# Role of PPARG in Chemosensitivity-Regulating Network for Hypopharyngeal Squamous Cell Carcinoma

**DOI:** 10.1155/2023/6019318

**Published:** 2023-09-25

**Authors:** Fanyong Kong, Boxuan Han, Jiaming Chen, Xixi Shen, Lizhen Hou, Jugao Fang, Meng Lian

**Affiliations:** ^1^Department of Otorhinolaryngology, Beijing Shunyi District Hospital, Shunyi Teaching Hospital of Capital Medical University, Beijing 101300, China; ^2^Department of Otorhinolaryngology Head and Neck Surgery, Beijing Tongren Hospital, Capital Medical University, Beijing 100730, China

## Abstract

PPARG has been reported to promote chemosensitivity in hypopharyngeal squamous cell carcinoma (HSCC). However, few studies tested its significance in the texture of a complex molecular network regulating chemosensitivity in HSCC. Here, we first employed RNA expression data analysis and literature data mining to uncover candidate genes related to HSCC chemosensitivity. Then, we constructed the molecular network regulating chemosensitivity in HSCC. After that, we employed degree centrality (DC) and weighted centrality (WC) to test the significance of PPARG within the regulating network. Pathway enrichment was done to study the cofunctions of PPARG and the rest of the genes within the network. The findings of our study contribute to the construction of a comprehensive network that regulates HSCC chemosensitivity, consisting of 57 genes, including PPARG. Notably, within this network, PPARG demonstrates a ranking of #5 and #13 based on DC and WC, respectively. Moreover, PPARG is connected to 29 out of the 57 genes and plays roles in multiple functional groups. These top related genes include AKT1, TP53, PTEN, MAPK1, NOTCH1, BECN1, PTGS2, SPP1, and RAC1. PPARG gets enriched in several key functional groups that have been implicated in the regulation of chemosensitivity, including those associated with the response to nutrients, vitamins, and peptides, the cellular response to chemical stress, and the regulation of hormone secretion and growth. Our results emphasize the involvement of PPARG and its interconnectedness with other genes in the regulation of HSCC chemosensitivity.

## 1. Introduction

Hypopharyngeal squamous cell carcinoma (HSCC) is one of the worst prognostic malignant tumors [1, 2], characterized by hidden location, strong infiltration, easy submucosal spread, and multicentric growth of the primary lesion. Because there are no apparent symptoms in the early stage, 70-85% of patients with HSCC are diagnosed at stage III or IV, with 5-year overall survival rates around 15-45% [3].

Combined chemotherapy, surgery, and radiation therapy are commonly used in the multidisciplinary treatment of HSCC, and cisplatin is the most widely used platinum-based chemotherapeutic agent for HSCC [4]. However, chemotherapy resistance could severely limit the clinical efficiency and the improvement of survival rate in patients with HSCC [5].

There is a suggestion that PPARG has the potential to enhance the chemosensitivity of HSCC tumor cells [6]. However, significant expression variation of PPARG exists in chemotherapy-sensitive patients, which may be influenced by multiple factors, including the TNM (tumor, node, and metastasis) stage, a predictor of chemosensitivity [7]. Moreover, multiple other chemosensitivity promoters have been shown to be inhibited by HSCC, such as TIPE2 [8, 9] and BECN1 [10, 11]. Zhao et al. showed that TIPE2 might enhance chemosensitivity by downregulating MDR1 transcription in hypopharyngeal carcinoma [9]. Sun et al. found that overexpression of the BECN1 gene could upregulate chemosensitivity to anticancer drugs by enhancing therapy-induced apoptosis in cervix squamous carcinoma CaSki cells [11]. However, the expression of both TIPE2 and BECN1 was found to be significantly downregulated in HSCC [8, 10]. Identifying the relationship between PPARG and these genetic markers influencing chemosensitivity may help in understanding the role of PPARG in the chemosensitivity of HSCC.

Here, we employed two gene expression datasets and literature-based knowledge data to construct genetic networks regulating chemosensitivity in HSCC and evaluate the significance of PPARG and its cofunctions with other genetic markers. Our results proposed a complex network that could influence the chemosensitivity of HSCC and highlighted the importance of PPARG within the network.

## 2. Materials and Methods

### 2.1. Extract CSP-Significant Genes from Gene Expression Datasets

Two microarray expression datasets, including 32 HSCC patients, were used to explore candidate genes that were related to chemotherapy in HSCC, including 19 chemotherapy-sensitive patients (CSP) and 13 chemotherapy-non-sensitive patients (CNSP). All the patients were recruited by the Department of Head and Neck Surgery, Beijing Tongren Hospital. We employed the two datasets in exploring the possible relationship between PPARG and chemosensitivity in HSCC [6, 7]. Both datasets, with the GEO IDs GSE85608 and GSE85607, were submitted to the Gene Expression Omnibus (GEO; https://www.ncbi.nlm.nih.gov/geo/). Written consent forms were obtained from all patients included in the study at the time of data acquisition for use of the datasets in publication. We noted during the previous studies that, besides PPARG, there were multiple other genes that presented significant expression changes and thus may play a role in HSCC chemosensitivity regulation. Here, we extracted and analyzed the genes identified from the CSP and CNSP comparison. These genes, together with literature data mining uncovered chemosensitivity genes, were used to construct the chemosensitivity-regulating network for HSCC. The significance of normalized degree centrality (DC) and weighted centrality (WC), widely employed metrics for assessing the significance of a vertex (gene) in a network, was utilized to evaluate the involvement of PPARG in the chemosensitivity-regulating network for HSCC [12]. The chemosensitivity gene identification using literature data mining was described in the following section.

### 2.2. Identify Chemosensitivity Genes for HSCC Using Literature Data Mining

Assisted by using Pathway Studio (http://www.pathwaystudio.com), we employed data mining to identify genes promoting chemosensitivity and also inhibited by HSCC, or genes inhibiting chemosensitivity but activated by HSCC. The union of the two gene groups was used as candidate genes that promote chemosensitivity genes in HSCC.

### 2.3. Construct the Chemosensitivity-Regulation Network for HSCC

For the genes that were identified from the above two steps, we constructed the gene-gene interaction (GGI) network following the instruction: https://supportcontent.elsevier.com/Support%20Hub/Pathway%20Studio/Network%20Builder%20basic%20_Interactive%20NB%20v114.pdf. These unconnected genes were removed from the network without further analysis. The exclusion of these genes was motivated by two primary reasons. Firstly, we posited that genes involved in the regulation of HSCC chemosensitivity should exhibit functional connections. Candidates lacking connections with other regulators may be considered outliers with limited or no significance in HSCC regulation. Therefore, removing such genes helps reduce noise in the potential HSCC regulating network. Secondly, the assessment of significance was based on two network centrality metrics (detailed descriptions are provided in the following section), which are not applicable to genes lacking connections since they would receive a score of zero. Moreover, we add the term “chemosensitivity” and “hypopharyngeal squamous cell carcinoma” to address their significance to chemosensitivity in HSCC.

### 2.4. Evaluate the Significance of PPARG in the Chemosensitivity-Regulation Network for HSCC

Vertex centrality is used to measure the significance of a vertex in the network. The simplest vertex centrality is degree centrality (DC), defined as the number of edges incident upon a vertex. For a graph with *n* vertices, the normalized degree centrality for vertex *v* is defined in

(1) $$\begin{array}{c} \text{DC}\left(v\right)=\frac{\text{Degree}\left(v\right)}{\sum_{i}\text{D}\text{egree}\left(v_{i}\right)}, \end{array}$$

where Degree(*v*_*i*_) is the degree for vertex *v*_*i*_, which equals the edges connected to the vertex; *i* = 1, ⋯, *n*.

In a weighted network, the corresponding vertex strength centrality is defined as the sum of the weights of these edges [12]. Considering that the literature-based relationships were reported by a different number of publications in different publication years, we employ a quality score (QScore) for each relationship (edge) as the weight [13]. QScore has been proposed as an effect metric evaluating literature-based relationships. Then, the normalized weighted degree centrality is defined as

(2) $$\begin{array}{c} \text{WC}\left(v\right)=\frac{\text{QScore}\left(v\right)}{\sum_{i}\text{Q}\text{Score}\left(v_{i}\right)}, \end{array}$$

where QScore(*v*_*i*_) is the sum of the QScores of all the relationships (edges) connecting the vertex *v*_*i*_; *i* = 1, ⋯, *n*.

### 2.5. Enrichment Analysis

To enhance our comprehension of the relationship between PPARG and its role in regulating HSCC chemosensitivity within the network, we carried out a gene set enrichment analysis utilizing Gene Ontology (GO) terms. This analysis involved utilizing the genes that make up the HSCC chemosensitivity-regulating network as input. The findings encompass the identification of highly enriched pathways and an evaluation of gene cofunctions, which are determined by shared GO terms.

## 3. Results

### 3.1. Significant Genes from CSP vs. CNSP Expression Comparison

For dataset GSE85607, 51 significant genes (LFC > 1 or < -1; *p* < 0.01) were identified from the comparison of the CSP and CNSP groups. The number of significant genes from dataset GSE85608 was 21, as shown in Table 1. The upregulated genes were highlighted in italics, and downregulated ones were not. Interestingly, no overlap was identified between the two groups of significant genes, suggesting the diversity of genetic markers influencing the chemosensitivity of HSCC. Although a significant expression change in CSP patients does not guarantee a gene regulating the chemosensitivity, it indicates that the gene could be a candidate worthy of further evaluation for its significance in the chemosensitivity of HSCC. In summary, the analysis of RNA expression data revealed a set of 31 genes that have the potential to act as inhibitors of HSCC chemosensitivity (downregulated in the CSP group), as well as 41 genes that show potential as promoters (upregulated in the CSP group).

### 3.2. Uncover HSCC Chemosensitivity-Related Genes

**Figure 1:**
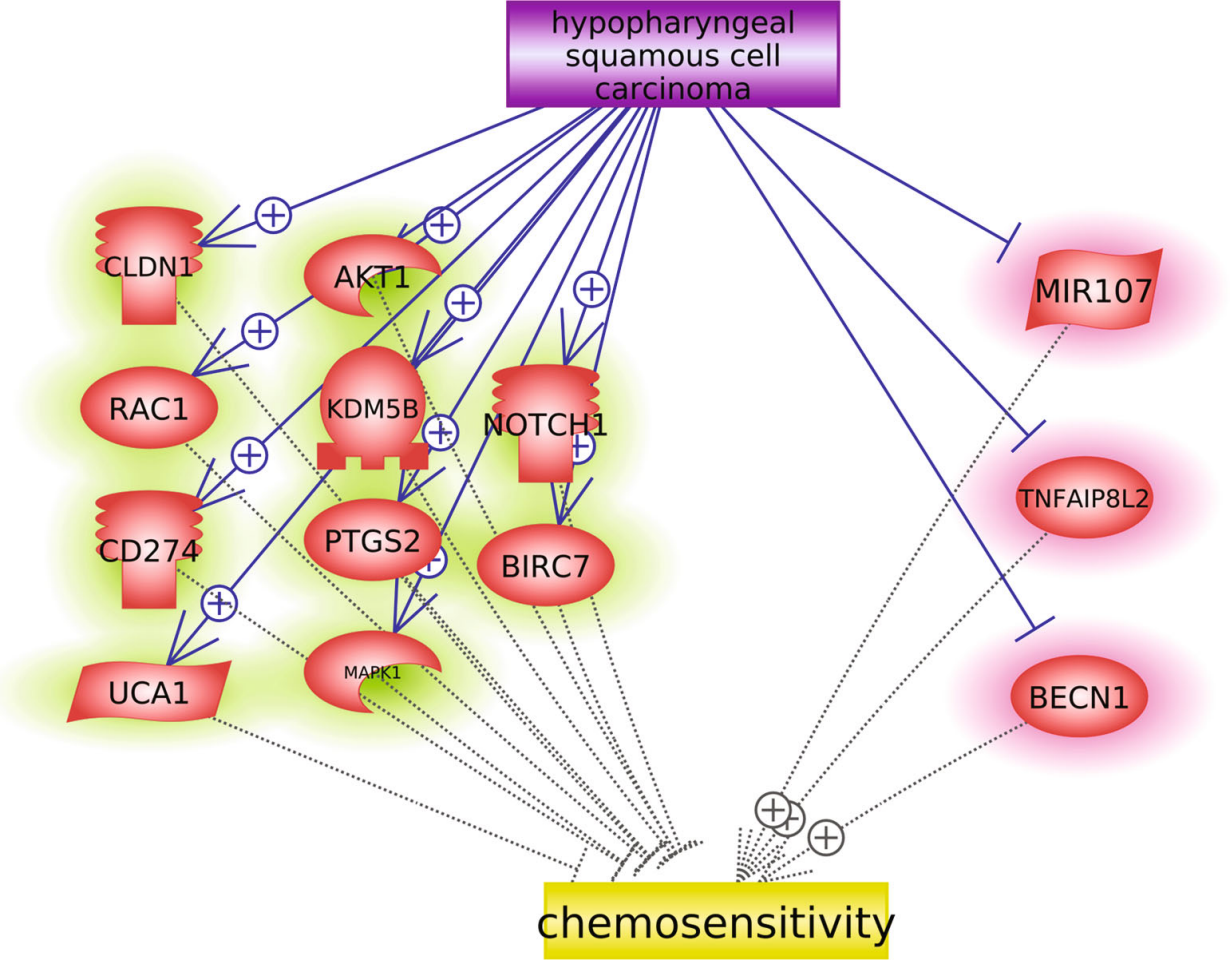
HSCC-driven molecules influencing chemosensitivity.

To explore the relationship between PPARG and other HSCC chemosensitivity-related genetic markers, we first used literature data mining that uncovered 523 genetic markers promoting chemosensitivity in different diseases. These 523 promoters were supported by over 2,900 scientific references (see Supplementary Table 1). However, only a few have been reported to have a direct role in promoting chemosensitivity in HSCC or hypopharyngeal cancer, including ING4, TP53, PPARG, and PTEN. Moreover, out of these 523 chemosensitivity promoters, three got inhibited in HSCC, as shown in Figure 1 (highlighted in red). Therefore, we identified seven literature-based molecules as chemosensitivity promoters in HSCC.

We also identified 593 genetic markers inhibiting chemosensitivity, which were supported by over 3,100 references (see Supplementary Table 2). However, only four genes have been reported to inhibit the chemosensitivity in HSCC or hypopharyngeal carcinoma, including PTGS2, PHF20, ABCC1, and MCL1. In addition, out of these 593 chemosensitivity inhibitors, ten were got promoted in HSCC, as shown in Figure 1 (highlighted in green). Thus, we identified 14 literature-based molecules as chemosensitivity inhibitors in HSCC.

### 3.3. Construct HSCC Chemosensitivity-Regulating Genetic Network

**Figure 2:**
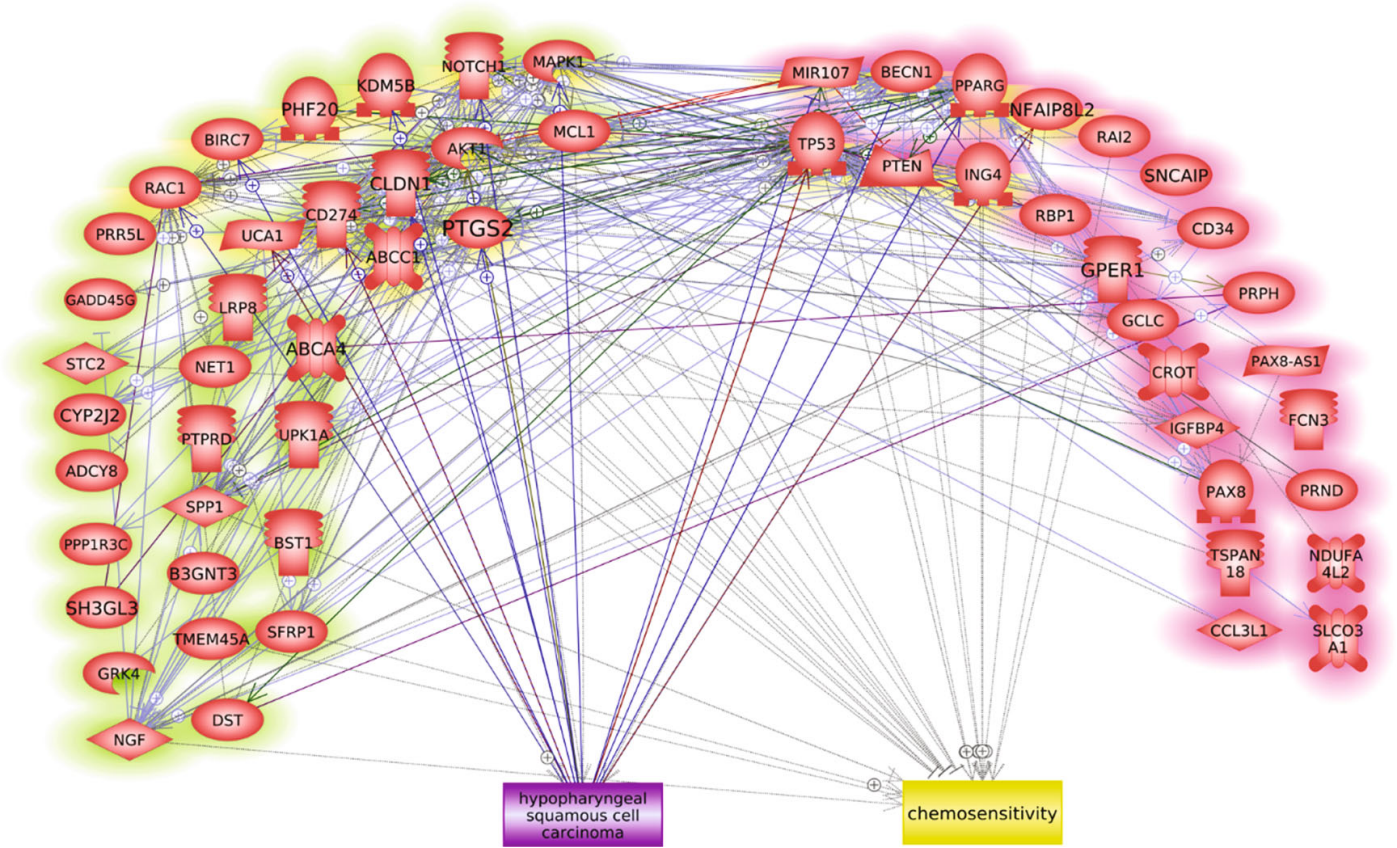
HSCC chemosensitivity-regulating network. Molecules highlighted in green are inhibitors of chemosensitivity; those in red are chemosensitivity promoters.

For the 48 chemosensitivity promoters and 45 inhibitors identified as mentioned above, a GGI analysis has been conducted by using Pathway Studio, as shown in Figure 2. In total, 36 genes presented no connection with any other genes, leaving 57 genes to compose the chemosensitivity-regulating network (Figure 2), including 24 inhibitors and 33 promoters. To address the significance of these molecules to HSCC and chemosensitivity, we added these two items into the GGI network for later evaluation purposes. In total, there were 467 edges within the network, supported by over 13,000 references (see Supplementary Table 3).

### 3.4. Significance Weight of PPARG

By using degree centrality (DC), PPARG ranked no. 5 out of 57 molecules with a DC = 5.15. The average DC of all 57 molecules is 1.65 ± 2.11. Our results indicate that PPARG is a hub molecular within the HSCC chemosensitivity-regulating network.

**Figure 3:**
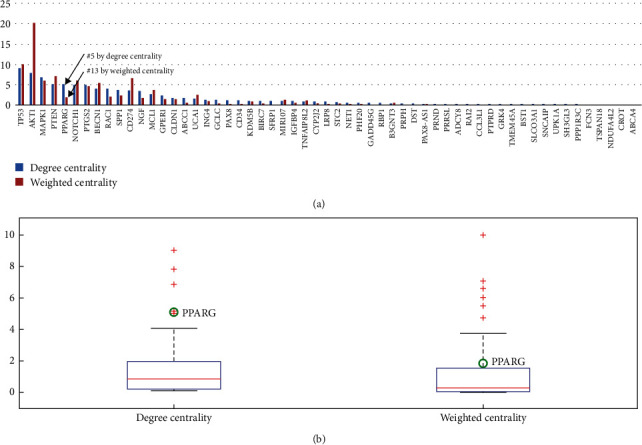
Weight of PPARG in the HSCC chemosensitivity-regulating network. (a) Bar plot of the weight of each molecule. (b) Boxplot depicting the results of centrality weighting using two different types.

By using weighted centrality, PPARG ranked no. 13 out of 57 molecules with a WC = 1.98. The average WC of all 57 molecules is 1.65 ± 3.32. These results indicate that the connections of PPARG to other vertex within the HSCC chemosensitivity-regulating network are also well supported by literature data (Figure 3).

### 3.5. Enrichment Analysis

**Figure 4:**
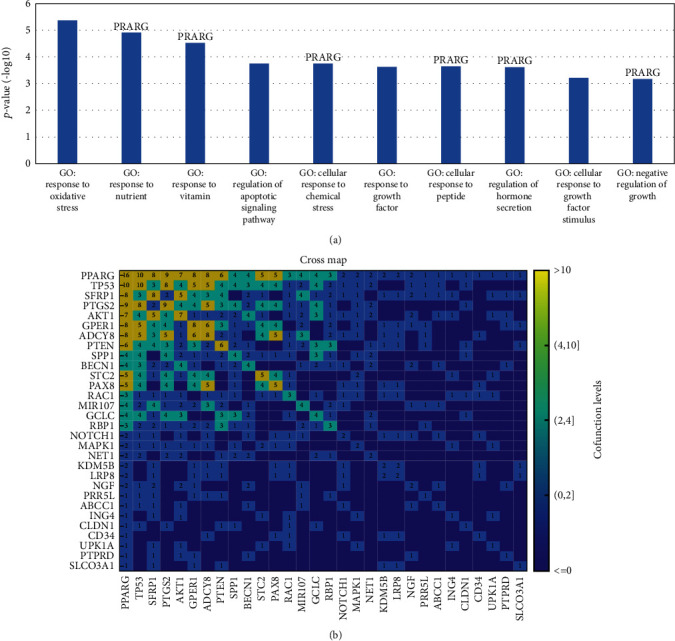
PPARG cofunctions with 29 genes in different GO terms implicated with chemosensitivity. (a) The top 10 GO terms are enriched by the 57 molecules regulating HSCC chemosensitivity. The pathways with “PPARG” on the top represent PPARG gets enriched within the GO term. (b) The cross-map shows the cofunction of PPARG with other molecules. The numbers represent the number of GO terms involving the two corresponding molecules.

Enrichment analysis showed that the 57 molecules were significantly enriched within 35 Gene Ontology (GO) terms. PPARG has been enriched within six out of the top ten GO terms (FDR corrected *p* value < 6.83*e* − 4), as shown in Figure 4(a). Most of these pathways have been implicated with chemosensitivity in different diseases, supporting the importance of PPARG in chemosensitivity regulation.

It is also worth mentioning that PPARG was connecting with 29 out of the 57 molecules in different GO terms influencing chemosensitivities, as shown in Figure 4(b). The numbers within the map indicate the number of GO terms the two corresponding molecules function in.

## 4. Discussion

In this study, we identified 93 candidate molecules regulating HSCC chemosensitivity using HSCC RNA expression data and literature data mining. Out of these 93 molecules, 57 presented complex connections with each other, including PPARG, composing a genetic network regulating HSCC chemosensitivity. The centrality score analysis suggested that PPARG is a hub gene within the network, and the relations (edges) connecting PPARG were well supported by literature data. Moreover, PPARG cofunctions with 29 out of the 57 genes in different GO terms, which may add to the understanding of the roles of PPARG in chemosensitivity regulation.

The four genes that outweigh PPARG in terms of degree centrality are TP53, AKT1, MAPK1, and PTEN. Two of these genes were implicated as inhibitors of chemosensitivity (MAPK1 and AKT1) that got activated in HSCC. Back in 2007, Shimada et al. showed that MAPK1 activation reduces the chemosensitivity in human prostate cancer cells [14]. Multiple other studies later pointed out that MAPK1 inhibition could induce apoptosis and enhances chemosensitivity in tumor cells [15, 16]. Similarly, the downregulation of AKT1 has been shown to enhance the chemosensitivity of multiple tumor cells, including non-small-cell lung cancer, esophageal squamous cell carcinoma, and head and neck squamous cell carcinoma [17–19]. The levels of AKT1 and MAPK1 could increase significantly with the progression of the clinical stage of HSCC [20], which is consistent with the stage-associated chemotherapy resistance in HSCC [7].

The other two genes that outweigh PPARG in terms of degree centrality are TP53 and PTEN. These two genes are chemosensitivity promoters that get inhibited in HSCC. PTEN has been found to enhance the chemosensitivity of multiple cancer cells, including hypopharyngeal cancer [21], endometrial carcinoma cells [22], ovarian cancer [23], and bladder cancer cells [23]. As a well-known tumor suppressor, P53 promotes DNA damage and apoptosis and plays a key role in the chemosensitivity of many tumor types, including hypopharyngeal cancer [24–26]. Recently, Sun et al. revealed that low expression of TP53 was associated with the advanced stage of HSCC [27]. TP53 is an activator of PTEN by binding to a site within the PTEN promoter region [28]. Therefore, the suppression of TP53 expression by HSCC may also influence the expression of PTEN.

To note, PPARG was linked to all these four genes with a higher degree centrality score. It has been shown that PPARG can induce the expression of TP53 and PTEN by binding directly to the promoter region of these two genes [29, 30] and therefore contribute to the chemosensitivity promotion in HSCC. PPARG activation has also been shown to inhibit AKT1 [31] and PDGF-BB-mediated phospho-MAPK1 activity by blocking its nuclear translocation [32]. This could also add to its mechanism in chemosensitivity promotion. Moreover, in the pathway enrichment analysis, TP53, AKT1, PTEN, and MAPK1 cofunction with PPARG in 10, 7, 6, and 2 GO terms, respectively (Figure 4(b)). These results provide further support to the connection between PPARG and chemosensitivity regulation in HSCC.

Most of the top enriched pathways have been implicated with chemosensitivity. For instance, oxidative stress and its effectors have been found to play critical roles in carcinogenesis and chemoresistance [33]. LINC01615 maintains cell survival in adaptation to nutrient starvation through the pentose phosphate pathway and modulates chemosensitivity in colorectal cancer [34]. Vitamin D has been found to enhance cisplatin chemotherapy and is suggested to be supplied during chemotherapy [35]. Through enhancing apoptosis, multiple molecules (e.g., Bcl-xL DNAzymes) promote radiosensitivity and chemosensitivity in cancer cells [36]. Recently, Jaidee et al. showed that the inhibition of fibroblast growth factor receptor 2 enhances chemosensitivity to gemcitabine in cholangiocarcinoma [37]. These results not only supported the validity of the 57 candidate chemosensitivity-related genes that we identified through the process of this study but also suggested the potential mechanisms of how these genes, including PPARG, influence the chemosensitivity in cancer cells.

Ranked by weighted centrality score, besides the four genes (AKT1, TP53, PTEN, and MAPK1) we mentioned above, there were also eight other genes that outweigh PPARG, including CD274, NOTCH1, BECN1, PTGS2, MCL1, UCA1, SPP1, and RAC1. As the weight was relationship QScore, which measures the strength of literature support for the relationship [13], a high weighted centrality indicates a large number of recent studies supporting the relations the gene presented within the chemosensitive regulation network. To note, PPARG got enriched in the same GO terms with most of these genes (9 out of 12; for NOTCH1, BECN1, PTGS2, SPP1, and RAC1, the number of shared GO terms is 2, 4, 9, 4, and 3, respectively), suggesting the functional connection between PPARG and these well-studied genes.

There are several limitations in this study that require attention in future research. Firstly, while the network-based significance ranking highlights the importance of PPARG in HSCC's chemosensitivity compared to other regulators, it does not provide a detailed understanding of PPARG's specific role in HSCC chemosensitivity. Secondly, although gene set enrichment analysis reveals that PPARG interacts with various molecules and participates in multiple functional pathways related to chemosensitivity, this pathway-based information remains somewhat vague when it comes to specifying how PPARG interacts with other regulators and their respective contributions to HSCC chemosensitivity. Therefore, further studies using more extensive data are needed to investigate the precise role of PPARG and its interactions with other regulators involved in HSCC chemosensitivity.

## 5. Conclusion

The outcomes of our study indicate that PPARG serves as a hub gene within a significant genetic network that regulates chemosensitivity in patients with HSCC. The construction of a comprehensive network comprising 57 genes, along with the identification of enriched GO terms, contributes to a deeper understanding of the roles played by PPARG and other genes in the regulation of HSCC chemosensitivity.

## Figures and Tables

**Table 1a:** (a) 51 genes from GSE85607

SPP1	SH3GL3	NGF	ABCA4	MPP4	Hs.552282	CORO6	TMEM45A	ADPRHL1
BST1	PTPRD	EHBP1	STC2	FEM1B	GADD45G	NET1	DST	LRP8
*C6orf52*	*KATNAL1*	*NT5M*	*CTRC*	*ADCY8*	*GRK4*	*POMZP3*	*Hs.21820*	*PRR5L*
*CROT*	*FAM169A*	*ITPRIPL1*	*RAI2*	*PAX8*	*Hs.533844*	*SELENBP1*	*ACOX2*	*Hs.565170*
*KIR2DL5B*	*CD34*	*DRC7*	*OR1V1*	*IGFBP4*	*CCDC184*	*PRND*	*GPER1*	*PAX8-AS1*
*GPER1*	*GGT6*	*RBP1*	*SNCAIP*	*NDUFA4L2*	*KREMEN2*			

**Table 1b:** (b) 21 genes from GSE85608

SFRP1	PPP1R3C	CYP2J2	C11orf44	LOC339535	RIMS2	C16orf73	UPK1A	B3GNT3
SPIN2B	MYOM3	ACOT11	SPTLC3	*LILRA5*	*FCN3*	*SLCO3A1*	*CCL3L1*	*GCLC*
*PRPH*	*LOC392437*	*TSPAN18*						

## Data Availability

The data of this study are available from the corresponding authors upon reasonable request.

## Supplementary Materials

Supplementary 1 Supplementary Table 1: the reference information supporting the 523 chemosensitivity promoters.

Supplementary 2 Supplementary Table 2: the reference information supporting the 593 chemosensitivity inhibitors.

Supplementary 3 Supplementary Table 3: the reference information supporting the HSCC chemosensitivity-regulating network.
