# Supplementary material for: Role of PPARG in Chemosensitivity-Regulating Network for Hypopharyngeal Squamous Cell Carcinoma
Source: PPAR Res. 2023 Sep 25;2023:6019318. doi: 10.1155/2023/6019318 (PMC10545467; doi:10.1155/2023/6019318)
Supplement: Supplementary 1 — Supplementary Table 1: the reference information supporting the 523 chemosensitivity promoters. [file 6019318.f1.pdf]

Ref info supporting the chemosensitivity_promoters

Relation Name	PMID	DOI
positive Regulation: ESRP1 --+> chemosensitivity	33495408	10.18632/aging.202
positive Regulation: ESRP1 --+> chemosensitivity	33495408	10.18632/aging.202
positive Regulation: ESRP1 --+> chemosensitivity	33495408	10.18632/aging.202
positive Regulation: FOXA3 --+> chemosensitivity	33292133	10.2174/156800962C
positive Regulation: ULK2 --+> chemosensitivity	32848049	10.1194/jlr.RA120C
positive Regulation: ULK2 --+> chemosensitivity	30655741	10.3892/ol.2018.96
positive Regulation: ULK2 --+> chemosensitivity	30655741	10.3892/ol.2018.96
positive Regulation: ULK2 --+> chemosensitivity	34350832	10.4149/gpb_20210C
positive Regulation: HTR7 --+> chemosensitivity	30866045	10.1113/JP27705210
positive Regulation: MME --+> chemosensitivity	15788637	
positive Regulation: MME --+> chemosensitivity	22895534	10.3892/ijo.2012.1
positive Regulation: MME --+> chemosensitivity	14734478	10.1158/1078-0432.
positive Regulation: SEMA3F --+> chemosensitivity	30824197	10.1016/j.molmed.2
positive Regulation: SEMA3F --+> chemosensitivity	36119535	10.3389/fonc.2022.
positive Regulation: SEMA3F --+> chemosensitivity	36119535	10.3389/fonc.2022.
positive Regulation: ALB --+> chemosensitivity	23063551	10.1016/j.jconrel.
positive Regulation: ALB --+> chemosensitivity	23063551	10.1016/j.jconrel.
positive Regulation: TNFRSF12A --+> chemosensitivity	34517088	10.1016/j.cellsig.
positive Regulation: CYP3A4 --+> chemosensitivity	19011599	10.1038/cgt.2008.9
positive Regulation: RHOA --+> chemosensitivity	15448013	10.1158/1078-0432.
positive Regulation: MIR129-1 --+> chemosensitivity	25218158	10.1016/j.bbrc.201
positive Regulation: MIR129-1 --+> chemosensitivity	25218158	10.1016/j.bbrc.201
positive Regulation: MIR129-1 --+> chemosensitivity	33333206	10.1016/j.addr.202
positive Regulation: MIR129-1 --+> chemosensitivity	28012924	10.1016/j.biopha.2
positive Regulation: MIR129-1 --+> chemosensitivity	28012924	10.1016/j.biopha.2
positive Regulation: MIR129-1 --+> chemosensitivity	29864913	10.1016/j.biopha.2
positive Regulation: MIR129-1 --+> chemosensitivity	29864913	10.1016/j.biopha.2
positive Regulation: MIR129-1 --+> chemosensitivity	29864913	10.1016/j.biopha.2
positive Regulation: MIR129-1 --+> chemosensitivity	30021343	10.1016/j.biopha.2
positive Regulation: MIR129-1 --+> chemosensitivity	30551491	10.1016/j.biopha.2
positive Regulation: MIR129-1 --+> chemosensitivity	28095367	10.1515/hsz-2016-C
positive Regulation: MIR129-1 --+> chemosensitivity	29531296	10.1038/s41419-018
positive Regulation: MIR129-1 --+> chemosensitivity	32893526	10.1631/jzus.B200C
positive Regulation: MIR129-1 --+> chemosensitivity	26518892	10.14715/cmb/2015.
positive Regulation: MIR129-1 --+> chemosensitivity	23744359	10.1038/cddis.2013
positive Regulation: MIR129-1 --+> chemosensitivity	25571061	10.1109/EMBC.2014.
positive Regulation: MIR129-1 --+> chemosensitivity	29864913	10.1016/j.biopha.2
positive Regulation: MIR129-1 --+> chemosensitivity	29864913	10.1016/j.biopha.2
positive Regulation: MIR129-1 --+> chemosensitivity	29864913	10.1016/j.biopha.2
positive Regulation: MIR129-1 --+> chemosensitivity		10.3390/molecules2
positive Regulation: MIR129-1 --+> chemosensitivity		10.3390/molecules2
positive Regulation: MIR129-1 --+> chemosensitivity	35578599	10.1155/2022/79817
positive Regulation: MIR129-1 --+> chemosensitivity	35637945	10.7150/ijbs.73504
positive Regulation: MIR129-1 --+> chemosensitivity	35031927	10.1007/s11033-021
positive Regulation: MIR129-1 --+> chemosensitivity	36119459	10.4084/MJHID.2022
positive Regulation: MIR129-1 --+> chemosensitivity	34132932	10.1007/s10565-021
positive Regulation: MIR129-1 --+> chemosensitivity	34132932	10.1007/s10565-021
positive Regulation: LINC00672 --+> chemosensitivity	28232485	10.1074/jbc.M116.7
positive Regulation: LINC00672 --+> chemosensitivity	28232485	10.1074/jbc.M116.7
positive Regulation: LINC00672 --+> chemosensitivity	29775889	10.1016/j.biopha.2

positive Regulation: LINC00672 --+> chemosensitivity	28232485	10.1074/jbc.M116.7
positive Regulation: LINC00672 --+> chemosensitivity	28232485	10.1074/jbc.M116.7
positive Regulation: LINC00672 --+> chemosensitivity	28232485	10.1074/jbc.M116.7
positive Regulation: LINC00672 --+> chemosensitivity	28232485	10.1074/jbc.M116.7
positive Regulation: LINC00672 --+> chemosensitivity	30127216	10.4103/0366-6999.
positive Regulation: LINC00672 --+> chemosensitivity	32633343	10.26355/eurrev_20
positive Regulation: LINC00672 --+> chemosensitivity	28232485	10.1074/jbc.M116.7
positive Regulation: SMAD2 --+> chemosensitivity	34453645	10.1007/s11010-021
positive Regulation: KLF4 --+> chemosensitivity		10.1016/j.exphem.2
positive Regulation: KLF4 --+> chemosensitivity	30176890	10.1186/s12964-018
positive Regulation: KLF4 --+> chemosensitivity	30176890	10.1186/s12964-018
positive Regulation: KLF4 --+> chemosensitivity	30176890	10.1186/s12964-018
positive Regulation: KLF4 --+> chemosensitivity	24551169	10.1371/journal.pc
positive Regulation: KLF4 --+> chemosensitivity	30176890	10.1186/s12964-018
positive Regulation: KLF4 --+> chemosensitivity	30176890	10.1186/s12964-018
positive Regulation: MIR524 --+> chemosensitivity	29221202	10.18632/oncotarge
positive Regulation: MIR524 --+> chemosensitivity	35922753	10.1186/s11658-022
positive Regulation: MAGI3 --+> chemosensitivity	35864508	10.1186/s12943-022
positive Regulation: MAGI3 --+> chemosensitivity	35864508	10.1186/s12943-022
positive Regulation: MAGI3 --+> chemosensitivity	35864508	10.1186/s12943-022
positive Regulation: MAGI3 --+> chemosensitivity	35864508	10.1186/s12943-022
positive Regulation: MAGI3 --+> chemosensitivity	35864508	10.1186/s12943-022
positive Regulation: TNFAIP8L2 --+> chemosensitivity	30114619	10.1016/j.molimm.2
positive Regulation: HNF4A --+> chemosensitivity	33462379	10.1038/s12276-020
positive Regulation: HNF4A --+> chemosensitivity	33462379	10.1038/s12276-020
positive Regulation: HNF4A --+> chemosensitivity	25175346	10.1002/jps.241281
positive Regulation: HNF4A --+> chemosensitivity	25175346	10.1002/jps.241281
positive Regulation: TP73 --+> chemosensitivity	22446689	10.1016/j.jhep.201
positive Regulation: TP73 --+> chemosensitivity	20156675	10.1016/j.ejca.201
positive Regulation: TP73 --+> chemosensitivity	15219617	10.1016/j.semcance
positive Regulation: TP73 --+> chemosensitivity	18054518	10.1016/j.drup.200
positive Regulation: TP73 --+> chemosensitivity	21195657	10.1016/j.drup.201
positive Regulation: TP73 --+> chemosensitivity	18620901	10.1016/j.oraloncc
positive Regulation: TP73 --+> chemosensitivity	16530485	10.1016/j.molmed.2
positive Regulation: TP73 --+> chemosensitivity	22340593	10.1016/j.ccr.2011
positive Regulation: TP73 --+> chemosensitivity	21293058	
positive Regulation: TP73 --+> chemosensitivity	21293058	
positive Regulation: TP73 --+> chemosensitivity	21293058	
positive Regulation: TP73 --+> chemosensitivity	21293058	
positive Regulation: TP73 --+> chemosensitivity	21293058	
positive Regulation: TP73 --+> chemosensitivity	21293058	
positive Regulation: TP73 --+> chemosensitivity	17446929	
positive Regulation: TP73 --+> chemosensitivity	17626635	
positive Regulation: TP73 --+> chemosensitivity	18806757	
positive Regulation: TP73 --+> chemosensitivity	16195739	
positive Regulation: TP73 --+> chemosensitivity	15153941	
positive Regulation: TP73 --+> chemosensitivity	15153941	
positive Regulation: TP73 --+> chemosensitivity	26459801	10.3892/ijo.2015.3
positive Regulation: TP73 --+> chemosensitivity	27376739	10.1007/978-981-10
positive Regulation: TP73 --+> chemosensitivity	27376730	10.1007/978-981-10
positive Regulation: TP73 --+> chemosensitivity	16773194	
positive Regulation: TP73 --+> chemosensitivity	21293058	10.1172/JCI43897
positive Regulation: TP73 --+> chemosensitivity	21293058	10.1172/JCI43897
positive Regulation: TP73 --+> chemosensitivity	21293058	10.1172/JCI43897

positive Regulation: TP73 --+> chemosensitivity	21391908	
positive Regulation: TP73 --+> chemosensitivity	21391909	
positive Regulation: TP73 --+> chemosensitivity	21391909	
positive Regulation: TP73 --+> chemosensitivity	21391904	
positive Regulation: TP73 --+> chemosensitivity	22762204	10.1042/BSR2011012
positive Regulation: TP73 --+> chemosensitivity	21861192	10.1007/s10495-011
positive Regulation: TP73 --+> chemosensitivity	24052075	10.1038/cddis.2013
positive Regulation: TP73 --+> chemosensitivity	23494264	10.1007/s12013-013
positive Regulation: TP73 --+> chemosensitivity	22900074	10.1371/journal.pc
positive Regulation: TP73 --+> chemosensitivity	24052409	10.1007/s10495-013
positive Regulation: TP73 --+> chemosensitivity		10.1007/978-94-007
positive Regulation: TP73 --+> chemosensitivity	24946002	10.1038/bjc.2014.2
positive Regulation: TP73 --+> chemosensitivity	24722210	10.1038/cdd.2014.4
positive Regulation: TP73 --+> chemosensitivity	15678153	10.1038/sj.cgt.770
positive Regulation: TP73 --+> chemosensitivity	16195739	10.1038/sj.cdd.440
positive Regulation: TP73 --+> chemosensitivity	17446929	10.1172/JCI30866
positive Regulation: TP73 --+> chemosensitivity	18565851	10.1182/blood-2007
positive Regulation: TP73 --+> chemosensitivity	19861456	10.1158/1078-0432.
positive Regulation: TP73 --+> chemosensitivity	20100536	10.1016/j.tox.2010
positive Regulation: TP73 --+> chemosensitivity	21293058	10.1172/JCI4389710
positive Regulation: TP73 --+> chemosensitivity	21436470	10.18632/oncotarge
positive Regulation: TP73 --+> chemosensitivity	36352215	10.1007/978-3-031-
positive Regulation: CAST --+> chemosensitivity	24589339	10.1016/j.ajpath.2
positive Regulation: CCNL2 --+> chemosensitivity	22799343	10.7314/apjcp.2012
positive Regulation: CCNL2 --+> chemosensitivity	22799343	10.7314/apjcp.2012
positive Regulation: CCNL2 --+> chemosensitivity	22799343	10.7314/apjcp.2012
positive Regulation: TFF1 --+> chemosensitivity	29809170	10.1172/JCI97755
positive Regulation: TFF1 --+> chemosensitivity	29210057	10.1002/ijc.311971
positive Regulation: TFF1 --+> chemosensitivity		10.1172/JCI97755
positive Regulation: MIR600HG --+> chemosensitivity	32270866	10.1042/BSR2020039
positive Regulation: MIR600HG --+> chemosensitivity	32270866	10.1042/BSR2020039
positive Regulation: MIR600HG --+> chemosensitivity	32270866	10.1042/BSR2020039
positive Regulation: MIR600HG --+> chemosensitivity	32270866	10.1042/BSR2020039
positive Regulation: TREX2 --+> chemosensitivity	34031538	10.1038/s41374-021
positive Regulation: ABCB5 --+> chemosensitivity		10.1053/j.gastro.2
positive Regulation: ABCB5 --+> chemosensitivity	34381520	10.1155/2021/39053
positive Regulation: DPEP1 --+> chemosensitivity	22363658	10.1371/journal.pc
positive Regulation: SFRP1 --+> chemosensitivity	24643460	10.2119/molmed.201
positive Regulation: SFRP1 --+> chemosensitivity	24643460	10.2119/molmed.201
positive Regulation: SFRP1 --+> chemosensitivity	24643460	10.2119/molmed.201
positive Regulation: MIR874 --+> chemosensitivity	28922711	10.1016/j.biopha.2
positive Regulation: MIR874 --+> chemosensitivity	29039607	10.3892/or.2017.60
positive Regulation: MIR874 --+> chemosensitivity	30106442	10.3892/or.2018.66
positive Regulation: MIR874 --+> chemosensitivity	30320370	10.3892/ijo.2018.4
positive Regulation: MIR874 --+> chemosensitivity	32016967	10.26355/eurrev_20
positive Regulation: MIR874 --+> chemosensitivity	32141542	10.26355/eurrev_20
positive Regulation: MIR874 --+> chemosensitivity	27221209	10.3892/or.2016.48
positive Regulation: MIR874 --+> chemosensitivity	30004169	10.1002/jbt.221681
positive Regulation: MIR874 --+> chemosensitivity	34716858	10.1007/s11010-021
positive Regulation: MIR874 --+> chemosensitivity	34716858	10.1007/s11010-021
positive Regulation: ING4 --+> chemosensitivity	15882981	10.1016/j.bbrc.200
positive Regulation: ING4 --+> chemosensitivity	27484725	10.3892/mmr.2016.5
positive Regulation: ING4 --+> chemosensitivity	20707719	10.1089/cbr.2010.0
positive Regulation: ING4 --+> chemosensitivity	22863759	10.1038/cgt.2012.5

positive Regulation: ING4 --+> chemosensitivity	21052098	10.1038/cgt.2010.6
positive Regulation: ING4 --+> chemosensitivity	23969950	10.3892/or.2013.26
positive Regulation: ING4 --+> chemosensitivity	23969950	10.3892/or.2013.26
positive Regulation: ING4 --+> chemosensitivity	23991130	10.1371/journal.pc
positive Regulation: ING4 --+> chemosensitivity	21553494	
positive Regulation: m_Hct --+> chemosensitivity	26747178	10.1007/s13277-015
positive Regulation: MIR584 --+> chemosensitivity	32346311	10.2147/CMAR.S2465
positive Regulation: MIR146A --+> chemosensitivity	23707524	10.1016/j.cellsig.
positive Regulation: MIR146A --+> chemosensitivity	28560455	10.3892/ijo.2017.4
positive Regulation: MIR146A --+> chemosensitivity	28975995	
positive Regulation: MIR146A --+> chemosensitivity	29966370	10.3390/ijms190719
positive Regulation: MIR146A --+> chemosensitivity	32626953	10.3892/ijmm.2020.
positive Regulation: MIR146A --+> chemosensitivity	32626953	10.3892/ijmm.2020.
positive Regulation: MIR146A --+> chemosensitivity	33390839	10.7150/ijbs.50773
positive Regulation: MIR146A --+> chemosensitivity	34281588	10.1186/s12967-021
positive Regulation: MIR146A --+> chemosensitivity	24565101	10.1186/1476-4598-
positive Regulation: MIR146A --+> chemosensitivity	27131313	10.3727/096504016X
positive Regulation: MIR146A --+> chemosensitivity	27131313	10.3727/096504016X
positive Regulation: MIR146A --+> chemosensitivity	27131313	10.3727/096504016X
positive Regulation: MIR146A --+> chemosensitivity	21610143	10.1158/1078-0432.
positive Regulation: MIR146A --+> chemosensitivity	28678319	
positive Regulation: MIR146A --+> chemosensitivity		
positive Regulation: MIR146A --+> chemosensitivity		10.7150/ijbs.50773
positive Regulation: MIR146A --+> chemosensitivity	35193602	10.1186/s12967-021
positive Regulation: MIR146A --+> chemosensitivity	36293478	10.3390/ijms232012
positive Regulation: APAF1 --+> chemosensitivity	14729468	10.1016/j.yexcr.20
positive Regulation: APAF1 --+> chemosensitivity	14729468	10.1016/j.yexcr.20
positive Regulation: APAF1 --+> chemosensitivity	15305193	
positive Regulation: APAF1 --+> chemosensitivity	33904968	10.1007/s12032-021
positive Regulation: MIR577 --+> chemosensitivity	31731194	10.1016/j.biopha.2
positive Regulation: MIR577 --+> chemosensitivity	33338745	10.1016/j.biopha.2
positive Regulation: MIR577 --+> chemosensitivity	30879950	10.1016/j.ymthe.20
positive Regulation: MIR577 --+> chemosensitivity	31115000	10.26355/eurrev_20
positive Regulation: MIR577 --+> chemosensitivity	30879950	10.1016/j.ymthe.20
positive Regulation: MIR577 --+> chemosensitivity	30879950	10.1016/j.ymthe.20
positive Regulation: MIR577 --+> chemosensitivity	33767583	10.7150/ijbs.45885
positive Regulation: MIR577 --+> chemosensitivity	34194499	10.1155/2021/99194
positive Regulation: MIR577 --+> chemosensitivity	28150434	10.1002/jbt.218881
positive Regulation: MIR206 --+> chemosensitivity	32800545	10.1016/j.bbrc.202
positive Regulation: MIR206 --+> chemosensitivity	32800545	10.1016/j.bbrc.202
positive Regulation: MIR206 --+> chemosensitivity		10.7150/ijbs.73949
positive Regulation: MIR144 --+> chemosensitivity	34020028	10.1016/j.semcance
positive Regulation: MIR144 --+> chemosensitivity	27508019	
positive Regulation: MIR144 --+> chemosensitivity	27508019	
positive Regulation: MIR144 --+> chemosensitivity	27508019	
positive Regulation: MIR144 --+> chemosensitivity	27508019	
positive Regulation: MIR144 --+> chemosensitivity	29504819	10.1080/15384047.2
positive Regulation: MIR144 --+> chemosensitivity	31659146	10.12659/MSM.91607
positive Regulation: MIR144 --+> chemosensitivity	31659146	10.12659/MSM.91607
positive Regulation: MIRLET7I --+> chemosensitivity	19776209	
positive Regulation: MIRLET7I --+> chemosensitivity		
positive Regulation: GAS5 --+> chemosensitivity	33631478	10.1016/j.prp.2021
positive Regulation: GAS5 --+> chemosensitivity	28686971	10.1016/j.biopha.2
positive Regulation: GAS5 --+> chemosensitivity	28686971	10.1016/j.biopha.2

positive Regulation: GAS5 --+> chemosensitivity	30606744	10.1042/BSR2018078
positive Regulation: GAS5 --+> chemosensitivity	31282278	10.1080/15384101.2
positive Regulation: GAS5 --+> chemosensitivity	31282278	10.1080/15384101.2
positive Regulation: GAS5 --+> chemosensitivity	31282278	10.1080/15384101.2
positive Regulation: GAS5 --+> chemosensitivity	33076450	10.3390/ijms212076
positive Regulation: GAS5 --+> chemosensitivity	33076450	10.3390/ijms212076
positive Regulation: GAS5 --+> chemosensitivity	28686971	10.1016/j.biopha.2
positive Regulation: GAS5 --+> chemosensitivity	28686971	10.1016/j.biopha.2
positive Regulation: GAS5 --+> chemosensitivity	32705220	10.3892/mmr.2020.1
positive Regulation: GAS5 --+> chemosensitivity	34425750	10.1186/s11658-021
positive Regulation: GAS5 --+> chemosensitivity	35337961	10.1016/j.drudis.2
positive Regulation: GAS5 --+> chemosensitivity	35295003	10.4103/aja2021117
positive Regulation: GAS5 --+> chemosensitivity	35295003	10.4103/aja2021117
positive Regulation: EXT1 --+> chemosensitivity	23457527	10.1371/journal.pc
positive Regulation: PMS2 --+> chemosensitivity	20178594	
positive Regulation: PMS2 --+> chemosensitivity	20178594	
positive Regulation: PMS2 --+> chemosensitivity	20178594	
positive Regulation: PMS2 --+> chemosensitivity	20178594	10.1186/1471-2407-
positive Regulation: MIR203A --+> chemosensitivity	32818543	10.1016/j.lfs.2020
positive Regulation: MIR203A --+> chemosensitivity	32818543	10.1016/j.lfs.2020
positive Regulation: MIR203A --+> chemosensitivity	27612668	10.1016/j.bbamcr.2
positive Regulation: MIR203A --+> chemosensitivity	29198335	10.1016/j.critrevc
positive Regulation: MIR203A --+> chemosensitivity	32961483	10.1016/j.neo.2020
positive Regulation: MIR203A --+> chemosensitivity	25843291	10.1016/j.canlet.2
positive Regulation: MIR203A --+> chemosensitivity	26790955	10.1016/j.yexcr.20
positive Regulation: MIR203A --+> chemosensitivity	27059735	10.1007/s13277-016
positive Regulation: MIR203A --+> chemosensitivity	27059735	10.1007/s13277-016
positive Regulation: MIR203A --+> chemosensitivity	27236538	10.1007/s13277-016
positive Regulation: MIR203A --+> chemosensitivity	28381160	10.1177/1010428317
positive Regulation: MIR203A --+> chemosensitivity	32961483	10.1016/j.neo.2020
positive Regulation: MIR203A --+> chemosensitivity	25482885	10.3892/or.2014.36
positive Regulation: MIR203A --+> chemosensitivity	25482885	10.3892/or.2014.36
positive Regulation: MIR203A --+> chemosensitivity	26782075	10.3892/ijmm.2016.
positive Regulation: MIR203A --+> chemosensitivity	25482885	10.3892/or.2014.36
positive Regulation: MIR203A --+> chemosensitivity	25482885	10.3892/or.2014.36
positive Regulation: MIR203A --+> chemosensitivity	32760222	10.1186/s12935-020
positive Regulation: MIR203A --+> chemosensitivity	33764270	10.1080/1120009X.2
positive Regulation: MIR203A --+> chemosensitivity	33764270	10.1080/1120009X.2
positive Regulation: MIR203A --+> chemosensitivity	35216281	10.3390/ijms230421
positive Regulation: MIR203A --+> chemosensitivity	35670956	10.1007/s13577-022
positive Regulation: MIR203A --+> chemosensitivity	35670956	10.1007/s13577-022
positive Regulation: MIR203A --+> chemosensitivity	35670956	10.1007/s13577-022
positive Regulation: MIR203A --+> chemosensitivity	35670956	10.1007/s13577-022
positive Regulation: FNDC5 --+> chemosensitivity	32353410	10.1016/j.cellsig.
positive Regulation: FNDC5 --+> chemosensitivity	32353410	10.1016/j.cellsig.
positive Regulation: FNDC5 --+> chemosensitivity	32353410	10.1016/j.cellsig.
positive Regulation: FNDC5 --+> chemosensitivity	32353410	10.1016/j.cellsig.
positive Regulation: FNDC5 --+> chemosensitivity	32353410	10.1016/j.cellsig.
positive Regulation: FNDC5 --+> chemosensitivity	31412018	10.12659/MSM.91762
positive Regulation: FNDC5 --+> chemosensitivity	31412018	10.12659/MSM.91762
positive Regulation: FNDC5 --+> chemosensitivity	31412018	10.12659/MSM.91762
positive Regulation: FNDC5 --+> chemosensitivity	31412018	10.12659/MSM.91762
positive Regulation: FNDC5 --+> chemosensitivity	31412018	10.12659/MSM.91762
positive Regulation: FNDC5 --+> chemosensitivity	31412018	10.12659/MSM.91762

positive Regulation: FNDC5 --+> chemosensitivity	31412018	10.12659/MSM.91762
positive Regulation: FNDC5 --+> chemosensitivity	31412018	10.12659/MSM.91762
positive Regulation: FNDC5 --+> chemosensitivity	31412018	10.12659/MSM.91762
positive Regulation: FNDC5 --+> chemosensitivity	31412018	10.12659/MSM.91762
positive Regulation: FNDC5 --+> chemosensitivity	31412018	10.12659/MSM.91762
positive Regulation: MIR126 --+> chemosensitivity	27622325	10.1016/j.bbrc.201
positive Regulation: MIR126 --+> chemosensitivity	27622325	10.1016/j.bbrc.201
positive Regulation: MIR126 --+> chemosensitivity	27622325	10.1016/j.bbrc.201
positive Regulation: MIR126 --+> chemosensitivity	33360300	10.1016/j.tranon.2
positive Regulation: MIR126 --+> chemosensitivity		10.1016/j.genrep.2
positive Regulation: MIR126 --+> chemosensitivity		10.12892/ejgo3658.
positive Regulation: MIR126 --+> chemosensitivity	30268144	10.1186/s12967-018
positive Regulation: MIR126 --+> chemosensitivity	25571061	10.1109/EMBC.2014.
positive Regulation: MIR126 --+> chemosensitivity	27622325	10.1016/j.bbrc.201
positive Regulation: MIR126 --+> chemosensitivity		10.1016/j.gendis.2
positive Regulation: MIR126 --+> chemosensitivity	36329030	10.1080/15384047.2
positive Regulation: TUSC7 --+> chemosensitivity	31837299	10.1016/j.cca.2019
positive Regulation: TUSC7 --+> chemosensitivity	31837299	10.1016/j.cca.2019
positive Regulation: HTR5A --+> chemosensitivity	12208084	10.1016/s1569-9048
positive Regulation: GRN --+> chemosensitivity		10.1053/j.gastro.2
positive Regulation: GRN --+> chemosensitivity	33087088	10.1186/s12885-020
positive Regulation: MUC13 --+> chemosensitivity	26673820	10.18632/oncotarge
positive Regulation: SDF-1alpha --+> chemosensitivity	23544165	10.1593/tlo.12268
positive Regulation: SDF-1alpha --+> chemosensitivity	24255072	10.1158/1078-0432.
positive Regulation: LRRFIP1 --+> chemosensitivity	33707114	10.1016/j.pan.2021
positive Regulation: BCL11B --+> chemosensitivity	33093445	10.1038/s41419-020
positive Regulation: IRAG1-AS1 --+> chemosensitivity	31273338	10.1038/s41388-019
positive Regulation: IRAG1-AS1 --+> chemosensitivity	31273338	10.1038/s41388-019
positive Regulation: IRAG1-AS1 --+> chemosensitivity	31273338	10.1038/s41388-019
positive Regulation: IRAG1-AS1 --+> chemosensitivity	31273338	10.1038/s41388-019
positive Regulation: IRAG1-AS1 --+> chemosensitivity	31273338	10.1038/s41388-019
positive Regulation: IRAG1-AS1 --+> chemosensitivity	35541920	10.7150/ijbs.70292
positive Regulation: FUT4 --+> chemosensitivity	24232099	10.1038/cddis.2013
positive Regulation: FUT4 --+> chemosensitivity	24232099	10.1038/cddis.2013
positive Regulation: WT1 --+> chemosensitivity	20820871	10.1007/s11060-010
positive Regulation: WT1 --+> chemosensitivity	35024404	10.1016/j.gore.202
positive Regulation: COL6A3 --+> chemosensitivity		10.1016/j.tem.2021
positive Regulation: PYCARD --+> chemosensitivity	21955833	10.1016/j.drup.201
positive Regulation: PYCARD --+> chemosensitivity	16331272	10.1038/sj.onc.120
positive Regulation: PYCARD --+> chemosensitivity	16331272	10.1038/sj.onc.120
positive Regulation: PYCARD --+> chemosensitivity	16331272	10.1038/sj.onc.120
positive Regulation: PYCARD --+> chemosensitivity	16331272	10.1038/sj.onc.120
positive Regulation: MIR513A1 --+> chemosensitivity	33592319	10.1016/j.vph.2021
positive Regulation: MIR513A1 --+> chemosensitivity	33469378	10.2147/CMAR.S2773
positive Regulation: MIR513A1 --+> chemosensitivity	36255069	10.1097/CAD.000000
positive Regulation: DDB2 --+> chemosensitivity	36190612	10.1007/s11033-022
positive Regulation: TBXT --+> chemosensitivity	24504414	10.3892/ijo.2014.2
positive Regulation: IFNB1 --+> chemosensitivity	32044356	10.1016/j.canlet.2
positive Regulation: IFNB1 --+> chemosensitivity	22542810	10.1016/j.canlet.2
positive Regulation: IFNB1 --+> chemosensitivity		10.1016/j.jns.2015
positive Regulation: IFNB1 --+> chemosensitivity	29557060	10.1007/s11060-018
positive Regulation: IFNB1 --+> chemosensitivity	29557060	10.1007/s11060-018
positive Regulation: IFNB1 --+> chemosensitivity	32367437	10.1007/s11060-020
positive Regulation: IFNB1 --+> chemosensitivity	32367437	10.1007/s11060-020

positive Regulation: IFNB1 --+> chemosensitivity	26329778	10.3892/or.2015.42
positive Regulation: IFNB1 --+> chemosensitivity	21327711	10.1007/s11060-011
positive Regulation: IFNB1 --+> chemosensitivity	22277391	10.5692/clinicalne
positive Regulation: MIR122 --+> chemosensitivity	30539797	10.1016/j.yexmp.20
positive Regulation: MIR122 --+> chemosensitivity	30539797	10.1016/j.yexmp.20
positive Regulation: MIR122 --+> chemosensitivity	31171380	10.1016/j.prp.2019
positive Regulation: MIR122 --+> chemosensitivity	28807240	10.1016/j.suronc.2
positive Regulation: MIR122 --+> chemosensitivity	32898724	10.1016/j.coph.202
positive Regulation: MIR122 --+> chemosensitivity	32505000	10.1016/j.omtn.202
positive Regulation: MIR122 --+> chemosensitivity		10.1016/j.livres.2
positive Regulation: MIR122 --+> chemosensitivity	27508026	
positive Regulation: MIR122 --+> chemosensitivity	27508026	
positive Regulation: MIR122 --+> chemosensitivity	27508026	
positive Regulation: MIR122 --+> chemosensitivity	27508026	
positive Regulation: MIR122 --+> chemosensitivity	28500493	10.1007/s11427-016
positive Regulation: MIR122 --+> chemosensitivity	30593276	10.1186/s12943-018
positive Regulation: MIR122 --+> chemosensitivity	30940145	10.1186/s12943-019
positive Regulation: MIR122 --+> chemosensitivity	31291956	10.1186/s12964-019
positive Regulation: MIR122 --+> chemosensitivity	31894326	10.3892/ijo.2019.4
positive Regulation: MIR122 --+> chemosensitivity	31795332	10.3390/ijms202360
positive Regulation: MIR122 --+> chemosensitivity	32310043	10.2174/1381612826
positive Regulation: MIR122 --+> chemosensitivity	33155220	10.26355/eurrev_20
positive Regulation: MIR122 --+> chemosensitivity	33743101	10.1007/s12032-021
positive Regulation: MIR122 --+> chemosensitivity	34076140	10.1590/1414-431X2
positive Regulation: MIR122 --+> chemosensitivity	24466275	10.1371/journal.pc
positive Regulation: MIR122 --+> chemosensitivity	26514126	10.1186/s13045-015
positive Regulation: MIR122 --+> chemosensitivity	26514126	10.1186/s13045-015
positive Regulation: MIR122 --+> chemosensitivity		10.1016/j.prp.2019
positive Regulation: MIR122 --+> chemosensitivity		10.1186/s12943-018
positive Regulation: MIR122 --+> chemosensitivity		10.1186/s12943-019
positive Regulation: MIR122 --+> chemosensitivity		10.1186/s12964-019
positive Regulation: MIR122 --+> chemosensitivity		10.3892/ijo.2019.4
positive Regulation: MIR122 --+> chemosensitivity		10.3390/ijms202360
positive Regulation: MIR122 --+> chemosensitivity	34745324	10.1155/2021/32675
positive Regulation: MIR621 --+> chemosensitivity	30266665	10.1016/j.ejphar.2
positive Regulation: MIR621 --+> chemosensitivity	31546023	10.1016/j.canlet.2
positive Regulation: MIR621 --+> chemosensitivity	29278851	10.1016/j.biopha.2
positive Regulation: MIR621 --+> chemosensitivity	29518611	10.1016/j.biopha.2
positive Regulation: MIR621 --+> chemosensitivity	30503969	10.1016/j.ymthe.20
positive Regulation: MIR621 --+> chemosensitivity	26427329	10.1016/j.semcance
positive Regulation: MIR621 --+> chemosensitivity	30807260	10.1089/gtmb.2018.
positive Regulation: MIR621 --+> chemosensitivity	25867061	10.1038/onc.2015.9
positive Regulation: MIR621 --+> chemosensitivity	25867061	10.1038/onc.2015.9
positive Regulation: MIR424 --+> chemosensitivity	32846190	10.1016/j.canlet.2
positive Regulation: MIR424 --+> chemosensitivity	32277626	
positive Regulation: MIR424 --+> chemosensitivity	33555529	10.1007/s11033-021
positive Regulation: MIR424 --+> chemosensitivity	27147225	10.1038/ncomms1140
positive Regulation: MIR424 --+> chemosensitivity	27500472	10.1002/mc.2253610
positive Regulation: MIR424 --+> chemosensitivity	33555529	10.1007/s11033-021
positive Regulation: MIR424 --+> chemosensitivity	33555529	10.1007/s11033-021
positive Regulation: MIR424 --+> chemosensitivity	34440380	10.3390/genes12081
positive Regulation: MIR424 --+> chemosensitivity	35409396	10.3390/ijms230740
positive Regulation: MIR424 --+> chemosensitivity	35409396	10.3390/ijms230740
positive Regulation: MIR424 --+> chemosensitivity	33038905	10.4149/neo_2020_2

positive Regulation: MAML3 --+> chemosensitivity	30061220	10.21873/anticanre
positive Regulation: SREBF1 --+> chemosensitivity	31601152	10.1080/21655979.2
positive Regulation: E2F1 --+> chemosensitivity	14684733	10.1074/jbc.M31368
positive Regulation: E2F1 --+> chemosensitivity	31870703	10.1016/j.bbcan.20
positive Regulation: E2F1 --+> chemosensitivity	16530485	10.1016/j.molmed.2
positive Regulation: E2F1 --+> chemosensitivity	14684733	
positive Regulation: E2F1 --+> chemosensitivity	12838312	
positive Regulation: E2F1 --+> chemosensitivity	11325040	10.1385/CBB:33:2:1
positive Regulation: E2F1 --+> chemosensitivity	11445852	10.3892/ijo.19.2.3
positive Regulation: E2F1 --+> chemosensitivity	12065845	10.1159/000059708
positive Regulation: E2F1 --+> chemosensitivity	34292880	10.18632/aging.203
positive Regulation: E2F1 --+> chemosensitivity		
positive Regulation: INPP4B --+> chemosensitivity	31123408	10.2147/OTT.S19683
positive Regulation: MGAT5 --+> chemosensitivity	30143259	10.1016/j.bbrc.201
positive Regulation: MGAT5 --+> chemosensitivity	30143259	10.1016/j.bbrc.201
positive Regulation: MGAT5 --+> chemosensitivity	30143259	10.1016/j.bbrc.201
positive Regulation: MGAT5 --+> chemosensitivity	30143259	10.1016/j.bbrc.201
positive Regulation: MGAT5 --+> chemosensitivity	30143259	10.1016/j.bbrc.201
positive Regulation: MGAT5 --+> chemosensitivity	25636501	10.1016/j.blre.201
positive Regulation: MGAT5 --+> chemosensitivity	23441047	10.1002/iub.11331C
positive Regulation: MGAT5 --+> chemosensitivity	33376510	10.3892/etm.2020.9
positive Regulation: MGAT5 --+> chemosensitivity	33376510	10.3892/etm.2020.9
positive Regulation: PADI2 --+> chemosensitivity	25240403	10.1186/1471-2407-
positive Regulation: PADI2 --+> chemosensitivity	25240403	10.1186/1471-2407-
positive Regulation: PADI2 --+> chemosensitivity	25240403	10.1186/1471-2407-
positive Regulation: SHQ1 --+> chemosensitivity	32522979	10.1038/s41419-020
positive Regulation: BECN1 --+> chemosensitivity	28242349	10.1016/j.bbcan.20
positive Regulation: BECN1 --+> chemosensitivity	28479384	10.1016/j.gene.201
positive Regulation: BECN1 --+> chemosensitivity	20207475	10.1016/j.canlet.2
positive Regulation: BECN1 --+> chemosensitivity	22771248	10.1016/j.amjoto.2
positive Regulation: BECN1 --+> chemosensitivity	23722017	10.1016/j.prp.2013
positive Regulation: BECN1 --+> chemosensitivity	25523227	10.1016/j.prp.2014
positive Regulation: BECN1 --+> chemosensitivity	27003389	10.1007/s10495-016
positive Regulation: BECN1 --+> chemosensitivity	29207161	10.3892/or.2017.61
positive Regulation: BECN1 --+> chemosensitivity	29322787	10.4149/neo_2018_1
positive Regulation: BECN1 --+> chemosensitivity	32251287	10.1038/s41419-020
positive Regulation: BECN1 --+> chemosensitivity	21350938	10.1007/s10059-011
positive Regulation: BECN1 --+> chemosensitivity	22805532	10.1097/IGC.0b013e
positive Regulation: BECN1 --+> chemosensitivity	22665050	10.1038/onc.2012.1
positive Regulation: BECN1 --+> chemosensitivity	26700674	10.1007/s13277-015
positive Regulation: BECN1 --+> chemosensitivity	20207475	10.1016/j.canlet.2
positive Regulation: BECN1 --+> chemosensitivity	22665050	10.1038/onc.2012.1
positive Regulation: BECN1 --+> chemosensitivity	23225331	10.1007/s13277-012
positive Regulation: BECN1 --+> chemosensitivity	27313746	10.3892/ol.2016.45
positive Regulation: BECN1 --+> chemosensitivity	31274516	10.1097/CAD.000000
positive Regulation: BECN1 --+> chemosensitivity	31966694	
positive Regulation: BECN1 --+> chemosensitivity	31966694	
positive Regulation: BECN1 --+> chemosensitivity	33670664	10.3390/biomedicin
positive Regulation: AQP1 --+> chemosensitivity	32814878	10.1038/s41418-020
positive Regulation: AQP1 --+> chemosensitivity	32814878	10.1038/s41418-020
positive Regulation: AQP1 --+> chemosensitivity	32814878	10.1038/s41418-020
positive Regulation: AQP1 --+> chemosensitivity	32814878	10.1038/s41418-020
positive Regulation: AQP1 --+> chemosensitivity	32814878	10.1038/s41418-020
positive Regulation: AQP1 --+> chemosensitivity	32814878	10.1038/s41418-020

positive Regulation: AQP1 --+> chemosensitivity	32814878	10.1038/s41418-020
positive Regulation: BMP6 --+> chemosensitivity	23674072	10.3892/or.2013.24
positive Regulation: BMP6 --+> chemosensitivity		
positive Regulation: FUT6 --+> chemosensitivity	24232099	10.1038/cddis.2013
positive Regulation: FUT6 --+> chemosensitivity	24232099	10.1038/cddis.2013
positive Regulation: MIR185 --+> chemosensitivity	31128298	10.1016/j.semcance
positive Regulation: MIR185 --+> chemosensitivity	29138830	10.3892/mmr.2017.8
positive Regulation: MIR185 --+> chemosensitivity	30210696	
positive Regulation: MIR185 --+> chemosensitivity	30210696	
positive Regulation: MIR185 --+> chemosensitivity	30210696	
positive Regulation: MIR185 --+> chemosensitivity	32382150	10.1038/s41419-020
positive Regulation: MIR185 --+> chemosensitivity	32633368	10.26355/eurrev_20
positive Regulation: MIR185 --+> chemosensitivity	32588739	10.1080/15384047.2
positive Regulation: MIR185 --+> chemosensitivity	24763054	10.1038/cddis.2014
positive Regulation: MIR185 --+> chemosensitivity	24763054	10.1038/cddis.2014
positive Regulation: MIR185 --+> chemosensitivity	24763054	10.1038/cddis.2014
positive Regulation: MIR185 --+> chemosensitivity	24763054	10.1038/cddis.2014
positive Regulation: MIR185 --+> chemosensitivity	24763054	10.1038/cddis.2014
positive Regulation: MIR185 --+> chemosensitivity	30210696	
positive Regulation: XAF1 --+> chemosensitivity	23685456	10.3892/ijo.2013.1
positive Regulation: LINC01614 --+> chemosensitivity	35083139	10.3389/fonc.2021.
positive Regulation: NKAIN2 --+> chemosensitivity	28381166	10.1177/1010428317
positive Regulation: TMPRSS4 --+> chemosensitivity	34379296	10.1007/s11605-021
positive Regulation: EZR --+> chemosensitivity	23435957	10.1007/s11010-013
positive Regulation: MIR137 --+> chemosensitivity	33892053	10.1016/j.bbcan.20
positive Regulation: MIR137 --+> chemosensitivity	32736290	10.1016/j.omtn.202
positive Regulation: MIR137 --+> chemosensitivity	29348676	10.1038/s41418-017
positive Regulation: MIR137 --+> chemosensitivity	33718112	10.3389/fonc.2020.
positive Regulation: WBSCR22 --+> chemosensitivity	25352209	10.3892/mmr.2014.2
positive Regulation: WBSCR22 --+> chemosensitivity	25352209	10.3892/mmr.2014.2
positive Regulation: WBSCR22 --+> chemosensitivity	25352209	10.3892/mmr.2014.2
positive Regulation: WBSCR22 --+> chemosensitivity	25352209	10.3892/mmr.2014.2
positive Regulation: WBSCR22 --+> chemosensitivity	25352209	10.3892/mmr.2014.2
positive Regulation: WBSCR22 --+> chemosensitivity	25352209	10.3892/mmr.2014.2
positive Regulation: MIR873 --+> chemosensitivity	33865701	10.1016/j.semcdb.2
positive Regulation: MIR873 --+> chemosensitivity	33865701	10.1016/j.semcdb.2
positive Regulation: MIR873 --+> chemosensitivity	31257462	10.3892/mmr.2019.1
positive Regulation: MIR590 --+> chemosensitivity	28922711	10.1016/j.biopha.2
positive Regulation: MIR590 --+> chemosensitivity	30111512	10.1016/j.ebiom.20
positive Regulation: MIR590 --+> chemosensitivity	30111512	10.1016/j.ebiom.20
positive Regulation: MIR590 --+> chemosensitivity	33022677	10.1159/000509811
positive Regulation: MIR590 --+> chemosensitivity	27757042	10.2147/OTT.S11092
positive Regulation: LAMP1 --+> chemosensitivity	27834032	10.1245/s10434-016
positive Regulation: KDM1A --+> chemosensitivity	28381185	10.1177/1010428317
positive Regulation: PPP2R2D --+> chemosensitivity	27074866	10.1186/s13046-016
positive Regulation: LAMTOR5-AS1 --+> chemosensitivity	34862368	10.1038/s41419-021
positive Regulation: MIR29B1 --+> chemosensitivity	33587978	10.1016/j.canlet.2
positive Regulation: MIR29B1 --+> chemosensitivity	28164574	10.7754/Clin.Lab.2
positive Regulation: MIR29B1 --+> chemosensitivity	25174983	10.3892/ijo.2014.2
positive Regulation: MIR29B1 --+> chemosensitivity	25174983	10.3892/ijo.2014.2
positive Regulation: MIR29B1 --+> chemosensitivity	25308719	
positive Regulation: MIR29B1 --+> chemosensitivity	24147037	10.1371/journal.pc
positive Regulation: MIR29B1 --+> chemosensitivity	25784815	10.2147/OTT.S76484
positive Regulation: MIR29B1 --+> chemosensitivity	28164574	10.7754/Clin.Lab.2

positive Regulation: FPGS --+> chemosensitivity	18035049	10.1016/j.bbrc.200
positive Regulation: FPGS --+> chemosensitivity	24045662	10.1038/bjc.2013.5
positive Regulation: FPGS --+> chemosensitivity	15542523	10.1136/gut.2004.0
positive Regulation: FPGS --+> chemosensitivity	18025275	10.1158/1535-7163.
positive Regulation: FPGS --+> chemosensitivity	32256983	10.4162/nrp.2020.1
positive Regulation: CASR --+> chemosensitivity	19038444	10.1016/j.ceca.200
positive Regulation: RASSF1 --+> chemosensitivity	31273338	10.1038/s41388-019
positive Regulation: RASSF1 --+> chemosensitivity	19567146	
positive Regulation: RASSF1 --+> chemosensitivity	19567146	
positive Regulation: RASSF1 --+> chemosensitivity	33442234	10.2147/DDDT.S2692
positive Regulation: RASSF1 --+> chemosensitivity	33442234	10.2147/DDDT.S2692
positive Regulation: UNC5B --+> chemosensitivity	32141538	10.26355/eurrev_20
positive Regulation: ERCC2 --+> chemosensitivity	30109500	10.1007/s00432-018
positive Regulation: ERCC2 --+> chemosensitivity	32377720	10.3892/ijmm.2020.
positive Regulation: ERCC2 --+> chemosensitivity	32377720	10.3892/ijmm.2020.
positive Regulation: ERCC2 --+> chemosensitivity	32377720	10.3892/ijmm.2020.
positive Regulation: ERCC2 --+> chemosensitivity	32377720	10.3892/ijmm.2020.
positive Regulation: ERCC2 --+> chemosensitivity	32377720	10.3892/ijmm.2020.
positive Regulation: ERCC2 --+> chemosensitivity	32377720	10.3892/ijmm.2020.
positive Regulation: ERCC2 --+> chemosensitivity	25571061	10.1109/EMBC.2014.
positive Regulation: ERCC2 --+> chemosensitivity	32377720	10.3892/ijmm.2020.
positive Regulation: ERCC2 --+> chemosensitivity	32377720	10.3892/ijmm.2020.
positive Regulation: CSRP2 --+> chemosensitivity	33324073	10.2147/OTT.S28180
positive Regulation: TP53 --+> chemosensitivity	28755993	10.1016/j.ijpharm.
positive Regulation: TP53 --+> chemosensitivity	29615301	10.1016/j.dld.2018
positive Regulation: TP53 --+> chemosensitivity	21211512	10.1016/j.abb.2010
positive Regulation: TP53 --+> chemosensitivity	15865929	10.1016/j.bbrc.200
positive Regulation: TP53 --+> chemosensitivity	20233581	10.1016/j.bbrc.201
positive Regulation: TP53 --+> chemosensitivity		10.1016/S0006-2952
positive Regulation: TP53 --+> chemosensitivity	12694871	10.1016/S0006-2952
positive Regulation: TP53 --+> chemosensitivity	12948852	10.1016/S0006-2952
positive Regulation: TP53 --+> chemosensitivity	17445779	10.1016/j.bcp.2007
positive Regulation: TP53 --+> chemosensitivity	19028473	10.1016/j.cbi.2008
positive Regulation: TP53 --+> chemosensitivity	11435891	10.1016/S0022-5347
positive Regulation: TP53 --+> chemosensitivity		10.1016/S0022-5347
positive Regulation: TP53 --+> chemosensitivity	10379742	10.1097/00005392-1
positive Regulation: TP53 --+> chemosensitivity	12175703	10.1016/S0024-3205
positive Regulation: TP53 --+> chemosensitivity	12409142	10.1016/S0024-3205
positive Regulation: TP53 --+> chemosensitivity	14761678	10.1016/j.taap.200
positive Regulation: TP53 --+> chemosensitivity	15891997	10.1016/j.humpath.
positive Regulation: TP53 --+> chemosensitivity	12893182	10.1016/S0090-8258
positive Regulation: TP53 --+> chemosensitivity	12893182	10.1016/S0090-8258
positive Regulation: TP53 --+> chemosensitivity	15790436	10.1016/j.ygyno.20
positive Regulation: TP53 --+> chemosensitivity	15790436	10.1016/j.ygyno.20
positive Regulation: TP53 --+> chemosensitivity	16380178	10.1016/j.tibtech.
positive Regulation: TP53 --+> chemosensitivity	22446689	10.1016/j.jhep.201
positive Regulation: TP53 --+> chemosensitivity	11557115	10.1016/S0169-5002
positive Regulation: TP53 --+> chemosensitivity	16307839	10.1016/j.fct.2005
positive Regulation: TP53 --+> chemosensitivity	10737712	10.1016/S0304-3835
positive Regulation: TP53 --+> chemosensitivity	10893449	10.1016/S0304-3835
positive Regulation: TP53 --+> chemosensitivity	12880966	10.1016/S0304-3835
positive Regulation: TP53 --+> chemosensitivity	15327837	10.1016/j.canlet.2
positive Regulation: TP53 --+> chemosensitivity	16023288	10.1016/j.canlet.2
positive Regulation: TP53 --+> chemosensitivity	16919866	10.1016/j.canlet.2

positive Regulation: TP53 --+> chemosensitivity	18657356	10.1016/j.canlet.2
positive Regulation: TP53 --+> chemosensitivity	19467788	10.1016/j.ctrv.200
positive Regulation: TP53 --+> chemosensitivity	21856084	10.1016/j.mehy.201
positive Regulation: TP53 --+> chemosensitivity	22353361	10.1016/j.gene.201
positive Regulation: TP53 --+> chemosensitivity		10.1016/j.tiv.2012
positive Regulation: TP53 --+> chemosensitivity	17875383	10.1016/j.ijom.200
positive Regulation: TP53 --+> chemosensitivity	17875383	10.1016/j.ijom.200
positive Regulation: TP53 --+> chemosensitivity	16337111	10.1016/j.colsurfb
positive Regulation: TP53 --+> chemosensitivity	16713222	10.1016/j.phymed.2
positive Regulation: TP53 --+> chemosensitivity	15691646	10.1016/j.ejca.200
positive Regulation: TP53 --+> chemosensitivity	16146690	10.1016/j.ejca.200
positive Regulation: TP53 --+> chemosensitivity	12850529	10.1016/S1040-8428
positive Regulation: TP53 --+> chemosensitivity	16253824	10.1053/j.semtcvs.
positive Regulation: TP53 --+> chemosensitivity	19508919	10.1016/j.drudis.2
positive Regulation: TP53 --+> chemosensitivity	21195657	10.1016/j.drup.201
positive Regulation: TP53 --+> chemosensitivity	12829013	10.1016/S1471-4914
positive Regulation: TP53 --+> chemosensitivity	12726865	10.1016/S1535-6108
positive Regulation: TP53 --+> chemosensitivity	20832747	10.1016/j.ccr.2010
positive Regulation: TP53 --+> chemosensitivity	17240826	10.1016/j.thorsurg
positive Regulation: TP53 --+> chemosensitivity	23040255	10.1053/j.seminonc
positive Regulation: TP53 --+> chemosensitivity	22445862	10.1016/j.tiv.2012
positive Regulation: TP53 --+> chemosensitivity	23313858	10.1016/j.yexcr.20
positive Regulation: TP53 --+> chemosensitivity	23973262	10.1016/j.canlet.2
positive Regulation: TP53 --+> chemosensitivity	24462821	10.1016/j.canlet.2
positive Regulation: TP53 --+> chemosensitivity	25117446	10.1016/j.bbrc.201
positive Regulation: TP53 --+> chemosensitivity	25220870	10.1016/j.biochi.2
positive Regulation: TP53 --+> chemosensitivity		10.1016/j.toxlet.2
positive Regulation: TP53 --+> chemosensitivity	24681512	10.1016/j.phrs.201
positive Regulation: TP53 --+> chemosensitivity	24681512	10.1016/j.phrs.201
positive Regulation: TP53 --+> chemosensitivity	25448278	10.1016/j.toxlet.2
positive Regulation: TP53 --+> chemosensitivity	21293058	
positive Regulation: TP53 --+> chemosensitivity	21293058	
positive Regulation: TP53 --+> chemosensitivity	11095436	
positive Regulation: TP53 --+> chemosensitivity	11095436	
positive Regulation: TP53 --+> chemosensitivity	9768682	
positive Regulation: TP53 --+> chemosensitivity	9661637	
positive Regulation: TP53 --+> chemosensitivity	20075077	
positive Regulation: TP53 --+> chemosensitivity	19055826	
positive Regulation: TP53 --+> chemosensitivity	17626635	
positive Regulation: TP53 --+> chemosensitivity	17150101	
positive Regulation: TP53 --+> chemosensitivity	17121812	
positive Regulation: TP53 --+> chemosensitivity	16159878	
positive Regulation: TP53 --+> chemosensitivity	16120770	
positive Regulation: TP53 --+> chemosensitivity	15262986	
positive Regulation: TP53 --+> chemosensitivity	12890671	
positive Regulation: TP53 --+> chemosensitivity	12890671	
positive Regulation: TP53 --+> chemosensitivity	12690107	
positive Regulation: TP53 --+> chemosensitivity	12615954	
positive Regulation: TP53 --+> chemosensitivity	12481433	
positive Regulation: TP53 --+> chemosensitivity	12481433	
positive Regulation: TP53 --+> chemosensitivity	18818514	10.4161/cc.7.19.67
positive Regulation: TP53 --+> chemosensitivity	27313779	10.7150/jca.145061
positive Regulation: TP53 --+> chemosensitivity	27484708	10.1080/15384101.2
positive Regulation: TP53 --+> chemosensitivity	32492656	10.18632/aging.103

positive Regulation: TP53 --+> chemosensitivity	27313779	10.7150/jca.145061
positive Regulation: TP53 --+> chemosensitivity	12615954	
positive Regulation: TP53 --+> chemosensitivity	12615954	
positive Regulation: TP53 --+> chemosensitivity	12615954	
positive Regulation: TP53 --+> chemosensitivity	9694807	
positive Regulation: TP53 --+> chemosensitivity	14769944	
positive Regulation: TP53 --+> chemosensitivity	11583579	
positive Regulation: TP53 --+> chemosensitivity	11248087	
positive Regulation: TP53 --+> chemosensitivity	9122197	
positive Regulation: TP53 --+> chemosensitivity	16755297	
positive Regulation: TP53 --+> chemosensitivity	15153941	
positive Regulation: TP53 --+> chemosensitivity	14739942	
positive Regulation: TP53 --+> chemosensitivity	11791171	
positive Regulation: TP53 --+> chemosensitivity	11791171	
positive Regulation: TP53 --+> chemosensitivity	11791171	
positive Regulation: TP53 --+> chemosensitivity	11791171	
positive Regulation: TP53 --+> chemosensitivity	11791171	
positive Regulation: TP53 --+> chemosensitivity	10698490	
positive Regulation: TP53 --+> chemosensitivity	10471039	
positive Regulation: TP53 --+> chemosensitivity	16260623	
positive Regulation: TP53 --+> chemosensitivity	20706634	
positive Regulation: TP53 --+> chemosensitivity	27329169	10.1007/s10549-016
positive Regulation: TP53 --+> chemosensitivity		10.1186/s13046-014
positive Regulation: TP53 --+> chemosensitivity	27484466	10.1186/s12885-016
positive Regulation: TP53 --+> chemosensitivity	27485825	10.3892/mmr.2016.5
positive Regulation: TP53 --+> chemosensitivity	27485825	10.3892/mmr.2016.5
positive Regulation: TP53 --+> chemosensitivity		10.1096/fj.2016012
positive Regulation: TP53 --+> chemosensitivity		10.1096/fj.2016012
positive Regulation: TP53 --+> chemosensitivity	16973289	10.1016/j.bbcan.20
positive Regulation: TP53 --+> chemosensitivity	18064040	
positive Regulation: TP53 --+> chemosensitivity	28112370	10.3892/or.2017.53
positive Regulation: TP53 --+> chemosensitivity	8642047	10.1007/bf01220804
positive Regulation: TP53 --+> chemosensitivity	12615954	
positive Regulation: TP53 --+> chemosensitivity	28467351	10.3390/ijms180509
positive Regulation: TP53 --+> chemosensitivity	22615851	10.1371/journal.pc
positive Regulation: TP53 --+> chemosensitivity	30106452	10.3892/or.2018.66
positive Regulation: TP53 --+> chemosensitivity	30106452	10.3892/or.2018.66
positive Regulation: TP53 --+> chemosensitivity		10.1186/s12935-018
positive Regulation: TP53 --+> chemosensitivity	30387834	10.3892/ijo.2018.4
positive Regulation: TP53 --+> chemosensitivity		10.4149/neo_2018_1
positive Regulation: TP53 --+> chemosensitivity	31108984	10.3390/ijms201024
positive Regulation: TP53 --+> chemosensitivity		10.1186/s12935-019
positive Regulation: TP53 --+> chemosensitivity	32377739	10.3892/mmr.2020.1
positive Regulation: TP53 --+> chemosensitivity		10.1042/BSR2019148
positive Regulation: TP53 --+> chemosensitivity		10.1042/BSR2019148
positive Regulation: TP53 --+> chemosensitivity	33184290	10.1038/s41419-020
positive Regulation: TP53 --+> chemosensitivity	33184290	10.1038/s41419-020
positive Regulation: TP53 --+> chemosensitivity	33256191	10.3390/ijms212389
positive Regulation: TP53 --+> chemosensitivity		10.1016/j.jbior.20
positive Regulation: TP53 --+> chemosensitivity	32876741	10.1007/s00508-020
positive Regulation: TP53 --+> chemosensitivity	33785447	10.1016/j.semcance
positive Regulation: TP53 --+> chemosensitivity		10.1016/j.omtn.202
positive Regulation: TP53 --+> chemosensitivity		10.1016/j.jtcms.20
positive Regulation: WWTR1 --+> chemosensitivity	32898567	10.1016/j.abb.2020

positive Regulation: WWTR1 --+> chemosensitivity	32898567	10.1016/j.abb.2020
positive Regulation: WWTR1 --+> chemosensitivity	28737828	10.3892/or.2017.58
positive Regulation: WWTR1 --+> chemosensitivity	28749195	10.1080/15384101.2
positive Regulation: WWTR1 --+> chemosensitivity	32898567	10.1016/j.abb.2020
positive Regulation: WWTR1 --+> chemosensitivity	35604626	10.1007/s11033-022
positive Regulation: TLE3 --+> chemosensitivity	33571907	10.1016/j.bbrc.202
positive Regulation: MIR455 --+> chemosensitivity	35582195	10.1515/med-2022-0
positive Regulation: MIR1278 --+> chemosensitivity	32407879	10.1016/j.mcp.2020
positive Regulation: CYC1 --+> chemosensitivity	31149728	10.14670/HH-18-130
positive Regulation: CYC1 --+> chemosensitivity	31149728	10.14670/HH-18-130
positive Regulation: CYC1 --+> chemosensitivity	31149728	10.14670/HH-18-130
positive Regulation: CTSS --+> chemosensitivity	25755832	
positive Regulation: TNFSF10 --+> chemosensitivity	21514380	10.1016/j.bbadis.2
positive Regulation: TNFSF10 --+> chemosensitivity	11472983	
positive Regulation: TNFSF10 --+> chemosensitivity	11803469	
positive Regulation: TNFSF10 --+> chemosensitivity	31119375	10.1007/s00280-019
positive Regulation: TNFSF10 --+> chemosensitivity	22372904	10.5858/arpa.2011-
positive Regulation: TNFSF10 --+> chemosensitivity	17283156	10.1158/0008-5472.
positive Regulation: TNFSF10 --+> chemosensitivity	34048180	10.31557/APJCP.202
positive Regulation: TNFSF10 --+> chemosensitivity		
positive Regulation: IKZF3 --+> chemosensitivity	25524659	10.3892/or.2014.36
positive Regulation: FHIT --+> chemosensitivity	27166255	10.18632/oncotarge
positive Regulation: FHIT --+> chemosensitivity	34901149	10.3389/fmolb.2021
positive Regulation: FBP1 --+> chemosensitivity	28990097	10.3892/mmr.2017.7
positive Regulation: FBP1 --+> chemosensitivity	28990097	10.3892/mmr.2017.7
positive Regulation: FBP1 --+> chemosensitivity	28990097	10.3892/mmr.2017.7
positive Regulation: EPHA3 --+> chemosensitivity	27101199	10.1007/s13277-016
positive Regulation: AMBN --+> chemosensitivity	28054649	10.1038/srep401871
positive Regulation: MIR489 --+> chemosensitivity	30551491	10.1016/j.biopha.2
positive Regulation: MIR489 --+> chemosensitivity	31378911	10.26355/eurrev_20
positive Regulation: MIR489 --+> chemosensitivity	27171498	10.1002/path.47431
positive Regulation: NRBP2 --+> chemosensitivity	27634758	10.1158/0008-5472.
positive Regulation: NRBP2 --+> chemosensitivity	35648115	10.1007/s11010-022
positive Regulation: PTPN6 --+> chemosensitivity	33615510	10.1002/cbf.360410
positive Regulation: PTPN6 --+> chemosensitivity	33615510	10.1002/cbf.360410
positive Regulation: MIR506 --+> chemosensitivity	30558723	10.1016/j.ajpath.2
positive Regulation: MIR506 --+> chemosensitivity	32681832	10.1016/j.abb.2020
positive Regulation: MIR506 --+> chemosensitivity	30553455	10.1016/j.bbrc.201
positive Regulation: MIR506 --+> chemosensitivity	33711308	10.1016/j.ejphar.2
positive Regulation: MIR506 --+> chemosensitivity	28254409	10.1016/j.canlet.2
positive Regulation: MIR506 --+> chemosensitivity	27065335	10.1038/onc.2016.9
positive Regulation: MIR506 --+> chemosensitivity	27065335	10.1038/onc.2016.9
positive Regulation: MIR506 --+> chemosensitivity	27065335	10.1038/onc.2016.9
positive Regulation: MIR506 --+> chemosensitivity	27065335	10.1038/onc.2016.9
positive Regulation: MIR506 --+> chemosensitivity	27065335	10.1038/onc.2016.9
positive Regulation: MIR506 --+> chemosensitivity	29788724	10.4149/neo_2018_1
positive Regulation: MIR506 --+> chemosensitivity	25793370	10.1371/journal.pc
positive Regulation: MIR506 --+> chemosensitivity	26935526	10.3892/or.2016.46
positive Regulation: MIR506 --+> chemosensitivity	24608427	10.1038/onc.2014.9
positive Regulation: MIR506 --+> chemosensitivity	27065335	10.1038/onc.2016.9
positive Regulation: MIR506 --+> chemosensitivity	35715750	10.1186/s11658-022
positive Regulation: SCN5A --+> chemosensitivity	33338532	10.1016/j.canlet.2
positive Regulation: SCN5A --+> chemosensitivity	33338532	10.1016/j.canlet.2
positive Regulation: SCN5A --+> chemosensitivity	34091433	10.1016/j.canlet.2

positive Regulation: SMAD3 --+> chemosensitivity	27110775	10.3390/ijms170406
positive Regulation: MIR125B1 --+> chemosensitivity	28122310	10.1016/j.biopha.2
positive Regulation: MIR125B1 --+> chemosensitivity	32243895	10.1016/j.ygeno.20
positive Regulation: MIR125B1 --+> chemosensitivity	32461153	10.1016/j.semcance
positive Regulation: MIR125B1 --+> chemosensitivity	25042866	10.1016/j.canlet.2
positive Regulation: MIR125B1 --+> chemosensitivity	28695772	10.1177/1010428317
positive Regulation: MIR125B1 --+> chemosensitivity	28628119	10.1038/onc.2017.1
positive Regulation: MIR125B1 --+> chemosensitivity	30896792	10.3892/ijo.2019.4
positive Regulation: MIR125B1 --+> chemosensitivity	33348804	10.3390/ijms212496
positive Regulation: MIR125B1 --+> chemosensitivity	23497288	10.1186/1475-2867-
positive Regulation: MIR125B1 --+> chemosensitivity	23497288	10.1186/1475-2867-
positive Regulation: MIR125B1 --+> chemosensitivity	23497288	10.1186/1475-2867-
positive Regulation: MIR125B1 --+> chemosensitivity	25532106	10.1371/journal.pc
positive Regulation: MIR125B1 --+> chemosensitivity	25756509	10.1080/15384047.2
positive Regulation: MIR125B1 --+> chemosensitivity	26596831	10.1007/s13277-015
positive Regulation: MIR125B1 --+> chemosensitivity	24643683	10.1007/s13277-014
positive Regulation: MIR125B1 --+> chemosensitivity	24846940	10.3724/sp.j.1005.
positive Regulation: MIR125B1 --+> chemosensitivity	26744308	10.1177/1533034615
positive Regulation: MIR125B1 --+> chemosensitivity	34888842	10.1007/978-3-030-
positive Regulation: MIR125B1 --+> chemosensitivity	35730423	10.5603/FHC.a2022.
positive Regulation: CACNA2D3 --+> chemosensitivity	31746409	10.3892/or.2019.73
positive Regulation: FASN --+> chemosensitivity	28339092	10.3892/or.2017.55
positive Regulation: FASN --+> chemosensitivity	31043565	10.1073/pnas.18194
positive Regulation: FASN --+> chemosensitivity	34131808	10.1007/s00018-021
positive Regulation: FASN --+> chemosensitivity	26484401	10.1667/RR14173.1
positive Regulation: FASN --+> chemosensitivity	33752312	10.3760/cma.j.cn11
positive Regulation: FASN --+> chemosensitivity		10.3390/ijms222111
positive Regulation: MICA --+> chemosensitivity	32724354	10.3892/ol.2020.11
positive Regulation: ATF3 --+> chemosensitivity	26961878	10.1074/jbc.M115.6
positive Regulation: ATF3 --+> chemosensitivity	31273338	10.1038/s41388-019
positive Regulation: ATF3 --+> chemosensitivity	31273338	10.1038/s41388-019
positive Regulation: ATF3 --+> chemosensitivity	31273338	10.1038/s41388-019
positive Regulation: ATF3 --+> chemosensitivity	31273338	10.1038/s41388-019
positive Regulation: ATF3 --+> chemosensitivity	35129760	10.1007/s11262-022
positive Regulation: SGK1 --+> chemosensitivity	28504714	10.1038/onc.2017.1
positive Regulation: KCNH2 --+> chemosensitivity	22020779	10.3892/or.2011.15
positive Regulation: KCNH2 --+> chemosensitivity	22020779	10.3892/or.2011.15
positive Regulation: KCNH2 --+> chemosensitivity	22020779	10.3892/or.2011.15
positive Regulation: MIR24-1 --+> chemosensitivity	32089062	10.1080/15384047.2
positive Regulation: MIR24-1 --+> chemosensitivity	31548657	10.1038/s41417-019
positive Regulation: MIR24-1 --+> chemosensitivity	27513190	10.2217/fon-2016-0
positive Regulation: MIR24-1 --+> chemosensitivity	31646794	
positive Regulation: MIR24-1 --+> chemosensitivity	31646794	
positive Regulation: MIR24-1 --+> chemosensitivity	32521898	
positive Regulation: MIR24-1 --+> chemosensitivity	32521898	
positive Regulation: MIR24-1 --+> chemosensitivity	35397087	10.1007/s11033-022
positive Regulation: DOP1B --+> chemosensitivity	34781999	10.1186/s13046-021
positive Regulation: DOP1B --+> chemosensitivity	34781999	10.1186/s13046-021
positive Regulation: DOP1B --+> chemosensitivity	35164815	10.1186/s13046-022
positive Regulation: DOP1B --+> chemosensitivity	35164815	10.1186/s13046-022
positive Regulation: PLK2 --+> chemosensitivity	22138223	10.1016/j.biocel.2
positive Regulation: PLK2 --+> chemosensitivity	33176854	10.1186/s13046-020
positive Regulation: PLK2 --+> chemosensitivity	33176854	10.1186/s13046-020
positive Regulation: PLK2 --+> chemosensitivity	33176854	10.1186/s13046-020

positive Regulation: PLK2 --+> chemosensitivity	33176854	10.1186/s13046-020
positive Regulation: PLK2 --+> chemosensitivity	33176854	10.1186/s13046-020
positive Regulation: PLK2 --+> chemosensitivity	33176854	10.1186/s13046-020
positive Regulation: PLK2 --+> chemosensitivity	21402713	10.1158/0008-5472.
positive Regulation: MIR330 --+> chemosensitivity	28521444	10.3892/ol.2017.58
positive Regulation: MIR330 --+> chemosensitivity	28521444	10.3892/ol.2017.58
positive Regulation: MIR29C --+> chemosensitivity	31047896	10.1016/j.lfs.2019
positive Regulation: MIR29C --+> chemosensitivity	29355660	10.1016/j.canlet.2
positive Regulation: MIR29C --+> chemosensitivity	33989708	10.1016/j.canlet.2
positive Regulation: MIR29C --+> chemosensitivity	33035835	10.1016/j.biopha.2
positive Regulation: MIR29C --+> chemosensitivity	29807360	10.1159/000490027
positive Regulation: MIR29C --+> chemosensitivity	29807360	10.1159/000490027
positive Regulation: MIR29C --+> chemosensitivity	29807360	10.1159/000490027
positive Regulation: MIR29C --+> chemosensitivity	29807360	10.1159/000490027
positive Regulation: MIR29C --+> chemosensitivity	29807360	10.1159/000490027
positive Regulation: MIR29C --+> chemosensitivity	29807360	10.1159/000490027
positive Regulation: MIR29C --+> chemosensitivity	31753063	10.3727/096504019X
positive Regulation: MIR29C --+> chemosensitivity	31753063	10.3727/096504019X
positive Regulation: MIR29C --+> chemosensitivity	29807360	10.1159/0004900271
positive Regulation: MIR29C --+> chemosensitivity	31753063	10.3727/096504019X
positive Regulation: MIR770 --+> chemosensitivity	28922711	10.1016/j.biopha.2
positive Regulation: MIR770 --+> chemosensitivity	29323124	10.1038/s41419-017
positive Regulation: MIR770 --+> chemosensitivity	29323124	10.1038/s41419-017
positive Regulation: MIR770 --+> chemosensitivity	29323124	10.1038/s41419-017
positive Regulation: MIR770 --+> chemosensitivity	30524203	10.1186/s12935-018
positive Regulation: MIR770 --+> chemosensitivity	33630753	10.1530/EJE-20-117
positive Regulation: MIR770 --+> chemosensitivity	27449101	10.18632/oncotarge
positive Regulation: STK11 --+> chemosensitivity	29257268	10.3892/mmr.2017.8
positive Regulation: STK11 --+> chemosensitivity	29257268	10.3892/mmr.2017.8
positive Regulation: STK11 --+> chemosensitivity	29257268	10.3892/mmr.2017.8
positive Regulation: IL1A --+> chemosensitivity	32760201	10.7150/ijbs.46651
positive Regulation: RYBP --+> chemosensitivity	27060496	10.1016/j.cellsig.
positive Regulation: NEK6 --+> chemosensitivity	30153958	10.1016/j.prp.2018
positive Regulation: UNC5D --+> chemosensitivity	24518784	10.1016/j.juro.201
positive Regulation: CD82 --+> chemosensitivity	32203165	10.1038/s41388-020
positive Regulation: ARID3A --+> chemosensitivity	35257428	10.1002/cbin.11789
positive Regulation: ARID3A --+> chemosensitivity	35257428	10.1002/cbin.11789
positive Regulation: ARID3A --+> chemosensitivity	35257428	10.1002/cbin.11789
positive Regulation: MIR497 --+> chemosensitivity	28705657	10.1016/j.freeradb
positive Regulation: MIR497 --+> chemosensitivity	28820062	10.2174/0929866524
positive Regulation: MIR497 --+> chemosensitivity	30535471	10.3892/ijmm.2018.
positive Regulation: MIR497 --+> chemosensitivity	30535471	10.3892/ijmm.2018.
positive Regulation: MIR497 --+> chemosensitivity	24667580	10.1371/journal.pc
positive Regulation: MIR497 --+> chemosensitivity	24667580	10.1371/journal.pc
positive Regulation: MIR497 --+> chemosensitivity	24667580	10.1371/journal.pc
positive Regulation: MIR497 --+> chemosensitivity	26673620	10.18632/oncotarge
positive Regulation: MIR497 --+> chemosensitivity	26673620	10.18632/oncotarge
positive Regulation: MIR497 --+> chemosensitivity	27904781	
positive Regulation: MIR497 --+> chemosensitivity	27904781	
positive Regulation: MIR497 --+> chemosensitivity	33015042	10.3389/fcell.2020
positive Regulation: MIR497 --+> chemosensitivity	33179318	10.1111/cas.147331
positive Regulation: MIR497 --+> chemosensitivity	34440380	10.3390/genes12081
positive Regulation: MIR497 --+> chemosensitivity	35116999	10.21037/tcr.2019.
positive Regulation: MIR497 --+> chemosensitivity		10.1016/j.semcance

positive Regulation: OSM --+> chemosensitivity	22469558	10.1016/j.ctrv.201
positive Regulation: OSM --+> chemosensitivity	23358473	10.4161/cbt.23622
positive Regulation: DKK1 --+> chemosensitivity	26799283	10.18632/oncotarge
positive Regulation: CDKN2D --+> chemosensitivity	22762204	10.1042/BSR2011012
positive Regulation: MIR107 --+> chemosensitivity	27498977	10.3892/ijo.2016.3
positive Regulation: MIR107 --+> chemosensitivity	27498977	10.3892/ijo.2016.3
positive Regulation: MIR107 --+> chemosensitivity	33901009	10.18632/aging.202
positive Regulation: MIR107 --+> chemosensitivity	33901009	10.18632/aging.202
positive Regulation: MIR107 --+> chemosensitivity	33901009	10.18632/aging.202
positive Regulation: MIR107 --+> chemosensitivity	33901009	10.18632/aging.202
positive Regulation: MIR107 --+> chemosensitivity	33901009	10.18632/aging.202
positive Regulation: MIR107 --+> chemosensitivity	25400821	
positive Regulation: MIR107 --+> chemosensitivity	28979809	
positive Regulation: MIR107 --+> chemosensitivity	33901009	10.18632/aging.202
positive Regulation: FGF10 --+> chemosensitivity	24865969	10.1128/MCB.00871-
positive Regulation: FGF10 --+> chemosensitivity		10.1128/MCB.00871-
positive Regulation: MIR127 --+> chemosensitivity	27590853	10.1016/j.biocel.2
positive Regulation: MIR127 --+> chemosensitivity	32742197	10.1186/s12935-020
positive Regulation: MIR127 --+> chemosensitivity	32742197	10.1186/s12935-020
positive Regulation: ESR2 --+> chemosensitivity	29669750	10.1124/pr.117.014
positive Regulation: ESR2 --+> chemosensitivity	31297554	10.1007/s00280-019
positive Regulation: ESR2 --+> chemosensitivity	22398780	10.1007/s00432-011
positive Regulation: ESR2 --+> chemosensitivity	24339738	10.1593/neo.131184
positive Regulation: ESR2 --+> chemosensitivity	24339738	10.1593/neo.131184
positive Regulation: ESR2 --+> chemosensitivity	29854295	10.18632/oncotarge
positive Regulation: MIRLET7B --+> chemosensitivity	30857828	10.1016/j.bcp.2019
positive Regulation: MIRLET7B --+> chemosensitivity	23806108	10.1186/1756-9966-
positive Regulation: MIRLET7B --+> chemosensitivity	25951903	10.1186/s12957-015
positive Regulation: CASC2 --+> chemosensitivity	28495512	10.1016/j.abb.2017
positive Regulation: CASC2 --+> chemosensitivity	28495512	10.1016/j.abb.2017
positive Regulation: CASC2 --+> chemosensitivity	28495512	10.1016/j.abb.2017
positive Regulation: CASC2 --+> chemosensitivity	29373811	10.1016/j.abb.2018
positive Regulation: CASC2 --+> chemosensitivity	32271431	10.26355/eurrev_20
positive Regulation: CASC2 --+> chemosensitivity	32787433	10.4149/neo_2020_1
positive Regulation: CASC2 --+> chemosensitivity	32787433	10.4149/neo_2020_1
positive Regulation: CASC2 --+> chemosensitivity	32787433	10.4149/neo_2020_1
positive Regulation: CASC2 --+> chemosensitivity	32787433	10.4149/neo_2020_1
positive Regulation: CASC2 --+> chemosensitivity	34229588	10.2174/1871520621
positive Regulation: SDHAP1 --+> chemosensitivity	32211849	10.1093/jb/mvaa036
positive Regulation: MIR15A --+> chemosensitivity	30317163	10.1016/j.omtn.201
positive Regulation: MIR15A --+> chemosensitivity	25945419	10.1080/15384047.2
positive Regulation: MIR15A --+> chemosensitivity	25945419	10.1080/15384047.2
positive Regulation: MIR15A --+> chemosensitivity	29467857	10.3892/etm.2017.5
positive Regulation: MIR15A --+> chemosensitivity	29467857	10.3892/etm.2017.5
positive Regulation: MIR15A --+> chemosensitivity	34785950	10.2147/CMAR.S3338
positive Regulation: SNORD3A --+> chemosensitivity	32382150	10.1038/s41419-020
positive Regulation: SNORD3A --+> chemosensitivity	32382150	10.1038/s41419-020
positive Regulation: SNORD3A --+> chemosensitivity	32382150	10.1038/s41419-020
positive Regulation: SNORD3A --+> chemosensitivity	32382150	10.1038/s41419-020
positive Regulation: SNORD3A --+> chemosensitivity	32382150	10.1038/s41419-020
positive Regulation: SNORD3A --+> chemosensitivity	32382150	10.1038/s41419-020
positive Regulation: SNORD3A --+> chemosensitivity	32382150	10.1038/s41419-020
positive Regulation: SNORD3A --+> chemosensitivity	32382150	10.1038/s41419-020
positive Regulation: SLX4 --+> chemosensitivity	27284361	10.3892/ol.2016.44

positive Regulation: TSC22D1 --+> chemosensitivity		10.1016/S0006-291X
positive Regulation: TSC22D1 --+> chemosensitivity	10854535	10.3892/or.7.4.737
positive Regulation: TSC22D1 --+> chemosensitivity	15379637	10.2174/1568009043
positive Regulation: MIR451A --+> chemosensitivity	29627370	10.1016/j.addr.201
positive Regulation: MIR451A --+> chemosensitivity	31708373	10.1016/j.prp.2019
positive Regulation: MIR451A --+> chemosensitivity	33926792	10.1016/j.semcdb.2
positive Regulation: MIR451A --+> chemosensitivity	31147293	10.1016/j.ebiom.20
positive Regulation: MIR451A --+> chemosensitivity	21329503	
positive Regulation: MIR451A --+> chemosensitivity	21329503	
positive Regulation: MIR451A --+> chemosensitivity	21329503	
positive Regulation: MIR451A --+> chemosensitivity	21329503	
positive Regulation: MIR451A --+> chemosensitivity	21329503	
positive Regulation: MIR451A --+> chemosensitivity	27573896	10.1007/978-3-319-
positive Regulation: MIR451A --+> chemosensitivity	27686452	10.1007/s11010-016
positive Regulation: MIR451A --+> chemosensitivity	34257614	10.3389/pore.2021.
positive Regulation: MIR451A --+> chemosensitivity	21329503	10.1186/1756-9966-
positive Regulation: MIR451A --+> chemosensitivity	21329503	10.1186/1756-9966-
positive Regulation: MIR451A --+> chemosensitivity	21329503	10.1186/1756-9966-
positive Regulation: MIR451A --+> chemosensitivity	21329503	10.1186/1756-9966-
positive Regulation: MIR451A --+> chemosensitivity	21329503	10.1186/1756-9966-
positive Regulation: MIR451A --+> chemosensitivity	24788655	10.1371/journal.pc
positive Regulation: MIR451A --+> chemosensitivity	25135964	10.1098/rstb.2013.
positive Regulation: MIR451A --+> chemosensitivity	25322930	10.3892/or.2014.35
positive Regulation: MIR451A --+> chemosensitivity	35126514	10.1155/2022/25210
positive Regulation: MIR451A --+> chemosensitivity	34993725	10.1007/s11033-021
positive Regulation: MIR451A --+> chemosensitivity	35031927	10.1007/s11033-021
positive Regulation: MIR512-1 --+> chemosensitivity	31777256	10.4149/neo_2019_1
positive Regulation: MIR512-1 --+> chemosensitivity	31777256	10.4149/neo_2019_1
positive Regulation: MIR512-1 --+> chemosensitivity	31777256	10.4149/neo_2019_1
positive Regulation: MIR512-1 --+> chemosensitivity	31777256	10.4149/neo_2019_1
positive Regulation: MIR512-1 --+> chemosensitivity	31777256	10.4149/neo_2019_1
positive Regulation: CREBZF --+> chemosensitivity	22983008	10.4161/cc.22133
positive Regulation: MIR34C --+> chemosensitivity	26975503	10.1186/s12885-016
positive Regulation: MIR34C --+> chemosensitivity	32228202	10.1177/0300060520
positive Regulation: MIR34C --+> chemosensitivity	32228202	10.1177/0300060520
positive Regulation: MIR34C --+> chemosensitivity	32228202	10.1177/0300060520
positive Regulation: MIR34C --+> chemosensitivity	32228202	10.1177/0300060520
positive Regulation: MIR34C --+> chemosensitivity	32228202	10.1177/0300060520
positive Regulation: MIR34C --+> chemosensitivity	32228202	10.1177/0300060520
positive Regulation: MIR34C --+> chemosensitivity	32700743	10.1042/BSR2019406
positive Regulation: MIR34C --+> chemosensitivity	24802328	10.1007/s12032-014
positive Regulation: MIR34C --+> chemosensitivity	23423488	10.1007/s00280-013
positive Regulation: HOXD10 --+> chemosensitivity	34790580	10.3389/fonc.2021.
positive Regulation: SIRT3 --+> chemosensitivity	29191657	10.1016/j.bbrc.201
positive Regulation: SIRT3 --+> chemosensitivity	32243871	10.1016/j.ejphar.2
positive Regulation: SIRT3 --+> chemosensitivity	32229158	10.1016/j.gene.202
positive Regulation: SIRT3 --+> chemosensitivity	32229158	10.1016/j.gene.202
positive Regulation: SIRT3 --+> chemosensitivity	32229158	10.1016/j.gene.202
positive Regulation: SIRT3 --+> chemosensitivity	32229158	10.1016/j.gene.202
positive Regulation: SIRT3 --+> chemosensitivity	32229158	10.1016/j.gene.202
positive Regulation: SIRT3 --+> chemosensitivity		10.3390/cimb440100
positive Regulation: SIRT3 --+> chemosensitivity	35159089	10.3390/cancers140
positive Regulation: SHH --+> chemosensitivity	30053447	10.1016/j.lfs.2018
positive Regulation: KCNQ1 --+> chemosensitivity	31454677	10.1016/j.omtn.201

positive Regulation: KCNQ1 --+> chemosensitivity	31454677	10.1016/j.omtn.201
positive Regulation: TOP2A --+> chemosensitivity	25710584	10.1097/PAI.000000
positive Regulation: MIR199B --+> chemosensitivity	30325582	10.1002/iub.188910
positive Regulation: EHMT2 --+> chemosensitivity	35640269	10.1093/carcin/bga
positive Regulation: MIR9-1 --+> chemosensitivity	32497683	10.1016/j.semcance
positive Regulation: MIR9-1 --+> chemosensitivity	28260112	10.3892/or.2017.54
positive Regulation: MIR9-1 --+> chemosensitivity	28260112	10.3892/or.2017.54
positive Regulation: MIR9-1 --+> chemosensitivity	28260112	10.3892/or.2017.54
positive Regulation: MIR9-1 --+> chemosensitivity	28260112	10.3892/or.2017.54
positive Regulation: MIR9-1 --+> chemosensitivity	28260112	10.3892/or.2017.54
positive Regulation: MIR9-1 --+> chemosensitivity	28260112	10.3892/or.2017.54
positive Regulation: MIR9-1 --+> chemosensitivity	28358371	10.1038/cddis.2017
positive Regulation: MIR9-1 --+> chemosensitivity	32071552	10.7150/ijbs.32460
positive Regulation: MIR9-1 --+> chemosensitivity	32071552	10.7150/ijbs.32460
positive Regulation: MIR9-1 --+> chemosensitivity	26152689	10.1186/s12885-015
positive Regulation: MIR9-1 --+> chemosensitivity	26152689	10.1186/s12885-015
positive Regulation: MIR9-1 --+> chemosensitivity	25571061	10.1109/EMBC.2014.
positive Regulation: MIR9-1 --+> chemosensitivity	34185245	10.1007/s11010-021
positive Regulation: BRIP1 --+> chemosensitivity	26680099	10.1016/j.gene.201
positive Regulation: BRIP1 --+> chemosensitivity	33790078	10.1248/CPB.C20-00
positive Regulation: BRIP1 --+> chemosensitivity	26680099	10.1016/j.gene.201
positive Regulation: DPP4 --+> chemosensitivity	22261805	10.1158/1078-0432.
positive Regulation: MIR202 --+> chemosensitivity	29559564	10.1042/BSR2017138
positive Regulation: ECM1 --+> chemosensitivity	31319137	10.1016/j.canlet.2
positive Regulation: ECM1 --+> chemosensitivity	31319137	10.1016/j.canlet.2
positive Regulation: ECM1 --+> chemosensitivity	31319137	10.1016/j.canlet.2
positive Regulation: ECM1 --+> chemosensitivity	31319137	10.1016/j.canlet.2
positive Regulation: CD99 --+> chemosensitivity		10.1016/j.clml.201
positive Regulation: MIR1182 --+> chemosensitivity	26772886	10.1016/j.bbrc.201
positive Regulation: MIR1182 --+> chemosensitivity	26772886	10.1016/j.bbrc.201
positive Regulation: MIR1182 --+> chemosensitivity	26772886	10.1016/j.bbrc.201
positive Regulation: MIR1182 --+> chemosensitivity	26772886	10.1016/j.bbrc.201
positive Regulation: MIR1182 --+> chemosensitivity	26772886	10.1016/j.bbrc.201
positive Regulation: MIR1182 --+> chemosensitivity	26772886	10.1016/j.bbrc.201
positive Regulation: MIR1182 --+> chemosensitivity	29526163	10.1186/s12929-018
positive Regulation: MIR1182 --+> chemosensitivity	26772886	10.1016/j.bbrc.201
positive Regulation: MIR1182 --+> chemosensitivity	26772886	10.1016/j.bbrc.201
positive Regulation: NOD1 --+> chemosensitivity	31872284	10.1007/s00109-019
positive Regulation: CYLD --+> chemosensitivity	27448305	10.1007/s13277-016
positive Regulation: CYLD --+> chemosensitivity	27448305	10.1007/s13277-016
positive Regulation: CYLD --+> chemosensitivity	27448305	10.1007/s13277-016
positive Regulation: CYLD --+> chemosensitivity	27448305	10.1007/s13277-016
positive Regulation: CYLD --+> chemosensitivity	27448305	10.1007/s13277-016
positive Regulation: CYLD --+> chemosensitivity	27448305	10.1007/s13277-016
positive Regulation: CYLD --+> chemosensitivity	27448305	10.1007/s13277-016
positive Regulation: OLA1 --+> chemosensitivity	32528278	10.3389/fphar.2020
positive Regulation: PIK3R3 --+> chemosensitivity	29370570	10.1080/15384047.2
positive Regulation: PIK3R3 --+> chemosensitivity	29370570	10.1080/15384047.2
positive Regulation: PIK3R3 --+> chemosensitivity	29370570	10.1080/15384047.2
positive Regulation: PIK3R3 --+> chemosensitivity	29370570	10.1080/15384047.2
positive Regulation: PIK3R3 --+> chemosensitivity	29370570	10.1080/15384047.2
positive Regulation: MIR217 --+> chemosensitivity	29401684	10.3390/ijms190204
positive Regulation: MIR217 --+> chemosensitivity	28599501	10.3892/ol.2017.60
positive Regulation: MIR217 --+> chemosensitivity	30774364	10.2147/OTT.S17661

positive Regulation: MIR217 --+> chemosensitivity	34510251	10.1007/s00280-021
positive Regulation: MIR217 --+> chemosensitivity	35457012	10.3390/ijms230841
positive Regulation: MIR217 --+> chemosensitivity	35628648	10.3390/ijms231058
positive Regulation: MIR221 --+> chemosensitivity	29627370	10.1016/j.addr.201
positive Regulation: MIR221 --+> chemosensitivity	32961483	10.1016/j.neo.2020
positive Regulation: MIR221 --+> chemosensitivity		10.1016/j.biopha.2
positive Regulation: MIR221 --+> chemosensitivity	26422796	10.1016/j.biopha.2
positive Regulation: MIR221 --+> chemosensitivity	26796268	10.1016/j.biopha.2
positive Regulation: MIR221 --+> chemosensitivity	28350128	10.3892/ijo.2017.3
positive Regulation: MIR221 --+> chemosensitivity	28887606	10.1007/s10616-017
positive Regulation: MIR221 --+> chemosensitivity		10.1042/BSR2019019
positive Regulation: MIR221 --+> chemosensitivity		10.1042/BSR2019019
positive Regulation: MIR221 --+> chemosensitivity	24899890	10.1155/2014/38656
positive Regulation: MIR221 --+> chemosensitivity	25308719	
positive Regulation: MIR221 --+> chemosensitivity	24147037	10.1371/journal.pc
positive Regulation: MIR221 --+> chemosensitivity	27501171	10.1097/SLA.000000
positive Regulation: MIR221 --+> chemosensitivity	32256084	10.2147/OTT.S23295
positive Regulation: MIR221 --+> chemosensitivity		
positive Regulation: MIR221 --+> chemosensitivity		10.1016/j.apsb.202
positive Regulation: DRD1 --+> chemosensitivity	35582277	10.20517/cdr.2019.
positive Regulation: LGALS9 --+> chemosensitivity	26717877	10.3892/or.2015.44
positive Regulation: CBLB --+> chemosensitivity	19508871	10.1016/j.febslet.
positive Regulation: CBLB --+> chemosensitivity	19508871	10.1016/j.febslet.
positive Regulation: CBLB --+> chemosensitivity	29416005	10.1038/s41419-017
positive Regulation: CBLB --+> chemosensitivity	24351824	10.3390/ijms141224
positive Regulation: CBLB --+> chemosensitivity	24351824	10.3390/ijms141224
positive Regulation: CBLB --+> chemosensitivity	24351824	10.3390/ijms141224
positive Regulation: CBLB --+> chemosensitivity	24351824	10.3390/ijms141224
positive Regulation: SFRP4 --+> chemosensitivity	28534940	10.3892/ijo.2017.4
positive Regulation: SFRP4 --+> chemosensitivity	23039795	10.1186/1471-2121-
positive Regulation: SFRP4 --+> chemosensitivity	23039795	10.1186/1471-2121-
positive Regulation: C2orf92 --+> chemosensitivity	31865363	10.12659/MSM.91682
positive Regulation: C2orf92 --+> chemosensitivity	31865363	10.12659/MSM.91682
positive Regulation: C2orf92 --+> chemosensitivity	31865363	10.12659/MSM.91682
positive Regulation: C2orf92 --+> chemosensitivity	31865363	10.12659/MSM.91682
positive Regulation: MIR509-3 --+> chemosensitivity	33790078	10.1248/CPB.C20-00
positive Regulation: MIR509-3 --+> chemosensitivity	33790078	10.1248/CPB.C20-00
positive Regulation: MIR509-3 --+> chemosensitivity	33790078	10.1248/CPB.C20-00
positive Regulation: MIR509-3 --+> chemosensitivity	33790078	10.1248/CPB.C20-00
positive Regulation: MIR509-3 --+> chemosensitivity	33790078	10.1248/cpb.c20-00
positive Regulation: PAWR --+> chemosensitivity	24164776	10.1186/bcr3562
positive Regulation: ABCB9 --+> chemosensitivity	30400960	10.1186/s13046-018
positive Regulation: ABCB9 --+> chemosensitivity	30400960	10.1186/s13046-018
positive Regulation: TFF3 --+> chemosensitivity	15769482	10.1016/j.lfs.2004
positive Regulation: TFF3 --+> chemosensitivity	15769482	10.1016/j.lfs.2004
positive Regulation: MIR3609 --+> chemosensitivity	34440380	10.3390/genes12081
positive Regulation: MIR212 --+> chemosensitivity	32427088	10.2174/1871530320
positive Regulation: KAT2B --+> chemosensitivity	31042625	10.1016/j.neo.2019
positive Regulation: MIR129-2 --+> chemosensitivity	35146917	10.1111/1759-7714.
positive Regulation: MIR129-2 --+> chemosensitivity	35146917	10.1111/1759-7714.
positive Regulation: MIR7-1 --+> chemosensitivity	29421518	10.1016/j.cbi.2018
positive Regulation: MIR7-1 --+> chemosensitivity	33152930	10.1016/j.biopha.2
positive Regulation: MIR7-1 --+> chemosensitivity	33497805	10.1016/j.phrs.202
positive Regulation: MIR7-1 --+> chemosensitivity	23206698	10.1016/j.bbrc.201

positive Regulation: MIR7-1 --+> chemosensitivity	28618418	10.1159/000477884
positive Regulation: MIR7-1 --+> chemosensitivity	28618418	10.1159/000477884
positive Regulation: MIR7-1 --+> chemosensitivity	34400888	10.7150/IJMS.62219
positive Regulation: IL24 --+> chemosensitivity	28228085	10.2174/1568009617
positive Regulation: IL24 --+> chemosensitivity	33894112	10.1515/hsz-2020-0
positive Regulation: IL24 --+> chemosensitivity	33894112	10.1515/hsz-2020-0
positive Regulation: IL24 --+> chemosensitivity	23783436	10.3892/or.2013.25
positive Regulation: IL24 --+> chemosensitivity	23982423	10.3892/or.2013.26
positive Regulation: IL24 --+> chemosensitivity	23982423	10.3892/or.2013.26
positive Regulation: IL24 --+> chemosensitivity	23982423	10.3892/or.2013.26
positive Regulation: IL24 --+> chemosensitivity	25778843	10.3892/or.2015.38
positive Regulation: IL24 --+> chemosensitivity	26168134	10.3727/096504015X
positive Regulation: IL24 --+> chemosensitivity	21553494	
positive Regulation: IL24 --+> chemosensitivity	23982423	10.3892/or.2013.26
positive Regulation: IL24 --+> chemosensitivity	23982423	10.3892/or.2013.26
positive Regulation: IL24 --+> chemosensitivity	23982423	10.3892/or.2013.26
positive Regulation: IL24 --+> chemosensitivity	25778843	10.3892/or.2015.38
positive Regulation: IL24 --+> chemosensitivity	26168134	10.3727/096504015X
positive Regulation: IL24 --+> chemosensitivity	35008495	10.3390/ijms230100
positive Regulation: MIR193A --+> chemosensitivity	26743123	10.1016/j.gene.201
positive Regulation: MIR193A --+> chemosensitivity	21293058	
positive Regulation: MIR193A --+> chemosensitivity	21293058	
positive Regulation: MIR193A --+> chemosensitivity	29848678	10.21873/anticanre
positive Regulation: MIR193A --+> chemosensitivity	30531834	10.1038/s41388-018
positive Regulation: MIR193A --+> chemosensitivity	32323741	10.3892/ijmm.2020.
positive Regulation: MIR193A --+> chemosensitivity	32209033	10.2174/1566524020
positive Regulation: MIR193A --+> chemosensitivity	21293058	10.1172/JCI43897
positive Regulation: MIR193A --+> chemosensitivity	25964554	
positive Regulation: MIR193A --+> chemosensitivity	26488287	10.1159/000439102
positive Regulation: MIR193A --+> chemosensitivity	29216925	10.1186/s13046-017
positive Regulation: MIR370 --+> chemosensitivity	31589963	10.1016/j.gene.201
positive Regulation: MIR370 --+> chemosensitivity	25063739	10.1016/j.canlet.2
positive Regulation: MIR370 --+> chemosensitivity	30576245	10.1152/ajpendo.00
positive Regulation: MIR370 --+> chemosensitivity	32052578	10.15252/embr.2019
positive Regulation: MIR370 --+> chemosensitivity	30712191	10.1007/s12253-019
positive Regulation: MIR370 --+> chemosensitivity	25063739	10.1016/j.canlet.2
positive Regulation: MIR370 --+> chemosensitivity	25063739	10.1016/j.canlet.2
positive Regulation: MIR370 --+> chemosensitivity	29242506	10.1038/s41408-017
positive Regulation: CNOT3 --+> chemosensitivity	31177396	10.1007/s10495-019
positive Regulation: BTG2 --+> chemosensitivity	27932314	10.1016/j.cellsig.
positive Regulation: MIR219A1 --+> chemosensitivity	33348804	10.3390/ijms212496
positive Regulation: MIR219A1 --+> chemosensitivity	28884131	10.1155/2017/90325
positive Regulation: USP17L2 --+> chemosensitivity	35383144	10.1038/s41420-022
positive Regulation: INS --+> chemosensitivity	36459572	10.1113/EP090584
positive Regulation: MIR423 --+> chemosensitivity	31424364	10.2174/1389200220
positive Regulation: SUFU --+> chemosensitivity	27810403	10.1016/j.canlet.2
positive Regulation: FZD5 --+> chemosensitivity	33311446	10.1038/s41419-020
positive Regulation: MIR5195 --+> chemosensitivity	31308851	10.1186/s11658-019
positive Regulation: CCL3 --+> chemosensitivity	35613826	10.1136/jitc-2021-
positive Regulation: CCL3 --+> chemosensitivity	35613826	10.1136/jitc-2021-
positive Regulation: CCL3 --+> chemosensitivity	35613826	10.1136/jitc-2021-
positive Regulation: MARCKS --+> chemosensitivity	32056006	10.1007/s00432-020
positive Regulation: MARCKS --+> chemosensitivity	32112147	10.1007/s00432-020
positive Regulation: MARCKS --+> chemosensitivity	32112147	10.1007/s00432-020

positive Regulation: DIRAS3 --+> chemosensitivity	26247722	10.1038/cddis.2015
positive Regulation: DIRAS3 --+> chemosensitivity	26247722	10.1038/cddis.2015
positive Regulation: DIRAS3 --+> chemosensitivity	26247722	10.1038/cddis.2015
positive Regulation: DIRAS3 --+> chemosensitivity	26247722	10.1038/cddis.2015
positive Regulation: DIRAS3 --+> chemosensitivity	26247722	10.1038/cddis.2015
positive Regulation: DIRAS3 --+> chemosensitivity	26247722	10.1038/cddis.2015
positive Regulation: MIR22 --+> chemosensitivity	32818543	10.1016/j.lfs.2020
positive Regulation: MIR22 --+> chemosensitivity	32634442	10.1016/j.mad.2020
positive Regulation: MIR22 --+> chemosensitivity	28242349	10.1016/j.bbcan.20
positive Regulation: MIR22 --+> chemosensitivity	32887025	10.1016/j.biopha.2
positive Regulation: MIR22 --+> chemosensitivity	31257059	10.1016/j.jphs.201
positive Regulation: MIR22 --+> chemosensitivity	31257059	10.1016/j.jphs.201
positive Regulation: MIR22 --+> chemosensitivity	27573902	10.1007/978-3-319-
positive Regulation: MIR22 --+> chemosensitivity	28000852	10.3892/ijo.2016.3
positive Regulation: MIR22 --+> chemosensitivity	28000852	10.3892/ijo.2016.3
positive Regulation: MIR22 --+> chemosensitivity	28000852	10.3892/ijo.2016.3
positive Regulation: MIR22 --+> chemosensitivity	28000852	10.3892/ijo.2016.3
positive Regulation: MIR22 --+> chemosensitivity	29568877	10.3892/mmr.2018.8
positive Regulation: MIR22 --+> chemosensitivity	30041677	10.1186/s13046-018
positive Regulation: MIR22 --+> chemosensitivity	30041677	10.1186/s13046-018
positive Regulation: MIR22 --+> chemosensitivity	30041677	10.1186/s13046-018
positive Regulation: MIR22 --+> chemosensitivity	30041677	10.1186/s13046-018
positive Regulation: MIR22 --+> chemosensitivity	31257059	10.1016/j.jphs.201
positive Regulation: MIR22 --+> chemosensitivity	31257059	10.1016/j.jphs.201
positive Regulation: MIR22 --+> chemosensitivity	31638211	10.3892/ijo.2019.4
positive Regulation: MIR22 --+> chemosensitivity	32323781	10.3892/or.2020.74
positive Regulation: MIR22 --+> chemosensitivity	33284712	10.1177/0300060520
positive Regulation: MIR22 --+> chemosensitivity	33284712	10.1177/0300060520
positive Regulation: MIR22 --+> chemosensitivity	33284712	10.1177/0300060520
positive Regulation: MIR22 --+> chemosensitivity	33319669	10.2174/1389557520
positive Regulation: MIR22 --+> chemosensitivity	30932352	10.1111/os.1244210
positive Regulation: LAMB3 --+> chemosensitivity	30414703	10.1016/j.ejso.201
positive Regulation: DEFA1 --+> chemosensitivity	24378474	10.3892/or.2013.29
positive Regulation: DEFA1 --+> chemosensitivity	24378474	10.3892/or.2013.29
positive Regulation: DEFA1 --+> chemosensitivity	24378474	10.3892/or.2013.29
positive Regulation: DEFA1 --+> chemosensitivity	24378474	10.3892/or.2013.29
positive Regulation: DEFA1 --+> chemosensitivity	24378474	10.3892/or.2013.29
positive Regulation: PRKAA1 --+> chemosensitivity	28065383	10.1016/j.pan.2016
positive Regulation: MIR509-1 --+> chemosensitivity	33152930	10.1016/j.biopha.2
positive Regulation: TLR5 --+> chemosensitivity	36140341	10.3390/biomedicin
positive Regulation: MAD2L1 --+> chemosensitivity	18597777	10.1016/j.jmb.2008
positive Regulation: MAD2L1 --+> chemosensitivity	16880791	
positive Regulation: MAD2L1 --+> chemosensitivity	16880791	
positive Regulation: MIR211 --+> chemosensitivity	29115509	10.3892/mmr.2017.8
positive Regulation: MIR211 --+> chemosensitivity	30821276	10.12659/MSM.91186
positive Regulation: MIR211 --+> chemosensitivity	30821276	10.12659/MSM.91186
positive Regulation: MIR211 --+> chemosensitivity	31235732	10.1038/s41419-019
positive Regulation: MIR211 --+> chemosensitivity	31235732	10.1038/s41419-019
positive Regulation: MIR211 --+> chemosensitivity	31235732	10.1038/s41419-019
positive Regulation: MIR211 --+> chemosensitivity	31235732	10.1038/s41419-019
positive Regulation: MIR211 --+> chemosensitivity	31235732	10.1038/s41419-019
positive Regulation: MIR211 --+> chemosensitivity	31235732	10.1038/s41419-019
positive Regulation: MIR211 --+> chemosensitivity	31235732	10.1038/s41419-019
positive Regulation: MIR211 --+> chemosensitivity	31235732	10.1038/s41419-019

positive Regulation: MIR211 --+> chemosensitivity	31235732	10.1038/s41419-019
positive Regulation: MIR211 --+> chemosensitivity	33223523	10.1038/s41417-020
positive Regulation: MIR211 --+> chemosensitivity	23966157	10.4161/cc.25950
positive Regulation: MIR211 --+> chemosensitivity	26035292	10.3892/ijo.2015.3
positive Regulation: MIR211 --+> chemosensitivity	23183822	10.18632/oncotarge
positive Regulation: MIR211 --+> chemosensitivity	31235732	10.1038/s41419-019
positive Regulation: MIR211 --+> chemosensitivity	31235732	10.1038/s41419-019
positive Regulation: MIR211 --+> chemosensitivity	33494674	10.2174/1389450122
positive Regulation: NDUFA13 --+> chemosensitivity	30032449	10.1007/s00280-018
positive Regulation: NDUFA13 --+> chemosensitivity	30032449	10.1007/s00280-018
positive Regulation: MIR494 --+> chemosensitivity	33892053	10.1016/j.bbcan.20
positive Regulation: MIR494 --+> chemosensitivity		10.14715/cmb/2017.
positive Regulation: MIR494 --+> chemosensitivity		10.14715/cmb/2017.
positive Regulation: MIR494 --+> chemosensitivity		10.14715/cmb/2017.
positive Regulation: MIR494 --+> chemosensitivity		10.14715/cmb/2017.
positive Regulation: MIR494 --+> chemosensitivity		10.14715/cmb/2017.
positive Regulation: MIR494 --+> chemosensitivity		10.14715/cmb/2017.
positive Regulation: MIR494 --+> chemosensitivity		10.14715/cmb/2017.
positive Regulation: MIR494 --+> chemosensitivity	30672438	
positive Regulation: MIR494 --+> chemosensitivity	30672438	
positive Regulation: ADARB1 --+> chemosensitivity	32052578	10.15252/embr.2019
positive Regulation: DFFB --+> chemosensitivity	14969818	10.1016/j.oralonco
positive Regulation: MIR1268B --+> chemosensitivity	29567542	10.1016/j.biopha.2
positive Regulation: MIR1268B --+> chemosensitivity	31136284	10.1161/STROKEAHA.
positive Regulation: MIR1268B --+> chemosensitivity	29163776	10.18632/oncotarge
positive Regulation: MIR1268B --+> chemosensitivity	29163776	10.18632/oncotarge
positive Regulation: MIR138-1 --+> chemosensitivity	27622325	10.1016/j.bbrc.201
positive Regulation: MIR138-1 --+> chemosensitivity	28922711	10.1016/j.biopha.2
positive Regulation: MIR138-1 --+> chemosensitivity	33360300	10.1016/j.tranon.2
positive Regulation: MIR138-1 --+> chemosensitivity	30594069	10.1016/j.omtn.201
positive Regulation: MIR138-1 --+> chemosensitivity	28378633	10.1177/1010428317
positive Regulation: MIR138-1 --+> chemosensitivity	28378633	10.1177/1010428317
positive Regulation: MIR138-1 --+> chemosensitivity	28378633	10.1177/1010428317
positive Regulation: MIR138-1 --+> chemosensitivity	29328459	10.3892/or.2018.61
positive Regulation: MIR138-1 --+> chemosensitivity	29328459	10.3892/or.2018.61
positive Regulation: MIR138-1 --+> chemosensitivity	32349783	10.1186/s40659-020
positive Regulation: MIR138-1 --+> chemosensitivity	32349783	10.1186/s40659-020
positive Regulation: MIR138-1 --+> chemosensitivity	32349783	10.1186/s40659-020
positive Regulation: MIR138-1 --+> chemosensitivity	25190487	10.3892/or.2014.34
positive Regulation: MIR138-1 --+> chemosensitivity	25190487	10.3892/or.2014.34
positive Regulation: MIR138-1 --+> chemosensitivity	27019355	10.1371/journal.po
positive Regulation: MIR138-1 --+> chemosensitivity	27019355	10.1371/journal.po
positive Regulation: MIR138-1 --+> chemosensitivity	27019355	10.1371/journal.po
positive Regulation: MIR138-1 --+> chemosensitivity	27019355	10.1371/journal.po
positive Regulation: MIR138-1 --+> chemosensitivity	26631041	10.1007/s13277-015
positive Regulation: MIR138-1 --+> chemosensitivity	25571061	10.1109/EMBC.2014.
positive Regulation: MIR138-1 --+> chemosensitivity	26631041	10.1007/s13277-015
positive Regulation: MIR138-1 --+> chemosensitivity	27019355	10.1371/journal.po
positive Regulation: MIR138-1 --+> chemosensitivity	31853324	10.3892/etm.2019.8
positive Regulation: MIR138-1 --+> chemosensitivity	32349783	10.1186/s40659-020
positive Regulation: MIR138-1 --+> chemosensitivity	34440380	10.3390/genes12081
positive Regulation: AQP9 --+> chemosensitivity	28640255	10.1038/cddis.2017
positive Regulation: AQP9 --+> chemosensitivity	28640255	10.1038/cddis.2017
positive Regulation: RAB37 --+> chemosensitivity	32858472	10.1016/j.ejmech.2

positive Regulation: RAB37 --+> chemosensitivity	33824475	10.1038/s41388-021
positive Regulation: RAB37 --+> chemosensitivity	30131385	10.1158/1078-0432.
positive Regulation: MIR218-1 --+> chemosensitivity	32681832	10.1016/j.abb.2020
positive Regulation: MIR218-1 --+> chemosensitivity	30921705	10.1016/j.biopha.2
positive Regulation: MIR218-1 --+> chemosensitivity	32711289	10.1016/j.oraloncc
positive Regulation: MIR218-1 --+> chemosensitivity	32711289	10.1016/j.oraloncc
positive Regulation: MIR218-1 --+> chemosensitivity	33965650	10.1016/j.jogoh.20
positive Regulation: MIR218-1 --+> chemosensitivity	25857406	10.1016/j.yexcr.20
positive Regulation: MIR218-1 --+> chemosensitivity	25857406	10.1016/j.yexcr.20
positive Regulation: MIR218-1 --+> chemosensitivity	25857406	10.1016/j.yexcr.20
positive Regulation: MIR218-1 --+> chemosensitivity	26662432	10.4238/2015.Decem
positive Regulation: MIR218-1 --+> chemosensitivity	27415661	10.1159/000445653
positive Regulation: MIR218-1 --+> chemosensitivity	28222430	10.1159/000460505
positive Regulation: MIR218-1 --+> chemosensitivity	28222430	10.1159/000460505
positive Regulation: MIR218-1 --+> chemosensitivity	28222430	10.1159/000460505
positive Regulation: MIR218-1 --+> chemosensitivity	28492560	10.1038/cddis.2017
positive Regulation: MIR218-1 --+> chemosensitivity	28192397	10.1038/onc.2016.4
positive Regulation: MIR218-1 --+> chemosensitivity	28192397	10.1038/onc.2016.4
positive Regulation: MIR218-1 --+> chemosensitivity	28631568	10.1177/1010428317
positive Regulation: MIR218-1 --+> chemosensitivity	30293085	10.12659/MSM.91029
positive Regulation: MIR218-1 --+> chemosensitivity	31257509	10.3892/mmr.2019.1
positive Regulation: MIR218-1 --+> chemosensitivity	32869841	10.1042/BSR2019450
positive Regulation: MIR218-1 --+> chemosensitivity	23443110	10.3390/ijms131216
positive Regulation: MIR218-1 --+> chemosensitivity	23443110	10.3390/ijms131216
positive Regulation: MIR218-1 --+> chemosensitivity	23443110	10.3390/ijms131216
positive Regulation: MIR218-1 --+> chemosensitivity	23443110	10.3390/ijms131216
positive Regulation: MIR218-1 --+> chemosensitivity	23443110	10.3390/ijms131216
positive Regulation: MIR218-1 --+> chemosensitivity	24705471	10.1371/journal.pc
positive Regulation: MIR218-1 --+> chemosensitivity	25482044	10.3892/or.2014.36
positive Regulation: MIR218-1 --+> chemosensitivity	25482044	10.3892/or.2014.36
positive Regulation: MIR218-1 --+> chemosensitivity	25482044	10.3892/or.2014.36
positive Regulation: MIR218-1 --+> chemosensitivity	25482044	10.3892/or.2014.36
positive Regulation: MIR218-1 --+> chemosensitivity	25482044	10.3892/or.2014.36
positive Regulation: MIR218-1 --+> chemosensitivity		
positive Regulation: MIR218-1 --+> chemosensitivity	25132800	
positive Regulation: MIR218-1 --+> chemosensitivity		
positive Regulation: MIR218-1 --+> chemosensitivity	26282001	10.1007/s13277-015
positive Regulation: MIR218-1 --+> chemosensitivity	26282001	10.1007/s13277-015
positive Regulation: MIR218-1 --+> chemosensitivity	26282001	10.1007/s13277-015
positive Regulation: MIR218-1 --+> chemosensitivity	26282001	10.1007/s13277-015
positive Regulation: MIR218-1 --+> chemosensitivity	26282001	10.1007/s13277-015
positive Regulation: MIR218-1 --+> chemosensitivity	26282001	10.1007/s13277-015
positive Regulation: MIR218-1 --+> chemosensitivity	23443110	10.3390/ijms131216
positive Regulation: MIR218-1 --+> chemosensitivity	23443110	10.3390/ijms131216
positive Regulation: MIR218-1 --+> chemosensitivity	25170221	10.3748/wjg.v20.i3
positive Regulation: MIR218-1 --+> chemosensitivity	25394901	10.1007/s13277-014
positive Regulation: MIR218-1 --+> chemosensitivity	25482044	10.3892/or.2014.36
positive Regulation: MIR218-1 --+> chemosensitivity	26282001	10.1007/s13277-015
positive Regulation: MIR218-1 --+> chemosensitivity	26442524	10.1093/carcin/bgv
positive Regulation: MIR218-1 --+> chemosensitivity	28052414	10.1002/mc.2261210
positive Regulation: MIR218-1 --+> chemosensitivity	28192397	10.1038/onc.2016.4
positive Regulation: MIR218-1 --+> chemosensitivity	28222430	10.1159/0004605051
positive Regulation: MIR218-1 --+> chemosensitivity	28222430	10.1159/0004605051
positive Regulation: MIR218-1 --+> chemosensitivity	34453645	10.1007/s11010-021

positive Regulation: MIR218-1 --+> chemosensitivity	35005988	10.1089/dna.2021.0
positive Regulation: MIR218-1 --+> chemosensitivity		10.32604/biocell.2
positive Regulation: MIR379 --+> chemosensitivity	33892053	10.1016/j.bbcan.20
positive Regulation: MIR379 --+> chemosensitivity	28051262	
positive Regulation: MIR379 --+> chemosensitivity	29286115	10.3892/mmr.2017.8
positive Regulation: MIR379 --+> chemosensitivity	33173959	10.3892/mmr.2020.1
positive Regulation: CLDN7 --+> chemosensitivity	24009024	10.3390/ijms140918
positive Regulation: LYVE1 --+> chemosensitivity	23743354	10.1016/j.canlet.2
positive Regulation: FUBP1 --+> chemosensitivity	28667493	10.1007/s12253-017
positive Regulation: CHL1 --+> chemosensitivity	32863924	10.3892/ol.2020.11
positive Regulation: MIR26A1 --+> chemosensitivity	30898716	10.1016/j.gene.201
positive Regulation: MIR26A1 --+> chemosensitivity	29518611	10.1016/j.biopha.2
positive Regulation: MIR26A1 --+> chemosensitivity	28614291	10.12659/MSM.90417
positive Regulation: MIR26A1 --+> chemosensitivity	28898169	10.1080/15384101.2
positive Regulation: MIR26A1 --+> chemosensitivity	28079894	10.1038/cddis.2016
positive Regulation: MIR26A1 --+> chemosensitivity	30425242	10.1038/s41419-018
positive Regulation: MIR26A1 --+> chemosensitivity	30425242	10.1038/s41419-018
positive Regulation: MIR26A1 --+> chemosensitivity	26398882	10.3892/or.2015.42
positive Regulation: MIR26A1 --+> chemosensitivity	28079894	10.1038/cddis.2016
positive Regulation: MIR26A1 --+> chemosensitivity	34639131	10.3390/ijms221910
positive Regulation: MIR26A1 --+> chemosensitivity	34510251	10.1007/s00280-021
positive Regulation: S100A16 --+> chemosensitivity	31894756	10.12659/MSM.91975
positive Regulation: S100A16 --+> chemosensitivity	30916375	10.1111/bjh.158781
positive Regulation: MIR133B --+> chemosensitivity	30653948	10.1016/j.clinbioc
positive Regulation: MIR133B --+> chemosensitivity	29328427	10.3892/ijmm.2018.
positive Regulation: MIR133B --+> chemosensitivity	29328427	10.3892/ijmm.2018.
positive Regulation: MIR133B --+> chemosensitivity	29327946	10.1089/dna.2017.3
positive Regulation: MIR133B --+> chemosensitivity	28881788	10.18632/oncotarge
positive Regulation: MIR133B --+> chemosensitivity	28881788	10.18632/oncotarge
positive Regulation: MIR133B --+> chemosensitivity	30317571	10.1002/jcp.272881
positive Regulation: MIR133B --+> chemosensitivity	35086110	10.1159/000521493
positive Regulation: MIR487B --+> chemosensitivity	33125503	10.3892/or.2020.78
positive Regulation: MIR487B --+> chemosensitivity	33390839	10.7150/ijbs.50773
positive Regulation: LIN28A --+> chemosensitivity	26687759	10.1007/s13277-015
positive Regulation: LIN28A --+> chemosensitivity	26687759	10.1007/s13277-015
positive Regulation: LIN28A --+> chemosensitivity	26687759	10.1007/s13277-015
positive Regulation: LIN28A --+> chemosensitivity	26687759	10.1007/s13277-015
positive Regulation: LIN28A --+> chemosensitivity	26687759	10.1007/s13277-015
positive Regulation: LIN28A --+> chemosensitivity	22808086	10.1371/journal.pc
positive Regulation: LIN28A --+> chemosensitivity	23335963	10.1371/journal.pc
positive Regulation: LIN28A --+> chemosensitivity	26123544	10.1186/s12943-015
positive Regulation: LIN28A --+> chemosensitivity	26687759	10.1007/s13277-015
positive Regulation: MIR148B --+> chemosensitivity	26554910	10.1016/j.jgg.2015
positive Regulation: MIR148B --+> chemosensitivity	33831655	10.1016/j.tranon.2
positive Regulation: MIR148B --+> chemosensitivity		
positive Regulation: MIR148B --+> chemosensitivity	25997710	10.1042/BSR2015008
positive Regulation: MIR148B --+> chemosensitivity	25997710	10.1042/BSR2015008
positive Regulation: MIR148B --+> chemosensitivity	25997710	10.1042/BSR2015008
positive Regulation: MIR148B --+> chemosensitivity	23171948	10.1158/1535-7163.
positive Regulation: MIR148B --+> chemosensitivity	23171948	10.1158/1535-7163.
positive Regulation: MIR148B --+> chemosensitivity	25997710	10.1042/BSR2015008
positive Regulation: MIR148B --+> chemosensitivity	33423167	10.1007/s13402-020
positive Regulation: MIR148B --+> chemosensitivity	34283401	10.1007/s13402-021
positive Regulation: MIR148B --+> chemosensitivity	34473617	10.2174/1566524021

positive Regulation: PBK --+> chemosensitivity	28373071	10.1016/j.bbrc.201
positive Regulation: PBK --+> chemosensitivity	30778048	10.1038/s41419-019
positive Regulation: PBK --+> chemosensitivity	28373071	10.1016/j.bbrc.201
positive Regulation: MIR433 --+> chemosensitivity	29495532	10.3390/ijms190306
positive Regulation: MIR433 --+> chemosensitivity		
positive Regulation: MIR433 --+> chemosensitivity	33485372	10.1186/s13048-020
positive Regulation: MIR433 --+> chemosensitivity	27926502	10.18632/oncotarge
positive Regulation: MIR433 --+> chemosensitivity	27926502	10.18632/oncotarge
positive Regulation: MIR433 --+> chemosensitivity		10.1007/s12038-021
positive Regulation: MIR433 --+> chemosensitivity	36303447	10.1002/brb3.2632
positive Regulation: TACC1 --+> chemosensitivity	23354013	10.3892/or.2013.22
positive Regulation: NEFL --+> chemosensitivity	26879754	10.1007/s10571-016
positive Regulation: NEFL --+> chemosensitivity	31057612	10.1155/2019/35181
positive Regulation: TRIM72 --+> chemosensitivity	35858925	10.1038/s41389-022
positive Regulation: WARS1 --+> chemosensitivity	33408886	10.4048/jbc.2020.2
positive Regulation: C4BPA --+> chemosensitivity	34167573	10.1186/s13046-021
positive Regulation: C4BPA --+> chemosensitivity	34167573	10.1186/s13046-021
positive Regulation: MIR139 --+> chemosensitivity	30336197	10.1016/j.canlet.2
positive Regulation: MIR139 --+> chemosensitivity	26299922	10.1016/j.bbrc.201
positive Regulation: MIR139 --+> chemosensitivity	27173050	10.1016/j.prp.2016
positive Regulation: MIR139 --+> chemosensitivity	28713954	10.3892/mmr.2017.6
positive Regulation: MIR139 --+> chemosensitivity	28095367	10.1515/hsz-2016-0
positive Regulation: MIR139 --+> chemosensitivity	29594361	10.1007/s00280-018
positive Regulation: MIR139 --+> chemosensitivity	29719173	10.1152/ajpcell.00
positive Regulation: MIR139 --+> chemosensitivity	30439707	10.1159/000495169
positive Regulation: MIR139 --+> chemosensitivity	35212928	10.1007/s11033-022
positive Regulation: ID3 --+> chemosensitivity	30008847	10.3892/ol.2018.88
positive Regulation: MIR30A --+> chemosensitivity	33892053	10.1016/j.bbcan.20
positive Regulation: MIR30A --+> chemosensitivity	30249919	10.2176/nmc.ra.201
positive Regulation: MIR30A --+> chemosensitivity	31638211	10.3892/ijo.2019.4
positive Regulation: MIR30A --+> chemosensitivity	33097691	10.1038/s41419-020
positive Regulation: MIR30A --+> chemosensitivity	29375703	10.3892/etm.2017.5
positive Regulation: MIR30A --+> chemosensitivity	29805498	10.3892/etm.2018.6
positive Regulation: MIR30A --+> chemosensitivity	29805498	10.3892/etm.2018.6
positive Regulation: MIR30A --+> chemosensitivity	31602254	10.7150/jca.311911
positive Regulation: MIR30A --+> chemosensitivity	35449123	10.1038/s41419-022
positive Regulation: MIR30A --+> chemosensitivity	35449123	10.1038/s41419-022
positive Regulation: MIR30A --+> chemosensitivity	35449123	10.1038/s41419-022
positive Regulation: KRT10 --+> chemosensitivity	24434152	10.1016/j.bbrc.201
positive Regulation: KRT10 --+> chemosensitivity	24434152	10.1016/j.bbrc.201
positive Regulation: KRT10 --+> chemosensitivity	24434152	10.1016/j.bbrc.201
positive Regulation: HDAC1 --+> chemosensitivity	33539818	10.1016/j.ejphar.2
positive Regulation: HDAC1 --+> chemosensitivity	33539818	10.1016/j.ejphar.2
positive Regulation: HDAC1 --+> chemosensitivity	30500418	10.1016/j.humpath.
positive Regulation: HDAC1 --+> chemosensitivity	30071534	10.1159/000492260
positive Regulation: HDAC1 --+> chemosensitivity	30071534	10.1159/000492260
positive Regulation: HDAC1 --+> chemosensitivity	30071534	10.1159/000492260
positive Regulation: HDAC1 --+> chemosensitivity	30071534	10.1159/000492260
positive Regulation: HDAC1 --+> chemosensitivity	30168013	10.1007/s10637-018
positive Regulation: HDAC1 --+> chemosensitivity	30071534	10.1159/0004922601
positive Regulation: EGF --+> chemosensitivity	23554613	10.1016/S1674-8301
positive Regulation: EGF --+> chemosensitivity	23554613	10.1016/S1674-8301
positive Regulation: EGF --+> chemosensitivity	24761873	10.7314/apjcp.2014
positive Regulation: MIR125A --+> chemosensitivity	28088556	10.1016/j.urology.

positive Regulation: MIR125A --+> chemosensitivity	29990840	10.1016/j.biopha.2
positive Regulation: MIR125A --+> chemosensitivity	29990840	10.1016/j.biopha.2
positive Regulation: MIR125A --+> chemosensitivity	33152930	10.1016/j.biopha.2
positive Regulation: MIR125A --+> chemosensitivity	32497683	10.1016/j.semcance
positive Regulation: MIR125A --+> chemosensitivity	28381182	10.1177/1010428317
positive Regulation: MIR125A --+> chemosensitivity	29739052	10.7754/Clin.Lab.2
positive Regulation: MIR125A --+> chemosensitivity	29767234	10.3892/ijo.2018.4
positive Regulation: MIR125A --+> chemosensitivity	29767234	10.3892/ijo.2018.4
positive Regulation: MIR125A --+> chemosensitivity	29767234	10.3892/ijo.2018.4
positive Regulation: MIR125A --+> chemosensitivity	30541899	10.1042/BSR2018089
positive Regulation: MIR125A --+> chemosensitivity	26758190	10.1093/abbs/gmv12
positive Regulation: MIR125A --+> chemosensitivity	26758190	10.1093/abbs/gmv12
positive Regulation: MIR125A --+> chemosensitivity	29990840	10.1016/j.biopha.2
positive Regulation: MIR125A --+> chemosensitivity	29990840	10.1016/j.biopha.2
positive Regulation: MIR125A --+> chemosensitivity	29990840	10.1016/j.biopha.2
positive Regulation: MIR125A --+> chemosensitivity		10.1016/j.semcance
positive Regulation: MOAP1 --+> chemosensitivity	27003254	10.1016/j.bbrc.201
positive Regulation: MOAP1 --+> chemosensitivity	27003254	10.1016/j.bbrc.201
positive Regulation: MOAP1 --+> chemosensitivity	27424190	10.1007/s00432-016
positive Regulation: MOAP1 --+> chemosensitivity	27424190	10.1007/s00432-016
positive Regulation: MOAP1 --+> chemosensitivity	27424190	10.1007/s00432-016
positive Regulation: MOAP1 --+> chemosensitivity	27424190	10.1007/s00432-016
positive Regulation: DACT1 --+> chemosensitivity	29037126	10.1177/1010428317
positive Regulation: DACT1 --+> chemosensitivity	29037126	10.1177/1010428317
positive Regulation: DACT1 --+> chemosensitivity	29037126	10.1177/1010428317
positive Regulation: MIR539 --+> chemosensitivity	30119173	10.1016/j.biopha.2
positive Regulation: MIR539 --+> chemosensitivity	30119173	10.1016/j.biopha.2
positive Regulation: MIR539 --+> chemosensitivity	33352449	10.1016/j.biopha.2
positive Regulation: MIR539 --+> chemosensitivity	31432162	10.3892/mmr.2019.1
positive Regulation: MIR539 --+> chemosensitivity	31432162	10.3892/mmr.2019.1
positive Regulation: MIR539 --+> chemosensitivity	30119173	10.1016/j.biopha.2
positive Regulation: MIR539 --+> chemosensitivity	30119173	10.1016/j.biopha.2
positive Regulation: MIR539 --+> chemosensitivity	36271518	10.1016/j.biopha.2
positive Regulation: IRF4 --+> chemosensitivity	22888789	10.1186/1476-4598-
positive Regulation: PHLDA2 --+> chemosensitivity	31638211	10.3892/ijo.2019.4
positive Regulation: SDCBP --+> chemosensitivity	32595209	10.1038/s41416-020
positive Regulation: MIRLET7A3 --+> chemosensitivity	23335963	10.1371/journal.pc
positive Regulation: MIR128-1 --+> chemosensitivity	31474314	10.1016/j.prp.2019
positive Regulation: MIR128-1 --+> chemosensitivity	30551451	10.1016/j.biopha.2
positive Regulation: MIR128-1 --+> chemosensitivity	33338745	10.1016/j.biopha.2
positive Regulation: MIR128-1 --+> chemosensitivity	27105614	10.1007/s13277-016
positive Regulation: MIR128-1 --+> chemosensitivity	30076414	10.1038/s41388-018
positive Regulation: MIR128-1 --+> chemosensitivity	30601194	10.1097/CAD.000000
positive Regulation: MIR128-1 --+> chemosensitivity	32016963	10.26355/eurrev_20
positive Regulation: MIR128-1 --+> chemosensitivity	31721699	10.2174/1381612825
positive Regulation: MIR128-1 --+> chemosensitivity	33743142	10.1007/s12013-021
positive Regulation: MIR128-1 --+> chemosensitivity	23526655	10.1002/cbin.10100
positive Regulation: MIR128-1 --+> chemosensitivity	25017996	10.3892/or.2014.33
positive Regulation: MIR128-1 --+> chemosensitivity	25921099	10.1093/jjco/hyv02
positive Regulation: MIR128-1 --+> chemosensitivity	28146425	10.18632/oncotarge
positive Regulation: MIR128-1 --+> chemosensitivity	30890168	10.1186/s12943-019
positive Regulation: MIR128-1 --+> chemosensitivity	32398152	10.1186/s12943-020
positive Regulation: MIR128-1 --+> chemosensitivity	27415661	10.1159/000445653
positive Regulation: MIR128-1 --+> chemosensitivity	25017996	10.3892/or.2014.33

positive Regulation: MIR128-1 --+> chemosensitivity	34510546	10.1096/fj.2021002
positive Regulation: MIR128-1 --+> chemosensitivity	34263426	10.1007/s12035-021
positive Regulation: MIR128-1 --+> chemosensitivity	34884646	10.3390/ijms222312
positive Regulation: MIR128-1 --+> chemosensitivity		10.3390/ijms230313
positive Regulation: MIR128-1 --+> chemosensitivity	35563265	10.3390/ijms230948
positive Regulation: MIR128-1 --+> chemosensitivity	35906618	10.1186/s12943-022
positive Regulation: MIR128-1 --+> chemosensitivity	35906618	10.1186/s12943-022
positive Regulation: MIR149 --+> chemosensitivity	33197838	10.1016/j.prp.2020
positive Regulation: MIR149 --+> chemosensitivity	33253912	10.1016/j.cellsig.
positive Regulation: MIR149 --+> chemosensitivity	33348804	10.3390/ijms212496
positive Regulation: MIR149 --+> chemosensitivity	33011952	10.1007/s11010-020
positive Regulation: MIR149 --+> chemosensitivity	33011952	10.1007/s11010-020
positive Regulation: MIR149 --+> chemosensitivity	34140005	10.1186/s12967-021
positive Regulation: MIR149 --+> chemosensitivity	27121091	10.3892/mmr.2016.5
positive Regulation: MIR149 --+> chemosensitivity	25017996	10.3892/or.2014.33
positive Regulation: MIR149 --+> chemosensitivity	26223974	10.1186/s13048-015
positive Regulation: MIR149 --+> chemosensitivity	31889909	10.1186/s12935-019
positive Regulation: MIR149 --+> chemosensitivity	31889909	10.1186/s12935-019
positive Regulation: MIR149 --+> chemosensitivity	31889909	10.1186/s12935-019
positive Regulation: MIR149 --+> chemosensitivity	31933761	
positive Regulation: MIR149 --+> chemosensitivity	31933761	
positive Regulation: MIR149 --+> chemosensitivity	33011952	10.1007/s11010-020
positive Regulation: MIR149 --+> chemosensitivity	27415661	10.1159/000445653
positive Regulation: MIR149 --+> chemosensitivity	25017996	10.3892/or.2014.33
positive Regulation: MIR149 --+> chemosensitivity		10.3390/molecules2
positive Regulation: MIR149 --+> chemosensitivity	35806286	10.3390/ijms231372
positive Regulation: MIR149 --+> chemosensitivity	36142734	10.3390/ijms231810
positive Regulation: MIR148A --+> chemosensitivity	20406806	10.1074/jbc.M109.0
positive Regulation: MIR148A --+> chemosensitivity	20406806	10.1074/jbc.M109.0
positive Regulation: MIR148A --+> chemosensitivity	30551544	10.1016/j.biopha.2
positive Regulation: MIR148A --+> chemosensitivity	23383211	10.1371/journal.pc
positive Regulation: MIR148A --+> chemosensitivity	35563265	10.3390/ijms230948
positive Regulation: BAX --+> chemosensitivity	12560233	10.1182/blood-2002
positive Regulation: BAX --+> chemosensitivity	12175703	10.1016/S0024-3205
positive Regulation: BAX --+> chemosensitivity	20206383	10.1016/j.leukres.
positive Regulation: BAX --+> chemosensitivity	11479005	10.1016/S0165-6147
positive Regulation: BAX --+> chemosensitivity	15327837	10.1016/j.canlet.2
positive Regulation: BAX --+> chemosensitivity	15629598	10.1016/j.ijrobp.2
positive Regulation: BAX --+> chemosensitivity	19480968	10.1016/j.ijrobp.2
positive Regulation: BAX --+> chemosensitivity	9793746	10.1016/S1040-8428
positive Regulation: BAX --+> chemosensitivity		10.1016/j.bionut.2
positive Regulation: BAX --+> chemosensitivity	23540284	10.1016/j.biopha.2
positive Regulation: BAX --+> chemosensitivity	23540284	10.1016/j.biopha.2
positive Regulation: BAX --+> chemosensitivity	14645705	
positive Regulation: BAX --+> chemosensitivity	9122197	
positive Regulation: BAX --+> chemosensitivity	9122197	
positive Regulation: BAX --+> chemosensitivity	10557106	
positive Regulation: BAX --+> chemosensitivity	24173654	10.3892/or.2013.28
positive Regulation: BAX --+> chemosensitivity	24637737	10.1371/journal.pc
positive Regulation: BAX --+> chemosensitivity	9122197	10.1073/pnas.94.6.
positive Regulation: BAX --+> chemosensitivity	9802058	
positive Regulation: BAX --+> chemosensitivity	10419903	
positive Regulation: BAX --+> chemosensitivity	23526655	10.1002/cbin.10100
positive Regulation: BAX --+> chemosensitivity	11803466	

positive Regulation: BAX --+> chemosensitivity	22590594	10.1371/journal.pc
positive Regulation: PIWIL1 --+> chemosensitivity	25701955	10.1007/s12013-015
positive Regulation: MIR1271 --+> chemosensitivity	36136988	10.1097/CAD.00000C
positive Regulation: CYTOR --+> chemosensitivity		
positive Regulation: CYTOR --+> chemosensitivity	29863253	10.26355/eurrev_2C
positive Regulation: CYTOR --+> chemosensitivity	31777553	10.3892/etm.2019.8
positive Regulation: CYTOR --+> chemosensitivity	31777553	10.3892/etm.2019.8
positive Regulation: CYTOR --+> chemosensitivity	34558643	10.3892/ijo.2021.5
positive Regulation: MSI2 --+> chemosensitivity	28223335	10.1096/fj.2016012
positive Regulation: MSI2 --+> chemosensitivity	28223335	10.1096/fj.2016012
positive Regulation: MSI2 --+> chemosensitivity	28223335	10.1096/fj.2016012
positive Regulation: MSI2 --+> chemosensitivity	28223335	10.1096/fj.2016012
positive Regulation: MSI2 --+> chemosensitivity	26308531	10.1371/journal.pc
positive Regulation: MSI2 --+> chemosensitivity	26308531	10.1371/journal.pc
positive Regulation: MSI2 --+> chemosensitivity	26308531	10.1371/journal.pc
positive Regulation: MSI2 --+> chemosensitivity	26308531	10.1371/journal.pc
positive Regulation: MSI2 --+> chemosensitivity	26308531	10.1371/journal.pc
positive Regulation: TPI1 --+> chemosensitivity	31461438	10.1371/journal.pb
positive Regulation: PAX5 --+> chemosensitivity	35191417	10.1097/CM9.00000C
positive Regulation: PAX5 --+> chemosensitivity	35191417	10.1097/CM9.00000C
positive Regulation: PAX5 --+> chemosensitivity	35191417	10.1097/CM9.00000C
positive Regulation: MIR98 --+> chemosensitivity	29670086	10.1038/s41419-018
positive Regulation: MIR98 --+> chemosensitivity	30387848	10.3892/ijo.2018.4
positive Regulation: MIR98 --+> chemosensitivity		10.4149/neo_2018_1
positive Regulation: MIR98 --+> chemosensitivity	33798550	10.1016/j.lfs.2021
positive Regulation: MIR486-1 --+> chemosensitivity	30015845	10.3892/mmr.2018.9
positive Regulation: MIR486-1 --+> chemosensitivity	31646573	10.26355/eurrev_2C
positive Regulation: MIR486-1 --+> chemosensitivity	31646573	10.26355/eurrev_2C
positive Regulation: MIR486-1 --+> chemosensitivity	32774717	
positive Regulation: MIR486-1 --+> chemosensitivity	32086739	10.1007/s12017-02C
positive Regulation: MIR486-1 --+> chemosensitivity	32086739	10.1007/s12017-02C
positive Regulation: MIR486-1 --+> chemosensitivity	25655186	10.1111/hepr.1250C
positive Regulation: MIR486-1 --+> chemosensitivity	25655186	10.1111/hepr.1250C
positive Regulation: MIR486-1 --+> chemosensitivity	32086739	10.1007/s12017-02C
positive Regulation: MIR486-1 --+> chemosensitivity	32086739	10.1007/s12017-02C
positive Regulation: FOXO1 --+> chemosensitivity	28351566	10.1016/j.oralonc c
positive Regulation: FOXO1 --+> chemosensitivity	27080594	10.1158/1940-6207.
positive Regulation: FOXO1 --+> chemosensitivity	27966721	10.1039/c6mb00678g
positive Regulation: FOXO1 --+> chemosensitivity	27966721	10.1039/c6mb00678g
positive Regulation: FOXO1 --+> chemosensitivity	23615915	10.3892/ijo.2013.1
positive Regulation: FOXO1 --+> chemosensitivity	25749387	10.18632/oncotarge
positive Regulation: FOXO1 --+> chemosensitivity	27966721	10.1039/c6mb00678g
positive Regulation: FOXO1 --+> chemosensitivity	30982496	10.3727/096504018X
positive Regulation: FOXO1 --+> chemosensitivity	31754475	10.1038/s41392-019
positive Regulation: FOXO1 --+> chemosensitivity	33199698	10.1038/s41392-019
positive Regulation: FOXO1 --+> chemosensitivity	35257265	10.1007/s10495-022
positive Regulation: CBLL1 --+> chemosensitivity	29786107	10.3892/ijmm.2018.
positive Regulation: CBLL1 --+> chemosensitivity	29786107	10.3892/ijmm.2018.
positive Regulation: NME1 --+> chemosensitivity	12898755	
positive Regulation: NME1 --+> chemosensitivity	14619565	
positive Regulation: NME1 --+> chemosensitivity	17953358	
positive Regulation: NME1 --+> chemosensitivity	21875478	
positive Regulation: NME1 --+> chemosensitivity	25277180	10.18632/oncotarge
positive Regulation: NME1 --+> chemosensitivity	15296908	

positive Regulation: CUL3 --+> chemosensitivity	22825334	10.4161/cbt.21046
positive Regulation: CUL3 --+> chemosensitivity	36198437	10.1136/jitc-2022-
positive Regulation: CUL3 --+> chemosensitivity	36198437	10.1136/jitc-2022-
positive Regulation: CUL3 --+> chemosensitivity	36198437	10.1136/jitc-2022-
positive Regulation: MIR200A --+> chemosensitivity	23874841	10.1371/journal.pc
positive Regulation: MIR200A --+> chemosensitivity	25997962	10.4149/neo_2015_C
positive Regulation: MIR200A --+> chemosensitivity	25997962	10.4149/neo_2015_C
positive Regulation: MIR200A --+> chemosensitivity	25327865	
positive Regulation: MIR200A --+> chemosensitivity	25997962	10.4149/neo_2015_C
positive Regulation: MIR200A --+> chemosensitivity	25997962	10.4149/neo_2015_C
positive Regulation: MIR200A --+> chemosensitivity	29329575	10.1186/s12885-017
positive Regulation: MIR200A --+> chemosensitivity	29329575	10.1186/s12885-017
positive Regulation: MIR200A --+> chemosensitivity	29329575	10.1186/s12885-017
positive Regulation: MIR324 --+> chemosensitivity	29221202	10.18632/oncotarge
positive Regulation: MIR324 --+> chemosensitivity	35922753	10.1186/s11658-022
positive Regulation: MIR105-1 --+> chemosensitivity	28618952	10.1177/1010428317
positive Regulation: MIR105-1 --+> chemosensitivity	28618952	10.1177/1010428317
positive Regulation: MIR105-1 --+> chemosensitivity	28618952	10.1177/1010428317
positive Regulation: MIR105-1 --+> chemosensitivity	28618952	10.1177/1010428317
positive Regulation: DKK3 --+> chemosensitivity	21982838	10.1016/j.bbcan.2C
positive Regulation: DKK3 --+> chemosensitivity	25760729	10.1089/cbr.2014.1
positive Regulation: DKK3 --+> chemosensitivity	25760729	10.1089/cbr.2014.1
positive Regulation: DKK3 --+> chemosensitivity	25760729	10.1089/cbr.2014.1
positive Regulation: DKK3 --+> chemosensitivity	25573172	10.3892/or.2014.37
positive Regulation: MIR670 --+> chemosensitivity	35805884	10.3390/ijms231368
positive Regulation: DUSP26 --+> chemosensitivity	20562916	10.1038/onc.2010.2
positive Regulation: GLP1R --+> chemosensitivity	31855560	10.1530/JME-19-018
positive Regulation: STX8 --+> chemosensitivity	19075597	10.2174/1568009087
positive Regulation: CTSD --+> chemosensitivity	16331270	
positive Regulation: CTSD --+> chemosensitivity	16331270	
positive Regulation: CTSD --+> chemosensitivity	27573911	10.3892/ijmm.2016.
positive Regulation: CTSD --+> chemosensitivity	16331270	10.1038/sj.onc.12C
positive Regulation: CTSD --+> chemosensitivity	18497069	10.1007/978-0-387-
positive Regulation: ID1 --+> chemosensitivity	26797271	10.1016/j.bbrc.201
positive Regulation: ID1 --+> chemosensitivity	23308043	10.1593/neo.121044
positive Regulation: ID1 --+> chemosensitivity	20388787	10.1158/0008-5472.
positive Regulation: ID1 --+> chemosensitivity	20388787	10.1158/0008-5472.
positive Regulation: REG1A --+> chemosensitivity	24065141	10.3892/or.2013.27
positive Regulation: FAP --+> chemosensitivity	25344051	10.1186/1471-2407-
positive Regulation: MIR1243 --+> chemosensitivity	33152930	10.1016/j.biopha.2
positive Regulation: STAT1 --+> chemosensitivity	29800921	10.1016/j.biopha.2
positive Regulation: STAT1 --+> chemosensitivity	28179302	10.21873/anticanre
positive Regulation: STAT1 --+> chemosensitivity	28179302	10.21873/anticanre
positive Regulation: MAPK14 --+> chemosensitivity	19435873	
positive Regulation: SELENBP1 --+> chemosensitivity	23483240	
positive Regulation: FOXO3 --+> chemosensitivity	29518547	10.1016/j.gene.201
positive Regulation: FOXO3 --+> chemosensitivity	29518547	10.1016/j.gene.201
positive Regulation: FOXO3 --+> chemosensitivity	28260024	10.3892/or.2017.54
positive Regulation: FOXO3 --+> chemosensitivity	29099416	10.1097/CAD.00000C
positive Regulation: FOXO3 --+> chemosensitivity	31695022	10.1038/s41419-019
positive Regulation: FOXO3 --+> chemosensitivity	32645698	10.1159/000508337
positive Regulation: FOXO3 --+> chemosensitivity	23255113	10.4161/cc.22962
positive Regulation: FOXO3 --+> chemosensitivity	22936386	10.1007/s10059-012
positive Regulation: FOXO3 --+> chemosensitivity	22313691	10.1016/j.cellsig.

positive Regulation: FOXO3 --+> chemosensitivity	29518547	10.1016/j.gene.201
positive Regulation: FOXO3 --+> chemosensitivity	34510251	10.1007/s00280-021
positive Regulation: FOXO3 --+> chemosensitivity	35765274	10.3892/ol.2022.13
positive Regulation: THRB --+> chemosensitivity	25820519	10.1007/s10549-015
positive Regulation: MIR30D --+> chemosensitivity	29504819	10.1080/15384047.2
positive Regulation: TGFBI --+> chemosensitivity	19211240	10.1016/j.gde.2009
positive Regulation: TGFBI --+> chemosensitivity	26503734	10.3892/or.2015.43
positive Regulation: TGFBI --+> chemosensitivity	26503734	10.3892/or.2015.43
positive Regulation: TGFBI --+> chemosensitivity	26503734	10.3892/or.2015.43
positive Regulation: PPARA --+> chemosensitivity	31539552	10.1016/j.ejphar.2
positive Regulation: PPARG --+> chemosensitivity	22684020	10.1016/j.freeradb
positive Regulation: PPARG --+> chemosensitivity	22684020	10.1016/j.freeradb
positive Regulation: PPARG --+> chemosensitivity	32373170	10.1155/2020/64521
positive Regulation: PPARG --+> chemosensitivity	32373170	10.1155/2020/64521
positive Regulation: PPARG --+> chemosensitivity	32373170	10.1155/2020/64521
positive Regulation: PPARG --+> chemosensitivity	32373170	10.1155/2020/64521
positive Regulation: PPARG --+> chemosensitivity	32373170	10.1155/2020/64521
positive Regulation: PPARG --+> chemosensitivity	32373170	10.1155/2020/64521
positive Regulation: PPARG --+> chemosensitivity	32373170	10.1155/2020/64521
positive Regulation: PPARG --+> chemosensitivity	32373170	10.1155/2020/64521
positive Regulation: PPARG --+> chemosensitivity	25333644	10.3892/ijo.2014.2
positive Regulation: PPARG --+> chemosensitivity	25333644	10.3892/ijo.2014.2
positive Regulation: PPARG --+> chemosensitivity	32373170	10.1155/2020/64521
positive Regulation: PPARG --+> chemosensitivity	23964924	10.1089/ars.2013.5
positive Regulation: ANXA7 --+> chemosensitivity	29970503	10.21873/anticanre
positive Regulation: CLDN4 --+> chemosensitivity	24482188	10.3892/or.2014.29
positive Regulation: CLDN4 --+> chemosensitivity	31040910	10.18632/oncotarge
positive Regulation: CLDN4 --+> chemosensitivity	31498559	10.1002/cam4.25471
positive Regulation: CLDN4 --+> chemosensitivity	32086991	10.1111/cas.143611
positive Regulation: MIR23B --+> chemosensitivity	34110366	10.1093/abbs/gmab0
positive Regulation: RPL23 --+> chemosensitivity	35733701	10.1007/s10616-022
positive Regulation: RPL23 --+> chemosensitivity	35733701	10.1007/s10616-022
positive Regulation: MIR1284 --+> chemosensitivity	30257412	10.1016/j.biopha.2
positive Regulation: ADORA2B --+> chemosensitivity	30926752	10.1523/JNEUROSCI.
positive Regulation: MIR623 --+> chemosensitivity	30551491	10.1016/j.biopha.2
positive Regulation: SIRT4 --+> chemosensitivity	30008852	10.3892/ol.2018.88
positive Regulation: SIRT4 --+> chemosensitivity	30008852	10.3892/ol.2018.88
positive Regulation: ATOH8 --+> chemosensitivity	26099525	10.1053/j.gastro.2
positive Regulation: ATOH8 --+> chemosensitivity	33165366	
positive Regulation: HIPK2 --+> chemosensitivity	33650652	10.3892/or.2020.79
positive Regulation: HIPK2 --+> chemosensitivity	21602882	10.1038/onc.2011.1
positive Regulation: HIPK2 --+> chemosensitivity	21602882	10.1038/onc.2011.1
positive Regulation: HIPK2 --+> chemosensitivity	21602882	10.1038/onc.2011.1
positive Regulation: HIPK2 --+> chemosensitivity	24145406	10.1073/pnas.13100
positive Regulation: HIPK2 --+> chemosensitivity	24196445	10.4161/cc.26857
positive Regulation: HIPK2 --+> chemosensitivity	21602882	10.1038/onc.2011.1
positive Regulation: HIPK2 --+> chemosensitivity	22889244	10.1186/1756-9966-
positive Regulation: HIPK2 --+> chemosensitivity	29434701	10.3892/etm.2017.5
positive Regulation: HIPK2 --+> chemosensitivity	33613771	10.7150/jca.521151
positive Regulation: GSK3B --+> chemosensitivity	27689729	10.1016/j.ejmech.2
positive Regulation: GSK3B --+> chemosensitivity	32422572	10.1016/j.tranon.2
positive Regulation: GSK3B --+> chemosensitivity	18606491	10.1016/j.canlet.2
positive Regulation: GSK3B --+> chemosensitivity	31323761	10.3390/ijms201435
positive Regulation: GSK3B --+> chemosensitivity	31665911	10.1177/0963689719

positive Regulation: GSK3B --+> chemosensitivity	31665911	10.1177/0963689719
positive Regulation: GSK3B --+> chemosensitivity	32422572	10.1016/j.tranon.2
positive Regulation: GSK3B --+> chemosensitivity	33125126	10.3892/or.2020.78
positive Regulation: GSK3B --+> chemosensitivity	33905062	10.1007/s11626-021
positive Regulation: GSK3B --+> chemosensitivity	24407515	10.4161/cc.27728
positive Regulation: GSK3B --+> chemosensitivity	15753396	10.1158/0008-5472.
positive Regulation: GSK3B --+> chemosensitivity	26036631	10.18632/oncotarge
positive Regulation: GSK3B --+> chemosensitivity	36012631	10.3390/ijms231693
positive Regulation: MYCN --+> chemosensitivity	32820040	10.1101/gad.340133
positive Regulation: MYCN --+> chemosensitivity	19885598	10.3892/or_0000058
positive Regulation: MYCN --+> chemosensitivity	32820040	10.1101/gad.340133
positive Regulation: MYCN --+> chemosensitivity		
positive Regulation: BNIP3 --+> chemosensitivity	28607593	10.7150/jca.181711
positive Regulation: CDH2 --+> chemosensitivity	33131267	10.4081/ejh.2020.3
positive Regulation: CDH2 --+> chemosensitivity	35887090	10.3390/ijms231477
positive Regulation: PHGDH --+> chemosensitivity	30857125	10.3390/ijms200511
positive Regulation: PRKAG2 --+> chemosensitivity	23457527	10.1371/journal.pc
positive Regulation: MPO --+> chemosensitivity	18273043	10.1038/leu.2008.8
positive Regulation: MPO --+> chemosensitivity	18273043	10.1038/leu.2008.8
positive Regulation: MCPH1 --+> chemosensitivity		10.1093/annonc/mdv
positive Regulation: PHOX2B --+> chemosensitivity	30036539	10.1016/j.yexcr.20
positive Regulation: PHOX2B --+> chemosensitivity	30036539	10.1016/j.yexcr.20
positive Regulation: PHOX2B --+> chemosensitivity	16410396	10.1152/ajpregu.00
positive Regulation: PHOX2B --+> chemosensitivity	18675942	10.1016/j.resp.200
positive Regulation: PHOX2B --+> chemosensitivity	23723037	10.1002/cphy.c1000
positive Regulation: CAV1 --+> chemosensitivity	30917935	10.1016/j.ebiom.20
positive Regulation: CAV1 --+> chemosensitivity	18482795	10.1016/j.canlet.2
positive Regulation: CAV1 --+> chemosensitivity	32186270	10.2174/1381612826
positive Regulation: CAV1 --+> chemosensitivity	24131927	10.4161/cc.26809
positive Regulation: CAV1 --+> chemosensitivity	26503358	10.3892/or.2015.43
positive Regulation: CAV1 --+> chemosensitivity	23598719	10.4161/cc.2449710
positive Regulation: CAV1 --+> chemosensitivity	30146689	10.1002/jcp.271961
positive Regulation: CAV1 --+> chemosensitivity	31900313	10.1158/1541-7786.
positive Regulation: CAV1 --+> chemosensitivity	34498146	10.1007/s00432-021
positive Regulation: CAV1 --+> chemosensitivity	34498146	10.1007/s00432-021
positive Regulation: MIR199A1 --+> chemosensitivity	32634442	10.1016/j.mad.2020
positive Regulation: MIR199A1 --+> chemosensitivity	24657660	10.1016/j.canlet.2
positive Regulation: MIR199A1 --+> chemosensitivity	28079894	10.1038/cddis.2016
positive Regulation: MIR199A1 --+> chemosensitivity	29436681	10.3892/or.2018.62
positive Regulation: MIR199A1 --+> chemosensitivity	29436681	10.3892/or.2018.62
positive Regulation: MIR199A1 --+> chemosensitivity	29204706	10.1007/s00345-017
positive Regulation: MIR199A1 --+> chemosensitivity		
positive Regulation: MIR199A1 --+> chemosensitivity	31636666	10.1155/2019/56134
positive Regulation: MIR199A1 --+> chemosensitivity	31636666	10.1155/2019/56134
positive Regulation: MIR199A1 --+> chemosensitivity	33245712	10.12659/MSM.92402
positive Regulation: MIR199A1 --+> chemosensitivity	30575906	10.26355/eurrev_20
positive Regulation: MIR199A1 --+> chemosensitivity	33526055	10.1186/s13046-021
positive Regulation: MIR199A1 --+> chemosensitivity	28126676	10.1016/j.biopha.2
positive Regulation: MIR199A1 --+> chemosensitivity	31898515	10.1186/s13046-019
positive Regulation: MIR199A1 --+> chemosensitivity		10.1155/2019/56134
positive Regulation: MIR199A1 --+> chemosensitivity		10.1155/2019/56134
positive Regulation: MIR199A1 --+> chemosensitivity		
positive Regulation: MIR199A1 --+> chemosensitivity		10.12659/MSM.92402
positive Regulation: MIR199A1 --+> chemosensitivity		10.26355/eurrev_20

positive Regulation: MIR199A1 --+> chemosensitivity	35054811	10.3390/ijms230206
positive Regulation: MIR199A1 --+> chemosensitivity		
positive Regulation: MIR199A1 --+> chemosensitivity		
positive Regulation: MIR199A1 --+> chemosensitivity	24240682	10.1038/onc.2013.4
positive Regulation: MIR199A1 --+> chemosensitivity	35676254	10.1038/s41419-022
positive Regulation: MIR199A1 --+> chemosensitivity	35082400	10.1038/s41417-022
positive Regulation: CHEK2 --+> chemosensitivity	17118344	10.1016/j.bcp.2006
positive Regulation: CHEK2 --+> chemosensitivity	17118344	10.1016/j.bcp.2006
positive Regulation: CHEK2 --+> chemosensitivity	17940507	
positive Regulation: CHEK2 --+> chemosensitivity	29352124	10.1038/s41419-017
positive Regulation: CHEK2 --+> chemosensitivity	23818585	10.1073/pnas.12208
positive Regulation: CHEK2 --+> chemosensitivity	29352124	10.1038/s41419-017
positive Regulation: MIR326 --+> chemosensitivity	27460077	10.1007/s13277-016
positive Regulation: MIR326 --+> chemosensitivity	28627598	10.3892/ijo.2017.4
positive Regulation: MIR326 --+> chemosensitivity	28713953	10.3892/or.2017.58
positive Regulation: MIR326 --+> chemosensitivity	30764896	10.3727/096504018X
positive Regulation: MIR326 --+> chemosensitivity	30764896	10.3727/096504018X
positive Regulation: MIR326 --+> chemosensitivity	30764896	10.3727/096504018X
positive Regulation: MIR326 --+> chemosensitivity	30764896	10.3727/096504018X
positive Regulation: MIR326 --+> chemosensitivity	31651347	10.1186/s12943-019
positive Regulation: MIR326 --+> chemosensitivity	30764896	10.3727/096504018X
positive Regulation: MIR326 --+> chemosensitivity		10.3727/096504018X
positive Regulation: MIR326 --+> chemosensitivity		10.3727/096504018X
positive Regulation: MIR326 --+> chemosensitivity		10.3727/096504018X
positive Regulation: MIR326 --+> chemosensitivity		10.3727/096504018X
positive Regulation: MIR326 --+> chemosensitivity		10.1186/s12943-019
positive Regulation: MIR326 --+> chemosensitivity	33176632	10.2174/0929867327
positive Regulation: BTG1 --+> chemosensitivity		10.1007/s12038-020
positive Regulation: MIR664B --+> chemosensitivity	28176879	10.1038/srep423191
positive Regulation: MIR664B --+> chemosensitivity	28176879	10.1038/srep423191
positive Regulation: MIR588 --+> chemosensitivity	29187727	10.12659/MSM.90512
positive Regulation: MIR588 --+> chemosensitivity	29187727	10.12659/MSM.90512
positive Regulation: MIR588 --+> chemosensitivity	29187727	10.12659/MSM.90512
positive Regulation: MIR588 --+> chemosensitivity	29187727	10.12659/MSM.90512
positive Regulation: MIR588 --+> chemosensitivity	29187727	10.12659/msm.90512
positive Regulation: IGFBP7 --+> chemosensitivity	24513543	10.1016/j.cca.2014
positive Regulation: IGFBP7 --+> chemosensitivity	25984556	10.1016/j.gendis.2
positive Regulation: IGFBP7 --+> chemosensitivity	23600329	
positive Regulation: IGFBP7 --+> chemosensitivity	23600329	
positive Regulation: MIR708 --+> chemosensitivity	32721464	10.1016/j.lfs.2020
positive Regulation: MIR708 --+> chemosensitivity	33865701	10.1016/j.semcdb.2
positive Regulation: MIR708 --+> chemosensitivity		10.32604/BIOCELL.2
positive Regulation: MIR708 --+> chemosensitivity	31273952	10.1111/jcmm.14462
positive Regulation: MIR200C --+> chemosensitivity	32818543	10.1016/j.lfs.2020
positive Regulation: MIR200C --+> chemosensitivity	29698868	10.1016/j.biomater
positive Regulation: MIR200C --+> chemosensitivity	30921705	10.1016/j.biopha.2
positive Regulation: MIR200C --+> chemosensitivity	23757365	10.1016/j.drup.201
positive Regulation: MIR200C --+> chemosensitivity	25281917	10.1016/j.addr.201
positive Regulation: MIR200C --+> chemosensitivity	25958353	10.1016/j.phrs.201
positive Regulation: MIR200C --+> chemosensitivity	26192966	10.1016/j.semcance
positive Regulation: MIR200C --+> chemosensitivity	26257289	10.1016/j.critrevc
positive Regulation: MIR200C --+> chemosensitivity	28724364	10.1186/s12885-017
positive Regulation: MIR200C --+> chemosensitivity	30226594	10.3892/ijo.2018.4
positive Regulation: MIR200C --+> chemosensitivity	30272330	10.3892/or.2018.67

positive Regulation: MIR200C --+> chemosensitivity	30272330	10.3892/or.2018.67
positive Regulation: MIR200C --+> chemosensitivity	30272330	10.3892/or.2018.67
positive Regulation: MIR200C --+> chemosensitivity	30087437	10.1038/s41388-018
positive Regulation: MIR200C --+> chemosensitivity	33650661	10.3892/or.2021.79
positive Regulation: MIR200C --+> chemosensitivity	23546450	10.3892/mmr.2013.1
positive Regulation: MIR200C --+> chemosensitivity	23834156	10.2174/1389450111
positive Regulation: MIR200C --+> chemosensitivity	25052237	10.1007/s12032-014
positive Regulation: MIR200C --+> chemosensitivity	25322930	10.3892/or.2014.35
positive Regulation: MIR200C --+> chemosensitivity	26202679	10.3892/ijo.2015.3
positive Regulation: MIR200C --+> chemosensitivity		
positive Regulation: MIR200C --+> chemosensitivity	19435871	10.1158/1535-7163.
positive Regulation: MIR200C --+> chemosensitivity	22101791	10.1007/s12032-011
positive Regulation: MIR200C --+> chemosensitivity	22991189	10.1002/jcb.243981
positive Regulation: MIR200C --+> chemosensitivity	23074172	10.1158/1535-7163.
positive Regulation: MIR200C --+> chemosensitivity	25746005	10.1038/onc.2015.4
positive Regulation: MIR200C --+> chemosensitivity	25746005	10.1038/onc.2015.4
positive Regulation: MIR200C --+> chemosensitivity	25746005	10.1038/onc.2015.4
positive Regulation: MIR200C --+> chemosensitivity	29051585	10.1038/s41598-017
positive Regulation: MIR200C --+> chemosensitivity	29051585	10.1038/s41598-017
positive Regulation: MIR200C --+> chemosensitivity	34440380	10.3390/genes12081
positive Regulation: KAT5 --+> chemosensitivity	26808162	10.1016/j.pharmthe
positive Regulation: KAT5 --+> chemosensitivity	26808162	10.1016/j.pharmthe
positive Regulation: KAT5 --+> chemosensitivity	22673729	10.1038/jid.2012.1
positive Regulation: MUC20 --+> chemosensitivity	26673820	10.18632/oncotarge
positive Regulation: VPS37A --+> chemosensitivity	31152734	10.1016/j.cbi.2019
positive Regulation: MRPL33 --+> chemosensitivity	30816492	10.3892/ijo.2019.4
positive Regulation: MRPL33 --+> chemosensitivity	30816492	10.3892/ijo.2019.4
positive Regulation: C1orf109 --+> chemosensitivity	32761833	10.1111/cpr.128751
positive Regulation: C1orf109 --+> chemosensitivity	32761833	10.1111/cpr.128751
positive Regulation: NOD2 --+> chemosensitivity	32659252	10.1016/j.bbcan.20
positive Regulation: NOD2 --+> chemosensitivity	32144252	10.1038/s41419-020
positive Regulation: SNX1 --+> chemosensitivity	26643894	10.1007/s13277-015
positive Regulation: SNX1 --+> chemosensitivity	26643894	10.1007/s13277-015
positive Regulation: BRCA1 --+> chemosensitivity	19269106	10.1016/j.ctrv.200
positive Regulation: BRCA1 --+> chemosensitivity	20060649	10.1016/j.ctrv.200
positive Regulation: BRCA1 --+> chemosensitivity		10.1016/j.biopha.2
positive Regulation: BRCA1 --+> chemosensitivity	23435357	10.1016/j.bcp.2013
positive Regulation: BRCA1 --+> chemosensitivity	23500037	10.1016/j.bbagrm.2
positive Regulation: BRCA1 --+> chemosensitivity	17229870	
positive Regulation: BRCA1 --+> chemosensitivity	17229870	
positive Regulation: BRCA1 --+> chemosensitivity	12698198	
positive Regulation: BRCA1 --+> chemosensitivity	11781837	
positive Regulation: BRCA1 --+> chemosensitivity	11781837	
positive Regulation: BRCA1 --+> chemosensitivity	11781837	
positive Regulation: BRCA1 --+> chemosensitivity	11781837	
positive Regulation: BRCA1 --+> chemosensitivity	11781837	
positive Regulation: BRCA1 --+> chemosensitivity	11781837	
positive Regulation: BRCA1 --+> chemosensitivity	11781837	
positive Regulation: BRCA1 --+> chemosensitivity	11781837	
positive Regulation: BRCA1 --+> chemosensitivity	11781837	
positive Regulation: BRCA1 --+> chemosensitivity	11781837	
positive Regulation: BRCA1 --+> chemosensitivity	11781837	
positive Regulation: BRCA1 --+> chemosensitivity	11781837	
positive Regulation: BRCA1 --+> chemosensitivity	11781837	

positive Regulation: BRCA1 --+> chemosensitivity	11521194	
positive Regulation: BRCA1 --+> chemosensitivity	11521194	
positive Regulation: BRCA1 --+> chemosensitivity	11521194	
positive Regulation: BRCA1 --+> chemosensitivity	11521194	
positive Regulation: BRCA1 --+> chemosensitivity	11521194	
positive Regulation: BRCA1 --+> chemosensitivity	11521194	
positive Regulation: BRCA1 --+> chemosensitivity	11521194	
positive Regulation: BRCA1 --+> chemosensitivity	11521194	
positive Regulation: BRCA1 --+> chemosensitivity	11521194	
positive Regulation: BRCA1 --+> chemosensitivity	11521194	
positive Regulation: BRCA1 --+> chemosensitivity	11521194	
positive Regulation: BRCA1 --+> chemosensitivity	11521194	
positive Regulation: BRCA1 --+> chemosensitivity	11521194	
positive Regulation: BRCA1 --+> chemosensitivity	11521194	
positive Regulation: BRCA1 --+> chemosensitivity	17993233	10.1007/978-0-387-
positive Regulation: BRCA1 --+> chemosensitivity	21119513	10.1097/CCO.0b013e
positive Regulation: BRCA1 --+> chemosensitivity	24657936	10.4161/cbt.28556
positive Regulation: BRCA1 --+> chemosensitivity	25758301	10.1016/j.bulcan.2
positive Regulation: BRCA1 --+> chemosensitivity	11521194	10.1038/sj.onc.120
positive Regulation: BRCA1 --+> chemosensitivity	11781837	10.1038/sj.onc.120
positive Regulation: BRCA1 --+> chemosensitivity	12698198	10.1038/sj.bjc.660
positive Regulation: BRCA1 --+> chemosensitivity	16739340	
positive Regulation: BRCA1 --+> chemosensitivity	17229870	10.1124/mol.106.02
positive Regulation: BRCA1 --+> chemosensitivity	19551867	10.1002/ijc.246841
positive Regulation: BRCA1 --+> chemosensitivity	20487263	10.1111/j.1349-700
positive Regulation: BRCA1 --+> chemosensitivity	20730959	10.1002/cbdv.20090
positive Regulation: BRCA1 --+> chemosensitivity	24131965	10.1093/annonc/mdt
positive Regulation: BRCA1 --+> chemosensitivity	25758301	10.1016/j.bulcan.2
positive Regulation: BRCA1 --+> chemosensitivity	33670664	10.3390/biomedicin
positive Regulation: BRCA1 --+> chemosensitivity	19150941	10.1093/annonc/mdn
positive Regulation: KEAP1 --+> chemosensitivity	22684020	10.1016/j.freeradb
positive Regulation: KEAP1 --+> chemosensitivity	28129239	10.1097/IGC.000000
positive Regulation: KEAP1 --+> chemosensitivity		10.1097/IGC.000000
positive Regulation: POU1F1 --+> chemosensitivity	34022282	10.1016/j.canlet.2
positive Regulation: RBM5 --+> chemosensitivity	22609235	10.1016/j.canep.20
positive Regulation: RBM5 --+> chemosensitivity	26923134	10.1186/s12957-016
positive Regulation: MEG3 --+> chemosensitivity	30173893	10.1016/j.bbrc.201
positive Regulation: MEG3 --+> chemosensitivity	31790697	10.1016/j.cca.2019
positive Regulation: MEG3 --+> chemosensitivity	29355660	10.1016/j.canlet.2
positive Regulation: MEG3 --+> chemosensitivity	30119236	10.1016/j.biopha.2
positive Regulation: MEG3 --+> chemosensitivity	29328401	10.3892/or.2018.61
positive Regulation: MEG3 --+> chemosensitivity	29328401	10.3892/or.2018.61
positive Regulation: MEG3 --+> chemosensitivity	29328401	10.3892/or.2018.61
positive Regulation: MEG3 --+> chemosensitivity	30439718	10.1159/000495168
positive Regulation: MEG3 --+> chemosensitivity	29940769	10.4149/neo_2018_1
positive Regulation: MEG3 --+> chemosensitivity	29940769	10.4149/neo_2018_1
positive Regulation: MEG3 --+> chemosensitivity	29940769	10.4149/neo_2018_1
positive Regulation: MEG3 --+> chemosensitivity	29940769	10.4149/neo_2018_1
positive Regulation: MEG3 --+> chemosensitivity	32472764	10.2741/4884
positive Regulation: MEG3 --+> chemosensitivity	32856219	10.1007/s10753-020
positive Regulation: MEG3 --+> chemosensitivity	26059239	10.3892/mmr.2015.3
positive Regulation: MEG3 --+> chemosensitivity	27121324	10.3892/or.2016.47
positive Regulation: MEG3 --+> chemosensitivity	27121324	10.3892/or.2016.47
positive Regulation: MEG3 --+> chemosensitivity	25992654	10.1371/journal.pc

positive Regulation: MEG3 --+> chemosensitivity	25992654	10.1371/journal.pc
positive Regulation: MEG3 --+> chemosensitivity	28677749	10.3892/mmr.2017.6
positive Regulation: MEG3 --+> chemosensitivity	32618397	10.1002/jcla.23369
positive Regulation: MEG3 --+> chemosensitivity	29328401	10.3892/or.2018.61
positive Regulation: MEG3 --+> chemosensitivity		10.1016/j.gendis.2
positive Regulation: MEG3 --+> chemosensitivity		10.1016/j.gendis.2
positive Regulation: MEG3 --+> chemosensitivity	35184425	10.3349/ymj.2022.6
positive Regulation: MEG3 --+> chemosensitivity		10.1016/j.gendis.2
positive Regulation: MEG3 --+> chemosensitivity	35761379	10.1186/s12967-022
positive Regulation: CISD2 --+> chemosensitivity	32633348	10.26355/eurrev_20
positive Regulation: KLF3-AS1 --+> chemosensitivity	34745937	10.3389/fonc.2021.
positive Regulation: MIR17 --+> chemosensitivity	29953965	10.1016/j.biocel.2
positive Regulation: MIR17 --+> chemosensitivity	26554910	10.1016/j.jgg.2015
positive Regulation: MIR17 --+> chemosensitivity	30249919	10.2176/nmc.ra.201
positive Regulation: MIR17 --+> chemosensitivity	33390839	10.7150/ijbs.50773
positive Regulation: MIR17 --+> chemosensitivity	33462751	10.1007/s10863-020
positive Regulation: MIR17 --+> chemosensitivity	29953965	10.1016/j.biocel.2
positive Regulation: MIR184 --+> chemosensitivity	33689813	10.1016/j.micpath.
positive Regulation: PDCD5 --+> chemosensitivity	22688731	10.4161/cbt.20565
positive Regulation: PDCD5 --+> chemosensitivity	22688731	10.4161/cbt.20565
positive Regulation: PDCD5 --+> chemosensitivity	22688731	10.4161/cbt.20565
positive Regulation: PDCD5 --+> chemosensitivity	25625867	10.3892/mmr.2015.3
positive Regulation: PDCD5 --+> chemosensitivity	25625867	10.3892/mmr.2015.3
positive Regulation: PDCD5 --+> chemosensitivity	26077467	10.1038/ncomms8390
positive Regulation: PDCD5 --+> chemosensitivity	26077467	10.1038/ncomms8390
positive Regulation: PDCD5 --+> chemosensitivity	22688731	10.4161/cbt.20565
positive Regulation: PDCD5 --+> chemosensitivity	24219296	10.1139/bcb-2013-0
positive Regulation: PDCD5 --+> chemosensitivity	25625867	10.3892/mmr.2015.3
positive Regulation: MIR520B --+> chemosensitivity	32355540	
positive Regulation: MIR520B --+> chemosensitivity	32355540	
positive Regulation: MIR638 --+> chemosensitivity	34257614	10.3389/pore.2021.
positive Regulation: MIR34A --+> chemosensitivity	32243871	10.1016/j.ejphar.2
positive Regulation: MIR34A --+> chemosensitivity	27591936	10.1016/j.canlet.2
positive Regulation: MIR34A --+> chemosensitivity	29102917	10.1016/j.biopha.2
positive Regulation: MIR34A --+> chemosensitivity	30551433	10.1016/j.biopha.2
positive Regulation: MIR34A --+> chemosensitivity	33894312	10.1016/j.cellsig.
positive Regulation: MIR34A --+> chemosensitivity	27263934	10.1016/j.critrevo
positive Regulation: MIR34A --+> chemosensitivity	31158463	10.1016/j.semcance
positive Regulation: MIR34A --+> chemosensitivity	29723707	10.1016/j.dnarep.2
positive Regulation: MIR34A --+> chemosensitivity	31760377	10.1016/j.omtn.201
positive Regulation: MIR34A --+> chemosensitivity	32304779	10.1016/j.jcmgh.20
positive Regulation: MIR34A --+> chemosensitivity	21145728	10.1016/j.ejca.201
positive Regulation: MIR34A --+> chemosensitivity	24786471	10.1016/j.febslet.
positive Regulation: MIR34A --+> chemosensitivity	30001529	10.1159/000491665
positive Regulation: MIR34A --+> chemosensitivity	30001529	10.1159/000491665
positive Regulation: MIR34A --+> chemosensitivity	30106161	10.3892/mmr.2018.9
positive Regulation: MIR34A --+> chemosensitivity	30282072	10.1159/000494004
positive Regulation: MIR34A --+> chemosensitivity	30282072	10.1159/000494004
positive Regulation: MIR34A --+> chemosensitivity	30365088	10.3892/mmr.2018.9
positive Regulation: MIR34A --+> chemosensitivity	30374014	10.12659/MSM.91158
positive Regulation: MIR34A --+> chemosensitivity	30387834	10.3892/ijo.2018.4
positive Regulation: MIR34A --+> chemosensitivity	32626991	10.3892/mmr.2020.1
positive Regulation: MIR34A --+> chemosensitivity	32626991	10.3892/mmr.2020.1
positive Regulation: MIR34A --+> chemosensitivity	32626991	10.3892/mmr.2020.1

positive Regulation: MIR34A --+> chemosensitivity	32504411	10.1007/s12079-020
positive Regulation: MIR34A --+> chemosensitivity	33562604	10.3390/ijms220416
positive Regulation: MIR34A --+> chemosensitivity	34103477	10.1038/s41419-021
positive Regulation: MIR34A --+> chemosensitivity	33423646	10.2174/1568009620
positive Regulation: MIR34A --+> chemosensitivity	23497288	10.1186/1475-2867-
positive Regulation: MIR34A --+> chemosensitivity	23824643	10.1007/s12032-013
positive Regulation: MIR34A --+> chemosensitivity	24418846	10.4161/auto.27418
positive Regulation: MIR34A --+> chemosensitivity	24423412	10.1186/1476-4598-
positive Regulation: MIR34A --+> chemosensitivity	25179842	10.1007/s13277-014
positive Regulation: MIR34A --+> chemosensitivity	26371779	10.1097/CCO.000000
positive Regulation: MIR34A --+> chemosensitivity	26499184	10.3892/or.2015.43
positive Regulation: MIR34A --+> chemosensitivity	26499184	10.3892/or.2015.43
positive Regulation: MIR34A --+> chemosensitivity	26782555	10.4238/2015.Decem
positive Regulation: MIR34A --+> chemosensitivity	21960059	10.1002/path.30071
positive Regulation: MIR34A --+> chemosensitivity	23085450	10.1016/j.arcmed.2
positive Regulation: MIR34A --+> chemosensitivity	24418846	10.4161/auto.27418
positive Regulation: MIR34A --+> chemosensitivity	25783790	10.1111/cas.126561
positive Regulation: MIR34A --+> chemosensitivity	25783790	10.1111/cas.126561
positive Regulation: MIR34A --+> chemosensitivity	26499184	10.3892/or.2015.43
positive Regulation: MIR34A --+> chemosensitivity	26499184	10.3892/or.2015.43
positive Regulation: MIR34A --+> chemosensitivity	30001529	10.1159/0004916651
positive Regulation: MIR34A --+> chemosensitivity	30001529	10.1159/0004916651
positive Regulation: MIR34A --+> chemosensitivity	30001529	10.1159/0004916651
positive Regulation: MIR34A --+> chemosensitivity	30282072	10.1159/0004940041
positive Regulation: MIR34A --+> chemosensitivity	31332647	10.1007/s13402-019
positive Regulation: MIR34A --+> chemosensitivity	32044957	10.1093/jb/mvaa012
positive Regulation: MIR34A --+> chemosensitivity	34440380	10.3390/genes12081
positive Regulation: MIR34A --+> chemosensitivity		10.4161/auto.27418
positive Regulation: MIR34A --+> chemosensitivity		10.1097/CCO.000000
positive Regulation: MIR34A --+> chemosensitivity	22762204	10.1042/BSR2011012
positive Regulation: MIR34A --+> chemosensitivity		
positive Regulation: MIR34A --+> chemosensitivity		
positive Regulation: MIR34A --+> chemosensitivity	33959850	10.1007/s10555-021
positive Regulation: MIR34A --+> chemosensitivity	34861419	10.1016/j.preteyer
positive Regulation: MIR34A --+> chemosensitivity		10.31083/j.ceog490
positive Regulation: MIR34A --+> chemosensitivity		10.31083/j.ceog490
positive Regulation: MIR34A --+> chemosensitivity		10.31083/j.ceog490
positive Regulation: MIR34A --+> chemosensitivity		10.31083/j.ceog490
positive Regulation: MIR34A --+> chemosensitivity		10.31083/j.ceog490
positive Regulation: MIR34A --+> chemosensitivity		10.31083/j.ceog490
positive Regulation: MIR34A --+> chemosensitivity		10.31083/j.ceog490
positive Regulation: MIR34A --+> chemosensitivity	35834883	10.1016/j.prp.2022
positive Regulation: MIR34A --+> chemosensitivity	34629041	10.2174/1389450122
positive Regulation: DDRGK1 --+> chemosensitivity	33219317	10.1038/s41388-020
positive Regulation: HDAC2 --+> chemosensitivity	33872660	10.1016/j.lfs.2021
positive Regulation: HDAC2 --+> chemosensitivity	22154511	10.1016/j.yexcr.20
positive Regulation: HDAC2 --+> chemosensitivity	22154511	10.1016/j.yexcr.20
positive Regulation: SLC38A3 --+> chemosensitivity	27467507	10.1371/journal.po
positive Regulation: SLC38A3 --+> chemosensitivity	27467507	10.1371/journal.po
positive Regulation: MIR570 --+> chemosensitivity	34440380	10.3390/genes12081
positive Regulation: MIR29A --+> chemosensitivity	24210072	10.1016/j.biopha.2
positive Regulation: MIR29A --+> chemosensitivity	28347239	10.1177/1010428317
positive Regulation: MIR29A --+> chemosensitivity	29217524	10.1042/BSR2017126
positive Regulation: SPRY2 --+> chemosensitivity	29373811	10.1016/j.abb.2018

positive Regulation: DCN --+> chemosensitivity	25550184	10.1016/j.bbrc.201
positive Regulation: DCN --+> chemosensitivity	25550184	10.1016/j.bbrc.201
positive Regulation: DCN --+> chemosensitivity	28631095	10.1007/s10637-017
positive Regulation: DCN --+> chemosensitivity	28631095	10.1007/s10637-017
positive Regulation: DCN --+> chemosensitivity	28631095	10.1007/s10637-017
positive Regulation: MMP3 --+> chemosensitivity	31432178	10.3892/or.2019.72
positive Regulation: CXCL12 --+> chemosensitivity	29903571	10.1016/j.cyto.201
positive Regulation: CXCL12 --+> chemosensitivity	31096215	10.1159/000497430
positive Regulation: CXCL12 --+> chemosensitivity	29903571	10.1016/j.cyto.201
positive Regulation: CXCL12 --+> chemosensitivity	30453100	10.1016/j.leukres.
positive Regulation: MIR498 --+> chemosensitivity	33186139	10.1097/CAD.000000
positive Regulation: TIMP2 --+> chemosensitivity	23474755	10.1038/onc.2013.6
positive Regulation: TIMP2 --+> chemosensitivity	23474755	10.1038/onc.2013.6
positive Regulation: TIMP2 --+> chemosensitivity	23474755	10.1038/onc.2013.6
positive Regulation: TIMP2 --+> chemosensitivity	35586209	10.1155/2022/32497
positive Regulation: TIMP2 --+> chemosensitivity	36585738	10.1186/s12935-022
positive Regulation: bradykinin --+> chemosensitivity	25914087	10.1016/j.lfs.2015
positive Regulation: MIR373 --+> chemosensitivity	27512943	10.1016/j.biomater
positive Regulation: MIR373 --+> chemosensitivity	30021382	10.1016/j.biopha.2
positive Regulation: MIR373 --+> chemosensitivity	30021382	10.1016/j.biopha.2
positive Regulation: MIR373 --+> chemosensitivity	30021382	10.1016/j.biopha.2
positive Regulation: MIR373 --+> chemosensitivity	34839317	10.1097/CM9.000000
positive Regulation: ERRFI1 --+> chemosensitivity	21190978	10.1210/jc.2010-18
positive Regulation: ING1 --+> chemosensitivity	12012016	
positive Regulation: ING1 --+> chemosensitivity	15191662	
positive Regulation: ING1 --+> chemosensitivity	15662138	10.4161/cbt.4.1.13
positive Regulation: ING1 --+> chemosensitivity	16325212	10.1016/j.lfs.2005
positive Regulation: ING1 --+> chemosensitivity	32831651	10.1186/s12935-020
positive Regulation: ING1 --+> chemosensitivity		
positive Regulation: ING1 --+> chemosensitivity		
positive Regulation: ING1 --+> chemosensitivity		
positive Regulation: ING1 --+> chemosensitivity	34786051	
positive Regulation: CASP3 --+> chemosensitivity	33711308	10.1016/j.ejphar.2
positive Regulation: CASP3 --+> chemosensitivity	19047049	10.1074/jbc.M80670
positive Regulation: CASP3 --+> chemosensitivity	32422231	10.1016/j.exphem.2
positive Regulation: CASP3 --+> chemosensitivity	23974068	10.1016/j.drudis.2
positive Regulation: CASP3 --+> chemosensitivity	19047049	
positive Regulation: CASP3 --+> chemosensitivity	11420687	
positive Regulation: CASP3 --+> chemosensitivity	24535252	10.3892/ijo.2014.2
positive Regulation: CASP3 --+> chemosensitivity	11420687	10.1038/sj.onc.120
positive Regulation: CASP3 --+> chemosensitivity	30774365	10.2147/OTT.S19123
positive Regulation: CASP3 --+> chemosensitivity	33363619	10.3892/ol.2020.12
positive Regulation: HBEGF --+> chemosensitivity	34360915	10.3390/ijms221581
positive Regulation: IRF1 --+> chemosensitivity	30980967	10.1016/j.pvr.2019
positive Regulation: IRF1 --+> chemosensitivity	24632547	10.1097/MPA.000000
positive Regulation: IRF1 --+> chemosensitivity	24632547	10.1097/MPA.000000
positive Regulation: IRF1 --+> chemosensitivity	24632547	10.1097/MPA.000000
positive Regulation: IRF1 --+> chemosensitivity	22531515	10.4103/0973-1482.
positive Regulation: IRF1 --+> chemosensitivity		10.1097/MPA.000000
positive Regulation: IRF1 --+> chemosensitivity		10.1097/MPA.000000
positive Regulation: IRF1 --+> chemosensitivity		10.1097/MPA.000000
positive Regulation: IRF1 --+> chemosensitivity	35092496	10.1007/s12032-021
positive Regulation: ARNTL --+> chemosensitivity	30621723	10.1186/s13046-018
positive Regulation: ARNTL --+> chemosensitivity	30959483	10.1530/ERC-19-009

positive Regulation: ARNTL --+> chemosensitivity	33824475	10.1038/s41388-021
positive Regulation: ARNTL --+> chemosensitivity	25175925	10.3892/ijo.2014.2
positive Regulation: ARNTL --+> chemosensitivity	25175925	10.3892/ijo.2014.2
positive Regulation: ARNTL --+> chemosensitivity	25175925	10.3892/ijo.2014.2
positive Regulation: ARNTL --+> chemosensitivity	25175925	10.3892/ijo.2014.2
positive Regulation: ARNTL --+> chemosensitivity	26253128	10.1186/s40880-015
positive Regulation: ARNTL --+> chemosensitivity	25175925	10.3892/ijo.2014.2
positive Regulation: ARNTL --+> chemosensitivity		10.1186/s13046-018
positive Regulation: ARNTL --+> chemosensitivity		10.1530/ERC-19-009
positive Regulation: MIR181B1 --+> chemosensitivity	29360452	10.1016/j.bbrc.201
positive Regulation: MIR181B1 --+> chemosensitivity	30119173	10.1016/j.biopha.2
positive Regulation: MIR181B1 --+> chemosensitivity	30551476	10.1016/j.biopha.2
positive Regulation: MIR181B1 --+> chemosensitivity	30551476	10.1016/j.biopha.2
positive Regulation: MIR181B1 --+> chemosensitivity	30551476	10.1016/j.biopha.2
positive Regulation: MIR181B1 --+> chemosensitivity	28501897	10.1007/s11060-017
positive Regulation: MIR181B1 --+> chemosensitivity	28501897	10.1007/s11060-017
positive Regulation: MIR181B1 --+> chemosensitivity	28501897	10.1007/s11060-017
positive Regulation: MIR181B1 --+> chemosensitivity	28501897	10.1007/s11060-017
positive Regulation: MIR181B1 --+> chemosensitivity	28501897	10.1007/s11060-017
positive Regulation: MIR181B1 --+> chemosensitivity	28501897	10.1007/s11060-017
positive Regulation: MIR181B1 --+> chemosensitivity	28501897	10.1007/s11060-017
positive Regulation: MIR181B1 --+> chemosensitivity	28501897	10.1007/s11060-017
positive Regulation: MIR181B1 --+> chemosensitivity	29725260	10.7150/ijbs.22243
positive Regulation: MIR181B1 --+> chemosensitivity	30182330	10.1007/s12017-018
positive Regulation: MIR181B1 --+> chemosensitivity	31087138	10.1007/s00280-019
positive Regulation: MIR181B1 --+> chemosensitivity	31858559	10.26355/eurrev_20
positive Regulation: MIR181B1 --+> chemosensitivity	23645289	10.1007/s00280-013
positive Regulation: MIR181B1 --+> chemosensitivity	26620926	10.1038/srep17618
positive Regulation: MIR181B1 --+> chemosensitivity	26620926	10.1038/srep176181
positive Regulation: MIR181B1 --+> chemosensitivity	26620926	10.1038/srep176181
positive Regulation: MIR181B1 --+> chemosensitivity	26620926	10.1038/srep176181
positive Regulation: MIR181B1 --+> chemosensitivity	28501897	10.1007/s11060-017
positive Regulation: MIR181B1 --+> chemosensitivity	30551476	10.1016/j.biopha.2
positive Regulation: MIR181B1 --+> chemosensitivity	35198024	10.1155/2022/24022
positive Regulation: MIR181B1 --+> chemosensitivity	35457012	10.3390/ijms230841
positive Regulation: MIR200B --+> chemosensitivity	28823964	10.1016/j.canlet.2
positive Regulation: MIR200B --+> chemosensitivity	33892053	10.1016/j.bbcan.20
positive Regulation: MIR200B --+> chemosensitivity	32187964	10.1016/j.biopha.2
positive Regulation: MIR200B --+> chemosensitivity	34023767	10.1016/j.ctarc.20
positive Regulation: MIR200B --+> chemosensitivity	34023767	10.1016/j.ctarc.20
positive Regulation: MIR200B --+> chemosensitivity	22795796	10.1016/j.biopha.2
positive Regulation: MIR200B --+> chemosensitivity	28152297	10.14348/molcells.
positive Regulation: MIR200B --+> chemosensitivity	29534583	10.4149/neo_2018_1
positive Regulation: MIR200B --+> chemosensitivity	30628651	10.3892/ijo.2019.4
positive Regulation: MIR200B --+> chemosensitivity	33390839	10.7150/ijbs.50773
positive Regulation: MIR200B --+> chemosensitivity	23806890	10.4161/rna.25481
positive Regulation: MIR200B --+> chemosensitivity	24317363	10.3892/or.2013.28
positive Regulation: MIR200B --+> chemosensitivity	24317363	10.3892/or.2013.28
positive Regulation: MIR200B --+> chemosensitivity	24317363	10.3892/or.2013.28
positive Regulation: MIR200B --+> chemosensitivity	24447584	10.1186/1479-5876-
positive Regulation: MIR200B --+> chemosensitivity	25065598	10.1038/onc.2014.2
positive Regulation: MIR200B --+> chemosensitivity	26648487	10.3892/ijo.2015.3
positive Regulation: MIR200B --+> chemosensitivity	24317363	10.3892/or.2013.28
positive Regulation: MIR200B --+> chemosensitivity	24317363	10.3892/or.2013.28

positive Regulation: MIR200B --+> chemosensitivity	27027446	10.18632/oncotarge
positive Regulation: KIZ --+> chemosensitivity	33000253	10.3892/or.2020.77
positive Regulation: KLF10 --+> chemosensitivity	18930345	10.1016/j.canlet.2
positive Regulation: KLF10 --+> chemosensitivity	18930345	10.1016/j.canlet.2
positive Regulation: MIR181A1 --+> chemosensitivity	28823541	10.1016/j.toxlet.2
positive Regulation: MIR181A1 --+> chemosensitivity	28823541	10.1016/j.toxlet.2
positive Regulation: MIR181A1 --+> chemosensitivity	24183997	10.1016/j.yexcr.20
positive Regulation: MIR181A1 --+> chemosensitivity	24183997	10.1016/j.yexcr.20
positive Regulation: MIR181A1 --+> chemosensitivity	30309296	10.1177/1933719118
positive Regulation: MIR181A1 --+> chemosensitivity	31832711	10.1007/s00253-019
positive Regulation: MIR181A1 --+> chemosensitivity	32875398	10.1007/s10565-020
positive Regulation: MIR181A1 --+> chemosensitivity	22762204	10.1042/BSR2011012
positive Regulation: MIR181A1 --+> chemosensitivity	24573637	10.1007/s12032-014
positive Regulation: MIR181A1 --+> chemosensitivity	31737213	
positive Regulation: MIR181A1 --+> chemosensitivity	32810491	10.1016/j.ejphar.2
positive Regulation: MIR181A1 --+> chemosensitivity	26910006	10.4238/gmr.150177
positive Regulation: MIR181A1 --+> chemosensitivity	24573637	10.1007/s12032-014
positive Regulation: MIR181A1 --+> chemosensitivity	34453645	10.1007/s11010-021
positive Regulation: CSF3 --+> chemosensitivity	25131807	10.1016/j.cytogfr.
positive Regulation: CSF3 --+> chemosensitivity	29243031	10.1007/s12185-017
positive Regulation: CSF3 --+> chemosensitivity	7563592	
positive Regulation: CSF3 --+> chemosensitivity	10086796	10.1046/j.1365-214
positive Regulation: CSF3 --+> chemosensitivity	25908586	10.1158/0008-5472.
positive Regulation: FBXW7 --+> chemosensitivity	27398136	
positive Regulation: FBXW7 --+> chemosensitivity	27247392	10.1073/pnas.16068
positive Regulation: FBXW7 --+> chemosensitivity	32572597	10.1007/s00401-020
positive Regulation: FBXW7 --+> chemosensitivity	29633504	10.1002/1878-0261.
positive Regulation: FBXW7 --+> chemosensitivity	30094882	10.1111/cpr.124731
positive Regulation: FBXW7 --+> chemosensitivity	33147048	10.4149/neo_2020_2
positive Regulation: ANXA1 --+> chemosensitivity	30292410	10.1016/j.bbrc.201
positive Regulation: ANXA1 --+> chemosensitivity	32006544	10.1016/j.cca.2020
positive Regulation: ANXA1 --+> chemosensitivity	31882477	10.21873/invivo.11
positive Regulation: ANXA1 --+> chemosensitivity	34439260	10.3390/cancers131
positive Regulation: ANXA1 --+> chemosensitivity		10.21873/invivo.11
positive Regulation: IL15 --+> chemosensitivity		10.1016/j.hbpd.202
positive Regulation: IL15 --+> chemosensitivity	34538570	10.1016/j.hbpd.202
positive Regulation: PKM --+> chemosensitivity	27045080	10.1016/j.bbrc.201
positive Regulation: PKM --+> chemosensitivity	31132429	10.1016/j.canlet.2
positive Regulation: PKM --+> chemosensitivity	28757126	10.1016/j.bbcan.20
positive Regulation: PKM --+> chemosensitivity	27593857	10.3349/ymj.2016.5
positive Regulation: PKM --+> chemosensitivity	33503959	10.3390/ijms220311
positive Regulation: PKM --+> chemosensitivity	27045080	10.1016/j.bbrc.201
positive Regulation: PKM --+> chemosensitivity	29312802	
positive Regulation: MIR181C --+> chemosensitivity	30537505	10.1016/j.ijbiomac
positive Regulation: MIR181C --+> chemosensitivity	28956120	10.1007/s00280-017
positive Regulation: MIR181C --+> chemosensitivity	33087705	10.1038/s41419-020
positive Regulation: MIR181C --+> chemosensitivity	33087705	10.1038/s41419-020
positive Regulation: MIR181C --+> chemosensitivity	33087705	10.1038/s41419-020
positive Regulation: MIR181C --+> chemosensitivity	33087705	10.1038/s41419-020
positive Regulation: MIR181C --+> chemosensitivity	30537505	10.1016/j.ijbiomac
positive Regulation: MIR181C --+> chemosensitivity	30537505	10.1016/j.ijbiomac
positive Regulation: TGFBR2 --+> chemosensitivity	23457527	10.1371/journal.pc
positive Regulation: NELL1 --+> chemosensitivity	30628703	10.3892/or.2019.69
positive Regulation: MIR198 --+> chemosensitivity	33684406	10.1016/j.jtbi.202

positive Regulation: MIR198 --+> chemosensitivity	23069480	10.1016/j.ejca.201
positive Regulation: MIR198 --+> chemosensitivity	28425046	10.1007/s11060-017
positive Regulation: MIR198 --+> chemosensitivity	28425046	10.1007/s11060-017
positive Regulation: MIR198 --+> chemosensitivity	28425046	10.1007/s11060-017
positive Regulation: MIR198 --+> chemosensitivity	28425046	10.1007/s11060-017
positive Regulation: MIR198 --+> chemosensitivity	28765921	10.3892/or.2017.58
positive Regulation: MIR198 --+> chemosensitivity	28765921	10.3892/or.2017.58
positive Regulation: MIR198 --+> chemosensitivity	29749457	10.3892/mmr.2018.8
positive Regulation: MIR198 --+> chemosensitivity	28425046	10.1007/s11060-017
positive Regulation: MIR198 --+> chemosensitivity	28425046	10.1007/s11060-017
positive Regulation: MIR198 --+> chemosensitivity	28425046	10.1007/s11060-017
positive Regulation: MIR198 --+> chemosensitivity	28765921	10.3892/or.2017.58
positive Regulation: MIR198 --+> chemosensitivity	35559396	
positive Regulation: MIR198 --+> chemosensitivity	35559396	
positive Regulation: PACAP 38 --+> chemosensitivity	22874427	10.1152/ajpregu.00
positive Regulation: MIR409 --+> chemosensitivity	28928082	10.1016/j.yexcr.20
positive Regulation: MIR409 --+> chemosensitivity	28922711	10.1016/j.biopha.2
positive Regulation: MIR409 --+> chemosensitivity	32582997	10.3892/or.2020.76
positive Regulation: MIR409 --+> chemosensitivity	32662828	10.1042/BSR2020172
positive Regulation: MIR409 --+> chemosensitivity	32875398	10.1007/s10565-020
positive Regulation: MIR409 --+> chemosensitivity	29242506	10.1038/s41408-017
positive Regulation: MIR568 --+> chemosensitivity	33499874	10.1186/s13046-021
positive Regulation: MIR568 --+> chemosensitivity	33499874	10.1186/s13046-021
positive Regulation: ARRB1 --+> chemosensitivity	30261591	10.3390/ijms191029
positive Regulation: MIR26B --+> chemosensitivity	27717846	10.1016/j.lfs.2016
positive Regulation: MIR26B --+> chemosensitivity	25571061	10.1109/EMBC.2014.
positive Regulation: MIR26B --+> chemosensitivity	32505000	10.1016/j.omtn.202
positive Regulation: MIR26B --+> chemosensitivity	27376741	10.1007/978-981-10
positive Regulation: MIR26B --+> chemosensitivity	28281961	10.3727/096504016X
positive Regulation: MIR26B --+> chemosensitivity	29291351	10.1038/nm.4461
positive Regulation: MIR26B --+> chemosensitivity	24565101	10.1186/1476-4598-
positive Regulation: MIR26B --+> chemosensitivity	24565101	10.1186/1476-4598-
positive Regulation: MIR26B --+> chemosensitivity	35416706	10.1128/iai.00120-
positive Regulation: RARA --+> chemosensitivity	28412739	10.18632/oncotarge
positive Regulation: MIRLET7A1 --+> chemosensitivity	28445844	10.1016/j.tranon.2
positive Regulation: MIRLET7A1 --+> chemosensitivity	26687759	10.1007/s13277-015
positive Regulation: MIRLET7A1 --+> chemosensitivity	28954272	10.1159/000481610
positive Regulation: MIRLET7A1 --+> chemosensitivity	28954272	10.1159/000481610
positive Regulation: MIRLET7A1 --+> chemosensitivity	28954272	10.1159/000481610
positive Regulation: MIRLET7A1 --+> chemosensitivity	28954272	10.1159/000481610
positive Regulation: MIRLET7A1 --+> chemosensitivity	28954272	10.1159/000481610
positive Regulation: MIRLET7A1 --+> chemosensitivity	31189742	10.1042/BSR2018210
positive Regulation: MIRLET7A1 --+> chemosensitivity	23676502	10.1172/JCI66553
positive Regulation: MIRLET7A1 --+> chemosensitivity	26218285	10.1371/journal.pc
positive Regulation: MIRLET7A1 --+> chemosensitivity	26218285	10.1371/journal.pc
positive Regulation: MIRLET7A1 --+> chemosensitivity	26218285	10.1371/journal.pc
positive Regulation: MIRLET7A1 --+> chemosensitivity	26218285	10.1371/journal.pc
positive Regulation: MIRLET7A1 --+> chemosensitivity	26218285	10.1371/journal.pc
positive Regulation: MIRLET7A1 --+> chemosensitivity	23335963	10.1371/journal.pc
positive Regulation: MIRLET7A1 --+> chemosensitivity	23676502	10.1172/JCI6655310
positive Regulation: MIRLET7A1 --+> chemosensitivity		10.1042/BSR2018210
positive Regulation: MIR142 --+> chemosensitivity	30639456	10.1016/j.bcp.2019
positive Regulation: MIR142 --+> chemosensitivity	23410826	10.1016/j.lungcan.
positive Regulation: MIR142 --+> chemosensitivity	29844410	10.1038/s41419-018

positive Regulation: MIR142 --+> chemosensitivity	30266744	10.1042/BSR2018051
positive Regulation: MIR142 --+> chemosensitivity	30092578	10.1159/000492517
positive Regulation: MIR142 --+> chemosensitivity	32582997	10.3892/or.2020.76
positive Regulation: MIR142 --+> chemosensitivity	33648498	10.1186/s12943-021
positive Regulation: MIR142 --+> chemosensitivity	33648498	10.1186/s12943-021
positive Regulation: MIR142 --+> chemosensitivity		
positive Regulation: MIR142 --+> chemosensitivity	28427045	10.1159/0004678961
positive Regulation: MIR142 --+> chemosensitivity	32642410	10.1016/j.apsb.201
positive Regulation: MIR142 --+> chemosensitivity		10.1042/BSR2018051
positive Regulation: MIR142 --+> chemosensitivity	35413227	10.1139/cjpp-2022-
positive Regulation: STK4 --+> chemosensitivity	30958437	10.1097/CM9.00000C
positive Regulation: STK4 --+> chemosensitivity	23419720	10.1093/abbs/gmt0C
positive Regulation: STK4 --+> chemosensitivity	35295003	10.4103/aja2021117
positive Regulation: MIR151A --+> chemosensitivity	30102952	10.1016/j.canlet.2
positive Regulation: MIR151A --+> chemosensitivity	30917935	10.1016/j.ebiom.2C
positive Regulation: MIR151A --+> chemosensitivity	30102952	10.1016/j.canlet.2
positive Regulation: MIR151A --+> chemosensitivity	33099951	
positive Regulation: MIR151A --+> chemosensitivity	33099951	
positive Regulation: MIR151A --+> chemosensitivity		10.1016/j.ebiom.2C
positive Regulation: MIR151A --+> chemosensitivity	35232010	10.18388/abp.2020_
positive Regulation: MIR151A --+> chemosensitivity	35232010	10.18388/abp.2020_
positive Regulation: MECP2 --+> chemosensitivity	27884797	10.1016/j.resp.201
positive Regulation: MECP2 --+> chemosensitivity	22475461	10.1016/j.conb.201
positive Regulation: MECP2 --+> chemosensitivity	21307341	10.1152/ajpcell.0C
positive Regulation: MECP2 --+> chemosensitivity	21307341	10.1152/ajpcell.0C
positive Regulation: MECP2 --+> chemosensitivity	21307341	10.1152/ajpcell.0C
positive Regulation: SALL4 --+> chemosensitivity	24012616	10.1016/j.jhep.201
positive Regulation: SALL4 --+> chemosensitivity	28943937	10.3892/ol.2017.67
positive Regulation: SALL4 --+> chemosensitivity		
positive Regulation: CDK2AP1 --+> chemosensitivity	30343278	10.21873/cgp.20103
positive Regulation: CDK2AP1 --+> chemosensitivity	23404055	10.3892/ijo.2013.1
positive Regulation: CDK2AP1 --+> chemosensitivity	23404055	10.3892/ijo.2013.1
positive Regulation: CDK2AP1 --+> chemosensitivity	23404055	10.3892/ijo.2013.1
positive Regulation: CDK2AP1 --+> chemosensitivity	23404055	10.3892/ijo.2013.1
positive Regulation: CDK2AP1 --+> chemosensitivity	23404055	10.3892/ijo.2013.1
positive Regulation: CDK2AP1 --+> chemosensitivity	23404055	10.3892/ijo.2013.1
positive Regulation: ZBTB4 --+> chemosensitivity	29425745	10.1016/j.biopha.2
positive Regulation: PDCD4 --+> chemosensitivity	26142886	10.1016/j.abb.2015
positive Regulation: PDCD4 --+> chemosensitivity	26166769	10.1038/cmi.2015.4
positive Regulation: PDCD4 --+> chemosensitivity	33650661	10.3892/or.2021.79
positive Regulation: PDCD4 --+> chemosensitivity	23486359	10.4161/auto.24069
positive Regulation: PDCD4 --+> chemosensitivity	23604124	10.1038/onc.2013.1
positive Regulation: PDCD4 --+> chemosensitivity	20735432	10.1111/j.1349-70C
positive Regulation: PDCD4 --+> chemosensitivity		10.3892/or.2021.79
positive Regulation: MIR16-1 --+> chemosensitivity	30679313	10.1074/jbc.RA118.
positive Regulation: MIR16-1 --+> chemosensitivity	32987354	10.1016/j.dnarep.2
positive Regulation: MIR16-1 --+> chemosensitivity	30679313	10.1074/jbc.RA118.
positive Regulation: MIR16-1 --+> chemosensitivity	25945419	10.1080/15384047.2
positive Regulation: MIR16-1 --+> chemosensitivity	26383521	10.1007/s13277-015
positive Regulation: MIR16-1 --+> chemosensitivity	25945419	10.1080/15384047.2
positive Regulation: MIR382 --+> chemosensitivity	31635836	10.1016/j.jss.2019
positive Regulation: MIR382 --+> chemosensitivity	28982593	10.1016/j.bbamcr.2
positive Regulation: MIR382 --+> chemosensitivity	27468717	10.1007/s13277-016
positive Regulation: MIR382 --+> chemosensitivity	29700213	10.1042/BSR2018044

positive Regulation: MIR382 --+> chemosensitivity	29700213	10.1042/BSR2018044
positive Regulation: MIR382 --+> chemosensitivity	29700213	10.1042/BSR2018044
positive Regulation: MIR382 --+> chemosensitivity		10.32604/biocell.2
positive Regulation: MIR382 --+> chemosensitivity	25998694	10.3892/or.2015.39
positive Regulation: MIR382 --+> chemosensitivity	25344865	10.18632/oncotarge
positive Regulation: MIR382 --+> chemosensitivity	29700213	10.1042/BSR2018044
positive Regulation: MIR382 --+> chemosensitivity	29700213	10.1042/BSR2018044
positive Regulation: MIR1207 --+> chemosensitivity	30341811	10.1002/1878-0261.
positive Regulation: PPIA --+> chemosensitivity	33121398	10.2174/0929867327
positive Regulation: CCDC144NL-AS1 --+> chemosensitivit	35083139	10.3389/fonc.2021.
positive Regulation: MIR920 --+> chemosensitivity	34633158	10.23736/S0026-480
positive Regulation: UCHL1 --+> chemosensitivity	29080080	10.1007/s10555-017
positive Regulation: UCHL1 --+> chemosensitivity	23499448	10.1016/j.celrep.2
positive Regulation: CD24 --+> chemosensitivity	28866120	10.1016/j.imlet.20
positive Regulation: CD24 --+> chemosensitivity	28179302	10.21873/anticanre
positive Regulation: CD24 --+> chemosensitivity	28179302	10.21873/anticanre
positive Regulation: CD24 --+> chemosensitivity	28179302	10.21873/anticanre
positive Regulation: CD24 --+> chemosensitivity	32124945	10.3892/ijo.2020.4
positive Regulation: CD24 --+> chemosensitivity	32572883	10.26355/eurrev_20
positive Regulation: CD24 --+> chemosensitivity	21960110	10.1007/s10549-011
positive Regulation: CD24 --+> chemosensitivity	23852892	
positive Regulation: CD24 --+> chemosensitivity	23852892	
positive Regulation: CD24 --+> chemosensitivity	23852892	
positive Regulation: CD24 --+> chemosensitivity	25308718	
positive Regulation: CD24 --+> chemosensitivity	23852892	
positive Regulation: CD24 --+> chemosensitivity	23852892	
positive Regulation: CD24 --+> chemosensitivity	23852892	
positive Regulation: CD24 --+> chemosensitivity	28179302	10.21873/anticanre
positive Regulation: CD24 --+> chemosensitivity	28179302	10.21873/anticanre
positive Regulation: CD24 --+> chemosensitivity	28179302	10.21873/anticanre
positive Regulation: MIR143 --+> chemosensitivity	29224950	10.1016/j.cca.2017
positive Regulation: MIR143 --+> chemosensitivity	28242349	10.1016/j.bbcan.20
positive Regulation: MIR143 --+> chemosensitivity	29879503	10.1016/j.gene.201
positive Regulation: MIR143 --+> chemosensitivity	31704502	10.1016/j.biocel.2
positive Regulation: MIR143 --+> chemosensitivity	25281917	10.1016/j.addr.201
positive Regulation: MIR143 --+> chemosensitivity	31863330	10.1007/s11033-019
positive Regulation: MIR143 --+> chemosensitivity	33077697	10.18632/aging.103
positive Regulation: MIR143 --+> chemosensitivity	33569751	10.1007/s43032-021
positive Regulation: MIR143 --+> chemosensitivity	33569751	10.1007/s43032-021
positive Regulation: MIR143 --+> chemosensitivity	33569751	10.1007/s43032-021
positive Regulation: MIR143 --+> chemosensitivity	33569751	10.1007/s43032-021
positive Regulation: MIR143 --+> chemosensitivity	33569751	10.1007/s43032-021
positive Regulation: MIR143 --+> chemosensitivity	23383988	10.1186/1471-2407-
positive Regulation: MIR143 --+> chemosensitivity	23202889	10.3390/ijms131012
positive Regulation: MIR143 --+> chemosensitivity	21197560	10.1007/s11010-010
positive Regulation: MIR143 --+> chemosensitivity	23574723	10.4161/cc.24477
positive Regulation: MIR143 --+> chemosensitivity	23574723	10.4161/cc.24477
positive Regulation: MIR143 --+> chemosensitivity	23574723	10.4161/cc.24477
positive Regulation: MIR143 --+> chemosensitivity	23574723	10.4161/cc.24477
positive Regulation: MIR143 --+> chemosensitivity	23574723	10.4161/cc.24477
positive Regulation: MIR143 --+> chemosensitivity	24722758	10.1371/journal.pc
positive Regulation: MIR143 --+> chemosensitivity	21197560	10.1007/s11010-010
positive Regulation: MIR143 --+> chemosensitivity	23574723	10.4161/cc.2447710
positive Regulation: MIR143 --+> chemosensitivity	28123579	10.3892/ol.2016.53

positive Regulation: HCP5 --+> chemosensitivity	33993846	10.1080/15384101.2
positive Regulation: SHC1 --+> chemosensitivity	31575671	10.3324/haematol.2
positive Regulation: SHC1 --+> chemosensitivity	30819907	10.3324/haematol.2
positive Regulation: CDH1 --+> chemosensitivity	16373333	
positive Regulation: CDH1 --+> chemosensitivity	28469776	
positive Regulation: CDH1 --+> chemosensitivity	22126395	10.1186/bcr3068
positive Regulation: CDH1 --+> chemosensitivity	24377525	10.7314/APJCP.2013
positive Regulation: CDH1 --+> chemosensitivity	24883070	10.1155/2014/73067
positive Regulation: CDH1 --+> chemosensitivity	26316041	10.3892/ijo.2015.3
positive Regulation: CDH1 --+> chemosensitivity	26316041	10.3892/ijo.2015.3
positive Regulation: CDH1 --+> chemosensitivity	26316041	10.3892/ijo.2015.3
positive Regulation: CDH1 --+> chemosensitivity	26316041	10.3892/ijo.2015.3
positive Regulation: CDH1 --+> chemosensitivity	26316041	10.3892/ijo.2015.3
positive Regulation: CDH1 --+> chemosensitivity	26316041	10.3892/ijo.2015.3
positive Regulation: CDH1 --+> chemosensitivity	26316041	10.3892/ijo.2015.3
positive Regulation: CDH1 --+> chemosensitivity	14532995	
positive Regulation: CDH1 --+> chemosensitivity	14532995	
positive Regulation: CDH1 --+> chemosensitivity	15361706	10.1159/000079141
positive Regulation: MIR664A --+> chemosensitivity	30896829	10.3892/ijo.2019.4
positive Regulation: MIR145 --+> chemosensitivity	32818543	10.1016/j.lfs.2020
positive Regulation: MIR145 --+> chemosensitivity	29627370	10.1016/j.addr.201
positive Regulation: MIR145 --+> chemosensitivity	31704502	10.1016/j.biocel.2
positive Regulation: MIR145 --+> chemosensitivity	32505000	10.1016/j.omtn.202
positive Regulation: MIR145 --+> chemosensitivity	26852750	10.1007/s13277-016
positive Regulation: MIR145 --+> chemosensitivity	30896792	10.3892/ijo.2019.4
positive Regulation: MIR145 --+> chemosensitivity	31582906	10.1186/s12935-019
positive Regulation: MIR145 --+> chemosensitivity	32339066	10.1177/0300060520
positive Regulation: MIR145 --+> chemosensitivity	32209033	10.2174/1566524020
positive Regulation: MIR145 --+> chemosensitivity	32209033	10.2174/1566524020
positive Regulation: MIR145 --+> chemosensitivity	30485503	10.1002/jcb.280791
positive Regulation: MIR145 --+> chemosensitivity	31440081	10.2147/CMAR.S2100
positive Regulation: MIR145 --+> chemosensitivity	31440081	10.2147/CMAR.S2100
positive Regulation: MIR145 --+> chemosensitivity		10.3892/ijo.2019.4
positive Regulation: MIR145 --+> chemosensitivity		10.1186/s12935-019
positive Regulation: MIR145 --+> chemosensitivity	34825319	10.1007/s11033-021
positive Regulation: MIR145 --+> chemosensitivity	34839317	10.1097/CM9.000000
positive Regulation: SERPINB5 --+> chemosensitivity	28648644	10.1016/j.bbamcr.2
positive Regulation: SERPINB5 --+> chemosensitivity	31732312	10.1016/j.amjoto.2
positive Regulation: SERPINB5 --+> chemosensitivity	22076034	10.3892/ijmm.2011.
positive Regulation: SERPINB5 --+> chemosensitivity	23530500	
positive Regulation: SERPINB5 --+> chemosensitivity	19906413	10.1016/j.ygyno.20
positive Regulation: SERPINB5 --+> chemosensitivity	26733306	10.1186/s13046-015
positive Regulation: SERPINB5 --+> chemosensitivity	26733306	10.1186/s13046-015
positive Regulation: SERPINB5 --+> chemosensitivity	26733306	10.1186/s13046-015
positive Regulation: SERPINB5 --+> chemosensitivity		10.1016/j.amjoto.2
positive Regulation: CYP2C9 --+> chemosensitivity	10991840	
positive Regulation: DNMT3B --+> chemosensitivity	35670956	10.1007/s13577-022
positive Regulation: UPF1 --+> chemosensitivity	34036905	10.2174/0929867328
positive Regulation: GRM8 --+> chemosensitivity	34950209	10.1155/2021/80020
positive Regulation: GRM8 --+> chemosensitivity	34950209	10.1155/2021/80020
positive Regulation: HOPX --+> chemosensitivity	28146149	10.1038/ncomms1405
positive Regulation: HOPX --+> chemosensitivity	28146149	10.1038/ncomms1405
positive Regulation: HOPX --+> chemosensitivity	28146149	10.1038/ncomms1405
positive Regulation: HOPX --+> chemosensitivity	28146149	10.1038/ncomms1405

positive Regulation: HOPX --+> chemosensitivity	33824475	10.1038/s41388-021
positive Regulation: HOPX --+> chemosensitivity	28146149	10.1038/ncomms1405
positive Regulation: MIR337 --+> chemosensitivity	28641487	10.1177/1010428317
positive Regulation: SLC5A8 --+> chemosensitivity	23167260	10.1042/BJ20121248
positive Regulation: MIR383 --+> chemosensitivity	30399596	10.1016/j.biopha.2
positive Regulation: MIR383 --+> chemosensitivity	30399596	10.1016/j.biopha.2
positive Regulation: MIR383 --+> chemosensitivity	31648164	10.1016/j.biopha.2
positive Regulation: MIR383 --+> chemosensitivity	31308851	10.1186/s11658-019
positive Regulation: MIR383 --+> chemosensitivity		10.1590/1414-431x2
positive Regulation: MIR383 --+> chemosensitivity	30399596	10.1016/j.biopha.2
positive Regulation: MIR383 --+> chemosensitivity	34440380	10.3390/genes12081
positive Regulation: MIR383 --+> chemosensitivity	34761351	10.1007/s12032-021
positive Regulation: MIR383 --+> chemosensitivity	34761351	10.1007/s12032-021
positive Regulation: MIR383 --+> chemosensitivity	34761351	10.1007/s12032-021
positive Regulation: MIR383 --+> chemosensitivity	34761351	10.1007/s12032-021
positive Regulation: MIR383 --+> chemosensitivity	34761351	10.1007/s12032-021
positive Regulation: MIR383 --+> chemosensitivity	34761351	10.1007/s12032-021
positive Regulation: MIR383 --+> chemosensitivity	34761351	10.1007/s12032-021
positive Regulation: MIR383 --+> chemosensitivity	35799305	10.1186/s13048-022
positive Regulation: SPHK2 --+> chemosensitivity	19240026	10.1074/jbc.M90073
positive Regulation: SPHK2 --+> chemosensitivity	19240026	
positive Regulation: SPHK2 --+> chemosensitivity	22469881	10.3892/or.2012.17
positive Regulation: SPHK2 --+> chemosensitivity	21707491	
positive Regulation: SPHK2 --+> chemosensitivity	26628299	10.1007/s13277-015
positive Regulation: SPHK2 --+> chemosensitivity	36066393	10.1097/CAD.000000
positive Regulation: LAPTM5 --+> chemosensitivity	35294039	10.3892/ijo.2022.5
positive Regulation: LAPTM5 --+> chemosensitivity	35294039	10.3892/ijo.2022.5
positive Regulation: LAPTM5 --+> chemosensitivity	35294039	10.3892/ijo.2022.5
positive Regulation: ECRG4 --+> chemosensitivity	23553029	10.1007/s13277-013
positive Regulation: ECRG4 --+> chemosensitivity	35464163	10.1007/s10616-022
positive Regulation: ECRG4 --+> chemosensitivity	35676661	10.1186/s12967-022
positive Regulation: ECRG4 --+> chemosensitivity	35676661	10.1186/s12967-022
positive Regulation: ECRG4 --+> chemosensitivity	35676661	10.1186/s12967-022
positive Regulation: HMGA2 --+> chemosensitivity	17222355	
positive Regulation: HMGA2 --+> chemosensitivity		10.3892/ijmm.2019.
positive Regulation: HMGA2 --+> chemosensitivity	30543177	10.2174/1871520619
positive Regulation: HMGA2 --+> chemosensitivity	28954272	10.1159/0004816101
positive Regulation: HMGA2 --+> chemosensitivity	31067275	10.1371/journal.pc
positive Regulation: HMGA2 --+> chemosensitivity	31173171	10.3892/ijmm.2019.
positive Regulation: HMGA2 --+> chemosensitivity		
positive Regulation: ABCF1 --+> chemosensitivity	31100412	10.1016/j.canlet.2
positive Regulation: IL2 --+> chemosensitivity	23972545	10.1016/j.cyto.201
positive Regulation: UGT1A1 --+> chemosensitivity	25260839	10.1016/j.biopha.2
positive Regulation: UGT1A1 --+> chemosensitivity	25260839	10.1016/j.biopha.2
positive Regulation: UGT1A1 --+> chemosensitivity	25260839	10.1016/j.biopha.2
positive Regulation: UGT1A1 --+> chemosensitivity	25260839	10.1016/j.biopha.2
positive Regulation: UGT1A1 --+> chemosensitivity	25260839	10.1016/j.biopha.2
positive Regulation: UGT1A1 --+> chemosensitivity	30016963	10.1186/s12967-018
positive Regulation: KISS1 --+> chemosensitivity	22226740	10.1016/j.ajpath.2
positive Regulation: KISS1 --+> chemosensitivity	21383688	10.1038/onc.2011.3
positive Regulation: RBM4 --+> chemosensitivity	32187743	10.1002/jcla.23272
positive Regulation: MIR146B --+> chemosensitivity	31546023	10.1016/j.canlet.2
positive Regulation: MIR146B --+> chemosensitivity	33279671	10.1016/j.cellsig.
positive Regulation: MIR146B --+> chemosensitivity	31877405	10.1016/j.drup.201

positive Regulation: MIR146B --+> chemosensitivity	29048680	10.3892/or.2017.59
positive Regulation: MIR146B --+> chemosensitivity	29048680	10.3892/or.2017.59
positive Regulation: MIR146B --+> chemosensitivity	29048680	10.3892/or.2017.59
positive Regulation: MIR146B --+> chemosensitivity	30409964	10.1038/s41419-018
positive Regulation: MIR146B --+> chemosensitivity	30409964	10.1038/s41419-018
positive Regulation: LNC-LBCS --+> chemosensitivity	30397178	10.1158/1078-0432.
positive Regulation: GGT5 --+> chemosensitivity	32640421	10.18632/aging.103
positive Regulation: TRIM21 --+> chemosensitivity	34856418	10.1016/j.bbrc.202
positive Regulation: MARVELD1 --+> chemosensitivity	23826386	10.1371/journal.pc
positive Regulation: MARVELD1 --+> chemosensitivity	22320884	10.1111/j.1349-700
positive Regulation: G3BP2 --+> chemosensitivity	31461651	10.1016/j.celrep.2
positive Regulation: H2AX --+> chemosensitivity	26687759	10.1007/s13277-015
positive Regulation: H2AX --+> chemosensitivity	27006338	10.15252/emmm.2015
positive Regulation: H2AX --+> chemosensitivity	25487737	10.1038/ncomms6691
positive Regulation: H2AX --+> chemosensitivity	25487737	10.1038/ncomms6691
positive Regulation: H2AX --+> chemosensitivity	29435013	10.3892/ol.2017.76
positive Regulation: MIR216B --+> chemosensitivity	28373071	10.1016/j.bbrc.201
positive Regulation: MIR216B --+> chemosensitivity	32387443	10.1016/j.canlet.2
positive Regulation: MIR216B --+> chemosensitivity	29787989	10.1016/j.biopha.2
positive Regulation: MIR216B --+> chemosensitivity	30778048	10.1038/s41419-019
positive Regulation: MIR216B --+> chemosensitivity	32972441	10.1186/s13046-020
positive Regulation: MIR216B --+> chemosensitivity	28373071	10.1016/j.bbrc.201
positive Regulation: MIR216B --+> chemosensitivity	35152275	10.1038/s41374-022
positive Regulation: RHOB --+> chemosensitivity	21872591	10.1016/j.febslet.
positive Regulation: RHOB --+> chemosensitivity	22982107	10.1016/j.febslet.
positive Regulation: RHOB --+> chemosensitivity	23153558	10.1016/j.taap.201
positive Regulation: RHOB --+> chemosensitivity	20473933	10.1002/ijc.254451
positive Regulation: MIR519A1 --+> chemosensitivity	34203727	10.3390/ijms221263
positive Regulation: MIR519A1 --+> chemosensitivity	29843746	10.1186/s13045-018
positive Regulation: MIR519A1 --+> chemosensitivity	34454479	10.1186/s12935-021
positive Regulation: MIR519A1 --+> chemosensitivity	35432536	10.1155/2022/25000
positive Regulation: MIR519A1 --+> chemosensitivity	35432536	10.1155/2022/25000
positive Regulation: MIR519A1 --+> chemosensitivity	36354672	10.3390/cimb441103
positive Regulation: MC4R --+> chemosensitivity	33624805	10.1093/sleep/zsab
positive Regulation: MIR101-1 --+> chemosensitivity	33989708	10.1016/j.canlet.2
positive Regulation: MIR101-1 --+> chemosensitivity	28242349	10.1016/j.bbcan.20
positive Regulation: MIR101-1 --+> chemosensitivity	32119915	10.1016/j.gene.202
positive Regulation: MIR101-1 --+> chemosensitivity	27155790	10.1016/j.molonc.2
positive Regulation: MIR101-1 --+> chemosensitivity		10.1016/j.jss.2011
positive Regulation: MIR101-1 --+> chemosensitivity		10.1016/j.jss.2011
positive Regulation: MIR101-1 --+> chemosensitivity	28384067	10.1089/dna.2016.3
positive Regulation: MIR101-1 --+> chemosensitivity	28560419	10.3892/or.2017.56
positive Regulation: MIR101-1 --+> chemosensitivity	28933259	10.1177/1010428317
positive Regulation: MIR101-1 --+> chemosensitivity	28079894	10.1038/cddis.2016
positive Regulation: MIR101-1 --+> chemosensitivity	28950928	10.3727/096504017X
positive Regulation: MIR101-1 --+> chemosensitivity	30596336	10.1080/15384101.2
positive Regulation: MIR101-1 --+> chemosensitivity	30249919	10.2176/nmc.ra.201
positive Regulation: MIR101-1 --+> chemosensitivity	31298406	10.26355/eurrev_20
positive Regulation: MIR101-1 --+> chemosensitivity	31638211	10.3892/ijo.2019.4
positive Regulation: MIR101-1 --+> chemosensitivity	25013866	10.1155/2014/79475
positive Regulation: MIR101-1 --+> chemosensitivity	25013866	10.1155/2014/79475
positive Regulation: MIR101-1 --+> chemosensitivity	25153722	10.18632/oncotarge
positive Regulation: MIR101-1 --+> chemosensitivity	26628987	
positive Regulation: MIR101-1 --+> chemosensitivity		10.3727/096504017X

positive Regulation: MIR101-1 --+> chemosensitivity		10.1080/15384101.2
positive Regulation: MIR101-1 --+> chemosensitivity		10.26355/eurrev_20
positive Regulation: MIR101-1 --+> chemosensitivity		10.3892/ijo.2019.4
positive Regulation: MIR152 --+> chemosensitivity	31128298	10.1016/j.semcance
positive Regulation: MIR410 --+> chemosensitivity	33035835	10.1016/j.biopha.2
positive Regulation: MIR410 --+> chemosensitivity	28138700	10.3892/mmr.2017.6
positive Regulation: MIR410 --+> chemosensitivity	32323781	10.3892/or.2020.74
positive Regulation: MIR223 --+> chemosensitivity	33310181	10.1016/j.ejphar.2
positive Regulation: MIR223 --+> chemosensitivity	28322994	10.1016/j.gene.201
positive Regulation: MIR223 --+> chemosensitivity	28322994	10.1016/j.gene.201
positive Regulation: MIR223 --+> chemosensitivity	28322994	10.1016/j.gene.201
positive Regulation: MIR223 --+> chemosensitivity	29518547	10.1016/j.gene.201
positive Regulation: MIR223 --+> chemosensitivity	29518547	10.1016/j.gene.201
positive Regulation: MIR223 --+> chemosensitivity	33865701	10.1016/j.semcdb.2
positive Regulation: MIR223 --+> chemosensitivity	27398136	
positive Regulation: MIR223 --+> chemosensitivity	31991775	10.3390/ijms210307
positive Regulation: MIR223 --+> chemosensitivity	29518547	10.1016/j.gene.201
positive Regulation: MIR223 --+> chemosensitivity	29518547	10.1016/j.gene.201
positive Regulation: MIR223 --+> chemosensitivity	34830408	10.3390/ijms222212
positive Regulation: MIR223 --+> chemosensitivity	36052513	10.15252/embj.2021
positive Regulation: MIR608 --+> chemosensitivity	32822816	10.1016/j.canlet.2
positive Regulation: LEP --+> chemosensitivity	20609602	10.1016/j.smrv.201
positive Regulation: LEP --+> chemosensitivity		10.1016/S1389-9457
positive Regulation: LEP --+> chemosensitivity	23201827	10.1016/j.neurosci
positive Regulation: LEP --+> chemosensitivity	23201827	10.1016/j.neurosci
positive Regulation: LEP --+> chemosensitivity	23541616	10.1016/j.brainres
positive Regulation: LEP --+> chemosensitivity	11834635	10.1378/chest.121.
positive Regulation: LEP --+> chemosensitivity	11834635	
positive Regulation: LEP --+> chemosensitivity	32603263	10.1165/rcmb.2020-
positive Regulation: LEP --+> chemosensitivity	32603263	10.1165/rcmb.2020-
positive Regulation: LEP --+> chemosensitivity	33624805	10.1093/sleep/zsab
positive Regulation: LEP --+> chemosensitivity	34201760	10.3390/ijms221367
positive Regulation: LEP --+> chemosensitivity	34131808	10.1007/s00018-021
positive Regulation: LEP --+> chemosensitivity	31289528	10.3892/ol.2019.10
positive Regulation: MIR302B --+> chemosensitivity	23845851	10.1016/j.toxlet.2
positive Regulation: ZNF185 --+> chemosensitivity	32565799	10.1155/2020/31815
positive Regulation: ZNF185 --+> chemosensitivity	32565799	10.1155/2020/31815
positive Regulation: ZNF185 --+> chemosensitivity	32565799	10.1155/2020/31815
positive Regulation: ZNF185 --+> chemosensitivity	32565799	10.1155/2020/31815
positive Regulation: ZNF185 --+> chemosensitivity	32565799	10.1155/2020/31815
positive Regulation: IPO7 --+> chemosensitivity	23178451	10.1016/j.canlet.2
positive Regulation: MIR193B --+> chemosensitivity	22819820	10.1016/j.febslet.
positive Regulation: MIR193B --+> chemosensitivity	26878873	10.1186/s12885-016
positive Regulation: MIR193B --+> chemosensitivity	26878873	10.1186/s12885-016
positive Regulation: MIR193B --+> chemosensitivity	26878873	10.1186/s12885-016
positive Regulation: MIR193B --+> chemosensitivity	26878873	10.1186/s12885-016
positive Regulation: MIR193B --+> chemosensitivity	35575588	10.1080/15384101.2
positive Regulation: CCL2 --+> chemosensitivity	35787939	10.1016/j.semcance
positive Regulation: MIR133A1 --+> chemosensitivity	31651347	10.1186/s12943-019
positive Regulation: PNMA5 --+> chemosensitivity	27424190	10.1007/s00432-016
positive Regulation: PNMA5 --+> chemosensitivity	27424190	10.1007/s00432-016
positive Regulation: PNMA5 --+> chemosensitivity	27424190	10.1007/s00432-016
positive Regulation: PNMA5 --+> chemosensitivity	27424190	10.1007/s00432-016
positive Regulation: PNMA5 --+> chemosensitivity	35559417	

positive Regulation: TET2 --+> chemosensitivity	32355773	10.21037/atm.2020.
positive Regulation: MIRLET7D --+> chemosensitivity	25281917	10.1016/j.addr.201
positive Regulation: MIRLET7D --+> chemosensitivity	29658612	10.3892/or.2018.63
positive Regulation: MIRLET7D --+> chemosensitivity	30816441	10.3892/ijo.2019.4
positive Regulation: MIRLET7D --+> chemosensitivity	30816441	10.3892/ijo.2019.4
positive Regulation: MIRLET7D --+> chemosensitivity	30816441	10.3892/ijo.2019.4
positive Regulation: MIRLET7D --+> chemosensitivity	30816441	10.3892/ijo.2019.4
positive Regulation: MIRLET7D --+> chemosensitivity	30816441	10.3892/ijo.2019.4
positive Regulation: MIRLET7D --+> chemosensitivity	30816441	10.3892/ijo.2019.4
positive Regulation: MIRLET7D --+> chemosensitivity	21725603	10.3892/or.2011.13
positive Regulation: MIRLET7D --+> chemosensitivity	23834156	10.2174/1389450111
positive Regulation: MIRLET7D --+> chemosensitivity	21725603	10.3892/or.2011.13
positive Regulation: MIRLET7D --+> chemosensitivity	33076802	10.2174/1381612826
positive Regulation: MIRLET7D --+> chemosensitivity	35715825	10.1186/s13048-022
positive Regulation: MIRLET7D --+> chemosensitivity	35864955	10.7150/ijbs.72952
positive Regulation: LINC00161 --+> chemosensitivity	28499919	10.1016/j.bbamcr.2
positive Regulation: LINC00161 --+> chemosensitivity	36539799	10.1186/s13046-022
positive Regulation: NR3C1 --+> chemosensitivity	34017210	10.1177/1179554921
positive Regulation: MIR381 --+> chemosensitivity	33137708	10.1016/j.tice.202
positive Regulation: MIR381 --+> chemosensitivity	32898724	10.1016/j.coph.202
positive Regulation: MIR381 --+> chemosensitivity	32763503	10.1016/j.tranon.2
positive Regulation: MIR381 --+> chemosensitivity	29048619	10.3892/or.2017.59
positive Regulation: MIR381 --+> chemosensitivity	29257334	10.3892/mmr.2017.8
positive Regulation: MIR381 --+> chemosensitivity	29257334	10.3892/mmr.2017.8
positive Regulation: MIR381 --+> chemosensitivity	31299041	10.12659/MSM.91552
positive Regulation: MIR381 --+> chemosensitivity	32763503	10.1016/j.tranon.2
positive Regulation: MIR381 --+> chemosensitivity	27612424	10.18632/oncotarge
positive Regulation: MIR381 --+> chemosensitivity	29242506	10.1038/s41408-017
positive Regulation: MIR381 --+> chemosensitivity		10.12659/MSM.91552
positive Regulation: SPARC --+> chemosensitivity	33910127	10.1016/j.bbrc.202
positive Regulation: SPARC --+> chemosensitivity	33910127	10.1016/j.bbrc.202
positive Regulation: SPARC --+> chemosensitivity	33910127	10.1016/j.bbrc.202
positive Regulation: SPARC --+> chemosensitivity	33910127	10.1016/j.bbrc.202
positive Regulation: SPARC --+> chemosensitivity	17897953	10.1074/jbc.M70445
positive Regulation: SPARC --+> chemosensitivity	18992864	10.1016/j.ajog.200
positive Regulation: SPARC --+> chemosensitivity	18992864	10.1016/j.ajog.200
positive Regulation: SPARC --+> chemosensitivity	18992864	10.1016/j.ajog.200
positive Regulation: SPARC --+> chemosensitivity	18992864	10.1016/j.ajog.200
positive Regulation: SPARC --+> chemosensitivity	18849185	10.1016/j.drup.200
positive Regulation: SPARC --+> chemosensitivity	18849185	10.1016/j.drup.200
positive Regulation: SPARC --+> chemosensitivity	25064829	10.1016/j.matbio.2
positive Regulation: SPARC --+> chemosensitivity	15902309	
positive Regulation: SPARC --+> chemosensitivity	17897953	
positive Regulation: SPARC --+> chemosensitivity	32595212	10.1038/s41416-020
positive Regulation: SPARC --+> chemosensitivity	22480225	10.1186/1476-4598-
positive Regulation: SPARC --+> chemosensitivity	22069448	10.1371/journal.pc
positive Regulation: SPARC --+> chemosensitivity	24309322	10.1038/modpathol.
positive Regulation: SPARC --+> chemosensitivity	26358255	10.1007/s13277-015
positive Regulation: SPARC --+> chemosensitivity	26358255	10.1007/s13277-015
positive Regulation: SPARC --+> chemosensitivity	25030587	
positive Regulation: SPARC --+> chemosensitivity	25030587	
positive Regulation: SPARC --+> chemosensitivity	25030587	
positive Regulation: SPARC --+> chemosensitivity	26358255	10.1007/s13277-015
positive Regulation: SPARC --+> chemosensitivity	26358255	10.1007/s13277-015

positive Regulation: SPARC --+> chemosensitivity	33910127	10.1016/j.bbrc.202
positive Regulation: SPARC --+> chemosensitivity	33910127	10.1016/j.bbrc.202
positive Regulation: SPARC --+> chemosensitivity		
positive Regulation: SPARC --+> chemosensitivity	36157225	10.1155/2022/79615
positive Regulation: MIR196A1 --+> chemosensitivity	23570052	
positive Regulation: MIR196A1 --+> chemosensitivity	26062455	10.3892/or.2015.40
positive Regulation: MIR196A1 --+> chemosensitivity	26936095	10.3892/ijmm.2016.
positive Regulation: MIR196A1 --+> chemosensitivity	26376998	10.1007/s13277-015
positive Regulation: MIR196A1 --+> chemosensitivity	25571061	10.1109/EMBC.2014.
positive Regulation: MIR1-1 --+> chemosensitivity	30551491	10.1016/j.biopha.2
positive Regulation: MIR1-1 --+> chemosensitivity	28358371	10.1038/cddis.2017
positive Regulation: MIR1-1 --+> chemosensitivity	29851226	10.1002/cbin.10995
positive Regulation: MIR1-1 --+> chemosensitivity	33277838	
positive Regulation: MIR1-1 --+> chemosensitivity	33277838	
positive Regulation: COL12A1 --+> chemosensitivity	26843134	10.1620/tjem.238.1
positive Regulation: LITAF --+> chemosensitivity	26716897	10.18632/oncotarge
positive Regulation: MIR637 --+> chemosensitivity	31631038	10.1016/j.ebiom.20
positive Regulation: MIR637 --+> chemosensitivity	31631038	10.1016/j.ebiom.20
positive Regulation: LINC01963 --+> chemosensitivity	35152275	10.1038/s41374-022
positive Regulation: LINC01963 --+> chemosensitivity	35152275	10.1038/s41374-022
positive Regulation: LINC01963 --+> chemosensitivity	35152275	10.1038/s41374-022
positive Regulation: LINC01963 --+> chemosensitivity	35152275	10.1038/s41374-022
positive Regulation: LINC01963 --+> chemosensitivity	35152275	10.1038/s41374-022
positive Regulation: LINC01963 --+> chemosensitivity	35152275	10.1038/s41374-022
positive Regulation: LINC01963 --+> chemosensitivity	35152275	10.1038/s41374-022
positive Regulation: IFNG --+> chemosensitivity	17627812	10.1016/j.ejca.200
positive Regulation: IFNG --+> chemosensitivity	33194045	
positive Regulation: IFNG --+> chemosensitivity	21327638	10.1007/s00262-011
positive Regulation: STK31 --+> chemosensitivity	30144069	10.1002/jcp.270791
positive Regulation: GJB1 --+> chemosensitivity	31213923	10.2147/CMAR.S2036
positive Regulation: MIR302A --+> chemosensitivity	33865878	10.1016/j.lfs.2021
positive Regulation: MIR302A --+> chemosensitivity	26644266	10.1016/j.ygyno.20
positive Regulation: MIR302A --+> chemosensitivity	28922711	10.1016/j.biopha.2
positive Regulation: MIR302A --+> chemosensitivity	26644266	10.1016/j.ygyno.20
positive Regulation: PTEN --+> chemosensitivity	24434152	10.1016/j.bbrc.201
positive Regulation: PTEN --+> chemosensitivity	11729185	10.1074/jbc.M10830
positive Regulation: PTEN --+> chemosensitivity	11495901	10.1074/jbc.M10363
positive Regulation: PTEN --+> chemosensitivity	11495901	10.1074/jbc.M10363
positive Regulation: PTEN --+> chemosensitivity	11495901	10.1074/jbc.M10363
positive Regulation: PTEN --+> chemosensitivity	11495901	10.1074/jbc.M10363
positive Regulation: PTEN --+> chemosensitivity	27721020	10.1016/j.canlet.2
positive Regulation: PTEN --+> chemosensitivity	33220333	10.1016/j.canlet.2
positive Regulation: PTEN --+> chemosensitivity	32827577	10.1016/j.bbcan.20
positive Regulation: PTEN --+> chemosensitivity	28755993	10.1016/j.ijpharm.
positive Regulation: PTEN --+> chemosensitivity	21187093	10.1016/j.febslet.
positive Regulation: PTEN --+> chemosensitivity	22465665	10.1016/j.febslet.
positive Regulation: PTEN --+> chemosensitivity	16545436	10.1016/j.ygyno.20
positive Regulation: PTEN --+> chemosensitivity	15072831	10.1016/j.canlet.2
positive Regulation: PTEN --+> chemosensitivity	15072831	10.1016/j.canlet.2
positive Regulation: PTEN --+> chemosensitivity	23920276	10.1016/j.phymed.2
positive Regulation: PTEN --+> chemosensitivity	25744176	10.1016/j.drudis.2
positive Regulation: PTEN --+> chemosensitivity	21108789	
positive Regulation: PTEN --+> chemosensitivity	21108789	
positive Regulation: PTEN --+> chemosensitivity	19299556	

positive Regulation: PTEN --+> chemosensitivity	11729185	
positive Regulation: PTEN --+> chemosensitivity	11495901	
positive Regulation: PTEN --+> chemosensitivity	11495901	
positive Regulation: PTEN --+> chemosensitivity	11495901	
positive Regulation: PTEN --+> chemosensitivity	11495901	
positive Regulation: PTEN --+> chemosensitivity	18458673	
positive Regulation: PTEN --+> chemosensitivity	16404430	
positive Regulation: PTEN --+> chemosensitivity	11103942	
positive Regulation: PTEN --+> chemosensitivity	28887606	10.1007/s10616-017
positive Regulation: PTEN --+> chemosensitivity	30616477	10.1177/0300060518
positive Regulation: PTEN --+> chemosensitivity	33076450	10.3390/ijms212076
positive Regulation: PTEN --+> chemosensitivity	33721218	10.1007/s13577-021
positive Regulation: PTEN --+> chemosensitivity	33721218	10.1007/s13577-021
positive Regulation: PTEN --+> chemosensitivity	33721218	10.1007/s13577-021
positive Regulation: PTEN --+> chemosensitivity	33721218	10.1007/s13577-021
positive Regulation: PTEN --+> chemosensitivity	33721218	10.1007/s13577-021
positive Regulation: PTEN --+> chemosensitivity	21108789	10.1186/1476-4598-
positive Regulation: PTEN --+> chemosensitivity	21108789	10.1186/1476-4598-
positive Regulation: PTEN --+> chemosensitivity	20736773	10.1097/PGP.0b013e
positive Regulation: PTEN --+> chemosensitivity	26043084	10.1371/journal.pc
positive Regulation: PTEN --+> chemosensitivity	11103942	10.1038/sj.onc.120
positive Regulation: PTEN --+> chemosensitivity	16404430	10.1038/sj.bjc.660
positive Regulation: PTEN --+> chemosensitivity	16438844	
positive Regulation: PTEN --+> chemosensitivity	21820606	10.1016/j.arcmed.2
positive Regulation: PTEN --+> chemosensitivity	28686971	10.1016/j.biopha.2
positive Regulation: PTEN --+> chemosensitivity	30925916	10.1186/s13046-019
positive Regulation: PTEN --+> chemosensitivity	33721218	10.1007/s13577-021
positive Regulation: PTEN --+> chemosensitivity		10.1016/j.febslet.
positive Regulation: PTEN --+> chemosensitivity		10.1177/0300060518
positive Regulation: PTEN --+> chemosensitivity	27147467	10.1007/s00018-016
positive Regulation: PTEN --+> chemosensitivity	22781804	
positive Regulation: PTEN --+> chemosensitivity	27955382	
positive Regulation: PTEN --+> chemosensitivity	34681603	10.3390/ijms222010
positive Regulation: MIR495 --+> chemosensitivity	33892053	10.1016/j.bbcan.20
positive Regulation: MIR495 --+> chemosensitivity	33892053	10.1016/j.bbcan.20
positive Regulation: MIR495 --+> chemosensitivity	27075472	10.1007/s13277-016
positive Regulation: MIR495 --+> chemosensitivity	30147110	10.12659/MSM.90945
positive Regulation: MIR495 --+> chemosensitivity	30147110	10.12659/MSM.90945
positive Regulation: MIR495 --+> chemosensitivity	30147110	10.12659/MSM.90945
positive Regulation: MIR495 --+> chemosensitivity	30147110	10.12659/MSM.90945
positive Regulation: MIR495 --+> chemosensitivity	30147110	10.12659/MSM.90945
positive Regulation: MIR495 --+> chemosensitivity	30147110	10.12659/MSM.90945
positive Regulation: MIR495 --+> chemosensitivity	30147110	10.12659/MSM.90945
positive Regulation: MIR124-1 --+> chemosensitivity	28242198	10.1016/j.bbrc.201
positive Regulation: MIR124-1 --+> chemosensitivity	33358904	10.1016/j.lfs.2020
positive Regulation: MIR124-1 --+> chemosensitivity	28163106	10.1016/j.addr.201
positive Regulation: MIR124-1 --+> chemosensitivity	32382656	10.1016/j.omto.202
positive Regulation: MIR124-1 --+> chemosensitivity	32382656	10.1016/j.omto.202
positive Regulation: MIR124-1 --+> chemosensitivity	32382656	10.1016/j.omto.202
positive Regulation: MIR124-1 --+> chemosensitivity	25281917	10.1016/j.addr.201
positive Regulation: MIR124-1 --+> chemosensitivity	29185191	10.1007/s12015-017
positive Regulation: MIR124-1 --+> chemosensitivity	29185191	10.1007/s12015-017
positive Regulation: MIR124-1 --+> chemosensitivity	29185191	10.1007/s12015-017
positive Regulation: MIR124-1 --+> chemosensitivity	30657585	10.26355/eurrev_20

positive Regulation: MIR124-1 --+> chemosensitivity	32096187	10.26355/eurrev_20
positive Regulation: MIR124-1 --+> chemosensitivity	24861879	10.1093/neuonc/nou
positive Regulation: MIR124-1 --+> chemosensitivity	24861879	10.1093/neuonc/nou
positive Regulation: MIR124-1 --+> chemosensitivity	28242198	10.1016/j.bbrc.201
positive Regulation: MIR124-1 --+> chemosensitivity	28242198	10.1016/j.bbrc.201
positive Regulation: MIR124-1 --+> chemosensitivity	28242198	10.1016/j.bbrc.201
positive Regulation: MIR124-1 --+> chemosensitivity	29185191	10.1007/s12015-017
positive Regulation: MIR124-1 --+> chemosensitivity	29185191	10.1007/s12015-017
positive Regulation: MIR124-1 --+> chemosensitivity	29185191	10.1007/s12015-017
positive Regulation: MIR124-1 --+> chemosensitivity		10.26355/eurrev_20
positive Regulation: MIR124-1 --+> chemosensitivity		
positive Regulation: MIR124-1 --+> chemosensitivity		
positive Regulation: MIR124-1 --+> chemosensitivity	28242198	10.1016/j.bbrc.201
positive Regulation: MIR124-1 --+> chemosensitivity	33886036	10.1007/s10571-021
positive Regulation: RASA1 --+> chemosensitivity	33968197	10.3892/ol.2021.12
positive Regulation: MIRLET7C --+> chemosensitivity	33539818	10.1016/j.ejphar.2
positive Regulation: MIRLET7C --+> chemosensitivity	28571552	10.2174/1381612823
positive Regulation: MIRLET7C --+> chemosensitivity	25951903	10.1186/s12957-015
positive Regulation: MIRLET7C --+> chemosensitivity	29582468	10.1111/jop.127111
positive Regulation: MIRLET7C --+> chemosensitivity	34603447	10.1155/2021/56706
positive Regulation: MIRLET7C --+> chemosensitivity		
positive Regulation: TP53BP2 --+> chemosensitivity		10.1016/j.gendis.2
positive Regulation: MIRLET7G --+> chemosensitivity	25972194	
positive Regulation: MIR556 --+> chemosensitivity	34936303	10.1615/JEnvironPa
positive Regulation: EMP3 --+> chemosensitivity	34511602	10.1038/s41419-021
positive Regulation: EMP3 --+> chemosensitivity	34511602	10.1038/s41419-021
positive Regulation: EMP3 --+> chemosensitivity	34511602	10.1038/s41419-021
positive Regulation: SPOP --+> chemosensitivity	36198437	10.1136/jitc-2022-
positive Regulation: NOS2 --+> chemosensitivity	20338478	10.1016/j.ijrobp.2
positive Regulation: PHF19 --+> chemosensitivity	30323224	10.1038/s41419-018
positive Regulation: PHF19 --+> chemosensitivity	30323224	10.1038/s41419-018
positive Regulation: CDR1-AS --+> chemosensitivity	31542615	10.1016/j.biopha.2
positive Regulation: CDR1-AS --+> chemosensitivity	31479922	10.1016/j.omtn.201
positive Regulation: CDR1-AS --+> chemosensitivity	31542615	10.1016/j.biopha.2
positive Regulation: CDR1-AS --+> chemosensitivity	31479922	10.1016/j.omtn.201
positive Regulation: CDR1-AS --+> chemosensitivity	31479922	10.1016/j.omtn.201
positive Regulation: CDR1-AS --+> chemosensitivity	31479922	10.1016/j.omtn.201
positive Regulation: CDR1-AS --+> chemosensitivity	32655321	10.1186/s12935-020
positive Regulation: CDR1-AS --+> chemosensitivity	30884120	10.1111/jcmm.14171
positive Regulation: CDR1-AS --+> chemosensitivity	31131537	10.1002/1878-0261.
positive Regulation: CDR1-AS --+> chemosensitivity	31131537	10.1002/1878-0261.
positive Regulation: CDR1-AS --+> chemosensitivity	34400888	10.7150/IJMS.62219
positive Regulation: CDR1-AS --+> chemosensitivity	35133881	10.1089/cbr.2021.0
positive Regulation: BBC3 --+> chemosensitivity	33139695	10.1038/s41419-020
positive Regulation: BBC3 --+> chemosensitivity	21863213	10.2119/molmed.201
positive Regulation: BBC3 --+> chemosensitivity	21863213	10.2119/molmed.201
positive Regulation: BBC3 --+> chemosensitivity	21863213	10.2119/molmed.201
positive Regulation: BBC3 --+> chemosensitivity	22312312	10.3390/ijms130111
positive Regulation: BBC3 --+> chemosensitivity	26698248	
positive Regulation: BBC3 --+> chemosensitivity	16481741	10.4161/cbt.5.4.24
positive Regulation: BBC3 --+> chemosensitivity	36595102	10.1007/s12672-022
positive Regulation: BBC3 --+> chemosensitivity	36595102	10.1007/s12672-022
positive Regulation: CRYAB --+> chemosensitivity	28648665	10.1016/j.ymthe.20
positive Regulation: SMAD4 --+> chemosensitivity	23298711	10.1016/j.ejca.201

positive Regulation: SMAD4 --+> chemosensitivity	28112370	10.3892/or.2017.53
positive Regulation: SMAD4 --+> chemosensitivity	28902368	10.3892/or.2017.59
positive Regulation: SMAD4 --+> chemosensitivity	28902368	10.3892/or.2017.59
positive Regulation: SMAD4 --+> chemosensitivity	28902368	10.3892/or.2017.59
positive Regulation: SMAD4 --+> chemosensitivity	32565799	10.1155/2020/31815
positive Regulation: SMAD4 --+> chemosensitivity	24384683	10.1038/bjc.2013.7
positive Regulation: SMAD4 --+> chemosensitivity	24384683	10.1038/bjc.2013.7
positive Regulation: SMAD4 --+> chemosensitivity	24384683	10.1038/bjc.2013.7
positive Regulation: SMAD4 --+> chemosensitivity	26647806	10.3892/or.2015.44
positive Regulation: SMAD4 --+> chemosensitivity	26647806	10.3892/or.2015.44
positive Regulation: SMAD4 --+> chemosensitivity	26647806	10.3892/or.2015.44
positive Regulation: SMAD4 --+> chemosensitivity	26647806	10.3892/or.2015.44
positive Regulation: SMAD4 --+> chemosensitivity	26647806	10.3892/or.2015.44
positive Regulation: SMAD4 --+> chemosensitivity	26647806	10.3892/or.2015.44
positive Regulation: SMAD4 --+> chemosensitivity	26647806	10.3892/or.2015.44
positive Regulation: SMAD4 --+> chemosensitivity	26647806	10.3892/or.2015.44
positive Regulation: SMAD4 --+> chemosensitivity	26647806	10.3892/or.2015.44
positive Regulation: SMAD4 --+> chemosensitivity	26647806	10.3892/or.2015.44
positive Regulation: SMAD4 --+> chemosensitivity	26647806	10.3892/or.2015.44
positive Regulation: SMAD4 --+> chemosensitivity	24384683	10.1038/bjc.2013.7
positive Regulation: SMAD4 --+> chemosensitivity	26647806	10.3892/or.2015.44
positive Regulation: SMAD4 --+> chemosensitivity	32565799	10.1155/2020/31815
positive Regulation: SMAD4 --+> chemosensitivity	34957936	10.1080/16078454.2
positive Regulation: SMAD4 --+> chemosensitivity	34957936	10.1080/16078454.2
positive Regulation: SMAD4 --+> chemosensitivity	34957936	10.1080/16078454.2
positive Regulation: SMAD4 --+> chemosensitivity	34957936	10.1080/16078454.2
positive Regulation: SMAD4 --+> chemosensitivity	34957936	10.1080/16078454.2
positive Regulation: MIR214 --+> chemosensitivity	33166593	10.1016/j.lfs.2020
positive Regulation: MIR214 --+> chemosensitivity	33894206	10.1016/j.pbiomolb
positive Regulation: MIR214 --+> chemosensitivity		10.1007/s12038-020
positive Regulation: MIR214 --+> chemosensitivity		10.1007/s12038-020
positive Regulation: MIR214 --+> chemosensitivity	32884449	10.1186/s12935-020
positive Regulation: MIR214 --+> chemosensitivity	22350790	10.1007/s10549-012
positive Regulation: MIR375 --+> chemosensitivity	33253912	10.1016/j.cellsig.
positive Regulation: MIR375 --+> chemosensitivity		
positive Regulation: MIR375 --+> chemosensitivity	28079894	10.1038/cddis.2016
positive Regulation: MIR375 --+> chemosensitivity	29115164	10.1177/0300060517
positive Regulation: MIR375 --+> chemosensitivity	32626943	10.3892/mmr.2020.1
positive Regulation: MIR375 --+> chemosensitivity	23696927	
positive Regulation: MIR375 --+> chemosensitivity	27466996	
positive Regulation: MIR375 --+> chemosensitivity	31983104	
positive Regulation: MIR375 --+> chemosensitivity	32073706	10.1111/cas.143561
positive Regulation: MIR375 --+> chemosensitivity	34961460	10.2174/1566524021
positive Regulation: ST3GAL4 --+> chemosensitivity	28512058	10.1016/j.gene.201
positive Regulation: PRMT1 --+> chemosensitivity	35068392	10.2174/0929866529
positive Regulation: MIR4282 --+> chemosensitivity		
positive Regulation: VCAM1 --+> chemosensitivity	33922612	10.3390/ijms220944
positive Regulation: VCAM1 --+> chemosensitivity	24583847	10.3390/ijms150335
positive Regulation: TFAP2A --+> chemosensitivity	16533807	10.1074/jbc.M60053
positive Regulation: TFAP2A --+> chemosensitivity	16533807	10.1074/jbc.M60053
positive Regulation: TFAP2A --+> chemosensitivity	20184741	
positive Regulation: TFAP2A --+> chemosensitivity	16533807	
positive Regulation: TFAP2A --+> chemosensitivity	19672266	
positive Regulation: TFAP2A --+> chemosensitivity	19672266	

positive Regulation: TFAP2A --+> chemosensitivity	21489314	10.1186/1471-2407-
positive Regulation: TFAP2A --+> chemosensitivity	22194984	10.1371/journal.pc
positive Regulation: TFAP2A --+> chemosensitivity	22194984	10.1371/journal.pc
positive Regulation: TFAP2A --+> chemosensitivity	19672266	10.1038/sj.bjc.660
positive Regulation: TFAP2A --+> chemosensitivity	22194984	10.1371/journal.pc
positive Regulation: UNC5C --+> chemosensitivity	21600761	10.1016/j.ejca.201
positive Regulation: UNC5C --+> chemosensitivity	21600761	10.1016/j.ejca.201
positive Regulation: UNC5C --+> chemosensitivity	21600761	10.1016/j.ejca.201
positive Regulation: UNC5C --+> chemosensitivity	21600761	10.1016/j.ejca.201
positive Regulation: UNC5C --+> chemosensitivity	21600761	10.1016/j.ejca.201
positive Regulation: UPRT --+> chemosensitivity	11094317	10.1016/S0959-8049
positive Regulation: MIR939 --+> chemosensitivity	30825424	10.1016/j.clinbioc
positive Regulation: MIR939 --+> chemosensitivity	31545400	10.3892/ijmm.2019.
positive Regulation: MIR939 --+> chemosensitivity	28114937	10.1186/s12943-017
positive Regulation: MIR208B --+> chemosensitivity	33905821	10.1016/j.ymthe.20
positive Regulation: MIR208B --+> chemosensitivity	33905821	10.1016/j.ymthe.20
positive Regulation: MIR195 --+> chemosensitivity	32898724	10.1016/j.coph.202
positive Regulation: MIR195 --+> chemosensitivity	31508486	10.1016/j.omto.201
positive Regulation: MIR195 --+> chemosensitivity	31508486	10.1016/j.omto.201
positive Regulation: MIR195 --+> chemosensitivity	31508486	10.1016/j.omto.201
positive Regulation: MIR195 --+> chemosensitivity	28901478	10.3892/mmr.2017.7
positive Regulation: MIR195 --+> chemosensitivity	28901478	10.3892/mmr.2017.7
positive Regulation: MIR195 --+> chemosensitivity	29863237	
positive Regulation: MIR195 --+> chemosensitivity	30200804	10.1080/15384101.2
positive Regulation: MIR195 --+> chemosensitivity	28969047	10.18632/oncotarge
positive Regulation: MIR195 --+> chemosensitivity	29635904	10.1016/j.biopha.2
positive Regulation: MIR195 --+> chemosensitivity	33953601	10.2147/PGPM.S3027
positive Regulation: MIR195 --+> chemosensitivity	33953601	10.2147/PGPM.S3027
positive Regulation: MIR195 --+> chemosensitivity	34440380	10.3390/genes12081
positive Regulation: NR1H2 --+> chemosensitivity		10.1016/j.gendis.2
positive Regulation: NR1H2 --+> chemosensitivity	31210377	10.1002/jcb.285581
positive Regulation: NR1H2 --+> chemosensitivity	31210377	10.1002/jcb.285581
positive Regulation: ALKBH5 --+> chemosensitivity	33376625	10.1016/j.omtn.202
positive Regulation: ALKBH5 --+> chemosensitivity		10.1016/j.apsb.202
positive Regulation: ALKBH5 --+> chemosensitivity	33376625	10.1016/j.omtn.202
positive Regulation: ALKBH5 --+> chemosensitivity	36471428	10.1186/s13046-022
positive Regulation: MIR1294 --+> chemosensitivity	29949161	10.26355/eurrev-20
positive Regulation: MIR1294 --+> chemosensitivity	30536306	10.26355/eurrev-20
positive Regulation: MIR1294 --+> chemosensitivity	31114997	10.26355/eurrev_20
positive Regulation: MIR1294 --+> chemosensitivity	32329296	10.23812/20-10A
positive Regulation: MIR1294 --+> chemosensitivity	29511599	
positive Regulation: MIR1294 --+> chemosensitivity	29511599	
positive Regulation: MIR33A --+> chemosensitivity	25971209	10.18632/oncotarge
positive Regulation: MIR328 --+> chemosensitivity	29627370	10.1016/j.addr.201
positive Regulation: MIR328 --+> chemosensitivity	31193825	10.1016/j.apsb.201
positive Regulation: SAA1 --+> chemosensitivity	25442283	10.1016/j.phymed.2
positive Regulation: SAA1 --+> chemosensitivity	26176734	10.1080/15384047.2
positive Regulation: GDPD5 --+> chemosensitivity	29635904	10.1016/j.biopha.2
positive Regulation: GDPD5 --+> chemosensitivity	29635904	10.1016/j.biopha.2
positive Regulation: AMOT --+> chemosensitivity	33414519	10.1038/s41417-020
positive Regulation: AMOT --+> chemosensitivity	33414519	10.1038/s41417-020
positive Regulation: ARL11 --+> chemosensitivity	21153650	10.1007/s00404-010
positive Regulation: MIR612 --+> chemosensitivity	24704424	10.1016/j.bbrc.201
positive Regulation: MIR194-1 --+> chemosensitivity	27810403	10.1016/j.canlet.2

positive Regulation: MIR194-1 --+> chemosensitivity	26909612	10.18632/oncotarge
positive Regulation: MIR485 --+> chemosensitivity	29678577	10.1016/j.bbrc.201
positive Regulation: MIR485 --+> chemosensitivity	29678577	10.1016/j.bbrc.201
positive Regulation: MIR485 --+> chemosensitivity	29678577	10.1016/j.bbrc.201
positive Regulation: MIR485 --+> chemosensitivity	29678577	10.1016/j.bbrc.201
positive Regulation: MIR485 --+> chemosensitivity	31493469	10.1016/j.biochi.2
positive Regulation: MIR485 --+> chemosensitivity		10.1016/j.sjbs.202
positive Regulation: MIR485 --+> chemosensitivity	29678577	10.1016/j.bbrc.201
positive Regulation: MIR485 --+> chemosensitivity	29678577	10.1016/j.bbrc.201
positive Regulation: MIR485 --+> chemosensitivity	29678577	10.1016/j.bbrc.201
positive Regulation: MIR485 --+> chemosensitivity	35235157	10.1007/s11033-022
positive Regulation: TNF --+> chemosensitivity	31733340	10.1016/j.resp.201
positive Regulation: TNF --+> chemosensitivity	23972545	10.1016/j.cyto.201
positive Regulation: TNF --+> chemosensitivity	31364751	10.3892/or.2019.72
positive Regulation: TNF --+> chemosensitivity	29564744	10.1007/s12253-018
positive Regulation: TNF --+> chemosensitivity	21990224	10.1002/hed.216481
positive Regulation: TNF --+> chemosensitivity		10.3892/or.2019.72
positive Regulation: TNF --+> chemosensitivity		
positive Regulation: TNF --+> chemosensitivity	34657251	10.1007/s11033-021
positive Regulation: MIR204 --+> chemosensitivity	34015412	10.1016/j.bbcan.20
positive Regulation: MIR204 --+> chemosensitivity	29353201	10.1016/j.biopha.2
positive Regulation: MIR204 --+> chemosensitivity	28861151	
positive Regulation: MIR204 --+> chemosensitivity	30566393	10.1152/ajpcell.00
positive Regulation: MIR204 --+> chemosensitivity	31384174	10.1186/s12935-019
positive Regulation: MIR204 --+> chemosensitivity	27095441	10.1242/bio.015008
positive Regulation: MIR204 --+> chemosensitivity	28280358	10.2147/OTT.S12881
positive Regulation: MIR204 --+> chemosensitivity	28861151	
positive Regulation: MIR204 --+> chemosensitivity	32618144	10.1002/cam4.32481
positive Regulation: MIR204 --+> chemosensitivity	35163397	10.3390/ijms230314
positive Regulation: LINC00261 --+> chemosensitivity	30226808	10.1096/fj.2018007
positive Regulation: LINC00261 --+> chemosensitivity	30226808	10.1096/fj.2018007
positive Regulation: LINC00261 --+> chemosensitivity	30226808	10.1096/fj.2018007
positive Regulation: LINC00261 --+> chemosensitivity	30226808	10.1096/fj.2018007
positive Regulation: LINC00261 --+> chemosensitivity	32744688	10.26355/eurrev_20
positive Regulation: LINC00261 --+> chemosensitivity	34424120	10.1080/15384101.2
positive Regulation: LINC00261 --+> chemosensitivity	34424120	10.1080/15384101.2
positive Regulation: MIR625 --+> chemosensitivity	30988674	10.1186/s11658-019
positive Regulation: MIR625 --+> chemosensitivity	30988674	10.1186/s11658-019
positive Regulation: MIR625 --+> chemosensitivity	31115501	10.3892/ijmm.2019.
positive Regulation: MIR625 --+> chemosensitivity	28979807	
positive Regulation: MIR625 --+> chemosensitivity	35955533	10.3390/ijms231583
positive Regulation: NR4A1 --+> chemosensitivity	27264242	10.1016/j.bbcan.20
positive Regulation: NR4A1 --+> chemosensitivity		10.1016/j.jsps.202
positive Regulation: MAPK13 --+> chemosensitivity	26512696	10.3390/cancers704
positive Regulation: PCDHGB7 --+> chemosensitivity	31379979	10.1155/2019/61315
positive Regulation: PCDHGB7 --+> chemosensitivity	31379979	10.1155/2019/61315
positive Regulation: TMEM100 --+> chemosensitivity	31188741	10.1515/hsz-2019-0
positive Regulation: TMEM100 --+> chemosensitivity	31188741	10.1515/hsz-2019-0
positive Regulation: TMEM100 --+> chemosensitivity	34422038	10.1155/2021/55523
positive Regulation: TMEM100 --+> chemosensitivity	34422038	10.1155/2021/55523
positive Regulation: CTBP1 --+> chemosensitivity	33613771	10.7150/jca.521151
positive Regulation: MIR4306 --+> chemosensitivity	34021861	10.1007/s12013-021
positive Regulation: LRIG1 --+> chemosensitivity	29230082	10.1155/2017/96735
positive Regulation: LRIG1 --+> chemosensitivity	29230082	10.1155/2017/96735

positive Regulation: LRIG1 --+> chemosensitivity	30182330	10.1007/s12017-018
positive Regulation: LRIG1 --+> chemosensitivity	25695283	10.3892/or.2015.38
positive Regulation: LRIG1 --+> chemosensitivity	25048475	10.3349/ymj.2014.5
positive Regulation: LRIG1 --+> chemosensitivity	25048475	10.3349/ymj.2014.5
positive Regulation: LRIG1 --+> chemosensitivity	25048475	10.3349/ymj.2014.5
positive Regulation: LRIG1 --+> chemosensitivity	25449296	10.1007/s12013-014
positive Regulation: LRIG1 --+> chemosensitivity	25449296	10.1007/s12013-014
positive Regulation: LRIG1 --+> chemosensitivity	25449296	10.1007/s12013-014
positive Regulation: LRIG1 --+> chemosensitivity	25695283	10.3892/or.2015.38
positive Regulation: LRIG1 --+> chemosensitivity	25695283	10.3892/or.2015.38
positive Regulation: LRIG1 --+> chemosensitivity	25801120	10.1016/j.brainres
positive Regulation: LRIG1 --+> chemosensitivity	29450946	10.1111/cas.135381
positive Regulation: MIR365A --+> chemosensitivity	30464615	10.2147/CMAR.S1748
positive Regulation: TCF4 --+> chemosensitivity	36116131	10.1002/ctm2.1042
positive Regulation: TSC22D3 --+> chemosensitivity	24895125	10.1074/jbc.M114.5
positive Regulation: FBXL5 --+> chemosensitivity	26506454	10.1016/j.semcance
positive Regulation: FBXL5 --+> chemosensitivity	25249620	10.1093/nar/gku876
positive Regulation: CERS2 --+> chemosensitivity	30953657	10.1016/j.plipres.
positive Regulation: CERS2 --+> chemosensitivity	24021978	10.1016/j.bbalip.2
positive Regulation: CERS2 --+> chemosensitivity	24021978	10.1016/j.bbalip.2
positive Regulation: CERS2 --+> chemosensitivity	27241665	10.1016/j.canlet.2
positive Regulation: CERS2 --+> chemosensitivity	25720066	10.1515/hsz-2014-0
positive Regulation: CERS2 --+> chemosensitivity	29138865	10.3892/or.2017.60
positive Regulation: CERS2 --+> chemosensitivity	30988071	10.1042/BSR2018174
positive Regulation: CERS2 --+> chemosensitivity	22580606	10.1038/onc.2012.1
positive Regulation: CERS2 --+> chemosensitivity	22580606	10.1038/onc.2012.1
positive Regulation: CERS2 --+> chemosensitivity	22580606	10.1038/onc.2012.1
positive Regulation: CERS2 --+> chemosensitivity	22580606	10.1038/onc.2012.1
positive Regulation: CERS2 --+> chemosensitivity	22580606	10.1038/onc.2012.1
positive Regulation: CERS2 --+> chemosensitivity	22580606	10.1038/onc.2012.1
positive Regulation: CERS2 --+> chemosensitivity	22580606	10.1038/onc.2012.1
positive Regulation: CERS2 --+> chemosensitivity	22580606	10.1038/onc.2012.1
positive Regulation: CERS2 --+> chemosensitivity		10.1016/j.drup.202
positive Regulation: CERS2 --+> chemosensitivity	35734966	10.14670/HH-18-483
positive Regulation: PDGFRA --+> chemosensitivity		10.1016/j.jamcolls
positive Regulation: DIABLO --+> chemosensitivity	28424988	10.1007/s10495-017
positive Regulation: DIABLO --+> chemosensitivity	24959694	10.1371/journal.pc
positive Regulation: DIABLO --+> chemosensitivity		
positive Regulation: DIABLO --+> chemosensitivity	21863213	10.2119/molmed.201
positive Regulation: DIABLO --+> chemosensitivity	22052903	10.1074/jbc.M111.2
positive Regulation: DIABLO --+> chemosensitivity	20210890	10.1111/j.1440-184
positive Regulation: DIABLO --+> chemosensitivity	24317109	10.3892/mmr.2013.1
positive Regulation: DIABLO --+> chemosensitivity	25807633	10.7754/clin.lab.2
positive Regulation: MIR205 --+> chemosensitivity	32353488	10.1016/j.jconrel.
positive Regulation: MIR205 --+> chemosensitivity	33152930	10.1016/j.biopha.2
positive Regulation: MIR205 --+> chemosensitivity	23279926	10.1016/j.ejcb.201
positive Regulation: MIR205 --+> chemosensitivity	27362808	10.1038/cddis.2016
positive Regulation: MIR205 --+> chemosensitivity	27362808	10.1038/cddis.2016
positive Regulation: MIR205 --+> chemosensitivity	27362808	10.1038/cddis.2016
positive Regulation: MIR205 --+> chemosensitivity	27362808	10.1038/cddis.2016
positive Regulation: MIR205 --+> chemosensitivity	27362808	10.1038/cddis.2016
positive Regulation: MIR205 --+> chemosensitivity	27362808	10.1038/cddis.2016
positive Regulation: MIR205 --+> chemosensitivity	27362808	10.1038/cddis.2016
positive Regulation: MIR205 --+> chemosensitivity	33650661	10.3892/or.2021.79

positive Regulation: MIR205 --+> chemosensitivity	24568460	10.7314/APJCP.2014
positive Regulation: MIR205 --+> chemosensitivity	25308719	
positive Regulation: MIR205 --+> chemosensitivity	27271572	10.1371/journal.pc
positive Regulation: MIR205 --+> chemosensitivity	23073476	10.1016/j.canlet.2
positive Regulation: MIR205 --+> chemosensitivity	24147037	10.1371/journal.pc
positive Regulation: MIR205 --+> chemosensitivity	27271572	10.1371/journal.pc
positive Regulation: MIR205 --+> chemosensitivity	27362808	10.1038/cddis.2016
positive Regulation: MIR205 --+> chemosensitivity	30982496	10.3727/096504018X
positive Regulation: MIR205 --+> chemosensitivity	32277654	
positive Regulation: MIR205 --+> chemosensitivity	32277654	
positive Regulation: MIR205 --+> chemosensitivity		10.3892/or.2021.79
positive Regulation: MIR205 --+> chemosensitivity	34453639	10.1007/s10555-021
positive Regulation: MIR205 --+> chemosensitivity	34839317	10.1097/CM9.000000
positive Regulation: MAEA --+> chemosensitivity	28931648	10.1042/BCJ2016078
positive Regulation: XBP1 --+> chemosensitivity	27045680	10.1016/j.jsbmb.20
positive Regulation: GLI3 --+> chemosensitivity	34453645	10.1007/s11010-021
positive Regulation: BAK1 --+> chemosensitivity	11803466	
positive Regulation: BAK1 --+> chemosensitivity	30592293	10.3892/or.2018.69
positive Regulation: BAK1 --+> chemosensitivity	30592293	10.3892/or.2018.69
positive Regulation: BAK1 --+> chemosensitivity	30592293	10.3892/or.2018.69
positive Regulation: BAK1 --+> chemosensitivity	33312200	10.1155/2020/26790
positive Regulation: BAK1 --+> chemosensitivity	23497288	10.1186/1475-2867-
positive Regulation: BAK1 --+> chemosensitivity	11803466	10.1038/sj.onc.120
positive Regulation: FA2H --+> chemosensitivity	30738828	10.1016/j.ebiom.20
positive Regulation: SLC27A4 --+> chemosensitivity	26662804	10.1007/s13277-015
positive Regulation: SLC27A4 --+> chemosensitivity	26662804	10.1007/s13277-015
positive Regulation: SST --+> chemosensitivity	14769829	
positive Regulation: SST --+> chemosensitivity	18785538	
positive Regulation: SST --+> chemosensitivity	19950772	
positive Regulation: SST --+> chemosensitivity	20359855	10.1016/j.biopha.2
positive Regulation: SST --+> chemosensitivity	20359855	10.1016/j.biopha.2

2295
2295
229510.18632/aging.202295
06662012071506321O.2174/156800962066620120715063 2
000895
60410.3892/ol.2018.9604
60410.3892/ol.2018.9604
08
0.1113/JP277052

586
ccr-0798-3
2019.01.010
899927
899927
2012.10.003
2012.10.003
2021.11014610.1016/j.cellsig.2021.110146
03
CCR-04-0242
4.08.150
4.08.150
20.12.007
2016.11.139
2016.11.139
2018.04.153
2018.04.153
2018.04.153
2018.06.050
2018.10.061
0308
3-0343-1
0076
61.6.4
3.19310.1038/cddis.2013.193
694469310.1109/EMBC.2014.6944693
2018.04.15310.1016/j.biopha.2018.04.153
2018.04.15310.1016/j.biopha.2018.04.153
2018.04.15310.1016/j.biopha.2018.04.153
27010099
27010099
711
4
-07040-2
2.062
-09614-z
-09614-z
758508
758508
2018.05.046

'58508
'58508
'58508
'58508
239308
)2006_21640
'5850810.1074/jbc.M116.758508
-04249-4
2017.06.181
3-0270-x
3-0270-x
3-0270-x
ne.0088827
3-0270-x10.1186/s12964-018-0270-x
3-0270-x10.1186/s12964-018-0270-x
t.2199610.18632/oncotarget.21996
2-00354-4
2-01622-910.1186/s12943-022-01622-9
2-01622-9
2-01622-9
2-01622-9
2-01622-9
2018.08.010
)-00551-1
)-00551-1
0.1002/jps.24128
0.1002/jps.24128
1.11.029
0.01.015
r.2004.04.003
7.09.001
0.12.001
logy.2008.04.003
2006.02.002
.12.025

3188
)-1498-7_10
)-1498-7_1

25
-0640-y
3.302
3-9557-0
one.0042985
3-0907-6
7-5590-1-5
260
41
00803
01774

7-09-11177310.1182/blood-2007-09-111773
CCR-09-122910.1158/1078-0432.CCR-09-1229
0.01.01110.1016/j.tox.2010.01.011
0.1172/JCI43897
t.244
08356-3_7
2014.01.027
2.13.4.1425
2.13.4.1425
2.13.4.1425

10.1002/ijc.31197

00
00
00
0010.1042/BSR20200390
-00613-6
2010.11.028
367
one.003150710.1371/journal.pone.0031507
13.00149
13.00149
13.00149
2017.08.084
041
624
4593
02001_20043
02002_20351
31010.3892/or.2016.4810
10.1002/jbt.22168
-04271-6
-04271-6
05.04.023
5552
0778
60

57
571
571
one.0072615

5-4691-5
54510.2147/CMAR.S246545
2013.05.017
4023

031
4634
4634
3
1-02985-1
-13-35
114562725373798
114562725373798
114562725373798
CCR-11-049410.1158/1078-0432.CCR-11-0494

3
1-03171-z
2625
003.10.024
003.10.024

1-01509-7
2019.109495
2020.111099
019.02.002
01905_17800
019.02.002
019.02.002
5
494
10.1002/jbt.21888
20.08.019
20.08.019
9
er.2021.05.016

2018.1433502
75
75

1.153373
2017.06.089
2017.06.089

39
2019.1635870
2019.1635870
2019.1635870
533
533
2017.06.08910.1016/j.biopha.2017.06.089
2017.06.08910.1016/j.biopha.2017.06.089
.133410.3892/mmr.2020.11334
.-00282-9
2022.03.014
'
'
ne.0056197

-10-5810.1186/1471-2407-10-58
).118255
).118255
2016.09.004
nc.2017.10.003
).08.005
2015.03.045
)16.01.004
5-5050-x
5-5050-x
5-5066-2
7697553
).08.005
546
546
2455
54610.3892/or.2014.3646
54610.3892/or.2014.3646
)-01441-210.1186/s12935-020-01441-2
2021.189944110.1080/1120009X.2021.1899441
2021.189944110.1080/1120009X.2021.1899441
.66
2-00728-y10.1007/s13577-022-00728-y10.3322/caac.2155110.1016/j.cell.2012.02.00510.
2-00728-y
2-00728-y
2-00728-y
2020.109665
2020.109665
2020.109665
2020.109665
2020.109665
25
25
25
25
25
25

25
25
25
2510.12659/MSM.917625
2510.12659/MSM.917625
16.09.040
16.09.040
16.09.040
2020.100987
2021.101109
2018
3-1639-8
694469310.1109/EMBC.2014.6944693
16.09.04010.1016/j.bbrc.2016.09.040
2022.05.024
2022.2132008
0.12.004
0.12.004
3(02)00119-2
2010.11.028
0-07523-8
et.655410.18632/oncotarget.6554

CCR-13-058210.1158/1078-0432.CCR-13-0582
1.02.018
0-03115-3
0-0858-7
0-0858-7
0-0858-7
0-0858-7
0-0858-710.1038/s41388-019-0858-7
2
3.450
3.450
0-0374-7
21.10091810.1016/j.gore.2021.100918
1.09.005
11.08.001
09204
09204
09204
09204
1.106842
39910.2147/CMAR.S277399
00000001359
2-07963-4
2292
2020.02.002
2012.04.013
5.08.630
3-2831-7
3-2831-7
0-03505-9
0-03505-9

32
-0529-110.1007/s11060-011-0529-1
urol.51.853
18.10.009
18.10.009
.152468
017.04.005
0.08.004
0.05.011
018.06.002

-0365-3
-0926-6
-0991-5
-0390-y
952
16
666200420144805
2010_23420
-01491-0
020e10390
ne.008687210.1371/journal.pone.0086872
-0220-710.1186/s13045-015-0220-7
-0220-710.1186/s13045-015-0220-7
.152468
-0926-6
-0991-5
-0390-y
952
16
3610.1155/2021/3267536
018.09.024
019.09.008
017.12.088
018.02.118
18.11.005
r.2015.09.014
0259
610.1038/onc.2015.96
610.1038/onc.2015.96
020.08.027

-06193-4
6
.1002/mc.22536
-06193-410.1007/s11033-021-06193-410.1016/j.gene.2019.14430110.1186/s40169-018-01
-06193-410.1007/s11033-021-06193-410.1016/j.gene.2019.14430110.1186/s40169-018-01
20610.3390/genes12081206
37
37
00707N705

es.12758
2019.167648510.1080/21655979.2019.1676485
32200
019.188336
2006.02.002

.99
559

3298

3210.2147/OTT.S196832
.8.08.107
.8.08.107
.8.08.107
.8.08.107
.8.08.107
.5.01.003
).1002/iub.1133
)56010.3892/etm.2020.9560
)56010.3892/etm.2020.9560
-14-686
-14-686
-14-686
)-2656-010.1038/s41419-020-2656-0
)17.02.004
.7.05.006
2010.02.001
2012.05.005
3.04.006
1.11.010
5-1237-2
.15
.61102N528
)-2390-7
.-0034-6
e31825e0caa
.76
5-4693-3
2010.02.00110.1016/j.canlet.2010.02.001
.7610.1038/onc.2012.176
2-0599-510.1007/s13277-012-0599-5
542
)000000080410.1097/CAD.0000000000000804

nes902020710.3390/biomedicines9020207
)-00607-9
)-00607-9
)-00607-9
)-00607-9
)-00607-9
)-00607-9

)-00607-9
462

3.450
3.450
er.2019.05.012
3050

)-2557-2
)2006_21665
2020.1761238
4.148
4.148
4.148
4.148
4.148

.949
769563 10.3389/fonc.2021.769563
7698364
1-05101-2
3-1586-x10.1007/s11010-013-1586-x
021.188552
20.06.028
7-0053-8
611699 10.3389/fonc.2020.611699
2764
2764
2764
2764
2764
2764
2021.04.001
2021.04.001
.0381
2017.08.084
018.08.010
018.08.010

23
6-5671-810.1245/s10434-016-5671-8
7698356
6-0341-z
1-04413-0
2021.02.007
2016.160204
2618
2618

one.007762310.1371/journal.pone.0077623
410.2147/OTT.S76484
2016.160204

7.11.043
79
42713
MCT-07-0449
4.2.9510.4162/nrp.2020.14.2.95
8.10.004
-0858-7

7710.2147/DDDT.S269277
7710.2147/DDDT.S269277
2002_20346
-2737-y
4593
4593
4593
4593
4593
4593
694469310.1109/EMBC.2014.6944693
459310.3892/ijmm.2020.4593
459310.3892/ijmm.2020.4593
210.2147/OTT.S281802
2017.07.056
.02.009
.12.034
5.03.153
0.03.082
(01)00733-X
(03)00078-9
(03)00397-6
.03.009
.10.048
(05)66150-2
(05)67323-5
99907000-00017
(02)01900-8
(02)02177-X
3.09.011
2005.01.018
(03)00282-8
(03)00282-8
04.12.053
04.12.053
2005.12.002
1.11.029
(01)00230-6
.10.003
(99)00345-6
(00)00480-8
(03)00088-0
004.03.044
005.06.002
006.06.013

2008.06.006
)9.04.008
.1.07.052
.2.01.099
2.03.004
)7.06.006
)7.06.006
).2005.10.006
2006.03.009
)4.10.014
)5.04.044
3(03)00066-0
2005.08.002
2009.03.008
.0.12.001
4(03)00084-4
3(03)00078-3
).08.016
g.2006.08.001
col.2012.08.003
2.03.004
)12.12.025
2013.08.028
2014.01.011
.4.08.014
2014.09.001
2014.11.016
.4.03.007
.4.03.007
2014.11.016

77
.0.7150/jca.14506
2016.1215390
37710.18632/aging.103377

.0.7150/jca.14506

5-3869-x
1-0091-8
5-2617-2
5561
5561
240R
240R
006.08.001

399
1

061
one.0036940
528
528
3-0690-0
4613
.80125N55
154
0-0910-2
.1121
38
38
0-03185-310.1038/s41419-020-03185-3
0-03185-3
091
020.100780
0-01729-3
er.2021.03.010
21.07.022
022.01.001
0.108571

).108571
347
2017.1356508
).10857110.1016/j.abb.2020.108571
2-07590-z
21.01.052
)47410.1515/med-2022-0474
).101597
)
)
)10.14670/HH-18-130

2011.04.004

)-03878-3
-0215-RA
CAN-06-2310
21.22.5.151310.31557/APJCP.2021.22.5.1513

57710.3892/or.2014.3677
t.917910.18632/oncotarget.9179
.71526310.3389/fmolb.2021.715263
7665
7665
766510.3892/mmr.2017.7665
5-5048-4
.0.1038/srep40187
2018.10.061
)1908_18559
.0.1002/path.4743
CAN-16-0937
2-04480-7
).1002/cbf.3604
).1002/cbf.3604
2018.10.005
).108480
.8.12.055
2021.174006
2017.02.020
)0
)0
)0
)0
)0
.70112N25
ne.0120258
551
)10.1038/onc.2014.9
)010.1038/onc.2016.90
2-00348-2
2020.12.01710.1016/j.canlet.2020.12.017
2020.12.01710.1016/j.canlet.2020.12.017
2021.05.02410.1016/j.canlet.2021.05.024

51010.3390/ijms17040610
2016.11.090
020.03.024
er.2020.05.011
2014.07.011
709991
.82
4751
530
-13-21
-13-21
-13-21
one.0114419
2014.1001287
5-4409-8
4-1821-410.1007/s13277-014-1821-4
2014.00119
5618849
-83282-7_1
0018
396
519
100116
1-03866-y

.2152-20190813-00517
.881
.64610.3892/ol.2020.11646
596609
0-0858-7
0-0858-7
0-0858-7
0-0858-7
2-01887-8
.9
515
515
515
2020.1727700
0-0137-3
0179

2-07401-5
1-02149-510.1186/s13046-021-02149-510.1136/bmj.l437310.1053/j.gastro.2017.08.02310
1-02149-5
2-02278-510.1186/s13046-022-02278-5
2-02278-5
2011.11.018
0-01750-4
0-01750-4
0-01750-4

-01750-4
-01750-4
-01750-4
CAN-10-204810.1158/0008-5472.CAN-10-2048
9510.3892/ol.2017.5895
9510.3892/ol.2017.5895
.04.063
018.01.041
021.05.004
020.110799

15742472027401
15742472027401
0.1159/000490027
1574247202740110.3727/096504019X15742472027401
017.08.084
-0030-7
-0030-7
-0030-7
-0694-9
8
t.1073610.18632/oncotarget.10736
229
229
22910.3892/mmr.2017.8229

2016.03.012
.07.030
4.01.108
-1261-0
10.1002/cbin.11789
10.1002/cbin.11789
10.1002/cbin.11789
iomed.2017.07.008
666170818161132
4019
4019
ne.0092847
ne.0092847
ne.0092847
t.654510.18632/oncotarget.6545
t.654510.18632/oncotarget.6545

.00840
0.1111/cas.14733
20610.3390/genes12081206
10.0310.21037/tcr.2019.10.03
r.2022.05.012

2.03.003

t.695110.18632/oncotarget.6951
5
628
628
908
908
908
908
908

90810.18632/aging.202908
13
13
016.08.026
-01402-9
-01402-910.1186/s12935-020-01402-9
944
-03891-6
-1145-3
[illegible]
[illegible]
t.2514710.18632/oncotarget.25147
.03.011
32-41
-0596-4
.05.001
.05.001
.05.001
.01.013
2003_20680
91029N1102
91029N1102
91029N1102
91029N1102
666210706113102
10.1093/jb/mvaa036
8.08.019
015.1040963
015.104096310.1080/15384047.2015.1040963
67510.3892/etm.2017.5675
67510.3892/etm.2017.5675
3010.2147/CMAR.S333830
-2557-2
-2557-2
-2557-2
-2557-2
-2557-2
-2557-2
-2557-2
-2557-2
89

[illegible](03)00854-4
[illegible]

[illegible]332844
[illegible]8.04.005
[illegible].152726
[illegible]021.04.006
[illegible]19.05.046

[illegible]42059-2_5
[illegible]-2827-6
[illegible]1609753
[illegible]30-20
[illegible]30-20
[illegible]30-20
[illegible]30-20
[illegible]30-20
[illegible]ne.0096228
[illegible]0503
[illegible]46
[illegible]25
[illegible]-07010-8
[illegible]-07040-2
[illegible]90106N18
[illegible]90106N18
[illegible]90106N18
[illegible]90106N1810.4149/neo_2019_190106N18
[illegible]90106N1810.4149/neo_2019_190106N18

[illegible]-2253-x
[illegible]904847
[illegible]904847
[illegible]904847
[illegible]904847
[illegible]904847
[illegible]904847
[illegible]2
[illegible]-0972-x
[illegible]-2108-y10.1007/s00280-013-2108-y
[illegible]77152810.3389/fonc.2021.771528
[illegible]7.11.164
[illegible]020.173094
[illegible]0.144629
[illegible]0.144629
[illegible]0.144629
[illegible]0.144629
[illegible]0.14462910.1016/j.gene.2020.144629
[illegible]05
[illegible]3082110.3390/cancers14030821
[illegible].07.046
[illegible]9.06.010

9.06.010
0000000154
.1002/iub.1889
c050
r.2020.05.017
64
64
64
64
64
64
.117
[illegible]
[illegible]
-1509-1
-1509-1
694469310.1109/EMBC.2014.6944693
-04208-z
5.12.007
600
5.12.00710.1016/j.gene.2015.12.007
CCR-11-199010.1158/1078-0432.CCR-11-1990
3
019.07.005
019.07.005
019.07.005
019.07.00510.1016/j.canlet.2019.07.005
4.06.041
6.01.014
6.01.014
6.01.014
6.01.014
6.01.014
6.01.014
-0422-8
6.01.01410.1016/j.bbrc.2016.01.014
6.01.014
-01868-910.1007/s00109-019-01868-9
-5157-0
-5157-0
-5157-0
-5157-0
-5157-0
-5157-0
-5157-0
.0066610.3389/fphar.2020.00666
017.1416936
017.1416936
017.1416936
017.1416936
017.1416936
60
7610.3892/ol.2017.6076
8

-04337-8
94
38
8.04.005
.08.005
015.07.018
015.07.018
015.12.002
898
-0134-z
8
8
1

ne.007762310.1371/journal.pone.0077623
0000001928
310.2147/OTT.S232953

1.03.008
8310.20517/cdr.2019.83
52
2009.05.054
2009.05.054
-0211-4
399
399
399
399
011
13-25
13-25
0
0
0
0
600
600
600
600
60010.1248/cpb.c20-00600

-0930-0
-0930-010.1186/s13046-018-0930-0
.11.014
.11.014
20610.3390/genes12081206
666200519075908
.03.011
1433610.1111/1759-7714.14336
1433610.1111/1759-7714.14336
.01.019
020.110768
1.105442
2.11.086

)
7666170222123406
)373
)373
550
595
595
595
353
X14298122915628

59510.3892/or.2013.2695
59510.3892/or.2013.2695
59510.3892/or.2013.2695
35310.3892/or.2015.3853
X1429812291562810.3727/096504015X14298122915628
)72
.5.12.060

es.12596
3-0602-8
4504
)666200226124336

7-0649-310.1186/s13046-017-0649-3
.9.144126
2014.07.026
)244.2018
)48467
)-00605-4
2014.07.02610.1016/j.canlet.2014.07.026
2014.07.02610.1016/j.canlet.2014.07.026
7-0033-810.1038/s41408-017-0033-8
)-01550-y
2016.12.001
530
50210.1155/2017/9032502
2-00988-510.1038/s41420-022-00988-5

)666190819151946
2016.10.035
)-03282-3
)-0168-710.1186/s11658-019-0168-7
-00379310.1136/jitc-2021-003793
-00379310.1136/jitc-2021-003793
-00379310.1136/jitc-2021-003793
)-03149-210.1007/s00432-020-03149-210.1111/dgd.1203910.1038/onc.2014.4010.1172/JCI
)-03165-210.1007/s00432-020-03165-2
)-03165-210.1007/s00432-020-03165-2

5.208
5.208
5.208
5.208
5.208
5.208
0.118255
0.111309
017.02.004
2020.110395
19.03.005
19.03.005
-42059-2_11
3811
3811
3811
3811
3790
3-0834-z
3-0834-z
3-0834-z
3-0834-z
19.03.005
19.03.005
1902
192
0967818
0967818
0967818
0999201214234421
0.1111/os.12442
18.10.543
047
047
047
047
047
5.12.013
2020.110768
nes10092240
3.05.080

3006
52
52
0-1715-x
0-1715-x
0-1715-x
0-1715-x
0-1715-x
0-1715-x
0-1715-x
0-1715-x

-1715-x
-00237-w

026
t.683
-1715-x10.1038/s41419-019-1715-x
-1715-x10.1038/s41419-019-1715-x
666210120141546
-3651-3
-3651-3
21.188552
64.15.10
64.15.10
64.15.10
64.15.10
64.15.10
64.15.10
64.15.10

48467
logy.2003.07.001
018.03.088
118.023648
t.2009910.18632/oncotarget.20099
t.2009910.18632/oncotarget.20099
6.09.040
017.08.084
020.100987
8.11.009
697575
697575
697575
87
87
-00286-3
-00286-3
-00286-3
61
61
ne.0150026
ne.0150026
ne.0150026
ne.0150026
-4528-2
694469310.1109/EMBC.2014.6944693
-4528-210.1007/s13277-015-4528-2
ne.015002610.1371/journal.pone.0150026
23810.3892/etm.2019.8238
-00286-310.1186/s40659-020-00286-3
20610.3390/genes12081206
.282
.282
020.112758

.-01768-8
CCR-18-053210.1158/1078-0432.CCR-18-0532
).108480
2019.108800
ology.2020.104916
ology.2020.104916
021.102159
015.03.027
015.03.027
015.03.027
nber.9.5

7.178
414
414
7703825
05
.0430
09
5053
5053
5053
5053
5053
one.0094065
557
557
557
557
557

5-3831-2
5-3831-2
5-3831-2
5-3831-2
5-3831-2
5-3831-2
505310.3390/ijms131216053
505310.3390/ijms131216053
32.1134710.3748/wjg.v20.i32.11347
4-2814-z10.1007/s13277-014-2814-z
55710.3892/or.2014.3657
5-3831-210.1007/s13277-015-3831-2
714510.1093/carcin/bgv145
).1002/mc.22612
41410.1038/onc.2016.414
10.1159/000460505
10.1159/000460505
.-04249-4

)240
2022.021510
)21.188552

3361
.1553
3148
2013.05.037
7-0263-x
.97210.3892/ol.2020.11972
.9.02.093
2018.02.118
77
2017.1367071
5.461
3-1176-7
3-1176-7
275
5.46110.1038/cddis.2016.461
)774
.-04337-8
57
.0.1111/bjh.15878
:hem.2019.01.002
.3382
.3382
3940
:t.1767710.18632/oncotarget.17677
:t.1767710.18632/oncotarget.17677
.0.1002/jcp.27288

314
3
5-4559-8
5-4559-8
5-4559-8
5-4559-8
5-4559-8
)ne.0040008
)ne.0053436
5-0402-5
5-4559-8
5.07.003
2021.101090

34
34
34
.MCT-12-0534-T10.1158/1535-7163.MCT-12-0534-T
.MCT-12-0534-T10.1158/1535-7163.MCT-12-0534-T
3410.1042/BSR20150084
)-00500-010.1007/s13402-020-00500-010.1016/j.eururo.2016.06.01010.1002/msj.202241C
.-00615-y10.1007/s13402-021-00615-y
.666210902113824

.7.03.162
)-1415-6
.7.03.16210.1016/j.bbrc.2017.03.162
571

)-00742-4
et.1378910.18632/oncotarget.13789
et.1378910.18632/oncotarget.13789
.-00198-8

253
5-0328-5
.49
2-00414-610.1038/s41389-022-00414-6
23.e6710.4048/jbc.2020.23.e67
.-02019-0
.-02019-0
2018.09.031
.5.08.053
5.04.011
5956
)308
3-3548-1
)283.2017

2-07227-1
34910.3892/ol.2018.8849
)21.188552
.8-0141
1902
)-03123-3
535410.3892/etm.2017.5354
500710.3892/etm.2018.6007
500710.3892/etm.2018.6007
.0.7150/jca.31191
2-04791-z
2-04791-z
2-04791-z
.4.01.014
.4.01.014
.4.01.014
2021.173923
2021.173923
.2018.10.031

3-0651-4
.0.1159/000492260
.(10)60010-310.1016/S1674-8301(10)60010-3
.(10)60010-310.1016/S1674-8301(10)60010-3
1.15.6.2619
.2017.01.001

2018.06.114
2018.06.114
2020.110768
er.2020.05.017
7695964
2018.171124
409
409
409
00
2910.1093/abbs/gmv129
2910.1093/abbs/gmv129
2018.06.11410.1016/j.biopha.2018.06.114
2018.06.11410.1016/j.biopha.2018.06.114
2018.06.11410.1016/j.biopha.2018.06.114
er.2022.08.003
6.03.083
6.03.083
-2205-5
-2205-5
-2205-5
-2205-510.1007/s00432-016-2205-5
711089
711089
71108910.1177/1010428317711089
2018.07.024
2018.07.024
2020.111111
0566
0566
2018.07.024
2018.07.024
2022.113813
-11-54
902
-0965-9
ne.0053436
.152612
2018.08.052
2020.111099
-4988-z
-0432-8
0000000745
2001_20038
666191112142943
-00975-0
010.1002/cbin.10100
1810.3892/or.2014.3318
2710.1093/jjco/hyv027
t.1483910.18632/oncotarget.14839
-0981-7
-01211-8

1810.3892/or.2014.3318

294RR
1-02474-y
2841
353
375
2-01627-410.1186/s12943-022-01627-4
2-01627-4
0.153266
2020.109858
530
0-03906-4
0-03906-4
1-02907-1
5205
31810.3892/or.2014.3318
5-0178-710.1186/s13048-015-0178-7
0-1082-910.1186/s12935-019-1082-9
0-1082-910.1186/s12935-019-1082-9
0-1082-910.1186/s12935-019-1082-9

0-03906-410.1007/s11010-020-03906-4

31810.3892/or.2014.3318
27010099
281
0823
079525
079525
2018.10.080
one.0055513
375
2-08-2501
5(02)01900-8
2010.01.030
7(00)01742-9
2004.03.044
2004.05.023
2009.03.003
3(98)00018-3
2010.12.006
2012.12.002
2012.12.002

317
one.0090180
2345

0

ɔne.0036722
5-0668-710.1007/s12013-015-0668-7
)0000001377

)1805_15067
306610.3892/etm.2019.8066
306610.3892/etm.2019.8066
5269
240R
240R
240R
240R
ɔne.0136484
ɔne.0136484
ɔne.0136484
ɔne.0136484
ɔne.013648410.1371/journal.pone.0136484
bio.300042510.1371/journal.pbio.3000425
)0000002018
)0000002018
)0000002018
3-0390-7
1610
180805N574
1.11940510.1016/j.lfs.2021.119405
)247
)1910_19155
)1910_19155

)-08592-5
)-08592-5
)10.1111/hepr.12500
)10.1111/hepr.12500
)-08592-510.1007/s12017-020-08592-5
)-08592-510.1007/s12017-020-08592-5
ɔlogy.2017.02.015
CAPR-15-0338
g
g
1918
et.3165
g10.1039/c6mb00678g
X1545218788883910.3727/096504018X15452187888839
)-0084-310.1038/s41392-019-0084-3
)-0084-310.1038/s41392-019-0084-3
2-01718-z10.1007/s10495-022-01718-z10.1016/j.critrevonc.2020.10298810.1016/S0925-5
3683
368310.3892/ijmm.2018.3683

et.1912

-005270
-005270
-005270
one.0068990
)66
)66

)66
)66
7-3930-010.1186/s12885-017-3930-0
7-3930-0
7-3930-0
et.2199610.18632/oncotarget.21996
2-00354-4
7705766
7705766
7705766
7705766
)11.09.003
.797
.797
.797
70410.3892/or.2014.3704
379
244
3610.1530/JME-19-0186
786733405

2709
)9221
-69080-3_4410.1007/978-0-387-69080-3_44
.6.01.090
[illegible]
CAN-09-318610.1158/0008-5472.CAN-09-3186
CAN-09-318610.1158/0008-5472.CAN-09-3186
739
-14-781
2020.110768
2018.05.079
es.11349
es.11349

.8.03.013
.8.03.013
459
)0000000572
)-2053-8

2-0118-y
2012.01.01210.1016/j.cellsig.2012.01.012

.8.03.01310.1016/j.gene.2018.03.013
.-04337-8
338010.3892/ol.2022.13380
5-3354-y
2018.1433502
).01.003
324
324
324
2019.172664
biomed.2012.05.041
biomed.2012.05.041
.82
.82
.82
.82
.82
.82
.82
.82
2709
2709
.8210.1155/2020/6452182
5295
es.12667
)95
et.2675810.18632/oncotarget.26758
.0.1002/cam4.2547
.0.1111/cas.14361
)6610.1093/abbs/gmab066
2-00535-110.1007/s10616-022-00535-1
2-00535-1
2018.08.059
1118-18.2019
2018.10.061
35010.3892/ol.2018.8850
35010.3892/ol.2018.8850
2015.06.010

)12
.82
.82
.82
)01110

.8210.1038/onc.2011.182
-31-6310.1186/1756-9966-31-63
546810.3892/etm.2017.5468
.0.7150/jca.52115
2016.09.058
2020.100769
2008.05.045
518
)885083

)885083
2020.100769
317
1-00583-5

CAN-04-2501
et.3896
365
3.120
36
3.120

10.7150/jca.18171
3168
740
180
one.0056197
310.1038/leu.2008.8
310.1038/leu.2008.8
7117.17
)18.07.032
)18.07.032
)875.2005
)8.07.00310.1016/j.resp.2008.07.003
)3710.1002/cphy.c100037
)19.03.016
2008.03.055
5666200318130625

320
).4161/cc.24497
10.1002/jcp.27196
MCR-19-085610.1158/1541-7786.MCR-19-0856
1-03793-2
1-03793-2
).111309
2014.03.013
5.461
259
259
7-2143-0

417
417
23
)1812_16630
1-01853-6
2017.01.05810.1016/j.biopha.2017.01.058
)-1512-510.1186/s13046-019-1512-5
417
417

23
)1812_16630

525

492
2-04976-6
2-00427-8
5.10.023
5.10.023

7-0097-1
398110
7-0097-110.1038/s41419-017-0097-1
5-5244-2
4037
310
X15420734828058
X15420734828058
X15420734828058
X15420734828058
9-1086-z
X1542073482805810.3727/096504018X15420734828058
X15420734828058
X15420734828058
X15420734828058
X15420734828058
9-1086-z
7666201111145212
9-00032-7
10.1038/srep42319
10.1038/srep42319
26
26
26
26
26
4.01.037
2014.10.004

0.118147
2021.04.001
2020.08613
210.1111/jcmm.14462
0.118255
rials.2018.04.027
2019.108800
13.05.001
14.09.010
15.04.015
er.2015.07.001
onc.2015.07.002
7-3457-4
4546
735

735
735
3-0433-7
025
.403
.3149990183
1-0134-1
546
3084

MCT-08-104610.1158/1535-7163.MCT-08-1046
1-0117-410.1007/s12032-011-0117-4
10.1002/jcb.24398
MCT-12-046310.1158/1535-7163.MCT-12-0463
4810.1038/onc.2015.48
4810.1038/onc.2015.48
4810.1038/onc.2015.48
7-14088-310.1038/s41598-017-14088-3
7-14088-310.1038/s41598-017-14088-3
120610.3390/genes12081206
era.2016.01.011
era.2016.01.011
193
et.655410.18632/oncotarget.6554
0.05.032
4728
472810.3892/ijo.2019.4728
10.1111/cpr.12875
10.1111/cpr.12875
020.188391
0-2368-510.1038/s41419-020-2368-5
6-3814-3
6-3814-3
09.02.002
09.12.002
2009.04.003
3.02.017
2013.03.002

-74039-3_5
:328341ee38

2015.01.010
)4666
)5033
)0859

29504
.0.1002/ijc.24684
)6.2010.01568.x10.1111/j.1349-7006.2010.01568.x
)030810.1002/cbdv.200900308
:30710.1093/annonc/mdt307
2015.01.01010.1016/j.bulcan.2015.01.010
ıes902020710.3390/biomedicines9020207
ı669
›iomed.2012.05.041
)0000000908
)0000000908
2021.05.013
)12.04.00410.1016/j.canep.2012.04.004
ɜ-0815-710.1186/s12957-016-0815-7
.8.08.142
).11.031
2018.01.041
2018.07.131
.78
.78
.78

.80125N55
.80125N55
.80125N55
.80125N55

)-01318-6
3897
'71
'71
›ne.011458610.1371/journal.pone.0114586

one.011458610.1371/journal.pone.0114586
689710.3892/mmr.2017.6897
010.1002/jcla.23369
.78
2021.11.004
2021.11.004
63.3.229
2021.11.004
2-03456-x10.1186/s12967-022-03456-x
02006_21645
70433910.3389/fonc.2021.704339
2018.06.007
5.07.003
.8-0141
3
0-09869-x
2018.06.00710.1016/j.biocel.2018.06.007
2021.104837

3252
3252
0
0

005210.1139/bcb-2013-0052
325210.3892/mmr.2015.3252

1609753
2020.173094
2016.08.015
2017.10.163
2018.11.003
2021.110016
onc.2016.05.010
er.2019.05.020
2018.04.002
.9.09.031
020.04.002
.0.11.005
2014.04.024

0365

0573
35
4613
.1238
.1238
.1238

)-00567-2
503
-03854-x
)666210108103134
-13-21
3-0653-1
3
-13-8
1-2547-z
)0000000235
331
331
nber.29.12
0.1002/path.3007
2012.09.00710.1016/j.arcmed.2012.09.007
310.4161/auto.27418
0.1111/cas.12656
0.1111/cas.12656
33110.3892/or.2015.4331
33110.3892/or.2015.4331
0.1159/000491665
0.1159/000491665
0.1159/000491665
0.1159/000494004
)-00461-z10.1007/s13402-019-00461-z
210.1093/jb/mvaa012
20610.3390/genes12081206
3
)0000000235
25

-09973-3
res.2021.101030
)2054
)2054
)2054
)2054
)2054
)2054
)2054
2.15400410.1016/j.prp.2022.154004
2666211008140811
)-01551-1
.119504
)11.11.010
)11.11.010
one.0160248
one.0160248
20610.3390/genes12081206
2013.10.005
7695920
55
3.01.013

.4.12.093
.4.12.093
7-0477-5
7-0477-5
7-0477-5
281
.8.02.020

.8.02.02010.1016/j.cyto.2018.02.020
2018.11.00110.1016/j.leukres.2018.11.001
00000001014
51
51
5110.1038/onc.2013.61
766
2-02838-x
5.03.023
rials.2016.07.040
2018.05.091
2018.05.091
2018.05.09110.1016/j.biopha.2018.05.091
00000001826
300

372
5.07.044
0-01489-010.1186/s12935-020-01489-0

2021.174006
08200
2020.05.003
2013.08.007

2301
04342
3910.2147/OTT.S191239
234310.3892/ol.2020.12343
.51
0.03.006
00000000116
00000000116
00000000116
9517510.4103/0973-1482.95175
00000000116
00000000116
00000000116
1-01638-z
3-0997-7
04

-01768-8
627
627
627
627
-0043-5
62710.3892/ijo.2014.2627
-0997-7
4
8.01.109
018.07.024
018.11.074
018.11.074
018.11.074
-2463-3
-2463-3
-2463-3
-2463-3
-2463-3
-2463-3
-2463-3
-2463-3

-8507-9
-03866-7
1912_19794
-2180-310.1007/s00280-013-2180-3

0.1038/srep17618
0.1038/srep17618
0.1038/srep17618
-2463-310.1007/s11060-017-2463-3
018.11.07410.1016/j.biopha.2018.11.074
12
94
017.08.011
21.188552
020.110037
21.100385
21.100385
012.06.002
2017.2258
70116N37
676

97
97
97
12-17
18
267
9710.3892/or.2013.2897
9710.3892/or.2013.2897

et.837610.18632/oncotarget.8376
742
2008.09.017
2008.09.01710.1016/j.canlet.2008.09.017
2017.08.014
2017.08.014
013.10.014
013.10.014
3805865
0-10257-8
0-09553-1
25
4-0892-910.1007/s12032-014-0892-9

2020.17348310.1016/j.ejphar.2020.173483
798
4-0892-910.1007/s12032-014-0892-9
1-04249-4
2014.07.011
7-2389-8

41.1999.01205.x
CAN-14-294610.1158/0008-5472.CAN-14-2946

376113
0-02180-4
1220010.1002/1878-0261.12200
10.1111/cpr.12473
200417N414
18.09.140
0.01.022
1759
16410610.3390/cancers13164106
1759
21.08.012
21.08.012
16.03.160
2019.04.033
017.07.002
57.6.1312
171
16.03.16010.1016/j.bbrc.2016.03.160

c.2018.12.075
7-3435-1
0-03113-5
0-03113-5
0-03113-5
0-03113-5
c.2018.12.07510.1016/j.ijbiomac.2018.12.075
c.2018.12.07510.1016/j.ijbiomac.2018.12.075
one.0056197
054
21.110662

.2.08.029
7-2425-9
7-2425-9
7-2425-9
7-2425-9
366
366
3979
7-2425-910.1007/s11060-017-2425-9
7-2425-910.1007/s11060-017-2425-9
7-2425-910.1007/s11060-017-2425-9
36610.3892/or.2017.5866

0202.2012
017.09.023
2017.08.084
555
27
0-09553-1
7-0033-810.1038/s41408-017-0033-8
1-01854-5
1-01854-5
019
5.10.002
694469310.1109/EMBC.2014.6944693
20.05.011
0-1498-7_12
114685034103437

-13-35
-13-3510.1186/1476-4598-13-35
-22
et.1544110.18632/oncotarget.15441
2017.03.003
5-4559-8

01

one.0133643
one.0133643
one.0133643
one.0133643
one.0133643
one.005343610.1371/journal.pone.0053436
0.1172/JCI66553
01
0.01.009
2013.01.013
3-0628-4

.6

555
-01332-8
-01332-8

0.1159/000467896
9.11.00910.1016/j.apsb.2019.11.009
.6
-0058
)0000000162
)610.1093/abbs/gmt006
'
2018.08.004
)19.03.016
2018.08.00410.1016/j.canlet.2018.08.004

)19.03.016
5777
5777
.6.11.011
2.03.009
)334.2010
)334.2010
)334.201010.1152/ajpcell.00334.2010
.3.08.024
'2210.3892/ol.2017.6722

3
.813
.813
.813
.813
.813
.81310.3892/ijo.2013.1813
2018.01.132
5.07.001
.7
)25
)
.50
)6.2010.01664.x10.1111/j.1349-7006.2010.01664.x
)25
005778
2020.102971
005778
2015.1040963
5-3966-1
2015.104096310.1080/15384047.2015.1040963
).09.018
2017.09.020
5-5199-3
.1

ɪ1
ɪ1
2020.08770
)86
t.2418
ɪ110.1042/BSR20180441
ɪ110.1042/BSR20180441
1239310.1002/1878-0261.12393
766620102916105510.2174/0929867327666201029161055
76956310.3389/fonc.2021.769563
)6.21.07709-010.23736/S0026-4806.21.07709-0
-9702-0
2013.02.01410.1016/j.celrep.2013.02.014
)17.08.028
s.11349
s.11349
s.11349
963
)2006_21513
-1759-9

s.11349
s.11349
s.11349
.12.009
)17.02.004
8.06.005
2019.105643
4.09.010
)-05228-1
614
-00479-5
-00479-5
-00479-5
-00479-5
-00479-5
13-61
2153
)-0700-6

ne.0094855
)-0700-610.1007/s11010-010-0700-6
).4161/cc.24477
8810.3892/ol.2016.5388

2021.1924948
2019.225904
2018.209981

3.14.11.6321
78
3137
3137
3137
3137
3137
3137
3137

4759
0.118255
18.04.005
2019.105643
20.05.011
5-4957-6
4751
0-0943-6
0920411
0666200226124336
0666200226124336
10.1002/jcb.28079
07610.2147/CMAR.S210076
07610.2147/CMAR.S210076
4751
0-0943-6
1-06872-2
00000001826
2017.06.017
2019.102322
833

009.10.05210.1016/j.ygyno.2009.10.052
5-0282-y10.1186/s13046-015-0282-y
5-0282-y10.1186/s13046-015-0282-y
5-0282-y10.1186/s13046-015-0282-y
2019.102322

2-00728-y
3666210521213352
087
087
53
53
53
53

1-01768-8
5310.1038/ncomms14053
7711323
3
2018.10.148
2018.10.148
2019.109551
9-0168-7
20198341
2018.10.14810.1016/j.biopha.2018.10.148
120610.3390/genes12081206
1-01606-710.1007/s12032-021-01606-710.1016/j.gene.2019.14430110.2174/0929867328666
1-01606-710.1007/s12032-021-01606-710.1016/j.gene.2019.14430110.2174/0929867328666
1-01606-7
1-01606-7
1-01606-7
1-01606-7
1-01606-7
2-01012-1
35200

743

5-4480-110.1007/s13277-015-4480-1
00000001348
5337
5337
5337
3-0768-110.1007/s13277-013-0768-1
2-00520-8
2-03446-z
2-03446-z
2-03446-z

4229
0666181213110258
10.1159/000481610
one.021569610.1371/journal.pone.0215696
422910.3892/ijmm.2019.4229

2019.05.010
13.07.029
2014.08.012
2014.08.012
2014.08.012
2014.08.012
2014.08.012
3-1579-3
2011.11.020
39
210.1002/jcla.23272
2019.09.008
2020.109871
19.100673

)70
)70
)70
3-1093-9
3-1093-910.1038/s41419-018-1093-9
CCR-18-165610.1158/1078-0432.CCR-18-1656
342910.18632/aging.103429
21.07.04010.1016/j.bbrc.2021.07.040
one.0068291
)6.2012.02220.x10.1111/j.1349-7006.2012.02220.x
2019.07.070
5-4559-8
50589110.15252/emmm.201505891

.10.1038/ncomms6691
56410.3892/ol.2017.7664
.7.03.162
2020.04.020
2018.05.048
)-1415-6
)-01670-3
.7.03.16210.1016/j.bbrc.2017.03.162
2-00736-4
2011.08.014
2012.08.024
.2.10.019
.0.1002/ijc.25445
385
3-0618-010.1186/s13045-018-0618-0
.-02153-x
)92
)92
360
5046
2021.05.004
)17.02.004
20.144518
2016.04.004
..11.357
..11.357
3612
566
7705762
5.461
215061902533715
2018.1560203
.8-0141
)1906_18183
1902
56
5610.1155/2014/794756
et.2157

215061902533715

2018.1560203
01906_18183
1902
er.2019.05.012
2020.110799
5149
192
2020.173660
.7.03.021
.7.03.021
.7.03.021
.8.03.013
.8.03.013
2021.04.001

788
.8.03.01310.1016/j.gene.2018.03.013
.8.03.01310.1016/j.gene.2018.03.013
2530
.109288
2020.08.012
.0.04.002
7(01)00098-3
ence.2012.11.018
ence.2012.11.018
s.2013.03.022
2.320

-01170C
-01170C
5046
742
-03866-y
038110.3892/ol.2019.10381
2013.06.242
596
596
596
596
59610.1155/2020/3181596
2012.11.006
2012.06.049
5-2123-6
5-2123-6
5-2123-6
5-2123-6
2022.2078614
er.2022.06.010
0-1086-z
5-2205-5
5-2205-5
5-2205-5
5-2205-510.1007/s00432-016-2205-5

02.14510.21037/atm.2020.02.145
4.09.010
366
4731
4731
4731
4731
4731
4731
360
.3149990183
36010.3892/or.2011.1360
5666201019102207
2-01002-3
2
2017.05.008
2-02569-x
.101266610.1177/11795549211012666
20.101451
20.08.004
2020.100841
956
3288
3288
24
2020.100841
et.1186110.18632/oncotarget.11861
7-0033-810.1038/s41408-017-0033-8
24
21.04.009
21.04.009
21.04.009
21.04.009
59200
08.08.047
08.08.047
08.08.047
08.08.047
08.08.005
08.08.005
2014.07.005

0-0953-0
-11-20
one.0026390
2013.204
5-4044-4
5-4044-4

5-4044-410.1007/s13277-015-4044-4
5-4044-410.1007/s13277-015-4044-4

21.04.00910.1016/j.bbrc.2021.04.009
21.04.00910.1016/j.bbrc.2021.04.009

537

049
2513
5-4017-7
694469310.1109/EMBC.2014.6944693
2018.10.061
7.117
510.1002/cbin.10995

.43
et.673910.18632/oncotarget.6739
019.09.051
019.09.051
2-00736-4
2-00736-4
2-00736-4
2-00736-4
2-00736-4
2-00736-4
2-00736-4
07.05.028

1-0981-y
10.1002/jcp.27079
5610.2147/CMAR.S203656
.119499
015.11.034
2017.08.084
015.11.03410.1016/j.ygyno.2015.11.034
4.01.014
02200
32200
32200
32200
32200
2016.10.003
2020.11.016
020.188405
2017.07.056
2010.12.027
2012.03.006
005.12.033
2003.10.028
2003.10.028
2013.07.002
2015.02.011

'-0134-z
3819606
533
.-00519-x
.-00519-x
.-00519-x
.-00519-x
.-00519-x
-9-305
-9-305
:3181de3068
one.0128886
)3918
)2926

2011.06.00810.1016/j.arcmed.2011.06.008
2017.06.08910.1016/j.biopha.2017.06.089
)-1119-x10.1186/s13046-019-1119-x
.-00519-x10.1007/s13577-021-00519-x10.3322/caac.2133810.1007/s11748-013-0246-010.1
2012.03.006
3819606
5-2253-7

)944
)21.188552
)21.188552
5-5052-8
58
58
58
58
58
58
5810.12659/MSM.909458
.7.02.120
).118874
.7.01.008
20.04.003
20.04.003
20.04.003
.4.09.010
'-9788-3
'-9788-3
'-9788-3
)1901_16773

)2002_20195
108410.1093/neuonc/nou084
108410.1093/neuonc/nou084
.7.02.12010.1016/j.bbrc.2017.02.120
.7.02.12010.1016/j.bbrc.2017.02.120
.7.02.12010.1016/j.bbrc.2017.02.120
'-9788-310.1007/s12015-017-9788-3
'-9788-3
'-9788-310.1007/s12015-017-9788-3
)1901_16773

.7.02.12010.1016/j.bbrc.2017.02.120
.-01091-6
!74210.3892/ol.2021.12742
!021.173923
;666170601100633
;-0596-4
.0.1111/jop.12711
;75

!022.08.014

ltholToxicolOncol.2021039471
.-04140-6
.-04140-6
.-04140-6
-005270
!009.10.047
;-1082-z
;-1082-z
!019.109462
.9.07.012
!019.109462
.9.07.012
.9.07.012
.9.07.012
)-01390-w
.10.1111/jcmm.14171
1252310.1002/1878-0261.12523
1252310.1002/1878-0261.12523
)
)339
)-03154-w
.1.00176
.1.00176
.1.00176
.86

!77
!-00535-9
!-00535-9
)17.05.018
.2.12.002

399
029
029
029
596
789
789
789
479
479
479
479
479
479
479
479
479
479
479
78910.1038/bjc.2013.789
47910.3892/or.2015.4479
59610.1155/2020/3181596
2021.201033110.1080/16078454.2021.2010331
2021.2010331
2021.2010331
2021.2010331
2021.2010331
0.118725
bio.2021.04.001
0-00054-1
0-00054-1
0-01523-1
2-1988-6
2020.109858

5.461
7734114
1196

0.1111/cas.14356
666211213122619
7.05.030
0666220124120208

426
56010.3390/ijms15033560
39200
39200

-11-135
one.0029043
one.0029043
)519010.1038/sj.bjc.6605190
one.002904310.1371/journal.pone.0029043
.1.04.021
.1.04.021
.1.04.021
.1.04.021
.1.04.02110.1016/j.ejca.2011.04.021
)(00)00338-5
chem.2019.02.013
4333
7-0586-y
)21.04.028
)21.04.028
20.08.004
.9.07.004
.9.07.004
.9.07.004
7460
7460

2018.1502574
et.1925610.18632/oncotarget.19256
2018.03.02810.1016/j.biopha.2018.03.028
75510.2147/PGPM.S302755
75510.2147/PGPM.S302755
.20610.3390/genes12081206
2021.04.008
.0.1002/jcb.28558
.0.1002/jcb.28558
20.10.031
22.01.003
20.10.03110.1016/j.omtn.2020.10.031
2-02538-4
)1806-15269
)1811-16381
)1905_17797

et.3885
.8.04.005
.8.12.002
2014.08.007
2015.1070990
2018.03.028
2018.03.028
)-00258-510.1038/s41417-020-00258-510.1038/nrclinonc.2013.19710.1016/j.beha.2018.0
)-00258-5
)-1782-910.1007/s00404-010-1782-9
.4.03.135
2016.10.035

t.754510.18632/oncotarget.7545
8.04.129
8.04.129
8.04.129
8.04.129
019.09.001
1.04.093
8.04.12910.1016/j.bbrc.2018.04.129
8.04.12910.1016/j.bbrc.2018.04.129
8.04.12910.1016/j.bbrc.2018.04.129
-07288-2
9.103337
3.07.029
52
-0405-9
0.1002/hed.21648
52

-06800-4
21.188569
018.01.055

142.2018
-0921-z
10.1242/bio.015008
910.2147/OTT.S128819

0.1002/cam4.3248
73
59R
59R
59R
59R
2007_22260
021.1949132
021.1949132
-0149-x
-0149-x
4203

98
16.06.001
1.12.009
088110.3390/cancers7040881
4810.1155/2019/6131548
4810.1155/2019/6131548
161
161
24
24
0.7150/jca.52115
-00994-x
37
37

3-8507-9
307
55.5.119610.3349/ymj.2014.55.5.1196
55.5.119610.3349/ymj.2014.55.5.1196
55.5.119610.3349/ymj.2014.55.5.1196
4-0139-610.1007/s12013-014-0139-6
4-0139-610.1007/s12013-014-0139-6
4-0139-610.1007/s12013-014-0139-6
30710.3892/or.2015.3807
30710.3892/or.2015.3807
s.2015.03.02310.1016/j.brainres.2015.03.023
10.1111/cas.13538
38910.2147/CMAR.S174889

569889
er.2015.10.003
510.1093/nar/gku876
.2019.04.002
2013.08.019
2013.08.019
2016.05.030
0310
092
43
.83
.83
.83
.83
.83
.83
.8310.1038/onc.2012.183
.8310.1038/onc.2012.183
21.100797
3
surg.2016.06.303
7-1375-1
one.0100127

.1.00176
271205
43.2010.01710.x10.1111/j.1440-1843.2010.01710.x
.84210.3892/mmr.2013.1842
2014.140607
.2020.04.041
2020.110768
.2.11.002
5.194
5.194
5.194
5.194
5.194
5.194
5.194
025

[illegible].15.2.577

[illegible]one.0156871
[illegible]012.10.00810.1016/j.canlet.2012.10.008
[illegible]one.007762310.1371/journal.pone.0077623
[illegible]one.015687110.1371/journal.pone.0156871
[illegible].19410.1038/cddis.2016.194
[illegible]1545218788883910.3727/096504018X15452187888839

[illegible]25
[illegible]-09979-x
[illegible]0000001826
[illegible]2
[illegible]16.03.036
[illegible]-04249-4

[illegible]51
[illegible]51
[illegible]51
[illegible]46
[illegible]13-21
[illegible]5010
[illegible]19.01.06610.1016/j.ebiom.2019.01.066
[illegible]-4587-4
[illegible]-4587-4

[illegible]010.01.01010.1016/j.biopha.2010.01.010
[illegible]010.01.01010.1016/j.biopha.2010.01.010

1038/nrc393210.1016/j.semcancer.2021.03.01410.1038/nrd.2016.24610.1016/S1470-2045

85-610.3390/cells809095710.3390/biom912078910.1517/14740338.6.5.60910.18632/oncot
85-610.3390/cells809095710.3390/biom912078910.1517/14740338.6.5.60910.18632/oncot

). 1056/NEJMoa111208810. 1371/journal. pone. 010600710. 1016/j. canlet. 2004. 01. 03810. 101

7043010.1164/rccm.201408-1505OC10.18632/oncotarget.382710.3892/ijo_0000046510.101

). 1007/s10238-014-0288-310. 1186/s12943-019-1019-x10. 1007/s13402-018-0388-210. 1186/

5710(98)00038-310.3109/07388551.2015.101595710.1038/nature0592510.1080/15548627.20

[illegible]21051412595510.3390/ijms2205265210.1002/jcp.2767010.1007/s11033-021-06193-410.103
[illegible]21051412595510.3390/ijms2205265210.1002/jcp.2767010.1007/s11033-021-06193-410.103

.016/j.athoracsur.2018.02.01810.21037/cco.2017.07.0210.1016/j.oralоncology.2008.06

)7.01410.3747/co.26.542110.1182/blood-2014-05-57718910.1083/jcb.152.6.124710.1016/

(11)70067-510.1038/s41419-019-1382-y10.1002/2211-5463.1224810.3389/fphar.2

:arget.1672310.1002/jcp.2882310.3390/ijms2019494010.1093/femspd/ftz03910.10
:arget.1672310.1002/jcp.2882310.3390/ijms2019494010.1093/femspd/ftz03910.10

6/bs.acr.2017.11.00110.1038/onc.2011.38410.1016/j.molcel.2018.06.03410.109

6/bs.acr.2014.10.00310.1016/0014-5793(93)81604-X10.1038/nature0145110.1007

's12943-018-0863-410.1007/s13402-018-0378-410.1186/s12943-017-0718-410.1002

)16.116435910.21037/atm.2019.02.2210.1371/journal.pone.009015110.1038/onc.2

38/sigtrans.2015.410.1016/j.biopha.2018.10.14810.3389/fcell.2020.0054010.32
38/sigtrans.2015.410.1016/j.biopha.2018.10.14810.3389/fcell.2020.0054010.32

[illegible]. 00210. 1016/j. tox. 2006. 07. 01110. 1016/j. toxlet. 2013. 10. 00410. 1289/ehp. 09010

'S0378-1119(02)00928-910.1074/jbc.M50391520010.1111/j.1742-4658.2006.05216.
