# Supplementary material for: Role of PPARG in Chemosensitivity-Regulating Network for Hypopharyngeal Squamous Cell Carcinoma
Source: PPAR Res. 2023 Sep 25;2023:6019318. doi: 10.1155/2023/6019318 (PMC10545467; doi:10.1155/2023/6019318)
Supplement: Supplementary 2 — Supplementary Table 2: the reference information supporting the 593 chemosensitivity inhibitors. [file 6019318.f2.pdf]

Ref info supporting the chemosensitivity inhibitors

Relation Name PMID DOI
negative Regulation: BIRC2 ---| chemosensitivity 29567488 10.1016/j.biocel.2018.03.01
negative Regulation: BIRC2 ---| chemosensitivity 26168135 10.3727/096504015X142981229
negative Regulation: BIRC2 ---| chemosensitivity 26168135 10.3727/096504015X142981229
negative Regulation: BIRC2 ---| chemosensitivity 26168135 10.3727/096504015X142981229
negative Regulation: BIRC2 ---| chemosensitivity 26168135 10.3727/096504015X142981229
negative Regulation: BIRC2 ---| chemosensitivity 26168135 10.3727/096504015X142981229
negative Regulation: LINC00461 ---| chemosensitivit33869744 10.1016/j.omto.2021.01.0081
negative Regulation: KRT81 ---| chemosensitivity 34591651 10.1089/dna.2021.0317
negative Regulation: KRT81 ---| chemosensitivity 34591651 10.1089/dna.2021.0317
negative Regulation: MIR182 ---| chemosensitivity 31540771 10.1016/j.prp.2019.152603
negative Regulation: MIR182 ---| chemosensitivity 31128298 10.1016/j.semcancer.2019.05
negative Regulation: MIR182 ---| chemosensitivity 26216350 10.1016/j.humpath.2015.06.0
negative Regulation: MIR182 ---| chemosensitivity 29925897 10.1038/s41417-018-0031-4
negative Regulation: MIR182 ---| chemosensitivity 31115013 10.26355/eurrev_201905_1781
negative Regulation: MIR182 ---| chemosensitivity 33437365
negative Regulation: MIR182 ---| chemosensitivity 25012722 10.1186/1746-1596-9-14310.1
negative Regulation: MIR182 ---| chemosensitivity 31697978 10.1016/j.canlet.2019.10.04
negative Regulation: STIM1 ---| chemosensitivity 28326487 10.1007/s13577-017-0167-9
negative Regulation: GAA ---| chemosensitivity 33931930 10.1111/cas.1492110.1111/ca
negative Regulation: BUB3 ---| chemosensitivity 33872216 10.18632/aging.202944
negative Regulation: BUB3 ---| chemosensitivity 30866167 10.1111/odi.1308910.1111/od
negative Regulation: TRIM37 ---| chemosensitivity 29163677 10.3892/ol.2017.705910.3892
negative Regulation: STAT3 ---| chemosensitivity 22205702 10.1074/jbc.M111.295964
negative Regulation: STAT3 ---| chemosensitivity 30145375 10.1016/j.ejmech.2018.08.03
negative Regulation: STAT3 ---| chemosensitivity 24076095 10.1016/j.mce.2013.08.019
negative Regulation: STAT3 ---| chemosensitivity 32160976 10.1016/j.canlet.2020.03.00
negative Regulation: STAT3 ---| chemosensitivity 28011380 10.1016/j.biopha.2016.12.03
negative Regulation: STAT3 ---| chemosensitivity 33378975 10.1016/j.biopha.2020.11107
negative Regulation: STAT3 ---| chemosensitivity 30668440 10.1016/j.phymed.2018.06.03
negative Regulation: STAT3 ---| chemosensitivity 29175114 10.1016/j.phrs.2017.11.024
negative Regulation: STAT3 ---| chemosensitivity 20026083 10.1016/j.bcp.2009.12.014
negative Regulation: STAT3 ---| chemosensitivity 21549414 10.1016/j.acthis.2011.04.00
negative Regulation: STAT3 ---| chemosensitivity 21549414 10.1016/j.acthis.2011.04.00
negative Regulation: STAT3 ---| chemosensitivity 23340171 10.1016/j.canlet.2013.01.00
negative Regulation: STAT3 ---| chemosensitivity 23246576 10.1016/j.bbagen.2012.12.00
negative Regulation: STAT3 ---| chemosensitivity 24735751 10.1016/j.canlet.2014.04.00
negative Regulation: STAT3 ---| chemosensitivity 25892518 10.1016/j.bbrc.2015.04.040
negative Regulation: STAT3 ---| chemosensitivity 27130669 10.1016/j.canlet.2016.04.03
negative Regulation: STAT3 ---| chemosensitivity 17531096
negative Regulation: STAT3 ---| chemosensitivity 27725789 10.1155/2016/5894347
negative Regulation: STAT3 ---| chemosensitivity 29293118 10.1097/CAD.0000000000000058
negative Regulation: STAT3 ---| chemosensitivity 30252677 10.1172/JCI120156
negative Regulation: STAT3 ---| chemosensitivity 22961117 10.1055/s-0032-1315303
negative Regulation: STAT3 ---| chemosensitivity 21887474 10.3892/or.2011.1396
negative Regulation: STAT3 ---| chemosensitivity 22205702 10.1074/jbc.M111.295964
negative Regulation: STAT3 ---| chemosensitivity 22205702 10.1074/jbc.M111.295964
negative Regulation: STAT3 ---| chemosensitivity 24220695 10.1038/bjc.2013.673
negative Regulation: STAT3 ---| chemosensitivity
negative Regulation: STAT3 ---| chemosensitivity 25867391 10.4238/2015.March.30.3
negative Regulation: STAT3 ---| chemosensitivity 18059167 10.4161/cbt.6.12.4970

negative Regulation: STAT3 ---| chemosensitivity	20106947	10.1093/toxsci/kfq02810.109
negative Regulation: STAT3 ---| chemosensitivity	21685938	10.1038/onc.2011.22210.1038
negative Regulation: STAT3 ---| chemosensitivity	22205702	10.1074/jbc.M111.29596410.1
negative Regulation: STAT3 ---| chemosensitivity	25867391	10.4238/2015.March.30.310.4
negative Regulation: STAT3 ---| chemosensitivity	25908586	10.1158/0008-5472.CAN-14-29
negative Regulation: STAT3 ---| chemosensitivity	26121837	
negative Regulation: STAT3 ---| chemosensitivity	27373041	
negative Regulation: STAT3 ---| chemosensitivity	27797972	10.1158/1078-0432.CCR-16-13
negative Regulation: STAT3 ---| chemosensitivity	34571081	10.1016/j.canlet.2021.09.03
negative Regulation: STAT3 ---| chemosensitivity		10.1097/CAD.000000000000058
negative Regulation: STAT3 ---| chemosensitivity		10.1172/JCI120156
negative Regulation: STAT3 ---| chemosensitivity		10.3390/ijms19123890
negative Regulation: STAT3 ---| chemosensitivity	34397325	10.2174/138620732466621081б
negative Regulation: STAT3 ---| chemosensitivity	35353612	10.1089/dna.2021.0936
negative Regulation: MACC1 ---| chemosensitivity	26936749	10.12659/MSM.897055
negative Regulation: MACC1 ---| chemosensitivity	26936749	10.12659/MSM.897055
negative Regulation: MACC1 ---| chemosensitivity	28339092	10.3892/or.2017.5519
negative Regulation: MACC1 ---| chemosensitivity	28339092	10.3892/or.2017.5519
negative Regulation: MACC1 ---| chemosensitivity	28339092	10.3892/or.2017.5519
negative Regulation: MACC1 ---| chemosensitivity	26794135	10.3892/or.2016.4585
negative Regulation: MACC1 ---| chemosensitivity	26794135	10.3892/or.2016.458510.3892
negative Regulation: MACC1 ---| chemosensitivity	28339092	10.3892/or.2017.551910.3892
negative Regulation: KRT7 ---| chemosensitivity		
negative Regulation: KRT7 ---| chemosensitivity		
negative Regulation: KRT7 ---| chemosensitivity		
negative Regulation: KRT7 ---| chemosensitivity		
negative Regulation: KRT7 ---| chemosensitivity		
negative Regulation: KRT7 ---| chemosensitivity	31317696	
negative Regulation: SEC62 ---| chemosensitivity	33858476	10.1186/s13046-021-01934-6
negative Regulation: SEC62 ---| chemosensitivity	33858476	10.1186/s13046-021-01934-6
negative Regulation: SEC62 ---| chemosensitivity	33858476	10.1186/s13046-021-01934-6
negative Regulation: CLU ---| chemosensitivity	28784314	10.1016/j.cbi.2017.08.002
negative Regulation: CLU ---| chemosensitivity	11502446	10.1016/S0090-4295(01)01241
negative Regulation: CLU ---| chemosensitivity	12350509	10.1016/S0090-4295(02)01806
negative Regulation: CLU ---| chemosensitivity	19903745	
negative Regulation: CLU ---| chemosensitivity	15689620	
negative Regulation: CLU ---| chemosensitivity	25778071	10.1186/s12957-015-0501-1
negative Regulation: CLU ---| chemosensitivity	28471806	10.1097/CAD.000000000000050
negative Regulation: CLU ---| chemosensitivity	31433751	10.2174/138920102066619082l
negative Regulation: CLU ---| chemosensitivity	32459606	10.2174/138955752066620052б
negative Regulation: CLU ---| chemosensitivity	32459606	10.2174/138955752066620052б
negative Regulation: CLU ---| chemosensitivity	32459606	10.2174/138955752066620052б
negative Regulation: CLU ---| chemosensitivity	34212987	10.3892/mmr.2021.12267
negative Regulation: CLU ---| chemosensitivity	22895628	10.3892/mmr.2012.1017
negative Regulation: CLU ---| chemosensitivity	21609464	10.1186/1477-7819-9-59
negative Regulation: CLU ---| chemosensitivity	25106434	10.1186/1477-7819-12-255
negative Regulation: CLU ---| chemosensitivity	25343293	10.1080/15287394.2014.95176
negative Regulation: CLU ---| chemosensitivity	11571636	10.1038/sj.neo.7900174
negative Regulation: CLU ---| chemosensitivity	16373699	10.1158/1535-7163.MCT-05-01
negative Regulation: CLU ---| chemosensitivity	21630085	10.1007/s00280-011-1682-01C
negative Regulation: CLU ---| chemosensitivity	22895628	10.3892/mmr.2012.101710.389
negative Regulation: CLU ---| chemosensitivity	22895628	10.3892/mmr.2012.101710.389
negative Regulation: CLU ---| chemosensitivity	22967941	10.1186/1756-9966-31-7310.1
negative Regulation: CLU ---| chemosensitivity	25106434	10.1186/1477-7819-12-25510.

negative Regulation: CLU ---| chemosensitivity	25884382	10.1186/s12957-015-0501-110
negative Regulation: CLU ---| chemosensitivity	26988917	10.18632/oncotarget.810510.
negative Regulation: CLU ---| chemosensitivity	34212987	10.3892/mmr.2021.1226710.38
negative Regulation: CLU ---| chemosensitivity		10.1097/CAD.00000000000050
negative Regulation: CLU ---| chemosensitivity	25106434	10.1186/1477-7819-12-255
negative Regulation: CLU ---| chemosensitivity	25778071	10.1186/s12957-015-0501-1
negative Regulation: CLU ---| chemosensitivity		
negative Regulation: CLU ---| chemosensitivity		10.2174/1389201020666190821
negative Regulation: CLU ---| chemosensitivity		10.33549/physiolres.934908
negative Regulation: H2AZ1 ---| chemosensitivity	35968782	10.1172/JCI158446
negative Regulation: FKBP5 ---| chemosensitivity	21530399	10.1016/j.coph.2011.04.001
negative Regulation: FKBP5 ---| chemosensitivity	23936393	10.1371/journal.pone.007021
negative Regulation: FKBP5 ---| chemosensitivity	24061475	10.1128/MCB.00695-13
negative Regulation: FKBP5 ---| chemosensitivity	32964982	10.26355/eurrev_202009_2283
negative Regulation: FKBP5 ---| chemosensitivity	35354796	10.1038/s41419-022-04727-7
negative Regulation: NOTCH4 ---| chemosensitivity	30497876	10.1016/j.prp.2018.10.013
negative Regulation: NOTCH4 ---| chemosensitivity	1150	10.1016/j.prp.2018.10.013
negative Regulation: KDM5B ---| chemosensitivity	32440821	10.1007/s12031-020-01587-8
negative Regulation: KDM5B ---| chemosensitivity	25951238	10.1371/journal.pone.012534
negative Regulation: TRPA1 ---| chemosensitivity	34015320	10.1016/j.ejphar.2021.17418
negative Regulation: TRPA1 ---| chemosensitivity	31682917	10.1016/j.freeradbiomed.201
negative Regulation: TRPA1 ---| chemosensitivity	29805077	10.1016/j.ccell.2018.05.001
negative Regulation: TRPA1 ---| chemosensitivity		10.1016/j.apsb.2021.11.001
negative Regulation: RPS6KB1 ---| chemosensitivity	25573956	10.1158/1535-7163.MCT-14-06
negative Regulation: CBR3-AS1 ---| chemosensitivity	35466320	10.1155/2022/2260211
negative Regulation: GRM5 ---| chemosensitivity	35396501	10.1038/s41417-022-00465-2
negative Regulation: GRM5 ---| chemosensitivity	35396501	10.1038/s41417-022-00465-2
negative Regulation: GRM5 ---| chemosensitivity	35396501	10.1038/s41417-022-00465-2
negative Regulation: GRM5 ---| chemosensitivity	35396501	10.1038/s41417-022-00465-2
negative Regulation: GRM5 ---| chemosensitivity	35396501	10.1038/s41417-022-00465-2
negative Regulation: GRM5 ---| chemosensitivity	35396501	10.1038/s41417-022-00465-2
negative Regulation: GRM5 ---| chemosensitivity	35396501	10.1038/s41417-022-00465-2
negative Regulation: GRM5 ---| chemosensitivity	35396501	10.1038/s41417-022-00465-2
negative Regulation: CCND1 ---| chemosensitivity	24480319	10.1016/j.bbcan.2014.01.007
negative Regulation: CCND1 ---| chemosensitivity	27155130	10.1016/j.dnarep.2016.04.01
negative Regulation: CCND1 ---| chemosensitivity	13679454	
negative Regulation: CCND1 ---| chemosensitivity	24100731	10.3892/ijo.2013.2119
negative Regulation: CCND1 ---| chemosensitivity	24303084	10.1371/journal.pone.008247
negative Regulation: CCND1 ---| chemosensitivity	25881299	10.1186/s12885-015-1240-y
negative Regulation: CCND1 ---| chemosensitivity		10.3727/096368915X687787
negative Regulation: CCND1 ---| chemosensitivity	16586549	
negative Regulation: CCND1 ---| chemosensitivity	16586549	
negative Regulation: CCND1 ---| chemosensitivity	19020753	
negative Regulation: CCND1 ---| chemosensitivity	28990111	10.3892/mmr.2017.7732
negative Regulation: EIF5A2 ---| chemosensitivity	27097942	
negative Regulation: EIF5A2 ---| chemosensitivity	28885268	10.1097/CAD.00000000000055
negative Regulation: EIF5A2 ---| chemosensitivity	33174013	10.3892/ijo.2020.5143
negative Regulation: EIF5A2 ---| chemosensitivity	25071013	10.18632/oncotarget.2236
negative Regulation: EIF5A2 ---| chemosensitivity	26317793	10.18632/oncotarget.458110.
negative Regulation: EIF5A2 ---| chemosensitivity	26581310	10.1186/s12935-015-0250-910
negative Regulation: EIF5A2 ---| chemosensitivity	35718769	10.1186/s13048-022-00998-y
negative Regulation: GSTP1 ---| chemosensitivity	31026507	10.1016/j.ejps.2019.04.021
negative Regulation: GSTP1 ---| chemosensitivity	28062686	10.1152/physiolgenomics.000
negative Regulation: GSTP1 ---| chemosensitivity	29328427	10.3892/ijmm.2018.3382

negative Regulation: GSTP1 ---| chemosensitivity 29328427 10.3892/ijmm.2018.3382
negative Regulation: GSTP1 ---| chemosensitivity 31024008 10.1038/s41419-019-1588-z
negative Regulation: GSTP1 ---| chemosensitivity 32713331 10.2174/1381612826666200724
negative Regulation: GSTP1 ---| chemosensitivity 26910073 10.1007/978-1-4939-3347-1_9
negative Regulation: GSTP1 ---| chemosensitivity 11436358
negative Regulation: GSTP1 ---| chemosensitivity 17513610 10.1158/1535-7163.MCT-06-05
negative Regulation: GSTP1 ---| chemosensitivity 24460306 10.7314/apjcp.2013.14.12.73
negative Regulation: GSTP1 ---| chemosensitivity 25010864 10.1038/bjc.2014.38610.1038
negative Regulation: GSTP1 ---| chemosensitivity 25010864 10.1038/bjc.2014.38610.1038
negative Regulation: GSTP1 ---| chemosensitivity 26711214
negative Regulation: FGF9 ---| chemosensitivity 30999114 10.1016/j.biopha.2019.10866
negative Regulation: RPS6KA2 ---| chemosensitivity 23108403 10.1038/onc.2012.472
negative Regulation: UCA1 ---| chemosensitivity 30825424 10.1016/j.clinbiochem.2019.
negative Regulation: UCA1 ---| chemosensitivity 33901464 10.1016/j.ejphar.2021.17411
negative Regulation: UCA1 ---| chemosensitivity 30107990 10.1016/j.prp.2018.07.036
negative Regulation: UCA1 ---| chemosensitivity 29674277 10.1016/j.biopha.2018.03.13
negative Regulation: UCA1 ---| chemosensitivity 32146422 10.1016/j.drup.2020.100683
negative Regulation: UCA1 ---| chemosensitivity 31611036 10.1016/j.oooo.2019.08.011
negative Regulation: UCA1 ---| chemosensitivity 29441929 10.1691/ph.2016.6625
negative Regulation: UCA1 ---| chemosensitivity 29441929 10.1691/ph.2016.6625
negative Regulation: UCA1 ---| chemosensitivity 29441929 10.1691/ph.2016.6625
negative Regulation: UCA1 ---| chemosensitivity 29441929 10.1691/ph.2016.6625
negative Regulation: UCA1 ---| chemosensitivity 29441929 10.1691/ph.2016.6625
negative Regulation: UCA1 ---| chemosensitivity 29441929 10.1691/ph.2016.6625
negative Regulation: UCA1 ---| chemosensitivity 29441929 10.1691/ph.2016.6625
negative Regulation: UCA1 ---| chemosensitivity 29367594 10.1038/s41419-017-0113-5
negative Regulation: UCA1 ---| chemosensitivity 30015983 10.3892/or.2018.6573
negative Regulation: UCA1 ---| chemosensitivity 30868851 10.7754/Clin.Lab.2018.18073
negative Regulation: UCA1 ---| chemosensitivity 31173289 10.26355/eurrev_201905_1792
negative Regulation: UCA1 ---| chemosensitivity 31528122 10.1186/s12935-019-0934-7
negative Regulation: UCA1 ---| chemosensitivity 33936199 10.1155/2021/5519720
negative Regulation: UCA1 ---| chemosensitivity 33936199 10.1155/2021/5519720
negative Regulation: UCA1 ---| chemosensitivity 27591936 10.1016/j.canlet.2016.08.01
negative Regulation: UCA1 ---| chemosensitivity 29441929 10.1691/ph.2016.662510.1691
negative Regulation: UCA1 ---| chemosensitivity 29441929 10.1691/ph.2016.662510.1691
negative Regulation: UCA1 ---| chemosensitivity 29441929 10.1691/ph.2016.662510.1691
negative Regulation: NIPBL ---| chemosensitivity 29670369 10.2147/OTT.S15865510.2147/
negative Regulation: MIR93 ---| chemosensitivity 10.1016/j.febslet.2012.03.0
negative Regulation: MIR93 ---| chemosensitivity 32796817 10.1038/s41419-020-02855-61
negative Regulation: MIR93 ---| chemosensitivity 10.1016/j.febslet.2012.03.0
negative Regulation: MIR93 ---| chemosensitivity 10.1016/j.febslet.2012.03.0
negative Regulation: MIR93 ---| chemosensitivity 10.1016/j.febslet.2012.03.0
negative Regulation: MIR93 ---| chemosensitivity 22465665 10.1016/j.febslet.2012.03.0
negative Regulation: MIR93 ---| chemosensitivity 22465665 10.1016/j.febslet.2012.03.0
negative Regulation: MIR93 ---| chemosensitivity 22465665 10.1016/j.febslet.2012.03.0
negative Regulation: MIR93 ---| chemosensitivity 22465665 10.1016/j.febslet.2012.03.0
negative Regulation: MIR93 ---| chemosensitivity 25064468 10.1016/j.mce.2014.07.014
negative Regulation: MIR93 ---| chemosensitivity 26363097 10.1016/j.febslet.2015.08.0
negative Regulation: MIR93 ---| chemosensitivity 29327155 10.1007/s00404-018-4649-0
negative Regulation: MIR93 ---| chemosensitivity 30213291 10.14715/cmb/2018.64.11.12
negative Regulation: MIR93 ---| chemosensitivity 10.1042/BSR20181743
negative Regulation: MIR93 ---| chemosensitivity 23591839 10.3390/ijms14048213
negative Regulation: MIR93 ---| chemosensitivity 24512727 10.3727/096504013X138324733
negative Regulation: MIR93 ---| chemosensitivity 27099514 10.2147/OTT.S9739910.2147/C

negative Regulation: MIR93 ---| chemosensitivity 27099514 10.2147/OTT.S9739910.2147/C
negative Regulation: MIR93 ---| chemosensitivity 27185265 10.1242/bio.01555210.1242/b
negative Regulation: MIR93 ---| chemosensitivity 32796817 10.1038/s41419-020-02855-61
negative Regulation: MIR93 ---| chemosensitivity 34292880 10.18632/aging.203298
negative Regulation: MIR432 ---| chemosensitivity 31246330 10.1111/bcpt.1328610.1111/b
negative Regulation: LILRB2 ---| chemosensitivity 27070574 10.3390/ijms17040517
negative Regulation: LILRB2 ---| chemosensitivity 28571552 10.2174/1381612823666170601
negative Regulation: LILRB2 ---| chemosensitivity 27002147 10.1074/jbc.M115.700021
negative Regulation: LILRB2 ---| chemosensitivity 26448762 10.1155/2015/98907010.1155/
negative Regulation: LILRB2 ---| chemosensitivity 27002147 10.1074/jbc.M115.70002110.1
negative Regulation: LILRB2 ---| chemosensitivity 10.1074/jbc.M115.700021
negative Regulation: MAGED1 ---| chemosensitivity 29778424 10.1016/j.gene.2018.05.060
negative Regulation: CDC20 ---| chemosensitivity 27633058 10.3892/ijo.2016.3671
negative Regulation: CDC20 ---| chemosensitivity 27633058 10.3892/ijo.2016.3671
negative Regulation: CDC20 ---| chemosensitivity 29901174 10.3892/or.2018.6467
negative Regulation: CDC20 ---| chemosensitivity 31081056 10.3892/ijo.2019.4791
negative Regulation: CDC20 ---| chemosensitivity 27633058 10.3892/ijo.2016.367110.389
negative Regulation: CDC20 ---| chemosensitivity 33118830 10.4149/neo_2020_200614N629
negative Regulation: CDC20 ---| chemosensitivity 33118830 10.4149/neo_2020_200614N629
negative Regulation: CDC20 ---| chemosensitivity 33118830 10.4149/neo_2020_200614N629
negative Regulation: CDC20 ---| chemosensitivity 33118830 10.4149/neo_2020_200614N629
negative Regulation: CDC20 ---| chemosensitivity 33118830 10.4149/neo_2020_200614N629
negative Regulation: MIR574 ---| chemosensitivity 31786621 10.1007/s00210-019-01772-6
negative Regulation: MIR574 ---| chemosensitivity 31786621 10.1007/s00210-019-01772-6
negative Regulation: MIR574 ---| chemosensitivity 31786621 10.1007/s00210-019-01772-6
negative Regulation: MIR574 ---| chemosensitivity 31786621 10.1007/s00210-019-01772-6
negative Regulation: MIR574 ---| chemosensitivity 31786621 10.1007/s00210-019-01772-6
negative Regulation: MIR574 ---| chemosensitivity 31786621 10.1007/s00210-019-01772-6
negative Regulation: MIR574 ---| chemosensitivity 31786621 10.1007/s00210-019-01772-61
negative Regulation: MIR574 ---| chemosensitivity 31786621 10.1007/s00210-019-01772-61
negative Regulation: LOXL2 ---| chemosensitivity 31462706 10.1038/s41388-019-0969-1
negative Regulation: MIR10B ---| chemosensitivity 31267531 10.1002/jcp.2904010.1002/jc
negative Regulation: MIR10B ---| chemosensitivity 35181582 10.21873/cgp.20308
negative Regulation: MIR10B ---| chemosensitivity 36142861 10.3390/ijms231810952
negative Regulation: MAPKAPK2 ---| chemosensitivity29704518 10.1016/j.canlet.2018.04.03
negative Regulation: MAPKAPK2 ---| chemosensitivity20727903 10.1016/j.mrfmmm.2010.07.01
negative Regulation: MAPKAPK2 ---| chemosensitivity
negative Regulation: MAPKAPK2 ---| chemosensitivity29704518 10.1016/j.canlet.2018.04.03
negative Regulation: MAPKAPK2 ---| chemosensitivity
negative Regulation: MUS81 ---| chemosensitivity 28347251 10.1177/1010428317694307
negative Regulation: MUS81 ---| chemosensitivity 28347251 10.1177/1010428317694307
negative Regulation: MUS81 ---| chemosensitivity 28347251 10.1177/1010428317694307
negative Regulation: MUS81 ---| chemosensitivity 28347251 10.1177/1010428317694307
negative Regulation: MUS81 ---| chemosensitivity 29393493 10.3892/or.2018.6229
negative Regulation: MUS81 ---| chemosensitivity 25364260 10.2147/OTT.S6433910.2147/C
negative Regulation: MUS81 ---| chemosensitivity 26714930 10.1002/cam4.58810.1002/cam
negative Regulation: MUS81 ---| chemosensitivity 26714930 10.1002/cam4.58810.1002/cam
negative Regulation: MUS81 ---| chemosensitivity 27284361 10.3892/ol.2016.4489
negative Regulation: MUS81 ---| chemosensitivity 28291626 10.1016/j.clinre.2017.01.01
negative Regulation: ANPEP ---| chemosensitivity 21354167 10.1053/j.gastro.2011.02.03
negative Regulation: ANPEP ---| chemosensitivity
negative Regulation: PCLAF ---| chemosensitivity 27708548 10.1186/s12935-016-0353-y
negative Regulation: MECOM ---| chemosensitivity 26742943 10.1016/j.jconrel.2015.12.0
negative Regulation: MECOM ---| chemosensitivity 29879503 10.1016/j.gene.2018.06.005

negative Regulation: MECOM ---| chemosensitivity	22295105	10.1371/journal.pone.003070
negative Regulation: ETS1 ---| chemosensitivity	27525970	10.1016/j.biopha.2016.08.02
negative Regulation: ETS1 ---| chemosensitivity	29950928	10.1186/s12935-018-0581-4
negative Regulation: ETS1 ---| chemosensitivity	29950928	10.1186/s12935-018-0581-4
negative Regulation: ETS1 ---| chemosensitivity	24602286	10.1186/1475-2867-14-22
negative Regulation: ETS1 ---| chemosensitivity	24602286	10.1186/1475-2867-14-22
negative Regulation: ETS1 ---| chemosensitivity	29950928	10.1186/s12935-018-0581-410
negative Regulation: ETS1 ---| chemosensitivity	28672814	10.3390/ijms18071354
negative Regulation: SPDEF ---| chemosensitivity	34139546	10.1016/j.phymed.2021.15360
negative Regulation: B4GALT1 ---| chemosensitivity	23744354	10.1038/cddis.2013.186
negative Regulation: HOXA5 ---| chemosensitivity	30267809	10.1016/j.gene.2018.09.051
negative Regulation: HOXA5 ---| chemosensitivity	26397212	10.3892/mmr.2015.4331
negative Regulation: HOXA5 ---| chemosensitivity	26397212	10.3892/mmr.2015.433110.389
negative Regulation: HOXA5 ---| chemosensitivity	26397212	10.3892/mmr.2015.433110.389
negative Regulation: GALNT14 ---| chemosensitivity	29227978	10.1159/000485400
negative Regulation: GALNT14 ---| chemosensitivity	29227978	10.1159/00048540010.1159/00
negative Regulation: GALNT14 ---| chemosensitivity	29227978	10.1159/00048540010.1159/00
negative Regulation: GALNT14 ---| chemosensitivity	29227978	10.1159/00048540010.1159/00
negative Regulation: AKT2 ---| chemosensitivity	32522594	10.1016/j.bcp.2020.114085
negative Regulation: AKT2 ---| chemosensitivity	22771706	10.1016/j.brainres.2012.06.
negative Regulation: AKT2 ---| chemosensitivity	22771706	10.1016/j.brainres.2012.06.
negative Regulation: AKT2 ---| chemosensitivity	27163202	10.1016/j.yexmp.2016.05.003
negative Regulation: AKT2 ---| chemosensitivity	26953242	10.2174/1389450117666160307
negative Regulation: AKT2 ---| chemosensitivity	26709804	
negative Regulation: PF4 ---| chemosensitivity	25822018	10.1172/JCI78752
negative Regulation: PF4 ---| chemosensitivity	25822018	10.1172/JCI78752
negative Regulation: PF4 ---| chemosensitivity	9116276	
negative Regulation: PF4 ---| chemosensitivity	9116276	
negative Regulation: USP2 ---| chemosensitivity	30918246	10.1038/s41419-019-1512-6
negative Regulation: USP2 ---| chemosensitivity	35384881	10.1097/PAI.000000000000101
negative Regulation: IL6R ---| chemosensitivity	25625841	10.3892/ijo.2015.2851
negative Regulation: KDM5A ---| chemosensitivity	36566915	10.1016/j.ejphar.2022.17546
negative Regulation: KDM5A ---| chemosensitivity	36566915	10.1016/j.ejphar.2022.17546
negative Regulation: BAP1 ---| chemosensitivity	33872660	10.1016/j.lfs.2021.119504
negative Regulation: MUC5B ---| chemosensitivity	26984395	10.3892/ijo.2016.3434
negative Regulation: MUC5B ---| chemosensitivity	26984395	10.3892/ijo.2016.3434
negative Regulation: CCN2 ---| chemosensitivity	33358571	10.1016/j.trecan.2020.12.00
negative Regulation: CCN2 ---| chemosensitivity	28617438	10.1038/cddis.2017.248
negative Regulation: CCN2 ---| chemosensitivity	28617438	10.1038/cddis.2017.248
negative Regulation: CCN2 ---| chemosensitivity	28617438	10.1038/cddis.2017.248
negative Regulation: CCN2 ---| chemosensitivity	28617438	10.1038/cddis.2017.248
negative Regulation: CCN2 ---| chemosensitivity	28617438	10.1038/cddis.2017.248
negative Regulation: CCN2 ---| chemosensitivity	28617438	10.1038/cddis.2017.248
negative Regulation: CCN2 ---| chemosensitivity	28617438	10.1038/cddis.2017.24810.10
negative Regulation: CCN2 ---| chemosensitivity		10.1016/j.trecan.2020.12.00
negative Regulation: NOTCH2 ---| chemosensitivity	30497876	10.1016/j.prp.2018.10.013
negative Regulation: CDKN2B-AS1 ---| chemosensitivi	30777616	10.1016/j.prp.2019.01.042
negative Regulation: CDKN2B-AS1 ---| chemosensitivi	30777616	10.1016/j.prp.2019.01.042
negative Regulation: CDKN2B-AS1 ---| chemosensitivi	31189742	10.1042/BSR20182101
negative Regulation: CDKN2B-AS1 ---| chemosensitivi	30777616	10.1016/j.prp.2019.01.04210
negative Regulation: SNHG1 ---| chemosensitivity	32505000	10.1016/j.omtn.2020.05.011
negative Regulation: AKR1C1 ---| chemosensitivity	32307891	10.1111/jcmm.1529110.1111/j
negative Regulation: AKR1C1 ---| chemosensitivity	34702254	10.1186/s12935-021-02267-2
negative Regulation: CLIC1 ---| chemosensitivity		

negative Regulation: CLIC1 ---| chemosensitivity		
negative Regulation: CLIC1 ---| chemosensitivity		
negative Regulation: CLIC1 ---| chemosensitivity		
negative Regulation: TRIM44 ---| chemosensitivity		10.1016/j.gendis.2021.10.00
negative Regulation: MIR31 ---| chemosensitivity	29969627	10.1016/j.bbrc.2018.06.175
negative Regulation: MIR31 ---| chemosensitivity	28918032	10.1016/j.omtn.2017.07.0011
negative Regulation: MIR31 ---| chemosensitivity	29969627	10.1016/j.bbrc.2018.06.175
negative Regulation: MIR31 ---| chemosensitivity	27573902	10.1007/978-3-319-42059-2_1
negative Regulation: MIR31 ---| chemosensitivity	30400960	10.1186/s13046-018-0930-0
negative Regulation: MIR31 ---| chemosensitivity	30400960	10.1186/s13046-018-0930-0
negative Regulation: MIR31 ---| chemosensitivity	30400960	10.1186/s13046-018-0930-0
negative Regulation: MIR31 ---| chemosensitivity	30400960	10.1186/s13046-018-0930-0
negative Regulation: MIR31 ---| chemosensitivity	30400960	10.1186/s13046-018-0930-0
negative Regulation: MIR31 ---| chemosensitivity	30400960	10.1186/s13046-018-0930-0
negative Regulation: MIR31 ---| chemosensitivity		10.1186/s13046-019-1158-3
negative Regulation: MIR31 ---| chemosensitivity	28918032	10.1016/j.omtn.2017.07.0011
negative Regulation: MIR31 ---| chemosensitivity	29115526	10.3892/or.2017.608210.3892
negative Regulation: MIR31 ---| chemosensitivity	30400960	10.1186/s13046-018-0930-010
negative Regulation: MIR31 ---| chemosensitivity	30400960	10.1186/s13046-018-0930-010
negative Regulation: MIR31 ---| chemosensitivity	30400960	10.1186/s13046-018-0930-010
negative Regulation: MIR31 ---| chemosensitivity	30677405	10.1016/j.abb.2019.01.02310
negative Regulation: MIR31 ---| chemosensitivity	30940159	10.1186/s13046-019-1158-310
negative Regulation: S100A14 ---| chemosensitivity	33579599	10.1016/j.pan.2021.01.011
negative Regulation: DUSP4 ---| chemosensitivity	24012960	10.1016/j.yexcr.2013.08.023
negative Regulation: DUSP4 ---| chemosensitivity	24012960	10.1016/j.yexcr.2013.08.023
negative Regulation: DUSP4 ---| chemosensitivity	24012960	10.1016/j.yexcr.2013.08.023
negative Regulation: DUSP4 ---| chemosensitivity	31801953	10.1038/s41419-019-2164-2
negative Regulation: DUSP4 ---| chemosensitivity	26202679	10.3892/ijo.2015.3084
negative Regulation: DUSP4 ---| chemosensitivity	24012960	10.1016/j.yexcr.2013.08.023
negative Regulation: DUSP4 ---| chemosensitivity	24012960	10.1016/j.yexcr.2013.08.023
negative Regulation: DUSP4 ---| chemosensitivity	36234680	10.3390/molecules27196146
negative Regulation: DUSP4 ---| chemosensitivity	36234680	10.3390/molecules27196146
negative Regulation: ZKSCAN1 ---| chemosensitivity	29956811	10.3892/or.2018.6524
negative Regulation: ZKSCAN1 ---| chemosensitivity	29956811	10.3892/or.2018.6524
negative Regulation: ZKSCAN1 ---| chemosensitivity	29956811	10.3892/or.2018.6524
negative Regulation: ZKSCAN1 ---| chemosensitivity	26622672	10.3892/ol.2015.3421
negative Regulation: ZKSCAN1 ---| chemosensitivity	29956811	10.3892/or.2018.652410.3892
negative Regulation: RAF1 ---| chemosensitivity	19631573	10.1016/j.oraloncology.2009
negative Regulation: RAF1 ---| chemosensitivity	21756541	10.1179/102453311X130255689
negative Regulation: RAF1 ---| chemosensitivity	23941244	10.1089/dna.2013.2138
negative Regulation: RAF1 ---| chemosensitivity	12536201	10.1038/sj.cgt.7700544
negative Regulation: MUTYH ---| chemosensitivity	27999205	10.18632/oncotarget.1398510
negative Regulation: NFE2L2 ---| chemosensitivity	31791583	10.1016/j.bbrc.2019.11.162
negative Regulation: NFE2L2 ---| chemosensitivity	33168189	10.1016/j.bbrc.2020.10.052
negative Regulation: NFE2L2 ---| chemosensitivity	33168189	10.1016/j.bbrc.2020.10.052
negative Regulation: NFE2L2 ---| chemosensitivity	31954771	10.1016/j.canlet.2020.01.01
negative Regulation: NFE2L2 ---| chemosensitivity	29857117	10.1016/j.toxlet.2018.05.03
negative Regulation: NFE2L2 ---| chemosensitivity	32858502	10.1016/j.biopha.2020.11067
negative Regulation: NFE2L2 ---| chemosensitivity	34020028	10.1016/j.semcancer.2021.05
negative Regulation: NFE2L2 ---| chemosensitivity	31648982	10.1016/j.ebiom.2019.09.046
negative Regulation: NFE2L2 ---| chemosensitivity	30505938	10.1016/j.omto.2018.10.002
negative Regulation: NFE2L2 ---| chemosensitivity	22684020	10.1016/j.freeradbiomed.201
negative Regulation: NFE2L2 ---| chemosensitivity	22684020	10.1016/j.freeradbiomed.201
negative Regulation: NFE2L2 ---| chemosensitivity	22684020	10.1016/j.freeradbiomed.201

negative Regulation: NFE2L2 ---|	chemosensitivity	22684020	10.1016/j.freeradbiomed.201
negative Regulation: NFE2L2 ---|	chemosensitivity	29061583	10.1124/dmd.117.078741
negative Regulation: NFE2L2 ---|	chemosensitivity	29061583	10.1124/dmd.117.078741
negative Regulation: NFE2L2 ---|	chemosensitivity	30852137	10.1016/j.ymthe.2019.02.010
negative Regulation: NFE2L2 ---|	chemosensitivity	30852137	10.1016/j.ymthe.2019.02.010
negative Regulation: NFE2L2 ---|	chemosensitivity	30852137	10.1016/j.ymthe.2019.02.010
negative Regulation: NFE2L2 ---|	chemosensitivity	32943607	10.1038/s41419-020-02938-4
negative Regulation: NFE2L2 ---|	chemosensitivity	33219317	10.1038/s41388-020-01551-1
negative Regulation: NFE2L2 ---|	chemosensitivity	33219317	10.1038/s41388-020-01551-1
negative Regulation: NFE2L2 ---|	chemosensitivity	33596893	10.1186/s12935-021-01822-1
negative Regulation: NFE2L2 ---|	chemosensitivity	33619640	10.1007/s11095-021-02997-y
negative Regulation: NFE2L2 ---|	chemosensitivity	23766517	10.1210/jc.2013-1510
negative Regulation: NFE2L2 ---|	chemosensitivity	22039513	10.1371/journal.pone.002659
negative Regulation: NFE2L2 ---|	chemosensitivity	28560379	10.3892/mmr.2017.662110.389
negative Regulation: NFE2L2 ---|	chemosensitivity	30505938	10.1016/j.omto.2018.10.0021
negative Regulation: NFE2L2 ---|	chemosensitivity	31590928	10.1016/j.ebiom.2019.08.037
negative Regulation: NFE2L2 ---|	chemosensitivity	31648982	10.1016/j.ebiom.2019.09.046
negative Regulation: NFE2L2 ---|	chemosensitivity	33461143	10.1016/j.phymed.2020.15341
negative Regulation: NFE2L2 ---|	chemosensitivity	34520771	10.1016/j.lfs.2021.119948
negative Regulation: NFE2L2 ---|	chemosensitivity	35111249	10.3892/ol.2022.1320010.389
negative Regulation: NFE2L2 ---|	chemosensitivity	35272669	10.1186/s13046-022-02270-z1
negative Regulation: NFE2L2 ---|	chemosensitivity	35272669	10.1186/s13046-022-02270-z1
negative Regulation: NFE2L2 ---|	chemosensitivity	35945195	10.1038/s41419-022-05126-8
negative Regulation: NFE2L2 ---|	chemosensitivity	35945195	10.1038/s41419-022-05126-8
negative Regulation: NFE2L2 ---|	chemosensitivity	36528059	10.1016/j.jbc.2022.102798
negative Regulation: GSTA1 ---|	chemosensitivity	21150160	10.1159/000320744
negative Regulation: GREM1 ---|	chemosensitivity	32096207	10.26355/eurrev_202002_2015
negative Regulation: GREM1 ---|	chemosensitivity	32096207	10.26355/eurrev_202002_2015
negative Regulation: KDM6B ---|	chemosensitivity	33742970	10.1515/hsz-2020-0345
negative Regulation: KDM6B ---|	chemosensitivity	33742970	10.1515/hsz-2020-0345
negative Regulation: KDM6B ---|	chemosensitivity	33742970	10.1515/hsz-2020-0345
negative Regulation: KDM6B ---|	chemosensitivity	33742970	10.1515/hsz-2020-0345
negative Regulation: KDM6B ---|	chemosensitivity	33742970	10.1515/hsz-2020-034510.151
negative Regulation: CRNDE ---|	chemosensitivity	32426817	10.1042/BSR20193450
negative Regulation: CRNDE ---|	chemosensitivity	31753063	10.3727/096504019X157424720
negative Regulation: CRNDE ---|	chemosensitivity	32426817	10.1042/BSR20193450
negative Regulation: CRNDE ---|	chemosensitivity	32826865	10.1038/s41419-020-02853-8
negative Regulation: CRNDE ---|	chemosensitivity	31753063	10.3727/096504019X157424720
negative Regulation: CRNDE ---|	chemosensitivity	34454479	10.1186/s12935-021-02153-x
negative Regulation: CRNDE ---|	chemosensitivity	34454479	10.1186/s12935-021-02153-x
negative Regulation: CRNDE ---|	chemosensitivity	34454479	10.1186/s12935-021-02153-x
negative Regulation: CRNDE ---|	chemosensitivity	34454479	10.1186/s12935-021-02153-x
negative Regulation: CRNDE ---|	chemosensitivity	34454479	10.1186/s12935-021-02153-x
negative Regulation: CRNDE ---|	chemosensitivity	34454479	10.1186/s12935-021-02153-x1
negative Regulation: CRNDE ---|	chemosensitivity	34454479	10.1186/s12935-021-02153-x1
negative Regulation: CRNDE ---|	chemosensitivity	35506469	10.3892/ijo.2022.5366
negative Regulation: PLD1 ---|	chemosensitivity	33191863	10.1080/16078454.2020.18455
negative Regulation: ATG7 ---|	chemosensitivity	27268264	10.1182/blood-2016-01-69224
negative Regulation: ATG7 ---|	chemosensitivity	29130102	10.3892/or.2017.6056
negative Regulation: ATG7 ---|	chemosensitivity	27268264	10.1182/blood-2016-01-69224
negative Regulation: ATG7 ---|	chemosensitivity	29130102	10.3892/or.2017.605610.3892
negative Regulation: PLK1 ---|	chemosensitivity	30611728	10.1016/j.bbcan.2018.12.007
negative Regulation: PLK1 ---|	chemosensitivity	28487115	10.1016/j.ymthe.2017.04.016
negative Regulation: PLK1 ---|	chemosensitivity	29753186	10.1016/j.tranon.2018.04.01

negative Regulation: PLK1 ---| chemosensitivity 30876762 10.1016/j.ebiom.2019.02.012
negative Regulation: PLK1 ---| chemosensitivity 22064247 10.1016/j.biocel.2011.10.01
negative Regulation: PLK1 ---| chemosensitivity 19775446
negative Regulation: PLK1 ---| chemosensitivity 27090640 10.1128/MCB.00130-16
negative Regulation: PLK1 ---| chemosensitivity 30836927 10.2174/1871520619666190301
negative Regulation: PLK1 ---| chemosensitivity 30836927 10.2174/1871520619666190301
negative Regulation: PLK1 ---| chemosensitivity 31244432 10.2174/1871520619666190618
negative Regulation: PLK1 ---| chemosensitivity 27220401 10.3892/or.2016.4820
negative Regulation: PLK1 ---| chemosensitivity 18266952 10.1111/j.1582-4934.2008.00
negative Regulation: PLK1 ---| chemosensitivity 21402713 10.1158/0008-5472.CAN-10-20
negative Regulation: PLK1 ---| chemosensitivity 30488440 10.1002/jcp.2677710.1002/jc
negative Regulation: PLK1 ---| chemosensitivity 30876762 10.1016/j.ebiom.2019.02.012
negative Regulation: PLK1 ---| chemosensitivity 31097408 10.1016/j.ebiom.2019.05.001
negative Regulation: PLK1 ---| chemosensitivity 33418498 10.1016/j.ebiom.2020.103041
negative Regulation: PLK1 ---| chemosensitivity 35659304 10.1186/s12957-022-02617-y
negative Regulation: PLK1 ---| chemosensitivity 36476988 10.1038/s41419-022-05478-1
negative Regulation: TFAP4 ---| chemosensitivity 22615908 10.1371/journal.pone.003709
negative Regulation: TFAP4 ---| chemosensitivity 22615908 10.1371/journal.pone.003709
negative Regulation: TFAP4 ---| chemosensitivity 22615908 10.1371/journal.pone.003709
negative Regulation: KIF2A ---| chemosensitivity 31858647 10.1002/jcla.2313510.1002/j
negative Regulation: KIF2A ---| chemosensitivity 34792179 10.3892/or.2021.8229
negative Regulation: KIF2A ---| chemosensitivity 34792179 10.3892/or.2021.8229
negative Regulation: KIF2A ---| chemosensitivity 34792179 10.3892/or.2021.8229
negative Regulation: KIF2A ---| chemosensitivity 34792179 10.3892/or.2021.8229
negative Regulation: KIF2A ---| chemosensitivity 34792179 10.3892/or.2021.8229
negative Regulation: KIF2A ---| chemosensitivity 35173830
negative Regulation: KIF2A ---| chemosensitivity 35173830
negative Regulation: KIF2A ---| chemosensitivity 35173830
negative Regulation: KIF2A ---| chemosensitivity 35173830
negative Regulation: KIF2A ---| chemosensitivity 35313389 10.1002/jcla.2431310.1002/j
negative Regulation: KIF2A ---| chemosensitivity 35313389 10.1002/jcla.2431310.1002/j
negative Regulation: DAZAP2 ---| chemosensitivity 33591310 10.1093/nar/gkab08410.1093/
negative Regulation: DAZAP2 ---| chemosensitivity 33591310 10.1093/nar/gkab08410.1093/
negative Regulation: PIK3CA ---| chemosensitivity 26747178 10.1007/s13277-015-4691-5
negative Regulation: PIK3CA ---| chemosensitivity 26747178 10.1007/s13277-015-4691-5
negative Regulation: PIK3CA ---| chemosensitivity 24071646 10.1038/cddis.2013.312
negative Regulation: CREB1 ---| chemosensitivity 35613826 10.1136/jitc-2021-00379310.
negative Regulation: CYP2A6 ---| chemosensitivity 28073588 10.1016/j.surg.2016.11.0361
negative Regulation: CD55 ---| chemosensitivity 20692039 10.1016/j.molimm.2010.07.00
negative Regulation: BCL2 ---| chemosensitivity 12560233 10.1182/blood-2002-08-2501
negative Regulation: BCL2 ---| chemosensitivity 11495901 10.1074/jbc.M103632200
negative Regulation: BCL2 ---| chemosensitivity 11495901 10.1074/jbc.M103632200
negative Regulation: BCL2 ---| chemosensitivity 11495901 10.1074/jbc.M103632200
negative Regulation: BCL2 ---| chemosensitivity 11495901 10.1074/jbc.M103632200
negative Regulation: BCL2 ---| chemosensitivity 15611130 10.1074/jbc.M409906200
negative Regulation: BCL2 ---| chemosensitivity 29879503 10.1016/j.gene.2018.06.005
negative Regulation: BCL2 ---| chemosensitivity 12056703 10.1093/annonc/mdf124
negative Regulation: BCL2 ---| chemosensitivity 27371891 10.1016/j.colsurfb.2016.06.
negative Regulation: BCL2 ---| chemosensitivity 28774835 10.1016/j.semcancer.2017.07
negative Regulation: BCL2 ---| chemosensitivity 21878904 10.1038/mt.2011.174
negative Regulation: BCL2 ---| chemosensitivity 21878904 10.1038/mt.2011.174
negative Regulation: BCL2 ---| chemosensitivity 30317163 10.1016/j.omtn.2018.08.019
negative Regulation: BCL2 ---| chemosensitivity 10612539 10.1016/S0002-9610(99)00209
negative Regulation: BCL2 ---| chemosensitivity 11226371 10.1016/S0006-2952(00)00538

negative Regulation: BCL2 ---|	chemosensitivity	12175703	10.1016/S0024-3205(02)01900
negative Regulation: BCL2 ---|	chemosensitivity	16458116	10.1016/j.canlet.2005.02.01
negative Regulation: BCL2 ---|	chemosensitivity	21840388	10.1016/j.jep.2011.07.056
negative Regulation: BCL2 ---|	chemosensitivity		10.1016/S0889-8588(05)70229
negative Regulation: BCL2 ---|	chemosensitivity		10.1016/S0889-8588(05)70231
negative Regulation: BCL2 ---|	chemosensitivity		10.1016/S0889-8588(05)70231
negative Regulation: BCL2 ---|	chemosensitivity	20106644	10.1016/j.phymed.2009.12.00
negative Regulation: BCL2 ---|	chemosensitivity	18840529	10.1016/j.phrs.2008.09.005
negative Regulation: BCL2 ---|	chemosensitivity	18342810	10.1016/j.beha.2007.11.009
negative Regulation: BCL2 ---|	chemosensitivity		10.1016/j.bionut.2010.12.00
negative Regulation: BCL2 ---|	chemosensitivity	23083798	10.1016/j.clgc.2012.09.002
negative Regulation: BCL2 ---|	chemosensitivity	25596645	10.1016/j.urolonc.2014.12.0
negative Regulation: BCL2 ---|	chemosensitivity	19445670	
negative Regulation: BCL2 ---|	chemosensitivity	15611130	
negative Regulation: BCL2 ---|	chemosensitivity	11495901	
negative Regulation: BCL2 ---|	chemosensitivity	11495901	
negative Regulation: BCL2 ---|	chemosensitivity	11495901	
negative Regulation: BCL2 ---|	chemosensitivity	11495901	
negative Regulation: BCL2 ---|	chemosensitivity	11040047	
negative Regulation: BCL2 ---|	chemosensitivity	11040047	
negative Regulation: BCL2 ---|	chemosensitivity	9396780	
negative Regulation: BCL2 ---|	chemosensitivity	17353918	
negative Regulation: BCL2 ---|	chemosensitivity	28440463	10.3892/or.2017.5584
negative Regulation: BCL2 ---|	chemosensitivity	17088977	
negative Regulation: BCL2 ---|	chemosensitivity	21756541	10.1179/102453311X130255689
negative Regulation: BCL2 ---|	chemosensitivity	21785821	10.3892/ijo.2011.1140
negative Regulation: BCL2 ---|	chemosensitivity	21936689	10.1089/cbr.2011.1031
negative Regulation: BCL2 ---|	chemosensitivity	23774697	10.1097/MPA.0b013e3182854ab
negative Regulation: BCL2 ---|	chemosensitivity	21878904	10.1038/mt.2011.174
negative Regulation: BCL2 ---|	chemosensitivity	23941244	10.1089/dna.2013.2138
negative Regulation: BCL2 ---|	chemosensitivity	23848200	10.2174/1871520611313999009
negative Regulation: BCL2 ---|	chemosensitivity	24619837	10.1055/s-0034-1368198
negative Regulation: BCL2 ---|	chemosensitivity	25405848	10.3892/mmr.2014.2951
negative Regulation: BCL2 ---|	chemosensitivity	26300071	10.3892/mmr.2015.4230
negative Regulation: BCL2 ---|	chemosensitivity	7845007	
negative Regulation: BCL2 ---|	chemosensitivity	9461199	10.1038/nm0298-232
negative Regulation: BCL2 ---|	chemosensitivity	10430080	
negative Regulation: BCL2 ---|	chemosensitivity	11040047	10.1124/mol.58.5.1001
negative Regulation: BCL2 ---|	chemosensitivity	11436358	
negative Regulation: BCL2 ---|	chemosensitivity	12393493	10.1182/blood-2002-06-1899
negative Regulation: BCL2 ---|	chemosensitivity	12536201	10.1038/sj.cgt.7700544
negative Regulation: BCL2 ---|	chemosensitivity	15085157	10.1038/sj.leu.2403363
negative Regulation: BCL2 ---|	chemosensitivity	15514848	10.1093/abbs/36.11.749
negative Regulation: BCL2 ---|	chemosensitivity	15824414	10.1200/JCO.2005.09.118
negative Regulation: BCL2 ---|	chemosensitivity	15826766	10.1016/j.eururo.2004.11.01
negative Regulation: BCL2 ---|	chemosensitivity	15837754	10.1158/1078-0432.CCR-04-15
negative Regulation: BCL2 ---|	chemosensitivity	16080514	
negative Regulation: BCL2 ---|	chemosensitivity	16412276	10.1111/j.1745-7254.2006.00
negative Regulation: BCL2 ---|	chemosensitivity	19520430	10.1016/j.leukres.2009.05.0
negative Regulation: BCL2 ---|	chemosensitivity	26599548	10.1002/mc.2243710.1002/mc.
negative Regulation: BCL2 ---|	chemosensitivity	32582369	10.3332/ecancer.2020.105410
negative Regulation: BCL2 ---|	chemosensitivity	34354046	10.1038/s41419-021-04042-7
negative Regulation: BCL2 ---|	chemosensitivity	34406846	10.1089/gtmb.2021.0009
negative Regulation: BCL2 ---|	chemosensitivity	34774019	10.1186/s12885-021-08961-8

negative Regulation: BCL2 ---|	chemosensitivity	36445321	10.18632/aging.204404
negative Regulation: HDAC6 ---|	chemosensitivity	23618908	10.1038/cddis.2013.127
negative Regulation: HDAC6 ---|	chemosensitivity	34524571	10.1007/s10565-021-09651-81
negative Regulation: B4GALT5 ---|	chemosensitivity	23744354	10.1038/cddis.2013.186
negative Regulation: FGFR1 ---|	chemosensitivity	24651014	10.1016/j.ccr.2014.02.005
negative Regulation: MIR587 ---|	chemosensitivity	26247730	10.1038/cddis.2015.200
negative Regulation: LINC00518 ---|	chemosensitivit	31993309	10.1016/j.apsb.2019.09.011
negative Regulation: LINC00518 ---|	chemosensitivit	30001527	10.1159/00049165910.1159/00
negative Regulation: FAM83H ---|	chemosensitivity	33571515	10.1016/j.lfs.2021.119205
negative Regulation: CES2 ---|	chemosensitivity	31981591	10.1016/j.yexcr.2020.111856
negative Regulation: LRP1 ---|	chemosensitivity	30458112	10.1096/fj.201801339RRR10.1
negative Regulation: LRP1 ---|	chemosensitivity	35628341	10.3390/ijms23105532
negative Regulation: HULC ---|	chemosensitivity	32711110	10.1016/j.cellsig.2020.1097
negative Regulation: HULC ---|	chemosensitivity	29168767	10.3390/ijms18122505
negative Regulation: HULC ---|	chemosensitivity	30224380	10.1042/BSR20180915
negative Regulation: HULC ---|	chemosensitivity	30472720	10.12659/MSM.911179
negative Regulation: HULC ---|	chemosensitivity	31614437	10.3390/ijms20205029
negative Regulation: HULC ---|	chemosensitivity	32627034	10.3892/or.2020.7608
negative Regulation: HULC ---|	chemosensitivity	32744688	10.26355/eurrev_202007_2226
negative Regulation: HULC ---|	chemosensitivity	33067424	10.1038/s41419-020-03080-x
negative Regulation: HULC ---|	chemosensitivity	34223709	10.1002/prp2.81510.1002/prp
negative Regulation: DROSHA ---|	chemosensitivity	27609577	
negative Regulation: B3GNT8 ---|	chemosensitivity	22579717	10.1016/j.biocel.2012.04.02
negative Regulation: SUV39H2 ---|	chemosensitivity	31636512	10.1186/s12935-019-0982-z
negative Regulation: SUV39H2 ---|	chemosensitivity	31636512	10.1186/s12935-019-0982-z
negative Regulation: SUV39H2 ---|	chemosensitivity	31636512	10.1186/s12935-019-0982-z
negative Regulation: SUV39H2 ---|	chemosensitivity	31636512	10.1186/s12935-019-0982-z
negative Regulation: SUV39H2 ---|	chemosensitivity	31636512	10.1186/s12935-019-0982-z
negative Regulation: SUV39H2 ---|	chemosensitivity	31636512	10.1186/s12935-019-0982-z10
negative Regulation: ABCC6 ---|	chemosensitivity	28582730	10.1016/j.redox.2017.05.025
negative Regulation: CCNE2 ---|	chemosensitivity	28176879	10.1038/srep4231910.1038/sr
negative Regulation: HOTTIP ---|	chemosensitivity	28254409	10.1016/j.canlet.2017.02.02
negative Regulation: HOTTIP ---|	chemosensitivity	31465841	10.1016/j.canlet.2019.08.01
negative Regulation: HOTTIP ---|	chemosensitivity	32335298	10.1016/j.biopha.2020.11015
negative Regulation: HOTTIP ---|	chemosensitivity	33207266	10.1016/j.semcancer.2020.11
negative Regulation: HOTTIP ---|	chemosensitivity	27546609	10.1039/c6mb00475j
negative Regulation: HOTTIP ---|	chemosensitivity	28586074	10.3892/or.2017.5691
negative Regulation: HOTTIP ---|	chemosensitivity	25779149	10.1186/s12967-015-0442-z
negative Regulation: HOTTIP ---|	chemosensitivity	25779149	10.1186/s12967-015-0442-z
negative Regulation: HOTTIP ---|	chemosensitivity	25779149	10.1186/s12967-015-0442-z
negative Regulation: HOTTIP ---|	chemosensitivity	30652087	10.21037/hbsn.2018.10.0710.
negative Regulation: HOTTIP ---|	chemosensitivity	30809864	10.1002/jcb.2785110.1002/jc
negative Regulation: HOTTIP ---|	chemosensitivity		10.1016/j.semcancer.2022.03
negative Regulation: HOTTIP ---|	chemosensitivity	34365949	10.2174/156652402166621080б
negative Regulation: HOTTIP ---|	chemosensitivity	34365949	10.2174/1566524021666210806
negative Regulation: HOTTIP ---|	chemosensitivity	36514224	10.3724/abbs.2022182
negative Regulation: BTK ---|	chemosensitivity	10092645	
negative Regulation: ALDOA ---|	chemosensitivity	28000858	10.3892/ijo.2016.3814
negative Regulation: ALDOA ---|	chemosensitivity	28000858	10.3892/ijo.2016.3814
negative Regulation: NFKBIA ---|	chemosensitivity	15327837	10.1016/j.canlet.2004.03.04
negative Regulation: NFKBIA ---|	chemosensitivity	12824895	10.1111/j.1349-7006.2003.tb
negative Regulation: NFKBIA ---|	chemosensitivity	12824895	10.1111/j.1349-7006.2003.tb
negative Regulation: NFKBIA ---|	chemosensitivity	12871783	10.1016/s0169-5002(03)00227
negative Regulation: FGD5 ---|	chemosensitivity	33416094	10.3892/ijmm.2020.4816

negative Regulation:	CCDC88A ---|	chemosensitivity	29989653	10.3892/or.2018.6511
negative Regulation:	CCDC88A ---|	chemosensitivity	25009397	10.3748/wjg.v20.i25.822910.
negative Regulation:	CCDC88A ---|	chemosensitivity	33854963	10.3389/fonc.2021.61876410.
negative Regulation:	CCDC88A ---|	chemosensitivity	33854963	10.3389/fonc.2021.61876410.
negative Regulation:	MIR3658 ---|	chemosensitivity	29739079	10.7754/Clin.Lab.2017.17102
negative Regulation:	MIR3658 ---|	chemosensitivity	30988071	10.1042/BSR20181743
negative Regulation:	MIR3658 ---|	chemosensitivity	29739079	10.7754/Clin.Lab.2017.17102
negative Regulation:	C4orf47 ---|	chemosensitivity		10.1093/annonc/mdx660.043
negative Regulation:	PTGS1 ---|	chemosensitivity	32653589	10.1016/j.bcp.2020.114147
negative Regulation:	PTGS1 ---|	chemosensitivity	33075476	10.1016/j.prostaglandins.20
negative Regulation:	EIF4G2 ---|	chemosensitivity	33526055	10.1186/s13046-021-01853-6
negative Regulation:	EIF4G2 ---|	chemosensitivity	28117895	10.1002/1873-3468.1256610.1
negative Regulation:	BIRC5 ---|	chemosensitivity	29678577	10.1016/j.bbrc.2018.04.129
negative Regulation:	BIRC5 ---|	chemosensitivity	28624478	10.1016/j.addr.2017.06.004
negative Regulation:	BIRC5 ---|	chemosensitivity	28505590	10.1016/j.jpba.2017.04.025
negative Regulation:	BIRC5 ---|	chemosensitivity	28577912	10.1016/j.drudis.2017.05.00
negative Regulation:	BIRC5 ---|	chemosensitivity	22169827	10.1016/j.biomaterials.2011
negative Regulation:	BIRC5 ---|	chemosensitivity	22169827	10.1016/j.biomaterials.2011
negative Regulation:	BIRC5 ---|	chemosensitivity	19318043	10.1016/j.pharmthera.2009.0
negative Regulation:	BIRC5 ---|	chemosensitivity	21130850	10.1016/j.ijpharm.2010.11.0
negative Regulation:	BIRC5 ---|	chemosensitivity	27459907	10.3892/or.2016.4958
negative Regulation:	BIRC5 ---|	chemosensitivity	27489375	10.1136/postgradmedj-2016-1
negative Regulation:	BIRC5 ---|	chemosensitivity	29328459	10.3892/or.2018.6187
negative Regulation:	BIRC5 ---|	chemosensitivity	27063218	10.1038/aps.2015.152
negative Regulation:	BIRC5 ---|	chemosensitivity	31711057	10.1159/000502913
negative Regulation:	BIRC5 ---|	chemosensitivity	32945459	10.3892/or.2020.7698
negative Regulation:	BIRC5 ---|	chemosensitivity	22426961	10.1007/s00432-012-1196-0
negative Regulation:	BIRC5 ---|	chemosensitivity	23254641	10.3892/or.2012.2196
negative Regulation:	BIRC5 ---|	chemosensitivity	23254641	10.3892/or.2012.2196
negative Regulation:	BIRC5 ---|	chemosensitivity	22117970	
negative Regulation:	BIRC5 ---|	chemosensitivity	20490863	10.1007/s00432-010-0914-8
negative Regulation:	BIRC5 ---|	chemosensitivity	20563741	10.1007/s11010-010-0525-3
negative Regulation:	BIRC5 ---|	chemosensitivity	22211100	
negative Regulation:	BIRC5 ---|	chemosensitivity	23091600	10.1371/journal.pone.004673
negative Regulation:	BIRC5 ---|	chemosensitivity	21034604	10.3760/cma.j.issn.0366-699
negative Regulation:	BIRC5 ---|	chemosensitivity	21034604	10.3760/cma.j.issn.0366-699
negative Regulation:	BIRC5 ---|	chemosensitivity	24330851	10.3727/096504013X137935557
negative Regulation:	BIRC5 ---|	chemosensitivity	23990130	10.1007/s12013-013-9722-5
negative Regulation:	BIRC5 ---|	chemosensitivity	23509217	10.1515/hsz-2013-0128
negative Regulation:	BIRC5 ---|	chemosensitivity	26081496	10.3892/or.2015.4067
negative Regulation:	BIRC5 ---|	chemosensitivity	26125270	
negative Regulation:	BIRC5 ---|	chemosensitivity	26392111	10.1007/s13277-015-4075-x
negative Regulation:	BIRC5 ---|	chemosensitivity	27050416	10.1371/journal.pone.015301
negative Regulation:	BIRC5 ---|	chemosensitivity	17686279	
negative Regulation:	BIRC5 ---|	chemosensitivity	17686279	
negative Regulation:	BIRC5 ---|	chemosensitivity	17686279	
negative Regulation:	BIRC5 ---|	chemosensitivity	18594980	10.1007/s10620-008-0329-410
negative Regulation:	BIRC5 ---|	chemosensitivity	19280061	10.1093/abbs/gmp005
negative Regulation:	BIRC5 ---|	chemosensitivity	19921309	10.1007/s11845-009-0448-810
negative Regulation:	BIRC5 ---|	chemosensitivity	19951584	
negative Regulation:	BIRC5 ---|	chemosensitivity	20441052	10.3727/096504010x126444223
negative Regulation:	BIRC5 ---|	chemosensitivity	20441052	10.3727/096504010x126444223
negative Regulation:	BIRC5 ---|	chemosensitivity	22068035	10.1158/0008-5472.CAN-11-23
negative Regulation:	BIRC5 ---|	chemosensitivity	22593446	

negative Regulation: BIRC5 ---| chemosensitivity	23254641	10.3892/or.2012.219610.3892
negative Regulation: BIRC5 ---| chemosensitivity	23358177	10.1097/IGC.0b013e31827ad2b
negative Regulation: BIRC5 ---| chemosensitivity	23591345	
negative Regulation: BIRC5 ---| chemosensitivity	24212789	10.3390/cancers302192910.33
negative Regulation: BIRC5 ---| chemosensitivity	24460358	10.7314/apjcp.2013.14.12.77
negative Regulation: BIRC5 ---| chemosensitivity	25070628	10.3892/mmr.2014.241310.389
negative Regulation: BIRC5 ---| chemosensitivity	25070628	10.3892/mmr.2014.241310.389
negative Regulation: BIRC5 ---| chemosensitivity	26123662	10.1016/j.canlet.2015.05.03
negative Regulation: BIRC5 ---| chemosensitivity	29678577	10.1016/j.bbrc.2018.04.1291
negative Regulation: BIRC5 ---| chemosensitivity	25707849	10.3892/mmr.2015.3380
negative Regulation: BIRC5 ---| chemosensitivity		10.1016/j.biomaterials.2011
negative Regulation: BIRC5 ---| chemosensitivity		10.1016/j.biomaterials.2011
negative Regulation: BIRC5 ---| chemosensitivity		10.1159/000502913
negative Regulation: BIRC5 ---| chemosensitivity	16224667	
negative Regulation: BIRC5 ---| chemosensitivity	35706019	10.1186/s12929-022-00824-z
negative Regulation: IL17RA ---| chemosensitivity	33243748	10.12122/j.issn.1673-4254.2
negative Regulation: CS ---| chemosensitivity	25545012	10.1371/journal.pone.011570
negative Regulation: CS ---| chemosensitivity	25545012	10.1371/journal.pone.011570
negative Regulation: FOXO4 ---| chemosensitivity	33152911	10.1016/j.biopha.2020.11072
negative Regulation: HGF ---| chemosensitivity	22820099	10.1016/j.abb.2012.07.003
negative Regulation: HGF ---| chemosensitivity	22741575	10.1186/1477-7819-10-128
negative Regulation: HGF ---| chemosensitivity	10924353	10.1006/bbrc.2000.3199
negative Regulation: HGF ---| chemosensitivity	22741575	10.1186/1477-7819-10-128
negative Regulation: HGF ---| chemosensitivity	22741575	10.1186/1477-7819-10-128
negative Regulation: BCAT1 ---| chemosensitivity	33760210	10.3892/OR.2021.8003
negative Regulation: BCAT1 ---| chemosensitivity	28052414	10.1002/mc.2261210.1002/mc.
negative Regulation: BCAT1 ---| chemosensitivity	33798550	10.1016/j.lfs.2021.11940510
negative Regulation: MIR663A ---| chemosensitivity	23436656	10.1074/jbc.M112.434340
negative Regulation: MIR663A ---| chemosensitivity	23436656	10.1074/jbc.M112.434340
negative Regulation: MIR663A ---| chemosensitivity	23436656	10.1074/jbc.M112.434340
negative Regulation: MIR663A ---| chemosensitivity	33116621	10.2147/OTT.S26157010.2147/
negative Regulation: BIRC3 ---| chemosensitivity	27002147	10.1074/jbc.M115.700021
negative Regulation: BIRC3 ---| chemosensitivity	32298617	10.1016/j.tim.2020.01.001
negative Regulation: BIRC3 ---| chemosensitivity	21952624	10.1038/bjc.2011.387
negative Regulation: BIRC3 ---| chemosensitivity	21952624	10.1038/bjc.2011.387
negative Regulation: BIRC3 ---| chemosensitivity	27002147	10.1074/jbc.M115.700021
negative Regulation: MIR329-1 ---| chemosensitivity	33989708	10.1016/j.canlet.2021.05.00
negative Regulation: PCGF2 ---| chemosensitivity	31186712	10.3892/ol.2019.1016010.389
negative Regulation: ATR ---| chemosensitivity	18413665	
negative Regulation: ATR ---| chemosensitivity	21927021	10.1038/onc.2011.399
negative Regulation: ATR ---| chemosensitivity	18413665	10.1124/mol.107.04474310.11
negative Regulation: ATR ---| chemosensitivity	21927021	10.1038/onc.2011.39910.1038
negative Regulation: ATR ---| chemosensitivity	30666155	10.2147/CMAR.S18709910.2147
negative Regulation: ATR ---| chemosensitivity	35530299	
negative Regulation: RAD50 ---| chemosensitivity	26951044	10.1186/s12885-016-2190-8
negative Regulation: LINC00337 ---| chemosensitivit	32239565	10.1096/fj.201900731RR10.10
negative Regulation: ELK1 ---| chemosensitivity	28987388	10.1016/j.canlet.2017.09.04
negative Regulation: NEDD4 ---| chemosensitivity	33288736	10.18632/aging.202185
negative Regulation: MAPK3 ---| chemosensitivity	22179790	10.3892/ijo.2011.1303
negative Regulation: MAPK3 ---| chemosensitivity	22179790	10.3892/ijo.2011.1303
negative Regulation: MAPK3 ---| chemosensitivity	22179790	10.3892/ijo.2011.1303
negative Regulation: MAPK3 ---| chemosensitivity	26316041	10.3892/ijo.2015.3137
negative Regulation: MAPK3 ---| chemosensitivity	22179790	10.3892/ijo.2011.1303
negative Regulation: MAPK3 ---| chemosensitivity	22179790	10.3892/ijo.2011.1303

negative Regulation: MAPK3 ---| chemosensitivity		
negative Regulation: VEGFA ---| chemosensitivity	28119236	10.1016/j.biopha.2017.01.05
negative Regulation: VEGFA ---| chemosensitivity	21397054	10.1016/j.resp.2011.03.006
negative Regulation: VEGFA ---| chemosensitivity	33838253	10.1016/j.matbio.2021.04.00
negative Regulation: VEGFA ---| chemosensitivity	28742793	10.1038/bjc.2017.238
negative Regulation: VEGFA ---| chemosensitivity	28742793	10.1038/bjc.2017.238
negative Regulation: VEGFA ---| chemosensitivity	27362808	10.1038/cddis.2016.194
negative Regulation: VEGFA ---| chemosensitivity	21057529	10.1038/onc.2010.496
negative Regulation: VEGFA ---| chemosensitivity	26199906	10.22074/cellj.2016.3730
negative Regulation: VEGFA ---| chemosensitivity	28742793	10.1038/bjc.2017.23810.1038
negative Regulation: DUSP6 ---| chemosensitivity		10.1016/j.mgene.2020.100692
negative Regulation: PDK4 ---| chemosensitivity	27330076	10.1074/jbc.M116.713735
negative Regulation: PDK4 ---| chemosensitivity	33667731	10.1016/j.clinre.2021.10166
negative Regulation: PDK4 ---| chemosensitivity	32577500	10.1016/j.omto.2020.05.008
negative Regulation: PDK4 ---| chemosensitivity	27330076	10.1074/jbc.M116.713735
negative Regulation: PDK4 ---| chemosensitivity	32444598	10.1038/s41467-020-16306-5
negative Regulation: PDK4 ---| chemosensitivity	32444598	10.1038/s41467-020-16306-5
negative Regulation: PLXND1 ---| chemosensitivity	35917012	10.1245/s10434-022-11945-y
negative Regulation: CSF1 ---| chemosensitivity	31629410	10.1186/s12929-019-0568-z
negative Regulation: CSF1 ---| chemosensitivity		10.1186/s12929-019-0568-z
negative Regulation: RIPK2 ---| chemosensitivity	24642040	10.1186/bcr3629
negative Regulation: PTGS2 ---| chemosensitivity	31494169	10.1016/j.lfs.2019.116825
negative Regulation: PTGS2 ---| chemosensitivity	30954568	10.1016/j.bbamcr.2019.04.00
negative Regulation: PTGS2 ---| chemosensitivity	31473273	10.1016/j.virusres.2019.197
negative Regulation: PTGS2 ---| chemosensitivity	16580341	10.1016/j.ajog.2005.07.086
negative Regulation: PTGS2 ---| chemosensitivity	19836060	10.1016/j.acthis.2009.09.00
negative Regulation: PTGS2 ---| chemosensitivity		10.1053/j.seminoncol.2003.1
negative Regulation: PTGS2 ---| chemosensitivity		10.1016/j.ijrobp.2007.07.17
negative Regulation: PTGS2 ---| chemosensitivity		10.1016/j.ijrobp.2010.07.14
negative Regulation: PTGS2 ---| chemosensitivity	24184161	10.1016/j.bbamcr.2013.10.01
negative Regulation: PTGS2 ---| chemosensitivity	24184161	10.1016/j.bbamcr.2013.10.01
negative Regulation: PTGS2 ---| chemosensitivity	18356920	
negative Regulation: PTGS2 ---| chemosensitivity	33138752	10.2174/1381612826666201102
negative Regulation: PTGS2 ---| chemosensitivity	23393346	
negative Regulation: PTGS2 ---| chemosensitivity	16912190	10.1158/0008-5472.CAN-06-12
negative Regulation: PTGS2 ---| chemosensitivity	17982644	
negative Regulation: PTGS2 ---| chemosensitivity	18592138	10.1007/s11010-008-9847-910
negative Regulation: PTGS2 ---| chemosensitivity	19276291	10.1158/1078-0432.CCR-08-06
negative Regulation: PTGS2 ---| chemosensitivity	28881683	10.18632/oncotarget.1851810
negative Regulation: PTGS2 ---| chemosensitivity	30365050	10.3892/ijmm.2018.393910.38
negative Regulation: PTGS2 ---| chemosensitivity	34084214	10.3892/ol.2021.1279410.389
negative Regulation: PTGS2 ---| chemosensitivity		10.1016/j.bbamcr.2019.04.00
negative Regulation: PTGS2 ---| chemosensitivity		
negative Regulation: IKBKB ---| chemosensitivity	19940859	
negative Regulation: IKBKB ---| chemosensitivity	19940859	10.1038/jid.2009.36510.1038
negative Regulation: SOX9 ---| chemosensitivity	33524528	10.1016/j.bbcan.2021.188517
negative Regulation: SOX9 ---| chemosensitivity	28984791	10.1097/MPA.000000000000094
negative Regulation: SOX9 ---| chemosensitivity	28984791	10.1097/MPA.000000000000094
negative Regulation: SOX9 ---| chemosensitivity	29749469	10.3892/ijo.2018.4382
negative Regulation: CHRNA7 ---| chemosensitivity	31962133	10.1016/j.lfs.2020.117332
negative Regulation: CHRNA7 ---| chemosensitivity	35972579	10.1007/s12032-022-01745-5
negative Regulation: CHRNA7 ---| chemosensitivity	35972579	10.1007/s12032-022-01745-5
negative Regulation: CHRNA7 ---| chemosensitivity	35972579	10.1007/s12032-022-01745-5
negative Regulation: CHRNA7 ---| chemosensitivity	35972579	10.1007/s12032-022-01745-5

negative Regulation: RPS6KA5 ---| chemosensitivity 28314603 10.1016/j.dld.2017.02.009
negative Regulation: RPS6KA5 ---| chemosensitivity 28314603 10.1016/j.dld.2017.02.00910
negative Regulation: CYRIB ---| chemosensitivity 34645466 10.1186/s12935-021-02244-9
negative Regulation: DKK4 ---| chemosensitivity 28981599 10.1093/abbs/gmx08610.1093/
negative Regulation: DKK4 ---| chemosensitivity 34255218 10.1007/s10528-021-10080-9
negative Regulation: S100A8 ---| chemosensitivity 27956388 10.1182/blood-2016-07-69270
negative Regulation: S100A8 ---| chemosensitivity 33801279 10.3390/molecules26051323
negative Regulation: S100A8 ---| chemosensitivity 24820971 10.1371/journal.pone.009724
negative Regulation: EPS8 ---| chemosensitivity 29192326 10.3892/or.2017.6102
negative Regulation: EPS8 ---| chemosensitivity 25359883 10.1242/jcs.157560
negative Regulation: EPS8 ---| chemosensitivity 25803781 10.1371/journal.pone.011938
negative Regulation: EPS8 ---| chemosensitivity 29192326 10.3892/or.2017.6102
negative Regulation: EPS8 ---| chemosensitivity 29192326 10.3892/or.2017.6102
negative Regulation: EPS8 ---| chemosensitivity 29357910 10.1186/s13046-018-0682-x
negative Regulation: EPS8 ---| chemosensitivity 29357910 10.1186/s13046-018-0682-x
negative Regulation: EPS8 ---| chemosensitivity 29357910 10.1186/s13046-018-0682-x
negative Regulation: EPS8 ---| chemosensitivity 33432368 10.3892/or.2021.7927
negative Regulation: TDGF1 ---| chemosensitivity 29445127 10.1038/s41418-018-0059-x
negative Regulation: SLC25A5 ---| chemosensitivity 22198296
negative Regulation: SLC25A5 ---| chemosensitivity 22198296
negative Regulation: SLC25A5 ---| chemosensitivity 22198296
negative Regulation: SLC25A5 ---| chemosensitivity 22198296 10.3858/emm.2012.44.4.01910
negative Regulation: NAMPT ---| chemosensitivity 30876976 10.1016/j.ejphar.2019.03.01
negative Regulation: NAMPT ---| chemosensitivity 33823623 10.1177/09603271211006168
negative Regulation: NAMPT ---| chemosensitivity 35506454 10.3892/ijo.2022.536510.389
negative Regulation: NAMPT ---| chemosensitivity 35506454 10.3892/ijo.2022.536510.389
negative Regulation: NAMPT ---| chemosensitivity 35506454 10.3892/ijo.2022.5365
negative Regulation: LASP1 ---| chemosensitivity 33915163 10.1016/j.brainres.2021.147
negative Regulation: LASP1 ---| chemosensitivity 29980193 10.1186/s12885-018-4649-2
negative Regulation: LASP1 ---| chemosensitivity 29980193 10.1186/s12885-018-4649-2
negative Regulation: LASP1 ---| chemosensitivity 29980193 10.1186/s12885-018-4649-2
negative Regulation: LASP1 ---| chemosensitivity 29980193 10.1186/s12885-018-4649-210
negative Regulation: FLT3LG ---| chemosensitivity 17942916 10.1158/0008-5472.CAN-07-01
negative Regulation: RFC4 ---| chemosensitivity 18492021 10.1111/j.1478-3231.2008.01
negative Regulation: PHF20 ---| chemosensitivity 33982773 10.3892/ijo.2021.5218
negative Regulation: PHF20 ---| chemosensitivity 33982773 10.3892/ijo.2021.5218
negative Regulation: PHF20 ---| chemosensitivity 33982773 10.3892/ijo.2021.5218
negative Regulation: PHF20 ---| chemosensitivity 33982773 10.3892/ijo.2021.521810.389
negative Regulation: PHF20 ---| chemosensitivity 33982773 10.3892/ijo.2021.521810.389
negative Regulation: P4HA1 ---| chemosensitivity 31239153 10.1016/j.bbrc.2019.06.096
negative Regulation: MAF1 ---| chemosensitivity 30628658 10.3892/ijo.2019.4678
negative Regulation: PROX1-AS1 ---| chemosensitivit34583623 10.1080/15384101.2021.19713
negative Regulation: LINC00667 ---| chemosensitivit36281649 10.4081/ejh.2022.3529
negative Regulation: USP22 ---| chemosensitivity 32687947 10.1016/j.gene.2020.144960
negative Regulation: USP22 ---| chemosensitivity 31133011 10.1186/s12929-019-0522-0
negative Regulation: USP22 ---| chemosensitivity 31776228 10.1136/gutjnl-2019-319616
negative Regulation: BRCA2 ---| chemosensitivity 25758301 10.1016/j.bulcan.2015.01.01
negative Regulation: BRCA2 ---| chemosensitivity 24131965 10.1093/annonc/mdt30710.109
negative Regulation: BRCA2 ---| chemosensitivity 25758301 10.1016/j.bulcan.2015.01.01
negative Regulation: SNHG8 ---| chemosensitivity
negative Regulation: SNHG8 ---| chemosensitivity
negative Regulation: SNHG8 ---| chemosensitivity 30556854 10.26355/eurrev_201812_1650
negative Regulation: SNHG8 ---| chemosensitivity 30556854 10.26355/eurrev_201812_1650
negative Regulation: SNHG8 ---| chemosensitivity 30556854 10.26355/eurrev_201812_1650

negative Regulation: NEDD9 ---| chemosensitivity		31411930	10.1089/cbr.2018.2761
negative Regulation: USP7 ---| chemosensitivity		29065837	10.2174/1573406413666171020
negative Regulation: USP7 ---| chemosensitivity		24136231	10.1038/cddis.2013.400
negative Regulation: USP7 ---| chemosensitivity			
negative Regulation: USP7 ---| chemosensitivity		36291159	10.3390/cells11203294
negative Regulation: CLPTM1L ---| chemosensitivity		23300716	10.1371/journal.pone.005259
negative Regulation: AQP5 ---| chemosensitivity		29390193	10.1139/bcb-2017-0162
negative Regulation: AQP5 ---| chemosensitivity		29390193	10.1139/bcb-2017-0162
negative Regulation: AQP5 ---| chemosensitivity		29390193	10.1139/bcb-2017-0162
negative Regulation: AQP5 ---| chemosensitivity		29390193	10.1139/bcb-2017-0162
negative Regulation: AQP5 ---| chemosensitivity		29390193	10.1139/bcb-2017-016210.113
negative Regulation: KRT16 ---| chemosensitivity		30782177	10.1186/s13046-019-1091-5
negative Regulation: KRT16 ---| chemosensitivity		30782177	10.1186/s13046-019-1091-5
negative Regulation: KRT16 ---| chemosensitivity		30782177	10.1186/s13046-019-1091-5
negative Regulation: KRT16 ---| chemosensitivity		30782177	10.1186/s13046-019-1091-510
negative Regulation: QKI ---| chemosensitivity		31786621	10.1007/s00210-019-01772-6
negative Regulation: ENPP1 ---| chemosensitivity		24531536	10.1038/cdd.2014.12
negative Regulation: ENPP1 ---| chemosensitivity		24531536	10.1038/cdd.2014.12
negative Regulation: ATG4B ---| chemosensitivity		31291988	10.1186/s13046-019-1287-8
negative Regulation: RNF8 ---| chemosensitivity		27618486	10.1016/j.molcel.2016.08.00
negative Regulation: RNF8 ---| chemosensitivity		32572597	10.1007/s00401-020-02180-4
negative Regulation: RNF8 ---| chemosensitivity		747	10.1016/j.molcel.2016.08.00
negative Regulation: CLDN1 ---| chemosensitivity		29524521	10.1016/j.bbamcr.2018.03.00
negative Regulation: CLDN1 ---| chemosensitivity		29524521	10.1016/j.bbamcr.2018.03.00
negative Regulation: CLDN1 ---| chemosensitivity		32824620	10.3390/ijms21165909
negative Regulation: CLDN1 ---| chemosensitivity		32824620	10.3390/ijms21165909
negative Regulation: CLDN1 ---| chemosensitivity		33828978	10.3389/fonc.2021.62087310.
negative Regulation: ABCC1 ---| chemosensitivity		32278855	10.1016/j.ejphar.2020.17310
negative Regulation: ABCC1 ---| chemosensitivity		21219875	10.1016/j.bcp.2010.12.018
negative Regulation: ABCC1 ---| chemosensitivity		11864633	10.1016/S0014-2999(02)01267
negative Regulation: ABCC1 ---| chemosensitivity		22771706	10.1016/j.brainres.2012.06.
negative Regulation: ABCC1 ---| chemosensitivity		30001527	10.1159/000491659
negative Regulation: ABCC1 ---| chemosensitivity		31582906	10.1186/s12935-019-0943-6
negative Regulation: ABCC1 ---| chemosensitivity		34054937	10.1155/2021/5525091
negative Regulation: ABCC1 ---| chemosensitivity		22460269	10.1038/bjc.2012.81
negative Regulation: ABCC1 ---| chemosensitivity		23686318	10.1124/dmd.113.052092
negative Regulation: ABCC1 ---| chemosensitivity		23686318	10.1124/dmd.113.052092
negative Regulation: ABCC1 ---| chemosensitivity		25895710	10.1007/978-3-319-16537-0_7
negative Regulation: ABCC1 ---| chemosensitivity			10.1186/s12935-019-0943-6
negative Regulation: ABCC1 ---| chemosensitivity		34510251	10.1007/s00280-021-04337-8
negative Regulation: DDIT4-AS1 ---| chemosensitivit		36056355	10.1186/s12943-022-01647-0
negative Regulation: DDIT4-AS1 ---| chemosensitivit		36056355	10.1186/s12943-022-01647-0
negative Regulation: DDIT4-AS1 ---| chemosensitivit		36056355	10.1186/s12943-022-01647-0
negative Regulation: DDIT4-AS1 ---| chemosensitivit		36056355	10.1186/s12943-022-01647-0
negative Regulation: DDIT4-AS1 ---| chemosensitivit		36056355	10.1186/s12943-022-01647-0
negative Regulation: DDIT4-AS1 ---| chemosensitivit		36056355	10.1186/s12943-022-01647-0
negative Regulation: DDIT4-AS1 ---| chemosensitivit		36056355	10.1186/s12943-022-01647-0
negative Regulation: DDIT4-AS1 ---| chemosensitivit		36056355	10.1186/s12943-022-01647-0
negative Regulation: DDIT4-AS1 ---| chemosensitivit		36056355	10.1186/s12943-022-01647-0
negative Regulation: MIR1238 ---| chemosensitivity		30917935	10.1016/j.ebiom.2019.03.016
negative Regulation: TERT ---| chemosensitivity		26772886	10.1016/j.bbrc.2016.01.014
negative Regulation: TERT ---| chemosensitivity		30716337	10.1016/j.lfs.2019.02.005
negative Regulation: TERT ---| chemosensitivity			10.1016/j.annonc.2020.08.46
negative Regulation: TERT ---| chemosensitivity		33631540	10.1016/j.ejca.2021.01.014

negative Regulation: TERT ---| chemosensitivity 18708758 10.4161/cbt.7.10.6539
negative Regulation: TERT ---| chemosensitivity 26497550 10.18632/oncotarget.5468
negative Regulation: MIR21 ---| chemosensitivity 28495512 10.1016/j.abb.2017.05.001
negative Regulation: MIR21 ---| chemosensitivity 29890129 10.1016/j.cbi.2018.06.006
negative Regulation: MIR21 ---| chemosensitivity 24140396 10.1053/j.gastro.2013.10.01
negative Regulation: MIR21 ---| chemosensitivity 30802686 10.1016/j.biomaterials.2019
negative Regulation: MIR21 ---| chemosensitivity 32035242 10.1016/j.gene.2020.144453
negative Regulation: MIR21 ---| chemosensitivity 32428836 10.1016/j.biopha.2020.11023
negative Regulation: MIR21 ---| chemosensitivity 28400205 10.1016/j.tiv.2017.04.007
negative Regulation: MIR21 ---| chemosensitivity 29196189 10.1016/j.semcancer.2017.11
negative Regulation: MIR21 ---| chemosensitivity 33865701 10.1016/j.semcdb.2021.04.00
negative Regulation: MIR21 ---| chemosensitivity 32828020 10.1016/j.oraloncology.2020
negative Regulation: MIR21 ---| chemosensitivity 27623176 10.1016/j.molmed.2016.08.00
negative Regulation: MIR21 ---| chemosensitivity 30579238 10.1016/j.canep.2018.12.008
negative Regulation: MIR21 ---| chemosensitivity 30597288 10.1016/j.tranon.2018.12.00
negative Regulation: MIR21 ---| chemosensitivity 28718444 10.1016/j.trecan.2016.12.00
negative Regulation: MIR21 ---| chemosensitivity 22130252 10.1016/j.acthis.2011.11.00
negative Regulation: MIR21 ---| chemosensitivity 22130252 10.1016/j.acthis.2011.11.00
negative Regulation: MIR21 ---| chemosensitivity 21376256 10.1016/j.arcmed.2011.01.00
negative Regulation: MIR21 ---| chemosensitivity 21376256 10.1016/j.arcmed.2011.01.00
negative Regulation: MIR21 ---| chemosensitivity 21820606 10.1016/j.arcmed.2011.06.00
negative Regulation: MIR21 ---| chemosensitivity 21820606 10.1016/j.arcmed.2011.06.00
negative Regulation: MIR21 ---| chemosensitivity 20219416 10.1016/j.oraloncology.2010
negative Regulation: MIR21 ---| chemosensitivity 23177026 10.1016/j.molonc.2012.10.01
negative Regulation: MIR21 ---| chemosensitivity 24148247 10.1016/j.bbrc.2013.10.051
negative Regulation: MIR21 ---| chemosensitivity 24472409 10.1016/j.ygyno.2014.01.034
negative Regulation: MIR21 ---| chemosensitivity 25304373 10.1016/j.canlet.2014.09.03
negative Regulation: MIR21 ---| chemosensitivity 24906642 10.1016/j.cancergen.2014.04
negative Regulation: MIR21 ---| chemosensitivity 24906642 10.1016/j.cancergen.2014.04
negative Regulation: MIR21 ---| chemosensitivity 26142886 10.1016/j.abb.2015.07.001
negative Regulation: MIR21 ---| chemosensitivity 27162073 10.1016/j.biomaterials.2016
negative Regulation: MIR21 ---| chemosensitivity 20113523
negative Regulation: MIR21 ---| chemosensitivity 20113523
negative Regulation: MIR21 ---| chemosensitivity 20113523
negative Regulation: MIR21 ---| chemosensitivity 26975392 10.1186/s12885-016-2231-3
negative Regulation: MIR21 ---| chemosensitivity 27070574 10.3390/ijms17040517
negative Regulation: MIR21 ---| chemosensitivity 27573902 10.1007/978-3-319-42059-2_1
negative Regulation: MIR21 ---| chemosensitivity 26223974 10.1186/s13048-015-0178-7
negative Regulation: MIR21 ---| chemosensitivity 27712594 10.3727/096504016X146850341
negative Regulation: MIR21 ---| chemosensitivity 27748936 10.3892/or.2016.5140
negative Regulation: MIR21 ---| chemosensitivity 27448297 10.1007/s13277-016-5216-6
negative Regulation: MIR21 ---| chemosensitivity 28407783 10.1186/s13046-017-0528-y
negative Regulation: MIR21 ---| chemosensitivity 28407783 10.1186/s13046-017-0528-y
negative Regulation: MIR21 ---| chemosensitivity 28799568 10.1038/cgt.2017.20
negative Regulation: MIR21 ---| chemosensitivity 29242381 10.1042/BCJ20170079
negative Regulation: MIR21 ---| chemosensitivity 27063218 10.1038/aps.2015.152
negative Regulation: MIR21 ---| chemosensitivity 30194558 10.1007/s11033-018-4358-6
negative Regulation: MIR21 ---| chemosensitivity 30597867 10.3390/ijms20010103
negative Regulation: MIR21 ---| chemosensitivity 30249919 10.2176/nmc.ra.2018-0141
negative Regulation: MIR21 ---| chemosensitivity 30421325 10.1007/s12192-018-0946-6
negative Regulation: MIR21 ---| chemosensitivity 30600466 10.1007/s10616-018-0291-8
negative Regulation: MIR21 ---| chemosensitivity 30088452 10.2174/1871520618666180808
negative Regulation: MIR21 ---| chemosensitivity 31225740 10.1089/dna.2018.4529
negative Regulation: MIR21 ---| chemosensitivity 31225740 10.1089/dna.2018.4529

negative Regulation: MIR21 ---| chemosensitivity 31225740 10.1089/dna.2018.4529
negative Regulation: MIR21 ---| chemosensitivity 31621692 10.12659/MSM.917082
negative Regulation: MIR21 ---| chemosensitivity 32098295 10.3390/ijms21041495
negative Regulation: MIR21 ---| chemosensitivity 32811810 10.1038/s41419-020-02821-2
negative Regulation: MIR21 ---| chemosensitivity 33000186 10.3892/mmr.2020.11447
negative Regulation: MIR21 ---| chemosensitivity 33112535 10.31557/APJCP.2020.21.10.2
negative Regulation: MIR21 ---| chemosensitivity 33650661 10.3892/or.2021.7925
negative Regulation: MIR21 ---| chemosensitivity 33481665 10.1089/dna.2020.6087
negative Regulation: MIR21 ---| chemosensitivity 34088891 10.1038/s41419-021-03803-8
negative Regulation: MIR21 ---| chemosensitivity 22753745
negative Regulation: MIR21 ---| chemosensitivity 22249446 10.1007/s11033-011-1350-9
negative Regulation: MIR21 ---| chemosensitivity 23321165
negative Regulation: MIR21 ---| chemosensitivity 20670480 10.1179/102453310X126470836
negative Regulation: MIR21 ---| chemosensitivity 20670480 10.1179/102453310X126470836
negative Regulation: MIR21 ---| chemosensitivity 21139417 10.4161/cbt.10.12.14252
negative Regulation: MIR21 ---| chemosensitivity 21468550 10.3892/or.2011.1245
negative Regulation: MIR21 ---| chemosensitivity 21468550 10.3892/or.2011.1245
negative Regulation: MIR21 ---| chemosensitivity 21468550 10.3892/or.2011.1245
negative Regulation: MIR21 ---| chemosensitivity 21468550 10.3892/or.2011.1245
negative Regulation: MIR21 ---| chemosensitivity 20143188 10.1007/s12032-009-9413-7
negative Regulation: MIR21 ---| chemosensitivity
negative Regulation: MIR21 ---| chemosensitivity 21685938 10.1038/onc.2011.222
negative Regulation: MIR21 ---| chemosensitivity 23527297 10.1371/journal.pone.006003
negative Regulation: MIR21 ---| chemosensitivity 24324076
negative Regulation: MIR21 ---| chemosensitivity 24899890 10.1155/2014/386561
negative Regulation: MIR21 ---| chemosensitivity 24696266 10.1007/s12035-014-8679-8
negative Regulation: MIR21 ---| chemosensitivity 25756509 10.1080/15384047.2014.10012
negative Regulation: MIR21 ---| chemosensitivity 26035292 10.3892/ijo.2015.3026
negative Regulation: MIR21 ---| chemosensitivity 26116372 10.1186/s12967-015-0570-5
negative Regulation: MIR21 ---| chemosensitivity 20113523 10.1186/1471-2407-10-2710.1
negative Regulation: MIR21 ---| chemosensitivity 21685938 10.1038/onc.2011.22210.1038
negative Regulation: MIR21 ---| chemosensitivity 21820606 10.1016/j.arcmed.2011.06.00
negative Regulation: MIR21 ---| chemosensitivity 22753745
negative Regulation: MIR21 ---| chemosensitivity 23564788
negative Regulation: MIR21 ---| chemosensitivity 24606718 10.1186/1476-4598-13-5210.1
negative Regulation: MIR21 ---| chemosensitivity 24961346 10.1007/s13277-014-2068-910
negative Regulation: MIR21 ---| chemosensitivity 27179559 10.1016/j.clinre.2016.02.01
negative Regulation: MIR21 ---| chemosensitivity 28714373 10.1177/101042831770737210.
negative Regulation: MIR21 ---| chemosensitivity 24609942 10.1007/s11010-014-1976-810
negative Regulation: MIR21 ---| chemosensitivity 24609942 10.1007/s11010-014-1976-810
negative Regulation: MIR21 ---| chemosensitivity 25232387
negative Regulation: MIR21 ---| chemosensitivity 1087 10.1016/j.trecan.2016.12.00
negative Regulation: MIR21 ---| chemosensitivity 10.1016/j.acthis.2011.11.00
negative Regulation: MIR21 ---| chemosensitivity 10.1016/j.acthis.2011.11.00
negative Regulation: MIR21 ---| chemosensitivity 10.3390/ijms20010103
negative Regulation: MIR21 ---| chemosensitivity 10.1007/s10616-018-0291-8
negative Regulation: MIR21 ---| chemosensitivity 10.1089/dna.2018.4529
negative Regulation: MIR21 ---| chemosensitivity 10.1089/dna.2018.4529
negative Regulation: MIR21 ---| chemosensitivity 10.1089/dna.2018.4529
negative Regulation: MIR21 ---| chemosensitivity 10.12659/MSM.917082
negative Regulation: MIR21 ---| chemosensitivity 23276982
negative Regulation: MIR21 ---| chemosensitivity 10.2174/1389450111314100001
negative Regulation: MIR21 ---| chemosensitivity 23834156 10.2174/1389450111314999018
negative Regulation: MIR21 ---| chemosensitivity 20711171 10.1038/modpathol.2010.135

negative Regulation: MIR21 ---| chemosensitivity 10.2174/1566524014666140603
negative Regulation: MIR21 ---| chemosensitivity 25845681 10.3892/or.2015.3891
negative Regulation: MIR21 ---| chemosensitivity 26116372 10.1186/s12967-015-0570-5
negative Regulation: MIR21 ---| chemosensitivity 10.1097/CCO.000000000000023
negative Regulation: MIR21 ---| chemosensitivity 27021436 10.1038/ncomms11150
negative Regulation: MIR21 ---| chemosensitivity 21820606 10.1016/j.arcmed.2011.06.00
negative Regulation: MIR21 ---| chemosensitivity 31033015 10.1002/jcb.2874710.1002/jc
negative Regulation: MIR21 ---| chemosensitivity 31033015 10.1002/jcb.2874710.1002/jc
negative Regulation: MIR21 ---| chemosensitivity 23834154
negative Regulation: MIR21 ---| chemosensitivity 24472409 10.1016/j.ygyno.2014.01.034
negative Regulation: MIR21 ---| chemosensitivity 28686971 10.1016/j.biopha.2017.06.08
negative Regulation: MIR21 ---| chemosensitivity 28714373 10.1177/101042831770737210.
negative Regulation: MIR21 ---| chemosensitivity 10.3892/or.2021.7925
negative Regulation: MIR21 ---| chemosensitivity 10.1016/j.semcdb.2021.04.00
negative Regulation: MIR21 ---| chemosensitivity 34453645 10.1007/s11010-021-04249-4
negative Regulation: MIR21 ---| chemosensitivity 10.1615/CritRevEukaryotGene
negative Regulation: MIR21 ---| chemosensitivity 34145425 10.1038/s41417-021-00356-y
negative Regulation: MIR21 ---| chemosensitivity 36083475 10.19852/j.cnki.jtcm.2022.0
negative Regulation: MIR21 ---| chemosensitivity 36293478 10.3390/ijms232012625
negative Regulation: WNT10A ---| chemosensitivity 23094073 10.1371/journal.pone.004764
negative Regulation: WNT10A ---| chemosensitivity 23094073 10.1371/journal.pone.004764
negative Regulation: FCHSD2 ---| chemosensitivity 22902056 10.1016/j.leukres.2012.06.0
negative Regulation: AURKA ---| chemosensitivity 28242198 10.1016/j.bbrc.2017.02.120
negative Regulation: AURKA ---| chemosensitivity 28242198 10.1016/j.bbrc.2017.02.120
negative Regulation: AURKA ---| chemosensitivity 18160664 10.1182/blood-2007-07-09932
negative Regulation: AURKA ---| chemosensitivity 27461831 10.1016/j.prp.2016.05.004
negative Regulation: AURKA ---| chemosensitivity 31362850 10.1016/j.tcb.2019.06.005
negative Regulation: AURKA ---| chemosensitivity 19157502 10.1016/j.humpath.2008.10.0
negative Regulation: AURKA ---| chemosensitivity 20598352 10.1016/j.acthis.2010.05.00
negative Regulation: AURKA ---| chemosensitivity 20598352 10.1016/j.acthis.2010.05.00
negative Regulation: AURKA ---| chemosensitivity 24480319 10.1016/j.bbcan.2014.01.007
negative Regulation: AURKA ---| chemosensitivity 24613085 10.1016/j.jnutbio.2014.01.0
negative Regulation: AURKA ---| chemosensitivity 24613085 10.1016/j.jnutbio.2014.01.0
negative Regulation: AURKA ---| chemosensitivity 27138904 10.1016/j.yexcr.2016.04.012
negative Regulation: AURKA ---| chemosensitivity 25987188 10.1186/s12943-015-0375-4
negative Regulation: AURKA ---| chemosensitivity 28167680 10.1242/jcs.196790
negative Regulation: AURKA ---| chemosensitivity 27396604 10.2174/1568009616666160630
negative Regulation: AURKA ---| chemosensitivity 28849180 10.3892/or.2017.5908
negative Regulation: AURKA ---| chemosensitivity 29333101 10.7150/ijms.22513
negative Regulation: AURKA ---| chemosensitivity 29367628 10.1038/s41419-017-0137-x
negative Regulation: AURKA ---| chemosensitivity 28786875 10.1097/IGC.000000000000108
negative Regulation: AURKA ---| chemosensitivity 31647033 10.1186/s12931-019-1194-8
negative Regulation: AURKA ---| chemosensitivity 32576205 10.1186/s12964-020-00591-0
negative Regulation: AURKA ---| chemosensitivity 23037716 10.1038/bjc.2012.450
negative Regulation: AURKA ---| chemosensitivity 23348568 10.1038/cdd.2012.169
negative Regulation: AURKA ---| chemosensitivity 23474708 10.1038/aps.2012.197
negative Regulation: AURKA ---| chemosensitivity 22281755 10.1038/cgt.2011.89
negative Regulation: AURKA ---| chemosensitivity 25625960 10.3892/ijo.2015.2842
negative Regulation: AURKA ---| chemosensitivity 25625960 10.3892/ijo.2015.2842
negative Regulation: AURKA ---| chemosensitivity 25889801 10.1186/s12943-015-0348-7
negative Regulation: AURKA ---| chemosensitivity
negative Regulation: AURKA ---| chemosensitivity 27082306 10.1080/15384047.2016.11776
negative Regulation: AURKA ---| chemosensitivity 17317845 10.1158/1078-0432.CCR-06-11
negative Regulation: AURKA ---| chemosensitivity 17317845 10.1158/1078-0432.CCR-06-11

negative Regulation: AURKA ---| chemosensitivity 19671766 10.1158/1535-7163.MCT-08-08
negative Regulation: AURKA ---| chemosensitivity 19912186 10.1111/j.1464-410X.2009.09
negative Regulation: AURKA ---| chemosensitivity 20232424 10.1002/pbc.2246510.1002/pb
negative Regulation: AURKA ---| chemosensitivity 22281755 10.1038/cgt.2011.8910.1038/
negative Regulation: AURKA ---| chemosensitivity 22848254 10.3892/ol.2011.295
negative Regulation: AURKA ---| chemosensitivity 22848254 10.3892/ol.2011.295
negative Regulation: AURKA ---| chemosensitivity 24277377 10.1007/s13277-013-1393-810
negative Regulation: AURKA ---| chemosensitivity 24613085 10.1016/j.jnutbio.2014.01.0
negative Regulation: AURKA ---| chemosensitivity 25428915 10.18632/oncotarget.2682
negative Regulation: AURKA ---| chemosensitivity 25625960 10.3892/ijo.2015.284210.389
negative Regulation: AURKA ---| chemosensitivity 27396604 10.2174/1568009616666160630
negative Regulation: AURKA ---| chemosensitivity 29207686 10.18632/oncotarget.2061010
negative Regulation: AURKA ---| chemosensitivity 30431113 10.3892/or.2018.6858
negative Regulation: AURKA ---| chemosensitivity 26045992
negative Regulation: AURKA ---| chemosensitivity 35216255 10.3390/ijms23042144
negative Regulation: AURKA ---| chemosensitivity 35428744 10.12659/MSM.934799
negative Regulation: AURKA ---| chemosensitivity 35917645 10.1016/j.tranon.2022.10149
negative Regulation: CASC9 ---| chemosensitivity 28146436 10.18632/oncotarget.1487110
negative Regulation: EPHA8 ---| chemosensitivity 34278497 10.3892/OR.2021.8134
negative Regulation: EPHA8 ---| chemosensitivity 34278497 10.3892/or.2021.813410.3892
negative Regulation: SNHG12 ---| chemosensitivity 31743769 10.1016/j.gene.2019.144145
negative Regulation: MIR224 ---| chemosensitivity 28272374 10.3390/molecules22030417
negative Regulation: MIR224 ---| chemosensitivity 28079894 10.1038/cddis.2016.461
negative Regulation: MIR224 ---| chemosensitivity 24921914 10.1038/bjc.2014.157
negative Regulation: MIR224 ---| chemosensitivity 25919696 10.1038/bjc.2015.125
negative Regulation: MIR224 ---| chemosensitivity 25919696 10.1038/bjc.2015.125
negative Regulation: MIR224 ---| chemosensitivity 25919696 10.1038/bjc.2015.12510.1038
negative Regulation: MIR224 ---| chemosensitivity 10.3892/or.2018.6929
negative Regulation: MIR224 ---| chemosensitivity 10.3892/or.2018.6929
negative Regulation: MIR224 ---| chemosensitivity 10.3892/or.2018.6929
negative Regulation: HNRNPA2B1 ---| chemosensitivit28351333 10.1177/1010428317694318
negative Regulation: HNRNPA2B1 ---| chemosensitivit23525071 10.1097/CAD.0b013e3283608bc
negative Regulation: HNRNPA2B1 ---| chemosensitivit23525071 10.1097/CAD.0b013e3283608bc
negative Regulation: ATG2B ---| chemosensitivity 33824300 10.1038/s41419-021-03650-7
negative Regulation: BCAR4 ---| chemosensitivity 36627684 10.1186/s13046-022-02588-8
negative Regulation: RPRD1A ---| chemosensitivity 34921137 10.1038/s41419-021-04447-4
negative Regulation: APOBEC3B ---| chemosensitivity30226610 10.3892/or.2018.669810.3892
negative Regulation: ABCG2 ---| chemosensitivity 21219875 10.1016/j.bcp.2010.12.018
negative Regulation: ABCG2 ---| chemosensitivity 16844360 10.1016/j.ejps.2006.06.001
negative Regulation: ABCG2 ---| chemosensitivity 23743354 10.1016/j.canlet.2013.05.03
negative Regulation: ABCG2 ---| chemosensitivity 27573902 10.1007/978-3-319-42059-2_1
negative Regulation: ABCG2 ---| chemosensitivity 28374336 10.1007/s13318-017-0411-4
negative Regulation: ABCG2 ---| chemosensitivity 28942499 10.1007/s00432-017-2515-2
negative Regulation: ABCG2 ---| chemosensitivity 30617282 10.1038/s12276-018-0197-8
negative Regulation: ABCG2 ---| chemosensitivity 32565864 10.1155/2020/5062942
negative Regulation: ABCG2 ---| chemosensitivity 32091329 10.2174/1381612826666200224
negative Regulation: ABCG2 ---| chemosensitivity 17016657
negative Regulation: ABCG2 ---| chemosensitivity 22460269 10.1038/bjc.2012.81
negative Regulation: ABCG2 ---| chemosensitivity 18248513 10.1111/j.1742-7843.2007.00
negative Regulation: ABCG2 ---| chemosensitivity 27012188 10.1016/j.canlet.2016.03.03
negative Regulation: ABCG2 ---| chemosensitivity 27293996
negative Regulation: ABCG2 ---| chemosensitivity 27671528 10.1158/1535-7163.MCT-15-07
negative Regulation: ABCG2 ---| chemosensitivity 30617282 10.1038/s12276-018-0197-810
negative Regulation: ABCG2 ---| chemosensitivity 34381520 10.1155/2021/3905367

negative Regulation: ABCG2 ---| chemosensitivity	34498146	10.1007/s00432-021-03793-2
negative Regulation: ABCG2 ---| chemosensitivity	34830383	10.3390/ijms22221250210.339
negative Regulation: ABCG2 ---| chemosensitivity	34830383	10.3390/ijms22221250210.339
negative Regulation: SPA17 ---| chemosensitivity	19744347	
negative Regulation: SPA17 ---| chemosensitivity	19744347	
negative Regulation: SPA17 ---| chemosensitivity	19744347	10.1186/1471-2407-9-32310.1
negative Regulation: SPA17 ---| chemosensitivity	19744347	10.1186/1471-2407-9-32310.1
negative Regulation: CA1 ---| chemosensitivity	32668392	10.1016/j.omtn.2020.06.016
negative Regulation: MAPK9 ---| chemosensitivity	27313779	10.7150/jca.1450610.7150/jc
negative Regulation: NCK1-DT ---| chemosensitivity	31570280	10.1016/j.prp.2019.152653
negative Regulation: NCK1-DT ---| chemosensitivity	32858372	10.1016/j.prp.2020.153157
negative Regulation: NCK1-DT ---| chemosensitivity	31972361	10.1016/j.biopha.2019.10974
negative Regulation: NCK1-DT ---| chemosensitivity	31257479	10.3892/ijmm.2019.4261
negative Regulation: NCK1-DT ---| chemosensitivity	31371629	10.1042/BSR20191226
negative Regulation: NCK1-DT ---| chemosensitivity	30221354	10.1002/jcp.2719810.1002/jc
negative Regulation: MAPK8 ---| chemosensitivity	27313779	10.7150/jca.1450610.7150/jc
negative Regulation: ADM ---| chemosensitivity	22294191	10.3892/or.2012.1655
negative Regulation: ADM ---| chemosensitivity	22294191	10.3892/or.2012.1655
negative Regulation: ADM ---| chemosensitivity	22294191	10.3892/or.2012.1655
negative Regulation: ADM ---| chemosensitivity	22294191	10.3892/or.2012.165510.3892
negative Regulation: TAGLN ---| chemosensitivity	32819569	10.1016/j.bbrc.2020.06.066
negative Regulation: TAGLN ---| chemosensitivity	32819569	10.1016/j.bbrc.2020.06.066
negative Regulation: TAGLN ---| chemosensitivity	32819569	10.1016/j.bbrc.2020.06.066
negative Regulation: TAGLN ---| chemosensitivity	32819569	10.1016/j.bbrc.2020.06.066
negative Regulation: TAGLN ---| chemosensitivity	32819569	10.1016/j.bbrc.2020.06.066
negative Regulation: TAGLN ---| chemosensitivity	32819569	10.1016/j.bbrc.2020.06.066
negative Regulation: TAGLN ---| chemosensitivity	32819569	10.1016/j.bbrc.2020.06.0661
negative Regulation: TAGLN ---| chemosensitivity	33478719	10.1016/j.bbrc.2021.01.0041
negative Regulation: SRI ---| chemosensitivity	30144438	10.1016/j.cca.2018.08.034
negative Regulation: SRI ---| chemosensitivity	27261590	10.1016/j.biopha.2016.04.00
negative Regulation: SRI ---| chemosensitivity	16859119	
negative Regulation: SRI ---| chemosensitivity	26045737	
negative Regulation: PIN1 ---| chemosensitivity	28598431	10.1038/ncomms15772
negative Regulation: PIN1 ---| chemosensitivity	30684192	10.1007/s12272-019-01122-3
negative Regulation: PIN1 ---| chemosensitivity	28598431	10.1038/ncomms1577210.1038/
negative Regulation: ATG5 ---| chemosensitivity	30862487	10.1016/j.canlet.2019.02.05
negative Regulation: ATG5 ---| chemosensitivity	32735773	10.1016/j.ymthe.2020.07.019
negative Regulation: ATG5 ---| chemosensitivity	30569180	10.3892/or.2018.6929
negative Regulation: ATG5 ---| chemosensitivity	30569180	10.3892/or.2018.6929
negative Regulation: ATG5 ---| chemosensitivity	30569180	10.3892/or.2018.6929
negative Regulation: ATG5 ---| chemosensitivity	30569180	10.3892/or.2018.6929
negative Regulation: ATG5 ---| chemosensitivity	32735773	10.1016/j.ymthe.2020.07.019
negative Regulation: ATG5 ---| chemosensitivity	32735773	10.1016/j.ymthe.2020.07.019
negative Regulation: ATG5 ---| chemosensitivity	30569180	10.3892/or.2018.692910.3892
negative Regulation: CDK2 ---| chemosensitivity	33555529	10.1007/s11033-021-06193-4
negative Regulation: CDK2 ---| chemosensitivity	24970653	10.3892/ijo.2014.2523
negative Regulation: CDK2 ---| chemosensitivity	24970653	10.3892/ijo.2014.252310.389
negative Regulation: FAIM2 ---| chemosensitivity	30617187	10.1042/CS20180814
negative Regulation: HMGN5 ---| chemosensitivity	29710817	10.3390/ijms19051320
negative Regulation: HMGN5 ---| chemosensitivity	31936777	10.3390/ijms21020449
negative Regulation: HMGN5 ---| chemosensitivity	24664583	10.1007/s13277-014-1833-010
negative Regulation: HMGN5 ---| chemosensitivity	26315299	10.3892/ijo.2015.313110.389
negative Regulation: HMGN5 ---| chemosensitivity	26315299	10.3892/ijo.2015.313110.389
negative Regulation: HMGN5 ---| chemosensitivity	29163683	10.3892/ol.2017.704510.3892

negative Regulation: HMGN5 ---| chemosensitivity	29163683	10.3892/ol.2017.704510.3892
negative Regulation: MIR487A ---| chemosensitivity	30194558	10.1007/s11033-018-4358-6
negative Regulation: MDM4 ---| chemosensitivity		10.31083/j.ceog4902054
negative Regulation: MDM4 ---| chemosensitivity		10.31083/j.ceog4902054
negative Regulation: PLAT ---| chemosensitivity	30458112	10.1096/fj.201801339RRR10.1
negative Regulation: H19 ---| chemosensitivity	30244121	10.1016/j.taap.2018.09.018
negative Regulation: H19 ---| chemosensitivity	32736275	10.1016/j.ijporl.2020.11024
negative Regulation: H19 ---| chemosensitivity	30244121	10.1016/j.taap.2018.09.0181
negative Regulation: H19 ---| chemosensitivity	30519041	10.2147/OTT.S17237910.2147/
negative Regulation: H19 ---| chemosensitivity	30843379	10.1002/cam4.186010.1002/ca
negative Regulation: H19 ---| chemosensitivity	32736275	10.1016/j.ijporl.2020.11024
negative Regulation: HSPA9 ---| chemosensitivity	30776464	10.1016/j.gene.2019.02.033
negative Regulation: CCAR2 ---| chemosensitivity	34021129	10.1038/s41419-021-03798-2
negative Regulation: IGF1R ---| chemosensitivity	28433634	10.1016/j.bbrc.2017.04.100
negative Regulation: IGF1R ---| chemosensitivity	28433634	10.1016/j.bbrc.2017.04.100
negative Regulation: IGF1R ---| chemosensitivity	33945846	10.1016/j.bbcan.2021.188557
negative Regulation: IGF1R ---| chemosensitivity	32887025	10.1016/j.biopha.2020.11039
negative Regulation: IGF1R ---| chemosensitivity	21782884	10.1016/j.mce.2011.07.002
negative Regulation: IGF1R ---| chemosensitivity	17506723	
negative Regulation: IGF1R ---| chemosensitivity	20543858	
negative Regulation: IGF1R ---| chemosensitivity	19603014	
negative Regulation: IGF1R ---| chemosensitivity	27405474	10.1186/s12885-016-2437-4
negative Regulation: IGF1R ---| chemosensitivity	28051262	
negative Regulation: IGF1R ---| chemosensitivity	28627639	10.3892/mmr.2017.6788
negative Regulation: IGF1R ---| chemosensitivity	29393438	10.3892/mmr.2018.8497
negative Regulation: IGF1R ---| chemosensitivity	29273680	10.1530/JME-17-0250
negative Regulation: IGF1R ---| chemosensitivity	29273680	10.1530/JME-17-0250
negative Regulation: IGF1R ---| chemosensitivity	31230061	10.12659/MSM.915503
negative Regulation: IGF1R ---| chemosensitivity	32341579	10.1038/s41591-020-0821-8
negative Regulation: IGF1R ---| chemosensitivity	20376536	10.1007/s11010-010-0453-2
negative Regulation: IGF1R ---| chemosensitivity	20376536	10.1007/s11010-010-0453-2
negative Regulation: IGF1R ---| chemosensitivity	20376536	10.1007/s11010-010-0453-2
negative Regulation: IGF1R ---| chemosensitivity	23574723	10.4161/cc.24477
negative Regulation: IGF1R ---| chemosensitivity		10.2174/18715206113130300003
negative Regulation: IGF1R ---| chemosensitivity	20543858	10.1038/onc.2010.205
negative Regulation: IGF1R ---| chemosensitivity	24103397	10.1186/1757-2215-6-71
negative Regulation: IGF1R ---| chemosensitivity	23990456	10.1007/s13277-013-1041-3
negative Regulation: IGF1R ---| chemosensitivity	23990456	10.1007/s13277-013-1041-3
negative Regulation: IGF1R ---| chemosensitivity	24960521	10.1038/clpt.2014.136
negative Regulation: IGF1R ---| chemosensitivity	12209582	10.1002/ijc.10537
negative Regulation: IGF1R ---| chemosensitivity	17446062	10.1016/j.ejca.2007.03.003
negative Regulation: IGF1R ---| chemosensitivity	17908353	10.1017/S0022215107000606
negative Regulation: IGF1R ---| chemosensitivity	18535748	10.1111/j.1745-7270.2008.00
negative Regulation: IGF1R ---| chemosensitivity	18535748	10.1111/j.1745-7270.2008.00
negative Regulation: IGF1R ---| chemosensitivity	20376536	10.1007/s11010-010-0453-210
negative Regulation: IGF1R ---| chemosensitivity	20451373	10.1016/j.ejca.2010.03.0381
negative Regulation: IGF1R ---| chemosensitivity	21442237	10.1007/s11010-011-0790-910
negative Regulation: IGF1R ---| chemosensitivity	22165963	10.2174/156800912798888974
negative Regulation: IGF1R ---| chemosensitivity	22275271	10.1002/jcb.2408010.1002/jc
negative Regulation: IGF1R ---| chemosensitivity	23589145	10.1007/s10620-013-2673-210
negative Regulation: IGF1R ---| chemosensitivity	27405474	10.1186/s12885-016-2437-410
negative Regulation: IGF1R ---| chemosensitivity		10.1530/JME-17-0250
negative Regulation: IGF1R ---| chemosensitivity		10.1530/JME-17-0250
negative Regulation: IGF1R ---| chemosensitivity	23574723	10.4161/cc.24477

negative Regulation: IGF1R ---| chemosensitivity 35102541 10.14670/HH-18-425
negative Regulation: IGF1R ---| chemosensitivity 36361620 10.3390/ijms232112829
negative Regulation: BCL6 ---| chemosensitivity 30420967 10.1155/2018/695350610.1155
negative Regulation: XIST ---| chemosensitivity 29130102 10.3892/or.2017.6056
negative Regulation: XIST ---| chemosensitivity 28961027 10.1080/15384101.2017.13610
negative Regulation: XIST ---| chemosensitivity 28961027 10.1080/15384101.2017.13610
negative Regulation: XIST ---| chemosensitivity 31646589 10.26355/eurrev_201910_1917
negative Regulation: XIST ---| chemosensitivity 31659146 10.12659/MSM.916075
negative Regulation: XIST ---| chemosensitivity 31659146 10.12659/MSM.916075
negative Regulation: XIST ---| chemosensitivity 31659146 10.12659/MSM.916075
negative Regulation: XIST ---| chemosensitivity 31659146 10.12659/MSM.916075
negative Regulation: XIST ---| chemosensitivity 31659146 10.12659/MSM.916075
negative Regulation: XIST ---| chemosensitivity 31659146 10.12659/MSM.916075
negative Regulation: XIST ---| chemosensitivity 33191714
negative Regulation: XIST ---| chemosensitivity 30207107 10.3802/jgo.2018.29.e9910.3
negative Regulation: XIST ---| chemosensitivity 32209729 10.18632/aging.10267310.186
negative Regulation: XIST ---| chemosensitivity 34930117 10.1186/s10020-021-00421-0
negative Regulation: XIST ---| chemosensitivity 34930117 10.1186/s10020-021-00421-0
negative Regulation: XIST ---| chemosensitivity 34930117 10.1186/s10020-021-00421-0
negative Regulation: CIRBP ---| chemosensitivity 19900510 10.1016/j.bbcan.2009.11.001
negative Regulation: CIRBP ---| chemosensitivity 26188505 10.1016/j.bbrc.2015.07.066
negative Regulation: CIRBP ---| chemosensitivity 29849797 10.3892/ol.2018.833810.3892
negative Regulation: CIRBP ---| chemosensitivity 35541895 10.7150/ijbs.69655
negative Regulation: CD274 ---| chemosensitivity 26806807 10.1016/j.canlet.2015.12.00
negative Regulation: CD274 ---| chemosensitivity 30172884 10.1016/j.tranon.2018.08.00
negative Regulation: CD274 ---| chemosensitivity 33073683 10.1177/172460082092617210.
negative Regulation: CD274 ---| chemosensitivity 33030957 10.4149/neo_2020_200531N589
negative Regulation: G6PD ---| chemosensitivity 35213227 10.1126/sciadv.abf909610.11
negative Regulation: G6PD ---| chemosensitivity 35597872 10.1038/s41416-022-01843-1
negative Regulation: TMEM98 ---| chemosensitivity 24608572 10.1158/1535-7163.MCT-13-08
negative Regulation: UGCG ---| chemosensitivity 33887691 10.1016/j.biopha.2021.11156
negative Regulation: UGCG ---| chemosensitivity 23133636 10.1371/journal.pone.004849
negative Regulation: MALAT1 ---| chemosensitivity 28034748 10.1016/j.bbrc.2016.12.167
negative Regulation: MALAT1 ---| chemosensitivity 28034748 10.1016/j.bbrc.2016.12.167
negative Regulation: MALAT1 ---| chemosensitivity 32423796 10.1016/j.bbrc.2020.03.170
negative Regulation: MALAT1 ---| chemosensitivity 31926239 10.1016/j.lfs.2020.117280
negative Regulation: MALAT1 ---| chemosensitivity 29505924 10.1016/j.biopha.2018.02.13
negative Regulation: MALAT1 ---| chemosensitivity 30970520 10.1016/j.biopha.2019.10872
negative Regulation: MALAT1 ---| chemosensitivity 33346133 10.1016/j.semcancer.2020.12
negative Regulation: MALAT1 ---| chemosensitivity 32036249 10.1016/j.omtn.2019.11.035
negative Regulation: MALAT1 ---| chemosensitivity 27376734 10.1007/978-981-10-1498-7_5
negative Regulation: MALAT1 ---| chemosensitivity 28394318 10.1038/labinvest.2017.41
negative Regulation: MALAT1 ---| chemosensitivity 29168767 10.3390/ijms18122505
negative Regulation: MALAT1 ---| chemosensitivity 31371629 10.1042/BSR20191226
negative Regulation: MALAT1 ---| chemosensitivity 32141554 10.26355/eurrev_202002_2036
negative Regulation: MALAT1 ---| chemosensitivity 32473641 10.1186/s12957-020-01884-x
negative Regulation: MALAT1 ---| chemosensitivity 32623440 10.12659/MSM.922672
negative Regulation: MALAT1 ---| chemosensitivity 32623440 10.12659/MSM.922672
negative Regulation: MALAT1 ---| chemosensitivity 32623440 10.12659/MSM.922672
negative Regulation: MALAT1 ---| chemosensitivity 33151424 10.1007/s00404-020-05858-y
negative Regulation: MALAT1 ---| chemosensitivity 32748748 10.2174/156800962099920073C
negative Regulation: MALAT1 ---| chemosensitivity 25811929 10.3390/ijms16046677
negative Regulation: MALAT1 ---| chemosensitivity 25811929 10.3390/ijms16046677
negative Regulation: MALAT1 ---| chemosensitivity 25811929 10.3390/ijms1604667710.3390

negative Regulation: MALAT1 ---|	chemosensitivity	30970520	10.1016/j.biopha.2019.10872
negative Regulation: MALAT1 ---|	chemosensitivity	31037832	10.1002/2211-5463.1264910.1
negative Regulation: MALAT1 ---|	chemosensitivity	31116509	10.1002/2211-5463.1267610.1
negative Regulation: MALAT1 ---|	chemosensitivity	34036905	10.2174/0929867328666210521
negative Regulation: MALAT1 ---|	chemosensitivity	35706002	10.1186/s12957-022-02655-6
negative Regulation: MALAT1 ---|	chemosensitivity	36229883	10.1186/s12935-022-02736-2
negative Regulation: PIM3 ---|	chemosensitivity	27016481	10.1016/j.bbrc.2016.03.099
negative Regulation: PIM3 ---|	chemosensitivity	27525970	10.1016/j.biopha.2016.08.02
negative Regulation: TMSB4X ---|	chemosensitivity	19251410	10.1016/j.suronc.2009.01.00
negative Regulation: BIRC7 ---|	chemosensitivity	20171199	10.1016/j.clinbiochem.2010.
negative Regulation: BIRC7 ---|	chemosensitivity	27448305	10.1007/s13277-016-5157-0
negative Regulation: BIRC7 ---|	chemosensitivity	27448305	10.1007/s13277-016-5157-0
negative Regulation: BIRC7 ---|	chemosensitivity	27448305	10.1007/s13277-016-5157-0
negative Regulation: BIRC7 ---|	chemosensitivity	27448305	10.1007/s13277-016-5157-0
negative Regulation: BIRC7 ---|	chemosensitivity	27448305	10.1007/s13277-016-5157-0
negative Regulation: BIRC7 ---|	chemosensitivity	27448305	10.1007/s13277-016-5157-0
negative Regulation: BIRC7 ---|	chemosensitivity	27448305	10.1007/s13277-016-5157-0
negative Regulation: BIRC7 ---|	chemosensitivity	27448305	10.1007/s13277-016-5157-0
negative Regulation: BIRC7 ---|	chemosensitivity	27448305	10.1007/s13277-016-5157-0
negative Regulation: BIRC7 ---|	chemosensitivity	28440463	10.3892/or.2017.5584
negative Regulation: BIRC7 ---|	chemosensitivity	28440463	10.3892/or.2017.5584
negative Regulation: BIRC7 ---|	chemosensitivity	28440463	10.3892/or.2017.5584
negative Regulation: BIRC7 ---|	chemosensitivity	28440463	10.3892/or.2017.5584
negative Regulation: BIRC7 ---|	chemosensitivity	28765921	10.3892/or.2017.5866
negative Regulation: BIRC7 ---|	chemosensitivity	28765921	10.3892/or.2017.5866
negative Regulation: BIRC7 ---|	chemosensitivity	28765921	10.3892/or.2017.5866
negative Regulation: BIRC7 ---|	chemosensitivity	31777256	10.4149/neo_2019_190106N18
negative Regulation: BIRC7 ---|	chemosensitivity	33649820	10.3892/or.2021.7969
negative Regulation: BIRC7 ---|	chemosensitivity	33649820	10.3892/or.2021.7969
negative Regulation: BIRC7 ---|	chemosensitivity	33649820	10.3892/or.2021.7969
negative Regulation: BIRC7 ---|	chemosensitivity	21883015	10.1089/cbr.2011.0962
negative Regulation: BIRC7 ---|	chemosensitivity	23632777	10.3892/ijo.2013.1925
negative Regulation: BIRC7 ---|	chemosensitivity	23632777	10.3892/ijo.2013.1925
negative Regulation: BIRC7 ---|	chemosensitivity	23632777	10.3892/ijo.2013.1925
negative Regulation: BIRC7 ---|	chemosensitivity	18340356	10.1038/cgt.2008.1610.1038/
negative Regulation: BIRC7 ---|	chemosensitivity	23632777	10.3892/ijo.2013.192510.389
negative Regulation: BIRC7 ---|	chemosensitivity	23632777	10.3892/ijo.2013.192510.389
negative Regulation: BIRC7 ---|	chemosensitivity	25695324	10.3892/mmr.2015.337210.389
negative Regulation: BIRC7 ---|	chemosensitivity	27448305	10.1007/s13277-016-5157-0
negative Regulation: BIRC7 ---|	chemosensitivity	28440463	10.3892/or.2017.558410.3892
negative Regulation: BIRC7 ---|	chemosensitivity	28765921	10.3892/or.2017.586610.3892
negative Regulation: BIRC7 ---|	chemosensitivity	31777256	10.4149/neo_2019_190106N181
negative Regulation: EEF1A1 ---|	chemosensitivity	23799104	10.1371/journal.pone.006643
negative Regulation: EEF1A1 ---|	chemosensitivity	23799104	10.1371/journal.pone.006643
negative Regulation: GGH ---|	chemosensitivity	24045662	10.1038/bjc.2013.57910.1038
negative Regulation: GGH ---|	chemosensitivity	24045662	10.1038/bjc.2013.579
negative Regulation: GGH ---|	chemosensitivity	24045662	10.1038/bjc.2013.57910.1038
negative Regulation: GGH ---|	chemosensitivity	24045662	10.1038/bjc.2013.57910.1038
negative Regulation: GGH ---|	chemosensitivity	24045662	10.1038/bjc.2013.57910.1038
negative Regulation: GGH ---|	chemosensitivity	24045662	10.1038/bjc.2013.57910.1038
negative Regulation: GGH ---|	chemosensitivity	24045662	10.1038/bjc.2013.57910.1038
negative Regulation: GGH ---|	chemosensitivity	24045662	10.1038/bjc.2013.579
negative Regulation: GGH ---|	chemosensitivity	24045662	10.1038/bjc.2013.579
negative Regulation: GGH ---|	chemosensitivity	24045662	10.1038/bjc.2013.579

negative Regulation: GGH ---| chemosensitivity	24045662	10.1038/bjc.2013.579
negative Regulation: GGH ---| chemosensitivity	24045662	10.1038/bjc.2013.579
negative Regulation: GGH ---| chemosensitivity	24045662	10.1038/bjc.2013.579
negative Regulation: GGH ---| chemosensitivity	24045662	10.1038/bjc.2013.579
negative Regulation: GGH ---| chemosensitivity	24045662	10.1038/bjc.2013.579
negative Regulation: GGH ---| chemosensitivity	24045662	10.1038/bjc.2013.579
negative Regulation: GGH ---| chemosensitivity	32256983	10.4162/nrp.2020.14.2.9510.
negative Regulation: MIR744 ---| chemosensitivity		10.1016/j.biopha.2021.11152
negative Regulation: MIR744 ---| chemosensitivity	31553714	10.12659/MSM.919219
negative Regulation: MIR744 ---| chemosensitivity	31884801	10.4149/neo_2019_190508N411
negative Regulation: MIR744 ---| chemosensitivity	33847207	10.1080/15384047.2021.18987
negative Regulation: MIR744 ---| chemosensitivity	31553714	10.12659/MSM.91921910.12659
negative Regulation: CALR ---| chemosensitivity	33591948	10.18632/aging.202488
negative Regulation: KLK11 ---| chemosensitivity	27882085	10.3892/etm.2016.3723
negative Regulation: DYRK1A ---| chemosensitivity	29773344	10.1016/j.ejcb.2018.04.005
negative Regulation: ZBED1 ---| chemosensitivity	31949791	
negative Regulation: ZBED1 ---| chemosensitivity	31949791	
negative Regulation: RNASEH2A ---| chemosensitivity	32509219	
negative Regulation: CHD4 ---| chemosensitivity	26095183	10.1016/j.jhep.2015.06.009
negative Regulation: CHD4 ---| chemosensitivity	26095183	10.1016/j.jhep.2015.06.009
negative Regulation: CLDN2 ---| chemosensitivity	31923533	10.1016/j.bbamcr.2019.11864
negative Regulation: CLDN2 ---| chemosensitivity	29597147	10.1016/j.jnutbio.2018.02.0
negative Regulation: FTCD ---| chemosensitivity	24686083	10.1016/j.cellsig.2014.03.0
negative Regulation: DUT ---| chemosensitivity	15322254	
negative Regulation: LINC01021 ---| chemosensitivit	29262524	10.18632/oncotarget.2224510
negative Regulation: AKT1 ---| chemosensitivity	27038543	10.1016/j.bbrc.2016.03.141
negative Regulation: AKT1 ---| chemosensitivity	27666600	10.1016/j.bcp.2016.09.022
negative Regulation: AKT1 ---| chemosensitivity	33011222	10.1016/j.lfs.2020.118513
negative Regulation: AKT1 ---| chemosensitivity	26276725	10.1016/j.canlet.2015.08.00
negative Regulation: AKT1 ---| chemosensitivity	29753186	10.1016/j.tranon.2018.04.01
negative Regulation: AKT1 ---| chemosensitivity	22387880	10.1016/j.regpep.2012.02.00
negative Regulation: AKT1 ---| chemosensitivity	20079806	10.1016/j.bbcan.2010.01.002
negative Regulation: AKT1 ---| chemosensitivity	22208970	10.1016/j.ijrobp.2011.09.04
negative Regulation: AKT1 ---| chemosensitivity	19651067	10.1016/j.jamcollsurg.2009.
negative Regulation: AKT1 ---| chemosensitivity	24184201	10.1016/j.biopha.2013.10.00
negative Regulation: AKT1 ---| chemosensitivity	21108789	
negative Regulation: AKT1 ---| chemosensitivity	19078992	
negative Regulation: AKT1 ---| chemosensitivity	15150572	
negative Regulation: AKT1 ---| chemosensitivity	12373610	
negative Regulation: AKT1 ---| chemosensitivity	27473470	10.1007/s10571-016-0410-z
negative Regulation: AKT1 ---| chemosensitivity	28440463	10.3892/or.2017.5584
negative Regulation: AKT1 ---| chemosensitivity	28849180	10.3892/or.2017.5908
negative Regulation: AKT1 ---| chemosensitivity	28887606	10.1007/s10616-017-0134-z
negative Regulation: AKT1 ---| chemosensitivity	30594239	10.1186/s40659-018-0205-4
negative Regulation: AKT1 ---| chemosensitivity		
negative Regulation: AKT1 ---| chemosensitivity	31665911	10.1177/0963689719885083
negative Regulation: AKT1 ---| chemosensitivity	31665911	10.1177/0963689719885083
negative Regulation: AKT1 ---| chemosensitivity	32945500	10.3892/or.2020.7710
negative Regulation: AKT1 ---| chemosensitivity	33499874	10.1186/s13046-021-01854-5
negative Regulation: AKT1 ---| chemosensitivity	33591948	10.18632/aging.202488
negative Regulation: AKT1 ---| chemosensitivity	22873219	
negative Regulation: AKT1 ---| chemosensitivity	22873219	
negative Regulation: AKT1 ---| chemosensitivity	20878074	10.3892/ijo-00000778
negative Regulation: AKT1 ---| chemosensitivity	21444628	10.1124/jpet.111.179663

negative Regulation: AKT1 ---| chemosensitivity	22312312	10.3390/ijms13011186
negative Regulation: AKT1 ---| chemosensitivity	21108789	10.1186/1476-4598-9-305
negative Regulation: AKT1 ---| chemosensitivity	23743572	10.3892/or.2013.2520
negative Regulation: AKT1 ---| chemosensitivity	23743572	10.3892/or.2013.2520
negative Regulation: AKT1 ---| chemosensitivity	23743572	10.3892/or.2013.2520
negative Regulation: AKT1 ---| chemosensitivity	23613505	10.1177/0300060513480920
negative Regulation: AKT1 ---| chemosensitivity	24232099	10.1038/cddis.2013.450
negative Regulation: AKT1 ---| chemosensitivity	24232099	10.1038/cddis.2013.450
negative Regulation: AKT1 ---| chemosensitivity	24255131	10.1074/mcp.M113.034025
negative Regulation: AKT1 ---| chemosensitivity	24454800	10.1371/journal.pone.008511
negative Regulation: AKT1 ---| chemosensitivity	24104394	10.1038/leu.2013.291
negative Regulation: AKT1 ---| chemosensitivity	24784001	10.1016/j.neo.2014.03.008
negative Regulation: AKT1 ---| chemosensitivity	24722292	10.1038/cddis.2014.136
negative Regulation: AKT1 ---| chemosensitivity	25230779	10.3892/or.2014.3488
negative Regulation: AKT1 ---| chemosensitivity	25230779	10.3892/or.2014.3488
negative Regulation: AKT1 ---| chemosensitivity	21273759	10.1159/000322986
negative Regulation: AKT1 ---| chemosensitivity	21597260	10.1159/000322849
negative Regulation: AKT1 ---| chemosensitivity	26820593	10.3892/or.2016.4590
negative Regulation: AKT1 ---| chemosensitivity	27220401	10.3892/or.2016.4820
negative Regulation: AKT1 ---| chemosensitivity	15150572	10.1038/sj.bjc.6601876
negative Regulation: AKT1 ---| chemosensitivity	18071906	10.1007/s10495-007-0165-6
negative Regulation: AKT1 ---| chemosensitivity	18224693	10.1002/ijc.2337110.1002/ij
negative Regulation: AKT1 ---| chemosensitivity	19375813	10.1016/j.lungcan.2009.03.0
negative Regulation: AKT1 ---| chemosensitivity	19625496	10.1158/1535-7163.MCT-09-01
negative Regulation: AKT1 ---| chemosensitivity	20944098	
negative Regulation: AKT1 ---| chemosensitivity	21273759	10.1159/00032298610.1159/00
negative Regulation: AKT1 ---| chemosensitivity	21597260	10.1159/00032284910.1159/00
negative Regulation: AKT1 ---| chemosensitivity	23530461	10.1111/ced.1213810.1111/ce
negative Regulation: AKT1 ---| chemosensitivity	23546174	10.3892/mmr.2013.140010.389
negative Regulation: AKT1 ---| chemosensitivity	26629248	
negative Regulation: AKT1 ---| chemosensitivity	27163202	10.1016/j.yexmp.2016.05.003
negative Regulation: AKT1 ---| chemosensitivity	27301639	10.1016/j.bbrc.2016.06.0571
negative Regulation: AKT1 ---| chemosensitivity	31001468	10.3389/fonc.2019.0018510.3
negative Regulation: AKT1 ---| chemosensitivity	32945500	10.3892/or.2020.771010.3892
negative Regulation: AKT1 ---| chemosensitivity	33876384	10.1007/s12031-021-01832-8
negative Regulation: AKT1 ---| chemosensitivity	34542810	10.1007/s11427-020-1978-4
negative Regulation: AKT1 ---| chemosensitivity	34792179	10.3892/or.2021.8229
negative Regulation: AKT1 ---| chemosensitivity	35257265	10.1007/s10495-022-01718-z1
negative Regulation: AKT1 ---| chemosensitivity	35354796	10.1038/s41419-022-04727-7
negative Regulation: AKT1 ---| chemosensitivity	33783376	10.1097/CAD.000000000000107
negative Regulation: TRIM59 ---| chemosensitivity	33240412	10.3892/ol.2020.1226710.389
negative Regulation: NUSAP1 ---| chemosensitivity	30476929	10.12659/MSM.910364
negative Regulation: NUSAP1 ---| chemosensitivity	32099387	10.2147/OTT.S23712710.2147/
negative Regulation: SAMD4B ---| chemosensitivity	34109425	10.3892/mmr.2021.12196
negative Regulation: SAMD4B ---| chemosensitivity	34109425	10.3892/mmr.2021.12196
negative Regulation: TXNDC5 ---| chemosensitivity	26810069	10.1007/s13277-016-4891-7
negative Regulation: TRIM27 ---| chemosensitivity	32825933	10.1016/j.prp.2020.153048
negative Regulation: TRIM27 ---| chemosensitivity	32825933	10.1016/j.prp.2020.153048
negative Regulation: TRIM27 ---| chemosensitivity	31648164	10.1016/j.biopha.2019.10955
negative Regulation: TRIM27 ---| chemosensitivity	33251042	10.1016/j.omtn.2020.10.012
negative Regulation: TRIM27 ---| chemosensitivity	33251042	10.1016/j.omtn.2020.10.012
negative Regulation: TLR7 ---| chemosensitivity	26134824	10.3892/ijo.2015.3069
negative Regulation: HSP90AA1 ---| chemosensitivity	31638211	10.3892/ijo.2019.4902
negative Regulation: HSP90AA1 ---| chemosensitivity	33766957	10.1136/jclinpath-2020-2071

negative Regulation: FOXC2 ---| chemosensitivity 27336949 10.1159/000445620
negative Regulation: FOXC2 ---| chemosensitivity 27336949 10.1159/000445620
negative Regulation: CD81 ---| chemosensitivity 32926125 10.1182/bloodadvances.20200
negative Regulation: CD81 ---| chemosensitivity 32926125 10.1182/bloodadvances.20200
negative Regulation: MIR1260B ---| chemosensitivity 31081086 10.26355/eurrev_201904_1769
negative Regulation: MIR1260B ---| chemosensitivity 30250581 10.3892/ol.2018.930710.3892
negative Regulation: IGFBP2 ---| chemosensitivity 29344663 10.3892/mmr.2018.8434
negative Regulation: IGFBP2 ---| chemosensitivity 29344663 10.3892/mmr.2018.8434
negative Regulation: IGFBP2 ---| chemosensitivity 34225729 10.1186/s12935-021-02040-5
negative Regulation: IGFBP2 ---| chemosensitivity 23515291 10.1210/en.2012-1970
negative Regulation: IGFBP2 ---| chemosensitivity 23515291 10.1210/en.2012-1970
negative Regulation: IGFBP2 ---| chemosensitivity 25093489 10.1038/bjc.2014.435
negative Regulation: IGFBP2 ---| chemosensitivity 25093489 10.1038/bjc.2014.435
negative Regulation: IGFBP2 ---| chemosensitivity 25093489 10.1038/bjc.2014.435
negative Regulation: IGFBP2 ---| chemosensitivity 31903164 10.18632/oncotarget.2735510
negative Regulation: INSR ---| chemosensitivity 31230061 10.12659/MSM.915503
negative Regulation: NHEJ1 ---| chemosensitivity 28526069 10.1186/s12885-017-3345-y
negative Regulation: GLI1 ---| chemosensitivity 31474360 10.1016/j.trecan.2019.07.00
negative Regulation: GLI1 ---| chemosensitivity 33743723 10.1186/s12967-021-02789-3
negative Regulation: GLI1 ---| chemosensitivity 33743723 10.1186/s12967-021-02789-3
negative Regulation: GLI1 ---| chemosensitivity 27008269 10.1159/000443075
negative Regulation: KIF4A ---| chemosensitivity 36217900 10.1111/cbdd.14153
negative Regulation: FUT8 ---| chemosensitivity 33571582 10.1016/j.bbagen.2021.12987
negative Regulation: FUT8 ---| chemosensitivity 24232099 10.1038/cddis.2013.450
negative Regulation: FUT8 ---| chemosensitivity 24232099 10.1038/cddis.2013.450
negative Regulation: FUT8 ---| chemosensitivity 33571582 10.1016/j.bbagen.2021.12987
negative Regulation: PARP1 ---| chemosensitivity 32360576 10.1016/j.addr.2020.04.010
negative Regulation: PARP1 ---| chemosensitivity 30466996 10.1016/j.phymed.2018.09.01
negative Regulation: PARP1 ---| chemosensitivity 21093979 10.1016/j.canlet.2010.10.02
negative Regulation: PARP1 ---| chemosensitivity 19229243
negative Regulation: PARP1 ---| chemosensitivity 22204321 10.2174/138945012799201711
negative Regulation: PARP1 ---| chemosensitivity 21270666 10.1097/JTO.0b013e31820c9e3
negative Regulation: PARP1 ---| chemosensitivity 18440222 10.1016/j.ejca.2008.03.0191
negative Regulation: PARP1 ---| chemosensitivity 28916446 10.1016/j.jconrel.2017.09.0
negative Regulation: PARP1 ---| chemosensitivity 33363619 10.3892/ol.2020.1234310.389
negative Regulation: PARP1 ---| chemosensitivity 35130631 10.3724/abbs.202101110.3724
negative Regulation: PARP1 ---| chemosensitivity 35130631 10.3724/abbs.202101110.3724
negative Regulation: MVP ---| chemosensitivity 23974068 10.1016/j.drudis.2013.08.00
negative Regulation: MVP ---| chemosensitivity
negative Regulation: RALBP1 ---| chemosensitivity 26125275
negative Regulation: RALBP1 ---| chemosensitivity 23292182 10.1007/s11060-013-1045-2
negative Regulation: RALBP1 ---| chemosensitivity 24839008 10.1007/s13277-014-2073-z
negative Regulation: RALBP1 ---| chemosensitivity 24839008 10.1007/s13277-014-2073-z
negative Regulation: RALBP1 ---| chemosensitivity 24839008 10.1007/s13277-014-2073-z
negative Regulation: RALBP1 ---| chemosensitivity 27473470 10.1007/s10571-016-0410-z10
negative Regulation: RALBP1 ---| chemosensitivity 23292182 10.1007/s11060-013-1045-210
negative Regulation: RALBP1 ---| chemosensitivity
negative Regulation: OLFM4 ---| chemosensitivity 34974280 10.1016/j.tranon.2021.10133
negative Regulation: OLFM4 ---| chemosensitivity 34974280 10.1016/j.tranon.2021.10133
negative Regulation: CDC25C ---| chemosensitivity 28487115 10.1016/j.ymthe.2017.04.016
negative Regulation: EDN1 ---| chemosensitivity 28013061 10.1016/j.resp.2016.12.005
negative Regulation: EDN1 ---| chemosensitivity 24184161 10.1016/j.bbamcr.2013.10.01
negative Regulation: EDN1 ---| chemosensitivity 24184161 10.1016/j.bbamcr.2013.10.01
negative Regulation: EDN1 ---| chemosensitivity 24468467 10.1016/j.resp.2014.01.015

negative Regulation: TLR8 ---| chemosensitivity	26134824	10.3892/ijo.2015.3069
negative Regulation: RNF13 ---| chemosensitivity	36551703	10.3390/cancers14246218
negative Regulation: RNF13 ---| chemosensitivity	36551703	10.3390/cancers14246218
negative Regulation: TBL1X ---| chemosensitivity	26070712	10.15252/emmm.20140483710.1
negative Regulation: NR2F1-AS1 ---| chemosensitivit	31651347	10.1186/s12943-019-1086-z
negative Regulation: PTK2 ---| chemosensitivity	32446364	10.1016/j.bbrc.2020.04.107
negative Regulation: PTK2 ---| chemosensitivity	33038490	10.1016/j.canlet.2020.10.00
negative Regulation: PTK2 ---| chemosensitivity	21176958	10.1016/j.leukres.2010.12.0
negative Regulation: PTK2 ---| chemosensitivity	25316657	10.1016/j.pharmthera.2014.1
negative Regulation: PTK2 ---| chemosensitivity	20021699	
negative Regulation: PTK2 ---| chemosensitivity	20021699	
negative Regulation: PTK2 ---| chemosensitivity	31236120	10.1186/s11658-019-0169-6
negative Regulation: PTK2 ---| chemosensitivity	29984656	10.2174/156800961866618070б
negative Regulation: PTK2 ---| chemosensitivity	32872257	10.3390/ijms21176234
negative Regulation: PTK2 ---| chemosensitivity	20021699	10.1186/1476-4598-8-12510.1
negative Regulation: PTK2 ---| chemosensitivity	24062525	10.1093/jnci/djt21010.1093/
negative Regulation: PTK2 ---| chemosensitivity	26631041	10.1007/s13277-015-4528-210
negative Regulation: PTK2 ---| chemosensitivity	31969973	10.1016/j.csbj.2019.12.0101
negative Regulation: PTK2 ---| chemosensitivity	35163650	10.3390/ijms23031726
negative Regulation: PTK2 ---| chemosensitivity	35350066	10.1158/0008-5472.CAN-20-38
negative Regulation: PTK2 ---| chemosensitivity	35820889	10.1186/s12885-022-09799-4
negative Regulation: ZNF649-AS1 ---| chemosensitivi	32735773	10.1016/j.ymthe.2020.07.019
negative Regulation: ZNF649-AS1 ---| chemosensitivi	32735773	10.1016/j.ymthe.2020.07.019
negative Regulation: ZNF649-AS1 ---| chemosensitivi	32735773	10.1016/j.ymthe.2020.07.019
negative Regulation: ZNF649-AS1 ---| chemosensitivi	32735773	10.1016/j.ymthe.2020.07.019
negative Regulation: LINC02883 ---| chemosensitivit	31850704	10.7754/Clin.Lab.2019.19053
negative Regulation: LINC02883 ---| chemosensitivit	31850704	10.7754/Clin.Lab.2019.19053
negative Regulation: LINC02883 ---| chemosensitivit	31850704	10.7754/Clin.Lab.2019.19053
negative Regulation: LINC02883 ---| chemosensitivit	31850704	10.7754/Clin.Lab.2019.19053
negative Regulation: METTL1 ---| chemosensitivity	31866582	10.18632/aging.10257510.186
negative Regulation: METTL1 ---| chemosensitivity	35217794	10.1038/s41388-022-02250-9
negative Regulation: METTL1 ---| chemosensitivity	35217794	10.1038/s41388-022-02250-9
negative Regulation: METTL1 ---| chemosensitivity	35217794	10.1038/s41388-022-02250-9
negative Regulation: METTL1 ---| chemosensitivity	35217794	10.1038/s41388-022-02250-9
negative Regulation: METTL1 ---| chemosensitivity	35217794	10.1038/s41388-022-02250-9
negative Regulation: TAOK3 ---| chemosensitivity	33087151	10.1186/s12964-020-00600-2
negative Regulation: NODAL ---| chemosensitivity	30699065	10.1515/hsz-2018-0392
negative Regulation: NODAL ---| chemosensitivity	30699065	10.1515/hsz-2018-0392
negative Regulation: NODAL ---| chemosensitivity	30699065	10.1515/hsz-2018-0392
negative Regulation: NODAL ---| chemosensitivity	21532621	10.1038/onc.2011.127
negative Regulation: PTN ---| chemosensitivity	30497491	10.1186/s12964-018-0304-4
negative Regulation: PTN ---| chemosensitivity	28969035	10.18632/oncotarget.1914810
negative Regulation: PTN ---| chemosensitivity		10.1186/s12964-018-0304-4
negative Regulation: ST8SIA1 ---| chemosensitivity	34577144	10.3390/molecules26185673
negative Regulation: METAP2 ---| chemosensitivity	30880771	10.4103/jcrt.JCRT_250_1710.
negative Regulation: DOT1L ---| chemosensitivity	26111464	10.1053/j.seminhematol.2015
negative Regulation: USP35 ---| chemosensitivity	35022505	10.1038/s41374-021-00725-z
negative Regulation: NOTCH1 ---| chemosensitivity	26302232	10.1016/j.biomaterials.2015
negative Regulation: NOTCH1 ---| chemosensitivity	30336197	10.1016/j.canlet.2018.09.03
negative Regulation: NOTCH1 ---| chemosensitivity	30497876	10.1016/j.prp.2018.10.013
negative Regulation: NOTCH1 ---| chemosensitivity	29879503	10.1016/j.gene.2018.06.005
negative Regulation: NOTCH1 ---| chemosensitivity	31545250	10.1016/j.biopha.2019.10930
negative Regulation: NOTCH1 ---| chemosensitivity	29723707	10.1016/j.dnarep.2018.04.00
negative Regulation: NOTCH1 ---| chemosensitivity	27727250	10.1038/mtna.2016.82

negative Regulation: NOTCH1 ---| chemosensitivity 21420785 10.1016/j.canlet.2011.02.03
negative Regulation: NOTCH1 ---| chemosensitivity 20600632 10.1016/j.bbcan.2010.06.001
negative Regulation: NOTCH1 ---| chemosensitivity 23500524 10.1016/j.biocel.2013.02.02
negative Regulation: NOTCH1 ---| chemosensitivity 23500524 10.1016/j.biocel.2013.02.02
negative Regulation: NOTCH1 ---| chemosensitivity 23810012 10.1016/B978-0-12-416673-8.
negative Regulation: NOTCH1 ---| chemosensitivity 28914645 10.1097/CCO.000000000000040
negative Regulation: NOTCH1 ---| chemosensitivity 32228202 10.1177/0300060520904847
negative Regulation: NOTCH1 ---| chemosensitivity 32913140 10.1124/MOLPHARM.120.000006
negative Regulation: NOTCH1 ---| chemosensitivity 32913140 10.1124/MOLPHARM.120.000006
negative Regulation: NOTCH1 ---| chemosensitivity 24451948 10.3760/cma.j.issn.0366-699
negative Regulation: NOTCH1 ---| chemosensitivity 24451948 10.3760/cma.j.issn.0366-699
negative Regulation: NOTCH1 ---| chemosensitivity 26927514 10.1038/labinvest.2015.163
negative Regulation: NOTCH1 ---| chemosensitivity 19550121
negative Regulation: NOTCH1 ---| chemosensitivity 20204271 10.3892/or_00000712
negative Regulation: NOTCH1 ---| chemosensitivity 20204271 10.3892/or_00000712
negative Regulation: NOTCH1 ---| chemosensitivity 23568245 10.1007/s00268-013-2010-010
negative Regulation: NOTCH1 ---| chemosensitivity 24316112 10.1016/j.arcmed.2013.10.00
negative Regulation: NOTCH1 ---| chemosensitivity 24782036 10.1007/s11010-014-2069-410
negative Regulation: NOTCH1 ---| chemosensitivity 24802328 10.1007/s12032-014-0972-x10
negative Regulation: NOTCH1 ---| chemosensitivity 31545250 10.1016/j.biopha.2019.10930
negative Regulation: NOTCH1 ---| chemosensitivity 1150 10.1016/j.prp.2018.10.013
negative Regulation: NOTCH1 ---| chemosensitivity 10.1097/CCO.000000000000040
negative Regulation: NOTCH1 ---| chemosensitivity 10.2298/ABS210423030R
negative Regulation: NOTCH1 ---| chemosensitivity 33882814 10.2174/1871530321666210421
negative Regulation: SLC34A2 ---| chemosensitivity 30592329 10.1002/jcb.2830510.1002/jc
negative Regulation: PLA2G4A ---| chemosensitivity 30829552 10.1080/15384047.2019.15799
negative Regulation: PLA2G4A ---| chemosensitivity 30829552 10.1080/15384047.2019.15799
negative Regulation: PLA2G4A ---| chemosensitivity 30829552 10.1080/15384047.2019.15799
negative Regulation: PLA2G4A ---| chemosensitivity 30829552 10.1080/15384047.2019.15799
negative Regulation: PLA2G4A ---| chemosensitivity 30829552 10.1080/15384047.2019.15799
negative Regulation: PLA2G4A ---| chemosensitivity 30829552 10.1080/15384047.2019.15799
negative Regulation: PLA2G4A ---| chemosensitivity 30829552 10.1080/15384047.2019.15799
negative Regulation: RELN ---| chemosensitivity 32764530 10.12659/MSM.925298
negative Regulation: RELN ---| chemosensitivity 32764530 10.12659/MSM.92529810.12659
negative Regulation: PRKCA ---| chemosensitivity 32203165 10.1038/s41388-020-1261-0
negative Regulation: BCL2L1 ---| chemosensitivity 15611130 10.1074/jbc.M409906200
negative Regulation: BCL2L1 ---| chemosensitivity 31102887 10.1016/j.phymed.2019.15294
negative Regulation: BCL2L1 ---| chemosensitivity 10.1016/S1525-0016(16)41190
negative Regulation: BCL2L1 ---| chemosensitivity 11458048 10.1016/S0022-5347(05)65964
negative Regulation: BCL2L1 ---| chemosensitivity 21277008 10.1016/j.acthis.2011.01.00
negative Regulation: BCL2L1 ---| chemosensitivity 15327837 10.1016/j.canlet.2004.03.04
negative Regulation: BCL2L1 ---| chemosensitivity 18840529 10.1016/j.phrs.2008.09.005
negative Regulation: BCL2L1 ---| chemosensitivity 16280040
negative Regulation: BCL2L1 ---| chemosensitivity 15611130
negative Regulation: BCL2L1 ---| chemosensitivity 17353918
negative Regulation: BCL2L1 ---| chemosensitivity 31810474 10.1186/s13048-019-0588-z
negative Regulation: BCL2L1 ---| chemosensitivity 22664653 10.3892/ijmm.2012.1013
negative Regulation: BCL2L1 ---| chemosensitivity 22664653 10.3892/ijmm.2012.1013
negative Regulation: BCL2L1 ---| chemosensitivity 23171055 10.1186/1471-2407-12-541
negative Regulation: BCL2L1 ---| chemosensitivity 23666471 10.1097/CCO.0b013e3283622c1
negative Regulation: BCL2L1 ---| chemosensitivity 24173654 10.3892/or.2013.2817
negative Regulation: BCL2L1 ---| chemosensitivity 16080514
negative Regulation: BCL2L1 ---| chemosensitivity 17224645 10.4161/cbt.6.2.3626
negative Regulation: BCL2L1 ---| chemosensitivity 34678222 10.1016/j.bcp.2021.114801

negative Regulation: PRMT5 ---| chemosensitivity	31665911	10.1177/0963689719885083
negative Regulation: PRMT5 ---| chemosensitivity	31665911	10.1177/0963689719885083
negative Regulation: PRMT5 ---| chemosensitivity	31665911	10.1177/0963689719885083
negative Regulation: PRMT5 ---| chemosensitivity	32766778	10.1042/CS20200680
negative Regulation: PRMT5 ---| chemosensitivity	31665911	10.1177/096368971988508310.
negative Regulation: CYBA ---| chemosensitivity	30800222	10.18632/oncotarget.2663710
negative Regulation: CYBA ---| chemosensitivity	34093828	10.7150/jca.5416310.7150/jc
negative Regulation: ANK2 ---| chemosensitivity	18441325	
negative Regulation: ANK2 ---| chemosensitivity	18441325	10.1074/jbc.M80010920010.10
negative Regulation: CX3CL1 ---| chemosensitivity	35294039	10.3892/ijo.2022.5337
negative Regulation: CX3CL1 ---| chemosensitivity	35294039	10.3892/ijo.2022.5337
negative Regulation: MYH9 ---| chemosensitivity	33220257	10.1016/j.yexcr.2020.112387
negative Regulation: HMGB1 ---| chemosensitivity	32553931	10.1016/j.lfs.2020.117967
negative Regulation: HMGB1 ---| chemosensitivity	33035835	10.1016/j.biopha.2020.11079
negative Regulation: HMGB1 ---| chemosensitivity	30612957	10.1016/j.redox.2018.101084
negative Regulation: HMGB1 ---| chemosensitivity	32388677	10.1007/s00262-020-02598-5
negative Regulation: HMGB1 ---| chemosensitivity	24418846	10.4161/auto.27418
negative Regulation: HMGB1 ---| chemosensitivity	25101674	10.1038/cddis.2014.300
negative Regulation: HMGB1 ---| chemosensitivity	25101674	10.1038/cddis.2014.300
negative Regulation: HMGB1 ---| chemosensitivity	25101674	10.1038/cddis.2014.300
negative Regulation: HMGB1 ---| chemosensitivity	26397184	10.3892/or.2015.4278
negative Regulation: HMGB1 ---| chemosensitivity	24418846	10.4161/auto.2741810.4161/a
negative Regulation: HMGB1 ---| chemosensitivity	26397184	10.3892/or.2015.427810.3892
negative Regulation: HMGB1 ---| chemosensitivity	28885675	10.5301/tj.500065610.5301/t
negative Regulation: HMGB1 ---| chemosensitivity	30154286	10.11817/j.issn.1672-7347.2
negative Regulation: HMGB1 ---| chemosensitivity	31664305	10.1590/1414-431X2019865710
negative Regulation: HMGB1 ---| chemosensitivity		10.4161/auto.27418
negative Regulation: HMGB1 ---| chemosensitivity	25934464	10.1016/j.thromres.2015.03.
negative Regulation: HMGB1 ---| chemosensitivity		
negative Regulation: HMGB1 ---| chemosensitivity	35193644	10.1186/s13046-022-02291-81
negative Regulation: HMGB1 ---| chemosensitivity	35193644	10.1186/s13046-022-02291-8
negative Regulation: HMGB1 ---| chemosensitivity	35193644	10.1186/s13046-022-02291-8
negative Regulation: HMGB1 ---| chemosensitivity	35193644	10.1186/s13046-022-02291-8
negative Regulation: PDRG1 ---| chemosensitivity	31882431	
negative Regulation: CLN3 ---| chemosensitivity	26299671	10.3892/mmr.2015.4238
negative Regulation: CLN3 ---| chemosensitivity	26299671	10.3892/mmr.2015.4238
negative Regulation: PRKDC ---| chemosensitivity	24530422	10.1016/j.bbagrm.2014.02.00
negative Regulation: PRKDC ---| chemosensitivity	28498431	10.3892/or.2017.5634
negative Regulation: PRKDC ---| chemosensitivity	28498431	10.3892/or.2017.5634
negative Regulation: PRKDC ---| chemosensitivity	32661323	10.1038/s41388-020-1384-3
negative Regulation: PRKDC ---| chemosensitivity	26065416	10.1371/journal.pone.012794
negative Regulation: PRKDC ---| chemosensitivity	26065416	10.1371/journal.pone.012794
negative Regulation: PRKDC ---| chemosensitivity	14532987	10.3892/ijo.23.5.1431
negative Regulation: PRKDC ---| chemosensitivity	27487130	10.18632/oncotarget.1086410
negative Regulation: PRKDC ---| chemosensitivity	28498431	10.3892/or.2017.563410.3892
negative Regulation: PRKDC ---| chemosensitivity	31255330	10.1016/j.prp.2019.15250910
negative Regulation: MIR124-3 ---| chemosensitivity	31708690	10.1186/s12935-019-1004-x
negative Regulation: MIR492 ---| chemosensitivity	32065219	10.1042/BSR20194342
negative Regulation: MIR629 ---| chemosensitivity	31257454	10.3892/ijmm.2019.4245
negative Regulation: ATF4 ---| chemosensitivity	33782384	10.1038/s41419-021-03574-2
negative Regulation: AK6 ---| chemosensitivity	32774758	
negative Regulation: RB1 ---| chemosensitivity	20594292	
negative Regulation: RB1 ---| chemosensitivity	18782450	
negative Regulation: RB1 ---| chemosensitivity	9342368	

negative Regulation: RB1 ---| chemosensitivity	9342368	
negative Regulation: RB1 ---| chemosensitivity	11521194	
negative Regulation: RB1 ---| chemosensitivity	28551632	10.21873/anticanres.11648
negative Regulation: RB1 ---| chemosensitivity	20594292	10.1186/1476-4598-9-173
negative Regulation: RB1 ---| chemosensitivity	24987858	10.1371/journal.pone.010095
negative Regulation: RB1 ---| chemosensitivity	17640669	10.1016/j.jss.2007.03.038
negative Regulation: RB1 ---| chemosensitivity	17640669	10.1016/j.jss.2007.03.038
negative Regulation: RB1 ---| chemosensitivity	18332869	10.1038/onc.2008.4310.1038/
negative Regulation: RB1 ---| chemosensitivity	34214844	10.1016/j.prp.2021.15353210
negative Regulation: HRAS ---| chemosensitivity	25189269	10.3892/or.2014.3466
negative Regulation: HRAS ---| chemosensitivity	25189269	10.3892/or.2014.3466
negative Regulation: HRAS ---| chemosensitivity	25189269	10.3892/or.2014.346610.3892
negative Regulation: SPP1 ---| chemosensitivity	28433634	10.1016/j.bbrc.2017.04.100
negative Regulation: SPP1 ---| chemosensitivity	30535434	10.3892/ijo.2018.4656
negative Regulation: SPP1 ---| chemosensitivity	21539449	10.1089/cbr.2010.0838
negative Regulation: SPP1 ---| chemosensitivity	30627777	10.1007/s00280-018-3759-510
negative Regulation: SPP1 ---| chemosensitivity		
negative Regulation: IL6 ---| chemosensitivity	22609487	10.1016/j.cca.2012.05.009
negative Regulation: IL6 ---| chemosensitivity	23298711	10.1016/j.ejca.2012.12.002
negative Regulation: IL6 ---| chemosensitivity	27725789	10.1155/2016/5894347
negative Regulation: IL6 ---| chemosensitivity	26923327	10.1038/onc.2016.34
negative Regulation: IL6 ---| chemosensitivity	29693201	10.1007/s00280-018-3584-x
negative Regulation: IL6 ---| chemosensitivity		10.1007/978-3-030-20301-6_9
negative Regulation: IL6 ---| chemosensitivity	15894559	10.1152/ajplung.00033.2005
negative Regulation: IL6 ---| chemosensitivity	31714645	10.1002/jbt.2241210.1002/jb
negative Regulation: IL6 ---| chemosensitivity	32933994	10.1158/1078-0432.CCR-19-27
negative Regulation: IL6 ---| chemosensitivity	33135338	10.1002/ctm2.21710.1002/ctm
negative Regulation: IL6 ---| chemosensitivity	33742970	10.1515/hsz-2020-034510.151
negative Regulation: IL6 ---| chemosensitivity	33742970	10.1515/hsz-2020-0345
negative Regulation: IL6 ---| chemosensitivity	33742970	10.1515/hsz-2020-0345
negative Regulation: TGFB1 ---| chemosensitivity	23298711	10.1016/j.ejca.2012.12.002
negative Regulation: TGFB1 ---| chemosensitivity	29204827	10.1007/978-3-319-69194-7_3
negative Regulation: TGFB1 ---| chemosensitivity		10.1186/s40659-018-0205-4
negative Regulation: TGFB1 ---| chemosensitivity		10.1186/s40659-018-0205-4
negative Regulation: TGFB1 ---| chemosensitivity		10.1186/s40659-018-0205-4
negative Regulation: TGFB1 ---| chemosensitivity	31127087	10.1038/s41419-019-1597-y
negative Regulation: TGFB1 ---| chemosensitivity	31087138	10.1007/s00280-019-03866-7
negative Regulation: TGFB1 ---| chemosensitivity	30594239	10.1186/s40659-018-0205-410
negative Regulation: TGFB1 ---| chemosensitivity	30594239	10.1186/s40659-018-0205-410
negative Regulation: TGFB1 ---| chemosensitivity	30594239	10.1186/s40659-018-0205-410
negative Regulation: TGFB1 ---| chemosensitivity	28918673	10.1080/21691401.2017.13742
negative Regulation: TGFB1 ---| chemosensitivity	31814893	
negative Regulation: WEE1 ---| chemosensitivity	25965828	10.18632/oncotarget.3865
negative Regulation: WEE1 ---| chemosensitivity	31387179	10.1016/j.biopha.2019.10918
negative Regulation: MIR543 ---| chemosensitivity	34347977	10.1615/CritRevEukaryotGene
negative Regulation: CDC42 ---| chemosensitivity	32532057	10.3390/cancers1206152110.3
negative Regulation: POU5F1 ---| chemosensitivity	23872274	10.1016/j.canlet.2013.07.00
negative Regulation: POU5F1 ---| chemosensitivity	33650661	10.3892/or.2021.7925
negative Regulation: POU5F1 ---| chemosensitivity	22732500	10.4161/cc.21021
negative Regulation: POU5F1 ---| chemosensitivity	26260289	10.1186/s12943-015-0417-y
negative Regulation: POU5F1 ---| chemosensitivity	26398480	10.3892/or.2015.4286
negative Regulation: POU5F1 ---| chemosensitivity		
negative Regulation: POU5F1 ---| chemosensitivity		10.3892/or.2021.7925
negative Regulation: LINC01270 ---| chemosensitivit	33199829	10.1038/s41417-020-00232-11

negative Regulation: LINC01270 ---| chemosensitivit	35277667	10.1038/s41417-022-00439-4
negative Regulation: MIR630 ---| chemosensitivity	29452092	10.1016/j.bbrc.2018.02.062
negative Regulation: MIR630 ---| chemosensitivity	29949157	10.26355/eurrev-201806-1526
negative Regulation: MIR630 ---| chemosensitivity	26345808	10.4238/2015.July.31.25
negative Regulation: MIR630 ---| chemosensitivity	26345808	10.4238/2015.July.31.25
negative Regulation: MIR630 ---| chemosensitivity	26345808	10.4238/2015.July.31.25
negative Regulation: MIR630 ---| chemosensitivity	26345808	10.4238/2015.July.31.25
negative Regulation: MIR630 ---| chemosensitivity	26345808	10.4238/2015.July.31.2510.4
negative Regulation: MIR630 ---| chemosensitivity	26345808	10.4238/2015.July.31.2510.4
negative Regulation: MIR630 ---| chemosensitivity	26345808	10.4238/2015.July.31.2510.4
negative Regulation: IDH2 ---| chemosensitivity	36637036	10.15252/embj.2022110620
negative Regulation: UBQLN1 ---| chemosensitivity	34528694	10.3892/or.2021.818710.3892
negative Regulation: NR5A2 ---| chemosensitivity	29545602	10.1038/s41388-018-0193-4
negative Regulation: NR5A2 ---| chemosensitivity	29545602	10.1038/s41388-018-0193-4
negative Regulation: ESRRA ---| chemosensitivity	35918761	10.1186/s12967-022-03549-7
negative Regulation: ALDH1A3 ---| chemosensitivity	30865894	10.1016/j.celrep.2019.02.03
negative Regulation: ALDH1A3 ---| chemosensitivity	32270866	10.1042/BSR20200390
negative Regulation: ALDH1A3 ---| chemosensitivity		10.1042/BSR20200390
negative Regulation: MST1R ---| chemosensitivity	27035413	10.3892/or.2016.4721
negative Regulation: MST1R ---| chemosensitivity	27035413	10.3892/or.2016.472110.3892
negative Regulation: TGM2 ---| chemosensitivity	30015899	10.3892/ijmm.2018.3753
negative Regulation: TGM2 ---| chemosensitivity	30015899	10.3892/ijmm.2018.3753
negative Regulation: TGM2 ---| chemosensitivity	30015899	10.3892/ijmm.2018.3753
negative Regulation: TGM2 ---| chemosensitivity	30015899	10.3892/ijmm.2018.3753
negative Regulation: TGM2 ---| chemosensitivity	30015899	10.3892/ijmm.2018.3753
negative Regulation: TGM2 ---| chemosensitivity	30015899	10.3892/ijmm.2018.3753
negative Regulation: TGM2 ---| chemosensitivity	30015899	10.3892/ijmm.2018.3753
negative Regulation: TGM2 ---| chemosensitivity	30015899	10.3892/ijmm.2018.3753
negative Regulation: TGM2 ---| chemosensitivity	30015899	10.3892/ijmm.2018.3753
negative Regulation: TGM2 ---| chemosensitivity	32572883	10.26355/eurrev_202006_2151
negative Regulation: TGM2 ---| chemosensitivity	32572883	10.26355/eurrev_202006_2151
negative Regulation: TGM2 ---| chemosensitivity	20596752	10.1007/s11060-010-0277-7
negative Regulation: TGM2 ---| chemosensitivity	16170020	10.1158/1535-7163.MCT-04-03
negative Regulation: TGM2 ---| chemosensitivity	26622499	10.3892/etm.2015.2679
negative Regulation: TGM2 ---| chemosensitivity	26622499	10.3892/etm.2015.2679
negative Regulation: TGM2 ---| chemosensitivity	30015899	10.3892/ijmm.2018.375310.38
negative Regulation: TGM2 ---| chemosensitivity	30015899	10.3892/ijmm.2018.375310.38
negative Regulation: TGM2 ---| chemosensitivity	34549308	10.3892/mmr.2021.12452
negative Regulation: TGM2 ---| chemosensitivity	34549308	10.3892/mmr.2021.12452
negative Regulation: SFRP5 ---| chemosensitivity	35269636	10.3390/ijms23052496
negative Regulation: KCNN1 ---| chemosensitivity	22833671	10.1074/jbc.M112.385765
negative Regulation: PDCD1 ---| chemosensitivity	33765543	10.1016/j.tranon.2021.10106
negative Regulation: PDCD1 ---| chemosensitivity	33710337	10.1182/bloodadvances.20200
negative Regulation: YBX1 ---| chemosensitivity	27044807	10.1016/j.biopha.2016.01.03
negative Regulation: YBX1 ---| chemosensitivity	27044807	10.1016/j.biopha.2016.01.03
negative Regulation: TIE1 ---| chemosensitivity	33812182	10.1016/j.ctarc.2021.100364
negative Regulation: PPBP ---| chemosensitivity	25822018	10.1172/JCI78752
negative Regulation: PPBP ---| chemosensitivity	25822018	10.1172/JCI78752
negative Regulation: PRKD1 ---| chemosensitivity	24665648	
negative Regulation: LINC00662 ---| chemosensitivit	30297104	10.1016/j.bbrc.2018.09.191
negative Regulation: LINC00662 ---| chemosensitivit	30297104	10.1016/j.bbrc.2018.09.1911
negative Regulation: DCLK1 ---| chemosensitivity	30119173	10.1016/j.biopha.2018.07.02
negative Regulation: DCLK1 ---| chemosensitivity	32533239	10.1007/s00424-020-02415-z
negative Regulation: DCLK1 ---| chemosensitivity	32533239	10.1007/s00424-020-02415-z

negative Regulation: DCLK1 ---| chemosensitivity	32533239	10.1007/s00424-020-02415-z1
negative Regulation: DCLK1 ---| chemosensitivity	32533239	10.1007/s00424-020-02415-z1
negative Regulation: TSPAN9 ---| chemosensitivity	31911756	10.1186/s12935-019-1089-2
negative Regulation: TSPAN9 ---| chemosensitivity	31911756	10.1186/s12935-019-1089-210
negative Regulation: ROS1 ---| chemosensitivity	27035628	10.3892/ijo.2016.3452
negative Regulation: ROS1 ---| chemosensitivity	27035628	10.3892/ijo.2016.3452
negative Regulation: DAZL ---| chemosensitivity	32682409	10.1186/s12885-020-07155-y
negative Regulation: DNMT3A ---| chemosensitivity	29115606	10.3892/mmr.2017.7923
negative Regulation: DNMT3A ---| chemosensitivity	29115606	10.3892/mmr.2017.792310.389
negative Regulation: DNMT3A ---| chemosensitivity	29115606	10.3892/mmr.2017.792310.389
negative Regulation: DNMT3A ---| chemosensitivity	29115606	10.3892/mmr.2017.792310.389
negative Regulation: XIAP ---| chemosensitivity	15196850	10.1016/j.ygyno.2004.03.029
negative Regulation: XIAP ---| chemosensitivity	20647037	10.1016/j.bbcan.2010.07.004
negative Regulation: XIAP ---| chemosensitivity	17210228	10.1016/j.ctrv.2006.11.002
negative Regulation: XIAP ---| chemosensitivity	22627131	10.1016/j.bbrc.2012.05.066
negative Regulation: XIAP ---| chemosensitivity	25776486	10.1016/j.biopha.2014.12.04
negative Regulation: XIAP ---| chemosensitivity	19445670	
negative Regulation: XIAP ---| chemosensitivity	19904270	
negative Regulation: XIAP ---| chemosensitivity	16322751	
negative Regulation: XIAP ---| chemosensitivity	20676365	
negative Regulation: XIAP ---| chemosensitivity	23242307	10.3892/or.2012.2189
negative Regulation: XIAP ---| chemosensitivity	23354694	10.3892/or.2013.2246
negative Regulation: XIAP ---| chemosensitivity	23354694	10.3892/or.2013.2246
negative Regulation: XIAP ---| chemosensitivity	23354694	10.3892/or.2013.2246
negative Regulation: XIAP ---| chemosensitivity	23479728	10.1074/jbc.M112.436113
negative Regulation: XIAP ---| chemosensitivity	20676365	10.1371/journal.pone.001181
negative Regulation: XIAP ---| chemosensitivity	24330851	10.3727/096504013X137935557
negative Regulation: XIAP ---| chemosensitivity	24330851	10.3727/096504013X137935557
negative Regulation: XIAP ---| chemosensitivity	24853184	10.1038/bjc.2014.255
negative Regulation: XIAP ---| chemosensitivity	24839986	10.3892/or.2014.3200
negative Regulation: XIAP ---| chemosensitivity	25572427	10.3892/or.2014.3698
negative Regulation: XIAP ---| chemosensitivity	17127230	10.1179/joc.2006.18.5.525
negative Regulation: XIAP ---| chemosensitivity	17611394	
negative Regulation: XIAP ---| chemosensitivity	22736919	10.3748/wjg.v18.i23.295610.
negative Regulation: XIAP ---| chemosensitivity	23591345	
negative Regulation: XIAP ---| chemosensitivity	25572427	10.3892/or.2014.369810.3892
negative Regulation: XIAP ---| chemosensitivity	29221164	10.18632/oncotarget.2132010
negative Regulation: XIAP ---| chemosensitivity	29221164	10.18632/oncotarget.2132010
negative Regulation: XIAP ---| chemosensitivity	29221164	10.18632/oncotarget.2132010
negative Regulation: XIAP ---| chemosensitivity	25707849	10.3892/mmr.2015.3380
negative Regulation: GHET1 ---| chemosensitivity	32720600	10.2174/1389201021999200727
negative Regulation: RPN2 ---| chemosensitivity	33087705	10.1038/s41419-020-03113-5
negative Regulation: RPN2 ---| chemosensitivity	33087705	10.1038/s41419-020-03113-5
negative Regulation: RPN2 ---| chemosensitivity	33087705	10.1038/s41419-020-03113-5
negative Regulation: RPN2 ---| chemosensitivity	33087705	10.1038/s41419-020-03113-5
negative Regulation: PRL ---| chemosensitivity	28204229	10.1210/en.2016-1903
negative Regulation: PRL ---| chemosensitivity	28204229	10.1210/en.2016-1903
negative Regulation: PRL ---| chemosensitivity	28204229	10.1210/en.2016-1903
negative Regulation: PRL ---| chemosensitivity	28204229	10.1210/en.2016-190310.1210
negative Regulation: FOSL1 ---| chemosensitivity	27685628	10.1038/cddis.2016.289
negative Regulation: FOSL1 ---| chemosensitivity	22586064	10.1158/0008-5472.CAN-11-25
negative Regulation: FOSL1 ---| chemosensitivity	27685628	10.1038/cddis.2016.28910.10
negative Regulation: AR ---| chemosensitivity	22851332	10.1007/s12020-012-9762-410
negative Regulation: FGFRL1 ---| chemosensitivity	31957179	10.1111/jcmm.1476310.1111/j

negative Regulation: YY1 ---| chemosensitivity		10.1615/CritRevEukaryotGene
negative Regulation: TFAP2C ---| chemosensitivity	29439714	10.1186/s13046-018-0683-9
negative Regulation: RAC1 ---| chemosensitivity	31787614	10.3324/haematol.2019.23134
negative Regulation: RAC1 ---| chemosensitivity	32193458	10.1038/s41467-020-15308-7
negative Regulation: RAC1 ---| chemosensitivity	32193458	10.1038/s41467-020-15308-7
negative Regulation: RAC1 ---| chemosensitivity	32193458	10.1038/s41467-020-15308-7
negative Regulation: RAC1 ---| chemosensitivity	32193458	10.1038/s41467-020-15308-7
negative Regulation: RAC1 ---| chemosensitivity	32193458	10.1038/s41467-020-15308-7
negative Regulation: RAC1 ---| chemosensitivity	33790078	10.1248/CPB.C20-00600
negative Regulation: RAC1 ---| chemosensitivity	21837360	10.3892/ijmm.2011.775
negative Regulation: RAC1 ---| chemosensitivity	23792636	10.4161/cbt.25091
negative Regulation: RAC1 ---| chemosensitivity	23760915	10.1177/0300060513479875
negative Regulation: RAC1 ---| chemosensitivity	23760915	10.1177/0300060513479875
negative Regulation: RAC1 ---| chemosensitivity	24441506	10.1097/CCO.000000000000004
negative Regulation: RAC1 ---| chemosensitivity	21837360	10.3892/ijmm.2011.77510.389
negative Regulation: RAC1 ---| chemosensitivity		10.3324/haematol.2019.23134
negative Regulation: RAC1 ---| chemosensitivity	36599922	10.1038/s41388-022-02574-6
negative Regulation: RNPS1 ---| chemosensitivity	36300671	10.1002/iub.2686
negative Regulation: CTNNB1 ---| chemosensitivity	31071331	10.1016/j.bcp.2019.05.001
negative Regulation: CTNNB1 ---| chemosensitivity	30081068	10.1016/j.canlet.2018.07.04
negative Regulation: CTNNB1 ---| chemosensitivity	32932156	10.1016/j.ctrv.2020.102103
negative Regulation: CTNNB1 ---| chemosensitivity	32563150	10.1016/j.biopha.2020.11039
negative Regulation: CTNNB1 ---| chemosensitivity	32268268	10.1016/j.ebiom.2020.102717
negative Regulation: CTNNB1 ---| chemosensitivity	28870999	10.21873/cgp.20042
negative Regulation: CTNNB1 ---| chemosensitivity		10.1007/s00280-018-3565-010
negative Regulation: CTNNB1 ---| chemosensitivity	31323761	10.3390/ijms20143518
negative Regulation: CTNNB1 ---| chemosensitivity	32814878	10.1038/s41418-020-00607-9
negative Regulation: CTNNB1 ---| chemosensitivity	32814878	10.1038/s41418-020-00607-9
negative Regulation: CTNNB1 ---| chemosensitivity	29228417	10.26355/eurrev_201711_1381
negative Regulation: CTNNB1 ---| chemosensitivity	23747891	10.2741/4187
negative Regulation: CTNNB1 ---| chemosensitivity	21858114	10.1371/journal.pone.002342
negative Regulation: CTNNB1 ---| chemosensitivity	21858114	10.1371/journal.pone.002342
negative Regulation: CTNNB1 ---| chemosensitivity	23094073	10.1371/journal.pone.004764
negative Regulation: CTNNB1 ---| chemosensitivity	23094073	10.1371/journal.pone.004764
negative Regulation: CTNNB1 ---| chemosensitivity	25658419	10.1371/journal.pone.011709
negative Regulation: CTNNB1 ---| chemosensitivity	26316041	10.3892/ijo.2015.3137
negative Regulation: CTNNB1 ---| chemosensitivity	26316041	10.3892/ijo.2015.3137
negative Regulation: CTNNB1 ---| chemosensitivity	26316041	10.3892/ijo.2015.3137
negative Regulation: CTNNB1 ---| chemosensitivity	26316041	10.3892/ijo.2015.3137
negative Regulation: CTNNB1 ---| chemosensitivity	26316041	10.3892/ijo.2015.3137
negative Regulation: CTNNB1 ---| chemosensitivity	26316041	10.3892/ijo.2015.3137
negative Regulation: CTNNB1 ---| chemosensitivity	26603103	10.1038/ncomms9904
negative Regulation: CTNNB1 ---| chemosensitivity	21517265	
negative Regulation: CTNNB1 ---| chemosensitivity	22550000	10.1002/jbmr.165010.1002/jb
negative Regulation: CTNNB1 ---| chemosensitivity	25658419	10.1371/journal.pone.011709
negative Regulation: CTNNB1 ---| chemosensitivity	26036631	10.18632/oncotarget.3896
negative Regulation: CTNNB1 ---| chemosensitivity	26316041	10.3892/ijo.2015.313710.389
negative Regulation: CTNNB1 ---| chemosensitivity	26316041	10.3892/ijo.2015.313710.389
negative Regulation: CTNNB1 ---| chemosensitivity	26316041	10.3892/ijo.2015.313710.389
negative Regulation: CTNNB1 ---| chemosensitivity	28121357	
negative Regulation: CTNNB1 ---| chemosensitivity	31305293	10.1097/CAD.000000000000074
negative Regulation: CTNNB1 ---| chemosensitivity	31882431	
negative Regulation: CTNNB1 ---| chemosensitivity	28967908	10.1038/onc.2017.328
negative Regulation: CTNNB1 ---| chemosensitivity		10.3390/ijms20143518

negative Regulation: CTNNB1 ---| chemosensitivity 29342970 10.3390/ijms19010251
negative Regulation: CTNNB1 ---| chemosensitivity 34498146 10.1007/s00432-021-03793-2
negative Regulation: CTNNB1 ---| chemosensitivity 35163465 10.3390/ijms23031545
negative Regulation: SAAL1 ---| chemosensitivity 32650537 10.3390/cancers1207184310.3
negative Regulation: SAAL1 ---| chemosensitivity 35963646 10.18632/aging.204224
negative Regulation: BCCIP ---| chemosensitivity 27995408 10.1007/s10620-016-4382-0
negative Regulation: PRDM14 ---| chemosensitivity 33355367 10.3892/mmr.2020.11788
negative Regulation: PRDM14 ---| chemosensitivity 33355367 10.3892/mmr.2020.11788
negative Regulation: PRDM14 ---| chemosensitivity 33355367 10.3892/mmr.2020.11788
negative Regulation: PRDM14 ---| chemosensitivity 33355367 10.3892/mmr.2020.1178810.38
negative Regulation: PRDM14 ---| chemosensitivity 33355367 10.3892/mmr.2020.1178810.38
negative Regulation: PRDM14 ---| chemosensitivity 21339739 10.1038/onc.2011.12
negative Regulation: PRDM14 ---| chemosensitivity
negative Regulation: TNNC1 ---| chemosensitivity 32946432 10.12659/MSM.922703
negative Regulation: MEDAG ---| chemosensitivity 33462219 10.1038/s41419-020-03340-w1
negative Regulation: RAD51 ---| chemosensitivity 30337244 10.1016/j.jphs.2018.09.011
negative Regulation: RAD51 ---| chemosensitivity 21168393 10.1016/j.bcp.2010.12.008
negative Regulation: RAD51 ---| chemosensitivity 21810436 10.1016/j.taap.2011.07.012
negative Regulation: RAD51 ---| chemosensitivity 17229870
negative Regulation: RAD51 ---| chemosensitivity 17229870
negative Regulation: RAD51 ---| chemosensitivity 17229870
negative Regulation: RAD51 ---| chemosensitivity 29620223 10.3892/or.2018.6336
negative Regulation: RAD51 ---| chemosensitivity 30483758 10.3892/ijo.2018.4642
negative Regulation: RAD51 ---| chemosensitivity 31638261 10.3892/or.2019.7384
negative Regulation: RAD51 ---| chemosensitivity 31638261 10.3892/or.2019.7384
negative Regulation: RAD51 ---| chemosensitivity 31638261 10.3892/or.2019.7384
negative Regulation: RAD51 ---| chemosensitivity 20855443 10.1124/jpet.110.173146
negative Regulation: RAD51 ---| chemosensitivity 21798026 10.1186/1476-4598-10-92
negative Regulation: RAD51 ---| chemosensitivity 17513613 10.1158/1535-7163.MCT-06-06
negative Regulation: RAD51 ---| chemosensitivity 20625943 10.1007/s12032-010-9605-110
negative Regulation: RAD51 ---| chemosensitivity 24971740 10.1371/journal.pone.010099
negative Regulation: RAD51 ---| chemosensitivity 27566579 10.18632/oncotarget.1154010
negative Regulation: RAD51 ---| chemosensitivity 27566579 10.18632/oncotarget.1154010
negative Regulation: RAD51 ---| chemosensitivity 31638261 10.3892/or.2019.738410.3892
negative Regulation: PFKFB3 ---| chemosensitivity 33420377 10.1038/s41388-020-01621-4
negative Regulation: PFKFB3 ---| chemosensitivity 33420377 10.1038/s41388-020-01621-4
negative Regulation: PFKFB3 ---| chemosensitivity 30226266 10.1002/ijc.3186810.1002/ij
negative Regulation: PFKFB3 ---| chemosensitivity 33420377 10.1038/s41388-020-01621-4
negative Regulation: PFKFB3 ---| chemosensitivity 35804016 10.1038/s41388-022-02391-x
negative Regulation: PFKFB3 ---| chemosensitivity 35553342 10.1007/s11033-022-07513-y
negative Regulation: CX3CR1 ---| chemosensitivity 35294039 10.3892/ijo.2022.5337
negative Regulation: CX3CR1 ---| chemosensitivity 35294039 10.3892/ijo.2022.5337
negative Regulation: ATP7A ---| chemosensitivity 31226634 10.1016/j.biopha.2019.10913
negative Regulation: ATP7A ---| chemosensitivity 29772714 10.3390/ijms19051486
negative Regulation: ATP7A ---| chemosensitivity 22304828 10.1186/1479-5876-10-21
negative Regulation: ATP7A ---| chemosensitivity 24150977 10.1002/ijc.2852610.1002/ij
negative Regulation: TNKS ---| chemosensitivity 24789807 10.3892/ijo.2014.2406
negative Regulation: TNKS ---| chemosensitivity 31585633 10.1016/j.mrgentox.2019.503
negative Regulation: TNKS ---| chemosensitivity 34713301 10.3892/IJMM.2021.5056
negative Regulation: TNKS ---| chemosensitivity 35750753 10.1038/s41417-022-00493-y
negative Regulation: BDNF ---| chemosensitivity 12438277
negative Regulation: BDNF ---| chemosensitivity 27852063 10.18632/oncotarget.1329110
negative Regulation: BDNF ---| chemosensitivity 12691830 10.1016/s0304-3835(02)00723
negative Regulation: BDNF ---| chemosensitivity 26868822 10.1007/s11010-016-2660-y10

negative Regulation: BDNF ---| chemosensitivity	36361620	10.3390/ijms232112829
negative Regulation: BDNF ---| chemosensitivity	36361620	10.3390/ijms232112829
negative Regulation: BDNF ---| chemosensitivity	36361620	10.3390/ijms232112829
negative Regulation: AURKB ---| chemosensitivity	22283874	10.1186/1423-0127-19-9
negative Regulation: ADCYAP1 ---| chemosensitivity	23597836	10.1016/j.resp.2013.04.009
negative Regulation: ADCYAP1 ---| chemosensitivity	21691246	10.1203/PDR.0b013e318229474
negative Regulation: BAG3 ---| chemosensitivity	29938246	10.1016/j.jacbts.2017.09.00
negative Regulation: BAG3 ---| chemosensitivity	24992675	10.1016/j.ygyno.2014.06.024
negative Regulation: BAG3 ---| chemosensitivity	26655271	10.1016/j.canlet.2015.11.03
negative Regulation: BAG3 ---| chemosensitivity	27120977	10.3892/ijo.2016.3494
negative Regulation: BAG3 ---| chemosensitivity	24992675	10.1016/j.ygyno.2014.06.024
negative Regulation: BAG3 ---| chemosensitivity	24992675	10.1016/j.ygyno.2014.06.024
negative Regulation: S100A7 ---| chemosensitivity	28923839	10.1158/1541-7786.MCR-17-03
negative Regulation: ANGPTL4 ---| chemosensitivity	30342537	10.1186/s12943-018-0904-z
negative Regulation: ANGPTL4 ---| chemosensitivity	33040824	10.14715/cmb/2020.66.5.21
negative Regulation: CCN1 ---| chemosensitivity	17993234	10.1007/978-0-387-74039-3_6
negative Regulation: CCN1 ---| chemosensitivity	31145521	10.1111/cas.1408310.1111/ca
negative Regulation: CCN1 ---| chemosensitivity	33753993	10.7150/jca.4889110.7150/jc
negative Regulation: CD9 ---| chemosensitivity	31624373	10.1038/s41375-019-0593-7
negative Regulation: CD9 ---| chemosensitivity	31624373	10.1038/s41375-019-0593-710
negative Regulation: CD9 ---| chemosensitivity		10.1038/s41375-019-0593-7
negative Regulation: NRP1 ---| chemosensitivity	30824197	10.1016/j.molmed.2019.01.01
negative Regulation: NRP1 ---| chemosensitivity	21338642	10.1016/j.jhep.2011.01.033
negative Regulation: NRP1 ---| chemosensitivity	20087344	
negative Regulation: NRP1 ---| chemosensitivity	15956974	
negative Regulation: NRP1 ---| chemosensitivity	24992616	
negative Regulation: NRP1 ---| chemosensitivity	24992616	
negative Regulation: NRP1 ---| chemosensitivity	25392102	
negative Regulation: NRP1 ---| chemosensitivity	25392102	
negative Regulation: NRP1 ---| chemosensitivity	15956974	10.1038/sj.bjc.6602663
negative Regulation: NRP1 ---| chemosensitivity	20087344	10.1038/sj.bjc.660553910.10
negative Regulation: NRP1 ---| chemosensitivity	20087344	10.1038/sj.bjc.660553910.10
negative Regulation: NRP1 ---| chemosensitivity	24992616	
negative Regulation: NRP1 ---| chemosensitivity	24992616	
negative Regulation: SIAH2 ---| chemosensitivity	29400343	10.12659/MSM.908553
negative Regulation: SIAH2 ---| chemosensitivity	25997740	10.14348/molcells.2015.0051
negative Regulation: SIAH2 ---| chemosensitivity	29400343	10.12659/msm.908553
negative Regulation: CHEK1 ---| chemosensitivity	28888100	10.1016/j.neo.2017.08.002
negative Regulation: CHEK1 ---| chemosensitivity	30055507	10.1016/j.dnarep.2018.07.00
negative Regulation: CHEK1 ---| chemosensitivity		
negative Regulation: CHEK1 ---| chemosensitivity	22641286	10.3892/or.2012.1833
negative Regulation: CHEK1 ---| chemosensitivity	24138717	10.2174/1381612811319666075
negative Regulation: CHEK1 ---| chemosensitivity	24804869	10.1097/IGC.000000000000013
negative Regulation: CHEK1 ---| chemosensitivity	24804869	10.1097/IGC.000000000000013
negative Regulation: RASAL2 ---| chemosensitivity	34826200	10.1002/tox.2341810.1002/tc
negative Regulation: POLR1H ---| chemosensitivity	23893241	10.1038/onc.2013.297
negative Regulation: DNER ---| chemosensitivity	32811806	10.1038/s41419-020-02903-1
negative Regulation: DNER ---| chemosensitivity	32811806	10.1038/s41419-020-02903-1
negative Regulation: MAPK1 ---| chemosensitivity	30257357	10.1016/j.biopha.2018.08.13
negative Regulation: MAPK1 ---| chemosensitivity	17690186	10.2353/ajpath.2007.070010
negative Regulation: MAPK1 ---| chemosensitivity	30257357	10.1016/j.biopha.2018.08.13
negative Regulation: MAPK1 ---| chemosensitivity	17482503	10.1016/j.drup.2007.03.003
negative Regulation: MAPK1 ---| chemosensitivity	23073477	10.1016/j.canlet.2012.10.00
negative Regulation: MAPK1 ---| chemosensitivity	28849180	10.3892/or.2017.5908

negative Regulation: MAPK1 ---| chemosensitivity	31133044	10.1186/s13046-019-1226-8
negative Regulation: MAPK1 ---| chemosensitivity	32039627	10.4149/neo_2020_190604N486
negative Regulation: MAPK1 ---| chemosensitivity	24104394	10.1038/leu.2013.291
negative Regulation: MAPK1 ---| chemosensitivity	30257357	10.1016/j.biopha.2018.08.13
negative Regulation: MAPK1 ---| chemosensitivity	22179790	10.3892/ijo.2011.1303
negative Regulation: MAPK1 ---| chemosensitivity	22179790	10.3892/ijo.2011.1303
negative Regulation: MAPK1 ---| chemosensitivity	22179790	10.3892/ijo.2011.1303
negative Regulation: MAPK1 ---| chemosensitivity	26316041	10.3892/ijo.2015.3137
negative Regulation: MAPK1 ---| chemosensitivity	22179790	10.3892/ijo.2011.1303
negative Regulation: MAPK1 ---| chemosensitivity	22179790	10.3892/ijo.2011.1303
negative Regulation: MAPK1 ---| chemosensitivity		
negative Regulation: MMP1 ---| chemosensitivity	35586209	10.1155/2022/3249766
negative Regulation: WNT5A ---| chemosensitivity	27612668	10.1016/j.bbamcr.2016.09.00
negative Regulation: WNT5A ---| chemosensitivity	27077077	10.1016/j.gendis.2015.12.00
negative Regulation: WNT5A ---| chemosensitivity	28538104	10.1139/bcb-2016-0216
negative Regulation: WNT5A ---| chemosensitivity	29531296	10.1038/s41419-018-0343-1
negative Regulation: WNT5A ---| chemosensitivity	21270611	10.1097/IGC.0b013e31820aaad
negative Regulation: WNT5A ---| chemosensitivity	21270611	10.1097/IGC.0b013e31820aaad
negative Regulation: WNT5A ---| chemosensitivity	21270611	10.1097/IGC.0b013e31820aaad
negative Regulation: WNT5A ---| chemosensitivity	21270611	10.1097/IGC.0b013e31820aaad
negative Regulation: WNT5A ---| chemosensitivity	24606488	10.7314/APJCP.2014.15.3.148
negative Regulation: WNT5A ---| chemosensitivity	24785108	
negative Regulation: WNT5A ---| chemosensitivity	21270611	10.1097/IGC.0b013e31820aaad
negative Regulation: WNT5A ---| chemosensitivity	21270611	10.1097/IGC.0b013e31820aaad
negative Regulation: SHFM1 ---| chemosensitivity	34031538	10.1038/s41374-021-00613-6
negative Regulation: SHFM1 ---| chemosensitivity	34031538	10.1038/s41374-021-00613-6
negative Regulation: SHFM1 ---| chemosensitivity	34031538	10.1038/s41374-021-00613-6
negative Regulation: SHFM1 ---| chemosensitivity	34031538	10.1038/s41374-021-00613-6
negative Regulation: SHFM1 ---| chemosensitivity	34031538	10.1038/s41374-021-00613-6
negative Regulation: FAM83A ---| chemosensitivity	34931434	10.1002/tox.2343510.1002/tc
negative Regulation: ABCE1 ---| chemosensitivity	25744244	10.1007/s12032-015-0557-3
negative Regulation: KIF14 ---| chemosensitivity	30404039	10.1016/j.bbadis.2018.10.03
negative Regulation: KIF14 ---| chemosensitivity	24784001	10.1016/j.neo.2014.03.008
negative Regulation: KIF14 ---| chemosensitivity	24784001	10.1016/j.neo.2014.03.008
negative Regulation: KIF14 ---| chemosensitivity	23479679	10.1158/1078-0432.CCR-13-00
negative Regulation: KIF14 ---| chemosensitivity	24784001	10.1016/j.neo.2014.03.00810
negative Regulation: KIF14 ---| chemosensitivity	33380832	10.2147/CMAR.S28536710.2147
negative Regulation: KIF14 ---| chemosensitivity	33380832	10.2147/CMAR.S28536710.2147
negative Regulation: KIF14 ---| chemosensitivity	35799605	10.1155/2022/7257738
negative Regulation: LGR5 ---| chemosensitivity	30792218	10.1136/jim-2018-000934
negative Regulation: LGR5 ---| chemosensitivity	31849331	10.1186/s12943-019-1116-x
negative Regulation: LGR5 ---| chemosensitivity	24789370	10.3892/ijmm.2014.1752
negative Regulation: LGR5 ---| chemosensitivity	24789370	10.3892/ijmm.2014.1752
negative Regulation: LGR5 ---| chemosensitivity	24789370	10.3892/ijmm.2014.1752
negative Regulation: LGR5 ---| chemosensitivity	24789370	10.3892/ijmm.2014.175210.38
negative Regulation: AHR ---| chemosensitivity	32877761	10.1016/j.semcancer.2020.08
negative Regulation: AHR ---| chemosensitivity	32877761	10.1016/j.semcancer.2020.08
negative Regulation: CASC11 ---| chemosensitivity	31181314	10.1016/j.gene.2019.06.011
negative Regulation: MIR222 ---| chemosensitivity	26796268	10.1016/j.biopha.2015.12.00
negative Regulation: MIR222 ---| chemosensitivity	28887606	10.1007/s10616-017-0134-z
negative Regulation: MIR222 ---| chemosensitivity	25474084	10.3390/ijms15122212810.339
negative Regulation: MIR222 ---| chemosensitivity	25474084	10.3390/ijms15122212810.339
negative Regulation: MIR222 ---| chemosensitivity		
negative Regulation: MIR222 ---| chemosensitivity		10.12659/MSM.913325

negative Regulation: EFNB2 ---| chemosensitivity	26494468	10.1038/cdd.2015.133
negative Regulation: EFNB2 ---| chemosensitivity	26494468	10.1038/cdd.2015.13310.1038
negative Regulation: BIRC6 ---| chemosensitivity	32922121	10.3906/biy-1912-71
negative Regulation: BIRC6 ---| chemosensitivity	23836645	10.1073/pnas.1300415110
negative Regulation: BIRC6 ---| chemosensitivity	22027771	
negative Regulation: BIRC6 ---| chemosensitivity	25216531	10.18632/oncotarget.2293
negative Regulation: BIRC6 ---| chemosensitivity	25216531	10.18632/oncotarget.2293
negative Regulation: BIRC6 ---| chemosensitivity	26191375	10.4172/1948-5956.1000335
negative Regulation: BIRC6 ---| chemosensitivity	28358418	10.3892/or.2017.553510.3892
negative Regulation: PART1 ---| chemosensitivity	34030482	10.1089/dna.2021.0059
negative Regulation: USP1 ---| chemosensitivity	29731868	10.3892/ol.2018.818010.3892
negative Regulation: SNHG14 ---| chemosensitivity	31121484	10.1016/j.biopha.2019.10899
negative Regulation: FZD1 ---| chemosensitivity	29058015	10.1007/s00018-017-2685-8
negative Regulation: SEMA4D ---| chemosensitivity	32888127	10.1007/s11033-020-05761-4
negative Regulation: SEMA4D ---| chemosensitivity	32888127	10.1007/s11033-020-05761-41
negative Regulation: SEMA4D ---| chemosensitivity	32888127	10.1007/s11033-020-05761-41
negative Regulation: SEMA4D ---| chemosensitivity	33404909	10.1007/s11033-020-06099-71
negative Regulation: MYC ---| chemosensitivity	28089283	10.1016/j.clbc.2016.12.005
negative Regulation: MYC ---| chemosensitivity	28089283	10.1016/j.clbc.2016.12.005
negative Regulation: MYC ---| chemosensitivity	16938463	10.1016/j.semcancer.2006.08
negative Regulation: MYC ---| chemosensitivity	26211592	10.1016/j.biopha.2015.05.01
negative Regulation: MYC ---| chemosensitivity	27233476	10.1016/j.canlet.2016.05.02
negative Regulation: MYC ---| chemosensitivity	19134217	
negative Regulation: MYC ---| chemosensitivity	19134217	
negative Regulation: MYC ---| chemosensitivity	10551811	
negative Regulation: MYC ---| chemosensitivity	28184929	10.3892/or.2017.5432
negative Regulation: MYC ---| chemosensitivity	30459529	10.1186/s12935-018-0674-0
negative Regulation: MYC ---| chemosensitivity	31761897	10.12659/MSM.917345
negative Regulation: MYC ---| chemosensitivity	31761897	10.12659/MSM.917345
negative Regulation: MYC ---| chemosensitivity	24303084	10.1371/journal.pone.008247
negative Regulation: MYC ---| chemosensitivity	25341931	
negative Regulation: MYC ---| chemosensitivity	26528706	10.1038/bjc.2015.305
negative Regulation: MYC ---| chemosensitivity	10961689	
negative Regulation: MYC ---| chemosensitivity	17159602	10.1097/CAD.0b013e328010942
negative Regulation: MYC ---| chemosensitivity	26211592	10.1016/j.biopha.2015.05.01
negative Regulation: MYC ---| chemosensitivity	27793037	10.18632/oncotarget.1291110
negative Regulation: MYC ---| chemosensitivity	28089283	10.1016/j.clbc.2016.12.0051
negative Regulation: MYC ---| chemosensitivity	31761897	10.12659/MSM.91734510.12659
negative Regulation: MYC ---| chemosensitivity		10.12659/MSM.917345
negative Regulation: MYC ---| chemosensitivity		10.12659/MSM.917345
negative Regulation: MYC ---| chemosensitivity	34803511	10.7150/ijbs.62236
negative Regulation: SIRT2 ---| chemosensitivity	25915617	10.1371/journal.pone.012467
negative Regulation: SIRT2 ---| chemosensitivity	33566315	10.1007/s11845-021-02516-31
negative Regulation: SIRT2 ---| chemosensitivity	33566315	10.1007/s11845-021-02516-31
negative Regulation: SIRT2 ---| chemosensitivity	33566315	10.1007/s11845-021-02516-31
negative Regulation: SIRT2 ---| chemosensitivity	33566315	10.1007/s11845-021-02516-3
negative Regulation: SIRT2 ---| chemosensitivity	33566315	10.1007/s11845-021-02516-3
negative Regulation: SIRT2 ---| chemosensitivity	33566315	10.1007/s11845-021-02516-3
negative Regulation: HMOX1 ---| chemosensitivity	29524413	10.1016/j.bbrc.2018.03.030
negative Regulation: HMOX1 ---| chemosensitivity	30076913	10.1016/j.fct.2018.07.059
negative Regulation: HMOX1 ---| chemosensitivity	34020028	10.1016/j.semcancer.2021.05
negative Regulation: HMOX1 ---| chemosensitivity	22684019	10.1016/j.freeradbiomed.201
negative Regulation: HMOX1 ---| chemosensitivity	24200599	10.1016/j.freeradbiomed.201
negative Regulation: HMOX1 ---| chemosensitivity	24413389	10.1097/CAD.000000000000006

negative Regulation: HMOX1 ---|	chemosensitivity	18006113	10.1016/j.lungcan.2007.09.0
negative Regulation: HMOX1 ---|	chemosensitivity	26801320	10.1007/s10495-016-1216-710
negative Regulation: HMOX1 ---|	chemosensitivity	29537718	10.1111/pin.1265710.1111/pi
negative Regulation: HMOX1 ---|	chemosensitivity	29620188	10.3892/ijo.2018.436310.389
negative Regulation: HMOX1 ---|	chemosensitivity	32663573	10.1016/j.lfs.2020.11808810
negative Regulation: HMOX1 ---|	chemosensitivity	31787334	10.1016/j.biomaterials.2019
negative Regulation: NIBAN2 ---|	chemosensitivity	31262713	10.1016/j.ebiom.2019.06.022
negative Regulation: NIBAN2 ---|	chemosensitivity	31262713	10.1016/j.ebiom.2019.06.022
negative Regulation: NIBAN2 ---|	chemosensitivity	28731151	10.3892/or.2017.582810.3892
negative Regulation: NIBAN2 ---|	chemosensitivity	31262713	10.1016/j.ebiom.2019.06.022
negative Regulation: NIBAN2 ---|	chemosensitivity	31262713	10.1016/j.ebiom.2019.06.022
negative Regulation: NIBAN2 ---|	chemosensitivity	34606782	10.1016/j.pharmthera.2021.1
negative Regulation: NIBAN2 ---|	chemosensitivity	33653247	10.2174/1566524021666210302
negative Regulation: NIBAN2 ---|	chemosensitivity	35761379	10.1186/s12967-022-03456-x
negative Regulation: TLR4 ---|	chemosensitivity	21167248	10.1016/j.humimm.2010.12.00
negative Regulation: TLR4 ---|	chemosensitivity	22648782	10.1007/s10565-012-9221-2
negative Regulation: TLR4 ---|	chemosensitivity	24977712	10.1371/journal.pone.010081
negative Regulation: TLR4 ---|	chemosensitivity	19200169	10.1111/j.1365-2362.2008.02
negative Regulation: TLR4 ---|	chemosensitivity	22648782	10.1007/s10565-012-9221-210
negative Regulation: TLR4 ---|	chemosensitivity	26276725	10.1016/j.canlet.2015.08.00
negative Regulation: TLR4 ---|	chemosensitivity	34110366	10.1093/abbs/gmab06610.1093
negative Regulation: TLR4 ---|	chemosensitivity		10.3390/molecules27030735
negative Regulation: CUL4A ---|	chemosensitivity		
negative Regulation: CUL4A ---|	chemosensitivity	30720173	10.26355/eurrev_201901_1687
negative Regulation: CUL4A ---|	chemosensitivity	26503734	10.3892/or.2015.4324
negative Regulation: CUL4A ---|	chemosensitivity	26503734	10.3892/or.2015.4324
negative Regulation: CUL4A ---|	chemosensitivity	26503734	10.3892/or.2015.4324
negative Regulation: CUL4A ---|	chemosensitivity	26503734	10.3892/or.2015.4324
negative Regulation: CUL4A ---|	chemosensitivity	26503734	10.3892/or.2015.4324
negative Regulation: CUL4A ---|	chemosensitivity	26969027	10.1111/jcmm.1281110.1111/j
negative Regulation: CUL4A ---|	chemosensitivity		
negative Regulation: CUL4A ---|	chemosensitivity		
negative Regulation: CUL4A ---|	chemosensitivity		
negative Regulation: CUL4A ---|	chemosensitivity		
negative Regulation: CUL4A ---|	chemosensitivity		
negative Regulation: CUL4A ---|	chemosensitivity	34786051	
negative Regulation: CUL4A ---|	chemosensitivity	34786051	
negative Regulation: BRD4 ---|	chemosensitivity	30728036	10.1186/s13046-019-1055-9
negative Regulation: BRD4 ---|	chemosensitivity	36352160	10.1007/s12032-022-01831-8
negative Regulation: MIR3151 ---|	chemosensitivity	26544558	10.1371/journal.pone.014242
negative Regulation: MIR640 ---|	chemosensitivity	35984538	10.1007/s10528-022-10264-x
negative Regulation: SATB1 ---|	chemosensitivity	30085330	10.3892/mmr.2018.9344
negative Regulation: CCAT2 ---|	chemosensitivity	27166155	10.1016/j.bbrc.2016.05.031
negative Regulation: CCAT2 ---|	chemosensitivity	24077681	10.18632/oncotarget.1292
negative Regulation: CCAT2 ---|	chemosensitivity	28531944	10.1016/j.biopha.2017.05.03
negative Regulation: CCAT2 ---|	chemosensitivity	28381160	10.1177/1010428317697553
negative Regulation: CCAT2 ---|	chemosensitivity	27938499	10.3727/096504016X147920983
negative Regulation: CCAT2 ---|	chemosensitivity	25992654	10.1371/journal.pone.011458
negative Regulation: CCAT2 ---|	chemosensitivity	25996380	10.1080/15384047.2015.10409
negative Regulation: CCAT2 ---|	chemosensitivity	35170195	10.1111/jcmm.1704110.1111/j
negative Regulation: CCAT2 ---|	chemosensitivity	35170195	10.1111/jcmm.1704110.1111/j
negative Regulation: HYOU1 ---|	chemosensitivity	33455947	10.14348/molcells.2020.0212
negative Regulation: CDCA5 ---|	chemosensitivity	33770322	10.1007/s10735-021-09969-x
negative Regulation: CDCA5 ---|	chemosensitivity	31324603	10.1016/j.ebiom.2019.07.030

negative Regulation: PPARGC1A ---| chemosensitivity	30244973	10.1016/j.cmet.2018.09.002
negative Regulation: MXD1 ---| chemosensitivity	32827577	10.1016/j.bbcan.2020.188405
negative Regulation: MIR6785 ---| chemosensitivity	33609542	10.1016/j.lfs.2021.119222
negative Regulation: MIR6785 ---| chemosensitivity	30551491	10.1016/j.biopha.2018.10.06
negative Regulation: MIR6785 ---| chemosensitivity	33152911	10.1016/j.biopha.2020.11072
negative Regulation: MIR6785 ---| chemosensitivity	32209033	10.2174/156652402066620022б
negative Regulation: MIR6785 ---| chemosensitivity	30551491	10.1016/j.biopha.2018.10.06
negative Regulation: GOLM1 ---| chemosensitivity	33538941	10.1007/s10863-021-09875-7
negative Regulation: GOLM1 ---| chemosensitivity	33538941	10.1007/s10863-021-09875-71
negative Regulation: NANOG ---| chemosensitivity	29198990	10.1016/j.cca.2017.11.037
negative Regulation: NANOG ---| chemosensitivity	33460610	10.1016/j.ejphar.2021.17387
negative Regulation: NANOG ---| chemosensitivity	33460610	10.1016/j.ejphar.2021.17387
negative Regulation: NANOG ---| chemosensitivity	20671266	10.2353/ajpath.2010.100316
negative Regulation: NANOG ---| chemosensitivity	23872274	10.1016/j.canlet.2013.07.00
negative Regulation: NANOG ---| chemosensitivity	26687709	10.1016/j.gene.2015.12.023
negative Regulation: NANOG ---| chemosensitivity	26939902	10.1007/s13277-016-5007-0
negative Regulation: NANOG ---| chemosensitivity	27884977	10.1042/BSR20160247
negative Regulation: NANOG ---| chemosensitivity	31165741	10.1038/s41419-019-1686-y
negative Regulation: NANOG ---| chemosensitivity	32899775	10.3390/ijms21186467
negative Regulation: NANOG ---| chemosensitivity	22714588	10.1007/s00432-012-1253-8
negative Regulation: NANOG ---| chemosensitivity	24647772	10.3892/ijo.2014.2347
negative Regulation: NANOG ---| chemosensitivity	24647772	10.3892/ijo.2014.2347
negative Regulation: NANOG ---| chemosensitivity		
negative Regulation: NANOG ---| chemosensitivity		
negative Regulation: NANOG ---| chemosensitivity	26676719	10.3892/or.2015.4483
negative Regulation: NANOG ---| chemosensitivity	26676719	10.3892/or.2015.4483
negative Regulation: NANOG ---| chemosensitivity	26676719	10.3892/or.2015.4483
negative Regulation: NANOG ---| chemosensitivity	26676719	10.3892/or.2015.4483
negative Regulation: NANOG ---| chemosensitivity	26936116	10.3892/or.2016.4639
negative Regulation: NANOG ---| chemosensitivity	26446457	10.1007/s13277-015-4155-y
negative Regulation: NANOG ---| chemosensitivity	19633292	10.1074/jbc.M109.02746610.1
negative Regulation: NANOG ---| chemosensitivity	21685938	10.1038/onc.2011.22210.1038
negative Regulation: NANOG ---| chemosensitivity	22714588	10.1007/s00432-012-1253-8
negative Regulation: NANOG ---| chemosensitivity	24647772	10.3892/ijo.2014.234710.389
negative Regulation: NANOG ---| chemosensitivity	25245189	10.18632/oncotarget.2298
negative Regulation: NANOG ---| chemosensitivity	26676719	10.3892/or.2015.448310.3892
negative Regulation: NANOG ---| chemosensitivity	34453639	10.1007/s10555-021-09979-x
negative Regulation: NANOG ---| chemosensitivity	35398618	10.1016/j.prp.2022.15386910
negative Regulation: NANOG ---| chemosensitivity	36209968	10.1016/j.bmcl.2022.129017
negative Regulation: PDZD7 ---| chemosensitivity	30786928	10.1186/s13046-019-1106-2
negative Regulation: PDZD7 ---| chemosensitivity	30786928	10.1186/s13046-019-1106-2
negative Regulation: PDZD7 ---| chemosensitivity	30786928	10.1186/s13046-019-1106-2
negative Regulation: PDZD7 ---| chemosensitivity	30786928	10.1186/s13046-019-1106-2
negative Regulation: PDZD7 ---| chemosensitivity	30786928	10.1186/s13046-019-1106-2
negative Regulation: PDZD7 ---| chemosensitivity	30786928	10.1186/s13046-019-1106-2
negative Regulation: PDZD7 ---| chemosensitivity	30786928	10.1186/s13046-019-1106-210
negative Regulation: SPHK1 ---| chemosensitivity	19240026	10.1074/jbc.M900735200
negative Regulation: SPHK1 ---| chemosensitivity	19240026	10.1074/jbc.M900735200
negative Regulation: SPHK1 ---| chemosensitivity	30347210	10.1016/j.pharmthera.2018.1
negative Regulation: SPHK1 ---| chemosensitivity	17669501	10.1016/j.pharmthera.2007.0
negative Regulation: SPHK1 ---| chemosensitivity	19240026	
negative Regulation: SPHK1 ---| chemosensitivity	19240026	
negative Regulation: SPHK1 ---| chemosensitivity	19240026	
negative Regulation: SPHK1 ---| chemosensitivity	27065335	10.1038/onc.2016.90

negative Regulation:	SPHK1	---|	chemosensitivity	27065335	10.1038/onc.2016.90
negative Regulation:	SPHK1	---|	chemosensitivity	27065335	10.1038/onc.2016.90
negative Regulation:	SPHK1	---|	chemosensitivity	27127108	
negative Regulation:	SPHK1	---|	chemosensitivity	28804221	10.1155/2017/7510496
negative Regulation:	SPHK1	---|	chemosensitivity	30607469	10.1007/s00210-018-01607-w
negative Regulation:	SPHK1	---|	chemosensitivity	31636343	10.1038/s41375-019-0577-7
negative Regulation:	SPHK1	---|	chemosensitivity	22469881	10.3892/or.2012.1743
negative Regulation:	SPHK1	---|	chemosensitivity	22812190	10.3727/096504012X133424637
negative Regulation:	SPHK1	---|	chemosensitivity	22833671	10.1074/jbc.M112.385765
negative Regulation:	SPHK1	---|	chemosensitivity	22984905	10.1089/lrb.2012.0010
negative Regulation:	SPHK1	---|	chemosensitivity	23232649	10.3892/ijo.2012.1733
negative Regulation:	SPHK1	---|	chemosensitivity	19240026	10.1074/jbc.M90073520010.10
negative Regulation:	SPHK1	---|	chemosensitivity	28502300	
negative Regulation:	SPHK1	---|	chemosensitivity	28502300	
negative Regulation:	SMO	---|	chemosensitivity	33743723	10.1186/s12967-021-02789-3
negative Regulation:	WASF1	---|	chemosensitivity	33558065	10.1016/j.pathol.2020.11.00
negative Regulation:	WASF1	---|	chemosensitivity	33558065	10.1016/j.pathol.2020.11.00
negative Regulation:	FOXM1	---|	chemosensitivity	32711110	10.1016/j.cellsig.2020.1097
negative Regulation:	FOXM1	---|	chemosensitivity	30017390	10.1016/j.cyto.2018.06.018
negative Regulation:	FOXM1	---|	chemosensitivity	31082791	10.1016/j.omtn.2019.04.008
negative Regulation:	FOXM1	---|	chemosensitivity	31082791	10.1016/j.omtn.2019.04.008
negative Regulation:	FOXM1	---|	chemosensitivity	31082791	10.1016/j.omtn.2019.04.008
negative Regulation:	FOXM1	---|	chemosensitivity	31082791	10.1016/j.omtn.2019.04.008
negative Regulation:	FOXM1	---|	chemosensitivity	23177020	10.1016/j.lungcan.2012.10.0
negative Regulation:	FOXM1	---|	chemosensitivity	27439614	10.1186/s12885-016-2542-4
negative Regulation:	FOXM1	---|	chemosensitivity	27415661	10.1159/000445653
negative Regulation:	FOXM1	---|	chemosensitivity	30992007	10.1186/s12964-019-0347-1
negative Regulation:	FOXM1	---|	chemosensitivity	31322278	10.3892/or.2019.7225
negative Regulation:	FOXM1	---|	chemosensitivity	33292277	10.1186/s12935-020-01631-y
negative Regulation:	FOXM1	---|	chemosensitivity	33311446	10.1038/s41419-020-03282-3
negative Regulation:	FOXM1	---|	chemosensitivity	33291076	10.18632/aging.202300
negative Regulation:	FOXM1	---|	chemosensitivity	33221995	10.1007/s00432-020-03438-w
negative Regulation:	FOXM1	---|	chemosensitivity	23912794	10.3892/or.2013.2654
negative Regulation:	FOXM1	---|	chemosensitivity	24148180	10.1186/1479-5876-11-265
negative Regulation:	FOXM1	---|	chemosensitivity	25760224	10.3892/mmr.2015.3469
negative Regulation:	FOXM1	---|	chemosensitivity	25760224	10.3892/mmr.2015.3469
negative Regulation:	FOXM1	---|	chemosensitivity	24314213	
negative Regulation:	FOXM1	---|	chemosensitivity	25760224	10.3892/mmr.2015.346910.389
negative Regulation:	FOXM1	---|	chemosensitivity	26560505	10.1111/cas.1284610.1111/ca
negative Regulation:	FOXM1	---|	chemosensitivity	27162541	10.7150/jca.1446110.7150/jc
negative Regulation:	FOXM1	---|	chemosensitivity	27439614	10.1186/s12885-016-2542-410
negative Regulation:	FOXM1	---|	chemosensitivity	31814893	
negative Regulation:	FOXM1	---|	chemosensitivity	682	10.1016/j.cyto.2018.06.018
negative Regulation:	FOXM1	---|	chemosensitivity		10.1186/s12964-019-0347-1
negative Regulation:	FOXM1	---|	chemosensitivity		10.3892/or.2019.7225
negative Regulation:	FOXM1	---|	chemosensitivity		10.1186/s12935-020-01631-y
negative Regulation:	FOXM1	---|	chemosensitivity	34812991	10.1007/s12032-021-01610-x
negative Regulation:	FOXM1	---|	chemosensitivity	34825342	10.1007/s13577-021-00650-9
negative Regulation:	BMI1	---|	chemosensitivity	22112692	10.1016/j.critrevonc.2011.1
negative Regulation:	BMI1	---|	chemosensitivity	28830551	10.1186/s12943-017-0711-y
negative Regulation:	BMI1	---|	chemosensitivity	29200967	10.1186/s12935-017-0481-z
negative Regulation:	BMI1	---|	chemosensitivity	24317363	10.3892/or.2013.2897
negative Regulation:	BMI1	---|	chemosensitivity	24317363	10.3892/or.2013.2897
negative Regulation:	BMI1	---|	chemosensitivity	24317363	10.3892/or.2013.2897

negative Regulation: BMI1 ---| chemosensitivity	25286028	10.1038/labinvest.2014.123
negative Regulation: BMI1 ---| chemosensitivity	25286028	10.1038/labinvest.2014.123
negative Regulation: BMI1 ---| chemosensitivity	25915207	10.1371/journal.pone.012320
negative Regulation: BMI1 ---| chemosensitivity	25915207	10.1371/journal.pone.012320
negative Regulation: BMI1 ---| chemosensitivity	18452707	10.1016/j.bbrc.2008.04.1171
negative Regulation: BMI1 ---| chemosensitivity	23205090	10.3892/ol.2012.805
negative Regulation: BMI1 ---| chemosensitivity	25286028	10.1038/labinvest.2014.1231
negative Regulation: BMI1 ---| chemosensitivity	26622537	10.3892/ol.2015.3361
negative Regulation: FLT4 ---| chemosensitivity	27931843	10.1016/j.critrevonc.2016.1
negative Regulation: FLT4 ---| chemosensitivity	29048623	10.3892/or.2017.5969
negative Regulation: FLT4 ---| chemosensitivity	29048623	10.3892/or.2017.5969
negative Regulation: FLT4 ---| chemosensitivity	34904812	10.1097/PPO.000000000000056
negative Regulation: XPA ---| chemosensitivity	32007918	10.1016/j.biopha.2020.10989
negative Regulation: XPA ---| chemosensitivity	32007918	10.1016/j.biopha.2020.10989
negative Regulation: ATG4D ---| chemosensitivity	31291988	10.1186/s13046-019-1287-8
negative Regulation: PLCG1 ---| chemosensitivity	28978037	10.18632/oncotarget.1863110
negative Regulation: PCAT1 ---| chemosensitivity	30530229	10.1016/j.biopha.2018.12.01
negative Regulation: PCAT1 ---| chemosensitivity	30530229	10.1016/j.biopha.2018.12.01
negative Regulation: PCAT1 ---| chemosensitivity	31273188	10.1038/s41419-019-1745-4
negative Regulation: PCAT1 ---| chemosensitivity	29314203	10.1002/cbf.331410.1002/cbf
negative Regulation: PCAT1 ---| chemosensitivity	33277833	
negative Regulation: AGGF1 ---| chemosensitivity	33168501	10.3969/j.issn.1673-4254.20
negative Regulation: MIR19A ---| chemosensitivity	33023313	10.1177/0963689720962460
negative Regulation: MIR19A ---| chemosensitivity	30655782	10.3892/ol.2018.959210.3892
negative Regulation: MIR19A ---| chemosensitivity	30655782	10.3892/ol.2018.959210.3892
negative Regulation: MIR19A ---| chemosensitivity	30655782	10.3892/ol.2018.959210.3892
negative Regulation: TRIM29 ---| chemosensitivity	30876939	10.1016/j.lfs.2019.03.028
negative Regulation: TRIM29 ---| chemosensitivity	30876939	10.1016/j.lfs.2019.03.028
negative Regulation: TRIM29 ---| chemosensitivity	33930453	10.1016/j.pharmthera.2021.1
negative Regulation: TRIM29 ---| chemosensitivity	28098872	10.3892/or.2017.5364
negative Regulation: TRIM29 ---| chemosensitivity	28098872	10.3892/or.2017.5364
negative Regulation: TRIM29 ---| chemosensitivity	32901838	10.3892/mmr.2020.11473
negative Regulation: TRIM29 ---| chemosensitivity	26273332	10.1111/1759-7714.1213010.1
negative Regulation: SNHG22 ---| chemosensitivity	32306838	10.1080/15384101.2020.17494
negative Regulation: POU4F1 ---| chemosensitivity	32988584	10.1016/j.bbrc.2020.09.003
negative Regulation: POU4F1 ---| chemosensitivity	32988584	10.1016/j.bbrc.2020.09.0031
negative Regulation: MFGE8 ---| chemosensitivity	32324268	10.1002/jcp.2971210.1002/jc
negative Regulation: FOXP3 ---| chemosensitivity	28935177	10.1016/j.prp.2017.09.004
negative Regulation: FOXP3 ---| chemosensitivity	28935177	10.1016/j.prp.2017.09.004
negative Regulation: FOXP3 ---| chemosensitivity	28935177	10.1016/j.prp.2017.09.004
negative Regulation: FOXP3 ---| chemosensitivity	28935177	10.1016/j.prp.2017.09.004
negative Regulation: FOXP3 ---| chemosensitivity	30360388	10.3390/ijms19103279
negative Regulation: FOXP3 ---| chemosensitivity	28935177	10.1016/j.prp.2017.09.00410
negative Regulation: FOXP3 ---| chemosensitivity	28935177	10.1016/j.prp.2017.09.00410
negative Regulation: FOXP3 ---| chemosensitivity	35401877	10.1155/2022/4534080
negative Regulation: FOXP3 ---| chemosensitivity	36235242	10.3390/molecules27196706
negative Regulation: MASTL ---| chemosensitivity	30445205	10.1016/j.canlet.2018.11.01
negative Regulation: MASTL ---| chemosensitivity	30068336	10.1186/s12943-018-0848-3
negative Regulation: r_Mir29b-3 ---| chemosensitivi	30747209	10.3892/ijo.2019.4695
negative Regulation: SLPI ---| chemosensitivity	36595102	10.1007/s12672-022-00535-9
negative Regulation: FOXA1 ---| chemosensitivity	29665655	10.1016/j.biopha.2018.01.07
negative Regulation: FOXA1 ---| chemosensitivity		
negative Regulation: FOXA1 ---| chemosensitivity		
negative Regulation: FOXA1 ---| chemosensitivity		

negative Regulation: FOXA1 ---| chemosensitivity		
negative Regulation: FOXA1 ---| chemosensitivity		
negative Regulation: FOXA1 ---| chemosensitivity		
negative Regulation: FOXA1 ---| chemosensitivity		
negative Regulation: FOXA1 ---| chemosensitivity	29072684	10.1038/cddis.2017.553
negative Regulation: FOXA1 ---| chemosensitivity	29442000	10.1691/ph.2016.676410.1691
negative Regulation: FOXA1 ---| chemosensitivity	31317696	
negative Regulation: FOXA1 ---| chemosensitivity	36230619	10.3390/cancers14194699
negative Regulation: NSD2 ---| chemosensitivity	30683853	10.1038/s41419-019-1347-1
negative Regulation: NSD2 ---| chemosensitivity	30683853	10.1038/s41419-019-1347-1
negative Regulation: NSD2 ---| chemosensitivity	30683853	10.1038/s41419-019-1347-1
negative Regulation: NSD2 ---| chemosensitivity	30683853	10.1038/s41419-019-1347-110
negative Regulation: NSD2 ---| chemosensitivity	33469000	10.1038/s41420-021-00402-61
negative Regulation: NSD2 ---| chemosensitivity	33469000	10.1038/s41420-021-00402-61
negative Regulation: NSD2 ---| chemosensitivity	35748228	10.14348/molcells.2022.0014
negative Regulation: CXCR2 ---| chemosensitivity	31731888	10.1080/15384101.2019.16894
negative Regulation: CXCR2 ---| chemosensitivity	21565970	10.1530/ERC-10-0343
negative Regulation: PAK5 ---| chemosensitivity	23877225	10.1007/s11010-013-1767-710
negative Regulation: RBM24 ---| chemosensitivity	34558639	10.3892/ijo.2021.526710.389
negative Regulation: UBE2C ---| chemosensitivity	31875843	10.1016/j.bbrc.2019.12.058
negative Regulation: UBE2C ---| chemosensitivity	30146342	10.1016/j.ebiom.2018.08.001
negative Regulation: UBE2C ---| chemosensitivity	30146342	10.1016/j.ebiom.2018.08.001
negative Regulation: UBE2C ---| chemosensitivity	29303411	10.1080/15384047.2017.14169
negative Regulation: UBE2C ---| chemosensitivity	32192022	10.3390/ijms21062041
negative Regulation: UBE2C ---| chemosensitivity	22760214	10.1007/s00432-012-1275-2
negative Regulation: UBE2C ---| chemosensitivity	22760214	10.1007/s00432-012-1275-210
negative Regulation: UBE2C ---| chemosensitivity	23275705	10.6026/9732063008104710.60
negative Regulation: UBE2C ---| chemosensitivity	25739083	10.3390/ijms1603469810.3390
negative Regulation: PTPMT1 ---| chemosensitivity	24709986	10.1371/journal.pone.009389
negative Regulation: PTPMT1 ---| chemosensitivity	24709986	10.1371/journal.pone.009389
negative Regulation: PTPMT1 ---| chemosensitivity	24709986	10.1371/journal.pone.009389
negative Regulation: LGALS3 ---| chemosensitivity	19124005	10.1016/j.bbrc.2008.12.153
negative Regulation: LGALS3 ---| chemosensitivity	27012551	10.1007/s13277-016-5004-3
negative Regulation: LGALS3 ---| chemosensitivity	28870921	10.21873/anticanres.11909
negative Regulation: LGALS3 ---| chemosensitivity	30626779	10.2131/jts.44.47
negative Regulation: LGALS3 ---| chemosensitivity	21368866	10.1038/cddis.2010.79
negative Regulation: LGALS3 ---| chemosensitivity	19199318	10.1002/path.251010.1002/pa
negative Regulation: LGALS3 ---| chemosensitivity	28146425	10.18632/oncotarget.1483910
negative Regulation: TCF3 ---| chemosensitivity	26961878	10.1074/jbc.M115.696609
negative Regulation: STK35 ---| chemosensitivity	33117809	10.3389/fcell.2020.58269510
negative Regulation: ATG3 ---| chemosensitivity	29966607	10.1016/j.lfs.2018.06.034
negative Regulation: SERPINB3 ---| chemosensitivity	30825353	10.1111/cas.1398610.1111/ca
negative Regulation: G3BP1 ---| chemosensitivity	33000280	10.3892/mmr.2020.11527
negative Regulation: TIGAR ---| chemosensitivity	33510458	10.1038/s41401-020-00588-y
negative Regulation: RIPK4 ---| chemosensitivity	24519546	10.5301/jbm.5000073
negative Regulation: CPE ---| chemosensitivity	25374060	10.1007/s13277-014-2564-y10
negative Regulation: FAM201A ---| chemosensitivity	36067543	10.1016/j.tranon.2022.10149
negative Regulation: ITGB3 ---| chemosensitivity	30563517	10.1186/s12943-018-0924-8
negative Regulation: AKR1B10 ---| chemosensitivity	24656094	10.1016/j.humpath.2013.12.0
negative Regulation: MTOR ---| chemosensitivity	32841911	10.1016/j.biomaterials.2020
negative Regulation: MTOR ---| chemosensitivity	33839294	10.1016/j.semcancer.2021.04
negative Regulation: MTOR ---| chemosensitivity	32422572	10.1016/j.tranon.2020.10076
negative Regulation: MTOR ---| chemosensitivity	18395956	10.1016/j.ejogrb.2007.12.02
negative Regulation: MTOR ---| chemosensitivity	16540312	10.1016/j.ejca.2005.12.018

negative Regulation: MTOR ---|	chemosensitivity	30419950	10.1186/s13046-018-0947-4
negative Regulation: MTOR ---|	chemosensitivity		
negative Regulation: MTOR ---|	chemosensitivity	32422572	10.1016/j.tranon.2020.10076
negative Regulation: MTOR ---|	chemosensitivity	32608202	10.3349/YMJ.2020.61.7.587
negative Regulation: MTOR ---|	chemosensitivity	32927648	10.3390/molecules25184136
negative Regulation: MTOR ---|	chemosensitivity	22785211	10.4161/cbt.20838
negative Regulation: MTOR ---|	chemosensitivity	22873368	
negative Regulation: MTOR ---|	chemosensitivity	20878074	10.3892/ijo-00000778
negative Regulation: MTOR ---|	chemosensitivity	23818585	10.1073/pnas.1220898110
negative Regulation: MTOR ---|	chemosensitivity		
negative Regulation: MTOR ---|	chemosensitivity	16540312	10.1016/j.ejca.2005.12.018
negative Regulation: MTOR ---|	chemosensitivity	18824293	10.1016/j.canlet.2008.08.01
negative Regulation: MTOR ---|	chemosensitivity	19694375	
negative Regulation: MTOR ---|	chemosensitivity	22785211	10.4161/cbt.2083810.4161/cb
negative Regulation: MTOR ---|	chemosensitivity	23433853	10.1016/j.biopha.2012.11.00
negative Regulation: MTOR ---|	chemosensitivity	24444656	10.1016/j.molonc.2013.12.01
negative Regulation: MTOR ---|	chemosensitivity	27301639	10.1016/j.bbrc.2016.06.0571
negative Regulation: MTOR ---|	chemosensitivity	33179318	10.1111/cas.1473310.1111/ca
negative Regulation: MTOR ---|	chemosensitivity	34358725	10.1016/j.jtho.2021.07.018
negative Regulation: MTOR ---|	chemosensitivity		10.7150/IJBS.61175
negative Regulation: MTOR ---|	chemosensitivity	34358725	10.1016/j.jtho.2021.07.018
negative Regulation: MTOR ---|	chemosensitivity	36396656	10.1038/s41467-022-34890-6
negative Regulation: MTOR ---|	chemosensitivity	36396656	10.1038/s41467-022-34890-6
negative Regulation: HOXB4 ---|	chemosensitivity	29660518	10.1016/j.gene.2018.04.033
negative Regulation: HOXB4 ---|	chemosensitivity	27779650	10.3892/ijo.2016.373810.389
negative Regulation: ATF6 ---|	chemosensitivity	30413206	10.1186/s13046-018-0935-8
negative Regulation: PRKCH ---|	chemosensitivity	24784886	10.1016/j.canlet.2014.04.02
negative Regulation: PRKCH ---|	chemosensitivity	25399563	10.1042/BST20140182
negative Regulation: PRKCH ---|	chemosensitivity		10.1042/BST20140182
negative Regulation: MED19 ---|	chemosensitivity	25735376	10.7314/apjcp.2015.16.3.875
negative Regulation: MED19 ---|	chemosensitivity	30161287	10.1002/jcb.2740610.1002/jc
negative Regulation: MED19 ---|	chemosensitivity	35637945	10.7150/ijbs.73504
negative Regulation: NTRK1 ---|	chemosensitivity	27264679	10.1016/j.bbcan.2016.05.003
negative Regulation: NTRK1 ---|	chemosensitivity	31485624	10.3892/or.2019.7296
negative Regulation: NTRK1 ---|	chemosensitivity	25785038	
negative Regulation: NTRK1 ---|	chemosensitivity	25785038	
negative Regulation: NTRK1 ---|	chemosensitivity		10.3892/or.2019.7296
negative Regulation: BAG1 ---|	chemosensitivity	34402163	10.1111/jcmm.1682210.1111/j
negative Regulation: RBM3 ---|	chemosensitivity	19900510	10.1016/j.bbcan.2009.11.001
negative Regulation: RBM3 ---|	chemosensitivity	27147467	10.1007/s00018-016-2253-7
negative Regulation: RBM3 ---|	chemosensitivity	23673116	10.3760/cma.j.issn.0366-699
negative Regulation: RBM3 ---|	chemosensitivity	35109796	10.1186/s12885-021-09168-7
negative Regulation: MSI1 ---|	chemosensitivity	30362859	10.1080/15476286.2018.15396
negative Regulation: MSI1 ---|	chemosensitivity	32786108	10.1002/jcb.2983310.1002/jc
negative Regulation: SOD2 ---|	chemosensitivity	31364751	10.3892/or.2019.7252
negative Regulation: SOD2 ---|	chemosensitivity	31364751	10.3892/or.2019.7252
negative Regulation: SOD2 ---|	chemosensitivity	23964924	10.1089/ars.2013.5295
negative Regulation: SOD2 ---|	chemosensitivity	23964924	10.1089/ars.2013.5295
negative Regulation: SOD2 ---|	chemosensitivity	23964924	10.1089/ars.2013.5295
negative Regulation: SOD2 ---|	chemosensitivity		10.3892/or.2019.7252
negative Regulation: SOD2 ---|	chemosensitivity		10.3892/or.2019.7252
negative Regulation: SOD2 ---|	chemosensitivity	23964924	10.1089/ars.2013.5295
negative Regulation: SOD2 ---|	chemosensitivity		
negative Regulation: LEF1-AS1 ---|	chemosensitivity	32786108	10.1002/jcb.2983310.1002/jc

negative Regulation: TRIM14 ---|	chemosensitivity		10.1038/s41388-018-0344-710
negative Regulation: UIMC1 ---|	chemosensitivity	22573342	10.1007/s10620-012-2132-5
negative Regulation: UIMC1 ---|	chemosensitivity	22573342	10.1007/s10620-012-2132-510
negative Regulation: UIMC1 ---|	chemosensitivity	27998448	10.3760/cma.j.issn.0253-376
negative Regulation: UIMC1 ---|	chemosensitivity	30705591	10.2147/OTT.S18698110.2147/
negative Regulation: L1CAM ---|	chemosensitivity	22210381	10.1016/j.canlet.2011.12.03
negative Regulation: L1CAM ---|	chemosensitivity	22472175	10.1158/1078-0432.CCR-11-27
negative Regulation: L1CAM ---|	chemosensitivity	22472175	10.1158/1078-0432.CCR-11-27
negative Regulation: L1CAM ---|	chemosensitivity	22472175	10.1158/1078-0432.CCR-11-27
negative Regulation: WFDC2 ---|	chemosensitivity		10.31083/J.EJGO.2020.06.223
negative Regulation: DEPTOR ---|	chemosensitivity	28599191	10.1016/j.leukres.2017.06.0
negative Regulation: DEPTOR ---|	chemosensitivity	33184290	10.1038/s41419-020-03185-3
negative Regulation: DEPTOR ---|	chemosensitivity	23503641	10.3892/ijmm.2013.1299
negative Regulation: DEPTOR ---|	chemosensitivity	23503641	10.3892/ijmm.2013.1299
negative Regulation: DEPTOR ---|	chemosensitivity	23503641	10.3892/ijmm.2013.1299
negative Regulation: DEPTOR ---|	chemosensitivity	23503641	10.3892/ijmm.2013.1299
negative Regulation: DEPTOR ---|	chemosensitivity	23613505	10.1177/0300060513480920
negative Regulation: DEPTOR ---|	chemosensitivity	23613505	10.1177/0300060513480920
negative Regulation: DEPTOR ---|	chemosensitivity	23613505	10.1177/0300060513480920
negative Regulation: DEPTOR ---|	chemosensitivity	23503641	10.3892/ijmm.2013.129910.38
negative Regulation: DEPTOR ---|	chemosensitivity	23503641	10.3892/ijmm.2013.129910.38
negative Regulation: DEPTOR ---|	chemosensitivity	23503641	10.3892/ijmm.2013.129910.38
negative Regulation: DEPTOR ---|	chemosensitivity	33184290	10.1038/s41419-020-03185-31
negative Regulation: SNHG16 ---|	chemosensitivity	33390845	10.7150/ijbs.50730
negative Regulation: MIR362 ---|	chemosensitivity	33989708	10.1016/j.canlet.2021.05.00
negative Regulation: FURIN ---|	chemosensitivity	35917012	10.1245/s10434-022-11945-y
negative Regulation: EZH2 ---|	chemosensitivity	27622325	10.1016/j.bbrc.2016.09.040
negative Regulation: EZH2 ---|	chemosensitivity	27622325	10.1016/j.bbrc.2016.09.040
negative Regulation: EZH2 ---|	chemosensitivity	29567536	10.1016/j.biopha.2018.03.07
negative Regulation: EZH2 ---|	chemosensitivity	28196596	10.1016/j.ccell.2017.01.006
negative Regulation: EZH2 ---|	chemosensitivity	33360300	10.1016/j.tranon.2020.10098
negative Regulation: EZH2 ---|	chemosensitivity	33360300	10.1016/j.tranon.2020.10098
negative Regulation: EZH2 ---|	chemosensitivity	28272374	10.3390/molecules22030417
negative Regulation: EZH2 ---|	chemosensitivity	29035367	10.1038/nm.4415
negative Regulation: EZH2 ---|	chemosensitivity	30786928	10.1186/s13046-019-1106-2
negative Regulation: EZH2 ---|	chemosensitivity	33743723	10.1186/s12967-021-02789-3
negative Regulation: EZH2 ---|	chemosensitivity	33743723	10.1186/s12967-021-02789-3
negative Regulation: EZH2 ---|	chemosensitivity	23603558	10.1016/j.canlet.2013.04.01
negative Regulation: EZH2 ---|	chemosensitivity	25400745	
negative Regulation: EZH2 ---|	chemosensitivity	29221202	10.18632/oncotarget.2199610
negative Regulation: EZH2 ---|	chemosensitivity	33240432	10.3892/ol.2020.1228710.389
negative Regulation: EZH2 ---|	chemosensitivity	33240432	10.3892/ol.2020.1228710.389
negative Regulation: EZH2 ---|	chemosensitivity	33240432	10.3892/ol.2020.1228710.389
negative Regulation: EZH2 ---|	chemosensitivity	34904810	10.1097/PPO.000000000000055
negative Regulation: EZH2 ---|	chemosensitivity	35216113	10.3390/ijms23042001
negative Regulation: PIK3CB ---|	chemosensitivity	22528234	10.1007/s11596-012-0049-z10
negative Regulation: FOXD2-AS1 ---|	chemosensitivit	34873418	10.1155/2021/967476110.1155
negative Regulation: MCL1 ---|	chemosensitivity	27847320	10.1016/j.bbrc.2016.11.049
negative Regulation: MCL1 ---|	chemosensitivity	12787138	10.1046/j.1523-1747.2003.12
negative Regulation: MCL1 ---|	chemosensitivity	23954445	10.1016/j.bbamcr.2013.08.00
negative Regulation: MCL1 ---|	chemosensitivity	28343875	10.1016/j.bmcl.2017.03.028
negative Regulation: MCL1 ---|	chemosensitivity	18601905	10.1016/j.bbrc.2008.06.098
negative Regulation: MCL1 ---|	chemosensitivity	20004446	10.1016/j.surg.2009.10.033
negative Regulation: MCL1 ---|	chemosensitivity	20004446	10.1016/j.surg.2009.10.033

negative Regulation: MCL1 ---| chemosensitivity	12787138	
negative Regulation: MCL1 ---| chemosensitivity	30221675	10.3892/ijo.2018.4557
negative Regulation: MCL1 ---| chemosensitivity	23257783	10.1038/leu.2012.367
negative Regulation: MCL1 ---| chemosensitivity	24223823	10.1371/journal.pone.007857
negative Regulation: MCL1 ---| chemosensitivity	24982331	
negative Regulation: MCL1 ---| chemosensitivity	18297287	10.1007/s00280-008-0697-710
negative Regulation: MCL1 ---| chemosensitivity	18297287	10.1007/s00280-008-0697-710
negative Regulation: MCL1 ---| chemosensitivity	18297287	10.1007/s00280-008-0697-710
negative Regulation: MCL1 ---| chemosensitivity	27049076	
negative Regulation: MCL1 ---| chemosensitivity	27686452	10.1007/s11010-016-2827-6
negative Regulation: MCL1 ---| chemosensitivity	31754782	10.1007/s00441-019-03105-81
negative Regulation: MCL1 ---| chemosensitivity	746	10.1016/j.bmcl.2017.03.028
negative Regulation: MCL1 ---| chemosensitivity	34781999	10.1186/s13046-021-02149-5
negative Regulation: MCL1 ---| chemosensitivity	35031927	10.1007/s11033-021-07040-2
negative Regulation: RECQL4 ---| chemosensitivity	26690729	10.1002/path.468110.1002/pa
negative Regulation: PPFIA4 ---| chemosensitivity	32137820	10.1093/annonc/mdy303.054
negative Regulation: PPFIA4 ---| chemosensitivity	30842147	10.21873/anticanres.13227
negative Regulation: PPFIA4 ---| chemosensitivity	30842147	10.21873/anticanres.13227
negative Regulation: PPFIA4 ---| chemosensitivity	30842147	10.21873/anticanres.13227
negative Regulation: PPFIA4 ---| chemosensitivity	30842147	10.21873/anticanres.1322710
negative Regulation: IDO1 ---| chemosensitivity	25955018	10.1371/journal.pone.012615
negative Regulation: NR1I2 ---| chemosensitivity	27085140	10.1016/j.bbagrm.2016.03.01
negative Regulation: NR1I2 ---| chemosensitivity	20691230	10.1016/j.addr.2010.07.008
negative Regulation: NR1I2 ---| chemosensitivity	26808161	10.1016/j.pharmthera.2016.0
negative Regulation: NR1I2 ---| chemosensitivity	23413096	
negative Regulation: DLEU1 ---| chemosensitivity	35152365	10.1007/s11010-022-04368-6
negative Regulation: TRIM28 ---| chemosensitivity	28381187	10.1177/1010428317695919
negative Regulation: TRIM28 ---| chemosensitivity	28381187	10.1177/1010428317695919
negative Regulation: TRIM28 ---| chemosensitivity	28381187	10.1177/1010428317695919
negative Regulation: SMG1 ---| chemosensitivity	25760059	10.3892/mmr.2015.3434
negative Regulation: SMG1 ---| chemosensitivity	25760059	10.3892/mmr.2015.3434
negative Regulation: TFEB ---| chemosensitivity	28603284	10.1038/aps.2017.25
negative Regulation: SOX2 ---| chemosensitivity	31412296	10.1016/j.semcancer.2019.08
negative Regulation: SOX2 ---| chemosensitivity	30272330	10.3892/or.2018.6735
negative Regulation: SOX2 ---| chemosensitivity	30272330	10.3892/or.2018.6735
negative Regulation: SOX2 ---| chemosensitivity	30272330	10.3892/or.2018.6735
negative Regulation: SOX2 ---| chemosensitivity	30272330	10.3892/or.2018.6735
negative Regulation: SOX2 ---| chemosensitivity	32439916	10.1038/s41419-020-2540-y
negative Regulation: SOX2 ---| chemosensitivity	32439916	10.1038/s41419-020-2540-y
negative Regulation: SOX2 ---| chemosensitivity	33953166	10.1038/s41419-021-03733-5
negative Regulation: SOX2 ---| chemosensitivity	22732500	10.4161/cc.21021
negative Regulation: SOX2 ---| chemosensitivity	26499463	10.1186/s12885-015-1826-4
negative Regulation: SOX2 ---| chemosensitivity	30272330	10.3892/or.2018.673510.3892
negative Regulation: SOX2 ---| chemosensitivity		
negative Regulation: MIR155HG ---| chemosensitivity	32529543	10.1007/s10571-020-00898-z
negative Regulation: EGR1 ---| chemosensitivity	23178451	10.1016/j.canlet.2012.11.00
negative Regulation: EGR1 ---| chemosensitivity	23478574	10.1016/j.biopha.2013.01.00
negative Regulation: EGR1 ---| chemosensitivity	23478574	10.1016/j.biopha.2013.01.00
negative Regulation: EGR1 ---| chemosensitivity	24704156	10.1016/j.canlet.2014.03.02
negative Regulation: EGR1 ---| chemosensitivity	26708617	10.1016/j.yexcr.2015.12.006
negative Regulation: EGR1 ---| chemosensitivity		
negative Regulation: IFI30 ---| chemosensitivity	34252711	10.1016/j.tranon.2021.10115
negative Regulation: IFI30 ---| chemosensitivity	34400904	10.7150/IJMS.62870
negative Regulation: RRM2 ---| chemosensitivity	29275122	10.1016/j.biomaterials.2017

negative Regulation: RRM2 ---| chemosensitivity	29275122	10.1016/j.biomaterials.2017
negative Regulation: RRM2 ---| chemosensitivity	33227712	10.1016/j.biopha.2020.11099
negative Regulation: RRM2 ---| chemosensitivity	15890238	10.1016/j.canlet.2004.10.01
negative Regulation: RRM2 ---| chemosensitivity	18414411	
negative Regulation: RRM2 ---| chemosensitivity	30898978	10.1042/BSR20182062
negative Regulation: RRM2 ---| chemosensitivity	30777929	10.1042/BSR20181374
negative Regulation: RRM2 ---| chemosensitivity	23335963	10.1371/journal.pone.005343
negative Regulation: RRM2 ---| chemosensitivity	23335963	10.1371/journal.pone.005343
negative Regulation: RRM2 ---| chemosensitivity		10.1016/j.gendis.2022.11.02
negative Regulation: GLI2 ---| chemosensitivity	27748762	10.1038/onc.2016.348
negative Regulation: CENPA ---| chemosensitivity	35013138	10.1038/s41420-021-00622-w1
negative Regulation: ST3GAL3 ---| chemosensitivity	27871859	10.1016/j.bbrc.2016.11.107
negative Regulation: ST3GAL3 ---| chemosensitivity	31702036	10.3892/mmr.2019.10712
negative Regulation: PGD ---| chemosensitivity	30841449	10.1016/j.biopha.2019.01.02
negative Regulation: PGD ---| chemosensitivity	30182212	10.1007/s12094-018-1937-x10
negative Regulation: HOXA7 ---| chemosensitivity	29455183	10.1515/hsz-2017-027410.151
negative Regulation: HOXA7 ---| chemosensitivity	29455183	10.1515/hsz-2017-027410.151
negative Regulation: TNC ---| chemosensitivity	33876384	10.1007/s12031-021-01832-8
negative Regulation: TNC ---| chemosensitivity	33876384	10.1007/s12031-021-01832-8
negative Regulation: TNC ---| chemosensitivity	33876384	10.1007/s12031-021-01832-8
negative Regulation: TNC ---| chemosensitivity	33876384	10.1007/s12031-021-01832-8
negative Regulation: HOXA13 ---| chemosensitivity	36514224	10.3724/abbs.2022182
negative Regulation: SP7 ---| chemosensitivity	29227978	10.1159/000485400
negative Regulation: SP7 ---| chemosensitivity	29227978	10.1159/000485400
negative Regulation: SP7 ---| chemosensitivity	29227978	10.1159/000485400
negative Regulation: SP7 ---| chemosensitivity	29227978	10.1159/000485400
negative Regulation: SP7 ---| chemosensitivity	29227978	10.1159/000485400
negative Regulation: SP7 ---| chemosensitivity	29227978	10.1159/000485400
negative Regulation: SP7 ---| chemosensitivity	29227978	10.1159/000485400
negative Regulation: SP7 ---| chemosensitivity	29227978	10.1159/000485400
negative Regulation: SP7 ---| chemosensitivity	29227978	10.1159/000485400
negative Regulation: SP7 ---| chemosensitivity	29227978	10.1159/000485400
negative Regulation: SP7 ---| chemosensitivity	29227978	10.1159/000485400
negative Regulation: SP7 ---| chemosensitivity	29227978	10.1159/000485400
negative Regulation: SP7 ---| chemosensitivity	29227978	10.1159/000485400
negative Regulation: SP7 ---| chemosensitivity	29227978	10.1159/000485400
negative Regulation: SP7 ---| chemosensitivity	32098271	10.3390/ijms21041491
negative Regulation: SP7 ---| chemosensitivity	29227978	10.1159/00048540010.1159/00
negative Regulation: SP7 ---| chemosensitivity	29227978	10.1159/00048540010.1159/00
negative Regulation: SP7 ---| chemosensitivity	29227978	10.1159/00048540010.1159/00
negative Regulation: PTTG1 ---| chemosensitivity	23523951	10.1016/j.cbi.2013.03.011
negative Regulation: RPS6KA3 ---| chemosensitivity	27082640	10.3892/ijo.2016.3481
negative Regulation: RPS6KA3 ---| chemosensitivity	27082640	10.3892/ijo.2016.3481
negative Regulation: RPS6KA3 ---| chemosensitivity	27082640	10.3892/ijo.2016.3481
negative Regulation: RPS6KA3 ---| chemosensitivity	27082640	10.3892/ijo.2016.3481
negative Regulation: RPS6KA3 ---| chemosensitivity	34373588	10.1038/s41374-021-00651-0
negative Regulation: RPS6KA3 ---| chemosensitivity	34373588	10.1038/s41374-021-00651-0
negative Regulation: STOML2 ---| chemosensitivity		
negative Regulation: STOML2 ---| chemosensitivity		
negative Regulation: CFTR ---| chemosensitivity	28592122	10.4149/neo_2017_508
negative Regulation: TYMS ---| chemosensitivity	27838412	10.1016/j.canlet.2016.11.00
negative Regulation: TYMS ---| chemosensitivity	29331423	10.1016/j.canlet.2018.01.02
negative Regulation: TYMS ---| chemosensitivity	17498667	10.1016/j.bcp.2007.04.007
negative Regulation: TYMS ---| chemosensitivity	19576666	10.1016/j.bjoms.2009.05.010

negative Regulation: TYMS ---| chemosensitivity	12084459	10.1016/S0925-4439(02)00080
negative Regulation: TYMS ---| chemosensitivity	12084459	10.1016/S0925-4439(02)00080
negative Regulation: TYMS ---| chemosensitivity	15737843	10.1016/j.jamcollsurg.2004.
negative Regulation: TYMS ---| chemosensitivity	18187583	
negative Regulation: TYMS ---| chemosensitivity	21380490	10.3892/or.2011.1206
negative Regulation: TYMS ---| chemosensitivity	21362378	10.3760/cma.j.issn.0366-699
negative Regulation: TYMS ---| chemosensitivity	21362378	10.3760/cma.j.issn.0366-699
negative Regulation: TYMS ---| chemosensitivity	25482885	10.3892/or.2014.3646
negative Regulation: TYMS ---| chemosensitivity	25482885	10.3892/or.2014.3646
negative Regulation: TYMS ---| chemosensitivity	27098147	10.3727/096504016X145627253
negative Regulation: TYMS ---| chemosensitivity	18187583	10.1124/mol.107.04238210.11
negative Regulation: TYMS ---| chemosensitivity	28521444	10.3892/ol.2017.589510.3892
negative Regulation: TYMS ---| chemosensitivity	35586209	10.1155/2022/3249766
negative Regulation: MIF ---| chemosensitivity	32893526	10.1631/jzus.B2000076
negative Regulation: MIF ---| chemosensitivity	25101674	10.1038/cddis.2014.300
negative Regulation: MIF ---| chemosensitivity	22430150	10.1038/cr.2012.4410.1038/c
negative Regulation: NUAK1 ---| chemosensitivity	28469776	
negative Regulation: NUAK1 ---| chemosensitivity	28469776	
negative Regulation: CDKL1 ---| chemosensitivity	30802495	10.1016/j.mcp.2019.02.00410
negative Regulation: HMGCR ---| chemosensitivity	17158228	10.1182/blood-2006-08-04444
negative Regulation: TRPM2 ---| chemosensitivity	30942446	10.3892/or.2019.7095
negative Regulation: TRPM2 ---| chemosensitivity	35089473	10.1007/s10495-022-01709-0
negative Regulation: OGT ---| chemosensitivity	30453909	10.1186/s12885-018-5033-y
negative Regulation: OGT ---| chemosensitivity	30453909	10.1186/s12885-018-5033-y
negative Regulation: PPP1R7 ---| chemosensitivity	27685628	10.1038/cddis.2016.289
negative Regulation: PPP1R7 ---| chemosensitivity	27685628	10.1038/cddis.2016.28910.10
negative Regulation: TRIM62 ---| chemosensitivity	35469851	10.1016/j.taap.2022.1160351
negative Regulation: ID4 ---| chemosensitivity	27537399	10.1097/CAD.000000000000041
negative Regulation: ID4 ---| chemosensitivity	27537399	10.1097/CAD.000000000000041
negative Regulation: ID4 ---| chemosensitivity	33993270	10.1093/carcin/bgab03710.10
negative Regulation: SSRP1 ---| chemosensitivity	27525970	10.1016/j.biopha.2016.08.02
negative Regulation: MIR10A ---| chemosensitivity	32786108	10.1002/jcb.2983310.1002/jc
negative Regulation: MIR10A ---| chemosensitivity	35863089	10.1016/j.bbrc.2022.07.017
negative Regulation: DPP9 ---| chemosensitivity	32734369	10.1245/s10434-020-08729-7
negative Regulation: DPP9 ---| chemosensitivity	32734369	10.1245/s10434-020-08729-7
negative Regulation: HSPA4L ---| chemosensitivity	31899217	10.1016/j.exphem.2019.12.00
negative Regulation: COIL ---| chemosensitivity	30065619	10.1186/s12935-018-0600-5
negative Regulation: COIL ---| chemosensitivity	35151311	10.1186/s12964-022-00820-8
negative Regulation: MEN1 ---| chemosensitivity	34711954	10.1038/s41388-021-02039-2
negative Regulation: JOSD1 ---| chemosensitivity	34261480	10.1186/s12935-021-02060-1
negative Regulation: JOSD1 ---| chemosensitivity	34261480	10.1186/s12935-021-02060-11
negative Regulation: EIF2S1 ---| chemosensitivity	29957018	10.1089/cbr.2018.2447
negative Regulation: SOX4 ---| chemosensitivity	28780934	10.1016/j.trecan.2017.06.00
negative Regulation: SOX4 ---| chemosensitivity	26555193	10.1186/s12885-015-1875-8
negative Regulation: PAK6 ---| chemosensitivity		10.1016/j.juro.2011.02.501
negative Regulation: PAK6 ---| chemosensitivity	28687498	10.1016/j.ebiom.2017.06.028
negative Regulation: PAK6 ---| chemosensitivity	28688823	10.1016/j.ebiom.2017.07.003
negative Regulation: PAK6 ---| chemosensitivity		10.1016/j.juro.2010.02.455
negative Regulation: PAK6 ---| chemosensitivity	26459798	10.3892/mmr.2015.4390
negative Regulation: PAK6 ---| chemosensitivity	19362342	10.1016/j.urology.2008.09.0
negative Regulation: PAK6 ---| chemosensitivity	19362342	10.1016/j.urology.2008.09.0
negative Regulation: PAK6 ---| chemosensitivity	35902562	10.1038/s41419-022-05118-8
negative Regulation: MIR3136 ---| chemosensitivity	30425571	10.2147/CMAR.S17272210.2147
negative Regulation: STAB1 ---| chemosensitivity	28631095	10.1007/s10637-017-0477-5

negative Regulation: KCNJ2 ---|	chemosensitivity	33892053	10.1016/j.bbcan.2021.188552
negative Regulation: WNT2B ---|	chemosensitivity	31985024	10.3892/ijmm.2020.4474
negative Regulation: MAPK14 ---|	chemosensitivity	28916425	10.1016/j.prp.2017.08.003
negative Regulation: MAPK14 ---|	chemosensitivity	23920402	10.1016/j.ejca.2013.07.015
negative Regulation: MAPK14 ---|	chemosensitivity	24333738	10.1016/j.canlet.2013.11.01
negative Regulation: MAPK14 ---|	chemosensitivity	19435873	10.1158/1535-7163.MCT-08-09
negative Regulation: MAPK14 ---|	chemosensitivity		
negative Regulation: ALOX12 ---|	chemosensitivity	31014671	10.1016/j.bbrc.2019.04.101
negative Regulation: ALOX12 ---|	chemosensitivity		10.1016/j.bbrc.2019.04.101
negative Regulation: YEATS4 ---|	chemosensitivity	30662802	
negative Regulation: PINK1 ---|	chemosensitivity	29022200	10.1245/s10434-017-6096-8
negative Regulation: CTSK ---|	chemosensitivity	31901727	10.1016/j.redox.2019.101422
negative Regulation: LINC00680 ---|	chemosensitivit	33499874	10.1186/s13046-021-01854-5
negative Regulation: LINC00680 ---|	chemosensitivit	33499874	10.1186/s13046-021-01854-5
negative Regulation: LINC00680 ---|	chemosensitivit	33499874	10.1186/s13046-021-01854-51
negative Regulation: WDR5 ---|	chemosensitivity	33257682	10.1038/s41419-020-03231-0
negative Regulation: WDR5 ---|	chemosensitivity	34154613	10.1186/s13046-021-01989-51
negative Regulation: WDR5 ---|	chemosensitivity	33754029	10.7150/thno.5581410.7150/t
negative Regulation: WDR5 ---|	chemosensitivity	33754029	10.7150/thno.5581410.7150/t
negative Regulation: WDR5 ---|	chemosensitivity	34154613	10.1186/s13046-021-01989-51
negative Regulation: WDR5 ---|	chemosensitivity	34154613	10.1186/s13046-021-01989-5
negative Regulation: WDR5 ---|	chemosensitivity	34154613	10.1186/s13046-021-01989-5
negative Regulation: WDR5 ---|	chemosensitivity	34830196	10.3390/ijms222212314
negative Regulation: AXL ---|	chemosensitivity	29496493	10.1016/j.lfs.2018.02.033
negative Regulation: AXL ---|	chemosensitivity	24184575	10.1016/j.brainres.2013.10.
negative Regulation: AXL ---|	chemosensitivity	27558819	10.1007/978-3-319-39406-0_5
negative Regulation: AXL ---|	chemosensitivity	27558819	10.1007/978-3-319-39406-0_5
negative Regulation: AXL ---|	chemosensitivity	27712586	10.3727/096504016X146487014
negative Regulation: AXL ---|	chemosensitivity	28468579	10.1177/1010428317699796
negative Regulation: AXL ---|	chemosensitivity	31243646	10.1007/s12272-019-01169-2
negative Regulation: AXL ---|	chemosensitivity	22890323	10.1038/onc.2012.355
negative Regulation: AXL ---|	chemosensitivity	22890323	10.1038/onc.2012.355
negative Regulation: AXL ---|	chemosensitivity	22890323	10.1038/onc.2012.355
negative Regulation: AXL ---|	chemosensitivity		
negative Regulation: AXL ---|	chemosensitivity	20423999	10.1158/1535-7163.MCT-09-07
negative Regulation: AXL ---|	chemosensitivity	22890323	10.1038/onc.2012.35510.1038
negative Regulation: AXL ---|	chemosensitivity	22890323	10.1038/onc.2012.35510.1038
negative Regulation: AXL ---|	chemosensitivity	24984960	10.1002/iub.128510.1002/iub
negative Regulation: AXL ---|	chemosensitivity	25596315	10.1016/j.bbrc.2015.01.0171
negative Regulation: AXL ---|	chemosensitivity	35708914	10.1172/JCI150517
negative Regulation: CCR2 ---|	chemosensitivity	28971904	10.3324/haematol.2016.15720
negative Regulation: APEX1 ---|	chemosensitivity	32641008	10.1186/s12885-020-07111-w
negative Regulation: APEX1 ---|	chemosensitivity	34006852	10.1038/s41419-021-03804-7
negative Regulation: APEX1 ---|	chemosensitivity	22788765	10.2174/092986712802002509
negative Regulation: APEX1 ---|	chemosensitivity	23418439	10.1371/journal.pone.005531
negative Regulation: APEX1 ---|	chemosensitivity		
negative Regulation: YWHAZ ---|	chemosensitivity	25770209	10.1074/jbc.M114.607580
negative Regulation: YWHAZ ---|	chemosensitivity	21334806	10.1016/j.canlet.2011.01.01
negative Regulation: YWHAZ ---|	chemosensitivity	24556826	10.1038/emm.2013.151
negative Regulation: YWHAZ ---|	chemosensitivity	24603438	10.1159/000357377
negative Regulation: YWHAZ ---|	chemosensitivity	24603438	10.1159/000357377
negative Regulation: YWHAZ ---|	chemosensitivity	24603438	10.1159/000357377
negative Regulation: YWHAZ ---|	chemosensitivity	25770209	10.1074/jbc.M114.607580
negative Regulation: YWHAZ ---|	chemosensitivity	21334806	10.1016/j.canlet.2011.01.01

negative Regulation: YWHAZ ---| chemosensitivity	29387222	10.3892/ol.2017.732610.3892
negative Regulation: YWHAZ ---| chemosensitivity		10.1074/jbc.M114.607580
negative Regulation: UBE2V1 ---| chemosensitivity	31827405	10.1186/s12935-019-1050-4
negative Regulation: UBE2V1 ---| chemosensitivity	31827405	10.1186/s12935-019-1050-410
negative Regulation: VTRNA2-1 ---| chemosensitivity	31966795	
negative Regulation: ATG16L1 ---| chemosensitivity	30320370	10.3892/ijo.2018.4593
negative Regulation: RAD18 ---| chemosensitivity	32356270	10.1245/s10434-020-08518-2
negative Regulation: RAD18 ---| chemosensitivity	32356270	10.1245/s10434-020-08518-2
negative Regulation: RAD18 ---| chemosensitivity	32356270	10.1245/s10434-020-08518-21
negative Regulation: RER1 ---| chemosensitivity	32380129	10.1016/j.canlet.2020.04.01
negative Regulation: MIR452 ---| chemosensitivity	33658394	10.18632/aging.202657
negative Regulation: MIR452 ---| chemosensitivity	33658394	10.18632/aging.202657
negative Regulation: COPS6 ---| chemosensitivity	36512632	10.1158/0008-5472.CAN-22-21
negative Regulation: LDHA ---| chemosensitivity	26454069	10.1016/j.semcancer.2015.10
negative Regulation: LDHA ---| chemosensitivity	34131808	10.1007/s00018-021-03866-y
negative Regulation: LDHA ---| chemosensitivity	24178759	10.1038/bjc.2013.681
negative Regulation: LDHA ---| chemosensitivity	35191522	10.3892/OR.2022.8288
negative Regulation: IFRD1 ---| chemosensitivity	33524870	10.1016/j.bbrc.2021.01.029
negative Regulation: IFRD1 ---| chemosensitivity	33524870	10.1016/j.bbrc.2021.01.029
negative Regulation: IFRD1 ---| chemosensitivity	33524870	10.1016/j.bbrc.2021.01.029
negative Regulation: IFRD1 ---| chemosensitivity	33524870	10.1016/j.bbrc.2021.01.029
negative Regulation: LOC112543491 ---| chemosensiti	27195675	10.1038/cddis.2016.124
negative Regulation: KCNQ1OT1 ---| chemosensitivity	31676070	10.1016/j.bbrc.2019.10.136
negative Regulation: KCNQ1OT1 ---| chemosensitivity	31706574	10.1016/j.bbrc.2019.10.180
negative Regulation: KCNQ1OT1 ---| chemosensitivity	31454677	10.1016/j.omtn.2019.06.010
negative Regulation: KCNQ1OT1 ---| chemosensitivity	31454677	10.1016/j.omtn.2019.06.010
negative Regulation: KCNQ1OT1 ---| chemosensitivity	29970910	10.1038/s41419-018-0793-5
negative Regulation: KCNQ1OT1 ---| chemosensitivity	33576460	10.3892/ijmm.2021.488710.38
negative Regulation: KCNQ1OT1 ---| chemosensitivity	33705625	10.4143/crt.2020.1208
negative Regulation: KCNQ1OT1 ---| chemosensitivity	35540579	10.1039/c9ra06378a10.1039/c
negative Regulation: MIR25 ---| chemosensitivity	29596304	10.3390/molecules23040787
negative Regulation: MIR25 ---| chemosensitivity	25945419	10.1080/15384047.2015.10409
negative Regulation: MIR25 ---| chemosensitivity		
negative Regulation: PATJ ---| chemosensitivity	33169673	10.1016/j.ygeno.2020.10.016
negative Regulation: COX5A ---| chemosensitivity	32758616	10.1016/j.canlet.2020.07.02
negative Regulation: CLEC3A ---| chemosensitivity	32319617	10.3892/mmr.2020.10986
negative Regulation: CLEC3A ---| chemosensitivity	32319617	10.3892/mmr.2020.10986
negative Regulation: CLEC3A ---| chemosensitivity	32319617	10.3892/mmr.2020.10986
negative Regulation: CLEC3A ---| chemosensitivity	32319617	10.3892/mmr.2020.10986
negative Regulation: CLEC3A ---| chemosensitivity	32319617	10.3892/mmr.2020.1098610.38
negative Regulation: ITGA6 ---| chemosensitivity	26742943	10.1016/j.jconrel.2015.12.0
negative Regulation: ARHGDIA ---| chemosensitivity	22668020	10.4149/neo_2012_07010.4149
negative Regulation: MIR134 ---| chemosensitivity	33892053	10.1016/j.bbcan.2021.188552
negative Regulation: MIR134 ---| chemosensitivity	27685628	10.1038/cddis.2016.289
negative Regulation: MIR134 ---| chemosensitivity	28325280	10.1016/j.omtn.2016.11.003
negative Regulation: MIR134 ---| chemosensitivity	31689617	10.1016/j.omtn.2019.09.025
negative Regulation: MIR134 ---| chemosensitivity	27685628	10.1038/cddis.2016.289
negative Regulation: MIR134 ---| chemosensitivity	27685628	10.1038/cddis.2016.289
negative Regulation: MIR134 ---| chemosensitivity	27685628	10.1038/cddis.2016.289
negative Regulation: MIR134 ---| chemosensitivity	27685628	10.1038/cddis.2016.289
negative Regulation: MIR134 ---| chemosensitivity	27685628	10.1038/cddis.2016.289
negative Regulation: MIR134 ---| chemosensitivity	33621196	10.18632/aging.202538
negative Regulation: MIR134 ---| chemosensitivity	33621196	10.18632/aging.202538
negative Regulation: MIR134 ---| chemosensitivity	27685628	10.1038/cddis.2016.28910.10

negative Regulation: DICER1 ---| chemosensitivity 30336979 10.1016/j.bbrc.2018.10.071
negative Regulation: DICER1 ---| chemosensitivity 25115815 10.1186/1476-4598-13-190
negative Regulation: DICER1 ---| chemosensitivity 25115815 10.1186/1476-4598-13-190
negative Regulation: DICER1 ---| chemosensitivity 25115815 10.1186/1476-4598-13-190
negative Regulation: DICER1 ---| chemosensitivity 25115815 10.1186/1476-4598-13-19010.
negative Regulation: DICER1 ---| chemosensitivity 28911000 10.1093/carcin/bgx05910.109
negative Regulation: DDX27 ---| chemosensitivity 36551703 10.3390/cancers14246218
negative Regulation: CHCHD6 ---| chemosensitivity 22228767 10.1074/jbc.M111.277103
negative Regulation: CHCHD6 ---| chemosensitivity 22228767 10.1074/jbc.M111.277103
negative Regulation: CHCHD6 ---| chemosensitivity 27479602 10.1371/journal.pone.016025
negative Regulation: CHCHD6 ---| chemosensitivity 22228767 10.1074/jbc.M111.277103
negative Regulation: CHCHD6 ---| chemosensitivity 22228767 10.1074/jbc.M111.277103
negative Regulation: CHCHD6 ---| chemosensitivity 22228767 10.1074/jbc.M111.277103
negative Regulation: CHCHD6 ---| chemosensitivity 22228767 10.1074/jbc.M111.277103
negative Regulation: CHCHD6 ---| chemosensitivity 22228767 10.1074/jbc.M111.27710310.1
negative Regulation: CREBBP ---| chemosensitivity 34149923 10.7150/jca.5613510.7150/jc
negative Regulation: CREBBP ---| chemosensitivity 34149923 10.7150/jca.5613510.7150/jc
negative Regulation: CREBBP ---| chemosensitivity 34812991 10.1007/s12032-021-01610-x
negative Regulation: ST8SIA4 ---| chemosensitivity 22579717 10.1016/j.biocel.2012.04.02
negative Regulation: MET ---| chemosensitivity 28939129 10.1016/j.jphs.2017.06.006
negative Regulation: MET ---| chemosensitivity 22820099 10.1016/j.abb.2012.07.003
negative Regulation: MET ---| chemosensitivity 22820099 10.1016/j.abb.2012.07.003
negative Regulation: MET ---| chemosensitivity 22820099 10.1016/j.abb.2012.07.003
negative Regulation: MET ---| chemosensitivity 24835851 10.1016/j.oraloncology.2014
negative Regulation: MET ---| chemosensitivity 33300049 10.3892/ijo.2020.5127
negative Regulation: MET ---| chemosensitivity 33213473 10.1186/s12967-020-02613-4
negative Regulation: MET ---| chemosensitivity 33526881 10.1038/s41388-020-01577-5
negative Regulation: MET ---| chemosensitivity 33846781 10.3892/IJO.2021.5208
negative Regulation: MET ---| chemosensitivity 21468575 10.3892/mmr.2011.426
negative Regulation: MET ---| chemosensitivity 23613505 10.1177/0300060513480920
negative Regulation: MET ---| chemosensitivity 25873032 10.1038/ncomms7776
negative Regulation: MET ---| chemosensitivity 21468575 10.3892/mmr.2011.42610.3892
negative Regulation: MET ---| chemosensitivity 21468575 10.3892/mmr.2011.42610.3892
negative Regulation: MET ---| chemosensitivity 21468575 10.3892/mmr.2011.42610.3892
negative Regulation: MET ---| chemosensitivity 22820099 10.1016/j.abb.2012.07.00310
negative Regulation: MET ---| chemosensitivity 24835851 10.1016/j.oraloncology.2014
negative Regulation: MET ---| chemosensitivity 27658187 10.1111/1440-1681.1267210.1
negative Regulation: MET ---| chemosensitivity 33441071 10.2174/1570159X19666210113
negative Regulation: MET ---| chemosensitivity 36283555 10.1016/j.ijbiomac.2022.10.
negative Regulation: SCD ---| chemosensitivity 22922095 10.1016/j.mce.2012.08.005
negative Regulation: PRKD2 ---| chemosensitivity 24665648
negative Regulation: PCID2 ---| chemosensitivity 34031538 10.1038/s41374-021-00613-6
negative Regulation: PCID2 ---| chemosensitivity 34031538 10.1038/s41374-021-00613-6
negative Regulation: PCID2 ---| chemosensitivity 34031538 10.1038/s41374-021-00613-6
negative Regulation: PCID2 ---| chemosensitivity 34031538 10.1038/s41374-021-00613-6
negative Regulation: STAT5B ---| chemosensitivity 25997700 10.3892/or.2015.3994
negative Regulation: STAT5B ---| chemosensitivity 27035235 10.3892/or.2016.4727
negative Regulation: STAT5B ---| chemosensitivity 25997700 10.3892/or.2015.3994
negative Regulation: STAT5B ---| chemosensitivity 25997700 10.3892/or.2015.3994
negative Regulation: STAT5B ---| chemosensitivity 25997700 10.3892/or.2015.3994
negative Regulation: STAT5B ---| chemosensitivity 25997700 10.3892/or.2015.3994
negative Regulation: STAT5B ---| chemosensitivity 25997700 10.3892/or.2015.399410.3892
negative Regulation: STAT5B ---| chemosensitivity 25997700 10.3892/or.2015.399410.3892
negative Regulation: STAT5B ---| chemosensitivity 25997700 10.3892/or.2015.399410.3892

negative Regulation:	STMN1 ---| chemosensitivity	18162179	10.1016/j.bbrc.2007.12.107
negative Regulation:	STMN1 ---| chemosensitivity	12480194	10.1016/S0169-5002(02)00171
negative Regulation:	STMN1 ---| chemosensitivity	26802649	10.3727/096504015X144525634
negative Regulation:	STMN1 ---| chemosensitivity	26802649	10.3727/096504015X144525634
negative Regulation:	STMN1 ---| chemosensitivity	29059154	10.1038/onc.2017.373
negative Regulation:	STMN1 ---| chemosensitivity	30169496	10.12659/MSM.910953
negative Regulation:	STMN1 ---| chemosensitivity	30616477	10.1177/0300060518819606
negative Regulation:	STMN1 ---| chemosensitivity	30422880	10.1097/CMR.000000000000055
negative Regulation:	STMN1 ---| chemosensitivity	32377743	10.3892/or.2020.7601
negative Regulation:	STMN1 ---| chemosensitivity	21546534	10.1167/iovs.10-6973
negative Regulation:	STMN1 ---| chemosensitivity	26782519	10.4238/2015.December.28.18
negative Regulation:	STMN1 ---| chemosensitivity	21546534	10.1167/iovs.10-697310.1167
negative Regulation:	STMN1 ---| chemosensitivity	26802649	10.3727/096504015X144525634
negative Regulation:	STMN1 ---| chemosensitivity	29021019	10.3727/096504015X144525634
negative Regulation:	STMN1 ---| chemosensitivity	35186166	10.1155/2022/3554100
negative Regulation:	HSF1 ---| chemosensitivity	35158144	10.1016/j.biopha.2022.11271
negative Regulation:	RABL6 ---| chemosensitivity	30197676	10.3892/ol.2018.920510.3892
negative Regulation:	HSPB1 ---| chemosensitivity	28012700	10.1016/j.tips.2016.11.009
negative Regulation:	HSPB1 ---| chemosensitivity	20833165	10.1016/j.yexcr.2010.08.014
negative Regulation:	HSPB1 ---| chemosensitivity		10.1016/j.ddstr.2008.02.007
negative Regulation:	HSPB1 ---| chemosensitivity	22498886	10.1016/j.jprot.2012.03.046
negative Regulation:	HSPB1 ---| chemosensitivity	26056726	10.1016/j.biomaterials.2015
negative Regulation:	HSPB1 ---| chemosensitivity	26877709	10.1186/s12935-016-0283-8
negative Regulation:	HSPB1 ---| chemosensitivity	31052354	10.3390/molecules24091700
negative Regulation:	HSPB1 ---| chemosensitivity		
negative Regulation:	HSPB1 ---| chemosensitivity	23404246	10.3892/or.2013.2274
negative Regulation:	HSPB1 ---| chemosensitivity	21472339	10.3892/mmr.2010.372
negative Regulation:	HSPB1 ---| chemosensitivity	22027750	10.1097/IGC.0b013e31822491d
negative Regulation:	HSPB1 ---| chemosensitivity	24902789	10.1038/aps.2014.22
negative Regulation:	HSPB1 ---| chemosensitivity	25502518	10.1371/journal.pone.011415
negative Regulation:	HSPB1 ---| chemosensitivity	26931434	10.3727/096504015X144969329
negative Regulation:	HSPB1 ---| chemosensitivity	26928556	10.1186/s13048-015-0210-y
negative Regulation:	HSPB1 ---| chemosensitivity	16525997	10.1002/pmic.200402031
negative Regulation:	HSPB1 ---| chemosensitivity	17524270	
negative Regulation:	HSPB1 ---| chemosensitivity	18758817	10.1007/s00432-008-0460-910
negative Regulation:	HSPB1 ---| chemosensitivity	19575256	10.1007/s00292-009-1147-y10
negative Regulation:	HSPB1 ---| chemosensitivity	19625496	10.1158/1535-7163.MCT-09-01
negative Regulation:	HSPB1 ---| chemosensitivity	26805817	10.3390/ijms1701013710.3390
negative Regulation:	HSPB1 ---| chemosensitivity	29487706	10.18632/oncotarget.2409110
negative Regulation:	HSPB1 ---| chemosensitivity	30471115	10.1002/jcp.2766610.1002/jc
negative Regulation:	HSPB1 ---| chemosensitivity	30651868	10.3892/etm.2018.704010.389
negative Regulation:	HSPB1 ---| chemosensitivity	36012138	10.3390/ijms23168875
negative Regulation:	TM4SF1 ---| chemosensitivity	31142317	10.1186/s12931-019-1071-5
negative Regulation:	TM4SF1 ---| chemosensitivity	31142317	10.1186/s12931-019-1071-5
negative Regulation:	TM4SF1 ---| chemosensitivity	31142317	10.1186/s12931-019-1071-5
negative Regulation:	TM4SF1 ---| chemosensitivity	31142317	10.1186/s12931-019-1071-5
negative Regulation:	TM4SF1 ---| chemosensitivity	31142317	10.1186/s12931-019-1071-5
negative Regulation:	TM4SF1 ---| chemosensitivity	31142317	10.1186/s12931-019-1071-510
negative Regulation:	FGFR4 ---| chemosensitivity	29763898	10.1159/000489759
negative Regulation:	FGFR4 ---| chemosensitivity	22508544	10.1530/ERC-12-0060
negative Regulation:	FGFR4 ---| chemosensitivity	29763898	10.1159/00048975910.1159/00
negative Regulation:	FGFR4 ---| chemosensitivity		
negative Regulation:	WTAP ---| chemosensitivity	30851419	10.1016/j.canlet.2019.02.04
negative Regulation:	WTAP ---| chemosensitivity	30851419	10.1016/j.canlet.2019.02.04

negative Regulation: WTAP ---| chemosensitivity 34081626 10.18632/aging.203062
negative Regulation: WTAP ---| chemosensitivity 30851419 10.1016/j.canlet.2019.02.04
negative Regulation: WTAP ---| chemosensitivity 35090469 10.1186/s12943-022-01508-w
negative Regulation: WTAP ---| chemosensitivity 36517820 10.1186/s12943-022-01680-z
negative Regulation: HIF1A ---| chemosensitivity 32278855 10.1016/j.ejphar.2020.17310
negative Regulation: HIF1A ---| chemosensitivity 27090014 10.1016/j.yexcr.2016.04.011
negative Regulation: HIF1A ---| chemosensitivity 29782974 10.1016/j.ijbiomac.2018.05.
negative Regulation: HIF1A ---| chemosensitivity 33321156 10.1016/j.jconrel.2020.12.0
negative Regulation: HIF1A ---| chemosensitivity 32137820 10.1093/annonc/mdy303.054
negative Regulation: HIF1A ---| chemosensitivity 17303624
negative Regulation: HIF1A ---| chemosensitivity 20706634
negative Regulation: HIF1A ---| chemosensitivity 28534514 10.1038/onc.2017.119
negative Regulation: HIF1A ---| chemosensitivity 29693166 10.3892/ijo.2018.4376
negative Regulation: HIF1A ---| chemosensitivity 30569180 10.3892/or.2018.6929
negative Regulation: HIF1A ---| chemosensitivity 30569180 10.3892/or.2018.6929
negative Regulation: HIF1A ---| chemosensitivity 30842147 10.21873/anticanres.13227
negative Regulation: HIF1A ---| chemosensitivity 30842147 10.21873/anticanres.13227
negative Regulation: HIF1A ---| chemosensitivity 30842147 10.21873/anticanres.13227
negative Regulation: HIF1A ---| chemosensitivity 30842147 10.21873/anticanres.13227
negative Regulation: HIF1A ---| chemosensitivity 30842147 10.21873/anticanres.13227
negative Regulation: HIF1A ---| chemosensitivity 30842147 10.21873/anticanres.13227
negative Regulation: HIF1A ---| chemosensitivity 31370155 10.3390/ijms20153736
negative Regulation: HIF1A ---| chemosensitivity 32432742 10.26355/eurrev_202005_2116
negative Regulation: HIF1A ---| chemosensitivity 32781585 10.3390/ijms21165631
negative Regulation: HIF1A ---| chemosensitivity 22785211 10.4161/cbt.20838
negative Regulation: HIF1A ---| chemosensitivity 23836645 10.1073/pnas.1300415110
negative Regulation: HIF1A ---| chemosensitivity 20706634 10.1371/journal.pone.001203
negative Regulation: HIF1A ---| chemosensitivity 20706634 10.1371/journal.pone.001203
negative Regulation: HIF1A ---| chemosensitivity 24795051
negative Regulation: HIF1A ---| chemosensitivity 24662981 10.1371/journal.pone.009282
negative Regulation: HIF1A ---| chemosensitivity 24737252 10.3892/or.2014.3140
negative Regulation: HIF1A ---| chemosensitivity 25920936 10.1080/15384047.2015.10409
negative Regulation: HIF1A ---| chemosensitivity 16628086 10.1097/00006676-200604000-
negative Regulation: HIF1A ---| chemosensitivity 22785211 10.4161/cbt.2083810.4161/cb
negative Regulation: HIF1A ---| chemosensitivity 27373041
negative Regulation: HIF1A ---| chemosensitivity 28656004 10.7150/ijbs.1896910.7150/i
negative Regulation: HIF1A ---| chemosensitivity 29275599 10.3760/cma.j.issn.0376-249
negative Regulation: HIF1A ---| chemosensitivity 30800222 10.18632/oncotarget.2663710
negative Regulation: HIF1A ---| chemosensitivity 30842147 10.21873/anticanres.1322710
negative Regulation: HIF1A ---| chemosensitivity 32432742 10.26355/eurrev_202005_2116
negative Regulation: HIF1A ---| chemosensitivity 33496749 10.1182/bloodadvances.20200
negative Regulation: HIF1A ---| chemosensitivity 35985176 10.1016/j.neo.2022.100821
negative Regulation: HIF1A ---| chemosensitivity 34383201 10.1007/s10620-021-07202-z
negative Regulation: ADORA1 ---| chemosensitivity 34115290 10.1007/s12017-021-08672-0
negative Regulation: ITGB4 ---| chemosensitivity 26742943 10.1016/j.jconrel.2015.12.0
negative Regulation: ITGB4 ---| chemosensitivity 30106452 10.3892/or.2018.6628
negative Regulation: ITGB4 ---| chemosensitivity 30106452 10.3892/or.2018.6628
negative Regulation: ITGB4 ---| chemosensitivity 30106452 10.3892/or.2018.6628
negative Regulation: ITGB4 ---| chemosensitivity 30106452 10.3892/or.2018.6628
negative Regulation: ITGB4 ---| chemosensitivity 22295105 10.1371/journal.pone.003070
negative Regulation: ITGB4 ---| chemosensitivity 30106452 10.3892/or.2018.662810.3892
negative Regulation: ALK ---| chemosensitivity 31832711 10.1007/s00253-019-10257-8
negative Regulation: ALK ---| chemosensitivity 32724469 10.7150/thno.4580310.7150/t
negative Regulation: MIR3617 ---| chemosensitivity 33545377 10.1016/j.etap.2021.103607

negative Regulation: MIR3617 ---| chemosensitivity 30425571 10.2147/CMAR.S17272210.2147
negative Regulation: PTPRZ1 ---| chemosensitivity 30497491 10.1186/s12964-018-0304-4
negative Regulation: VASH2 ---| chemosensitivity 31233186 10.3892/or.2019.7194
negative Regulation: VASH2 ---| chemosensitivity 24595063 10.1371/journal.pone.009035
negative Regulation: VASH2 ---| chemosensitivity 33710778 10.1002/cam4.384110.1002/ca
negative Regulation: SRPX2 ---| chemosensitivity 30551519 10.1016/j.biopha.2018.10.04
negative Regulation: SRPX2 ---| chemosensitivity 32455867 10.3390/ijms21103655
negative Regulation: SRPX2 ---| chemosensitivity 30551519 10.1016/j.biopha.2018.10.04
negative Regulation: SRPX2 ---| chemosensitivity 35935582 10.1155/2022/2931214
negative Regulation: GFI1 ---| chemosensitivity 29494986 10.1016/j.biopha.2018.02.03
negative Regulation: THOC1 ---| chemosensitivity 32669125 10.1186/s13046-020-01634-71
negative Regulation: SRC ---| chemosensitivity 29079189 10.1016/j.bbrc.2017.10.118
negative Regulation: SRC ---| chemosensitivity 32446364 10.1016/j.bbrc.2020.04.107
negative Regulation: SRC ---| chemosensitivity 26690339 10.1016/j.drup.2015.10.003
negative Regulation: SRC ---| chemosensitivity 18078709 10.1016/j.canlet.2007.10.03
negative Regulation: SRC ---| chemosensitivity 15194078 10.1016/j.jamcollsurg.2004.
negative Regulation: SRC ---| chemosensitivity 15194078 10.1016/j.jamcollsurg.2004.
negative Regulation: SRC ---| chemosensitivity 16621676 10.1016/j.drup.2006.02.002
negative Regulation: SRC ---| chemosensitivity 24388104 10.1016/j.critrevonc.2013.1
negative Regulation: SRC ---| chemosensitivity 19158362
negative Regulation: SRC ---| chemosensitivity 21152443
negative Regulation: SRC ---| chemosensitivity 29749405 10.1038/s41419-018-0574-1
negative Regulation: SRC ---| chemosensitivity 29749405 10.1038/s41419-018-0574-1
negative Regulation: SRC ---| chemosensitivity 29978609 10.3349/ymj.2018.59.6.727
negative Regulation: SRC ---| chemosensitivity 31113938 10.1038/s41419-019-1633-y
negative Regulation: SRC ---| chemosensitivity 31113938 10.1038/s41419-019-1633-y
negative Regulation: SRC ---| chemosensitivity 21720550 10.1371/journal.pone.002149
negative Regulation: SRC ---| chemosensitivity 21720550 10.1371/journal.pone.002149
negative Regulation: SRC ---| chemosensitivity 18794807 10.1038/onc.2008.32610.1038
negative Regulation: SRC ---| chemosensitivity 27825112 10.18632/oncotarget.1306710
negative Regulation: SRCIN1 ---| chemosensitivity 28393242 10.3892/ijo.2017.3952
negative Regulation: CXCL8 ---| chemosensitivity 32898567 10.1016/j.abb.2020.108571
negative Regulation: CXCL8 ---| chemosensitivity 33539818 10.1016/j.ejphar.2021.17392
negative Regulation: CXCL8 ---| chemosensitivity 25687885 10.1016/j.canlet.2015.02.02
negative Regulation: CXCL8 ---| chemosensitivity 19646263
negative Regulation: CXCL8 ---| chemosensitivity 29693201 10.1007/s00280-018-3584-x
negative Regulation: CXCL8 ---| chemosensitivity 22617157 10.1038/bjc.2012.177
negative Regulation: CXCL8 ---| chemosensitivity 25972907 10.1155/2015/917345
negative Regulation: CXCL8 ---| chemosensitivity 23321310 10.1016/j.bbrc.2013.01.0221
negative Regulation: CXCL8 ---| chemosensitivity 34847866 10.1186/s12885-021-09025-71
negative Regulation: CXCL8 ---| chemosensitivity 34847866 10.1186/s12885-021-09025-7
negative Regulation: CXCL8 ---| chemosensitivity 34847866 10.1186/s12885-021-09025-7
negative Regulation: CXCL8 ---| chemosensitivity 34847866 10.1186/s12885-021-09025-7
negative Regulation: MYO1B ---| chemosensitivity 35861939 10.1002/jcp.30831
negative Regulation: POSTN ---| chemosensitivity 30978348 10.1016/j.lfs.2019.04.021
negative Regulation: POSTN ---| chemosensitivity 29404445 10.1002/hep4.111410.1002/he
negative Regulation: MIR520G ---| chemosensitivity 25616665 10.1074/jbc.M114.620252
negative Regulation: MIR520G ---| chemosensitivity 25616665 10.1074/jbc.M114.620252
negative Regulation: KIF18B ---| chemosensitivity 34058791 10.1002/biof.175710.1002/bi
negative Regulation: LAMTOR5 ---| chemosensitivity 29309885 10.1016/j.gene.2018.01.019
negative Regulation: LAMTOR5 ---| chemosensitivity 34606782 10.1016/j.pharmthera.2021.1
negative Regulation: NDRG1 ---| chemosensitivity 30443171 10.7150/ijms.28055
negative Regulation: JAK2 ---| chemosensitivity 32160976 10.1016/j.canlet.2020.03.00
negative Regulation: JAK2 ---| chemosensitivity 24220695 10.1038/bjc.2013.673

negative Regulation: MERTK ---	chemosensitivity	32417270	10.1016/j.pharmthera.2020.1
negative Regulation: MERTK ---	chemosensitivity	24184575	10.1016/j.brainres.2013.10.
negative Regulation: MERTK ---	chemosensitivity	27558819	10.1007/978-3-319-39406-0_5
negative Regulation: MERTK ---	chemosensitivity	27558819	10.1007/978-3-319-39406-0_5
negative Regulation: MERTK ---	chemosensitivity	27558819	10.1007/978-3-319-39406-0_5
negative Regulation: MERTK ---	chemosensitivity	27558819	10.1007/978-3-319-39406-0_5
negative Regulation: MERTK ---	chemosensitivity	27712586	10.3727/096504016X146487014
negative Regulation: MERTK ---	chemosensitivity	28468579	10.1177/1010428317699796
negative Regulation: MERTK ---	chemosensitivity	31636733	10.1155/2019/2387614
negative Regulation: MERTK ---	chemosensitivity	22005534	10.1097/01.JTO.0000407562.0
negative Regulation: MERTK ---	chemosensitivity	33509214	10.1186/s12964-020-00694-8
negative Regulation: MERTK ---	chemosensitivity	22179835	10.1038/onc.2011.588
negative Regulation: MERTK ---	chemosensitivity	22179835	10.1038/onc.2011.588
negative Regulation: MERTK ---	chemosensitivity	22179835	10.1038/onc.2011.588
negative Regulation: MERTK ---	chemosensitivity	22890323	10.1038/onc.2012.355
negative Regulation: MERTK ---	chemosensitivity	22890323	10.1038/onc.2012.355
negative Regulation: MERTK ---	chemosensitivity	22890323	10.1038/onc.2012.355
negative Regulation: MERTK ---	chemosensitivity		
negative Regulation: MERTK ---	chemosensitivity	20423999	10.1158/1535-7163.MCT-09-07
negative Regulation: MERTK ---	chemosensitivity	22179835	10.1038/onc.2011.58810.1038
negative Regulation: MERTK ---	chemosensitivity	22890323	10.1038/onc.2012.35510.1038
negative Regulation: MERTK ---	chemosensitivity	23353780	10.1038/bcj.2012.4610.1038/
negative Regulation: MERTK ---	chemosensitivity	23997116	10.1158/1535-7163.MCT-13-00
negative Regulation: MERTK ---	chemosensitivity	25596315	10.1016/j.bbrc.2015.01.0171
negative Regulation: MERTK ---	chemosensitivity	27783662	10.1371/journal.pone.016510
negative Regulation: MERTK ---	chemosensitivity		10.1155/2019/2387614
negative Regulation: MERTK ---	chemosensitivity		10.1007/s12272-019-01169-2
negative Regulation: MERTK ---	chemosensitivity	34779503	10.3892/mmr.2021.12529
negative Regulation: MERTK ---	chemosensitivity	35708914	10.1172/JCI150517
negative Regulation: ROR2 ---	chemosensitivity	24158497	10.1007/s00018-013-1485-z10
negative Regulation: HOTAIR ---	chemosensitivity	29224950	10.1016/j.cca.2017.12.009
negative Regulation: HOTAIR ---	chemosensitivity	31901481	10.1016/j.cca.2019.12.028
negative Regulation: HOTAIR ---	chemosensitivity	33785336	10.1016/j.lfs.2021.119419
negative Regulation: HOTAIR ---	chemosensitivity	27717733	10.1016/j.bbcan.2016.10.001
negative Regulation: HOTAIR ---	chemosensitivity	32505000	10.1016/j.omtn.2020.05.011
negative Regulation: HOTAIR ---	chemosensitivity	27875938	10.1080/10245332.2016.12581
negative Regulation: HOTAIR ---	chemosensitivity	29168767	10.3390/ijms18122505
negative Regulation: HOTAIR ---	chemosensitivity	29455183	10.1515/hsz-2017-0274
negative Regulation: HOTAIR ---	chemosensitivity	30113217	10.1089/dna.2018.4312
negative Regulation: HOTAIR ---	chemosensitivity		10.1007/s12038-018-9820-z
negative Regulation: HOTAIR ---	chemosensitivity	31746420	10.3892/ijo.2019.4909
negative Regulation: HOTAIR ---	chemosensitivity	32349783	10.1186/s40659-020-00286-3
negative Regulation: HOTAIR ---	chemosensitivity	32349783	10.1186/s40659-020-00286-3
negative Regulation: HOTAIR ---	chemosensitivity	32705261	10.3892/or.2020.7685
negative Regulation: HOTAIR ---	chemosensitivity	33238475	10.3390/ijms21228855
negative Regulation: HOTAIR ---	chemosensitivity	25422887	10.1371/journal.pone.011067
negative Regulation: HOTAIR ---	chemosensitivity	26800519	10.1371/journal.pone.014723
negative Regulation: HOTAIR ---	chemosensitivity	26341496	10.1007/s13277-015-3998-6
negative Regulation: HOTAIR ---	chemosensitivity	26341496	10.1007/s13277-015-3998-6
negative Regulation: HOTAIR ---	chemosensitivity	26781446	10.1007/s00280-016-2964-310
negative Regulation: HOTAIR ---	chemosensitivity	26781446	10.1007/s00280-016-2964-310
negative Regulation: HOTAIR ---	chemosensitivity	26781446	10.1007/s00280-016-2964-310
negative Regulation: HOTAIR ---	chemosensitivity	29236333	10.1002/jcb.2659410.1002/jc
negative Regulation: HOTAIR ---	chemosensitivity	29250186	10.3892/ol.2017.723710.3892

negative Regulation: HOTAIR ---| chemosensitivity	29250186	10.3892/ol.2017.723710.3892
negative Regulation: HOTAIR ---| chemosensitivity	29455183	10.1515/hsz-2017-027410.151
negative Regulation: HOTAIR ---| chemosensitivity	29455183	10.1515/hsz-2017-027410.151
negative Regulation: HOTAIR ---| chemosensitivity	32349783	10.1186/s40659-020-00286-31
negative Regulation: HOTAIR ---| chemosensitivity	34790046	10.7150/IJMS.64253
negative Regulation: HOTAIR ---| chemosensitivity	33502891	10.4149/neo_2021_201112N121
negative Regulation: PTRF ---| chemosensitivity	24747515	10.1371/journal.pone.009343
negative Regulation: PTRF ---| chemosensitivity	24747515	10.1371/journal.pone.009343
negative Regulation: PTRF ---| chemosensitivity	24747515	10.1371/journal.pone.009343
negative Regulation: PROM1 ---| chemosensitivity	23268401	
negative Regulation: PROM1 ---| chemosensitivity	32246247	10.1007/s11033-020-05411-91
negative Regulation: ATG4A ---| chemosensitivity	31291988	10.1186/s13046-019-1287-8
negative Regulation: ATG4A ---| chemosensitivity	35088889	10.3892/mmr.2022.1261710.38
negative Regulation: TIMP1 ---| chemosensitivity	32522594	10.1016/j.bcp.2020.114085
negative Regulation: TIMP1 ---| chemosensitivity	21684102	10.1016/j.biopha.2011.02.00
negative Regulation: TIMP1 ---| chemosensitivity	21684102	10.1016/j.biopha.2011.02.00
negative Regulation: ZEB1 ---| chemosensitivity	30107990	10.1016/j.prp.2018.07.036
negative Regulation: ZEB1 ---| chemosensitivity	29352223	10.1038/s41419-017-0087-3
negative Regulation: ZEB1 ---| chemosensitivity	31323761	10.3390/ijms20143518
negative Regulation: ZEB1 ---| chemosensitivity	22116519	10.1159/000334449
negative Regulation: ZEB1 ---| chemosensitivity	25890268	10.1186/s12943-015-0357-6
negative Regulation: ZEB1 ---| chemosensitivity	23255418	10.1002/jcb.2448110.1002/jc
negative Regulation: ZEB1 ---| chemosensitivity	25890268	10.1186/s12943-015-0357-610
negative Regulation: ZEB1 ---| chemosensitivity	26036631	10.18632/oncotarget.3896
negative Regulation: PRKCQ ---| chemosensitivity	32600444	10.1186/s13058-020-01302-w1
negative Regulation: MIR499A ---| chemosensitivity	34453645	10.1007/s11010-021-04249-4
negative Regulation: UBD ---| chemosensitivity	23812429	10.1038/onc.2013.236
negative Regulation: MIR642A ---| chemosensitivity	33152911	10.1016/j.biopha.2020.11072
negative Regulation: GFER ---| chemosensitivity	29048676	10.3892/or.2017.5984
negative Regulation: GFER ---| chemosensitivity	29048676	10.3892/or.2017.598410.3892
negative Regulation: HHLA2 ---| chemosensitivity	36535461	10.1016/j.gene.2022.147086
negative Regulation: HHLA2 ---| chemosensitivity	36535461	10.1016/j.gene.2022.147086
negative Regulation: GLS ---| chemosensitivity	35294039	10.3892/ijo.2022.5337
negative Regulation: TUG1 ---| chemosensitivity	29543785	10.12659/MSM.906616
negative Regulation: TUG1 ---| chemosensitivity	29543785	10.12659/MSM.906616
negative Regulation: TUG1 ---| chemosensitivity	31141943	10.3390/ijms20112615
negative Regulation: TUG1 ---| chemosensitivity	32705269	10.3892/ijo.2020.5100
negative Regulation: TUG1 ---| chemosensitivity	32804119	10.3233/CH-200906
negative Regulation: TUG1 ---| chemosensitivity	35184425	10.3349/ymj.2022.63.3.229
negative Regulation: TUG1 ---| chemosensitivity	35643221	10.1016/j.semcancer.2022.05
negative Regulation: TUG1 ---| chemosensitivity	36539367	10.1097/CAD.000000000000139
negative Regulation: TUG1 ---| chemosensitivity	36539367	10.1097/CAD.000000000000139
negative Regulation: TUG1 ---| chemosensitivity	36539367	10.1097/CAD.000000000000139
negative Regulation: TUG1 ---| chemosensitivity	36539367	10.1097/CAD.000000000000139
negative Regulation: TUG1 ---| chemosensitivity	36539367	10.1097/CAD.000000000000139
negative Regulation: TUG1 ---| chemosensitivity	36539367	10.1097/CAD.000000000000139
negative Regulation: TUG1 ---| chemosensitivity	36539367	10.1097/CAD.000000000000139
negative Regulation: TUG1 ---| chemosensitivity	36539367	10.1097/CAD.000000000000139
negative Regulation: TUG1 ---| chemosensitivity	36539367	10.1097/CAD.000000000000139
negative Regulation: TUG1 ---| chemosensitivity	36539367	10.1097/CAD.000000000000139
negative Regulation: TUG1 ---| chemosensitivity	36539367	10.1097/CAD.000000000000139
negative Regulation: ICMT ---| chemosensitivity	29746868	10.1016/j.bbrc.2018.05.038
negative Regulation: ICMT ---| chemosensitivity	29331763	10.1016/j.biopha.2018.01.04
negative Regulation: ICMT ---| chemosensitivity	30882399	10.1097/CAD.000000000000077

negative Regulation: BANCR ---|	chemosensitivity	33816780	10.1016/j.omto.2021.01.003
negative Regulation: TUBB3 ---|	chemosensitivity	33429249	10.1016/j.drup.2020.100742
negative Regulation: TUBB3 ---|	chemosensitivity	19122647	
negative Regulation: TUBB3 ---|	chemosensitivity	31098996	10.1007/978-1-4939-9220-1_6
negative Regulation: TUBB3 ---|	chemosensitivity	17285590	10.1002/ijc.22557
negative Regulation: TUBB3 ---|	chemosensitivity	31099013	10.1007/978-1-4939-9220-1_2
negative Regulation: RNF4 ---|	chemosensitivity	35236966	10.1038/s41388-022-02247-4
negative Regulation: FZD7 ---|	chemosensitivity	28669726	10.1016/j.bbrc.2017.06.185
negative Regulation: FZD7 ---|	chemosensitivity	28669726	10.1016/j.bbrc.2017.06.185
negative Regulation: FZD7 ---|	chemosensitivity	28669726	10.1016/j.bbrc.2017.06.185
negative Regulation: FZD7 ---|	chemosensitivity	28669726	10.1016/j.bbrc.2017.06.185
negative Regulation: FZD7 ---|	chemosensitivity	28669726	10.1016/j.bbrc.2017.06.185
negative Regulation: FZD7 ---|	chemosensitivity	30142536	10.1016/j.biopha.2018.07.15
negative Regulation: FZD7 ---|	chemosensitivity	28669726	10.1016/j.bbrc.2017.06.1851
negative Regulation: FZD7 ---|	chemosensitivity	28669726	10.1016/j.bbrc.2017.06.1851
negative Regulation: TBXT ---|	chemosensitivity	24504414	10.3892/ijo.2014.2292
negative Regulation: TBXT ---|	chemosensitivity		
negative Regulation: AREG ---|	chemosensitivity	31370819	10.1186/s12885-019-5843-6
negative Regulation: ERP29 ---|	chemosensitivity	30569094	10.3892/or.2018.6943
negative Regulation: ERP29 ---|	chemosensitivity	25734833	10.1097/CAD.000000000000022
negative Regulation: ERP29 ---|	chemosensitivity	25734833	10.1097/CAD.000000000000022
negative Regulation: ERP29 ---|	chemosensitivity	25734833	10.1097/CAD.000000000000022
negative Regulation: ERP29 ---|	chemosensitivity	25734833	10.1097/CAD.000000000000022
negative Regulation: ERP29 ---|	chemosensitivity	25734833	10.1097/CAD.000000000000022
negative Regulation: ERP29 ---|	chemosensitivity	25734833	10.1097/CAD.000000000000022
negative Regulation: ERP29 ---|	chemosensitivity	30651868	10.3892/etm.2018.704010.389
negative Regulation: ERP29 ---|	chemosensitivity	30651868	10.3892/etm.2018.704010.389
negative Regulation: MAP4K4 ---|	chemosensitivity	24013097	10.1158/1535-7163.MCT-13-02
negative Regulation: MAP4K4 ---|	chemosensitivity	34930918	10.1038/s41419-021-04474-11
negative Regulation: SIRT1 ---|	chemosensitivity	33971612	10.1016/j.colsurfb.2021.111
negative Regulation: SIRT1 ---|	chemosensitivity	28288414	10.1016/j.redox.2017.03.006
negative Regulation: SIRT1 ---|	chemosensitivity	18573234	10.1016/j.bbrc.2008.06.045
negative Regulation: SIRT1 ---|	chemosensitivity	18573234	10.1016/j.bbrc.2008.06.045
negative Regulation: SIRT1 ---|	chemosensitivity	23104101	10.1016/j.freeradbiomed.201
negative Regulation: SIRT1 ---|	chemosensitivity	25445714	10.1016/j.bbagen.2014.11.00
negative Regulation: SIRT1 ---|	chemosensitivity	25843411	10.1016/j.ejphar.2015.03.06
negative Regulation: SIRT1 ---|	chemosensitivity	29584552	10.1089/omi.2018.0006
negative Regulation: SIRT1 ---|	chemosensitivity	30864709	10.3892/mmr.2019.10002
negative Regulation: SIRT1 ---|	chemosensitivity	31043584	10.1038/s41419-019-1592-3
negative Regulation: SIRT1 ---|	chemosensitivity	31043584	10.1038/s41419-019-1592-3
negative Regulation: SIRT1 ---|	chemosensitivity	31043584	10.1038/s41419-019-1592-3
negative Regulation: SIRT1 ---|	chemosensitivity	31101119	10.1186/s13058-019-1150-z
negative Regulation: SIRT1 ---|	chemosensitivity	32051395	10.1038/s41419-020-2308-4
negative Regulation: SIRT1 ---|	chemosensitivity	31746308	10.2174/1871520619666191028
negative Regulation: SIRT1 ---|	chemosensitivity	31746308	10.2174/1871520619666191028
negative Regulation: SIRT1 ---|	chemosensitivity	21947960	10.1007/s10930-011-9354-9
negative Regulation: SIRT1 ---|	chemosensitivity	21947960	10.1007/s10930-011-9354-9
negative Regulation: SIRT1 ---|	chemosensitivity	21677689	10.1038/gt.2011.81
negative Regulation: SIRT1 ---|	chemosensitivity	21677689	10.1038/gt.2011.81
negative Regulation: SIRT1 ---|	chemosensitivity	24223900	10.1371/journal.pone.007916
negative Regulation: SIRT1 ---|	chemosensitivity	24223900	10.1371/journal.pone.007916
negative Regulation: SIRT1 ---|	chemosensitivity	24223900	10.1371/journal.pone.007916
negative Regulation: SIRT1 ---|	chemosensitivity	24223900	10.1371/journal.pone.007916
negative Regulation: SIRT1 ---|	chemosensitivity	24223900	10.1371/journal.pone.007916

negative Regulation: SIRT1 ---| chemosensitivity	24223900	10.1371/journal.pone.007916
negative Regulation: SIRT1 ---| chemosensitivity	23542177	10.1038/onc.2013.88
negative Regulation: SIRT1 ---| chemosensitivity	23542177	10.1038/onc.2013.88
negative Regulation: SIRT1 ---| chemosensitivity	25032850	10.1038/cddis.2014.270
negative Regulation: SIRT1 ---| chemosensitivity	26662958	10.1007/s13277-015-4459-y
negative Regulation: SIRT1 ---| chemosensitivity	21947960	10.1007/s10930-011-9354-910
negative Regulation: SIRT1 ---| chemosensitivity	23542177	10.1038/onc.2013.8810.1038/
negative Regulation: SIRT1 ---| chemosensitivity	24223900	10.1371/journal.pone.007916
negative Regulation: SIRT1 ---| chemosensitivity	26398583	10.3892/or.2015.428710.3892
negative Regulation: SIRT1 ---| chemosensitivity	32831651	10.1186/s12935-020-01489-01
negative Regulation: SIRT1 ---| chemosensitivity	34590923	10.1177/03000605211040762
negative Regulation: LIF ---| chemosensitivity	34265288	10.1016/j.yexcr.2021.112734
negative Regulation: FN1 ---| chemosensitivity	29512696	10.3892/mmr.2018.8656
negative Regulation: SUB1 ---| chemosensitivity	33524870	10.1016/j.bbrc.2021.01.0291
negative Regulation: CYP1B1 ---| chemosensitivity	25860934	10.18632/oncotarget.3484
negative Regulation: CYP1B1 ---| chemosensitivity	33469378	10.2147/CMAR.S27739910.2147
negative Regulation: CYP1B1 ---| chemosensitivity	33469378	10.2147/CMAR.S27739910.2147
negative Regulation: CYP1B1 ---| chemosensitivity	36077068	10.3390/ijms23179670
negative Regulation: TWIST1 ---| chemosensitivity	32853985	10.1016/j.ebiom.2020.102955
negative Regulation: TWIST1 ---| chemosensitivity	18331824	10.1016/j.bbrc.2008.02.143
negative Regulation: TWIST1 ---| chemosensitivity	18331824	10.1016/j.bbrc.2008.02.143
negative Regulation: TWIST1 ---| chemosensitivity	18331824	10.1016/j.bbrc.2008.02.143
negative Regulation: TWIST1 ---| chemosensitivity	18331824	10.1016/j.bbrc.2008.02.143
negative Regulation: TWIST1 ---| chemosensitivity	28214899	10.1159/000447238
negative Regulation: TWIST1 ---| chemosensitivity	22673193	10.1038/cddis.2012.63
negative Regulation: TWIST1 ---| chemosensitivity	23741524	10.1371/journal.pone.006607
negative Regulation: TWIST1 ---| chemosensitivity	24805866	10.3892/mmr.2014.2212
negative Regulation: TWIST1 ---| chemosensitivity	24805866	10.3892/mmr.2014.2212
negative Regulation: TWIST1 ---| chemosensitivity	24805866	10.3892/mmr.2014.2212
negative Regulation: TWIST1 ---| chemosensitivity	24805866	10.3892/mmr.2014.2212
negative Regulation: TWIST1 ---| chemosensitivity	18331824	10.1016/j.bbrc.2008.02.143
negative Regulation: TWIST1 ---| chemosensitivity	24805866	10.3892/mmr.2014.2212
negative Regulation: TWIST1 ---| chemosensitivity	19272800	10.1016/j.urolonc.2008.12.0

.5
)15664
)15664
)15664
)15664
)15664
.0. 1016/j. omto. 2021. 01. 008

;. 012
)13

.3

.186/1746-1596-9-143
410. 1016/j. canlet. 2019. 10. 044

ıs. 14921

li. 13089
!/ol. 2017. 7059

;7

)6
;6
'7
;8

)2
)2
)9
)5
)6

;7

;8

3/toxsci/kfq028
/onc.2011.222
074/jbc.M111.295964
238/2015.March.30.3
4610.1158/0008-5472.CAN-14-2946

1710.1158/1078-0432.CCR-16-1317
510.1016/j.canlet.2021.09.035
8

141436

/or.2016.4585
/or.2017.5519

-9
-X

7
151120
183353
183353
183353

0

78
.1007/s00280-011-1682-0
2/mmr.2012.1017
2/mmr.2012.1017
186/1756-9966-31-73
1186/1477-7819-12-255

).1186/s12957-015-0501-1
18632/oncotarget.8105
392/mmr.2021.12267
)7

.151120

.6

3310.26355/eurrev_202009_22833

:3
35
.9.10.415
.10.1016/j.ccell.2018.05.001

548

,
.1

'8

50

18632/oncotarget.4581
).1186/s12935-015-0250-9

)14.2016

154711
)

580
375
3/bjc.2014.386
3/bjc.2014.386

52

02.013
.4

38

39
21

.510.1016/j.canlet.2016.08.015
./ph.2016.6625
./ph.2016.6625
./ph.2016.6625
/OTT.S158655
)06
.0.1038/s41419-020-02855-6
)06
)06
)06
)06
)06
)06
)06

)47

329999
)TT.S97399

)TT.S97399
)io.015552
.0.1038/s41419-020-02855-6

)cpt.13286

.100633

'2015/989070
.074/jbc.M115.700021

)2/ijo.2016.3671
)10.4149/neo_2020_200614N629
)
)
)
)

.0.1007/s00210-019-01772-6
.0.1007/s00210-019-01772-6

:p.29040

30
.2

3010.1016/j.canlet.2018.04.030

)TT.S64339
14.588
14.588

.110.1016/j.clinre.2017.01.011
39

)52

)6
:2

). 1186/s12935-018-0581-4

)110. 1016/j. phymed. 2021. 153601

)2/mmr. 2015. 4331
)2/mmr. 2015. 4331

)0485400
)0485400
)0485400

043
043
;
'145236

.4

i5
i5

)1

)38/cddis. 2017. 248
)1

). 1016/j. prp. 2019. 01. 042

icmm. 15291

)8

.0.1016/j.omtn.2017.07.001

.1

.0.1016/j.omtn.2017.07.001
2/or.2017.6082
).1186/s13046-018-0930-0
).1186/s13046-018-0930-0
).1186/s13046-018-0930-0
).1016/j.abb.2019.01.023
).1186/s13046-019-1158-3

3
3
3

310.1016/j.yexcr.2013.08.023
310.1016/j.yexcr.2013.08.023

2/or.2018.6524
).05.642
)41961

).18632/oncotarget.13985

.0
33
76
5.016
5

.2.05.041
.2.05.041
.2.05.041

.2.05.041

)
)
)

)0
)2/mmr.2017.6621
.0.1016/j.omto.2018.10.002
'10.1016/j.ebiom.2019.08.037
510.1016/j.ebiom.2019.09.046
.4

)2/ol.2022.13200
.0.1186/s13046-022-02270-z
.0.1186/s13046-022-02270-z

57
57

.5/hsz-2020-0345

)27401

)2740110.3727/096504019X15742472027401

.0.1186/s12935-021-02153-x10.1016/S0140-6736(18)30990-510.7150/jca.2027710.3322/ca
.0.1186/s12935-021-02153-x10.1016/S0140-6736(18)30990-510.7150/jca.2027710.3322/ca

501
.4

.410.1182/blood-2016-01-692244
2/or.2017.6056
'
5
.1

2
.7

.145637
.145637
3162828

0257.x10.1111/j.1582-4934.2008.00257.x
04810.1158/0008-5472.CAN-10-2048
:p.26777
210.1016/j.ebiom.2019.02.012
.10.1016/j.ebiom.2019.05.001
.10.1016/j.ebiom.2020.103041

06
06
06
jcla.23135

jcla.24313
jcla.24313
'nar/gkab084
'nar/gkab084

1136/jitc-2021-003793
.0.1016/j.surg.2016.11.036
02

034
'.004

0-3
3-4

-8
8

-X
-8
-8
9

6

05

41961

0

1

3
05

247. x
0910. 1016/j. leukres. 2009. 05. 009
22437
. 3332/ecancer. 2020. 1054

.0.1007/s10565-021-09651-810.1016/j.celrep.2015.12.08610.1016/j.bcp.2017.11.00810.

)0491659

;
.096/fj.201801339RRR

'20

;0

;2.815

:6

).1186/s12935-019-0982-z
;
'ep42319
:0
.0
;8
..004

21037/hbsn.2018.10.07
:b.27851
;.024
;162848
;162848

:4
;01466.x
;01466.x
'-7

3748/wjg.v20.i25.8229
3389/fonc.2021.618764
3389/fonc.2021.618764
26

2610.7754/Clin.Lab.2017.171026

020.106502

.002/1873-3468.12566

09
.11.039
.11.039
01.001
047

.34105

37
09.2010.20.028
09.2010.20.028
706722

.1

0.1007/s10620-008-0329-4

0.1007/s11845-009-0448-8

320663
320663
31510.1158/0008-5472.CAN-11-2315

2/or.2012.2196
)810.1097/IGC.0b013e31827ad2b8

390/cancers3021929
719
02/mmr.2014.2413
02/mmr.2014.2413
3310.1016/j.canlet.2015.05.033
10.1016/j.bbrc.2018.04.129

.11.039
.11.039

2020.11.0310.12122/j.issn.1673-4254.2020.11.03
08
08
29

22612
0.1016/j.lfs.2021.119405

/OTT.S261570

04
02/ol.2019.10160

.24/mol.107.044743
3/onc.2011.399
7/CMAR.S187099

096/fj.201900731RR
41

;2

)1

3/bjc.2017.238
2

;4

)3
'735

)4
.2.010
'57
.54
.5
.5

2101758

215

). 1007/s11010-008-9847-9
;2910.1158/1078-0432.CCR-08-0629
). 18632/oncotarget.18518
392/ijmm.2018.3939
)2/ol.2021.12794
)3

3/jid.2009.365
'

.5
.5

).1016/j.dld.2017.02.009

/abbs/gmx086

)7

210.1371/journal.pone.0097242

33

).3858/emm.2012.44.4.019
.6

)2/ijo.2022.5365
)2/ijo.2022.5365

7501

).1186/s12885-018-4649-2
.05
.792.x10.1111/j.1478-3231.2008.01792.x

)2/ijo.2021.5218
)2/ijo.2021.5218

352

.0
)3/annonc/mdt307
.010.1016/j.bulcan.2015.01.010

)8
)8
)810.26355/eurrev_201812_16508

)115539

)8

39/bcb-2017-0162

). 1186/s13046-019-1091-5

)9

)9
)1
)1

3389/fonc.2021.620873
)5

'-0
043

,

;

;9

.3
). 02. 016

31

. 009
)1
). 104950
)6
3
)4
)5
)1
)1
)6
)6
)8
)8
). 02. 002
.1

[illegible]
39
. 003
. 003

. 04. 027

.1

.03356

3125459

:817

;20840
;20840

;4

:87

.186/1471-2407-10-27
;/onc.2011.222
)810.1016/j.arcmed.2011.06.008

.186/1476-4598-13-52
).1007/s13277-014-2068-9
.010.1016/j.clinre.2016.02.010
1177/1010428317707372
).1007/s11010-014-1976-8
).1007/s11010-014-1976-8

)5
)1
)1

;3

3095414

35

0810.1016/j.arcmed.2011.06.008
cb.28747
cb.28747

410.1016/j.ygyno.2014.01.034
3910.1016/j.biopha.2017.06.089
1177/1010428317707372

01

eExpr.2021039983

05.003

49
49
01110.1016/j.leukres.2012.06.011

25

011
04
04

003
003
2

0182344

31

576
.92
.92

35710.1158/1535-7163.MCT-08-0857
9047.x10.1111/j.1464-410X.2009.09047.x
bc.22465
/cgt.2011.89

0.1007/s13277-013-1393-8
00310.1016/j.jnutbio.2014.01.003

02/ijo.2015.2842
018234410.2174/1568009616666160630182344
0.18632/oncotarget.20610

07
0.18632/oncotarget.14871

2/or.2021.8134

3/bjc.2015.125

c5
c510.1097/CAD.0b013e3283608bc5

2/or.2018.6698

37
.1

112141

0199.x10.1111/j.1742-7843.2007.00199.x
3010.1016/j.canlet.2016.03.030

789
0.1038/s12276-018-0197-8

)0/ijms222212502
)0/ijms222212502

.186/1471-2407-9-323
.186/1471-2407-9-323

:a.14506

:0

:p.27198
:a.14506

2/or.2012.1655

.0.1016/j.bbrc.2020.06.066
.0.1016/j.bbrc.2021.01.004

)5

/ncomms15772
52
)

)
)
2/or.2018.6929

)2/ijo.2014.2523

).1007/s13277-014-1833-0
)2/ijo.2015.3131
)2/ijo.2015.3131
2/ol.2017.7045

2/ol.2017.7045

.096/fj.201801339RRR

19
.0.1016/j.taap.2018.09.018
/OTT.S172379
m4.1860
910.1016/j.ijporl.2020.110249

,

)5

3

)429.x
)429.x
).1007/s11010-010-0453-2
.0.1016/j.ejca.2010.03.038
).1007/s11010-011-0790-9

b.24080
).1007/s10620-013-2673-2
).1186/s12885-016-2437-4

/2018/6953506

71
71
2

802/jgo.2018.29.e99
32/aging.102673

/ol.2018.8338

6
5
1177/1724600820926172

26/sciadv.abf9096

0610.1158/1535-7163.MCT-13-0806
5
2

0
0
.012

3

183110

/ijms16046677

:010.1016/j.biopha.2019.108720
.002/2211-5463.12649
.002/2211-5463.12676
.213352

:2
)4
02.004

/cgt.2008.16
)2/ijo.2013.1925
)2/ijo.2013.1925
)2/mmr.2015.3372

:/or.2017.5584
:/or.2017.5866
.0.4149/neo_2019_190106N18
36
36
3/bjc.2013.579

3/bjc.2013.579
3/bjc.2013.579
3/bjc.2013.579
3/bjc.2013.579
3/bjc.2013.579

4162/nrp.2020.14.2.95
29

728
)/MSM.919219

42
016
)1610.1016/j.cellsig.2014.03.016

). 18632/oncotarget.22245

)4
1
)3
2
40
03.006
)3

.3

jc.23371
)1510.1016/j.lungcan.2009.03.015
.4810.1158/1535-7163.MCT-09-0148

)0322986
)0322849
ed.12138
)2/mmr.2013.1400

310.1016/j.yexmp.2016.05.003
.0.1016/j.bbrc.2016.06.057
3389/fonc.2019.00185
2/or.2020.7710

.0.1007/s10495-022-01718-z10.1016/j.critrevonc.2020.10298810.1016/S0925-5710(98)00

'4
)2/ol.2020.12267

'OTT.S237127

51

.06

)0159210.1182/bloodadvances.2020001592
)0159210.1182/bloodadvances.2020001592
)5
:/ol.2018.9307

).18632/oncotarget.27355

)5

'0

'010.1016/j.bbagen.2021.129870

.2
:6

}c
.0.1016/j.ejca.2008.03.019
)1310.1016/j.jconrel.2017.09.013
)2/ol.2020.12343
:/abbs.2021011
:/abbs.2021011
)7

).1007/s10571-016-0410-z
).1007/s11060-013-1045-2

}1
}1
;

.5
.5

.5252/emmm.201404837

)5
)02
.0.001

i165222

.186/1476-4598-8-125
'jnci/djt210
). 1007/s13277-015-4528-2
.0.1016/j.csbj.2019.12.010

307

)
)
)
)
32
32
32
32
i32/aging.102575

). 18632/oncotarget.19148

4103/jcrt.JCRT_250_17
i.04.003

i.08.026
31

)6
)2

34

22
22
00013-7
06

5
5
09.20120230
09.20120230

0.1007/s00268-013-2010-0
0110.1016/j.arcmed.2013.10.001
0.1007/s11010-014-2069-4
0.1007/s12032-014-0972-x
0610.1016/j.biopha.2019.109306

06

125231
b.28305
061
061
061
061
061
061
061

0/MSM.925298

47
0-1
4-2
02
44

b

1177/0963689719885083
). 18632/oncotarget. 26637
:a. 54163

)74/jbc. M800109200

'10. 1016/j. yexcr. 2020. 112387

)9
[illegible]

ıuto. 27418
!/or. 2015. 4278
:j. 5000656
!018. 01. 00310. 11817/j. issn. 1672-7347. 2018. 01. 003
). 1590/1414-431X20198657

029

.0. 1186/s13046-022-02291-8

)4

:3
:3

). 18632/oncotarget. 10864
!/or. 2017. 5634
). 1016/j. prp. 2019. 152509

;0

/onc.2008.43
).1016/j.prp.2021.153532

?/or.2014.3466

).1007/s00280-018-3759-5

)

ot.22412
70010.1158/1078-0432.CCR-19-2700
a2.217
.5/hsz-2020-0345

;

).1186/s40659-018-0205-4
).1186/s40659-018-0205-4
).1186/s40659-018-0205-4
?8510.1080/21691401.2017.1374285

;510.1016/j.biopha.2019.109185
Expr.2021037227
;390/cancers12061521
)9

.0.1038/s41417-020-00232-110.1002/jso.2459210.3748/wjg.v21.i26.793310.1158/1078-04

5

238/2015.July.31.25
238/2015.July.31.25
238/2015.July.31.25

/or.2021.8187

2

/or.2016.4721

3
3

28

92/ijmm.2018.3753
92/ijmm.2018.3753

2
03556
7
710.1016/j.biopha.2016.01.037
10.1016/j.ctarc.2021.100364

0.1016/j.bbrc.2018.09.191
4

0.1007/s00424-020-02415-z
0.1007/s00424-020-02415-z
.1186/s12935-019-1089-2

2/mmr.2017.7923
2/mmr.2017.7923
2/mmr.2017.7923
4

3
06722
06722
3748/wjg.v18.i23.2956

/or.2014.3698
.18632/oncotarget.21320
.18632/oncotarget.21320
.18632/oncotarget.21320
163238

/en.2016-1903

3610.1158/0008-5472.CAN-11-2536
38/cddis.2016.289
.1007/s12020-012-9762-4
cmm.14763

eExpr.2022041766

40

46
02/ijmm.2011.775
40

40

00
,

0.1007/s00280-018-3590-z

13

29
29
49
49
07

omr.1650
0710.1371/journal.pone.0117097

02/ijo.2015.3137
02/ijo.2015.3137
02/ijo.2015.3137

4710.1097/CAD.0000000000000747

390/cancers12071843

92/mmr.2020.11788
92/mmr.2020.11788

0.1038/s41419-020-03340-w

36
.1007/s12032-010-9605-1
310.1371/journal.pone.0100993
.18632/oncotarget.11540
.18632/oncotarget.11540
/or.2019.7384

c.31868

5

c.28526

08410.1016/j.mrgentox.2019.503084

.18632/oncotarget.13291
-1
.1007/s11010-016-2660-y

ld
)9

31

10.1016/j.ygyno.2014.06.024
10.1016/j.ygyno.2014.06.024
34910.1158/1541-7786.MCR-17-0349

5
is.14083
a.48891

).1038/s41375-019-0593-7

.0

)38/sj.bjc.6605539
)38/sj.bjc.6605539

)5

59
36
3610.1097/IGC.0000000000000136
ox.23418

32

32

)4

5

3210.1016/j.biopha.2018.08.132

04
04

lb
lb
lb
lb
39

lb10.1097/IGC.0b013e31820aaadb
lb10.1097/IGC.0b013e31820aaadb

ox.23435

39

08210.1158/1078-0432.CCR-13-0082
0.1016/j.neo.2014.03.008
7/CMAR.S285367
7/CMAR.S285367

392/ijmm.2014.1752
3.014
3.014

02

00/ijms151222128
00/ijms151222128

3/cdd.2015.133

2/or.2017.5535

2/ol.2018.8180
05

.0.1007/s11033-020-05761-4
.0.1007/s11033-020-05761-4
.0.1007/s11033-020-06099-7

3.004
.9
24

78

24
.910.1016/j.biopha.2015.05.019
).18632/oncotarget.12911
.0.1016/j.clbc.2016.12.005
)/MSM.917345

70
.0.1007/s11845-021-02516-310.1016/S0140-6736(15)00130-010.1016/j.soncn.2019.02.002
.0.1007/s11845-021-02516-310.1016/S0140-6736(15)00130-010.1016/j.soncn.2019.02.002
.0.1007/s11845-021-02516-310.1016/S0140-6736(15)00130-010.1016/j.soncn.2019.02.002

5.016
.2.05.038
.3.10.819
58

2110.1016/j.lungcan.2007.09.021
.1007/s10495-016-1216-7
n.12657
2/ijo.2018.4363
.1016/j.lfs.2020.118088
.11965110.1016/j.biomaterials.2019.119651
2
2
/or.2017.5828
10.1016/j.ebiom.2019.06.022
10.1016/j.ebiom.2019.06.022
07992
143802
9
6
070.x10.1111/j.1365-2362.2008.02070.x
.1007/s10565-012-9221-2
410.1016/j.canlet.2015.08.004
/abbs/gmab066
9
cmm.12811
2
0
07036
6
57
cmm.17041
cmm.17041
2
10.1016/j.ebiom.2019.07.030

;

;1
:9
;124336
;110.1016/j.biopha.2018.10.061

.0.1007/s10863-021-09875-710.1038/sj.bjc.660271510.1016/j.brainres.2019.03.03510.1

'1
'1

)9

.074/jbc.M109.027466
}/onc.2011.222

)2/ijo.2014.2347

!/or.2015.4483

).1016/j.prp.2022.153869

).1186/s13046-019-1106-2

.0.011
)5.011

'47450

)74/jbc.M900735200

)3
)3
'20

)19

)2/mmr.2015.3469
ıs.12846
:a.14461
).1186/s12885-016-2542-4

.0.007

08
08
0.1016/j.bbrc.2008.04.117

0.1038/labinvest.2014.123

0.010

62
06
06

.18632/oncotarget.18631
4
4

.3314

018.07.1510.3969/j.issn.1673-4254.2018.07.15

/ol.2018.9592
/ol.2018.9592
/ol.2018.9592

07881

111/1759-7714.12130
66

0.1016/j.bbrc.2020.09.003
p.29712

.1016/j.prp.2017.09.004
.1016/j.prp.2017.09.004

0

6

./ph.2016.6764

).1038/s41419-019-1347-1
.0.1038/s41420-021-00402-610.1038/s41575-019-0189-810.1146/annurev-pathol-011110-1
.0.1038/s41420-021-00402-610.1038/s41575-019-0189-810.1146/annurev-pathol-011110-1
:71

).1007/s11010-013-1767-7
)2/ijo.2021.5267

)39

).1007/s00432-012-1275-2
)26/97320630081047
)/ijms16034698
)6
)6
)610.1371/journal.pone.0093896

ıth.2510
).18632/oncotarget.14839

).3389/fcell.2020.582695

ıs.13986

).1007/s13277-014-2564-y
)0

)02
).120308
:.005
i9
!1

ì9

.810.1016/j.canlet.2008.08.018

ıt.20838
)710.1016/j.biopha.2012.11.007
.410.1016/j.molonc.2013.12.014
.0.1016/j.bbrc.2016.06.057
ıs.14733

)2/ijo.2016.3738

20

;
:b.27406

;

jcmm.16822

)9.20130169

ì07
:b.29833

:b.29833

).1038/s41388-018-0467-x

).1007/s10620-012-2132-5
36.2016.11.01310.3760/cma.j.issn.0253-3766.2016.11.013
'OTT.S186981
35
'5710.1158/1078-0432.CCR-11-2757
'5710.1158/1078-0432.CCR-11-2757
'5710.1158/1078-0432.CCR-11-2757
39
)02

392/ijmm.2013.1299
392/ijmm.2013.1299
392/ijmm.2013.1299
.0.1038/s41419-020-03185-3

)4

'7
5
37
37

.210.1016/j.canlet.2013.04.012

).18632/oncotarget.21996
)2/ol.2020.12287
)2/ol.2020.12287
)2/ol.2020.12287
55

).1007/s11596-012-0049-z
5/2021/9674761

2252.x
)6

'0

). 1007/s00280-008-0697-7
). 1007/s00280-008-0697-7
). 1007/s00280-008-0697-7

.0. 1007/s00441-019-03105-8

ıth. 4681

). 21873/anticanres. 13227
;9
.6

)1. 009

}. 007

?/or. 2018. 6735

)6
)1
)1
?5
;

;910. 1016/j. tranon. 2021. 101159

'. 12. 015

'.12.015
)6
.9

36
3610.1371/journal.pone.0053436
22

.0.1038/s41420-021-00622-w

28
).1007/s12094-018-1937-x
.5/hsz-2017-0274
.5/hsz-2017-0274

)0485400
)0485400
)0485400

)5
26

)

)-7
)-7
10.035

)9.2011.02.021
)9.2011.02.021

373671
.24/mol.107.042382
2/ol.2017.5895

r.2012.44

). 1016/j.mcp.2019.02.004
.6

)38/cddis.2016.289
.0.1016/j.taap.2022.116035
.4
.4
)93/carcin/bgab037
2
b.29833

)7

.0.1186/s12935-021-02060-110.3322/caac.2149210.1038/s41572-020-00224-310.1186/s404

)2

3
3

)4110.1016/j.urology.2008.09.041
)4110.1016/j.urology.2008.09.041

7/CMAR.S172722

2

.9
004

2

.0. 1186/s13046-021-01854-5

.0. 1186/s13046-021-01989-5
:hno. 55814
:hno. 55814
.0. 1186/s13046-021-01989-510. 3322/caac. 2165410. 1038/ncb303810. 1016/j. eururo. 2016. 0

049
5
5
.47814

707
3/onc. 2012. 355
3/onc. 2012. 355
). 1285
.0. 1016/j. bbrc. 2015. 01. 017

06

.3

.5

.510. 1016/j. canlet. 2011. 01. 015

2/ol.2017.7326

).1186/s12935-019-1050-4

.0.1245/s10434-020-08518-2
.8

.45
).002

392/ijmm.2021.4887

:9ra06378a

)63

;
2710.1016/j.canlet.2020.07.027

392/mmr.2020.10986
)52
)/neo_2012_070
2

)38/cddis.2016.289

1186/1476-4598-13-190
03/carcin/bgx059

68

074/jbc.M111.277103
ca.56135
ca.56135

26

4.04.004

2/mmr.2011.426
2/mmr.2011.426
2/mmr.2011.426
0.1016/j.abb.2012.07.003
4.04.00410.1016/j.oraloncology.2014.04.004
111/1440-1681.12672
3152108
167

2/or.2015.3994
2/or.2015.3994
2/or.2015.3994

-X
86057
86057

0

/iovs.10-6973
8605710.3727/096504015X14452563486057
8605710.3727/096504015X14452563486057

310.1016/j.biopha.2022.112713
/ol.2018.9205

.04.037

0

8
33656

.1007/s00432-008-0460-9
.1007/s00292-009-1147-y
4810.1158/1535-7163.MCT-09-0148
/ijms17010137
.18632/oncotarget.24091
p.27666
2/etm.2018.7040

.1186/s12931-019-1071-5

0489759

3
3

:310.1016/j.canlet.2019.02.043

)5

.077
)12

i8

38
38

24

)58
-00010
)t.20838

.jbs.18969
)1.2017.45.01310.3760/cma.j.issn.0376-2491.2017.45.013
).18632/oncotarget.26637
).21873/anticanres.13227
i810.26355/eurrev_202005_21168
)0283210.1182/bloodadvances.2020002832

)52

)6
2/or.2018.6628

:hno.45803

/CMAR.S172722

8
m4.3841
2

210.1016/j.biopha.2018.10.042

9
0.1186/s13046-020-01634-7

5
01.037
01.037

2.009

6
6
/onc.2008.326
.18632/oncotarget.13067

3
1

0.1016/j.bbrc.2013.01.022
0.1186/s12885-021-09025-7

p4.1114

of.1757

07992

6

.07577
049
;
;
;
;
:47814

)7029.52

'07
;/onc.2011.588
;/onc.2012.355
'bcj.2012.46
)4010.1158/1535-7163.MCT-13-0040
.0.1016/j.bbrc.2015.01.017
)710.1371/journal.pone.0165107

).1007/s00018-013-1485-z

.52

'4
;6

).1007/s00280-016-2964-3
).1007/s00280-016-2964-3
).1007/s00280-016-2964-3
:b.26594
!/ol.2017.7237

2/ol.2017.7237
.5/hsz-2017-0274
.5/hsz-2017-0274
.0.1186/s40659-020-00286-3

.2
39
39
3910.1371/journal.pone.0093439

.0.1007/s11033-020-05411-9

392/mmr.2022.12617

)410.1016/j.biopha.2011.02.004
)410.1016/j.biopha.2011.02.004

:b.24481
).1186/s12943-015-0357-6

.0.1186/s13058-020-01302-w

29

2/or.2017.5984

5.013
)3
)3
)3
)3
)3
)3
)3
)3
)3
)3
)3

48
79

;

2310.1007/978-1-4939-9220-1_23

i0
.0.1016/j.bbrc.2017.06.185
.0.1016/j.bbrc.2017.06.185

25
25
25
25
25
2510.1097/CAD.0000000000000225
)2/etm.2018.7040
)2/etm.2018.7040
29610.1158/1535-7163.MCT-13-0296
.0.1038/s41419-021-04474-110.1038/346240a010.1038/346245a010.1016/j.stem.2012.12.0
.821
;

.2.10.525
)7
;4

3100405
3100405

;2
;2
;2
;2
;2

52

). 1007/s10930-011-9354-9
'onc. 2013. 88
5210. 1371/journal. pone. 0079162
2/or. 2015. 4287
.0. 1186/s12935-020-01489-0

110. 1016/j. yexcr. 2021. 112734

.0. 1016/j. bbrc. 2021. 01. 029

7/CMAR. S277399
7/CMAR. S277399

5

'0

)18

ıac. 2155110. 1056/NEJMoa04333110. 1080/15548627. 2019. 159875210. 1096/fj. 201901021RR10
ıac. 2155110. 1056/NEJMoa04333110. 1080/15548627. 2019. 159875210. 1096/fj. 201901021RR10

1002/bies.2021010.1002/jcb.2561210.1111/jcmm.1412310.1007/978-3-030-43085-6_410.1

)038-310.3109/07388551.2015.101595710.1038/nature0592510.1080/15548627.2016.116435

32. CCR-14-219110. 1016/j. molonc. 2013. 10. 00710. 1016/j. canlet. 2013. 06. 01310. 1186/147

210.1093/jnci/djx21410.1097/GCO.000000000000033810.1016/j.tem.2013.12.00110.1016/j
210.1093/jnci/djx21410.1097/GCO.000000000000033810.1016/j.tem.2013.12.00110.1016/j
210.1093/jnci/djx21410.1097/GCO.000000000000033810.1016/j.tem.2013.12.00110.1016/j

.016/j.mayocp.2019.01.01310.1172/jci3187110.1016/s0140-6736(16)30958-810.1016/j.bb

3023510.1053/j.gastro.2019.06.04710.1002/(SICI)1097-0142(19960115)77:2<255::AID-C
3023510.1053/j.gastro.2019.06.04710.1002/(SICI)1097-0142(19960115)77:2<255::AID-C

25-019-0662-510. 1016/j. oraloncology. 2016. 12. 01010. 1146/annurev-biochem-061516-044

)6.02010.1001/jama.2020.1759810.1016/j.cub.2016.06.01110.1172/JCI13959710.1038/nsm

)0710.1242/dev.09179310.1006/dbio.2000.988310.1158/1078-0432.CCR-10-115510.1002/cn

). 1093/neuonc/noy07210. 1007/s12035-011-8196-y10. 1038/nrg. 2015. 1010
). 1093/neuonc/noy07210. 1007/s12035-011-8196-y10. 1038/nrg. 2015. 1010

038/ncomms1215610.1016/j.cmet.2007.03.00710.18632/oncotarget.1278

[illegible]910. 21037/atm. 2019. 02. 2210. 1371/journal. pone. 009015110. 1038/onc. 2

'6-4598-10-3810.1002/mc.2194410.1186/1476-4598-14-310.1186/s13045-

i.arr.2019.10096110.1007/s13277-015-3300-y10.1002/cmdc.20170041410
i.arr.2019.10096110.1007/s13277-015-3300-y10.1002/cmdc.20170041410
i.arr.2019.10096110.1007/s13277-015-3300-y10.1002/cmdc.20170041410

rc.2006.10.11210.1038/s41598-017-03368-710.18632/oncotarget.21022

NCR6>3.0.CO;2-L10.1038/cdd.2014.17010.1158/1078-0432.CCR-16-25061
NCR6>3.0.CO;2-L10.1038/cdd.2014.17010.1158/1078-0432.CCR-16-25061

91610. 1016/j. trecan. 2019. 08. 00510. 1038/s41580-019-0099-110. 1111/a

ıb112810.1016/j.molcel.2017.10.01310.3389/fonc.2018.0050210.1038/n

ıcr. 2656610. 1016/j. canep. 2013. 05. 00210. 1182/blood-2010-04-28235010
