# Supplementary material for: Role of PPARG in Chemosensitivity-Regulating Network for Hypopharyngeal Squamous Cell Carcinoma
Source: PPAR Res. 2023 Sep 25;2023:6019318. doi: 10.1155/2023/6019318 (PMC10545467; doi:10.1155/2023/6019318)
Supplement: Supplementary 3 — Supplementary Table 3: the reference information supporting the HSCC chemosensitivity-regulating network. [file 6019318.f3.pdf]

Ref info supporting the HSCC-chemosensitivity regulating network

Relation Name	PMID	DOI
Regulation: PPARG ---> MAPK1	30278161	10.1016/j.cbi.2018.09.019
Regulation: PPARG ---> MAPK1	15649892	10.1074/jbc.M410445200
Regulation: PPARG ---> MAPK1	16524877	10.1074/jbc.M513786200
Regulation: PPARG ---> MAPK1	15632188	10.1074/jbc.M409447200
Regulation: PPARG ---> MAPK1	18411276	10.1074/jbc.M705822200
Regulation: PPARG ---> MAPK1	28527699	10.1016/j.phrs.2017.05.008
Regulation: PPARG ---> MAPK1	27792919	10.1016/j.intimp.2016.09.028
Regulation: PPARG ---> MAPK1	33539723	10.1016/j.stem.2020.12.008
Regulation: PPARG ---> MAPK1	28580280	10.1016/j.molmet.2017.03.003
Regulation: PPARG ---> MAPK1	22591774	10.1016/j.archoralbio.2012.04.015
Regulation: PPARG ---> MAPK1	16945329	10.1016/j.bbrc.2006.08.085
Regulation: PPARG ---> MAPK1	17583675	10.1016/j.bbrc.2007.05.222
Regulation: PPARG ---> MAPK1	19397913	10.1016/j.yjmcc.2009.04.011
Regulation: PPARG ---> MAPK1	19010350	10.1016/j.pharmthera.2008.10.002
Regulation: PPARG ---> MAPK1	18848847	10.1016/j.bbamcr.2008.09.002
Regulation: PPARG ---> MAPK1	20537761	10.1016/j.ejcb.2010.04.002
Regulation: PPARG ---> MAPK1	18513951	10.1016/j.jsbmb.2008.04.005
Regulation: PPARG ---> MAPK1	19443277	10.1016/j.clim.2009.04.004
Regulation: PPARG ---> MAPK1	23219716	10.1016/j.ajpath.2012.10.007
Regulation: PPARG ---> MAPK1		10.1016/j.jalz.2014.05.748
Regulation: PPARG ---> MAPK1	25557278	10.1016/j.freeradbiomed.2014.12.019
Regulation: PPARG ---> MAPK1	26607460	10.1016/j.ejphar.2015.11.040
Regulation: PPARG ---> MAPK1	26556104	10.1016/j.cyto.2015.11.001
Regulation: PPARG ---> MAPK1	18382765	
Regulation: PPARG ---> MAPK1	18411276	
Regulation: PPARG ---> MAPK1	18411276	
Regulation: PPARG ---> MAPK1	16971554	
Regulation: PPARG ---> MAPK1	16971554	
Regulation: PPARG ---> MAPK1	16524877	
Regulation: PPARG ---> MAPK1	16524877	
Regulation: PPARG ---> MAPK1	16524877	
Regulation: PPARG ---> MAPK1	15649892	
Regulation: PPARG ---> MAPK1	15632188	
Regulation: PPARG ---> MAPK1	11231915	
Regulation: PPARG ---> MAPK1	17389706	
Regulation: PPARG ---> MAPK1	25632270	10.1186/s12950-014-0044-1
Regulation: PPARG ---> MAPK1	26818512	10.1523/JNEUROSCI.3280-15.2016
Regulation: PPARG ---> MAPK1	27718123	10.1007/s13105-016-0523-3
Regulation: PPARG ---> MAPK1	27718123	10.1007/s13105-016-0523-3
Regulation: PPARG ---> MAPK1	27718123	10.1007/s13105-016-0523-3
Regulation: PPARG ---> MAPK1	28673319	10.1186/s13046-017-0557-6
Regulation: PPARG ---> MAPK1	28466688	10.1177/1074248417707049
Regulation: PPARG ---> MAPK1	29350308	10.1007/s10549-017-4649-y
Regulation: PPARG ---> MAPK1	28534168	10.1007/s00394-017-1473-4
Regulation: PPARG ---> MAPK1	31233701	10.1071/RD19108
Regulation: PPARG ---> MAPK1	32417836	10.1159/000505209
Regulation: PPARG ---> MAPK1	32417836	10.1159/000505209
Regulation: PPARG ---> MAPK1	32417836	10.1159/000505209
Regulation: PPARG ---> MAPK1	32417836	10.1159/000505209
Regulation: PPARG ---> MAPK1	32417836	10.1159/000505209
Regulation: PPARG ---> MAPK1	32958825	10.1038/s41416-020-01088-w

Regulation: PPARG ---> MAPK1	32867639	10.2174/0929867327999200820173853
Regulation: PPARG ---> MAPK1	22502865	10.1186/1755-1536-5-5
Regulation: PPARG ---> MAPK1	22502865	10.1186/1755-1536-5-5
Regulation: PPARG ---> MAPK1	22426809	10.1007/s12253-012-9517-9
Regulation: PPARG ---> MAPK1	21208163	10.2174/156720211794520224
Regulation: PPARG ---> MAPK1	21532147	10.1248/bpb.34.618
Regulation: PPARG ---> MAPK1	21955520	10.1158/1940-6207.CAPR-11-0256
Regulation: PPARG ---> MAPK1	23175826	10.1523/JNEUROSCI.2153-12.2012
Regulation: PPARG ---> MAPK1	24259663	10.1242/jcs.128306
Regulation: PPARG ---> MAPK1	24092354	10.1007/978-3-642-38664-0-21
Regulation: PPARG ---> MAPK1	24623782	10.1523/JNEUROSCI.4024-13.2014
Regulation: PPARG ---> MAPK1	24623782	10.1523/JNEUROSCI.4024-13.2014
Regulation: PPARG ---> MAPK1	24623782	10.1523/JNEUROSCI.4024-13.2014
Regulation: PPARG ---> MAPK1	24623782	10.1523/JNEUROSCI.4024-13.2014
Regulation: PPARG ---> MAPK1	24623782	10.1523/JNEUROSCI.4024-13.2014
Regulation: PPARG ---> MAPK1	24623782	10.1523/JNEUROSCI.4024-13.2014
Regulation: PPARG ---> MAPK1	24623782	10.1523/JNEUROSCI.4024-13.2014
Regulation: PPARG ---> MAPK1	26398946	10.1242/dmm.021550
Regulation: PPARG ---> MAPK1	26398946	10.1242/dmm.021550
Regulation: PPARG ---> MAPK1	26398946	10.1242/dmm.021550
Regulation: PPARG ---> MAPK1	20300579	10.1155/2009/43867310.1155/2009/438673
Regulation: PPARG ---> MAPK1	20537761	10.1016/j.ejcb.2010.04.00210.1016/j.ejcb.2010
Regulation: PPARG ---> MAPK1	24623782	10.1523/JNEUROSCI.4024-13.201410.1523/JNEUROS
Regulation: PPARG ---> MAPK1	25557278	10.1016/j.freeradbiomed.2014.12.01910.1016/j.
Regulation: PPARG ---> MAPK1	26125905	10.4238/2015.June.26.510.4238/2015.June.26.5
Regulation: PPARG ---> MAPK1	30018246	10.3390/md1607023810.3390/md16070238
Regulation: PPARG ---> MAPK1	30189217	10.1016/j.lfs.2018.09.00410.1016/j.lfs.2018.0
Regulation: PPARG ---> MAPK1	31233701	10.1071/RD1910810.1071/RD19108
Regulation: PPARG ---> MAPK1	32417836	10.1159/00050520910.1159/000505209
Regulation: PPARG ---> MAPK1		10.1016/j.archoralbio.2012.04.015
Regulation: PPARG ---> MAPK1		10.1071/RD19108
Regulation: PPARG ---> MAPK1		10.1007/s12253-012-9517-9
Regulation: PPARG ---> MAPK1		10.2174/156720211794520224
Regulation: PPARG ---> MAPK1		10.1523/JNEUROSCI.4024-13.2014
Regulation: PPARG ---> MAPK1		10.1523/JNEUROSCI.4024-13.2014
Regulation: PPARG ---> MAPK1		10.1523/JNEUROSCI.4024-13.2014
Regulation: PPARG ---> MAPK1		10.1523/JNEUROSCI.4024-13.2014
Regulation: PPARG ---> MAPK1		10.1523/JNEUROSCI.4024-13.2014
Regulation: PPARG ---> MAPK1		10.1523/JNEUROSCI.4024-13.2014
Regulation: PPARG ---> MAPK1		10.1523/JNEUROSCI.4024-13.2014
Regulation: PPARG ---> MAPK1	25874449	10.1371/journal.pone.0123724
Regulation: PPARG ---> MAPK1	27084771	10.1007/s11064-016-1913-1
Regulation: PPARG ---> MAPK1	19142226	
Regulation: PPARG ---> MAPK1		10.1016/j.archoralbio.2012.04.015
Regulation: PPARG ---> MAPK1		10.1016/j.archoralbio.2012.04.015
Regulation: PPARG ---> MAPK1		10.1016/j.bbrc.2004.08.101
Regulation: PPARG ---> MAPK1	18423937	10.1016/j.peptides.2008.03.001
Regulation: PPARG ---> MAPK1	21664456	10.1016/j.freeradbiomed.2011.05.027
Regulation: PPARG ---> MAPK1	22633083	10.1016/j.cyto.2012.04.042
Regulation: PPARG ---> MAPK1	22591774	10.1016/j.archoralbio.2012.04.015
Regulation: PPARG ---> MAPK1	22591774	10.1016/j.archoralbio.2012.04.015
Regulation: PPARG ---> MAPK1	25557278	10.1016/j.freeradbiomed.2014.12.019
Regulation: PPARG ---> MAPK1	25557278	10.1016/j.freeradbiomed.2014.12.019
Regulation: PPARG ---> MAPK1	25557278	10.1016/j.freeradbiomed.2014.12.019

Regulation: PPARG ---> MAPK1	25917921	10.1016/j.prostaglandins.2015.04.007
Regulation: PPARG ---> MAPK1	26526086	10.1016/j.intimp.2015.10.017
Regulation: PPARG ---> MAPK1	16815847	
Regulation: PPARG ---> MAPK1	16815847	
Regulation: PPARG ---> MAPK1	16815847	
Regulation: PPARG ---> MAPK1	16815847	
Regulation: PPARG ---> MAPK1	16815847	
Regulation: PPARG ---> MAPK1	16815847	
Regulation: PPARG ---> MAPK1	16815847	
Regulation: PPARG ---> MAPK1	16815847	
Regulation: PPARG ---> MAPK1	16815847	
Regulation: PPARG ---> MAPK1	16051889	
Regulation: PPARG ---> MAPK1	17519361	
Regulation: PPARG ---> MAPK1	22934105	10.1155/2012/362085
Regulation: PPARG ---> MAPK1	21840940	10.1096/fj.11-188607
Regulation: PPARG ---> MAPK1	24520152	10.1152/physiolgenomics.00158.2013
Regulation: PPARG ---> MAPK1	16815847	
Regulation: PPARG ---> MAPK1	16815847	
Regulation: PPARG ---> MAPK1	16815847	
Regulation: PPARG ---> MAPK1	20672248	10.1002/cbf.1681
Regulation: PPARG ---> MAPK1	25557278	10.1016/j.freeradbiomed.2014.12.019
Regulation: PPARG ---> MAPK1	18996358	10.1016/j.bcp.2008.10.013
Regulation: PPARG ---> MAPK1	15050417	10.1016/j.taap.2003.12.005
Regulation: PPARG ---> MAPK1		10.1016/j.jnutbio.2015.06.005
Regulation: PPARG ---> MAPK1	18382765	
Regulation: PPARG ---> MAPK1	16815847	
Regulation: PPARG ---> MAPK1	16815847	
Regulation: PPARG ---> MAPK1	21840940	10.1096/fj.11-188607
Regulation: PPARG ---> MAPK1	24223584	10.1155/2013/582809
Regulation: PPARG ---> MAPK1		10.1523/JNEUROSCI.4024-13.2014
Regulation: PPARG ---> MAPK1		
Regulation: PPARG ---> MAPK1	29351409	10.1152/ajpcell.00143.2017
Regulation: PPARG ---> MAPK1	29351409	10.1152/ajpcell.00143.2017
Regulation: PPARG ---> MAPK1	29351409	10.1152/ajpcell.00143.2017
Regulation: PPARG ---> MAPK1	35563317	10.3390/ijms23094925
Regulation: PPARG ---> MAPK1	34370683	10.1530/JME-20-0334
negative Expression: AKT1 ---| RA	33388318	10.1053/j.gastro.2020.12.061
negative Expression: AKT1 ---| RA	21282107	10.1074/jbc.M110.217653
negative Expression: AKT1 ---| RA	25378391	10.1074/jbc.M114.593285
negative Expression: AKT1 ---| RA	14960575	10.1074/jbc.M400905200
negative Expression: AKT1 ---| RA	23871831	10.1016/j.bbamcr.2013.07.005
negative Expression: AKT1 ---| RA	14960575	
negative Expression: AKT1 ---| RA	21282107	10.1074/jbc.M110.217653
negative Expression: AKT1 ---| RA	25378391	10.1074/jbc.M114.593285
negative Expression: AKT1 ---| RA	26631033	10.1007/s13277-015-4504-x
positive Regulation: RAC1 --+> AK	15308574	10.1182/blood-2004-03-0781
positive Regulation: RAC1 --+> AK	18684863	10.1182/blood-2008-03-144667
positive Regulation: RAC1 --+> AK	16873675	10.1182/blood-2006-02-005959
positive Regulation: RAC1 --+> AK	12393431	10.1182/blood-2002-04-1251
positive Regulation: RAC1 --+> AK	12393431	10.1182/blood-2002-04-1251
positive Regulation: RAC1 --+> AK	12393431	10.1182/blood-2002-04-1251
positive Regulation: RAC1 --+> AK	12393431	10.1182/blood-2002-04-1251
positive Regulation: RAC1 --+> AK	12393431	10.1182/blood-2002-04-1251
positive Regulation: RAC1 --+> AK	12393431	10.1182/blood-2002-04-1251

positive Regulation: RAC1 --+> AK 12393431 10.1182/blood-2002-04-1251
positive Regulation: RAC1 --+> AK 27018294 10.1016/j.brainres.2016.03.025
positive Regulation: RAC1 --+> AK 29129468 10.1016/j.expneurol.2017.10.030
positive Regulation: RAC1 --+> AK 33388318 10.1053/j.gastro.2020.12.061
positive Regulation: RAC1 --+> AK 25586182 10.1074/jbc.M114.633701
positive Regulation: RAC1 --+> AK 25586182 10.1074/jbc.M114.633701
positive Regulation: RAC1 --+> AK 16551621 10.1074/jbc.M513243200
positive Regulation: RAC1 --+> AK 17519230 10.1074/jbc.M702182200
positive Regulation: RAC1 --+> AK 17519230 10.1074/jbc.M702182200
positive Regulation: RAC1 --+> AK 17519230 10.1074/jbc.M702182200
positive Regulation: RAC1 --+> AK 23640896 10.1074/jbc.M113.467647
positive Regulation: RAC1 --+> AK 23640896 10.1074/jbc.M113.467647
positive Regulation: RAC1 --+> AK 23640896 10.1074/jbc.M113.467647
positive Regulation: RAC1 --+> AK 23640896 10.1074/jbc.M113.467647
positive Regulation: RAC1 --+> AK 16698790 10.1074/jbc.M603508200
positive Regulation: RAC1 --+> AK 25378391 10.1074/jbc.M114.593285
positive Regulation: RAC1 --+> AK 15677464 10.1074/jbc.M411625200
positive Regulation: RAC1 --+> AK 17604276 10.1074/jbc.M703132200
positive Regulation: RAC1 --+> AK 15579468 10.1074/jbc.M410508200
positive Regulation: RAC1 --+> AK 16339142 10.1074/jbc.M510434200
positive Regulation: RAC1 --+> AK 16339142 10.1074/jbc.M510434200
positive Regulation: RAC1 --+> AK 16339142 10.1074/jbc.M510434200
positive Regulation: RAC1 --+> AK 16339142 10.1074/jbc.M510434200
positive Regulation: RAC1 --+> AK 16339142 10.1074/jbc.M510434200
positive Regulation: RAC1 --+> AK 16339142 10.1074/jbc.M510434200
positive Regulation: RAC1 --+> AK 11689561 10.1074/jbc.M107474200
positive Regulation: RAC1 --+> AK 32244053 10.1016/j.gene.2020.144635
positive Regulation: RAC1 --+> AK 31018152 10.1016/j.toxlet.2019.04.017
positive Regulation: RAC1 --+> AK 31018152 10.1016/j.toxlet.2019.04.017
positive Regulation: RAC1 --+> AK 29329780 10.1016/j.cellsig.2018.01.004
positive Regulation: RAC1 --+> AK 29329780 10.1016/j.cellsig.2018.01.004
positive Regulation: RAC1 --+> AK 33152487 10.1016/j.semcancer.2020.10.012
positive Regulation: RAC1 --+> AK 27453460 10.1016/j.pan.2016.07.002
positive Regulation: RAC1 --+> AK 31257073 10.1016/j.ccell.2019.05.015
positive Regulation: RAC1 --+> AK 31257073 10.1016/j.ccell.2019.05.015
positive Regulation: RAC1 --+> AK 31257073 10.1016/j.ccell.2019.05.015
positive Regulation: RAC1 --+> AK 28007913 10.1074/mcp.M116.064428
positive Regulation: RAC1 --+> AK 30590131 10.1016/j.jprot.2018.12.019
positive Regulation: RAC1 --+> AK 32460002 10.1016/j.trecan.2020.02.021
positive Regulation: RAC1 --+> AK 32460002 10.1016/j.trecan.2020.02.021
positive Regulation: RAC1 --+> AK 15093747 10.1016/j.yexcr.2004.02.002
positive Regulation: RAC1 --+> AK 15093747 10.1016/j.yexcr.2004.02.002
positive Regulation: RAC1 --+> AK 17292356 10.1016/j.exer.2006.12.012
positive Regulation: RAC1 --+> AK 20538003 10.1016/j.yjmcc.2010.05.007
positive Regulation: RAC1 --+> AK 20538003 10.1016/j.yjmcc.2010.05.007
positive Regulation: RAC1 --+> AK 21925199 10.1016/j.advenzreg.2011.09.001
positive Regulation: RAC1 --+> AK 19118901 10.1016/j.molimm.2008.11.006
positive Regulation: RAC1 --+> AK 18086571 10.1016/j.bbamcr.2007.10.024
positive Regulation: RAC1 --+> AK 20144672 10.1016/j.neuint.2010.01.015
positive Regulation: RAC1 --+> AK 20307615 10.1016/j.neuint.2010.03.005
positive Regulation: RAC1 --+> AK 20307615 10.1016/j.neuint.2010.03.005
positive Regulation: RAC1 --+> AK 15388231 10.1016/j.biochi.2004.07.008
positive Regulation: RAC1 --+> AK 15013523 10.1016/j.canlet.2003.08.035
positive Regulation: RAC1 --+> AK 20074642 10.1016/j.cellsig.2010.01.001

positive Regulation: RAC1 --+>	AK	20074642	10.1016/j.cellsig.2010.01.001
positive Regulation: RAC1 --+>	AK	20074642	10.1016/j.cellsig.2010.01.001
positive Regulation: RAC1 --+>	AK	20074642	10.1016/j.cellsig.2010.01.001
positive Regulation: RAC1 --+>	AK	21683139	10.1016/j.cellsig.2011.05.022
positive Regulation: RAC1 --+>	AK	21251810	10.1016/j.gde.2010.12.004
positive Regulation: RAC1 --+>	AK	15752980	10.1016/j.tcb.2005.01.002
positive Regulation: RAC1 --+>	AK	21474067	10.1016/j.molcel.2011.03.017
positive Regulation: RAC1 --+>	AK	21474067	10.1016/j.molcel.2011.03.017
positive Regulation: RAC1 --+>	AK	21474067	10.1016/j.molcel.2011.03.017
positive Regulation: RAC1 --+>	AK	21474067	10.1016/j.molcel.2011.03.017
positive Regulation: RAC1 --+>	AK	20060361	10.1016/j.coph.2009.11.004
positive Regulation: RAC1 --+>	AK	12086876	10.1016/S1535-6108(02)00033-8
positive Regulation: RAC1 --+>	AK	23623748	10.1016/j.cmet.2013.03.017
positive Regulation: RAC1 --+>	AK	23706742	10.1016/j.cell.2013.04.031
positive Regulation: RAC1 --+>	AK	23706742	10.1016/j.cell.2013.04.031
positive Regulation: RAC1 --+>	AK	24216610	10.1016/j.cellsig.2013.11.007
positive Regulation: RAC1 --+>	AK	24508121	10.1016/j.bbagen.2014.01.033
positive Regulation: RAC1 --+>	AK	24508121	10.1016/j.bbagen.2014.01.033
positive Regulation: RAC1 --+>	AK	24880064	10.1016/j.cellsig.2014.05.015
positive Regulation: RAC1 --+>	AK	24721474	10.1016/j.semcdb.2014.04.008
positive Regulation: RAC1 --+>	AK	19129452	10.1128/JVI.01972-0810.1128/JVI.01972-08
positive Regulation: RAC1 --+>	AK	32275841	10.1080/15384101.2020.174979010.1080/15384101
positive Regulation: RAC1 --+>	AK	31257073	10.1016/j.ccell.2019.05.01510.1016/j.ccell.20
positive Regulation: RAC1 --+>	AK	18579797	10.1182/blood-2008-01-13206810.1182/blood-200
positive Regulation: RAC1 --+>	AK	19293637	10.3858/emm.2009.41.3.02110.3858/emm.2009.41.
positive Regulation: RAC1 --+>	AK	23554696	10.1016/S1674-8301(11)60032-810.1016/S1674-83
positive Regulation: RAC1 --+>	AK	32823607	10.3390/ijms21165812
positive Regulation: RAC1 --+>	AKT1		10.1016/j.trecan.2020.02.021
positive Regulation: RAC1 --+>	AKT1		10.1016/j.trecan.2020.02.021
positive Regulation: RAC1 --+>	AKT1		10.1016/j.advenzreg.2011.09.001
positive Regulation: RAC1 --+>	AKT1		10.1016/j.cellsig.2010.01.001
positive Regulation: RAC1 --+>	AKT1		10.1016/j.cellsig.2010.01.001
positive Regulation: RAC1 --+>	AKT1		10.1016/j.cellsig.2010.01.001
positive Regulation: RAC1 --+>	AKT1		10.1016/j.cellsig.2010.01.001
positive Regulation: RAC1 --+>	AK	25022196	10.1016/j.cytogfr.2014.06.002
positive Regulation: RAC1 --+>	AK	26463037	10.1016/j.ejphar.2015.10.018
positive Regulation: RAC1 --+>	AK	25843002	10.1016/j.ccell.2015.02.014
positive Regulation: RAC1 --+>	AK	16321803	
positive Regulation: RAC1 --+>	AK	19497102	
positive Regulation: RAC1 --+>	AK	17855349	
positive Regulation: RAC1 --+>	AK	17604276	
positive Regulation: RAC1 --+>	AK	17519230	
positive Regulation: RAC1 --+>	AK	17519230	
positive Regulation: RAC1 --+>	AK	16774691	
positive Regulation: RAC1 --+>	AK	16551621	
positive Regulation: RAC1 --+>	AK	16339142	
positive Regulation: RAC1 --+>	AK	16339142	
positive Regulation: RAC1 --+>	AK	16339142	
positive Regulation: RAC1 --+>	AK	16339142	
positive Regulation: RAC1 --+>	AK	16339142	
positive Regulation: RAC1 --+>	AK	16339142	
positive Regulation: RAC1 --+>	AK	16339142	
positive Regulation: RAC1 --+>	AK	16014609	
positive Regulation: RAC1 --+>	AK	15677464	

positive Regulation: RAC1 --+>	AK	15579468	
positive Regulation: RAC1 --+>	AK	11805146	
positive Regulation: RAC1 --+>	AK	11689561	
positive Regulation: RAC1 --+>	AK	11369774	
positive Regulation: RAC1 --+>	AK	11369774	
positive Regulation: RAC1 --+>	AK	18599867	
positive Regulation: RAC1 --+>	AK	15027896	
positive Regulation: RAC1 --+>	AK	11803464	
positive Regulation: RAC1 --+>	AK	11803464	
positive Regulation: RAC1 --+>	AK	11803464	
positive Regulation: RAC1 --+>	AK	11803464	
positive Regulation: RAC1 --+>	AK	19179620	
positive Regulation: RAC1 --+>	AK	19179620	
positive Regulation: RAC1 --+>	AK	17804488	
positive Regulation: RAC1 --+>	AK	17804488	
positive Regulation: RAC1 --+>	AK	17682064	
positive Regulation: RAC1 --+>	AK	16798728	
positive Regulation: RAC1 --+>	AK	16798728	
positive Regulation: RAC1 --+>	AK	16798728	
positive Regulation: RAC1 --+>	AK	16670255	
positive Regulation: RAC1 --+>	AK	15226424	
positive Regulation: RAC1 --+>	AK	12842860	
positive Regulation: RAC1 --+>	AK	12842860	
positive Regulation: RAC1 --+>	AK	12842860	
positive Regulation: RAC1 --+>	AK	12842860	
positive Regulation: RAC1 --+>	AK	12842860	
positive Regulation: RAC1 --+>	AK	12842860	
positive Regulation: RAC1 --+>	AK	12842860	
positive Regulation: RAC1 --+>	AK	32432925	10.1152/ajpcell.00505.2019
positive Regulation: RAC1 --+>	AK	32432925	10.1152/ajpcell.00505.2019
positive Regulation: RAC1 --+>	AK	32432925	10.1152/ajpcell.00505.2019
positive Regulation: RAC1 --+>	AK	32568828	10.1097/CAD.000000000000096110.1097/CAD.0000[illegible]
positive Regulation: RAC1 --+>	AK	12842860	
positive Regulation: RAC1 --+>	AK	12842860	
positive Regulation: RAC1 --+>	AKT1		10.1074/mcp.M116.064428
positive Regulation: RAC1 --+>	AK	29749029	10.1113/JP27560210.1113/JP275602
positive Regulation: RAC1 --+>	AK	9601639	10.1016/S0960-9822(98)70224-6
positive Regulation: RAC1 --+>	AK	24216610	10.1016/j.cellsig.2013.11.007
positive Regulation: RAC1 --+>	AK	16698790	
positive Regulation: RAC1 --+>	AK	16339142	
positive Regulation: RAC1 --+>	AK	10973919	
positive Regulation: RAC1 --+>	AK	16514078	
positive Regulation: RAC1 --+>	AK	27726098	10.1007/s13277-016-5374-6
positive Regulation: RAC1 --+>	AK	28805822	10.1038/nm.4379
positive Regulation: RAC1 --+>	AKT1		10.1042/BCJ20170279
positive Regulation: RAC1 --+>	AK	21984807	10.1242/jcs.092601
positive Regulation: RAC1 --+>	AK	21537609	10.1590/S0100-879X2011007500057
positive Regulation: RAC1 --+>	AK	21110188	10.1007/s00223-010-9435-3
positive Regulation: RAC1 --+>	AK	21110188	10.1007/s00223-010-9435-3
positive Regulation: RAC1 --+>	AK	17467682	10.1016/j.ydbio.2007.03.520
positive Regulation: RAC1 --+>	AK	15093747	10.1016/j.yexcr.2004.02.002
positive Regulation: RAC1 --+>	AK	20538003	10.1016/j.yjmcc.2010.05.007
positive Regulation: RAC1 --+>	AK	17188364	10.1016/j.brainresrev.2006.09.004
positive Regulation: RAC1 --+>	AK	15752980	10.1016/j.tcb.2005.01.002

positive Regulation: RAC1 --+> AK 12621032
positive Regulation: RAC1 --+> AK 11369774
positive Regulation: RAC1 --+> AK 11956329
positive Regulation: RAC1 --+> AK 20811053 10.1167/iovs.10-6140
positive Regulation: RAC1 --+> AK 22207728 10.1161/ATVBAHA.111.243675
positive Regulation: RAC1 --+> AK 22207728 10.1161/ATVBAHA.111.243675
positive Regulation: RAC1 --+> AK 22207728 10.1161/ATVBAHA.111.243675
positive Regulation: RAC1 --+> AK 22995516 10.1161/ATVBAHA.112.254920
positive Regulation: RAC1 --+> AK 22995516 10.1161/ATVBAHA.112.254920
positive Regulation: RAC1 --+> AK 23446149 10.1189/jlb.0912473
positive Regulation: RAC1 --+> AK 23640896 10.1074/jbc.M113.467647
positive Regulation: RAC1 --+> AK 23640896 10.1074/jbc.M113.467647
positive Regulation: RAC1 --+> AK 23640896 10.1074/jbc.M113.467647
positive Regulation: RAC1 --+> AK 23640896 10.1074/jbc.M113.467647
positive Regulation: RAC1 --+> AK 23640896 10.1074/jbc.M113.467647
positive Regulation: RAC1 --+> AK 23640896 10.1074/jbc.M113.467647
positive Regulation: RAC1 --+> AK 21339740 10.1038/onc.2011.22
positive Regulation: RAC1 --+> AK 21339740 10.1038/onc.2011.22
positive Regulation: RAC1 --+> AK 21455314 10.1371/journal.pone.0018002
positive Regulation: RAC1 --+> AK 20124168 10.1200/JCO.2009.25.8624
positive Regulation: RAC1 --+> AK 22723286 10.1099/mic.0.059972-0
positive Regulation: RAC1 --+> AK 22723286 10.1099/mic.0.059972-0
positive Regulation: RAC1 --+> AK 22723286 10.1099/mic.0.059972-0
positive Regulation: RAC1 --+> AK 22723286 10.1099/mic.0.059972-0
positive Regulation: RAC1 --+> AK 23898047 10.1189/jlb.0313174
positive Regulation: RAC1 --+> AK 23919665 10.1089/ars.2013.5526
positive Regulation: RAC1 --+> AK 22668020 10.4149/neo_2012_070
positive Regulation: RAC1 --+> AK 24604047
positive Regulation: RAC1 --+> AK 24667766 10.1371/journal.pone.0092398
positive Regulation: RAC1 --+> AK 24667766 10.1371/journal.pone.0092398
positive Regulation: RAC1 --+> AK 24667766 10.1371/journal.pone.0092398
positive Regulation: RAC1 --+> AK 24571616 10.1186/1742-4690-11-20
positive Regulation: RAC1 --+> AKT1 10.1172/JCI73579
positive Regulation: RAC1 --+> AK 25268615 10.3390/ijms151017411
positive Regulation: RAC1 --+> AK 23686855 10.1152/ajplung.00298.2012
positive Regulation: RAC1 --+> AK 23686855 10.1152/ajplung.00298.2012
positive Regulation: RAC1 --+> AKT1 10.1074/jbc.M114.633701
positive Regulation: RAC1 --+> AKT1 10.1074/jbc.M114.633701
positive Regulation: RAC1 --+> AKT1 10.1074/jbc.M114.633701
positive Regulation: RAC1 --+> AKT1 10.1074/jbc.M114.633701
positive Regulation: RAC1 --+> AKT1 10.1074/jbc.M114.633701
positive Regulation: RAC1 --+> AK 23817184 10.1159/000350108
positive Regulation: RAC1 --+> AK 23817184 10.1159/000350108
positive Regulation: RAC1 --+> AKT1
positive Regulation: RAC1 --+> AK 25968904 10.1038/ncomms8166
positive Regulation: RAC1 --+> AK 25968904 10.1038/ncomms8166
positive Regulation: RAC1 --+> AK 25968904 10.1038/ncomms8166
positive Regulation: RAC1 --+> AK 25968904 10.1038/ncomms8166
positive Regulation: RAC1 --+> AK 25968904 10.1038/ncomms8166
positive Regulation: RAC1 --+> AK 10891487
positive Regulation: RAC1 --+> AK 10891487
positive Regulation: RAC1 --+> AK 11803464
positive Regulation: RAC1 --+> AK 14685694
positive Regulation: RAC1 --+> AK 15187145

positive Regulation: RAC1 --+>	AK	15308574	
positive Regulation: RAC1 --+>	AK	15516977	
positive Regulation: RAC1 --+>	AK	16294277	
positive Regulation: RAC1 --+>	AK	16339142	
positive Regulation: RAC1 --+>	AK	17855349	
positive Regulation: RAC1 --+>	AK	18086571	
positive Regulation: RAC1 --+>	AK	19074832	10.1158/1541-7786.MCR-08-0084
positive Regulation: RAC1 --+>	AK	20175116	10.1002/jcp.22080
positive Regulation: RAC1 --+>	AK	20307615	10.1016/j.neuint.2010.03.005
positive Regulation: RAC1 --+>	AKT1		10.1091/mbc.E15-04-0257
positive Regulation: RAC1 --+>	AK	26687235	10.1007/s12035-015-9583-6
positive Regulation: RAC1 --+>	AK	26148238	10.1038/onc.2015.240
positive Regulation: RAC1 --+>	AKT1		
positive Regulation: RAC1 --+>	AK	29046393	10.1042/BCJ2017027910.1042/BCJ20170279
positive Regulation: RAC1 --+>	AK	29046393	10.1042/BCJ2017027910.1042/BCJ20170279
positive Regulation: RAC1 --+>	AK	29046393	10.1042/BCJ2017027910.1042/BCJ20170279
positive Regulation: RAC1 --+>	AK	29046393	10.1042/BCJ2017027910.1042/BCJ20170279
positive Regulation: RAC1 --+>	AKT1		10.1042/BCJ20170279
positive Regulation: RAC1 --+>	AKT1		10.1042/BCJ20170279
positive Regulation: RAC1 --+>	AKT1		10.1042/BCJ20170279
positive Regulation: RAC1 --+>	AKT1		10.1042/BCJ20170279
positive Regulation: RAC1 --+>	AKT1		10.1042/BCJ20170279
positive Regulation: RAC1 --+>	AKT1		10.1042/BCJ20170279
positive Regulation: RAC1 --+>	AKT1		10.1042/BCJ20170279
positive Regulation: RAC1 --+>	AKT1		10.1042/BCJ20170279
positive Regulation: RAC1 --+>	AKT1		10.1042/BCJ20170279
positive Regulation: RAC1 --+>	AKT1		10.1042/BCJ20170279
positive Regulation: RAC1 --+>	AK	29329780	10.1016/j.cellsig.2018.01.00410.1016/j.cellsi
positive Regulation: RAC1 --+>	AK	30013448	10.7150/ijms.25527
positive Regulation: RAC1 --+>	AKT1		10.1007/s12035-018-0881-710.1007/s12035-018-1
positive Regulation: RAC1 --+>	AK	30064133	10.1159/000492255
positive Regulation: RAC1 --+>	AKT1		10.3727/096504018X15166204902353
positive Regulation: RAC1 --+>	AK	29186971	10.1089/ars.2017.7441
positive Regulation: RAC1 --+>	AK	30067154	10.1152/physrev.00063.2017
positive Regulation: RAC1 --+>	AKT1		10.1007/978-3-030-30651-9_7
positive Regulation: RAC1 --+>	AKT1		10.1515/hsz-2019-0329
positive Regulation: RAC1 --+>	AK	32275841	10.1080/15384101.2020.1749790
positive Regulation: RAC1 --+>	AK	32275841	10.1080/15384101.2020.1749790
positive Regulation: RAC1 --+>	AK	32275841	10.1080/15384101.2020.1749790
positive Regulation: RAC1 --+>	AK	32275841	10.1080/15384101.2020.1749790
positive Regulation: RAC1 --+>	AK	32275841	10.1080/15384101.2020.1749790
positive Regulation: RAC1 --+>	AK	32275841	10.1080/15384101.2020.1749790
positive Regulation: RAC1 --+>	AK	32275841	10.1080/15384101.2020.1749790
positive Regulation: RAC1 --+>	AK	32275841	10.1080/15384101.2020.1749790
positive Regulation: RAC1 --+>	AK	32275841	10.1080/15384101.2020.1749790
positive Regulation: RAC1 --+>	AK	32275841	10.1080/15384101.2020.1749790
positive Regulation: RAC1 --+>	AK	32275841	10.1080/15384101.2020.1749790
positive Regulation: RAC1 --+>	AK	32275841	10.1080/15384101.2020.1749790
positive Regulation: RAC1 --+>	AK	32275841	10.1080/15384101.2020.1749790
positive Regulation: RAC1 --+>	AK	32275841	10.1080/15384101.2020.1749790
positive Regulation: RAC1 --+>	AK	32275841	10.1080/15384101.2020.1749790
positive Regulation: RAC1 --+>	AK	32275841	10.1080/15384101.2020.1749790
positive Regulation: RAC1 --+>	AK	32275841	10.1080/15384101.2020.1749790
positive Regulation: RAC1 --+>	AK	32275841	10.1080/15384101.2020.1749790

positive Regulation: RAC1 --+> AK	33081375	10.3390/ijms2120767910.3390/ijms21207679
positive Regulation: RAC1 --+> AK	33081375	10.3390/ijms21207679
positive Regulation: RAC1 --+> AK	32772212	10.1007/s10555-020-09923-5
positive Regulation: RAC1 --+> AK	33318149	10.1242/dev.184341
positive Regulation: RAC1 --+> AK	33318149	10.1242/dev.184341
positive Regulation: RAC1 --+> AK	33879799	10.1038/s41467-021-22531-3
positive Regulation: RAC1 --+> AK	34882832	10.1096/fj.202100554R
positive Regulation: RAC1 --+> AK	35462099	10.1016/j.bbrc.2022.03.15210.1016/j.bbrc.2022
positive Regulation: RAC1 --+> AK	35962329	10.1186/s10020-022-00511-7
positive Regulation: RAC1 --+> AK	35796938	10.1007/s13577-022-00733-1
positive Regulation: RAC1 --+> AK	35816228	10.1007/s13577-022-00745-x
positive Regulation: RAC1 --+> AK	35816228	10.1007/s13577-022-00745-x
positive Regulation: RAC1 --+> AK	36045410	10.1186/s12931-022-02153-z
positive Regulation: RAC1 --+> AKT1		10.7150/ijms.76664
positive Regulation: ADCY8 --+> M	18448650	
Binding: GPER1 ---- PTEN	23921077	10.1016/j.steroids.2013.07.007
negative Expression: GRK4 ---| BE	33280021	10.1093/eurheartj/ehaa878
negative Expression: UCA1 ---| NC	31301558	10.1016/j.intimp.2019.105734
positive Regulation: UPK1A --+> N	25978409	10.1371/journal.pgen.1005221
Regulation: PRND ---> AKT1	32895288	10.1242/dev.193094
Regulation: PRND ---> AKT1	26950422	10.1172/JCI83427
Regulation: PRND ---> AKT1	26950422	10.1172/JCI83427
Regulation: PRND ---> AKT1	26950422	10.1172/JCI83427
Expression: MAPK1 ---> NET1	21986943	10.1038/onc.2011.457
Expression: MAPK1 ---> NET1	21986943	10.1038/onc.2011.457
negative Regulation: CD274 ---| F	28231855	10.1186/s12967-017-1150-7
negative Regulation: CD274 ---| F	28231855	10.1186/s12967-017-1150-710.1186/s12967-017-1
positive Expression: SPP1 --+> AK	29079264	10.1016/j.yexcr.2017.10.018
positive Expression: SPP1 --+> AK	28935249	10.1016/j.lfs.2017.09.019
positive Expression: SPP1 --+> AK	31879711	10.1016/j.heliyon.2019.e03006
positive Expression: SPP1 --+> AK	24999034	10.1016/j.bbagrm.2014.06.020
positive Expression: SPP1 --+> AK	25732057	10.1016/j.bbcan.2015.02.003
positive Expression: SPP1 --+> AK	25732057	10.1016/j.bbcan.2015.02.003
positive Expression: SPP1 --+> AKT1		10.12659/MSM.905700
positive Expression: SPP1 --+> AKT1		10.12659/MSM.905700
positive Expression: SPP1 --+> AK	30627777	10.1007/s00280-018-3759-5
positive Expression: SPP1 --+> AK	30627777	10.1007/s00280-018-3759-5
positive Expression: SPP1 --+> AK	28032860	10.1038/cddis.2016.422
positive Expression: SPP1 --+> AK	28032860	10.1038/cddis.2016.422
positive Expression: SPP1 --+> AK	28032860	10.1038/cddis.2016.422
positive Expression: SPP1 --+> AK	28032860	10.1038/cddis.2016.422
positive Expression: SPP1 --+> AK	28032860	10.1038/cddis.2016.422
positive Expression: SPP1 --+> AK	28032860	10.1038/cddis.2016.422
positive Expression: SPP1 --+> AK	28032860	10.1038/cddis.2016.422
positive Expression: SPP1 --+> AK	28032860	10.1038/cddis.2016.422
positive Expression: SPP1 --+> AK	28032860	10.1038/cddis.2016.422
positive Expression: SPP1 --+> AK	28032860	10.1038/cddis.2016.422
positive Expression: SPP1 --+> AK	31295749	10.1055/a-0926-3991
positive Expression: SPP1 --+> AK	24714722	10.3892/ijo.2014.2370
positive Expression: SPP1 --+> AK	24714722	10.3892/ijo.2014.2370
positive Expression: SPP1 --+> AK	24961145	10.1186/1756-9966-33-53
positive Expression: SPP1 --+> AK	26099282	10.3892/mmr.2015.3975
positive Expression: SPP1 --+> AK	19597469	10.1038/onc.2009.18910.1038/onc.2009.189
positive Expression: SPP1 --+> AK	24999034	10.1016/j.bbagrm.2014.06.02010.1016/j.bbagrm.

positive Expression: SPP1 --+> AI	27063991	10.1016/j.lfs.2016.04.00310.1016/j.lfs.2016.0
positive Expression: SPP1 --+> AI	28875010	
positive Expression: SPP1 --+> AI	29550832	10.12659/msm.905700
positive Expression: SPP1 --+> AI	28505114	10.3390/ijms18051058
positive Expression: SPP1 --+> AKT1		10.1007/s00280-018-3759-5
positive Expression: SPP1 --+> AKT1		10.1007/s00280-018-3759-5
negative Expression: SNCAIP ---|	16495229	10.1074/jbc.M508619200
negative Expression: SNCAIP ---|	20807766	10.1074/jbc.M110.169581
negative Expression: SNCAIP ---|	16495229	
negative Expression: SNCAIP ---|	16495229	
negative Expression: SNCAIP ---|	20807766	10.1074/jbc.M110.169581
negative Expression: SNCAIP ---|	16495229	10.1074/jbc.M508619200
negative Expression: SNCAIP ---|	22832581	10.1038/nature11327
positive Expression: AKT1 --+> SF	16807238	10.1074/jbc.M602694200
positive Expression: AKT1 --+> SF	30107166	10.1016/j.lfs.2018.08.025
positive Expression: AKT1 --+> SF	27496181	10.1016/j.pbiomolbio.2016.08.003
positive Expression: AKT1 --+> SF	27794492	10.1016/j.jconrel.2016.10.024
positive Expression: AKT1 --+> SF	32858177	10.1016/j.gene.2020.145059
positive Expression: AKT1 --+> SF	19356721	10.1016/j.ejphar.2009.03.063
positive Expression: AKT1 --+> SF	19356721	10.1016/j.ejphar.2009.03.063
positive Expression: AKT1 --+> SF	19767100	10.1016/j.leukres.2009.08.028
positive Expression: AKT1 --+> SF	21056654	10.1016/j.cellsig.2010.10.021
positive Expression: AKT1 --+> SF	21056654	10.1016/j.cellsig.2010.10.021
positive Expression: AKT1 --+> SF	18952487	10.1016/j.cytogfr.2008.08.001
positive Expression: AKT1 --+> SF	17161983	10.1016/j.dld.2006.10.015
positive Expression: AKT1 --+> SF	22947282	10.1016/j.acthis.2012.08.004
positive Expression: AKT1 --+> SF	24530412	10.1016/j.intimp.2014.01.028
positive Expression: AKT1 --+> SF	25986148	10.1016/j.yjmcc.2015.05.006
positive Expression: AKT1 --+> SF	25986148	10.1016/j.yjmcc.2015.05.006
positive Expression: AKT1 --+> SF	16946008	
positive Expression: AKT1 --+> SF	16807238	
positive Expression: AKT1 --+> SF	15347645	
positive Expression: AKT1 --+> SF	15854023	
positive Expression: AKT1 --+> SF	12944477	
positive Expression: AKT1 --+> SF	12944477	
positive Expression: AKT1 --+> SF	12944477	
positive Expression: AKT1 --+> SF	12944477	
positive Expression: AKT1 --+> SF	12944477	
positive Expression: AKT1 --+> SF	12944477	
positive Expression: AKT1 --+> SF	12944477	
positive Expression: AKT1 --+> SF	28715969	10.1089/ars.2017.7275
positive Expression: AKT1 --+> SF	29620276	10.3892/mmr.2018.8845
positive Expression: AKT1 --+> SF	22965338	10.3892/mmr.2012.1074
positive Expression: AKT1 --+> SF	22965338	10.3892/mmr.2012.1074
positive Expression: AKT1 --+> SF	21406114	10.1186/1471-2121-12-11
positive Expression: AKT1 --+> SF	21406114	10.1186/1471-2121-12-11
positive Expression: AKT1 --+> SF	21406114	10.1186/1471-2121-12-11
positive Expression: AKT1 --+> SF	21406114	10.1186/1471-2121-12-11
positive Expression: AKT1 --+> SF	21406114	10.1186/1471-2121-12-11
positive Expression: AKT1 --+> SF	22675553	10.1371/journal.pone.0038378
positive Expression: AKT1 --+> SF	22675553	10.1371/journal.pone.0038378
positive Expression: AKT1 --+> SF	22675553	10.1371/journal.pone.0038378
positive Expression: AKT1 --+> SF	22675553	10.1371/journal.pone.0038378
positive Expression: AKT1 --+> SF	22675553	10.1371/journal.pone.0038378

positive Expression: AKT1 --+> SF	22675553	10.1371/journal.pone.0038378
positive Expression: AKT1 --+> SF	25884410	10.1371/journal.pone.0123318
positive Expression: AKT1 --+> SF	26280346	10.1371/journal.pone.0134243
positive Expression: AKT1 --+> SF	12944477	10.1128/mcb.23.18.6507-6519.2003
positive Expression: AKT1 --+> SF	12944477	10.1128/mcb.23.18.6507-6519.2003
positive Expression: AKT1 --+> SF	15705860	
positive Expression: AKT1 --+> SF	15705860	
positive Expression: AKT1 --+> SF	16614705	
positive Expression: AKT1 --+> SF	21406114	10.1186/1471-2121-12-1110.1186/1471-2121-12-1
positive Expression: AKT1 --+> SF	24530412	10.1016/j.intimp.2014.01.02810.1016/j.intimp.
positive Expression: AKT1 --+> SF	34234236	10.1038/s41598-021-93484-210.1038/s41598-021-
positive Expression: AKT1 --+> SF	17287359	
positive Expression: AKT1 --+> SF	21337710	10.1002/ar.2132410.1002/ar.21324
Regulation: PTGS2 ---> NGF	19129380	
Regulation: PTGS2 ---> NGF		10.1097/BRS.0000000000001379
Regulation: PTGS2 ---> NGF	16879691	10.1111/j.1464-410X.2006.06207.x
Regulation: PTGS2 ---> NGF	23799042	10.1371/journal.pone.006575110.1371/journal.p
Regulation: PTGS2 ---> NGF	26876621	10.1016/j.jos.2016.01.00410.1016/j.jos.2016.0
Regulation: PTGS2 ---> NGF	28759126	10.1111/sms.1295410.1111/sms.12954
ProtModification: AKT1 ---> RAC1	11733515	10.1074/jbc.M108233200
ProtModification: AKT1 ---> RAC1	14960575	10.1074/jbc.M400905200
ProtModification: AKT1 ---> RAC1	31669133	10.1016/j.semcdb.2019.10.007
ProtModification: AKT1 ---> RAC1	16018989	10.1016/j.brainres.2005.05.043
ProtModification: AKT1 ---> RAC1	17399794	10.1016/j.pharmthera.2007.02.002
ProtModification: AKT1 ---> RAC1	18191273	10.1016/j.jhep.2007.11.018
ProtModification: AKT1 ---> RAC1	18255190	10.1016/j.neurobiolaging.2007.12.015
ProtModification: AKT1 ---> RAC1	15013523	10.1016/j.canlet.2003.08.035
ProtModification: AKT1 ---> RAC1	16973299	10.1016/j.neuroscience.2006.07.049
ProtModification: AKT1 ---> RAC1	16973299	10.1016/j.neuroscience.2006.07.049
ProtModification: AKT1 ---> RAC1	16973299	10.1016/j.neuroscience.2006.07.049
ProtModification: AKT1 ---> RAC1	22572609	10.1016/j.tcb.2012.04.004
ProtModification: AKT1 ---> RAC1	16839863	10.1016/j.tcm.2006.03.010
ProtModification: AKT1 ---> RAC1	14523036	
ProtModification: AKT1 ---> RAC1	16195373	10.1096/fj.05-3928com
ProtModification: AKT1 ---> RAC1	14960575	10.1074/jbc.M400905200
ProtModification: AKT1 ---> RAC1	10617634	
ProtModification: AKT1 ---> RAC1	10617634	
ProtModification: AKT1 ---> RAC1	10617634	
ProtModification: AKT1 ---> RAC1	10617634	
ProtModification: AKT1 ---> RAC1	10617634	
ProtModification: AKT1 ---> RAC1	10617634	
ProtModification: AKT1 ---> RAC1	10617634	
ProtModification: AKT1 ---> RAC1	10617634	
ProtModification: AKT1 ---> RAC1	10617634	
ProtModification: AKT1 ---> RAC1	10617634	
ProtModification: AKT1 ---> RAC1	10617634	
ProtModification: AKT1 ---> RAC1	10617634	
ProtModification: AKT1 ---> RAC1	10617634	
ProtModification: AKT1 ---> RAC1	10617634	
ProtModification: AKT1 ---> RAC1	10617634	
ProtModification: AKT1 ---> RAC1	10617634	
ProtModification: AKT1 ---> RAC1	10617634	
ProtModification: AKT1 ---> RAC1	11686294	
ProtModification: AKT1 ---> RAC1	12728249	

ProtModification: AKT1 ---> RAC1 19889994 10.1523/JNEUROSCI.3574-09.2009
ProtModification: AKT1 ---> RAC1 27600824 10.1152/ajpendo.00189.2016
ProtModification: AKT1 ---> RAC1 29036929 10.3390/ijms18102148
ProtModification: AKT1 ---> RAC1 29036929 10.3390/ijms18102148
ProtModification: AKT1 ---> RAC1 31248165 10.3390/ijms20122997
ProtModification: AKT1 ---> RAC1 23512198 10.1096/fj.12-223099
ProtModification: AKT1 ---> RAC1 23512198 10.1096/fj.12-223099
ProtModification: AKT1 ---> RAC1 23512198 10.1096/fj.12-223099
ProtModification: AKT1 ---> RAC1 23512198 10.1096/fj.12-223099
ProtModification: AKT1 ---> RAC1 23512198 10.1096/fj.12-223099
ProtModification: AKT1 ---> RAC1 24043306 10.1128/MCB.00822-13
ProtModification: AKT1 ---> RAC1 24043306 10.1128/MCB.00822-13
ProtModification: AKT1 ---> RAC1 26816343 10.1371/journal.pone.0147103
ProtModification: AKT1 ---> RAC1 10617634 10.1074/jbc.275.1.423
ProtModification: AKT1 ---> RAC1 10617634 10.1074/jbc.275.1.423
ProtModification: AKT1 ---> RAC1 23512198 10.1096/fj.12-223099
ProtModification: AKT1 ---> RAC1 10617634 10.1074/jbc.275.1.423
ProtModification: AKT1 ---> RAC1 24043306 10.1128/MCB.00822-13
ProtModification: AKT1 ---> RAC1 29036929 10.3390/ijms18102148
ProtModification: AKT1 ---> RAC1 16839863 10.1016/j.tcm.2006.03.010
ProtModification: AKT1 ---> RAC1 14960575 10.1074/jbc.M400905200
ProtModification: AKT1 ---> RAC1 18255190 10.1016/j.neurobiolaging.2007.12.015
ProtModification: AKT1 ---> RAC1 16973299 10.1016/j.neuroscience.2006.07.049
ProtModification: AKT1 ---> RAC1 22572609 10.1016/j.tcb.2012.04.004
ProtModification: AKT1 ---> RAC1 15013523 10.1016/j.canlet.2003.08.035
ProtModification: AKT1 ---> RAC1 26816343 10.1371/journal.pone.0147103
ProtModification: AKT1 ---> RAC1 18191273 10.1016/j.jhep.2007.11.018
ProtModification: AKT1 ---> RAC1 17399794 10.1016/j.pharmthera.2007.02.002
ProtModification: AKT1 ---> RAC1 27600824 10.1152/ajpendo.00189.2016
ProtModification: AKT1 ---> RAC1 16018989 10.1016/j.brainres.2005.05.043
ProtModification: AKT1 ---> RAC1 19889994 10.1523/JNEUROSCI.3574-09.2009
ProtModification: AKT1 ---> RAC1 16195373 10.1096/fj.05-3928com
ProtModification: AKT1 ---> RAC1 12728249 10.1038/sj.cdd.4401172
ProtModification: AKT1 ---> RAC1 22970203 10.1371/journal.pone.0044358
ProtModification: AKT1 ---> RAC1 11134000 10.1074/jbc.M004617200
ProtModification: AKT1 ---> RAC1 100427475 10.1016/j.tcb.2012.04.004
ProtModification: AKT1 ---> RAC1 10.1016/j.semcdb.2019.10.007
ProtModification: AKT1 ---> RAC1 100427475 10.1016/j.tcb.2012.04.004
ProtModification: AKT1 ---> RAC1 10.3390/ijms20122997
ProtModification: AKT1 ---> RAC1
ProtModification: AKT1 ---> RAC1 12728249 10.1038/sj.cdd.4401172
ProtModification: AKT1 ---> RAC1 22970203 10.1371/journal.pone.0044358
ProtModification: AKT1 ---> RAC1 11134000 10.1074/jbc.M004617200
ProtModification: AKT1 ---> RAC1 18255190 10.1016/j.neurobiolaging.2007.12.015
ProtModification: AKT1 ---> RAC1 10617634
ProtModification: AKT1 ---> RAC1 12728249
ProtModification: AKT1 ---> RAC1 23512198 10.1096/fj.12-223099
Expression: MAPK1 ---> PPARG 26940012 10.1016/j.yexcr.2016.02.019
Expression: MAPK1 ---> PPARG 30573683 10.1074/jbc.RA118.004411
Expression: MAPK1 ---> PPARG 30573683 10.1074/jbc.RA118.004411
Expression: MAPK1 ---> PPARG 30573683 10.1074/jbc.RA118.004411
Expression: MAPK1 ---> PPARG 24550383 10.1074/jbc.M113.546382
Expression: MAPK1 ---> PPARG 16574647 10.1074/jbc.M602623200
Expression: MAPK1 ---> PPARG 29656183 10.1016/j.biopha.2018.04.014

Expression: MAPK1 ---> PPARG	26872696	10.1016/j.immuni.2016.01.002
Expression: MAPK1 ---> PPARG		10.1016/j.chmed.2018.01.009
Expression: MAPK1 ---> PPARG	29198236	10.1017/S1751731117003275
Expression: MAPK1 ---> PPARG	28580280	10.1016/j.molmet.2017.03.003
Expression: MAPK1 ---> PPARG	16945329	10.1016/j.bbrc.2006.08.085
Expression: MAPK1 ---> PPARG	17173860	10.1016/j.bbrc.2006.11.137
Expression: MAPK1 ---> PPARG	17331472	10.1016/j.bbrc.2007.02.057
Expression: MAPK1 ---> PPARG	17543283	10.1016/j.bbrc.2007.05.070
Expression: MAPK1 ---> PPARG	18983975	10.1016/j.bbrc.2008.10.131
Expression: MAPK1 ---> PPARG	21439945	10.1016/j.bcp.2011.03.012
Expression: MAPK1 ---> PPARG	18037376	10.1016/j.febslet.2007.11.051
Expression: MAPK1 ---> PPARG	19385085	10.1016/j.lfs.2009.01.018
Expression: MAPK1 ---> PPARG	19385085	10.1016/j.lfs.2009.01.018
Expression: MAPK1 ---> PPARG	20346961	10.1016/j.lfs.2010.03.010
Expression: MAPK1 ---> PPARG	20553736	10.1016/j.lfs.2010.05.007
Expression: MAPK1 ---> PPARG	20553736	10.1016/j.lfs.2010.05.007
Expression: MAPK1 ---> PPARG	21315734	10.1016/j.lfs.2011.02.001
Expression: MAPK1 ---> PPARG		10.1016/S0041-008X(03)00083-8
Expression: MAPK1 ---> PPARG		10.1016/S0041-008X(03)00083-8
Expression: MAPK1 ---> PPARG		10.1016/S0041-008X(03)00083-8
Expression: MAPK1 ---> PPARG	22137902	10.1016/j.fct.2011.11.020
Expression: MAPK1 ---> PPARG	18790715	10.1016/j.mce.2008.08.018
Expression: MAPK1 ---> PPARG		10.1016/j.foodchem.2010.08.052
Expression: MAPK1 ---> PPARG	21333731	10.1016/j.tiv.2011.02.004
Expression: MAPK1 ---> PPARG	21664967	10.1016/j.cellsig.2011.05.018
Expression: MAPK1 ---> PPARG	21447440	10.1016/j.jnutbio.2010.11.016
Expression: MAPK1 ---> PPARG	23058985	10.1016/j.bcp.2012.09.033
Expression: MAPK1 ---> PPARG	23058985	10.1016/j.bcp.2012.09.033
Expression: MAPK1 ---> PPARG	22981159	10.1016/j.npep.2012.08.006
Expression: MAPK1 ---> PPARG	23684777	10.1016/j.freeradbiomed.2013.05.013
Expression: MAPK1 ---> PPARG	25562427	10.1016/j.cellsig.2014.12.018
Expression: MAPK1 ---> PPARG	26796578	10.1016/j.lfs.2016.01.024
Expression: MAPK1 ---> PPARG	26631505	10.1016/j.lfs.2015.11.028
Expression: MAPK1 ---> PPARG	18703634	
Expression: MAPK1 ---> PPARG	17312274	
Expression: MAPK1 ---> PPARG	16574647	
Expression: MAPK1 ---> PPARG	15067015	
Expression: MAPK1 ---> PPARG	18320708	
Expression: MAPK1 ---> PPARG	15252128	
Expression: MAPK1 ---> PPARG	12457461	
Expression: MAPK1 ---> PPARG	17210639	
Expression: MAPK1 ---> PPARG	15657420	
Expression: MAPK1 ---> PPARG	15486348	
Expression: MAPK1 ---> PPARG	27669202	10.3390/molecules21101278
Expression: MAPK1 ---> PPARG	28840539	10.1007/s12272-017-0948-4
Expression: MAPK1 ---> PPARG	28922143	10.3233/CH-170246
Expression: MAPK1 ---> PPARG	29283422	10.3390/ijms19010090
Expression: MAPK1 ---> PPARG	29283422	10.3390/ijms19010090
Expression: MAPK1 ---> PPARG	29283422	10.3390/ijms19010090
Expression: MAPK1 ---> PPARG	29589999	10.1089/thy.2017.0338
Expression: MAPK1 ---> PPARG	29990990	10.1159/000491619
Expression: MAPK1 ---> PPARG	30249030	10.3390/molecules23102433
Expression: MAPK1 ---> PPARG	30244245	10.1159/000492415
Expression: MAPK1 ---> PPARG	30431093	10.3892/mmr.2018.9627

Expression: MAPK1 ---> PPARG	30573683	10.1074/jbc.RA118.004411
Expression: MAPK1 ---> PPARG	30573683	10.1074/jbc.RA118.004411
Expression: MAPK1 ---> PPARG	30573683	10.1074/jbc.RA118.004411
Expression: MAPK1 ---> PPARG	30573683	10.1074/jbc.RA118.004411
Expression: MAPK1 ---> PPARG	30647194	10.21775/cimb.031.001
Expression: MAPK1 ---> PPARG	31292134	10.2337/db18-1055
Expression: MAPK1 ---> PPARG	31292134	10.2337/db18-1055
Expression: MAPK1 ---> PPARG	31292134	10.2337/db18-1055
Expression: MAPK1 ---> PPARG	34634123	10.1093/jas/skab287
Expression: MAPK1 ---> PPARG	19191008	10.1007/s11010-009-0027-310.1007/s11010-009-0
Expression: MAPK1 ---> PPARG	19191008	10.1007/s11010-009-0027-310.1007/s11010-009-0
Expression: MAPK1 ---> PPARG	19243475	10.1111/j.1582-4934.2009.00709.x10.1111/j.158
Expression: MAPK1 ---> PPARG	19243475	10.1111/j.1582-4934.2009.00709.x10.1111/j.158
Expression: MAPK1 ---> PPARG	20339310	10.3275/694810.1007/BF03346688
Expression: MAPK1 ---> PPARG	21669876	10.1074/jbc.M111.24843510.1074/jbc.M111.24843
Expression: MAPK1 ---> PPARG	22805337	10.1111/j.1582-4934.2012.01602.x10.1111/j.158
Expression: MAPK1 ---> PPARG		10.1016/j.fct.2011.11.020
Expression: MAPK1 ---> PPARG		10.3233/CH-170246
Expression: MAPK1 ---> PPARG		10.1074/jbc.RA118.004411
Expression: MAPK1 ---> PPARG		10.1074/jbc.RA118.004411
Expression: MAPK1 ---> PPARG		10.1074/jbc.RA118.004411
Expression: MAPK1 ---> PPARG		10.1074/jbc.RA118.004411
Expression: MAPK1 ---> PPARG		10.21775/cimb.031.001
Expression: MAPK1 ---> PPARG		10.2337/db18-1055
Expression: MAPK1 ---> PPARG		10.2337/db18-1055
Expression: MAPK1 ---> PPARG		10.2337/db18-1055
Expression: MAPK1 ---> PPARG		10.1071/RD19108
Expression: MAPK1 ---> PPARG	22675336	10.1155/2012/541204
Expression: MAPK1 ---> PPARG	20643830	10.1084/jem.20091022
Expression: MAPK1 ---> PPARG	21155050	10.3349/ymj.2011.52.1.165
Expression: MAPK1 ---> PPARG	21155050	10.3349/ymj.2011.52.1.165
Expression: MAPK1 ---> PPARG	21669876	10.1074/jbc.M111.248435
Expression: MAPK1 ---> PPARG	21701568	10.1038/oby.2011.176
Expression: MAPK1 ---> PPARG	23746272	10.2174/09298673113209990136
Expression: MAPK1 ---> PPARG	22479600	10.1371/journal.pone.0034315
Expression: MAPK1 ---> PPARG		10.1074/jbc.M113.546382
Expression: MAPK1 ---> PPARG	24626526	10.3892/mmr.2014.2041
Expression: MAPK1 ---> PPARG	24959884	10.1371/journal.pone.0100862
Expression: MAPK1 ---> PPARG		10.1194/jlr.M049551
Expression: MAPK1 ---> PPARG		10.1194/jlr.M050658
Expression: MAPK1 ---> PPARG	21368268	10.1152/ajpregu.00806.2010
Expression: MAPK1 ---> PPARG	21737780	10.1152/ajpgi.00508.2010
Expression: MAPK1 ---> PPARG	18635913	10.3177/jnsv.54.250
Expression: MAPK1 ---> PPARG	15733737	10.1016/j.biochi.2004.10.018
Expression: MAPK1 ---> PPARG	23780847	10.1152/physiolgenomics.00009.2013
Expression: MAPK1 ---> PPARG	25796354	10.12659/MSM.893144
Expression: MAPK1 ---> PPARG	12270934	10.1074/jbc.M207776200
Expression: MAPK1 ---> PPARG	15193259	10.1016/j.ccr.2004.05.014
Expression: MAPK1 ---> PPARG	15252128	10.1242/jcs.01207
Expression: MAPK1 ---> PPARG	18783396	10.1111/j.1530-0277.2008.00781.x10.1111/j.153
Expression: MAPK1 ---> PPARG	18783396	10.1111/j.1530-0277.2008.00781.x10.1111/j.153
Expression: MAPK1 ---> PPARG	21352814	10.1016/j.bbrc.2011.02.095
Expression: MAPK1 ---> PPARG	21352814	10.1016/j.bbrc.2011.02.095
Expression: MAPK1 ---> PPARG	20346961	10.1016/j.lfs.2010.03.010

Expression: MAPK1 ---> PPARG	18485432	10.1016/j.taap.2008.03.016
Expression: MAPK1 ---> PPARG	18790715	10.1016/j.mce.2008.08.018
Expression: MAPK1 ---> PPARG	18790715	10.1016/j.mce.2008.08.018
Expression: MAPK1 ---> PPARG	18790715	10.1016/j.mce.2008.08.018
Expression: MAPK1 ---> PPARG		10.1016/j.mce.2010.03.005
Expression: MAPK1 ---> PPARG		10.1016/j.mce.2010.03.005
Expression: MAPK1 ---> PPARG		10.1016/j.mce.2010.03.005
Expression: MAPK1 ---> PPARG		10.1016/j.mce.2010.03.005
Expression: MAPK1 ---> PPARG		10.1016/j.mce.2010.03.005
Expression: MAPK1 ---> PPARG	21839191	10.1016/j.bone.2011.07.038
Expression: MAPK1 ---> PPARG	23623642	10.1016/j.atherosclerosis.2013.03.023
Expression: MAPK1 ---> PPARG	23684777	10.1016/j.freeradbiomed.2013.05.013
Expression: MAPK1 ---> PPARG	23684777	10.1016/j.freeradbiomed.2013.05.013
Expression: MAPK1 ---> PPARG	23684777	10.1016/j.freeradbiomed.2013.05.013
Expression: MAPK1 ---> PPARG	24412557	10.1016/j.fct.2014.01.007
Expression: MAPK1 ---> PPARG	24355793	10.1016/j.jnutbio.2013.09.009
Expression: MAPK1 ---> PPARG	25557278	10.1016/j.freeradbiomed.2014.12.019
Expression: MAPK1 ---> PPARG	16766531	
Expression: MAPK1 ---> PPARG	16766531	
Expression: MAPK1 ---> PPARG	20553736	10.1016/j.lfs.2010.05.007
Expression: MAPK1 ---> PPARG		10.1016/j.fct.2011.11.020
Expression: MAPK1 ---> PPARG		10.1016/j.fct.2011.11.020
Expression: MAPK1 ---> PPARG	22137902	10.1016/j.fct.2011.11.020
Expression: MAPK1 ---> PPARG	22137902	10.1016/j.fct.2011.11.020
Expression: MAPK1 ---> PPARG	25283330	10.1016/j.jnutbio.2014.07.007
Expression: MAPK1 ---> PPARG	19221061	
Expression: MAPK1 ---> PPARG	21155050	10.3349/ymj.2011.52.1.165
Expression: MAPK1 ---> PPARG	24735204	10.1089/dna.2013.2290
Expression: MAPK1 ---> PPARG	20226837	10.1016/j.mce.2010.03.005
Expression: MAPK1 ---> PPARG	20553736	10.1016/j.lfs.2010.05.007
Expression: MAPK1 ---> PPARG	21155050	10.3349/ymj.2011.52.1.165
Expression: MAPK1 ---> PPARG	21664967	10.1016/j.cellsig.2011.05.018
Expression: MAPK1 ---> PPARG	23684777	10.1016/j.freeradbiomed.2013.05.013
Expression: MAPK1 ---> PPARG	25937083	10.1016/j.mvr.2015.04.008
Expression: MAPK1 ---> PPARG		
Expression: MAPK1 ---> PPARG	26675800	
Expression: MAPK1 ---> PPARG	28838346	10.14715/cmb/2017.63.7.15
Expression: MAPK1 ---> PPARG	29099637	10.1089/dna.2017.3706
Expression: MAPK1 ---> PPARG	29299041	10.1186/s12986-017-0234-y
Expression: MAPK1 ---> PPARG	29299041	10.1186/s12986-017-0234-y
Expression: MAPK1 ---> PPARG		10.1091/mbc.E17-03-0141
Expression: MAPK1 ---> PPARG		10.1091/mbc.E17-03-0141
Expression: MAPK1 ---> PPARG	29990990	10.1159/000491619
Expression: MAPK1 ---> PPARG	29383561	10.1007/s11010-018-3299-7
Expression: MAPK1 ---> PPARG	29383561	10.1007/s11010-018-3299-7
Expression: MAPK1 ---> PPARG	30639475	10.1016/j.molimm.2019.01.00110.1016/j.molimm.
Expression: MAPK1 ---> PPARG	30641938	10.3390/ijms2002027410.3390/ijms20020274
Expression: MAPK1 ---> PPARG	33370564	10.1016/j.bbagen.2020.12983610.1016/j.bbagen.
Expression: MAPK1 ---> PPARG	33468065	10.1186/s12864-020-07361-9
Expression: MAPK1 ---> PPARG	33991355	10.1111/pim.1286810.1111/pim.12868
Expression: MAPK1 ---> PPARG	34948240	10.3390/ijms222413446
Expression: MAPK1 ---> PPARG	35109789	10.1186/s11658-022-00314-y
Expression: MAPK1 ---> PPARG	35859055	10.1007/s00018-022-04475-z
ProtModification: MAPK1 ---> PPAF	27871858	10.1016/j.bbrc.2016.11.102

ProtModification: MAPK1 ---> PPAF 21525007 10.1074/jbc.M111.236398
ProtModification: MAPK1 ---> PPAF 15632188 10.1074/jbc.M409447200
ProtModification: MAPK1 ---> PPAF 26105050 10.1074/jbc.M115.638924
ProtModification: MAPK1 ---> PPAF 28424263 10.1074/jbc.M116.771428
ProtModification: MAPK1 ---> PPAF 24368768 10.1074/jbc.M113.536805
ProtModification: MAPK1 ---> PPAF 16574647 10.1074/jbc.M602623200
ProtModification: MAPK1 ---> PPAF 12837769 10.1074/jbc.M210747200
ProtModification: MAPK1 ---> PPAF 15067015 10.1074/jbc.M401766200
ProtModification: MAPK1 ---> PPAF 12475986 10.1074/jbc.M208394200
ProtModification: MAPK1 ---> PPAF 12475986 10.1074/jbc.M208394200
ProtModification: MAPK1 ---> PPAF 27475846 10.1016/j.mce.2016.07.030
ProtModification: MAPK1 ---> PPAF 27475846 10.1016/j.mce.2016.07.030
ProtModification: MAPK1 ---> PPAF 27984083 10.1016/j.mce.2016.10.028
ProtModification: MAPK1 ---> PPAF 27984083 10.1016/j.mce.2016.10.028
ProtModification: MAPK1 ---> PPAF 27352979 10.1016/j.freeradbiomed.2016.06.023
ProtModification: MAPK1 ---> PPAF 31654717 10.1016/j.cellsig.2019.109429
ProtModification: MAPK1 ---> PPAF 32088371 10.1016/j.cellsig.2020.109575
ProtModification: MAPK1 ---> PPAF 33359696 10.1016/j.bbadis.2020.166050
ProtModification: MAPK1 ---> PPAF 33359696 10.1016/j.bbadis.2020.166050
ProtModification: MAPK1 ---> PPAF 27268145 10.1016/j.phrs.2016.02.028
ProtModification: MAPK1 ---> PPAF 27268145 10.1016/j.phrs.2016.02.028
ProtModification: MAPK1 ---> PPAF 30745182 10.1016/j.cmet.2019.01.005
ProtModification: MAPK1 ---> PPAF 31708444 10.1016/j.cmet.2019.10.003
ProtModification: MAPK1 ---> PPAF 28580280 10.1016/j.molmet.2017.03.003
ProtModification: MAPK1 ---> PPAF 12110438 10.1016/S8756-3282(02)00807-4
ProtModification: MAPK1 ---> PPAF 17331472 10.1016/j.bbrc.2007.02.057
ProtModification: MAPK1 ---> PPAF 21486568 10.1016/j.febslet.2011.03.066
ProtModification: MAPK1 ---> PPAF 15993441 10.1016/j.neuropharm.2005.03.009
ProtModification: MAPK1 ---> PPAF 12729718 10.1016/S0041-008X(03)00015-2
ProtModification: MAPK1 ---> PPARG 10.1016/S0041-008X(03)00083-8
ProtModification: MAPK1 ---> PPAF 16338464 10.1016/j.placenta.2005.01.012
ProtModification: MAPK1 ---> PPAF 21354647 10.1016/j.lungcan.2011.01.019
ProtModification: MAPK1 ---> PPAF 22137902 10.1016/j.fct.2011.11.020
ProtModification: MAPK1 ---> PPAF 15733737 10.1016/j.biochi.2004.10.018
ProtModification: MAPK1 ---> PPAF 15733734 10.1016/j.biochi.2004.11.010
ProtModification: MAPK1 ---> PPAF 21645960 10.1016/j.diff.2011.05.008
ProtModification: MAPK1 ---> PPAF 14597384 10.1016/S0378-1119(03)00816-3
ProtModification: MAPK1 ---> PPAF 18423978 10.1016/j.cellsig.2008.02.022
ProtModification: MAPK1 ---> PPAF 18571911 10.1016/j.etp.2008.04.009
ProtModification: MAPK1 ---> PPAF 15780600 10.1016/j.ceb.2005.02.002
ProtModification: MAPK1 ---> PPAF 12943712 10.1016/S0960-0760(03)00214-0
ProtModification: MAPK1 ---> PPAF 17560826 10.1016/j.bbalip.2007.04.018
ProtModification: MAPK1 ---> PPAF 17560826 10.1016/j.bbalip.2007.04.018
ProtModification: MAPK1 ---> PPAF 23058985 10.1016/j.bcp.2012.09.033
ProtModification: MAPK1 ---> PPAF 23058985 10.1016/j.bcp.2012.09.033
ProtModification: MAPK1 ---> PPAF 22901559 10.1016/j.nutres.2012.06.001
ProtModification: MAPK1 ---> PPAF 24036109 10.1016/j.ejphar.2013.08.035
ProtModification: MAPK1 ---> PPAF 24036109 10.1016/j.ejphar.2013.08.035
ProtModification: MAPK1 ---> PPAF 24845100 10.1016/j.abb.2014.05.008
ProtModification: MAPK1 ---> PPAF 24915420 10.1016/j.bcp.2014.06.002
ProtModification: MAPK1 ---> PPAF 25813581 10.1016/j.cellsig.2015.03.010
ProtModification: MAPK1 ---> PPAF 25813581 10.1016/j.cellsig.2015.03.010
ProtModification: MAPK1 ---> PPAF 26830970 10.1016/j.ceca.2015.12.006
ProtModification: MAPK1 ---> PPAF 26556104 10.1016/j.cyto.2015.11.001

ProtModification: MAPK1 ---> PPAF	9886850	
ProtModification: MAPK1 ---> PPAF	9886850	
ProtModification: MAPK1 ---> PPAF	9886850	
ProtModification: MAPK1 ---> PPAF	9886850	
ProtModification: MAPK1 ---> PPAF	9886850	
ProtModification: MAPK1 ---> PPAF	16574647	
ProtModification: MAPK1 ---> PPAF	15632188	
ProtModification: MAPK1 ---> PPAF	15067015	
ProtModification: MAPK1 ---> PPAF	12837769	
ProtModification: MAPK1 ---> PPAF	12475986	
ProtModification: MAPK1 ---> PPAF	12475986	
ProtModification: MAPK1 ---> PPAF	12356758	
ProtModification: MAPK1 ---> PPAF	12356758	
ProtModification: MAPK1 ---> PPAF	9030579	
ProtModification: MAPK1 ---> PPAF	8943212	
ProtModification: MAPK1 ---> PPAF	15677498	
ProtModification: MAPK1 ---> PPAF	15054105	
ProtModification: MAPK1 ---> PPAF	18287349	
ProtModification: MAPK1 ---> PPAF	10064336	
ProtModification: MAPK1 ---> PPAF	17210639	
ProtModification: MAPK1 ---> PPAF	34339734	10.1016/j.jbc.2021.101030
ProtModification: MAPK1 ---> PPAF	18287349	10.1093/jn/138.3.455
ProtModification: MAPK1 ---> PPARG		10.1016/j.fct.2011.11.020
ProtModification: MAPK1 ---> PPARG		10.1016/j.ceca.2015.12.006
ProtModification: MAPK1 ---> PPAF	15486348	
ProtModification: MAPK1 ---> PPAF	15486348	
ProtModification: MAPK1 ---> PPAF	15486348	
ProtModification: MAPK1 ---> PPAF	14656714	
ProtModification: MAPK1 ---> PPAF	14656714	
ProtModification: MAPK1 ---> PPAF	14656714	
ProtModification: MAPK1 ---> PPARG		10.1530/JME-15-0122
ProtModification: MAPK1 ---> PPARG		10.1074/jbc.M116.771428
ProtModification: MAPK1 ---> PPAF	29141245	10.1159/000485008
ProtModification: MAPK1 ---> PPAF	29990990	10.1159/000491619
ProtModification: MAPK1 ---> PPAF	29383561	10.1007/s11010-018-3299-7
ProtModification: MAPK1 ---> PPAF	30287730	10.3390/ijms19103022
ProtModification: MAPK1 ---> PPARG		10.1074/jbc.RA118.004411
ProtModification: MAPK1 ---> PPARG		10.15252/embr.201947928
ProtModification: MAPK1 ---> PPAF	20590410	10.1089/scd.2010.0071
ProtModification: MAPK1 ---> PPAF	20590410	10.1089/scd.2010.0071
ProtModification: MAPK1 ---> PPAF	21525007	10.1074/jbc.M111.236398
ProtModification: MAPK1 ---> PPAF	22448169	10.1155/2012/929052
ProtModification: MAPK1 ---> PPAF	22481913	10.1155/2012/641087
ProtModification: MAPK1 ---> PPAF	20508203	10.1161/ATVBAHA.109.202309
ProtModification: MAPK1 ---> PPAF	23549795	10.3349/ymj.2013.54.3.545
ProtModification: MAPK1 ---> PPAF	22292086	10.1371/journal.pone.0030983
ProtModification: MAPK1 ---> PPAF	23175826	10.1523/JNEUROSCI.2153-12.2012
ProtModification: MAPK1 ---> PPAF	24194944	10.1371/journal.pone.0078536
ProtModification: MAPK1 ---> PPAF	24194944	10.1371/journal.pone.0078536
ProtModification: MAPK1 ---> PPAF	24304723	10.1254/jphs.13R09CP
ProtModification: MAPK1 ---> PPARG		10.1074/jbc.M113.536805
ProtModification: MAPK1 ---> PPAF	24189421	10.1248/bpb.b13-00351
ProtModification: MAPK1 ---> PPARG		10.1530/ERC-13-0529
ProtModification: MAPK1 ---> PPAF	25409143	10.1038/nature13887

ProtModification: MAPK1 ---> PPAF	25409143	10.1038/nature13887
ProtModification: MAPK1 ---> PPAF	25790378	10.1371/journal.pone.0120073
ProtModification: MAPK1 ---> PPAF	26125905	10.4238/2015.June.26.5
ProtModification: MAPK1 ---> PPARG		10.1074/jbc.M115.638924
ProtModification: MAPK1 ---> PPAF	25409143	10.1038/nature1388710.1038/nature13887
ProtModification: MAPK1 ---> PPAF	17632092	10.1016/j.cbi.2007.05.010
ProtModification: MAPK1 ---> PPAF	23490586	10.1016/j.metabol.2013.01.025
ProtModification: MAPK1 ---> PPAF	24915420	10.1016/j.bcp.2014.06.002
ProtModification: MAPK1 ---> PPAF	27268145	10.1016/j.phrs.2016.02.028
ProtModification: MAPK1 ---> PPAF	16873530	
ProtModification: MAPK1 ---> PPAF	16835228	
ProtModification: MAPK1 ---> PPAF	16155293	
ProtModification: MAPK1 ---> PPAF	15067015	
ProtModification: MAPK1 ---> PPAF	18287349	
ProtModification: MAPK1 ---> PPAF	22593575	10.1074/jbc.M112.350181
ProtModification: MAPK1 ---> PPAF	22593575	10.1074/jbc.M112.350181
ProtModification: MAPK1 ---> PPAF	22593575	10.1074/jbc.M112.350181
ProtModification: MAPK1 ---> PPAF	22342903	10.1016/j.biochi.2012.01.008
ProtModification: MAPK1 ---> PPAF	22561024	10.1016/j.rbmo.2012.02.012
ProtModification: MAPK1 ---> PPAF	20809049	10.2119/molmed.2010.00063
ProtModification: MAPK1 ---> PPAF	21854054	10.1021/jf2014233
ProtModification: MAPK1 ---> PPARG		
ProtModification: MAPK1 ---> PPAF	29299041	10.1186/s12986-017-0234-y
ProtModification: MAPK1 ---> PPAF	29299041	10.1186/s12986-017-0234-y
ProtModification: MAPK1 ---> PPAF	946	10.1016/j.ejcb.2018.03.001
ProtModification: MAPK1 ---> PPAF	32878272	10.3390/ijms21176300
ProtModification: MAPK1 ---> PPAF	35040437	10.1172/JCI148552
ProtModification: MAPK1 ---> PPAF	35254645	10.1007/s40265-022-01689-0
ProtModification: MAPK1 ---> PPAF	35501783	10.1186/s12915-022-01287-2
negative Regulation: KDM5B ---| c	32440821	10.1007/s12031-020-01587-8
negative Regulation: KDM5B ---| c	25951238	10.1371/journal.pone.0125343
positive Expression: CD34 --+> G(	30909158	10.1016/j.redox.2019.101153
positive Regulation: TNFAIP8L2 --	30114619	10.1016/j.molimm.2018.08.010
Regulation: PRR5L ---> AKT1	29344641	10.3892/ijo.2018.4244
Regulation: PRR5L ---> AKT1	25486193	10.4161/15384101.2014.941747
positive Expression: KDM5B --+> F	33304897	10.3389/fcell.2020.55373310.3389/fcell.2020.5
positive Expression: KDM5B --+> F	35946516	10.1097/CAD.0000000000001329
positive Expression: KDM5B --+> F	35946516	10.1097/CAD.0000000000001329
positive Expression: KDM5B --+> F	35946516	10.1097/CAD.0000000000001329
positive Expression: KDM5B --+> F	25909289	10.18632/oncotarget.3713
positive Expression: KDM5B --+> F	30628696	10.3892/mmr.2019.9832
positive Expression: KDM5B --+> F	28958389	10.1016/j.trecan.2017.08.004
positive Expression: KDM5B --+> F	35946516	10.1097/CAD.0000000000001329
positive Expression: KDM5B --+> F	28958389	10.1016/j.trecan.2017.08.004
positive Expression: NOTCH1 --+>	33587255	10.1007/s12020-021-02651-y
negative Regulation: UCA1 ---| ch	30825424	10.1016/j.clinbiochem.2019.02.013
negative Regulation: UCA1 ---| ch	33901464	10.1016/j.ejphar.2021.174114
negative Regulation: UCA1 ---| ch	30107990	10.1016/j.prp.2018.07.036
negative Regulation: UCA1 ---| ch	29674277	10.1016/j.biopha.2018.03.138
negative Regulation: UCA1 ---| ch	32146422	10.1016/j.drup.2020.100683
negative Regulation: UCA1 ---| ch	31611036	10.1016/j.oooo.2019.08.011
negative Regulation: UCA1 ---| ch	29441929	10.1691/ph.2016.6625
negative Regulation: UCA1 ---| ch	29441929	10.1691/ph.2016.6625
negative Regulation: UCA1 ---| ch	29441929	10.1691/ph.2016.6625

negative Regulation: UCA1 ---| ch 29441929 10.1691/ph.2016.6625
negative Regulation: UCA1 ---| ch 29441929 10.1691/ph.2016.6625
negative Regulation: UCA1 ---| ch 29441929 10.1691/ph.2016.6625
negative Regulation: UCA1 ---| ch 29441929 10.1691/ph.2016.6625
negative Regulation: UCA1 ---| ch 29367594 10.1038/s41419-017-0113-5
negative Regulation: UCA1 ---| ch 30015983 10.3892/or.2018.6573
negative Regulation: UCA1 ---| ch 30868851 10.7754/Clin.Lab.2018.180739
negative Regulation: UCA1 ---| ch 31173289 10.26355/eurrev_201905_17921
negative Regulation: UCA1 ---| ch 31528122 10.1186/s12935-019-0934-7
negative Regulation: UCA1 ---| ch 33936199 10.1155/2021/5519720
negative Regulation: UCA1 ---| ch 33936199 10.1155/2021/5519720
negative Regulation: UCA1 ---| ch 27591936 10.1016/j.canlet.2016.08.01510.1016/j.canlet.
negative Regulation: UCA1 ---| ch 29441929 10.1691/ph.2016.662510.1691/ph.2016.6625
negative Regulation: UCA1 ---| ch 29441929 10.1691/ph.2016.662510.1691/ph.2016.6625
negative Regulation: UCA1 ---| ch 29441929 10.1691/ph.2016.662510.1691/ph.2016.6625
negative Regulation: PAX8 ---| NC 31391063 10.1186/s13046-019-1332-7
Regulation: LRP8 ---> AKT1 12376533 10.1074/jbc.M209205200
Regulation: LRP8 ---> AKT1 12376533 10.1074/jbc.M209205200
Regulation: LRP8 ---> AKT1 12376533 10.1074/jbc.M209205200
Regulation: LRP8 ---> AKT1 30227220 10.1016/j.canlet.2018.09.022
Regulation: LRP8 ---> AKT1 12376533
Regulation: LRP8 ---> AKT1 12376533
Regulation: LRP8 ---> AKT1 12376533
Regulation: LRP8 ---> AKT1 12376533
Regulation: LRP8 ---> AKT1 28129662 10.1055/s-0036-1597286
Regulation: LRP8 ---> AKT1 30451721 10.1097/MOH.0000000000000473
Regulation: LRP8 ---> AKT1 26474194 10.1002/art.3945310.1002/art.39453
Regulation: LRP8 ---> AKT1 28386352
Regulation: LRP8 ---> AKT1 10.1097/MOH.0000000000000473
Regulation: LRP8 ---> AKT1
Regulation: LRP8 ---> AKT1 33626909 10.1161/ATVBAHA.121.316000
Regulation: LRP8 ---> AKT1 36446154 10.1016/j.bbrc.2022.10.101
Expression: AKT1 ---> CD34 27645691 10.1016/j.exphem.2016.09.003
Expression: AKT1 ---> CD34 26073521 10.1016/j.exphem.2015.06.001
Expression: AKT1 ---> CD34 34928395 10.1007/s00421-021-04865-4
positive Regulation: CD34 --+> PT 11158605
ProtModification: PTEN ---> AKT1 32201226 10.1016/j.jaut.2020.102440
ProtModification: PTEN ---> AKT1 19264909 10.2353/ajpath.2009.080460
ProtModification: PTEN ---> AKT1 20515660 10.1016/j.bbrc.2010.05.140
ProtModification: PTEN ---> AKT1 22074824 10.1016/j.bbrc.2011.10.120
ProtModification: PTEN ---> AKT1 22074824 10.1016/j.bbrc.2011.10.120
ProtModification: PTEN ---> AKT1 20223231 10.1016/j.cca.2010.02.074
ProtModification: PTEN ---> AKT1 16919259 10.1016/j.ydbio.2006.06.001
ProtModification: PTEN ---> AKT1 15652487 10.1016/j.cell.2004.12.033
ProtModification: PTEN ---> AKT1 22244698 10.1016/j.biomaterials.2011.12.051
ProtModification: PTEN ---> AKT1 20347952 10.1016/j.exger.2010.03.015
ProtModification: PTEN ---> AKT1 20678571 10.1016/j.freeradbiomed.2010.07.020
ProtModification: PTEN ---> AKT1 18495460 10.1016/j.jnutbio.2007.12.001
ProtModification: PTEN ---> AKT1 12946479 10.1016/S0960-7404(03)00037-9
ProtModification: PTEN ---> AKT1 15172356 10.1016/S1470-2045(04)01492-5
ProtModification: PTEN ---> AKT1 10.1016/S1535-6108(02)00024-7
ProtModification: PTEN ---> AKT1 10.1016/S1535-6108(03)00331-3
ProtModification: PTEN ---> AKT1 20801415 10.1016/j.trsl.2010.06.003
ProtModification: PTEN ---> AKT1 23403165 10.1016/j.bbcan.2013.01.004

ProtModification: PTEN ---> AKT1 23458710 10.1016/j.neuroscience.2013.02.046
ProtModification: PTEN ---> AKT1 24041630 10.1016/j.critrevonc.2013.08.003
ProtModification: PTEN ---> AKT1 24561200 10.1016/j.febslet.2014.02.005
ProtModification: PTEN ---> AKT1 24911372 10.1016/j.ajpath.2014.05.004
ProtModification: PTEN ---> AKT1 25533812 10.1016/j.phrs.2014.12.004
ProtModification: PTEN ---> AKT1 25721811 10.1016/j.semcdb.2015.02.012
ProtModification: PTEN ---> AKT1 27207584 10.1016/j.yexcr.2016.05.011
ProtModification: PTEN ---> AKT1 27017291 10.1016/j.ejca.2016.02.016
ProtModification: PTEN ---> AKT1 27297622 10.1016/j.biocel.2016.06.006
ProtModification: PTEN ---> AKT1 20630061
ProtModification: PTEN ---> AKT1 18258752
ProtModification: PTEN ---> AKT1 18258752
ProtModification: PTEN ---> AKT1 17142836
ProtModification: PTEN ---> AKT1 10400703
ProtModification: PTEN ---> AKT1 18268330
ProtModification: PTEN ---> AKT1 15539459
ProtModification: PTEN ---> AKT1 15087424
ProtModification: PTEN ---> AKT1 10988233
ProtModification: PTEN ---> AKT1 17657248
ProtModification: PTEN ---> AKT1 10435616
ProtModification: PTEN ---> AKT1 10435616
ProtModification: PTEN ---> AKT1 11997518
ProtModification: PTEN ---> AKT1 21253589
ProtModification: PTEN ---> AKT1 21170325
ProtModification: PTEN ---> AKT1 20436671
ProtModification: PTEN ---> AKT1 26563373 10.1007/s13277-015-4392-0
ProtModification: PTEN ---> AKT1 27793908 10.21873/anticanres.11170
ProtModification: PTEN ---> AKT1 28289208 10.1073/pnas.1612284114
ProtModification: PTEN ---> AKT1 28229220 10.1007/s00432-016-2333-y
ProtModification: PTEN ---> AKT1 28384045 10.1089/gtmb.2016.0281
ProtModification: PTEN ---> AKT1 28358430 10.3892/mmr.2017.6415
ProtModification: PTEN ---> AKT1 28358430 10.3892/mmr.2017.6415
ProtModification: PTEN ---> AKT1 28498467 10.3892/ijo.2017.3982
ProtModification: PTEN ---> AKT1 28886696 10.1186/s12885-017-3639-0
ProtModification: PTEN ---> AKT1 29038421 10.1038/s41467-017-00906-9
ProtModification: PTEN ---> AKT1 29121869 10.1186/s12885-017-3714-6
ProtModification: PTEN ---> AKT1 28982275 10.1177/1933719117734700
ProtModification: PTEN ---> AKT1 29463952 10.1155/2017/5458178
ProtModification: PTEN ---> AKT1 29488607 10.3892/mmr.2018.8640
ProtModification: PTEN ---> AKT1 30134935 10.1186/s13046-018-0869-1
ProtModification: PTEN ---> AKT1 31138169 10.1186/s12885-019-5731-0
ProtModification: PTEN ---> AKT1 31006103 10.1007/s10549-019-05239-3
ProtModification: PTEN ---> AKT1 10.1038/s41374-018-0185-6
ProtModification: PTEN ---> AKT1 30318001 10.2174/1871520618666181012093056
ProtModification: PTEN ---> AKT1 10.1155/2019/2902985
ProtModification: PTEN ---> AKT1 32230887 10.3390/ijms21072322
ProtModification: PTEN ---> AKT1 32493394 10.1186/s12964-020-00586-x
ProtModification: PTEN ---> AKT1 22393246 10.1124/jpet.112.191536
ProtModification: PTEN ---> AKT1 23023710 10.1172/JCI61269
ProtModification: PTEN ---> AKT1 22465967 10.1007/s00262-012-1249-x
ProtModification: PTEN ---> AKT1 23239155 10.1164/rccm.201205-0851OC
ProtModification: PTEN ---> AKT1 20795550 10.1007/978-1-4419-6451-9_15
ProtModification: PTEN ---> AKT1 21953448 10.1074/jbc.M111.270884
ProtModification: PTEN ---> AKT1 22233681 10.1017/S1462399411002109

ProtModification: PTEN ---> AKT1 20630061 10.1186/1471-2407-10-368
ProtModification: PTEN ---> AKT1 22035408 10.1186/1475-2867-11-36
ProtModification: PTEN ---> AKT1 22107784 10.1186/1479-5876-9-200
ProtModification: PTEN ---> AKT1 21263312 10.1097/CAD.0b013e328341ccdd
ProtModification: PTEN ---> AKT1 21556061 10.1038/ncomms1309
ProtModification: PTEN ---> AKT1 23970032 10.3892/ijo.2013.2064
ProtModification: PTEN ---> AKT1 20436671 10.1371/journal.pone.0010350
ProtModification: PTEN ---> AKT1 21170325 10.1371/journal.pone.0015170
ProtModification: PTEN ---> AKT1 21253589 10.1371/journal.pone.0015909
ProtModification: PTEN ---> AKT1 21494560 10.1371/journal.pone.0018702
ProtModification: PTEN ---> AKT1 22194952 10.1371/journal.pone.0028930
ProtModification: PTEN ---> AKT1 24113181 10.1038/cddis.2013.375
ProtModification: PTEN ---> AKT1 24040957 10.1089/ars.2013.5619
ProtModification: PTEN ---> AKT1 24786832 10.1038/cdd.2014.53
ProtModification: PTEN ---> AKT1 25677517 10.1161/CIRCRESAHA.116.305348
ProtModification: PTEN ---> AKT1 21421815 10.1152/ajpheart.01138.2010
ProtModification: PTEN ---> AKT1 23812635 10.1152/ajplung.00432.2012
ProtModification: PTEN ---> AKT1 24651020 10.1515/revneuro-2014-0002
ProtModification: PTEN ---> AKT1 25778370 10.1186/s13058-015-0534-y
ProtModification: PTEN ---> AKT1 25982630 10.1038/aps.2015.24
ProtModification: PTEN ---> AKT1 259 10.1016/j.ceca.2017.01.003
ProtModification: PTEN ---> AKT1 23953228 10.1016/j.humpath.2013.05.014
ProtModification: PTEN ---> AKT1 17478730
ProtModification: PTEN ---> AKT1 11103942
ProtModification: PTEN ---> AKT1 27634014 10.1152/ajpgi.00292.2016
ProtModification: PTEN ---> AKT1 28358430 10.3892/mmr.2017.6415
ProtModification: PTEN ---> AKT1 28524854 10.1038/cdd.2017.73
ProtModification: PTEN ---> AKT1 28957811 10.1159/000481648
ProtModification: PTEN ---> AKT1 28982275 10.1177/1933719117734700
ProtModification: PTEN ---> AKT1 28982275 10.1177/1933719117734700
ProtModification: PTEN ---> AKT1 28982275 10.1177/1933719117734700
ProtModification: PTEN ---> AKT1 28982275 10.1177/1933719117734700
ProtModification: PTEN ---> AKT1 20596601 10.3892/ijmm-00000455
ProtModification: PTEN ---> AKT1 21267808 10.1055/s-0030-1250735
ProtModification: PTEN ---> AKT1 22364283 10.1042/BJ20112037
ProtModification: PTEN ---> AKT1 24736893 10.3892/mmr.2014.2155
ProtModification: PTEN ---> AKT1 25234167 10.1038/leu.2014.278
ProtModification: PTEN ---> AKT1 25825815 10.1210/en.2014-1362
ProtModification: PTEN ---> AKT1 27829222 10.18632/oncotarget.1311310.18632/oncotarget.
ProtModification: PTEN ---> AKT1 27829222 10.18632/oncotarget.1311310.18632/oncotarget.
ProtModification: PTEN ---> AKT1 21874010 10.1038/modpathol.2011.133
ProtModification: PTEN ---> AKT1 21874010 10.1038/modpathol.2011.133
ProtModification: PTEN ---> AKT1 25333206 10.3892/mmr.2014.2700
ProtModification: PTEN ---> AKT1 10.1074/jbc.M115.684746
ProtModification: PTEN ---> AKT1
ProtModification: PTEN ---> AKT1 26692175 10.1038/ni.3311
ProtModification: PTEN ---> AKT1 27035337 10.3892/mmr.2016.5048
ProtModification: PTEN ---> AKT1 26589417 10.1007/s13277-015-4456-1
ProtModification: PTEN ---> AKT1 20515660 10.1016/j.bbrc.2010.05.140
ProtModification: PTEN ---> AKT1 28259931 10.3892/mmr.2017.6204
ProtModification: PTEN ---> AKT1 10.1200/JCO.18.01207
ProtModification: PTEN ---> AKT1 31006103 10.1007/s10549-019-05239-310.1007/s10549-019-
ProtModification: PTEN ---> AKT1 33189865 10.1016/j.mce.2020.111080
ProtModification: PTEN ---> AKT1 33189865 10.1016/j.mce.2020.111080

ProtModification: PTEN ---> AKT1 33554779 10.1152/AJPENDO.00275.2020
ProtModification: PTEN ---> AKT1 33554779 10.1152/AJPENDO.00275.2020
ProtModification: PTEN ---> AKT1 33895342 10.1016/j.neuroscience.2021.04.01010.1016/j.n
ProtModification: PTEN ---> AKT1 34248141 10.1038/s41392-021-00571-x10.1038/s41392-021-
ProtModification: PTEN ---> AKT1 12010818 10.1182/blood.V99.11.4138
ProtModification: PTEN ---> AKT1 17142836 10.1074/jbc.M604195200
ProtModification: PTEN ---> AKT1 26324707 10.1074/jbc.M115.684746
ProtModification: PTEN ---> AKT1 21953448 10.1074/jbc.M111.270884
ProtModification: PTEN ---> AKT1 31884408 10.1016/j.ejmech.2019.111972
ProtModification: PTEN ---> AKT1 29136769 10.1016/j.biopha.2017.11.014
ProtModification: PTEN ---> AKT1 15851405 10.1093/annonc/mdi182
ProtModification: PTEN ---> AKT1 31492635 10.1016/j.molcel.2019.08.006
ProtModification: PTEN ---> AKT1 33098764 10.1016/j.molcel.2020.10.003
ProtModification: PTEN ---> AKT1 29729889 10.1016/j.biocel.2018.04.020
ProtModification: PTEN ---> AKT1 28578144 10.1016/j.etap.2017.05.013
ProtModification: PTEN ---> AKT1 32058031 10.1016/j.bbalip.2020.158658
ProtModification: PTEN ---> AKT1 30598407 10.1016/j.oooo.2018.11.011
ProtModification: PTEN ---> AKT1 25482988 10.1016/j.jbior.2014.10.004
ProtModification: PTEN ---> AKT1 30683902 10.1038/s41374-018-0185-6
ProtModification: PTEN ---> AKT1 31929796 10.1155/2019/2902985
ProtModification: PTEN ---> AKT1 34635142 10.1186/s13046-021-02097-0
negative Expression: PTEN ---| CI 23470565 10.1038/cgt.2013.14
negative Expression: PTEN ---| CI 23470565 10.1038/cgt.2013.1410.1038/cgt.2013.14
Regulation: SPP1 ---> PTEN 28928218 10.1074/jbc.M117.797787
Regulation: SPP1 ---> PTEN 28928218 10.1074/jbc.M117.797787
Regulation: SPP1 ---> PTEN 19286136 10.1016/j.chom.2009.01.005
Regulation: SPP1 ---> PTEN 19683677 10.1016/j.chom.2009.07.008
Regulation: SPP1 ---> PTEN 24039584 10.1371/journal.ppat.1003623
Expression: RBP1 ---> PPARG 26936961 10.2337/db15-1315
Expression: RBP1 ---> PPARG 20498279 10.1128/MCB.00014-1010.1128/MCB.00014-10
negative Expression: GPER1 ---| F 23142288 10.1016/j.canlet.2012.10.037
negative Expression: GPER1 ---| F 30093934
negative Expression: GPER1 ---| F 34860303 10.1007/s10522-021-09945-8
positive Expression: NOTCH1 --+> 18237408
positive Expression: NOTCH1 --+> 29158034 10.1016/j.yjmcc.2017.11.00910.1016/j.yjmcc.20
positive Regulation: SPP1 --+> R 18174176 10.1074/jbc.M706712200
positive Regulation: SPP1 --+> R 27576187 10.1016/j.mce.2016.08.042
positive Regulation: SPP1 --+> R 27593345 10.1016/j.ccell.2016.08.002
positive Regulation: SPP1 --+> R 18174176
positive Regulation: SPP1 --+> R 18174176
positive Regulation: SPP1 --+> R 27006454 10.1161/STROKEAHA.115.011552
positive Regulation: SPP1 --+> R 1117 10.1016/j.ccell.2016.08.002
positive Regulation: SPP1 --+> RAC1 10.1161/STROKEAHA.115.011552
positive Regulation: SPP1 --+> R 18174176
positive Regulation: SPP1 --+> R 18174176 10.1074/jbc.M706712200
positive Regulation: SFRP1 --+> c 24643460 10.2119/molmed.2013.00149
positive Regulation: SFRP1 --+> c 24643460 10.2119/molmed.2013.00149
positive Regulation: SFRP1 --+> c 24643460 10.2119/molmed.2013.00149
Regulation: NOTCH1 ---> hypophary 31778670 10.1016/j.yexcr.2019.111746
positive QuantitativeChange: hypo 26704053 10.1016/j.yexmp.2015.12.008
positive QuantitativeChange: hypo 30425527 10.2147/OTT.S17542310.2147/OTT.S175423
positive Expression: PPARG --+> (28237650 10.1016/j.bcp.2017.02.017
positive Expression: PPARG --+> (25389363 10.1152/ajpendo.00118.2014
positive Expression: PPARG --+> (25389363 10.1152/ajpendo.00118.2014

positive Expression: PPARG --+> C	19944311	10.1016/j.jjcc.2009.06.00410.1016/j.jjcc.2009
positive Expression: PPARG --+> C	25618409	10.1093/cvr/cvv01810.1093/cvr/cvv018
positive Expression: PPARG --+> C	22223859	10.1194/jlr.M017319
positive Expression: PPARG --+> C	22223859	10.1194/jlr.M017319
positive Expression: PPARG --+> C	22223859	10.1194/jlr.M017319
positive Expression: PPARG --+> C	23446230	10.1194/jlr.M036533
positive Expression: PPARG --+> CYP2J2		
positive Regulation: ING4 --+> ch	29494959	10.1016/j.biopha.2018.02.066
positive Regulation: ING4 --+> ch	15882981	10.1016/j.bbrc.2005.04.023
positive Regulation: ING4 --+> ch	27484725	10.3892/mmr.2016.5552
positive Regulation: ING4 --+> ch	27484725	10.3892/mmr.2016.5552
positive Regulation: ING4 --+> ch	20707719	10.1089/cbr.2010.0778
positive Regulation: ING4 --+> ch	22863759	10.1038/cgt.2012.50
positive Regulation: ING4 --+> ch	21052098	10.1038/cgt.2010.67
positive Regulation: ING4 --+> ch	23969950	10.3892/or.2013.2671
positive Regulation: ING4 --+> ch	23969950	10.3892/or.2013.2671
positive Regulation: ING4 --+> ch	23991130	10.1371/journal.pone.0072615
positive Regulation: ING4 --+> ch	21553494	
negative Regulation: NDUFA4L2 ---	28344054	10.1016/j.yexcr.2017.03.052
Regulation: TP53 ---> PAX8	9722166	10.1016/S0303-7207(98)00027-6
Regulation: TP53 ---> PAX8	12050209	
Regulation: TP53 ---> PAX8	23868062	10.1038/cddis.2013.262
Regulation: TP53 ---> PAX8		
positive QuantitativeChange: hypo	26436506	10.4238/2015.October.2.15
MolTransport: RAC1 ---> IGFBP4	26305333	10.1371/journal.pgen.1005458
MolTransport: RAC1 ---> IGFBP4	26305333	10.1371/journal.pgen.1005458
MolTransport: RAC1 ---> IGFBP4	26305333	10.1371/journal.pgen.1005458
Expression: CYP2J2 ---> PPARG	22223859	10.1194/jlr.M017319
Expression: CYP2J2 ---> PPARG	23313758	10.1016/j.ejphar.2012.12.025
Expression: CYP2J2 ---> PPARG	22223859	10.1194/jlr.M017319
Expression: CYP2J2 ---> PPARG	23515292	10.1210/en.2012-2012
Expression: CYP2J2 ---> PPARG	23515292	10.1210/en.2012-2012
Expression: CYP2J2 ---> PPARG	23446230	10.1194/jlr.M036533
Expression: CYP2J2 ---> PPARG	23696562	10.1210/en.2012-2166
Expression: CYP2J2 ---> PPARG	23696562	10.1210/en.2012-2166
Expression: CYP2J2 ---> PPARG	23696562	10.1210/en.2012-2166
Expression: CYP2J2 ---> PPARG	25389363	10.1152/ajpendo.00118.2014
Expression: CYP2J2 ---> PPARG	25389363	10.1152/ajpendo.00118.2014
Expression: CYP2J2 ---> PPARG	25389363	10.1152/ajpendo.00118.2014
Expression: CYP2J2 ---> PPARG	25389363	10.1152/ajpendo.00118.2014
Expression: CYP2J2 ---> PPARG	22223859	10.1194/jlr.M01731910.1194/jlr.M017319
Expression: CYP2J2 ---> PPARG	34774051	10.1186/s12931-021-01891-w
positive Expression: AKT1 --+> CI	29679564	10.1016/j.bbrc.2018.04.120
positive Expression: AKT1 --+> CI	32422139	10.1016/j.bcp.2020.114038
positive Expression: AKT1 --+> CI	32603752	10.1016/j.jid.2020.05.113
positive Expression: AKT1 --+> CI	30927618	10.1016/j.jss.2019.02.038
positive Expression: AKT1 --+> CI	32070710	10.1016/j.lfs.2020.117437
positive Expression: AKT1 --+> CI	32593711	10.1016/j.lfs.2020.118005
positive Expression: AKT1 --+> CI	32593711	10.1016/j.lfs.2020.118005
positive Expression: AKT1 --+> CI	33513401	10.1016/j.lfs.2021.119110
positive Expression: AKT1 --+> CI	33798547	10.1016/j.lfs.2021.119353
positive Expression: AKT1 --+> CI	32120136	10.1016/j.mrfmmm.2020.111690
positive Expression: AKT1 --+> CI	33159965	10.1016/j.humpath.2020.10.014
positive Expression: AKT1 --+> CI	33159965	10.1016/j.humpath.2020.10.014

positive Expression: AKT1 --+> CI 32183989 10.1016/j.acthis.2020.151538
positive Expression: AKT1 --+> CI 32183989 10.1016/j.acthis.2020.151538
positive Expression: AKT1 --+> CI 32183989 10.1016/j.acthis.2020.151538
positive Expression: AKT1 --+> CI 28720430 10.1016/j.pharmthera.2017.07.004
positive Expression: AKT1 --+> CI 30825473 10.1016/j.pharmthera.2019.02.006
positive Expression: AKT1 --+> CI 32473961 10.1016/j.pharmthera.2020.107585
positive Expression: AKT1 --+> CI 33421449 10.1016/j.pharmthera.2021.107800
positive Expression: AKT1 --+> CI 28223102 10.1016/j.imlet.2017.02.006
positive Expression: AKT1 --+> CI 33647320 10.1016/j.bbamcr.2021.118992
positive Expression: AKT1 --+> CI 30249348 10.1016/j.radonc.2018.08.023
positive Expression: AKT1 --+> CI 28213003 10.1016/j.lungcan.2016.12.006
positive Expression: AKT1 --+> CI 29413045 10.1016/j.lungcan.2017.12.003
positive Expression: AKT1 --+> CI 29800685 10.1016/j.canlet.2018.05.015
positive Expression: AKT1 --+> CI 31830559 10.1016/j.canlet.2019.12.009
positive Expression: AKT1 --+> CI 29598887 10.1016/j.prp.2018.03.001
positive Expression: AKT1 --+> CI 32057515 10.1016/j.prp.2020.152852
positive Expression: AKT1 --+> CI 29031195 10.1016/j.biopha.2017.09.129
positive Expression: AKT1 --+> CD274 10.1093/annonc/mdv528.08
positive Expression: AKT1 --+> CI 26681673 10.1093/annonc/mdv615
positive Expression: AKT1 --+> CI 26681673 10.1093/annonc/mdv615
positive Expression: AKT1 --+> CI 26681673 10.1093/annonc/mdv615
positive Expression: AKT1 --+> CI 26681673 10.1093/annonc/mdv615
positive Expression: AKT1 --+> CI 32136408 10.1093/annonc/mdy268.004
positive Expression: AKT1 --+> CI 30462163 10.1093/annonc/mdy507
positive Expression: AKT1 --+> CD274 10.1016/j.ejca.2018.07.165
positive Expression: AKT1 --+> CI 32294525 10.1016/j.phrs.2020.104806
positive Expression: AKT1 --+> CI 32294525 10.1016/j.phrs.2020.104806
positive Expression: AKT1 --+> CI 32294525 10.1016/j.phrs.2020.104806
positive Expression: AKT1 --+> CI 32687951 10.1016/j.phrs.2020.105086
positive Expression: AKT1 --+> CI 28476657 10.1016/j.semcancer.2017.04.018
positive Expression: AKT1 --+> CI 30951826 10.1016/j.semcancer.2019.04.001
positive Expression: AKT1 --+> CI 31422157 10.1016/j.semcancer.2019.08.015
positive Expression: AKT1 --+> CI 31874279 10.1016/j.semcancer.2019.11.011
positive Expression: AKT1 --+> CI 33301862 10.1016/j.semcancer.2020.12.001
positive Expression: AKT1 --+> CI 33301862 10.1016/j.semcancer.2020.12.001
positive Expression: AKT1 --+> CI 27192563 10.1016/j.immuni.2016.05.002
positive Expression: AKT1 --+> CI 29562194 10.1016/j.immuni.2018.03.014
positive Expression: AKT1 --+> CI 33308976 10.1016/j.urolonc.2020.11.029
positive Expression: AKT1 --+> CI 33909988 10.1016/j.molcel.2021.03.037
positive Expression: AKT1 --+> CI 33909988 10.1016/j.molcel.2021.03.037
positive Expression: AKT1 --+> CI 33909988 10.1016/j.molcel.2021.03.037
positive Expression: AKT1 --+> CI 31005030 10.1016/j.drup.2019.04.001
positive Expression: AKT1 --+> CI 31005030 10.1016/j.drup.2019.04.001
positive Expression: AKT1 --+> CI 31005030 10.1016/j.drup.2019.04.001
positive Expression: AKT1 --+> CI 31005030 10.1016/j.drup.2019.04.001
positive Expression: AKT1 --+> CI 33707115 10.1016/j.pan.2021.01.023
positive Expression: AKT1 --+> CI 27924752 10.1016/S1470-2045(16)30406-5
positive Expression: AKT1 --+> CI 29153898 10.1016/j.cllc.2017.10.014
positive Expression: AKT1 --+> CI 28478231 10.1016/j.jtho.2017.04.014
positive Expression: AKT1 --+> CI 28846888 10.1016/j.intimp.2017.08.017
positive Expression: AKT1 --+> CI 32866782 10.1016/j.intimp.2020.106864
positive Expression: AKT1 --+> CI 33838550 10.1016/j.dnarep.2021.103112
positive Expression: AKT1 --+> CI 31660091 10.1016/j.jdsr.2019.09.001
positive Expression: AKT1 --+> CI 29177095 10.1136/esmoopen-2017-000255

positive Expression: AKT1 --+> CI 33171331 10.1016/j.redox.2020.101780
positive Expression: AKT1 --+> CI 33171331 10.1016/j.redox.2020.101780
positive Expression: AKT1 --+> CI 33171331 10.1016/j.redox.2020.101780
positive Expression: AKT1 --+> CI 33171331 10.1016/j.redox.2020.101780
positive Expression: AKT1 --+> CI 33667716 10.1016/j.jcmgh.2021.02.010
positive Expression: AKT1 --+> CI 19010538 10.1016/j.placenta.2008.10.001
positive Expression: AKT1 --+> CI 17920123 10.1016/j.molimm.2007.08.013
positive Expression: AKT1 --+> CI 19944971 10.1016/j.nec.2009.09.002
positive Expression: AKT1 --+> CI 25499621 10.1016/j.cellsig.2014.12.003
positive Expression: AKT1 --+> CD274 10.1016/j.omtn.2021.05.011
positive Expression: AKT1 --+> CD274 10.7150/ijbs.62280
positive Expression: AKT1 --+> CI 34445735 10.3390/ijms22169030
positive Expression: AKT1 --+> CI 33860125 10.1002/hep4.165910.1002/hep4.1659
positive Expression: AKT1 --+> CI 34486197 10.1002/cbin.1169710.1002/cbin.11697
positive Expression: AKT1 --+> CI 34486197 10.1002/cbin.1169710.1002/cbin.11697
positive Expression: AKT1 --+> CI 33994852 10.7150/ijbs.5796410.7150/ijbs.57964
positive Expression: AKT1 --+> CI 19010538 10.1016/j.placenta.2008.10.00110.1016/j.place
positive Expression: AKT1 --+> CI 1109 10.1016/j.cllc.2017.10.014
positive Expression: AKT1 --+> CI 597 10.1016/j.prp.2018.03.001
positive Expression: AKT1 --+> CI 558 10.1016/j.radonc.2018.08.023
positive Expression: AKT1 --+> CD274 10.1016/j.semcancer.2019.04.001
positive Expression: AKT1 --+> CD274 10.1016/j.semcancer.2019.08.015
positive Expression: AKT1 --+> CD274 10.1016/j.semcancer.2019.11.011
positive Expression: AKT1 --+> CD274 10.1016/j.prp.2020.152852
positive Expression: AKT1 --+> CD274 10.1016/j.acthis.2020.151538
positive Expression: AKT1 --+> CD274 10.1016/j.acthis.2020.151538
positive Expression: AKT1 --+> CD274 10.1016/j.acthis.2020.151538
positive Expression: AKT1 --+> CI 25499621 10.1016/j.cellsig.2014.12.003
positive Expression: AKT1 --+> CI 26707383 10.1016/j.cllc.2015.11.006
positive Expression: AKT1 --+> CI 20626886
positive Expression: AKT1 --+> CI 16199861
positive Expression: AKT1 --+> CI 27572267 10.1038/ncomms12632
positive Expression: AKT1 --+> CI 26833481 10.1007/s12032-016-0733-0
positive Expression: AKT1 --+> CI 27617403 10.1038/labinvest.2016.96
positive Expression: AKT1 --+> CI 27793883 10.21873/anticanres.11145
positive Expression: AKT1 --+> CI 28231855 10.1186/s12967-017-1150-7
positive Expression: AKT1 --+> CI 27934877 10.1038/modpathol.2016.202
positive Expression: AKT1 --+> CI 28341875 10.1007/s00262-017-1986-y
positive Expression: AKT1 --+> CI 28341875 10.1007/s00262-017-1986-y
positive Expression: AKT1 --+> CI 28627635 10.3892/or.2017.5733
positive Expression: AKT1 --+> CI 28627635 10.3892/or.2017.5733
positive Expression: AKT1 --+> CI 28344319 10.1038/leu.2017.103
positive Expression: AKT1 --+> CI 28605599 10.1177/0022034517712435
positive Expression: AKT1 --+> CI 28714960 10.1038/onc.2017.217
positive Expression: AKT1 --+> CI 28436428 10.1038/ncomms14709
positive Expression: AKT1 --+> CI 28436428 10.1038/ncomms14709
positive Expression: AKT1 --+> CI 28910818 10.1159/000479231
positive Expression: AKT1 --+> CI 28969535 10.2174/1568009617666170927114440
positive Expression: AKT1 --+> CI 29765155 10.1038/s41388-018-0303-3
positive Expression: AKT1 --+> CI 29786078 10.1038/s41388-018-0314-0
positive Expression: AKT1 --+> CI 30120503 10.1007/s00262-018-2226-9
positive Expression: AKT1 --+> CI 30120503 10.1007/s00262-018-2226-9
positive Expression: AKT1 --+> CI 29855617 10.1038/s41388-018-0347-4
positive Expression: AKT1 --+> CD274 10.1172/JCI96610

positive Expression: AKT1 --+> CI30264678 10.2174/1389203719666180928105632
positive Expression: AKT1 --+> CD274 10.1172/JCI12127
positive Expression: AKT1 --+> CD274 10.1186/s12943-019-0953-y
positive Expression: AKT1 --+> CI30765874 10.1038/s41416-019-0397-6
positive Expression: AKT1 --+> CD274 10.1186/s12935-019-0789-y
positive Expression: AKT1 --+> CI29954840 10.1136/gutjnl-2018-316039
positive Expression: AKT1 --+> CI29954840 10.1136/gutjnl-2018-316039
positive Expression: AKT1 --+> CI30770752 10.1186/s12885-019-5364-3
positive Expression: AKT1 --+> CI30770752 10.1186/s12885-019-5364-3
positive Expression: AKT1 --+> CI30770752 10.1186/s12885-019-5364-3
positive Expression: AKT1 --+> CI31113825 10.1136/jclinpath-2019-205818
positive Expression: AKT1 --+> CD274 10.1007/s12272-019-01169-2
positive Expression: AKT1 --+> CD274 10.1038/s41388-019-0877-4
positive Expression: AKT1 --+> CD274 10.1042/BSR20190362
positive Expression: AKT1 --+> CI29187498 10.21873/anticanres.12180
positive Expression: AKT1 --+> CI30368626 10.1007/s10637-018-0678-6
positive Expression: AKT1 --+> CI32194893
positive Expression: AKT1 --+> CD274 10.1038/s41388-019-1001-5
positive Expression: AKT1 --+> CD274 10.1038/s41416-019-0697-x
positive Expression: AKT1 --+> CI32359357 10.1186/s12943-020-01200-x
positive Expression: AKT1 --+> CI32359357 10.1186/s12943-020-01200-x
positive Expression: AKT1 --+> CI21374075 10.1007/s12185-011-0799-6
positive Expression: AKT1 --+> CI23785454 10.1371/journal.pone.0065821
positive Expression: AKT1 --+> CI24551119 10.1371/journal.pone.0088557
positive Expression: AKT1 --+> CI26019170 10.1158/1078-0432.CCR-15-001610.1158/1078-043
positive Expression: AKT1 --+> CI26245467 10.1186/s12943-015-0421-210.1186/s12943-015-0
positive Expression: AKT1 --+> CI26637667 10.1158/0008-5472.CAN-14-336210.1158/0008-547
positive Expression: AKT1 --+> CI26637667 10.1158/0008-5472.CAN-14-336210.1158/0008-547
positive Expression: AKT1 --+> CI28223102 10.1016/j.imlet.2017.02.00610.1016/j.imlet.20
positive Expression: AKT1 --+> CI28341875 10.1007/s00262-017-1986-y10.1007/s00262-017-1
positive Expression: AKT1 --+> CI28627635 10.3892/or.2017.573310.3892/or.2017.5733
positive Expression: AKT1 --+> CI30266108 10.24920/2180410.24920/21804
positive Expression: AKT1 --+> CI31436252 10.4103/jcrt.JCRT_636_1810.4103/jcrt.JCRT_636
positive Expression: AKT1 --+> CI32294525 10.1016/j.phrs.2020.10480610.1016/j.phrs.2020
positive Expression: AKT1 --+> CI32294525 10.1016/j.phrs.2020.10480610.1016/j.phrs.2020
positive Expression: AKT1 --+> CD274 10.12659/MSM.923624
positive Expression: AKT1 --+> CI32963532 10.1155/2020/7314195
positive Expression: AKT1 --+> CI32860047 10.1084/JEM.20191115
positive Expression: AKT1 --+> CI32860047 10.1084/JEM.20191115
positive Expression: AKT1 --+> CI32860047 10.1084/JEM.20191115
positive Expression: AKT1 --+> CI32860047 10.1084/JEM.20191115
positive Expression: AKT1 --+> CI32860047 10.1084/JEM.20191115
positive Expression: AKT1 --+> CI32860047 10.1084/JEM.20191115
positive Expression: AKT1 --+> CI32860047 10.1084/jem.2019111510.1084/jem.20191115
positive Expression: AKT1 --+> CI33042623
positive Expression: AKT1 --+> CI27006653 10.1155/2016/6261721
positive Expression: AKT1 --+> CI27617403 10.1038/labinvest.2016.96
positive Expression: AKT1 --+> CI28436428 10.1038/ncomms14709
positive Expression: AKT1 --+> CI26149216 10.1016/j.febslet.2015.06.037
positive Expression: AKT1 --+> CI1975 10.1016/j.cllc.2015.11.006
positive Expression: AKT1 --+> CI28231855 10.1186/s12967-017-1150-7
positive Expression: AKT1 --+> CD274
positive Expression: AKT1 --+> CI28629173 10.3390/ijms18061297
positive Expression: AKT1 --+> CI30770752 10.1186/s12885-019-5364-3

positive Expression: AKT1 --+> CD274   10.1016/j.semcancer.2019.06.021
positive Expression: AKT1 --+> CD274   10.1016/j.semcancer.2019.11.011
positive Expression: AKT1 --+> CI32992449 10.3390/ijms21197117
positive Expression: AKT1 --+> CI32992658 10.3390/ijms21197139
positive Expression: AKT1 --+> CI33116132 10.1038/s41467-020-19288-6
positive Expression: AKT1 --+> CI33241021 10.21037/atm-20-586510.21037/atm-20-5865
positive Expression: AKT1 --+> CD274   10.1016/j.urolonc.2020.11.029
positive Expression: AKT1 --+> CI33301862 10.1016/j.semcancer.2020.12.001
positive Expression: AKT1 --+> CI33271941 10.3390/ijms21239165
positive Expression: AKT1 --+> CI32152439 10.1038/s41401-020-0366-x
positive Expression: AKT1 --+> CD274   10.3727/096504020X15925659763817
positive Expression: AKT1 --+> CI32603752 10.1016/j.jid.2020.05.113
positive Expression: AKT1 --+> CI33967272 10.1038/s41419-021-03745-1
positive Expression: AKT1 --+> CI34098945 10.1186/s12935-021-02003-w
positive Expression: AKT1 --+> CI34088360 10.1186/s13046-021-01987-7
positive Expression: AKT1 --+> CI34088360 10.1186/s13046-021-01987-7
positive Expression: AKT1 --+> CI34088360 10.1186/s13046-021-01987-7
positive Expression: AKT1 --+> CI34108491 10.1038/s41467-021-23864-9
positive Expression: AKT1 --+> CI34319029 10.31557/APJCP.2021.22.7.2069
positive Expression: AKT1 --+> CI34639059 10.3390/ijms221910719
positive Expression: AKT1 --+> CI34599143 10.1038/s41419-021-04171-z
positive Expression: AKT1 --+> CI34548615 10.1038/s12276-021-00670-3
positive Expression: AKT1 --+> CI34548615 10.1038/s12276-021-00670-3
positive Expression: AKT1 --+> CI34666670 10.1186/s10020-021-00384-2
positive Expression: AKT1 --+> CI34666670 10.1186/s10020-021-00384-2
positive Expression: AKT1 --+> CI34499223 10.1007/s00432-021-03792-3
positive Expression: AKT1 --+> CI34718325 10.1038/s41419-021-04293-410.1038/s41419-021-
positive Expression: AKT1 --+> CI34718325 10.1038/s41419-021-04293-4
positive Expression: AKT1 --+> CI34718325 10.1038/s41419-021-04293-4
positive Expression: AKT1 --+> CI34830179 10.3390/ijms222212297
positive Expression: AKT1 --+> CI34830179 10.3390/ijms222212297
positive Expression: AKT1 --+> CI34830179 10.3390/ijms222212297
positive Expression: AKT1 --+> CI34830209 10.3390/ijms222212330
positive Expression: AKT1 --+> CI34480337 10.1007/s12035-021-02495-7
positive Expression: AKT1 --+> CI35062949 10.1186/s12943-021-01489-2
positive Expression: AKT1 --+> CI34839353 10.1038/s41388-021-02124-6
positive Expression: AKT1 --+> CI35203446 10.3390/biomedicines1002023610.3390/biomedici
positive Expression: AKT1 --+> CI35031925 10.1007/s11033-021-07063-9
positive Expression: AKT1 --+> CI35305624 10.1186/s12935-022-02550-w
positive Expression: AKT1 --+> CI32917780 10.2967/jnumed.120.250720
positive Expression: AKT1 --+> CI32917780 10.2967/jnumed.120.250720
positive Expression: AKT1 --+> CI32917780 10.2967/jnumed.120.250720
positive Expression: AKT1 --+> CI35414774 10.7150/ijbs.67200
positive Expression: AKT1 --+> CI35610613 10.1186/s12885-022-09675-1
positive Expression: AKT1 --+> CI35628647 10.3390/ijms23105837
positive Expression: AKT1 --+> CI35682571 10.3390/ijms23115892
positive Expression: AKT1 --+> CI35682571 10.3390/ijms23115892
positive Expression: AKT1 --+> CI35817272 10.1016/j.ijpharm.2022.122002
positive Expression: AKT1 --+> CI35917090 10.1007/s13258-022-01291-4
positive Expression: AKT1 --+> CI35638450 10.1042/BSR20212051
positive Expression: AKT1 --+> CI35955729 10.3390/ijms23158599
positive Expression: AKT1 --+> CI36233088 10.3390/ijms231911789
positive Expression: AKT1 --+> CI36245982 10.1155/2022/5233222
positive Expression: AKT1 --+> CI36245982 10.1155/2022/5233222

positive Expression: AKT1 --+> CI	36313041	10.7150/jca.77619
positive Expression: AKT1 --+> CI	36313041	10.7150/jca.77619
positive Expression: AKT1 --+> CI	36348350	10.1186/s12964-022-00981-6
positive Expression: AKT1 --+> CI	36430322	10.3390/ijms232213846
positive Expression: AKT1 --+> CI	36499710	10.3390/ijms232315384
positive Expression: AKT1 --+> CI	36528587	10.1186/s12935-022-02805-6
Regulation: CD274 ---> AKT1	31668739	10.1016/j.cellimm.2019.103957
Regulation: CD274 ---> AKT1	33771483	10.1016/j.yexcr.2021.112575
Regulation: CD274 ---> AKT1	22337882	10.1074/jbc.M111.283705
Regulation: CD274 ---> AKT1	33065462	10.1016/j.lungcan.2020.10.004
Regulation: CD274 ---> AKT1	32428662	10.1016/j.canlet.2020.05.003
Regulation: CD274 ---> AKT1	30318472	10.1016/j.bbcan.2018.08.001
Regulation: CD274 ---> AKT1	28477990	10.1016/j.maturitas.2017.04.014
Regulation: CD274 ---> AKT1	29510196	10.1016/j.bbadis.2018.03.002
Regulation: CD274 ---> AKT1	31063806	10.1016/j.phrs.2019.104258
Regulation: CD274 ---> AKT1	33301862	10.1016/j.semcancer.2020.12.001
Regulation: CD274 ---> AKT1	33301862	10.1016/j.semcancer.2020.12.001
Regulation: CD274 ---> AKT1	28887097	10.1016/j.urolonc.2017.08.010
Regulation: CD274 ---> AKT1	31005030	10.1016/j.drup.2019.04.001
Regulation: CD274 ---> AKT1	27964820	10.1016/j.it.2016.11.005
Regulation: CD274 ---> AKT1	29576280	10.1016/j.hbpd.2018.03.001
Regulation: CD274 ---> AKT1	32663768	10.1016/j.intimp.2020.106759
Regulation: CD274 ---> AKT1	33581579	10.1016/j.intimp.2021.107443
Regulation: CD274 ---> AKT1	33581579	10.1016/j.intimp.2021.107443
Regulation: CD274 ---> AKT1	26321679	10.1016/j.cell.2015.08.016
Regulation: CD274 ---> AKT1	28703774	10.3390/ijms18071517
Regulation: CD274 ---> AKT1	28692048	10.1038/onc.2017.209
Regulation: CD274 ---> AKT1	28692048	10.1038/onc.2017.209
Regulation: CD274 ---> AKT1	28692048	10.1038/onc.2017.209
Regulation: CD274 ---> AKT1	28982322	10.2174/1381612823666171004120152
Regulation: CD274 ---> AKT1	30078052	10.1007/s00262-018-2221-1
Regulation: CD274 ---> AKT1	30808044	10.1055/s-0039-1679909
Regulation: CD274 ---> AKT1	32020232	10.3892/or.2020.7477
Regulation: CD274 ---> AKT1	32020232	10.3892/or.2020.7477
Regulation: CD274 ---> AKT1	30368626	10.1007/s10637-018-0678-6
Regulation: CD274 ---> AKT1	32161124	10.1073/pnas.1921445117
Regulation: CD274 ---> AKT1	32161124	10.1073/pnas.1921445117
Regulation: CD274 ---> AKT1	32161124	10.1073/pnas.1921445117
Regulation: CD274 ---> AKT1	32161124	10.1073/pnas.1921445117
Regulation: CD274 ---> AKT1	31853007	10.1038/s41416-019-0697-x
Regulation: CD274 ---> AKT1	32868929	10.1038/s41590-020-0769-3
Regulation: CD274 ---> AKT1	33472580	10.1186/s11658-020-00246-5
Regulation: CD274 ---> AKT1		10.5483/BMBRep.2021.54.1.241
Regulation: CD274 ---> AKT1		10.5483/BMBRep.2021.54.1.241
Regulation: CD274 ---> AKT1		10.5483/BMBRep.2021.54.1.241
Regulation: CD274 ---> AKT1	33112410	10.14670/HH-18-272
Regulation: CD274 ---> AKT1	34326692	10.7150/ijbs.60114
Regulation: CD274 ---> AKT1	21899511	
Regulation: CD274 ---> AKT1	22337882	10.1074/jbc.M111.283705
Regulation: CD274 ---> AKT1	23267208	10.2332/allergolint.12-OA-0450
Regulation: CD274 ---> AKT1	25057927	10.1097/SHK.0000000000000247
Regulation: CD274 ---> AKT1	25609381	10.1038/ncomms6997
Regulation: CD274 ---> AKT1	26927206	10.1146/annurev-immunol-032414-112049
Regulation: CD274 ---> AKT1	27671847	10.1002/stem.250910.1002/stem.2509

Regulation: CD274 ---> AKT1	29245948	10.18632/oncotarget.2191410.18632/oncotarget.
Regulation: CD274 ---> AKT1	31850228	10.3389/fonc.2019.0134710.3389/fonc.2019.0134
Regulation: CD274 ---> AKT1	32277292	10.1007/s12032-020-01372-y10.1007/s12032-020-
Regulation: CD274 ---> AKT1	919	10.1016/j.it.2016.11.005
Regulation: CD274 ---> AKT1	445	10.1016/j.maturitas.2017.04.014
Regulation: CD274 ---> AKT1		10.1016/j.cellimm.2019.103957
Regulation: CD274 ---> AKT1		10.3892/or.2020.7477
Regulation: CD274 ---> AKT1		10.3892/or.2020.7477
Regulation: CD274 ---> AKT1		10.1038/s41416-019-0697-x
Regulation: CD274 ---> AKT1		10.1097/SHK.0000000000000247
Regulation: CD274 ---> AKT1		10.14670/HH-18-272
Regulation: CD274 ---> AKT1	34847253	10.1096/fj.202101306R
Regulation: CD274 ---> AKT1	34482409	10.1007/s00262-021-03041-z
Regulation: CD274 ---> AKT1	35409396	10.3390/ijms23074037
Regulation: CD274 ---> AKT1	33030957	10.4149/neo_2020_200531N589
Regulation: CD274 ---> AKT1	33030957	10.4149/neo_2020_200531N589
Regulation: CD274 ---> AKT1	35444235	10.1038/s41419-022-04821-w
Regulation: CD274 ---> AKT1	35684481	10.3390/molecules27113545
Regulation: CD274 ---> AKT1	35301651	10.1007/s11033-022-07246-y
Regulation: CD274 ---> AKT1	36175691	10.1007/s12032-022-01842-5
Regulation: CD274 ---> AKT1	36232331	10.3390/ijms231911031
positive Expression: MAPK1 --+> /	23051672	10.1016/j.ejphar.2012.09.046
positive Expression: MAPK1 --+> /	30834839	10.2174/1568026619666190305130141
positive Expression: MAPK1 --+> /	22787275	10.1091/mbc.E12-02-0132
positive Expression: MAPK1 --+> /	22787275	10.1091/mbc.E12-02-0132
positive Expression: MAPK1 --+> /	22787275	10.1091/mbc.E12-02-0132
positive Expression: MAPK1 --+> /	22787275	10.1091/mbc.E12-02-0132
positive Expression: MAPK1 --+> /	24324065	
positive Expression: MAPK1 --+> /	26883473	10.3892/or.2016.4622
positive Expression: MAPK1 --+> /	22787275	10.1091/mbc.E12-02-013210.1091/mbc.E12-02-013
positive Expression: MAPK1 --+> /	16997801	
positive Expression: MAPK1 --+> E	25446102	10.1016/j.bbrc.2014.11.038
positive Expression: MAPK1 --+> E	19520853	10.1074/jbc.M109.026013
positive Expression: MAPK1 --+> E	29203148	10.1016/j.lfs.2017.12.001
positive Expression: MAPK1 --+> E	33412209	10.1016/j.lfs.2020.118978
positive Expression: MAPK1 --+> E	33581126	10.1016/j.lfs.2021.119188
positive Expression: MAPK1 --+> E	27667695	10.1016/j.toxlet.2016.09.007
positive Expression: MAPK1 --+> E	32322463	10.1016/j.apsb.2019.10.003
positive Expression: MAPK1 --+> E	32322463	10.1016/j.apsb.2019.10.003
positive Expression: MAPK1 --+> E	20150434	10.2353/ajpath.2010.090850
positive Expression: MAPK1 --+> E	22634147	10.1016/j.freeradbiomed.2012.05.018
positive Expression: MAPK1 --+> E	24735948	10.1016/j.freeradbiomed.2014.04.004
positive Expression: MAPK1 --+> E	19520853	
positive Expression: MAPK1 --+> E	19520853	
positive Expression: MAPK1 --+> E	29115504	10.3892/mmr.2017.7967
positive Expression: MAPK1 --+> E	29405820	10.1080/15384047.2018.1436024
positive Expression: MAPK1 --+> E	29943652	10.1089/ars.2017.7234
positive Expression: MAPK1 --+> E	31776989	10.1007/978-981-15-0602-4_12
positive Expression: MAPK1 --+> E	32271449	10.26355/eurrev_202003_20699
positive Expression: MAPK1 --+> E	33287140	10.3390/ijms21239210
positive Expression: MAPK1 --+> E	22466960	10.1254/jphs.11181FP
positive Expression: MAPK1 --+> E	21768780	10.4161/cbt.12.8.15978
positive Expression: MAPK1 --+> E	24305604	10.1186/1756-9966-32-99
positive Expression: MAPK1 --+> E	24416349	10.1371/journal.pone.0085116

positive Expression: MAPK1 --+> E 24416349 10.1371/journal.pone.0085116
positive Expression: MAPK1 --+> E 24416349 10.1371/journal.pone.0085116
positive Expression: MAPK1 --+> E 24603327 10.1038/cddis.2014.64
positive Expression: MAPK1 --+> E 22674982 10.1128/JVI.00616-12
positive Expression: MAPK1 --+> E 25760151 10.1055/s-0034-1398494
positive Expression: MAPK1 --+> E 25866220 10.4149/neo_2015_047
positive Expression: MAPK1 --+> E 24416349 10.1371/journal.pone.008511610.1371/journal.p
positive Expression: MAPK1 --+> E 25348263 10.1111/jnc.1298010.1111/jnc.12980
positive Expression: MAPK1 --+> BECN1 10.1016/j.apsb.2019.10.003
positive Expression: MAPK1 --+> BECN1 10.1016/j.apsb.2019.10.003
positive Expression: MAPK1 --+> BECN1 10.1016/j.bbrc.2014.11.038
positive Expression: MAPK1 --+> BECN1 10.1007/978-981-15-0602-4_12
positive Expression: MAPK1 --+> E 19748465 10.1016/j.chom.2009.08.004
positive Expression: MAPK1 --+> E 25934479 10.1016/j.healun.2014.12.008
positive Expression: MAPK1 --+> E 29405820 10.1080/15384047.2018.1436024
positive Expression: MAPK1 --+> E 912 10.1016/j.cytogfr.2018.07.001
positive Expression: MAPK1 --+> E 29620175 10.3892/mmr.2018.8818
positive Expression: MAPK1 --+> E 35588334 10.1007/s00018-022-04319-w
positive Expression: NGF --+> BE(31698410 10.1038/s41390-019-0657-4
negative Expression: AKT1 ---| TF 18948082 10.1016/j.bbrc.2008.10.054
negative Expression: AKT1 ---| TF 29944886 10.1016/j.bbrc.2018.06.127
negative Expression: AKT1 ---| TF 16728701 10.1182/blood-2006-02-001354
negative Expression: AKT1 ---| TF 32315388 10.1182/blood.2019003538
negative Expression: AKT1 ---| TF 26764128 10.1016/j.ydbio.2015.12.027
negative Expression: AKT1 ---| TF 32949604 10.1016/j.ejphar.2020.173580
negative Expression: AKT1 ---| TF 33345853 10.1016/j.ejphar.2020.173796
negative Expression: AKT1 ---| TF 33662352 10.1016/j.ejphar.2021.173957
negative Expression: AKT1 ---| TF 32561481 10.1016/j.exer.2020.108116
negative Expression: AKT1 ---| TF 26878368 10.1016/j.atherosclerosis.2016.02.002
negative Expression: AKT1 ---| TF 11054421 10.1074/jbc.M008552200
negative Expression: AKT1 ---| TF 14559897 10.1074/jbc.M310208200
negative Expression: AKT1 ---| TF 19376776 10.1074/jbc.M109.002105
negative Expression: AKT1 ---| TF 19376776 10.1074/jbc.M109.002105
negative Expression: AKT1 ---| TF 14701854 10.1074/jbc.M312333200
negative Expression: AKT1 ---| TF 19605357 10.1074/jbc.M109.034629
negative Expression: AKT1 ---| TF 25210032 10.1074/jbc.M114.565796
negative Expression: AKT1 ---| TF 25210032 10.1074/jbc.M114.565796
negative Expression: AKT1 ---| TF 22334668 10.1074/jbc.M111.333922
negative Expression: AKT1 ---| TF 22334668 10.1074/jbc.M111.333922
negative Expression: AKT1 ---| TF 22334668 10.1074/jbc.M111.333922
negative Expression: AKT1 ---| TF 21778238 10.1074/jbc.M111.267633
negative Expression: AKT1 ---| TF 21778238 10.1074/jbc.M111.267633
negative Expression: AKT1 ---| TF 15799971 10.1074/jbc.M414464200
negative Expression: AKT1 ---| TF 15805113 10.1074/jbc.M414091200
negative Expression: AKT1 ---| TF 15805113 10.1074/jbc.M414091200
negative Expression: AKT1 ---| TF 18467333 10.1074/jbc.M709695200
negative Expression: AKT1 ---| TF 27373978 10.1016/j.jid.2016.06.610
negative Expression: AKT1 ---| TF 32931807 10.1016/j.jid.2020.08.019
negative Expression: AKT1 ---| TF 28202289 10.1016/j.lfs.2017.02.005
negative Expression: AKT1 ---| TF 31678282 10.1016/j.lfs.2019.116880
negative Expression: AKT1 ---| TF 33121990 10.1016/j.lfs.2020.118672
negative Expression: AKT1 ---| TF 28844836 10.1016/j.bbamcr.2017.08.008
negative Expression: AKT1 ---| TF 24801417 10.1016/j.jhep.2014.04.035
negative Expression: AKT1 ---| TF 24801417 10.1016/j.jhep.2014.04.035

negative Expression: AKT1 ---| TF32222662 10.1016/j.ejmech.2020.112207
negative Expression: AKT1 ---| TF31918176 10.1016/j.nutres.2019.12.004
negative Expression: AKT1 ---| TF31150783 10.1016/j.fct.2019.05.041
negative Expression: AKT1 ---| TF32871194 10.1016/j.fct.2020.111714
negative Expression: AKT1 ---| TF33232793 10.1016/j.biochi.2020.11.015
negative Expression: AKT1 ---| TF26902421 10.1016/j.canlet.2016.02.023
negative Expression: AKT1 ---| TF26902421 10.1016/j.canlet.2016.02.023
negative Expression: AKT1 ---| TF26902421 10.1016/j.canlet.2016.02.023
negative Expression: AKT1 ---| TF26902421 10.1016/j.canlet.2016.02.023
negative Expression: AKT1 ---| TF30419311 10.1016/j.bbcan.2018.10.001
negative Expression: AKT1 ---| TF32050108 10.1016/j.ctrv.2020.101975
negative Expression: AKT1 ---| TF27461822 10.1016/j.prp.2016.06.005
negative Expression: AKT1 ---| TF28322991 10.1016/j.gene.2017.03.014
negative Expression: AKT1 ---| TF28922731 10.1016/j.biopha.2017.09.023
negative Expression: AKT1 ---| TF29324311 10.1016/j.biopha.2018.01.030
negative Expression: AKT1 ---| TF29635125 10.1016/j.biopha.2018.03.126
negative Expression: AKT1 ---| TF29635125 10.1016/j.biopha.2018.03.126
negative Expression: AKT1 ---| TF29635125 10.1016/j.biopha.2018.03.126
negative Expression: AKT1 ---| TF20943647 10.1093/annonc/mdq366
negative Expression: AKT1 ---| TF31323288 10.1016/j.semcancer.2019.07.009
negative Expression: AKT1 ---| TF27818144 10.1016/j.molcel.2016.10.002
negative Expression: AKT1 ---| TF32442683 10.1016/j.micinf.2020.04.010
negative Expression: AKT1 ---| TP53 10.1016/j.procbio.2018.12.010
negative Expression: AKT1 ---| TF28993289 10.1016/j.arr.2017.10.001
negative Expression: AKT1 ---| TF32846356 10.1016/j.dnarep.2020.102952
negative Expression: AKT1 ---| TF26522297 10.1016/j.apjtm.2015.09.008
negative Expression: AKT1 ---| TF33869685 10.1016/j.reth.2021.02.002
negative Expression: AKT1 ---| TF28844411 10.1016/j.ebiom.2017.08.018
negative Expression: AKT1 ---| TF20079716 10.1016/j.bbrc.2010.01.038
negative Expression: AKT1 ---| TF20079716 10.1016/j.bbrc.2010.01.038
negative Expression: AKT1 ---| TF20079716 10.1016/j.bbrc.2010.01.038
negative Expression: AKT1 ---| TF14988039 10.1016/j.brainres.2003.12.021
negative Expression: AKT1 ---| TF20096282 10.1016/j.ejphar.2010.01.004
negative Expression: AKT1 ---| TF21930127 10.1016/j.yexcr.2011.08.013
negative Expression: AKT1 ---| TF16616141 10.1016/j.febslet.2006.03.055
negative Expression: AKT1 ---| TF18325394 10.1053/j.gastro.2008.01.079
negative Expression: AKT1 ---| TF21723221 10.1053/j.gastro.2011.06.056
negative Expression: AKT1 ---| TF17306348 10.1016/j.ygyno.2006.12.030
negative Expression: AKT1 ---| TF18485879 10.1016/j.cell.2008.03.021
negative Expression: AKT1 ---| TF14613025 10.1053/j.seminoncol.2003.08.006
negative Expression: AKT1 ---| TF12085086 10.1067/mcn.2002.125874
negative Expression: AKT1 ---| TF21440011 10.1016/j.bbamcr.2011.03.010
negative Expression: AKT1 ---| TF20691487 10.1016/j.tibtech.2010.06.005
negative Expression: AKT1 ---| TF17337084 10.1016/j.lungcan.2007.01.027
negative Expression: AKT1 ---| TF19457607 10.1016/j.canlet.2009.04.035
negative Expression: AKT1 ---| TF19457607 10.1016/j.canlet.2009.04.035
negative Expression: AKT1 ---| TF22266095 10.1016/j.canlet.2012.01.003
negative Expression: AKT1 ---| TF17293044 10.1016/j.neulet.2006.06.050
negative Expression: AKT1 ---| TF21625365 10.1016/j.foodchem.2011.03.073
negative Expression: AKT1 ---| TF26902421 10.1016/j.canlet.2016.02.023
negative Expression: AKT1 ---| TF18325394 10.1053/j.gastro.2008.01.079
negative Expression: AKT1 ---| TF12941408 10.1016/s1369-5274(03)00085-7
negative Expression: AKT1 ---| TF23375534 10.1016/j.jcms.2012.12.006
negative Expression: AKT1 ---| TF26878368 10.1016/j.atherosclerosis.2016.02.002

negative Expression: AKT1 ---| TF25172547 10.1016/j.imbio.2014.08.010
negative Expression: AKT1 ---| TF22033326 10.1016/j.ejca.2011.09.014
negative Expression: AKT1 ---| TF18174288 10.1210/en.2007-0844
negative Expression: AKT1 ---| TF23137567 10.1016/j.bbalip.2012.10.008
negative Expression: AKT1 ---| TF16697215 10.1016/j.semcancer.2006.03.009
negative Expression: AKT1 ---| TF19539777 10.1016/j.biocel.2009.06.004
negative Expression: AKT1 ---| TF24801417 10.1016/j.jhep.2014.04.035
negative Expression: AKT1 ---| TF26066464 10.1016/j.meegid.2015.06.008
negative Expression: AKT1 ---| TF17416211 10.1016/j.dnarep.2007.02.028
negative Expression: AKT1 ---| TF17306348 10.1016/j.ygyno.2006.12.030
negative Expression: AKT1 ---| TF25466518 10.1016/j.jss.2014.10.042
negative Expression: AKT1 ---| TF22266095 10.1016/j.canlet.2012.01.003
negative Expression: AKT1 ---| TF18485879 10.1016/j.cell.2008.03.021
negative Expression: AKT1 ---| TF897 10.1007/978-1-4684-3261-9_15
negative Expression: AKT1 ---| TF25976245 10.1016/j.ajpath.2015.02.023
negative Expression: AKT1 ---| TF20691487 10.1016/j.tibtech.2010.06.005
negative Expression: AKT1 ---| TF22270165 10.1016/j.ijrobp.2011.08.005
negative Expression: AKT1 ---| TF19457607 10.1016/j.canlet.2009.04.035
negative Expression: AKT1 ---| TF25289770 10.1016/j.cbi.2014.09.023
negative Expression: AKT1 ---| TF21440011 10.1016/j.bbamcr.2011.03.010
negative Expression: AKT1 ---| TF20399660 10.1016/j.tcb.2010.03.004
negative Expression: AKT1 ---| TF21930127 10.1016/j.yexcr.2011.08.013
negative Expression: AKT1 ---| TF22841670 10.1016/j.ejphar.2012.06.045
negative Expression: AKT1 ---| TF12620407 10.1016/s1535-6108(03)00021-7
negative Expression: AKT1 ---| TF25217104 10.1016/j.molonc.2014.08.005
negative Expression: AKT1 ---| TF26764128 10.1016/j.ydbio.2015.12.027
negative Expression: AKT1 ---| TF25617127 10.1016/j.biomaterials.2014.12.023
negative Expression: AKT1 ---| TF26033200 10.1016/j.pbiomolbio.2015.05.004
negative Expression: AKT1 ---| TF27373978 10.1016/j.jid.2016.06.610
negative Expression: AKT1 ---| TF17293044 10.1016/j.neulet.2006.06.050
negative Expression: AKT1 ---| TF11983168 10.1016/s1097-2765(02)00504-x
negative Expression: AKT1 ---| TF26522297 10.1016/j.apjtm.2015.09.008
negative Expression: AKT1 ---| TF14988039 10.1016/j.brainres.2003.12.021
negative Expression: AKT1 ---| TF21625365 10.1016/j.foodchem.2011.03.073
negative Expression: AKT1 ---| TF24607498 10.1016/j.biocel.2014.02.020
negative Expression: AKT1 ---| TF27208405 10.1016/j.placenta.2016.03.001
negative Expression: AKT1 ---| TF17337084 10.1016/j.lungcan.2007.01.027
negative Expression: AKT1 ---| TF24998604 10.1016/j.bbamcr.2014.06.017
negative Expression: AKT1 ---| TF16616141 10.1016/j.febslet.2006.03.055
negative Expression: AKT1 ---| TF17336087 10.1016/j.critrevonc.2007.02.001
negative Expression: AKT1 ---| TF12049739 10.1016/s1097-2765(02)00520-8
negative Expression: AKT1 ---| TF27461822 10.1016/j.prp.2016.06.005
negative Expression: AKT1 ---| TF16527552 10.1016/j.dld.2006.01.012
negative Expression: AKT1 ---| TF23142241 10.1016/j.lfs.2012.10.023
negative Expression: AKT1 ---| TF16026734 10.1016/j.beha.2005.01.003
negative Expression: AKT1 ---| TF24801546 10.1016/j.neuint.2014.04.016
negative Expression: AKT1 ---| TF20096282 10.1016/j.ejphar.2010.01.004
negative Expression: AKT1 ---| TF1050 10.1016/j.ebiom.2017.08.018
negative Expression: AKT1 ---| TF21723221 10.1053/j.gastro.2011.06.056
negative Expression: AKT1 ---| TP53 10.1016/j.ejca.2011.09.014
negative Expression: AKT1 ---| TP53 10.1016/j.jss.2014.10.042
negative Expression: AKT1 ---| TP53 10.1016/j.ijrobp.2011.08.005
negative Expression: AKT1 ---| TP53 10.1016/j.semcdb.2004.01.002
negative Expression: AKT1 ---| TP53 10.1016/j.bbrc.2008.10.054

negative Expression: AKT1 ---| TP53 10.1053/j.seminoncol.2003.08.006
negative Expression: AKT1 ---| TP53 10.1016/j.foodchem.2011.03.073
negative Expression: AKT1 ---| TP53 10.1016/j.jbiotec.2014.07.331
negative Expression: AKT1 ---| TP53 10.1016/j.path.2010.04.001
negative Expression: AKT1 ---| TP53 10.1016/j.ejca.2005.02.011
negative Expression: AKT1 ---| TP53 10.1067/mcn.2002.125874
negative Expression: AKT1 ---| TP53 10.1016/S1535-6108(03)00248-4
negative Expression: AKT1 ---| TP53 10.1016/S0959-437X(02)00274-5
negative Expression: AKT1 ---| TP53 10.1016/S1097-2765(02)00776-1
negative Expression: AKT1 ---| TF34445509 10.3390/ijms22168805
negative Expression: AKT1 ---| TF34417460 10.1038/s41467-021-25390-0
negative Expression: AKT1 ---| TF21040738 10.1016/j.mrfmmm.2010.10.00710.1016/j.mrfmmm.
negative Expression: AKT1 ---| TF23238568 10.1038/cdd.2012.15310.1038/cdd.2012.153
negative Expression: AKT1 ---| TF23843462 10.1074/jbc.M112.44514810.1074/jbc.M112.44514
negative Expression: AKT1 ---| TF24525337 10.18632/oncotarget.1328
negative Expression: AKT1 ---| TF24525337 10.18632/oncotarget.1328
negative Expression: AKT1 ---| TF32404543 10.1248/bpb.b20-00094
negative Expression: AKT1 ---| TF897 10.1016/j.molcel.2016.10.002
negative Expression: AKT1 ---| TF1050 10.1016/j.ebiom.2017.08.018
negative Expression: AKT1 ---| TP53 10.1016/j.semcancer.2019.07.009
negative Expression: AKT1 ---| TF20399660 10.1016/j.tcb.2010.03.004
negative Expression: AKT1 ---| TF17336087 10.1016/j.critrevonc.2007.02.001
negative Expression: AKT1 ---| TF16697215 10.1016/j.semcancer.2006.03.009
negative Expression: AKT1 ---| TF11983168 10.1016/S1097-2765(02)00504-X
negative Expression: AKT1 ---| TF12049739 10.1016/S1097-2765(02)00520-8
negative Expression: AKT1 ---| TF19539777 10.1016/j.biocel.2009.06.004
negative Expression: AKT1 ---| TF12941408 10.1016/S1369-5274(03)00085-7
negative Expression: AKT1 ---| TF16026734 10.1016/j.beha.2005.01.003
negative Expression: AKT1 ---| TF12620407 10.1016/S1535-6108(03)00021-7
negative Expression: AKT1 ---| TF12620407 10.1016/S1535-6108(03)00021-7
negative Expression: AKT1 ---| TF12620407 10.1016/S1535-6108(03)00021-7
negative Expression: AKT1 ---| TF17416211 10.1016/j.dnarep.2007.02.028
negative Expression: AKT1 ---| TF16527552 10.1016/j.dld.2006.01.012
negative Expression: AKT1 ---| TF22841670 10.1016/j.ejphar.2012.06.045
negative Expression: AKT1 ---| TF22270165 10.1016/j.ijrobp.2011.08.005
negative Expression: AKT1 ---| TF22033326 10.1016/j.ejca.2011.09.014
negative Expression: AKT1 ---| TF23142241 10.1016/j.lfs.2012.10.023
negative Expression: AKT1 ---| TF23375534 10.1016/j.jcms.2012.12.006
negative Expression: AKT1 ---| TF23137567 10.1016/j.bbalip.2012.10.008
negative Expression: AKT1 ---| TF24607498 10.1016/j.biocel.2014.02.020
negative Expression: AKT1 ---| TF25289770 10.1016/j.cbi.2014.09.023
negative Expression: AKT1 ---| TF24998604 10.1016/j.bbamcr.2014.06.017
negative Expression: AKT1 ---| TF25172547 10.1016/j.imbio.2014.08.010
negative Expression: AKT1 ---| TF24801546 10.1016/j.neuint.2014.04.016
negative Expression: AKT1 ---| TF25217104 10.1016/j.molonc.2014.08.005
negative Expression: AKT1 ---| TF25976245 10.1016/j.ajpath.2015.02.023
negative Expression: AKT1 ---| TF25466518 10.1016/j.jss.2014.10.042
negative Expression: AKT1 ---| TF25617127 10.1016/j.biomaterials.2014.12.023
negative Expression: AKT1 ---| TF26066464 10.1016/j.meegid.2015.06.008
negative Expression: AKT1 ---| TF26066464 10.1016/j.meegid.2015.06.008
negative Expression: AKT1 ---| TF27208405 10.1016/j.placenta.2016.03.001
negative Expression: AKT1 ---| TP53 10.1042/BCJ20190888
negative Expression: AKT1 ---| TF32855630 10.1155/2020/3608315
negative Expression: AKT1 ---| TF27746632 10.5021/ad.2016.28.5.548

negative Expression: AKT1 ---| TF30697266 10.5114/aoms.2018.7402610.5114/aoms.2018.7402
negative Expression: AKT1 ---| TF31086728 10.7717/peerj.671610.7717/peerj.6716
negative Expression: AKT1 ---| TF32581555 10.2147/OTT.S24670610.2147/OTT.S246706
negative Expression: AKT1 ---| TF26033200 10.1016/j.pbiomolbio.2015.05.004
negative Expression: AKT1 ---| TF18174288 10.1210/en.2007-0844
negative Expression: AKT1 ---| TF27629785 10.1007/s11010-016-2809-8
negative Expression: AKT1 ---| TF27560518 10.1371/journal.pone.0161508
negative Expression: AKT1 ---| TF25144704 10.1371/journal.pntd.0003106
negative Expression: AKT1 ---| TF25144704 10.1371/journal.pntd.0003106
negative Expression: AKT1 ---| TF16540659
negative Expression: AKT1 ---| TF16330492
negative Expression: AKT1 ---| TF24213576 10.1038/onc.2013.477
negative Expression: AKT1 ---| TF11923280
negative Expression: AKT1 ---| TF17684018
negative Expression: AKT1 ---| TF22975685 10.1016/j.cellsig.2012.09.009
negative Expression: AKT1 ---| TF15862750
negative Expression: AKT1 ---| TF11715018
negative Expression: AKT1 ---| TF25301184 10.1152/ajpgi.00151.2014
negative Expression: AKT1 ---| TF19376776 10.1074/jbc.M109.002105
negative Expression: AKT1 ---| TF22395446 10.1007/s10495-012-0712-7
negative Expression: AKT1 ---| TF24149576 10.1038/ncomms3544
negative Expression: AKT1 ---| TF17725107
negative Expression: AKT1 ---| TF22417301 10.1111/j.1600-0625.2012.01448.x
negative Expression: AKT1 ---| TF17709487
negative Expression: AKT1 ---| TF25109960 10.1042/BST20140070
negative Expression: AKT1 ---| TP53 10.1016/j.apsb.2012.05.003
negative Expression: AKT1 ---| TF23462616 10.1016/j.freeradbiomed.2013.02.009
negative Expression: AKT1 ---| TF12738763
negative Expression: AKT1 ---| TF12738763
negative Expression: AKT1 ---| TF19376776
negative Expression: AKT1 ---| TF19376776
negative Expression: AKT1 ---| TF19376776
negative Expression: AKT1 ---| TF18467333
negative Expression: AKT1 ---| TF17684018
negative Expression: AKT1 ---| TF15805113
negative Expression: AKT1 ---| TF15799971
negative Expression: AKT1 ---| TF15746177
negative Expression: AKT1 ---| TF14701854
negative Expression: AKT1 ---| TF14580260
negative Expression: AKT1 ---| TF12517744
negative Expression: AKT1 ---| TF12517744
negative Expression: AKT1 ---| TF12042314
negative Expression: AKT1 ---| TF11923280
negative Expression: AKT1 ---| TF11923280
negative Expression: AKT1 ---| TF11923280
negative Expression: AKT1 ---| TF11923280
negative Expression: AKT1 ---| TF11923280
negative Expression: AKT1 ---| TF11923280
negative Expression: AKT1 ---| TF11923280
negative Expression: AKT1 ---| TF11923280
negative Expression: AKT1 ---| TF11923280
negative Expression: AKT1 ---| TF11923280
negative Expression: AKT1 ---| TF17122210
negative Expression: AKT1 ---| TF14670833

negative Expression: AKT1 ---| TF11504915
negative Expression: AKT1 ---| TF11504915
negative Expression: AKT1 ---| TF16007156
negative Expression: AKT1 ---| TF15933740
negative Expression: AKT1 ---| TF15150572
negative Expression: AKT1 ---| TF14713953
negative Expression: AKT1 ---| TF16982679
negative Expression: AKT1 ---| TF20676365
negative Expression: AKT1 ---| TF20436671
negative Expression: AKT1 ---| TF22641480 10.3892/or.2012.1827
negative Expression: AKT1 ---| TF23095642 10.4161/cc.22413
negative Expression: AKT1 ---| TF22020565 10.1007/s00394-011-0260-x
negative Expression: AKT1 ---| TF23390564
negative Expression: AKT1 ---| TF23267122
negative Expression: AKT1 ---| TF20491628 10.2174/138920310791824039
negative Expression: AKT1 ---| TF21460185 10.1091/mbc.E10-11-0923
negative Expression: AKT1 ---| TF21460185 10.1091/mbc.E10-11-0923
negative Expression: AKT1 ---| TF21460185 10.1091/mbc.E10-11-0923
negative Expression: AKT1 ---| TF21460185 10.1091/mbc.E10-11-0923
negative Expression: AKT1 ---| TF21460185 10.1091/mbc.E10-11-0923
negative Expression: AKT1 ---| TF21235352 10.1089/ars.2010.3540
negative Expression: AKT1 ---| TF21778238 10.1074/jbc.M111.267633
negative Expression: AKT1 ---| TF21990374 10.1096/fj.11-190025
negative Expression: AKT1 ---| TF22334668 10.1074/jbc.M111.333922
negative Expression: AKT1 ---| TF22334668 10.1074/jbc.M111.333922
negative Expression: AKT1 ---| TF22334668 10.1074/jbc.M111.333922
negative Expression: AKT1 ---| TF22334668 10.1074/jbc.M111.333922
negative Expression: AKT1 ---| TF22334668 10.1074/jbc.M111.333922
negative Expression: AKT1 ---| TF22334668 10.1074/jbc.M111.333922
negative Expression: AKT1 ---| TF22481935 10.1155/2012/951724
negative Expression: AKT1 ---| TF23202921 10.3390/ijms131012710
negative Expression: AKT1 ---| TF19728186 10.1007/s12032-009-9295-8
negative Expression: AKT1 ---| TF21870170 10.1007/s12020-011-9524-8
negative Expression: AKT1 ---| TF22157439 10.1097/MOG.0b013e32834dda51
negative Expression: AKT1 ---| TF22611192 10.1073/pnas.1110287109
negative Expression: AKT1 ---| TF23530877 10.1186/1475-2840-12-52
negative Expression: AKT1 ---| TF23410153 10.2174/09298673113209990106
negative Expression: AKT1 ---| TF23693014 10.1186/1476-4598-12-44
negative Expression: AKT1 ---| TF23693014 10.1186/1476-4598-12-44
negative Expression: AKT1 ---| TF23693014 10.1186/1476-4598-12-44
negative Expression: AKT1 ---| TF23693014 10.1186/1476-4598-12-44
negative Expression: AKT1 ---| TP53 10.1186/1743-422X-10-182
negative Expression: AKT1 ---| TF20489726 10.1038/cdd.2010.63
negative Expression: AKT1 ---| TF22735455 10.1038/ncomms1933
negative Expression: AKT1 ---| TF23843462 10.1074/jbc.M112.445148
negative Expression: AKT1 ---| TF21685937 10.1038/onc.2011.198
negative Expression: AKT1 ---| TF21743495 10.1038/onc.2011.284
negative Expression: AKT1 ---| TF21909130 10.1038/onc.2011.394
negative Expression: AKT1 ---| TF21909130 10.1038/onc.2011.394
negative Expression: AKT1 ---| TF22510990 10.1038/embor.2012.53
negative Expression: AKT1 ---| TF23470565 10.1038/cgt.2013.14
negative Expression: AKT1 ---| TF23787999 10.1038/cdd.2013.63
negative Expression: AKT1 ---| TF20676365 10.1371/journal.pone.0011813
negative Expression: AKT1 ---| TF20386595 10.1371/journal.pone.0009999

negative Expression: AKT1 ---| TF20436671 10.1371/journal.pone.0010350
negative Expression: AKT1 ---| TF22928011 10.1371/journal.pone.0043646
negative Expression: AKT1 ---| TF23285096 10.1371/journal.pone.0052572
negative Expression: AKT1 ---| TF23114628 10.1099/vir.0.046052-0
negative Expression: AKT1 ---| TF23114628 10.1099/vir.0.046052-0
negative Expression: AKT1 ---| TF23638184 10.1371/journal.pone.0063179
negative Expression: AKT1 ---| TF24149576 10.1038/ncomms3544
negative Expression: AKT1 ---| TF24149576 10.1038/ncomms3544
negative Expression: AKT1 ---| TF24149576 10.1038/ncomms3544
negative Expression: AKT1 ---| TF24149576 10.1038/ncomms3544
negative Expression: AKT1 ---| TF24212374 10.1247/csf.13018
negative Expression: AKT1 ---| TF22296497 10.4149/neo_2012_035
negative Expression: AKT1 ---| TF23396362 10.1038/onc.2013.8
negative Expression: AKT1 ---| TF23396362 10.1038/onc.2013.8
negative Expression: AKT1 ---| TF23396362 10.1038/onc.2013.8
negative Expression: AKT1 ---| TF24479877 10.1210/me.2013-1266
negative Expression: AKT1 ---| TF23394468 10.3727/096368913X663578
negative Expression: AKT1 ---| TP53
negative Expression: AKT1 ---| TF24292837 10.1007/978-3-642-41199-1-16
negative Expression: AKT1 ---| TF24603851 10.3892/ijo.2014.2322
negative Expression: AKT1 ---| TF24762959 10.1038/bjc.2014.220
negative Expression: AKT1 ---| TF24762959 10.1038/bjc.2014.220
negative Expression: AKT1 ---| TF24762959 10.1038/bjc.2014.220
negative Expression: AKT1 ---| TF24971451 10.1371/journal.pone.0101277
negative Expression: AKT1 ---| TF24929707 10.1186/1471-2407-14-440
negative Expression: AKT1 ---| TF24096477 10.1038/onc.2013.410
negative Expression: AKT1 ---| TP53 10.1074/jbc.M114.565796
negative Expression: AKT1 ---| TP53 10.1074/jbc.M114.565796
negative Expression: AKT1 ---| TF25117830 10.1089/scd.2014.0058
negative Expression: AKT1 ---| TF25142166 10.1007/s11010-014-2198-9
negative Expression: AKT1 ---| TF25501204 10.4238/2014.November.27.22
negative Expression: AKT1 ---| TF25667479
negative Expression: AKT1 ---| TF25301184 10.1152/ajpgi.00151.2014
negative Expression: AKT1 ---| TF21372320 10.1128/MMBR.00031-10
negative Expression: AKT1 ---| TF21357737 10.1128/MCB.01321-10
negative Expression: AKT1 ---| TF21742795 10.1152/physrev.00024.2010
negative Expression: AKT1 ---| TF21791616 10.1128/MCB.05744-11
negative Expression: AKT1 ---| TF25738369 10.3892/mmr.2015.3393
negative Expression: AKT1 ---| TF25713322 10.1152/ajplung.00334.2014
negative Expression: AKT1 ---| TF25932965 10.1371/journal.pone.0125903
negative Expression: AKT1 ---| TF25932965 10.1371/journal.pone.0125903
negative Expression: AKT1 ---| TP53 10.3727/096504015X14298122915583
negative Expression: AKT1 ---| TP53 10.3727/096504015X14298122915583
negative Expression: AKT1 ---| TF11923280
negative Expression: AKT1 ---| TF19376776 10.1074/jbc.M109.002105
negative Expression: AKT1 ---| TF26459696 10.3892/mmr.2015.4381
negative Expression: AKT1 ---| TF25482935 10.4161/15384047.2014.961876
negative Expression: AKT1 ---| TF26902421 10.1016/j.canlet.2016.02.023
negative Expression: AKT1 ---| TP53
negative Expression: AKT1 ---| TP53
negative Expression: AKT1 ---| TP53 10.1172/JCI80396
negative Expression: AKT1 ---| TF26433377 10.1007/s12035-015-9445-2
negative Expression: AKT1 ---| TF26433377 10.1007/s12035-015-9445-2
negative Expression: AKT1 ---| TF24292837 10.1007/978-3-642-41199-1_16

negative Expression: AKT1 ---| TP53 10.2174/1381612822666161006160524
negative Expression: AKT1 ---| TF28107044 10.1080/01635581.2017.1267775
negative Expression: AKT1 ---| TF28484034 10.1073/pnas.1702412114
negative Expression: AKT1 ---| TF27837015 10.1177/0022034516678203
negative Expression: AKT1 ---| TF28635633 10.3390/ijms18061325
negative Expression: AKT1 ---| TF28635633 10.3390/ijms18061325
negative Expression: AKT1 ---| TF28691365 10.1111/acel.1263910.1111/acel.12639
negative Expression: AKT1 ---| TF28401302 10.1007/s00432-017-2415-5
negative Expression: AKT1 ---| TF28553961 10.1038/nsmb.3414
negative Expression: AKT1 ---| TF28938844 10.1089/ars.2017.699010.1089/ars.2017.6990
negative Expression: AKT1 ---| TF29048667 10.3892/ijo.2017.4145
negative Expression: AKT1 ---| TF29048667 10.3892/ijo.2017.4145
negative Expression: AKT1 ---| TF29048640 10.3892/or.2017.6000
negative Expression: AKT1 ---| TF29139175 10.1002/stem.274110.1002/stem.2741
negative Expression: AKT1 ---| TF28696156 10.1080/15384047.2017.1345397
negative Expression: AKT1 ---| TF28853983 10.1080/15384047.2017.1360446
negative Expression: AKT1 ---| TF29297700 10.1080/01635581.2018.1412486
negative Expression: AKT1 ---| TF29209894 10.1007/s10863-017-9737-5
negative Expression: AKT1 ---| TF29596872 10.1016/j.cellsig.2018.03.01310.1016/j.cellsi
negative Expression: AKT1 ---| TF29436685 10.3892/or.2018.6256
negative Expression: AKT1 ---| TF28938844 10.1089/ars.2017.6990
negative Expression: AKT1 ---| TF28938844 10.1089/ars.2017.6990
negative Expression: AKT1 ---| TP53 10.1161/ATVBAHA.117.309378
negative Expression: AKT1 ---| TF28681127 10.1007/s12011-017-1088-x
negative Expression: AKT1 ---| TP53 10.1096/fj.201700570RR
negative Expression: AKT1 ---| TF29739064 10.7754/Clin.Lab.2018.171129
negative Expression: AKT1 ---| TF30011966 10.3390/ijms19072060
negative Expression: AKT1 ---| TF29926229 10.1007/s00424-018-2162-8
negative Expression: AKT1 ---| TF29926229 10.1007/s00424-018-2162-8
negative Expression: AKT1 ---| TF29873596 10.1152/physrev.00038.2017
negative Expression: AKT1 ---| TF30208932 10.1186/s13058-018-1028-5
negative Expression: AKT1 ---| TF30106128 10.3892/ijmm.2018.3825
negative Expression: AKT1 ---| TF30226561 10.3892/or.2018.6703
negative Expression: AKT1 ---| TP53 10.3892/or.2018.6759
negative Expression: AKT1 ---| TF30181579 10.1038/s41401-018-0034-6
negative Expression: AKT1 ---| TF30349992 10.1007/s00018-018-2949-y
negative Expression: AKT1 ---| TF30132214 10.1007/s11010-018-3422-9
negative Expression: AKT1 ---| TF30251117 10.1007/s11010-018-3448-z
negative Expression: AKT1 ---| TP53 10.3892/or.2019.7041
negative Expression: AKT1 ---| TP53 10.1186/s12885-019-5475-x
negative Expression: AKT1 ---| TP53 10.1038/s41388-018-0648-7
negative Expression: AKT1 ---| TP53 10.1091/mbc.E18-12-0770
negative Expression: AKT1 ---| TF31592121 10.15171/apb.2019.05610.15171/apb.2019.056
negative Expression: AKT1 ---| TP53 10.12659/MSM.916461
negative Expression: AKT1 ---| TF32148649 10.1155/2020/404870610.1155/2020/4048706
negative Expression: AKT1 ---| TP53 10.1042/BSR20181881
negative Expression: AKT1 ---| TP53 10.1038/s41418-019-0384-8
negative Expression: AKT1 ---| TP53 10.3390/ijms21031060
negative Expression: AKT1 ---| TP53 10.1242/JCS.230029
negative Expression: AKT1 ---| TP53 10.1080/15476286.2019.1705610
negative Expression: AKT1 ---| TF33468664 10.1073/pnas.2003193118
negative Expression: AKT1 ---| TF33562110 10.3390/ijms22041653
negative Expression: AKT1 ---| TF33111210 10.1007/s11010-020-03951-z
negative Expression: AKT1 ---| TF33542215 10.1038/s41419-021-03419-y

negative Expression: AKT1 ---| TF 32931807 10.1016/j.jid.2020.08.019
negative Expression: AKT1 ---| TF 34065345 10.3390/ijms22105377
negative Expression: AKT1 ---| TF 34109989 10.3892/or.2021.8104
negative Expression: AKT1 ---| TF 34209135 10.3390/ijms22137064
negative Expression: AKT1 ---| TF 34511432 10.18632/aging.203499
negative Expression: AKT1 ---| TF 34681745 10.3390/ijms222011088
negative Expression: AKT1 ---| TF 34759291 10.1038/s41419-021-04371-7
negative Expression: AKT1 ---| TF 34948338 10.3390/ijms222413543
negative Expression: AKT1 ---| TF 35216283 10.3390/ijms23042168
negative Expression: AKT1 ---| TP53 10.1016/j.gendis.2021.12.025
negative Expression: AKT1 ---| TF 35031925 10.1007/s11033-021-07063-9
negative Expression: AKT1 ---| TF 35305553 10.1186/s11658-022-00324-w
negative Expression: AKT1 ---| TF 35431190 10.3233/TUB-211538
negative Expression: AKT1 ---| TF 35414774 10.7150/ijbs.67200
negative Expression: AKT1 ---| TF 35414774 10.7150/ijbs.67200
negative Expression: AKT1 ---| TF 34328200 10.3892/or.2021.8159
negative Expression: AKT1 ---| TP53 10.1016/j.apsb.2022.06.004
negative Expression: AKT1 ---| TF 35725771 10.1038/s41375-022-01627-9
negative Expression: AKT1 ---| TF 36012464 10.3390/ijms23169196
negative Expression: AKT1 ---| TF 35094248 10.1007/s11064-021-03516-6
negative Expression: AKT1 ---| TF 35094248 10.1007/s11064-021-03516-6
negative Expression: AKT1 ---| TF 36180910 10.1186/s12929-022-00860-9
negative Expression: AKT1 ---| TF 36232606 10.3390/ijms231911300
negative Expression: AKT1 ---| TF 36221123 10.1186/s12943-022-01664-z
negative Expression: AKT1 ---| TF 35792922 10.1080/15384101.2022.2094577
negative Expression: AKT1 ---| TF 34951363 10.2174/1566524022666211222161637
ProtModification: AKT1 ---> TP53 28359723 10.1016/j.cbi.2017.03.015
ProtModification: AKT1 ---> TP53 11054421 10.1074/jbc.M008552200
ProtModification: AKT1 ---> TP53 27518560 10.1016/j.cell.2016.07.031
ProtModification: AKT1 ---> TP53 15809051 10.1016/j.bbrc.2005.03.044
ProtModification: AKT1 ---> TP53 16956580 10.1016/j.bbrc.2006.08.120
ProtModification: AKT1 ---> TP53 19147016 10.1016/j.ijrobp.2008.09.036
ProtModification: AKT1 ---> TP53 21406225 10.1016/j.cellsig.2011.03.009
ProtModification: AKT1 ---> TP53 10.1016/S0959-437X(02)00274-5
ProtModification: AKT1 ---> TP53 15574337 10.1016/j.molcel.2004.11.003
ProtModification: AKT1 ---> TP53 27208405 10.1016/j.placenta.2016.03.001
ProtModification: AKT1 ---> TP53 16452501
ProtModification: AKT1 ---> TP53 20200230
ProtModification: AKT1 ---> TP53 11960368
ProtModification: AKT1 ---> TP53 16982679
ProtModification: AKT1 ---> TP53 28696156 10.1080/15384047.2017.1345397
ProtModification: AKT1 ---> TP53 29895969 10.1038/s41388-018-0333-x
ProtModification: AKT1 ---> TP53 30865562 10.1091/mbc.E18-12-0770
ProtModification: AKT1 ---> TP53 21455568 10.3892/ijo.2011.984
ProtModification: AKT1 ---> TP53 22316005 10.1089/ars.2012.4529
ProtModification: AKT1 ---> TP53 20157733 10.1007/s00432-010-0779-x
ProtModification: AKT1 ---> TP53 20820992 10.1007/s00134-010-2023-1
ProtModification: AKT1 ---> TP53 21562588 10.1038/cddis.2011.39
ProtModification: AKT1 ---> TP53 24285894 10.1523/JNEUROSCI.3293-13.2013
ProtModification: AKT1 ---> TP53 26096928 10.1038/onc.2015.228
ProtModification: AKT1 ---> TP53 34645107 10.31083/j.jin2003078
ProtModification: AKT1 ---> TP53 10.1016/j.bbrc.2005.03.044
ProtModification: AKT1 ---> TP53 10.1091/mbc.E18-12-0770
ProtModification: AKT1 ---> TP53 10.1523/JNEUROSCI.3293-13.2013

ProtModification: AKT1 ---> TP53 14967490 10.1016/j.virol.2003.11.008
ProtModification: AKT1 ---> TP53 20036733 10.1016/j.freeradbiomed.2009.12.014
ProtModification: AKT1 ---> TP53 25942376 10.1080/15384101.2015.1041693
positive Expression: NOTCH1 --+> 27770236 10.1007/s10565-016-9364-7
positive Expression: NOTCH1 --+> 27770236 10.1007/s10565-016-9364-7
positive Expression: NOTCH1 --+> 27770236 10.1007/s10565-016-9364-7
positive Expression: NOTCH1 --+> 30042932 10.3389/fcimb.2018.0024110.3389/fcimb.2018.00
positive Expression: NOTCH1 --+> 36266281 10.1038/s41467-022-33739-2
positive Expression: NOTCH1 --+> 36266281 10.1038/s41467-022-33739-2
positive Expression: NOTCH1 --+> 36266281 10.1038/s41467-022-33739-2
positive Expression: NOTCH1 --+> 36266281 10.1038/s41467-022-33739-2
positive Expression: NOTCH1 --+> 36266281 10.1038/s41467-022-33739-2
positive Expression: NOTCH1 --+> 36266281 10.1038/s41467-022-33739-2
positive Expression: NOTCH1 --+> 36266281 10.1038/s41467-022-33739-2
positive Expression: NOTCH1 --+> 36266281 10.1038/s41467-022-33739-2
negative miRNAEffect: MIR107 ---|28393193 10.3892/or.2017.5546
negative miRNAEffect: MIR107 ---|28393193 10.3892/or.2017.5546
negative miRNAEffect: MIR107 ---|28393193 10.3892/or.2017.5546
negative miRNAEffect: MIR107 ---|30569100 10.3892/or.2018.6936
negative miRNAEffect: MIR107 ---|25386925 10.1371/journal.pone.0111860
negative miRNAEffect: MIR107 ---|25386925 10.1371/journal.pone.0111860
negative miRNAEffect: MIR107 ---|25386925 10.1371/journal.pone.0111860
negative miRNAEffect: MIR107 ---|25386925 10.1371/journal.pone.0111860
negative miRNAEffect: MIR107 ---|25386925 10.1371/journal.pone.0111860
negative miRNAEffect: MIR107 ---|25386925 10.1371/journal.pone.011186010.1371/journal.p
negative miRNAEffect: MIR107 ---|25386925 10.1371/journal.pone.011186010.1371/journal.p
negative miRNAEffect: MIR107 ---| MCL1 10.3892/or.2018.6936
negative miRNAEffect: MIR107 ---| MCL1 10.1615/JEnvironPatholToxicolOncol.2022041924
positive Regulation: PTGS2 --+> S10844593 10.1046/j.1523-1755.2000.00083.x
positive Regulation: PTGS2 --+> S10844593 10.1046/j.1523-1755.2000.00083.x
positive Regulation: PTGS2 --+> S10844593
positive Regulation: PTGS2 --+> S10844593
positive Regulation: PTGS2 --+> S10844593
positive Regulation: PTGS2 --+> S21266034 10.1186/1756-9966-30-13
positive Regulation: PTGS2 --+> S21266034 10.1186/1756-9966-30-13
positive Regulation: PTGS2 --+> S21266034 10.1186/1756-9966-30-13
positive Regulation: PTGS2 --+> S21266034 10.1186/1756-9966-30-13
positive Regulation: PTGS2 --+> S22355367 10.1371/journal.pone.0031458
positive Regulation: PTGS2 --+> S22355367 10.1371/journal.pone.0031458
positive Regulation: PTGS2 --+> S10844593 10.1046/j.1523-1755.2000.00083.x
positive Regulation: PTGS2 --+> S21266034 10.1186/1756-9966-30-1310.1186/1756-9966-30-1
positive Regulation: PTGS2 --+> SPP1
positive Expression: SPP1 --+> PT24657887 10.1016/j.matbio.2014.03.001
positive Expression: SPP1 --+> PT12933657
positive Expression: SPP1 --+> PT28505114 10.3390/ijms18051058
positive Expression: SPP1 --+> PT28716167 10.2500/ajra.2017.31.4449
positive Expression: SPP1 --+> PT28769537 10.1155/2017/4049098
positive Expression: SPP1 --+> PT29500465 10.1038/s41419-018-0391-6
positive Expression: SPP1 --+> PT28978514 10.1152/ajpregu.00443.2016
positive Expression: SPP1 --+> PT30307407 10.1172/JCI121266
positive Expression: SPP1 --+> PT33740245 10.1007/978-981-33-6785-2_6
positive Expression: SPP1 --+> PT23166604 10.1371/journal.pone.0049014
positive Expression: SPP1 --+> PT23166604 10.1371/journal.pone.0049014
positive Expression: SPP1 --+> PT23728342 10.1038/onc.2013.184

positive Expression: SPP1 --+> P123728342 10.1038/onc.2013.184
positive Expression: SPP1 --+> P123728342 10.1038/onc.2013.184
positive Expression: SPP1 --+> P123728342 10.1038/onc.2013.184
positive Expression: SPP1 --+> P123728342 10.1038/onc.2013.184
positive Expression: SPP1 --+> P123728342 10.1038/onc.2013.184
positive Expression: SPP1 --+> P123728342 10.1038/onc.2013.184
positive Expression: SPP1 --+> P123728342 10.1038/onc.2013.184
positive Expression: SPP1 --+> P123728342 10.1038/onc.2013.184
positive Expression: SPP1 --+> P123728342 10.1038/onc.2013.184
positive Expression: SPP1 --+> P123728342 10.1038/onc.2013.184
positive Expression: SPP1 --+> P123728342 10.1038/onc.2013.184
positive Expression: SPP1 --+> P116818637 10.1158/0008-5472.CAN-06-0661
positive Expression: SPP1 --+> P116818637 10.1158/0008-5472.CAN-06-0661
positive Expression: SPP1 --+> P123728342 10.1038/onc.2013.18410.1038/onc.2013.184
positive Expression: SPP1 --+> P126473949 10.1038/onc.2015.31510.1038/onc.2015.315
positive Expression: SPP1 --+> P132356572 10.1002/pros.2398610.1002/pros.23986
positive Expression: SPP1 --+> PTGS2 10.1172/JCI121266
positive Expression: SPP1 --+> P112933657 10.1210/en.2003-0039
positive Expression: SPP1 --+> P120459645
positive Expression: SPP1 --+> PTGS2
Regulation: TP53 ---> AKT1 26022125 10.1016/j.bbrc.2015.05.069
Regulation: TP53 ---> AKT1 30827507 10.1016/j.bbrc.2019.02.089
Regulation: TP53 ---> AKT1 31733192 10.1016/j.bcp.2019.113713
Regulation: TP53 ---> AKT1 12393587 10.1182/blood-2002-02-0504
Regulation: TP53 ---> AKT1 32687844 10.1016/j.cbi.2020.109196
Regulation: TP53 ---> AKT1 31870856 10.1016/j.yexmp.2019.104362
Regulation: TP53 ---> AKT1 30528266 10.1016/j.yexcr.2018.12.002
Regulation: TP53 ---> AKT1 30528266 10.1016/j.yexcr.2018.12.002
Regulation: TP53 ---> AKT1 30528266 10.1016/j.yexcr.2018.12.002
Regulation: TP53 ---> AKT1 30528266 10.1016/j.yexcr.2018.12.002
Regulation: TP53 ---> AKT1 30528266 10.1016/j.yexcr.2018.12.002
Regulation: TP53 ---> AKT1 20685653 10.1074/jbc.M110.134650
Regulation: TP53 ---> AKT1 20685653 10.1074/jbc.M110.134650
Regulation: TP53 ---> AKT1 29358327 10.1074/jbc.M117.791145
Regulation: TP53 ---> AKT1 24451373 10.1074/jbc.M113.532523
Regulation: TP53 ---> AKT1 24451373 10.1074/jbc.M113.532523
Regulation: TP53 ---> AKT1 24451373 10.1074/jbc.M113.532523
Regulation: TP53 ---> AKT1 24451373 10.1074/jbc.M113.532523
Regulation: TP53 ---> AKT1 19339243 10.1074/jbc.M809757200
Regulation: TP53 ---> AKT1 30473026 10.1016/j.lfs.2018.10.051
Regulation: TP53 ---> AKT1 33333052 10.1016/j.lfs.2020.118814
Regulation: TP53 ---> AKT1 32693135 10.1016/j.ijbiomac.2020.07.137
Regulation: TP53 ---> AKT1 30170033 10.1016/j.dci.2018.08.017
Regulation: TP53 ---> AKT1 26875545 10.1016/j.plipres.2016.02.001
Regulation: TP53 ---> AKT1 30296496 10.1016/j.bbamcr.2018.10.005
Regulation: TP53 ---> AKT1 32738450 10.1016/j.jhep.2020.07.036
Regulation: TP53 ---> AKT1 32738450 10.1016/j.jhep.2020.07.036
Regulation: TP53 ---> AKT1 27600832 10.1016/j.bbcan.2016.09.001
Regulation: TP53 ---> AKT1 31320250 10.1016/j.prp.2019.152531
Regulation: TP53 ---> AKT1 33711551 10.1016/j.biopha.2021.111445
Regulation: TP53 ---> AKT1 24355212 10.1016/j.freeradbiomed.2013.12.010
Regulation: TP53 ---> AKT1 31953216 10.1016/j.bbadis.2020.165684
Regulation: TP53 ---> AKT1 28673691 10.1016/j.mod.2017.06.006
Regulation: TP53 ---> AKT1 33773191 10.1016/j.phymed.2021.153534

Regulation: TP53 ---> AKT1	31843464	10.1016/j.bmc.2019.115235
Regulation: TP53 ---> AKT1	32652464	10.1016/j.jphotobiol.2020.111951
Regulation: TP53 ---> AKT1	30641278	10.1016/j.phrs.2019.01.021
Regulation: TP53 ---> AKT1	30641278	10.1016/j.phrs.2019.01.021
Regulation: TP53 ---> AKT1	33540047	10.1016/j.phrs.2021.105428
Regulation: TP53 ---> AKT1	32502598	10.1016/j.semcancer.2020.05.015
Regulation: TP53 ---> AKT1	33785447	10.1016/j.semcancer.2021.03.010
Regulation: TP53 ---> AKT1	33785447	10.1016/j.semcancer.2021.03.010
Regulation: TP53 ---> AKT1	33785447	10.1016/j.semcancer.2021.03.010
Regulation: TP53 ---> AKT1	30389549	10.1016/j.biocel.2018.10.008
Regulation: TP53 ---> AKT1	31846838	10.1016/j.drup.2019.100670
Regulation: TP53 ---> AKT1	32846356	10.1016/j.dnarep.2020.102952
Regulation: TP53 ---> AKT1	33717651	10.1016/j.omtn.2021.02.005
Regulation: TP53 ---> AKT1	24857662	10.1016/j.celrep.2014.04.046
Regulation: TP53 ---> AKT1	24857662	10.1016/j.celrep.2014.04.046
Regulation: TP53 ---> AKT1	32670818	10.1016/j.jtcme.2020.02.003
Regulation: TP53 ---> AKT1		10.1016/j.gendis.2020.06.001
Regulation: TP53 ---> AKT1	15001399	10.1016/j.abb.2004.01.004
Regulation: TP53 ---> AKT1	15001399	10.1016/j.abb.2004.01.004
Regulation: TP53 ---> AKT1	15001399	10.1016/j.abb.2004.01.004
Regulation: TP53 ---> AKT1	15001399	10.1016/j.abb.2004.01.004
Regulation: TP53 ---> AKT1	12213580	10.1016/S0006-2952(02)01149-8
Regulation: TP53 ---> AKT1	20362557	10.1016/j.bcp.2010.03.031
Regulation: TP53 ---> AKT1	22142888	10.1016/j.bcp.2011.11.022
Regulation: TP53 ---> AKT1	16648169	10.1529/biophysj.105.077693
Regulation: TP53 ---> AKT1	16648169	10.1529/biophysj.105.077693
Regulation: TP53 ---> AKT1	20858413	10.1016/j.bpj.2010.07.042
Regulation: TP53 ---> AKT1	20858413	10.1016/j.bpj.2010.07.042
Regulation: TP53 ---> AKT1	17935714	10.1016/j.yexcr.2007.08.022
Regulation: TP53 ---> AKT1	22192357	10.1016/j.febslet.2011.11.034
Regulation: TP53 ---> AKT1	15001356	10.1016/j.jmb.2003.10.081
Regulation: TP53 ---> AKT1	19203586	10.1016/j.cell.2008.12.002
Regulation: TP53 ---> AKT1	19203586	10.1016/j.cell.2008.12.002
Regulation: TP53 ---> AKT1	19203586	10.1016/j.cell.2008.12.002
Regulation: TP53 ---> AKT1	21377268	10.1016/j.canlet.2011.02.008
Regulation: TP53 ---> AKT1	20951769	10.1016/j.bbcan.2010.10.003
Regulation: TP53 ---> AKT1	21632182	10.1016/j.mehy.2011.05.006
Regulation: TP53 ---> AKT1	15475174	10.1016/j.toxlet.2004.06.013
Regulation: TP53 ---> AKT1	18448277	10.1016/j.toxlet.2008.03.012
Regulation: TP53 ---> AKT1	19840842	10.1016/j.exger.2009.10.009
Regulation: TP53 ---> AKT1	19840842	10.1016/j.exger.2009.10.009
Regulation: TP53 ---> AKT1	18534819	10.1016/j.cellsig.2008.04.011
Regulation: TP53 ---> AKT1	14624950	10.1016/S0899-9007(03)00174-6
Regulation: TP53 ---> AKT1	15862750	10.1016/j.ejca.2005.02.011
Regulation: TP53 ---> AKT1	15862750	10.1016/j.ejca.2005.02.011
Regulation: TP53 ---> AKT1	15862750	10.1016/j.ejca.2005.02.011
Regulation: TP53 ---> AKT1	16084079	10.1016/j.ejca.2005.03.033
Regulation: TP53 ---> AKT1	22079189	10.1016/j.tibs.2011.10.002
Regulation: TP53 ---> AKT1	15209377	10.1016/j.semcdb.2004.01.002
Regulation: TP53 ---> AKT1	11545734	10.1016/S1097-2765(01)00323-9
Regulation: TP53 ---> AKT1	12742394	10.1016/S1350-9462(03)00035-1
Regulation: TP53 ---> AKT1	19520599	10.1016/j.cytogfr.2009.05.007
Regulation: TP53 ---> AKT1	34502416	10.3390/ijms22179507
Regulation: TP53 ---> AKT1	34502416	10.3390/ijms22179507

Regulation:	TP53 ---> AKT1	34445495	10.3390/ijms22168784
Regulation:	TP53 ---> AKT1	34416907	10.1186/s13046-021-02061-y
Regulation:	TP53 ---> AKT1	32275841	10.1080/15384101.2020.174979010.1080/15384101
Regulation:	TP53 ---> AKT1	32275841	10.1080/15384101.2020.174979010.1080/15384101
Regulation:	TP53 ---> AKT1	32275841	10.1080/15384101.2020.174979010.1080/15384101
Regulation:	TP53 ---> AKT1	19197146	10.4161/cbt.8.3.7443
Regulation:	TP53 ---> AKT1	21999842	10.1186/1476-4598-10-12910.1186/1476-4598-10-
Regulation:	TP53 ---> AKT1	24451373	10.1074/jbc.M113.53252310.1074/jbc.M113.53252
Regulation:	TP53 ---> AKT1	24469034	10.1038/onc.2013.58510.1038/onc.2013.585
Regulation:	TP53 ---> AKT1	24488098	10.1038/cdd.2014.210.1038/cdd.2014.2
Regulation:	TP53 ---> AKT1	24613379	10.1016/j.freeradbiomed.2014.02.02310.1016/j.
Regulation:	TP53 ---> AKT1	25268131	10.1371/journal.pone.010681210.1371/journal.p
Regulation:	TP53 ---> AKT1		10.1016/j.bbrc.2019.02.089
Regulation:	TP53 ---> AKT1		10.1042/BCJ20190888
Regulation:	TP53 ---> AKT1		10.1016/j.prp.2019.152531
Regulation:	TP53 ---> AKT1		10.1016/j.bmc.2019.115235
Regulation:	TP53 ---> AKT1		10.1016/j.jtcme.2020.02.003
Regulation:	TP53 ---> AKT1		10.1016/j.bpj.2010.07.042
Regulation:	TP53 ---> AKT1		10.1016/j.bpj.2010.07.042
Regulation:	TP53 ---> AKT1		10.1016/j.ejca.2005.02.011
Regulation:	TP53 ---> AKT1		10.1016/j.ejca.2005.02.011
Regulation:	TP53 ---> AKT1		10.1016/j.ejca.2005.02.011
Regulation:	TP53 ---> AKT1		10.1016/j.tibs.2011.10.002
Regulation:	TP53 ---> AKT1		10.1016/j.semcdb.2004.01.002
Regulation:	TP53 ---> AKT1	19648052	10.1016/j.drup.2009.07.001
Regulation:	TP53 ---> AKT1	19411072	10.1016/j.ccr.2009.03.021
Regulation:	TP53 ---> AKT1	19796712	10.1016/j.mito.2009.09.006
Regulation:	TP53 ---> AKT1	22999923	10.1016/j.neulet.2012.09.022
Regulation:	TP53 ---> AKT1	22975375	10.1016/j.ccr.2012.08.001
Regulation:	TP53 ---> AKT1	23870914	10.1016/j.foodchem.2013.05.021
Regulation:	TP53 ---> AKT1	24113167	10.1016/j.bbapap.2013.09.019
Regulation:	TP53 ---> AKT1	24060999	10.1016/j.jprot.2013.09.009
Regulation:	TP53 ---> AKT1	24333688	10.1016/j.pharmthera.2013.12.005
Regulation:	TP53 ---> AKT1	24374014	10.1016/j.canlet.2013.12.025
Regulation:	TP53 ---> AKT1		10.1016/j.bbamem.2014.10.036
Regulation:	TP53 ---> AKT1		10.1016/j.bbrc.2014.11.018
Regulation:	TP53 ---> AKT1	25016051	10.1016/j.ygcen.2014.06.029
Regulation:	TP53 ---> AKT1	24915467	10.1016/j.mad.2014.06.002
Regulation:	TP53 ---> AKT1	25907832	10.1016/j.ajpath.2015.01.025
Regulation:	TP53 ---> AKT1	25446094	10.1016/j.bbrc.2014.11.018
Regulation:	TP53 ---> AKT1	25939952	10.1016/j.toxlet.2015.04.015
Regulation:	TP53 ---> AKT1	26066464	10.1016/j.meegid.2015.06.008
Regulation:	TP53 ---> AKT1	25450343	10.1016/j.bbamem.2014.10.036
Regulation:	TP53 ---> AKT1	26718407	10.1016/j.bbrc.2015.12.070
Regulation:	TP53 ---> AKT1	20124728	
Regulation:	TP53 ---> AKT1	15472230	
Regulation:	TP53 ---> AKT1	12639981	
Regulation:	TP53 ---> AKT1	21138557	
Regulation:	TP53 ---> AKT1	18441250	
Regulation:	TP53 ---> AKT1	12529380	
Regulation:	TP53 ---> AKT1	32664542	10.3390/ijms2114490210.3390/ijms21144902
Regulation:	TP53 ---> AKT1	32922194	10.7150/ijms.4895510.7150/ijms.48955
Regulation:	TP53 ---> AKT1	33038921	10.1186/s40779-020-00275-310.1186/s40779-020-
Regulation:	TP53 ---> AKT1	11959846	

Regulation: TP53 ---> AKT1	10579725	
Regulation: TP53 ---> AKT1	10579725	
Regulation: TP53 ---> AKT1		10.3727/096504018X15424918479652
Regulation: TP53 ---> AKT1	11782444	10.1101/gad.942902
Regulation: TP53 ---> AKT1	29190376	10.1093/jmcb/mjx05110.1093/jmcb/mjx051
Regulation: TP53 ---> AKT1	10579725	10.1083/jcb.147.5.1063
Regulation: TP53 ---> AKT1	10579725	10.1083/jcb.147.5.1063
Regulation: TP53 ---> AKT1	19917135	
Regulation: TP53 ---> AKT1	15792956	
Regulation: TP53 ---> AKT1	10579725	
Regulation: TP53 ---> AKT1	10579725	
Regulation: TP53 ---> AKT1	18445683	
Regulation: TP53 ---> AKT1	18445683	
Regulation: TP53 ---> AKT1	18270270	
Regulation: TP53 ---> AKT1	17921246	
Regulation: TP53 ---> AKT1	12972501	
Regulation: TP53 ---> AKT1	12972501	
Regulation: TP53 ---> AKT1	11368782	
Regulation: TP53 ---> AKT1	19057511	
Regulation: TP53 ---> AKT1		10.1016/j.cytogfr.2014.09.003
Regulation: TP53 ---> AKT1	25438737	10.1016/j.cytogfr.2014.09.003
Regulation: TP53 ---> AKT1	22030255	10.1016/j.canlet.2011.09.040
Regulation: TP53 ---> AKT1	15308206	10.1016/j.tcb.2004.07.006
Regulation: TP53 ---> AKT1	23211536	10.1016/j.canlet.2012.11.042
Regulation: TP53 ---> AKT1	24468652	10.1016/j.semcdb.2014.01.006
Regulation: TP53 ---> AKT1	25016051	10.1016/j.ygcen.2014.06.029
Regulation: TP53 ---> AKT1	25016051	10.1016/j.ygcen.2014.06.029
Regulation: TP53 ---> AKT1	24857548	10.1016/j.molcel.2014.04.024
Regulation: TP53 ---> AKT1	24857662	10.1016/j.celrep.2014.04.046
Regulation: TP53 ---> AKT1	19339243	
Regulation: TP53 ---> AKT1	18356162	
Regulation: TP53 ---> AKT1	10446202	
Regulation: TP53 ---> AKT1	15150542	
Regulation: TP53 ---> AKT1	12719720	
Regulation: TP53 ---> AKT1	12719720	
Regulation: TP53 ---> AKT1	12483525	
Regulation: TP53 ---> AKT1	12149653	
Regulation: TP53 ---> AKT1	11850850	
Regulation: TP53 ---> AKT1	11850850	
Regulation: TP53 ---> AKT1	11850850	
Regulation: TP53 ---> AKT1	11850850	
Regulation: TP53 ---> AKT1	11850850	
Regulation: TP53 ---> AKT1	11296226	
Regulation: TP53 ---> AKT1	11296226	
Regulation: TP53 ---> AKT1	11296226	
Regulation: TP53 ---> AKT1	11296226	
Regulation: TP53 ---> AKT1	11296226	
Regulation: TP53 ---> AKT1	11296226	
Regulation: TP53 ---> AKT1	12446788	
Regulation: TP53 ---> AKT1	19197384	
Regulation: TP53 ---> AKT1	22559167	10.1186/1471-2407-12-166
Regulation: TP53 ---> AKT1	22961366	10.3892/ijo.2012.1623
Regulation: TP53 ---> AKT1		
Regulation: TP53 ---> AKT1		

Regulation: TP53 ---> AKT1		
Regulation: TP53 ---> AKT1		
Regulation: TP53 ---> AKT1		
Regulation: TP53 ---> AKT1	20685653	10.1074/jbc.M110.134650
Regulation: TP53 ---> AKT1	21293193	10.4161/cc.10.4.14864
Regulation: TP53 ---> AKT1	21395546	10.2174/156800911795538129
Regulation: TP53 ---> AKT1	21743969	10.3892/or.2011.1380
Regulation: TP53 ---> AKT1		10.1253/circj.CJ-11-0025
Regulation: TP53 ---> AKT1	21138557	10.1186/1476-4598-9-311
Regulation: TP53 ---> AKT1	22592924	10.1165/rcmb.2011-0390OC
Regulation: TP53 ---> AKT1	23733230	10.3892/or.2013.2518
Regulation: TP53 ---> AKT1	21487429	10.1038/bjc.2011.84
Regulation: TP53 ---> AKT1	20972462	10.1038/onc.2010.486
Regulation: TP53 ---> AKT1	20972462	10.1038/onc.2010.486
Regulation: TP53 ---> AKT1	20972462	10.1038/onc.2010.486
Regulation: TP53 ---> AKT1	20972462	10.1038/onc.2010.486
Regulation: TP53 ---> AKT1	21478913	10.1038/onc.2011.117
Regulation: TP53 ---> AKT1	22231450	10.1038/onc.2011.576
Regulation: TP53 ---> AKT1	23970333	10.3892/ijo.2013.2070
Regulation: TP53 ---> AKT1	22076154	10.1371/journal.pone.0027355
Regulation: TP53 ---> AKT1		10.1378/chest.11-2746
Regulation: TP53 ---> AKT1		10.1378/chest.11-2746
Regulation: TP53 ---> AKT1	23028800	10.1371/journal.pone.0045129
Regulation: TP53 ---> AKT1	23285096	10.1371/journal.pone.0052572
Regulation: TP53 ---> AKT1	24343302	10.1038/ncomms3935
Regulation: TP53 ---> AKT1		10.1074/jbc.M113.532523
Regulation: TP53 ---> AKT1		10.1074/jbc.M113.532523
Regulation: TP53 ---> AKT1		10.1074/jbc.M113.532523
Regulation: TP53 ---> AKT1		10.1074/jbc.M113.532523
Regulation: TP53 ---> AKT1		10.1074/jbc.M113.532523
Regulation: TP53 ---> AKT1		10.4161/auto.27286
Regulation: TP53 ---> AKT1	24488098	10.1038/cdd.2014.2
Regulation: TP53 ---> AKT1	24971482	10.1038/cdd.2014.69
Regulation: TP53 ---> AKT1	25268131	10.1371/journal.pone.0106812
Regulation: TP53 ---> AKT1	25501339	10.3892/or.2014.3665
Regulation: TP53 ---> AKT1	25663065	10.1007/s12032-015-0483-4
Regulation: TP53 ---> AKT1	25748921	10.1371/journal.pone.0118926
Regulation: TP53 ---> AKT1	24052005	10.1159/000353164
Regulation: TP53 ---> AKT1	25692338	10.1080/15384047.2015.1009269
Regulation: TP53 ---> AKT1	25692338	10.1080/15384047.2015.1009269
Regulation: TP53 ---> AKT1	25607549	10.1089/dna.2014.2745
Regulation: TP53 ---> AKT1	25719246	10.1038/cddis.2015.33
Regulation: TP53 ---> AKT1		10.1172/JCI80402
Regulation: TP53 ---> AKT1		10.1172/JCI80402
Regulation: TP53 ---> AKT1		10.1172/JCI80402
Regulation: TP53 ---> AKT1		10.1172/JCI80402
Regulation: TP53 ---> AKT1		10.1172/JCI82058
Regulation: TP53 ---> AKT1		10.1172/JCI82058
Regulation: TP53 ---> AKT1		
Regulation: TP53 ---> AKT1	20220043	10.1177/0091270010362904
Regulation: TP53 ---> AKT1	11959846	
Regulation: TP53 ---> AKT1	15001399	
Regulation: TP53 ---> AKT1	16061640	
Regulation: TP53 ---> AKT1	16894566	

Regulation: TP53 ---> AKT1	17935714	
Regulation: TP53 ---> AKT1	21073655	10.1111/j.1742-4658.2010.07911.x
Regulation: TP53 ---> AKT1	23194110	10.1111/j.1742-481X.2012.12000.x
Regulation: TP53 ---> AKT1	21575475	
Regulation: TP53 ---> AKT1	25045020	10.1002/cbin.10334
Regulation: TP53 ---> AKT1	25501339	10.3892/or.2014.3665
Regulation: TP53 ---> AKT1	25623760	
Regulation: TP53 ---> AKT1	25961455	10.1172/JCI80402
Regulation: TP53 ---> AKT1	26181206	10.1038/cddis.2015.191
Regulation: TP53 ---> AKT1	26723251	10.1016/j.bbrc.2015.12.096
Regulation: TP53 ---> AKT1	26239548	10.3892/mmr.2015.4137
Regulation: TP53 ---> AKT1	26790143	10.1016/j.ejca.2015.12.00210.1016/j.ejca.2015
Regulation: TP53 ---> AKT1	26496776	10.1007/s10495-015-1188-z
Regulation: TP53 ---> AKT1	26181206	10.1038/cddis.2015.191
Regulation: TP53 ---> AKT1	26181206	10.1038/cddis.2015.191
Regulation: TP53 ---> AKT1	26181206	10.1038/cddis.2015.191
Regulation: TP53 ---> AKT1	26181206	10.1038/cddis.2015.191
Regulation: TP53 ---> AKT1	26181206	10.1038/cddis.2015.191
Regulation: TP53 ---> AKT1	26181206	10.1038/cddis.2015.191
Regulation: TP53 ---> AKT1	26181206	10.1038/cddis.2015.191
Regulation: TP53 ---> AKT1	26181206	10.1038/cddis.2015.191
Regulation: TP53 ---> AKT1		
Regulation: TP53 ---> AKT1	27869737	10.3390/ijms17111928
Regulation: TP53 ---> AKT1	28229963	10.1007/s12038-017-9667-8
Regulation: TP53 ---> AKT1	28322991	10.1016/j.gene.2017.03.01410.1016/j.gene.2017
Regulation: TP53 ---> AKT1	28339062	10.3892/ijo.2017.392210.3892/ijo.2017.3922
Regulation: TP53 ---> AKT1		10.1530/JME-16-0189
Regulation: TP53 ---> AKT1	28339062	10.3892/ijo.2017.3922
Regulation: TP53 ---> AKT1	28339062	10.3892/ijo.2017.3922
Regulation: TP53 ---> AKT1	28439015	10.1073/pnas.1619832114
Regulation: TP53 ---> AKT1	28498433	10.3892/or.2017.5637
Regulation: TP53 ---> AKT1	28513299	10.1177/1010428317705330
Regulation: TP53 ---> AKT1	28166194	10.1038/onc.2016.518
Regulation: TP53 ---> AKT1	28667123	10.1073/pnas.170099611410.1073/pnas.170099611
Regulation: TP53 ---> AKT1	28691365	10.1111/acel.1263910.1111/acel.12639
Regulation: TP53 ---> AKT1	28667123	10.1073/pnas.1700996114
Regulation: TP53 ---> AKT1	28667123	10.1073/pnas.1700996114
Regulation: TP53 ---> AKT1	28667123	10.1073/pnas.1700996114
Regulation: TP53 ---> AKT1	28667123	10.1073/pnas.1700996114
Regulation: TP53 ---> AKT1	28667123	10.1073/pnas.1700996114
Regulation: TP53 ---> AKT1	28667123	10.1073/pnas.1700996114
Regulation: TP53 ---> AKT1	28667123	10.1073/pnas.1700996114
Regulation: TP53 ---> AKT1	28263966	10.1038/onc.2017.14
Regulation: TP53 ---> AKT1	27714633	10.1007/s12035-016-0126-6
Regulation: TP53 ---> AKT1	27278236	10.1007/s00232-016-9909-y
Regulation: TP53 ---> AKT1	28727928	10.1139/cjpp-2017-0149
Regulation: TP53 ---> AKT1	28643165	10.1007/s00018-017-2575-0
Regulation: TP53 ---> AKT1	29147906	10.1007/978-981-10-6583-5_8
Regulation: TP53 ---> AKT1	29147906	10.1007/978-981-10-6583-5_8
Regulation: TP53 ---> AKT1	28696156	10.1080/15384047.2017.1345397
Regulation: TP53 ---> AKT1	28696156	10.1080/15384047.2017.1345397
Regulation: TP53 ---> AKT1	28696156	10.1080/15384047.2017.1345397
Regulation: TP53 ---> AKT1	28696156	10.1080/15384047.2017.1345397
Regulation: TP53 ---> AKT1	28696156	10.1080/15384047.2017.1345397

Regulation: TP53 ---> AKT1	28696156	10.1080/15384047.2017.1345397
Regulation: TP53 ---> AKT1	28696156	10.1080/15384047.2017.1345397
Regulation: TP53 ---> AKT1	29242354	10.1124/mol.117.110759
Regulation: TP53 ---> AKT1	29434218	10.1038/s41419-017-0191-4
Regulation: TP53 ---> AKT1	29504895	10.1186/s12864-018-4474-7
Regulation: TP53 ---> AKT1	29681985	10.1155/2018/8324696
Regulation: TP53 ---> AKT1	29693151	10.3892/or.2018.6380
Regulation: TP53 ---> AKT1	29845212	10.3892/ijo.2018.4416
Regulation: TP53 ---> AKT1	30431131	10.3892/or.2018.6870
Regulation: TP53 ---> AKT1	30576070	10.1002/tox.2268510.1002/tox.22685
Regulation: TP53 ---> AKT1		10.1186/s12881-019-0752-8
Regulation: TP53 ---> AKT1	30289354	10.1080/15384047.2018.1523858
Regulation: TP53 ---> AKT1	30337688	10.1038/s41388-018-0521-8
Regulation: TP53 ---> AKT1		10.1097/PPO.0000000000000365
Regulation: TP53 ---> AKT1	31126310	10.1186/s12943-019-1030-2
Regulation: TP53 ---> AKT1		10.1186/s12935-019-0910-2
Regulation: TP53 ---> AKT1		10.1186/s12935-019-0910-2
Regulation: TP53 ---> AKT1	32014919	10.21873/anticanres.1400810.21873/anticanres.
Regulation: TP53 ---> AKT1	32014919	10.21873/anticanres.1400810.21873/anticanres.
Regulation: TP53 ---> AKT1	32161037	10.1523/ENEURO.0438-19.202010.1523/ENEURO.043
Regulation: TP53 ---> AKT1		10.1007/s13238-019-0640-1
Regulation: TP53 ---> AKT1	31115790	10.1007/s13238-019-0634-z
Regulation: TP53 ---> AKT1	31115790	10.1007/s13238-019-0634-z
Regulation: TP53 ---> AKT1	31115790	10.1007/s13238-019-0634-z
Regulation: TP53 ---> AKT1	31115790	10.1007/s13238-019-0634-z
Regulation: TP53 ---> AKT1	31115790	10.1007/s13238-019-0634-z
Regulation: TP53 ---> AKT1	31115790	10.1007/s13238-019-0634-z
Regulation: TP53 ---> AKT1		10.1038/s41388-019-1022-0
Regulation: TP53 ---> AKT1	32242617	10.1042/BCJ2019088810.1042/BCJ20190888
Regulation: TP53 ---> AKT1	32274755	10.1007/978-3-030-38266-7_5
Regulation: TP53 ---> AKT1	32223728	10.2174/0929866527666200330172646
Regulation: TP53 ---> AKT1	32223728	10.2174/0929866527666200330172646
Regulation: TP53 ---> AKT1	32223728	10.2174/0929866527666200330172646
Regulation: TP53 ---> AKT1	33184290	10.1038/s41419-020-03185-3
Regulation: TP53 ---> AKT1	33184290	10.1038/s41419-020-03185-3
Regulation: TP53 ---> AKT1	33446037	10.1080/15384047.2020.1839278
Regulation: TP53 ---> AKT1	33688358	10.1155/2021/4652492
Regulation: TP53 ---> AKT1	33164579	10.1152/AJPHEART.00304.2020
Regulation: TP53 ---> AKT1	33164579	10.1152/AJPHEART.00304.2020
Regulation: TP53 ---> AKT1	33238278	10.1159/000511505
Regulation: TP53 ---> AKT1	26359920	10.1139/cjpp-2015-0159
Regulation: TP53 ---> AKT1	33761957	10.1186/s12929-021-00715-9
Regulation: TP53 ---> AKT1	34064134	10.3390/ijms22115445
Regulation: TP53 ---> AKT1	33920054	10.3390/molecules26082278
Regulation: TP53 ---> AKT1	34205414	10.3390/ijms22126587
Regulation: TP53 ---> AKT1	34209215	10.3390/ijms22137059
Regulation: TP53 ---> AKT1	34209215	10.3390/ijms22137059
Regulation: TP53 ---> AKT1	34209215	10.3390/ijms22137059
Regulation: TP53 ---> AKT1	34830349	10.3390/ijms222212466
Regulation: TP53 ---> AKT1	34813650	10.1042/BCJ20210077
Regulation: TP53 ---> AKT1	34907345	10.1038/s41388-021-02147-z10.1038/s41388-021-
Regulation: TP53 ---> AKT1	34907345	10.1038/s41388-021-02147-z10.1038/s41388-021-
Regulation: TP53 ---> AKT1	34907345	10.1038/s41388-021-02147-z10.1038/s41388-021-
Regulation: TP53 ---> AKT1	34911439	10.1186/s11658-021-00293-6

Regulation: TP53 ---> AKT1 34911439 10.1186/s11658-021-00293-6
Regulation: TP53 ---> AKT1 10.3390/molecules27010199
Regulation: TP53 ---> AKT1 10.3390/molecules27010199
Regulation: TP53 ---> AKT1 10.1016/j.ygyno.2022.01.027
positive Expression: CD34 --+> BE 22617440 10.4161/auto.20053
positive QuantitativeChange: hypc 25268615 10.3390/ijms151017411
positive Regulation: NET1 --+> TF 29769144 10.1186/s13058-018-0966-2
negative QuantitativeChange: hypc 35894468 10.2174/1389203723666220727090317
negative QuantitativeChange: hypc 35894468 10.2174/1389203723666220727090317
negative Expression: ING4 ---| AE 23969950 10.3892/or.2013.267110.3892/or.2013.2671
negative Expression: CYP2J2 ---| 28160853 10.1016/j.phymed.2016.12.001
negative Expression: CYP2J2 ---| 31295013 10.1096/fj.201900756R
negative Expression: CYP2J2 ---| 31295013 10.1096/fj.201900756R
negative Expression: CYP2J2 ---| 31295013 10.1096/fj.201900756R
negative Expression: CYP2J2 ---| 31295013 10.1096/fj.201900756R10.1096/fj.201900756R
negative Expression: CYP2J2 ---| 31295013 10.1096/fj.201900756R
negative Expression: CYP2J2 ---| 31295013 10.1096/fj.201900756R
negative Expression: CYP2J2 ---| 31295013 10.1096/fj.201900756R
negative Expression: CYP2J2 ---| 31295013 10.1096/fj.201900756R
negative Expression: CYP2J2 ---| 31295013 10.1096/fj.201900756R
negative Expression: CYP2J2 ---| 35897843 10.3390/ijms23158267
positive Regulation: CCL3L1 --+> 35437648 10.1007/s11427-021-2097-1
Expression: SPP1 ---> TP53 30537505 10.1016/j.ijbiomac.2018.12.075
Expression: SPP1 ---> TP53 17785423
Expression: SPP1 ---> TP53 29110207 10.1007/s10571-017-0563-4
Expression: SPP1 ---> TP53 20676127 10.1038/onc.2010.325
Expression: SPP1 ---> TP53 22052905 10.1074/jbc.M111.257519
Expression: SPP1 ---> TP53 19016748 10.1111/j.1349-7006.2008.00911.x
Expression: SPP1 ---> TP53
positive Expression: TP53 --+> SF 15501463 10.1016/j.jss.2004.03.028
positive Expression: TP53 --+> SF 17113338 10.1016/j.cytogfr.2006.09.010
positive Expression: TP53 --+> SF 32931483 10.1172/JCI137186
positive Expression: TP53 --+> SF 11807984 10.1002/gcc.10020
positive Expression: TP53 --+> SF 11807984 10.1002/gcc.10020
positive Expression: TP53 --+> SF 32638492 10.1111/acel.1318310.1111/acel.13183
positive Expression: TP53 --+> SF 16567167 10.1016/j.jpedsurg.2005.12.001
positive Expression: TP53 --+> SF 22405968 10.1016/j.yexcr.2012.02.022
positive Expression: TP53 --+> SF 20053348 10.1016/j.yexmp.2009.12.008
positive Expression: TP53 --+> SF 33785447 10.1016/j.semcancer.2021.03.010
negative MolTransport: TP53 ---| 19620778
negative MolTransport: TP53 ---| 32931483 10.1172/JCI137186
negative Regulation: PTEN ---| AK 11991859 10.2741/tsugawa
negative Regulation: PTEN ---| AK 10760291 10.1073/pnas.97.8.4233
negative Regulation: PTEN ---| AK 15889265 10.1007/s00418-005-0783-4
negative Regulation: PTEN ---| AK 20444542 10.1016/j.canlet.2010.04.006
negative Regulation: PTEN ---| AK 10748157 10.1074/jbc.M909741199
negative Regulation: PTEN ---| AK 19001549 10.1152/ajpendo.90581.2008
negative Regulation: PTEN ---| AK 10823906 10.1073/pnas.110149597
negative Regulation: PTEN ---| AK 17420249 10.1074/jbc.M608810200
negative Regulation: PTEN ---| AK 15379721 10.2174/1568008043339857
negative Regulation: PTEN ---| AK 17006756 10.1007/s10549-006-9295-8
negative Regulation: PTEN ---| AKT1
negative Regulation: PTEN ---| AK 11991859
negative Regulation: PTEN ---| AK 10760291

negative Regulation: PTEN ---| AK 15379721
positive Expression: ABCC1 --+> F 31571144 10.1007/s12272-019-01183-4
negative miRNAEffect: MIR107 ---| 30885433 10.1016/j.bbrc.2019.03.009
negative miRNAEffect: MIR107 ---| 33785379 10.1016/j.neulet.2021.135855
negative miRNAEffect: MIR107 ---| 33785379 10.1016/j.neulet.2021.135855
negative miRNAEffect: MIR107 ---| 29857052 10.1016/j.biocel.2018.05.016
negative miRNAEffect: MIR107 ---| 29328395 10.3892/or.2018.6205
negative miRNAEffect: MIR107 ---| 30149689 10.3390/ijms19092531
negative miRNAEffect: MIR107 ---| 30149689 10.3390/ijms19092531
negative miRNAEffect: MIR107 ---| 30149689 10.3390/ijms19092531
negative miRNAEffect: MIR107 ---| 30149689 10.3390/ijms19092531
negative miRNAEffect: MIR107 ---| 30149689 10.3390/ijms19092531
negative miRNAEffect: MIR107 ---| 30149689 10.3390/ijms19092531
negative miRNAEffect: MIR107 ---| 29361817 10.4143/crt.2017.537
negative miRNAEffect: MIR107 ---| 29361817 10.4143/crt.2017.537
negative miRNAEffect: MIR107 ---| 29361817 10.4143/crt.2017.537
negative miRNAEffect: MIR107 ---| 29361817 10.4143/crt.2017.537
negative miRNAEffect: MIR107 ---| 29361817 10.4143/crt.2017.537
negative miRNAEffect: MIR107 ---| 29361817 10.4143/crt.2017.537
negative miRNAEffect: MIR107 ---| 29361817 10.4143/crt.2017.537
negative miRNAEffect: MIR107 ---| 29361817 10.4143/crt.2017.537
negative miRNAEffect: MIR107 ---| 29361817 10.4143/crt.2017.537
negative miRNAEffect: MIR107 ---| 30123092 10.1186/s12935-018-0612-1
negative miRNAEffect: MIR107 ---| PTEN
negative miRNAEffect: MIR107 ---| PTEN
negative miRNAEffect: MIR107 ---| PTEN
negative miRNAEffect: MIR107 ---| 30777076 10.1186/s12943-019-0958-6
negative miRNAEffect: MIR107 ---| 31799671 10.26355/eurrev_201911_19568
negative miRNAEffect: MIR107 ---| 32246301 10.1007/s10522-020-09876-w
negative miRNAEffect: MIR107 ---| 21158707 10.2174/138945011798184155
negative miRNAEffect: MIR107 ---| 22408395 10.3390/ijms13021347
negative miRNAEffect: MIR107 ---| 29361817 10.4143/crt.2017.53710.4143/crt.2017.537
negative miRNAEffect: MIR107 ---| 30575901 10.26355/eurrev_201812_1662510.26355/eurrev_2
negative miRNAEffect: MIR107 ---| 31190980 10.2147/CMAR.S19888610.2147/CMAR.S198886
negative miRNAEffect: MIR107 ---| 31933853
negative miRNAEffect: MIR107 ---| PTEN 10.26355/eurrev_201911_19568
negative miRNAEffect: MIR107 ---| 29111166 10.1016/j.yexcr.2017.10.03310.1016/j.yexcr.20
negative miRNAEffect: MIR107 ---| 130 10.1016/j.yexcr.2017.10.033
negative miRNAEffect: MIR107 ---| 130 10.1016/j.yexcr.2017.10.033
negative miRNAEffect: MIR107 ---| 30149689 10.3390/ijms19092531
negative miRNAEffect: MIR107 ---| 30149689 10.3390/ijms19092531
negative miRNAEffect: MIR107 ---| 30149689 10.3390/ijms19092531
negative miRNAEffect: MIR107 ---| 30149689 10.3390/ijms19092531
negative miRNAEffect: MIR107 ---| 34368895 10.1007/s00709-021-01695-1
negative miRNAEffect: MIR107 ---| 34368895 10.1007/s00709-021-01695-1
negative miRNAEffect: MIR107 ---| 34368895 10.1007/s00709-021-01695-1
negative miRNAEffect: MIR107 ---| 35717938 10.1159/00052547610.1159/000525476
Expression: PTEN ---> ABCC1 18702307
positive PromoterBinding: TP53 -- 18234542 10.1016/j.oraloncology.2007.10.006
positive PromoterBinding: TP53 -- 10196169 10.1074/jbc.274.16.10911
positive PromoterBinding: TP53 -- 16865247
positive PromoterBinding: TP53 -- 17043422 10.3346/jkms.2006.21.5.871
positive PromoterBinding: TP53 -- 15608668 10.1038/sj.onc.1208353
positive PromoterBinding: TP53 -- 16682957 10.1038/sj.onc.1209579

positive PromoterBinding: TP53 --17594159 10.3349/ymj.2007.48.3.495
positive PromoterBinding: TP53 --17929263 10.1002/jso.20907
positive PromoterBinding: TP53 --15921850 10.1016/j.canlet.2005.03.023
positive PromoterBinding: TP53 --19148357 10.1590/s0004-28032008000400007
positive PromoterBinding: TP53 --16515599 10.1111/j.1525-1438.2006.00477.x
positive PromoterBinding: TP53 --+> PTGS2
positive PromoterBinding: TP53 --10196169
positive PromoterBinding: TP53 --16865247
positive PromoterBinding: TP53 --17043422 10.3346/jkms.2006.21.5.871
positive PromoterBinding: TP53 --15608668 10.1038/sj.onc.1208353
positive PromoterBinding: TP53 --16682957 10.1038/sj.onc.1209579
positive PromoterBinding: TP53 --17594159 10.3349/ymj.2007.48.3.495
positive PromoterBinding: TP53 --17929263 10.1002/jso.20907
positive PromoterBinding: TP53 --15921850 10.1016/j.canlet.2005.03.023
positive PromoterBinding: TP53 --19148357
positive PromoterBinding: TP53 --16515599 10.1111/j.1525-1438.2006.00477.x
positive Expression: FCN3 --+> TP53 10.7150/ijbs.69784
Binding: SH3GL3 ---- RAC1 17341688
Expression: TP53 ---> SPP1 15501463 10.1016/j.jss.2004.03.028
Expression: TP53 ---> SPP1 32638492 10.1111/acel.1318310.1111/acel.13183
Expression: TP53 ---> SPP1 17113338 10.1016/j.cytogfr.2006.09.010
Expression: TP53 ---> SPP1 11807984 10.1002/gcc.10020
Expression: TP53 ---> SPP1 11807984 10.1002/gcc.10020
Expression: TP53 ---> SPP1 16567167 10.1016/j.jpedsurg.2005.12.001
Expression: TP53 ---> SPP1 22405968 10.1016/j.yexcr.2012.02.022
Expression: TP53 ---> SPP1 20053348 10.1016/j.yexmp.2009.12.008
Expression: TP53 ---> SPP1 22099172 10.1016/j.diff.2011.08.008
Expression: TP53 ---> SPP1 22099172 10.1016/j.diff.2011.08.008
Expression: TP53 ---> SPP1
Expression: TP53 ---> SPP1 10.1172/JCI137186
Expression: TP53 ---> SPP1 33785447 10.1016/j.semcancer.2021.03.010
Expression: GADD45G ---> TP53 10597261
Expression: GADD45G ---> TP53 10597261
negative Regulation: PTGS2 ---| 133741331 10.1016/j.bcp.2021.114523
negative Regulation: PTGS2 ---| 133741331 10.1016/j.bcp.2021.114523
negative Regulation: PTGS2 ---| 120558725 10.1074/jbc.M110.135251
negative Regulation: PTGS2 ---| 120236942 10.1074/jbc.M109.078576
negative Regulation: PTGS2 ---| 120236942 10.1074/jbc.M109.078576
negative Regulation: PTGS2 ---| 120236942 10.1074/jbc.M109.078576
negative Regulation: PTGS2 ---| 115550400 10.1074/jbc.M406577200
negative Regulation: PTGS2 ---| 115550400 10.1074/jbc.M406577200
negative Regulation: PTGS2 ---| 115550400 10.1074/jbc.M406577200
negative Regulation: PTGS2 ---| 115550400 10.1074/jbc.M406577200
negative Regulation: PTGS2 ---| 115550400 10.1074/jbc.M406577200
negative Regulation: PTGS2 ---| 115550400 10.1074/jbc.M406577200
negative Regulation: PTGS2 ---| 115550400 10.1074/jbc.M406577200
negative Regulation: PTGS2 ---| 115550400 10.1074/jbc.M406577200
negative Regulation: PTGS2 ---| 115550400 10.1074/jbc.M406577200
negative Regulation: PTGS2 ---| 133794285 10.1016/j.jtbi.2021.110696
negative Regulation: PTGS2 ---| 129959028 10.1016/j.lfs.2018.06.028
negative Regulation: PTGS2 ---| 133551224 10.1016/j.arcmed.2021.01.005
negative Regulation: PTGS2 ---| 129242151 10.1016/j.fct.2017.12.011
negative Regulation: PTGS2 ---| 129242151 10.1016/j.fct.2017.12.011
negative Regulation: PTGS2 ---| 129242151 10.1016/j.fct.2017.12.011

negative Regulation: PTGS2 ---| 130784938 10.1016/j.biopha.2019.108669
negative Regulation: PTGS2 ---| 130784938 10.1016/j.biopha.2019.108669
negative Regulation: PTGS2 ---| 130118847 10.1016/j.ejps.2018.08.019
negative Regulation: PTGS2 ---| 126774195 10.1016/j.semcancer.2015.11.001
negative Regulation: PTGS2 ---| 126424600 10.1074/mcp.M115.053926
negative Regulation: PTGS2 ---| 129247872 10.1016/j.gpb.2017.03.005
negative Regulation: PTGS2 ---| 129247872 10.1016/j.gpb.2017.03.005
negative Regulation: PTGS2 ---| 129247872 10.1016/j.gpb.2017.03.005
negative Regulation: PTGS2 ---| 116936256 10.2353/ajpath.2006.060079
negative Regulation: PTGS2 ---| 121356386 10.1016/j.ajpath.2010.11.074
negative Regulation: PTGS2 ---| 116996907 10.1016/j.athoracsur.2006.04.069
negative Regulation: PTGS2 ---| 115707991 10.1016/j.bbrc.2005.01.072
negative Regulation: PTGS2 ---| 115707991 10.1016/j.bbrc.2005.01.072
negative Regulation: PTGS2 ---| 122561872 10.1016/j.taap.2012.04.023
negative Regulation: PTGS2 ---| 119465063 10.1016/j.bbamcr.2009.05.006
negative Regulation: PTGS2 ---| 119465063 10.1016/j.bbamcr.2009.05.006
negative Regulation: PTGS2 ---| 119465063 10.1016/j.bbamcr.2009.05.006
negative Regulation: PTGS2 ---| 119465063 10.1016/j.bbamcr.2009.05.006
negative Regulation: PTGS2 ---| 119465063 10.1016/j.bbamcr.2009.05.006
negative Regulation: PTGS2 ---| 119465063 10.1016/j.bbamcr.2009.05.006
negative Regulation: PTGS2 ---| 119465063 10.1016/j.bbamcr.2009.05.006
negative Regulation: PTGS2 ---| 119465063 10.1016/j.bbamcr.2009.05.006
negative Regulation: PTGS2 ---| 119465063 10.1016/j.bbamcr.2009.05.006
negative Regulation: PTGS2 ---| 119465063 10.1016/j.bbamcr.2009.05.006
negative Regulation: PTGS2 ---| 119465063 10.1016/j.bbamcr.2009.05.006
negative Regulation: PTGS2 ---| 119465063 10.1016/j.bbamcr.2009.05.006
negative Regulation: PTGS2 ---| 119465063 10.1016/j.bbamcr.2009.05.006
negative Regulation: PTGS2 ---| 119465063 10.1016/j.bbamcr.2009.05.006
negative Regulation: PTGS2 ---| 119465063 10.1016/j.bbamcr.2009.05.006
negative Regulation: PTGS2 ---| 119465063 10.1016/j.bbamcr.2009.05.006
negative Regulation: PTGS2 ---| 119465063 10.1016/j.bbamcr.2009.05.006
negative Regulation: PTGS2 ---| 119465063 10.1016/j.bbamcr.2009.05.006
negative Regulation: PTGS2 ---| 119465063 10.1016/j.bbamcr.2009.05.006
negative Regulation: PTGS2 ---| 115246187 10.1016/j.lungcan.2004.01.013
negative Regulation: PTGS2 ---| 119239949 10.1016/j.amjoto.2008.02.012
negative Regulation: PTGS2 ---| 121763744 10.1016/j.neuint.2011.06.018
negative Regulation: PTGS2 ---| 120685028 10.1016/j.ejogrb.2010.06.006
negative Regulation: PTGS2 ---| 119217709 10.1016/j.canlet.2009.01.021
negative Regulation: PTGS2 ---| 118334284 10.1016/j.ctrv.2008.01.006
negative Regulation: PTGS2 ---| 119695440 10.1016/j.ijrobp.2009.04.086
negative Regulation: PTGS2 ---| 116140209 10.1016/j.freeradbiomed.2005.05.015
negative Regulation: PTGS2 ---| 122561872 10.1016/j.taap.2012.04.023
negative Regulation: PTGS2 ---| 124333447 10.1016/j.bbrc.2013.12.038
negative Regulation: PTGS2 ---| 133972599 10.1038/s41598-021-89220-510.1038/s41598-021-
negative Regulation: PTGS2 ---| 133972599 10.1038/s41598-021-89220-510.1038/s41598-021-
negative Regulation: PTGS2 ---| TP53 10.1016/j.ajpath.2010.11.074
negative Regulation: PTGS2 ---| TP53 10.1016/j.taap.2012.04.023
negative Regulation: PTGS2 ---| 124792472 10.1016/j.jphotobiol.2014.03.015
negative Regulation: PTGS2 ---| 124792472 10.1016/j.jphotobiol.2014.03.015
negative Regulation: PTGS2 ---| 124792472 10.1016/j.jphotobiol.2014.03.015
negative Regulation: PTGS2 ---| 124792472 10.1016/j.jphotobiol.2014.03.015
negative Regulation: PTGS2 ---| 124792472 10.1016/j.jphotobiol.2014.03.015
negative Regulation: PTGS2 ---| 124792472 10.1016/j.jphotobiol.2014.03.015
negative Regulation: PTGS2 ---| 117389748

negative Regulation: PTGS2 ---| 119706164
negative Regulation: PTGS2 ---| 115550400
negative Regulation: PTGS2 ---| 115550400
negative Regulation: PTGS2 ---| 115550400
negative Regulation: PTGS2 ---| 115550400
negative Regulation: PTGS2 ---| 115550400
negative Regulation: PTGS2 ---| 115550400
negative Regulation: PTGS2 ---| 115550400
negative Regulation: PTGS2 ---| 115550400
negative Regulation: PTGS2 ---| 115550400
negative Regulation: PTGS2 ---| 115550400
negative Regulation: PTGS2 ---| 111756433
negative Regulation: PTGS2 ---| 116800815
negative Regulation: PTGS2 ---| 111756671
negative Regulation: PTGS2 ---| 111756671
negative Regulation: PTGS2 ---| 118356920
negative Regulation: PTGS2 ---| 112411481
negative Regulation: PTGS2 ---| 121209926
negative Regulation: PTGS2 ---| 128346420 10.1038/onc.2017.73
negative Regulation: PTGS2 ---| TP53 10.1530/ERC-17-0450
negative Regulation: PTGS2 ---| TP53 10.1530/ERC-17-0376
negative Regulation: PTGS2 ---| 120558725 10.1074/jbc.M110.135251
negative Regulation: PTGS2 ---| 124792472 10.1016/j.jphotobiol.2014.03.015
negative Regulation: PTGS2 ---| 121254973 10.2174/092986711794940833
negative Regulation: PTGS2 ---| 126212257 10.1016/j.biocel.2015.07.009
negative Regulation: PTGS2 ---| 115550400
negative Regulation: PTGS2 ---| 123328527 10.1164/rccm.201208-1361OC
negative Regulation: PTGS2 ---| 116650963 10.1016/j.ejso.2006.02.024
negative Regulation: PTGS2 ---| 121340650 10.1007/s00011-011-0316-7
negative Regulation: PTGS2 ---| 121765464 10.1038/onc.2011.234
negative Regulation: PTGS2 ---| 121765464 10.1038/onc.2011.234
negative Regulation: PTGS2 ---| 121765464 10.1038/onc.2011.234
negative Regulation: PTGS2 ---| 121765464 10.1038/onc.2011.234
negative Regulation: PTGS2 ---| 121209926 10.1371/journal.pone.0015174
negative Regulation: PTGS2 ---| 124926985 10.1371/journal.pone.0099743
negative Regulation: PTGS2 ---| 123907425 10.1515/hsz-2012-0355
negative Regulation: PTGS2 ---| 123907425 10.1515/hsz-2012-0355
negative Regulation: PTGS2 ---| 111756433
negative Regulation: PTGS2 ---| 114500353
negative Regulation: PTGS2 ---| 115698513
negative Regulation: PTGS2 ---| 115707991
negative Regulation: PTGS2 ---| 115707991
negative Regulation: PTGS2 ---| 115608668
negative Regulation: PTGS2 ---| 117582597
negative Regulation: PTGS2 ---| 119346994 10.1097/MPA.0b013e31819fef8b
negative Regulation: PTGS2 ---| 119465063 10.1016/j.bbamcr.2009.05.006
negative Regulation: PTGS2 ---| 119465063 10.1016/j.bbamcr.2009.05.006
negative Regulation: PTGS2 ---| 119465063 10.1016/j.bbamcr.2009.05.006
negative Regulation: PTGS2 ---| 119465063 10.1016/j.bbamcr.2009.05.006
negative Regulation: PTGS2 ---| 120236942 10.1074/jbc.M109.078576
negative Regulation: PTGS2 ---| 121668942 10.1186/1471-2407-11-238
negative Regulation: PTGS2 ---| 124792472 10.1016/j.jphotobiol.2014.03.015
negative Regulation: PTGS2 ---| TP53 10.1074/mcp.M115.053926
negative Regulation: PTGS2 ---| 127633132 10.3892/or.2016.5089

negative Regulation: PTGS2 ---| TP53
negative Regulation: PTGS2 ---| T29242151 10.1016/j.fct.2017.12.01110.1016/j.fct.2017.1
negative Regulation: PTGS2 ---| T29883595 10.1016/j.bbcan.2018.06.001
negative Regulation: PTGS2 ---| T28960214 10.1038/emm.2017.144
negative Regulation: PTGS2 ---| TP53 10.1016/j.arcmed.2021.01.005
negative Regulation: PTGS2 ---| T34711017 10.31557/APJCP.2021.22.10.3393
negative Regulation: PTGS2 ---| T34711017 10.31557/APJCP.2021.22.10.3393
Regulation: TP53 ---> GADD45G 15644399
Regulation: TP53 ---> GADD45G 15644399 10.1210/jc.2004-1848
Regulation: TP53 ---> GADD45G 30072642 10.3390/ijms19082269
Regulation: TP53 ---> GADD45G 30072642 10.3390/ijms19082269
Regulation: TP53 ---> GADD45G 34326251 10.1073/pnas.2025539118
Regulation: TP53 ---> GADD45G 35099007 10.1242/DMM.049327
Expression: SPP1 ---> CD274 28830685 10.1016/j.yexcr.2017.08.028
Expression: SPP1 ---> CD274 28830685 10.1016/j.yexcr.2017.08.028
Expression: SPP1 ---> CD274 28830685 10.1016/j.yexcr.2017.08.028
Expression: SPP1 ---> CD274 28830685 10.1016/j.yexcr.2017.08.02810.1016/j.yexcr.20
Expression: SPP1 ---> CD274 28830685 10.1016/j.yexcr.2017.08.028
Expression: SPP1 ---> CD274 28830685 10.1016/j.yexcr.2017.08.028
Expression: SPP1 ---> CD274 30206757 10.1007/s10620-018-5282-2
positive Expression: MAPK1 --+> C23274518 10.1016/j.taap.2012.12.014
positive Expression: MAPK1 --+> C19749156
positive Expression: MAPK1 --+> C19749156
positive Expression: MAPK1 --+> C21266576 10.1074/jbc.M110.172247
positive Expression: MAPK1 --+> C22430216 10.1038/onc.2012.97
positive Expression: MAPK1 --+> C22430216 10.1038/onc.2012.97
positive Expression: MAPK1 --+> C21266576 10.1074/jbc.M110.17224710.1074/jbc.M110.17224
positive Expression: MAPK1 --+> C22430216 10.1038/onc.2012.9710.1038/onc.2012.97
positive Expression: MAPK1 --+> C23947803 10.1186/bcr3458
positive Expression: MAPK1 --+> C23947803 10.1186/bcr3458
positive Expression: MAPK1 --+> C25805831 10.1096/fj.14-265603
positive Regulation: GPER1 --+> N29996493 10.3390/ijms19072011
positive Regulation: GPER1 --+> N30576226 10.1139/cjpp-2018-0544
positive Regulation: GPER1 --+> N33237585 10.1111/jcmm.1599710.1111/jcmm.15997
positive Regulation: GPER1 --+> N24275097 10.1016/j.biocel.2013.11.011
positive Regulation: GPER1 --+> N24275097 10.1016/j.biocel.2013.11.011
positive Regulation: GPER1 --+> N27548161 10.3390/ijms17081357
positive Regulation: GPER1 --+> N25423896 10.1038/nrn3856
positive Regulation: GPER1 --+> N29996493 10.3390/ijms19072011
positive Regulation: GPER1 --+> N35216398 10.3390/ijms23042284
positive Expression: NOTCH1 --+> 25670080 10.1016/j.ccell.2014.11.013
positive Expression: NOTCH1 --+> 23342113 10.1371/journal.pone.0054262
positive Expression: NOTCH1 --+> 23342113 10.1371/journal.pone.0054262
positive Expression: NOTCH1 --+> 23342113 10.1371/journal.pone.0054262
positive Expression: NOTCH1 --+> AKT1 10.1128/MCB.01074-15
positive Expression: NOTCH1 --+> AKT1 10.1128/MCB.01074-15
positive Expression: NOTCH1 --+> AKT1 10.1128/MCB.01074-15
positive Expression: NOTCH1 --+> AKT1 10.1128/MCB.01074-15
positive Expression: NOTCH1 --+> AKT1 10.1128/MCB.01074-15
positive Expression: NOTCH1 --+> AKT1 10.1071/RD14212
positive Expression: NOTCH1 --+> 18440314 10.1016/j.febslet.2008.04.024
positive Expression: NOTCH1 --+> 23342113 10.1371/journal.pone.0054262
positive Expression: NOTCH1 --+> 23196196 10.1016/j.bbagen.2012.11.008
positive Expression: NOTCH1 --+> 25380967 10.1186/1476-4598-13-247

positive Expression: NOTCH1 --+>	19771395	10.1007/s11060-009-0007-1
positive Expression: NOTCH1 --+>	AKT1	10.1128/MCB.01074-15
positive Expression: NOTCH1 --+>	AKT1	10.1530/REP-16-0689
positive Expression: NOTCH1 --+>	28286920	10.1007/s12026-017-8913-3
Expression: TP53 ---> CD274	32522567	10.1016/j.lfs.2020.117923
Expression: TP53 ---> CD274	33753114	10.1016/j.lfs.2021.119359
Expression: TP53 ---> CD274	33159965	10.1016/j.humpath.2020.10.014
Expression: TP53 ---> CD274	29572005	10.1016/j.lungcan.2018.02.005
Expression: TP53 ---> CD274	31097096	10.1016/j.lungcan.2019.04.005
Expression: TP53 ---> CD274	31097096	10.1016/j.lungcan.2019.04.005
Expression: TP53 ---> CD274	26947605	10.1016/j.eururo.2016.02.048
Expression: TP53 ---> CD274	33993033	10.1016/j.ctrv.2021.102219
Expression: TP53 ---> CD274	32057515	10.1016/j.prp.2020.152852
Expression: TP53 ---> CD274		10.1093/annonc/mdx711.018
Expression: TP53 ---> CD274	27687306	10.1093/annonc/mdw436
Expression: TP53 ---> CD274	31046082	10.1093/annonc/mdz135
Expression: TP53 ---> CD274	33798656	10.1016/j.annonc.2021.03.203
Expression: TP53 ---> CD274	31005030	10.1016/j.drup.2019.04.001
Expression: TP53 ---> CD274	32846356	10.1016/j.dnarep.2020.102952
Expression: TP53 ---> CD274	31495715	10.1016/j.oooo.2019.07.008
Expression: TP53 ---> CD274	28741502	10.1016/j.trecan.2016.09.007
Expression: TP53 ---> CD274	33199193	10.1016/j.trecan.2020.10.005
Expression: TP53 ---> CD274	33199193	10.1016/j.trecan.2020.10.005
Expression: TP53 ---> CD274		10.1016/j.asjsur.2021.06.030
Expression: TP53 ---> CD274	27499357	10.3892/ijo.2016.3632
Expression: TP53 ---> CD274	29855617	10.1038/s41388-018-0347-4
Expression: TP53 ---> CD274	30264678	10.2174/1389203719666180928105632
Expression: TP53 ---> CD274	31399545	10.1073/pnas.1901765116
Expression: TP53 ---> CD274	31506076	10.1186/s13046-019-1403-9
Expression: TP53 ---> CD274	31506076	10.1186/s13046-019-1403-9
Expression: TP53 ---> CD274	31506076	10.1186/s13046-019-1403-9
Expression: TP53 ---> CD274	31506076	10.1186/s13046-019-1403-9
Expression: TP53 ---> CD274	31506076	10.1186/s13046-019-1403-9
Expression: TP53 ---> CD274	31506076	10.1186/s13046-019-1403-9
Expression: TP53 ---> CD274	31506076	10.1186/s13046-019-1403-9
Expression: TP53 ---> CD274	31726940	10.1080/15384101.2019.1688951
Expression: TP53 ---> CD274	32060814	10.1007/s10620-020-06126-4
Expression: TP53 ---> CD274	32929059	10.1038/s41419-020-02940-w
Expression: TP53 ---> CD274	32929059	10.1038/s41419-020-02940-w
Expression: TP53 ---> CD274	32992658	10.3390/ijms21197139
Expression: TP53 ---> CD274	33116132	10.1038/s41467-020-19288-6
Expression: TP53 ---> CD274		10.5483/BMBRep.2021.54.1.241
Expression: TP53 ---> CD274	34088360	10.1186/s13046-021-01987-7
Expression: TP53 ---> CD274	29707124	10.18632/oncotarget.2477010.18632/oncotarget.
Expression: TP53 ---> CD274	30915036	10.3389/fendo.2019.0015210.3389/fendo.2019.00
Expression: TP53 ---> CD274	31506076	10.1186/s13046-019-1403-910.1186/s13046-019-1
Expression: TP53 ---> CD274	31506076	10.1186/s13046-019-1403-910.1186/s13046-019-1
Expression: TP53 ---> CD274	32545367	10.3390/cancers1206153510.3390/cancers1206153
Expression: TP53 ---> CD274	33731702	10.1038/s41419-021-03556-410.1038/s41419-021-
Expression: TP53 ---> CD274		10.3390/ijms22189843
Expression: TP53 ---> CD274	1287	10.1016/j.trecan.2016.09.007
Expression: TP53 ---> CD274		10.1016/j.prp.2020.152852
Expression: TP53 ---> CD274		10.1073/pnas.1901765116
Expression: TP53 ---> CD274		10.1186/s13046-019-1403-9

Expression: TP53 ---> CD274		10.1186/s13046-019-1403-9
Expression: TP53 ---> CD274		10.1186/s13046-019-1403-9
Expression: TP53 ---> CD274		10.1186/s13046-019-1403-9
Expression: TP53 ---> CD274		10.1186/s13046-019-1403-9
Expression: TP53 ---> CD274		10.1186/s13046-019-1403-9
Expression: TP53 ---> CD274		10.1186/s13046-019-1403-9
Expression: TP53 ---> CD274		10.1080/15384101.2019.1688951
Expression: TP53 ---> CD274	29707344	10.21037/jtd.2018.03.1410.21037/jtd.2018.03.1
Expression: TP53 ---> CD274	27687306	10.1093/annonc/mdw436
Expression: TP53 ---> CD274	27281199	10.1038/nature18294
Expression: TP53 ---> CD274	404	10.1016/j.eururo.2016.02.048
Expression: TP53 ---> CD274	27793883	10.21873/anticanres.11145
Expression: TP53 ---> CD274	28714919	10.3390/ijms18071542
Expression: TP53 ---> CD274	29970498	10.21873/anticanres.12662
Expression: TP53 ---> CD274	35090497	10.1186/s12964-021-00816-w
Expression: TP53 ---> CD274	35373730	10.1017/erm.2022.9
Expression: TP53 ---> CD274	35373730	10.1017/erm.2022.9
Expression: TP53 ---> CD274	35373730	10.1017/erm.2022.9
Expression: TP53 ---> CD274	35414774	10.7150/ijbs.67200
Expression: TP53 ---> CD274		10.1016/j.semcancer.2022.05.012
Expression: TP53 ---> CD274	35905642	10.1016/j.tranon.2022.101493
Expression: TP53 ---> CD274	36104100	10.1136/jitc-2022-005068
Expression: TP53 ---> CD274	36233088	10.3390/ijms231911789
Expression: TP53 ---> CD274	36361836	10.3390/ijms232113055
negative Expression: PPARG ---| (	22895549	10.3892/mmr.2012.1034
negative Expression: PPARG ---| (	22895549	10.3892/mmr.2012.1034
negative Expression: PPARG ---| (	22895549	10.3892/mmr.2012.1034
positive QuantitativeChange: hypc	31620898	10.1007/s00432-019-03047-210.1007/s00432-019-
negative Regulation: KDM5B ---| 1	24778210	10.1073/pnas.1324036111
negative Regulation: KDM5B ---| 1	24778210	10.1073/pnas.1324036111
negative Regulation: KDM5B ---| TP53		
Expression: NOTCH1 ---> NGF	26491050	10.1152/ajpcell.00163.2015
Expression: NOTCH1 ---> NGF	25817714	10.1016/j.visres.2015.01.030
positive Regulation: BECN1 --+> c	28242349	10.1016/j.bbcan.2017.02.004
positive Regulation: BECN1 --+> c	28479384	10.1016/j.gene.2017.05.006
positive Regulation: BECN1 --+> c	20207475	10.1016/j.canlet.2010.02.001
positive Regulation: BECN1 --+> c	22771248	10.1016/j.amjoto.2012.05.005
positive Regulation: BECN1 --+> c	23722017	10.1016/j.prp.2013.04.006
positive Regulation: BECN1 --+> c	25523227	10.1016/j.prp.2014.11.010
positive Regulation: BECN1 --+> c	27003389	10.1007/s10495-016-1237-2
positive Regulation: BECN1 --+> c	29207161	10.3892/or.2017.6115
positive Regulation: BECN1 --+> c	29322787	10.4149/neo_2018_161102N528
positive Regulation: BECN1 --+> c	32251287	10.1038/s41419-020-2390-7
positive Regulation: BECN1 --+> c	21350938	10.1007/s10059-011-0034-6
positive Regulation: BECN1 --+> c	22805532	10.1097/IGC.0b013e31825e0caa
positive Regulation: BECN1 --+> c	22665050	10.1038/onc.2012.176
positive Regulation: BECN1 --+> c	26700674	10.1007/s13277-015-4693-3
positive Regulation: BECN1 --+> c	20207475	10.1016/j.canlet.2010.02.00110.1016/j.canlet.
positive Regulation: BECN1 --+> c	22665050	10.1038/onc.2012.17610.1038/onc.2012.176
positive Regulation: BECN1 --+> c	23225331	10.1007/s13277-012-0599-510.1007/s13277-012-0
positive Regulation: BECN1 --+> c	27313746	10.3892/ol.2016.4542
positive Regulation: BECN1 --+> c	31274516	10.1097/CAD.000000000000080410.1097/CAD.00000
positive Regulation: BECN1 --+> c	31966694	
positive Regulation: BECN1 --+> c	31966694	

positive Regulation: BECN1 --+> c 33670664 10.3390/biomedicines902020710.3390/biomedicin
StateChange: hypopharyngeal squamous cell 10.5483/BMBRep.2021.54.1.241
Regulation: TMEM45A ---> chemoser 31801939 10.1038/s41419-019-2088-x
Regulation: TMEM45A ---> chemoser 31801939 10.1038/s41419-019-2088-x10.1038/s41419-019-2
negative Regulation: STC2 ---| I(28964325 10.1016/j.metabol.2017.07.003
negative Regulation: STC2 ---| I(32693362 10.1016/j.ghir.2020.101330
negative Regulation: STC2 ---| I(28911149 10.1210/jc.2017-00033
negative Regulation: STC2 ---| I(25533459 10.1074/jbc.M114.611665
negative Regulation: STC2 ---| I(25533459 10.1074/jbc.M114.61166510.1074/jbc.M114.61166
negative Regulation: STC2 ---| I(36257932 10.1038/s41467-022-33698-8
Binding: PRPH ---- ABCA4 22034031 10.1007/978-1-61779-370-7_8
Expression: TP53 ---> STC2 23664860 10.1016/j.mce.2013.04.024
Expression: TP53 ---> STC2
negative Expression: PTEN ---| M(24239354 10.1016/j.celrep.2013.10.022
negative Expression: PTEN ---| M(16051596
negative Expression: PTEN ---| M(26648536 10.1038/leu.2015.335
negative Expression: PTEN ---| M(16051596 10.1074/jbc.M508187200
negative Expression: PTEN ---| M(27517917 10.3390/ijms1708129810.3390/ijms17081298
negative Expression: PTEN ---| M(24434521 10.1038/cddis.2013.519
negative Expression: PTEN ---| M(16051596
negative Expression: PTEN ---| M(22325465 10.1016/j.imbio.2012.01.001
negative Expression: PTEN ---| M(16051596 10.1074/jbc.M508187200
negative Expression: PTEN ---| M(36381235 10.1158/2767-9764.crc-22-0024
Regulation: MCL1 ---> hypopharyng 27847320 10.1016/j.bbrc.2016.11.049
Regulation: MCL1 ---> hypopharyng 27847320 10.1016/j.bbrc.2016.11.049
positive Regulation: PPARG --+> F 19016747 10.1111/j.1349-7006.2008.00904.x10.1111/j.134
Biomarker: hypopharyngeal squamou 33460997 10.1016/j.anndiagpath.2020.151695
Biomarker: hypopharyngeal squamou 29282038 10.1186/s12885-017-3913-1
Biomarker: hypopharyngeal squamou 29282038 10.1186/s12885-017-3913-1
Biomarker: hypopharyngeal squamou 29282038 10.1186/s12885-017-3913-110.1186/s12885-017-3
Biomarker: hypopharyngeal squamou 35894468 10.2174/1389203723666220727090317
positive Expression: NGF --+> PPA 15632188 10.1074/jbc.M409447200
positive Expression: NGF --+> PPA 15632188 10.1074/jbc.M409447200
positive Expression: NGF --+> PPA 18423377 10.1016/j.bbrc.2008.04.035
positive Expression: NGF --+> PPA 18423377 10.1016/j.bbrc.2008.04.035
positive Expression: NGF --+> PPA 15632188
positive Expression: NGF --+> PPA 15632188
positive Expression: NGF --+> PPA 15632188
positive Expression: NGF --+> PPA 33923652 10.3390/ijms22084137
positive Expression: NGF --+> PPA 15632188 10.1074/jbc.M409447200
positive Expression: NGF --+> PPA 15632188 10.1074/jbc.M409447200
positive Expression: NGF --+> PPA 29523844 10.1038/s41419-018-0429-9
positive Expression: NGF --+> PPA 15632188
positive Expression: NGF --+> PPA 15632188
positive Expression: NGF --+> PPA 15632188
positive Expression: NGF --+> PPARG
GeneticChange: hypopharyngeal squ 15236881 10.1016/j.radonc.2004.05.003
GeneticChange: hypopharyngeal squ 23199662 10.1016/j.nic.2012.08.007
GeneticChange: hypopharyngeal squ 29282038 10.1186/s12885-017-3913-1
GeneticChange: hypopharyngeal squ 29282038 10.1186/s12885-017-3913-1
GeneticChange: hypopharyngeal squ 29282038 10.1186/s12885-017-3913-1
GeneticChange: hypopharyngeal squ 29282038 10.1186/s12885-017-3913-1
GeneticChange: hypopharyngeal squ 29282038 10.1186/s12885-017-3913-1
GeneticChange: hypopharyngeal squ 29282038 10.1186/s12885-017-3913-1

GeneticChange: hypopharyngeal squ	16477330	10.1016/S0929-6646(09)60330-2
GeneticChange: hypopharyngeal squ	29282038	10.1186/s12885-017-3913-110.1186/s12885-017-3
GeneticChange: hypopharyngeal squ	29282038	10.1186/s12885-017-3913-110.1186/s12885-017-3
GeneticChange: hypopharyngeal squ	29282038	10.1186/s12885-017-3913-110.1186/s12885-017-3
GeneticChange: hypopharyngeal squ	34775496	10.1038/s41419-021-04367-3
GeneticChange: hypopharyngeal squ	35849264	10.1007/s10495-022-01750-z
negative Regulation: GPER1 ---| (	34098945	10.1186/s12935-021-02003-w
positive Expression: MAPK1 --+> (	29666117	10.1182/blood-2017-11-764357
positive Expression: MAPK1 --+> CD274		10.1016/j.juro.2018.02.1848
positive Expression: MAPK1 --+> (	32822718	10.1016/j.lfs.2020.118297
positive Expression: MAPK1 --+> (	29428449	10.1016/j.leukres.2018.01.014
positive Expression: MAPK1 --+> (	30851544	10.1016/j.leukres.2019.02.010
positive Expression: MAPK1 --+> (	28720430	10.1016/j.pharmthera.2017.07.004
positive Expression: MAPK1 --+> (	32947221	10.1016/j.lungcan.2020.09.005
positive Expression: MAPK1 --+> (	31336132	10.1016/j.fct.2019.110693
positive Expression: MAPK1 --+> (	31336132	10.1016/j.fct.2019.110693
positive Expression: MAPK1 --+> (	31883986	10.1016/j.fct.2019.111092
positive Expression: MAPK1 --+> (	31883986	10.1016/j.fct.2019.111092
positive Expression: MAPK1 --+> (	33675984	10.1016/j.canlet.2021.02.023
positive Expression: MAPK1 --+> (	33220359	10.1016/j.jep.2020.113598
positive Expression: MAPK1 --+> CD274		10.1093/annonc/mdv528.08
positive Expression: MAPK1 --+> CD274		10.1093/annonc/mdw521.048
positive Expression: MAPK1 --+> (	32177601	10.1093/annonc/mdy315.001
positive Expression: MAPK1 --+> (	33310309	10.1016/j.phymed.2020.153425
positive Expression: MAPK1 --+> (	30287140	10.1016/j.tibs.2018.09.004
positive Expression: MAPK1 --+> (	32294525	10.1016/j.phrs.2020.104806
positive Expression: MAPK1 --+> (	29246442	10.1016/j.immuni.2017.11.016
positive Expression: MAPK1 --+> (	29562194	10.1016/j.immuni.2018.03.014
positive Expression: MAPK1 --+> (	29562194	10.1016/j.immuni.2018.03.014
positive Expression: MAPK1 --+> (	29562194	10.1016/j.immuni.2018.03.014
positive Expression: MAPK1 --+> (	29562194	10.1016/j.immuni.2018.03.014
positive Expression: MAPK1 --+> (	27258185	10.1016/j.intimp.2016.05.016
positive Expression: MAPK1 --+> (	31780372	10.1016/j.intimp.2019.106006
positive Expression: MAPK1 --+> (	30172884	10.1016/j.tranon.2018.08.005
positive Expression: MAPK1 --+> CD274		10.1016/j.apsb.2021.03.008
positive Expression: MAPK1 --+> CD274		10.1016/j.apsb.2021.03.010
positive Expression: MAPK1 --+> (	33171331	10.1016/j.redox.2020.101780
positive Expression: MAPK1 --+> (	16085391	10.1016/j.jdermsci.2005.06.008
positive Expression: MAPK1 --+> (	20971039	10.1016/j.molmed.2010.09.006
positive Expression: MAPK1 --+> (	25500094	10.1016/j.oraloncology.2014.11.014
positive Expression: MAPK1 --+> (	26707383	10.1016/j.cllc.2015.11.006
positive Expression: MAPK1 --+> (	26707383	10.1016/j.cllc.2015.11.006
positive Expression: MAPK1 --+> CD274		10.1016/S1556-0864(16)30012-0
positive Expression: MAPK1 --+> CD274		10.1016/S1556-0864(16)30012-0
positive Expression: MAPK1 --+> (	27617403	10.1038/labinvest.2016.96
positive Expression: MAPK1 --+> (	28344319	10.1038/leu.2017.103
positive Expression: MAPK1 --+> (	28451792	10.1007/s00262-017-2005-z
positive Expression: MAPK1 --+> (	28451792	10.1007/s00262-017-2005-z
positive Expression: MAPK1 --+> (	29765155	10.1038/s41388-018-0303-3
positive Expression: MAPK1 --+> (	31382969	10.1186/s12964-019-0391-x
positive Expression: MAPK1 --+> (	31382969	10.1186/s12964-019-0391-x
positive Expression: MAPK1 --+> (	31113825	10.1136/jclinpath-2019-205818
positive Expression: MAPK1 --+> (	31657787	10.1172/JCI132534
positive Expression: MAPK1 --+> (	31503548	10.1172/JCI127721

positive Expression: MAPK1 --+> (31503548 10.1172/JCI127721
positive Expression: MAPK1 --+> (32194893
positive Expression: MAPK1 --+> (32194893
positive Expression: MAPK1 --+> (32194893
positive Expression: MAPK1 --+> (32194893
positive Expression: MAPK1 --+> (32194893
positive Expression: MAPK1 --+> (32194893
positive Expression: MAPK1 --+> (32194893
positive Expression: MAPK1 --+> (31748880 10.1007/s12253-019-00735-9
positive Expression: MAPK1 --+> (32963532 10.1155/2020/7314195
positive Expression: MAPK1 --+> (34088360 10.1186/s13046-021-01987-7
positive Expression: MAPK1 --+> (34167551 10.1186/s13046-021-01972-0
positive Expression: MAPK1 --+> (34167551 10.1186/s13046-021-01972-0
positive Expression: MAPK1 --+> (34167551 10.1186/s13046-021-01972-0
positive Expression: MAPK1 --+> (34319029 10.31557/APJCP.2021.22.7.2069
positive Expression: MAPK1 --+> (22695874 10.1007/s00262-012-1293-6
positive Expression: MAPK1 --+> (23785454 10.1371/journal.pone.0065821
positive Expression: MAPK1 --+> (24551119 10.1371/journal.pone.0088557
positive Expression: MAPK1 --+> (24606459 10.7314/APJCP.2014.15.3.1321
positive Expression: MAPK1 --+> (24606459 10.7314/APJCP.2014.15.3.1321
positive Expression: MAPK1 --+> (24606459 10.7314/APJCP.2014.15.3.1321
positive Expression: MAPK1 --+> (25889536 10.1186/s12964-015-0093-y
positive Expression: MAPK1 --+> (25889536 10.1186/s12964-015-0093-y
positive Expression: MAPK1 --+> (17363736 10.1182/blood-2006-10-051482
positive Expression: MAPK1 --+> (19703193 10.1111/j.1349-7006.2009.01302.x10.1111/j.134
positive Expression: MAPK1 --+> (19703193 10.1111/j.1349-7006.2009.01302.x10.1111/j.134
positive Expression: MAPK1 --+> (19703193 10.1111/j.1349-7006.2009.01302.x10.1111/j.134
positive Expression: MAPK1 --+> (19703193 10.1111/j.1349-7006.2009.01302.x10.1111/j.134
positive Expression: MAPK1 --+> (19830728 10.1002/eji.20093928910.1002/eji.200939289
positive Expression: MAPK1 --+> (24606459 10.7314/apjcp.2014.15.3.1321
positive Expression: MAPK1 --+> (26019170 10.1158/1078-0432.CCR-15-001610.1158/1078-043
positive Expression: MAPK1 --+> (28218497 10.1002/1878-0261.1203210.1002/1878-0261.1203
positive Expression: MAPK1 --+> (31406255 10.1038/s41388-019-0919-y10.1038/s41388-019-0
positive Expression: MAPK1 --+> (31798578 10.3389/fimmu.2019.0262710.3389/fimmu.2019.02
positive Expression: MAPK1 --+> (32134157 10.1002/mc.2317610.1002/mc.23176
positive Expression: MAPK1 --+> (32194893
positive Expression: MAPK1 --+> (32194893
positive Expression: MAPK1 --+> (34185141 10.1007/s00432-021-03694-4
positive Expression: MAPK1 --+> (34202040 10.3390/medsci903004810.3390/medsci9030048
positive Expression: MAPK1 --+> (34349003 10.1158/1535-7163.MCT-20-111210.1158/1535-716
positive Expression: MAPK1 --+> (34349003 10.1158/1535-7163.MCT-20-111210.1158/1535-716
positive Expression: MAPK1 --+> (34486197 10.1002/cbin.1169710.1002/cbin.11697
positive Expression: MAPK1 --+> (34486197 10.1002/cbin.1169710.1002/cbin.11697
positive Expression: MAPK1 --+> (34599981 10.1016/j.bbamcr.2021.11914410.1016/j.bbamcr.
positive Expression: MAPK1 --+> (741 10.1016/j.tibs.2018.09.004
positive Expression: MAPK1 --+> CD274 10.1016/j.molmed.2010.09.006
positive Expression: MAPK1 --+> CD274 10.1186/s12964-019-0391-x
positive Expression: MAPK1 --+> CD274 10.1186/s12964-019-0391-x
positive Expression: MAPK1 --+> CD274 10.1172/JCI132534
positive Expression: MAPK1 --+> CD274 10.1172/JCI127721
positive Expression: MAPK1 --+> CD274 10.1172/JCI127721
positive Expression: MAPK1 --+> CD274 10.1007/s12253-019-00735-9
positive Expression: MAPK1 --+> (21094289 10.1016/j.intimp.2010.11.009
positive Expression: MAPK1 --+> (28222426 10.1159/000460504

positive Expression: MAPK1 --+> (29090321 10.1007/s00262-017-2086-8
positive Expression: MAPK1 --+> (27617403 10.1038/labinvest.2016.96
positive Expression: MAPK1 --+> (27221508 10.1016/j.steroids.2016.05.006
positive Expression: MAPK1 --+> (27221508 10.1016/j.steroids.2016.05.006
positive Expression: MAPK1 --+> (20937224
positive Expression: MAPK1 --+> (27784882 10.12659/MSM.900661
positive Expression: MAPK1 --+> (28720430 10.1016/j.pharmthera.2017.07.004
positive Expression: MAPK1 --+> (28720430 10.1016/j.pharmthera.2017.07.004
positive Expression: MAPK1 --+> (28451792 10.1007/s00262-017-2005-z
positive Expression: MAPK1 --+> (20937224 10.1170/156
positive Expression: MAPK1 --+> (26707383 10.1016/j.cllc.2015.11.006
positive Expression: MAPK1 --+> (1975 10.1016/j.cllc.2015.11.006
positive Expression: MAPK1 --+> (1975 10.1016/j.cllc.2015.11.006
positive Expression: MAPK1 --+> (1975 10.1016/j.cllc.2015.11.006
positive Expression: MAPK1 --+> CD274
positive Expression: MAPK1 --+> (34729305 10.1016/j.apsb.2021.03.01010.1016/j.apsb.2021
positive Expression: MAPK1 --+> (34805266 10.3389/fmolb.2021.68730510.3389/fmolb.2021.6
positive Expression: MAPK1 --+> (34830179 10.3390/ijms222212297
positive Expression: MAPK1 --+> (34830179 10.3390/ijms222212297
positive Expression: MAPK1 --+> (34830196 10.3390/ijms222212314
positive Expression: MAPK1 --+> (34830209 10.3390/ijms222212330
positive Expression: MAPK1 --+> (34480337 10.1007/s12035-021-02495-7
positive Expression: MAPK1 --+> (35237605 10.3389/fcell.2022.82978810.3389/fcell.2022.8
positive Expression: MAPK1 --+> (35031925 10.1007/s11033-021-07063-9
positive Expression: MAPK1 --+> (35305624 10.1186/s12935-022-02550-w10.1186/s12935-022-
positive Expression: MAPK1 --+> (34167551 10.1186/s13046-021-01972-010.1186/s13046-021-
positive Expression: MAPK1 --+> (35305624 10.1186/s12935-022-02550-w
positive Expression: MAPK1 --+> (35305624 10.1186/s12935-022-02550-w
positive Expression: MAPK1 --+> (35305624 10.1186/s12935-022-02550-w
positive Expression: MAPK1 --+> (35305624 10.1186/s12935-022-02550-w
positive Expression: MAPK1 --+> (35305624 10.1186/s12935-022-02550-w
positive Expression: MAPK1 --+> (35305624 10.1186/s12935-022-02550-w
positive Expression: MAPK1 --+> (35305624 10.1186/s12935-022-02550-w
positive Expression: MAPK1 --+> (35305624 10.1186/s12935-022-02550-w
positive Expression: MAPK1 --+> (33030957 10.4149/neo_2020_200531N589
positive Expression: MAPK1 --+> (33030957 10.4149/neo_2020_200531N589
positive Expression: MAPK1 --+> (33030957 10.4149/neo_2020_200531N589
positive Expression: MAPK1 --+> (35948200 10.1016/j.trsl.2022.08.005
positive Expression: MAPK1 --+> (35955729 10.3390/ijms23158599
positive Expression: MAPK1 --+> (36239572 10.1002/mc.23471
positive Expression: MAPK1 --+> (36528587 10.1186/s12935-022-02805-6
positive Regulation: CD274 --+> \33771483 10.1016/j.yexcr.2021.112575
positive Regulation: CD274 --+> \30898647 10.1016/j.lfs.2019.03.040
positive Regulation: CD274 --+> \29510196 10.1016/j.bbadis.2018.03.002
positive Regulation: CD274 --+> \29510196 10.1016/j.bbadis.2018.03.002
positive Regulation: CD274 --+> \29510196 10.1016/j.bbadis.2018.03.002
positive Regulation: CD274 --+> \29510196 10.1016/j.bbadis.2018.03.002
positive Regulation: CD274 --+> \31422157 10.1016/j.semcancer.2019.08.015
positive Regulation: CD274 --+> \33301862 10.1016/j.semcancer.2020.12.001
positive Regulation: CD274 --+> \31005030 10.1016/j.drup.2019.04.001
positive Regulation: CD274 --+> \29576280 10.1016/j.hbpd.2018.03.001
positive Regulation: CD274 --+> \32663768 10.1016/j.intimp.2020.106759
positive Regulation: CD274 --+> \30603685 10.1016/j.heliyon.2018.e01039
positive Regulation: CD274 --+> \23792703 10.1016/j.autrev.2013.05.003

positive Regulation: CD274 --+> M 28703774 10.3390/ijms18071517
positive Regulation: CD274 --+> M 28982322 10.2174/1381612823666171004120152
positive Regulation: CD274 --+> M 30377341 10.1038/s41416-018-0298-0
positive Regulation: CD274 --+> M 30377341 10.1038/s41416-018-0298-0
positive Regulation: CD274 --+> M 30377341 10.1038/s41416-018-0298-0
positive Regulation: CD274 --+> M 31098772 10.1007/s10616-019-00316-3
positive Regulation: CD274 --+> M 32484460 10.1172/JCI133334
positive Regulation: CD274 --+> M 27140316 10.1038/bjc.2016.120
positive Regulation: CD274 --+> M 29245948 10.18632/oncotarget.2191410.18632/oncotarget.
positive Regulation: CD274 --+> M 29510196 10.1016/j.bbadis.2018.03.00210.1016/j.bbadis.
positive Regulation: CD274 --+> M 29510196 10.1016/j.bbadis.2018.03.00210.1016/j.bbadis.
positive Regulation: CD274 --+> M 30603685 10.1016/j.heliyon.2018.e0103910.1016/j.heliyc
positive Regulation: CD274 --+> M 32277292 10.1007/s12032-020-01372-y10.1007/s12032-020-
positive Regulation: CD274 --+> M 33901179 10.1371/journal.pbio.300119910.1371/journal.p
positive Regulation: CD274 --+> M 34185141 10.1007/s00432-021-03694-4
positive Regulation: CD274 --+> MAPK1 10.1016/j.semcancer.2019.08.015
positive Regulation: CD274 --+> MAPK1 10.1172/JCI133334
positive Regulation: CD274 --+> M 20008522
positive Regulation: CD274 --+> M 20008522
positive Regulation: CD274 --+> MAPK1 10.3390/ijms23010245
positive Regulation: CD274 --+> M 34847253 10.1096/fj.202101306R
positive Regulation: CD274 --+> M 34847253 10.1096/fj.202101306R
positive Regulation: CD274 --+> M 35305624 10.1186/s12935-022-02550-w
positive Regulation: CD274 --+> M 35449134 10.1038/s41467-022-29930-0
positive Regulation: CD274 --+> M 36050267 10.1111/1440-1681.13717
positive Regulation: CD274 --+> M 34018092 10.1007/s10565-021-09613-0
positive Regulation: CD274 --+> M 36232331 10.3390/ijms231911031
positive Regulation: CD274 --+> M 36232331 10.3390/ijms231911031
positive Regulation: CD274 --+> M 36375639 10.1016/j.jbc.2022.102694
positive Regulation: CD274 --+> M 36375639 10.1016/j.jbc.2022.102694
negative Expression: KDM5B ---| T 34528900 10.18632/aging.203528
negative Expression: KDM5B ---| T 25877751 10.1007/s13277-015-3418-y10.1007/s13277-015-3
negative Expression: KDM5B ---| T 34528900 10.18632/aging.203528
negative Expression: KDM5B ---| T 25877751 10.1007/s13277-015-3418-y10.1007/s13277-015-3
negative Expression: KDM5B ---| T 31949850
negative Expression: KDM5B ---| T 31949850
negative Expression: KDM5B ---| T 34528900 10.18632/aging.203528
negative Expression: KDM5B ---| T 30343192 10.1016/j.ejmech.2018.10.040
positive Regulation: TP53 --+> ch 28755993 10.1016/j.ijpharm.2017.07.056
positive Regulation: TP53 --+> ch 29615301 10.1016/j.dld.2018.02.009
positive Regulation: TP53 --+> ch 21211512 10.1016/j.abb.2010.12.034
positive Regulation: TP53 --+> ch 15865929 10.1016/j.bbrc.2005.03.153
positive Regulation: TP53 --+> ch 20233581 10.1016/j.bbrc.2010.03.082
positive Regulation: TP53 --+> chemosensit 10.1016/S0006-2952(01)00733-X
positive Regulation: TP53 --+> ch 12694871 10.1016/S0006-2952(03)00078-9
positive Regulation: TP53 --+> ch 12948852 10.1016/S0006-2952(03)00397-6
positive Regulation: TP53 --+> ch 17445779 10.1016/j.bcp.2007.03.009
positive Regulation: TP53 --+> ch 19028473 10.1016/j.cbi.2008.10.048
positive Regulation: TP53 --+> ch 11435891 10.1016/S0022-5347(05)66150-2
positive Regulation: TP53 --+> chemosensit 10.1016/S0022-5347(05)67323-5
positive Regulation: TP53 --+> ch 10379742 10.1097/00005392-199907000-00017
positive Regulation: TP53 --+> ch 12175703 10.1016/S0024-3205(02)01900-8
positive Regulation: TP53 --+> ch 12409142 10.1016/S0024-3205(02)02177-X
positive Regulation: TP53 --+> ch 14761678 10.1016/j.taap.2003.09.011

positive Regulation: TP53 --+> cł 15891997 10.1016/j.humpath.2005.01.018
positive Regulation: TP53 --+> cł 12893182 10.1016/S0090-8258(03)00282-8
positive Regulation: TP53 --+> cł 12893182 10.1016/S0090-8258(03)00282-8
positive Regulation: TP53 --+> cł 15790436 10.1016/j.ygyno.2004.12.053
positive Regulation: TP53 --+> cł 15790436 10.1016/j.ygyno.2004.12.053
positive Regulation: TP53 --+> cł 16380178 10.1016/j.tibtech.2005.12.002
positive Regulation: TP53 --+> cł 22446689 10.1016/j.jhep.2011.11.029
positive Regulation: TP53 --+> cł 11557115 10.1016/S0169-5002(01)00230-6
positive Regulation: TP53 --+> cł 16307839 10.1016/j.fct.2005.10.003
positive Regulation: TP53 --+> cł 10737712 10.1016/S0304-3835(99)00345-6
positive Regulation: TP53 --+> cł 10893449 10.1016/S0304-3835(00)00480-8
positive Regulation: TP53 --+> cł 12880966 10.1016/S0304-3835(03)00088-0
positive Regulation: TP53 --+> cł 15327837 10.1016/j.canlet.2004.03.044
positive Regulation: TP53 --+> cł 16023288 10.1016/j.canlet.2005.06.002
positive Regulation: TP53 --+> cł 16919866 10.1016/j.canlet.2006.06.013
positive Regulation: TP53 --+> cł 18657356 10.1016/j.canlet.2008.06.006
positive Regulation: TP53 --+> cł 19467788 10.1016/j.ctrv.2009.04.008
positive Regulation: TP53 --+> cł 21856084 10.1016/j.mehy.2011.07.052
positive Regulation: TP53 --+> cł 22353361 10.1016/j.gene.2012.01.099
positive Regulation: TP53 --+> chemosensit 10.1016/j.tiv.2012.03.004
positive Regulation: TP53 --+> cł 17875383 10.1016/j.ijom.2007.06.006
positive Regulation: TP53 --+> cł 17875383 10.1016/j.ijom.2007.06.006
positive Regulation: TP53 --+> cł 16337111 10.1016/j.colsurfb.2005.10.006
positive Regulation: TP53 --+> cł 16713222 10.1016/j.phymed.2006.03.009
positive Regulation: TP53 --+> cł 15691646 10.1016/j.ejca.2004.10.014
positive Regulation: TP53 --+> cł 16146690 10.1016/j.ejca.2005.04.044
positive Regulation: TP53 --+> cł 12850529 10.1016/S1040-8428(03)00066-0
positive Regulation: TP53 --+> cł 16253824 10.1053/j.semtcvs.2005.08.002
positive Regulation: TP53 --+> cł 19508919 10.1016/j.drudis.2009.03.008
positive Regulation: TP53 --+> cł 21195657 10.1016/j.drup.2010.12.001
positive Regulation: TP53 --+> cł 12829013 10.1016/S1471-4914(03)00084-4
positive Regulation: TP53 --+> cł 12726865 10.1016/S1535-6108(03)00078-3
positive Regulation: TP53 --+> cł 20832747 10.1016/j.ccr.2010.08.016
positive Regulation: TP53 --+> cł 17240826 10.1016/j.thorsurg.2006.08.001
positive Regulation: TP53 --+> cł 23040255 10.1053/j.seminoncol.2012.08.003
positive Regulation: TP53 --+> cł 22445862 10.1016/j.tiv.2012.03.004
positive Regulation: TP53 --+> cł 23313858 10.1016/j.yexcr.2012.12.025
positive Regulation: TP53 --+> cł 23973262 10.1016/j.canlet.2013.08.028
positive Regulation: TP53 --+> cł 24462821 10.1016/j.canlet.2014.01.011
positive Regulation: TP53 --+> cł 25117446 10.1016/j.bbrc.2014.08.014
positive Regulation: TP53 --+> cł 25220870 10.1016/j.biochi.2014.09.001
positive Regulation: TP53 --+> chemosensit 10.1016/j.toxlet.2014.11.016
positive Regulation: TP53 --+> cł 24681512 10.1016/j.phrs.2014.03.007
positive Regulation: TP53 --+> cł 24681512 10.1016/j.phrs.2014.03.007
positive Regulation: TP53 --+> cł 25448278 10.1016/j.toxlet.2014.11.016
positive Regulation: TP53 --+> cł 21293058
positive Regulation: TP53 --+> cł 21293058
positive Regulation: TP53 --+> cł 11095436
positive Regulation: TP53 --+> cł 11095436
positive Regulation: TP53 --+> cł 9768682
positive Regulation: TP53 --+> cł 9661637
positive Regulation: TP53 --+> cł 20075077
positive Regulation: TP53 --+> cł 19055826
positive Regulation: TP53 --+> cł 17626635

positive Regulation: TP53 --+> ch 17150101
positive Regulation: TP53 --+> ch 17121812
positive Regulation: TP53 --+> ch 16159878
positive Regulation: TP53 --+> ch 16120770
positive Regulation: TP53 --+> ch 15262986
positive Regulation: TP53 --+> ch 12890671
positive Regulation: TP53 --+> ch 12890671
positive Regulation: TP53 --+> ch 12690107
positive Regulation: TP53 --+> ch 12615954
positive Regulation: TP53 --+> ch 12481433
positive Regulation: TP53 --+> ch 12481433
positive Regulation: TP53 --+> ch 18818514 10.4161/cc.7.19.6777
positive Regulation: TP53 --+> ch 27313779 10.7150/jca.1450610.7150/jca.14506
positive Regulation: TP53 --+> ch 27484708 10.1080/15384101.2016.1215390
positive Regulation: TP53 --+> ch 32492656 10.18632/aging.10337710.18632/aging.103377
positive Regulation: TP53 --+> ch 27313779 10.7150/jca.1450610.7150/jca.14506
positive Regulation: TP53 --+> ch 12615954
positive Regulation: TP53 --+> ch 12615954
positive Regulation: TP53 --+> ch 12615954
positive Regulation: TP53 --+> ch 9694807
positive Regulation: TP53 --+> ch 14769944
positive Regulation: TP53 --+> ch 11583579
positive Regulation: TP53 --+> ch 11248087
positive Regulation: TP53 --+> ch 9122197
positive Regulation: TP53 --+> ch 16755297
positive Regulation: TP53 --+> ch 15153941
positive Regulation: TP53 --+> ch 14739942
positive Regulation: TP53 --+> ch 11791171
positive Regulation: TP53 --+> ch 11791171
positive Regulation: TP53 --+> ch 11791171
positive Regulation: TP53 --+> ch 11791171
positive Regulation: TP53 --+> ch 11791171
positive Regulation: TP53 --+> ch 10698490
positive Regulation: TP53 --+> ch 10471039
positive Regulation: TP53 --+> ch 16260623
positive Regulation: TP53 --+> ch 20706634
positive Regulation: TP53 --+> ch 27329169 10.1007/s10549-016-3869-x
positive Regulation: TP53 --+> chemosensit 10.1186/s13046-014-0091-8
positive Regulation: TP53 --+> ch 27484466 10.1186/s12885-016-2617-2
positive Regulation: TP53 --+> ch 27484725 10.3892/mmr.2016.5552
positive Regulation: TP53 --+> ch 27485825 10.3892/mmr.2016.5561
positive Regulation: TP53 --+> ch 27485825 10.3892/mmr.2016.5561
positive Regulation: TP53 --+> chemosensit 10.1096/fj.201601240R
positive Regulation: TP53 --+> chemosensit 10.1096/fj.201601240R
positive Regulation: TP53 --+> ch 16973289 10.1016/j.bbcan.2006.08.001
positive Regulation: TP53 --+> ch 18064040
positive Regulation: TP53 --+> ch 28112370 10.3892/or.2017.5399
positive Regulation: TP53 --+> ch 8642047 10.1007/bf01220804
positive Regulation: TP53 --+> ch 12615954
positive Regulation: TP53 --+> ch 28467351 10.3390/ijms18050961
positive Regulation: TP53 --+> ch 22615851 10.1371/journal.pone.0036940
positive Regulation: TP53 --+> ch 30106452 10.3892/or.2018.6628
positive Regulation: TP53 --+> ch 30106452 10.3892/or.2018.6628
positive Regulation: TP53 --+> chemosensit 10.1186/s12935-018-0690-0

positive Regulation: TP53 --+> ch 30387834 10.3892/ijo.2018.4613
positive Regulation: TP53 --+> chemosensit 10.4149/neo_2018_180125N55
positive Regulation: TP53 --+> chemosensit 10.1042/BSR20180773
positive Regulation: TP53 --+> ch 31108984 10.3390/ijms20102454
positive Regulation: TP53 --+> chemosensit 10.1186/s12935-019-0910-2
positive Regulation: TP53 --+> ch 32377739 10.3892/mmr.2020.11121
positive Regulation: TP53 --+> chemosensit 10.1042/BSR20191488
positive Regulation: TP53 --+> chemosensit 10.1042/BSR20191488
positive Regulation: TP53 --+> ch 33184290 10.1038/s41419-020-03185-310.1038/s41419-020-
positive Regulation: TP53 --+> ch 33184290 10.1038/s41419-020-03185-3
positive Regulation: TP53 --+> ch 33256191 10.3390/ijms21238991
positive Regulation: TP53 --+> chemosensit 10.1016/j.jbior.2020.100780
positive Regulation: TP53 --+> ch 32876741 10.1007/s00508-020-01729-3
positive Regulation: TP53 --+> ch 33785447 10.1016/j.semcancer.2021.03.010
positive Regulation: TP53 --+> chemosensit 10.1016/j.omtn.2021.07.022
positive Regulation: TP53 --+> chemosensit 10.1016/j.jtcms.2022.01.001
Binding: UCA1 ---- TP53 28738810 10.1186/s12943-017-0699-3
Binding: UCA1 ---- TP53 10.1016/j.semcancer.2020.12.019
negative Regulation: LRP8 ---| PF 24840660 10.1016/j.bbadis.2014.05.009
negative Regulation: LRP8 ---| PF 24840660 10.1016/j.bbadis.2014.05.009
negative Regulation: LRP8 ---| PPARG
positive DirectRegulation: PRND - 24339999 10.1371/journal.pone.0082130
positive DirectRegulation: PRND - 24339999 10.1371/journal.pone.0082130
positive DirectRegulation: PRND - 24339999 10.1371/journal.pone.0082130
positive DirectRegulation: PRND - 24339999 10.1371/journal.pone.0082130
positive DirectRegulation: PRND - 24339999 10.1371/journal.pone.0082130
positive DirectRegulation: PRND - 24339999 10.1371/journal.pone.0082130
positive DirectRegulation: PRND - 22715097 10.1074/jbc.M112.340638
positive DirectRegulation: PRND - 27145840 10.1146/annurev-biochem-060815-014710
positive DirectRegulation: PRND - 24005412 10.1038/nature12481
positive DirectRegulation: PRND - 24003888 10.1042/BSR20130065
positive DirectRegulation: PRND - 23263379 10.1038/ncb2641
positive DirectRegulation: PRND - 24339999 10.1371/journal.pone.0082130
positive DirectRegulation: PRND - 24775734 10.1016/j.tibs.2014.04.001
positive DirectRegulation: PRND - 28489421 10.1146/annurev-biochem-060815-014616
positive DirectRegulation: PRND --+> TP53 10.1128/MCB.00118-17
positive DirectRegulation: PRND - 30341616 10.1007/978-1-4939-8820-4_17
positive Expression: GPER1 --+> (30227306 10.1016/j.neo.2018.08.010
positive Expression: GPER1 --+> (30227306 10.1016/j.neo.2018.08.010
positive Expression: GPER1 --+> (30227306 10.1016/j.neo.2018.08.010
positive Expression: GPER1 --+> (30227306 10.1016/j.neo.2018.08.010
positive Expression: GPER1 --+> (31483053 10.14670/HH-18-157
positive Expression: GPER1 --+> (26731262 10.1371/journal.pone.0145212
positive Expression: GPER1 --+> (30227306 10.1016/j.neo.2018.08.01010.1016/j.neo.2018.0
positive Expression: GPER1 --+> (30227306 10.1016/j.neo.2018.08.010
positive Expression: GPER1 --+> (30227306 10.1016/j.neo.2018.08.010
Regulation: IGFBP4 ---> AKT1 28179206 10.1016/j.neulet.2017.01.066
Regulation: IGFBP4 ---> AKT1 23499909 10.1016/j.cellsig.2013.02.018
Regulation: IGFBP4 ---> AKT1 23499909 10.1016/j.cellsig.2013.02.018
Regulation: IGFBP4 ---> AKT1 23639626 10.1016/j.tox.2013.04.014
Regulation: IGFBP4 ---> AKT1 15572521
Regulation: IGFBP4 ---> AKT1 23499909 10.1016/j.cellsig.2013.02.01810.1016/j.cellsi
Regulation: IGFBP4 ---> AKT1 23971193 10.2478/pjvs-2013-0036
Regulation: IGFBP4 ---> AKT1 29992318 10.1093/nar/gky58910.1093/nar/gky589

Regulation: IGFBP4 ---> AKT1 28150906 10.1002/cbin.1073210.1002/cbin.10732
Regulation: IGFBP4 ---> AKT1
Regulation: IGFBP4 ---> AKT1 10.2174/1381612828666220608122934
Regulation: IGFBP4 ---> AKT1 10.2174/1381612828666220608122934
negative Expression: PHF20 ---| M33982773 10.3892/ijo.2021.5218
positive DirectRegulation: ING4 -26530780 10.3892/or.2015.438510.3892/or.2015.4385
positive DirectRegulation: ING4 -25968091 10.1159/00043010810.1159/000430108
positive DirectRegulation: ING4 -23967213 10.1371/journal.pone.007145310.1371/journal.p
positive DirectRegulation: ING4 -21454715 10.1074/jbc.M111.23096110.1074/jbc.M111.23096
positive DirectRegulation: ING4 -21454715 10.1074/jbc.M111.23096110.1074/jbc.M111.23096
positive DirectRegulation: ING4 -21454715 10.1074/jbc.M111.23096110.1074/jbc.M111.23096
positive DirectRegulation: ING4 -21177815 10.1128/JVI.02279-1010.1128/JVI.02279-10
positive DirectRegulation: ING4 -12750254
positive DirectRegulation: ING4 -12750254
positive DirectRegulation: ING4 -26936485 10.3892/or.2016.4626
positive DirectRegulation: ING4 -26936485 10.3892/or.2016.4626
positive DirectRegulation: ING4 -26882029
positive DirectRegulation: ING4 -26882029
positive DirectRegulation: ING4 -26530780 10.3892/or.2015.4385
positive DirectRegulation: ING4 -26530780 10.3892/or.2015.4385
positive DirectRegulation: ING4 -26530780 10.3892/or.2015.4385
positive DirectRegulation: ING4 -26530780 10.3892/or.2015.4385
positive DirectRegulation: ING4 -26530780 10.3892/or.2015.4385
positive DirectRegulation: ING4 -26530780 10.3892/or.2015.4385
positive DirectRegulation: ING4 -21177815 10.1128/JVI.02279-10
positive DirectRegulation: ING4 -21177815 10.1128/JVI.02279-10
positive DirectRegulation: ING4 -21177815 10.1128/JVI.02279-10
positive DirectRegulation: ING4 -21177815 10.1128/JVI.02279-10
positive DirectRegulation: ING4 -21177815 10.1128/JVI.02279-10
positive DirectRegulation: ING4 -21177815 10.1128/JVI.02279-10
positive DirectRegulation: ING4 -21177815 10.1128/JVI.02279-10
positive DirectRegulation: ING4 -21177815 10.1128/JVI.02279-10
positive DirectRegulation: ING4 -21177815 10.1128/JVI.02279-10
positive DirectRegulation: ING4 -21177815 10.1128/JVI.02279-10
positive DirectRegulation: ING4 -21177815 10.1128/JVI.02279-10
positive DirectRegulation: ING4 -21177815 10.1128/JVI.02279-10
positive DirectRegulation: ING4 -21177815 10.1128/JVI.02279-10
positive DirectRegulation: ING4 -21177815 10.1128/JVI.02279-10
positive DirectRegulation: ING4 -25792601 10.1101/gad.254292.114
positive DirectRegulation: ING4 -25792601 10.1101/gad.254292.114
positive DirectRegulation: ING4 -24581166 10.3779/j.issn.1009-3419.2014.02.13
positive DirectRegulation: ING4 -23967213 10.1371/journal.pone.0071453
positive DirectRegulation: ING4 -23967213 10.1371/journal.pone.0071453
positive DirectRegulation: ING4 -23967213 10.1371/journal.pone.0071453
positive DirectRegulation: ING4 -23967213 10.1371/journal.pone.0071453
positive DirectRegulation: ING4 -23967213 10.1371/journal.pone.0071453
positive DirectRegulation: ING4 -23967213 10.1371/journal.pone.0071453
positive DirectRegulation: ING4 -21454715 10.1074/jbc.M111.230961
positive DirectRegulation: ING4 -21454715 10.1074/jbc.M111.230961
positive DirectRegulation: ING4 -21454715 10.1074/jbc.M111.230961
positive DirectRegulation: ING4 -21454715 10.1074/jbc.M111.230961
positive DirectRegulation: ING4 -21454715 10.1074/jbc.M111.230961
positive DirectRegulation: ING4 -21454715 10.1074/jbc.M111.230961
positive DirectRegulation: ING4 -21454715 10.1074/jbc.M111.230961

positive DirectRegulation: ING4 -21454715 10.1074/jbc.M111.230961
positive DirectRegulation: ING4 -21454715 10.1074/jbc.M111.230961
positive DirectRegulation: ING4 -21454715 10.1074/jbc.M111.230961
positive DirectRegulation: ING4 -21454715 10.1074/jbc.M111.230961
positive DirectRegulation: ING4 -21454715 10.1074/jbc.M111.230961
positive DirectRegulation: ING4 -21454715 10.1074/jbc.M111.230961
positive DirectRegulation: ING4 -21454715 10.1074/jbc.M111.230961
positive DirectRegulation: ING4 -21454715 10.1074/jbc.M111.230961
positive DirectRegulation: ING4 -21454715 10.1074/jbc.M111.230961
positive DirectRegulation: ING4 -21454715 10.1074/jbc.M111.230961
positive DirectRegulation: ING4 -21052543 10.1155/2011/963614
positive DirectRegulation: ING4 --+> TP53 10.1042/BSR20180773
positive DirectRegulation: ING4 -30403588 10.14715/cmb/2018.64.13.2
positive DirectRegulation: ING4 -27466989
positive DirectRegulation: ING4 -28050784 10.1007/s10620-016-4429-2
positive DirectRegulation: ING4 -27544390 10.1007/s00432-016-2219-z
positive DirectRegulation: ING4 -27544390 10.1007/s00432-016-2219-z
positive DirectRegulation: ING4 -26803518 10.1007/s13277-016-4842-3
positive DirectRegulation: ING4 -16024799
positive DirectRegulation: ING4 -17646389
positive DirectRegulation: ING4 -16520380
positive DirectRegulation: ING4 -26072331 10.1016/j.exphem.2015.05.010
positive DirectRegulation: ING4 -24333729 10.1016/j.canlet.2013.11.016
positive DirectRegulation: ING4 -23603392 10.1016/j.febslet.2013.04.004
positive DirectRegulation: ING4 -23603392 10.1016/j.febslet.2013.04.004
positive DirectRegulation: ING4 -15743678 10.1016/j.biocel.2004.09.008
positive DirectRegulation: ING4 -22366145 10.1016/j.jbspin.2012.01.008
positive DirectRegulation: ING4 -18571892 10.1016/j.biopha.2008.04.006
positive DirectRegulation: ING4 -14572637 10.1016/S0014-5793(03)01024-X
positive DirectRegulation: ING4 -18775696 10.1016/j.yexcr.2008.08.002
positive DirectRegulation: ING4 -18775696 10.1016/j.yexcr.2008.08.002
positive DirectRegulation: ING4 -15748897 10.1016/j.yexcr.2004.11.023
positive DirectRegulation: ING4 -15882981 10.1016/j.bbrc.2005.04.023
positive DirectRegulation: ING4 -15882981 10.1016/j.bbrc.2005.04.023
positive DirectRegulation: ING4 -15882981 10.1016/j.bbrc.2005.04.023
positive DirectRegulation: ING4 -15882981 10.1016/j.bbrc.2005.04.023
positive DirectRegulation: ING4 -12750254
positive DirectRegulation: ING4 -21177815 10.1128/JVI.02279-10
positive DirectRegulation: ING4 -21177815 10.1128/JVI.02279-10
positive DirectRegulation: ING4 -21177815 10.1128/JVI.02279-10
positive DirectRegulation: ING4 -21177815 10.1128/JVI.02279-10
positive DirectRegulation: ING4 -21177815 10.1128/JVI.02279-10
positive DirectRegulation: ING4 -23967213 10.1371/journal.pone.0071453
positive DirectRegulation: ING4 -23967213 10.1371/journal.pone.0071453
positive DirectRegulation: ING4 -23967213 10.1371/journal.pone.0071453
positive DirectRegulation: ING4 -23967213 10.1371/journal.pone.0071453
positive DirectRegulation: ING4 -23967213 10.1371/journal.pone.0071453
positive DirectRegulation: ING4 -23967213 10.1371/journal.pone.0071453
positive DirectRegulation: ING4 -23967213 10.1371/journal.pone.0071453
positive DirectRegulation: ING4 -23967213 10.1371/journal.pone.0071453
positive DirectRegulation: ING4 -20534538 10.1073/pnas.0912116107
positive DirectRegulation: ING4 -22228137 10.1007/s00432-011-1099-5
positive DirectRegulation: ING4 -21454715 10.1074/jbc.M111.230961
positive DirectRegulation: ING4 -21454715 10.1074/jbc.M111.230961

positive DirectRegulation: ING4 --+> TP53 10.1042/BSR20180773
positive DirectRegulation: ING4 --+> TP53 10.14670/HH-11-979
positive DirectRegulation: ING4 -27544390 10.1007/s00432-016-2219-z
positive DirectRegulation: ING4 -17954561
positive DirectRegulation: ING4 -17954561
positive DirectRegulation: ING4 -15528276
positive DirectRegulation: ING4 -20534538
positive DirectRegulation: ING4 -16520380
positive DirectRegulation: ING4 -23603392 10.1016/j.febslet.2013.04.004
positive DirectRegulation: ING4 -16387653 10.1016/j.molcel.2005.12.007
positive DirectRegulation: ING4 -15935570 10.1016/j.gene.2005.02.014
positive DirectRegulation: ING4 -15251430 10.1016/j.febslet.2004.06.010
positive DirectRegulation: ING4 -15251430 10.1016/j.febslet.2004.06.010
positive DirectRegulation: ING4 -18775696 10.1016/j.yexcr.2008.08.002
positive DirectRegulation: ING4 -18775696 10.1016/j.yexcr.2008.08.002
positive DirectRegulation: ING4 -15748897 10.1016/j.yexcr.2004.11.023
positive DirectRegulation: ING4 -15882981 10.1016/j.bbrc.2005.04.023
positive DirectRegulation: ING4 -15882981 10.1016/j.bbrc.2005.04.023
positive DirectRegulation: ING4 -15882981 10.1016/j.bbrc.2005.04.023
positive DirectRegulation: ING4 -15882981 10.1016/j.bbrc.2005.04.023
positive DirectRegulation: ING4 -15882981 10.1016/j.bbrc.2005.04.023
positive DirectRegulation: ING4 -15882981 10.1016/j.bbrc.2005.04.023
positive DirectRegulation: ING4 -15882981 10.1016/j.bbrc.2005.04.023
positive DirectRegulation: ING4 -15882981 10.1016/j.bbrc.2005.04.023
positive DirectRegulation: ING4 -15882981 10.1016/j.bbrc.2005.04.023
positive DirectRegulation: ING4 -20707719 10.1089/cbr.2010.077810.1089/cbr.2010.0778
positive DirectRegulation: ING4 -22595793 10.1038/cgt.2012.24
positive DirectRegulation: ING4 -21177815 10.1128/JVI.02279-10
positive DirectRegulation: ING4 -21177815 10.1128/JVI.02279-10
positive DirectRegulation: ING4 -23308284 10.1371/journal.pone.0053791
positive DirectRegulation: ING4 -21454715 10.1074/jbc.M111.230961
positive DirectRegulation: ING4 -15935570 10.1016/j.gene.2005.02.014
positive DirectRegulation: ING4 -15935570 10.1016/j.gene.2005.02.014
positive DirectRegulation: ING4 -15882981 10.1016/j.bbrc.2005.04.023
positive DirectRegulation: ING4 -21177815 10.1128/JVI.02279-10
positive DirectRegulation: ING4 -16520380
positive DirectRegulation: ING4 -15882981 10.1016/j.bbrc.2005.04.023
positive DirectRegulation: ING4 --+> TP53
positive DirectRegulation: ING4 --+> TP53 10.3390/ijms21041351
positive DirectRegulation: ING4 -33076322 10.3390/ijms21207622
negative Regulation: NOTCH1 ---| 19473993
negative Regulation: NOTCH1 ---| 19473992
negative DirectRegulation: PTGS2 21254973 10.2174/092986711794940833
negative DirectRegulation: PTGS2 20558725 10.1074/jbc.M110.135251
negative DirectRegulation: PTGS2 ---| TP53 10.1530/ERC-17-0450
negative DirectRegulation: PTGS2 28346420 10.1038/onc.2017.73
negative DirectRegulation: PTGS2 21209926
negative DirectRegulation: PTGS2 12411481
negative DirectRegulation: PTGS2 18356920
negative DirectRegulation: PTGS2 11756671
negative DirectRegulation: PTGS2 11756671
negative DirectRegulation: PTGS2 16800815
negative DirectRegulation: PTGS2 11756433
negative DirectRegulation: PTGS2 15550400

negative DirectRegulation: PTGS2 15550400
negative DirectRegulation: PTGS2 15550400
negative DirectRegulation: PTGS2 15550400
negative DirectRegulation: PTGS2 15550400
negative DirectRegulation: PTGS2 15550400
negative DirectRegulation: PTGS2 15550400
negative DirectRegulation: PTGS2 15550400
negative DirectRegulation: PTGS2 15550400
negative DirectRegulation: PTGS2 19706164
negative DirectRegulation: PTGS2 17389748
negative DirectRegulation: PTGS2 26774195 10.1016/j.semcancer.2015.11.001
negative DirectRegulation: PTGS2 24792472 10.1016/j.jphotobiol.2014.03.015
negative DirectRegulation: PTGS2 24792472 10.1016/j.jphotobiol.2014.03.015
negative DirectRegulation: PTGS2 24792472 10.1016/j.jphotobiol.2014.03.015
negative DirectRegulation: PTGS2 24792472 10.1016/j.jphotobiol.2014.03.015
negative DirectRegulation: PTGS2 24792472 10.1016/j.jphotobiol.2014.03.015
negative DirectRegulation: PTGS2 24792472 10.1016/j.jphotobiol.2014.03.015
negative DirectRegulation: PTGS2 24792472 10.1016/j.jphotobiol.2014.03.015
negative DirectRegulation: PTGS2 24333447 10.1016/j.bbrc.2013.12.038
negative DirectRegulation: PTGS2 22561872 10.1016/j.taap.2012.04.023
negative DirectRegulation: PTGS2 16140209 10.1016/j.freeradbiomed.2005.05.015
negative DirectRegulation: PTGS2 19695440 10.1016/j.ijrobp.2009.04.086
negative DirectRegulation: PTGS2 18334284 10.1016/j.ctrv.2008.01.006
negative DirectRegulation: PTGS2 19217709 10.1016/j.canlet.2009.01.021
negative DirectRegulation: PTGS2 20685028 10.1016/j.ejogrb.2010.06.006
negative DirectRegulation: PTGS2 21763744 10.1016/j.neuint.2011.06.018
negative DirectRegulation: PTGS2 19239949 10.1016/j.amjoto.2008.02.012
negative DirectRegulation: PTGS2 19465063 10.1016/j.bbamcr.2009.05.006
negative DirectRegulation: PTGS2 19465063 10.1016/j.bbamcr.2009.05.006
negative DirectRegulation: PTGS2 19465063 10.1016/j.bbamcr.2009.05.006
negative DirectRegulation: PTGS2 19465063 10.1016/j.bbamcr.2009.05.006
negative DirectRegulation: PTGS2 19465063 10.1016/j.bbamcr.2009.05.006
negative DirectRegulation: PTGS2 19465063 10.1016/j.bbamcr.2009.05.006
negative DirectRegulation: PTGS2 19465063 10.1016/j.bbamcr.2009.05.006
negative DirectRegulation: PTGS2 19465063 10.1016/j.bbamcr.2009.05.006
negative DirectRegulation: PTGS2 19465063 10.1016/j.bbamcr.2009.05.006
negative DirectRegulation: PTGS2 19465063 10.1016/j.bbamcr.2009.05.006
negative DirectRegulation: PTGS2 19465063 10.1016/j.bbamcr.2009.05.006
negative DirectRegulation: PTGS2 19465063 10.1016/j.bbamcr.2009.05.006
negative DirectRegulation: PTGS2 19465063 10.1016/j.bbamcr.2009.05.006
negative DirectRegulation: PTGS2 19465063 10.1016/j.bbamcr.2009.05.006
negative DirectRegulation: PTGS2 19465063 10.1016/j.bbamcr.2009.05.006
negative DirectRegulation: PTGS2 19465063 10.1016/j.bbamcr.2009.05.006
negative DirectRegulation: PTGS2 19465063 10.1016/j.bbamcr.2009.05.006
negative DirectRegulation: PTGS2 19465063 10.1016/j.bbamcr.2009.05.006
negative DirectRegulation: PTGS2 ---| TP53 10.1016/j.taap.2012.04.023
negative DirectRegulation: PTGS2 15707991 10.1016/j.bbrc.2005.01.072
negative DirectRegulation: PTGS2 15707991 10.1016/j.bbrc.2005.01.072
negative DirectRegulation: PTGS2 16996907 10.1016/j.athoracsur.2006.04.069
negative DirectRegulation: PTGS2 ---| TP53 10.1016/j.ajpath.2010.11.074
negative DirectRegulation: PTGS2 16936256 10.2353/ajpath.2006.060079
negative DirectRegulation: PTGS2 29247872 10.1016/j.gpb.2017.03.005
negative DirectRegulation: PTGS2 29247872 10.1016/j.gpb.2017.03.005

negative DirectRegulation: PTGS2	29247872	10.1016/j.gpb.2017.03.005
negative DirectRegulation: PTGS2	29242151	10.1016/j.fct.2017.12.01110.1016/j.fct.2017.1
negative DirectRegulation: PTGS2	22561872	10.1016/j.taap.2012.04.023
negative DirectRegulation: PTGS2	19465063	10.1016/j.bbamcr.2009.05.006
negative DirectRegulation: PTGS2	19465063	10.1016/j.bbamcr.2009.05.006
negative DirectRegulation: PTGS2	15707991	10.1016/j.bbrc.2005.01.072
negative DirectRegulation: PTGS2	15608668	10.1038/sj.onc.1208353
negative DirectRegulation: PTGS2	15608668	10.1038/sj.onc.1208353
negative DirectRegulation: PTGS2	11687965	10.1038/sj.onc.1204907
negative DirectRegulation: PTGS2	24069219	10.1371/journal.pone.0073656
negative DirectRegulation: PTGS2	21765464	10.1038/onc.2011.234
negative DirectRegulation: PTGS2	21765464	10.1038/onc.2011.234
negative DirectRegulation: PTGS2	21765464	10.1038/onc.2011.234
negative DirectRegulation: PTGS2	29578814	10.1080/01635581.2018.1446091
negative DirectRegulation: PTGS2	16540558	
negative DirectRegulation: PTGS2	11687965	
negative DirectRegulation: PTGS2	24792472	10.1016/j.jphotobiol.2014.03.015
negative DirectRegulation: PTGS2	24792472	10.1016/j.jphotobiol.2014.03.015
negative DirectRegulation: PTGS2	22561872	10.1016/j.taap.2012.04.023
negative DirectRegulation: PTGS2	19457709	10.1016/j.oraloncology.2009.03.016
negative DirectRegulation: PTGS2	21763744	10.1016/j.neuint.2011.06.018
negative DirectRegulation: PTGS2	19465063	10.1016/j.bbamcr.2009.05.006
negative DirectRegulation: PTGS2	19465063	10.1016/j.bbamcr.2009.05.006
negative DirectRegulation: PTGS2	19465063	10.1016/j.bbamcr.2009.05.006
negative DirectRegulation: PTGS2	19465063	10.1016/j.bbamcr.2009.05.006
negative DirectRegulation: PTGS2	19465063	10.1016/j.bbamcr.2009.05.006
negative DirectRegulation: PTGS2	19465063	10.1016/j.bbamcr.2009.05.006
negative DirectRegulation: PTGS2	19465063	10.1016/j.bbamcr.2009.05.006
negative DirectRegulation: PTGS2	19465063	10.1016/j.bbamcr.2009.05.006
negative DirectRegulation: PTGS2 ---| TP5[illegible]		10.1016/j.taap.2012.04.023
negative DirectRegulation: PTGS2	15707991	10.1016/j.bbrc.2005.01.072
negative DirectRegulation: PTGS2	15707991	10.1016/j.bbrc.2005.01.072
negative DirectRegulation: PTGS2	15707991	10.1016/j.bbrc.2005.01.072
negative DirectRegulation: PTGS2	15707991	10.1016/j.bbrc.2005.01.072
negative DirectRegulation: PTGS2	23328527	10.1164/rccm.201208-1361OC
negative DirectRegulation: PTGS2	15550400	
negative DirectRegulation: PTGS2	26212257	10.1016/j.biocel.2015.07.009
Regulation: CD274 ---> MCL1	34193942	10.1038/s41388-021-01910-6
Regulation: CD274 ---> MCL1	33581579	10.1016/j.intimp.2021.107443
Expression: PPARG ---> NGF	33453816	10.1074/jbc.RA119.012019
Expression: PPARG ---> NGF	29032144	10.1016/j.pneurobio.2017.10.002
Expression: PPARG ---> NGF	15713689	
Expression: PPARG ---> NGF	15713689	
Expression: PPARG ---> NGF	15100092	
Expression: PPARG ---> NGF	33082140	10.1074/jbc.RA119.012019
Expression: PPARG ---> NGF	24603727	10.1371/journal.ppat.1003980
Expression: PPARG ---> NGF	25159899	10.1186/s12967-014-0235-9
Expression: PPARG ---> NGF	24934600	10.1089/neu.2014.3429
Expression: PPARG ---> NGF		
Expression: PPARG ---> NGF		10.1074/jbc.RA119.012019
negative Expression: BIRC7 ---| [illegible]	26622485	10.3892/etm.2015.2675
Regulation: SPP1 ---> RAC1	18174176	
Regulation: SPP1 ---> RAC1	18174176	
Regulation: SPP1 ---> RAC1	18174176	

Regulation: SPP1 ---> RAC1	18174176	10.1074/jbc.M706712200
Regulation: SPP1 ---> RAC1		10.1161/STROKEAHA.115.011552
Regulation: SPP1 ---> RAC1	1117	10.1016/j.ccell.2016.08.002
Regulation: SPP1 ---> RAC1		
positive Regulation: PTGS2 --+> /	21689625	10.1016/j.ajpath.2011.04.004
positive Regulation: PTGS2 --+> /	21147091	10.1016/j.ejphar.2010.11.022
positive Regulation: PTGS2 --+> /	19505457	10.1016/j.yexcr.2009.06.002
positive Regulation: PTGS2 --+> /	18722373	10.1053/j.gastro.2008.07.012
positive Regulation: PTGS2 --+> /	17418864	10.1016/j.jss.2007.01.039
positive Regulation: PTGS2 --+> /	17198778	10.1016/j.jtcvs.2006.07.042
positive Regulation: PTGS2 --+> /	18343442	10.1016/j.juro.2007.12.037
positive Regulation: PTGS2 --+> /	16360450	10.1016/j.urology.2005.06.076
positive Regulation: PTGS2 --+> /	15179620	10.1053/j.seminoncol.2004.03.040
positive Regulation: PTGS2 --+> /	18375246	10.1016/j.arcmed.2007.12.005
positive Regulation: PTGS2 --+> /	12618325	10.1016/S0304-3835(02)00524-4
positive Regulation: PTGS2 --+> /	18790560	10.1016/j.canlet.2008.08.012
positive Regulation: PTGS2 --+> /	16337744	10.1016/j.ctrv.2005.10.004
positive Regulation: PTGS2 --+> AKT1		10.1016/j.mehy.2012.04.012
positive Regulation: PTGS2 --+> /	18571350	10.1016/j.jep.2008.05.001
positive Regulation: PTGS2 --+> /	18571350	10.1016/j.jep.2008.05.001
positive Regulation: PTGS2 --+> /	17572069	10.1016/j.cellsig.2007.05.003
positive Regulation: PTGS2 --+> /	20362052	10.1016/j.cellsig.2010.03.014
positive Regulation: PTGS2 --+> /	16002278	10.1016/j.ejca.2005.04.013
positive Regulation: PTGS2 --+> /	18178094	10.1016/j.nbd.2007.11.010
positive Regulation: PTGS2 --+> /	15661561	10.1016/j.critrevonc.2004.10.001
positive Regulation: PTGS2 --+> /	16531064	10.1016/j.critrevonc.2006.01.003
positive Regulation: PTGS2 --+> /	21807123	10.1016/j.intimp.2011.07.011
positive Regulation: PTGS2 --+> AKT1		10.1016/j.jecm.2010.08.003
positive Regulation: PTGS2 --+> /	20854947	10.1016/j.bone.2010.09.020
positive Regulation: PTGS2 --+> /	20854947	10.1016/j.bone.2010.09.020
positive Regulation: PTGS2 --+> /	20854947	10.1016/j.bone.2010.09.020
positive Regulation: PTGS2 --+> /	20854947	10.1016/j.bone.2010.09.020
positive Regulation: PTGS2 --+> /	20854947	10.1016/j.bone.2010.09.020
positive Regulation: PTGS2 --+> /	20854947	10.1016/j.bone.2010.09.020
positive Regulation: PTGS2 --+> /	20854947	10.1016/j.bone.2010.09.020
positive Regulation: PTGS2 --+> /	20854947	10.1016/j.bone.2010.09.020
positive Regulation: PTGS2 --+> /	20854947	10.1016/j.bone.2010.09.020
positive Regulation: PTGS2 --+> /	20854947	10.1016/j.bone.2010.09.020
positive Regulation: PTGS2 --+> /	20854947	10.1016/j.bone.2010.09.020
positive Regulation: PTGS2 --+> /	20854947	10.1016/j.bone.2010.09.020
positive Regulation: PTGS2 --+> /	22546756	10.1016/j.mehy.2012.04.012
positive Regulation: PTGS2 --+> /	23422333	10.1016/j.jep.2013.02.001
positive Regulation: PTGS2 --+> /	23563277	10.1016/j.pharmthera.2013.04.001
positive Regulation: PTGS2 --+> /	24465039	10.1016/j.curtheres.2013.06.001
positive Regulation: PTGS2 --+> /	23907017	10.1016/j.jsbmb.2013.07.008
positive Regulation: PTGS2 --+> /	24296130	10.1016/j.fct.2013.11.035
positive Regulation: PTGS2 --+> /	25064845	10.1016/j.febslet.2014.07.012
positive Regulation: PTGS2 --+> /	25064845	10.1016/j.febslet.2014.07.012
positive Regulation: PTGS2 --+> /	25770423	10.1016/j.bbrc.2015.03.008
positive Regulation: PTGS2 --+> /	25770423	10.1016/j.bbrc.2015.03.008
positive Regulation: PTGS2 --+> /	25770423	10.1016/j.bbrc.2015.03.008
positive Regulation: PTGS2 --+> /	25770423	10.1016/j.bbrc.2015.03.008
positive Regulation: PTGS2 --+> /	25770423	10.1016/j.bbrc.2015.03.008
positive Regulation: PTGS2 --+> /	25770423	10.1016/j.bbrc.2015.03.008

positive Regulation: PTGS2 --+> A20022932
positive Regulation: PTGS2 --+> A21078168
positive Regulation: PTGS2 --+> A21078168
positive Regulation: PTGS2 --+> A20042609
positive Regulation: PTGS2 --+> A19052170
positive Regulation: PTGS2 --+> A16966336
positive Regulation: PTGS2 --+> A16966336
positive Regulation: PTGS2 --+> A15016316
positive Regulation: PTGS2 --+> A12704185
positive Regulation: PTGS2 --+> A19099539
positive Regulation: PTGS2 --+> A16800815
positive Regulation: PTGS2 --+> A20661229
positive Regulation: PTGS2 --+> A17285134
positive Regulation: PTGS2 --+> A16160697
positive Regulation: PTGS2 --+> A16396944
positive Regulation: PTGS2 --+> A16396944
positive Regulation: PTGS2 --+> A16014934
positive Regulation: PTGS2 --+> A16014934
positive Regulation: PTGS2 --+> A16014934
positive Regulation: PTGS2 --+> A17259595
positive Regulation: PTGS2 --+> A22563076 10.1074/jbc.M112.347245
positive Regulation: PTGS2 --+> A23229294 10.3892/or.2012.2158
positive Regulation: PTGS2 --+> A23243000 10.1091/mbc.E12-08-0637
positive Regulation: PTGS2 --+> A20203246 10.1164/rccm.200905-0674OC
positive Regulation: PTGS2 --+> A20203246 10.1164/rccm.200905-0674OC
positive Regulation: PTGS2 --+> A19687189 10.1093/ecam/nep108
positive Regulation: PTGS2 --+> A21561856 10.1074/jbc.M111.222349
positive Regulation: PTGS2 --+> A21561856 10.1074/jbc.M111.222349
positive Regulation: PTGS2 --+> A19293251 10.1093/ecam/nep020
positive Regulation: PTGS2 --+> A20467837 10.1007/s12035-010-8139-z
positive Regulation: PTGS2 --+> A20803081 10.1245/s10434-010-1268-9
positive Regulation: PTGS2 --+> A21078168 10.1186/1471-2407-10-626
positive Regulation: PTGS2 --+> A21078168 10.1186/1471-2407-10-626
positive Regulation: PTGS2 --+> A21457561 10.1186/1743-422X-8-155
positive Regulation: PTGS2 --+> A21457561 10.1186/1743-422X-8-155
positive Regulation: PTGS2 --+> A22048655 10.1007/s00432-011-1080-3
positive Regulation: PTGS2 --+> A22350613 10.1007/s00423-012-0916-x
positive Regulation: PTGS2 --+> A22983360 10.1038/ni.2408
positive Regulation: PTGS2 --+> A23900573 10.3892/ijo.2013.2041
positive Regulation: PTGS2 --+> A23877542 10.3892/ijo.2013.2024
positive Regulation: PTGS2 --+> A22349822 10.1038/onc.2012.47
positive Regulation: PTGS2 --+> A23658755 10.1371/journal.pone.0062627
positive Regulation: PTGS2 --+> A23737956 10.1371/journal.pone.0063872
positive Regulation: PTGS2 --+> A23922859 10.1371/journal.pone.0069928
positive Regulation: PTGS2 --+> A23922175 10.1007/s13277-013-1024-4
positive Regulation: PTGS2 --+> A24988184 10.1371/journal.pone.0100079
positive Regulation: PTGS2 --+> A24988184 10.1371/journal.pone.0100079
positive Regulation: PTGS2 --+> A25823424 10.3892/ijo.2015.2940
positive Regulation: PTGS2 --+> A25997834 10.3892/ijo.2015.3012
positive Regulation: PTGS2 --+> A12939602
positive Regulation: PTGS2 --+> A15309882
positive Regulation: PTGS2 --+> A15312381
positive Regulation: PTGS2 --+> A15492414
positive Regulation: PTGS2 --+> A15767780

positive Regulation: PTGS2 --+> A 15922857
positive Regulation: PTGS2 --+> A 16641552
positive Regulation: PTGS2 --+> A 17198778
positive Regulation: PTGS2 --+> A 17070098
positive Regulation: PTGS2 --+> A 18571350 10.1016/j.jep.2008.05.001
positive Regulation: PTGS2 --+> A 18798061 10.1080/07357900801941860
positive Regulation: PTGS2 --+> A 19671676 10.1158/1541-7786.MCR-09-0004
positive Regulation: PTGS2 --+> A 19621391 10.1002/ijc.24757
positive Regulation: PTGS2 --+> A 19621391 10.1002/ijc.24757
positive Regulation: PTGS2 --+> A 20486853 10.3109/07357907.2010.483504
positive Regulation: PTGS2 --+> A 20854947 10.1016/j.bone.2010.09.020
positive Regulation: PTGS2 --+> A 21807123 10.1016/j.intimp.2011.07.011
positive Regulation: PTGS2 --+> A 22221825 10.1111/j.1872-034X.2011.00947.x
positive Regulation: PTGS2 --+> A 22546756 10.1016/j.mehy.2012.04.012
positive Regulation: PTGS2 --+> A 22784710 10.1158/1535-7163.MCT-12-0342
positive Regulation: PTGS2 --+> A 23149922 10.1158/0008-5472.CAN-12-2220
positive Regulation: PTGS2 --+> A 23435965 10.1007/s10495-013-0825-7
positive Regulation: PTGS2 --+> A 23435965 10.1007/s10495-013-0825-7
positive Regulation: PTGS2 --+> A 24260338 10.1371/journal.pone.0080076
positive Regulation: PTGS2 --+> A 24296130 10.1016/j.fct.2013.11.035
positive Regulation: PTGS2 --+> A 25849942
positive Regulation: PTGS2 --+> A 25770423 10.1016/j.bbrc.2015.03.008
positive Regulation: PTGS2 --+> A 25770423 10.1016/j.bbrc.2015.03.008
positive Regulation: PTGS2 --+> A 25582828 10.1111/cmi.12414
positive Regulation: PTGS2 --+> A 26323553 10.3892/or.2015.4202
positive Regulation: PTGS2 --+> A 26418808 10.1002/jbt.21769
positive Regulation: PTGS2 --+> A 26408666 10.4049/jimmunol.150070410.4049/jimmunol.150[illegible]
positive Regulation: PTGS2 --+> A 26794001 10.3892/or.2016.4584
positive Regulation: PTGS2 --+> AKT1
positive Regulation: PTGS2 --+> A 27725865
Expression: PPARG ---> CD274 21435906 10.1016/j.healun.2011.01.704
Expression: PPARG ---> CD274 25072357 10.1016/j.lfs.2014.07.02110.1016/j.lfs.2014.[illegible]
Expression: PPARG ---> CD274 22425227 10.1016/j.cellimm.2012.02.007
Expression: PPARG ---> CD274
Expression: PPARG ---> CD274 34026331 10.1080/2162402X.2021.190650010.1080/2162402X
Expression: PPARG ---> CD274 33766508 10.1016/j.jid.2021.02.748
positive Regulation: CLDN1 --+> M 33189716 10.1016/j.bbamem.2020.183503
positive Regulation: CLDN1 --+> M 29700355 10.1038/s12276-018-0076-3
positive Regulation: CLDN1 --+> M 33629735 10.14670/HH-18-320
positive Regulation: CLDN1 --+> M 23160379 10.1038/onc.2012.505
positive Regulation: CLDN1 --+> M 23160379 10.1038/onc.2012.505
positive Regulation: CLDN1 --+> M 23160379 10.1038/onc.2012.505
positive Regulation: CLDN1 --+> M 23160379 10.1038/onc.2012.505
positive Regulation: CLDN1 --+> M 23160379 10.1038/onc.2012.505
positive Regulation: CLDN1 --+> M 23160379 10.1038/onc.2012.505
positive Regulation: CLDN1 --+> M 25798937 10.1038/nbt.3179
Binding: ING4 ---- BECN1 29163768 10.18632/oncotarget.1903310.18632/oncotarget.
Binding: ING4 ---- BECN1 29163768 10.18632/oncotarget.1903310.18632/oncotarget.
positive Expression: PTEN --+> IN 24762396 10.1158/0008-5472.CAN-13-307610.1158/0008-547
negative Regulation: PTGS2 ---| c 31494169 10.1016/j.lfs.2019.116825
negative Regulation: PTGS2 ---| c 30954568 10.1016/j.bbamcr.2019.04.003
negative Regulation: PTGS2 ---| c 31473273 10.1016/j.virusres.2019.197735
negative Regulation: PTGS2 ---| c 16580341 10.1016/j.ajog.2005.07.086
negative Regulation: PTGS2 ---| c 19836060 10.1016/j.acthis.2009.09.004

negative Regulation: PTGS2 ---| chemosensi 10.1053/j.seminoncol.2003.12.010
negative Regulation: PTGS2 ---| chemosensi 10.1016/j.ijrobp.2007.07.1757
negative Regulation: PTGS2 ---| chemosensi 10.1016/j.ijrobp.2010.07.1454
negative Regulation: PTGS2 ---| c 24184161 10.1016/j.bbamcr.2013.10.015
negative Regulation: PTGS2 ---| c 24184161 10.1016/j.bbamcr.2013.10.015
negative Regulation: PTGS2 ---| c 18356920
negative Regulation: PTGS2 ---| c 33138752 10.2174/1381612826666201102101758
negative Regulation: PTGS2 ---| c 23393346
negative Regulation: PTGS2 ---| c 16912190 10.1158/0008-5472.CAN-06-1215
negative Regulation: PTGS2 ---| c 17982644
negative Regulation: PTGS2 ---| c 18592138 10.1007/s11010-008-9847-910.1007/s11010-008-9
negative Regulation: PTGS2 ---| c 19276291 10.1158/1078-0432.CCR-08-062910.1158/1078-043
negative Regulation: PTGS2 ---| c 28881683 10.18632/oncotarget.1851810.18632/oncotarget.
negative Regulation: PTGS2 ---| c 30365050 10.3892/ijmm.2018.393910.3892/ijmm.2018.3939
negative Regulation: PTGS2 ---| c 34084214 10.3892/ol.2021.1279410.3892/ol.2021.12794
negative Regulation: PTGS2 ---| chemosensi 10.1016/j.bbamcr.2019.04.003
negative Regulation: PTGS2 ---| chemosensitivity
Expression: UCA1 ---> CD274 31733291 10.1016/j.semcancer.2019.11.006
Expression: UCA1 ---> CD274 10.1016/j.mgene.2020.100687
Expression: UCA1 ---> CD274 33486611 10.1007/s00262-020-02753-y
Expression: UCA1 ---> CD274 33486611 10.1007/s00262-020-02753-y
Expression: UCA1 ---> CD274 33486611 10.1007/s00262-020-02753-y
Expression: UCA1 ---> CD274 33486611 10.1007/s00262-020-02753-y
Expression: UCA1 ---> CD274 33486611 10.1007/s00262-020-02753-y
Expression: UCA1 ---> CD274 33486611 10.1007/s00262-020-02753-y
Expression: UCA1 ---> CD274 33301862 10.1016/j.semcancer.2020.12.001
Expression: UCA1 ---> CD274 33301862 10.1016/j.semcancer.2020.12.001
Expression: UCA1 ---> CD274 35331183 10.1186/s12885-022-09377-8
positive Regulation: TP53 --+> G 15644399 10.1210/jc.2004-1848
positive Regulation: TP53 --+> GADD45G
Regulation: TNFAIP8L2 ---> hypoph 35894468 10.2174/1389203723666220727090317
Expression: GPER1 ---> CD34 30391242 10.1016/j.ygcen.2018.10.021
Expression: GPER1 ---> CD34 24894716 10.1158/0008-5472.CAN-13-359010.1158/0008-547
negative DirectRegulation: TNFAIF 33933441 10.1016/j.yexcr.2021.11263110.1016/j.yexcr.20
negative DirectRegulation: TNFAIF 32460619 10.1080/15548627.2020.176174810.1080/15548627
negative DirectRegulation: TNFAIF 30186591 10.1186/s13578-018-0247-x10.1186/s13578-018-0
negative DirectRegulation: TNFAIF 26530498 10.3892/ijo.2015.322410.3892/ijo.2015.3224
negative DirectRegulation: TNFAIF 24274578 10.1186/1476-4598-12-14910.1186/1476-4598-12-
negative DirectRegulation: TNFAIF 26987289 10.1038/cgt.2016.6
negative DirectRegulation: TNFAIF 26530498 10.3892/ijo.2015.3224
negative DirectRegulation: TNFAIF 25946186 10.1371/journal.pone.0126176
negative DirectRegulation: TNFAIF 24274578 10.1186/1476-4598-12-149
negative DirectRegulation: TNFAIF 24274578 10.1186/1476-4598-12-149
negative DirectRegulation: TNFAIF 24274578 10.1186/1476-4598-12-149
negative DirectRegulation: TNFAIF 24274578 10.1186/1476-4598-12-149
negative DirectRegulation: TNFAIF 24274578 10.1186/1476-4598-12-149
negative DirectRegulation: TNFAIF 24274578 10.1186/1476-4598-12-149
negative DirectRegulation: TNFAIF 24274578 10.1186/1476-4598-12-149
negative DirectRegulation: TNFAIF 32460619 10.1080/15548627.2020.1761748
negative DirectRegulation: TNFAIF 32460619 10.1080/15548627.2020.1761748
negative DirectRegulation: TNFAIF 32460619 10.1080/15548627.2020.1761748
negative DirectRegulation: TNFAIF 32460619 10.1080/15548627.2020.1761748
negative DirectRegulation: TNFAIF 32460619 10.1080/15548627.2020.1761748
negative DirectRegulation: TNFAIF 32460619 10.1080/15548627.2020.1761748

negative DirectRegulation: TNFAIF	32460619	10.1080/15548627.2020.1761748
negative DirectRegulation: TNFAIF	32460619	10.1080/15548627.2020.1761748
negative DirectRegulation: TNFAIF	32460619	10.1080/15548627.2020.1761748
negative DirectRegulation: TNFAIF	32460619	10.1080/15548627.2020.1761748
negative DirectRegulation: TNFAIF	32460619	10.1080/15548627.2020.1761748
negative DirectRegulation: TNFAIF	32460619	10.1080/15548627.2020.1761748
negative DirectRegulation: TNFAIF	31661000	10.1186/s12985-019-1224-7
negative DirectRegulation: TNFAIF	27779698	10.3892/or.2016.5192
negative DirectRegulation: TNFAIF	25912734	10.1016/j.bbadis.2015.04.018
negative DirectRegulation: TNFAIF	25912734	10.1016/j.bbadis.2015.04.018
negative DirectRegulation: TNFAIF	25912734	10.1016/j.bbadis.2015.04.018
negative DirectRegulation: TNFAIF	29920292	10.1016/j.canlet.2018.06.017
negative DirectRegulation: TNFAIF	33933441	10.1016/j.yexcr.2021.112631
negative DirectRegulation: TNFAIF	24274578	10.1186/1476-4598-12-149
negative DirectRegulation: TNFAIF	22949657	10.1073/pnas.1204525109
negative DirectRegulation: TNFAIF	22949657	10.1073/pnas.1204525109
negative DirectRegulation: TNFAIF	32460619	10.1080/15548627.2020.1761748
negative DirectRegulation: TNFAIF	32460619	10.1080/15548627.2020.1761748
negative DirectRegulation: TNFAIF	32460619	10.1080/15548627.2020.1761748
negative DirectRegulation: TNFAIF	30015980	10.3892/or.2018.6576
negative DirectRegulation: TNFAIF	25912734	10.1016/j.bbadis.2015.04.018
negative DirectRegulation: TNFAIP8L2 ---|		10.1186/s12985-019-1224-7
negative DirectRegulation: TNFAIF	24274578	10.1186/1476-4598-12-149
negative DirectRegulation: TNFAIF	24274578	10.1186/1476-4598-12-149
negative DirectRegulation: TNFAIF	22949657	10.1073/pnas.1204525109
negative DirectRegulation: TNFAIF	32460619	10.1080/15548627.2020.1761748
negative Regulation: PHF20 ---| c	33982773	10.3892/ijo.2021.5218
negative Regulation: PHF20 ---| c	33982773	10.3892/ijo.2021.5218
negative Regulation: PHF20 ---| c	33982773	10.3892/ijo.2021.5218
negative Regulation: PHF20 ---| c	33982773	10.3892/ijo.2021.521810.3892/ijo.2021.5218
negative Regulation: PHF20 ---| c	33982773	10.3892/ijo.2021.521810.3892/ijo.2021.5218
Regulation: ING4 ---> MAPK1	17517644	
Regulation: ING4 ---> MAPK1	17517644	
Regulation: ING4 ---> MAPK1	17517644	
Regulation: ING4 ---> MAPK1	17517644	
Regulation: ING4 ---> MAPK1	17517644	
Regulation: ING4 ---> MAPK1	17517644	
Regulation: ING4 ---> MAPK1	17517644	10.1073/pnas.0703383104
Regulation: ING4 ---> MAPK1		10.1016/j.acthis.2019.06.005
negative Expression: UCA1 ---| BI	33773561	10.31557/APJCP.2021.22.3.947
negative Expression: UCA1 ---| BI	31106532	
Regulation: NOTCH1 ---> AKT1	28104225	10.1016/j.biopsych.2016.12.021
Regulation: NOTCH1 ---> AKT1	33538798	10.1182/blood.2020005734
Regulation: NOTCH1 ---> AKT1	31228468	10.1016/j.cbi.2019.108724
Regulation: NOTCH1 ---> AKT1	29454609	10.1016/j.ejphar.2018.02.020
Regulation: NOTCH1 ---> AKT1	14583609	10.1074/jbc.M309924200
Regulation: NOTCH1 ---> AKT1	28893903	10.1074/jbc.M117.790121
Regulation: NOTCH1 ---> AKT1	28893903	10.1074/jbc.M117.790121
Regulation: NOTCH1 ---> AKT1	28893903	10.1074/jbc.M117.790121
Regulation: NOTCH1 ---> AKT1	28893903	10.1074/jbc.M117.790121
Regulation: NOTCH1 ---> AKT1	28893903	10.1074/jbc.M117.790121
Regulation: NOTCH1 ---> AKT1	28302721	10.1074/jbc.M116.759134
Regulation: NOTCH1 ---> AKT1	28302721	10.1074/jbc.M116.759134
Regulation: NOTCH1 ---> AKT1	23788636	10.1074/jbc.M113.451625

Regulation: NOTCH1 ---> AKT1	23788636	10.1074/jbc.M113.451625
Regulation: NOTCH1 ---> AKT1	23788636	10.1074/jbc.M113.451625
Regulation: NOTCH1 ---> AKT1	23788636	10.1074/jbc.M113.451625
Regulation: NOTCH1 ---> AKT1	23788636	10.1074/jbc.M113.451625
Regulation: NOTCH1 ---> AKT1	23788636	10.1074/jbc.M113.451625
Regulation: NOTCH1 ---> AKT1	23788636	10.1074/jbc.M113.451625
Regulation: NOTCH1 ---> AKT1	26967479	10.1016/j.jid.2015.11.006
Regulation: NOTCH1 ---> AKT1	31437519	10.1016/j.humpath.2019.08.003
Regulation: NOTCH1 ---> AKT1	30629981	10.1016/j.imlet.2019.01.004
Regulation: NOTCH1 ---> AKT1	29908894	10.1016/j.brainresbull.2018.06.005
Regulation: NOTCH1 ---> AKT1	33032902	10.1053/j.semdp.2020.09.010
Regulation: NOTCH1 ---> AKT1	30119264	10.1016/j.biopha.2018.07.018
Regulation: NOTCH1 ---> AKT1	30119264	10.1016/j.biopha.2018.07.018
Regulation: NOTCH1 ---> AKT1	27639599	10.1016/j.phrs.2016.09.001
Regulation: NOTCH1 ---> AKT1	27888063	10.1016/j.biocel.2016.11.013
Regulation: NOTCH1 ---> AKT1	28609656	10.1016/j.ccell.2017.05.006
Regulation: NOTCH1 ---> AKT1	29723707	10.1016/j.dnarep.2018.04.002
Regulation: NOTCH1 ---> AKT1	22465038	10.1016/j.yjmcc.2012.03.007
Regulation: NOTCH1 ---> AKT1	22056375	10.1016/j.lfs.2011.10.012
Regulation: NOTCH1 ---> AKT1	21193018	10.1016/j.bbcan.2010.12.002
Regulation: NOTCH1 ---> AKT1	22293023	10.1016/j.tcm.2011.11.006
Regulation: NOTCH1 ---> AKT1	21993533	10.1016/j.urolonc.2011.07.006
Regulation: NOTCH1 ---> AKT1	21993533	10.1016/j.urolonc.2011.07.006
Regulation: NOTCH1 ---> AKT1	20675210	10.1016/j.scr.2010.06.002
Regulation: NOTCH1 ---> AKT1	24095799	10.1016/j.yexcr.2013.09.019
Regulation: NOTCH1 ---> AKT1	24095799	10.1016/j.yexcr.2013.09.019
Regulation: NOTCH1 ---> AKT1	24140475	10.1016/j.cellsig.2013.09.021
Regulation: NOTCH1 ---> AKT1	24140475	10.1016/j.cellsig.2013.09.021
Regulation: NOTCH1 ---> AKT1	24140475	10.1016/j.cellsig.2013.09.021
Regulation: NOTCH1 ---> AKT1	24140475	10.1016/j.cellsig.2013.09.021
Regulation: NOTCH1 ---> AKT1	24140475	10.1016/j.cellsig.2013.09.021
Regulation: NOTCH1 ---> AKT1	24140475	10.1016/j.cellsig.2013.09.021
Regulation: NOTCH1 ---> AKT1	24140475	10.1016/j.cellsig.2013.09.021
Regulation: NOTCH1 ---> AKT1	24140475	10.1016/j.cellsig.2013.09.021
Regulation: NOTCH1 ---> AKT1	24140475	10.1016/j.cellsig.2013.09.021
Regulation: NOTCH1 ---> AKT1	24140475	10.1016/j.cellsig.2013.09.021
Regulation: NOTCH1 ---> AKT1	24140475	10.1016/j.cellsig.2013.09.021
Regulation: NOTCH1 ---> AKT1	24140475	10.1016/j.cellsig.2013.09.021
Regulation: NOTCH1 ---> AKT1	24529510	10.1016/j.jcpa.2013.11.213
Regulation: NOTCH1 ---> AKT1	25109840	10.1016/j.abb.2014.07.023
Regulation: NOTCH1 ---> AKT1	24775271	10.1016/j.imbio.2014.03.020
Regulation: NOTCH1 ---> AKT1	24775271	10.1016/j.imbio.2014.03.020
Regulation: NOTCH1 ---> AKT1	24939178	10.1016/j.ejcb.2014.05.004
Regulation: NOTCH1 ---> AKT1	25799457	10.1016/j.tips.2015.02.002
Regulation: NOTCH1 ---> AKT1	26850133	10.1016/j.amjsurg.2015.11.029
Regulation: NOTCH1 ---> AKT1	18924608	
Regulation: NOTCH1 ---> AKT1	18830414	
Regulation: NOTCH1 ---> AKT1	20030805	
Regulation: NOTCH1 ---> AKT1	14583609	
Regulation: NOTCH1 ---> AKT1	14583609	
Regulation: NOTCH1 ---> AKT1	20616044	
Regulation: NOTCH1 ---> AKT1	20616044	
Regulation: NOTCH1 ---> AKT1	18369158	
Regulation: NOTCH1 ---> AKT1	19838160	

Regulation: NOTCH1 ---> AKT1 19265851
Regulation: NOTCH1 ---> AKT1 15650752
Regulation: NOTCH1 ---> AKT1 27573203 10.3892/ijo.2016.3598
Regulation: NOTCH1 ---> AKT1 26846632 10.1007/s13365-015-0394-3
Regulation: NOTCH1 ---> AKT1 27548161 10.3390/ijms17081357
Regulation: NOTCH1 ---> AKT1 27719637 10.2174/1381612822666161006160524
Regulation: NOTCH1 ---> AKT1 28241766 10.1186/s12967-017-1151-6
Regulation: NOTCH1 ---> AKT1 27593934 10.1038/onc.2016.275
Regulation: NOTCH1 ---> AKT1 34502291 10.3390/ijms22179382
Regulation: NOTCH1 ---> AKT1 34495871 10.18632/aging.203261
Regulation: NOTCH1 ---> AKT1 34495871 10.18632/aging.203261
Regulation: NOTCH1 ---> AKT1 34465724 10.1038/s41389-021-00348-510.1038/s41389-021-
Regulation: NOTCH1 ---> AKT1 33015852 10.1111/fcp.1260810.1111/fcp.12608
Regulation: NOTCH1 ---> AKT1 34495871 10.18632/aging.20326110.18632/aging.203261
Regulation: NOTCH1 ---> AKT1 18369158 10.1161/CIRCRESAHA.107.16474910.1161/CIRCRESA
Regulation: NOTCH1 ---> AKT1 23788636 10.1074/jbc.M113.45162510.1074/jbc.M113.45162
Regulation: NOTCH1 ---> AKT1 23788636 10.1074/jbc.M113.45162510.1074/jbc.M113.45162
Regulation: NOTCH1 ---> AKT1 10.2298/ABS210423030R
Regulation: NOTCH1 ---> AKT1 10.1016/j.jsps.2021.12.009
Regulation: NOTCH1 ---> AKT1 34995552 10.1016/j.mvr.2021.10430810.1016/j.mvr.2021.1
Regulation: NOTCH1 ---> AKT1 35961388 10.1016/j.yexcr.2022.113305
Regulation: NOTCH1 ---> AKT1 10.7150/ijbs.71694
Regulation: NOTCH1 ---> AKT1 36183290 10.1007/s00018-022-04529-2
Regulation: NOTCH1 ---> AKT1 35704163 10.1007/s12033-022-00476-2
Expression: PTGS2 ---> CD274 24004819 10.1186/bcr3469
Expression: PTGS2 ---> CD274 28096371 10.1073/pnas.161292011410.1073/pnas.161292011
Expression: PTGS2 ---> CD274 28096371 10.1073/pnas.161292011410.1073/pnas.161292011
Expression: PTGS2 ---> CD274
Expression: PTPRD ---> BECN1 33075361 10.1016/j.pharmthera.2020.107707
negative Expression: GPER1 ---| F 26241029 10.1016/j.jsbmb.2015.07.019
negative Expression: GPER1 ---| PTGS2 10.1042/CS20180919
negative Expression: GPER1 ---| F 30705108 10.1042/CS2018091910.1042/CS20180919
negative Expression: GPER1 ---| F 26241029 10.1016/j.jsbmb.2015.07.019
negative Expression: GPER1 ---| F 26241029 10.1016/j.jsbmb.2015.07.019
negative Expression: GPER1 ---| PTGS2 10.1016/j.bbrc.2019.05.045
negative Expression: GPER1 ---| F 28347854 10.1016/j.jsbmb.2017.03.021
negative Regulation: CLDN1 ---| c 29524521 10.1016/j.bbamcr.2018.03.001
negative Regulation: CLDN1 ---| c 29524521 10.1016/j.bbamcr.2018.03.001
negative Regulation: CLDN1 ---| c 32824620 10.3390/ijms21165909
negative Regulation: CLDN1 ---| c 32824620 10.3390/ijms21165909
negative Regulation: CLDN1 ---| c 33828978 10.3389/fonc.2021.62087310.3389/fonc.2021.620
negative Regulation: ABCC1 ---| c 32278855 10.1016/j.ejphar.2020.173105
negative Regulation: ABCC1 ---| c 21219875 10.1016/j.bcp.2010.12.018
negative Regulation: ABCC1 ---| c 11864633 10.1016/S0014-2999(02)01267-0
negative Regulation: ABCC1 ---| c 22771706 10.1016/j.brainres.2012.06.043
negative Regulation: ABCC1 ---| c 30001527 10.1159/000491659
negative Regulation: ABCC1 ---| c 31582906 10.1186/s12935-019-0943-6
negative Regulation: ABCC1 ---| c 34054937 10.1155/2021/5525091
negative Regulation: ABCC1 ---| c 22460269 10.1038/bjc.2012.81
negative Regulation: ABCC1 ---| c 23686318 10.1124/dmd.113.052092
negative Regulation: ABCC1 ---| c 23686318 10.1124/dmd.113.052092
negative Regulation: ABCC1 ---| c 25895710 10.1007/978-3-319-16537-0_7
negative Regulation: ABCC1 ---| chemosensi 10.1186/s12935-019-0943-6
negative Regulation: ABCC1 ---| c 34510251 10.1007/s00280-021-04337-8

positive Regulation: NGF --+> LRF 34073639 10.3390/ijms22115692
positive Regulation: NGF --+> LRF 25233900 10.1186/1471-2202-15-108
positive Regulation: NGF --+> LRF 25233900 10.1186/1471-2202-15-108
positive Regulation: NGF --+> LRF 25233900 10.1186/1471-2202-15-108
positive Regulation: NGF --+> LRF 25233900 10.1186/1471-2202-15-108
positive Regulation: NGF --+> LRF 25233900 10.1186/1471-2202-15-108
positive Regulation: NGF --+> LRF 25233900 10.1186/1471-2202-15-108
positive Regulation: NGF --+> LRF 25233900 10.1186/1471-2202-15-108
positive Regulation: NGF --+> LRF 25233900 10.1186/1471-2202-15-108
positive Regulation: NGF --+> LRF 25233900 10.1186/1471-2202-15-108
positive Regulation: NGF --+> LRF 25233900 10.1186/1471-2202-15-108
positive Regulation: NGF --+> LRF 25233900 10.1186/1471-2202-15-108
positive Regulation: NGF --+> LRF 25233900 10.1186/1471-2202-15-108
positive Regulation: NGF --+> LRF 25233900 10.1186/1471-2202-15-108
positive Regulation: NGF --+> LRF 25233900 10.1186/1471-2202-15-108
positive Regulation: NGF --+> LRF 25233900 10.1186/1471-2202-15-108
positive Regulation: NGF --+> LRF 25233900 10.1186/1471-2202-15-10810.1186/1471-2202-15-
positive Expression: IGFBP4 --+> 35688891 10.1038/s41431-022-01129-3
positive Expression: IGFBP4 --+> 35688891 10.1038/s41431-022-01129-3
positive Expression: IGFBP4 --+> 35688891 10.1038/s41431-022-01129-3
positive Expression: IGFBP4 --+> 35688891 10.1038/s41431-022-01129-3
positive Expression: IGFBP4 --+> 35688891 10.1038/s41431-022-01129-3
positive Expression: IGFBP4 --+> 35688891 10.1038/s41431-022-01129-3
positive Expression: IGFBP4 --+> 35688891 10.1038/s41431-022-01129-3
negative Regulation: TNFAIP8L2 -- 30638932 10.1016/j.bbrc.2019.01.008
negative Regulation: TNFAIP8L2 -- 25912734 10.1016/j.bbadis.2015.04.018
negative Regulation: TNFAIP8L2 -- 28186089 10.1038/cgt.2017.3
negative Regulation: TNFAIP8L2 -- 29568914 10.3892/mmr.2018.8730
negative Regulation: TNFAIP8L2 -- 30015980 10.3892/or.2018.6576
negative Regulation: TNFAIP8L2 -- 26987289 10.1038/cgt.2016.6
negative Regulation: TNFAIP8L2 -- 26987289 10.1038/cgt.2016.6
negative Regulation: TNFAIP8L2 -- 34702807 10.1038/s41419-021-04289-0
positive Expression: AKT1 --+> N(27816488 10.1016/j.canlet.2016.10.039
positive Expression: AKT1 --+> N(18440314 10.1016/j.febslet.2008.04.024
positive Expression: AKT1 --+> N(18440314 10.1016/j.febslet.2008.04.024
positive Expression: AKT1 --+> N(21338656 10.1016/j.bbcan.2011.02.001
positive Expression: AKT1 --+> N(21338656 10.1016/j.bbcan.2011.02.001
positive Expression: AKT1 --+> N(17920278 10.1016/j.tibs.2007.09.002
positive Expression: AKT1 --+> N(26850133 10.1016/j.amjsurg.2015.11.029
positive Expression: AKT1 --+> N(18924608
positive Expression: AKT1 --+> N(18924608
positive Expression: AKT1 --+> N(18924608
positive Expression: AKT1 --+> N(18924608
positive Expression: AKT1 --+> N(18924608
positive Expression: AKT1 --+> N(17158336
positive Expression: AKT1 --+> N(12482957
positive Expression: AKT1 --+> N(27348588 10.1172/JCI80396
positive Expression: AKT1 --+> N(28629170 10.3390/ijms18061295
positive Expression: AKT1 --+> N(29857516 10.3390/ijms19061631
positive Expression: AKT1 --+> N(30486896 10.1186/s13046-018-0972-3
positive Expression: AKT1 --+> N(30636930 10.1186/s12935-018-0714-9
positive Expression: AKT1 --+> N(22258620 10.1038/emboj.2011.443
positive Expression: AKT1 --+> N(22258620 10.1038/emboj.2011.443
positive Expression: AKT1 --+> N(24113181 10.1038/cddis.2013.375

positive Expression: AKT1 --+> N(	24113181	10.1038/cddis.2013.375
positive Expression: AKT1 --+> N(	24113181	10.1038/cddis.2013.375
positive Expression: AKT1 --+> N(	24113181	10.1038/cddis.2013.375
positive Expression: AKT1 --+> N(	24113181	10.1038/cddis.2013.375
positive Expression: AKT1 --+> N(	24113181	10.1038/cddis.2013.375
positive Expression: AKT1 --+> N(	24113181	10.1038/cddis.2013.375
positive Expression: AKT1 --+> N(	21730286	10.1128/MCB.05821-11
positive Expression: AKT1 --+> N(	24113181	10.1038/cddis.2013.37510.1038/cddis.2013.375
positive Expression: AKT1 --+> N(	26117016	10.7534/j.issn.1009-2137.2015.03.01510.7534/j
positive Expression: AKT1 --+> N(	29725418	10.3892/ol.2018.819310.3892/ol.2018.8193
positive Expression: AKT1 --+> N(	33015852	10.1111/fcp.1260810.1111/fcp.12608
positive Expression: AKT1 --+> NOTCH1		10.1016/j.amjsurg.2015.11.029
positive Expression: AKT1 --+> NOTCH1		10.1172/JCI80396
positive Expression: AKT1 --+> NOTCH1		10.1186/s12935-018-0714-9
positive Expression: AKT1 --+> N(	36050315	10.1038/s41419-022-05178-w
Regulation: ADCY8 ---> AKT1	26992697	10.1016/j.alcohol.2015.11.008
Regulation: ADCY8 ---> AKT1		10.1016/j.alcohol.2015.11.008
Expression: RAC1 ---> CD274	29432733	10.1016/j.bbrc.2018.02.073
Expression: RAC1 ---> CD274	30544828	10.3390/ijms19123949
Expression: RAC1 ---> CD274	26176707	10.1111/pcmr.1239210.1111/pcmr.12392
positive Expression: TP53 --+> CF	22819212	10.1016/j.tem.2012.06.007
positive Expression: TP53 --+> CF	32151151	10.1089/ars.2020.8074
positive Expression: TP53 --+> CROT		
positive Regulation: MIR107 --+>	27498977	10.3892/ijo.2016.3628
positive Regulation: MIR107 --+>	27498977	10.3892/ijo.2016.3628
positive Regulation: MIR107 --+>	33901009	10.18632/aging.202908
positive Regulation: MIR107 --+>	33901009	10.18632/aging.202908
positive Regulation: MIR107 --+>	33901009	10.18632/aging.202908
positive Regulation: MIR107 --+>	33901009	10.18632/aging.202908
positive Regulation: MIR107 --+>	33901009	10.18632/aging.202908
positive Regulation: MIR107 --+>	25400821	
positive Regulation: MIR107 --+>	28979809	
positive Regulation: MIR107 --+>	33901009	10.18632/aging.20290810.18632/aging.202908
Expression: TP53 ---> BECN1	33743351	10.1016/j.bbrc.2021.03.034
Expression: TP53 ---> BECN1	36567312	10.1186/s12935-022-02831-4
Expression: TP53 ---> BECN1	29410006	10.1016/j.canlet.2018.01.061
Expression: TP53 ---> BECN1	29775897	10.1016/j.biopha.2018.04.013
Expression: TP53 ---> BECN1	29186924	10.3390/ijms18122541
Expression: TP53 ---> BECN1	30132535	10.3892/or.2018.6648
Expression: TP53 ---> BECN1	34065345	10.3390/ijms22105377
Expression: TP53 ---> BECN1	30132535	10.3892/or.2018.664810.3892/or.2018.6648
Expression: TP53 ---> BECN1	25208472	10.1111/jcmm.1238610.1111/jcmm.12386
negative Expression: PTEN ---| AI	29339153	10.1016/j.bbrc.2018.01.061
negative Expression: PTEN ---| AI	28844858	10.1016/j.cbi.2017.08.014
negative Expression: PTEN ---| AI	33647255	10.1016/j.ejphar.2021.173983
negative Expression: PTEN ---| AI	31067440	10.1016/j.yexmp.2019.05.002
negative Expression: PTEN ---| AI	32919989	10.1016/j.exer.2020.108224
negative Expression: PTEN ---| AI	32919989	10.1016/j.exer.2020.108224
negative Expression: PTEN ---| AI	16170201	10.1074/jbc.M504155200
negative Expression: PTEN ---| AI	16963781	10.1074/jbc.M606450200
negative Expression: PTEN ---| AI	16963781	10.1074/jbc.M606450200
negative Expression: PTEN ---| AI	30951746	10.1016/j.lfs.2019.04.001
negative Expression: PTEN ---| AI	31004658	10.1016/j.lfs.2019.04.040
negative Expression: PTEN ---| AI	28095306	10.1016/j.taap.2017.01.005

negative Expression: PTEN ---| AK 26801100 10.1016/j.dci.2016.01.004
negative Expression: PTEN ---| AK 32524999 10.1016/j.bbamcr.2020.118770
negative Expression: PTEN ---| AK 27818223 10.1016/j.tox.2016.11.004
negative Expression: PTEN ---| AK 32931887 10.1016/j.canlet.2020.09.010
negative Expression: PTEN ---| AK 29288045 10.1016/j.neulet.2017.12.049
negative Expression: PTEN ---| AKT1 10.1016/j.jacc.2014.06.407
negative Expression: PTEN ---| AK 29807230 10.1016/j.biopha.2018.04.028
negative Expression: PTEN ---| AK 26459109 10.1016/j.nbd.2015.10.002
negative Expression: PTEN ---| AK 28306514 10.1016/j.molcel.2017.02.019
negative Expression: PTEN ---| AK 30195065 10.1016/j.biocel.2018.09.003
negative Expression: PTEN ---| AKT1 10.1016/j.pan.2017.05.035
negative Expression: PTEN ---| AK 30248461 10.1016/j.jprot.2018.09.005
negative Expression: PTEN ---| AK 28647188 10.1016/j.apjtm.2017.05.007
negative Expression: PTEN ---| AK 20079711 10.1016/j.bbrc.2010.01.045
negative Expression: PTEN ---| AK 20163786 10.1016/j.biopsych.2009.12.031
negative Expression: PTEN ---| AK 21111719 10.1016/j.brainres.2010.11.062
negative Expression: PTEN ---| AK 17540362 10.1016/j.ydbio.2007.04.051
negative Expression: PTEN ---| AK 19628092 10.1016/j.surg.2009.04.007
negative Expression: PTEN ---| AK 18662848 10.1016/j.canlet.2008.06.018
negative Expression: PTEN ---| AK 18996641 10.1016/j.canlet.2008.09.019
negative Expression: PTEN ---| AK 22266095 10.1016/j.canlet.2012.01.003
negative Expression: PTEN ---| AK 21481725 10.1016/j.ijrobp.2010.12.049
negative Expression: PTEN ---| AK 12946479 10.1016/S0960-7404(03)00037-9
negative Expression: PTEN ---| AK 22922290 10.1016/j.bbagen.2012.08.006
negative Expression: PTEN ---| AK 23137704 10.1016/j.febslet.2012.10.034
negative Expression: PTEN ---| AK 23142288 10.1016/j.canlet.2012.10.037
negative Expression: PTEN ---| AK 23458710 10.1016/j.neuroscience.2013.02.046
negative Expression: PTEN ---| AK 23817095 10.1016/j.ijbiomac.2013.06.027
negative Expression: PTEN ---| AK 25771877 10.1016/j.mrfmmm.2014.08.008
negative Expression: PTEN ---| AK 19895837 10.1016/j.advenzreg.2009.10.016
negative Expression: PTEN ---| AK 25907832 10.1016/j.ajpath.2015.01.025
negative Expression: PTEN ---| AK 25542847 10.1016/j.mce.2014.12.005
negative Expression: PTEN ---| AK 26253360 10.1016/j.critrevonc.2015.07.005
negative Expression: PTEN ---| AK 25737250 10.1016/j.biocel.2015.02.015
negative Expression: PTEN ---| AK 26045339 10.1016/j.molonc.2015.04.014
negative Expression: PTEN ---| AK 26647161 10.1016/j.survophthal.2015.11.006
negative Expression: PTEN ---| AK 26647161 10.1016/j.survophthal.2015.11.006
negative Expression: PTEN ---| AK 21108789
negative Expression: PTEN ---| AK 19115995
negative Expression: PTEN ---| AK 16963781
negative Expression: PTEN ---| AK 16963781
negative Expression: PTEN ---| AK 16170201
negative Expression: PTEN ---| AK 14561749
negative Expression: PTEN ---| AK 19075230
negative Expression: PTEN ---| AK 18032782
negative Expression: PTEN ---| AK 16787946
negative Expression: PTEN ---| AK 14747659
negative Expression: PTEN ---| AK 10698713
negative Expression: PTEN ---| AK 20436671
negative Expression: PTEN ---| AK 18523065
negative Expression: PTEN ---| AK 26984887 10.1530/ERC-16-0039
negative Expression: PTEN ---| AK 27572739 10.3892/or.2016.5030
negative Expression: PTEN ---| AK 27460260 10.1089/scd.2016.0120
negative Expression: PTEN ---| AK 27465551 10.1007/s13277-016-5155-2

negative Expression: PTEN ---| AK 28289760 10.1007/s00018-017-2500-6
negative Expression: PTEN ---| AK 28495999 10.1124/mol.116.108035
negative Expression: PTEN ---| AK 28718798 10.3390/ijms18071560
negative Expression: PTEN ---| AK 28135838 10.1089/ars.2016.6851
negative Expression: PTEN ---| AK 34554926 10.18632/aging.203553
negative Expression: PTEN ---| AK 34307670 10.1155/2021/759097610.1155/2021/7590976
negative Expression: PTEN ---| AK 18231751 10.1007/s11596-007-0625-910.1007/s11596-007-0
negative Expression: PTEN ---| AK 20347952 10.1016/j.exger.2010.03.01510.1016/j.exger.20
negative Expression: PTEN ---| AK 21544242 10.1371/journal.pone.001913910.1371/journal.p
negative Expression: PTEN ---| AK 22678116 10.1093/carcin/bgs19810.1093/carcin/bgs198
negative Expression: PTEN ---| AK 24339862 10.1371/journal.pone.007792210.1371/journal.p
negative Expression: PTEN ---| AK 25009983 10.1371/journal.pone.010211810.1371/journal.p
negative Expression: PTEN ---| AK 34659902
negative Expression: PTEN ---| AKT1 10.1016/j.mrfmmm.2014.08.008
negative Expression: PTEN ---| AKT1 10.1016/j.critrevonc.2015.07.005
negative Expression: PTEN ---| AKT1 10.1530/ERC-16-0039
negative Expression: PTEN ---| AK 29192321 10.3892/or.2017.6099
negative Expression: PTEN ---| AK 28733702 10.1007/s10637-017-0494-4
negative Expression: PTEN ---| AK 29412697 10.1089/cbr.2017.2306
negative Expression: PTEN ---| AK 29412697 10.1089/cbr.2017.2306
negative Expression: PTEN ---| AK 29717662 10.1615/CritRevImmunol.2018025184
negative Expression: PTEN ---| AK 29687845 10.26355/eurrev-201804-14717
negative Expression: PTEN ---| AK 29767245 10.3892/or.2018.6439
negative Expression: PTEN ---| AKT1 10.3727/096504017X15061902533715
negative Expression: PTEN ---| AK 29713056 10.1038/s41388-018-0261-9
negative Expression: PTEN ---| AK 30089904 10.1038/s41586-018-0409-3
negative Expression: PTEN ---| AK 29953987 10.1159/000490256
negative Expression: PTEN ---| AK 30134988 10.1186/s13046-018-0795-2
negative Expression: PTEN ---| AK 29936638 10.1007/s11011-018-0276-5
negative Expression: PTEN ---| AK 30106086 10.3892/ijmm.2018.3814
negative Expression: PTEN ---| AK 29941984 10.1038/s41434-018-0020-0
negative Expression: PTEN ---| AK 30280788 10.26355/eurrev_201809_15940
negative Expression: PTEN ---| AK 30136167 10.1007/s10571-018-0615-4
negative Expression: PTEN ---| AK 30468465 10.26355/eurrev-201811-16256
negative Expression: PTEN ---| AK 30338819 10.26355/eurrev_201810_16062
negative Expression: PTEN ---| AK 30338819 10.26355/eurrev_201810_16062
negative Expression: PTEN ---| AK 30418964 10.12659/MSM.913307
negative Expression: PTEN ---| AK 30418964 10.12659/MSM.913307
negative Expression: PTEN ---| AK 30365133 10.3892/or.2018.6823
negative Expression: PTEN ---| AKT1 10.1038/s41419-019-1547-8
negative Expression: PTEN ---| AKT1 10.26355/eurrev_201906_18084
negative Expression: PTEN ---| AK 31088038 10.33594/000000101
negative Expression: PTEN ---| AK 31173315 10.26355/eurrev_201905_1794810.26355/eurrev_2
negative Expression: PTEN ---| AK 30094104
negative Expression: PTEN ---| AK 30680530 10.1007/s12079-018-00504-410.1007/s12079-018-
negative Expression: PTEN ---| AK 31124340 10.3349/ymj.2019.60.6.561
negative Expression: PTEN ---| AK 31415720 10.1139/bcb-2018-036410.1139/bcb-2018-0364
negative Expression: PTEN ---| AK 22172720 10.1016/j.ccr.2011.10.030
negative Expression: PTEN ---| AK 22549727 10.1101/gad.187252.112
negative Expression: PTEN ---| AK 22989520 10.1124/mol.112.081539
negative Expression: PTEN ---| AK 21360018 10.1007/s12032-011-9867-2
negative Expression: PTEN ---| AK 21360018 10.1007/s12032-011-9867-2
negative Expression: PTEN ---| AK 22607099 10.1089/ars.2011.4322
negative Expression: PTEN ---| AK 22011761 10.3892/mmr.2011.634

negative Expression: PTEN ---|	AK	19728186	10.1007/s12032-009-9295-8
negative Expression: PTEN ---|	AK	21108789	10.1186/1476-4598-9-305
negative Expression: PTEN ---|	AK	21492444	10.1186/bcr2865
negative Expression: PTEN ---|	AK	22298084	10.1007/s00395-012-0248-6
negative Expression: PTEN ---|	AK	22298084	10.1007/s00395-012-0248-6
negative Expression: PTEN ---|	AK	22298084	10.1007/s00395-012-0248-6
negative Expression: PTEN ---|	AK	22796579	10.1161/ATVBAHA.111.241463
negative Expression: PTEN ---|	AK	23754155	10.3892/ijmm.2013.1411
negative Expression: PTEN ---|	AK	22370645	10.1038/onc.2012.37
negative Expression: PTEN ---|	AK	20436671	10.1371/journal.pone.0010350
negative Expression: PTEN ---|	AK	22427991	10.1371/journal.pone.0033211
negative Expression: PTEN ---|	AK	23144940	10.1371/journal.pone.0048715
negative Expression: PTEN ---|	AK	23675511	10.1371/journal.pone.0063857
negative Expression: PTEN ---|	AK	23597193	10.2174/15680096113139990031
negative Expression: PTEN ---|	AK	24449917	10.1073/pnas.1318185111
negative Expression: PTEN ---|	AK	24312220	10.1371/journal.pone.0080429
negative Expression: PTEN ---|	AK	24971153	10.1155/2014/724670
negative Expression: PTEN ---|	AK	24206455	10.1089/ars.2013.5718
negative Expression: PTEN ---|	AK	25009983	10.1371/journal.pone.0102118
negative Expression: PTEN ---|	AK	25009983	10.1371/journal.pone.0102118
negative Expression: PTEN ---|	AK	25084828	10.1186/s13046-014-0062-0
negative Expression: PTEN ---|	AK	25600527	
negative Expression: PTEN ---|	AK	25254897	10.1055/s-0034-1385877
negative Expression: PTEN ---|	AK	21836055	10.1152/ajpgi.00178.2011
negative Expression: PTEN ---|	AK	25683168	10.3892/or.2015.3804
negative Expression: PTEN ---|	AK	24854844	10.1159/000358719
negative Expression: PTEN ---|	AK	21536651	10.1128/MCB.01323-10
negative Expression: PTEN ---|	AK	25881601	10.4103/0366-6999.155092
negative Expression: PTEN ---|	AK	26243299	10.1186/s13046-015-0192-z
negative Expression: PTEN ---|	AK	26390244	10.1038/nm.3955
negative Expression: PTEN ---|	AKT1		10.1042/CS20150496
negative Expression: PTEN ---|	AK	26848951	10.1371/journal.pone.0148508
negative Expression: PTEN ---|	AK	26928575	10.1371/journal.pcbi.1004787
negative Expression: PTEN ---|	AK	27747225	10.1155/2016/2860516
negative Expression: PTEN ---|	AK	11875759	10.1086/340026
negative Expression: PTEN ---|	AK	21627959	10.1016/j.bbrc.2011.05.074
negative Expression: PTEN ---|	AKT1		10.1016/S0022-5347(01)69142-0
negative Expression: PTEN ---|	AKT1		10.1097/01.ju.0000067603.56853.0f
negative Expression: PTEN ---|	AK	20600153	10.1016/j.lfs.2010.06.006
negative Expression: PTEN ---|	AKT1		10.1016/j.urolonc.2011.03.018
negative Expression: PTEN ---|	AK	23262204	10.1016/j.ygyno.2012.12.022
negative Expression: PTEN ---|	AK	22297255	10.1016/j.clml.2011.12.002
negative Expression: PTEN ---|	AK	23524257	10.1016/j.bbrc.2013.03.018
negative Expression: PTEN ---|	AK	21795074	10.1016/j.urolonc.2011.03.018
negative Expression: PTEN ---|	AKT1		10.1016/j.bbamcr.2014.11.004
negative Expression: PTEN ---|	AK	25447541	10.1016/j.bbamcr.2014.11.004
negative Expression: PTEN ---|	AK	25440090	10.1016/j.molmed.2014.10.009
negative Expression: PTEN ---|	AK	19276401	
negative Expression: PTEN ---|	AK	19623194	
negative Expression: PTEN ---|	AK	19039330	
negative Expression: PTEN ---|	AK	11103942	
negative Expression: PTEN ---|	AK	16055704	
negative Expression: PTEN ---|	AK	22505453	10.1172/JCI57477
negative Expression: PTEN ---|	AK	22962422	10.1210/jc.2012-1991

negative Expression: PTEN ---|	AK20605778	10.1074/jbc.M110.117093
negative Expression: PTEN ---|	AK21663495	10.1089/jmf.2010.1428
negative Expression: PTEN ---|	AK22556157	10.1165/rcmb.2012-0025OCon
negative Expression: PTEN ---|	AK20857258	10.1007/s13277-010-0111-z
negative Expression: PTEN ---|	AK21637289	10.1038/cdd.2011.73
negative Expression: PTEN ---|	AK23419514	10.1038/cr.2013.27
negative Expression: PTEN ---|	AK23912224	10.3892/mmr.2013.1612
negative Expression: PTEN ---|	AK23909904	10.1089/jmf.2012.2534
negative Expression: PTEN ---|	AK23909904	10.1089/jmf.2012.2534
negative Expression: PTEN ---|	AK24026200	10.3892/ijmm.2013.1487
negative Expression: PTEN ---|	AK22509396	10.1371/journal.pone.0035153
negative Expression: PTEN ---|	AK23874880	10.1371/journal.pone.0069104
negative Expression: PTEN ---|	AK24312220	10.1371/journal.pone.0080429
negative Expression: PTEN ---|	AK24736893	10.3892/mmr.2014.2155
negative Expression: PTEN ---|	AK24850856	10.1242/dev.105130
negative Expression: PTEN ---|	AK25816150	10.3892/mmr.2015.3509
negative Expression: PTEN ---|	AK25816150	10.3892/mmr.2015.3509
negative Expression: PTEN ---|	AK25707849	10.3892/mmr.2015.3380
negative Expression: PTEN ---|	AK26000878	10.1371/journal.pone.0126149
negative Expression: PTEN ---|	AK10958682	
negative Expression: PTEN ---|	AK15851405	
negative Expression: PTEN ---|	AK17483362	10.1158/0008-5472.CAN-06-3398
negative Expression: PTEN ---|	AK17826033	
negative Expression: PTEN ---|	AK17983517	
negative Expression: PTEN ---|	AK19726333	
negative Expression: PTEN ---|	AK22561258	10.1016/j.yexmp.2012.04.019
negative Expression: PTEN ---|	AK22556157	10.1165/rcmb.2012-0025OC
negative Expression: PTEN ---|	AK22982856	10.1016/j.trim.2012.09.001
negative Expression: PTEN ---|	AK24425042	10.3892/mmr.2014.1892
negative Expression: PTEN ---|	AKT1	
negative Expression: PTEN ---|	AK26531778	10.3892/or.2015.4375
negative Expression: PTEN ---|	AK27004535	10.3892/or.2016.4694
negative Expression: PTEN ---|	AK27004535	10.3892/or.2016.4694
negative Expression: PTEN ---|	AK27004535	10.3892/or.2016.4694
negative Expression: PTEN ---|	AK27955380	
negative Expression: PTEN ---|	AK1130	10.1016/j.apjtm.2017.05.007
negative Expression: PTEN ---|	AK29412697	10.1089/cbr.2017.230610.1089/cbr.2017.2306
negative Expression: PTEN ---|	AK30229821	10.26355/eurrev_201809_1581010.26355/eurrev_2
negative Expression: PTEN ---|	AK30583634	10.19540/j.cnki.cjcmm.20180601.00110.19540/j.
negative Expression: PTEN ---|	AK30979496	10.1016/j.bbrc.2019.04.03410.1016/j.bbrc.2019
negative Expression: PTEN ---|	AKT1	10.3892/or.2020.7457
negative Expression: PTEN ---|	AKT1	10.12659/MSM.919654
negative Expression: PTEN ---|	AK32086725	10.1007/s11011-020-00544-z
negative Expression: PTEN ---|	AKT1	10.1007/s10528-020-09947-0
negative Expression: PTEN ---|	AKT1	10.1042/BSR20201901
negative Expression: PTEN ---|	AK33670518	10.3390/biom1102030410.3390/biom11020304
negative Expression: PTEN ---|	AK33202406	10.1159/000509052
negative Expression: PTEN ---|	AK33918290	10.3390/molecules26072029
negative Expression: PTEN ---|	AK33867846	10.7150/ijbs.56933
negative Expression: PTEN ---|	AK34006834	10.1038/s41419-021-03735-3
negative Expression: PTEN ---|	AK33813678	10.1007/s12033-021-00322-x
negative Expression: PTEN ---|	AK34135128	10.18632/aging.203154
negative Expression: PTEN ---|	AK34344439	10.1186/s13058-021-01457-0
negative Expression: PTEN ---|	AK34633595	10.1007/s11011-021-00744-1

negative Expression: PTEN ---| AK 34633595 10.1007/s11011-021-00744-1
negative Expression: PTEN ---| AK 34635817 10.1038/s41418-021-00861-5
negative Expression: PTEN ---| AK 34368895 10.1007/s00709-021-01695-1
negative Expression: PTEN ---| AK 35397519 10.1186/s12891-022-05266-0
negative Expression: PTEN ---| AK 35774615 10.3389/fphar.2022.90769510.3389/fphar.2022.9
negative Expression: PTEN ---| AKT1 10.1016/j.chmed.2022.08.001
negative Expression: PTEN ---| AK 36217693 10.1111/jcpe.13730
negative Expression: PTEN ---| AK 36381235 10.1158/2767-9764.crc-22-0024
negative Expression: PTEN ---| AK 36330380 10.1155/2022/4766992
negative Expression: PTEN ---| AKT1 10.32604/or.2022.026459
negative Expression: PTEN ---| AK 36547074 10.3390/cimb44120413
negative Expression: PTEN ---| AK 36547074 10.3390/cimb44120413
Expression: AKT1 ---> PTEN 31493871 10.1016/j.bbrc.2019.08.164
Expression: AKT1 ---> PTEN 28583434 10.1016/j.chemphyslip.2017.05.012
Expression: AKT1 ---> PTEN 26764128 10.1016/j.ydbio.2015.12.027
Expression: AKT1 ---> PTEN 20100827 10.1074/jbc.M109.091637
Expression: AKT1 ---> PTEN 25873394 10.1074/jbc.M115.641407
Expression: AKT1 ---> PTEN 22184110 10.1074/jbc.M111.246397
Expression: AKT1 ---> PTEN 12743124 10.1074/jbc.M303318200
Expression: AKT1 ---> PTEN 32610163 10.1016/j.lfs.2020.118022
Expression: AKT1 ---> PTEN 28095306 10.1016/j.taap.2017.01.005
Expression: AKT1 ---> PTEN 32045588 10.1016/j.taap.2020.114918
Expression: AKT1 ---> PTEN 10.1016/j.ygyno.2016.04.198
Expression: AKT1 ---> PTEN 32524999 10.1016/j.bbamcr.2020.118770
Expression: AKT1 ---> PTEN 32805344 10.1016/j.fct.2020.111684
Expression: AKT1 ---> PTEN 29136769 10.1016/j.biopha.2017.11.014
Expression: AKT1 ---> PTEN 29567542 10.1016/j.biopha.2018.03.088
Expression: AKT1 ---> PTEN 29438745 10.1016/j.vascn.2018.02.005
Expression: AKT1 ---> PTEN 33606974 10.1016/j.molcel.2021.01.033
Expression: AKT1 ---> PTEN 33606974 10.1016/j.molcel.2021.01.033
Expression: AKT1 ---> PTEN 27210502 10.1016/j.biocel.2016.05.013
Expression: AKT1 ---> PTEN 28501723 10.1016/j.advms.2016.11.011
Expression: AKT1 ---> PTEN 27843613 10.1136/esmoopen-2016-000060
Expression: AKT1 ---> PTEN 29514093 10.1016/j.celrep.2018.02.044
Expression: AKT1 ---> PTEN 31201145 10.1016/j.ebiom.2019.05.055
Expression: AKT1 ---> PTEN 16169518 10.1016/j.bbrc.2005.08.256
Expression: AKT1 ---> PTEN 19000654 10.1016/j.bbrc.2008.10.157
Expression: AKT1 ---> PTEN 21320485 10.1016/j.yexcr.2011.02.003
Expression: AKT1 ---> PTEN 17239858 10.1016/j.febslet.2006.12.055
Expression: AKT1 ---> PTEN 17681183 10.1053/j.gastro.2007.05.022
Expression: AKT1 ---> PTEN 21756853 10.1016/j.jhep.2011.05.023
Expression: AKT1 ---> PTEN 11115729 10.1016/S0301-0082(00)00052-6
Expression: AKT1 ---> PTEN 21193018 10.1016/j.bbcan.2010.12.002
Expression: AKT1 ---> PTEN 17317031 10.1016/j.prp.2007.01.003
Expression: AKT1 ---> PTEN 17826953 10.1016/j.cellsig.2007.07.020
Expression: AKT1 ---> PTEN 10.1016/S0959-437X(99)00041-6
Expression: AKT1 ---> PTEN 16697215 10.1016/j.semcancer.2006.03.009
Expression: AKT1 ---> PTEN 19962668 10.1016/j.ccr.2009.10.014
Expression: AKT1 ---> PTEN 23603119 10.1016/j.molcel.2013.03.015
Expression: AKT1 ---> PTEN 24183943 10.1016/j.bbcan.2013.10.003
Expression: AKT1 ---> PTEN 24412320 10.1016/j.cca.2014.01.001
Expression: AKT1 ---> PTEN 26216598 10.1016/j.smim.2015.07.001
Expression: AKT1 ---> PTEN 26774676 10.1016/j.cytogfr.2015.12.004
Expression: AKT1 ---> PTEN 20100827

Expression: AKT1 ---> PTEN	19201817	
Expression: AKT1 ---> PTEN	12743124	
Expression: AKT1 ---> PTEN	19057511	
Expression: AKT1 ---> PTEN	19057511	
Expression: AKT1 ---> PTEN	19057511	
Expression: AKT1 ---> PTEN	19057511	
Expression: AKT1 ---> PTEN	19057511	
Expression: AKT1 ---> PTEN	27349269	10.3892/or.2016.4907
Expression: AKT1 ---> PTEN	26568302	10.1038/onc.2015.402
Expression: AKT1 ---> PTEN	26259914	10.1189/jlb.4A0415-171RR
Expression: AKT1 ---> PTEN	28570839	10.1089/dna.2017.3712
Expression: AKT1 ---> PTEN	27758700	10.2174/1381612822666161018155247
Expression: AKT1 ---> PTEN	29207060	10.3892/mmr.2017.8123
Expression: AKT1 ---> PTEN	29568880	10.3892/ijmm.2018.3568
Expression: AKT1 ---> PTEN	29693151	10.3892/or.2018.6380
Expression: AKT1 ---> PTEN	29966227	10.3390/ijms19071907
Expression: AKT1 ---> PTEN	29969760	10.1159/000491465
Expression: AKT1 ---> PTEN	30106086	10.3892/ijmm.2018.3814
Expression: AKT1 ---> PTEN	28233182	10.1007/164_2017_4
Expression: AKT1 ---> PTEN	30730015	10.1007/s10620-019-5461-9
Expression: AKT1 ---> PTEN	31270502	10.1038/s41591-019-0499-y
Expression: AKT1 ---> PTEN	31524278	10.3892/or.2019.7259
Expression: AKT1 ---> PTEN	31511778	10.1155/2019/4301238
Expression: AKT1 ---> PTEN	30733438	10.1038/s41467-019-08481-x
Expression: AKT1 ---> PTEN	32917956	10.1038/s41388-020-01450-5
Expression: AKT1 ---> PTEN	33549103	10.1186/s12935-021-01793-3
Expression: AKT1 ---> PTEN	33843453	10.1080/19336918.2021.1909899
Expression: AKT1 ---> PTEN	33843453	10.1080/19336918.2021.1909899
Expression: AKT1 ---> PTEN	22552728	10.3892/mmr.2012.889
Expression: AKT1 ---> PTEN	22956257	10.1055/s-0032-1311644
Expression: AKT1 ---> PTEN	23223894	10.1242/jcs.093765
Expression: AKT1 ---> PTEN	23314408	10.3892/ijo.2013.1765
Expression: AKT1 ---> PTEN		10.1016/j.clgc.2021.08.004
Expression: AKT1 ---> PTEN		10.1016/j.clgc.2021.08.004
Expression: AKT1 ---> PTEN	32959108	10.1007/s00403-020-02139-1
Expression: AKT1 ---> PTEN	34273059	10.1007/s11010-021-04221-2
Expression: AKT1 ---> PTEN	34402627	10.1302/2046-3758.108.BJR-2020-0127.R210.1302
Expression: AKT1 ---> PTEN	18414037	10.4161/cc.7.8.5753
Expression: AKT1 ---> PTEN	21756853	10.1016/j.jhep.2011.05.02310.1016/j.jhep.2011
Expression: AKT1 ---> PTEN	21952748	10.1007/s11010-011-1082-010.1007/s11010-011-1
Expression: AKT1 ---> PTEN	22301279	10.1093/carcin/bgs02210.1093/carcin/bgs022
Expression: AKT1 ---> PTEN	23129115	10.1002/jbt.2145110.1002/jbt.21451
Expression: AKT1 ---> PTEN		10.1016/j.ebiom.2019.05.055
Expression: AKT1 ---> PTEN		10.1016/j.bbrc.2019.08.164
Expression: AKT1 ---> PTEN	20679434	
Expression: AKT1 ---> PTEN		10.1189/jlb.4A0415-171RR
Expression: AKT1 ---> PTEN		10.1007/s10620-019-5461-9
Expression: AKT1 ---> PTEN		10.1038/s41591-019-0499-y
Expression: AKT1 ---> PTEN		10.3892/or.2019.7259
Expression: AKT1 ---> PTEN		10.1155/2019/4301238
Expression: AKT1 ---> PTEN		10.1038/s41467-019-08481-x
Expression: AKT1 ---> PTEN	20679434	10.1083/jcb.201003004
Expression: AKT1 ---> PTEN	20940316	10.1242/dmm.004671
Expression: AKT1 ---> PTEN	21870170	10.1007/s12020-011-9524-8

Expression: AKT1 ---> PTEN	23339168	10.1161/HYPERTENSIONAHA.111.00514
Expression: AKT1 ---> PTEN	21996732	10.1038/onc.2011.419
Expression: AKT1 ---> PTEN	23392169	10.1038/cddis.2013.3
Expression: AKT1 ---> PTEN	24025251	10.4161/cbt.26234
Expression: AKT1 ---> PTEN	21998680	10.1371/journal.pone.0025676
Expression: AKT1 ---> PTEN	24113181	10.1038/cddis.2013.375
Expression: AKT1 ---> PTEN	24113181	10.1038/cddis.2013.375
Expression: AKT1 ---> PTEN		10.1074/jbc.M114.607846
Expression: AKT1 ---> PTEN	25416384	10.1152/ajplung.00242.2014
Expression: AKT1 ---> PTEN	23255786	10.1128/JVI.02319-12
Expression: AKT1 ---> PTEN	25799148	10.1371/journal.pone.0121547
Expression: AKT1 ---> PTEN	25826666	10.1080/15476286.2015.1017205
Expression: AKT1 ---> PTEN		10.1691/ph.2015.4853
Expression: AKT1 ---> PTEN		10.1074/jbc.M115.641407
Expression: AKT1 ---> PTEN	26183061	10.1038/ncomms8769
Expression: AKT1 ---> PTEN	26265437	10.1186/s12943-015-0423-0
Expression: AKT1 ---> PTEN	26535660	10.4238/2015.October.28.7
Expression: AKT1 ---> PTEN	26544868	10.1371/journal.pone.0142373
Expression: AKT1 ---> PTEN		10.3233/JAD-150763
Expression: AKT1 ---> PTEN	25527506	10.1074/jbc.M114.60784610.1074/jbc.M114.60784
Expression: AKT1 ---> PTEN	29725418	10.3892/ol.2018.819310.3892/ol.2018.8193
Expression: AKT1 ---> PTEN	21111719	10.1016/j.brainres.2010.11.06210.1016/j.brain
Expression: AKT1 ---> PTEN	21297666	10.1038/onc.2011.610.1038/onc.2011.6
Expression: AKT1 ---> PTEN	22021428	10.1093/hmg/ddr49110.1093/hmg/ddr491
Expression: AKT1 ---> PTEN	31240704	10.1002/1873-3468.1350510.1002/1873-3468.1350
Expression: AKT1 ---> PTEN	22869142	10.1038/onc.2012.342
Expression: AKT1 ---> PTEN	12077256	
Expression: AKT1 ---> PTEN	22858201	10.1016/j.nut.2012.03.02010.1016/j.nut.2012.0
Expression: AKT1 ---> PTEN	23989801	10.1007/s00395-013-0373-x10.1007/s00395-013-0
Expression: AKT1 ---> PTEN	24975844	10.1016/j.cellsig.2014.06.00710.1016/j.cellsi
Expression: AKT1 ---> PTEN	25810340	10.1002/path.4538
Expression: AKT1 ---> PTEN	26183061	10.1038/ncomms8769
Expression: AKT1 ---> PTEN	26535660	10.4238/2015.October.28.7
Expression: AKT1 ---> PTEN	21320485	10.1016/j.yexcr.2011.02.003
Expression: AKT1 ---> PTEN	20691487	10.1016/j.tibtech.2010.06.005
Expression: AKT1 ---> PTEN	17169462	10.1016/j.neuint.2006.10.010
Expression: AKT1 ---> PTEN	19057511	
Expression: AKT1 ---> PTEN	20940316	10.1242/dmm.004671
Expression: AKT1 ---> PTEN	20712893	10.1186/1476-4598-9-216
Expression: AKT1 ---> PTEN	21709207	10.1161/HYPERTENSIONAHA.111.172189
Expression: AKT1 ---> PTEN	23271053	10.1096/fj.12-221994
Expression: AKT1 ---> PTEN	25389442	10.1155/2014/628712
Expression: AKT1 ---> PTEN	12535639	
Expression: AKT1 ---> PTEN	26568302	10.1038/onc.2015.402
Expression: AKT1 ---> PTEN		10.1042/CS20150405
Expression: AKT1 ---> PTEN		
Expression: AKT1 ---> PTEN	31592121	10.15171/apb.2019.05610.15171/apb.2019.056
Expression: AKT1 ---> PTEN	32281513	10.1177/153303382091795910.1177/1533033820917
Expression: AKT1 ---> PTEN	32889668	10.1007/s10571-020-00945-9
Expression: AKT1 ---> PTEN	32889668	10.1007/s10571-020-00945-9
Expression: AKT1 ---> PTEN	34958114	10.3892/or.2021.824810.3892/or.2021.8248
Expression: AKT1 ---> PTEN	35408777	10.3390/ijms23073416
Expression: AKT1 ---> PTEN	35682571	10.3390/ijms23115892
Expression: AKT1 ---> PTEN	36133813	10.3389/fphar.2022.985084

Expression: AKT1 ---> PTEN	35704163	10.1007/s12033-022-00476-2
Expression: AKT1 ---> PTEN	36402791	10.1038/s41467-022-34831-3
ProtModification: PTEN ---> AKT1	12010818	10.1182/blood.V99.11.4138
ProtModification: PTEN ---> AKT1	17142836	10.1074/jbc.M604195200
ProtModification: PTEN ---> AKT1	26324707	10.1074/jbc.M115.684746
ProtModification: PTEN ---> AKT1	21953448	10.1074/jbc.M111.270884
ProtModification: PTEN ---> AKT1	31884408	10.1016/j.ejmech.2019.111972
ProtModification: PTEN ---> AKT1	33189865	10.1016/j.mce.2020.111080
ProtModification: PTEN ---> AKT1	33189865	10.1016/j.mce.2020.111080
ProtModification: PTEN ---> AKT1	29136769	10.1016/j.biopha.2017.11.014
ProtModification: PTEN ---> AKT1	32201226	10.1016/j.jaut.2020.102440
ProtModification: PTEN ---> AKT1	15851405	10.1093/annonc/mdi182
ProtModification: PTEN ---> AKT1	27017291	10.1016/j.ejca.2016.02.016
ProtModification: PTEN ---> AKT1	31492635	10.1016/j.molcel.2019.08.006
ProtModification: PTEN ---> AKT1	33098764	10.1016/j.molcel.2020.10.003
ProtModification: PTEN ---> AKT1	27297622	10.1016/j.biocel.2016.06.006
ProtModification: PTEN ---> AKT1	29729889	10.1016/j.biocel.2018.04.020
ProtModification: PTEN ---> AKT1	28578144	10.1016/j.etap.2017.05.013
ProtModification: PTEN ---> AKT1	32058031	10.1016/j.bbalip.2020.158658
ProtModification: PTEN ---> AKT1	30598407	10.1016/j.oooo.2018.11.011
ProtModification: PTEN ---> AKT1	25482988	10.1016/j.jbior.2014.10.004
ProtModification: PTEN ---> AKT1	19264909	10.2353/ajpath.2009.080460
ProtModification: PTEN ---> AKT1	20515660	10.1016/j.bbrc.2010.05.140
ProtModification: PTEN ---> AKT1	22074824	10.1016/j.bbrc.2011.10.120
ProtModification: PTEN ---> AKT1	22074824	10.1016/j.bbrc.2011.10.120
ProtModification: PTEN ---> AKT1	20223231	10.1016/j.cca.2010.02.074
ProtModification: PTEN ---> AKT1	16919259	10.1016/j.ydbio.2006.06.001
ProtModification: PTEN ---> AKT1	15652487	10.1016/j.cell.2004.12.033
ProtModification: PTEN ---> AKT1	22244698	10.1016/j.biomaterials.2011.12.051
ProtModification: PTEN ---> AKT1	20347952	10.1016/j.exger.2010.03.015
ProtModification: PTEN ---> AKT1	20678571	10.1016/j.freeradbiomed.2010.07.020
ProtModification: PTEN ---> AKT1	18495460	10.1016/j.jnutbio.2007.12.001
ProtModification: PTEN ---> AKT1	12946479	10.1016/S0960-7404(03)00037-9
ProtModification: PTEN ---> AKT1	15172356	10.1016/S1470-2045(04)01492-5
ProtModification: PTEN ---> AKT1		10.1016/S1535-6108(02)00024-7
ProtModification: PTEN ---> AKT1		10.1016/S1535-6108(03)00331-3
ProtModification: PTEN ---> AKT1	20801415	10.1016/j.trsl.2010.06.003
ProtModification: PTEN ---> AKT1	23403165	10.1016/j.bbcan.2013.01.004
ProtModification: PTEN ---> AKT1	23458710	10.1016/j.neuroscience.2013.02.046
ProtModification: PTEN ---> AKT1	24041630	10.1016/j.critrevonc.2013.08.003
ProtModification: PTEN ---> AKT1	24561200	10.1016/j.febslet.2014.02.005
ProtModification: PTEN ---> AKT1	24911372	10.1016/j.ajpath.2014.05.004
ProtModification: PTEN ---> AKT1	25533812	10.1016/j.phrs.2014.12.004
ProtModification: PTEN ---> AKT1	25721811	10.1016/j.semcdb.2015.02.012
ProtModification: PTEN ---> AKT1	27207584	10.1016/j.yexcr.2016.05.011
ProtModification: PTEN ---> AKT1	20630061	
ProtModification: PTEN ---> AKT1	18258752	
ProtModification: PTEN ---> AKT1	18258752	
ProtModification: PTEN ---> AKT1	17142836	
ProtModification: PTEN ---> AKT1	10400703	
ProtModification: PTEN ---> AKT1	18268330	
ProtModification: PTEN ---> AKT1	15539459	
ProtModification: PTEN ---> AKT1	15087424	
ProtModification: PTEN ---> AKT1	10988233	

ProtModification: PTEN ---> AKT1 17657248
ProtModification: PTEN ---> AKT1 10435616
ProtModification: PTEN ---> AKT1 10435616
ProtModification: PTEN ---> AKT1 11997518
ProtModification: PTEN ---> AKT1 21253589
ProtModification: PTEN ---> AKT1 21170325
ProtModification: PTEN ---> AKT1 20436671
ProtModification: PTEN ---> AKT1 26563373 10.1007/s13277-015-4392-0
ProtModification: PTEN ---> AKT1 27793908 10.21873/anticanres.11170
ProtModification: PTEN ---> AKT1 28289208 10.1073/pnas.1612284114
ProtModification: PTEN ---> AKT1 28229220 10.1007/s00432-016-2333-y
ProtModification: PTEN ---> AKT1 28384045 10.1089/gtmb.2016.0281
ProtModification: PTEN ---> AKT1 28358430 10.3892/mmr.2017.6415
ProtModification: PTEN ---> AKT1 28358430 10.3892/mmr.2017.6415
ProtModification: PTEN ---> AKT1 28498467 10.3892/ijo.2017.3982
ProtModification: PTEN ---> AKT1 28886696 10.1186/s12885-017-3639-0
ProtModification: PTEN ---> AKT1 29038421 10.1038/s41467-017-00906-9
ProtModification: PTEN ---> AKT1 29121869 10.1186/s12885-017-3714-6
ProtModification: PTEN ---> AKT1 28982275 10.1177/1933719117734700
ProtModification: PTEN ---> AKT1 29463952 10.1155/2017/5458178
ProtModification: PTEN ---> AKT1 29488607 10.3892/mmr.2018.8640
ProtModification: PTEN ---> AKT1 30134935 10.1186/s13046-018-0869-1
ProtModification: PTEN ---> AKT1 31138169 10.1186/s12885-019-5731-0
ProtModification: PTEN ---> AKT1 31006103 10.1007/s10549-019-05239-3
ProtModification: PTEN ---> AKT1 30683902 10.1038/s41374-018-0185-6
ProtModification: PTEN ---> AKT1 30318001 10.2174/1871520618666181012093056
ProtModification: PTEN ---> AKT1 31929796 10.1155/2019/2902985
ProtModification: PTEN ---> AKT1 32230887 10.3390/ijms21072322
ProtModification: PTEN ---> AKT1 32493394 10.1186/s12964-020-00586-x
ProtModification: PTEN ---> AKT1 33554779 10.1152/AJPENDO.00275.2020
ProtModification: PTEN ---> AKT1 33554779 10.1152/AJPENDO.00275.2020
ProtModification: PTEN ---> AKT1 22393246 10.1124/jpet.112.191536
ProtModification: PTEN ---> AKT1 23023710 10.1172/JCI61269
ProtModification: PTEN ---> AKT1 22465967 10.1007/s00262-012-1249-x
ProtModification: PTEN ---> AKT1 10.1038/s41374-018-0185-6
ProtModification: PTEN ---> AKT1 23239155 10.1164/rccm.201205-0851OC
ProtModification: PTEN ---> AKT1 20795550 10.1007/978-1-4419-6451-9_15
ProtModification: PTEN ---> AKT1 21953448 10.1074/jbc.M111.270884
ProtModification: PTEN ---> AKT1 22233681 10.1017/S1462399411002109
ProtModification: PTEN ---> AKT1 20630061 10.1186/1471-2407-10-368
ProtModification: PTEN ---> AKT1 22035408 10.1186/1475-2867-11-36
ProtModification: PTEN ---> AKT1 22107784 10.1186/1479-5876-9-200
ProtModification: PTEN ---> AKT1 21263312 10.1097/CAD.0b013e328341ccdd
ProtModification: PTEN ---> AKT1 21556061 10.1038/ncomms1309
ProtModification: PTEN ---> AKT1 23970032 10.3892/ijo.2013.2064
ProtModification: PTEN ---> AKT1 20436671 10.1371/journal.pone.0010350
ProtModification: PTEN ---> AKT1 21170325 10.1371/journal.pone.0015170
ProtModification: PTEN ---> AKT1 21253589 10.1371/journal.pone.0015909
ProtModification: PTEN ---> AKT1 21494560 10.1371/journal.pone.0018702
ProtModification: PTEN ---> AKT1 22194952 10.1371/journal.pone.0028930
ProtModification: PTEN ---> AKT1 24113181 10.1038/cddis.2013.375
ProtModification: PTEN ---> AKT1 24040957 10.1089/ars.2013.5619
ProtModification: PTEN ---> AKT1 24786832 10.1038/cdd.2014.53
ProtModification: PTEN ---> AKT1 25677517 10.1161/CIRCRESAHA.116.305348

ProtModification: PTEN ---> AKT1 21421815 10.1152/ajpheart.01138.2010
ProtModification: PTEN ---> AKT1 23812635 10.1152/ajplung.00432.2012
ProtModification: PTEN ---> AKT1 24651020 10.1515/revneuro-2014-0002
ProtModification: PTEN ---> AKT1 25778370 10.1186/s13058-015-0534-y
ProtModification: PTEN ---> AKT1 25982630 10.1038/aps.2015.24
ProtModification: PTEN ---> AKT1 25825815 10.1210/en.2014-1362
ProtModification: PTEN ---> AKT1 10.1074/jbc.M115.684746
ProtModification: PTEN ---> AKT1
ProtModification: PTEN ---> AKT1 26692175 10.1038/ni.3311
ProtModification: PTEN ---> AKT1 259 10.1016/j.ceca.2017.01.003
ProtModification: PTEN ---> AKT1 23953228 10.1016/j.humpath.2013.05.014
ProtModification: PTEN ---> AKT1 17478730
ProtModification: PTEN ---> AKT1 11103942
ProtModification: PTEN ---> AKT1 20596601 10.3892/ijmm-00000455
ProtModification: PTEN ---> AKT1 21267808 10.1055/s-0030-1250735
ProtModification: PTEN ---> AKT1 22364283 10.1042/BJ20112037
ProtModification: PTEN ---> AKT1 21874010 10.1038/modpathol.2011.133
ProtModification: PTEN ---> AKT1 21874010 10.1038/modpathol.2011.133
ProtModification: PTEN ---> AKT1 24736893 10.3892/mmr.2014.2155
ProtModification: PTEN ---> AKT1 25333206 10.3892/mmr.2014.2700
ProtModification: PTEN ---> AKT1 25234167 10.1038/leu.2014.278
ProtModification: PTEN ---> AKT1 27035337 10.3892/mmr.2016.5048
ProtModification: PTEN ---> AKT1 26589417 10.1007/s13277-015-4456-1
ProtModification: PTEN ---> AKT1 20515660 10.1016/j.bbrc.2010.05.140
ProtModification: PTEN ---> AKT1 27634014 10.1152/ajpgi.00292.2016
ProtModification: PTEN ---> AKT1 27829222 10.18632/oncotarget.1311310.18632/oncotarget.
ProtModification: PTEN ---> AKT1 27829222 10.18632/oncotarget.1311310.18632/oncotarget.
ProtModification: PTEN ---> AKT1 17018242 10.1016/j.neuint.2006.08.008
ProtModification: PTEN ---> AKT1 23453810 10.1016/j.molcel.2013.01.037
ProtModification: PTEN ---> AKT1 23639247 10.1016/j.toxlet.2013.04.018
ProtModification: PTEN ---> AKT1 26708722 10.1016/j.bbagrm.2015.12.006
ProtModification: PTEN ---> AKT1
ProtModification: PTEN ---> AKT1 28259931 10.3892/mmr.2017.6204
ProtModification: PTEN ---> AKT1 28358430 10.3892/mmr.2017.6415
ProtModification: PTEN ---> AKT1 28524854 10.1038/cdd.2017.73
ProtModification: PTEN ---> AKT1 28957811 10.1159/000481648
ProtModification: PTEN ---> AKT1 28982275 10.1177/1933719117734700
ProtModification: PTEN ---> AKT1 28982275 10.1177/1933719117734700
ProtModification: PTEN ---> AKT1 28982275 10.1177/1933719117734700
ProtModification: PTEN ---> AKT1 28982275 10.1177/1933719117734700
ProtModification: PTEN ---> AKT1 10.1155/2019/2902985
ProtModification: PTEN ---> AKT1 34635142 10.1186/s13046-021-02097-0
ProtModification: PTEN ---> AKT1 33895342 10.1016/j.neuroscience.2021.04.01010.1016/j.n
ProtModification: PTEN ---> AKT1 34998390 10.1186/s12964-021-00805-z
ProtModification: PTEN ---> AKT1 34248141 10.1038/s41392-021-00571-x10.1038/s41392-021-
ProtModification: PTEN ---> AKT1 36219319 10.1007/s11033-022-07903-2
Regulation: TSPAN18 ---> RAC1 35293964 10.1083/jcb.202105147
negative MolTransport: PTEN ---| 19910471 10.1074/jbc.M109.075630
negative MolTransport: PTEN ---| 26302980 10.1194/jlr.R059154
negative MolTransport: PTEN ---| 21756853 10.1016/j.jhep.2011.05.023
negative MolTransport: PTEN ---| 23981302 10.1016/j.cellsig.2013.08.005
negative MolTransport: PTEN ---| 24680857 10.1016/j.neuroscience.2014.03.039
negative MolTransport: PTEN ---| 25857437 10.1016/j.drudis.2015.03.009
negative MolTransport: PTEN ---| 19910471

negative MolTransport: PTEN ---| 12869565
negative MolTransport: PTEN ---| 18566335
negative MolTransport: PTEN ---| 21873248 10.1073/pnas.1019386108
negative MolTransport: PTEN ---| 24486596 10.1177/1073858413514634
negative MolTransport: PTEN ---| 26302980 10.1194/jlr.R059154
negative MolTransport: PTEN ---| 12869565 10.1074/jbc.M303621200
negative MolTransport: PTEN ---| AKT1 10.1194/jlr.R059154
Regulation: BST1 ---> AKT1 24753259 10.1074/jbc.M113.535070
Regulation: BST1 ---> AKT1 29936181 10.1016/j.imlet.2018.06.007
Regulation: BST1 ---> AKT1 10.1016/j.cyto.2009.07.552
Regulation: BST1 ---> AKT1 21478153 10.1074/jbc.M111.227876
DirectRegulation: UCA1 ---> TP53 26551349 10.1016/j.trsl.2015.10.002
DirectRegulation: UCA1 ---> TP53 32112905 10.1016/j.canlet.2020.02.003
DirectRegulation: UCA1 ---> TP53 28738810 10.1186/s12943-017-0699-3
DirectRegulation: UCA1 ---> TP53 33421618 10.1016/j.semcancer.2020.12.019
Expression: BECN1 ---> PTGS2 29307663 10.1016/j.biocel.2018.01.002
Expression: BECN1 ---> PTGS2 24905997 10.4161/auto.28771
Expression: BECN1 ---> PTGS2 34212828 10.2174/0929867328666210629160647
Expression: NGF ---> MCL1 10915813 10.1016/S0006-8993(00)02512-9
Expression: NGF ---> MCL1 27551499 10.1038/cddiscovery.2016.410.1038/cddiscovery
Expression: SPP1 ---> BECN1 28139205 10.1016/j.atherosclerosis.2017.01.017
Expression: SPP1 ---> BECN1 10.1016/j.toxlet.2010.03.308
Expression: SPP1 ---> BECN1 36505285
positive Regulation: BST1 --+> M/21478153 10.1074/jbc.M111.227876
PromoterBinding: PAX8 ---> AKT1 18202153
PromoterBinding: PAX8 ---> AKT1 21974806 10.1016/j.canlet.2011.09.010
negative Expression: AKT1 ---| P/26872612 10.1016/j.mce.2016.02.006
negative Expression: AKT1 ---| P/26872612 10.1016/j.mce.2016.02.006
Regulation: CD274 ---> PTGS2 28231855 10.1186/s12967-017-1150-7
Regulation: CD274 ---> PTGS2 10.1530/ERC-17-0376
Regulation: CD274 ---> PTGS2 30897754 10.3390/ijms20061405
Regulation: CD274 ---> PTGS2 28231855 10.1186/s12967-017-1150-710.1186/s12967-017-1
positive PromoterBinding: TP53 --19173293 10.1002/ijc.24221
positive PromoterBinding: TP53 --16778187 10.1158/0008-5472.CAN-06-0772
positive PromoterBinding: TP53 --16061640 10.1158/0008-5472.CAN-04-4184
positive PromoterBinding: TP53 --12917634 10.1038/sj.onc.1206783
positive PromoterBinding: TP53 --10865036 10.1007/s002680010164
positive PromoterBinding: TP53 --15843377 10.1074/jbc.M503026200
positive PromoterBinding: TP53 --19079342 10.1038/onc.2008.451
positive PromoterBinding: TP53 --15016317 10.1111/j.1349-7006.2004.tb02203.x
positive PromoterBinding: TP53 --20554748 10.1093/carcin/bgq123
positive PromoterBinding: TP53 --11729185 10.1074/jbc.M108302200
positive PromoterBinding: TP53 --+> PTEN 10.1016/j.semcancer.2019.02.001
positive PromoterBinding: TP53 --+> PTEN 10.1016/j.bbrc.2012.04.015
positive PromoterBinding: TP53 --+> PTEN 10.1016/S0006-291X(02)02983-2
positive PromoterBinding: TP53 --22290228 10.1016/j.bbrc.2012.01.065
positive PromoterBinding: TP53 --17258789 10.1016/j.humpath.2006.10.007
positive PromoterBinding: TP53 --16545436 10.1016/j.ygyno.2005.12.033
positive PromoterBinding: TP53 --21664656 10.1016/j.ygyno.2011.05.017
positive PromoterBinding: TP53 --16051030 10.1016/j.canlet.2004.12.006
positive PromoterBinding: TP53 --16996682 10.1016/j.canlet.2006.08.007
positive PromoterBinding: TP53 --12217521 10.1016/S0968-0004(02)02166-7
positive PromoterBinding: TP53 --12217521 10.1016/S0968-0004(02)02166-7
positive PromoterBinding: TP53 --11545734 10.1016/S1097-2765(01)00323-9

positive PromoterBinding: TP53 --11545734 10.1016/S1097-2765(01)00323-9
positive PromoterBinding: TP53 --11545734 10.1016/S1097-2765(01)00323-9
positive PromoterBinding: TP53 --11545734 10.1016/S1097-2765(01)00323-9
positive PromoterBinding: TP53 --11545734 10.1016/S1097-2765(01)00323-9
positive PromoterBinding: TP53 --18950730 10.1016/j.biocel.2008.09.022
positive PromoterBinding: TP53 --18718806 10.1016/j.drup.2008.07.001
positive PromoterBinding: TP53 --12620407 10.1016/S1535-6108(03)00021-7
positive PromoterBinding: TP53 --21146147 10.1016/j.trsl.2010.09.004
positive PromoterBinding: TP53 --22510407 10.1016/j.bbrc.2012.04.015
positive PromoterBinding: TP53 --23122111 10.1016/j.foodchem.2012.07.112
positive PromoterBinding: TP53 --25744176 10.1016/j.drudis.2015.02.011
positive PromoterBinding: TP53 --17998413
positive PromoterBinding: TP53 --18032782
positive PromoterBinding: TP53 --15193142
positive PromoterBinding: TP53 --16495927
positive PromoterBinding: TP53 --18172008
positive PromoterBinding: TP53 --18172008
positive PromoterBinding: TP53 --28301379 10.1097/CAD.0000000000000476
positive PromoterBinding: TP53 --28301379 10.1097/CAD.0000000000000476
positive PromoterBinding: TP53 --28301379 10.1097/CAD.0000000000000476
positive PromoterBinding: TP53 --30217221 10.1186/s13046-018-0898-9
positive PromoterBinding: TP53 --30486451 10.3390/ijms19123773
positive PromoterBinding: TP53 --+> PTEN 10.1007/978-981-15-0602-4_6
positive PromoterBinding: TP53 --22489661 10.1089/cbr.2011.1154
positive PromoterBinding: TP53 --20862607 10.1007/s10528-010-9379-3
positive PromoterBinding: TP53 --21990315 10.1210/en.2011-1207
positive PromoterBinding: TP53 --22218495 10.3892/ijmm.2012.879
positive PromoterBinding: TP53 --20736773 10.1097/PGP.0b013e3181de3068
positive PromoterBinding: TP53 --21297666 10.1038/onc.2011.6
positive PromoterBinding: TP53 --24083690 10.7314/APJCP.2013.14.8.4485
positive PromoterBinding: TP53 --24866016 10.1371/journal.pone.0098113
positive PromoterBinding: TP53 --24905788 10.1146/annurev-biochem-082411-113907
positive PromoterBinding: TP53 --19079342 10.1038/onc.2008.45110.1038/onc.2008.451
positive PromoterBinding: TP53 --20554748 10.1093/carcin/bgq12310.1093/carcin/bgq123
positive PromoterBinding: TP53 --+> PTEN
positive PromoterBinding: TP53 --19234438
positive PromoterBinding: TP53 --19173293 10.1002/ijc.24221
positive PromoterBinding: TP53 --16778187 10.1158/0008-5472.CAN-06-0772
positive PromoterBinding: TP53 --16061640 10.1158/0008-5472.CAN-04-4184
positive PromoterBinding: TP53 --12917634 10.1038/sj.onc.1206783
positive PromoterBinding: TP53 --10865036
positive PromoterBinding: TP53 --15843377 10.1074/jbc.M503026200
positive PromoterBinding: TP53 --15016317
positive PromoterBinding: TP53 --11729185 10.1074/jbc.M108302200
positive PromoterBinding: TP53 --28602818 10.1016/j.jmb.2017.06.004
positive PromoterBinding: TP53 --33194023
positive PromoterBinding: TP53 --32974790 10.1007/s10549-020-05937-3
positive QuantitativeChange: hypo33964809 10.1016/j.intimp.2021.107675
positive QuantitativeChange: hypo33964809 10.1016/j.intimp.2021.107675
positive QuantitativeChange: hypo33964809 10.1016/j.intimp.2021.10767510.1016/j.intimp.
Biomarker: hypopharyngeal squamou32765090 10.2147/CMAR.S25729910.2147/CMAR.S257299
Biomarker: hypopharyngeal squamou33964809 10.1016/j.intimp.2021.10767510.1016/j.intimp.
positive QuantitativeChange: hypo27175933 10.3892/mmr.2016.5242
negative Expression: ING4 ---| PT18789575 10.1016/j.canlet.2008.05.050

negative Expression: ING4 ---| PT19903538 10.1016/j.arr.2009.11.001
negative Expression: ING4 ---| PT22863759 10.1038/cgt.2012.50
negative Expression: ING4 ---| PT24581166 10.3779/j.issn.1009-3419.2014.02.13
negative Expression: ING4 ---| PT24581166 10.3779/j.issn.1009-3419.2014.02.1310.3779/j.
negative Expression: ING4 ---| PT27806345 10.18632/oncotarget.1298410.18632/oncotarget.
negative Expression: ING4 ---| PT22863759 10.1038/cgt.2012.50
negative Expression: ING4 ---| PT22863759 10.1038/cgt.2012.50
negative Expression: ING4 ---| PT30643005 10.1042/BSR20180773
negative Expression: ING4 ---| PTGS2 10.1042/BSR20180773
positive Expression: NGF --+> RA(11278419 10.1074/jbc.M008546200
positive Expression: NGF --+> RA(11279039 10.1074/jbc.M100254200
positive Expression: NGF --+> RA(32533921 10.1016/j.devcel.2020.05.020
positive Expression: NGF --+> RA(21816277 10.1016/j.cell.2011.07.008
positive Expression: NGF --+> RA(11279039
positive Expression: NGF --+> RA(11278419
positive Expression: NGF --+> RA(30522553 10.1691/ph.2018.8726
positive Expression: NGF --+> RA(30522553 10.1691/ph.2018.8726
positive Expression: NGF --+> RA(30522553 10.1691/ph.2018.8726
positive Expression: NGF --+> RA(30522553 10.1691/ph.2018.8726
positive Expression: NGF --+> RA(30522553 10.1691/ph.2018.8726
positive Expression: NGF --+> RA(30522553 10.1691/ph.2018.872610.1691/ph.2018.8726
Regulation: RAI2 ---> chemosensit35299743 10.3389/fonc.2022.80529010.3389/fonc.2022.805
Regulation: RAI2 ---> chemosensit35299743 10.3389/fonc.2022.80529010.3389/fonc.2022.805
positive Expression: NGF --+> PT(25231981 10.1074/jbc.M114.587188
positive Expression: NGF --+> PT(28844566 10.1016/j.joca.2017.08.006
positive Expression: NGF --+> PT(10841984 10.1016/S0304-3940(00)01158-7
positive Expression: NGF --+> PT(11108289
positive Expression: NGF --+> PT(11108289
positive Expression: NGF --+> PT(26370988 10.3390/ijms160921759
positive Expression: NGF --+> PT(28673205 10.1177/1933719117716778
positive Expression: NGF --+> PT(28673205 10.1177/1933719117716778
positive Expression: NGF --+> PT(28673205 10.1177/1933719117716778
positive Expression: NGF --+> PT(28673205 10.1177/1933719117716778
positive Expression: NGF --+> PT(28673205 10.1177/1933719117716778
positive Expression: NGF --+> PT(28673205 10.1177/1933719117716778
positive Expression: NGF --+> PT(28673205 10.1177/1933719117716778
positive Expression: NGF --+> PT(28673205 10.1177/1933719117716778
positive Expression: NGF --+> PTGS2
positive Expression: NGF --+> PT(23825664 10.1371/journal.pone.0067470
positive Expression: NGF --+> PT(25231981 10.1074/jbc.M114.587188
positive Expression: NGF --+> PTGS2 10.1074/jbc.M114.587188
positive Expression: NGF --+> PT(15145552 10.1016/j.neuint.2003.06.001
positive Expression: NGF --+> PT(28245631 10.3390/ijms18030507
positive Expression: NGF --+> PT(9794438
positive Expression: NGF --+> PT(9794438
positive Expression: NGF --+> PT(9794438
positive Expression: NGF --+> PT(12045671
positive Expression: NGF --+> PTGS2
negative Expression: PTGS2 ---| N33857603 10.1016/j.prostaglandins.2021.106550
negative Expression: PTGS2 ---| N33857603 10.1016/j.prostaglandins.2021.106550
negative Expression: PTGS2 ---| N24993611 10.1016/j.addr.2014.06.009
negative Expression: PTGS2 ---| N28668088 10.1186/s12891-017-1648-4
negative Expression: PTGS2 ---| N34074169 10.1177/17448069211021252
negative Expression: PTGS2 ---| N23574818 10.1097/BRS.0b013e318294edb1

negative Expression: PTGS2 ---| N	26656048	10.1097/BRS.0000000000001379
negative Expression: PTGS2 ---| N	16879691	10.1111/j.1464-410X.2006.06207.x
negative Expression: PTGS2 ---| N	28759126	10.1111/sms.1295410.1111/sms.12954
negative Expression: PTGS2 ---| N	26876621	10.1016/j.jos.2016.01.00410.1016/j.jos.2016.0
negative Expression: PTGS2 ---| NGF		10.1097/BRS.0000000000001379
negative Expression: PTGS2 ---| N	23799042	10.1371/journal.pone.006575110.1371/journal.p
negative Expression: PTGS2 ---| NGF		10.1097/BRS.0000000000001379
negative Expression: PTGS2 ---| N	19129380	
negative Expression: PTGS2 ---| N	10766843	
negative Expression: PTGS2 ---| NGF		
negative Regulation: CD274 ---| c	26806807	10.1016/j.canlet.2015.12.006
negative Regulation: CD274 ---| c	30172884	10.1016/j.tranon.2018.08.005
negative Regulation: CD274 ---| c	33073683	10.1177/172460082092617210.1177/1724600820926
negative Regulation: CD274 ---| c	33030957	10.4149/neo_2020_200531N589
positive Expression: BIRC7 --+> (	25203888	10.1097/SLA.0000000000000916
PromoterBinding: PPARG ---> MCL1	607	10.1016/j.tim.2018.08.001
PromoterBinding: PPARG ---> MCL1	29928066	10.1371/journal.ppat.1007100
PromoterBinding: PPARG ---> MCL1	29928066	10.1371/journal.ppat.1007100
PromoterBinding: PPARG ---> MCL1	29928066	10.1371/journal.ppat.1007100
PromoterBinding: PPARG ---> MCL1	29928066	10.1371/journal.ppat.1007100
PromoterBinding: PPARG ---> MCL1	29928066	10.1371/journal.ppat.1007100
PromoterBinding: PPARG ---> MCL1	607	10.1016/j.tim.2018.08.001
PromoterBinding: PPARG ---> MCL1	29928066	10.1371/journal.ppat.100710010.1371/journal.p
PromoterBinding: PPARG ---> MCL1	25944662	10.1016/j.bbrc.2015.04.023
PromoterBinding: PPARG ---> MCL1		10.1016/S1074-7613(04)00030-5
negative QuantitativeChange: hypc	23935917	10.1371/journal.pone.0069038
negative QuantitativeChange: hypc	25923669	10.1371/journal.pone.0126147
negative Regulation: BIRC7 ---| c	20171199	10.1016/j.clinbiochem.2010.02.004
negative Regulation: BIRC7 ---| c	27448305	10.1007/s13277-016-5157-0
negative Regulation: BIRC7 ---| c	27448305	10.1007/s13277-016-5157-0
negative Regulation: BIRC7 ---| c	27448305	10.1007/s13277-016-5157-0
negative Regulation: BIRC7 ---| c	27448305	10.1007/s13277-016-5157-0
negative Regulation: BIRC7 ---| c	27448305	10.1007/s13277-016-5157-0
negative Regulation: BIRC7 ---| c	27448305	10.1007/s13277-016-5157-0
negative Regulation: BIRC7 ---| c	27448305	10.1007/s13277-016-5157-0
negative Regulation: BIRC7 ---| c	27448305	10.1007/s13277-016-5157-0
negative Regulation: BIRC7 ---| c	27448305	10.1007/s13277-016-5157-0
negative Regulation: BIRC7 ---| c	28440463	10.3892/or.2017.5584
negative Regulation: BIRC7 ---| c	28440463	10.3892/or.2017.5584
negative Regulation: BIRC7 ---| c	28440463	10.3892/or.2017.5584
negative Regulation: BIRC7 ---| c	28440463	10.3892/or.2017.5584
negative Regulation: BIRC7 ---| c	28765921	10.3892/or.2017.5866
negative Regulation: BIRC7 ---| c	28765921	10.3892/or.2017.5866
negative Regulation: BIRC7 ---| c	28765921	10.3892/or.2017.5866
negative Regulation: BIRC7 ---| c	31777256	10.4149/neo_2019_190106N18
negative Regulation: BIRC7 ---| c	33649820	10.3892/or.2021.7969
negative Regulation: BIRC7 ---| c	33649820	10.3892/or.2021.7969
negative Regulation: BIRC7 ---| c	33649820	10.3892/or.2021.7969
negative Regulation: BIRC7 ---| c	21883015	10.1089/cbr.2011.0962
negative Regulation: BIRC7 ---| c	23632777	10.3892/ijo.2013.1925
negative Regulation: BIRC7 ---| c	23632777	10.3892/ijo.2013.1925
negative Regulation: BIRC7 ---| c	23632777	10.3892/ijo.2013.1925
negative Regulation: BIRC7 ---| c	18340356	10.1038/cgt.2008.1610.1038/cgt.2008.16
negative Regulation: BIRC7 ---| c	23632777	10.3892/ijo.2013.192510.3892/ijo.2013.1925

negative Regulation: BIRC7 ---| c 23632777 10.3892/ijo.2013.192510.3892/ijo.2013.1925
negative Regulation: BIRC7 ---| c 25695324 10.3892/mmr.2015.337210.3892/mmr.2015.3372
negative Regulation: BIRC7 ---| c 27448305 10.1007/s13277-016-5157-0
negative Regulation: BIRC7 ---| c 28440463 10.3892/or.2017.558410.3892/or.2017.5584
negative Regulation: BIRC7 ---| c 28765921 10.3892/or.2017.586610.3892/or.2017.5866
negative Regulation: BIRC7 ---| c 31777256 10.4149/neo_2019_190106N1810.4149/neo_2019_19
Regulation: TP53 ---> chemosensit 34015314 10.1016/j.yexcr.2021.112649
Regulation: TP53 ---> chemosensit 16159878 10.1074/jbc.M507192200
Regulation: TP53 ---> chemosensit 20075077 10.1074/jbc.M109.047985
Regulation: TP53 ---> chemosensit 25112877 10.1074/jbc.M114.594812
Regulation: TP53 ---> chemosensit 15262986 10.1074/jbc.M401530200
Regulation: TP53 ---> chemosensit 12890671 10.1074/jbc.M307149200
Regulation: TP53 ---> chemosensit 12890671 10.1074/jbc.M307149200
Regulation: TP53 ---> chemosensit 31614146 10.1016/j.lfs.2019.116933
Regulation: TP53 ---> chemosensit 27681874 10.1016/j.bbcan.2016.09.006
Regulation: TP53 ---> chemosensit 28755993 10.1016/j.ijpharm.2017.07.056
Regulation: TP53 ---> chemosensit 29494959 10.1016/j.biopha.2018.02.066
Regulation: TP53 ---> chemosensit 33785447 10.1016/j.semcancer.2021.03.010
Regulation: TP53 ---> chemosensit 29615301 10.1016/j.dld.2018.02.009
Regulation: TP53 ---> chemosensit 30898612 10.1016/j.jbior.2019.03.002
Regulation: TP53 ---> chemosensit 33451973 10.1016/j.jbior.2020.100780
Regulation: TP53 ---> chemosensit 21211512 10.1016/j.abb.2010.12.034
Regulation: TP53 ---> chemosensit 15865929 10.1016/j.bbrc.2005.03.153
Regulation: TP53 ---> chemosensit 20233581 10.1016/j.bbrc.2010.03.082
Regulation: TP53 ---> chemosensitivity 10.1016/S0006-2952(01)00733-X
Regulation: TP53 ---> chemosensit 12694871 10.1016/S0006-2952(03)00078-9
Regulation: TP53 ---> chemosensit 12948852 10.1016/S0006-2952(03)00397-6
Regulation: TP53 ---> chemosensit 17445779 10.1016/j.bcp.2007.03.009
Regulation: TP53 ---> chemosensit 19028473 10.1016/j.cbi.2008.10.048
Regulation: TP53 ---> chemosensit 11435891 10.1016/S0022-5347(05)66150-2
Regulation: TP53 ---> chemosensitivity 10.1016/S0022-5347(05)67323-5
Regulation: TP53 ---> chemosensit 10379742 10.1097/00005392-199907000-00017
Regulation: TP53 ---> chemosensit 12175703 10.1016/S0024-3205(02)01900-8
Regulation: TP53 ---> chemosensit 12409142 10.1016/S0024-3205(02)02177-X
Regulation: TP53 ---> chemosensit 14761678 10.1016/j.taap.2003.09.011
Regulation: TP53 ---> chemosensit 15891997 10.1016/j.humpath.2005.01.018
Regulation: TP53 ---> chemosensit 12893182 10.1016/S0090-8258(03)00282-8
Regulation: TP53 ---> chemosensit 12893182 10.1016/S0090-8258(03)00282-8
Regulation: TP53 ---> chemosensit 15790436 10.1016/j.ygyno.2004.12.053
Regulation: TP53 ---> chemosensit 15790436 10.1016/j.ygyno.2004.12.053
Regulation: TP53 ---> chemosensit 16380178 10.1016/j.tibtech.2005.12.002
Regulation: TP53 ---> chemosensit 22446689 10.1016/j.jhep.2011.11.029
Regulation: TP53 ---> chemosensit 11557115 10.1016/S0169-5002(01)00230-6
Regulation: TP53 ---> chemosensit 16307839 10.1016/j.fct.2005.10.003
Regulation: TP53 ---> chemosensit 10737712 10.1016/S0304-3835(99)00345-6
Regulation: TP53 ---> chemosensit 10893449 10.1016/S0304-3835(00)00480-8
Regulation: TP53 ---> chemosensit 12880966 10.1016/S0304-3835(03)00088-0
Regulation: TP53 ---> chemosensit 15327837 10.1016/j.canlet.2004.03.044
Regulation: TP53 ---> chemosensit 16023288 10.1016/j.canlet.2005.06.002
Regulation: TP53 ---> chemosensit 16919866 10.1016/j.canlet.2006.06.013
Regulation: TP53 ---> chemosensit 18657356 10.1016/j.canlet.2008.06.006
Regulation: TP53 ---> chemosensit 19467788 10.1016/j.ctrv.2009.04.008
Regulation: TP53 ---> chemosensit 21856084 10.1016/j.mehy.2011.07.052
Regulation: TP53 ---> chemosensit 22353361 10.1016/j.gene.2012.01.099

Regulation: TP53 ---> chemosensit22445862 10.1016/j.tiv.2012.03.004
Regulation: TP53 ---> chemosensit17875383 10.1016/j.ijom.2007.06.006
Regulation: TP53 ---> chemosensit17875383 10.1016/j.ijom.2007.06.006
Regulation: TP53 ---> chemosensit16337111 10.1016/j.colsurfb.2005.10.006
Regulation: TP53 ---> chemosensit16713222 10.1016/j.phymed.2006.03.009
Regulation: TP53 ---> chemosensit15691646 10.1016/j.ejca.2004.10.014
Regulation: TP53 ---> chemosensit16146690 10.1016/j.ejca.2005.04.044
Regulation: TP53 ---> chemosensit12850529 10.1016/S1040-8428(03)00066-0
Regulation: TP53 ---> chemosensit16253824 10.1053/j.semtcvs.2005.08.002
Regulation: TP53 ---> chemosensit19508919 10.1016/j.drudis.2009.03.008
Regulation: TP53 ---> chemosensit21195657 10.1016/j.drup.2010.12.001
Regulation: TP53 ---> chemosensit12829013 10.1016/S1471-4914(03)00084-4
Regulation: TP53 ---> chemosensit12726865 10.1016/S1535-6108(03)00078-3
Regulation: TP53 ---> chemosensit20832747 10.1016/j.ccr.2010.08.016
Regulation: TP53 ---> chemosensit17240826 10.1016/j.thorsurg.2006.08.001
Regulation: TP53 ---> chemosensit23040255 10.1053/j.seminoncol.2012.08.003
Regulation: TP53 ---> chemosensit23313858 10.1016/j.yexcr.2012.12.025
Regulation: TP53 ---> chemosensit23973262 10.1016/j.canlet.2013.08.028
Regulation: TP53 ---> chemosensit24462821 10.1016/j.canlet.2014.01.011
Regulation: TP53 ---> chemosensit25117446 10.1016/j.bbrc.2014.08.014
Regulation: TP53 ---> chemosensit25220870 10.1016/j.biochi.2014.09.001
Regulation: TP53 ---> chemosensit25448278 10.1016/j.toxlet.2014.11.016
Regulation: TP53 ---> chemosensit24681512 10.1016/j.phrs.2014.03.007
Regulation: TP53 ---> chemosensit24681512 10.1016/j.phrs.2014.03.007
Regulation: TP53 ---> chemosensit21293058
Regulation: TP53 ---> chemosensit21293058
Regulation: TP53 ---> chemosensit11095436
Regulation: TP53 ---> chemosensit11095436
Regulation: TP53 ---> chemosensit9768682
Regulation: TP53 ---> chemosensit9661637
Regulation: TP53 ---> chemosensit20075077
Regulation: TP53 ---> chemosensit19055826
Regulation: TP53 ---> chemosensit17150101
Regulation: TP53 ---> chemosensit17121812
Regulation: TP53 ---> chemosensit15262986
Regulation: TP53 ---> chemosensit12890671
Regulation: TP53 ---> chemosensit12690107
Regulation: TP53 ---> chemosensit12615954
Regulation: TP53 ---> chemosensit12615954
Regulation: TP53 ---> chemosensit12615954
Regulation: TP53 ---> chemosensit12615954
Regulation: TP53 ---> chemosensit9694807
Regulation: TP53 ---> chemosensit14769944
Regulation: TP53 ---> chemosensit11583579
Regulation: TP53 ---> chemosensit11248087
Regulation: TP53 ---> chemosensit9122197
Regulation: TP53 ---> chemosensit16755297
Regulation: TP53 ---> chemosensit15153941
Regulation: TP53 ---> chemosensit14739942
Regulation: TP53 ---> chemosensit11791171
Regulation: TP53 ---> chemosensit11791171
Regulation: TP53 ---> chemosensit11791171
Regulation: TP53 ---> chemosensit11791171
Regulation: TP53 ---> chemosensit11791171

Regulation: TP53 ---> chemosensit	10698490	
Regulation: TP53 ---> chemosensit	10471039	
Regulation: TP53 ---> chemosensit	16260623	
Regulation: TP53 ---> chemosensit	20706634	
Regulation: TP53 ---> chemosensit	27329169	10.1007/s10549-016-3869-x
Regulation: TP53 ---> chemosensitivity		10.1016/j.omtn.2021.07.022
Regulation: TP53 ---> chemosensit	21293058	10.1172/JCI4389710.1172/JCI43897
Regulation: TP53 ---> chemosensit	21364617	10.1038/cddis.2009.1510.1038/cddis.2009.15
Regulation: TP53 ---> chemosensit	23224642	10.1007/s10072-012-1257-910.1007/s10072-012-1
Regulation: TP53 ---> chemosensit	25158956	10.1158/1541-7786.MCR-14-017710.1158/1541-778
Regulation: TP53 ---> chemosensit	21248371	10.18632/aging.100254
Regulation: TP53 ---> chemosensit	25296977	10.18632/oncotarget.2528
Regulation: TP53 ---> chemosensitivity		10.1016/j.jtcms.2022.01.001
Regulation: TP53 ---> chemosensit	35437704	10.1007/s13577-022-00700-w
Regulation: TP53 ---> chemosensit	36361682	10.3390/ijms232112893
Expression: NOTCH1 ---> TP53	16449526	10.1182/blood-2005-09-3551
Expression: NOTCH1 ---> TP53	19376776	10.1074/jbc.M109.002105
Expression: NOTCH1 ---> TP53	19376776	10.1074/jbc.M109.002105
Expression: NOTCH1 ---> TP53	19376776	10.1074/jbc.M109.002105
Expression: NOTCH1 ---> TP53	19376776	10.1074/jbc.M109.002105
Expression: NOTCH1 ---> TP53	30336197	10.1016/j.canlet.2018.09.031
Expression: NOTCH1 ---> TP53	27888063	10.1016/j.biocel.2016.11.013
Expression: NOTCH1 ---> TP53	27888063	10.1016/j.biocel.2016.11.013
Expression: NOTCH1 ---> TP53	27888063	10.1016/j.biocel.2016.11.013
Expression: NOTCH1 ---> TP53	22673505	10.1016/j.febslet.2012.03.017
Expression: NOTCH1 ---> TP53	21723221	10.1053/j.gastro.2011.06.056
Expression: NOTCH1 ---> TP53	21723221	10.1053/j.gastro.2011.06.056
Expression: NOTCH1 ---> TP53	24140475	10.1016/j.cellsig.2013.09.021
Expression: NOTCH1 ---> TP53	24140475	10.1016/j.cellsig.2013.09.021
Expression: NOTCH1 ---> TP53	25704190	10.1016/j.clineuro.2015.01.018
Expression: NOTCH1 ---> TP53	19376776	
Expression: NOTCH1 ---> TP53	19376776	
Expression: NOTCH1 ---> TP53	19376776	
Expression: NOTCH1 ---> TP53	19376776	
Expression: NOTCH1 ---> TP53	19376776	
Expression: NOTCH1 ---> TP53	18049480	
Expression: NOTCH1 ---> TP53	17186020	
Expression: NOTCH1 ---> TP53	17186020	
Expression: NOTCH1 ---> TP53	17186020	
Expression: NOTCH1 ---> TP53	17186020	
Expression: NOTCH1 ---> TP53	17186020	
Expression: NOTCH1 ---> TP53	16341148	
Expression: NOTCH1 ---> TP53	29857516	10.3390/ijms19061631
Expression: NOTCH1 ---> TP53	29717634	10.1089/scd.2017.0260
Expression: NOTCH1 ---> TP53	20491628	10.2174/138920310791824039
Expression: NOTCH1 ---> TP53	21743969	10.3892/or.2011.1380
Expression: NOTCH1 ---> TP53	22065781	10.1073/pnas.1104943108
Expression: NOTCH1 ---> TP53	24504124	10.3892/ijo.2014.2286
Expression: NOTCH1 ---> TP53	24950189	10.1371/journal.pone.0100359
Expression: NOTCH1 ---> TP53		
Expression: NOTCH1 ---> TP53	25380967	10.1186/1476-4598-13-247
Expression: NOTCH1 ---> TP53	15817159	10.1016/j.yexcr.2005.01.015
Expression: NOTCH1 ---> TP53	17186020	10.1038/sj.cdd.4402083
Expression: NOTCH1 ---> TP53	18359760	10.1093/carcin/bgn07910.1093/carcin/bgn079

Expression: NOTCH1 ---> TP53 19122673 10.1038/aps.2008.1610.1038/aps.2008.16
Expression: NOTCH1 ---> TP53 19376776 10.1074/jbc.M109.00210510.1074/jbc.M109.00210
Expression: NOTCH1 ---> TP53 21743969 10.3892/or.2011.138010.3892/or.2011.1380
Expression: NOTCH1 ---> TP53 31410150 10.3892/etm.2019.776510.3892/etm.2019.7765
Expression: NOTCH1 ---> TP53 31497354
Expression: NOTCH1 ---> TP53 10.1016/j.febslet.2012.03.017
Expression: NOTCH1 ---> TP53 19304334 10.1016/j.jhep.2008.12.032
Expression: NOTCH1 ---> TP53 27600900 10.1016/j.coviro.2016.08.011
Expression: NOTCH1 ---> TP53 23219912 10.1016/j.cellsig.2012.11.025
Expression: NOTCH1 ---> TP53 10.1016/j.bbrc.2014.10.023
Expression: NOTCH1 ---> TP53 19376776
Expression: NOTCH1 ---> TP53 18049480
Expression: NOTCH1 ---> TP53 18049480
Expression: NOTCH1 ---> TP53 17186020
Expression: NOTCH1 ---> TP53 17186020
Expression: NOTCH1 ---> TP53 17186020
Expression: NOTCH1 ---> TP53 17186020
Expression: NOTCH1 ---> TP53 21084867 10.4161/cbt.10.10.13923
Expression: NOTCH1 ---> TP53 22065781 10.1073/pnas.1104943108
Expression: NOTCH1 ---> TP53 22065781 10.1073/pnas.1104943108
Expression: NOTCH1 ---> TP53 23359069 10.1084/jem.20121527
Expression: NOTCH1 ---> TP53 23359069 10.1084/jem.20121527
Expression: NOTCH1 ---> TP53 26496776 10.1007/s10495-015-1188-z
Expression: NOTCH1 ---> TP53 26985633 10.1080/15384101.2016.1160975
Expression: NOTCH1 ---> TP53 21084383 10.1210/me.2010-0248
Expression: NOTCH1 ---> TP53 19376776
Expression: NOTCH1 ---> TP53 19376776
Expression: NOTCH1 ---> TP53 19376776
Expression: NOTCH1 ---> TP53 18367647
Expression: NOTCH1 ---> TP53 12208848
Expression: NOTCH1 ---> TP53 18192969
Expression: NOTCH1 ---> TP53 23737838 10.1155/2013/590393
Expression: NOTCH1 ---> TP53 22249263 10.1038/onc.2011.589
Expression: NOTCH1 ---> TP53 23936265 10.1371/journal.pone.0071213
Expression: NOTCH1 ---> TP53 21480340 10.1002/hep.24208
Expression: NOTCH1 ---> TP53
Expression: NOTCH1 ---> TP53 33874861 10.2174/0929867328666210419123200
PromoterBinding: TP53 ---> NOTCH128757263 10.1053/j.gastro.2017.06.066
PromoterBinding: TP53 ---> NOTCH129358327 10.1074/jbc.M117.791145
PromoterBinding: TP53 ---> NOTCH131102753 10.1016/j.brainresbull.2019.05.009
PromoterBinding: TP53 ---> NOTCH119103190 10.1016/j.ydbio.2008.11.021
PromoterBinding: TP53 ---> NOTCH123810012 10.1016/B978-0-12-416673-8.00013-7
PromoterBinding: TP53 ---> NOTCH117344417
PromoterBinding: TP53 ---> NOTCH117344417
PromoterBinding: TP53 ---> NOTCH117344417
PromoterBinding: TP53 ---> NOTCH117353266
PromoterBinding: TP53 ---> NOTCH117353266
PromoterBinding: TP53 ---> NOTCH120442780
PromoterBinding: TP53 ---> NOTCH120442780
PromoterBinding: TP53 ---> NOTCH120442780
PromoterBinding: TP53 ---> NOTCH120442780
PromoterBinding: TP53 ---> NOTCH129358327 10.1074/jbc.M117.791145
PromoterBinding: TP53 ---> NOTCH128847161 10.1089/ars.2017.7262
PromoterBinding: TP53 ---> NOTCH130673937 10.1007/s12272-019-01118-z

PromoterBinding: TP53 ---> NOTCH131689961 10.3390/ijms20215482
PromoterBinding: TP53 ---> NOTCH122833673 10.1074/jbc.M112.354522
PromoterBinding: TP53 ---> NOTCH122833673 10.1074/jbc.M112.354522
PromoterBinding: TP53 ---> NOTCH122833673 10.1074/jbc.M112.354522
PromoterBinding: TP53 ---> NOTCH122990386 10.4161/cc.22265
PromoterBinding: TP53 ---> NOTCH121042738 10.3892/or-00001004
PromoterBinding: TP53 ---> NOTCH121679465 10.1186/bcr2900
PromoterBinding: TP53 ---> NOTCH122583596 10.1186/1471-2121-13-12
PromoterBinding: TP53 ---> NOTCH123860128 10.1038/emboj.2013.156
PromoterBinding: TP53 ---> NOTCH120442780 10.1371/journal.pone.0010369
PromoterBinding: TP53 ---> NOTCH120442780 10.1371/journal.pone.0010369
PromoterBinding: TP53 ---> NOTCH120442780 10.1371/journal.pone.0010369
PromoterBinding: TP53 ---> NOTCH120442780 10.1371/journal.pone.0010369
PromoterBinding: TP53 ---> NOTCH122833673 10.1074/jbc.M112.35452210.1074/jbc.M112.35452
PromoterBinding: TP53 ---> NOTCH1 10.1074/jbc.M117.791145
PromoterBinding: TP53 ---> NOTCH1 10.3390/ijms20215482
PromoterBinding: TP53 ---> NOTCH123946688 10.4196/kjpp.2013.17.4.291
PromoterBinding: TP53 ---> NOTCH1
PromoterBinding: TP53 ---> NOTCH1 10.1016/j.gendis.2022.04.013
PromoterBinding: TP53 ---> NOTCH1 10.1016/j.gendis.2022.04.013
PromoterBinding: TP53 ---> NOTCH135617425 10.1073/pnas.2123202119
positive Expression: MAPK1 --+> S18390540 10.1074/jbc.M800668200
positive Expression: MAPK1 --+> S20427276 10.1074/jbc.M110.110312
positive Expression: MAPK1 --+> S20427276 10.1074/jbc.M110.110312
positive Expression: MAPK1 --+> S20427276 10.1074/jbc.M110.110312
positive Expression: MAPK1 --+> S20427276 10.1074/jbc.M110.110312
positive Expression: MAPK1 --+> S20007708 10.1074/jbc.M109.033951
positive Expression: MAPK1 --+> S27794492 10.1016/j.jconrel.2016.10.024
positive Expression: MAPK1 --+> S32707422 10.1016/j.cyto.2020.155193
positive Expression: MAPK1 --+> S18082140 10.1016/j.febslet.2007.12.006
positive Expression: MAPK1 --+> S17544501 10.1016/j.biomaterials.2007.05.007
positive Expression: MAPK1 --+> S14563548 10.1016/S0378-1119(03)00748-0
positive Expression: MAPK1 --+> S20211246 10.1016/j.freeradbiomed.2010.02.022
positive Expression: MAPK1 --+> S20667472 10.1016/j.bbadis.2010.07.010
positive Expression: MAPK1 --+> S21810479 10.1016/j.biocel.2011.07.008
positive Expression: MAPK1 --+> S18242159 10.1016/j.bone.2007.11.019
positive Expression: MAPK1 --+> S20850578 10.1016/j.bone.2010.09.016
positive Expression: MAPK1 --+> S25150530 10.1016/j.archoralbio.2014.07.013
positive Expression: MAPK1 --+> S25986148 10.1016/j.yjmcc.2015.05.006
positive Expression: MAPK1 --+> S26828266 10.1016/j.bbrc.2016.01.142
positive Expression: MAPK1 --+> S15905316
positive Expression: MAPK1 --+> S15905316
positive Expression: MAPK1 --+> S18390540
positive Expression: MAPK1 --+> S15084239
positive Expression: MAPK1 --+> S11278573
positive Expression: MAPK1 --+> S12970113
positive Expression: MAPK1 --+> S12522128
positive Expression: MAPK1 --+> S12234285
positive Expression: MAPK1 --+> S18156195
positive Expression: MAPK1 --+> S28978514 10.1152/ajpregu.00443.2016
positive Expression: MAPK1 --+> S30001635 10.1177/0963689718756070
positive Expression: MAPK1 --+> S29845241 10.3892/mmr.2018.9052
positive Expression: MAPK1 --+> S29917166 10.3892/or.2018.6504
positive Expression: MAPK1 --+> S30587607 10.21873/invivo.11443

positive Expression: MAPK1 --+> S 31766280 10.3390/ijms20225690
positive Expression: MAPK1 --+> S 32924371 10.1515/hsz-2020-0125
positive Expression: MAPK1 --+> S 22797927 10.1242/jcs.093005
positive Expression: MAPK1 --+> S 21406114 10.1186/1471-2121-12-11
positive Expression: MAPK1 --+> S 21406114 10.1186/1471-2121-12-11
positive Expression: MAPK1 --+> S 21406114 10.1186/1471-2121-12-11
positive Expression: MAPK1 --+> S 21406114 10.1186/1471-2121-12-11
positive Expression: MAPK1 --+> S 21406114 10.1186/1471-2121-12-11
positive Expression: MAPK1 --+> S 23744338 10.1007/s13238-013-3003-3
positive Expression: MAPK1 --+> S 23781879 10.1089/dna.2013.2070
positive Expression: MAPK1 --+> S 24099083 10.1146/annurev-cellbio-101512-122340
positive Expression: MAPK1 --+> S 24446436 10.1074/jbc.M113.536300
positive Expression: MAPK1 --+> S 21849669 10.1152/ajpcell.00518.2010
positive Expression: MAPK1 --+> S 11278573 10.1074/jbc.M009846200
positive Expression: MAPK1 --+> S 11716472 10.1006/abbi.2001.2578
positive Expression: MAPK1 --+> S 11716472 10.1006/abbi.2001.2578
positive Expression: MAPK1 --+> S 12815619 10.1002/dvdy.10319
positive Expression: MAPK1 --+> S 16440309 10.1002/jcb.20697
positive Expression: MAPK1 --+> S 16604287 10.1007/s00223-005-0231-4
positive Expression: MAPK1 --+> S 16778192 10.1158/0008-5472.CAN-05-3735
positive Expression: MAPK1 --+> S 18156195 10.1152/ajpheart.00913.2007
positive Expression: MAPK1 --+> S 18712054 10.1291/hypres.31.98710.1291/hypres.31.987
positive Expression: MAPK1 --+> S 19664780 10.1016/j.jtcvs.2009.06.02410.1016/j.jtcvs.20
positive Expression: MAPK1 --+> S 19798549 10.1007/s11010-009-0276-110.1007/s11010-009-0
positive Expression: MAPK1 --+> S 20427276 10.1074/jbc.M110.11031210.1074/jbc.M110.11031
positive Expression: MAPK1 --+> S 21406114 10.1186/1471-2121-12-1110.1186/1471-2121-12-1
positive Expression: MAPK1 --+> S 22588951 10.1007/s12020-012-9675-210.1007/s12020-012-9
positive Expression: MAPK1 --+> S 24446436 10.1074/jbc.M113.53630010.1074/jbc.M113.53630
positive Expression: MAPK1 --+> S 24530412 10.1016/j.intimp.2014.01.02810.1016/j.intimp.
positive Expression: MAPK1 --+> S 25150530 10.1016/j.archoralbio.2014.07.01310.1016/j.ar
positive Expression: MAPK1 --+> S 30001635 10.1177/096368971875607010.1177/0963689718756
positive Expression: MAPK1 --+> SPP1 10.21873/invivo.11443
positive Expression: MAPK1 --+> SPP1 10.3390/ijms20225690
positive Expression: MAPK1 --+> SPP1 10.1074/jbc.M113.536300
positive Expression: MAPK1 --+> S 19573532 10.1016/j.yjmcc.2009.06.015
positive Expression: MAPK1 --+> S 19573532 10.1016/j.yjmcc.2009.06.015
positive Expression: MAPK1 --+> S 19664780 10.1016/j.jtcvs.2009.06.024
positive Expression: MAPK1 --+> S 19664780 10.1016/j.jtcvs.2009.06.024
positive Expression: MAPK1 --+> S 21147284 10.1016/j.bone.2010.12.002
positive Expression: MAPK1 --+> S 21147284 10.1016/j.bone.2010.12.002
positive Expression: MAPK1 --+> SPP1 10.1016/j.bone.2012.02.197
positive Expression: MAPK1 --+> S 18390540
positive Expression: MAPK1 --+> S 18390540
positive Expression: MAPK1 --+> S 18390540
positive Expression: MAPK1 --+> S 15557322
positive Expression: MAPK1 --+> S 15557322
positive Expression: MAPK1 --+> S 12920127
positive Expression: MAPK1 --+> S 12920127
positive Expression: MAPK1 --+> S 12920127
positive Expression: MAPK1 --+> S 12920127
positive Expression: MAPK1 --+> S 20127409 10.1007/s10741-010-9158-6
positive Expression: MAPK1 --+> S 22814749 10.1161/ATVBAHA.112.255216
positive Expression: MAPK1 --+> S 22814749 10.1161/ATVBAHA.112.255216
positive Expression: MAPK1 --+> S 23744338 10.1007/s13238-013-3003-3

positive Expression: MAPK1 --+> S 25352086 10.3892/ijmm.2014.1978
positive Expression: MAPK1 --+> S 14755545
positive Expression: MAPK1 --+> S 14755545
positive Expression: MAPK1 --+> S 15557322
positive Expression: MAPK1 --+> S 18390540 10.1074/jbc.M800668200
positive Expression: MAPK1 --+> S 19358273 10.1002/ijc.24388
positive Expression: MAPK1 --+> S 16978937 10.1016/j.bone.2006.07.024
positive Expression: MAPK1 --+> S 16978937 10.1016/j.bone.2006.07.024
positive Expression: MAPK1 --+> S 24530412 10.1016/j.intimp.2014.01.028
positive Expression: MAPK1 --+> S 15084239
positive Expression: MAPK1 --+> S 19246678
positive Expression: MAPK1 --+> S 15671080
positive Expression: MAPK1 --+> S 21406114 10.1186/1471-2121-12-11
positive Expression: MAPK1 --+> S 21468757 10.1007/s00726-011-0901-7
positive Expression: MAPK1 --+> S 23781879 10.1089/dna.2013.2070
positive Expression: MAPK1 --+> S 23233539 10.1152/ajpendo.00460.2012
positive Expression: MAPK1 --+> SPP1
positive Expression: MAPK1 --+> S 29138812 10.3892/mmr.2017.8021
positive Expression: MAPK1 --+> S 29359350 10.1111/imcb.1201010.1111/imcb.12010
positive Expression: MAPK1 --+> SPP1 10.1074/jbc.M117.807339
positive Expression: MAPK1 --+> S 29359350 10.1111/imcb.12010
positive Expression: MAPK1 --+> S 29359350 10.1111/imcb.12010
positive Expression: MAPK1 --+> S 29694967 10.1159/000489206
positive Expression: MAPK1 --+> S 30497779 10.1016/j.bbrc.2018.11.07010.1016/j.bbrc.2018
positive Expression: MAPK1 --+> S 34435888 10.1089/ars.2020.8159
positive Expression: MAPK1 --+> S 35510038 10.1155/2022/5013622
positive Regulation: SPP1 --+> MA 14704150 10.1074/jbc.M310256200
positive Regulation: SPP1 --+> MA 14704150 10.1074/jbc.M310256200
positive Regulation: SPP1 --+> MA 14704150 10.1074/jbc.M310256200
positive Regulation: SPP1 --+> MA 14704150 10.1074/jbc.M310256200
positive Regulation: SPP1 --+> MA 14704150 10.1074/jbc.M310256200
positive Regulation: SPP1 --+> MA 14704150 10.1074/jbc.M310256200
positive Regulation: SPP1 --+> MA 14704150 10.1074/jbc.M310256200
positive Regulation: SPP1 --+> MA 14704150 10.1074/jbc.M310256200
positive Regulation: SPP1 --+> MA 14704150 10.1074/jbc.M310256200
positive Regulation: SPP1 --+> MA 14704150 10.1074/jbc.M310256200
positive Regulation: SPP1 --+> MA 14704150 10.1074/jbc.M310256200
positive Regulation: SPP1 --+> MA 15757900 10.1074/jbc.M414204200
positive Regulation: SPP1 --+> MA 15757900 10.1074/jbc.M414204200
positive Regulation: SPP1 --+> MA 15757900 10.1074/jbc.M414204200
positive Regulation: SPP1 --+> MA 15757900 10.1074/jbc.M414204200
positive Regulation: SPP1 --+> MA 15757900 10.1074/jbc.M414204200
positive Regulation: SPP1 --+> MA 15757900 10.1074/jbc.M414204200
positive Regulation: SPP1 --+> MA 15757900 10.1074/jbc.M414204200
positive Regulation: SPP1 --+> MA 15757900 10.1074/jbc.M414204200
positive Regulation: SPP1 --+> MA 15757900 10.1074/jbc.M414204200
positive Regulation: SPP1 --+> MA 15757900 10.1074/jbc.M414204200
positive Regulation: SPP1 --+> MA 15247285 10.1074/jbc.M404674200
positive Regulation: SPP1 --+> MA 15247285 10.1074/jbc.M404674200
positive Regulation: SPP1 --+> MA 15180992 10.1074/jbc.M404907200
positive Regulation: SPP1 --+> MA 11278600 10.1074/jbc.M010021200
positive Regulation: SPP1 --+> MA 30537505 10.1016/j.ijbiomac.2018.12.075
positive Regulation: SPP1 --+> MA 21703197 10.1016/j.jhep.2011.02.036
positive Regulation: SPP1 --+> MA 21703197 10.1016/j.jhep.2011.02.036

positive Regulation: SPP1 --+> M/28668580 10.1016/j.jhep.2017.06.020
positive Regulation: SPP1 --+> M/27702700 10.1016/j.arr.2016.09.008
positive Regulation: SPP1 --+> M/32339903 10.1016/j.scr.2020.101799
positive Regulation: SPP1 --+> MAPK1 10.1016/j.mgene.2018.05.006
positive Regulation: SPP1 --+> M/20363925 10.2353/ajpath.2010.090810
positive Regulation: SPP1 --+> M/18632157 10.1016/j.molimm.2008.06.008
positive Regulation: SPP1 --+> M/18632157 10.1016/j.molimm.2008.06.008
positive Regulation: SPP1 --+> M/18996613 10.1016/j.lungcan.2008.09.003
positive Regulation: SPP1 --+> M/18996613 10.1016/j.lungcan.2008.09.003
positive Regulation: SPP1 --+> M/18996613 10.1016/j.lungcan.2008.09.003
positive Regulation: SPP1 --+> M/18996613 10.1016/j.lungcan.2008.09.003
positive Regulation: SPP1 --+> M/18996613 10.1016/j.lungcan.2008.09.003
positive Regulation: SPP1 --+> M/19857547 10.1016/j.exphem.2009.10.010
positive Regulation: SPP1 --+> M/20211246 10.1016/j.freeradbiomed.2010.02.022
positive Regulation: SPP1 --+> M/16406521 10.1016/j.tcb.2005.12.005
positive Regulation: SPP1 --+> M/11872370 10.1016/S1056-8727(01)00189-1
positive Regulation: SPP1 --+> M/11872370 10.1016/S1056-8727(01)00189-1
positive Regulation: SPP1 --+> M/11872370 10.1016/S1056-8727(01)00189-1
positive Regulation: SPP1 --+> M/12618529
positive Regulation: SPP1 --+> M/15757900
positive Regulation: SPP1 --+> M/15757900
positive Regulation: SPP1 --+> M/15757900
positive Regulation: SPP1 --+> M/15757900
positive Regulation: SPP1 --+> M/15757900
positive Regulation: SPP1 --+> M/15757900
positive Regulation: SPP1 --+> M/15757900
positive Regulation: SPP1 --+> M/15757900
positive Regulation: SPP1 --+> M/15757900
positive Regulation: SPP1 --+> M/15757900
positive Regulation: SPP1 --+> M/15757900
positive Regulation: SPP1 --+> M/15247285
positive Regulation: SPP1 --+> M/15247285
positive Regulation: SPP1 --+> M/15180992
positive Regulation: SPP1 --+> M/14704150
positive Regulation: SPP1 --+> M/14704150
positive Regulation: SPP1 --+> M/14704150
positive Regulation: SPP1 --+> M/14704150
positive Regulation: SPP1 --+> M/14704150
positive Regulation: SPP1 --+> M/14704150
positive Regulation: SPP1 --+> M/14704150
positive Regulation: SPP1 --+> M/14704150
positive Regulation: SPP1 --+> M/14704150
positive Regulation: SPP1 --+> M/14704150
positive Regulation: SPP1 --+> M/14704150
positive Regulation: SPP1 --+> M/14704150
positive Regulation: SPP1 --+> M/14704150
positive Regulation: SPP1 --+> M/11278600
positive Regulation: SPP1 --+> M/10712383
positive Regulation: SPP1 --+> M/10712383
positive Regulation: SPP1 --+> M/10712383
positive Regulation: SPP1 --+> M/10712383
positive Regulation: SPP1 --+> M/18156195
positive Regulation: SPP1 --+> M/24818727 10.1007/978-3-0348-0837-8_11
positive Regulation: SPP1 --+> M/29415992 10.1038/s41419-017-0226-x

positive Regulation: SPP1 --+>	M/20828639	10.1016/j.bone.2010.08.02510.1016/j.bone.2010
positive Regulation: SPP1 --+>	M/29415992	10.1038/s41419-017-0226-x
positive Regulation: SPP1 --+>	M/29415992	10.1038/s41419-017-0226-x
positive Regulation: SPP1 --+>	M/29415992	10.1038/s41419-017-0226-x
positive Regulation: SPP1 --+>	M/29415992	10.1038/s41419-017-0226-x
positive Regulation: SPP1 --+>	M/29415992	10.1038/s41419-017-0226-x
positive Regulation: SPP1 --+>	MAPK1	10.1042/BCJ20170205
positive Regulation: SPP1 --+>	M/30535434	10.3892/ijo.2018.4656
positive Regulation: SPP1 --+>	M/32351594	10.1155/2020/3573240
positive Regulation: SPP1 --+>	M/21713404	10.1007/s11033-011-1128-0
positive Regulation: SPP1 --+>	M/23086356	10.1007/s12013-012-9449-8
positive Regulation: SPP1 --+>	M/23086356	10.1007/s12013-012-9449-8
positive Regulation: SPP1 --+>	M/20874252	10.1089/jir.2010.0082
positive Regulation: SPP1 --+>	M/21467192	10.1210/en.2010-1328
positive Regulation: SPP1 --+>	M/25777876	10.1186/s13054-015-0782-3
positive Regulation: SPP1 --+>	M/25777876	10.1186/s13054-015-0782-3
positive Regulation: SPP1 --+>	M/25777876	10.1186/s13054-015-0782-3
positive Regulation: SPP1 --+>	M/26458814	10.1186/s12943-015-0449-3
positive Regulation: SPP1 --+>	M/26458814	10.1186/s12943-015-0449-3
positive Regulation: SPP1 --+>	M/10712383	10.1161/01.atv.20.3.624
positive Regulation: SPP1 --+>	M/11872370	10.1016/s1056-8727(01)00189-1
positive Regulation: SPP1 --+>	M/14704150	10.1074/jbc.M310256200
positive Regulation: SPP1 --+>	M/15757900	10.1074/jbc.M414204200
positive Regulation: SPP1 --+>	M/18996613	10.1016/j.lungcan.2008.09.00310.1016/j.lungca
positive Regulation: SPP1 --+>	M/19475568	10.1002/jcp.2183510.1002/jcp.21835
positive Regulation: SPP1 --+>	M/19597469	10.1038/onc.2009.18910.1038/onc.2009.189
positive Regulation: SPP1 --+>	M/20056194	10.1016/j.archoralbio.2009.12.00310.1016/j.ar
positive Regulation: SPP1 --+>	M/25208103	10.1097/MIB.000000000000019210.1097/MIB.00000
positive Regulation: SPP1 --+>	M/29415992	10.1038/s41419-017-0226-x10.1038/s41419-017-0
positive Regulation: SPP1 --+>	M/19285028	10.1016/j.bbrc.2009.01.157
positive Regulation: SPP1 --+>	M/18632157	10.1016/j.molimm.2008.06.008
positive Regulation: SPP1 --+>	M/18996613	10.1016/j.lungcan.2008.09.003
positive Regulation: SPP1 --+>	M/20385232	10.1016/j.matbio.2010.04.001
positive Regulation: SPP1 --+>	M/20828639	10.1016/j.bone.2010.08.025
positive Regulation: SPP1 --+>	M/21147284	10.1016/j.bone.2010.12.002
positive Regulation: SPP1 --+>	M/23089492	10.1016/j.humpath.2012.07.007
positive Regulation: SPP1 --+>	M/20868520	
positive Regulation: SPP1 --+>	M/20868520	
positive Regulation: SPP1 --+>	M/20868520	
positive Regulation: SPP1 --+>	M/20868520	
positive Regulation: SPP1 --+>	M/20868520	
positive Regulation: SPP1 --+>	M/20868520	
positive Regulation: SPP1 --+>	M/20868520	
positive Regulation: SPP1 --+>	M/20868520	
positive Regulation: SPP1 --+>	M/15757900	
positive Regulation: SPP1 --+>	M/15247285	
positive Regulation: SPP1 --+>	M/15247285	
positive Regulation: SPP1 --+>	M/15247285	
positive Regulation: SPP1 --+>	M/15247285	
positive Regulation: SPP1 --+>	M/15247285	
positive Regulation: SPP1 --+>	M/15247285	
positive Regulation: SPP1 --+>	M/15180992	
positive Regulation: SPP1 --+>	M/14704150	
positive Regulation: SPP1 --+>	M/14704150	

positive Regulation: SPP1 --+> M 14704150
positive Regulation: SPP1 --+> M 14704150
positive Regulation: SPP1 --+> M 14704150
positive Regulation: SPP1 --+> M 14704150
positive Regulation: SPP1 --+> M 14704150
positive Regulation: SPP1 --+> M 14704150
positive Regulation: SPP1 --+> M 14704150
positive Regulation: SPP1 --+> M 14704150
positive Regulation: SPP1 --+> M 14704150
positive Regulation: SPP1 --+> M 14704150
positive Regulation: SPP1 --+> M 15247285
positive Regulation: SPP1 --+> M 14704150
positive Regulation: SPP1 --+> M 14704150
positive Regulation: SPP1 --+> M 14704150
positive Regulation: SPP1 --+> M 14704150
positive Regulation: SPP1 --+> M 14704150
positive Regulation: SPP1 --+> M 12920127
positive Regulation: SPP1 --+> M 20868520 10.1186/1476-4598-9-260
positive Regulation: SPP1 --+> M 20868520 10.1186/1476-4598-9-260
positive Regulation: SPP1 --+> M 20868520 10.1186/1476-4598-9-260
positive Regulation: SPP1 --+> M 20868520 10.1186/1476-4598-9-260
positive Regulation: SPP1 --+> M 20868520 10.1186/1476-4598-9-260
positive Regulation: SPP1 --+> M 20868520 10.1186/1476-4598-9-260
positive Regulation: SPP1 --+> M 20868520 10.1186/1476-4598-9-260
positive Regulation: SPP1 --+> M 23728342 10.1038/onc.2013.184
positive Regulation: SPP1 --+> M 24756245 10.3892/ijo.2014.2393
positive Regulation: SPP1 --+> M 24756245 10.3892/ijo.2014.2393
positive Regulation: SPP1 --+> MAPK1 10.1161/ATVBAHA.113.303017
positive Regulation: SPP1 --+> M 26063293 10.3892/ijmm.2015.2243
positive Regulation: SPP1 --+> M 26099282 10.3892/mmr.2015.3975
positive Regulation: SPP1 --+> M 14704150
positive Regulation: SPP1 --+> M 16691505
positive Regulation: SPP1 --+> M 19597469 10.1038/onc.2009.189
positive Regulation: SPP1 --+> M 20868520 10.1186/1476-4598-9-260
positive Regulation: SPP1 --+> M 20868520 10.1186/1476-4598-9-260
positive Regulation: SPP1 --+> M 28716167 10.2500/ajra.2017.31.4449
positive Regulation: SPP1 --+> M 29065446 10.3390/ijms18102198
positive Regulation: SPP1 --+> M 29223704 10.1016/j.actbio.2017.11.046
positive Regulation: SPP1 --+> M 29500465 10.1038/s41419-018-0391-6
positive Regulation: SPP1 --+> M 29500465 10.1038/s41419-018-0391-6
positive Regulation: SPP1 --+> M 33603163 10.1038/s41388-021-01663-2
positive Regulation: SPP1 --+> M 33603163 10.1038/s41388-021-01663-2
positive Regulation: SPP1 --+> MAPK1 10.1530/ERC-20-0517
positive Regulation: SPP1 --+> M 33527556 10.1002/JLB.2A0220-127R
positive Regulation: SPP1 --+> M 34664250 10.1007/978-3-030-73119-9_20
positive Regulation: SPP1 --+> MAPK1
positive Regulation: SPP1 --+> MAPK1 10.1016/j.jceh.2022.05.004
positive Regulation: SPP1 --+> M 35580963 10.1136/gutjnl-2022-327099
negative Regulation: AKT1 ---| c 27038543 10.1016/j.bbrc.2016.03.141
negative Regulation: AKT1 ---| c 27666600 10.1016/j.bcp.2016.09.022
negative Regulation: AKT1 ---| c 33011222 10.1016/j.lfs.2020.118513
negative Regulation: AKT1 ---| c 26276725 10.1016/j.canlet.2015.08.004
negative Regulation: AKT1 ---| c 29753186 10.1016/j.tranon.2018.04.011
negative Regulation: AKT1 ---| c 22387880 10.1016/j.regpep.2012.02.003

negative Regulation: AKT1 ---| cł20079806 10.1016/j.bbcan.2010.01.002
negative Regulation: AKT1 ---| cł22208970 10.1016/j.ijrobp.2011.09.040
negative Regulation: AKT1 ---| cł19651067 10.1016/j.jamcollsurg.2009.03.006
negative Regulation: AKT1 ---| cł24184201 10.1016/j.biopha.2013.10.003
negative Regulation: AKT1 ---| cł21108789
negative Regulation: AKT1 ---| cł19078992
negative Regulation: AKT1 ---| cł15150572
negative Regulation: AKT1 ---| cł12373610
negative Regulation: AKT1 ---| cł27473470 10.1007/s10571-016-0410-z
negative Regulation: AKT1 ---| cł28440463 10.3892/or.2017.5584
negative Regulation: AKT1 ---| cł28849180 10.3892/or.2017.5908
negative Regulation: AKT1 ---| cł28887606 10.1007/s10616-017-0134-z
negative Regulation: AKT1 ---| cł30594239 10.1186/s40659-018-0205-4
negative Regulation: AKT1 ---| chemosensitivity
negative Regulation: AKT1 ---| cł31665911 10.1177/0963689719885083
negative Regulation: AKT1 ---| cł31665911 10.1177/0963689719885083
negative Regulation: AKT1 ---| cł32945500 10.3892/or.2020.7710
negative Regulation: AKT1 ---| cł33499874 10.1186/s13046-021-01854-5
negative Regulation: AKT1 ---| cł33591948 10.18632/aging.202488
negative Regulation: AKT1 ---| cł22873219
negative Regulation: AKT1 ---| cł22873219
negative Regulation: AKT1 ---| cł20878074 10.3892/ijo-00000778
negative Regulation: AKT1 ---| cł21444628 10.1124/jpet.111.179663
negative Regulation: AKT1 ---| cł22312312 10.3390/ijms13011186
negative Regulation: AKT1 ---| cł21108789 10.1186/1476-4598-9-305
negative Regulation: AKT1 ---| cł23743572 10.3892/or.2013.2520
negative Regulation: AKT1 ---| cł23743572 10.3892/or.2013.2520
negative Regulation: AKT1 ---| cł23743572 10.3892/or.2013.2520
negative Regulation: AKT1 ---| cł23613505 10.1177/0300060513480920
negative Regulation: AKT1 ---| cł24232099 10.1038/cddis.2013.450
negative Regulation: AKT1 ---| cł24232099 10.1038/cddis.2013.450
negative Regulation: AKT1 ---| cł24255131 10.1074/mcp.M113.034025
negative Regulation: AKT1 ---| cł24454800 10.1371/journal.pone.0085113
negative Regulation: AKT1 ---| cł24104394 10.1038/leu.2013.291
negative Regulation: AKT1 ---| cł24784001 10.1016/j.neo.2014.03.008
negative Regulation: AKT1 ---| cł24722292 10.1038/cddis.2014.136
negative Regulation: AKT1 ---| cł25230779 10.3892/or.2014.3488
negative Regulation: AKT1 ---| cł25230779 10.3892/or.2014.3488
negative Regulation: AKT1 ---| cł21273759 10.1159/000322986
negative Regulation: AKT1 ---| cł21597260 10.1159/000322849
negative Regulation: AKT1 ---| cł26820593 10.3892/or.2016.4590
negative Regulation: AKT1 ---| cł27220401 10.3892/or.2016.4820
negative Regulation: AKT1 ---| cł15150572 10.1038/sj.bjc.6601876
negative Regulation: AKT1 ---| cł18071906 10.1007/s10495-007-0165-6
negative Regulation: AKT1 ---| cł18224693 10.1002/ijc.2337110.1002/ijc.23371
negative Regulation: AKT1 ---| cł19375813 10.1016/j.lungcan.2009.03.01510.1016/j.lungca
negative Regulation: AKT1 ---| cł19625496 10.1158/1535-7163.MCT-09-014810.1158/1535-716
negative Regulation: AKT1 ---| cł20944098
negative Regulation: AKT1 ---| cł21273759 10.1159/00032298610.1159/000322986
negative Regulation: AKT1 ---| cł21597260 10.1159/00032284910.1159/000322849
negative Regulation: AKT1 ---| cł23530461 10.1111/ced.1213810.1111/ced.12138
negative Regulation: AKT1 ---| cł23546174 10.3892/mmr.2013.140010.3892/mmr.2013.1400
negative Regulation: AKT1 ---| cł26629248
negative Regulation: AKT1 ---| cł27163202 10.1016/j.yexmp.2016.05.00310.1016/j.yexmp.20

negative Regulation: AKT1 ---| ch	27301639	10.1016/j.bbrc.2016.06.05710.1016/j.bbrc.2016
negative Regulation: AKT1 ---| ch	31001468	10.3389/fonc.2019.0018510.3389/fonc.2019.0018
negative Regulation: AKT1 ---| ch	32945500	10.3892/or.2020.771010.3892/or.2020.7710
negative Regulation: AKT1 ---| ch	33876384	10.1007/s12031-021-01832-8
negative Regulation: AKT1 ---| ch	34542810	10.1007/s11427-020-1978-4
negative Regulation: AKT1 ---| ch	34792179	10.3892/or.2021.8229
negative Regulation: AKT1 ---| ch	35257265	10.1007/s10495-022-01718-z10.1007/s10495-022-
negative Regulation: AKT1 ---| ch	35354796	10.1038/s41419-022-04727-7
negative Regulation: AKT1 ---| ch	33783376	10.1097/CAD.0000000000001074
positive Expression: UCA1 --+> AI	33183321	10.1186/s13000-020-01054-3
positive ProtModification: MAPK1	11409876	10.1006/bbrc.2001.5043
positive ProtModification: MAPK1	10958792	10.1074/jbc.M004267200
positive ProtModification: MAPK1	11821415	10.1074/jbc.M111598200
positive ProtModification: MAPK1	11546806	10.1074/jbc.M105669200
positive ProtModification: MAPK1	14734651	10.1242/jcs.00897
positive ProtModification: MAPK1	15078887	10.1074/jbc.M312135200
positive ProtModification: MAPK1	10781582	10.1074/jbc.M001020200
positive ProtModification: MAPK1	12890671	10.1074/jbc.M307149200
positive ProtModification: MAPK1	15116093	10.1038/sj.onc.1207426
positive ProtModification: MAPK1	10547071	
positive ProtModification: MAPK1 --+> TP53		
positive ProtModification: MAPK1 --+> TP53		
positive Regulation: TMEM45A --+	31585906	10.2741/4823
Expression: MAPK1 ---> GCLC	29066411	10.1016/j.fct.2017.10.033
Expression: MAPK1 ---> GCLC	26424114	10.1016/j.freeradbiomed.2015.08.011
Expression: MAPK1 ---> GCLC	28188924	10.1016/j.freeradbiomed.2017.02.016
Expression: MAPK1 ---> GCLC		10.1016/S0006-291X(02)00816-1
Expression: MAPK1 ---> GCLC	19328227	10.1016/j.freeradbiomed.2009.03.016
Expression: MAPK1 ---> GCLC	19328227	10.1016/j.freeradbiomed.2009.03.016
Expression: MAPK1 ---> GCLC	19328227	10.1016/j.freeradbiomed.2009.03.016
Expression: MAPK1 ---> GCLC	25201354	10.1016/j.tox.2014.09.002
Expression: MAPK1 ---> GCLC	22864849	10.1007/s00204-012-0913-410.1007/s00204-012-0
Expression: MAPK1 ---> GCLC	26424114	10.1016/j.freeradbiomed.2015.08.01110.1016/j.
positive Expression: CYP2J2 --+>	29427791	10.1016/j.freeradbiomed.2018.02.009
positive Expression: MAPK1 --+> N	20085809	10.1016/j.mod.2010.01.002
positive Expression: MAPK1 --+> N	29472716	10.1038/s41418-018-0077-8
positive Expression: MAPK1 --+> N	29472716	10.1038/s41418-018-0077-8
positive Expression: MAPK1 --+> N	24392017	10.1371/journal.pone.0085502
positive Expression: MAPK1 --+> N	32792479	10.1038/s41419-020-02873-4
positive Expression: MAPK1 --+> N	22787275	10.1091/mbc.E12-02-0132
positive Expression: MAPK1 --+> N	22646022	10.1139/Y2012-053
positive Expression: MAPK1 --+> N	23566955	10.1016/j.taap.2013.02.017
positive Expression: MAPK1 --+> N	22455903	10.1016/j.tice.2012.03.001
positive Expression: MAPK1 --+> N	17643270	10.1016/j.mod.2007.06.004
positive Expression: MAPK1 --+> NOTCH1		10.1016/j.tice.2012.03.001
positive Expression: MAPK1 --+> N	9390685	10.1016/S1074-7613(00)80382-9
positive Expression: MAPK1 --+> N	22682244	10.1016/j.cell.2012.03.048
positive Expression: MAPK1 --+> N	16522205	
positive Expression: MAPK1 --+> N	21552419	
positive Expression: MAPK1 --+> N	25723392	10.1371/journal.pone.0119723
positive Expression: MAPK1 --+> N	25894826	10.1038/nm.3849
positive Expression: MAPK1 --+> NOTCH1		
negative Regulation: GCLC ---| RA	31283930	10.1016/j.bcp.2019.07.001
positive Expression: CD274 --+> P	31799599	10.1042/BSR20191041

positive Expression: CD274 --+> E 31799599 10.1042/BSR20191041
positive Expression: CD274 --+> E 31799599 10.1042/BSR2019104110.1042/BSR20191041
Regulation: BECN1 ---> CD274 31503548 10.1172/JCI127721
Regulation: BECN1 ---> CD274 33323966 10.1038/s41388-020-01592-6
Regulation: NGF ---> chemosensiti 9831249 10.1016/S0168-0102(98)00060-1
Regulation: NGF ---> chemosensiti 11164258 10.1016/S0168-0102(00)00201-7
Regulation: NGF ---> chemosensiti 29875100 10.2337/db17-1306
Regulation: NGF ---> chemosensiti 21499209 10.1097/MPA.0b013e318214fb77
Regulation: NGF ---> chemosensiti 7623122
Regulation: NGF ---> chemosensiti 7623122
Regulation: NGF ---> chemosensitivity
negative Expression: ING4 ---| CI 22842937 10.3892/or.2012.1930
negative Expression: ING4 ---| CI 22842937 10.3892/or.2012.1930
negative Expression: ING4 ---| CI 22842937 10.3892/or.2012.1930
negative Expression: ING4 ---| CI 20707719 10.1089/cbr.2010.0778
negative Expression: ING4 ---| CI 21988227 10.1089/cbr.2011.1047
negative Expression: ING4 ---| CI 21052098 10.1038/cgt.2010.67
negative Expression: ING4 ---| CI 21052098 10.1038/cgt.2010.67
negative Expression: ING4 ---| CI 21052098 10.1038/cgt.2010.67
negative Expression: ING4 ---| CI 21660060 10.1038/cgt.2011.31
negative Expression: ING4 ---| CI 21052098 10.1038/cgt.2010.6710.1038/cgt.2010.67
negative Expression: ING4 ---| CI 22842937 10.3892/or.2012.193010.3892/or.2012.1930
positive DirectRegulation: KDM5B 27584795 10.1038/cddis.2016.26210.1038/cddis.2016.262
positive DirectRegulation: KDM5B 25909289 10.18632/oncotarget.3713
positive DirectRegulation: KDM5B 30628696 10.3892/mmr.2019.9832
positive DirectRegulation: KDM5B 30728054 10.1186/s13046-019-1063-9
positive DirectRegulation: KDM5B 27584795 10.1038/cddis.2016.262
positive DirectRegulation: KDM5B 27584795 10.1038/cddis.2016.262
positive DirectRegulation: KDM5B 27584795 10.1038/cddis.2016.26210.1038/cddis.2016.262
positive DirectRegulation: KDM5B 27584795 10.1038/cddis.2016.262
positive DirectRegulation: KDM5B 32868382 10.1158/0008-5472.CAN-20-050510.1158/0008-547
positive DirectRegulation: KDM5B --+> AKT1 10.3892/mmr.2019.9832
positive DirectRegulation: KDM5B --+> AKT1 10.1186/s13046-019-1063-9
positive DirectRegulation: KDM5B 35563264 10.3390/ijms23094874
Expression: PPARG ---> MCL1 10.1016/S1074-7613(04)00030-5
Expression: PPARG ---> MCL1 25944662 10.1016/j.bbrc.2015.04.023
Expression: PPARG ---> MCL1 29928066 10.1371/journal.ppat.100710010.1371/journal.p
Expression: PPARG ---> MCL1
Expression: PPARG ---> MCL1 607 10.1016/j.tim.2018.08.001
PromoterBinding: TP53 ---> DST 16343436 10.1016/j.bbrc.2005.11.118
PromoterBinding: TP53 ---> DST 15982302
PromoterBinding: TP53 ---> DST
negative Expression: PAX8 ---| TF 14627715 10.1074/jbc.M306824200
negative Expression: PAX8 ---| TF 10980123 10.1016/S0002-9440(10)64597-X
negative Expression: PAX8 ---| TF 16904651 10.1016/j.bcp.2006.06.024
negative Expression: PAX8 ---| TF 9459485 10.1016/S0167-4889(97)00119-5
negative Expression: PAX8 ---| TF 14627715
negative Expression: PAX8 ---| TF 11914272
negative Expression: PAX8 ---| TF 19139719
negative Expression: PAX8 ---| TF 29379162 10.1038/s41388-017-0040-z
negative Expression: PAX8 ---| TF 29379162 10.1038/s41388-017-0040-z
negative Expression: PAX8 ---| TF 29379162 10.1038/s41388-017-0040-z
negative Expression: PAX8 ---| TF 29379162 10.1038/s41388-017-0040-z
negative Expression: PAX8 ---| TF 29379162 10.1038/s41388-017-0040-z

negative Expression: PAX8 ---| TF 29997452 10.1186/s12935-018-0591-2
negative Expression: PAX8 ---| TF 21979593 10.1097/PGP.0b013e31821ac6c3
negative Expression: PAX8 ---| TF 25083965 10.1097/PGP.0b013e3182a54afa
negative Expression: PAX8 ---| TF 29379162 10.1038/s41388-017-0040-z10.1038/s41388-017-0
negative Expression: PAX8 ---| TF 34244607 10.1038/s41388-021-01925-z
negative Expression: PAX8 ---| TF 34244607 10.1038/s41388-021-01925-z
negative Expression: PAX8 ---| TF 34244607 10.1038/s41388-021-01925-z
negative Expression: PAX8 ---| TF 34244607 10.1038/s41388-021-01925-z
negative Expression: PAX8 ---| TF 23868062 10.1038/cddis.2013.262
negative Expression: PAX8 ---| TF 21876729 10.1371/journal.pone.0022312
negative Expression: PAX8 ---| TF 23868062 10.1038/cddis.2013.262
negative Expression: PAX8 ---| TF 29379162 10.1038/s41388-017-0040-z
negative Expression: PAX8 ---| TF 29379162 10.1038/s41388-017-0040-z
negative Expression: PAX8 ---| TP53 10.1016/j.pneurobio.2011.08.012
negative Expression: PAX8 ---| TP53
positive Regulation: SPP1 --+> AK 28359791 10.1016/j.bmcl.2017.03.047
positive Regulation: SPP1 --+> AK 29223704 10.1016/j.actbio.2017.11.046
positive Regulation: SPP1 --+> AK 29410240 10.1016/j.jprot.2018.01.017
positive Regulation: SPP1 --+> AK 20363925 10.2353/ajpath.2010.090810
positive Regulation: SPP1 --+> AK 17537408 10.1016/j.bbrc.2007.05.104
positive Regulation: SPP1 --+> AK 17537408 10.1016/j.bbrc.2007.05.104
positive Regulation: SPP1 --+> AK 19285028 10.1016/j.bbrc.2009.01.157
positive Regulation: SPP1 --+> AK 17306792 10.1016/j.yexcr.2006.12.026
positive Regulation: SPP1 --+> AK 19850036 10.1016/j.yexcr.2009.10.012
positive Regulation: SPP1 --+> AK 19850036 10.1016/j.yexcr.2009.10.012
positive Regulation: SPP1 --+> AK 19850036 10.1016/j.yexcr.2009.10.012
positive Regulation: SPP1 --+> AK 19850036 10.1016/j.yexcr.2009.10.012
positive Regulation: SPP1 --+> AK 19850036 10.1016/j.yexcr.2009.10.012
positive Regulation: SPP1 --+> AK 17977560 10.1016/j.lfs.2007.09.024
positive Regulation: SPP1 --+> AK 18632157 10.1016/j.molimm.2008.06.008
positive Regulation: SPP1 --+> AK 12965255 10.1016/S0165-5728(03)00223-6
positive Regulation: SPP1 --+> AK 21703197 10.1016/j.jhep.2011.02.036
positive Regulation: SPP1 --+> AK 21703197 10.1016/j.jhep.2011.02.036
positive Regulation: SPP1 --+> AK 18996613 10.1016/j.lungcan.2008.09.003
positive Regulation: SPP1 --+> AK 18996613 10.1016/j.lungcan.2008.09.003
positive Regulation: SPP1 --+> AK 21616556 10.1016/j.ejcb.2011.03.005
positive Regulation: SPP1 --+> AK 18694621 10.1016/j.canlet.2008.07.004
positive Regulation: SPP1 --+> AK 22542685 10.1016/j.ijdevneu.2012.04.003
positive Regulation: SPP1 --+> AK 20385232 10.1016/j.matbio.2010.04.001
positive Regulation: SPP1 --+> AK 16406521 10.1016/j.tcb.2005.12.005
positive Regulation: SPP1 --+> AK 16406521 10.1016/j.tcb.2005.12.005
positive Regulation: SPP1 --+> AK 20022267 10.1016/j.urolonc.2009.10.009
positive Regulation: SPP1 --+> AK 22750751 10.1016/j.bbcan.2012.06.005
positive Regulation: SPP1 --+> AK 24657887 10.1016/j.matbio.2014.03.001
positive Regulation: SPP1 --+> AK 24999034 10.1016/j.bbagrm.2014.06.020
positive Regulation: SPP1 --+> AK 25749339 10.1016/j.bbrc.2015.02.153
positive Regulation: SPP1 --+> AK 25779039 10.1016/j.brainres.2015.03.015
positive Regulation: SPP1 --+> AK 25779039 10.1016/j.brainres.2015.03.015
positive Regulation: SPP1 --+> AK 26116893 10.1016/j.cca.2015.06.017
positive Regulation: SPP1 --+> AK 25732057 10.1016/j.bbcan.2015.02.003
positive Regulation: SPP1 --+> AKT1 10.1378/chest.130.4_MeetingAbstracts.230S-c
positive Regulation: SPP1 --+> AKT1 10.1378/chest.130.4_MeetingAbstracts.230S-c
positive Regulation: SPP1 --+> AK 27063991 10.1016/j.lfs.2016.04.003
positive Regulation: SPP1 --+> AK 27063991 10.1016/j.lfs.2016.04.003

positive Regulation: SPP1 --+> AK 27063991 10.1016/j.lfs.2016.04.003
positive Regulation: SPP1 --+> AK 20868520
positive Regulation: SPP1 --+> AK 20868520
positive Regulation: SPP1 --+> AK 20868520
positive Regulation: SPP1 --+> AK 20868520
positive Regulation: SPP1 --+> AK 20868520
positive Regulation: SPP1 --+> AK 20868520
positive Regulation: SPP1 --+> AK 20868520
positive Regulation: SPP1 --+> AK 20459645
positive Regulation: SPP1 --+> AK 12771144
positive Regulation: SPP1 --+> AK 12771144
positive Regulation: SPP1 --+> AK 12771144
positive Regulation: SPP1 --+> AK 12771144
positive Regulation: SPP1 --+> AK 12771144
positive Regulation: SPP1 --+> AK 12771144
positive Regulation: SPP1 --+> AK 12771144
positive Regulation: SPP1 --+> AK 12771144
positive Regulation: SPP1 --+> AK 12771144
positive Regulation: SPP1 --+> AK 12771144
positive Regulation: SPP1 --+> AK 12771144
positive Regulation: SPP1 --+> AK 12771144
positive Regulation: SPP1 --+> AK 12771144
positive Regulation: SPP1 --+> AK 12771144
positive Regulation: SPP1 --+> AK 12771144
positive Regulation: SPP1 --+> AK 12771144
positive Regulation: SPP1 --+> AK 12771144
positive Regulation: SPP1 --+> AK 12771144
positive Regulation: SPP1 --+> AK 12771144
positive Regulation: SPP1 --+> AK 12460992
positive Regulation: SPP1 --+> AK 11590166
positive Regulation: SPP1 --+> AK 19706414
positive Regulation: SPP1 --+> AK 19706414
positive Regulation: SPP1 --+> AK 19706414
positive Regulation: SPP1 --+> AK 19706414
positive Regulation: SPP1 --+> AK 19706414
positive Regulation: SPP1 --+> AK 19706414
positive Regulation: SPP1 --+> AK 19234104
positive Regulation: SPP1 --+> AK 20224789
positive Regulation: SPP1 --+> AK 27411914 10.1186/s12885-016-2529-1
positive Regulation: SPP1 --+> AKT1
positive Regulation: SPP1 --+> AK 28716167 10.2500/ajra.2017.31.4449
positive Regulation: SPP1 --+> AK 28769537 10.1155/2017/4049098
positive Regulation: SPP1 --+> AK 29065446 10.3390/ijms18102198
positive Regulation: SPP1 --+> AK 29065446 10.3390/ijms18102198
positive Regulation: SPP1 --+> AK 29115557 10.3892/or.2017.6068
positive Regulation: SPP1 --+> AK 19850036 10.1016/j.yexcr.2009.10.012
positive Regulation: SPP1 --+> AK 20868520
positive Regulation: SPP1 --+> AK 22804248 10.2174/156652412802480826
positive Regulation: SPP1 --+> AK 22433222 10.1186/1479-5876-10-53
positive Regulation: SPP1 --+> AK 22847114 10.1007/s10719-012-9426-x
positive Regulation: SPP1 --+> AK 22847114 10.1007/s10719-012-9426-x
positive Regulation: SPP1 --+> AK 21940634 10.1074/jbc.M111.295048
positive Regulation: SPP1 --+> AK 21965655 10.1074/jbc.M111.250035
positive Regulation: SPP1 --+> AK 21965655 10.1074/jbc.M111.250035

positive Regulation: SPP1 --+> AK 16406521 10.1016/j.tcb.2005.12.005
positive Regulation: SPP1 --+> AK 20868520
positive Regulation: SPP1 --+> AK 12771144
positive Regulation: SPP1 --+> AK 12771144
positive Regulation: SPP1 --+> AK 19706414
positive Regulation: SPP1 --+> AK 12944477
positive Regulation: SPP1 --+> AK 22058329 10.1167/iovs.10-7050
positive Regulation: SPP1 --+> AK 20868520 10.1186/1476-4598-9-260
positive Regulation: SPP1 --+> AK 20868520 10.1186/1476-4598-9-260
positive Regulation: SPP1 --+> AK 20868520 10.1186/1476-4598-9-260
positive Regulation: SPP1 --+> AK 20868520 10.1186/1476-4598-9-260
positive Regulation: SPP1 --+> AK 20868520 10.1186/1476-4598-9-260
positive Regulation: SPP1 --+> AK 20868520 10.1186/1476-4598-9-260
positive Regulation: SPP1 --+> AK 20868520 10.1186/1476-4598-9-260
positive Regulation: SPP1 --+> AK 22444159 10.1186/1756-9966-31-26
positive Regulation: SPP1 --+> AK 21273567 10.1161/STROKEAHA.110.599118
positive Regulation: SPP1 --+> AK 23508962 10.1074/jbc.M112.432302
positive Regulation: SPP1 --+> AK 23630254 10.1073/pnas.1300492110
positive Regulation: SPP1 --+> AK 24008322 10.3892/ijmm.2013.1483
positive Regulation: SPP1 --+> AK 24008322 10.3892/ijmm.2013.1483
positive Regulation: SPP1 --+> AK 24008322 10.3892/ijmm.2013.1483
positive Regulation: SPP1 --+> AK 21909361 10.1371/journal.pone.0023831
positive Regulation: SPP1 --+> AK 21915300 10.1371/journal.pone.0024217
positive Regulation: SPP1 --+> AK 23152823 10.1371/journal.pone.0048933
positive Regulation: SPP1 --+> AK 24240095 10.1074/jbc.M113.492314
positive Regulation: SPP1 --+> AK 23728336 10.1038/onc.2013.171
positive Regulation: SPP1 --+> AK 24714722 10.3892/ijo.2014.2370
positive Regulation: SPP1 --+> AK 24714722 10.3892/ijo.2014.2370
positive Regulation: SPP1 --+> AK 24714722 10.3892/ijo.2014.2370
positive Regulation: SPP1 --+> AK 24810044 10.1038/cddis.2014.174
positive Regulation: SPP1 --+> AK 24498111 10.1371/journal.pone.0087464
positive Regulation: SPP1 --+> AK 24498111 10.1371/journal.pone.0087464
positive Regulation: SPP1 --+> AK 24756245 10.3892/ijo.2014.2393
positive Regulation: SPP1 --+> AK 24961145 10.1186/1756-9966-33-53
positive Regulation: SPP1 --+> AKT1 10.1161/ATVBAHA.113.303017
positive Regulation: SPP1 --+> AKT1 10.1530/JOE-14-0203
positive Regulation: SPP1 --+> AKT1 10.1530/JOE-14-0203
positive Regulation: SPP1 --+> AKT1
positive Regulation: SPP1 --+> AK 26099282 10.3892/mmr.2015.3975
positive Regulation: SPP1 --+> AK 26099282 10.3892/mmr.2015.3975
positive Regulation: SPP1 --+> AK 15678124
positive Regulation: SPP1 --+> AK 16012053
positive Regulation: SPP1 --+> AK 16012053
positive Regulation: SPP1 --+> AK 17483319
positive Regulation: SPP1 --+> AK 18849546 10.3181/0805-RM-164
positive Regulation: SPP1 --+> AK 18849546 10.3181/0805-RM-164
positive Regulation: SPP1 --+> AK 18694621 10.1016/j.canlet.2008.07.004
positive Regulation: SPP1 --+> AK 18985031 10.1038/mt.2008.235
positive Regulation: SPP1 --+> AK 18996613 10.1016/j.lungcan.2008.09.003
positive Regulation: SPP1 --+> AK 19597469 10.1038/onc.2009.189
positive Regulation: SPP1 --+> AK 19597469 10.1038/onc.2009.189
positive Regulation: SPP1 --+> AK 19706414 10.1073/pnas.0903704106
positive Regulation: SPP1 --+> AK 19850036 10.1016/j.yexcr.2009.10.012
positive Regulation: SPP1 --+> AK 19850036 10.1016/j.yexcr.2009.10.012

positive Regulation: SPP1 --+> AK	19850036	10.1016/j.yexcr.2009.10.012
positive Regulation: SPP1 --+> AK	19767100	10.1016/j.leukres.2009.08.028
positive Regulation: SPP1 --+> AK	20868520	10.1186/1476-4598-9-260
positive Regulation: SPP1 --+> AK	20868520	10.1186/1476-4598-9-260
positive Regulation: SPP1 --+> AK	21616556	10.1016/j.ejcb.2011.03.005
positive Regulation: SPP1 --+> AK	21909361	10.1371/journal.pone.0023831
positive Regulation: SPP1 --+> AK	25060796	10.1161/ATVBAHA.113.303017
positive Regulation: SPP1 --+> AK	25060796	10.1161/ATVBAHA.113.303017
positive Regulation: SPP1 --+> AK	24714722	10.3892/ijo.2014.2370
positive Regulation: SPP1 --+> AK	25208103	10.1097/MIB.0000000000000192
positive Regulation: SPP1 --+> AK	25352284	10.1530/JOE-14-0203
positive Regulation: SPP1 --+> AK	25817589	10.1016/j.bmcl.2015.03.024
positive Regulation: SPP1 --+> AK	25146847	10.1007/s12035-014-8859-6
positive Regulation: SPP1 --+> AKT1		
positive Regulation: SPP1 --+> AK	26681550	10.1021/acs.jnatprod.5b00796
positive Regulation: SPP1 --+> AK	25146847	10.1007/s12035-014-8859-6
positive Regulation: SPP1 --+> AK	25146847	10.1007/s12035-014-8859-6
positive Regulation: SPP1 --+> AK	28453726	10.1093/cvr/cvx01610.1093/cvr/cvx016
positive Regulation: SPP1 --+> AK	28716167	10.2500/ajra.2017.31.444910.2500/ajra.2017.31
positive Regulation: SPP1 --+> AK	29500465	10.1038/s41419-018-0391-6
positive Regulation: SPP1 --+> AKT1		10.12659/MSM.905700
positive Regulation: SPP1 --+> AK	28866734	10.1007/s12035-017-0734-9
positive Regulation: SPP1 --+> AK	28866734	10.1007/s12035-017-0734-9
positive Regulation: SPP1 --+> AK	30001635	10.1177/0963689718756070
positive Regulation: SPP1 --+> AKT1		10.1042/BCJ20170205
positive Regulation: SPP1 --+> AK	30250599	10.3892/ol.2018.928110.3892/ol.2018.9281
positive Regulation: SPP1 --+> AKT1		10.1128/JVI.02116-17
positive Regulation: SPP1 --+> AKT1		10.1128/JVI.02116-17
positive Regulation: SPP1 --+> AK	30463876	10.1084/jem.20181211
positive Regulation: SPP1 --+> AK	30483813	10.3892/or.2018.6882
positive Regulation: SPP1 --+> AK	30535434	10.3892/ijo.2018.4656
positive Regulation: SPP1 --+> AKT1		10.1536/ihj.18-017
Regulation: CLDN1 ---> AKT1	23095753	10.1074/jbc.M112.414789
Regulation: CLDN1 ---> AKT1	23095753	10.1074/jbc.M112.414789
Regulation: CLDN1 ---> AKT1	23095753	10.1074/jbc.M112.414789
Regulation: CLDN1 ---> AKT1	23449973	10.1074/jbc.M112.428920
Regulation: CLDN1 ---> AKT1	23449973	10.1074/jbc.M112.428920
Regulation: CLDN1 ---> AKT1	30518037	10.3390/ijms19123862
Regulation: CLDN1 ---> AKT1	30971761	10.1038/s41388-019-0795-5
Regulation: CLDN1 ---> AKT1	23095753	10.1074/jbc.M112.414789
Regulation: CLDN1 ---> AKT1	23095753	10.1074/jbc.M112.414789
Regulation: CLDN1 ---> AKT1	23095753	10.1074/jbc.M112.414789
Regulation: CLDN1 ---> AKT1	23449973	10.1074/jbc.M112.428920
Regulation: CLDN1 ---> AKT1	23095753	10.1074/jbc.M112.41478910.1074/jbc.M112.41478
Regulation: CLDN1 ---> AKT1		10.3390/ijms19123862
Regulation: CLDN1 ---> AKT1	26954146	10.5713/ajas.15.041510.5713/ajas.15.0415
Regulation: CLDN1 ---> AKT1		10.1038/s41388-019-0795-5
Regulation: CLDN1 ---> AKT1	35301287	10.1038/s41420-022-00918-5
Regulation: CLDN1 ---> AKT1	34276052	10.1038/s41417-021-00328-2
Regulation: CLDN1 ---> AKT1	34276052	10.1038/s41417-021-00328-2
Regulation: CLDN1 ---> AKT1	34276052	10.1038/s41417-021-00328-2
PromoterBinding: PPARG ---> PTEN		10.1016/S0006-2952(03)00488-X
PromoterBinding: PPARG ---> PTEN	22902055	10.1016/j.biopha.2012.04.004
PromoterBinding: PPARG ---> PTEN	18032782	

PromoterBinding: PPARG ---> PTEN	21990315	10.1210/en.2011-1207
PromoterBinding: PPARG ---> PTEN	841	10.1016/j.tjog.2016.05.006
PromoterBinding: PPARG ---> PTEN	841	10.1016/j.tjog.2016.05.006
PromoterBinding: PPARG ---> PTEN	21489309	10.1186/1465-9921-12-47
PromoterBinding: PPARG ---> PTEN	25889730	10.1186/s12943-015-0347-8
PromoterBinding: PPARG ---> PTEN	26674985	10.1038/srep1831910.1038/srep18319
PromoterBinding: PPARG ---> PTEN		
PromoterBinding: PPARG ---> PTEN	26861561	10.1007/s13277-016-4919-z
Expression: AKT1 ---> CLDN1	29524521	10.1016/j.bbamcr.2018.03.001
Expression: AKT1 ---> CLDN1	29524521	10.1016/j.bbamcr.2018.03.001
Expression: AKT1 ---> CLDN1	29524521	10.1016/j.bbamcr.2018.03.001
Expression: AKT1 ---> CLDN1	28993008	10.1016/j.ejcb.2017.09.004
Expression: AKT1 ---> CLDN1	28498331	10.3390/ijms18051051
Expression: AKT1 ---> CLDN1	31262888	10.21873/anticanres.13510
Expression: AKT1 ---> CLDN1	31467400	10.1038/s41598-019-49021-310.1038/s41598-019-
Expression: AKT1 ---> CLDN1	32553076	10.3967/bes2020.04410.3967/bes2020.044
Expression: AKT1 ---> CLDN1	35568758	10.1007/s00277-022-04867-y
Expression: NOTCH1 ---> MCL1	28319728	10.1016/j.mib.2017.03.001
Expression: NOTCH1 ---> MCL1	22249262	10.1038/onc.2011.615
Expression: NOTCH1 ---> MCL1	22249262	10.1038/onc.2011.615
Expression: NOTCH1 ---> MCL1	22249262	10.1038/onc.2011.615
Expression: NOTCH1 ---> MCL1	22249262	10.1038/onc.2011.615
Expression: NOTCH1 ---> MCL1	22249262	10.1038/onc.2011.615
Expression: NOTCH1 ---> MCL1	23872918	10.1038/cmi.2013.22
Expression: NOTCH1 ---> MCL1	23872918	10.1038/cmi.2013.22
Expression: NOTCH1 ---> MCL1	23872918	10.1038/cmi.2013.22
Expression: NOTCH1 ---> MCL1	25231743	10.1038/leu.2014.276
Expression: NOTCH1 ---> MCL1	23872918	10.1038/cmi.2013.2210.1038/cmi.2013.22
Expression: NOTCH1 ---> MCL1	23872918	10.1038/cmi.2013.2210.1038/cmi.2013.22
positive Expression: LRP8 --+> CI	33054597	10.1177/0300891620952872
positive Expression: LRP8 --+> CI	33054597	10.1177/0300891620952872
positive Expression: LRP8 --+> CI	33054597	10.1177/0300891620952872
positive Expression: LRP8 --+> CI	33054597	10.1177/0300891620952872
positive Expression: LRP8 --+> CI	36012187	10.3390/ijms23168921
Expression: PTEN ---> TP53	33667713	10.1016/j.bbrc.2021.02.093
Expression: PTEN ---> TP53	16728701	10.1182/blood-2006-02-001354
Expression: PTEN ---> TP53	21873427	10.1074/jbc.M110.217620
Expression: PTEN ---> TP53	17684018	10.1074/jbc.M703796200
Expression: PTEN ---> TP53	17684018	10.1074/jbc.M703796200
Expression: PTEN ---> TP53	12860987	10.1074/jbc.M305135200
Expression: PTEN ---> TP53	30763577	10.1016/j.lfs.2019.02.021
Expression: PTEN ---> TP53	26801100	10.1016/j.dci.2016.01.004
Expression: PTEN ---> TP53	30036566	10.1016/j.bbamcr.2018.07.016
Expression: PTEN ---> TP53	32172081	10.1016/j.ejmech.2020.112177
Expression: PTEN ---> TP53	32898647	10.1016/j.biochi.2020.08.021
Expression: PTEN ---> TP53	31978509	10.1016/j.gene.2020.144353
Expression: PTEN ---> TP53	32976933	10.1016/j.reprotox.2020.09.006
Expression: PTEN ---> TP53	29414645	10.1016/j.intimp.2018.01.029
Expression: PTEN ---> TP53	26579470	10.1016/j.apsb.2015.07.001
Expression: PTEN ---> TP53	29450148	10.1016/j.biopen.2017.03.005
Expression: PTEN ---> TP53	20413683	10.2353/ajpath.2010.090571
Expression: PTEN ---> TP53	15865934	10.1016/j.bbrc.2005.03.183
Expression: PTEN ---> TP53	19765550	10.1016/j.bcp.2009.09.013
Expression: PTEN ---> TP53	20858413	10.1016/j.bpj.2010.07.042

Expression: PTEN ---> TP53	21040738	10.1016/j.mrfmmm.2010.10.007
Expression: PTEN ---> TP53	20018398	10.1016/j.lungcan.2009.11.012
Expression: PTEN ---> TP53	22266095	10.1016/j.canlet.2012.01.003
Expression: PTEN ---> TP53	22266095	10.1016/j.canlet.2012.01.003
Expression: PTEN ---> TP53	18166163	10.1016/j.bbcan.2007.12.001
Expression: PTEN ---> TP53	22033326	10.1016/j.ejca.2011.09.014
Expression: PTEN ---> TP53	18061438	10.1016/j.jsbmb.2007.09.020
Expression: PTEN ---> TP53	14697668	10.1016/j.mcn.2003.08.010
Expression: PTEN ---> TP53	14697668	10.1016/j.mcn.2003.08.010
Expression: PTEN ---> TP53	16916644	10.1016/j.molcel.2006.06.028
Expression: PTEN ---> TP53	16916644	10.1016/j.molcel.2006.06.028
Expression: PTEN ---> TP53	16916644	10.1016/j.molcel.2006.06.028
Expression: PTEN ---> TP53	16916644	10.1016/j.molcel.2006.06.028
Expression: PTEN ---> TP53	21474066	10.1016/j.molcel.2011.02.020
Expression: PTEN ---> TP53	12620407	10.1016/S1535-6108(03)00021-7
Expression: PTEN ---> TP53	12620407	10.1016/S1535-6108(03)00021-7
Expression: PTEN ---> TP53	12620407	10.1016/S1535-6108(03)00021-7
Expression: PTEN ---> TP53	12620407	10.1016/S1535-6108(03)00021-7
Expression: PTEN ---> TP53	12620407	10.1016/S1535-6108(03)00021-7
Expression: PTEN ---> TP53	12620407	10.1016/S1535-6108(03)00021-7
Expression: PTEN ---> TP53	12620407	10.1016/S1535-6108(03)00021-7
Expression: PTEN ---> TP53	12620407	10.1016/S1535-6108(03)00021-7
Expression: PTEN ---> TP53	12620407	10.1016/S1535-6108(03)00021-7
Expression: PTEN ---> TP53	12620407	10.1016/S1535-6108(03)00021-7
Expression: PTEN ---> TP53	12620402	10.1016/S1535-6108(03)00022-9
Expression: PTEN ---> TP53	16697957	10.1016/j.ccr.2006.03.031
Expression: PTEN ---> TP53	21040901	10.1016/j.stem.2010.09.015
Expression: PTEN ---> TP53	21040901	10.1016/j.stem.2010.09.015
Expression: PTEN ---> TP53	23123091	10.1016/j.febslet.2012.10.036
Expression: PTEN ---> TP53	23123091	10.1016/j.febslet.2012.10.036
Expression: PTEN ---> TP53	24607404	10.1016/j.stem.2014.02.008
Expression: PTEN ---> TP53	24739178	10.1016/j.bpj.2014.02.032
Expression: PTEN ---> TP53	24818748	10.1016/j.tibs.2014.04.004
Expression: PTEN ---> TP53	26655093	10.1016/j.arr.2015.12.001
Expression: PTEN ---> TP53	18174288	
Expression: PTEN ---> TP53	17684018	
Expression: PTEN ---> TP53	17684018	
Expression: PTEN ---> TP53	15090541	
Expression: PTEN ---> TP53	15090541	
Expression: PTEN ---> TP53	12860987	
Expression: PTEN ---> TP53	19099539	
Expression: PTEN ---> TP53	19234438	
Expression: PTEN ---> TP53	17962806	
Expression: PTEN ---> TP53	18391023	
Expression: PTEN ---> TP53	18391023	
Expression: PTEN ---> TP53	18332125	
Expression: PTEN ---> TP53	18332125	
Expression: PTEN ---> TP53	18332125	
Expression: PTEN ---> TP53	16055704	
Expression: PTEN ---> TP53	27033080	10.1007/978-1-4939-3299-3_15
Expression: PTEN ---> TP53	28677502	10.2174/1381612823666170704124611
Expression: PTEN ---> TP53	29103158	10.1007/s00432-017-2537-9
Expression: PTEN ---> TP53		10.3390/ijms22189838
Expression: PTEN ---> TP53	33667713	10.1016/j.bbrc.2021.02.09310.1016/j.bbrc.2021

Expression: PTEN ---> TP53	34504554	10.3892/etm.2021.1053410.3892/etm.2021.10534
Expression: PTEN ---> TP53	18363844	10.1111/j.1582-4934.2008.00311.x10.1111/j.158
Expression: PTEN ---> TP53	20926450	10.1093/hmg/ddq43410.1093/hmg/ddq434
Expression: PTEN ---> TP53		10.1016/j.bpj.2010.07.042
Expression: PTEN ---> TP53		10.1016/j.ejca.2011.09.014
Expression: PTEN ---> TP53		10.1016/j.mcn.2003.08.010
Expression: PTEN ---> TP53		10.1016/j.mcn.2003.08.010
Expression: PTEN ---> TP53		10.1016/j.apsb.2015.07.001
Expression: PTEN ---> TP53	29103158	10.1007/s00432-017-2537-9
Expression: PTEN ---> TP53	29209894	10.1007/s10863-017-9737-5
Expression: PTEN ---> TP53	29488607	10.3892/mmr.2018.8640
Expression: PTEN ---> TP53	30095997	10.1152/ajpheart.00121.2018
Expression: PTEN ---> TP53	30396902	10.1136/gutjnl-2018-316822
Expression: PTEN ---> TP53		10.3390/molecules24132411
Expression: PTEN ---> TP53		10.1507/endocrj.EJ18-0538
Expression: PTEN ---> TP53		10.3892/or.2020.7457
Expression: PTEN ---> TP53		10.3892/or.2020.7457
Expression: PTEN ---> TP53	23114644	10.4161/cbt.22627
Expression: PTEN ---> TP53	23114644	10.4161/cbt.22627
Expression: PTEN ---> TP53	21164280	10.4161/cam.5.2.14375
Expression: PTEN ---> TP53	21873427	10.1074/jbc.M110.217620
Expression: PTEN ---> TP53	22394167	10.2174/138161212799859701
Expression: PTEN ---> TP53	22413754	10.1042/BJ20120098
Expression: PTEN ---> TP53	22592924	10.1165/rcmb.2011-0390OC
Expression: PTEN ---> TP53	23908595	10.1593/neo.13376
Expression: PTEN ---> TP53	21436840	10.1038/leu.2011.46
Expression: PTEN ---> TP53	21436840	10.1038/leu.2011.46
Expression: PTEN ---> TP53	32246301	10.1007/s10522-020-09876-w
Expression: PTEN ---> TP53	23727861	10.1038/ng.2654
Expression: PTEN ---> TP53	21642958	10.1038/emboj.2011.178
Expression: PTEN ---> TP53	24127214	10.1083/jcb.201303110
Expression: PTEN ---> TP53	21695255	10.1371/journal.pone.0020715
Expression: PTEN ---> TP53	22164291	10.1371/journal.pone.0028433
Expression: PTEN ---> TP53	24063548	10.1089/ars.2013.5498
Expression: PTEN ---> TP53	24063548	10.1089/ars.2013.5498
Expression: PTEN ---> TP53	24081673	10.1007/s13277-013-1210-4
Expression: PTEN ---> TP53	24905788	10.1146/annurev-biochem-082411-113907
Expression: PTEN ---> TP53	24959190	10.1155/2014/724658
Expression: PTEN ---> TP53	24959190	10.1155/2014/724658
Expression: PTEN ---> TP53	20733006	10.1128/MCB.00004-10
Expression: PTEN ---> TP53		10.1681/ASN.2014121161
Expression: PTEN ---> TP53	26647729	10.3892/ijo.2015.3268
Expression: PTEN ---> TP53	26869029	10.1038/ncomms10689
Expression: PTEN ---> TP53	26477313	10.1038/onc.2015.253
Expression: PTEN ---> TP53	12620407	
Expression: PTEN ---> TP53	14575575	
Expression: PTEN ---> TP53	15619344	
Expression: PTEN ---> TP53	16424003	
Expression: PTEN ---> TP53	30971627	10.1507/endocrj.EJ18-053810.1507/endocrj.EJ18
Expression: PTEN ---> TP53	33256086	10.3390/ijms21238982
Expression: PTEN ---> TP53	34205272	10.3390/molecules26123737
Expression: PTEN ---> TP53	36180910	10.1186/s12929-022-00860-9
Expression: PTEN ---> TP53	34951363	10.2174/1566524022666211222161637
PromoterBinding: TP53 ---> PTEN	28602818	10.1016/j.jmb.2017.06.004

PromoterBinding: TP53 ---> PTEN 19173293 10.1002/ijc.24221
PromoterBinding: TP53 ---> PTEN 29803738 10.1016/j.virol.2018.05.011
PromoterBinding: TP53 ---> PTEN 29179029 10.1016/j.leukres.2017.08.007
PromoterBinding: TP53 ---> PTEN 32035215 10.1016/j.fct.2020.111179
PromoterBinding: TP53 ---> PTEN 23406730 10.1093/annonc/mdt013
PromoterBinding: TP53 ---> PTEN 30738865 10.1016/j.semcancer.2019.02.001
PromoterBinding: TP53 ---> PTEN 33675995 10.1016/j.biocel.2021.105965
PromoterBinding: TP53 ---> PTEN 22510407 10.1016/j.bbrc.2012.04.015
PromoterBinding: TP53 ---> PTEN 10.1016/S0006-291X(02)02983-2
PromoterBinding: TP53 ---> PTEN 22290228 10.1016/j.bbrc.2012.01.065
PromoterBinding: TP53 ---> PTEN 17258789 10.1016/j.humpath.2006.10.007
PromoterBinding: TP53 ---> PTEN 16545436 10.1016/j.ygyno.2005.12.033
PromoterBinding: TP53 ---> PTEN 21664656 10.1016/j.ygyno.2011.05.017
PromoterBinding: TP53 ---> PTEN 16051030 10.1016/j.canlet.2004.12.006
PromoterBinding: TP53 ---> PTEN 16996682 10.1016/j.canlet.2006.08.007
PromoterBinding: TP53 ---> PTEN 12217521 10.1016/S0968-0004(02)02166-7
PromoterBinding: TP53 ---> PTEN 12217521 10.1016/S0968-0004(02)02166-7
PromoterBinding: TP53 ---> PTEN 11545734 10.1016/S1097-2765(01)00323-9
PromoterBinding: TP53 ---> PTEN 11545734 10.1016/S1097-2765(01)00323-9
PromoterBinding: TP53 ---> PTEN 11545734 10.1016/S1097-2765(01)00323-9
PromoterBinding: TP53 ---> PTEN 11545734 10.1016/S1097-2765(01)00323-9
PromoterBinding: TP53 ---> PTEN 11545734 10.1016/S1097-2765(01)00323-9
PromoterBinding: TP53 ---> PTEN 18950730 10.1016/j.biocel.2008.09.022
PromoterBinding: TP53 ---> PTEN 18718806 10.1016/j.drup.2008.07.001
PromoterBinding: TP53 ---> PTEN 12620407 10.1016/S1535-6108(03)00021-7
PromoterBinding: TP53 ---> PTEN 21146147 10.1016/j.trsl.2010.09.004
PromoterBinding: TP53 ---> PTEN 23122111 10.1016/j.foodchem.2012.07.112
PromoterBinding: TP53 ---> PTEN 25744176 10.1016/j.drudis.2015.02.011
PromoterBinding: TP53 ---> PTEN 17998413
PromoterBinding: TP53 ---> PTEN 18032782
PromoterBinding: TP53 ---> PTEN 15193142
PromoterBinding: TP53 ---> PTEN 16495927
PromoterBinding: TP53 ---> PTEN 18172008
PromoterBinding: TP53 ---> PTEN 18172008
PromoterBinding: TP53 ---> PTEN 28301379 10.1097/CAD.0000000000000476
PromoterBinding: TP53 ---> PTEN 28301379 10.1097/CAD.0000000000000476
PromoterBinding: TP53 ---> PTEN 28301379 10.1097/CAD.0000000000000476
PromoterBinding: TP53 ---> PTEN 30217221 10.1186/s13046-018-0898-9
PromoterBinding: TP53 ---> PTEN 30486451 10.3390/ijms19123773
PromoterBinding: TP53 ---> PTEN 31776983 10.1007/978-981-15-0602-4_6
PromoterBinding: TP53 ---> PTEN 33194023
PromoterBinding: TP53 ---> PTEN 32974790 10.1007/s10549-020-05937-3
PromoterBinding: TP53 ---> PTEN 22489661 10.1089/cbr.2011.1154
PromoterBinding: TP53 ---> PTEN 20862607 10.1007/s10528-010-9379-3
PromoterBinding: TP53 ---> PTEN 21990315 10.1210/en.2011-1207
PromoterBinding: TP53 ---> PTEN 22218495 10.3892/ijmm.2012.879
PromoterBinding: TP53 ---> PTEN 20736773 10.1097/PGP.0b013e3181de3068
PromoterBinding: TP53 ---> PTEN 21297666 10.1038/onc.2011.6
PromoterBinding: TP53 ---> PTEN 24083690 10.7314/APJCP.2013.14.8.4485
PromoterBinding: TP53 ---> PTEN 24866016 10.1371/journal.pone.0098113
PromoterBinding: TP53 ---> PTEN 24905788 10.1146/annurev-biochem-082411-113907
PromoterBinding: TP53 ---> PTEN 19079342 10.1038/onc.2008.45110.1038/onc.2008.451
PromoterBinding: TP53 ---> PTEN 20554748 10.1093/carcin/bgq12310.1093/carcin/bgq123
PromoterBinding: TP53 ---> PTEN 16778187 10.1158/0008-5472.CAN-06-0772

PromoterBinding: TP53 ---> PTEN 16061640 10.1158/0008-5472.CAN-04-4184
PromoterBinding: TP53 ---> PTEN 12917634 10.1038/sj.onc.1206783
PromoterBinding: TP53 ---> PTEN 10865036 10.1007/s002680010164
PromoterBinding: TP53 ---> PTEN 15843377 10.1074/jbc.M503026200
PromoterBinding: TP53 ---> PTEN 19079342 10.1038/onc.2008.451
PromoterBinding: TP53 ---> PTEN 15016317 10.1111/j.1349-7006.2004.tb02203.x
PromoterBinding: TP53 ---> PTEN 20554748 10.1093/carcin/bgq123
PromoterBinding: TP53 ---> PTEN 11729185 10.1074/jbc.M108302200
PromoterBinding: TP53 ---> PTEN 10.1016/j.bbrc.2012.04.015
PromoterBinding: TP53 ---> PTEN 19234438
PromoterBinding: TP53 ---> PTEN 36430560 10.3390/ijms232214083
negative Expression: NGF ---| GA 10100865 10.1016/S0014-5793(99)00234-3
negative Expression: NGF ---| GA 10100865 10.1016/S0014-5793(99)00234-3
negative PromoterBinding: TP53 ---| PPARG 10.1016/j.brainresbull.2011.07.021
negative PromoterBinding: TP53 -- 21854837 10.1016/j.brainresbull.2011.07.021
negative PromoterBinding: TP53 -- 21510869 10.1186/1471-2407-11-149
negative PromoterBinding: TP53 -- 21445621 10.1007/s00394-011-0187-2
negative PromoterBinding: TP53 -- 30181511 10.3390/ijms19092622
negative PromoterBinding: TP53 -- 30143607 10.1038/s41467-018-05711-6
negative PromoterBinding: TP53 -- 29593329 10.1038/s41556-018-0072-9
negative PromoterBinding: TP53 -- 29593329 10.1038/s41556-018-0072-9
negative PromoterBinding: TP53 -- 29593329 10.1038/s41556-018-0072-9
negative PromoterBinding: TP53 -- 29593329 10.1038/s41556-018-0072-9
negative PromoterBinding: TP53 -- 24675459 10.1038/cddis.2014.87
negative PromoterBinding: TP53 -- 24312426 10.1371/journal.pone.0082494
negative PromoterBinding: TP53 -- 29735908 10.3390/ijms19051387
negative PromoterBinding: TP53 ---| PPARG 10.2741/4475
negative PromoterBinding: TP53 -- 28155209 10.1007/s11999-017-5259-z
negative PromoterBinding: TP53 -- 21421225 10.1016/j.juro.2010.12.101
negative PromoterBinding: TP53 -- 19168033 10.1016/j.bbrc.2009.01.060
negative PromoterBinding: TP53 -- 30181511 10.3390/ijms19092622
positive Regulation: RAC1 --+> M 32898553 10.1016/j.yexcr.2020.112265
positive Regulation: RAC1 --+> M 10681583 10.1074/jbc.275.8.5911
positive Regulation: RAC1 --+> M 17145753 10.1074/jbc.M607989200
positive Regulation: RAC1 --+> M 17145753 10.1074/jbc.M607989200
positive Regulation: RAC1 --+> M 11756405 10.1074/jbc.M104766200
positive Regulation: RAC1 --+> M 11279039 10.1074/jbc.M100254200
positive Regulation: RAC1 --+> M 20650890 10.1074/jbc.M110.105700
positive Regulation: RAC1 --+> M 17908694 10.1074/jbc.M702175200
positive Regulation: RAC1 --+> M 20529870 10.1074/jbc.M109.099655
positive Regulation: RAC1 --+> M 20529870 10.1074/jbc.M109.099655
positive Regulation: RAC1 --+> M 20529870 10.1074/jbc.M109.099655
positive Regulation: RAC1 --+> M 17008315 10.1074/jbc.M605288200
positive Regulation: RAC1 --+> M 14557270 10.1074/jbc.M307330200
positive Regulation: RAC1 --+> M 17088251 10.1074/jbc.M605817200
positive Regulation: RAC1 --+> M 10945974 10.1074/jbc.M002138200
positive Regulation: RAC1 --+> MAPK1 10.1016/j.jid.2017.07.075
positive Regulation: RAC1 --+> M 29329780 10.1016/j.cellsig.2018.01.004
positive Regulation: RAC1 --+> M 29329780 10.1016/j.cellsig.2018.01.004
positive Regulation: RAC1 --+> M 29329780 10.1016/j.cellsig.2018.01.004
positive Regulation: RAC1 --+> M 29329780 10.1016/j.cellsig.2018.01.004
positive Regulation: RAC1 --+> M 31063759 10.1016/j.devcel.2019.04.007
positive Regulation: RAC1 --+> M 31063759 10.1016/j.devcel.2019.04.007
positive Regulation: RAC1 --+> M 32497487 10.1016/j.devcel.2020.05.011

positive Regulation: RAC1 --+> M/31257073 10.1016/j.ccell.2019.05.015
positive Regulation: RAC1 --+> M/27070502 10.1016/j.intimp.2016.03.025
positive Regulation: RAC1 --+> M/27930970 10.1016/j.intimp.2016.10.001
positive Regulation: RAC1 --+> M/26923603 10.1016/j.celrep.2016.02.016
positive Regulation: RAC1 --+> M/26923603 10.1016/j.celrep.2016.02.016
positive Regulation: RAC1 --+> M/26923603 10.1016/j.celrep.2016.02.016
positive Regulation: RAC1 --+> M/26923603 10.1016/j.celrep.2016.02.016
positive Regulation: RAC1 --+> M/17568564 10.1016/j.bbrc.2007.05.214
positive Regulation: RAC1 --+> M/17568564 10.1016/j.bbrc.2007.05.214
positive Regulation: RAC1 --+> M/15093747 10.1016/j.yexcr.2004.02.002
positive Regulation: RAC1 --+> M/19852956 10.1016/j.yexcr.2009.10.017
positive Regulation: RAC1 --+> M/15013523 10.1016/j.canlet.2003.08.035
positive Regulation: RAC1 --+> M/15893875 10.1016/j.canlet.2005.02.031
positive Regulation: RAC1 --+> M/9024622 10.1016/S0960-9822(06)00052-2
positive Regulation: RAC1 --+> M/16595175 10.1016/j.tcb.2006.03.002
positive Regulation: RAC1 --+> M/24140422 10.1016/j.molcel.2013.09.015
positive Regulation: RAC1 --+> M/24140422 10.1016/j.molcel.2013.09.015
positive Regulation: RAC1 --+> M/25284480 10.1016/j.ccell.2014.08.017
positive Regulation: RAC1 --+> M/25284480 10.1016/j.ccell.2014.08.017
positive Regulation: RAC1 --+> M/12843210
positive Regulation: RAC1 --+> M/12843210
positive Regulation: RAC1 --+> M/11075808
positive Regulation: RAC1 --+> M/18793427
positive Regulation: RAC1 --+> M/18003981
positive Regulation: RAC1 --+> M/17908694
positive Regulation: RAC1 --+> M/17145753
positive Regulation: RAC1 --+> M/17145753
positive Regulation: RAC1 --+> M/17088251
positive Regulation: RAC1 --+> M/17088251
positive Regulation: RAC1 --+> M/17008315
positive Regulation: RAC1 --+> M/16014609
positive Regulation: RAC1 --+> M/15339911
positive Regulation: RAC1 --+> M/15155836
positive Regulation: RAC1 --+> M/15155836
positive Regulation: RAC1 --+> M/15155836
positive Regulation: RAC1 --+> M/14557270
positive Regulation: RAC1 --+> M/12070151
positive Regulation: RAC1 --+> M/12070151
positive Regulation: RAC1 --+> M/11756405
positive Regulation: RAC1 --+> M/11369774
positive Regulation: RAC1 --+> M/11369774
positive Regulation: RAC1 --+> M/11369774
positive Regulation: RAC1 --+> M/11369774
positive Regulation: RAC1 --+> M/11369774
positive Regulation: RAC1 --+> M/11369774
positive Regulation: RAC1 --+> M/11279039
positive Regulation: RAC1 --+> M/10945984
positive Regulation: RAC1 --+> M/10945974
positive Regulation: RAC1 --+> M/10681583
positive Regulation: RAC1 --+> M/10681583
positive Regulation: RAC1 --+> M/10419534
positive Regulation: RAC1 --+> M/10419534
positive Regulation: RAC1 --+> M/17389689
positive Regulation: RAC1 --+> M/16574914

positive Regulation: RAC1 --+> MA 15027896
positive Regulation: RAC1 --+> MA 15027896
positive Regulation: RAC1 --+> MA 15027896
positive Regulation: RAC1 --+> MA 15027896
positive Regulation: RAC1 --+> MA 12902401
positive Regulation: RAC1 --+> MA 12902401
positive Regulation: RAC1 --+> MA 34294609 10.14348/molcells.2021.0031
positive Regulation: RAC1 --+> MA 20650890 10.1074/jbc.M110.10570010.1074/jbc.M110.10570
positive Regulation: RAC1 --+> MA 22494620 10.1186/bcr3164
positive Regulation: RAC1 --+> MA 22519702 10.1042/BJ2012005310.1042/BJ20120053
positive Regulation: RAC1 --+> MA 22617030 10.1016/j.cellsig.2012.05.01110.1016/j.cellsi
positive Regulation: RAC1 --+> MA 24371145 10.1074/jbc.M113.51851410.1074/jbc.M113.51851
positive Regulation: RAC1 --+> MA 31817229 10.3390/cells812156910.3390/cells8121569
positive Regulation: RAC1 --+> MA 15027896
positive Regulation: RAC1 --+> MA 12902401
positive Regulation: RAC1 --+> MA 10827130
positive Regulation: RAC1 --+> MA 18542102
positive Regulation: RAC1 --+> MA 15482467
positive Regulation: RAC1 --+> MA 19179620
positive Regulation: RAC1 --+> MA 14729984
positive Regulation: RAC1 --+> MAPK1
positive Regulation: RAC1 --+> MA 26561781 10.1038/cddis.2015.329
positive Regulation: RAC1 --+> MAPK1 10.1042/BST20160269
positive Regulation: RAC1 --+> MAPK1 10.1042/BST20160269
positive Regulation: RAC1 --+> MA 29620286 10.3892/or.2018.6345
positive Regulation: RAC1 --+> MA 29954442 10.1186/s13046-018-0787-2
positive Regulation: RAC1 --+> MA 30545369 10.1186/s12964-018-0312-4
positive Regulation: RAC1 --+> MA 31152053 10.1242/jcs.230706
positive Regulation: RAC1 --+> MAPK1 10.1074/jbc.RA119.008685
positive Regulation: RAC1 --+> MAPK1 10.1073/pnas.1914153117
positive Regulation: RAC1 --+> MA 32193458 10.1038/s41467-020-15308-7
positive Regulation: RAC1 --+> MA 20529870 10.1074/jbc.M109.099655
positive Regulation: RAC1 --+> MA 20529870 10.1074/jbc.M109.099655
positive Regulation: RAC1 --+> MA 20529870 10.1074/jbc.M109.099655
positive Regulation: RAC1 --+> MA 20529870 10.1074/jbc.M109.099655
positive Regulation: RAC1 --+> MA 20529870 10.1074/jbc.M109.099655
positive Regulation: RAC1 --+> MA 20529870 10.1074/jbc.M109.099655
positive Regulation: RAC1 --+> MA 20650890 10.1074/jbc.M110.105700
positive Regulation: RAC1 --+> MA 20650890 10.1074/jbc.M110.105700
positive Regulation: RAC1 --+> MA 21482718 10.1083/jcb.201009100
positive Regulation: RAC1 --+> MA 21478669 10.4161/cc.10.10.15612
positive Regulation: RAC1 --+> MA 21170593 10.1007/s11033-010-0630-0
positive Regulation: RAC1 --+> MA 22519702 10.1042/BJ20120053
positive Regulation: RAC1 --+> MA 32471868 10.1074/jbc.RA119.01098110.1074/jbc.RA119.010
positive Regulation: RAC1 --+> MA 27551466 10.1038/cddiscovery.2015.3510.1038/cddiscover
positive Regulation: RAC1 --+> MA 12468535
positive Regulation: RAC1 --+> MA 22519702 10.1042/BJ20120053
positive Regulation: RAC1 --+> MA 22519702 10.1042/BJ20120053
positive Regulation: RAC1 --+> MA 11369774
positive Regulation: RAC1 --+> MA 15027896
positive Regulation: RAC1 --+> MA 14993270
positive Regulation: RAC1 --+> MA 23023511 10.1161/CIRCRESAHA.111.255216
positive Regulation: RAC1 --+> MA 23337888 10.1038/jid.2013.23
positive Regulation: RAC1 --+> MA 23781022 10.1242/jcs.126714

positive Regulation: RAC1 --+> M/ 23781022 10.1242/jcs.126714
positive Regulation: RAC1 --+> M/ 22701712 10.1371/journal.pone.0038777
positive Regulation: RAC1 --+> M/ 22701712 10.1371/journal.pone.0038777
positive Regulation: RAC1 --+> M/ 22970259 10.1371/journal.pone.0044590
positive Regulation: RAC1 --+> M/ 23734197 10.1371/journal.pone.0064350
positive Regulation: RAC1 --+> M/ 24327733 10.1073/pnas.1314124110
positive Regulation: RAC1 --+> M/ 24152593 10.4161/cam.26596
positive Regulation: RAC1 --+> M/ 22668020 10.4149/neo_2012_070
positive Regulation: RAC1 --+> M/ 25121739 10.1371/journal.pone.0104746
positive Regulation: RAC1 --+> M/ 25121739 10.1371/journal.pone.0104746
positive Regulation: RAC1 --+> M/ 27497523 10.1016/j.bbadis.2016.07.019
positive Regulation: RAC1 --+> M/ 17568564 10.1016/j.bbrc.2007.05.214
positive Regulation: RAC1 --+> M/ 19450605 10.1016/j.yjmcc.2009.05.002
positive Regulation: RAC1 --+> M/ 19450605 10.1016/j.yjmcc.2009.05.002
positive Regulation: RAC1 --+> M/ 19450605 10.1016/j.yjmcc.2009.05.002
positive Regulation: RAC1 --+> M/ 22504301 10.1016/j.bbamcr.2012.03.015
positive Regulation: RAC1 --+> M/ 18086571 10.1016/j.bbamcr.2007.10.024
positive Regulation: RAC1 --+> M/ 18353489 10.1016/j.ejcb.2008.02.003
positive Regulation: RAC1 --+> M/ 21726627 10.1016/j.cellsig.2011.06.014
positive Regulation: RAC1 --+> M/ 22061968 10.1016/j.mcn.2011.10.008
positive Regulation: RAC1 --+> M/ 22061968 10.1016/j.mcn.2011.10.008
positive Regulation: RAC1 --+> M/ 22061968 10.1016/j.mcn.2011.10.008
positive Regulation: RAC1 --+> M/ 22061968 10.1016/j.mcn.2011.10.008
positive Regulation: RAC1 --+> M/ 15905109 10.1016/j.semcdb.2005.03.010
positive Regulation: RAC1 --+> M/ 25725286 10.1016/j.cellsig.2015.02.020
positive Regulation: RAC1 --+> M/ 26707876 10.1016/j.bbrc.2015.12.056
positive Regulation: RAC1 --+> M/ 18385518
positive Regulation: RAC1 --+> M/ 16698790
positive Regulation: RAC1 --+> M/ 17941827
positive Regulation: RAC1 --+> M/ 11031201
positive Regulation: RAC1 --+> M/ 12417261 10.1016/S0006-2952(02)01388-6
positive Regulation: RAC1 --+> M/ 19852956 10.1016/j.yexcr.2009.10.017
positive Regulation: RAC1 --+> M/ 23639442 10.1016/j.devcel.2013.03.019
positive Regulation: RAC1 --+> M/ 18508927
positive Regulation: RAC1 --+> M/ 18385518
positive Regulation: RAC1 --+> M/ 17088251
positive Regulation: RAC1 --+> M/ 11369774
positive Regulation: RAC1 --+> M/ 15027896
positive Regulation: RAC1 --+> M/ 16798728
positive Regulation: RAC1 --+> M/ 16798728
positive Regulation: RAC1 --+> M/ 16798728
positive Regulation: RAC1 --+> M/ 22494620 10.1186/bcr3164
positive Regulation: RAC1 --+> M/ 22494620 10.1186/bcr3164
positive Regulation: RAC1 --+> M/ 23093703 10.1210/en.2012-1626
positive Regulation: RAC1 --+> M/ 20529870 10.1074/jbc.M109.099655
positive Regulation: RAC1 --+> M/ 23446149 10.1189/jlb.0912473
positive Regulation: RAC1 --+> M/ 23657011 10.1073/pnas.1215902110
positive Regulation: RAC1 --+> M/ 23657011 10.1073/pnas.1215902110
positive Regulation: RAC1 --+> M/ 23657011 10.1073/pnas.1215902110
positive Regulation: RAC1 --+> M/ 23657011 10.1073/pnas.1215902110
positive Regulation: RAC1 --+> M/ 23657011 10.1073/pnas.1215902110
positive Regulation: RAC1 --+> M/ 24086766 10.1371/journal.pone.0077027
positive Regulation: RAC1 --+> M/ 24152593 10.4161/cam.26596
positive Regulation: RAC1 --+> M/ 24371145 10.1074/jbc.M113.518514

positive Regulation: RAC1 --+> M/ 24757372 10.4196/kjpp.2014.18.2.109
positive Regulation: RAC1 --+> M/ 24571616 10.1186/1742-4690-11-20
positive Regulation: RAC1 --+> M/ 25091805 10.1186/s12929-014-0067-1
positive Regulation: RAC1 --+> M/ 25714841 10.1371/journal.pone.0115325
positive Regulation: RAC1 --+> M/ 24760984 10.1152/ajpcell.00380.2013
positive Regulation: RAC1 --+> M/ 23817184 10.1159/000350108
positive Regulation: RAC1 --+> M/ 23817184 10.1159/000350108
positive Regulation: RAC1 --+> M/ 23817184 10.1159/000350108
positive Regulation: RAC1 --+> M/ 23817184 10.1159/000350108
positive Regulation: RAC1 --+> M/ 24043306 10.1128/MCB.00822-13
positive Regulation: RAC1 --+> M/ 11158304
positive Regulation: RAC1 --+> M/ 12485852
positive Regulation: RAC1 --+> M/ 15009099
positive Regulation: RAC1 --+> M/ 15027896
positive Regulation: RAC1 --+> M/ 17088251
positive Regulation: RAC1 --+> M/ 17568564
positive Regulation: RAC1 --+> M/ 18086571
positive Regulation: RAC1 --+> M/ 19450605 10.1016/j.yjmcc.2009.05.002
positive Regulation: RAC1 --+> M/ 19450605 10.1016/j.yjmcc.2009.05.002
positive Regulation: RAC1 --+> M/ 20175116 10.1002/jcp.22080
positive Regulation: RAC1 --+> M/ 20383193 10.1038/onc.2010.95
positive Regulation: RAC1 --+> M/ 21660950 10.1002/jcp.22563
positive Regulation: RAC1 --+> M/ 22114025 10.1152/ajpendo.00227.2011
positive Regulation: RAC1 --+> M/ 23524339 10.1016/j.cellsig.2013.03.011
positive Regulation: RAC1 --+> M/ 23817184 10.1159/000350108
positive Regulation: RAC1 --+> M/ 25725286 10.1016/j.cellsig.2015.02.020
positive Regulation: RAC1 --+> M/ 25136657 10.1155/2014/340271
positive Regulation: RAC1 --+> M/ 26379158 10.1371/journal.pone.0138381
positive Regulation: RAC1 --+> MAPK1
positive Regulation: RAC1 --+> M/ 26923603 10.1016/j.celrep.2016.02.016
positive Regulation: RAC1 --+> M/ 192 10.1016/j.bbrc.2015.12.056
positive Regulation: RAC1 --+> MAPK1 10.1074/jbc.M115.681544
positive Regulation: RAC1 --+> M/ 28287114 10.1038/cmi.2017.4
positive Regulation: RAC1 --+> M/ 28726720 10.3390/ijms1807157410.3390/ijms18071574
positive Regulation: RAC1 --+> M/ 29229918 10.1038/s41598-017-15170-610.1038/s41598-017-
positive Regulation: RAC1 --+> M/ 29329780 10.1016/j.cellsig.2018.01.00410.1016/j.cellsi
positive Regulation: RAC1 --+> M/ 29537337 10.1152/physrev.00025.2017
positive Regulation: RAC1 --+> M/ 33685622 10.1074/jbc.RA119.01098110.1074/jbc.RA119.010
positive Regulation: RAC1 --+> M/ 33879799 10.1038/s41467-021-22531-3
positive Regulation: RAC1 --+> M/ 28571533 10.2174/0929867324666170602082956
positive Regulation: RAC1 --+> M/ 34109430 10.3892/mmr.2021.1220710.3892/mmr.2021.12207
positive Regulation: RAC1 --+> M/ 34109430 10.3892/mmr.2021.12207
positive Regulation: RAC1 --+> M/ 33107320 10.1089/ars.2020.8199
positive Regulation: RAC1 --+> M/ 34326687 10.7150/ijbs.61790
ProtModification: MAPK1 ---> RAC129036929 10.3390/ijms18102148
ProtModification: MAPK1 ---> RAC130062409 10.1007/978-1-4939-8612-5_9
ProtModification: MAPK1 ---> RAC130062409 10.1007/978-1-4939-8612-5_9
ProtModification: MAPK1 ---> RAC130062409 10.1007/978-1-4939-8612-5_9
ProtModification: MAPK1 ---> RAC130062409 10.1007/978-1-4939-8612-5_9
ProtModification: MAPK1 ---> RAC130062409 10.1007/978-1-4939-8612-5_9
ProtModification: MAPK1 ---> RAC124043306 10.1128/MCB.00822-13
ProtModification: MAPK1 ---> RAC124043306 10.1128/MCB.00822-13
ProtModification: MAPK1 ---> RAC124043306 10.1128/MCB.00822-13
ProtModification: MAPK1 ---> RAC124043306 10.1128/MCB.00822-13

ProtModification: MAPK1 ---> RAC124043306 10.1128/MCB.00822-13
ProtModification: MAPK1 ---> RAC124043306 10.1128/MCB.00822-13
ProtModification: MAPK1 ---> RAC124043306 10.1128/MCB.00822-13
ProtModification: MAPK1 ---> RAC124043306 10.1128/MCB.00822-13
ProtModification: MAPK1 ---> RAC124043306 10.1128/MCB.00822-13
ProtModification: MAPK1 ---> RAC124043306 10.1128/MCB.00822-13
ProtModification: MAPK1 ---> RAC124043306 10.1128/MCB.00822-13
ProtModification: MAPK1 ---> RAC124043306 10.1128/MCB.00822-13
ProtModification: MAPK1 ---> RAC124043306 10.1128/MCB.00822-13
ProtModification: MAPK1 ---> RAC124043306 10.1128/MCB.00822-13
ProtModification: MAPK1 ---> RAC124043306 10.1128/MCB.00822-13
ProtModification: MAPK1 ---> RAC126816343 10.1371/journal.pone.0147103
ProtModification: MAPK1 ---> RAC126816343 10.1371/journal.pone.0147103
ProtModification: MAPK1 ---> RAC126816343 10.1371/journal.pone.0147103
ProtModification: MAPK1 ---> RAC126816343 10.1371/journal.pone.0147103
ProtModification: MAPK1 ---> RAC124043306 10.1128/MCB.00822-1310.1128/MCB.00822-13
ProtModification: MAPK1 ---> RAC124043306 10.1128/MCB.00822-1310.1128/MCB.00822-13
ProtModification: MAPK1 ---> RAC130062409 10.1007/978-1-4939-8612-5_910.1007/978-1-4939
ProtModification: MAPK1 ---> RAC130062409 10.1007/978-1-4939-8612-5_910.1007/978-1-4939
ProtModification: MAPK1 ---> RAC124043306 10.1128/MCB.00822-13
ProtModification: MAPK1 ---> RAC124462914 10.1016/j.bcp.2014.01.017
ProtModification: MAPK1 ---> RAC117853895
ProtModification: MAPK1 ---> RAC1
ProtModification: MAPK1 ---> RAC130062409 10.1007/978-1-4939-8612-5_9
positive PromoterBinding: PPARG -34026331 10.1080/2162402X.2021.190650010.1080/2162402X
positive PromoterBinding: PPARG -25072357 10.1016/j.lfs.2014.07.02110.1016/j.lfs.2014.0
positive PromoterBinding: PPARG -21435906 10.1016/j.healun.2011.01.704
positive PromoterBinding: PPARG -33766508 10.1016/j.jid.2021.02.748
positive PromoterBinding: PPARG -33766508 10.1016/j.jid.2021.02.748
positive PromoterBinding: PPARG -34697644 10.1007/s11882-021-01022-x
positive PromoterBinding: PPARG -35242187 10.1155/2022/3224373
positive Expression: AKT1 --+> MA11535584 10.1074/jbc.M101220200
positive Expression: AKT1 --+> MA20595063 10.1016/j.bbamcr.2010.05.007
positive Expression: AKT1 --+> MA12086645 10.1016/S0896-6273(02)00717-1
positive Expression: AKT1 --+> MA24333213 10.1016/j.ejphar.2013.12.005
positive Expression: AKT1 --+> MA24333213 10.1016/j.ejphar.2013.12.005
positive Expression: AKT1 --+> MA11535584
positive Expression: AKT1 --+> MA11535584
positive Expression: AKT1 --+> MA16195541
positive Expression: AKT1 --+> MA19668241
positive Expression: AKT1 --+> MA17172275
positive Expression: AKT1 --+> MA27147558 10.1152/ajpcell.00003.2016
positive Expression: AKT1 --+> MA26919896 10.1152/ajplung.00306.2015
positive Expression: AKT1 --+> MA27752996 10.1007/s13277-016-5433-z
positive Expression: AKT1 --+> MA21617941 10.1007/s11033-011-0908-x
positive Expression: AKT1 --+> MA23303682 10.1095/biolreprod.112.104778
positive Expression: AKT1 --+> MA23949281 10.1038/cgt.2013.44
positive Expression: AKT1 --+> MA24598933 10.1371/journal.pone.0089841
positive Expression: AKT1 --+> MA25569101 10.1038/cddis.2014.550
positive Expression: AKT1 --+> MA25872526 10.3892/ijmm.2015.2170
positive Expression: AKT1 --+> MA11535584 10.1074/jbc.M101220200
positive Expression: AKT1 --+> MA25872526 10.3892/ijmm.2015.217010.3892/ijmm.2015.2170
positive Expression: AKT1 --+> MA26578388 10.1038/srep1681310.1038/srep16813
positive Expression: AKT1 --+> MA27085942 10.1016/j.jep.2016.04.01910.1016/j.jep.2016.0

positive Expression: AKT1 --+> MA27752996 10.1007/s13277-016-5433-z
positive Expression: AKT1 --+> MA31518562 10.1016/j.ejphar.2019.17265810.1016/j.ejphar.
positive Expression: AKT1 --+> MA32553076 10.3967/bes2020.04410.3967/bes2020.044
positive Expression: AKT1 --+> MA14992744 10.1016/j.jamcollsurg.2003.10.004
positive Expression: AKT1 --+> MAPK1 10.3892/ijmm.2019.4275
Expression: MAPK1 ---> AKT1 16864584 10.1074/jbc.M602516200
Expression: MAPK1 ---> AKT1 22490886 10.1016/j.neulet.2012.03.068
Expression: MAPK1 ---> AKT1 23562504 10.1016/j.neulet.2013.02.067
Expression: MAPK1 ---> AKT1 24291216 10.1016/j.drudis.2013.11.018
Expression: MAPK1 ---> AKT1 16864584
Expression: MAPK1 ---> AKT1 12095987
Expression: MAPK1 ---> AKT1 21119656
Expression: MAPK1 ---> AKT1 21119656
Expression: MAPK1 ---> AKT1 19668241
Expression: MAPK1 ---> AKT1
Expression: MAPK1 ---> AKT1 27655635 10.1007/s00403-016-1686-z
Expression: MAPK1 ---> AKT1 29344654 10.3892/mmr.2018.8444
Expression: MAPK1 ---> AKT1 30621146 10.3390/ijms20010160
Expression: MAPK1 ---> AKT1 21904562 10.1155/2012/368196
Expression: MAPK1 ---> AKT1 23496764 10.1042/BJ20121578
Expression: MAPK1 ---> AKT1 24598933 10.1371/journal.pone.0089841
Expression: MAPK1 ---> AKT1 24787708 10.1371/journal.pone.009513610.1371/journal.p
Expression: MAPK1 ---> AKT1 27085942 10.1016/j.jep.2016.04.01910.1016/j.jep.2016.0
Expression: MAPK1 ---> AKT1 29344654 10.3892/mmr.2018.844410.3892/mmr.2018.8444
Expression: MAPK1 ---> AKT1 32553076 10.3967/bes2020.04410.3967/bes2020.044
Expression: MAPK1 ---> AKT1 10.1016/j.neulet.2012.03.068
Expression: MAPK1 ---> AKT1 10.3390/ijms20010160
Expression: MAPK1 ---> AKT1 10.1042/BJ20121578
Expression: MAPK1 ---> AKT1 28739729 10.21873/anticanres.11830
Expression: MAPK1 ---> AKT1 21762482 10.1186/1471-2202-12-69
Expression: MAPK1 ---> AKT1 10.1016/j.bbrc.2003.09.065
Expression: MAPK1 ---> AKT1 16858009
Expression: MAPK1 ---> AKT1 21503582 10.3892/or.2011.1264
Expression: MAPK1 ---> AKT1 20963488 10.1007/s10616-010-9310-0
Expression: MAPK1 ---> AKT1 23749166 10.1007/s11010-013-1710-y
Expression: MAPK1 ---> AKT1 25116684 10.3390/ijms150813833
Expression: MAPK1 ---> AKT1 25118589 10.1038/ncomms5657
Expression: MAPK1 ---> AKT1 27382014 10.1124/mol.116.104398
Expression: MAPK1 ---> AKT1 29770357 10.1515/med-2018-002910.1515/med-2018-0029
Expression: MAPK1 ---> AKT1 30364730 10.11909/j.issn.1671-5411.2018.07.00110.11909
ProtModification: AKT1 ---> MAPK130471866 10.1016/j.bbrc.2018.11.108
ProtModification: AKT1 ---> MAPK116282323 10.1074/jbc.M505546200
ProtModification: AKT1 ---> MAPK120693286 10.1074/jbc.M110.171058
ProtModification: AKT1 ---> MAPK120693286 10.1074/jbc.M110.171058
ProtModification: AKT1 ---> MAPK120693286 10.1074/jbc.M110.171058
ProtModification: AKT1 ---> MAPK120693286 10.1074/jbc.M110.171058
ProtModification: AKT1 ---> MAPK120693286 10.1074/jbc.M110.171058
ProtModification: AKT1 ---> MAPK118974095 10.1074/jbc.M805165200
ProtModification: AKT1 ---> MAPK129128169 10.1016/j.surg.2017.04.031
ProtModification: AKT1 ---> MAPK128522271 10.1016/j.mce.2017.05.015
ProtModification: AKT1 ---> MAPK132861756 10.1016/j.bbagen.2020.129723
ProtModification: AKT1 ---> MAPK1 10.1016/j.apsb.2021.04.016
ProtModification: AKT1 ---> MAPK132420479 10.1016/j.heliyon.2020.e03892
ProtModification: AKT1 ---> MAPK122178087 10.1016/j.mce.2011.11.024

ProtModification: AKT1 ---> MAPK119022565 10.1016/j.canlet.2008.10.013
ProtModification: AKT1 ---> MAPK120627457 10.1016/j.schres.2010.05.008
ProtModification: AKT1 ---> MAPK126844774 10.1016/j.bbamcr.2016.01.025
ProtModification: AKT1 ---> MAPK118974095
ProtModification: AKT1 ---> MAPK117931419
ProtModification: AKT1 ---> MAPK116282323 10.1074/jbc.M505546200
ProtModification: AKT1 ---> MAPK115355992
ProtModification: AKT1 ---> MAPK112468535
ProtModification: AKT1 ---> MAPK111303022
ProtModification: AKT1 ---> MAPK116763222
ProtModification: AKT1 ---> MAPK115319270
ProtModification: AKT1 ---> MAPK114640974
ProtModification: AKT1 ---> MAPK114640974
ProtModification: AKT1 ---> MAPK116858399
ProtModification: AKT1 ---> MAPK116648865
ProtModification: AKT1 ---> MAPK119151252
ProtModification: AKT1 ---> MAPK117893321
ProtModification: AKT1 ---> MAPK120844138
ProtModification: AKT1 ---> MAPK127521423 10.1152/ajpheart.00956.2015
ProtModification: AKT1 ---> MAPK129177675 10.1007/978-1-4939-7477-1_29
ProtModification: AKT1 ---> MAPK130205384 10.1159/000493411
ProtModification: AKT1 ---> MAPK133198383 10.3390/ijms21228542
ProtModification: AKT1 ---> MAPK133712543 10.1073/PNAS.2015786118
ProtModification: AKT1 ---> MAPK134299610 10.3390/molecules26144333
ProtModification: AKT1 ---> MAPK120693286 10.1074/jbc.M110.171058
ProtModification: AKT1 ---> MAPK120693286 10.1074/jbc.M110.171058
ProtModification: AKT1 ---> MAPK120693286 10.1074/jbc.M110.171058
ProtModification: AKT1 ---> MAPK120693286 10.1074/jbc.M110.171058
ProtModification: AKT1 ---> MAPK120693286 10.1074/jbc.M110.171058
ProtModification: AKT1 ---> MAPK120693286 10.1074/jbc.M110.171058
ProtModification: AKT1 ---> MAPK120693286 10.1074/jbc.M110.171058
ProtModification: AKT1 ---> MAPK120693286 10.1074/jbc.M110.171058
ProtModification: AKT1 ---> MAPK122101421 10.3892/mmr.2011.682
ProtModification: AKT1 ---> MAPK122449991
ProtModification: AKT1 ---> MAPK122134701
ProtModification: AKT1 ---> MAPK120844138 10.1523/JNEUROSCI.0895-10.2010
ProtModification: AKT1 ---> MAPK124068831 10.1523/JNEUROSCI.2010-13.2013
ProtModification: AKT1 ---> MAPK121519026 10.1200/JCO.2010.33.9473
ProtModification: AKT1 ---> MAPK121519026 10.1200/JCO.2010.33.9473
ProtModification: AKT1 ---> MAPK123593332 10.1371/journal.pone.0060863
ProtModification: AKT1 ---> MAPK124351840 10.3390/ijms141224656
ProtModification: AKT1 ---> MAPK124664246 10.1371/journal.pone.0092986
ProtModification: AKT1 ---> MAPK125028931 10.1371/journal.pone.0102521
ProtModification: AKT1 ---> MAPK125150498 10.1038/nm.3664
ProtModification: AKT1 ---> MAPK126504021
ProtModification: AKT1 ---> MAPK126808499 10.1172/JCI82788
ProtModification: AKT1 ---> MAPK112481435
ProtModification: AKT1 ---> MAPK116311120 10.1016/j.humpath.2005.09.009
ProtModification: AKT1 ---> MAPK116707604 10.1158/1078-0432.CCR-05-1732
ProtModification: AKT1 ---> MAPK120693286 10.1074/jbc.M110.17105810.1074/jbc.M110.17105
ProtModification: AKT1 ---> MAPK125504417 10.1002/mabi.20140042810.1002/mabi.201400428
ProtModification: AKT1 ---> MAPK127652596 10.1159/00044852410.1159/000448524
ProtModification: AKT1 ---> MAPK130076720 10.1002/jcp.2689110.1002/jcp.26891
ProtModification: AKT1 ---> MAPK120693286 10.1074/jbc.M110.171058

ProtModification: AKT1 ---> MAPK123585556 10.1530/EJE-13-0093
ProtModification: AKT1 ---> MAPK123970788 10.1210/en.2013-1197
ProtModification: AKT1 ---> MAPK124988468 10.1371/journal.pone.0101172
ProtModification: AKT1 ---> MAPK116311120 10.1016/j.humpath.2005.09.009
ProtModification: AKT1 ---> MAPK125028931 10.1371/journal.pone.0102521
ProtModification: AKT1 ---> MAPK123593332 10.1371/journal.pone.0060863
ProtModification: AKT1 ---> MAPK121519026 10.1200/JCO.2010.33.9473
ProtModification: AKT1 ---> MAPK116282323 10.1074/jbc.M505546200
ProtModification: AKT1 ---> MAPK126844774 10.1016/j.bbamcr.2016.01.025
ProtModification: AKT1 ---> MAPK121126166 10.1089/ars.2010.3401
ProtModification: AKT1 ---> MAPK122449991 10.2152/jmi.59.36
ProtModification: AKT1 ---> MAPK122101421 10.3892/mmr.2011.682
ProtModification: AKT1 ---> MAPK124351840 10.3390/ijms141224656
ProtModification: AKT1 ---> MAPK112468535 10.1074/jbc.M210578200
ProtModification: AKT1 ---> MAPK121220344 10.1073/pnas.1012975108
ProtModification: AKT1 ---> MAPK112481435
ProtModification: AKT1 ---> MAPK116648865 10.1038/modpathol.3800619
ProtModification: AKT1 ---> MAPK119371940 10.1016/j.urology.2008.12.058
ProtModification: AKT1 ---> MAPK111359783 10.1074/jbc.M103250200
ProtModification: AKT1 ---> MAPK122134701 10.1007/s11010-011-1137-2
ProtModification: AKT1 ---> MAPK117931419 10.1186/1476-4598-6-63
ProtModification: AKT1 ---> MAPK118974095 10.1074/jbc.M805165200
ProtModification: AKT1 ---> MAPK122178087 10.1016/j.mce.2011.11.024
ProtModification: AKT1 ---> MAPK119022565 10.1016/j.canlet.2008.10.013
ProtModification: AKT1 ---> MAPK125504417 10.1002/mabi.201400428
ProtModification: AKT1 ---> MAPK116952986 10.1161/CIRCULATIONAHA.105.602953
ProtModification: AKT1 ---> MAPK114640974 10.1042/BJ20031398
ProtModification: AKT1 ---> MAPK127652596 10.1159/000448524
ProtModification: AKT1 ---> MAPK114734530 10.1083/jcb.200307056
ProtModification: AKT1 ---> MAPK124664246 10.1371/journal.pone.0092986
ProtModification: AKT1 ---> MAPK115355992 10.1074/jbc.M408374200
ProtModification: AKT1 ---> MAPK111303022 10.1074/jbc.M011647200
ProtModification: AKT1 ---> MAPK1289 10.1007/bf02891714
ProtModification: AKT1 ---> MAPK115987944 10.1523/JNEUROSCI.1005-05.2005
ProtModification: AKT1 ---> MAPK120844138 10.1523/JNEUROSCI.0895-10.2010
ProtModification: AKT1 ---> MAPK119151252 10.1152/ajpheart.01147.2008
ProtModification: AKT1 ---> MAPK120926579 10.1210/en.2010-0792
ProtModification: AKT1 ---> MAPK120627457 10.1016/j.schres.2010.05.008
ProtModification: AKT1 ---> MAPK119372237 10.1210/me.2009-0011
ProtModification: AKT1 ---> MAPK111882599 10.1161/hy02t2.102907
ProtModification: AKT1 ---> MAPK115319270 10.1161/01.ATV.0000143096.15099.ce
ProtModification: AKT1 ---> MAPK1 10.2174/1566524016666160802145700
ProtModification: AKT1 ---> MAPK1 10.1172/JCI82788
ProtModification: AKT1 ---> MAPK134105991 10.1152/AJPLUNG.00181.2020
ProtModification: AKT1 ---> MAPK1289 10.1016/j.surg.2017.04.031
ProtModification: AKT1 ---> MAPK115998799
ProtModification: AKT1 ---> MAPK1
ProtModification: AKT1 ---> MAPK119371940 10.1016/j.urology.2008.12.058
ProtModification: AKT1 ---> MAPK119372237
ProtModification: AKT1 ---> MAPK114734530
ProtModification: AKT1 ---> MAPK111359783
ProtModification: AKT1 ---> MAPK116952986
ProtModification: AKT1 ---> MAPK111882599
ProtModification: AKT1 ---> MAPK115987944

ProtModification: AKT1 ---> MAPK120926579 10.1210/en.2010-0792
ProtModification: AKT1 ---> MAPK121126166 10.1089/ars.2010.3401
ProtModification: AKT1 ---> MAPK121220344 10.1073/pnas.1012975108
ProtModification: AKT1 ---> MAPK123585556 10.1530/EJE-13-0093
ProtModification: AKT1 ---> MAPK115894115 10.1016/j.imlet.2005.01.005
ProtModification: AKT1 ---> MAPK121406225 10.1016/j.cellsig.2011.03.009
ProtModification: AKT1 ---> MAPK115588981 10.1016/j.ymeth.2004.07.003
ProtModification: AKT1 ---> MAPK124927915 10.1016/j.jnutbio.2014.04.007
ProtModification: AKT1 ---> MAPK125749171 10.1016/j.tranon.2014.11.001
ProtModification: AKT1 ---> MAPK119955661
ProtModification: AKT1 ---> MAPK118577570
ProtModification: AKT1 ---> MAPK117463060
ProtModification: AKT1 ---> MAPK116395408
ProtModification: AKT1 ---> MAPK114593075
ProtModification: AKT1 ---> MAPK120955573
ProtModification: AKT1 ---> MAPK115256384
ProtModification: AKT1 ---> MAPK117535857
ProtModification: AKT1 ---> MAPK122406993 10.4161/cbt.19609
ProtModification: AKT1 ---> MAPK122403294 10.2337/db11-1578
ProtModification: AKT1 ---> MAPK120955573 10.1186/1471-2121-11-78
ProtModification: AKT1 ---> MAPK122683727 10.1097/SHK.0b013e31825da754
ProtModification: AKT1 ---> MAPK124098385 10.1371/journal.pone.0075440
ProtModification: AKT1 ---> MAPK123970788 10.1210/en.2013-1197
ProtModification: AKT1 ---> MAPK124423728 10.1038/ncomms4039
ProtModification: AKT1 ---> MAPK124983470 10.1371/journal.pone.0101349
ProtModification: AKT1 ---> MAPK124988468 10.1371/journal.pone.0101172
ProtModification: AKT1 ---> MAPK124988468 10.1371/journal.pone.0101172
ProtModification: AKT1 ---> MAPK122431920 10.1593/neo.111602
ProtModification: AKT1 ---> MAPK125832422 10.1038/aps.2014.156
ProtModification: AKT1 ---> MAPK126164453 10.1097/WNR.0000000000000401
ProtModification: AKT1 ---> MAPK1
ProtModification: AKT1 ---> MAPK124394804 10.1038/ncomms4051
ProtModification: AKT1 ---> MAPK112444071
ProtModification: AKT1 ---> MAPK121293001
ProtModification: AKT1 ---> MAPK119717735
ProtModification: AKT1 ---> MAPK121293001 10.1161/CIRCRESAHA.110.238949
ProtModification: AKT1 ---> MAPK124394804 10.1038/ncomms4051
ProtModification: AKT1 ---> MAPK128481866 10.1038/onc.2017.114
ProtModification: AKT1 ---> MAPK128589317 10.1007/s12079-017-0395-5
ProtModification: AKT1 ---> MAPK129412164 10.1016/j.bbrc.2018.02.002
ProtModification: AKT1 ---> MAPK129517106 10.3892/or.2018.6305
ProtModification: AKT1 ---> MAPK129762523 10.3390/ijms19051470
ProtModification: AKT1 ---> MAPK129551770 10.1038/s41388-018-0170-y
ProtModification: AKT1 ---> MAPK129741098 10.1152/ajprenal.00033.2018
ProtModification: AKT1 ---> MAPK129741098 10.1152/ajprenal.00033.2018
ProtModification: AKT1 ---> MAPK1 10.4143/crt.2018.526
ProtModification: AKT1 ---> MAPK1 10.1097/WNR.0000000000001241
ProtModification: AKT1 ---> MAPK135109876 10.1186/s12974-022-02405-1
ProtModification: MAPK1 ---> AKT126952643 10.1016/j.ajpath.2016.01.003
ProtModification: MAPK1 ---> AKT128007595 10.1016/j.bbrc.2016.12.117
ProtModification: MAPK1 ---> AKT130007439 10.1016/j.bbrc.2018.07.021
ProtModification: MAPK1 ---> AKT112435727 10.1074/jbc.M209148200
ProtModification: MAPK1 ---> AKT1 10.1074/jbc.M005911200
ProtModification: MAPK1 ---> AKT114701865 10.1074/jbc.M312048200

ProtModification: MAPK1 ---> AKT114973141 10.1074/jbc.M312996200
ProtModification: MAPK1 ---> AKT115020586 10.1074/jbc.M314099200
ProtModification: MAPK1 ---> AKT118974095 10.1074/jbc.M805165200
ProtModification: MAPK1 ---> AKT117200115 10.1074/jbc.M609712200
ProtModification: MAPK1 ---> AKT111359783 10.1074/jbc.M103250200
ProtModification: MAPK1 ---> AKT131931009 10.1016/j.jmb.2020.01.002
ProtModification: MAPK1 ---> AKT129909367 10.1016/j.molimm.2018.05.021
ProtModification: MAPK1 ---> AKT129981429 10.1016/j.canlet.2018.06.040
ProtModification: MAPK1 ---> AKT126424114 10.1016/j.freeradbiomed.2015.08.011
ProtModification: MAPK1 ---> AKT131479709 10.1016/j.cellsig.2019.109409
ProtModification: MAPK1 ---> AKT132169603 10.1016/j.bone.2020.115316
ProtModification: MAPK1 ---> AKT112054675 10.1016/S0006-291X(02)00385-6
ProtModification: MAPK1 ---> AKT112543452 10.1016/S0006-8993(02)03774-5
ProtModification: MAPK1 ---> AKT118590724 10.1016/j.ejphar.2008.06.040
ProtModification: MAPK1 ---> AKT121316056 10.1016/j.atherosclerosis.2011.01.019
ProtModification: MAPK1 ---> AKT118715655 10.1016/j.jneuroim.2008.05.018
ProtModification: MAPK1 ---> AKT118359096 10.1016/j.tips.2008.02.002
ProtModification: MAPK1 ---> AKT119046792 10.1016/j.lungcan.2008.10.009
ProtModification: MAPK1 ---> AKT122178087 10.1016/j.mce.2011.11.024
ProtModification: MAPK1 ---> AKT116427189 10.1016/j.canlet.2005.11.001
ProtModification: MAPK1 ---> AKT112086645 10.1016/S0896-6273(02)00717-1
ProtModification: MAPK1 ---> AKT115567066 10.1016/j.cellsig.2004.08.002
ProtModification: MAPK1 ---> AKT120627457 10.1016/j.schres.2010.05.008
ProtModification: MAPK1 ---> AKT123043959 10.1016/j.yexcr.2012.09.018
ProtModification: MAPK1 ---> AKT122944602 10.1016/j.neuint.2012.08.010
ProtModification: MAPK1 ---> AKT123376282 10.1016/j.jep.2013.01.032
ProtModification: MAPK1 ---> AKT124362258 10.1016/j.ygcen.2013.12.006
ProtModification: MAPK1 ---> AKT114523037
ProtModification: MAPK1 ---> AKT117200115
ProtModification: MAPK1 ---> AKT115632127
ProtModification: MAPK1 ---> AKT115020586
ProtModification: MAPK1 ---> AKT114973141
ProtModification: MAPK1 ---> AKT114701865
ProtModification: MAPK1 ---> AKT112435727
ProtModification: MAPK1 ---> AKT111359783
ProtModification: MAPK1 ---> AKT111340082
ProtModification: MAPK1 ---> AKT114979873
ProtModification: MAPK1 ---> AKT114640974
ProtModification: MAPK1 ---> AKT112761576
ProtModification: MAPK1 ---> AKT119151252
ProtModification: MAPK1 ---> AKT115870293
ProtModification: MAPK1 ---> AKT112969887
ProtModification: MAPK1 ---> AKT128259922 10.3892/mmr.2017.6195
ProtModification: MAPK1 ---> AKT128498467 10.3892/ijo.2017.3982
ProtModification: MAPK1 ---> AKT128731146 10.3892/ijmm.2017.3069
ProtModification: MAPK1 ---> AKT130348829 10.1042/CS20180557
ProtModification: MAPK1 ---> AKT130348829 10.1042/CS20180557
ProtModification: MAPK1 ---> AKT130348829 10.1042/CS20180557
ProtModification: MAPK1 ---> AKT131138788 10.1038/s41419-019-1656-4
ProtModification: MAPK1 ---> AKT130758982 10.1152/ajpregu.00251.2018
ProtModification: MAPK1 ---> AKT130758982 10.1152/ajpregu.00251.2018
ProtModification: MAPK1 ---> AKT131117055 10.1530/JOE-18-0704
ProtModification: MAPK1 ---> AKT131619201 10.1186/s12885-019-6161-8
ProtModification: MAPK1 ---> AKT132564026 10.1159/000506956

ProtModification: MAPK1 ---> AKT132772213 10.1007/s10555-020-09920-8
ProtModification: MAPK1 ---> AKT133498448 10.3390/ijms22031021
ProtModification: MAPK1 ---> AKT133972443 10.1073/pnas.2026104118
ProtModification: MAPK1 ---> AKT122304473 10.1089/ars.2011.4005
ProtModification: MAPK1 ---> AKT122499584 10.1096/fj.11-202994
ProtModification: MAPK1 ---> AKT123027608 10.1530/JOE-12-0335
ProtModification: MAPK1 ---> AKT124065093 10.3390/ijms140918861
ProtModification: MAPK1 ---> AKT121795553 10.1523/JNEUROSCI.2125-11.2011
ProtModification: MAPK1 ---> AKT121795553 10.1523/JNEUROSCI.2125-11.2011
ProtModification: MAPK1 ---> AKT123922774 10.1371/journal.pone.0069668
ProtModification: MAPK1 ---> AKT124324260 10.1074/jbc.M113.505065
ProtModification: MAPK1 ---> AKT124874473 10.1038/bjc.2014.282
ProtModification: MAPK1 ---> AKT125028931 10.1371/journal.pone.0102521
ProtModification: MAPK1 ---> AKT112481435
ProtModification: MAPK1 ---> AKT116311120 10.1016/j.humpath.2005.09.009
ProtModification: MAPK1 ---> AKT117634416 10.1152/ajpcell.00535.2006
ProtModification: MAPK1 ---> AKT148 10.1016/j.bbrc.2018.07.021
ProtModification: MAPK1 ---> AKT1 10.1016/j.jmb.2020.01.002
ProtModification: MAPK1 ---> AKT1 10.1016/j.cellsig.2004.08.002
ProtModification: MAPK1 ---> AKT110477751
ProtModification: MAPK1 ---> AKT1 10.1042/CS20180557
ProtModification: MAPK1 ---> AKT1 10.1042/CS20180557
ProtModification: MAPK1 ---> AKT1 10.1042/CS20180557
ProtModification: MAPK1 ---> AKT1 10.1152/ajpregu.00251.2018
ProtModification: MAPK1 ---> AKT1 10.1152/ajpregu.00251.2018
ProtModification: MAPK1 ---> AKT1 10.1530/JOE-18-0704
ProtModification: MAPK1 ---> AKT1 10.1186/s12885-019-6161-8
ProtModification: MAPK1 ---> AKT1 10.1074/jbc.M113.505065
ProtModification: MAPK1 ---> AKT125892444 10.1016/j.mce.2015.04.008
ProtModification: MAPK1 ---> AKT118362153
ProtModification: MAPK1 ---> AKT112133838
ProtModification: MAPK1 ---> AKT110488121
ProtModification: MAPK1 ---> AKT116952986
ProtModification: MAPK1 ---> AKT116151019
ProtModification: MAPK1 ---> AKT126683901 10.1152/ajpheart.00642.2015
ProtModification: MAPK1 ---> AKT128692379 10.1080/15384047.2017.1345384
ProtModification: MAPK1 ---> AKT122454147 10.1210/en.2011-1967
ProtModification: MAPK1 ---> AKT121285867 10.1097/CAD.0b013e3283439781
ProtModification: MAPK1 ---> AKT123766351 10.1161/CIRCULATIONAHA.112.000029
ProtModification: MAPK1 ---> AKT123584088 10.1038/nm.3150
ProtModification: MAPK1 ---> AKT123986484 10.1242/jcs.133421
ProtModification: MAPK1 ---> AKT123986484 10.1242/jcs.133421
ProtModification: MAPK1 ---> AKT123986484 10.1242/jcs.133421
ProtModification: MAPK1 ---> AKT123986484 10.1242/jcs.133421
ProtModification: MAPK1 ---> AKT121408177 10.1371/journal.pone.0017621
ProtModification: MAPK1 ---> AKT124130815 10.1371/journal.pone.0076951
ProtModification: MAPK1 ---> AKT126718891 10.3892/mmr.2015.4619
ProtModification: MAPK1 ---> AKT116176809 10.1016/j.bbrc.2005.08.260
ProtModification: MAPK1 ---> AKT115922325 10.1016/j.ejphar.2005.04.035
ProtModification: MAPK1 ---> AKT117950068 10.1016/j.jss.2007.01.051
ProtModification: MAPK1 ---> AKT119914172 10.1016/j.cell.2009.10.023
ProtModification: MAPK1 ---> AKT116083987 10.1016/j.jhep.2005.04.011
ProtModification: MAPK1 ---> AKT118407408 10.1016/j.canlet.2008.02.064
ProtModification: MAPK1 ---> AKT122444502 10.1016/j.jnutbio.2011.11.002

ProtModification: MAPK1 ---> AKT1 10.1016/j.fob.2012.02.002
ProtModification: MAPK1 ---> AKT123399764 10.1016/j.ejphar.2013.01.048
ProtModification: MAPK1 ---> AKT123650581 10.1016/j.fob.2012.02.002
ProtModification: MAPK1 ---> AKT118045963
ProtModification: MAPK1 ---> AKT112183360
ProtModification: MAPK1 ---> AKT117616749
ProtModification: MAPK1 ---> AKT118493596
ProtModification: MAPK1 ---> AKT122895181 10.4161/cc.21194
ProtModification: MAPK1 ---> AKT123254561 10.3892/or.2012.2195
ProtModification: MAPK1 ---> AKT120630868 10.1074/jbc.M110.108241
ProtModification: MAPK1 ---> AKT121876713 10.1155/2012/486568
ProtModification: MAPK1 ---> AKT121107779 10.1007/s13105-010-0063-1
ProtModification: MAPK1 ---> AKT123776078 10.1084/jem.20112495
ProtModification: MAPK1 ---> AKT122733137 10.1038/onc.2012.253
ProtModification: MAPK1 ---> AKT123358187 10.1038/mt.2013.9
ProtModification: MAPK1 ---> AKT122880006 10.1371/journal.pone.0042501
ProtModification: MAPK1 ---> AKT124423728 10.1038/ncomms4039
ProtModification: MAPK1 ---> AKT123193052 10.1152/ajpendo.00409.2012
ProtModification: MAPK1 ---> AKT123275616 10.1152/ajpgi.00418.2012
ProtModification: MAPK1 ---> AKT112147707
ProtModification: MAPK1 ---> AKT124642271 10.1186/1476-4598-13-61
ProtModification: MAPK1 ---> AKT125962684 10.3892/or.2015.3941
ProtModification: MAPK1 ---> AKT121107779 10.1007/s13105-010-0063-1
ProtModification: MAPK1 ---> AKT1
ProtModification: MAPK1 ---> AKT127879454 10.1177/1933719116678689
ProtModification: MAPK1 ---> AKT128784627 10.1084/jem.20161855
ProtModification: MAPK1 ---> AKT129258294 10.1038/nature25167
ProtModification: MAPK1 ---> AKT129369798 10.1016/j.jamcollsurg.2017.12.040
ProtModification: MAPK1 ---> AKT129517103 10.3892/or.2018.6306
ProtModification: MAPK1 ---> AKT130042178 10.1242/dev.162594
ProtModification: MAPK1 ---> AKT130226884 10.1371/journal.pone.0203952
ProtModification: MAPK1 ---> AKT129856035 10.1007/978-1-4939-7825-0_25
ProtModification: MAPK1 ---> AKT1 10.1007/s00011-019-01218-y
ProtModification: MAPK1 ---> AKT134386919 10.1007/s11064-021-03418-7
ProtModification: MAPK1 ---> AKT1 10.1016/j.ejmech.2021.113996
ProtModification: MAPK1 ---> AKT136207295 10.1038/s41467-022-33521-4
ProtModification: MAPK1 ---> AKT136555673 10.3390/ijms232416028
positive Expression: PTGS2 --+> S29736687 10.1007/s10787-018-0489-6
negative Regulation: SFRP1 ---| F20367282 10.1089/scd.2009.0326
negative Regulation: NOTCH1 ---| 26302232 10.1016/j.biomaterials.2015.08.026
negative Regulation: NOTCH1 ---| 30336197 10.1016/j.canlet.2018.09.031
negative Regulation: NOTCH1 ---| 30497876 10.1016/j.prp.2018.10.013
negative Regulation: NOTCH1 ---| 29879503 10.1016/j.gene.2018.06.005
negative Regulation: NOTCH1 ---| 31545250 10.1016/j.biopha.2019.109306
negative Regulation: NOTCH1 ---| 29723707 10.1016/j.dnarep.2018.04.002
negative Regulation: NOTCH1 ---| 27727250 10.1038/mtna.2016.82
negative Regulation: NOTCH1 ---| 21420785 10.1016/j.canlet.2011.02.034
negative Regulation: NOTCH1 ---| 20600632 10.1016/j.bbcan.2010.06.001
negative Regulation: NOTCH1 ---| 23500524 10.1016/j.biocel.2013.02.022
negative Regulation: NOTCH1 ---| 23500524 10.1016/j.biocel.2013.02.022
negative Regulation: NOTCH1 ---| 23810012 10.1016/B978-0-12-416673-8.00013-7
negative Regulation: NOTCH1 ---| 28914645 10.1097/CCO.0000000000000406
negative Regulation: NOTCH1 ---| 32228202 10.1177/0300060520904847
negative Regulation: NOTCH1 ---| 32913140 10.1124/MOLPHARM.120.000006

negative Regulation: NOTCH1 ---|	32913140	10.1124/MOLPHARM.120.000006
negative Regulation: NOTCH1 ---|	24451948	10.3760/cma.j.issn.0366-6999.20120230
negative Regulation: NOTCH1 ---|	24451948	10.3760/cma.j.issn.0366-6999.20120230
negative Regulation: NOTCH1 ---|	26927514	10.1038/labinvest.2015.163
negative Regulation: NOTCH1 ---|	19550121	
negative Regulation: NOTCH1 ---|	20204271	10.3892/or_00000712
negative Regulation: NOTCH1 ---|	20204271	10.3892/or_00000712
negative Regulation: NOTCH1 ---|	23568245	10.1007/s00268-013-2010-010.1007/s00268-013-2
negative Regulation: NOTCH1 ---|	24316112	10.1016/j.arcmed.2013.10.00110.1016/j.arcmed.
negative Regulation: NOTCH1 ---|	24782036	10.1007/s11010-014-2069-410.1007/s11010-014-2
negative Regulation: NOTCH1 ---|	24802328	10.1007/s12032-014-0972-x10.1007/s12032-014-0
negative Regulation: NOTCH1 ---|	31545250	10.1016/j.biopha.2019.10930610.1016/j.biopha.
negative Regulation: NOTCH1 ---|	1150	10.1016/j.prp.2018.10.013
negative Regulation: NOTCH1 ---|	chemosens	10.1097/CCO.0000000000000406
negative Regulation: NOTCH1 ---|	chemosens	10.2298/ABS210423030R
negative Regulation: NOTCH1 ---|	33882814	10.2174/1871530321666210421125231
positive Regulation: CD274 --+> A	32560747	10.3727/096504020X15925659763817
Expression: MAPK1 ---> BIRC7	32359099	10.1113/EP08858410.1113/EP088584
Expression: MAPK1 ---> BIRC7	32359099	10.1113/EP08858410.1113/EP088584
positive Expression: UCA1 --+> AK	29864621	10.1016/j.biopha.2018.05.141
positive Expression: UCA1 --+> AK	27765938	10.12659/MSM.900689
positive Expression: UCA1 --+> AK	29328452	10.3892/or.2018.6182
positive Expression: UCA1 --+> AK	29762839	10.26355/eurrev-201804-14828
positive Expression: UCA1 --+> AK	29762839	10.26355/eurrev-201804-14828
positive Expression: UCA1 --+> AK	30208633	10.3390/ijms19092711
positive Expression: UCA1 --+> AK	30272271	10.3892/ijo.2018.4574
positive Expression: UCA1 --+> AK	31799676	10.26355/eurrev_201911_19573
positive Expression: UCA1 --+> AK	24312245	10.1371/journal.pone.0080817
positive Expression: UCA1 --+> AK	33777227	10.3892/ol.2021.1266510.3892/ol.2021.12665
positive Expression: UCA1 --+> AKT1		10.26355/eurrev_201911_19573
positive Expression: UCA1 --+> AK	32601922	10.1007/s11033-020-05609-x
positive Expression: UCA1 --+> AK	32627021	10.3892/or.2020.7638
positive Expression: UCA1 --+> AK	29212166	10.18632/oncotarget.1928110.18632/oncotarget.
positive Expression: UCA1 --+> AK	28569779	10.1038/cddis.2017.14310.1038/cddis.2017.143
positive Expression: UCA1 --+> AK	26464647	
positive Expression: UCA1 --+> AK	29328452	10.3892/or.2018.6182
positive Expression: UCA1 --+> AK	28751619	10.18632/oncotarget.1928110.18632/oncotarget.
positive Expression: UCA1 --+> AKT1		10.1691/ph.2016.6625
positive Expression: UCA1 --+> AKT1		10.1691/ph.2016.6625
positive Expression: UCA1 --+> AK	30015961	10.3892/mmr.2018.9278
positive Expression: UCA1 --+> AKT1		10.1042/BSR20190590
positive Expression: UCA1 --+> AKT1		10.3892/ijo.2019.4679
positive Expression: UCA1 --+> AK	30272345	10.3892/ijo.2018.4575
positive Expression: UCA1 --+> AK	30272271	10.3892/ijo.2018.4574
positive Expression: UCA1 --+> AKT1		10.1016/j.jid.2019.04.029
positive Expression: UCA1 --+> AK	33565716	10.1002/prp2.72010.1002/prp2.720
positive Expression: UCA1 --+> AK	28569779	10.1038/cddis.2017.143
positive Expression: UCA1 --+> AK	33743811	10.1186/s13048-021-00792-2
positive Expression: UCA1 --+> AK	33535952	10.2174/1568009621666210203110305
positive Expression: UCA1 --+> AK	33432087	10.1038/s41417-020-00272-7
positive Expression: UCA1 --+> AK	35117446	10.21037/tcr.2019.12.4710.21037/tcr.2019.12.4
positive Expression: UCA1 --+> AKT1		10.1016/j.hbpd.2022.12.002
PromoterBinding: PHF20 ---> TP53	22334668	10.1074/jbc.M111.333922
PromoterBinding: PHF20 ---> TP53	22334668	10.1074/jbc.M111.333922

PromoterBinding: PHF20 ---> TP53 22334668 10.1074/jbc.M111.333922
PromoterBinding: PHF20 ---> TP53 22334668 10.1074/jbc.M111.333922
PromoterBinding: PHF20 ---> TP53 22334668 10.1074/jbc.M111.333922
PromoterBinding: PHF20 ---> TP53 32555448 10.1038/s41418-020-0580-6
PromoterBinding: PHF20 ---> TP53 22334668 10.1074/jbc.M111.333922
PromoterBinding: PHF20 ---> TP53 22334668 10.1074/jbc.M111.333922
PromoterBinding: PHF20 ---> TP53 22334668 10.1074/jbc.M111.333922
PromoterBinding: PHF20 ---> TP53 22334668 10.1074/jbc.M111.333922
PromoterBinding: PHF20 ---> TP53 22334668 10.1074/jbc.M111.333922
PromoterBinding: PHF20 ---> TP53 22334668 10.1074/jbc.M111.333922
positive PromoterBinding: PPARG -30119946 10.1016/j.tim.2018.08.001
positive PromoterBinding: PPARG -29928066 10.1371/journal.ppat.100710010.1371/journal.p
positive PromoterBinding: PPARG -29928066 10.1371/journal.ppat.1007100
positive PromoterBinding: PPARG -29928066 10.1371/journal.ppat.1007100
positive PromoterBinding: PPARG -29928066 10.1371/journal.ppat.1007100
positive PromoterBinding: PPARG -29928066 10.1371/journal.ppat.1007100
positive PromoterBinding: PPARG -29928066 10.1371/journal.ppat.1007100
positive PromoterBinding: PPARG -25944662 10.1016/j.bbrc.2015.04.023
positive PromoterBinding: PPARG --+> MCL1 10.1016/S1074-7613(04)00030-5
positive PromoterBinding: PPARG -30119946 10.1016/j.tim.2018.08.001
positive PromoterBinding: PPARG -607 10.1016/j.tim.2018.08.001
positive PromoterBinding: PPARG -607 10.1016/j.tim.2018.08.001
negative Expression: MAPK1 ---| (21669876 10.1074/jbc.M111.248435
negative Expression: MAPK1 ---| (21669876 10.1074/jbc.M111.248435
negative Expression: MAPK1 ---| (23846224 10.1038/cddis.2013.253
negative Expression: MAPK1 ---| (19498045
negative Expression: MAPK1 ---| (22748238 10.1016/j.cytogfr.2012.05.008
Regulation: BIRC7 ---> AKT1 23524337 10.1016/j.cellsig.2013.03.012
Regulation: BIRC7 ---> AKT1 29039608 10.3892/or.2017.6017
Regulation: BIRC7 ---> AKT1 33673050 10.3390/ijms22041820
Regulation: BIRC7 ---> AKT1 27175933 10.3892/mmr.2016.5242
Regulation: BIRC7 ---> AKT1 27175933 10.3892/mmr.2016.5242
Regulation: BIRC7 ---> AKT1 27175933 10.3892/mmr.2016.5242
Regulation: BIRC7 ---> AKT1 27175933 10.3892/mmr.2016.5242
Regulation: BIRC7 ---> AKT1 27175933 10.3892/mmr.2016.524210.3892/mmr.2016.5242
negative Expression: NOTCH1 ---| 22613711 10.1074/jbc.M111.292516
negative Expression: NOTCH1 ---| 22613711 10.1074/jbc.M111.292516
negative Expression: NOTCH1 ---| 22613711 10.1074/jbc.M111.292516
negative Expression: NOTCH1 ---| 22613711 10.1074/jbc.M111.292516
negative Expression: NOTCH1 ---| 22613711 10.1074/jbc.M111.292516
negative Expression: NOTCH1 ---| 22613711 10.1074/jbc.M111.292516
negative Expression: NOTCH1 ---| 22613711 10.1074/jbc.M111.292516
negative Expression: NOTCH1 ---| 22613711 10.1074/jbc.M111.292516
negative Expression: NOTCH1 ---| 22613711 10.1074/jbc.M111.292516
negative Expression: NOTCH1 ---| 22613711 10.1074/jbc.M111.292516
negative Expression: NOTCH1 ---| 22613711 10.1074/jbc.M111.292516
negative Expression: NOTCH1 ---| 22613711 10.1074/jbc.M111.292516
negative Expression: PTEN ---| PI28844858 10.1016/j.cbi.2017.08.014
negative Expression: PTEN ---| PI32890667 10.1016/j.cellsig.2020.109767
negative Expression: PTEN ---| PI22898050 10.1016/j.bbrc.2012.07.165
negative Expression: PTEN ---| PI25058027 10.1016/j.ajpath.2014.05.023
negative Expression: PTEN ---| PI25058027 10.1016/j.ajpath.2014.05.023
negative Expression: PTEN ---| PI25176064 10.1016/j.biomaterials.2014.08.016
negative Expression: PTEN ---| PI25176064 10.1016/j.biomaterials.2014.08.016

negative Expression: PTEN ---|	PT	26984887	10.1530/ERC-16-0039
negative Expression: PTEN ---|	PT	29582250	10.1007/s12264-018-0219-5
negative Expression: PTEN ---|	PT	29687845	10.26355/eurrev-201804-14717
negative Expression: PTEN ---|	PT	30406888	10.1007/s11033-018-4378-2
negative Expression: PTEN ---|	PT	22746381	10.1089/ars.2011.4408
negative Expression: PTEN ---|	PT	23589525	10.1210/jc.2013-1106
negative Expression: PTEN ---|	PT	23633918	10.1593/neo.121024
negative Expression: PTEN ---|	PT	25176064	10.1016/j.biomaterials.2014.08.01610.1016/j.b
negative Expression: PTEN ---|	PTGS2		10.1530/ERC-16-0039
negative Expression: PTEN ---|	PTGS2		
negative Expression: PTGS2 ---|	P	29339153	10.1016/j.bbrc.2018.01.061
negative Expression: PTGS2 ---|	P	29339153	10.1016/j.bbrc.2018.01.061
negative Expression: PTGS2 ---|	P	30243936	10.1016/j.gene.2018.09.034
negative Expression: PTGS2 ---|	P	25770423	10.1016/j.bbrc.2015.03.008
negative Expression: PTGS2 ---|	P	25770423	10.1016/j.bbrc.2015.03.008
negative Expression: PTGS2 ---|	P	25770423	10.1016/j.bbrc.2015.03.008
negative Expression: PTGS2 ---|	P	25770423	10.1016/j.bbrc.2015.03.008
negative Expression: PTGS2 ---|	P	25770423	10.1016/j.bbrc.2015.03.008
negative Expression: PTGS2 ---|	P	28851377	10.1186/s12943-017-0718-4
negative Expression: PTGS2 ---|	P	29048666	10.3892/or.2017.5981
negative Expression: PTGS2 ---|	P	29596308	10.3390/ijms19041019
negative Expression: PTGS2 ---|	P	26554780	10.1097/MD.000000000000180610.1097/MD.0000000
negative Expression: PTGS2 ---|	P	27385366	10.18632/aging.10098710.18632/aging.100987
negative Expression: PTGS2 ---|	P	28502821	10.1016/j.mad.2017.05.004
negative Expression: PTGS2 ---|	P	25770423	10.1016/j.bbrc.2015.03.008
negative Expression: PTGS2 ---|	P	20854947	10.1016/j.bone.2010.09.020
negative Expression: PTGS2 ---|	P	20854947	10.1016/j.bone.2010.09.020
negative Expression: PTGS2 ---|	P	22898050	10.1016/j.bbrc.2012.07.165
negative Expression: PTGS2 ---|	P	15767780	
negative Expression: PTGS2 ---|	PTEN		
negative Expression: PTGS2 ---|	P	28791404	10.3892/or.2017.5872
Regulation: NOTCH1 --->	MAPK1	29080702	10.1016/j.pharmthera.2017.10.017
Regulation: NOTCH1 --->	MAPK1	15817159	10.1016/j.yexcr.2005.01.015
Regulation: NOTCH1 --->	MAPK1	20616044	
Regulation: NOTCH1 --->	MAPK1		10.1074/jbc.M116.759134
Regulation: NOTCH1 --->	MAPK1		10.1096/fj.201601341RRR
Regulation: NOTCH1 --->	MAPK1	29874112	10.1152/ajpcell.00182.2017
Regulation: NOTCH1 --->	MAPK1	22205705	10.1074/jbc.M111.310375
Regulation: NOTCH1 --->	MAPK1	22004682	10.1186/1476-4598-10-130
Regulation: NOTCH1 --->	MAPK1	20616044	10.1073/pnas.1000132107
Regulation: NOTCH1 --->	MAPK1	23531541	10.3390/ijms14046863
Regulation: NOTCH1 --->	MAPK1	23542173	10.1038/onc.2013.74
Regulation: NOTCH1 --->	MAPK1	24472723	10.1124/jpet.113.207563
Regulation: NOTCH1 --->	MAPK1		10.1074/jbc.M116.720698
Regulation: NOTCH1 --->	MAPK1		10.1074/jbc.M116.720698
Regulation: NOTCH1 --->	MAPK1	23533157	10.1002/stem.137310.1002/stem.1373
Regulation: NOTCH1 --->	MAPK1	24472723	10.1124/jpet.113.20756310.1124/jpet.113.20756
Regulation: NOTCH1 --->	MAPK1	21622856	10.4049/jimmunol.100360610.4049/jimmunol.1003
Regulation: NOTCH1 --->	MAPK1	17320900	10.1016/j.jmb.2006.10.043
Regulation: NOTCH1 --->	MAPK1		10.1016/S0197-4580(98)00041-4
Regulation: NOTCH1 --->	MAPK1	18332140	
Regulation: NOTCH1 --->	MAPK1	17664272	
Regulation: NOTCH1 --->	MAPK1	17090547	
Regulation: NOTCH1 --->	MAPK1	17090547	

Regulation: NOTCH1 ---> MAPK1	14602722	
Regulation: NOTCH1 ---> MAPK1	20616044	
Regulation: NOTCH1 ---> MAPK1	17210639	
Regulation: NOTCH1 ---> MAPK1	27572051	10.3892/mmr.2016.5688
Regulation: NOTCH1 ---> MAPK1	21768114	10.1074/jbc.M111.232413
Regulation: NOTCH1 ---> MAPK1	21768114	10.1074/jbc.M111.232413
Regulation: NOTCH1 ---> MAPK1	22205705	10.1074/jbc.M111.310375
Regulation: NOTCH1 ---> MAPK1	22205705	10.1074/jbc.M111.310375
Regulation: NOTCH1 ---> MAPK1	22205705	10.1074/jbc.M111.310375
Regulation: NOTCH1 ---> MAPK1	22205705	10.1074/jbc.M111.310375
Regulation: NOTCH1 ---> MAPK1	20616044	10.1073/pnas.1000132107
Regulation: NOTCH1 ---> MAPK1	20616044	10.1073/pnas.1000132107
Regulation: NOTCH1 ---> MAPK1	21273559	10.1161/ATVBAHA.110.221945
Regulation: NOTCH1 ---> MAPK1	24714220	10.4161/cam.28437
Regulation: NOTCH1 ---> MAPK1	25194568	10.1038/nm.3678
Regulation: NOTCH1 ---> MAPK1	25194568	10.1038/nm.3678
Regulation: NOTCH1 ---> MAPK1	21622856	10.4049/jimmunol.1003606
Regulation: NOTCH1 ---> MAPK1	25194568	10.1038/nm.3678
Regulation: NOTCH1 ---> MAPK1	25194568	10.1038/nm.3678
Regulation: NOTCH1 ---> MAPK1	27578780	10.1242/dev.137919
Regulation: NOTCH1 ---> MAPK1	27578780	10.1242/dev.137919
Regulation: NOTCH1 ---> MAPK1	24504340	10.1242/dev.098905
Regulation: NOTCH1 ---> MAPK1	25380967	10.1186/1476-4598-13-247
Regulation: NOTCH1 ---> MAPK1		
Regulation: NOTCH1 ---> MAPK1		10.1074/jbc.M116.759134
Regulation: NOTCH1 ---> MAPK1	28536843	10.1007/s00726-017-2432-3
Regulation: NOTCH1 ---> MAPK1	29769419	10.26402/jpp.2018.1.04
Regulation: NOTCH1 ---> MAPK1	29743588	10.1038/s41388-018-0251-y
Regulation: NOTCH1 ---> MAPK1	29743588	10.1038/s41388-018-0251-y
Regulation: NOTCH1 ---> MAPK1	29874112	10.1152/ajpcell.00182.2017
Regulation: NOTCH1 ---> MAPK1	30206308	10.1038/s41375-018-0248-010.1038/s41375-018-0
Regulation: GPER1 ---> TP53		10.1507/endocrj.EJ15-0571
Regulation: GPER1 ---> TP53	25275589	10.1038/cddis.2014.398
Regulation: GPER1 ---> TP53	25275589	10.1038/cddis.2014.398
Regulation: GPER1 ---> TP53	26225773	10.1038/cddis.2015.201
Regulation: GPER1 ---> TP53	28595943	10.1016/j.jsbmb.2017.05.005
Regulation: GPER1 ---> TP53	25275589	10.1038/cddis.2014.398
Regulation: GPER1 ---> TP53	25275589	10.1038/cddis.2014.398
Regulation: GPER1 ---> TP53		
negative Regulation: SPP1 ---| ch	28433634	10.1016/j.bbrc.2017.04.100
negative Regulation: SPP1 ---| ch	30535434	10.3892/ijo.2018.4656
negative Regulation: SPP1 ---| ch	21539449	10.1089/cbr.2010.0838
negative Regulation: SPP1 ---| ch	30627777	10.1007/s00280-018-3759-510.1007/s00280-018-3
negative Regulation: SPP1 ---| chemosensitivity		
Binding: BECN1 ---- TP53	30981885	10.1016/j.ejps.2019.04.011
Binding: BECN1 ---- TP53	23088850	10.1016/j.mito.2012.10.010
Binding: BECN1 ---- TP53	23088850	10.1016/j.mito.2012.10.010
Binding: BECN1 ---- TP53	30563957	10.3390/ijms18091883
Binding: BECN1 ---- TP53	30258193	10.1038/s41419-018-1016-9
Binding: BECN1 ---- TP53	33375363	10.3390/ijms22010179
Binding: BECN1 ---- TP53	23151455	10.4161/cc.22778
Binding: BECN1 ---- TP53	22356768	10.4161/cc.11.6.19670
Binding: BECN1 ---- TP53		
Binding: BECN1 ---- TP53	24554038	10.1590/1414-431X2013323110.1590/1414-431X201

Binding: BECN1 ---- TP53	25208472	10.1111/jcmm.1238610.1111/jcmm.12386
Binding: BECN1 ---- TP53	31945648	10.1016/j.jinorgbio.2019.11098910.1016/j.jinc
Binding: BECN1 ---- TP53	34335587	10.3389/fimmu.2021.68552310.3389/fimmu.2021.6
Binding: BECN1 ---- TP53		10.3390/ijms18091883
Binding: BECN1 ---- TP53	35482278	10.1007/s12264-022-00861-6
Expression: GPER1 ---> CLDN1	26731262	10.1371/journal.pone.0145212
Expression: GPER1 ---> CLDN1	30227306	10.1016/j.neo.2018.08.01010.1016/j.neo.2018.0
Expression: GPER1 ---> CLDN1		10.14670/HH-18-157
negative Expression: BECN1 ---| T	31356866	10.1016/j.semcancer.2019.07.013
negative Expression: BECN1 ---| T	32800272	10.1016/j.mrrev.2020.108321
negative Expression: BECN1 ---| T	32800272	10.1016/j.mrrev.2020.108321
negative Expression: BECN1 ---| T	24858802	10.1016/j.bcp.2014.05.009
negative Expression: BECN1 ---| T	24858802	10.1016/j.bcp.2014.05.009
negative Expression: BECN1 ---| T	27559856	10.1080/15548627.2016.1217380
negative Expression: BECN1 ---| T	29230017	10.1038/s41556-017-0007-x
negative Expression: BECN1 ---| T	29991800	10.1038/s41388-018-0395-9
negative Expression: BECN1 ---| T	30328348	10.1530/REP-18-0243
negative Expression: BECN1 ---| T	31776978	10.1007/978-981-15-0602-4_1
negative Expression: BECN1 ---| T	23486312	10.4161/auto.24132
negative Expression: BECN1 ---| T	23542691	10.1038/nn.3365
negative Expression: BECN1 ---| T	24256242	10.1042/BST20130185
negative Expression: BECN1 ---| T	25180518	10.4161/auto.34398
negative Expression: BECN1 ---| T	26530936	10.3892/ijo.2015.3227
negative Expression: BECN1 ---| T	21962518	10.1016/j.cell.2011.08.03710.1016/j.cell.2011
negative Expression: BECN1 ---| T	30539860	10.4103/0973-1482.20489810.4103/0973-1482.204
negative Expression: BECN1 ---| T	34678225	10.1016/j.bcp.2021.114797
negative Expression: BECN1 ---| T	36528652	10.1038/s41467-022-35557-y
negative Expression: BECN1 ---| T	36528652	10.1038/s41467-022-35557-y
negative Expression: BECN1 ---| T	36528652	10.1038/s41467-022-35557-y
negative Expression: BECN1 ---| T	36528652	10.1038/s41467-022-35557-y
negative Expression: BECN1 ---| T	36528652	10.1038/s41467-022-35557-y
negative Expression: BECN1 ---| T	36528652	10.1038/s41467-022-35557-y
Binding: TP53 ---- RAC1	30059710	10.1016/j.mam.2018.07.002
Binding: TP53 ---- RAC1	28947497	10.1101/gad.301564.117
Binding: TP53 ---- RAC1	28947497	10.1101/gad.301564.117
Binding: TP53 ---- RAC1	28947497	10.1101/gad.301564.117
Binding: TP53 ---- RAC1	28947497	10.1101/gad.301564.117
Binding: TP53 ---- RAC1	28947497	10.1101/gad.301564.117
Binding: TP53 ---- RAC1	28947497	10.1101/gad.301564.117
Binding: TP53 ---- RAC1	28947497	10.1101/gad.301564.117
Binding: TP53 ---- RAC1	28947497	10.1101/gad.301564.117
Binding: TP53 ---- RAC1	32275841	10.1080/15384101.2020.1749790
Binding: TP53 ---- RAC1	28947497	10.1101/gad.301564.11710.1101/gad.301564.117
Binding: TP53 ---- RAC1	28947497	10.1101/gad.301564.117
Expression: BECN1 ---> SPP1	24657887	10.1016/j.matbio.2014.03.001
Expression: BECN1 ---> SPP1	34716503	10.1007/s11033-021-06866-0
negative Expression: RAC1 ---| TP	15303276	10.1016/j.bbrc.2004.06.088
negative Expression: RAC1 ---| TP	15303276	10.1016/j.bbrc.2004.06.088
negative Expression: RAC1 ---| TP	28825724	10.1038/onc.2017.292
negative Expression: RAC1 ---| TP	23750283	10.1371/journal.pone.0066275
negative Expression: RAC1 ---| TP	23750283	10.1371/journal.pone.0066275
negative Expression: RAC1 ---| TP	23750283	10.1371/journal.pone.0066275
negative Expression: RAC1 ---| TP	23750283	10.1371/journal.pone.0066275
negative Expression: RAC1 ---| TP	23750283	10.1371/journal.pone.0066275

negative Expression: RAC1 ---| TF 23750283 10.1371/journal.pone.0066275
negative Expression: RAC1 ---| TF 23750283 10.1371/journal.pone.0066275
negative Expression: RAC1 ---| TF 23750283 10.1371/journal.pone.0066275
negative Expression: RAC1 ---| TF 23750283 10.1371/journal.pone.0066275
negative Expression: RAC1 ---| TF 16395716 10.1002/ijc.21763
negative Expression: RAC1 ---| TF 23750283 10.1371/journal.pone.006627510.1371/journal.p
negative Expression: RAC1 ---| TP53
negative Expression: RAC1 ---| TF 34765009 10.1155/2021/8428130
Regulation: TP53 ---> MAPK1 29654721 10.1016/j.ajpath.2018.03.006
Regulation: TP53 ---> MAPK1 27616194 10.1016/j.bbrc.2016.09.035
Regulation: TP53 ---> MAPK1 27616194 10.1016/j.bbrc.2016.09.035
Regulation: TP53 ---> MAPK1 27616194 10.1016/j.bbrc.2016.09.035
Regulation: TP53 ---> MAPK1 27616194 10.1016/j.bbrc.2016.09.035
Regulation: TP53 ---> MAPK1 27616194 10.1016/j.bbrc.2016.09.035
Regulation: TP53 ---> MAPK1 27616194 10.1016/j.bbrc.2016.09.035
Regulation: TP53 ---> MAPK1 30037885 10.1182/blood-2018-03-828269
Regulation: TP53 ---> MAPK1 22039050 10.1074/jbc.M111.265397
Regulation: TP53 ---> MAPK1 22039050 10.1074/jbc.M111.265397
Regulation: TP53 ---> MAPK1 15078887 10.1074/jbc.M312135200
Regulation: TP53 ---> MAPK1 15078887 10.1074/jbc.M312135200
Regulation: TP53 ---> MAPK1 15078887 10.1074/jbc.M312135200
Regulation: TP53 ---> MAPK1 15078887 10.1074/jbc.M312135200
Regulation: TP53 ---> MAPK1 15078887 10.1074/jbc.M312135200
Regulation: TP53 ---> MAPK1 11821415 10.1074/jbc.M111598200
Regulation: TP53 ---> MAPK1 29852310 10.1016/j.bioorg.2018.05.020
Regulation: TP53 ---> MAPK1 29852310 10.1016/j.bioorg.2018.05.020
Regulation: TP53 ---> MAPK1 32738450 10.1016/j.jhep.2020.07.036
Regulation: TP53 ---> MAPK1 29232553 10.1016/j.ccell.2017.11.003
Regulation: TP53 ---> MAPK1 29232553 10.1016/j.ccell.2017.11.003
Regulation: TP53 ---> MAPK1 29232553 10.1016/j.ccell.2017.11.003
Regulation: TP53 ---> MAPK1 31269434 10.1016/j.celrep.2019.06.004
Regulation: TP53 ---> MAPK1 32075732 10.1016/j.celrep.2020.01.074
Regulation: TP53 ---> MAPK1 33812333 10.1016/j.redox.2021.101924
Regulation: TP53 ---> MAPK1 19497412 10.1016/j.cbi.2009.03.012
Regulation: TP53 ---> MAPK1 17208232 10.1016/j.febslet.2006.12.035
Regulation: TP53 ---> MAPK1 17208232 10.1016/j.febslet.2006.12.035
Regulation: TP53 ---> MAPK1 17208232 10.1016/j.febslet.2006.12.035
Regulation: TP53 ---> MAPK1 17208232 10.1016/j.febslet.2006.12.035
Regulation: TP53 ---> MAPK1 18448277 10.1016/j.toxlet.2008.03.012
Regulation: TP53 ---> MAPK1 16814113 10.1016/j.freeradbiomed.2006.04.019
Regulation: TP53 ---> MAPK1 16814113 10.1016/j.freeradbiomed.2006.04.019
Regulation: TP53 ---> MAPK1 18295447 10.1016/j.cellsig.2007.12.020
Regulation: TP53 ---> MAPK1 18396012 10.1016/j.cellsig.2008.02.012
Regulation: TP53 ---> MAPK1 18440285 10.1016/j.dnarep.2008.03.003
Regulation: TP53 ---> MAPK1 22921303 10.1016/j.lfs.2012.08.012
Regulation: TP53 ---> MAPK1 22921303 10.1016/j.lfs.2012.08.012
Regulation: TP53 ---> MAPK1 22796517 10.1016/j.pharmthera.2012.07.003
Regulation: TP53 ---> MAPK1 22749133 10.1016/j.jnutbio.2012.01.012
Regulation: TP53 ---> MAPK1 23108049 10.1016/j.febslet.2012.10.031
Regulation: TP53 ---> MAPK1 23583409 10.1016/j.bbrc.2013.03.124
Regulation: TP53 ---> MAPK1 23870914 10.1016/j.foodchem.2013.05.021
Regulation: TP53 ---> MAPK1 25190107 10.1016/j.acthis.2014.08.005
Regulation: TP53 ---> MAPK1 25459512 10.1016/j.bbagen.2014.10.008
Regulation: TP53 ---> MAPK1 12639981

Regulation: TP53 ---> MAPK1	21138557	
Regulation: TP53 ---> MAPK1	19088181	
Regulation: TP53 ---> MAPK1	15078887	
Regulation: TP53 ---> MAPK1	15078887	
Regulation: TP53 ---> MAPK1	15078887	
Regulation: TP53 ---> MAPK1	15078887	
Regulation: TP53 ---> MAPK1	15078887	
Regulation: TP53 ---> MAPK1	15078887	
Regulation: TP53 ---> MAPK1	15078887	
Regulation: TP53 ---> MAPK1	15078887	
Regulation: TP53 ---> MAPK1	15078887	
Regulation: TP53 ---> MAPK1	11821415	
Regulation: TP53 ---> MAPK1	11834713	
Regulation: TP53 ---> MAPK1	10890907	
Regulation: TP53 ---> MAPK1	10890907	
Regulation: TP53 ---> MAPK1	10890907	
Regulation: TP53 ---> MAPK1	10890907	
Regulation: TP53 ---> MAPK1	10890907	
Regulation: TP53 ---> MAPK1	10890907	
Regulation: TP53 ---> MAPK1	10890907	
Regulation: TP53 ---> MAPK1	10890907	
Regulation: TP53 ---> MAPK1	10890907	
Regulation: TP53 ---> MAPK1	10890907	
Regulation: TP53 ---> MAPK1	10890907	
Regulation: TP53 ---> MAPK1	15150542	
Regulation: TP53 ---> MAPK1	15150542	
Regulation: TP53 ---> MAPK1	15150542	
Regulation: TP53 ---> MAPK1	20035047	10.2353/ajpath.2010.09022210.2353/ajpath.2010
Regulation: TP53 ---> MAPK1	22039050	10.1074/jbc.M111.26539710.1074/jbc.M111.26539
Regulation: TP53 ---> MAPK1	22039050	10.1074/jbc.M111.26539710.1074/jbc.M111.26539
Regulation: TP53 ---> MAPK1	22576881	10.1007/s11033-012-1647-310.1007/s11033-012-1
Regulation: TP53 ---> MAPK1	23814485	10.1593/neo.121862
Regulation: TP53 ---> MAPK1	25268131	10.1371/journal.pone.010681210.1371/journal.p
Regulation: TP53 ---> MAPK1		10.1016/j.bbagen.2014.10.008
Regulation: TP53 ---> MAPK1	15557124	
Regulation: TP53 ---> MAPK1	15150542	
Regulation: TP53 ---> MAPK1	15150542	
Regulation: TP53 ---> MAPK1	15150542	
Regulation: TP53 ---> MAPK1	15150542	
Regulation: TP53 ---> MAPK1	15150542	
Regulation: TP53 ---> MAPK1	15150542	
Regulation: TP53 ---> MAPK1	15150542	
Regulation: TP53 ---> MAPK1	15150542	
Regulation: TP53 ---> MAPK1	15150542	
Regulation: TP53 ---> MAPK1	15150542	
Regulation: TP53 ---> MAPK1	15150542	
Regulation: TP53 ---> MAPK1	15150542	
Regulation: TP53 ---> MAPK1	15150542	
Regulation: TP53 ---> MAPK1	12483525	
Regulation: TP53 ---> MAPK1	12411481	
Regulation: TP53 ---> MAPK1	12411481	
Regulation: TP53 ---> MAPK1	12411481	
Regulation: TP53 ---> MAPK1	17579708	
Regulation: TP53 ---> MAPK1	26970968	10.1007/s10555-016-9617-1

Regulation: TP53 ---> MAPK1	28035374	10.3892/ijo.2016.3827
Regulation: TP53 ---> MAPK1	28229963	10.1007/s12038-017-9667-8
Regulation: TP53 ---> MAPK1		10.1134/S1990519X16030056
Regulation: TP53 ---> MAPK1	28045446	10.3390/molecules22010070
Regulation: TP53 ---> MAPK1	28560457	10.3892/ijo.2017.4025
Regulation: TP53 ---> MAPK1	29061914	10.1042/BCJ20170080
Regulation: TP53 ---> MAPK1	29238070	10.1038/s41418-017-0026-y
Regulation: TP53 ---> MAPK1	32650804	10.1186/s12943-020-01223-4
Regulation: TP53 ---> MAPK1	32650804	10.1186/s12943-020-01223-4
Regulation: TP53 ---> MAPK1	32650804	10.1186/s12943-020-01223-4
Regulation: TP53 ---> MAPK1	17208232	10.1016/j.febslet.2006.12.035
Regulation: TP53 ---> MAPK1		10.1155/2019/8323592
Regulation: TP53 ---> MAPK1	22466960	10.1254/jphs.11181fp
Regulation: TP53 ---> MAPK1	15150542	
Regulation: TP53 ---> MAPK1	17208316	10.1016/j.bbamcr.2006.12.002
Regulation: TP53 ---> MAPK1		10.1016/j.earlhumdev.2011.12.021
Regulation: TP53 ---> MAPK1	21741467	10.1016/j.tiv.2011.06.013
Regulation: TP53 ---> MAPK1	16214361	10.1016/j.tem.2005.09.007
Regulation: TP53 ---> MAPK1		10.1016/j.bbcan.2014.11.001
Regulation: TP53 ---> MAPK1		10.1016/j.cytogfr.2014.09.003
Regulation: TP53 ---> MAPK1	25450826	10.1016/j.bbcan.2014.11.001
Regulation: TP53 ---> MAPK1	25438737	10.1016/j.cytogfr.2014.09.003
Regulation: TP53 ---> MAPK1	19818165	
Regulation: TP53 ---> MAPK1	19818165	
Regulation: TP53 ---> MAPK1	15557124	
Regulation: TP53 ---> MAPK1	19910580	
Regulation: TP53 ---> MAPK1	15383658	
Regulation: TP53 ---> MAPK1	16701773	10.1016/j.taap.2006.03.009
Regulation: TP53 ---> MAPK1	21741467	10.1016/j.tiv.2011.06.013
Regulation: TP53 ---> MAPK1	22884810	10.1016/j.lfs.2012.07.027
Regulation: TP53 ---> MAPK1	25128958	10.1016/j.tips.2014.07.004
Regulation: TP53 ---> MAPK1	15150542	
Regulation: TP53 ---> MAPK1	15009734	
Regulation: TP53 ---> MAPK1	17875924	
Regulation: TP53 ---> MAPK1	17875924	
Regulation: TP53 ---> MAPK1	17875924	
Regulation: TP53 ---> MAPK1	23314357	10.3892/ijo.2013.1764
Regulation: TP53 ---> MAPK1	23314357	10.3892/ijo.2013.1764
Regulation: TP53 ---> MAPK1	23314357	10.3892/ijo.2013.1764
Regulation: TP53 ---> MAPK1	23314357	10.3892/ijo.2013.1764
Regulation: TP53 ---> MAPK1	23314357	10.3892/ijo.2013.1764
Regulation: TP53 ---> MAPK1	23314357	10.3892/ijo.2013.1764
Regulation: TP53 ---> MAPK1	23314357	10.3892/ijo.2013.1764
Regulation: TP53 ---> MAPK1	19818165	10.1186/bcr2361
Regulation: TP53 ---> MAPK1	19818165	10.1186/bcr2361
Regulation: TP53 ---> MAPK1	21852236	10.1074/jbc.M110.205708
Regulation: TP53 ---> MAPK1	22087839	10.2174/092986711798194388
Regulation: TP53 ---> MAPK1	22039050	10.1074/jbc.M111.265397
Regulation: TP53 ---> MAPK1	22039050	10.1074/jbc.M111.265397
Regulation: TP53 ---> MAPK1	22039050	10.1074/jbc.M111.265397
Regulation: TP53 ---> MAPK1	22039050	10.1074/jbc.M111.265397
Regulation: TP53 ---> MAPK1	22039050	10.1074/jbc.M111.265397
Regulation: TP53 ---> MAPK1	22039050	10.1074/jbc.M111.265397
Regulation: TP53 ---> MAPK1	22039050	10.1074/jbc.M111.265397

Regulation: TP53 ---> MAPK1	22336915	10.4161/cc.11.5.19452
Regulation: TP53 ---> MAPK1	21138557	10.1186/1476-4598-9-311
Regulation: TP53 ---> MAPK1	21468663	10.1007/s10495-011-0597-x
Regulation: TP53 ---> MAPK1	21107428	10.1038/nature09535
Regulation: TP53 ---> MAPK1	24114360	10.1007/s10495-013-0913-8
Regulation: TP53 ---> MAPK1	24504121	10.1371/journal.pone.0087229
Regulation: TP53 ---> MAPK1	24329111	10.2174/1389200214666131211155613
Regulation: TP53 ---> MAPK1	24867259	10.3892/ijo.2014.2463
Regulation: TP53 ---> MAPK1	24867259	10.3892/ijo.2014.2463
Regulation: TP53 ---> MAPK1	23873029	10.1038/onc.2013.287
Regulation: TP53 ---> MAPK1	25268131	10.1371/journal.pone.0106812
Regulation: TP53 ---> MAPK1		10.1074/jbc.M114.590943
Regulation: TP53 ---> MAPK1		10.1074/jbc.M114.590943
Regulation: TP53 ---> MAPK1		10.1074/jbc.M114.590943
Regulation: TP53 ---> MAPK1		10.1074/jbc.M114.590943
Regulation: TP53 ---> MAPK1		10.1074/jbc.M114.590943
Regulation: TP53 ---> MAPK1	22740414	10.1128/JVI.06279-11
Regulation: TP53 ---> MAPK1	22740414	10.1128/JVI.06279-11
Regulation: TP53 ---> MAPK1	25815718	10.1371/journal.pone.0119366
Regulation: TP53 ---> MAPK1	25815718	10.1371/journal.pone.0119366
Regulation: TP53 ---> MAPK1	25815718	10.1371/journal.pone.0119366
Regulation: TP53 ---> MAPK1	15150542	10.1038/sj.cdd.4401368
Regulation: TP53 ---> MAPK1	15150542	10.1038/sj.cdd.4401368
Regulation: TP53 ---> MAPK1	15150542	10.1038/sj.cdd.4401368
Regulation: TP53 ---> MAPK1	15078887	
Regulation: TP53 ---> MAPK1	15880691	
Regulation: TP53 ---> MAPK1	15880691	
Regulation: TP53 ---> MAPK1	17132229	
Regulation: TP53 ---> MAPK1	17215959	
Regulation: TP53 ---> MAPK1	17208232	
Regulation: TP53 ---> MAPK1	19910580	10.1161/CIRCRESAHA.109.195834
Regulation: TP53 ---> MAPK1	22039050	10.1074/jbc.M111.265397
Regulation: TP53 ---> MAPK1	23108049	10.1016/j.febslet.2012.10.031
Regulation: TP53 ---> MAPK1	23314357	10.3892/ijo.2013.1764
Regulation: TP53 ---> MAPK1	23559009	10.1038/cddis.2013.97
Regulation: TP53 ---> MAPK1	24486524	10.1016/j.bcp.2014.01.025
Regulation: TP53 ---> MAPK1	25936182	
Regulation: TP53 ---> MAPK1		
Regulation: TP53 ---> MAPK1		10.1007/s11055-016-0342-6
Regulation: TP53 ---> MAPK1		10.1007/s11055-016-0342-6
Regulation: TP53 ---> MAPK1	28573231	10.21010/ajtcam.v14i2.1610.21010/ajtcam.v14i2
Regulation: TP53 ---> MAPK1	208	10.1016/j.bbrc.2017.12.085
Regulation: TP53 ---> MAPK1	29852310	10.1016/j.bioorg.2018.05.02010.1016/j.bioorg.
Regulation: TP53 ---> MAPK1	29298131	10.1080/13510002.2017.1416773
Regulation: TP53 ---> MAPK1	28669336	10.2174/1568009617666170630124643
Regulation: TP53 ---> MAPK1		10.3390/ijms19123898
Regulation: TP53 ---> MAPK1		10.3892/or.2019.6964
Regulation: TP53 ---> MAPK1	31670863	10.1002/1878-0261.1259310.1002/1878-0261.1259
Regulation: TP53 ---> MAPK1	34947982	10.3390/ijms222413187
Regulation: TP53 ---> MAPK1	34749615	10.2174/1568026621666211105093658
Regulation: TP53 ---> MAPK1		10.55782/ane-2022-030
Regulation: TP53 ---> MAPK1	36589747	10.3389/fcell.2022.1049566
negative Regulation: TP53 ---| RA	30562482	10.1016/j.yexcr.2018.12.011
negative Regulation: TP53 ---| RA	12750388	

negative Regulation: TP53 ---| RA 12578823
negative Regulation: TP53 ---| RA 12578823
negative Regulation: TP53 ---| RA 18413607
negative Regulation: TP53 ---| RA 14729984
negative Regulation: TP53 ---| RA 14729984
negative Regulation: TP53 ---| RA 14729984
negative Regulation: TP53 ---| RA 14729984
negative Regulation: TP53 ---| RA 28947497 10.1101/gad.301564.117
negative Regulation: TP53 ---| RA 28947497 10.1101/gad.301564.117
negative Regulation: TP53 ---| RA 28947497 10.1101/gad.301564.117
negative Regulation: TP53 ---| RA 28947497 10.1101/gad.301564.117
negative Regulation: TP53 ---| RA 28947497 10.1101/gad.301564.117
negative Regulation: TP53 ---| RA 28947497 10.1101/gad.301564.117
negative Regulation: TP53 ---| RA 28947497 10.1101/gad.301564.117
negative Regulation: TP53 ---| RA 28947497 10.1101/gad.301564.117
negative Regulation: TP53 ---| RA 28947497 10.1101/gad.301564.117
negative Regulation: TP53 ---| RA 28947497 10.1101/gad.301564.117
negative Regulation: TP53 ---| RA 28947497 10.1101/gad.301564.117
negative Regulation: TP53 ---| RA 28947497 10.1101/gad.301564.117
negative Regulation: TP53 ---| RA 28947497 10.1101/gad.301564.117
negative Regulation: TP53 ---| RA 28947497 10.1101/gad.301564.117
negative Regulation: TP53 ---| RA 28947497 10.1101/gad.301564.117
negative Regulation: TP53 ---| RA 28947497 10.1101/gad.301564.117
negative Regulation: TP53 ---| RA 28947497 10.1101/gad.301564.117
negative Regulation: TP53 ---| RA 28947497 10.1101/gad.301564.117
negative Regulation: TP53 ---| RA 28947497 10.1101/gad.301564.117
negative Regulation: TP53 ---| RA 28947497 10.1101/gad.301564.117
negative Regulation: TP53 ---| RA 28947497 10.1101/gad.301564.117
negative Regulation: TP53 ---| RA 28947497 10.1101/gad.301564.117
negative Regulation: TP53 ---| RA 28947497 10.1101/gad.301564.117
negative Regulation: TP53 ---| RA 28947497 10.1101/gad.301564.117
negative Regulation: TP53 ---| RA 28947497 10.1101/gad.301564.117
negative Regulation: TP53 ---| RA 28869598 10.1038/onc.2017.306
negative Regulation: TP53 ---| RA 28869598 10.1038/onc.2017.306
negative Regulation: TP53 ---| RA 28869598 10.1038/onc.2017.306
negative Regulation: TP53 ---| RA 28869598 10.1038/onc.2017.306
negative Regulation: TP53 ---| RA 28869598 10.1038/onc.2017.306
negative Regulation: TP53 ---| RA 31726940 10.1080/15384101.2019.1688951
negative Regulation: TP53 ---| RA 31726940 10.1080/15384101.2019.1688951
negative Regulation: TP53 ---| RA 32128961 10.15252/embr.201949269
negative Regulation: TP53 ---| RA 32275841 10.1080/15384101.2020.1749790
negative Regulation: TP53 ---| RA 32275841 10.1080/15384101.2020.1749790
negative Regulation: TP53 ---| RA 32275841 10.1080/15384101.2020.1749790
negative Regulation: TP53 ---| RA 32275841 10.1080/15384101.2020.1749790
negative Regulation: TP53 ---| RA 32275841 10.1080/15384101.2020.1749790
negative Regulation: TP53 ---| RA 32275841 10.1080/15384101.2020.1749790
negative Regulation: TP53 ---| RA 32275841 10.1080/15384101.2020.1749790
negative Regulation: TP53 ---| RA 32275841 10.1080/15384101.2020.1749790
negative Regulation: TP53 ---| RA 32275841 10.1080/15384101.2020.1749790
negative Regulation: TP53 ---| RA 32275841 10.1080/15384101.2020.1749790
negative Regulation: TP53 ---| RA 32275841 10.1080/15384101.2020.1749790
negative Regulation: TP53 ---| RA 32275841 10.1080/15384101.2020.1749790
negative Regulation: TP53 ---| RA 32275841 10.1080/15384101.2020.1749790
negative Regulation: TP53 ---| RA 32275841 10.1080/15384101.2020.1749790

negative Regulation: TP53 ---| RA	32275841	10.1080/15384101.2020.1749790
negative Regulation: TP53 ---| RA	32275841	10.1080/15384101.2020.1749790
negative Regulation: TP53 ---| RA	32275841	10.1080/15384101.2020.1749790
negative Regulation: TP53 ---| RA	32275841	10.1080/15384101.2020.1749790
negative Regulation: TP53 ---| RA	22745590	10.1593/neo.12352
negative Regulation: TP53 ---| RA	22888789	10.1186/1476-4598-11-54
negative Regulation: TP53 ---| RA	21391906	
negative Regulation: TP53 ---| RA	26036474	10.1124/jpet.115.223610
negative Regulation: TP53 ---| RA	12578823	10.1074/jbc.M300341200
negative Regulation: TP53 ---| RA	28947497	10.1101/gad.301564.11710.1101/gad.301564.117
negative Regulation: TP53 ---| RA	34183772	10.1038/s41388-021-01921-3
negative Regulation: TP53 ---| RA	34183772	10.1038/s41388-021-01921-3
negative Regulation: TP53 ---| RA	34161151	10.1152/ajpcell.00142.2021
negative Regulation: TP53 ---| RA	91	10.1016/j.yexcr.2018.12.011
negative Regulation: TP53 ---| RAC1		10.1080/15384101.2019.1688951
negative Regulation: TP53 ---| RAC1		10.1080/15384101.2019.1688951
negative Regulation: TP53 ---| RAC1		10.1186/1476-4598-11-54
negative Regulation: TP53 ---| RA	34541899	10.1152/physrev.00045.2020
Expression: MAPK1 ---> TP53	27616194	10.1016/j.bbrc.2016.09.035
Expression: MAPK1 ---> TP53	27616194	10.1016/j.bbrc.2016.09.035
Expression: MAPK1 ---> TP53	26324703	10.1182/blood-2015-04-639138
Expression: MAPK1 ---> TP53	32315388	10.1182/blood.2019003538
Expression: MAPK1 ---> TP53	29477545	10.1016/j.brainres.2018.02.023
Expression: MAPK1 ---> TP53	33275947	10.1016/j.yexmp.2020.104587
Expression: MAPK1 ---> TP53	17244621	10.1074/jbc.M605649200
Expression: MAPK1 ---> TP53	21147777	10.1074/jbc.M110.175174
Expression: MAPK1 ---> TP53	28202289	10.1016/j.lfs.2017.02.005
Expression: MAPK1 ---> TP53	28558962	10.1016/j.taap.2017.05.034
Expression: MAPK1 ---> TP53	32660739	10.1016/j.blre.2020.100712
Expression: MAPK1 ---> TP53	29673545	10.1016/j.nutres.2017.12.011
Expression: MAPK1 ---> TP53	31201898	10.1016/j.fct.2019.05.052
Expression: MAPK1 ---> TP53	27461822	10.1016/j.prp.2016.06.005
Expression: MAPK1 ---> TP53	27638054	10.1016/j.tiv.2016.09.011
Expression: MAPK1 ---> TP53	31866342	10.1016/j.mcp.2019.101496
Expression: MAPK1 ---> TP53		10.1016/j.procbio.2018.12.010
Expression: MAPK1 ---> TP53	33187910	10.1016/j.intimp.2020.107158
Expression: MAPK1 ---> TP53	32075732	10.1016/j.celrep.2020.01.074
Expression: MAPK1 ---> TP53	33997178	10.1016/j.gendis.2019.12.009
Expression: MAPK1 ---> TP53	28844411	10.1016/j.ebiom.2017.08.018
Expression: MAPK1 ---> TP53	33997178	10.1016/j.gendis.2019.12.009
Expression: MAPK1 ---> TP53	20450891	10.1016/j.bcp.2010.04.029
Expression: MAPK1 ---> TP53	18272192	10.1016/j.taap.2007.12.028
Expression: MAPK1 ---> TP53	18272192	10.1016/j.taap.2007.12.028
Expression: MAPK1 ---> TP53	17337084	10.1016/j.lungcan.2007.01.027
Expression: MAPK1 ---> TP53	22252116	10.1016/j.canlet.2012.01.001
Expression: MAPK1 ---> TP53	15337531	10.1016/j.cellsig.2004.04.003
Expression: MAPK1 ---> TP53	16527552	10.1016/j.dld.2006.01.012
Expression: MAPK1 ---> TP53	22884810	10.1016/j.lfs.2012.07.027
Expression: MAPK1 ---> TP53	23603476	10.1016/j.tiv.2013.04.006
Expression: MAPK1 ---> TP53	23827354	10.1016/j.steroids.2013.06.005
Expression: MAPK1 ---> TP53	24094550	10.1016/j.ijcard.2013.08.076
Expression: MAPK1 ---> TP53	24094550	10.1016/j.ijcard.2013.08.076
Expression: MAPK1 ---> TP53	24035753	10.1016/j.toxlet.2013.09.002
Expression: MAPK1 ---> TP53	24548678	10.1016/j.toxlet.2014.02.006

Expression: MAPK1 ---> TP53	24792472	10.1016/j.jphotobiol.2014.03.015
Expression: MAPK1 ---> TP53	25043688	10.1016/j.biocel.2014.07.006
Expression: MAPK1 ---> TP53	25652123	10.1016/j.bbadis.2015.01.014
Expression: MAPK1 ---> TP53	26915871	10.1016/j.transproceed.2016.01.009
Expression: MAPK1 ---> TP53	21138557	
Expression: MAPK1 ---> TP53	21138557	
Expression: MAPK1 ---> TP53	17244621	
Expression: MAPK1 ---> TP53	16000378	
Expression: MAPK1 ---> TP53	15723837	
Expression: MAPK1 ---> TP53	10958792	
Expression: MAPK1 ---> TP53	12444545	
Expression: MAPK1 ---> TP53	11420662	
Expression: MAPK1 ---> TP53	11420662	
Expression: MAPK1 ---> TP53	16507997	
Expression: MAPK1 ---> TP53		10.14670/HH-11-88
Expression: MAPK1 ---> TP53	28587524	10.1089/ars.2017.7167
Expression: MAPK1 ---> TP53	29119536	10.1007/s12035-017-0814-x
Expression: MAPK1 ---> TP53	30208932	10.1186/s13058-018-1028-5
Expression: MAPK1 ---> TP53	30534001	10.1186/s12935-018-0692-y
Expression: MAPK1 ---> TP53	30538214	10.12659/MSM.911271
Expression: MAPK1 ---> TP53	30538214	10.12659/MSM.911271
Expression: MAPK1 ---> TP53	30538214	10.12659/MSM.911271
Expression: MAPK1 ---> TP53	30651129	10.1186/s13058-018-1071-2
Expression: MAPK1 ---> TP53	30569130	10.3892/or.2018.6926
Expression: MAPK1 ---> TP53	32003661	10.2174/1381612826666200128091506
Expression: MAPK1 ---> TP53	33562110	10.3390/ijms22041653
Expression: MAPK1 ---> TP53	22466960	10.1254/jphs.11181FP
Expression: MAPK1 ---> TP53	22843228	10.1007/s10456-012-9289-6
Expression: MAPK1 ---> TP53	22843228	10.1007/s10456-012-9289-6
Expression: MAPK1 ---> TP53	22843228	10.1007/s10456-012-9289-6
Expression: MAPK1 ---> TP53	21239614	10.1210/me.2010-0424
Expression: MAPK1 ---> TP53	21239614	10.1210/me.2010-0424
Expression: MAPK1 ---> TP53		10.2174/1381612826666200128091506
Expression: MAPK1 ---> TP53	1050	10.1016/j.ebiom.2017.08.018
Expression: MAPK1 ---> TP53		10.1016/j.mcp.2019.101496
Expression: MAPK1 ---> TP53		10.1016/j.gendis.2019.12.009
Expression: MAPK1 ---> TP53		10.1186/s13058-018-1071-2
Expression: MAPK1 ---> TP53		10.3892/or.2018.6926
Expression: MAPK1 ---> TP53	21239614	10.1210/me.2010-0424
Expression: MAPK1 ---> TP53	21239614	10.1210/me.2010-0424
Expression: MAPK1 ---> TP53	21147777	10.1074/jbc.M110.175174
Expression: MAPK1 ---> TP53	21964832	10.3892/or.2011.1480
Expression: MAPK1 ---> TP53	21138557	10.1186/1476-4598-9-311
Expression: MAPK1 ---> TP53	21138557	10.1186/1476-4598-9-311
Expression: MAPK1 ---> TP53	23844043	10.1371/journal.pone.0067632
Expression: MAPK1 ---> TP53	24114360	10.1007/s10495-013-0913-8
Expression: MAPK1 ---> TP53	24866016	10.1371/journal.pone.0098113
Expression: MAPK1 ---> TP53	24866016	10.1371/journal.pone.0098113
Expression: MAPK1 ---> TP53	24806432	10.3892/ijo.2014.2420
Expression: MAPK1 ---> TP53	24806432	10.3892/ijo.2014.2420
Expression: MAPK1 ---> TP53	24806432	10.3892/ijo.2014.2420
Expression: MAPK1 ---> TP53	25353185	10.1210/en.2014-1629
Expression: MAPK1 ---> TP53	23959801	10.1128/MCB.00021-13
Expression: MAPK1 ---> TP53	25962735	10.3892/or.2015.3923

Expression: MAPK1 ---> TP53	27196001	10.1371/journal.pone.0155893
Expression: MAPK1 ---> TP53	10547071	
Expression: MAPK1 ---> TP53	11420662	10.1038/sj.onc.1204353
Expression: MAPK1 ---> TP53	15723837	10.1074/jbc.M412334200
Expression: MAPK1 ---> TP53	15899123	
Expression: MAPK1 ---> TP53	20564491	10.1002/ptr.321410.1002/ptr.3214
Expression: MAPK1 ---> TP53	22843228	10.1007/s10456-012-9289-610.1007/s10456-012-9
Expression: MAPK1 ---> TP53	25146923	10.1038/cdd.2014.116
Expression: MAPK1 ---> TP53	21239614	10.1210/me.2010-0424
Expression: MAPK1 ---> TP53	21239614	10.1210/me.2010-042410.1210/me.2010-0424
Expression: MAPK1 ---> TP53	21239614	10.1210/me.2010-042410.1210/me.2010-0424
Expression: MAPK1 ---> TP53	27703489	10.1155/2016/6093783
Expression: MAPK1 ---> TP53	18154926	10.1016/j.brainres.2007.11.016
Expression: MAPK1 ---> TP53	23538210	10.1016/j.peptides.2013.03.023
Expression: MAPK1 ---> TP53	9417035	
Expression: MAPK1 ---> TP53	21340650	10.1007/s00011-011-0316-7
Expression: MAPK1 ---> TP53	24078446	10.1007/s13277-013-1193-1
Expression: MAPK1 ---> TP53	25932965	10.1371/journal.pone.0125903
Expression: MAPK1 ---> TP53	20138958	10.1016/j.mce.2010.02.004
Expression: MAPK1 ---> TP53	23538210	10.1016/j.peptides.2013.03.023
Expression: MAPK1 ---> TP53	15150542	
Expression: MAPK1 ---> TP53	21239614	10.1210/me.2010-0424
Expression: MAPK1 ---> TP53	22020547	10.3892/or.2011.1511
Expression: MAPK1 ---> TP53	25117830	10.1089/scd.2014.0058
Expression: MAPK1 ---> TP53	15899123	
Expression: MAPK1 ---> TP53	24469921	10.1007/s11481-014-9523-7
Expression: MAPK1 ---> TP53		
Expression: MAPK1 ---> TP53	29097252	10.1016/j.jep.2017.10.02610.1016/j.jep.2017.1
Expression: MAPK1 ---> TP53	29596872	10.1016/j.cellsig.2018.03.01310.1016/j.cellsi
Expression: MAPK1 ---> TP53	30226561	10.3892/or.2018.6703
Expression: MAPK1 ---> TP53		10.1016/j.intimp.2020.107158
Expression: MAPK1 ---> TP53	33402084	10.2174/1389557521666210105113831
Expression: MAPK1 ---> TP53	34992144	10.1158/1541-7786.MCR-21-014910.1158/1541-778
Expression: MAPK1 ---> TP53	35764642	10.1038/s41467-022-31418-w
Expression: MAPK1 ---> TP53	36165020	10.1134/S0026893322050089
Expression: MAPK1 ---> TP53	36005738	10.1080/15384101.2022.2108117
ProtModification: MAPK1 ---> TP5	29981726	10.1016/j.cbi.2018.07.005
ProtModification: MAPK1 ---> TP5	11409876	10.1006/bbrc.2001.5043
ProtModification: MAPK1 ---> TP5	16330547	10.1074/jbc.M507951200
ProtModification: MAPK1 ---> TP5	10781582	10.1074/jbc.M001020200
ProtModification: MAPK1 ---> TP5	11546806	10.1074/jbc.M105669200
ProtModification: MAPK1 ---> TP5	21147777	10.1074/jbc.M110.175174
ProtModification: MAPK1 ---> TP5	12890671	10.1074/jbc.M307149200
ProtModification: MAPK1 ---> TP5	11821415	10.1074/jbc.M111598200
ProtModification: MAPK1 ---> TP5	11689560	10.1074/jbc.M107231200
ProtModification: MAPK1 ---> TP5	10958792	10.1074/jbc.M004267200
ProtModification: MAPK1 ---> TP5	10958792	10.1074/jbc.M004267200
ProtModification: MAPK1 ---> TP5	10958792	10.1074/jbc.M004267200
ProtModification: MAPK1 ---> TP5	10958792	10.1074/jbc.M004267200
ProtModification: MAPK1 ---> TP5	33141030	10.1016/j.freeradbiomed.2020.10.304
ProtModification: MAPK1 ---> TP5	33246174	10.1016/j.phrs.2020.105307
ProtModification: MAPK1 ---> TP5	12510016	10.1016/S1383-5742(02)00069-8
ProtModification: MAPK1 ---> TP5	27776972	10.1016/j.jbior.2016.10.001
ProtModification: MAPK1 ---> TP5	21679689	10.1016/j.bbrc.2011.06.016

ProtModification: MAPK1 ---> TP53 11996883 10.1016/S0006-2952(02)00908-5
ProtModification: MAPK1 ---> TP53 15476743 10.1016/j.ejphar.2004.09.006
ProtModification: MAPK1 ---> TP53 15212949 10.1016/j.yexcr.2004.03.033
ProtModification: MAPK1 ---> TP53 15212949 10.1016/j.yexcr.2004.03.033
ProtModification: MAPK1 ---> TP53 15212949 10.1016/j.yexcr.2004.03.033
ProtModification: MAPK1 ---> TP53 20353787 10.1016/j.febslet.2010.03.040
ProtModification: MAPK1 ---> TP53 10430472 10.1016/S0306-4522(99)00137-2
ProtModification: MAPK1 ---> TP53 22627294 10.1016/j.tox.2012.05.013
ProtModification: MAPK1 ---> TP53 22749133 10.1016/j.jnutbio.2012.01.012
ProtModification: MAPK1 ---> TP53 23206596 10.1016/B978-0-12-398309-1.00005-6
ProtModification: MAPK1 ---> TP53 25058905 10.1016/j.ejphar.2014.07.025
ProtModification: MAPK1 ---> TP53 26751246 10.1016/j.exphem.2015.12.008
ProtModification: MAPK1 ---> TP53 18847491
ProtModification: MAPK1 ---> TP53 16330547
ProtModification: MAPK1 ---> TP53 15899039
ProtModification: MAPK1 ---> TP53 12890671 10.1074/jbc.M307149200
ProtModification: MAPK1 ---> TP53 11821415
ProtModification: MAPK1 ---> TP53 11689560
ProtModification: MAPK1 ---> TP53 11546806 10.1074/jbc.M105669200
ProtModification: MAPK1 ---> TP53 10958792
ProtModification: MAPK1 ---> TP53 10958792
ProtModification: MAPK1 ---> TP53 10958792
ProtModification: MAPK1 ---> TP53 10958792
ProtModification: MAPK1 ---> TP53 10958792
ProtModification: MAPK1 ---> TP53 10958792
ProtModification: MAPK1 ---> TP53 10958792 10.1074/jbc.M004267200
ProtModification: MAPK1 ---> TP53 10958792
ProtModification: MAPK1 ---> TP53 10781582
ProtModification: MAPK1 ---> TP53 10212189
ProtModification: MAPK1 ---> TP53 15750624
ProtModification: MAPK1 ---> TP53 11896587
ProtModification: MAPK1 ---> TP53 11426644
ProtModification: MAPK1 ---> TP53 11314025
ProtModification: MAPK1 ---> TP53 10602521
ProtModification: MAPK1 ---> TP53 10602521
ProtModification: MAPK1 ---> TP53 10.1007/s11055-016-0277-y
ProtModification: MAPK1 ---> TP53 26497030 10.1007/s12035-015-9490-x
ProtModification: MAPK1 ---> TP53 28533220 10.1128/MCB.00149-17
ProtModification: MAPK1 ---> TP53 10.14670/HH-11-88
ProtModification: MAPK1 ---> TP53 29405768 10.1177/0960327118755255
ProtModification: MAPK1 ---> TP53 32641480 10.1128/JVI.00722-20
ProtModification: MAPK1 ---> TP53 20664969 10.3892/or-00000903
ProtModification: MAPK1 ---> TP53 20818156 10.4161/cc.9.17.12799
ProtModification: MAPK1 ---> TP53 20085492 10.1089/ars.2009.2779
ProtModification: MAPK1 ---> TP53 21147777 10.1074/jbc.M110.175174
ProtModification: MAPK1 ---> TP53 22087839 10.2174/092986711798194388
ProtModification: MAPK1 ---> TP53 23638878 10.1186/1476-4598-12-35
ProtModification: MAPK1 ---> TP53 21901106 10.1371/journal.pgen.1002238
ProtModification: MAPK1 ---> TP53 24806680 10.1523/JNEUROSCI.5119-13.2014
ProtModification: MAPK1 ---> TP53 26408703
ProtModification: MAPK1 ---> TP53 10602521 10.1038/sj.onc.1203137
ProtModification: MAPK1 ---> TP53 11409876 10.1006/bbrc.2001.5043
ProtModification: MAPK1 ---> TP53 11409876 10.1006/bbrc.2001.5043
ProtModification: MAPK1 ---> TP53 11409876 10.1006/bbrc.2001.5043

ProtModification: MAPK1 ---> TP5 11409876 10.1006/bbrc.2001.5043
ProtModification: MAPK1 ---> TP5 15116093 10.1038/sj.onc.1207426
ProtModification: MAPK1 ---> TP5 28533220 10.1128/MCB.00149-1710.1128/MCB.00149-17
ProtModification: MAPK1 ---> TP5 10958792 10.1074/jbc.M004267200
ProtModification: MAPK1 ---> TP5 11821415 10.1074/jbc.M111598200
ProtModification: MAPK1 ---> TP5 11546806 10.1074/jbc.M105669200
ProtModification: MAPK1 ---> TP5 14734651 10.1242/jcs.00897
ProtModification: MAPK1 ---> TP5 15078887 10.1074/jbc.M312135200
ProtModification: MAPK1 ---> TP5 10781582 10.1074/jbc.M001020200
ProtModification: MAPK1 ---> TP5 12890671 10.1074/jbc.M307149200
ProtModification: MAPK1 ---> TP5 15116093 10.1038/sj.onc.1207426
ProtModification: MAPK1 ---> TP5 10547071
ProtModification: MAPK1 ---> TP53 10.1128/MCB.00149-17
ProtModification: MAPK1 ---> TP53
ProtModification: MAPK1 ---> TP5 11406180 10.1016/S0027-5107(01)00137-3
ProtModification: MAPK1 ---> TP53 10.1128/MCB.00149-17
ProtModification: MAPK1 ---> TP5 16970927 10.1016/j.bcp.2006.08.012
ProtModification: MAPK1 ---> TP5 19914607 10.1016/j.cellimm.2009.10.008
ProtModification: MAPK1 ---> TP5 19914607 10.1016/j.cellimm.2009.10.008
ProtModification: MAPK1 ---> TP5 21762708 10.1016/j.mrfmmm.2011.06.011
ProtModification: MAPK1 ---> TP5 15862828 10.1016/j.steroids.2005.03.001
ProtModification: MAPK1 ---> TP5 21262251 10.1016/j.taap.2011.01.011
ProtModification: MAPK1 ---> TP5 21262251 10.1016/j.taap.2011.01.011
ProtModification: MAPK1 ---> TP5 20036733 10.1016/j.freeradbiomed.2009.12.014
ProtModification: MAPK1 ---> TP5 10781582
ProtModification: MAPK1 ---> TP5 10781582
ProtModification: MAPK1 ---> TP5 10781582
ProtModification: MAPK1 ---> TP5 10781582
ProtModification: MAPK1 ---> TP53 10.1007/s11055-016-0277-y
ProtModification: MAPK1 ---> TP5 24944268 10.1152/ajprenal.00127.2014
ProtModification: MAPK1 ---> TP5 10781582 10.1074/jbc.M001020200
ProtModification: MAPK1 ---> TP5 27776972 10.1016/j.jbior.2016.10.001
ProtModification: MAPK1 ---> TP5 10547071
ProtModification: MAPK1 ---> TP5 11821415 10.1074/jbc.M111598200
ProtModification: MAPK1 ---> TP53
ProtModification: MAPK1 ---> TP5 14734651 10.1242/jcs.00897
ProtModification: MAPK1 ---> TP5 15078887 10.1074/jbc.M312135200
ProtModification: MAPK1 ---> TP5 21860418 10.1038/onc.2011.348
ProtModification: MAPK1 ---> TP53 10.1128/JVI.00722-20
ProtModification: MAPK1 ---> TP5 35477123 10.18632/aging.204038
ProtModification: MAPK1 ---> TP5 35807258 10.3390/molecules27134014
positive Expression: GPER1 --+> N 18262661 10.1016/j.tips.2008.01.001
positive Expression: GPER1 --+> N 19464786 10.1016/j.mce.2009.03.026
positive Expression: GPER1 --+> N 23545157 10.1016/j.mce.2013.03.018
positive Expression: GPER1 --+> N 14632195
positive Expression: GPER1 --+> N 24566361 10.1515/revneuro-2013-0055
positive Expression: GPER1 --+> N 22645130 10.1074/jbc.M112.341867
positive Expression: GPER1 --+> N 24859647 10.1016/j.mce.2014.05.008
positive Expression: GPER1 --+> N 19442754 10.1016/j.prostaglandins.2009.05.001
positive Expression: GPER1 --+> N 22645130 10.1074/jbc.M112.341867
positive Expression: GPER1 --+> NGF
MolTransport: GPER1 ---> NGF 14632195
MolTransport: GPER1 ---> NGF 14632195
negative Expression: PTPRD ---| (35117921 10.21037/tcr-20-242510.21037/tcr-20-2425

negative Expression: PTPRD ---| (	35117921	10.21037/tcr-20-242510.21037/tcr-20-2425
negative Expression: PTPRD ---| (	35117921	10.21037/tcr-20-242510.21037/tcr-20-2425
negative Expression: PTPRD ---| (	35117921	10.21037/tcr-20-242510.21037/tcr-20-2425
positive Expression: MAPK1 --+> M	1246	10.1016/j.apsb.2017.06.006
positive Expression: MAPK1 --+> M	21241747	10.1016/j.neures.2010.12.016
positive Expression: MAPK1 --+> M	17651887	10.1016/j.tox.2007.05.029
positive Expression: MAPK1 --+> M	23454433	10.1016/j.brainres.2013.02.026
positive Expression: MAPK1 --+> M	12925217	
positive Expression: MAPK1 --+> M	17942717	
positive Expression: MAPK1 --+> M	23182412	10.1017/S0007114512004710
positive Expression: MAPK1 --+> M	23207416	10.1159/000345043
positive Expression: MAPK1 --+> M	23000062	10.1016/j.neures.2012.09.001
positive Expression: MAPK1 --+> M	25957627	10.1007/s10266-015-0206-5
positive Expression: MAPK1 --+> M	29532850	10.3892/ijo.2018.4311
positive Expression: MAPK1 --+> NGF		10.2478/s11756-018-0099-1
positive Expression: MAPK1 --+> NGF		10.2478/s11756-018-0099-1
positive Expression: UCA1 --+> SF	33829718	10.18388/abp.2020_5448
positive Expression: UCA1 --+> SF	33829718	10.18388/abp.2020_5448
positive Expression: UCA1 --+> SF	33829718	10.18388/abp.2020_5448
positive Expression: UCA1 --+> SF	33829718	10.18388/abp.2020_5448
positive Expression: UCA1 --+> SF	33829718	10.18388/abp.2020_5448
positive Expression: UCA1 --+> SF	33829718	10.18388/abp.2020_5448
positive Expression: UCA1 --+> SF	33829718	10.18388/abp.2020_5448
negative Regulation: B3GNT3 ---|	31968179	10.1089/dna.2019.5089
positive Expression: PTGS2 --+> M	14530048	10.1016/S0003-4975(03)00334-5
positive Expression: PTGS2 --+> M	16083714	10.1053/j.gastro.2005.06.003
positive Expression: PTGS2 --+> M	18722373	10.1053/j.gastro.2008.07.012
positive Expression: PTGS2 --+> M	19933775	
positive Expression: PTGS2 --+> M	11585835	
positive Expression: PTGS2 --+> M	11585835	
positive Expression: PTGS2 --+> M	16528365	
positive Expression: PTGS2 --+> M	26302803	10.2174/1389450116666150825113035
positive Expression: PTGS2 --+> M	29928066	10.1371/journal.ppat.1007100
positive Expression: PTGS2 --+> M	23393346	
positive Expression: PTGS2 --+> M	11585835	10.1074/jbc.M107829200
positive Expression: PTGS2 --+> M	11585835	10.1074/jbc.M107829200
positive Expression: PTGS2 --+> M	16000874	10.1038/emm.2005.27
positive Expression: PTGS2 --+> M	23393346	
positive Expression: PTGS2 --+> M	24156425	10.7534/j.issn.1009-2137.2013.04.014
positive Expression: PTGS2 --+> MCL1		10.2174/1389450116666150825113035
positive Expression: PTGS2 --+> M	21525012	10.1074/jbc.M110.207571
positive Expression: PTGS2 --+> M	16000874	
positive Expression: PTGS2 --+> MCL1		
positive Expression: AKT1 --+> PF	18423396	10.1016/j.bbrc.2008.04.029
positive Expression: AKT1 --+> PF	19385085	10.1016/j.lfs.2009.01.018
positive Expression: AKT1 --+> PF	19385085	10.1016/j.lfs.2009.01.018
positive Expression: AKT1 --+> PF	21315734	10.1016/j.lfs.2011.02.001
positive Expression: AKT1 --+> PPARG		10.1016/S0083-6729(03)01005-7
positive Expression: AKT1 --+> PF	21333731	10.1016/j.tiv.2011.02.004
positive Expression: AKT1 --+> PF	21840191	10.1016/j.jnutbio.2011.03.021
positive Expression: AKT1 --+> PF	21840191	10.1016/j.jnutbio.2011.03.021
positive Expression: AKT1 --+> PPARG		10.1016/j.biocel.2012.03.019
positive Expression: AKT1 --+> PPARG		10.1016/j.biocel.2012.03.019
positive Expression: AKT1 --+> PF	19782949	10.1016/j.biocel.2009.04.004

positive Expression: AKT1 --+> PF	22497928	10.1016/j.biocel.2012.03.019
positive Expression: AKT1 --+> PF	22497928	10.1016/j.biocel.2012.03.019
positive Expression: AKT1 --+> PF	25283330	10.1016/j.jnutbio.2014.07.007
positive Expression: AKT1 --+> PF	26615875	10.1016/j.bbalip.2015.11.006
positive Expression: AKT1 --+> PF	26940012	10.1016/j.yexcr.2016.02.019
positive Expression: AKT1 --+> PF	27117918	10.1016/j.fct.2016.04.017
positive Expression: AKT1 --+> PF	17540722	
positive Expression: AKT1 --+> PF	17540722	
positive Expression: AKT1 --+> PF	17540722	
positive Expression: AKT1 --+> PF	17540722	
positive Expression: AKT1 --+> PF	17540722	
positive Expression: AKT1 --+> PF	17312274	
positive Expression: AKT1 --+> PF	17312274	
positive Expression: AKT1 --+> PF	19221061	
positive Expression: AKT1 --+> PF	16407258	
positive Expression: AKT1 --+> PF	15545269	
positive Expression: AKT1 --+> PF	16478789	
positive Expression: AKT1 --+> PF	16478789	
positive Expression: AKT1 --+> PF	16478789	
positive Expression: AKT1 --+> PF	11717164	
positive Expression: AKT1 --+> PF	11717164	
positive Expression: AKT1 --+> PF	11717164	
positive Expression: AKT1 --+> PF	11717164	
positive Expression: AKT1 --+> PF	11717164	
positive Expression: AKT1 --+> PF	18388859	
positive Expression: AKT1 --+> PF	28840539	10.1007/s12272-017-0948-4
positive Expression: AKT1 --+> PF	29283422	10.3390/ijms19010090
positive Expression: AKT1 --+> PF	29283422	10.3390/ijms19010090
positive Expression: AKT1 --+> PF	29899319	10.3390/ijms19061760
positive Expression: AKT1 --+> PPARG		10.1096/fj.201700208RR
positive Expression: AKT1 --+> PF	30250490	10.1155/2018/4573254
positive Expression: AKT1 --+> PF	30176712	10.4014/jmb.1802.01065
positive Expression: AKT1 --+> PF	30176712	10.4014/jmb.1802.01065
positive Expression: AKT1 --+> PPARG		10.21775/cimb.031.001
positive Expression: AKT1 --+> PPARG		10.21775/cimb.031.001
positive Expression: AKT1 --+> PPARG		10.21775/cimb.031.001
positive Expression: AKT1 --+> PF	31295749	10.1055/a-0926-3991
positive Expression: AKT1 --+> PF	21673026	10.1530/JOE-11-0175
positive Expression: AKT1 --+> PF	23592774	10.1074/jbc.M113.459792
positive Expression: AKT1 --+> PF	23746272	10.2174/09298673113209990136
positive Expression: AKT1 --+> PF	23166699	10.1371/journal.pone.0049530
positive Expression: AKT1 --+> PF	23936120	10.1371/journal.pone.0069925
positive Expression: AKT1 --+> PF	23396401	10.2337/db12-1229
positive Expression: AKT1 --+> PF	26295369	10.1210/en.2015-1184
positive Expression: AKT1 --+> PF	26295369	10.1210/en.2015-1184
positive Expression: AKT1 --+> PF	19191008	10.1007/s11010-009-0027-310.1007/s11010-009-0
positive Expression: AKT1 --+> PF	19191008	10.1007/s11010-009-0027-310.1007/s11010-009-0
positive Expression: AKT1 --+> PF	26615875	10.1016/j.bbalip.2015.11.00610.1016/j.bbalip.
positive Expression: AKT1 --+> PF	27940057	10.1016/j.ejphar.2016.12.00610.1016/j.ejphar.
positive Expression: AKT1 --+> PF	12782654	
positive Expression: AKT1 --+> PF	12782654	
positive Expression: AKT1 --+> PF	12782654	
positive Expression: AKT1 --+> PF	12782654	
positive Expression: AKT1 --+> PF	12782654	

positive Expression: AKT1 --+> PF	12782654	
positive Expression: AKT1 --+> PF	26700098	10.1016/j.tips.2015.11.011
positive Expression: AKT1 --+> PF	26976717	10.1016/j.diff.2016.02.003
positive Expression: AKT1 --+> PF	17312274	
positive Expression: AKT1 --+> PF	16478789	
positive Expression: AKT1 --+> PF	21315734	10.1016/j.lfs.2011.02.001
positive Expression: AKT1 --+> PF	14597716	
positive Expression: AKT1 --+> PF	11717164	
positive Expression: AKT1 --+> PF	18388859	
positive Expression: AKT1 --+> PF	11717164	
positive Expression: AKT1 --+> PPARG		
positive Expression: AKT1 --+> PF	33152931	10.1016/j.biopha.2020.11076910.1016/j.biopha.
positive Expression: AKT1 --+> PF	33199780	10.1038/s41467-020-19668-y
positive Expression: AKT1 --+> PF	32976570	10.1210/endocr/bqaa175
positive Expression: AKT1 --+> PF	32976570	10.1210/endocr/bqaa175
positive Expression: AKT1 --+> PF	32976570	10.1210/endocr/bqaa175
positive Expression: AKT1 --+> PF	32976570	10.1210/endocr/bqaa175
positive Expression: AKT1 --+> PF	33541789	10.1016/j.orcp.2021.01.003
positive Expression: AKT1 --+> PF	33712900	10.1007/s00431-021-03977-5
positive Expression: AKT1 --+> PF	34833928	10.3390/molecules26226836
Expression: PPARG ---> BECN1	28433567	10.1016/j.neulet.2017.02.075
Expression: PPARG ---> BECN1	26272430	10.1016/j.bbagen.2015.08.004
Expression: PPARG ---> BECN1	26272430	10.1016/j.bbagen.2015.08.004
Expression: PPARG ---> BECN1	27352979	10.1016/j.freeradbiomed.2016.06.023
Expression: PPARG ---> BECN1	26790955	10.1016/j.yexcr.2016.01.004
Expression: PPARG ---> BECN1	21700710	10.1074/jbc.M111.273292
Expression: PPARG ---> BECN1	23574724	10.4161/cc.24289
Expression: PPARG ---> BECN1	23574724	10.4161/cc.24289
Expression: PPARG ---> BECN1	21700710	10.1074/jbc.M111.273292
Expression: PPARG ---> BECN1	35944278	10.1089/dna.2022.0087
Regulation: PAX8 ---> PTEN	26138515	10.1016/j.ctrv.2015.06.005
Regulation: PAX8 ---> PTEN	29367766	10.1038/s41388-017-0097-8
Regulation: PAX8 ---> PTEN	29367766	10.1038/s41388-017-0097-8
positive Expression: PTEN --+> PA	31807182	10.3892/ol.2019.1102810.3892/ol.2019.11028
positive Expression: PTEN --+> PA	31807182	10.3892/ol.2019.1102810.3892/ol.2019.11028
positive Expression: NGF --+> NOT	29991107	10.3340/jkns.2017.021910.3340/jkns.2017.0219
positive Expression: NGF --+> NOT	29991107	10.3340/jkns.2017.021910.3340/jkns.2017.0219
positive Expression: NOTCH1 --+>	17141050	10.1016/S0076-6879(06)19002-3
positive Expression: NOTCH1 --+>	22249262	10.1038/onc.2011.615
positive Expression: NOTCH1 --+>	22249262	10.1038/onc.2011.615
positive Expression: NOTCH1 --+>	22249262	10.1038/onc.2011.615
positive Expression: NOTCH1 --+>	22249262	10.1038/onc.2011.615
positive Expression: NOTCH1 --+>	23872918	10.1038/cmi.2013.22
positive Expression: NOTCH1 --+>	23872918	10.1038/cmi.2013.22
positive Expression: NOTCH1 --+>	23872918	10.1038/cmi.2013.22
positive Expression: NOTCH1 --+>	23872918	10.1038/cmi.2013.22
positive Expression: NOTCH1 --+>	23872918	10.1038/cmi.2013.22
positive Expression: NOTCH1 --+>	23872918	10.1038/cmi.2013.22
positive Expression: NOTCH1 --+>	25231743	10.1038/leu.2014.276
positive Expression: NOTCH1 --+>	23872918	10.1038/cmi.2013.22
positive Expression: NOTCH1 --+>	23872918	10.1038/cmi.2013.22
positive Expression: NOTCH1 --+>	23872918	10.1038/cmi.2013.22
positive Expression: NOTCH1 --+>	26041884	
positive Expression: NOTCH1 --+>	26041884	

positive Expression: NOTCH1 --+> 26041884
positive Expression: NOTCH1 --+> 26041884
positive Expression: NOTCH1 --+> 30224339 10.1158/1078-0432.CCR-18-086710.1158/1078-043
Expression: TP53 ---> PPP1R3C   23837542 10.1089/dna.2013.2084
Expression: TP53 ---> PPP1R3C   23837542 10.1089/dna.2013.2084
Expression: TP53 ---> PPP1R3C
DirectRegulation: ING4 ---> TP53 21177815 10.1128/JVI.02279-10
DirectRegulation: ING4 ---> TP53 21177815 10.1128/JVI.02279-10
DirectRegulation: ING4 ---> TP53 21177815 10.1128/JVI.02279-10
DirectRegulation: ING4 ---> TP53 21177815 10.1128/JVI.02279-10
DirectRegulation: ING4 ---> TP53 21177815 10.1128/JVI.02279-10
DirectRegulation: ING4 ---> TP53 21177815 10.1128/JVI.02279-10
DirectRegulation: ING4 ---> TP53 21177815 10.1128/JVI.02279-10
DirectRegulation: ING4 ---> TP53 21177815 10.1128/JVI.02279-10
DirectRegulation: ING4 ---> TP53 21177815 10.1128/JVI.02279-10
DirectRegulation: ING4 ---> TP53 21177815 10.1128/JVI.02279-10
DirectRegulation: ING4 ---> TP53 25792601 10.1101/gad.254292.114
DirectRegulation: ING4 ---> TP53 25792601 10.1101/gad.254292.114
DirectRegulation: ING4 ---> TP53 24581166 10.3779/j.issn.1009-3419.2014.02.13
DirectRegulation: ING4 ---> TP53 23967213 10.1371/journal.pone.0071453
DirectRegulation: ING4 ---> TP53 23967213 10.1371/journal.pone.0071453
DirectRegulation: ING4 ---> TP53 23967213 10.1371/journal.pone.0071453
DirectRegulation: ING4 ---> TP53 23967213 10.1371/journal.pone.0071453
DirectRegulation: ING4 ---> TP53 23967213 10.1371/journal.pone.0071453
DirectRegulation: ING4 ---> TP53 23967213 10.1371/journal.pone.0071453
DirectRegulation: ING4 ---> TP53 21454715 10.1074/jbc.M111.230961
DirectRegulation: ING4 ---> TP53 21454715 10.1074/jbc.M111.230961
DirectRegulation: ING4 ---> TP53 21454715 10.1074/jbc.M111.230961
DirectRegulation: ING4 ---> TP53 21454715 10.1074/jbc.M111.230961
DirectRegulation: ING4 ---> TP53 21454715 10.1074/jbc.M111.230961
DirectRegulation: ING4 ---> TP53 21454715 10.1074/jbc.M111.230961
DirectRegulation: ING4 ---> TP53 21454715 10.1074/jbc.M111.230961
DirectRegulation: ING4 ---> TP53 21454715 10.1074/jbc.M111.230961
DirectRegulation: ING4 ---> TP53 21454715 10.1074/jbc.M111.230961
DirectRegulation: ING4 ---> TP53 21454715 10.1074/jbc.M111.230961
DirectRegulation: ING4 ---> TP53 21454715 10.1074/jbc.M111.230961
DirectRegulation: ING4 ---> TP53 21454715 10.1074/jbc.M111.230961
DirectRegulation: ING4 ---> TP53 21454715 10.1074/jbc.M111.230961
DirectRegulation: ING4 ---> TP53 21454715 10.1074/jbc.M111.230961
DirectRegulation: ING4 ---> TP53 21454715 10.1074/jbc.M111.230961
DirectRegulation: ING4 ---> TP53 21454715 10.1074/jbc.M111.230961
DirectRegulation: ING4 ---> TP53 21454715 10.1074/jbc.M111.230961
DirectRegulation: ING4 ---> TP53 21052543 10.1155/2011/963614
DirectRegulation: ING4 ---> TP53 34116679 10.1186/s12943-021-01373-z
DirectRegulation: ING4 ---> TP53 33299127 10.1038/s41374-020-00516-y
DirectRegulation: ING4 ---> TP53 33076322 10.3390/ijms21207622
DirectRegulation: ING4 ---> TP53 32079300 10.3390/ijms21041351
DirectRegulation: ING4 ---> TP53 30643005 10.1042/BSR20180773
DirectRegulation: ING4 ---> TP53 30403588 10.14715/cmb/2018.64.13.2
DirectRegulation: ING4 ---> TP53 27466989
DirectRegulation: ING4 ---> TP53 28050784 10.1007/s10620-016-4429-2
DirectRegulation: ING4 ---> TP53 27544390 10.1007/s00432-016-2219-z
DirectRegulation: ING4 ---> TP53 27544390 10.1007/s00432-016-2219-z
DirectRegulation: ING4 ---> TP53 26803518 10.1007/s13277-016-4842-3

DirectRegulation: ING4 ---> TP53 16024799
DirectRegulation: ING4 ---> TP53 17646389
DirectRegulation: ING4 ---> TP53 16520380
DirectRegulation: ING4 ---> TP53 26072331 10.1016/j.exphem.2015.05.010
DirectRegulation: ING4 ---> TP53 24333729 10.1016/j.canlet.2013.11.016
DirectRegulation: ING4 ---> TP53 23603392 10.1016/j.febslet.2013.04.004
DirectRegulation: ING4 ---> TP53 23603392 10.1016/j.febslet.2013.04.004
DirectRegulation: ING4 ---> TP53 15743678 10.1016/j.biocel.2004.09.008
DirectRegulation: ING4 ---> TP53 22366145 10.1016/j.jbspin.2012.01.008
DirectRegulation: ING4 ---> TP53 22366145 10.1016/j.jbspin.2012.01.008
DirectRegulation: ING4 ---> TP53 18571892 10.1016/j.biopha.2008.04.006
DirectRegulation: ING4 ---> TP53 14572637 10.1016/S0014-5793(03)01024-X
DirectRegulation: ING4 ---> TP53 18775696 10.1016/j.yexcr.2008.08.002
DirectRegulation: ING4 ---> TP53 18775696 10.1016/j.yexcr.2008.08.002
DirectRegulation: ING4 ---> TP53 15748897 10.1016/j.yexcr.2004.11.023
DirectRegulation: ING4 ---> TP53 15882981 10.1016/j.bbrc.2005.04.023
DirectRegulation: ING4 ---> TP53 28844713 10.1016/j.canlet.2017.08.019
DirectRegulation: ING4 ---> TP53 21454715 10.1074/jbc.M111.230961
DirectRegulation: ING4 ---> TP53 21454715 10.1074/jbc.M111.230961
DirectRegulation: ING4 ---> TP53 21454715 10.1074/jbc.M111.230961
DirectRegulation: ING4 ---> TP53 21454715 10.1074/jbc.M111.230961
DirectRegulation: ING4 ---> TP53 21454715 10.1074/jbc.M111.230961
DirectRegulation: ING4 ---> TP53 21454715 10.1074/jbc.M111.230961
DirectRegulation: ING4 ---> TP53 21454715 10.1074/jbc.M111.230961
DirectRegulation: ING4 ---> TP53 21454715 10.1074/jbc.M111.230961
DirectRegulation: ING4 ---> TP53 21454715 10.1074/jbc.M111.230961
DirectRegulation: ING4 ---> TP53 21454715 10.1074/jbc.M111.230961
DirectRegulation: ING4 ---> TP53 21454715 10.1074/jbc.M111.230961
DirectRegulation: ING4 ---> TP53 21454715 10.1074/jbc.M111.230961
DirectRegulation: ING4 ---> TP53 16520380 10.1074/jbc.M511309200
DirectRegulation: ING4 ---> TP53 15882981 10.1016/j.bbrc.2005.04.023
DirectRegulation: ING4 ---> TP53 15882981 10.1016/j.bbrc.2005.04.023
DirectRegulation: ING4 ---> TP53 15882981 10.1016/j.bbrc.2005.04.023
DirectRegulation: ING4 ---> TP53 12750254
DirectRegulation: ING4 ---> TP53 12750254
DirectRegulation: ING4 ---> TP53 26530780 10.3892/or.2015.4385
DirectRegulation: ING4 ---> TP53 21177815 10.1128/JVI.02279-10
DirectRegulation: ING4 ---> TP53 21177815 10.1128/JVI.02279-10
DirectRegulation: ING4 ---> TP53 21177815 10.1128/JVI.02279-10
DirectRegulation: ING4 ---> TP53 21177815 10.1128/JVI.02279-10
DirectRegulation: ING4 ---> TP53 21177815 10.1128/JVI.02279-10
DirectRegulation: ING4 ---> TP53 23967213 10.1371/journal.pone.0071453
DirectRegulation: ING4 ---> TP53 23967213 10.1371/journal.pone.0071453
DirectRegulation: ING4 ---> TP53 23967213 10.1371/journal.pone.0071453
DirectRegulation: ING4 ---> TP53 23967213 10.1371/journal.pone.0071453
DirectRegulation: ING4 ---> TP53 23967213 10.1371/journal.pone.0071453
DirectRegulation: ING4 ---> TP53 23967213 10.1371/journal.pone.0071453
DirectRegulation: ING4 ---> TP53 23967213 10.1371/journal.pone.0071453
DirectRegulation: ING4 ---> TP53 23967213 10.1371/journal.pone.0071453
DirectRegulation: ING4 ---> TP53 23969950 10.3892/or.2013.2671
DirectRegulation: ING4 ---> TP53 20534538 10.1073/pnas.0912116107
DirectRegulation: ING4 ---> TP53 22228137 10.1007/s00432-011-1099-5
DirectRegulation: ING4 ---> TP53 21454715 10.1074/jbc.M111.230961
DirectRegulation: ING4 ---> TP53 21454715 10.1074/jbc.M111.230961

DirectRegulation: ING4 ---> TP53 30643005 10.1042/BSR20180773
DirectRegulation: ING4 ---> TP53 29489009 10.14670/HH-11-979
DirectRegulation: ING4 ---> TP53 27544390 10.1007/s00432-016-2219-z
DirectRegulation: ING4 ---> TP53 17954561
DirectRegulation: ING4 ---> TP53 17954561
DirectRegulation: ING4 ---> TP53 15528276
DirectRegulation: ING4 ---> TP53 20534538
DirectRegulation: ING4 ---> TP53 16520380
DirectRegulation: ING4 ---> TP53 23603392 10.1016/j.febslet.2013.04.004
DirectRegulation: ING4 ---> TP53 19903538 10.1016/j.arr.2009.11.001
DirectRegulation: ING4 ---> TP53 16387653 10.1016/j.molcel.2005.12.007
DirectRegulation: ING4 ---> TP53 15935570 10.1016/j.gene.2005.02.014
DirectRegulation: ING4 ---> TP53 15251430 10.1016/j.febslet.2004.06.010
DirectRegulation: ING4 ---> TP53 15251430 10.1016/j.febslet.2004.06.010
DirectRegulation: ING4 ---> TP53 18775696 10.1016/j.yexcr.2008.08.002
DirectRegulation: ING4 ---> TP53 18775696 10.1016/j.yexcr.2008.08.002
DirectRegulation: ING4 ---> TP53 15748897 10.1016/j.yexcr.2004.11.023
DirectRegulation: ING4 ---> TP53 15882981 10.1016/j.bbrc.2005.04.023
DirectRegulation: ING4 ---> TP53 15882981 10.1016/j.bbrc.2005.04.023
DirectRegulation: ING4 ---> TP53 15882981 10.1016/j.bbrc.2005.04.023
DirectRegulation: ING4 ---> TP53 15882981 10.1016/j.bbrc.2005.04.023
DirectRegulation: ING4 ---> TP53 15882981 10.1016/j.bbrc.2005.04.023
DirectRegulation: ING4 ---> TP53 15882981 10.1016/j.bbrc.2005.04.023
DirectRegulation: ING4 ---> TP53 15882981 10.1016/j.bbrc.2005.04.023
DirectRegulation: ING4 ---> TP53 15882981 10.1016/j.bbrc.2005.04.023
DirectRegulation: ING4 ---> TP53 15882981 10.1016/j.bbrc.2005.04.023
DirectRegulation: ING4 ---> TP53 21454715 10.1074/jbc.M111.230961
DirectRegulation: ING4 ---> TP53 21454715 10.1074/jbc.M111.230961
DirectRegulation: ING4 ---> TP53 16520380 10.1074/jbc.M511309200
DirectRegulation: ING4 ---> TP53 23967213 10.1371/journal.pone.007145310.1371/journal.p
DirectRegulation: ING4 ---> TP53 21454715 10.1074/jbc.M111.23096110.1074/jbc.M111.23096
DirectRegulation: ING4 ---> TP53 21454715 10.1074/jbc.M111.23096110.1074/jbc.M111.23096
DirectRegulation: ING4 ---> TP53 21454715 10.1074/jbc.M111.23096110.1074/jbc.M111.23096
DirectRegulation: ING4 ---> TP53 21177815 10.1128/JVI.02279-1010.1128/JVI.02279-10
DirectRegulation: ING4 ---> TP53 34261347 10.1161/CIRCRESAHA.120.318364
DirectRegulation: ING4 ---> TP53 34569542 10.1159/000518414
DirectRegulation: ING4 ---> TP53 34569542 10.1159/000518414
positive Expression: ING4 --+> TF 33433356 10.1016/j.biopha.2020.111078
positive Expression: ING4 --+> TF 22436625 10.1016/j.humpath.2011.11.018
positive Expression: ING4 --+> TF 26803518 10.1007/s13277-016-4842-3
positive Expression: ING4 --+> TF 19895734
positive Expression: ING4 --+> TF 23967213 10.1371/journal.pone.0071453
positive Expression: ING4 --+> TF 23967213 10.1371/journal.pone.0071453
positive Expression: ING4 --+> TF 21177815 10.1128/JVI.02279-10
positive Expression: ING4 --+> TF 17594054 10.1007/s11060-007-9421-4
positive Expression: ING4 --+> TF 19895734 10.5732/cjc.009.10311
positive Expression: ING4 --+> TF 27806345 10.18632/oncotarget.1298410.18632/oncotarget.
positive Expression: ING4 --+> TF 23603392 10.1016/j.febslet.2013.04.004
positive Expression: ING4 --+> TF 27421660 10.1093/abbs/gmw06210.1093/abbs/gmw062
positive Expression: ING4 --+> TF 23603392 10.1016/j.febslet.2013.04.004
positive Expression: ING4 --+> TP53
positive Expression: ING4 --+> TF 35075768 10.1111/jcmm.1719210.1111/jcmm.17192
positive Expression: NOTCH1 --+> 28677782 10.3892/mmr.2017.6878
positive Expression: NOTCH1 --+> 28677782 10.3892/mmr.2017.687810.3892/mmr.2017.6878

positive Expression: NOTCH1 --+> 31250610 10.12047/j.cjap.5729.2019.03610.12047/j.cjap.
negative Regulation: RAC1 ---| ch 31787614 10.3324/haematol.2019.231340
negative Regulation: RAC1 ---| ch 32193458 10.1038/s41467-020-15308-7
negative Regulation: RAC1 ---| ch 32193458 10.1038/s41467-020-15308-7
negative Regulation: RAC1 ---| ch 32193458 10.1038/s41467-020-15308-7
negative Regulation: RAC1 ---| ch 32193458 10.1038/s41467-020-15308-7
negative Regulation: RAC1 ---| ch 32193458 10.1038/s41467-020-15308-7
negative Regulation: RAC1 ---| ch 33790078 10.1248/CPB.C20-00600
negative Regulation: RAC1 ---| ch 21837360 10.3892/ijmm.2011.775
negative Regulation: RAC1 ---| ch 23792636 10.4161/cbt.25091
negative Regulation: RAC1 ---| ch 23760915 10.1177/0300060513479875
negative Regulation: RAC1 ---| ch 23760915 10.1177/0300060513479875
negative Regulation: RAC1 ---| ch 24441506 10.1097/CCO.0000000000000046
negative Regulation: RAC1 ---| ch 21837360 10.3892/ijmm.2011.77510.3892/ijmm.2011.775
negative Regulation: RAC1 ---| chemosensit 10.3324/haematol.2019.231340
negative Regulation: RAC1 ---| ch 36599922 10.1038/s41388-022-02574-6
Binding: SH3GL3 ---- AKT1 17341688
QuantitativeChange: hypopharyngea 34054937 10.1155/2021/5525091
QuantitativeChange: hypopharyngea 34054937 10.1155/2021/5525091
Regulation: PPARG ---> hypopharyn 32373170 10.1155/2020/6452182
negative Expression: PAX8-AS1 --- 34012023 10.1038/s12276-021-00621-y
positive Expression: PPARG --+> T 19168033 10.1016/j.bbrc.2009.01.060
positive Expression: PPARG --+> T 18343364 10.1016/j.ejphar.2008.01.041
positive Expression: PPARG --+> T 19193378 10.1016/j.atherosclerosis.2008.12.036
positive Expression: PPARG --+> T 17870167 10.1016/j.molimm.2007.08.004
positive Expression: PPARG --+> T 21946416 10.1016/j.neures.2011.09.003
positive Expression: PPARG --+> T 25944662 10.1016/j.bbrc.2015.04.023
positive Expression: PPARG --+> T 16887883
positive Expression: PPARG --+> T 16887883
positive Expression: PPARG --+> T 16887883
positive Expression: PPARG --+> T 15190077
positive Expression: PPARG --+> T 23213321 10.1155/2012/968040
positive Expression: PPARG --+> T 21445621 10.1007/s00394-011-0187-2
positive Expression: PPARG --+> T 21445621 10.1007/s00394-011-0187-2
positive Expression: PPARG --+> TP53 10.1074/jbc.M113.453886
positive Expression: PPARG --+> T 25881671 10.1042/CS20130463
positive Expression: PPARG --+> T 25881671 10.1042/CS20130463
positive Expression: PPARG --+> T 25881671 10.1042/CS20130463
positive Expression: PPARG --+> TP53 10.1042/CS20140631
positive Expression: PPARG --+> T 26942868 10.1080/01635581.2016.1142581
positive Expression: PPARG --+> T 26942868 10.1080/01635581.2016.1142581
positive Expression: PPARG --+> T 16887883
positive Expression: PPARG --+> T 19168033 10.1016/j.bbrc.2009.01.060
positive Expression: PPARG --+> TP53
positive Expression: PPARG --+> T 765 10.1016/j.biopha.2017.11.075
positive Expression: PPARG --+> T 29311623 10.1038/s41418-017-0004-4
ProtModification: AKT1 ---> PRPH 17569669 10.1074/jbc.M611703200
ProtModification: AKT1 ---> PRPH 17569669 10.1074/jbc.M611703200
ProtModification: AKT1 ---> PRPH 17569669 10.1074/jbc.M611703200
ProtModification: AKT1 ---> PRPH 17569669 10.1074/jbc.M611703200
ProtModification: AKT1 ---> PRPH 17569669 10.1074/jbc.M611703200
ProtModification: AKT1 ---> PRPH 17569669 10.1074/jbc.M611703200
ProtModification: AKT1 ---> PRPH 17569669 10.1074/jbc.M611703200
ProtModification: AKT1 ---> PRPH 17569669 10.1074/jbc.M611703200

ProtModification: AKT1 ---> PRPH 18502206 10.1016/j.febslet.2008.04.064
ProtModification: AKT1 ---> PRPH 17569669
ProtModification: AKT1 ---> PRPH 17569669
ProtModification: AKT1 ---> PRPH 17569669
ProtModification: AKT1 ---> PRPH 17569669
ProtModification: AKT1 ---> PRPH 17569669
ProtModification: AKT1 ---> PRPH 17569669
ProtModification: AKT1 ---> PRPH 17569669
ProtModification: AKT1 ---> PRPH 17569669
ProtModification: AKT1 ---> PRPH 17569669
ProtModification: AKT1 ---> PRPH 17569669
ProtModification: AKT1 ---> PRPH 17569669 10.1074/jbc.M611703200
ProtModification: AKT1 ---> PRPH 36499746 10.3390/ijms232315416
Binding: BECN1 ---- RAC1 22393062 10.1074/jbc.M112.348375
Binding: BECN1 ---- RAC1 22393062 10.1074/jbc.M112.348375
Binding: BECN1 ---- RAC1 22393062 10.1074/jbc.M112.348375
Binding: BECN1 ---- RAC1 22393062 10.1074/jbc.M112.348375
Binding: BECN1 ---- RAC1 22393062 10.1074/jbc.M112.348375
Binding: BECN1 ---- RAC1 22393062 10.1074/jbc.M112.348375
Binding: BECN1 ---- RAC1 22393062 10.1074/jbc.M112.348375
negative Regulation: PPARG ---| F 18209083
negative Regulation: PPARG ---| RAC1
positive Expression: RAC1 --+> BE 32913260 10.1038/s41419-020-02951-7
positive Expression: RAC1 --+> BE 32913260 10.1038/s41419-020-02951-7
positive Expression: RAC1 --+> BE 32913260 10.1038/s41419-020-02951-710.1038/s41419-020-
positive Regulation: GPER1 --+> M 30360801 10.1016/j.amjms.2018.06.009
positive Regulation: GPER1 --+> M 29964007 10.1016/j.yhbeh.2018.06.012
positive Regulation: GPER1 --+> M 15090535 10.1074/jbc.M403588200
positive Regulation: GPER1 --+> M 29378226 10.1016/j.steroids.2018.01.010
positive Regulation: GPER1 --+> M 33711400 10.1016/j.neuint.2021.105011
positive Regulation: GPER1 --+> M 27519631 10.1016/j.mce.2016.08.011
positive Regulation: GPER1 --+> M 27940299 10.1016/j.mce.2016.12.007
positive Regulation: GPER1 --+> M 27940299 10.1016/j.mce.2016.12.007
positive Regulation: GPER1 --+> M 27773847 10.1016/j.mce.2016.10.020
positive Regulation: GPER1 --+> M 27773847 10.1016/j.mce.2016.10.020
positive Regulation: GPER1 --+> M 28736254 10.1016/j.mce.2017.07.016
positive Regulation: GPER1 --+> M 32777349 10.1016/j.neulet.2020.135299
positive Regulation: GPER1 --+> M 32325111 10.1016/j.tiv.2020.104871
positive Regulation: GPER1 --+> M 32325111 10.1016/j.tiv.2020.104871
positive Regulation: GPER1 --+> M 32325111 10.1016/j.tiv.2020.104871
positive Regulation: GPER1 --+> M 27369115 10.1016/j.jsbmb.2016.06.014
positive Regulation: GPER1 --+> M 28465157 10.1016/j.jsbmb.2017.04.012
positive Regulation: GPER1 --+> M 28465157 10.1016/j.jsbmb.2017.04.012
positive Regulation: GPER1 --+> M 26482875 10.1016/j.tem.2015.09.002
positive Regulation: GPER1 --+> M 26482875 10.1016/j.tem.2015.09.002
positive Regulation: GPER1 --+> M 27423484 10.1016/j.jphs.2016.06.005
positive Regulation: GPER1 --+> M 33161112 10.1016/j.etap.2020.103544
positive Regulation: GPER1 --+> M 31394165 10.1016/j.bbalip.2019.08.001
positive Regulation: GPER1 --+> M 32791253 10.1016/j.cbpc.2020.108868
positive Regulation: GPER1 --+> M 20875808 10.1016/j.brainres.2010.09.069
positive Regulation: GPER1 --+> M 22285849 10.1016/j.steroids.2011.12.023
positive Regulation: GPER1 --+> M 22198527 10.1016/j.steroids.2011.11.011
positive Regulation: GPER1 --+> M 17011908 10.1016/j.surg.2006.07.007
positive Regulation: GPER1 --+> M 11744082 10.1016/S0165-0173(01)00114-X

positive Regulation: GPER1 --+> M17931703 10.1016/j.brainresrev.2007.07.020
positive Regulation: GPER1 --+> M17222505 10.1016/j.mce.2006.12.010
positive Regulation: GPER1 --+> M20138962 10.1016/j.mce.2010.02.006
positive Regulation: GPER1 --+> M20138962 10.1016/j.mce.2010.02.006
positive Regulation: GPER1 --+> M20138962 10.1016/j.mce.2010.02.006
positive Regulation: GPER1 --+> M18556135 10.1016/j.neuroscience.2008.05.001
positive Regulation: GPER1 --+> M11897506 10.1016/S0960-0760(01)00190-X
positive Regulation: GPER1 --+> M16019207 10.1016/j.jsbmb.2005.05.006
positive Regulation: GPER1 --+> M19442754 10.1016/j.prostaglandins.2009.05.001
positive Regulation: GPER1 --+> M19442754 10.1016/j.prostaglandins.2009.05.001
positive Regulation: GPER1 --+> M22917634 10.1016/j.steroids.2012.07.019
positive Regulation: GPER1 --+> M23219795 10.1016/j.ejphar.2012.11.042
positive Regulation: GPER1 --+> M23385013 10.1016/j.steroids.2012.12.015
positive Regulation: GPER1 --+> M23402905 10.1016/j.ygyno.2013.02.005
positive Regulation: GPER1 --+> M23085545 10.1016/j.brainresbull.2012.10.004
positive Regulation: GPER1 --+> M23466742 10.1016/j.phrs.2013.02.008
positive Regulation: GPER1 --+> M23545157 10.1016/j.mce.2013.03.018
positive Regulation: GPER1 --+> M23419549 10.1016/j.neuroscience.2013.02.005
positive Regulation: GPER1 --+> M24161432 10.1016/j.cbi.2013.10.014
positive Regulation: GPER1 --+> M24240011 10.1016/j.steroids.2013.11.004
positive Regulation: GPER1 --+> M24240011 10.1016/j.steroids.2013.11.004
positive Regulation: GPER1 --+> M24240011 10.1016/j.steroids.2013.11.004
positive Regulation: GPER1 --+> M24239983 10.1016/j.mce.2013.11.005
positive Regulation: GPER1 --+> M23907016 10.1016/j.jsbmb.2013.07.011
positive Regulation: GPER1 --+> M23907016 10.1016/j.jsbmb.2013.07.011
positive Regulation: GPER1 --+> M23907016 10.1016/j.jsbmb.2013.07.011
positive Regulation: GPER1 --+> M24560890 10.1016/j.yhbeh.2014.02.004
positive Regulation: GPER1 --+> M24560890 10.1016/j.yhbeh.2014.02.004
positive Regulation: GPER1 --+> M24530924 10.1016/j.mce.2014.02.002
positive Regulation: GPER1 --+> M24594140 10.1016/j.mce.2014.01.024
positive Regulation: GPER1 --+> M24495410 10.1016/j.toxlet.2014.01.035
positive Regulation: GPER1 --+> M25263582 10.1016/j.expneurol.2014.09.011
positive Regulation: GPER1 --+> M25173820 10.1016/j.steroids.2014.08.013
positive Regulation: GPER1 --+> M29365274 10.1152/AJPCELL.00152.2017
positive Regulation: GPER1 --+> M29365274 10.1152/AJPCELL.00152.2017
positive Regulation: GPER1 --+> M34445359 10.3390/ijms22168654
positive Regulation: GPER1 --+> M34182071 10.1016/j.freeradbiomed.2021.06.02510.1016/j.
positive Regulation: GPER1 --+> M22251451 10.1186/bcr3096
positive Regulation: GPER1 --+> M19741198 10.1152/ajpcell.00185.200910.1152/ajpcell.001
positive Regulation: GPER1 --+> M852 10.1016/j.jphs.2016.06.005
positive Regulation: GPER1 --+> MAPK1 10.1016/j.steroids.2011.12.023
positive Regulation: GPER1 --+> M24835924 10.1016/j.mce.2014.05.005
positive Regulation: GPER1 --+> M25150623 10.1016/j.mce.2014.07.023
positive Regulation: GPER1 --+> M25150623 10.1016/j.mce.2014.07.023
positive Regulation: GPER1 --+> M25150623 10.1016/j.mce.2014.07.023
positive Regulation: GPER1 --+> M25312822 10.1016/j.biopha.2014.09.003
positive Regulation: GPER1 --+> M24930125 10.1016/j.etap.2014.05.007
positive Regulation: GPER1 --+> M26006730 10.1016/j.taap.2015.05.010
positive Regulation: GPER1 --+> MAPK1 10.1016/j.biopha.2015.07.031
positive Regulation: GPER1 --+> M25766520 10.1016/j.jsbmb.2015.02.008
positive Regulation: GPER1 --+> M17308128 10.1158/0008-5472.CAN-06-2909
positive Regulation: GPER1 --+> M20138962 10.1016/j.mce.2010.02.00610.1016/j.mce.2010.0
positive Regulation: GPER1 --+> M23907016 10.1016/j.jsbmb.2013.07.01110.1016/j.jsbmb.20
positive Regulation: GPER1 --+> M25847233 10.1074/jbc.M114.62874310.1074/jbc.M114.62874

positive Regulation: GPER1 --+> M27940299 10.1016/j.mce.2016.12.00710.1016/j.mce.2016.1
positive Regulation: GPER1 --+> M28736254 10.1016/j.mce.2017.07.01610.1016/j.mce.2017.0
positive Regulation: GPER1 --+> M31545985 10.1016/j.ejphar.2019.17266710.1016/j.ejphar.
positive Regulation: GPER1 --+> M29777703 10.1016/j.bbrc.2018.05.100
positive Regulation: GPER1 --+> M23274518 10.1016/j.taap.2012.12.014
positive Regulation: GPER1 --+> MAPK1 10.1016/j.bpj.2011.11.775
positive Regulation: GPER1 --+> M19410597 10.1016/j.yfrne.2009.04.014
positive Regulation: GPER1 --+> M18262661 10.1016/j.tips.2008.01.001
positive Regulation: GPER1 --+> M20875696 10.1016/j.envpol.2010.09.004
positive Regulation: GPER1 --+> M20875696 10.1016/j.envpol.2010.09.004
positive Regulation: GPER1 --+> M18775472 10.1016/j.mce.2008.08.003
positive Regulation: GPER1 --+> M21036202 10.1016/j.bbcan.2010.10.005
positive Regulation: GPER1 --+> M22265196 10.1016/j.psyneuen.2011.12.018
positive Regulation: GPER1 --+> MAPK1 10.1016/j.genm.2012.01.008
positive Regulation: GPER1 --+> M22265196 10.1016/j.psyneuen.2011.12.018
positive Regulation: GPER1 --+> M23466742 10.1016/j.phrs.2013.02.008
positive Regulation: GPER1 --+> M23466742 10.1016/j.phrs.2013.02.008
positive Regulation: GPER1 --+> M24188886 10.1016/j.ygcen.2013.10.015
positive Regulation: GPER1 --+> M24240011 10.1016/j.steroids.2013.11.004
positive Regulation: GPER1 --+> M24239983 10.1016/j.mce.2013.11.005
positive Regulation: GPER1 --+> M24440569 10.1016/j.taap.2014.01.005
positive Regulation: GPER1 --+> M24530924 10.1016/j.mce.2014.02.002
positive Regulation: GPER1 --+> M24495410 10.1016/j.toxlet.2014.01.035
positive Regulation: GPER1 --+> M24495410 10.1016/j.toxlet.2014.01.035
positive Regulation: GPER1 --+> M24495410 10.1016/j.toxlet.2014.01.035
positive Regulation: GPER1 --+> M24495410 10.1016/j.toxlet.2014.01.035
positive Regulation: GPER1 --+> M24565562 10.1016/j.jsbmb.2014.02.010
positive Regulation: GPER1 --+> M27908592 10.1016/j.yexcr.2016.11.022
positive Regulation: GPER1 --+> M27908592 10.1016/j.yexcr.2016.11.022
positive Regulation: GPER1 --+> M27481507 10.1007/s00441-016-2466-y
positive Regulation: GPER1 --+> M26954778 10.1159/000445190
positive Regulation: GPER1 --+> M26954778 10.1159/000445190
positive Regulation: GPER1 --+> M28347854 10.1016/j.jsbmb.2017.03.021
positive Regulation: GPER1 --+> M28450397 10.1074/jbc.M116.76587510.1074/jbc.M116.76587
positive Regulation: GPER1 --+> M28450397 10.1074/jbc.M116.76587510.1074/jbc.M116.76587
positive Regulation: GPER1 --+> M28450143 10.1016/j.ygcen.2017.04.013
positive Regulation: GPER1 --+> M28535016 10.3892/or.2017.564910.3892/or.2017.5649
positive Regulation: GPER1 --+> M28595943 10.1016/j.jsbmb.2017.05.005
positive Regulation: GPER1 --+> M28557972 10.12659/MSM.901850
positive Regulation: GPER1 --+> M28468276 10.3390/molecules22050705
positive Regulation: GPER1 --+> M28535016 10.3892/or.2017.5649
positive Regulation: GPER1 --+> M28535016 10.3892/or.2017.5649
positive Regulation: GPER1 --+> M28535016 10.3892/or.2017.5649
positive Regulation: GPER1 --+> M28535016 10.3892/or.2017.5649
positive Regulation: GPER1 --+> MAPK1 10.1074/jbc.M116.765875
positive Regulation: GPER1 --+> MAPK1 10.1074/jbc.M116.765875
positive Regulation: GPER1 --+> MAPK1 10.1074/jbc.M116.765875
positive Regulation: GPER1 --+> MAPK1 10.1074/jbc.M116.765875
positive Regulation: GPER1 --+> MAPK1 10.1074/jbc.M116.765875
positive Regulation: GPER1 --+> MAPK1 10.1074/jbc.M116.765875
positive Regulation: GPER1 --+> MAPK1 10.1074/jbc.M116.765875
positive Regulation: GPER1 --+> MAPK1 10.1074/jbc.M116.765875
positive Regulation: GPER1 --+> MAPK1 10.1074/jbc.M116.765875
positive Regulation: GPER1 --+> MAPK1 10.1074/jbc.M116.765875

positive Regulation: GPER1 --+> MAPK1 10.1074/jbc.M116.765875
positive Regulation: GPER1 --+> MAPK1 10.1074/jbc.M116.765875
positive Regulation: GPER1 --+> MAPK1 10.1074/jbc.M116.765875
positive Regulation: GPER1 --+> MAPK1 10.1074/jbc.M116.765875
positive Regulation: GPER1 --+> MAPK1 10.1074/jbc.M116.765875
positive Regulation: GPER1 --+> MAPK1 10.1074/jbc.M116.765875
positive Regulation: GPER1 --+> MAPK1 10.1074/jbc.M116.765875
positive Regulation: GPER1 --+> MAPK1 10.1074/jbc.M116.765875
positive Regulation: GPER1 --+> MAPK1 10.1074/jbc.M116.765875
positive Regulation: GPER1 --+> MAPK1 10.1074/jbc.M116.765875
positive Regulation: GPER1 --+> MAPK1 10.1074/jbc.M116.765875
positive Regulation: GPER1 --+> MAPK1 10.1042/BCJ20170630
positive Regulation: GPER1 --+> N29224106 10.1007/978-3-319-70178-3_20
positive Regulation: GPER1 --+> N28736254 10.1016/j.mce.2017.07.016
positive Regulation: GPER1 --+> N29167148 10.1152/ajpcell.00060.2017
positive Regulation: GPER1 --+> N25588050 10.4161/19336918.2014.990781
positive Regulation: GPER1 --+> N25588050 10.4161/19336918.2014.990781
positive Regulation: GPER1 --+> N29964007 10.1016/j.yhbeh.2018.06.012
positive Regulation: GPER1 --+> N29566418 10.1055/s-0044-100920
positive Regulation: GPER1 --+> N30041006 10.1016/j.bbr.2018.07.017
positive Regulation: GPER1 --+> N30195206 10.1016/j.ecoenv.2018.08.10210.1016/j.ecoenv.
positive Regulation: GPER1 --+> N30478301 10.1038/s41419-018-1144-2
positive Regulation: GPER1 --+> N21241678 10.1016/j.brainres.2011.01.034
positive Regulation: GPER1 --+> N20875696 10.1016/j.envpol.2010.09.004
positive Regulation: GPER1 --+> N19549587 10.1016/j.mce.2009.03.009
positive Regulation: GPER1 --+> N25312822 10.1016/j.biopha.2014.09.003
positive Regulation: GPER1 --+> N25744307 10.1016/j.cbi.2015.02.014
positive Regulation: GPER1 --+> N26190834 10.1016/j.mce.2015.07.016
positive Regulation: GPER1 --+> N26190834 10.1016/j.mce.2015.07.016
positive Regulation: GPER1 --+> N26190834 10.1016/j.mce.2015.07.016
positive Regulation: GPER1 --+> N26190834 10.1016/j.mce.2015.07.016
positive Regulation: GPER1 --+> N26208432 10.1016/j.canlet.2015.07.017
positive Regulation: GPER1 --+> MAPK1 10.1016/j.biopha.2015.07.031
positive Regulation: GPER1 --+> N19608654
positive Regulation: GPER1 --+> N19608654
positive Regulation: GPER1 --+> N19608654
positive Regulation: GPER1 --+> N19420011
positive Regulation: GPER1 --+> N19307418
positive Regulation: GPER1 --+> N18566133
positive Regulation: GPER1 --+> N18566127
positive Regulation: GPER1 --+> N18467434
positive Regulation: GPER1 --+> N18467434
positive Regulation: GPER1 --+> N18467434
positive Regulation: GPER1 --+> N17916740
positive Regulation: GPER1 --+> N17823264
positive Regulation: GPER1 --+> N16645038
positive Regulation: GPER1 --+> N16239258
positive Regulation: GPER1 --+> N16123167
positive Regulation: GPER1 --+> N12446589
positive Regulation: GPER1 --+> N12446589
positive Regulation: GPER1 --+> N12446589
positive Regulation: GPER1 --+> N12446589
positive Regulation: GPER1 --+> N12446589
positive Regulation: GPER1 --+> N12446589

positive Regulation: GPER1 --+> M12446589
positive Regulation: GPER1 --+> M12446589
positive Regulation: GPER1 --+> M12446589
positive Regulation: GPER1 --+> M12446589
positive Regulation: GPER1 --+> M12446589
positive Regulation: GPER1 --+> M12193550
positive Regulation: GPER1 --+> M12193550
positive Regulation: GPER1 --+> M11773440
positive Regulation: GPER1 --+> M11043579
positive Regulation: GPER1 --+> M11043579
positive Regulation: GPER1 --+> M11043579
positive Regulation: GPER1 --+> M11043579
positive Regulation: GPER1 --+> M9973260
positive Regulation: GPER1 --+> M19818165
positive Regulation: GPER1 --+> M18768737
positive Regulation: GPER1 --+> M18768737
positive Regulation: GPER1 --+> M16636149
positive Regulation: GPER1 --+> M15090535
positive Regulation: GPER1 --+> M15090535
positive Regulation: GPER1 --+> M11029403
positive Regulation: GPER1 --+> M19357782
positive Regulation: GPER1 --+> M19079715
positive Regulation: GPER1 --+> M22414008
positive Regulation: GPER1 --+> M22495674 10.1210/en.2012-1061
positive Regulation: GPER1 --+> MAPK1
positive Regulation: GPER1 --+> M23163984 10.1186/1757-2215-5-35
positive Regulation: GPER1 --+> M23163984 10.1186/1757-2215-5-35
positive Regulation: GPER1 --+> M23232940 10.3892/or.2012.2180
positive Regulation: GPER1 --+> M23273253 10.1186/1471-2407-12-624
positive Regulation: GPER1 --+> M19818165 10.1186/bcr2361
positive Regulation: GPER1 --+> M21149639 10.1124/mol.110.069500
positive Regulation: GPER1 --+> M21303939 10.1210/en.2010-1368
positive Regulation: GPER1 --+> M21303939 10.1210/en.2010-1368
positive Regulation: GPER1 --+> M21303939 10.1210/en.2010-1368
positive Regulation: GPER1 --+> M21303939 10.1210/en.2010-1368
positive Regulation: GPER1 --+> M21303939 10.1210/en.2010-1368
positive Regulation: GPER1 --+> M21303939 10.1210/en.2010-1368
positive Regulation: GPER1 --+> M21303939 10.1210/en.2010-1368
positive Regulation: GPER1 --+> M21372633 10.4161/cbt.11.8.15272
positive Regulation: GPER1 --+> M21673097 10.1210/en.2011-0091
positive Regulation: GPER1 --+> M21673097 10.1210/en.2011-0091
positive Regulation: GPER1 --+> M21673097 10.1210/en.2011-0091
positive Regulation: GPER1 --+> M21673097 10.1210/en.2011-0091
positive Regulation: GPER1 --+> M22355098 10.1530/JME-11-0145
positive Regulation: GPER1 --+> MAPK1 10.1186/bcr3096
positive Regulation: GPER1 --+> MAPK1 10.1186/bcr3096
positive Regulation: GPER1 --+> M21930911 10.1073/pnas.1102821108
positive Regulation: GPER1 --+> M20661136 10.1097/FBP.0b013e32833da5c3
positive Regulation: GPER1 --+> M21242460 10.1161/HYPERTENSIONAHA.110.161653
positive Regulation: GPER1 --+> M21242460 10.1161/HYPERTENSIONAHA.110.161653
positive Regulation: GPER1 --+> M21242460 10.1161/HYPERTENSIONAHA.110.161653
positive Regulation: GPER1 --+> M21242460 10.1161/HYPERTENSIONAHA.110.161653
positive Regulation: GPER1 --+> M21482958 10.1161/HYPERTENSIONAHA.110.159459
positive Regulation: GPER1 --+> M21536991 10.1161/HYPERTENSIONAHA.110.167650

positive Regulation: GPER1 --+> M23460719 10.1210/er.2012-1055
positive Regulation: GPER1 --+> M23756388
positive Regulation: GPER1 --+> M23610132 10.1210/en.2012-2119
positive Regulation: GPER1 --+> M23519092 10.1097/MED.0b013e328360c200
positive Regulation: GPER1 --+> M23590758 10.2174/1566524011313060015
positive Regulation: GPER1 --+> M23590758 10.2174/1566524011313060015
positive Regulation: GPER1 --+> M20203690 10.1038/cdd.2010.20
positive Regulation: GPER1 --+> M20203690 10.1038/cdd.2010.20
positive Regulation: GPER1 --+> M20203690 10.1038/cdd.2010.20
positive Regulation: GPER1 --+> M20203690 10.1038/cdd.2010.20
positive Regulation: GPER1 --+> M20203690 10.1038/cdd.2010.20
positive Regulation: GPER1 --+> M20203690 10.1038/cdd.2010.20
positive Regulation: GPER1 --+> M20203690 10.1038/cdd.2010.20
positive Regulation: GPER1 --+> M22167233 10.1038/jcbfm.2011.179
positive Regulation: GPER1 --+> M22492045 10.1523/JNEUROSCI.5828-11.2012
positive Regulation: GPER1 --+> M22492045 10.1523/JNEUROSCI.5828-11.2012
positive Regulation: GPER1 --+> M22492045 10.1523/JNEUROSCI.5828-11.2012
positive Regulation: GPER1 --+> M22492045 10.1523/JNEUROSCI.5828-11.2012
positive Regulation: GPER1 --+> M22552965 10.1289/ehp.1104526
positive Regulation: GPER1 --+> M22552965 10.1289/ehp.1104526
positive Regulation: GPER1 --+> M22552965 10.1289/ehp.1104526
positive Regulation: GPER1 --+> M23840305 10.1371/journal.pone.0064771
positive Regulation: GPER1 --+> M24136233 10.1038/cddis.2013.397
positive Regulation: GPER1 --+> M24343167 10.1007/s11906-013-0408-6
positive Regulation: GPER1 --+> M24481325 10.1530/ERC-13-0237
positive Regulation: GPER1 --+> M25251996 10.1146/annurev-pharmtox-010814-124302
positive Regulation: GPER1 --+> M23283935 10.1152/ajpcell.00203.2012
positive Regulation: GPER1 --+> M25005496 10.1152/ajpendo.00534.2013
positive Regulation: GPER1 --+> M25012534 10.1159/000365574
positive Regulation: GPER1 --+> M25275589 10.1038/cddis.2014.398
positive Regulation: GPER1 --+> M25275589 10.1038/cddis.2014.398
positive Regulation: GPER1 --+> M25275589 10.1038/cddis.2014.398
positive Regulation: GPER1 --+> M25275589 10.1038/cddis.2014.398
positive Regulation: GPER1 --+> M25275589 10.1038/cddis.2014.398
positive Regulation: GPER1 --+> M25275589 10.1038/cddis.2014.398
positive Regulation: GPER1 --+> MAPK1
positive Regulation: GPER1 --+> MAPK1 10.1074/jbc.M114.628743
positive Regulation: GPER1 --+> MAPK1 10.1074/jbc.M114.628743
positive Regulation: GPER1 --+> MAPK1 10.1074/jbc.M114.628743
positive Regulation: GPER1 --+> M25763610 10.1210/me.2015-1009
positive Regulation: GPER1 --+> M26023144 10.1124/pr.114.009712
positive Regulation: GPER1 --+> M26023144 10.1124/pr.114.009712
positive Regulation: GPER1 --+> M10988335
positive Regulation: GPER1 --+> M11043579
positive Regulation: GPER1 --+> M15090535
positive Regulation: GPER1 --+> M16644474
positive Regulation: GPER1 --+> M20875696 10.1016/j.envpol.2010.09.004
positive Regulation: GPER1 --+> M20875696 10.1016/j.envpol.2010.09.004
positive Regulation: GPER1 --+> M21278053 10.1093/toxsci/kfr019
positive Regulation: GPER1 --+> M21303939 10.1210/en.2010-1368
positive Regulation: GPER1 --+> M24782323 10.1002/glia.22679
positive Regulation: GPER1 --+> M25753185 10.1002/cbf.3097
positive Regulation: GPER1 --+> M25805831 10.1096/fj.14-265603
positive Regulation: GPER1 --+> MAPK1

positive Regulation: GPER1 --+> M	26069273	10.1530/JME-15-0086
positive Regulation: GPER1 --+> M	26921679	10.1016/j.steroids.2016.02.016
positive Regulation: GPER1 --+> M	26954778	10.1159/000445190
positive Regulation: GPER1 --+> M	26987903	10.1074/jbc.M115.697334
positive Regulation: GPER1 --+> MAPK1		10.1074/jbc.M115.697334
positive Regulation: GPER1 --+> MAPK1		10.1074/jbc.M115.697334
positive Regulation: GPER1 --+> MAPK1		10.1074/jbc.M115.697334
positive Regulation: GPER1 --+> MAPK1		10.1074/jbc.M115.697334
positive Regulation: GPER1 --+> MAPK1		10.1074/jbc.M115.697334
positive Regulation: GPER1 --+> MAPK1		10.1074/jbc.M115.697334
positive Regulation: GPER1 --+> M	27213340	10.3390/ijms17050760
positive Regulation: GPER1 --+> M	26475489	10.1007/s00204-015-1615-5
positive Regulation: GPER1 --+> M	27589731	10.3390/ijms17091434
positive Regulation: GPER1 --+> M	27589731	10.3390/ijms17091434
positive Regulation: GPER1 --+> MAPK1		10.1042/BSR20160134
positive Regulation: GPER1 --+> M	29515057	10.1262/jrd.2017-153
positive Regulation: GPER1 --+> M	32297302	10.1007/s12035-020-01911-8
positive Regulation: GPER1 --+> M	32297302	10.1007/s12035-020-01911-8
positive Regulation: GPER1 --+> M	33097048	10.1186/s12974-020-01998-9
positive Regulation: GPER1 --+> M	33287384	10.3390/ijms21239229
positive Regulation: GPER1 --+> M	29137141	10.3390/ijms18112394
positive Regulation: GPER1 --+> M	33430254	10.3390/ijms22020525
positive Regulation: GPER1 --+> M	34098945	10.1186/s12935-021-02003-w
positive Regulation: GPER1 --+> M	32570260	10.1159/000509583
positive Regulation: GPER1 --+> M	34182538	10.18632/aging.203145
positive Regulation: GPER1 --+> M	34830207	10.3390/ijms222212326
positive Regulation: GPER1 --+> M	36558071	10.3390/molecules27248943
positive Regulation: GPER1 --+> M	36558071	10.3390/molecules27248943
positive Regulation: GPER1 --+> M	36558071	10.3390/molecules27248943
negative Regulation: GPER1 ---| F	34330822	10.1124/MOLPHARM.121.000259
negative Regulation: GPER1 ---| F	34330822	10.1124/MOLPHARM.121.000259
positive Regulation: STC2 --+> MA	25816401	10.1177/0394632015572745
positive Regulation: STC2 --+> MA	23187001	
positive Regulation: STC2 --+> MA	27878259	10.3892/mmr.2016.595110.3892/mmr.2016.5951
positive Regulation: STC2 --+> MA	27878259	10.3892/mmr.2016.595110.3892/mmr.2016.5951
Regulation: PPARG ---> AKT1	26220473	10.1016/j.brainres.2015.07.025
Regulation: PPARG ---> AKT1	27373852	10.1016/j.ejphar.2016.06.051
Regulation: PPARG ---> AKT1	27725163	10.1016/j.yexmp.2016.10.002
Regulation: PPARG ---> AKT1	29080795	10.1016/j.yexcr.2017.10.024
Regulation: PPARG ---> AKT1	12048207	10.1074/jbc.M203913200
Regulation: PPARG ---> AKT1	20212046	10.1074/jbc.M109.087718
Regulation: PPARG ---> AKT1	20212046	10.1074/jbc.M109.087718
Regulation: PPARG ---> AKT1	20212046	10.1074/jbc.M109.087718
Regulation: PPARG ---> AKT1	22223859	10.1194/jlr.M017319
Regulation: PPARG ---> AKT1	31786192	10.1016/j.lfs.2019.117108
Regulation: PPARG ---> AKT1	32209360	10.1016/j.metabol.2020.154215
Regulation: PPARG ---> AKT1	29655655	10.1016/j.npep.2018.04.002
Regulation: PPARG ---> AKT1	31163220	10.1016/j.fct.2019.05.060
Regulation: PPARG ---> AKT1	31163220	10.1016/j.fct.2019.05.060
Regulation: PPARG ---> AKT1	32919185	10.1016/j.nut.2020.110968
Regulation: PPARG ---> AKT1	24269521	10.1016/j.pupt.2013.11.003
Regulation: PPARG ---> AKT1	24269521	10.1016/j.pupt.2013.11.003
Regulation: PPARG ---> AKT1	24269521	10.1016/j.pupt.2013.11.003
Regulation: PPARG ---> AKT1	29522896	10.1016/j.bbalip.2018.03.001

Regulation: PPARG ---> AKT1	30134163	10.1016/j.celrep.2018.07.063
Regulation: PPARG ---> AKT1		10.1016/S0006-291X(02)02983-2
Regulation: PPARG ---> AKT1	15369766	10.1016/j.bbrc.2004.08.101
Regulation: PPARG ---> AKT1		10.1016/S0014-5793(01)02674-6
Regulation: PPARG ---> AKT1	21974806	10.1016/j.canlet.2011.09.010
Regulation: PPARG ---> AKT1	17548167	10.1016/j.mehy.2006.12.064
Regulation: PPARG ---> AKT1	18950730	10.1016/j.biocel.2008.09.022
Regulation: PPARG ---> AKT1	23562554	10.1016/j.bbagen.2013.03.029
Regulation: PPARG ---> AKT1	24094981	10.1016/j.mehy.2013.08.014
Regulation: PPARG ---> AKT1	24856644	10.1016/j.thromres.2014.05.005
Regulation: PPARG ---> AKT1	26878778	10.1016/j.jnutbio.2015.09.015
Regulation: PPARG ---> AKT1	16204373	
Regulation: PPARG ---> AKT1	15199412	
Regulation: PPARG ---> AKT1	19246449	
Regulation: PPARG ---> AKT1	17337625	
Regulation: PPARG ---> AKT1	17337625	
Regulation: PPARG ---> AKT1	12048207	
Regulation: PPARG ---> AKT1	16051889	
Regulation: PPARG ---> AKT1	16051889	
Regulation: PPARG ---> AKT1	15677515	
Regulation: PPARG ---> AKT1	12411472	
Regulation: PPARG ---> AKT1	12411472	
Regulation: PPARG ---> AKT1	27356604	10.2220/biomedres.37.167
Regulation: PPARG ---> AKT1	26885270	
Regulation: PPARG ---> AKT1	26885270	
Regulation: PPARG ---> AKT1	26885270	
Regulation: PPARG ---> AKT1	28177764	10.1139/bcb-2016-0110
Regulation: PPARG ---> AKT1	28673319	10.1186/s13046-017-0557-6
Regulation: PPARG ---> AKT1	28466688	10.1177/1074248417707049
Regulation: PPARG ---> AKT1	29457786	10.1172/JCI97702
Regulation: PPARG ---> AKT1	29686697	10.1155/2018/6970407
Regulation: PPARG ---> AKT1	29568880	10.3892/ijmm.2018.3568
Regulation: PPARG ---> AKT1	30087458	10.1038/s41374-018-0098-4
Regulation: PPARG ---> AKT1	30845932	10.1186/s12885-019-5426-6
Regulation: PPARG ---> AKT1	31173165	10.3892/ijmm.2019.4210
Regulation: PPARG ---> AKT1	30758990	10.1152/ajplung.00403.2018
Regulation: PPARG ---> AKT1	31404995	10.3390/ijms20163891
Regulation: PPARG ---> AKT1	31814555	10.2174/1568009619666191209102015
Regulation: PPARG ---> AKT1	32929062	10.1038/s41419-020-02969-x
Regulation: PPARG ---> AKT1	32710395	10.1007/s43440-020-00140-z
Regulation: PPARG ---> AKT1	32279581	10.1161/CIRCRESAHA.119.315558
Regulation: PPARG ---> AKT1	22190906	10.1155/2011/29165610.1155/2011/291656
Regulation: PPARG ---> AKT1	23766271	10.1002/stem.145510.1002/stem.1455
Regulation: PPARG ---> AKT1	145	10.1016/j.yexcr.2017.10.024
Regulation: PPARG ---> AKT1	276	10.1016/j.npep.2018.04.002
Regulation: PPARG ---> AKT1		10.1016/j.bbrc.2004.08.101
Regulation: PPARG ---> AKT1		10.1016/j.mehy.2006.12.064
Regulation: PPARG ---> AKT1		10.1016/j.jnutbio.2015.09.015
Regulation: PPARG ---> AKT1		10.1172/JCI97702
Regulation: PPARG ---> AKT1		10.3892/ijmm.2019.4210
Regulation: PPARG ---> AKT1		10.1152/ajplung.00403.2018
Regulation: PPARG ---> AKT1		10.3390/ijms20163891
Regulation: PPARG ---> AKT1		10.2174/1568009619666191209102015
Regulation: PPARG ---> AKT1	22502865	10.1186/1755-1536-5-5

Regulation: PPARG ---> AKT1	22502865	10.1186/1755-1536-5-5
Regulation: PPARG ---> AKT1	22502865	10.1186/1755-1536-5-5
Regulation: PPARG ---> AKT1	22474127	10.1210/me.2011-1253
Regulation: PPARG ---> AKT1	22474127	10.1210/me.2011-1253
Regulation: PPARG ---> AKT1	22934112	10.1155/2012/408131
Regulation: PPARG ---> AKT1	20862376	10.1155/2010/417646
Regulation: PPARG ---> AKT1	21471100	10.1164/rccm.201010-1585OC
Regulation: PPARG ---> AKT1	22190906	10.1155/2011/291656
Regulation: PPARG ---> AKT1	22190906	10.1155/2011/291656
Regulation: PPARG ---> AKT1	22190906	10.1155/2011/291656
Regulation: PPARG ---> AKT1	22190906	10.1155/2011/291656
Regulation: PPARG ---> AKT1	22190906	10.1155/2011/291656
Regulation: PPARG ---> AKT1	22190906	10.1155/2011/291656
Regulation: PPARG ---> AKT1	22481913	10.1155/2012/641087
Regulation: PPARG ---> AKT1	22481913	10.1155/2012/641087
Regulation: PPARG ---> AKT1	20127524	10.1007/s12035-010-8103-y
Regulation: PPARG ---> AKT1	20505521	10.1097/FJC.0b013e3181e74d83
Regulation: PPARG ---> AKT1	22879581	10.1161/ATVBAHA.112.253823
Regulation: PPARG ---> AKT1	22874998	10.1038/cddis.2012.99
Regulation: PPARG ---> AKT1	24096869	10.1038/cdd.2013.129
Regulation: PPARG ---> AKT1	23372643	10.1371/journal.pone.0050128
Regulation: PPARG ---> AKT1	23650507	10.1371/journal.pone.0062012
Regulation: PPARG ---> AKT1	24890395	10.1007/s40265-014-0235-3
Regulation: PPARG ---> AKT1	25748432	10.1371/journal.pone.0118902
Regulation: PPARG ---> AKT1		10.4014/jmb.1501.01052
Regulation: PPARG ---> AKT1	30405422	10.3389/fphar.2018.0121110.3389/fphar.2018.01
Regulation: PPARG ---> AKT1	31267531	10.1002/jcp.2904010.1002/jcp.29040
Regulation: PPARG ---> AKT1	32612951	10.3389/fonc.2020.0091510.3389/fonc.2020.0091
Regulation: PPARG ---> AKT1	26299428	10.3892/mmr.2015.4224
Regulation: PPARG ---> AKT1	27150726	10.3892/mmr.2016.5208
Regulation: PPARG ---> AKT1	24856644	10.1016/j.thromres.2014.05.005
Regulation: PPARG ---> AKT1	24703963	10.1016/j.peptides.2014.03.014
Regulation: PPARG ---> AKT1	22874998	10.1038/cddis.2012.99
Regulation: PPARG ---> AKT1	16051889	10.1161/01.RES.0000179226.34112.6d
Regulation: PPARG ---> AKT1	17577098	10.1097/FJC.0b013e31804654d7
Regulation: PPARG ---> AKT1	18300869	10.1097/HJH.0b013e3282f343e110.1097/HJH.0b013
Regulation: PPARG ---> AKT1	20212046	10.1074/jbc.M109.08771810.1074/jbc.M109.08771
Regulation: PPARG ---> AKT1	25748432	10.1371/journal.pone.0118902
Regulation: PPARG ---> AKT1	26643367	10.1038/srep17878
Regulation: PPARG ---> AKT1	26643367	10.1038/srep17878
Regulation: PPARG ---> AKT1	26643367	10.1038/srep17878
Regulation: PPARG ---> AKT1	26643367	10.1038/srep17878
Regulation: PPARG ---> AKT1	16204373	
Regulation: PPARG ---> AKT1	16204373	
Regulation: PPARG ---> AKT1		
Regulation: PPARG ---> AKT1	29350308	10.1007/s10549-017-4649-y10.1007/s10549-017-4
Regulation: PPARG ---> AKT1	30454889	10.1016/j.bbrc.2018.11.04510.1016/j.bbrc.2018
Regulation: PPARG ---> AKT1		10.1161/CIRCRESAHA.119.315558
Regulation: PPARG ---> AKT1		10.1161/CIRCRESAHA.119.315558
Regulation: PPARG ---> AKT1	33199780	10.1038/s41467-020-19668-y
Regulation: PPARG ---> AKT1	33805942	10.3390/molecules26071844
Regulation: PPARG ---> AKT1	34373417	10.1507/endocrj.EJ21-0110
Regulation: PPARG ---> AKT1	35181582	10.21873/cgp.20308
Regulation: PPARG ---> AKT1	35397087	10.1007/s11033-022-07401-5

Expression: AKT1 ---> PPARG	28069607	10.1182/blood-2016-10-743757
Expression: AKT1 ---> PPARG	31760068	10.1016/j.ejphar.2019.172822
Expression: AKT1 ---> PPARG	26940012	10.1016/j.yexcr.2016.02.019
Expression: AKT1 ---> PPARG	16407258	10.1074/jbc.M508799200
Expression: AKT1 ---> PPARG	23592774	10.1074/jbc.M113.459792
Expression: AKT1 ---> PPARG	15545269	10.1074/jbc.M410480200
Expression: AKT1 ---> PPARG	30694771	10.1016/j.jss.2018.11.056
Expression: AKT1 ---> PPARG	28887057	10.1016/j.lfs.2017.09.008
Expression: AKT1 ---> PPARG	28887057	10.1016/j.lfs.2017.09.008
Expression: AKT1 ---> PPARG	28887057	10.1016/j.lfs.2017.09.008
Expression: AKT1 ---> PPARG	30107270	10.1016/j.biomaterials.2018.07.046
Expression: AKT1 ---> PPARG	31585207	10.1016/j.freeradbiomed.2019.09.029
Expression: AKT1 ---> PPARG	31132754	10.1016/j.phymed.2019.152952
Expression: AKT1 ---> PPARG	26615875	10.1016/j.bbalip.2015.11.006
Expression: AKT1 ---> PPARG	33541789	10.1016/j.orcp.2021.01.003
Expression: AKT1 ---> PPARG		10.1016/j.coemr.2019.04.002
Expression: AKT1 ---> PPARG	18423396	10.1016/j.bbrc.2008.04.029
Expression: AKT1 ---> PPARG	19385085	10.1016/j.lfs.2009.01.018
Expression: AKT1 ---> PPARG	19385085	10.1016/j.lfs.2009.01.018
Expression: AKT1 ---> PPARG	21315734	10.1016/j.lfs.2011.02.001
Expression: AKT1 ---> PPARG		10.1016/S0083-6729(03)01005-7
Expression: AKT1 ---> PPARG	21333731	10.1016/j.tiv.2011.02.004
Expression: AKT1 ---> PPARG	21840191	10.1016/j.jnutbio.2011.03.021
Expression: AKT1 ---> PPARG	21840191	10.1016/j.jnutbio.2011.03.021
Expression: AKT1 ---> PPARG	22497928	10.1016/j.biocel.2012.03.019
Expression: AKT1 ---> PPARG	22497928	10.1016/j.biocel.2012.03.019
Expression: AKT1 ---> PPARG	19782949	10.1016/j.biocel.2009.04.004
Expression: AKT1 ---> PPARG	25283330	10.1016/j.jnutbio.2014.07.007
Expression: AKT1 ---> PPARG	27117918	10.1016/j.fct.2016.04.017
Expression: AKT1 ---> PPARG	17540722	
Expression: AKT1 ---> PPARG	17540722	
Expression: AKT1 ---> PPARG	17540722	
Expression: AKT1 ---> PPARG	17540722	
Expression: AKT1 ---> PPARG	17540722	
Expression: AKT1 ---> PPARG	17312274	
Expression: AKT1 ---> PPARG	17312274	
Expression: AKT1 ---> PPARG	19221061	
Expression: AKT1 ---> PPARG	16407258	
Expression: AKT1 ---> PPARG	15545269	
Expression: AKT1 ---> PPARG	16478789	
Expression: AKT1 ---> PPARG	16478789	
Expression: AKT1 ---> PPARG	16478789	
Expression: AKT1 ---> PPARG	16478789	
Expression: AKT1 ---> PPARG	11717164	
Expression: AKT1 ---> PPARG	11717164	
Expression: AKT1 ---> PPARG	11717164	
Expression: AKT1 ---> PPARG	11717164	
Expression: AKT1 ---> PPARG	11717164	
Expression: AKT1 ---> PPARG	18388859	
Expression: AKT1 ---> PPARG	28840539	10.1007/s12272-017-0948-4
Expression: AKT1 ---> PPARG	29283422	10.3390/ijms19010090
Expression: AKT1 ---> PPARG	29283422	10.3390/ijms19010090
Expression: AKT1 ---> PPARG	29899319	10.3390/ijms19061760
Expression: AKT1 ---> PPARG	29533735	10.1096/fj.201700208RR

Expression: AKT1 ---> PPARG	30250490	10.1155/2018/4573254
Expression: AKT1 ---> PPARG	30176712	10.4014/jmb.1802.01065
Expression: AKT1 ---> PPARG	30176712	10.4014/jmb.1802.01065
Expression: AKT1 ---> PPARG	30647194	10.21775/cimb.031.001
Expression: AKT1 ---> PPARG	30647194	10.21775/cimb.031.001
Expression: AKT1 ---> PPARG	30647194	10.21775/cimb.031.001
Expression: AKT1 ---> PPARG	31295749	10.1055/a-0926-3991
Expression: AKT1 ---> PPARG	33199780	10.1038/s41467-020-19668-y
Expression: AKT1 ---> PPARG	32976570	10.1210/endocr/bqaa175
Expression: AKT1 ---> PPARG	32976570	10.1210/endocr/bqaa175
Expression: AKT1 ---> PPARG	32976570	10.1210/endocr/bqaa175
Expression: AKT1 ---> PPARG	32976570	10.1210/endocr/bqaa175
Expression: AKT1 ---> PPARG	33712900	10.1007/s00431-021-03977-5
Expression: AKT1 ---> PPARG	21673026	10.1530/JOE-11-0175
Expression: AKT1 ---> PPARG	23592774	10.1074/jbc.M113.459792
Expression: AKT1 ---> PPARG	23746272	10.2174/09298673113209990136
Expression: AKT1 ---> PPARG	23166699	10.1371/journal.pone.0049530
Expression: AKT1 ---> PPARG	23936120	10.1371/journal.pone.0069925
Expression: AKT1 ---> PPARG	23396401	10.2337/db12-1229
Expression: AKT1 ---> PPARG	26295369	10.1210/en.2015-1184
Expression: AKT1 ---> PPARG	26295369	10.1210/en.2015-1184
Expression: AKT1 ---> PPARG	19191008	10.1007/s11010-009-0027-310.1007/s11010-009-0
Expression: AKT1 ---> PPARG	19191008	10.1007/s11010-009-0027-310.1007/s11010-009-0
Expression: AKT1 ---> PPARG	26615875	10.1016/j.bbalip.2015.11.00610.1016/j.bbalip.
Expression: AKT1 ---> PPARG	27940057	10.1016/j.ejphar.2016.12.00610.1016/j.ejphar.
Expression: AKT1 ---> PPARG	33152931	10.1016/j.biopha.2020.11076910.1016/j.biopha.
Expression: AKT1 ---> PPARG	34833928	10.3390/molecules26226836
Expression: AKT1 ---> PPARG	35807476	10.3390/molecules27134232
Regulation: SFRP1 ---> MAPK1	20595636	10.2353/ajpath.2010.090803
Regulation: SFRP1 ---> MAPK1	23266471	10.1016/j.cellsig.2012.12.006
Regulation: SFRP1 ---> MAPK1	20595636	10.2353/ajpath.2010.09080310.2353/ajpath.2010
negative Expression: BIRC7 ---| F	32064154	
positive PromoterBinding: PPARG -	24026200	10.3892/ijmm.2013.1487
positive PromoterBinding: PPARG -	31634455	10.1016/j.bcp.2019.113674
positive PromoterBinding: PPARG -	19684609	10.1038/aps.2009.119
positive PromoterBinding: PPARG -	16425225	10.1002/ijc.21799
positive PromoterBinding: PPARG -	22615735	10.1371/journal.pone.0035283
positive PromoterBinding: PPARG -	24905788	10.1146/annurev-biochem-082411-113907
positive PromoterBinding: PPARG -	21955520	10.1158/1940-6207.CAPR-11-0256
positive PromoterBinding: PPARG -	22902055	10.1016/j.biopha.2012.04.004
positive PromoterBinding: PPARG -	24098526	10.1371/journal.pone.0076541
positive PromoterBinding: PPARG -	21990315	10.1210/en.2011-1207
positive PromoterBinding: PPARG -	21471100	10.1164/rccm.201010-1585OC
positive PromoterBinding: PPARG -	21267808	10.1055/s-0030-1250735
positive PromoterBinding: PPARG -	1355	10.1007/bf02082676
positive PromoterBinding: PPARG -	21700710	10.1074/jbc.M111.273292
positive PromoterBinding: PPARG -	18032782	10.1242/jcs.015230
positive PromoterBinding: PPARG -	100041034	10.1016/S0006-2952(03)00488-X
positive PromoterBinding: PPARG -	28330809	10.1016/j.ebiom.2017.03.021
positive PromoterBinding: PPARG -	100041034	10.1016/S0006-2952(03)00488-X
positive PromoterBinding: PPARG -	22902055	10.1016/j.biopha.2012.04.004
positive PromoterBinding: PPARG -	18032782	10.1242/jcs.015230
positive PromoterBinding: PPARG -	33194023	
positive PromoterBinding: PPARG -	32807067	10.2174/1871520620666200817101533

positive PromoterBinding: PPARG -21990315 10.1210/en.2011-1207
positive PromoterBinding: PPARG -23867870 10.1097/MIB.0b013e318297d74110.1097/MIB.0b013
positive PromoterBinding: PPARG -1355 10.1016/j.ebiom.2017.03.021
positive PromoterBinding: PPARG --+> PTEN
positive PromoterBinding: PPARG -21267808 10.1055/s-0030-1250735
positive PromoterBinding: PPARG -21955520 10.1158/1940-6207.CAPR-11-0256
positive PromoterBinding: PPARG -21700710 10.1074/jbc.M111.273292
positive PromoterBinding: PPARG -21471100 10.1164/rccm.201010-1585OC
positive PromoterBinding: PPARG -24905788 10.1146/annurev-biochem-082411-113907
positive PromoterBinding: PPARG -24098526 10.1371/journal.pone.0076541
positive PromoterBinding: PPARG -24026200 10.3892/ijmm.2013.1487
positive PromoterBinding: PPARG -24026200 10.3892/ijmm.2013.1487
positive PromoterBinding: PPARG -24026200 10.3892/ijmm.2013.1487
positive PromoterBinding: PPARG -24026200 10.3892/ijmm.2013.1487
positive PromoterBinding: PPARG -19684609 10.1038/aps.2009.119
positive PromoterBinding: PPARG -16425225 10.1002/ijc.21799
positive PromoterBinding: PPARG -22615735 10.1371/journal.pone.0035283
positive PromoterBinding: PPARG --+> PTEN
positive PromoterBinding: PPARG -21625550 10.1371/journal.pone.0019652
positive PromoterBinding: PPARG --+> PTEN
positive PromoterBinding: PPARG --+> PTEN
positive PromoterBinding: PPARG --+> PTEN
positive PromoterBinding: PPARG --+> PTEN
positive PromoterBinding: PPARG -20198337
positive PromoterBinding: PPARG --+> PTEN
positive PromoterBinding: PPARG -24026200 10.3892/ijmm.2013.1487
positive PromoterBinding: PPARG -24026200 10.3892/ijmm.2013.1487
positive PromoterBinding: PPARG -22615735 10.1371/journal.pone.0035283
negative Expression: UCA1 ---| PT32414649 10.1016/j.clbc.2019.11.001
negative Expression: UCA1 ---| PT29061150 10.1186/s12943-017-0734-4
negative Expression: UCA1 ---| PT29328452 10.3892/or.2018.6182
negative Expression: UCA1 ---| PT29328452 10.3892/or.2018.6182
negative Expression: UCA1 ---| PT29328452 10.3892/or.2018.6182
negative Expression: UCA1 ---| PT32916254 10.1016/j.phrs.2020.105195
negative Expression: UCA1 ---| PT29908280 10.1016/j.gene.2018.06.031
negative Expression: UCA1 ---| PT29908280 10.1016/j.gene.2018.06.031
negative Expression: UCA1 ---| PT29908280 10.1016/j.gene.2018.06.031
negative Expression: UCA1 ---| PT29408336 10.1016/j.bbrc.2018.02.006
negative Regulation: PTEN ---| UC34051619 10.1016/j.tranon.2021.101134
negative Regulation: PTEN ---| UC26513297 10.18632/oncotarget.580510.18632/oncotarget.5
Expression: NGF ---> NOTCH1 21861782 10.1515/RNS.2011.041
Expression: NGF ---> NOTCH1 26491050 10.1152/ajpcell.00163.2015
Expression: NOTCH1 ---> PPARG 12960086
Expression: NOTCH1 ---> PPARG 12960086
Expression: NOTCH1 ---> PPARG 12960086
Expression: NOTCH1 ---> PPARG 9368042
Expression: NOTCH1 ---> PPARG 12107827
Expression: NOTCH1 ---> PPARG 29115530 10.3892/mmr.2017.7980
Expression: NOTCH1 ---> PPARG 33021719 10.1007/s11033-020-05884-8
Expression: NOTCH1 ---> PPARG 33021719 10.1007/s11033-020-05884-8
Expression: NOTCH1 ---> PPARG 24696378 10.1007/s10495-014-0985-0
Expression: NOTCH1 ---> PPARG 25704852 10.1016/j.intimp.2015.02.019
Expression: NOTCH1 ---> PPARG 23070070 10.1007/s00395-012-0306-01
Expression: NOTCH1 ---> PPARG 22854710 10.1159/000341451

Expression: NOTCH1 ---> PPARG 26438692 10.1242/dmm.021055
Expression: NOTCH1 ---> PPARG 22056382 10.1172/JCI45797
Expression: NOTCH1 ---> PPARG 23046039 10.1111/j.1365-2184.2012.00850.x
Expression: NOTCH1 ---> PPARG 10.2174/1381612822666161006160524
positive Regulation: SFRP1 --+> F 18156211 10.2353/ajpath.2008.070130
positive Regulation: SFRP1 --+> F 18156211 10.2353/ajpath.2008.070130
positive Regulation: SFRP1 --+> F 30542739 10.3892/or.2018.6838
positive Regulation: SFRP1 --+> F 30542739 10.3892/or.2018.6838
positive Regulation: SFRP1 --+> F 30542739 10.3892/or.2018.6838
positive Regulation: SFRP1 --+> F 30542739 10.3892/or.2018.6838
positive Regulation: SFRP1 --+> F 30542739 10.3892/or.2018.6838
positive Regulation: SFRP1 --+> F 30542739 10.3892/or.2018.6838
positive Regulation: SFRP1 --+> F 22076635 10.1161/CIRCRESAHA.111.250936
positive Regulation: SFRP1 --+> RAC1
Expression: CLDN1 ---> SPP1 31952355 10.3390/ijms21020569
Expression: CLDN1 ---> SPP1 26982264 10.1089/dna.2015.3188
positive Expression: BECN1 --+> F 30700382 10.1016/j.bbrc.2019.01.091
positive Expression: BECN1 --+> F 32713934 10.12659/MSM.922032
negative Regulation: CD34 ---| MC 27032651 10.1007/s10495-016-1238-1
negative Regulation: CD34 ---| MC 27032651 10.1007/s10495-016-1238-1
Expression: AKT1 ---> PTGS2 23911786 10.1016/j.bbrc.2013.07.110
Expression: AKT1 ---> PTGS2 23911786 10.1016/j.bbrc.2013.07.110
Expression: AKT1 ---> PTGS2 24583133 10.1016/j.bbrc.2014.02.085
Expression: AKT1 ---> PTGS2 27286705 10.1016/j.bbrc.2016.05.070
Expression: AKT1 ---> PTGS2 31629465 10.1016/j.bbrc.2019.10.092
Expression: AKT1 ---> PTGS2 30391477 10.1016/j.bcp.2018.10.034
Expression: AKT1 ---> PTGS2 28438649 10.1016/j.ejphar.2017.04.027
Expression: AKT1 ---> PTGS2 12372818 10.1074/jbc.M210106200
Expression: AKT1 ---> PTGS2 26518876 10.1074/jbc.M115.668277
Expression: AKT1 ---> PTGS2 26518876 10.1074/jbc.M115.668277
Expression: AKT1 ---> PTGS2 18326857 10.1074/jbc.M703811200
Expression: AKT1 ---> PTGS2 16982614 10.1074/jbc.M605368200
Expression: AKT1 ---> PTGS2 12072439 10.1074/jbc.M203218200
Expression: AKT1 ---> PTGS2 12960176 10.1074/jbc.M303789200
Expression: AKT1 ---> PTGS2 12865424 10.1074/jbc.M305213200
Expression: AKT1 ---> PTGS2 12865424 10.1074/jbc.M305213200
Expression: AKT1 ---> PTGS2 10930401 10.1074/jbc.M002324200
Expression: AKT1 ---> PTGS2 25371970 10.1038/jid.2014.477
Expression: AKT1 ---> PTGS2 23873269 10.1194/jlr.R037762
Expression: AKT1 ---> PTGS2 26898122 10.1016/j.lfs.2016.02.060
Expression: AKT1 ---> PTGS2 30726711 10.1016/j.lfs.2019.02.007
Expression: AKT1 ---> PTGS2 28407559 10.1016/j.molimm.2017.04.004
Expression: AKT1 ---> PTGS2 20298753 10.1016/j.neulet.2010.03.026
Expression: AKT1 ---> PTGS2 27015762 10.1016/j.bbagen.2016.03.030
Expression: AKT1 ---> PTGS2 31866556 10.1016/j.neuroscience.2019.12.005
Expression: AKT1 ---> PTGS2 32535239 10.1016/j.jep.2020.113052
Expression: AKT1 ---> PTGS2 32512147 10.1016/j.tiv.2020.104912
Expression: AKT1 ---> PTGS2 32512147 10.1016/j.tiv.2020.104912
Expression: AKT1 ---> PTGS2 30683268 10.1016/j.ogc.2018.09.006
Expression: AKT1 ---> PTGS2 27823627 10.1016/j.phymed.2016.10.007
Expression: AKT1 ---> PTGS2 26241029 10.1016/j.jsbmb.2015.07.019
Expression: AKT1 ---> PTGS2 27590705 10.1016/j.bmcl.2016.08.074
Expression: AKT1 ---> PTGS2 29031211 10.1016/j.jphotobiol.2017.10.006
Expression: AKT1 ---> PTGS2 29031211 10.1016/j.jphotobiol.2017.10.006

Expression: AKT1 ---> PTGS2 29031211 10.1016/j.jphotobiol.2017.10.006
Expression: AKT1 ---> PTGS2 31911246 10.1016/j.phrs.2020.104632
Expression: AKT1 ---> PTGS2 26485540 10.1016/j.jmgm.2015.10.004
Expression: AKT1 ---> PTGS2 26485540 10.1016/j.jmgm.2015.10.004
Expression: AKT1 ---> PTGS2 26485540 10.1016/j.jmgm.2015.10.004
Expression: AKT1 ---> PTGS2 26485540 10.1016/j.jmgm.2015.10.004
Expression: AKT1 ---> PTGS2 28389370 10.1016/j.prostaglandins.2017.04.001
Expression: AKT1 ---> PTGS2 28495310 10.1016/j.biocel.2017.05.015
Expression: AKT1 ---> PTGS2 28495310 10.1016/j.biocel.2017.05.015
Expression: AKT1 ---> PTGS2 28495310 10.1016/j.biocel.2017.05.015
Expression: AKT1 ---> PTGS2 30529656 10.1016/j.biocel.2018.12.001
Expression: AKT1 ---> PTGS2 28219839 10.1016/j.intimp.2017.01.032
Expression: AKT1 ---> PTGS2 29310107 10.1016/j.intimp.2018.01.001
Expression: AKT1 ---> PTGS2 20167866 10.2353/ajpath.2010.090714
Expression: AKT1 ---> PTGS2 20167866 10.2353/ajpath.2010.090714
Expression: AKT1 ---> PTGS2 20167866 10.2353/ajpath.2010.090714
Expression: AKT1 ---> PTGS2 20167866 10.2353/ajpath.2010.090714
Expression: AKT1 ---> PTGS2 17825254 10.1016/j.bbrc.2007.08.119
Expression: AKT1 ---> PTGS2 17067555 10.1016/j.bcp.2006.09.018
Expression: AKT1 ---> PTGS2 17067555 10.1016/j.bcp.2006.09.018
Expression: AKT1 ---> PTGS2 18606397 10.1016/j.bcp.2008.05.023
Expression: AKT1 ---> PTGS2 19426678 10.1016/j.bcp.2009.01.009
Expression: AKT1 ---> PTGS2 21669192 10.1016/j.bcp.2011.05.027
Expression: AKT1 ---> PTGS2 17481595 10.1016/j.brainres.2007.03.006
Expression: AKT1 ---> PTGS2 16005454 10.1016/j.ydbio.2005.05.033
Expression: AKT1 ---> PTGS2 17208222 10.1016/j.ejphar.2006.11.060
Expression: AKT1 ---> PTGS2 17070520 10.1016/j.yexcr.2006.09.019
Expression: AKT1 ---> PTGS2 18395088 10.1053/j.gastro.2008.01.040
Expression: AKT1 ---> PTGS2 18395088 10.1053/j.gastro.2008.01.040
Expression: AKT1 ---> PTGS2 19302827 10.1016/j.yjmcc.2009.01.006
Expression: AKT1 ---> PTGS2 19302827 10.1016/j.yjmcc.2009.01.006
Expression: AKT1 ---> PTGS2 19302827 10.1016/j.yjmcc.2009.01.006
Expression: AKT1 ---> PTGS2 19302827 10.1016/j.yjmcc.2009.01.006
Expression: AKT1 ---> PTGS2 15854673 10.1016/j.jss.2004.12.022
Expression: AKT1 ---> PTGS2 17198778 10.1016/j.jtcvs.2006.07.042
Expression: AKT1 ---> PTGS2 15748645 10.1016/j.mrfmmm.2004.07.019
Expression: AKT1 ---> PTGS2 17889918 10.1016/j.taap.2007.08.012
Expression: AKT1 ---> PTGS2 18597804 10.1016/j.taap.2008.05.017
Expression: AKT1 ---> PTGS2 18840457 10.1016/j.taap.2008.09.003
Expression: AKT1 ---> PTGS2 17223196 10.1016/j.molimm.2006.12.003
Expression: AKT1 ---> PTGS2 19118901 10.1016/j.molimm.2008.11.006
Expression: AKT1 ---> PTGS2 19118901 10.1016/j.molimm.2008.11.006
Expression: AKT1 ---> PTGS2 22154837 10.1016/j.molimm.2011.11.011
Expression: AKT1 ---> PTGS2 19815299 10.1016/j.jneuroim.2009.09.004
Expression: AKT1 ---> PTGS2 19815299 10.1016/j.jneuroim.2009.09.004
Expression: AKT1 ---> PTGS2 17084486 10.1016/j.neuint.2006.09.016
Expression: AKT1 ---> PTGS2 22542552 10.1016/j.fct.2012.04.024
Expression: AKT1 ---> PTGS2 22542552 10.1016/j.fct.2012.04.024
Expression: AKT1 ---> PTGS2 22542552 10.1016/j.fct.2012.04.024
Expression: AKT1 ---> PTGS2 22353212 10.1016/j.fct.2012.02.006
Expression: AKT1 ---> PTGS2 19339108 10.1016/j.canlet.2009.03.003
Expression: AKT1 ---> PTGS2 21924548 10.1016/j.canlet.2011.08.001
Expression: AKT1 ---> PTGS2 15921858 10.1016/j.bbcan.2005.04.002
Expression: AKT1 ---> PTGS2 22001128 10.1016/j.mehy.2011.09.039

Expression: AKT1 ---> PTGS2	18571350	10.1016/j.jep.2008.05.001
Expression: AKT1 ---> PTGS2	34547381	10.1016/j.jsbmb.2021.10600110.1016/j.jsbmb.20
Expression: AKT1 ---> PTGS2	18597804	10.1016/j.taap.2008.05.01710.1016/j.taap.2008
Expression: AKT1 ---> PTGS2	21533119	10.1371/journal.pone.001882310.1371/journal.p
Expression: AKT1 ---> PTGS2	21745389	10.1186/1471-2199-12-2910.1186/1471-2199-12-2
Expression: AKT1 ---> PTGS2	21745389	10.1186/1471-2199-12-2910.1186/1471-2199-12-2
Expression: AKT1 ---> PTGS2	22214188	10.1186/1742-2094-9-210.1186/1742-2094-9-2
Expression: AKT1 ---> PTGS2	22214188	10.1186/1742-2094-9-210.1186/1742-2094-9-2
Expression: AKT1 ---> PTGS2	23263903	10.1007/s11010-012-1539-910.1007/s11010-012-1
Expression: AKT1 ---> PTGS2	23263903	10.1007/s11010-012-1539-910.1007/s11010-012-1
Expression: AKT1 ---> PTGS2	24520260	10.3892/etm.2014.1484
Expression: AKT1 ---> PTGS2	757	10.1016/j.bmcl.2016.08.074
Expression: AKT1 ---> PTGS2	32655777	
Expression: AKT1 ---> PTGS2		10.1016/j.bbrc.2019.10.092
Expression: AKT1 ---> PTGS2	17223196	10.1016/j.molimm.2006.12.003
Expression: AKT1 ---> PTGS2		10.1016/j.molimm.2011.11.011
Expression: AKT1 ---> PTGS2		10.1016/j.fct.2012.02.006
Expression: AKT1 ---> PTGS2		10.1016/j.biopha.2003.09.009
Expression: AKT1 ---> PTGS2	20152896	10.1016/j.tiv.2010.02.008
Expression: AKT1 ---> PTGS2	20036733	10.1016/j.freeradbiomed.2009.12.014
Expression: AKT1 ---> PTGS2	20036733	10.1016/j.freeradbiomed.2009.12.014
Expression: AKT1 ---> PTGS2		10.1016/j.cellsig.2004.10.001
Expression: AKT1 ---> PTGS2		10.1016/j.cellsig.2004.10.001
Expression: AKT1 ---> PTGS2		10.1016/j.cellsig.2004.10.001
Expression: AKT1 ---> PTGS2		10.1016/j.cellsig.2004.10.001
Expression: AKT1 ---> PTGS2	15894170	10.1016/j.cellsig.2004.11.015
Expression: AKT1 ---> PTGS2	17341442	10.1016/j.cellsig.2007.01.028
Expression: AKT1 ---> PTGS2	17692505	10.1016/j.cellsig.2007.07.006
Expression: AKT1 ---> PTGS2	17692505	10.1016/j.cellsig.2007.07.006
Expression: AKT1 ---> PTGS2	17692505	10.1016/j.cellsig.2007.07.006
Expression: AKT1 ---> PTGS2	17692505	10.1016/j.cellsig.2007.07.006
Expression: AKT1 ---> PTGS2	19007880	10.1016/j.cellsig.2008.10.014
Expression: AKT1 ---> PTGS2	20206688	10.1016/j.cellsig.2010.02.010
Expression: AKT1 ---> PTGS2	19914807	10.1016/j.jdermsci.2009.10.005
Expression: AKT1 ---> PTGS2	18656337	10.1016/j.jnutbio.2008.02.004
Expression: AKT1 ---> PTGS2	22071130	10.1016/j.ejca.2011.09.026
Expression: AKT1 ---> PTGS2	16531064	10.1016/j.critrevonc.2006.01.003
Expression: AKT1 ---> PTGS2	20883815	10.1016/j.biocel.2010.09.011
Expression: AKT1 ---> PTGS2	20883815	10.1016/j.biocel.2010.09.011
Expression: AKT1 ---> PTGS2	11597520	10.1016/S1471-4914(01)02107-4
Expression: AKT1 ---> PTGS2	11597520	10.1016/S1471-4914(01)02107-4
Expression: AKT1 ---> PTGS2	19298870	10.1016/j.intimp.2009.03.005
Expression: AKT1 ---> PTGS2	20451669	10.1016/j.intimp.2010.04.026
Expression: AKT1 ---> PTGS2	20451669	10.1016/j.intimp.2010.04.026
Expression: AKT1 ---> PTGS2	20840837	10.1016/j.intimp.2010.08.026
Expression: AKT1 ---> PTGS2	20937401	10.1016/j.intimp.2010.09.011
Expression: AKT1 ---> PTGS2		10.1016/j.intimp.2011.02.008
Expression: AKT1 ---> PTGS2		10.1016/j.intimp.2011.02.008
Expression: AKT1 ---> PTGS2		10.1016/j.intimp.2011.02.008
Expression: AKT1 ---> PTGS2	22974512	10.1016/j.ygcen.2012.08.020
Expression: AKT1 ---> PTGS2	23017720	10.1016/B978-0-12-398313-8.00011-7
Expression: AKT1 ---> PTGS2	22561121	10.1016/j.intimp.2012.04.013
Expression: AKT1 ---> PTGS2	22940184	10.1016/j.intimp.2012.08.011
Expression: AKT1 ---> PTGS2	23376120	10.1016/j.bcp.2013.01.020

Expression: AKT1 ---> PTGS2	23376120	10.1016/j.bcp.2013.01.020
Expression: AKT1 ---> PTGS2	23429041	10.1016/j.fct.2013.02.017
Expression: AKT1 ---> PTGS2	23429041	10.1016/j.fct.2013.02.017
Expression: AKT1 ---> PTGS2	23429041	10.1016/j.fct.2013.02.017
Expression: AKT1 ---> PTGS2	23376640	10.1016/j.canlet.2012.12.030
Expression: AKT1 ---> PTGS2	32904445	10.2147/OTT.S26397710.2147/OTT.S263977
Expression: AKT1 ---> PTGS2	32904445	10.2147/OTT.S26397710.2147/OTT.S263977
Expression: AKT1 ---> PTGS2	32904445	10.2147/OTT.S26397710.2147/OTT.S263977
Expression: AKT1 ---> PTGS2	15980035	10.1152/ajplung.00197.2005
Expression: AKT1 ---> PTGS2	15980035	10.1152/ajplung.00197.2005
Expression: AKT1 ---> PTGS2	15980035	10.1152/ajplung.00197.2005
Expression: AKT1 ---> PTGS2	19717552	10.1152/ajplung.00151.200910.1152/ajplung.001
Expression: AKT1 ---> PTGS2	22773695	10.1152/ajplung.00090.201210.1152/ajplung.000
Expression: AKT1 ---> PTGS2	27795708	10.1155/2016/5616807
Expression: AKT1 ---> PTGS2		10.1691/ph.2019.9373
Expression: AKT1 ---> PTGS2		10.1124/jpet.119.257501
Expression: AKT1 ---> PTGS2		10.1124/jpet.119.257501
Expression: AKT1 ---> PTGS2		10.1124/jpet.119.257501
Expression: AKT1 ---> PTGS2		10.1248/bpb.b19-00222
Expression: AKT1 ---> PTGS2	31300611	10.1124/jpet.119.25750110.1124/jpet.119.25750
Expression: AKT1 ---> PTGS2	31300611	10.1124/jpet.119.25750110.1124/jpet.119.25750
Expression: AKT1 ---> PTGS2	15894170	10.1016/j.cellsig.2004.11.015
Expression: AKT1 ---> PTGS2	20883815	10.1016/j.biocel.2010.09.011
Expression: AKT1 ---> PTGS2	23500011	10.1016/j.taap.2013.03.004
Expression: AKT1 ---> PTGS2	17223196	10.1016/j.molimm.2006.12.003
Expression: AKT1 ---> PTGS2	17223196	10.1016/j.molimm.2006.12.003
Expression: AKT1 ---> PTGS2	18771702	10.1016/j.mce.2008.08.004
Expression: AKT1 ---> PTGS2	15964692	10.1016/j.bbcan.2005.04.001
Expression: AKT1 ---> PTGS2	17644291	10.1016/j.jep.2007.05.023
Expression: AKT1 ---> PTGS2	15894170	10.1016/j.cellsig.2004.11.015
Expression: AKT1 ---> PTGS2	19914807	10.1016/j.jdermsci.2009.10.005
Expression: AKT1 ---> PTGS2	23872401	10.1016/j.ejphar.2013.07.010
Expression: AKT1 ---> PTGS2	24016823	10.1016/j.yexcr.2013.08.026
Expression: AKT1 ---> PTGS2	24239652	10.1016/j.taap.2013.11.002
Expression: AKT1 ---> PTGS2	24239652	10.1016/j.taap.2013.11.002
Expression: AKT1 ---> PTGS2	24239652	10.1016/j.taap.2013.11.002
Expression: AKT1 ---> PTGS2	24239652	10.1016/j.taap.2013.11.002
Expression: AKT1 ---> PTGS2	24239652	10.1016/j.taap.2013.11.002
Expression: AKT1 ---> PTGS2	23968725	10.1016/j.toxlet.2013.08.008
Expression: AKT1 ---> PTGS2	23968725	10.1016/j.toxlet.2013.08.008
Expression: AKT1 ---> PTGS2	24467851	10.1016/j.neuropharm.2014.01.022
Expression: AKT1 ---> PTGS2	24296130	10.1016/j.fct.2013.11.035
Expression: AKT1 ---> PTGS2	24365262	10.1016/j.fct.2013.12.011
Expression: AKT1 ---> PTGS2	24612689	10.1016/j.biopha.2014.01.006
Expression: AKT1 ---> PTGS2	24747121	10.1016/j.canlet.2014.03.037
Expression: AKT1 ---> PTGS2	24747121	10.1016/j.canlet.2014.03.037
Expression: AKT1 ---> PTGS2	24910117	10.1016/j.gene.2014.06.006
Expression: AKT1 ---> PTGS2	24910117	10.1016/j.gene.2014.06.006
Expression: AKT1 ---> PTGS2		10.1016/j.jep.2014.10.046
Expression: AKT1 ---> PTGS2	25869400	10.1016/j.mce.2015.04.005
Expression: AKT1 ---> PTGS2	25869400	10.1016/j.mce.2015.04.005
Expression: AKT1 ---> PTGS2	25869400	10.1016/j.mce.2015.04.005
Expression: AKT1 ---> PTGS2	25446583	10.1016/j.jep.2014.10.046
Expression: AKT1 ---> PTGS2	25917838	10.1016/j.jep.2015.04.022

Expression: AKT1 ---> PTGS2	25917838	10.1016/j.jep.2015.04.022
Expression: AKT1 ---> PTGS2	25743463	10.1016/j.bpg.2014.12.003
Expression: AKT1 ---> PTGS2	20124728	
Expression: AKT1 ---> PTGS2	16998585	
Expression: AKT1 ---> PTGS2	16945991	
Expression: AKT1 ---> PTGS2	16740977	
Expression: AKT1 ---> PTGS2	11733561	
Expression: AKT1 ---> PTGS2	19933775	
Expression: AKT1 ---> PTGS2	19933775	
Expression: AKT1 ---> PTGS2	19933775	
Expression: AKT1 ---> PTGS2	19933775	
Expression: AKT1 ---> PTGS2	19933775	
Expression: AKT1 ---> PTGS2	18326857	
Expression: AKT1 ---> PTGS2	18326857	
Expression: AKT1 ---> PTGS2	18326857	
Expression: AKT1 ---> PTGS2	16982614	
Expression: AKT1 ---> PTGS2	16540596	
Expression: AKT1 ---> PTGS2	16540596	
Expression: AKT1 ---> PTGS2	16540596	
Expression: AKT1 ---> PTGS2	15322248	
Expression: AKT1 ---> PTGS2	15322248	
Expression: AKT1 ---> PTGS2	15322248	
Expression: AKT1 ---> PTGS2	15322248	
Expression: AKT1 ---> PTGS2	15322248	
Expression: AKT1 ---> PTGS2	15322248	
Expression: AKT1 ---> PTGS2	15322248	
Expression: AKT1 ---> PTGS2	15322248	
Expression: AKT1 ---> PTGS2	15322248	
Expression: AKT1 ---> PTGS2	15322248	
Expression: AKT1 ---> PTGS2	15117884	
Expression: AKT1 ---> PTGS2	15016316	
Expression: AKT1 ---> PTGS2	15016316	
Expression: AKT1 ---> PTGS2	15016316	
Expression: AKT1 ---> PTGS2	15016316	
Expression: AKT1 ---> PTGS2	15016316	
Expression: AKT1 ---> PTGS2	15016316	
Expression: AKT1 ---> PTGS2	15016316	
Expression: AKT1 ---> PTGS2	12960176	
Expression: AKT1 ---> PTGS2	12865424	
Expression: AKT1 ---> PTGS2	12865424	
Expression: AKT1 ---> PTGS2	12865424	
Expression: AKT1 ---> PTGS2	12865424	
Expression: AKT1 ---> PTGS2	12372818	
Expression: AKT1 ---> PTGS2	12072439	
Expression: AKT1 ---> PTGS2	10930401	
Expression: AKT1 ---> PTGS2	10930401	
Expression: AKT1 ---> PTGS2	10930401	
Expression: AKT1 ---> PTGS2	10930401	
Expression: AKT1 ---> PTGS2	10930401	
Expression: AKT1 ---> PTGS2	19218449	
Expression: AKT1 ---> PTGS2	17680773	
Expression: AKT1 ---> PTGS2	17272774	
Expression: AKT1 ---> PTGS2	15653566	
Expression: AKT1 ---> PTGS2	15653566	

Expression: AKT1 ---> PTGS2	15653566	
Expression: AKT1 ---> PTGS2	15653566	
Expression: AKT1 ---> PTGS2	11742537	
Expression: AKT1 ---> PTGS2	18703638	
Expression: AKT1 ---> PTGS2	17287275	
Expression: AKT1 ---> PTGS2	22805054	10.1158/1940-6207.CAPR-11-0397
Expression: AKT1 ---> PTGS2	22767315	10.3892/ijmm.2012.1052
Expression: AKT1 ---> PTGS2	23037157	10.1248/bpb.b12-00037
Expression: AKT1 ---> PTGS2	22747786	10.1186/1742-2094-9-152
Expression: AKT1 ---> PTGS2	22962522	10.3164/jcbn.11-94
Expression: AKT1 ---> PTGS2	22962522	10.3164/jcbn.11-94
Expression: AKT1 ---> PTGS2	22962522	10.3164/jcbn.11-94
Expression: AKT1 ---> PTGS2	23229294	10.3892/or.2012.2158
Expression: AKT1 ---> PTGS2	21877194	10.1007/s12032-011-0056-0
Expression: AKT1 ---> PTGS2	23254481	10.3892/ijmm.2012.1216
Expression: AKT1 ---> PTGS2	23243000	10.1091/mbc.E12-08-0637
Expression: AKT1 ---> PTGS2	20954072	10.1080/15287394.2010.511551
Expression: AKT1 ---> PTGS2	21525012	10.1074/jbc.M110.207571
Expression: AKT1 ---> PTGS2	21776820	10.3727/096504011X13021877989711
Expression: AKT1 ---> PTGS2	21776820	10.3727/096504011X13021877989711
Expression: AKT1 ---> PTGS2	22109885	10.1210/en.2011-1438
Expression: AKT1 ---> PTGS2	22109885	10.1210/en.2011-1438
Expression: AKT1 ---> PTGS2	22280146	10.1089/jmf.2011.1701
Expression: AKT1 ---> PTGS2	19517063	10.1007/s10637-009-9274-0
Expression: AKT1 ---> PTGS2	19517063	10.1007/s10637-009-9274-0
Expression: AKT1 ---> PTGS2	20224909	10.1007/s11033-010-0035-0
Expression: AKT1 ---> PTGS2	20446020	10.1007/s11010-010-0469-7
Expression: AKT1 ---> PTGS2	20446020	10.1007/s11010-010-0469-7
Expression: AKT1 ---> PTGS2	20446020	10.1007/s11010-010-0469-7
Expression: AKT1 ---> PTGS2	21140284	10.1007/s11010-010-0682-4
Expression: AKT1 ---> PTGS2	21745389	10.1186/1471-2199-12-29
Expression: AKT1 ---> PTGS2	21745389	10.1186/1471-2199-12-29
Expression: AKT1 ---> PTGS2	21745389	10.1186/1471-2199-12-29
Expression: AKT1 ---> PTGS2	21745389	10.1186/1471-2199-12-29
Expression: AKT1 ---> PTGS2	21745389	10.1186/1471-2199-12-29
Expression: AKT1 ---> PTGS2	21745389	10.1186/1471-2199-12-29
Expression: AKT1 ---> PTGS2	21745389	10.1186/1471-2199-12-29
Expression: AKT1 ---> PTGS2	21745389	10.1186/1471-2199-12-29
Expression: AKT1 ---> PTGS2	21745389	10.1186/1471-2199-12-29
Expression: AKT1 ---> PTGS2	21813027	10.1186/1471-2407-11-334
Expression: AKT1 ---> PTGS2	21813027	10.1186/1471-2407-11-334
Expression: AKT1 ---> PTGS2	23146919	10.1097/MPA.0b013e318269d076
Expression: AKT1 ---> PTGS2	23515290	10.1210/en.2012-2074
Expression: AKT1 ---> PTGS2	23747884	10.2741/4180
Expression: AKT1 ---> PTGS2	23859016	10.1139/bcb-2012-0077
Expression: AKT1 ---> PTGS2	23272913	10.2174/18715206113139990119
Expression: AKT1 ---> PTGS2	24174713	10.1155/2013/964012
Expression: AKT1 ---> PTGS2	24174713	10.1155/2013/964012
Expression: AKT1 ---> PTGS2	21533119	10.1371/journal.pone.0018823
Expression: AKT1 ---> PTGS2	21533119	10.1371/journal.pone.0018823
Expression: AKT1 ---> PTGS2	21533119	10.1371/journal.pone.0018823
Expression: AKT1 ---> PTGS2	21533119	10.1371/journal.pone.0018823
Expression: AKT1 ---> PTGS2		10.1194/jlr.R037762
Expression: AKT1 ---> PTGS2		10.1194/jlr.R037762

Expression: AKT1 ---> PTGS2	24092824	10.1210/jc.2013-2662
Expression: AKT1 ---> PTGS2	24220687	10.3892/or.2013.2843
Expression: AKT1 ---> PTGS2	24395886	10.1084/jem.20131080
Expression: AKT1 ---> PTGS2	24395886	10.1084/jem.20131080
Expression: AKT1 ---> PTGS2	24395886	10.1084/jem.20131080
Expression: AKT1 ---> PTGS2		10.1194/jlr.R039297
Expression: AKT1 ---> PTGS2	24377653	10.1080/01635581.2014.864420
Expression: AKT1 ---> PTGS2	24329111	10.2174/1389200214666131211155613
Expression: AKT1 ---> PTGS2	24634601	10.4196/kjpp.2014.18.1.79
Expression: AKT1 ---> PTGS2	24400871	10.1089/thy.2013.0614
Expression: AKT1 ---> PTGS2	24969217	10.1530/ERC-13-0450
Expression: AKT1 ---> PTGS2	24969217	10.1530/ERC-13-0450
Expression: AKT1 ---> PTGS2	24969217	10.1530/ERC-13-0450
Expression: AKT1 ---> PTGS2	24969217	10.1530/ERC-13-0450
Expression: AKT1 ---> PTGS2	24969217	10.1530/ERC-13-0450
Expression: AKT1 ---> PTGS2	24969217	10.1530/ERC-13-0450
Expression: AKT1 ---> PTGS2	24969217	10.1530/ERC-13-0450
Expression: AKT1 ---> PTGS2	24969217	10.1530/ERC-13-0450
Expression: AKT1 ---> PTGS2	24969217	10.1530/ERC-13-0450
Expression: AKT1 ---> PTGS2		10.1194/jlr.M047738
Expression: AKT1 ---> PTGS2	24062397	10.1200/JCO.2013.50.0322
Expression: AKT1 ---> PTGS2	25177723	10.1155/2014/397430
Expression: AKT1 ---> PTGS2		
Expression: AKT1 ---> PTGS2	25553470	10.2741/4328
Expression: AKT1 ---> PTGS2		10.1530/JOE-14-0470
Expression: AKT1 ---> PTGS2	22442157	10.1152/ajpgi.00460.2011
Expression: AKT1 ---> PTGS2	22773695	10.1152/ajplung.00090.2012
Expression: AKT1 ---> PTGS2	22773695	10.1152/ajplung.00090.2012
Expression: AKT1 ---> PTGS2	22773695	10.1152/ajplung.00090.2012
Expression: AKT1 ---> PTGS2	22773695	10.1152/ajplung.00090.2012
Expression: AKT1 ---> PTGS2	22773695	10.1152/ajplung.00090.2012
Expression: AKT1 ---> PTGS2	22773695	10.1152/ajplung.00090.2012
Expression: AKT1 ---> PTGS2	22773695	10.1152/ajplung.00090.2012
Expression: AKT1 ---> PTGS2	22613973	10.1159/000171028
Expression: AKT1 ---> PTGS2	22613973	10.1159/000171028
Expression: AKT1 ---> PTGS2	20943963	10.1128/JVI.01065-10
Expression: AKT1 ---> PTGS2	23899566	10.1152/physrev.00004.2012
Expression: AKT1 ---> PTGS2	25710724	10.3390/ijms16034453
Expression: AKT1 ---> PTGS2	25710724	10.3390/ijms16034453
Expression: AKT1 ---> PTGS2	26159723	10.1186/s12943-015-0406-1
Expression: AKT1 ---> PTGS2	11289146	
Expression: AKT1 ---> PTGS2	11289146	
Expression: AKT1 ---> PTGS2	15067356	
Expression: AKT1 ---> PTGS2	15067356	
Expression: AKT1 ---> PTGS2	15016316	
Expression: AKT1 ---> PTGS2	15322248	
Expression: AKT1 ---> PTGS2	15322248	
Expression: AKT1 ---> PTGS2	15322248	
Expression: AKT1 ---> PTGS2	16044407	
Expression: AKT1 ---> PTGS2	16044407	
Expression: AKT1 ---> PTGS2	16508949	
Expression: AKT1 ---> PTGS2	16508949	
Expression: AKT1 ---> PTGS2	16508949	
Expression: AKT1 ---> PTGS2	17143555	

Expression: AKT1 ---> PTGS2	17223196	
Expression: AKT1 ---> PTGS2	17272774	
Expression: AKT1 ---> PTGS2	17472471	
Expression: AKT1 ---> PTGS2	17825254	
Expression: AKT1 ---> PTGS2	18097584	
Expression: AKT1 ---> PTGS2	18192694	10.1093/carcin/bgm299
Expression: AKT1 ---> PTGS2	18771702	10.1016/j.mce.2008.08.004
Expression: AKT1 ---> PTGS2	19118901	10.1016/j.molimm.2008.11.006
Expression: AKT1 ---> PTGS2	19106818	10.1097/SHK.0b013e31819940cb
Expression: AKT1 ---> PTGS2	19616084	10.1016/j.toxlet.2009.07.012
Expression: AKT1 ---> PTGS2	19616084	10.1016/j.toxlet.2009.07.012
Expression: AKT1 ---> PTGS2	19781629	10.1016/j.cellsig.2009.09.014
Expression: AKT1 ---> PTGS2	19781629	10.1016/j.cellsig.2009.09.014
Expression: AKT1 ---> PTGS2	20333648	10.1002/jcp.22133
Expression: AKT1 ---> PTGS2	20451669	10.1016/j.intimp.2010.04.026
Expression: AKT1 ---> PTGS2	21678415	10.1002/jcp.22880
Expression: AKT1 ---> PTGS2	22390933	10.1016/j.bbrc.2012.02.103
Expression: AKT1 ---> PTGS2	22613973	10.1159/000171028
Expression: AKT1 ---> PTGS2	22747786	10.1186/1742-2094-9-152
Expression: AKT1 ---> PTGS2	23254481	10.3892/ijmm.2012.1216
Expression: AKT1 ---> PTGS2	24016823	10.1016/j.yexcr.2013.08.026
Expression: AKT1 ---> PTGS2	24296130	10.1016/j.fct.2013.11.035
Expression: AKT1 ---> PTGS2	24486459	10.1016/j.neuint.2014.01.010
Expression: AKT1 ---> PTGS2	24859472	10.3892/mmr.2014.2259
Expression: AKT1 ---> PTGS2	24969217	10.1530/ERC-13-0450
Expression: AKT1 ---> PTGS2	25454762	10.1016/j.nutres.2014.10.002
Expression: AKT1 ---> PTGS2	25869400	10.1016/j.mce.2015.04.005
Expression: AKT1 ---> PTGS2	25869400	10.1016/j.mce.2015.04.005
Expression: AKT1 ---> PTGS2	26522220	10.1016/j.bbrc.2015.10.133
Expression: AKT1 ---> PTGS2	26546510	10.1016/j.expneurol.2015.11.001
Expression: AKT1 ---> PTGS2	26712086	10.1016/j.clon.2015.11.008
Expression: AKT1 ---> PTGS2	26341413	10.1016/j.intimp.2015.08.028
Expression: AKT1 ---> PTGS2	26238650	10.3892/ijo.2015.3110
Expression: AKT1 ---> PTGS2	26238650	10.3892/ijo.2015.3110
Expression: AKT1 ---> PTGS2	26238650	10.3892/ijo.2015.3110
Expression: AKT1 ---> PTGS2	26604433	10.1155/2015/831059
Expression: AKT1 ---> PTGS2	26608500	10.1007/s00011-015-0895-910.1007/s00011-015-0
Expression: AKT1 ---> PTGS2	25665524	10.1038/ncomms7221
Expression: AKT1 ---> PTGS2		
Expression: AKT1 ---> PTGS2	26898122	10.1016/j.lfs.2016.02.060
Expression: AKT1 ---> PTGS2	1348	10.1016/j.clon.2015.11.008
Expression: AKT1 ---> PTGS2		10.1074/jbc.M115.668277
Expression: AKT1 ---> PTGS2		10.1074/jbc.M115.668277
Expression: AKT1 ---> PTGS2		10.1074/jbc.M115.668277
Expression: AKT1 ---> PTGS2		10.1074/jbc.M115.668277
Expression: AKT1 ---> PTGS2		10.1074/jbc.M115.668277
Expression: AKT1 ---> PTGS2		
Expression: AKT1 ---> PTGS2		
Expression: AKT1 ---> PTGS2	27703516	10.3892/etm.2016.3642
Expression: AKT1 ---> PTGS2	28585217	10.1007/978-3-319-48382-5_25
Expression: AKT1 ---> PTGS2	28647573	10.1016/j.cellsig.2017.06.01210.1016/j.cellsi
Expression: AKT1 ---> PTGS2	29031211	10.1016/j.jphotobiol.2017.10.00610.1016/j.jph
Expression: AKT1 ---> PTGS2	29154739	10.1017/S002202991700067X10.1017/S0022029917O
Expression: AKT1 ---> PTGS2	29068698	10.1080/01635581.2017.1367940

Expression: AKT1 ---> PTGS2	29894800	10.1016/j.tiv.2018.06.00810.1016/j.tiv.2018.0
Expression: AKT1 ---> PTGS2	29906814	10.1055/a-0637-993610.1055/a-0637-9936
Expression: AKT1 ---> PTGS2		10.1097/CMR.0000000000000368
Expression: AKT1 ---> PTGS2	29962177	10.4077/CJP.2018.BAG562
Expression: AKT1 ---> PTGS2	29906814	10.1055/a-0637-9936
Expression: AKT1 ---> PTGS2	30406888	10.1007/s11033-018-4378-2
Expression: AKT1 ---> PTGS2	30806292	10.1615/JEnvironPatholToxicolOncol.2018026684
Expression: AKT1 ---> PTGS2	30877761	10.1093/abbs/gmz01410.1093/abbs/gmz014
Expression: AKT1 ---> PTGS2	31300611	10.1124/jpet.119.25750110.1124/jpet.119.25750
Expression: AKT1 ---> PTGS2	31300611	10.1124/jpet.119.25750110.1124/jpet.119.25750
Expression: AKT1 ---> PTGS2	31377156	10.1016/j.ejphar.2019.17258610.1016/j.ejphar.
Expression: AKT1 ---> PTGS2		10.1042/BSR20191255
Expression: AKT1 ---> PTGS2		10.1186/s13058-019-1224-y
Expression: AKT1 ---> PTGS2		10.1007/s00403-019-01968-z
Expression: AKT1 ---> PTGS2		10.2174/1568009620666200101094736
Expression: AKT1 ---> PTGS2		10.1155/2020/7642019
Expression: AKT1 ---> PTGS2	33081347	10.3390/ijms2120767510.3390/ijms21207675
Expression: AKT1 ---> PTGS2	33081347	10.3390/ijms21207675
Expression: AKT1 ---> PTGS2	33132637	10.3748/wjg.v26.i38.582210.3748/wjg.v26.i38.5
Expression: AKT1 ---> PTGS2	32859885	10.4103/CJP.CJP_44_20
Expression: AKT1 ---> PTGS2		10.5603/FHC.a2020.0025
Expression: AKT1 ---> PTGS2	32981501	10.2174/1389450121666200925150022
Expression: AKT1 ---> PTGS2	33167824	10.2174/0929867327666201109122025
Expression: AKT1 ---> PTGS2	35130451	10.1128/iai.00672-21
Expression: AKT1 ---> PTGS2	35038134	10.1007/s12640-022-00473-y
Expression: AKT1 ---> PTGS2	35575922	10.1007/978-981-19-1592-5_6
Expression: AKT1 ---> PTGS2	36430378	10.3390/ijms232213892
Expression: AKT1 ---> PTGS2	36552163	10.3390/brainsci12121703
positive Regulation: GPER1 --+> A	27301862	10.1182/blood-2016-03-708263
positive Regulation: GPER1 --+> A	29382535	10.1016/j.ejphar.2018.01.040
positive Regulation: GPER1 --+> A	29382535	10.1016/j.ejphar.2018.01.040
positive Regulation: GPER1 --+> A	30641053	10.1053/j.gastro.2019.01.010
positive Regulation: GPER1 --+> A	25960081	10.1016/j.yhbeh.2015.05.001
positive Regulation: GPER1 --+> A	12904304	10.1074/jbc.M303180200
positive Regulation: GPER1 --+> A	30905784	10.1016/j.lfs.2019.03.049
positive Regulation: GPER1 --+> A	31891720	10.1016/j.lfs.2019.117211
positive Regulation: GPER1 --+> A	26850467	10.1016/j.steroids.2016.01.021
positive Regulation: GPER1 --+> A	27940299	10.1016/j.mce.2016.12.007
positive Regulation: GPER1 --+> A	27940299	10.1016/j.mce.2016.12.007
positive Regulation: GPER1 --+> A	33444670	10.1016/j.mce.2021.111158
positive Regulation: GPER1 --+> A	29526689	10.1016/j.neuroscience.2018.02.048
positive Regulation: GPER1 --+> A	28595943	10.1016/j.jsbmb.2017.05.005
positive Regulation: GPER1 --+> A	28595943	10.1016/j.jsbmb.2017.05.005
positive Regulation: GPER1 --+> A	32289430	10.1016/j.jsbmb.2020.105671
positive Regulation: GPER1 --+> A	27423484	10.1016/j.jphs.2016.06.005
positive Regulation: GPER1 --+> A	26842883	10.1016/j.molonc.2016.01.002
positive Regulation: GPER1 --+> A	26842883	10.1016/j.molonc.2016.01.002
positive Regulation: GPER1 --+> A	31706983	10.1016/j.chembiol.2019.10.009
positive Regulation: GPER1 --+> AKT1		10.1016/j.endmts.2021.100080
positive Regulation: GPER1 --+> A	23142288	10.1016/j.canlet.2012.10.037
positive Regulation: GPER1 --+> A	23545157	10.1016/j.mce.2013.03.018
positive Regulation: GPER1 --+> A	24240011	10.1016/j.steroids.2013.11.004
positive Regulation: GPER1 --+> A	23911898	10.1016/j.mce.2013.07.026
positive Regulation: GPER1 --+> A	23907016	10.1016/j.jsbmb.2013.07.011

positive Regulation: GPER1 --+> A23907016 10.1016/j.jsbmb.2013.07.011
positive Regulation: GPER1 --+> A23907016 10.1016/j.jsbmb.2013.07.011
positive Regulation: GPER1 --+> A23907016 10.1016/j.jsbmb.2013.07.011
positive Regulation: GPER1 --+> A24594140 10.1016/j.mce.2014.01.024
positive Regulation: GPER1 --+> A24726471 10.1016/j.neurobiolaging.2014.03.017
positive Regulation: GPER1 --+> A24835924 10.1016/j.mce.2014.05.005
positive Regulation: GPER1 --+> A25892506 10.1016/j.neuropharm.2015.04.006
positive Regulation: GPER1 --+> A26189910 10.1016/j.jsbmb.2015.06.014
positive Regulation: GPER1 --+> A26497404 10.1016/j.yjmcc.2015.10.024
positive Regulation: GPER1 --+> A26432358 10.1016/j.mce.2015.09.032
positive Regulation: GPER1 --+> A12904304
positive Regulation: GPER1 --+> A18635483
positive Regulation: GPER1 --+> A17878225
positive Regulation: GPER1 --+> A27193386 10.1007/s40263-016-0343-6
positive Regulation: GPER1 --+> A27407175 10.1042/BSR20160134
positive Regulation: GPER1 --+> A27481507 10.1007/s00441-016-2466-y
positive Regulation: GPER1 --+> A27796870 10.1007/s12031-016-0856-y
positive Regulation: GPER1 --+> A27796870 10.1007/s12031-016-0856-y
positive Regulation: GPER1 --+> A28535016 10.3892/or.2017.5649
positive Regulation: GPER1 --+> A28535016 10.3892/or.2017.5649
positive Regulation: GPER1 --+> A28535016 10.3892/or.2017.5649
positive Regulation: GPER1 --+> A28535016 10.3892/or.2017.5649
positive Regulation: GPER1 --+> A29436590 10.3892/ijmm.2018.3470
positive Regulation: GPER1 --+> A29167148 10.1152/ajpcell.00060.2017
positive Regulation: GPER1 --+> A29167148 10.1152/ajpcell.00060.2017
positive Regulation: GPER1 --+> A25588050 10.4161/19336918.2014.990781
positive Regulation: GPER1 --+> A25588050 10.4161/19336918.2014.990781
positive Regulation: GPER1 --+> A25588050 10.4161/19336918.2014.990781
positive Regulation: GPER1 --+> A29566418 10.1055/s-0044-100920
positive Regulation: GPER1 --+> A30173650 10.2174/1871520618666180830162517
positive Regulation: GPER1 --+> A33097048 10.1186/s12974-020-01998-9
positive Regulation: GPER1 --+> A32180008 10.1007/s00709-020-01488-y
positive Regulation: GPER1 --+> A34098945 10.1186/s12935-021-02003-w
positive Regulation: GPER1 --+> A34182538 10.18632/aging.203145
positive Regulation: GPER1 --+> A22922893 10.3892/ijo.2012.1591
positive Regulation: GPER1 --+> A22075757 10.3892/or.2011.1536
positive Regulation: GPER1 --+> A19911269 10.1007/s10549-009-0624-6
positive Regulation: GPER1 --+> A19911269 10.1007/s10549-009-0624-6
positive Regulation: GPER1 --+> A23756388
positive Regulation: GPER1 --+> A23840305 10.1371/journal.pone.0064771
positive Regulation: GPER1 --+> A23840305 10.1371/journal.pone.0064771
positive Regulation: GPER1 --+> A25744245 10.1007/s12032-015-0558-2
positive Regulation: GPER1 --+> A26183213 10.1242/dmm.021071
positive Regulation: GPER1 --+> A29365274 10.1152/AJPCELL.00152.2017
positive Regulation: GPER1 --+> A34445359 10.3390/ijms22168654
positive Regulation: GPER1 --+> A34576301 10.3390/ijms221810138
positive Regulation: GPER1 --+> A32873626 10.1158/1541-7786.MCR-20-017310.1158/1541-778
positive Regulation: GPER1 --+> A23840305 10.1371/journal.pone.006477110.1371/journal.p
positive Regulation: GPER1 --+> A852 10.1016/j.jphs.2016.06.005
positive Regulation: GPER1 --+> AKT1 10.1016/j.molonc.2016.01.002
positive Regulation: GPER1 --+> AKT1 10.1016/j.molonc.2016.01.002
positive Regulation: GPER1 --+> AKT1 10.1042/BSR20160134
positive Regulation: GPER1 --+> A26183213 10.1242/dmm.021071
positive Regulation: GPER1 --+> A26356837 10.1371/journal.pone.0135988

positive Regulation: GPER1 --+> /26356837 10.1371/journal.pone.0135988
positive Regulation: GPER1 --+> /26356837 10.1371/journal.pone.0135988
positive Regulation: GPER1 --+> /21278053 10.1093/toxsci/kfr01910.1093/toxsci/kfr019
positive Regulation: GPER1 --+> /22075757 10.3892/or.2011.153610.3892/or.2011.1536
positive Regulation: GPER1 --+> /23235274 10.1097/IGC.0b013e31827912b810.1097/IGC.0b013
positive Regulation: GPER1 --+> /23545157 10.1016/j.mce.2013.03.01810.1016/j.mce.2013.0
positive Regulation: GPER1 --+> /23907016 10.1016/j.jsbmb.2013.07.01110.1016/j.jsbmb.20
positive Regulation: GPER1 --+> /26850467 10.1016/j.steroids.2016.01.02110.1016/j.sterc
positive Regulation: GPER1 --+> /27940299 10.1016/j.mce.2016.12.00710.1016/j.mce.2016.1
positive Regulation: GPER1 --+> /28535016 10.3892/or.2017.564910.3892/or.2017.5649
positive Regulation: GPER1 --+> /29382535 10.1016/j.ejphar.2018.01.04010.1016/j.ejphar.
positive Regulation: GPER1 --+> /30195206 10.1016/j.ecoenv.2018.08.10210.1016/j.ecoenv.
positive Regulation: GPER1 --+> /23235274 10.1097/IGC.0b013e31827912b8
positive Regulation: GPER1 --+> /23235274 10.1097/IGC.0b013e31827912b8
positive Regulation: GPER1 --+> /23142288 10.1016/j.canlet.2012.10.037
positive Regulation: GPER1 --+> /19477925
positive Regulation: GPER1 --+> /27222231 10.1177/1933719116649696
positive Regulation: GPER1 --+> /21272573 10.1016/j.ejphar.2011.01.016
positive Regulation: GPER1 --+> /25892506 10.1016/j.neuropharm.2015.04.006
positive Regulation: GPER1 --+> /21673097 10.1210/en.2011-0091
positive Regulation: GPER1 --+> /22075757 10.3892/or.2011.1536
positive Regulation: GPER1 --+> AKT1
positive Regulation: GPER1 --+> AKT1 10.12659/MSM.912986
positive Regulation: GPER1 --+> /32873626 10.1158/1541-7786.MCR-20-017310.1158/1541-778
positive Regulation: GPER1 --+> /35158255 10.1016/j.ecoenv.2022.11329010.1016/j.ecoenv.
positive Regulation: GPER1 --+> /34860303 10.1007/s10522-021-09945-8
positive Regulation: GPER1 --+> /35560348 10.1096/fasebj.2022.36.S1.R512210.1096/fasebj
positive Regulation: GPER1 --+> /35659445 10.1080/15384101.2022.2083708
positive Regulation: GPER1 --+> /35659445 10.1080/15384101.2022.2083708
positive Regulation: GPER1 --+> /36558071 10.3390/molecules27248943
positive Regulation: GPER1 --+> /36558071 10.3390/molecules27248943
positive Regulation: GPER1 --+> /36558071 10.3390/molecules27248943
positive Regulation: PPARG --+> (22684020 10.1016/j.freeradbiomed.2012.05.041
positive Regulation: PPARG --+> (22684020 10.1016/j.freeradbiomed.2012.05.041
positive Regulation: PPARG --+> (32373170 10.1155/2020/6452182
positive Regulation: PPARG --+> (32373170 10.1155/2020/6452182
positive Regulation: PPARG --+> (32373170 10.1155/2020/6452182
positive Regulation: PPARG --+> (32373170 10.1155/2020/6452182
positive Regulation: PPARG --+> (32373170 10.1155/2020/6452182
positive Regulation: PPARG --+> (32373170 10.1155/2020/6452182
positive Regulation: PPARG --+> (32373170 10.1155/2020/6452182
positive Regulation: PPARG --+> (32373170 10.1155/2020/6452182
positive Regulation: PPARG --+> (32373170 10.1155/2020/6452182
positive Regulation: PPARG --+> (32373170 10.1155/2020/6452182
positive Regulation: PPARG --+> (32373170 10.1155/2020/6452182
positive Regulation: PPARG --+> (34054937 10.1155/2021/5525091
positive Regulation: PPARG --+> (25333644 10.3892/ijo.2014.2709
positive Regulation: PPARG --+> (25333644 10.3892/ijo.2014.2709
positive Regulation: PPARG --+> (32373170 10.1155/2020/645218210.1155/2020/6452182
positive Regulation: PPARG --+> (32373170 10.1155/2020/645218210.1155/2020/6452182
positive Regulation: PPARG --+> (34054937 10.1155/2021/552509110.1155/2021/5525091
positive Regulation: PPARG --+> (23964924 10.1089/ars.2013.5295
positive Expression: PPARG --+> (24873723 10.1016/j.freeradbiomed.2014.05.020
positive Expression: PPARG --+> (24873723 10.1016/j.freeradbiomed.2014.05.020

positive Expression: PPARG --+> (18332871
positive Expression: PPARG --+> (18332871
positive Expression: PPARG --+> (18332871
positive Expression: PPARG --+> (18332871
positive Expression: PPARG --+> (16306131
positive Expression: PPARG --+> GCLC
Regulation: SFRP1 ---> AKT1 11278246 10.1074/jbc.C000880200
Regulation: SFRP1 ---> AKT1 11278246
Regulation: SFRP1 ---> AKT1 15920021
Regulation: SFRP1 ---> AKT1
Regulation: PTGS2 ---> AKT1 27916554 10.1016/j.ejphar.2016.11.052
Regulation: PTGS2 ---> AKT1 29750896 10.1016/j.yexcr.2018.05.008
Regulation: PTGS2 ---> AKT1 25064845 10.1016/j.febslet.2014.07.012
Regulation: PTGS2 ---> AKT1 25064845 10.1016/j.febslet.2014.07.012
Regulation: PTGS2 ---> AKT1 21561856 10.1074/jbc.M111.222349
Regulation: PTGS2 ---> AKT1 21561856 10.1074/jbc.M111.222349
Regulation: PTGS2 ---> AKT1 16966336 10.1074/jbc.M600135200
Regulation: PTGS2 ---> AKT1 20042609 10.1074/jbc.M109.027086
Regulation: PTGS2 ---> AKT1 28407559 10.1016/j.molimm.2017.04.004
Regulation: PTGS2 ---> AKT1 28479391 10.1016/j.fct.2017.05.006
Regulation: PTGS2 ---> AKT1 32640310 10.1016/j.gene.2020.144932
Regulation: PTGS2 ---> AKT1 21551001 10.1093/annonc/mdr266
Regulation: PTGS2 ---> AKT1 29625858 10.1016/j.cyto.2018.03.032
Regulation: PTGS2 ---> AKT1 31005030 10.1016/j.drup.2019.04.001
Regulation: PTGS2 ---> AKT1 33152664 10.1016/j.redox.2020.101774
Regulation: PTGS2 ---> AKT1 33152664 10.1016/j.redox.2020.101774
Regulation: PTGS2 ---> AKT1 33152664 10.1016/j.redox.2020.101774
Regulation: PTGS2 ---> AKT1 33152664 10.1016/j.redox.2020.101774
Regulation: PTGS2 ---> AKT1 21689625 10.1016/j.ajpath.2011.04.004
Regulation: PTGS2 ---> AKT1 21147091 10.1016/j.ejphar.2010.11.022
Regulation: PTGS2 ---> AKT1 18722373 10.1053/j.gastro.2008.07.012
Regulation: PTGS2 ---> AKT1 17418864 10.1016/j.jss.2007.01.039
Regulation: PTGS2 ---> AKT1 17198778 10.1016/j.jtcvs.2006.07.042
Regulation: PTGS2 ---> AKT1 18343442 10.1016/j.juro.2007.12.037
Regulation: PTGS2 ---> AKT1 16360450 10.1016/j.urology.2005.06.076
Regulation: PTGS2 ---> AKT1 15179620 10.1053/j.seminoncol.2004.03.040
Regulation: PTGS2 ---> AKT1 18812189 10.1016/j.imlet.2008.08.004
Regulation: PTGS2 ---> AKT1 18375246 10.1016/j.arcmed.2007.12.005
Regulation: PTGS2 ---> AKT1 12618325 10.1016/S0304-3835(02)00524-4
Regulation: PTGS2 ---> AKT1 18790560 10.1016/j.canlet.2008.08.012
Regulation: PTGS2 ---> AKT1 22546756 10.1016/j.mehy.2012.04.012
Regulation: PTGS2 ---> AKT1 18571350 10.1016/j.jep.2008.05.001
Regulation: PTGS2 ---> AKT1 18571350 10.1016/j.jep.2008.05.001
Regulation: PTGS2 ---> AKT1 20362052 10.1016/j.cellsig.2010.03.014
Regulation: PTGS2 ---> AKT1 16002278 10.1016/j.ejca.2005.04.013
Regulation: PTGS2 ---> AKT1 18178094 10.1016/j.nbd.2007.11.010
Regulation: PTGS2 ---> AKT1 15661561 10.1016/j.critrevonc.2004.10.001
Regulation: PTGS2 ---> AKT1 16531064 10.1016/j.critrevonc.2006.01.003
Regulation: PTGS2 ---> AKT1 21807123 10.1016/j.intimp.2011.07.011
Regulation: PTGS2 ---> AKT1 10.1016/j.jecm.2010.08.003
Regulation: PTGS2 ---> AKT1 20854947 10.1016/j.bone.2010.09.020
Regulation: PTGS2 ---> AKT1 20854947 10.1016/j.bone.2010.09.020
Regulation: PTGS2 ---> AKT1 20854947 10.1016/j.bone.2010.09.020
Regulation: PTGS2 ---> AKT1 20854947 10.1016/j.bone.2010.09.020

Regulation: PTGS2 ---> AKT1	20854947	10.1016/j.bone.2010.09.020
Regulation: PTGS2 ---> AKT1	20854947	10.1016/j.bone.2010.09.020
Regulation: PTGS2 ---> AKT1	20854947	10.1016/j.bone.2010.09.020
Regulation: PTGS2 ---> AKT1	20854947	10.1016/j.bone.2010.09.020
Regulation: PTGS2 ---> AKT1	20854947	10.1016/j.bone.2010.09.020
Regulation: PTGS2 ---> AKT1	20854947	10.1016/j.bone.2010.09.020
Regulation: PTGS2 ---> AKT1	20854947	10.1016/j.bone.2010.09.020
Regulation: PTGS2 ---> AKT1	20854947	10.1016/j.bone.2010.09.020
Regulation: PTGS2 ---> AKT1	23422333	10.1016/j.jep.2013.02.001
Regulation: PTGS2 ---> AKT1	23563277	10.1016/j.pharmthera.2013.04.001
Regulation: PTGS2 ---> AKT1	24465039	10.1016/j.curtheres.2013.06.001
Regulation: PTGS2 ---> AKT1	23907017	10.1016/j.jsbmb.2013.07.008
Regulation: PTGS2 ---> AKT1	24296130	10.1016/j.fct.2013.11.035
Regulation: PTGS2 ---> AKT1	25770423	10.1016/j.bbrc.2015.03.008
Regulation: PTGS2 ---> AKT1	25770423	10.1016/j.bbrc.2015.03.008
Regulation: PTGS2 ---> AKT1	25770423	10.1016/j.bbrc.2015.03.008
Regulation: PTGS2 ---> AKT1	25770423	10.1016/j.bbrc.2015.03.008
Regulation: PTGS2 ---> AKT1	25770423	10.1016/j.bbrc.2015.03.008
Regulation: PTGS2 ---> AKT1	25770423	10.1016/j.bbrc.2015.03.008
Regulation: PTGS2 ---> AKT1	20022932	
Regulation: PTGS2 ---> AKT1	21078168	
Regulation: PTGS2 ---> AKT1	21078168	
Regulation: PTGS2 ---> AKT1	20042609	
Regulation: PTGS2 ---> AKT1	19052170	
Regulation: PTGS2 ---> AKT1	16966336	
Regulation: PTGS2 ---> AKT1	15016316	
Regulation: PTGS2 ---> AKT1	19099539	
Regulation: PTGS2 ---> AKT1	16800815	
Regulation: PTGS2 ---> AKT1	20661229	
Regulation: PTGS2 ---> AKT1	17285134	
Regulation: PTGS2 ---> AKT1	16160697	
Regulation: PTGS2 ---> AKT1	16396944	
Regulation: PTGS2 ---> AKT1	16396944	
Regulation: PTGS2 ---> AKT1	34404424	10.1186/s13046-021-02050-1
Regulation: PTGS2 ---> AKT1	33662521	10.1016/j.alcohol.2020.08.00710.1016/j.alcohc
Regulation: PTGS2 ---> AKT1	22784710	10.1158/1535-7163.MCT-12-034210.1158/1535-716
Regulation: PTGS2 ---> AKT1	723	10.1016/j.cyto.2018.03.032
Regulation: PTGS2 ---> AKT1		10.1016/j.mehy.2012.04.012
Regulation: PTGS2 ---> AKT1	16014934	
Regulation: PTGS2 ---> AKT1	16014934	
Regulation: PTGS2 ---> AKT1	17259595	
Regulation: PTGS2 ---> AKT1	25422106	10.2337/db14-0979
Regulation: PTGS2 ---> AKT1	27056726	10.1152/ajpgi.00428.2015
Regulation: PTGS2 ---> AKT1		10.2174/1389557516666160505115743
Regulation: PTGS2 ---> AKT1		10.2174/1389557516666160505115743
Regulation: PTGS2 ---> AKT1		10.2174/1573406412666160622130036
Regulation: PTGS2 ---> AKT1	27994517	10.7150/ijbs.15943
Regulation: PTGS2 ---> AKT1	29048666	10.3892/or.2017.5981
Regulation: PTGS2 ---> AKT1	29110215	10.1007/s10456-017-9583-4
Regulation: PTGS2 ---> AKT1		10.1097/CMR.0000000000000368
Regulation: PTGS2 ---> AKT1		10.1097/IGC.0000000000001269
Regulation: PTGS2 ---> AKT1		10.1002/JLB.3HI0518-193R
Regulation: PTGS2 ---> AKT1		10.1186/s13046-019-1361-2
Regulation: PTGS2 ---> AKT1		10.1186/s13046-019-1361-2

Regulation: PTGS2 ---> AKT1 10.1186/s13046-019-1361-2
Regulation: PTGS2 ---> AKT1 10.1186/s13046-019-1361-2
Regulation: PTGS2 ---> AKT1 23229294 10.3892/or.2012.2158
Regulation: PTGS2 ---> AKT1 23243000 10.1091/mbc.E12-08-0637
Regulation: PTGS2 ---> AKT1 20203246 10.1164/rccm.200905-0674OC
Regulation: PTGS2 ---> AKT1 20203246 10.1164/rccm.200905-0674OC
Regulation: PTGS2 ---> AKT1 10.1186/s13046-019-1361-2
Regulation: PTGS2 ---> AKT1 10.1248/bpb.b19-00222
Regulation: PTGS2 ---> AKT1 31380437 10.1155/2019/758745110.1155/2019/7587451
Regulation: PTGS2 ---> AKT1 31380437 10.1155/2019/758745110.1155/2019/7587451
Regulation: PTGS2 ---> AKT1 31438982 10.1186/s13046-019-1361-210.1186/s13046-019-1
Regulation: PTGS2 ---> AKT1 31438982 10.1186/s13046-019-1361-210.1186/s13046-019-1
Regulation: PTGS2 ---> AKT1 15492414 10.1111/j.1346-8138.2004.tb00547.x
Regulation: PTGS2 ---> AKT1 23149922 10.1158/0008-5472.CAN-12-222010.1158/0008-547
Regulation: PTGS2 ---> AKT1 19505457 10.1016/j.yexcr.2009.06.002
Regulation: PTGS2 ---> AKT1 16014934
Regulation: PTGS2 ---> AKT1 19687189 10.1093/ecam/nep108
Regulation: PTGS2 ---> AKT1 21561856 10.1074/jbc.M111.222349
Regulation: PTGS2 ---> AKT1 21561856 10.1074/jbc.M111.222349
Regulation: PTGS2 ---> AKT1 19293251 10.1093/ecam/nep020
Regulation: PTGS2 ---> AKT1 20467837 10.1007/s12035-010-8139-z
Regulation: PTGS2 ---> AKT1 20803081 10.1245/s10434-010-1268-9
Regulation: PTGS2 ---> AKT1 21078168 10.1186/1471-2407-10-626
Regulation: PTGS2 ---> AKT1 21078168 10.1186/1471-2407-10-626
Regulation: PTGS2 ---> AKT1 21457561 10.1186/1743-422X-8-155
Regulation: PTGS2 ---> AKT1 30367494 10.1002/jcp.2751510.1002/jcp.27515
Regulation: PTGS2 ---> AKT1 32797349 10.1007/s11033-020-05709-8
Regulation: PTGS2 ---> AKT1 33152664 10.1016/j.redox.2020.10177410.1016/j.redox.20
Regulation: PTGS2 ---> AKT1 33152664 10.1016/j.redox.2020.10177410.1016/j.redox.20
Regulation: PTGS2 ---> AKT1 32902794 10.1007/s00432-020-03383-8
Regulation: PTGS2 ---> AKT1 29630111 10.26355/eurrev_201803_14579
Regulation: PTGS2 ---> AKT1 10.1124/jpet.118.254359
Regulation: PTGS2 ---> AKT1 33893997 10.1007/s12033-021-00326-7
Regulation: PTGS2 ---> AKT1 33893997 10.1007/s12033-021-00326-7
Regulation: PTGS2 ---> AKT1 34127813 10.1038/s41388-021-01858-7
Regulation: PTGS2 ---> AKT1 34664237 10.1007/978-3-030-73119-9_7
Regulation: PTGS2 ---> AKT1
Regulation: PTGS2 ---> AKT1 34917200 10.1155/2021/4114593
Regulation: PTGS2 ---> AKT1 34917200 10.1155/2021/4114593
Regulation: PTGS2 ---> AKT1 35031925 10.1007/s11033-021-07063-9
Regulation: PTGS2 ---> AKT1 35732325 10.21873/cgp.20335
Regulation: PTGS2 ---> AKT1 35780223 10.1007/s00018-022-04430-y
negative Regulation: MAPK1 ---| c 30257357 10.1016/j.biopha.2018.08.132
negative Regulation: MAPK1 ---| c 17690186 10.2353/ajpath.2007.070010
negative Regulation: MAPK1 ---| c 30257357 10.1016/j.biopha.2018.08.132
negative Regulation: MAPK1 ---| c 17482503 10.1016/j.drup.2007.03.003
negative Regulation: MAPK1 ---| c 23073477 10.1016/j.canlet.2012.10.004
negative Regulation: MAPK1 ---| c 28849180 10.3892/or.2017.5908
negative Regulation: MAPK1 ---| c 31133044 10.1186/s13046-019-1226-8
negative Regulation: MAPK1 ---| c 32039627 10.4149/neo_2020_190604N486
negative Regulation: MAPK1 ---| c 24104394 10.1038/leu.2013.291
negative Regulation: MAPK1 ---| c 30257357 10.1016/j.biopha.2018.08.13210.1016/j.biopha.
negative Regulation: MAPK1 ---| c 22179790 10.3892/ijo.2011.1303
negative Regulation: MAPK1 ---| c 22179790 10.3892/ijo.2011.1303

negative Regulation: MAPK1 ---| c 22179790 10.3892/ijo.2011.1303
negative Regulation: MAPK1 ---| c 26316041 10.3892/ijo.2015.3137
negative Regulation: MAPK1 ---| c 22179790 10.3892/ijo.2011.1303
negative Regulation: MAPK1 ---| c 22179790 10.3892/ijo.2011.1303
negative Regulation: MAPK1 ---| chemosensitivity
negative Regulation: CLDN1 ---| F 35686527 10.1631/jzus.B2100988
Binding: NOTCH1 ---- LRP8 30304853 10.3390/ijms19103090
Regulation: CD274 ---> PPARG 33218268 10.1080/15384047.2020.183803210.1080/15384047
Regulation: CD274 ---> PPARG 19535643 10.4049/jimmunol.0804260
Regulation: CD274 ---> PPARG
positive Regulation: LRP8 --+> NC 21253854 10.1007/s11064-011-0407-4
positive Regulation: LRP8 --+> NOTCH1
negative Regulation: TNFAIP8L2 -- 30114619 10.1016/j.molimm.2018.08.010
negative Regulation: TNFAIP8L2 -- 29920292 10.1016/j.canlet.2018.06.017
negative Regulation: TNFAIP8L2 -- 27712587 10.3727/096504016X14666990347437
negative Regulation: TNFAIP8L2 -- 27779698 10.3892/or.2016.5192
negative Regulation: TNFAIP8L2 -- 27779698 10.3892/or.2016.5192
negative Regulation: TNFAIP8L2 -- 27779698 10.3892/or.2016.5192
negative Regulation: TNFAIP8L2 -- 28186089 10.1038/cgt.2017.3
negative Regulation: TNFAIP8L2 -- 28186089 10.1038/cgt.2017.3
negative Regulation: TNFAIP8L2 -- 28186089 10.1038/cgt.2017.3
negative Regulation: TNFAIP8L2 -- 28287114 10.1038/cmi.2017.4
negative Regulation: TNFAIP8L2 -- 28849057 10.3892/mmr.2017.7364
negative Regulation: TNFAIP8L2 -- 30015980 10.3892/or.2018.6576
negative Regulation: TNFAIP8L2 -- 30157801 10.1186/s12885-018-4761-3
negative Regulation: TNFAIP8L2 -- 30157801 10.1186/s12885-018-4761-3
negative Regulation: TNFAIP8L2 -- 30157801 10.1186/s12885-018-4761-3
negative Regulation: TNFAIP8L2 -- 30157801 10.1186/s12885-018-4761-3
negative Regulation: TNFAIP8L2 -- 30157801 10.1186/s12885-018-4761-3
negative Regulation: TNFAIP8L2 -- 31591376 10.12659/MSM.917229
negative Regulation: TNFAIP8L2 -- 32493031 10.1152/ajplung.00069.2020
negative Regulation: TNFAIP8L2 -- 25946186 10.1371/journal.pone.0126176
negative Regulation: TNFAIP8L2 -- 25946186 10.1371/journal.pone.0126176
negative Regulation: TNFAIP8L2 -- 26530498 10.3892/ijo.2015.3224
negative Regulation: TNFAIP8L2 -- 26987289 10.1038/cgt.2016.6
negative Regulation: TNFAIP8L2 -- 26987289 10.1038/cgt.2016.6
negative Regulation: TNFAIP8L2 -- 25946186 10.1371/journal.pone.012617610.1371/journal.p
negative Regulation: TNFAIP8L2 -- 27712587 10.3727/096504016X1466699034743710.3727/09650
negative Regulation: TNFAIP8L2 -- 27779698 10.3892/or.2016.519210.3892/or.2016.5192
negative Regulation: TNFAIP8L2 -- 30157801 10.1186/s12885-018-4761-310.1186/s12885-018-4
negative Regulation: TNFAIP8L2 -- 34446024 10.1186/s12931-021-01826-5
negative Regulation: TNFAIP8L2 -- 34446024 10.1186/s12931-021-01826-510.1186/s12931-021-
negative Regulation: TNFAIP8L2 ---| AKT1 10.1016/j.jsps.2022.05.009
negative Regulation: TNFAIP8L2 ---| AKT1 10.1016/j.jsps.2022.05.009
negative Regulation: TNFAIP8L2 -- 35903524 10.1016/j.jsps.2022.05.00910.1016/j.jsps.2022
Regulation: STC2 ---> AKT1 30661222 10.1007/s12253-018-00576-y
Regulation: STC2 ---> AKT1 32900244 10.1089/hum.2020.090
Regulation: STC2 ---> AKT1 32900244 10.1089/hum.2020.090
Regulation: STC2 ---> AKT1 27863406 10.18632/oncotarget.1335510.18632/oncotarget.
Regulation: STC2 ---> AKT1 27863406 10.18632/oncotarget.1335510.18632/oncotarget.
negative Expression: ING4 ---| SF 17848618 10.1182/blood-2007-02-074617
negative Expression: ING4 ---| SF 17848618 10.1182/blood-2007-02-074617
negative Expression: ING4 ---| SF 17848618 10.1182/blood-2007-02-074617
negative Expression: ING4 ---| SF 30643005 10.1042/BSR20180773

negative Expression: ING4 ---| SF 17848618 10.1182/blood-2007-02-074617
negative Expression: ING4 ---| SF 17848618 10.1182/blood-2007-02-074617
negative Expression: ING4 ---| SF 17848618 10.1182/blood-2007-02-074617
negative Expression: ING4 ---| SF 17848618 10.1182/blood-2007-02-074617
negative Expression: ING4 ---| SF 17848618 10.1182/blood-2007-02-074617
negative Expression: ING4 ---| SF 17848618 10.1182/blood-2007-02-074617
negative Expression: ING4 ---| SF 35576491 10.1089/jir.2021.0214
negative Regulation: BECN1 ---| M 32800766 10.1016/j.ygeno.2020.08.009
negative Regulation: BECN1 ---| M 26837467 10.1038/ncomms10533
Regulation: CLDN1 ---> SPP1 26982264 10.1089/dna.2015.3188
positive Regulation: PTGS2 --+> F 15525653 10.1074/jbc.M410006200
positive Regulation: PTGS2 --+> F 16309946 10.1016/j.biocel.2005.10.006
positive Regulation: PTGS2 --+> F 15525653
positive Regulation: PTGS2 --+> F 20339539
positive Regulation: PTGS2 --+> F 21175416
positive Regulation: PTGS2 --+> F 21989184 10.1038/bjc.2011.403
positive Regulation: PTGS2 --+> F 25595899 10.18632/oncotarget.2783
positive Regulation: PTGS2 --+> RAC1
positive Expression: RAC1 --+> PT 15860667 10.1182/blood-2004-12-4975
positive Expression: RAC1 --+> PT 15860667 10.1182/blood-2004-12-4975
positive Expression: RAC1 --+> PT 15860667 10.1182/blood-2004-12-4975
positive Expression: RAC1 --+> PT 20167866 10.2353/ajpath.2010.090714
positive Expression: RAC1 --+> PT 20167866 10.2353/ajpath.2010.090714
positive Expression: RAC1 --+> PT 20167866 10.2353/ajpath.2010.090714
positive Expression: RAC1 --+> PT 20167866 10.2353/ajpath.2010.090714
positive Expression: RAC1 --+> PT 20167866 10.2353/ajpath.2010.090714
positive Expression: RAC1 --+> PT 19729484 10.2353/ajpath.2009.090443
positive Expression: RAC1 --+> PT 21907186 10.1016/j.bbrc.2011.08.116
positive Expression: RAC1 --+> PT 19118901 10.1016/j.molimm.2008.11.006
positive Expression: RAC1 --+> PT 20004474 10.1016/j.molimm.2009.11.017
positive Expression: RAC1 --+> PT 20004474 10.1016/j.molimm.2009.11.017
positive Expression: RAC1 --+> PT 20004474 10.1016/j.molimm.2009.11.017
positive Expression: RAC1 --+> PT 19110011 10.1016/j.bbamcr.2008.11.009
positive Expression: RAC1 --+> PT 18599158 10.1016/j.neurobiolaging.2008.05.017
positive Expression: RAC1 --+> PT 15588766 10.1016/j.bbcan.2004.10.002
positive Expression: RAC1 --+> PT 15588766 10.1016/j.bbcan.2004.10.002
positive Expression: RAC1 --+> PTGS2 10.1016/S1359-6446(03)02977-5
positive Expression: RAC1 --+> PT 20451669 10.1016/j.intimp.2010.04.026
positive Expression: RAC1 --+> PT 12857884
positive Expression: RAC1 --+> PT 12857884
positive Expression: RAC1 --+> PT 12857884
positive Expression: RAC1 --+> PT 12857884
positive Expression: RAC1 --+> PT 12857884
positive Expression: RAC1 --+> PT 12857884
positive Expression: RAC1 --+> PT 12360401
positive Expression: RAC1 --+> PT 27442365 10.1515/revneuro-2016-0021
positive Expression: RAC1 --+> PT 22113496 10.2119/molmed.2011.00447
positive Expression: RAC1 --+> PT 24667766 10.1371/journal.pone.0092398
positive Expression: RAC1 --+> PT 12857884 10.1091/mbc.e03-01-0016
positive Expression: RAC1 --+> PT 16088958 10.1002/jcb.20568
positive Expression: RAC1 --+> PT 16088958 10.1002/jcb.20568
positive Expression: RAC1 --+> PT 17592548 10.2119/2007－00005.Wu
positive Expression: RAC1 --+> PT 19118901 10.1016/j.molimm.2008.11.00610.1016/j.molimm.
positive Expression: RAC1 --+> PT 20131316 10.1002/ijc.2522610.1002/ijc.25226

positive Expression: RAC1 --+> P]12857884
positive Expression: RAC1 --+> PTGS2   10.1158/1940-6207.CAPR-15-0205-T
positive Expression: RAC1 --+> PTGS2
positive Expression: RAC1 --+> P]36018772 10.1530/REP-21-0186
positive Expression: RAC1 --+> P]36018772 10.1530/REP-21-0186
positive Expression: RAC1 --+> P]36018772 10.1530/REP-21-0186
positive Expression: RAC1 --+> P]36018772 10.1530/REP-21-0186
positive Expression: RAC1 --+> P]36018772 10.1530/REP-21-0186
positive Expression: RAC1 --+> P]36018772 10.1530/REP-21-0186
positive Expression: RAC1 --+> P]36018772 10.1530/REP-21-0186
positive Expression: RAC1 --+> P]36018772 10.1530/REP-21-0186
Regulation: BIRC7 ---> PTEN   21092516
Regulation: GPER1 ---> chemosensi26175858
Regulation: GPER1 ---> chemosensi35335125 10.3390/molecules27061762
negative Expression: PTEN ---| B]29436592 10.3892/ijmm.2018.3475
Regulation: MCL1 ---> AKT1   33831733 10.1016/j.archoralbio.2021.105114
Regulation: MCL1 ---> AKT1   21472003 10.1038/cddis.2011.2210.1038/cddis.2011.22
Regulation: MCL1 ---> AKT1   30415256 10.1159/00049506010.1159/000495060
Regulation: MCL1 ---> AKT1   33831733 10.1016/j.archoralbio.2021.105114
Regulation: MCL1 ---> AKT1   32814093 10.1016/j.jconrel.2020.08.018
Regulation: MCL1 ---> AKT1   29361380 10.1016/j.cytogfr.2018.01.002
Regulation: MCL1 ---> AKT1   22484311 10.1016/j.bcp.2012.03.015
Regulation: MCL1 ---> AKT1   22484311 10.1016/j.bcp.2012.03.015
Regulation: MCL1 ---> AKT1   20227039 10.1016/j.ccr.2010.01.021
Regulation: MCL1 ---> AKT1   23524145 10.1016/j.yexcr.2013.03.012
Regulation: MCL1 ---> AKT1   25801032 10.1016/j.celrep.2015.02.056
Regulation: MCL1 ---> AKT1   15782127
Regulation: MCL1 ---> AKT1   27102561 10.1152/ajpgi.00327.2015
Regulation: MCL1 ---> AKT1   27757735 10.1007/s10495-016-1307-5
Regulation: MCL1 ---> AKT1   30415256 10.1159/000495060
Regulation: MCL1 ---> AKT1   30415256 10.1159/000495060
Regulation: MCL1 ---> AKT1   30415256 10.1159/000495060
Regulation: MCL1 ---> AKT1   23158473 10.1186/1476-4598-11-84
Regulation: MCL1 ---> AKT1   23517112 10.1186/1471-2407-13-140
Regulation: MCL1 ---> AKT1   21472003 10.1038/cddis.2011.22
Regulation: MCL1 ---> AKT1   25032861 10.1038/cddis.2014.303
Regulation: MCL1 ---> AKT1   26739047 10.1128/JVI.02879-15
Regulation: MCL1 ---> AKT1   15782127 10.1038/sj.onc.1208530
Regulation: MCL1 ---> AKT1   31662324 10.1158/0008-5472.CAN-19-095010.1158/0008-547
Regulation: MCL1 ---> AKT1   35701409 10.1038/s41419-022-04958-810.1038/s41419-022-
Regulation: MCL1 ---> AKT1   35744786 10.3390/molecules27123661
Regulation: MCL1 ---> AKT1   35701409 10.1038/s41419-022-04958-8
Regulation: MCL1 ---> AKT1   35701409 10.1038/s41419-022-04958-8
Regulation: MCL1 ---> AKT1   35701409 10.1038/s41419-022-04958-8
Regulation: MCL1 ---> AKT1   35701409 10.1038/s41419-022-04958-8
Regulation: MCL1 ---> AKT1   35701409 10.1038/s41419-022-04958-8
Regulation: MCL1 ---> AKT1   36241868 10.1038/s41388-022-02482-9
Regulation: B3GNT3 ---> CD274   31874279 10.1016/j.semcancer.2019.11.011
Regulation: B3GNT3 ---> CD274   29438695 10.1016/j.ccell.2018.01.009
Regulation: B3GNT3 ---> CD274   29438695 10.1016/j.ccell.2018.01.009
Regulation: B3GNT3 ---> CD274   29438695 10.1016/j.ccell.2018.01.009
Regulation: B3GNT3 ---> CD274   29438695 10.1016/j.ccell.2018.01.009
Regulation: B3GNT3 ---> CD274   31287993 10.1016/j.ccell.2019.06.006
Regulation: B3GNT3 ---> CD274   32183950 10.1016/j.ccell.2020.02.006

Regulation: B3GNT3 ---> CD274	29765039	10.1038/s41467-018-04313-6
Regulation: B3GNT3 ---> CD274	32377193	10.1155/2020/5497015
Regulation: B3GNT3 ---> CD274	33159034	10.1038/s41419-020-03140-2
Regulation: B3GNT3 ---> CD274	32940722	10.1007/s00018-020-03637-1
Regulation: B3GNT3 ---> CD274	35197058	10.1186/s12943-021-01447-y
Expression: AKT1 ---> SPP1	27496181	10.1016/j.pbiomolbio.2016.08.003
Expression: AKT1 ---> SPP1	27794492	10.1016/j.jconrel.2016.10.024
Expression: AKT1 ---> SPP1	19356721	10.1016/j.ejphar.2009.03.063
Expression: AKT1 ---> SPP1	19356721	10.1016/j.ejphar.2009.03.063
Expression: AKT1 ---> SPP1	19767100	10.1016/j.leukres.2009.08.028
Expression: AKT1 ---> SPP1	21056654	10.1016/j.cellsig.2010.10.021
Expression: AKT1 ---> SPP1	21056654	10.1016/j.cellsig.2010.10.021
Expression: AKT1 ---> SPP1	18952487	10.1016/j.cytogfr.2008.08.001
Expression: AKT1 ---> SPP1	17161983	10.1016/j.dld.2006.10.015
Expression: AKT1 ---> SPP1	22947282	10.1016/j.acthis.2012.08.004
Expression: AKT1 ---> SPP1	24530412	10.1016/j.intimp.2014.01.028
Expression: AKT1 ---> SPP1	25986148	10.1016/j.yjmcc.2015.05.006
Expression: AKT1 ---> SPP1	25986148	10.1016/j.yjmcc.2015.05.006
Expression: AKT1 ---> SPP1	16946008	
Expression: AKT1 ---> SPP1	16807238	
Expression: AKT1 ---> SPP1	15347645	
Expression: AKT1 ---> SPP1	15854023	
Expression: AKT1 ---> SPP1	12944477	
Expression: AKT1 ---> SPP1	12944477	
Expression: AKT1 ---> SPP1	12944477	
Expression: AKT1 ---> SPP1	12944477	
Expression: AKT1 ---> SPP1	12944477	
Expression: AKT1 ---> SPP1	12944477	
Expression: AKT1 ---> SPP1	12944477	
Expression: AKT1 ---> SPP1	28715969	10.1089/ars.2017.7275
Expression: AKT1 ---> SPP1	29620276	10.3892/mmr.2018.8845
Expression: AKT1 ---> SPP1	22965338	10.3892/mmr.2012.1074
Expression: AKT1 ---> SPP1	22965338	10.3892/mmr.2012.1074
Expression: AKT1 ---> SPP1	21406114	10.1186/1471-2121-12-11
Expression: AKT1 ---> SPP1	21406114	10.1186/1471-2121-12-11
Expression: AKT1 ---> SPP1	21406114	10.1186/1471-2121-12-11
Expression: AKT1 ---> SPP1	21406114	10.1186/1471-2121-12-11
Expression: AKT1 ---> SPP1	21406114	10.1186/1471-2121-12-11
Expression: AKT1 ---> SPP1	22675553	10.1371/journal.pone.0038378
Expression: AKT1 ---> SPP1	22675553	10.1371/journal.pone.0038378
Expression: AKT1 ---> SPP1	22675553	10.1371/journal.pone.0038378
Expression: AKT1 ---> SPP1	22675553	10.1371/journal.pone.0038378
Expression: AKT1 ---> SPP1	22675553	10.1371/journal.pone.0038378
Expression: AKT1 ---> SPP1	22675553	10.1371/journal.pone.0038378
Expression: AKT1 ---> SPP1	25884410	10.1371/journal.pone.0123318
Expression: AKT1 ---> SPP1	26280346	10.1371/journal.pone.0134243
Expression: AKT1 ---> SPP1	12944477	10.1128/mcb.23.18.6507-6519.2003
Expression: AKT1 ---> SPP1	12944477	10.1128/mcb.23.18.6507-6519.2003
Expression: AKT1 ---> SPP1	15705860	
Expression: AKT1 ---> SPP1	15705860	
Expression: AKT1 ---> SPP1	16614705	
Expression: AKT1 ---> SPP1	21406114	10.1186/1471-2121-12-1110.1186/1471-2121-12-1
Expression: AKT1 ---> SPP1	24530412	10.1016/j.intimp.2014.01.02810.1016/j.intimp.
Expression: AKT1 ---> SPP1	34234236	10.1038/s41598-021-93484-210.1038/s41598-021-

Regulation: NGF ---> GCLC	15877105	
Regulation: NGF ---> GCLC	7901332	10.1111/j.1471-4159.1993.tb09808.x
Regulation: NGF ---> GCLC	8930687	
Regulation: NGF ---> GCLC	8973794	10.1016/s0006-8993(96)00844-x
positive Expression: NGF --+> MAF	28636927	10.1016/j.neulet.2017.06.025
positive Expression: NGF --+> MAF	33985879	10.1016/j.phymed.2021.153576
positive Expression: NGF --+> MAF	11024454	10.1016/S0014-5793(00)02037-8
positive Expression: NGF --+> MAF	18328626	10.1016/j.neulet.2008.01.078
positive Expression: NGF --+> MAF	20138971	10.1016/j.neulet.2010.01.070
positive Expression: NGF --+> MAF	15207334	10.1016/j.neuroscience.2004.04.036
positive Expression: NGF --+> MAF	23954826	10.1016/j.neulet.2013.07.057
positive Expression: NGF --+> MAF	12606768	
positive Expression: NGF --+> MAF	15385613	
positive Expression: NGF --+> MAF	15385613	
positive Expression: NGF --+> MAF	15385613	
positive Expression: NGF --+> MAF	28005222	10.1007/s11064-016-2134-3
positive Expression: NGF --+> MAF	32362584	10.1124/mol.119.118950
positive Expression: NGF --+> MAF	32567558	10.1530/REP-19-0601
positive Expression: NGF --+> MAF	34257579	10.3389/pore.2021.612375
positive Expression: NGF --+> MAF	22207192	10.1007/s00210-011-0721-z
positive Expression: NGF --+> MAF	24686445	10.1038/ncomms4527
positive Expression: NGF --+> MAF	15207334	10.1016/j.neuroscience.2004.04.036
positive Expression: NGF --+> MAF	22024079	10.5754/hge1122010.5754/hge11220
positive Expression: NGF --+> MAF	22280975	10.1016/j.neuroscience.2012.01.00210.1016/j.n
positive Expression: NGF --+> MAF	15153516	
positive Expression: NGF --+> MAF	990	10.1016/j.pan.2016.09.005
positive Expression: NGF --+> MAF	15555922	10.1016/j.mcn.2004.07.007
positive Expression: NGF --+> MAF	15555922	10.1016/j.mcn.2004.07.007
positive Expression: NGF --+> MAF	12764099	
positive Expression: NGF --+> MAF	24006492	10.1091/mbc.E12-06-0445
positive Expression: NGF --+> MAF	12154004	
positive Expression: NGF --+> MAF	20663926	10.1158/1535-7163.MCT-10-0336
positive Expression: NGF --+> MAF	16999937	10.1016/j.bcp.2006.08.013
positive Expression: NGF --+> MAF	21871873	10.1016/j.cbi.2011.08.001
positive Expression: NGF --+> MAF	15480997	10.1053/j.gastro.2004.06.023
positive Expression: NGF --+> MAF	12086645	10.1016/S0896-6273(02)00717-1
positive Expression: NGF --+> MAF	14550783	10.1016/S1044-7431(03)00126-X
positive Expression: NGF --+> MAF	9658404	
positive Expression: NGF --+> MAF	18367547	
positive Expression: NGF --+> MAF	17380122	
positive Expression: NGF --+> MAF	12764099	
positive Expression: NGF --+> MAF	10349848	
positive Expression: NGF --+> MAF	28762104	10.1007/s11064-017-2333-6
positive Expression: NGF --+> MAF	28762104	10.1007/s11064-017-2333-6
positive Expression: NGF --+> MAPK1		10.1530/REP-19-0601
positive Expression: NGF --+> MAPK1		10.1016/j.phymed.2021.153576
positive Expression: NGF --+> MAPK1		10.3389/pore.2021.612375
positive Expression: NGF --+> MAF	35610050	10.1523/JNEUROSCI.1133-21.2022
positive Expression: NGF --+> MAPK1		10.1016/j.burns.2022.11.001
Expression: MAPK1 ---> NGF	28924549	10.1016/j.apsb.2017.06.006
Expression: MAPK1 ---> NGF	29872754	10.1016/j.heliyon.2018.e00617
Expression: MAPK1 ---> NGF	29872754	10.1016/j.heliyon.2018.e00617
Expression: MAPK1 ---> NGF	21241747	10.1016/j.neures.2010.12.016
Expression: MAPK1 ---> NGF	17651887	10.1016/j.tox.2007.05.029

Expression: MAPK1 ---> NGF 17651887 10.1016/j.tox.2007.05.029
Expression: MAPK1 ---> NGF 23454433 10.1016/j.brainres.2013.02.026
Expression: MAPK1 ---> NGF 12925217
Expression: MAPK1 ---> NGF 17942717
Expression: MAPK1 ---> NGF 29532850 10.3892/ijo.2018.4311
Expression: MAPK1 ---> NGF 10.2478/s11756-018-0099-1
Expression: MAPK1 ---> NGF 10.2478/s11756-018-0099-1
Expression: MAPK1 ---> NGF 30893117 10.1097/ACO.0000000000000723
Expression: MAPK1 ---> NGF 23182412 10.1017/S0007114512004710
Expression: MAPK1 ---> NGF 23207416 10.1159/000345043
Expression: MAPK1 ---> NGF 25957627 10.1007/s10266-015-0206-510.1007/s10266-015-0
Expression: MAPK1 ---> NGF 1246 10.1016/j.apsb.2017.06.006
Expression: MAPK1 ---> NGF 10.1097/ACO.0000000000000723
positive MolTransport: NGF --+> M33910125 10.1016/j.compbiomed.2021.104339
positive MolTransport: NGF --+> M10858459 10.1074/jbc.275.25.19306
positive MolTransport: NGF --+> M10858459 10.1074/jbc.275.25.19306
positive MolTransport: NGF --+> M10858459 10.1074/jbc.275.25.19306
positive MolTransport: NGF --+> M12706116 10.1016/S0014-4827(03)00037-5
positive MolTransport: NGF --+> M10858459
positive MolTransport: NGF --+> M10858459
positive MolTransport: NGF --+> M10858459
positive MolTransport: NGF --+> M8626780
positive MolTransport: NGF --+> M16107706
positive MolTransport: NGF --+> M15121859
positive MolTransport: NGF --+> M15772344
positive MolTransport: NGF --+> M27342992 10.1007/s00018-016-2297-8
positive MolTransport: NGF --+> M22358092
positive MolTransport: NGF --+> M12615082 10.1016/S0736-5748(02)00139-9
positive MolTransport: NGF --+> M16306406
positive MolTransport: NGF --+> M15056710
Expression: KDM5B ---> TP53 25877751 10.1007/s13277-015-3418-y10.1007/s13277-015-3
Expression: KDM5B ---> TP53 25877751 10.1007/s13277-015-3418-y10.1007/s13277-015-3
Expression: KDM5B ---> TP53 31949850
Expression: KDM5B ---> TP53 31949850
positive Expression: NGF --+> AKT30578789 10.1016/j.exer.2018.12.005
positive Expression: NGF --+> AKT18660503 10.1074/jbc.M803019200
positive Expression: NGF --+> AKT30928478 10.1016/j.neulet.2019.03.049
positive Expression: NGF --+> AKT27263974 10.1016/j.neuron.2016.05.002
positive Expression: NGF --+> AKT32531260 10.1016/j.bbadis.2020.165858
positive Expression: NGF --+> AKT33985879 10.1016/j.phymed.2021.153576
positive Expression: NGF --+> AKT21036149 10.1016/j.bbrc.2010.10.110
positive Expression: NGF --+> AKT20307615 10.1016/j.neuint.2010.03.005
positive Expression: NGF --+> AKT20138971 10.1016/j.neulet.2010.01.070
positive Expression: NGF --+> AKT15555922 10.1016/j.mcn.2004.07.007
positive Expression: NGF --+> AKT1 10.1016/j.clim.2009.03.456
positive Expression: NGF --+> AKT18094051
positive Expression: NGF --+> AKT15626752
positive Expression: NGF --+> AKT15626752
positive Expression: NGF --+> AKT21179413
positive Expression: NGF --+> AKT27186265
positive Expression: NGF --+> AKT28005222 10.1007/s11064-016-2134-3
positive Expression: NGF --+> AKT28762104 10.1007/s11064-017-2333-6
positive Expression: NGF --+> AKT28762104 10.1007/s11064-017-2333-6
positive Expression: NGF --+> AKT32362584 10.1124/mol.119.118950

positive Expression: NGF --+> AK123598414 10.1038/cddis.2013.99
positive Expression: NGF --+> AKT1
positive Expression: NGF --+> AK121179413 10.1371/journal.pone.0015600
positive Expression: NGF --+> AK124006492 10.1091/mbc.E12-06-0445
positive Expression: NGF --+> AK124667984 10.1371/journal.pone.0093223
positive Expression: NGF --+> AK112065668 10.1046/j.1471-4159.2002.00841.x
positive Expression: NGF --+> AK117182181 10.1016/j.neulet.2006.11.040
positive Expression: NGF --+> AK120663926 10.1158/1535-7163.MCT-10-033610.1158/1535-716
positive Expression: NGF --+> AK130543144 10.1080/09168451.2018.155360710.1080/09168451
positive Expression: NGF --+> AK130578789 10.1016/j.exer.2018.12.00510.1016/j.exer.2018
positive Expression: NGF --+> AK132997750 10.1097/ALN.000000000000357610.1097/ALN.00000
positive Expression: NGF --+> AKT1 10.1016/j.phymed.2021.153576
positive Expression: NGF --+> AK135109876 10.1186/s12974-022-02405-1
Expression: CLDN1 ---> NOTCH1 28316062 10.1007/s11010-017-3000-6
Expression: CLDN1 ---> NOTCH1 28316062 10.1007/s11010-017-3000-6
Expression: CLDN1 ---> NOTCH1 28316062 10.1007/s11010-017-3000-6
Expression: CLDN1 ---> NOTCH1 28316062 10.1007/s11010-017-3000-610.1007/s11010-017-3
Expression: CLDN1 ---> NOTCH1 23766441 10.1136/gutjnl-2012-304241
Expression: CLDN1 ---> NOTCH1 28316062 10.1007/s11010-017-3000-6
Expression: CLDN1 ---> NOTCH1 28316062 10.1007/s11010-017-3000-6
Expression: SPP1 ---> NOTCH1 21747408 10.1038/icb.2011.47
Expression: SPP1 ---> NOTCH1 24131213 10.1089/scd.2013.0300
Expression: SPP1 ---> NOTCH1 25734817 10.1371/journal.pone.0118201
Expression: SPP1 ---> NOTCH1 25734817 10.1371/journal.pone.0118201
Expression: SPP1 ---> NOTCH1 29410240 10.1016/j.jprot.2018.01.017
Expression: STC2 ---> CLDN1 25830567 10.1371/journal.pone.012217910.1371/journal.p
Regulation: NOTCH1 ---> IGFBP4 15262980 10.1074/jbc.M405222200
Regulation: NOTCH1 ---> IGFBP4 17314100 10.1074/jbc.M607903200
Regulation: NOTCH1 ---> IGFBP4 15262980
Regulation: NOTCH1 ---> IGFBP4 31518159 10.1152/ajpheart.00395.2019
Regulation: NOTCH1 ---> IGFBP4 23161866 10.1210/en.2012-1552
negative Expression: IGFBP4 ---| 28150906 10.1002/cbin.1073210.1002/cbin.10732
negative Expression: PRR5L ---| I21964062 10.1016/j.cellsig.2011.09.01510.1016/j.cellsi
PromoterBinding: TP53 ---> CLDN1 18648642
PromoterBinding: TP53 ---> CLDN1 18648642
PromoterBinding: TP53 ---> CLDN1
Regulation: B3GNT3 ---> AKT1 31968179 10.1089/dna.2019.5089
Regulation: B3GNT3 ---> AKT1 24118321 10.1111/cas.1229410.1111/cas.12294
positive PromoterBinding: PPARG -23473036 10.1016/j.cmet.2013.01.016
positive PromoterBinding: PPARG -25562426 10.1016/j.cellsig.2014.12.015
positive PromoterBinding: PPARG -25562426 10.1016/j.cellsig.2014.12.015
positive PromoterBinding: PPARG -25562426 10.1016/j.cellsig.2014.12.015
positive PromoterBinding: PPARG -25562426 10.1016/j.cellsig.2014.12.015
positive PromoterBinding: PPARG -28711511 10.1016/j.micpath.2017.07.019
positive PromoterBinding: PPARG -23589827 10.1152/physrev.00019.2012
positive PromoterBinding: PPARG -31185306 10.1016/j.bbalip.2019.06.006
positive PromoterBinding: PPARG -18688264
positive PromoterBinding: PPARG --+> CLDN1
Expression: NOTCH1 ---> RBP1 32971095 10.1016/j.exer.2020.108256
Expression: NOTCH1 ---> RBP1 20100467 10.1016/j.bbrc.2010.01.084
Expression: NOTCH1 ---> RBP1 20100467 10.1016/j.bbrc.2010.01.084
Expression: NOTCH1 ---> RBP1 17991461 10.1016/j.ydbio.2007.09.052
Expression: NOTCH1 ---> RBP1 18805411 10.1016/j.ydbio.2008.08.034
Expression: NOTCH1 ---> RBP1 17681135 10.1016/j.devcel.2007.06.012

Expression: NOTCH1 ---> RBP1	17681135	10.1016/j.devcel.2007.06.012
Expression: NOTCH1 ---> RBP1	23293291	10.1242/dev.082842
Expression: NOTCH1 ---> RBP1	17681135	10.1016/j.devcel.2007.06.012
positive Expression: PTGS2 --+>	22826468	10.1124/mol.112.079129
positive Expression: PTGS2 --+>	26600514	10.4238/2015.November.18.18
positive Expression: PTGS2 --+>	15517878	
positive Expression: PTGS2 --+>	16170027	10.1158/1535-7163.MCT-05-0139
positive Expression: PTGS2 --+>	18695918	
positive Expression: PTGS2 --+>	26600514	10.4238/2015.November.18.1810.4238/2015.Novem
Regulation: ABCC1 ---> PTGS2	21803151	10.1016/j.cellsig.2011.07.010
Regulation: ABCC1 ---> PTGS2	21803151	10.1016/j.cellsig.2011.07.010
positive QuantitativeChange: hyp	27584795	10.1038/cddis.2016.262
positive QuantitativeChange: hyp	27584795	10.1038/cddis.2016.262
Regulation: SPP1 ---> MCL1	30107166	10.1016/j.lfs.2018.08.025
Regulation: SPP1 ---> MCL1	24947165	10.1186/1477-7819-12-189
Expression: AKT1 ---> NOTCH1	27816488	10.1016/j.canlet.2016.10.039
Expression: AKT1 ---> NOTCH1	18440314	10.1016/j.febslet.2008.04.024
Expression: AKT1 ---> NOTCH1	18440314	10.1016/j.febslet.2008.04.024
Expression: AKT1 ---> NOTCH1		10.1016/j.lfs.2011.10.012
Expression: AKT1 ---> NOTCH1	21338656	10.1016/j.bbcan.2011.02.001
Expression: AKT1 ---> NOTCH1	21338656	10.1016/j.bbcan.2011.02.001
Expression: AKT1 ---> NOTCH1	17920278	10.1016/j.tibs.2007.09.002
Expression: AKT1 ---> NOTCH1	22056375	10.1016/j.lfs.2011.10.012
Expression: AKT1 ---> NOTCH1		10.1016/j.amjsurg.2015.11.029
Expression: AKT1 ---> NOTCH1	26850133	10.1016/j.amjsurg.2015.11.029
Expression: AKT1 ---> NOTCH1	18924608	
Expression: AKT1 ---> NOTCH1	18924608	
Expression: AKT1 ---> NOTCH1	18924608	
Expression: AKT1 ---> NOTCH1	18924608	
Expression: AKT1 ---> NOTCH1	18924608	
Expression: AKT1 ---> NOTCH1	17158336	
Expression: AKT1 ---> NOTCH1	12482957	
Expression: AKT1 ---> NOTCH1		10.1172/JCI80396
Expression: AKT1 ---> NOTCH1	28629170	10.3390/ijms18061295
Expression: AKT1 ---> NOTCH1	22258620	10.1038/emboj.2011.443
Expression: AKT1 ---> NOTCH1	22258620	10.1038/emboj.2011.443
Expression: AKT1 ---> NOTCH1	24113181	10.1038/cddis.2013.375
Expression: AKT1 ---> NOTCH1	24113181	10.1038/cddis.2013.375
Expression: AKT1 ---> NOTCH1	24113181	10.1038/cddis.2013.375
Expression: AKT1 ---> NOTCH1	24113181	10.1038/cddis.2013.375
Expression: AKT1 ---> NOTCH1	24113181	10.1038/cddis.2013.375
Expression: AKT1 ---> NOTCH1	21730286	10.1128/MCB.05821-11
Expression: AKT1 ---> NOTCH1	24113181	10.1038/cddis.2013.37510.1038/cddis.2013.375
Expression: AKT1 ---> NOTCH1	26117016	10.7534/j.issn.1009-2137.2015.03.015
Expression: AKT1 ---> NOTCH1	29725418	10.3892/ol.2018.819310.3892/ol.2018.8193
Expression: AKT1 ---> NOTCH1	12768030	
Expression: AKT1 ---> NOTCH1	22258620	10.1038/emboj.2011.443
Expression: AKT1 ---> NOTCH1	23802886	10.1111/jgh.12291
Expression: AKT1 ---> NOTCH1	24062466	10.1073/pnas.1309276110
Expression: AKT1 ---> NOTCH1	26530164	10.3892/ijo.2015.3221
Expression: AKT1 ---> NOTCH1		
Expression: AKT1 ---> NOTCH1	29857516	10.3390/ijms19061631
Expression: AKT1 ---> NOTCH1	30478302	10.1038/s41419-018-1185-6
Expression: AKT1 ---> NOTCH1		10.1186/s12935-018-0714-9

positive Expression: PPARG --+> l18571911 10.1016/j.etp.2008.04.009
positive Expression: PPARG --+> l18688264
positive Expression: PPARG --+> l18688264
positive Expression: PPARG --+> l18688264 10.1038/cdd.2008.11610.1038/cdd.2008.116
positive Expression: PPARG --+> UPK1A
positive Expression: PPARG --+> l34697317 10.1038/s41467-021-26421-6
negative Expression: NOTCH1 ---| 29496446 10.1016/j.bbrc.2018.02.187
negative Expression: NOTCH1 ---| 29496446 10.1016/j.bbrc.2018.02.187
negative Expression: NOTCH1 ---| 29496446 10.1016/j.bbrc.2018.02.187
negative Expression: NOTCH1 ---| 28302721 10.1074/jbc.M116.759134
negative Expression: NOTCH1 ---| 33129879 10.1016/j.lfs.2020.118686
negative Expression: NOTCH1 ---| 30394310 10.1016/j.toxlet.2018.10.024
negative Expression: NOTCH1 ---| 30394310 10.1016/j.toxlet.2018.10.024
negative Expression: NOTCH1 ---| 30394310 10.1016/j.toxlet.2018.10.024
negative Expression: NOTCH1 ---| 30119264 10.1016/j.biopha.2018.07.018
negative Expression: NOTCH1 ---| 28645565 10.1016/j.cellsig.2017.06.011
negative Expression: NOTCH1 ---| 27823652 10.1016/j.critrevonc.2016.08.006
negative Expression: NOTCH1 ---| 30738865 10.1016/j.semcancer.2019.02.001
negative Expression: NOTCH1 ---| 32871474 10.1016/j.intimp.2020.106911
negative Expression: NOTCH1 ---| 32871474 10.1016/j.intimp.2020.106911
negative Expression: NOTCH1 ---| 32871474 10.1016/j.intimp.2020.106911
negative Expression: NOTCH1 ---| 31523031 10.1016/j.jbior.2019.100649
negative Expression: NOTCH1 ---| 21624358 10.1016/j.ydbio.2011.05.659
negative Expression: NOTCH1 ---| 18455982 10.1016/j.cell.2008.04.013
negative Expression: NOTCH1 ---| 21993533 10.1016/j.urolonc.2011.07.006
negative Expression: NOTCH1 ---| 21993533 10.1016/j.urolonc.2011.07.006
negative Expression: NOTCH1 ---| 21993533 10.1016/j.urolonc.2011.07.006
negative Expression: NOTCH1 ---| 23348702 10.1016/j.canlet.2013.01.020
negative Expression: NOTCH1 ---| 24090996 10.1016/j.leukres.2013.09.007
negative Expression: NOTCH1 ---| 24780321 10.1016/j.lfs.2014.04.017
negative Expression: NOTCH1 ---| 26123366 10.1016/j.exphem.2015.05.017
negative Expression: NOTCH1 ---| 26392376 10.1016/j.semcancer.2015.09.009
negative Expression: NOTCH1 ---| 20015880
negative Expression: NOTCH1 ---| 27070592 10.3390/ijms17040531
negative Expression: NOTCH1 ---| 28627671 10.3892/mmr.2017.6791
negative Expression: NOTCH1 ---| 28627671 10.3892/mmr.2017.6791
negative Expression: NOTCH1 ---| 28627671 10.3892/mmr.2017.6791
negative Expression: NOTCH1 ---| 28951560 10.1038/leu.2017.301
negative Expression: NOTCH1 ---| 29532851 10.3892/ijmm.2018.3548
negative Expression: NOTCH1 ---| 29743588 10.1038/s41388-018-0251-y
negative Expression: NOTCH1 ---| 29743588 10.1038/s41388-018-0251-y
negative Expression: NOTCH1 ---| 29743588 10.1038/s41388-018-0251-y
negative Expression: NOTCH1 ---| 29743588 10.1038/s41388-018-0251-y
negative Expression: NOTCH1 ---| 29743588 10.1038/s41388-018-0251-y
negative Expression: NOTCH1 ---| 29743588 10.1038/s41388-018-0251-y
negative Expression: NOTCH1 ---| 29858604 10.1038/s41580-018-0015-0
negative Expression: NOTCH1 ---| 30145641 10.1007/s11033-018-4321-6
negative Expression: NOTCH1 ---| 30145641 10.1007/s11033-018-4321-6
negative Expression: NOTCH1 ---| 31732769 10.1007/s00280-019-03990-4
negative Expression: NOTCH1 ---| 32893590 10.19540/j.cnki.cjcmm.20200405.403
negative Expression: NOTCH1 ---| 33158122 10.3390/ijms21218255
negative Expression: NOTCH1 ---| 33051595 10.1038/s41388-020-01498-3
negative Expression: NOTCH1 ---| PTEN 10.20944/preprints201609.0074.v1
negative Expression: NOTCH1 ---| 32467322 10.1136/jclinpath-2020-206693

negative Expression: NOTCH1 ---| 22623890 10.1100/2012/252457
negative Expression: NOTCH1 ---| 22074495 10.1186/1476-4598-10-138
negative Expression: NOTCH1 ---| PTEN
negative Expression: NOTCH1 ---| 23634247
negative Expression: NOTCH1 ---| 22002304 10.1038/onc.2011.467
negative Expression: NOTCH1 ---| 22002304 10.1038/onc.2011.467
negative Expression: NOTCH1 ---| 22002304 10.1038/onc.2011.467
negative Expression: NOTCH1 ---| 24113181 10.1038/cddis.2013.375
negative Expression: NOTCH1 ---| 24113181 10.1038/cddis.2013.375
negative Expression: NOTCH1 ---| 24113181 10.1038/cddis.2013.375
negative Expression: NOTCH1 ---| 24113181 10.1038/cddis.2013.375
negative Expression: NOTCH1 ---| 24113181 10.1038/cddis.2013.375
negative Expression: NOTCH1 ---| 24113181 10.1038/cddis.2013.375
negative Expression: NOTCH1 ---| 24113181 10.1038/cddis.2013.375
negative Expression: NOTCH1 ---| 24113181 10.1038/cddis.2013.375
negative Expression: NOTCH1 ---| 24113181 10.1038/cddis.2013.375
negative Expression: NOTCH1 ---| 24286423 10.3760/cma.j.issn.0366-6999.20130721
negative Expression: NOTCH1 ---| 24905788 10.1146/annurev-biochem-082411-113907
negative Expression: NOTCH1 ---| 24988225 10.1371/journal.pone.0101024
negative Expression: NOTCH1 ---| 25043004 10.1038/nature13495
negative Expression: NOTCH1 ---| 26491213 10.1155/2015/520590
negative Expression: NOTCH1 ---| 17245125 10.4161/cc.6.1.3648
negative Expression: NOTCH1 ---| 17245125 10.4161/cc.6.1.3648
negative Expression: NOTCH1 ---| 17873882 10.1038/nm1636
negative Expression: NOTCH1 ---| 19479935 10.1002/jcb.2219910.1002/jcb.22199
negative Expression: NOTCH1 ---| 19479935 10.1002/jcb.2219910.1002/jcb.22199
negative Expression: NOTCH1 ---| 19479935 10.1002/jcb.2219910.1002/jcb.22199
negative Expression: NOTCH1 ---| 21993533 10.1016/j.urolonc.2011.07.00610.1016/j.urolon
negative Expression: NOTCH1 ---| 24780321 10.1016/j.lfs.2014.04.01710.1016/j.lfs.2014.0
negative Expression: NOTCH1 ---| 25110169 10.3892/ijmm.2014.188910.3892/ijmm.2014.1889
negative Expression: NOTCH1 ---| 29496446 10.1016/j.bbrc.2018.02.18710.1016/j.bbrc.2018
negative Expression: NOTCH1 ---| PTEN 10.1016/j.semcancer.2019.02.001
negative Expression: NOTCH1 ---| PTEN 10.1016/j.jbior.2019.100649
negative Expression: NOTCH1 ---| PTEN 10.1016/j.urolonc.2011.07.006
negative Expression: NOTCH1 ---| PTEN 10.1016/j.urolonc.2011.07.006
negative Expression: NOTCH1 ---| PTEN 10.1016/j.urolonc.2011.07.006
negative Expression: NOTCH1 ---| PTEN 10.1007/s00280-019-03990-4
negative Expression: NOTCH1 ---| 29496446 10.1016/j.bbrc.2018.02.18710.1016/j.bbrc.2018
negative Expression: NOTCH1 ---| 28951560 10.1038/leu.2017.301
negative Expression: NOTCH1 ---| 21035501 10.1016/j.advenzreg.2010.09.012
negative Expression: NOTCH1 ---| 20223597 10.1016/j.ctrv.2010.02.005
negative Expression: NOTCH1 ---| 18951834 10.1016/j.drup.2008.09.002
negative Expression: NOTCH1 ---| 22897852 10.1016/j.ccr.2012.06.014
negative Expression: NOTCH1 ---| 26683354 10.1016/j.cca.2015.12.010
negative Expression: NOTCH1 ---| 26505911 10.1016/j.exphem.2015.10.004
negative Expression: NOTCH1 ---| 18830414
negative Expression: NOTCH1 ---| 18830414
negative Expression: NOTCH1 ---| 20015880
negative Expression: NOTCH1 ---| 19117948
negative Expression: NOTCH1 ---| 19117947
negative Expression: NOTCH1 ---| 27138341 10.1371/journal.pone.0154771
negative Expression: NOTCH1 ---| PTEN 10.1172/JCI84637
negative Expression: NOTCH1 ---| 22074495 10.1186/1476-4598-10-138
negative Expression: NOTCH1 ---| 22074495 10.1186/1476-4598-10-138

negative Expression: NOTCH1 ---| 21566651 10.1038/leu.2011.103
negative Expression: NOTCH1 ---| 21743488 10.1038/onc.2011.279
negative Expression: NOTCH1 ---| 23950991 10.1371/journal.pone.0070737
negative Expression: NOTCH1 ---| 23699387 10.1095/biolreprod.113.108100
negative Expression: NOTCH1 ---| 24415757 10.1074/jbc.M113.519686
negative Expression: NOTCH1 ---| 25781910 10.1080/15384047.2015.1016662
negative Expression: NOTCH1 ---| 26228240 10.1038/ncomms8935
negative Expression: NOTCH1 ---| 26228240 10.1038/ncomms8935
negative Expression: NOTCH1 ---| 26228240 10.1038/ncomms8935
negative Expression: NOTCH1 ---| 25781910 10.1080/15384047.2015.1016662
negative Expression: NOTCH1 ---| PTEN 10.1071/RD14212
negative Expression: NOTCH1 ---| PTEN 10.1071/RD14212
negative Expression: NOTCH1 ---| 28437168 10.1080/15384101.2017.1310351
negative Expression: NOTCH1 ---| 28627671 10.3892/mmr.2017.6791
negative Expression: NOTCH1 ---| 28603282 10.1038/cmi.2017.30
negative Expression: NOTCH1 ---| 29286063 10.3892/ijmm.2017.3319
negative Expression: NOTCH1 ---| 417 10.1016/j.bbamcr.2018.07.006
negative Expression: NOTCH1 ---| 21212363 10.1073/pnas.1006937108
negative Expression: NOTCH1 ---| 22258620 10.1038/emboj.2011.443
negative Expression: NOTCH1 ---| 19479935 10.1002/jcb.22199
negative Expression: NOTCH1 ---| PTEN
negative Expression: NOTCH1 ---| PTEN 10.1016/j.lfs.2020.118686
negative Expression: NOTCH1 ---| 34374886 10.1007/s10911-021-09496-1
negative Expression: NOTCH1 ---| 36067712 10.1016/j.cyto.2022.156013
negative Expression: NOTCH1 ---| 36286020 10.3390/cimb44100305
Binding: TP53 ---- RBP1 23376032 10.1016/j.freeradbiomed.2013.01.026
Regulation: TP53 ---> RBP1 14585279 10.1016/S1521-6616(03)00208-0
positive Regulation: GPER1 --+> T28595943 10.1016/j.jsbmb.2017.05.005
positive Regulation: GPER1 --+> T26888479 10.1507/endocrj.EJ15-0571
positive Regulation: GPER1 --+> T25275589 10.1038/cddis.2014.398
positive Regulation: GPER1 --+> T25275589 10.1038/cddis.2014.398
positive Regulation: GPER1 --+> T26225773 10.1038/cddis.2015.201
negative Expression: PTEN ---| CI32898555 10.1016/j.yexcr.2020.112259
negative Expression: PTEN ---| CI32522567 10.1016/j.lfs.2020.117923
negative Expression: PTEN ---| CI31931443 10.1016/j.lungcan.2020.01.005
negative Expression: PTEN ---| CI29800685 10.1016/j.canlet.2018.05.015
negative Expression: PTEN ---| CI32120023 10.1016/j.canlet.2020.02.036
negative Expression: PTEN ---| CI29126881 10.1016/j.bbcan.2017.10.007
negative Expression: PTEN ---| CI33607461 10.1016/j.ctrv.2021.102157
negative Expression: PTEN ---| CI32057515 10.1016/j.prp.2020.152852
negative Expression: PTEN ---| CI26967649 10.1016/j.coi.2016.02.001
negative Expression: PTEN ---| CI28208102 10.1016/j.coi.2017.01.004
negative Expression: PTEN ---| CI30287140 10.1016/j.tibs.2018.09.004
negative Expression: PTEN ---| CI33341506 10.1016/j.critrevonc.2020.103185
negative Expression: PTEN ---| CI31422157 10.1016/j.semcancer.2019.08.015
negative Expression: PTEN ---| CI31874279 10.1016/j.semcancer.2019.11.011
negative Expression: PTEN ---| CI27192563 10.1016/j.immuni.2016.05.002
negative Expression: PTEN ---| CI29562194 10.1016/j.immuni.2018.03.014
negative Expression: PTEN ---| CI33865701 10.1016/j.semcdb.2021.04.001
negative Expression: PTEN ---| CI31668929 10.1016/j.molcel.2019.09.030
negative Expression: PTEN ---| CI31005030 10.1016/j.drup.2019.04.001
negative Expression: PTEN ---| CI28233639 10.1016/j.it.2017.01.002
negative Expression: PTEN ---| CI27258185 10.1016/j.intimp.2016.05.016
negative Expression: PTEN ---| CI32416456 10.1016/j.intimp.2020.106594

negative Expression: PTEN ---|	CI33581502	10.1016/j.intimp.2021.107403
negative Expression: PTEN ---|	CI33581579	10.1016/j.intimp.2021.107443
negative Expression: PTEN ---|	CI33171331	10.1016/j.redox.2020.101780
negative Expression: PTEN ---|	CI18384938	10.1016/j.canlet.2008.02.055
negative Expression: PTEN ---|	CI20971039	10.1016/j.molmed.2010.09.006
negative Expression: PTEN ---|	CI24468585	10.1016/j.humimm.2014.01.006
negative Expression: PTEN ---|	CI24468585	10.1016/j.humimm.2014.01.006
negative Expression: PTEN ---|	CI25106088	10.1016/j.molonc.2014.07.011
negative Expression: PTEN ---|	CI25662388	10.1016/j.lungcan.2015.01.016
negative Expression: PTEN ---|	CI25440090	10.1016/j.molmed.2014.10.009
negative Expression: PTEN ---|	CI26762740	10.1016/j.jtho.2015.09.010
negative Expression: PTEN ---|	CI27764823	10.1159/000451015
negative Expression: PTEN ---|	CI27764823	10.1159/000451015
negative Expression: PTEN ---|	CI28368867	10.1097/WCO.0000000000000451
negative Expression: PTEN ---|	CI28064317	10.1134/S0026893316060248
negative Expression: PTEN ---|	CI28293764	10.1007/s11060-017-2390-3
negative Expression: PTEN ---|	CI28627635	10.3892/or.2017.5733
negative Expression: PTEN ---|	CD274	10.12659/MSM.903098
negative Expression: PTEN ---|	CI28528510	10.1007/s00428-017-2140-0
negative Expression: PTEN ---|	CI28822012	10.1007/s10555-017-9688-7
negative Expression: PTEN ---|	CI28910818	10.1159/000479231
negative Expression: PTEN ---|	CI28910818	10.1159/000479231
negative Expression: PTEN ---|	CI29422611	10.1038/s41388-018-0125-3
negative Expression: PTEN ---|	CI29767245	10.3892/or.2018.6439
negative Expression: PTEN ---|	CI29765155	10.1038/s41388-018-0303-3
negative Expression: PTEN ---|	CI29855617	10.1038/s41388-018-0347-4
negative Expression: PTEN ---|	CI29996881	10.1186/s13058-018-1004-0
negative Expression: PTEN ---|	CI30277471	10.1172/JCI96610
negative Expression: PTEN ---|	CI30264678	10.2174/1389203719666180928105632
negative Expression: PTEN ---|	CI28315073	10.1007/164_2017_11
negative Expression: PTEN ---|	CI30546089	10.1038/s41388-018-0627-z
negative Expression: PTEN ---|	CI30546089	10.1038/s41388-018-0627-z
negative Expression: PTEN ---|	CI30546089	10.1038/s41388-018-0627-z
negative Expression: PTEN ---|	CI32000802	10.1186/s12943-020-1144-6
negative Expression: PTEN ---|	CI32000802	10.1186/s12943-020-1144-6
negative Expression: PTEN ---|	CD274	10.1152/AJPGI.00197.2019
negative Expression: PTEN ---|	CD274	10.1152/AJPGI.00197.2019
negative Expression: PTEN ---|	CD274	10.1152/AJPGI.00197.2019
negative Expression: PTEN ---|	CD274	10.1152/AJPGI.00197.2019
negative Expression: PTEN ---|	CD274	10.1152/AJPGI.00197.2019
negative Expression: PTEN ---|	CD274	10.1152/AJPGI.00197.2019
negative Expression: PTEN ---|	CD274	10.1152/AJPGI.00197.2019
negative Expression: PTEN ---|	CD274	10.1152/AJPGI.00197.2019
negative Expression: PTEN ---|	CD274	10.1152/AJPGI.00197.2019
negative Expression: PTEN ---|	CD274	10.1152/AJPGI.00197.2019
negative Expression: PTEN ---|	CD274	10.1152/AJPGI.00197.2019
negative Expression: PTEN ---|	CD274	10.1152/AJPGI.00197.2019
negative Expression: PTEN ---|	CD274	10.1152/AJPGI.00197.2019
negative Expression: PTEN ---|	CD274	10.1152/AJPGI.00197.2019
negative Expression: PTEN ---|	CD274	10.1152/AJPGI.00197.2019
negative Expression: PTEN ---|	CD274	10.1152/AJPGI.00197.2019
negative Expression: PTEN ---|	CD274	10.1152/AJPGI.00197.2019
negative Expression: PTEN ---|	CD274	10.1152/AJPGI.00197.2019
negative Expression: PTEN ---|	CD274	10.1152/AJPGI.00197.2019

negative Expression: PTEN ---| CD274 10.1152/AJPGI.00197.2019
negative Expression: PTEN ---| CD274 10.1152/AJPGI.00197.2019
negative Expression: PTEN ---| CD274 10.1152/AJPGI.00197.2019
negative Expression: PTEN ---| CI 32225076 10.3390/ijms21072286
negative Expression: PTEN ---| CI 32327707 10.1038/s41416-020-0834-6
negative Expression: PTEN ---| CI 31714809 10.5858/arpa.2018-0584-CP
negative Expression: PTEN ---| CI 34445735 10.3390/ijms22169030
negative Expression: PTEN ---| CI 34385702 10.1038/s41591-021-01450-2
negative Expression: PTEN ---| CI 23785454 10.1371/journal.pone.006582110.1371/journal.p
negative Expression: PTEN ---| CI 23785454 10.1371/journal.pone.006582110.1371/journal.p
negative Expression: PTEN ---| CI 1170 10.1016/j.it.2017.01.002
negative Expression: PTEN ---| CI 741 10.1016/j.tibs.2018.09.004
negative Expression: PTEN ---| CD274 10.1016/j.semcancer.2019.08.015
negative Expression: PTEN ---| CD274 10.1016/j.molcel.2019.09.030
negative Expression: PTEN ---| CD274 10.1016/j.prp.2020.152852
negative Expression: PTEN ---| CD274 10.1016/j.molmed.2010.09.006
negative Expression: PTEN ---| CD274 10.1172/JCI96610
negative Expression: PTEN ---| CD274 10.1186/s12943-020-1144-6
negative Expression: PTEN ---| CD274 10.1186/s12943-020-1144-6
negative Expression: PTEN ---| CI 21899511
negative Expression: PTEN ---| CI 23625198 10.1189/jlb.1212621
negative Expression: PTEN ---| CI 23785454 10.1371/journal.pone.0065821
negative Expression: PTEN ---| CI 23785454 10.1371/journal.pone.0065821
negative Expression: PTEN ---| CI 23785454 10.1371/journal.pone.0065821
negative Expression: PTEN ---| CI 23785454 10.1371/journal.pone.0065821
negative Expression: PTEN ---| CI 23785454 10.1371/journal.pone.0065821
negative Expression: PTEN ---| CI 24779345 10.1186/1479-5876-12-107
negative Expression: PTEN ---| CI 25342040 10.3779/j.issn.1009-3419.2014.10.05
negative Expression: PTEN ---| CI 25844720 10.1371/journal.pone.0123410
negative Expression: PTEN ---| CI 26452385
negative Expression: PTEN ---| CI 26762740 10.1016/j.jtho.2015.09.010
negative Expression: PTEN ---| CI 27276060 10.1371/journal.pone.0157176
negative Expression: PTEN ---| CI 24624456
negative Expression: PTEN ---| CI 27133741 10.1016/j.lungcan.2016.03.00110.1016/j.lungca
negative Expression: PTEN ---| CI 30100056 10.1016/j.bbrc.2018.08.03710.1016/j.bbrc.2018
negative Expression: PTEN ---| CI 31604033 10.1152/ajpgi.00197.201910.1152/ajpgi.00197.2
negative Expression: PTEN ---| CI 31604033 10.1152/ajpgi.00197.201910.1152/ajpgi.00197.2
negative Expression: PTEN ---| CI 31604033 10.1152/ajpgi.00197.201910.1152/ajpgi.00197.2
negative Expression: PTEN ---| CD274 10.5858/arpa.2018-0584-CP
negative Expression: PTEN ---| CI 32860047 10.1084/JEM.20191115
negative Expression: PTEN ---| CI 32860047 10.1084/JEM.20191115
negative Expression: PTEN ---| CI 32860047 10.1084/JEM.20191115
negative Expression: PTEN ---| CI 32860047 10.1084/JEM.20191115
negative Expression: PTEN ---| CI 28714940 10.3390/ijms18071547
negative Expression: PTEN ---| CI 24468585 10.1016/j.humimm.2014.01.006
negative Expression: PTEN ---| CD274
negative Expression: PTEN ---| CD274 10.1016/j.semcdb.2021.04.001
negative Expression: PTEN ---| CI 34804985 10.3389/fonc.2021.78172010.3389/fonc.2021.781
negative Expression: PTEN ---| CI 34948346 10.3390/ijms222413550
negative Expression: PTEN ---| CI 35034965 10.1038/s41388-021-02144-2
negative Expression: PTEN ---| CI 35350588 10.3892/ol.2022.1326910.3892/ol.2022.13269
negative Expression: PTEN ---| CI 35350588 10.3892/ol.2022.1326910.3892/ol.2022.13269
negative Expression: PTEN ---| CI 32917780 10.2967/jnumed.120.250720
negative Expression: PTEN ---| CI 35021970 10.2174/1389450123666220111114649

negative Expression: PTEN ---| CI	35236543	10.3727/096504022X16462176651719
negative Expression: PTEN ---| CI	35541919	10.7150/ijbs.72534
negative Expression: PTEN ---| CI	35783156	10.1155/2022/1531353
negative Expression: PTEN ---| CI	35301651	10.1007/s11033-022-07246-y
negative Expression: PTEN ---| CI	35301651	10.1007/s11033-022-07246-y
negative Expression: PTEN ---| CI	35301651	10.1007/s11033-022-07246-y
negative Expression: PTEN ---| CD274		10.1016/j.gendis.2022.07.024
negative Expression: PTEN ---| CI	35817840	10.1038/s41388-022-02406-7
negative Expression: PTEN ---| CI	35817840	10.1038/s41388-022-02406-7
negative Expression: PTEN ---| CI	35817840	10.1038/s41388-022-02406-7
negative Expression: PTEN ---| CI	36233088	10.3390/ijms231911789
Regulation: CD274 ---> PTEN	27660198	10.1016/j.cellimm.2016.09.009
Regulation: CD274 ---> PTEN	32392177	10.1136/esmoopen-2019-000573
Regulation: CD274 ---> PTEN	26779629	10.1007/s13277-016-4812-9
Regulation: CD274 ---> PTEN	28703774	10.3390/ijms18071517
Regulation: CD274 ---> PTEN	28982322	10.2174/1381612823666171004120152
Regulation: CD274 ---> PTEN	30506105	10.1007/s10753-018-0939-8
Regulation: CD274 ---> PTEN	31792623	10.1007/s00404-019-05401-8
Regulation: CD274 ---> PTEN	32522267	10.1186/s12964-020-00599-6
Regulation: CD274 ---> PTEN	21899511	
Regulation: CD274 ---> PTEN	24551119	10.1371/journal.pone.0088557
Regulation: CD274 ---> PTEN	35409396	10.3390/ijms23074037
Regulation: CD274 ---> PTEN	35301651	10.1007/s11033-022-07246-y
positive Expression: NGF --+> SPF	12593600	10.1902/jop.2003.74.1.76
positive Expression: NGF --+> SPF	17720554	10.1016/j.cellbi.2007.06.012
positive Expression: NGF --+> SPF	17392476	
positive Expression: NGF --+> SPF	18045880	
positive Expression: NGF --+> SPF	15686872	10.1016/j.reprotox.2004.09.004
positive Expression: NGF --+> SPF	18045880	10.1074/jbc.M704913200
positive Expression: NGF --+> SPP1		
positive Expression: NGF --+> SPF	35683016	10.3390/ijms23116337
positive Regulation: RAC1 --+> NF	15684429	10.1074/jbc.M405073200
positive Regulation: RAC1 --+> NF	15684429	10.1074/jbc.M405073200
positive Regulation: RAC1 --+> NF	15684429	10.1074/jbc.M405073200
positive Regulation: RAC1 --+> NF	15684429	10.1074/jbc.M405073200
positive Regulation: RAC1 --+> NE	22330068	10.1016/j.freeradbiomed.2012.02.004
positive Regulation: RAC1 --+> NF	15684429	
positive Regulation: RAC1 --+> NF	15684429	
positive Regulation: RAC1 --+> NF	15684429	
positive Regulation: RAC1 --+> NF	15684429	
positive Regulation: RAC1 --+> NF	15684429	
positive Regulation: RAC1 --+> NE	27910850	10.1038/cr.2016.141
positive Regulation: RAC1 --+> NE	23792411	
positive Regulation: RAC1 --+> NE	23792411	
positive Regulation: RAC1 --+> NE	23792411	
positive Regulation: RAC1 --+> NE	23792411	
positive Regulation: RAC1 --+> NE	23792411	
positive Regulation: RAC1 --+> NE	23792411	
positive Regulation: RAC1 --+> NE	24589508	10.4161/cam.28058
positive Regulation: RAC1 --+> NE	25486363	10.4161/15384101.2015.945865
positive Regulation: RAC1 --+> NE	23184663	10.1128/MCB.00980-12
positive Regulation: RAC1 --+> NE	23184663	10.1128/MCB.00980-12
positive Regulation: RAC1 --+> NE	23184663	10.1128/MCB.00980-12
positive Regulation: RAC1 --+> NE	23184663	10.1128/MCB.00980-12

positive Regulation: RAC1 --+> NF 23184663 10.1128/MCB.00980-12
positive Regulation: RAC1 --+> NF 23184663 10.1128/MCB.00980-12
positive Regulation: RAC1 --+> NF 23184663 10.1128/MCB.00980-12
positive Regulation: RAC1 --+> NF 23184663 10.1128/MCB.00980-12
positive Regulation: RAC1 --+> NF 23184663 10.1128/MCB.00980-12
positive Regulation: RAC1 --+> NF 23184663 10.1128/MCB.00980-12
positive Regulation: RAC1 --+> NF 23184663 10.1128/MCB.00980-12
positive Regulation: RAC1 --+> NF 23184663 10.1128/MCB.00980-12
positive Regulation: RAC1 --+> NF 23184663 10.1128/MCB.00980-12
positive Regulation: RAC1 --+> NF 23184663 10.1128/MCB.00980-12
positive Regulation: RAC1 --+> NF 23184663 10.1128/MCB.00980-12
positive Regulation: RAC1 --+> NF 23184663 10.1128/MCB.00980-12
positive Regulation: RAC1 --+> NF 23184663 10.1128/MCB.00980-12
positive Regulation: RAC1 --+> NF 25588829 10.1242/jcs.158121
positive Regulation: RAC1 --+> NF 25588829 10.1242/jcs.158121
positive Regulation: RAC1 --+> NF 25588829 10.1242/jcs.158121
positive Regulation: RAC1 --+> NF 23184663 10.1128/MCB.00980-1210.1128/MCB.00980-12
positive Regulation: RAC1 --+> NF 23184663 10.1128/MCB.00980-1210.1128/MCB.00980-12
positive Regulation: RAC1 --+> NF 23792411 10.4161/cam.2527610.4161/cam.25276
positive Regulation: RAC1 --+> NF 23792411 10.4161/cam.2527610.4161/cam.25276
positive Expression: NGF --+> PRF 17428473 10.1016/j.yexcr.2007.02.014
positive Expression: NGF --+> PRF 11113316 10.1016/S0306-4522(00)00423-1
positive Expression: NGF --+> PRF 11113316 10.1016/S0306-4522(00)00423-1
positive Expression: NGF --+> PRF 16418002 10.1016/j.conb.2006.01.002
positive Expression: NGF --+> PRF 14595112
positive Expression: NGF --+> PRF 8798778
positive Expression: NGF --+> PRF 7896848
positive Expression: NGF --+> PRF 15673657
positive Expression: NGF --+> PRF 23695304 10.3892/ijmm.2013.1387
positive Expression: NGF --+> PRF 1588954 10.1128/mcb.12.6.2501
positive Expression: NGF --+> PRF 7510697
positive Expression: NGF --+> PRF 7510697
positive Expression: NGF --+> PRF 7896848 10.1074/jbc.270.12.6975
positive Expression: NGF --+> PRF 8798778 10.1074/jbc.271.40.24976
positive Expression: NGF --+> PRF 11573983 10.1006/exnr.2001.7744
negative Regulation: CD274 ---| / 919 10.1016/j.it.2016.11.005
negative Regulation: CD274 ---| / 445 10.1016/j.maturitas.2017.04.014
negative Regulation: CD274 ---| / 26321679 10.1016/j.cell.2015.08.016
negative Regulation: CD274 ---| / 28703774 10.3390/ijms18071517
negative Regulation: CD274 ---| / 28692048 10.1038/onc.2017.209
negative Regulation: CD274 ---| / 28692048 10.1038/onc.2017.209
negative Regulation: CD274 ---| / 28692048 10.1038/onc.2017.209
negative Regulation: CD274 ---| / 28982322 10.2174/1381612823666171004120152
negative Regulation: CD274 ---| / 30078052 10.1007/s00262-018-2221-1
negative Regulation: CD274 ---| / 30808044 10.1055/s-0039-1679909
negative Regulation: CD274 ---| / 21899511
negative Regulation: CD274 ---| / 22337882 10.1074/jbc.M111.283705
negative Regulation: CD274 ---| / 23267208 10.2332/allergolint.12-OA-0450
negative Regulation: CD274 ---| AKT1 10.1097/SHK.0000000000000247
negative Regulation: CD274 ---| / 25609381 10.1038/ncomms6997
negative Regulation: CD274 ---| / 26927206 10.1146/annurev-immunol-032414-112049
negative Regulation: CD274 ---| / 27671847 10.1002/stem.250910.1002/stem.2509
negative Regulation: CD274 ---| / 29245948 10.18632/oncotarget.2191410.18632/oncotarget.
negative Regulation: CD274 ---| / 20008522

negative Regulation: CD274 ---| /28692048 10.1038/onc.2017.209
negative Regulation: CD274 ---| /18941206
negative Regulation: CD274 ---| /27798164 10.4049/jimmunol.1601083
negative Regulation: CD274 ---| AKT1 10.1016/j.humimm.2011.12.011
negative Regulation: CD274 ---| /28887097 10.1016/j.urolonc.2017.08.010
negative Regulation: CD274 ---| /29706653 10.1038/s41388-018-0252-x
negative Regulation: CD274 ---| AKT1
negative Regulation: CD274 ---| AKT1 10.1016/j.semcancer.2019.08.015
negative Regulation: CD274 ---| AKT1 10.1016/j.cellimm.2019.103957
negative Regulation: CD274 ---| /31850228 10.3389/fonc.2019.0134710.3389/fonc.2019.0134
negative Regulation: CD274 ---| AKT1 10.3892/or.2020.7477
negative Regulation: CD274 ---| AKT1 10.3892/or.2020.7477
negative Regulation: CD274 ---| /30368626 10.1007/s10637-018-0678-6
negative Regulation: CD274 ---| /32161124 10.1073/pnas.1921445117
negative Regulation: CD274 ---| /32161124 10.1073/pnas.1921445117
negative Regulation: CD274 ---| /32161124 10.1073/pnas.1921445117
negative Regulation: CD274 ---| /32161124 10.1073/pnas.1921445117
negative Regulation: CD274 ---| /32277292 10.1007/s12032-020-01372-y10.1007/s12032-020-
negative Regulation: CD274 ---| AKT1 10.1038/s41416-019-0697-x
negative Regulation: CD274 ---| /32868929 10.1038/s41590-020-0769-3
negative Expression: BECN1 ---| F24905997 10.4161/auto.28771
negative Expression: BECN1 ---| PTGS2
positive Expression: NOTCH1 --+> 21156799 10.1074/jbc.M110.192393
positive Expression: NOTCH1 --+> 21156799 10.1074/jbc.M110.192393
positive Expression: NOTCH1 --+> 20381235 10.1016/j.canlet.2010.03.015
positive Expression: NOTCH1 --+> 23735298 10.1016/j.scr.2013.04.009
positive Expression: NOTCH1 --+> 19228814
positive Expression: NOTCH1 --+> 19228814
positive Expression: NOTCH1 --+> 19228814
positive Expression: NOTCH1 --+> 19710690
positive Expression: NOTCH1 --+> 19290049
positive Expression: NOTCH1 --+> 19290049
positive Expression: NOTCH1 --+> 19290049
positive Expression: NOTCH1 --+> 19290049
positive Expression: NOTCH1 --+> 19290049
positive Expression: NOTCH1 --+> 28586004 10.3892/ijmm.2017.3011
positive Expression: NOTCH1 --+> 29781036 10.3892/or.2018.6447
positive Expression: NOTCH1 --+> 29781036 10.3892/or.2018.6447
positive Expression: NOTCH1 --+> 29781036 10.3892/or.2018.6447
positive Expression: NOTCH1 --+> 21156799 10.1074/jbc.M110.192393
positive Expression: NOTCH1 --+> 21156799 10.1074/jbc.M110.192393
positive Expression: NOTCH1 --+> 21156799 10.1074/jbc.M110.192393
positive Expression: NOTCH1 --+> 21156799 10.1074/jbc.M110.192393
positive Expression: NOTCH1 --+> 23468978 10.1371/journal.pone.0057382
positive Expression: NOTCH1 --+> 23468978 10.1371/journal.pone.0057382
positive Expression: NOTCH1 --+> 23468978 10.1371/journal.pone.0057382
positive Expression: NOTCH1 --+> 26648421 10.3892/ijo.2015.3280
positive Expression: NOTCH1 --+> 19290049 10.1371/journal.pone.000491110.1371/journal.p
positive Expression: NOTCH1 --+> 23053901 10.1007/s10620-012-2434-710.1007/s10620-012-2
positive Expression: NOTCH1 --+> 30794693 10.1371/journal.pone.021274910.1371/journal.p
positive Expression: NOTCH1 --+> 31948324 10.1080/15384101.2020.171157810.1080/15384101
positive Expression: NOTCH1 --+> 20488613 10.1016/j.canlet.2010.04.025
positive Expression: NOTCH1 --+> 24875648 10.1016/j.biocel.2014.05.021
positive Expression: NOTCH1 --+> 19290049

positive Expression: NOTCH1 --+> 19290049
positive Expression: NOTCH1 --+> 19228814
positive Expression: NOTCH1 --+> 19290049
positive Expression: NOTCH1 --+> 19290049 10.1371/journal.pone.0004911
positive Expression: NOTCH1 --+> 19228814 10.1189/jlb.0908561
positive Expression: NOTCH1 --+> PTGS2
positive Expression: PTEN --+> SF 19549529 10.1053/j.gastro.2009.06.039
positive Expression: PTEN --+> SF 24893171 10.1371/journal.pone.0097544
positive Expression: PTEN --+> SF 15705860
positive Expression: PTEN --+> SF 15705860
positive Expression: PTEN --+> SF 12460992
positive Expression: PTEN --+> SF 10970827
positive Expression: PTEN --+> SF 25754821 10.1016/j.neuron.2015.02.017
positive Expression: PTEN --+> SF 28505114 10.3390/ijms18051058
positive Expression: NOTCH1 --+> 23468978 10.1371/journal.pone.0057382
positive Expression: NOTCH1 --+> 26398771 10.3892/or.2015.4274
positive Expression: NOTCH1 --+> 26398771 10.3892/or.2015.427410.3892/or.2015.4274
positive Expression: NOTCH1 --+> 32792479 10.1038/s41419-020-02873-4
positive Expression: NOTCH1 --+> 32792479 10.1038/s41419-020-02873-4
positive Expression: NOTCH1 --+> 24472723 10.1124/jpet.113.20756310.1124/jpet.113.20756
positive Expression: NOTCH1 --+> 23533157 10.1002/stem.137310.1002/stem.1373
positive Expression: NOTCH1 --+> MAPK1 10.1074/jbc.M116.720698
positive Expression: NOTCH1 --+> MAPK1 10.1074/jbc.M116.720698
positive Expression: NOTCH1 --+> 24472723 10.1124/jpet.113.207563
positive Regulation: IGFBP4 --+> 28179206 10.1016/j.neulet.2017.01.066
positive Regulation: IGFBP4 --+> 35688891 10.1038/s41431-022-01129-3
positive Regulation: IGFBP4 --+> 23499909 10.1016/j.cellsig.2013.02.018
positive Regulation: IGFBP4 --+> 23499909 10.1016/j.cellsig.2013.02.018
positive Regulation: IGFBP4 --+> 23639626 10.1016/j.tox.2013.04.014
positive Regulation: IGFBP4 --+> 15572521
positive Regulation: IGFBP4 --+> 23499909 10.1016/j.cellsig.2013.02.01810.1016/j.cellsi
positive Regulation: IGFBP4 --+> 23971193 10.2478/pjvs-2013-0036
positive Regulation: IGFBP4 --+> 29992318 10.1093/nar/gky58910.1093/nar/gky589
positive Regulation: IGFBP4 --+> AKT1 10.2174/1381612828666220608122934
positive Regulation: IGFBP4 --+> AKT1 10.2174/1381612828666220608122934
positive Regulation: IGFBP4 --+> 35688891 10.1038/s41431-022-01129-3
positive Regulation: IGFBP4 --+> 35688891 10.1038/s41431-022-01129-3
positive Regulation: IGFBP4 --+> 35688891 10.1038/s41431-022-01129-3
positive Expression: NGF --+> TP5 10978315 10.1074/jbc.M003146200
positive Expression: NGF --+> TP5 18036557 10.1016/j.bbrc.2007.11.051
positive Expression: NGF --+> TP5 10707967 10.1016/S0896-6273(00)80866-1
positive Expression: NGF --+> TP5 21406225 10.1016/j.cellsig.2011.03.009
positive Expression: NGF --+> TP5 21406225 10.1016/j.cellsig.2011.03.009
positive Expression: NGF --+> TP5 23036615 10.1016/j.neuro.2012.09.011
positive Expression: NGF --+> TP5 10978315
positive Expression: NGF --+> TP5 9295352
positive Expression: NGF --+> TP53 10.1007/s11055-016-0342-6
positive Expression: NGF --+> TP5 18177422 10.1111/j.1432-0436.2007.00261.x10.1111/j.143
positive Expression: NGF --+> TP5 9852160
positive Expression: NGF --+> TP5 23778138 10.1172/JCI65988
positive Expression: NGF --+> TP5 12679365
positive Expression: NGF --+> TP53
positive Expression: NGF --+> TP5 35916975 10.1007/s12035-022-02966-5
negative Expression: CYP2J2 ---| 22028915 10.1371/journal.pone.0026591

positive MolTransport: NGF --+> 115120643 10.1016/j.bbrc.2004.04.065
positive MolTransport: NGF --+> 114525955
positive MolTransport: NGF --+> 114525955
positive MolTransport: NGF --+> 114525955
positive MolTransport: NGF --+> 114525955
positive MolTransport: NGF --+> 114525955
positive MolTransport: NGF --+> 114525955
positive MolTransport: NGF --+> 114525955
positive MolTransport: NGF --+> 114525955 10.1210/me.2003-0190
positive MolTransport: NGF --+> TP53
Expression: MAPK1 ---> SLCO3A1 30063921 10.1053/j.gastro.2018.07.031
Expression: MAPK1 ---> SLCO3A1 30063921 10.1053/j.gastro.2018.07.031
Regulation: SLCO3A1 ---> MAPK1 24945726 10.1371/journal.pone.0100515
Regulation: SLCO3A1 ---> MAPK1 24945726 10.1371/journal.pone.0100515
Regulation: SLCO3A1 ---> MAPK1 24945726 10.1371/journal.pone.0100515
Regulation: SLCO3A1 ---> MAPK1 24945726 10.1371/journal.pone.0100515
Regulation: SLCO3A1 ---> MAPK1 24945726 10.1371/journal.pone.0100515
Regulation: SLCO3A1 ---> MAPK1 24945726 10.1371/journal.pone.0100515
Regulation: SLCO3A1 ---> MAPK1 24945726 10.1371/journal.pone.010051510.1371/journal.p
negative miRNAEffect: MIR107 ---|30149689 10.3390/ijms19092531
negative miRNAEffect: MIR107 ---|25197016 10.1186/1471-2407-14-656
negative miRNAEffect: MIR107 ---|31346547 10.1515/med-2019-000910.1515/med-2019-0009
negative miRNAEffect: MIR107 ---|29111166 10.1016/j.yexcr.2017.10.03310.1016/j.yexcr.20
negative miRNAEffect: MIR107 ---|33901009 10.18632/aging.202908
negative miRNAEffect: MIR107 ---|29111166 10.1016/j.yexcr.2017.10.033
negative miRNAEffect: MIR107 ---|30885433 10.1016/j.bbrc.2019.03.009
negative miRNAEffect: MIR107 ---|30909133 10.1016/j.intimp.2019.03.00510.1016/j.intimp.
negative miRNAEffect: MIR107 ---|33901009 10.18632/aging.202908
negative miRNAEffect: MIR107 ---|33292283 10.1186/s12935-020-01660-7
negative miRNAEffect: MIR107 ---|130 10.1016/j.yexcr.2017.10.033
negative miRNAEffect: MIR107 ---|25503114
negative miRNAEffect: MIR107 ---| AKT1
negative miRNAEffect: MIR107 ---|30149689 10.3390/ijms19092531
negative miRNAEffect: MIR107 ---| AKT1
negative miRNAEffect: MIR107 ---| AKT1 10.1042/BSR20200098
negative miRNAEffect: MIR107 ---|33243106 10.2174/0929867326666191016113631
negative miRNAEffect: MIR107 ---| AKT1 10.1186/s12935-020-01660-7
negative miRNAEffect: MIR107 ---|35041330 10.1097/MPA.0000000000001934
negative Regulation: TP53 ---| AKT1 10.1016/j.bbrc.2019.02.089
negative Regulation: TP53 ---| AK32161037 10.1523/ENEURO.0438-19.202010.1523/ENEURO.043
negative Regulation: TP53 ---| AKT1 10.1016/j.prp.2019.152531
negative Regulation: TP53 ---| AK15001399 10.1016/j.abb.2004.01.004
negative Regulation: TP53 ---| AK15001399 10.1016/j.abb.2004.01.004
negative Regulation: TP53 ---| AK15001399 10.1016/j.abb.2004.01.004
negative Regulation: TP53 ---| AK15001399 10.1016/j.abb.2004.01.004
negative Regulation: TP53 ---| AK12213580 10.1016/S0006-2952(02)01149-8
negative Regulation: TP53 ---| AK20362557 10.1016/j.bcp.2010.03.031
negative Regulation: TP53 ---| AK22142888 10.1016/j.bcp.2011.11.022
negative Regulation: TP53 ---| AK16648169 10.1529/biophysj.105.077693
negative Regulation: TP53 ---| AK16648169 10.1529/biophysj.105.077693
negative Regulation: TP53 ---| AKT1 10.1016/j.bpj.2010.07.042
negative Regulation: TP53 ---| AKT1 10.1016/j.bpj.2010.07.042
negative Regulation: TP53 ---| AK17935714 10.1016/j.yexcr.2007.08.022
negative Regulation: TP53 ---| AK22192357 10.1016/j.febslet.2011.11.034

negative Regulation: TP53 ---| AK 15001356 10.1016/j.jmb.2003.10.081
negative Regulation: TP53 ---| AK 19203586 10.1016/j.cell.2008.12.002
negative Regulation: TP53 ---| AK 19203586 10.1016/j.cell.2008.12.002
negative Regulation: TP53 ---| AK 19203586 10.1016/j.cell.2008.12.002
negative Regulation: TP53 ---| AK 21377268 10.1016/j.canlet.2011.02.008
negative Regulation: TP53 ---| AK 20951769 10.1016/j.bbcan.2010.10.003
negative Regulation: TP53 ---| AK 21632182 10.1016/j.mehy.2011.05.006
negative Regulation: TP53 ---| AK 15475174 10.1016/j.toxlet.2004.06.013
negative Regulation: TP53 ---| AK 18448277 10.1016/j.toxlet.2008.03.012
negative Regulation: TP53 ---| AK 19840842 10.1016/j.exger.2009.10.009
negative Regulation: TP53 ---| AK 19840842 10.1016/j.exger.2009.10.009
negative Regulation: TP53 ---| AK 18534819 10.1016/j.cellsig.2008.04.011
negative Regulation: TP53 ---| AK 14624950 10.1016/S0899-9007(03)00174-6
negative Regulation: TP53 ---| AKT1 10.1016/j.ejca.2005.02.011
negative Regulation: TP53 ---| AKT1 10.1016/j.ejca.2005.02.011
negative Regulation: TP53 ---| AKT1 10.1016/j.ejca.2005.02.011
negative Regulation: TP53 ---| AK 16084079 10.1016/j.ejca.2005.03.033
negative Regulation: TP53 ---| AKT1 10.1016/j.tibs.2011.10.002
negative Regulation: TP53 ---| AKT1 10.1016/j.semcdb.2004.01.002
negative Regulation: TP53 ---| AK 11545734 10.1016/S1097-2765(01)00323-9
negative Regulation: TP53 ---| AK 12742394 10.1016/S1350-9462(03)00035-1
negative Regulation: TP53 ---| AK 19520599 10.1016/j.cytogfr.2009.05.007
negative Regulation: TP53 ---| AK 19648052 10.1016/j.drup.2009.07.001
negative Regulation: TP53 ---| AK 19411072 10.1016/j.ccr.2009.03.021
negative Regulation: TP53 ---| AK 19796712 10.1016/j.mito.2009.09.006
negative Regulation: TP53 ---| AK 22999923 10.1016/j.neulet.2012.09.022
negative Regulation: TP53 ---| AK 22975375 10.1016/j.ccr.2012.08.001
negative Regulation: TP53 ---| AK 23870914 10.1016/j.foodchem.2013.05.021
negative Regulation: TP53 ---| AK 24113167 10.1016/j.bbapap.2013.09.019
negative Regulation: TP53 ---| AK 24060999 10.1016/j.jprot.2013.09.009
negative Regulation: TP53 ---| AK 24333688 10.1016/j.pharmthera.2013.12.005
negative Regulation: TP53 ---| AK 24374014 10.1016/j.canlet.2013.12.025
negative Regulation: TP53 ---| AK 24355212 10.1016/j.freeradbiomed.2013.12.010
negative Regulation: TP53 ---| AKT1 10.1016/j.bbamem.2014.10.036
negative Regulation: TP53 ---| AKT1 10.1016/j.bbrc.2014.11.018
negative Regulation: TP53 ---| AK 25016051 10.1016/j.ygcen.2014.06.029
negative Regulation: TP53 ---| AK 24915467 10.1016/j.mad.2014.06.002
negative Regulation: TP53 ---| AK 24857662 10.1016/j.celrep.2014.04.046
negative Regulation: TP53 ---| AK 25907832 10.1016/j.ajpath.2015.01.025
negative Regulation: TP53 ---| AK 25446094 10.1016/j.bbrc.2014.11.018
negative Regulation: TP53 ---| AK 26022125 10.1016/j.bbrc.2015.05.069
negative Regulation: TP53 ---| AK 20858413 10.1016/j.bpj.2010.07.042
negative Regulation: TP53 ---| AK 20858413 10.1016/j.bpj.2010.07.042
negative Regulation: TP53 ---| AK 25939952 10.1016/j.toxlet.2015.04.015
negative Regulation: TP53 ---| AK 26066464 10.1016/j.meegid.2015.06.008
negative Regulation: TP53 ---| AK 25450343 10.1016/j.bbamem.2014.10.036
negative Regulation: TP53 ---| AK 26718407 10.1016/j.bbrc.2015.12.070
negative Regulation: TP53 ---| AK 26875545 10.1016/j.plipres.2016.02.001
negative Regulation: TP53 ---| AK 20124728
negative Regulation: TP53 ---| AK 15472230
negative Regulation: TP53 ---| AK 12639981
negative Regulation: TP53 ---| AK 21138557
negative Regulation: TP53 ---| AK 18441250
negative Regulation: TP53 ---| AK 12529380

negative Regulation: TP53 ---| AK 11959846
negative Regulation: TP53 ---| AK 10579725
negative Regulation: TP53 ---| AK 10579725
negative Regulation: TP53 ---| AKT1 10.3727/096504018X15424918479652
negative Regulation: TP53 ---| AK 11782444 10.1101/gad.942902
negative Regulation: TP53 ---| AK 29190376 10.1093/jmcb/mjx05110.1093/jmcb/mjx051
negative Regulation: TP53 ---| AK 10579725 10.1083/jcb.147.5.1063
negative Regulation: TP53 ---| AK 10579725 10.1083/jcb.147.5.1063
negative Regulation: TP53 ---| AKT1 10.1016/j.bmc.2019.115235
negative Regulation: TP53 ---| AK 32014919 10.21873/anticanres.1400810.21873/anticanres.
negative Regulation: TP53 ---| AK 32014919 10.21873/anticanres.1400810.21873/anticanres.
negative Regulation: TP53 ---| AKT1 10.1016/j.jtcme.2020.02.003
negative Regulation: TP53 ---| AKT1 10.1007/s13238-019-0640-1
negative Regulation: TP53 ---| AK 31115790 10.1007/s13238-019-0634-z
negative Regulation: TP53 ---| AK 31115790 10.1007/s13238-019-0634-z
negative Regulation: TP53 ---| AK 31115790 10.1007/s13238-019-0634-z
negative Regulation: TP53 ---| AK 31115790 10.1007/s13238-019-0634-z
negative Regulation: TP53 ---| AK 31115790 10.1007/s13238-019-0634-z
negative Regulation: TP53 ---| AK 31115790 10.1007/s13238-019-0634-z
negative Regulation: TP53 ---| AKT1 10.1038/s41388-019-1022-0
negative Regulation: TP53 ---| AK 32242617 10.1042/BCJ2019088810.1042/BCJ20190888
negative Regulation: TP53 ---| AK 32502598 10.1016/j.semcancer.2020.05.015
negative Regulation: TP53 ---| AK 32274755 10.1007/978-3-030-38266-7_5
negative Regulation: TP53 ---| AK 32275841 10.1080/15384101.2020.174979010.1080/15384101
negative Regulation: TP53 ---| AK 32275841 10.1080/15384101.2020.174979010.1080/15384101
negative Regulation: TP53 ---| AK 32275841 10.1080/15384101.2020.174979010.1080/15384101
negative Regulation: TP53 ---| AK 32275841 10.1080/15384101.2020.1749790
negative Regulation: TP53 ---| AK 32275841 10.1080/15384101.2020.1749790
negative Regulation: TP53 ---| AK 32275841 10.1080/15384101.2020.1749790
negative Regulation: TP53 ---| AK 32275841 10.1080/15384101.2020.1749790
negative Regulation: TP53 ---| AK 32275841 10.1080/15384101.2020.1749790
negative Regulation: TP53 ---| AK 32275841 10.1080/15384101.2020.1749790
negative Regulation: TP53 ---| AK 32275841 10.1080/15384101.2020.1749790
negative Regulation: TP53 ---| AK 32275841 10.1080/15384101.2020.1749790
negative Regulation: TP53 ---| AK 32275841 10.1080/15384101.2020.1749790
negative Regulation: TP53 ---| AK 32275841 10.1080/15384101.2020.1749790
negative Regulation: TP53 ---| AK 32275841 10.1080/15384101.2020.1749790
negative Regulation: TP53 ---| AK 32275841 10.1080/15384101.2020.1749790
negative Regulation: TP53 ---| AK 32275841 10.1080/15384101.2020.1749790
negative Regulation: TP53 ---| AK 32275841 10.1080/15384101.2020.1749790
negative Regulation: TP53 ---| AK 32275841 10.1080/15384101.2020.1749790
negative Regulation: TP53 ---| AK 32275841 10.1080/15384101.2020.1749790
negative Regulation: TP53 ---| AK 32275841 10.1080/15384101.2020.1749790
negative Regulation: TP53 ---| AK 32275841 10.1080/15384101.2020.1749790
negative Regulation: TP53 ---| AK 32275841 10.1080/15384101.2020.1749790
negative Regulation: TP53 ---| AK 32275841 10.1080/15384101.2020.1749790
negative Regulation: TP53 ---| AK 32275841 10.1080/15384101.2020.1749790
negative Regulation: TP53 ---| AK 32275841 10.1080/15384101.2020.1749790
negative Regulation: TP53 ---| AK 32275841 10.1080/15384101.2020.1749790
negative Regulation: TP53 ---| AK 32275841 10.1080/15384101.2020.1749790
negative Regulation: TP53 ---| AK 32275841 10.1080/15384101.2020.1749790
negative Regulation: TP53 ---| AK 32275841 10.1080/15384101.2020.1749790
negative Regulation: TP53 ---| AK 32275841 10.1080/15384101.2020.1749790
negative Regulation: TP53 ---| AK 32275841 10.1080/15384101.2020.1749790

negative Regulation: TP53 ---|	AKT1		10.1016/j.gendis.2020.06.001
negative Regulation: TP53 ---|	AK	32664542	10.3390/ijms2114490210.3390/ijms21144902
negative Regulation: TP53 ---|	AKT1		10.1042/BCJ20190888
negative Regulation: TP53 ---|	AK	32922194	10.7150/ijms.4895510.7150/ijms.48955
negative Regulation: TP53 ---|	AK	33038921	10.1186/s40779-020-00275-310.1186/s40779-020-
negative Regulation: TP53 ---|	AK	32738450	10.1016/j.jhep.2020.07.036
negative Regulation: TP53 ---|	AK	32738450	10.1016/j.jhep.2020.07.036
negative Regulation: TP53 ---|	AK	32223728	10.2174/0929866527666200330172646
negative Regulation: TP53 ---|	AK	32223728	10.2174/0929866527666200330172646
negative Regulation: TP53 ---|	AK	32223728	10.2174/0929866527666200330172646
negative Regulation: TP53 ---|	AK	33184290	10.1038/s41419-020-03185-3
negative Regulation: TP53 ---|	AK	33184290	10.1038/s41419-020-03185-3
negative Regulation: TP53 ---|	AK	33446037	10.1080/15384047.2020.1839278
negative Regulation: TP53 ---|	AK	33688358	10.1155/2021/4652492
negative Regulation: TP53 ---|	AK	33164579	10.1152/AJPHEART.00304.2020
negative Regulation: TP53 ---|	AK	33164579	10.1152/AJPHEART.00304.2020
negative Regulation: TP53 ---|	AK	33238278	10.1159/000511505
negative Regulation: TP53 ---|	AK	34209215	10.3390/ijms22137059
negative Regulation: TP53 ---|	AK	34209215	10.3390/ijms22137059
negative Regulation: TP53 ---|	AK	34209215	10.3390/ijms22137059
negative Regulation: TP53 ---|	AK	34502416	10.3390/ijms22179507
negative Regulation: TP53 ---|	AK	34502416	10.3390/ijms22179507
negative Regulation: TP53 ---|	AK	34416907	10.1186/s13046-021-02061-y
negative Regulation: TP53 ---|	AK	34830349	10.3390/ijms222212466
negative Regulation: TP53 ---|	AK	34813650	10.1042/BCJ20210077
negative Regulation: TP53 ---|	AK	34907345	10.1038/s41388-021-02147-z
negative Regulation: TP53 ---|	AK	34907345	10.1038/s41388-021-02147-z
negative Regulation: TP53 ---|	AK	34907345	10.1038/s41388-021-02147-z
negative Regulation: TP53 ---|	AK	34911439	10.1186/s11658-021-00293-6
negative Regulation: TP53 ---|	AK	34911439	10.1186/s11658-021-00293-6
negative Regulation: TP53 ---|	AKT1		10.3390/molecules27010199
negative Regulation: TP53 ---|	AKT1		10.3390/molecules27010199
negative Regulation: TP53 ---|	AKT1		10.1016/j.ygyno.2022.01.027
negative Regulation: TP53 ---|	AK	19197146	10.4161/cbt.8.3.7443
negative Regulation: TP53 ---|	AK	33785447	10.1016/j.semcancer.2021.03.010
negative Regulation: TP53 ---|	AK	33785447	10.1016/j.semcancer.2021.03.010
negative Regulation: TP53 ---|	AK	33785447	10.1016/j.semcancer.2021.03.010
negative Regulation: TP53 ---|	AK	26359920	10.1139/cjpp-2015-0159
negative Regulation: TP53 ---|	AK	33761957	10.1186/s12929-021-00715-9
negative Regulation: TP53 ---|	AK	34064134	10.3390/ijms22115445
negative Regulation: TP53 ---|	AK	33920054	10.3390/molecules26082278
negative Regulation: TP53 ---|	AK	34205414	10.3390/ijms22126587
negative Regulation: TP53 ---|	AK	34445495	10.3390/ijms22168784
negative Regulation: TP53 ---|	AK	34907345	10.1038/s41388-021-02147-z
negative Regulation: TP53 ---|	AK	34907345	10.1038/s41388-021-02147-z
negative Regulation: TP53 ---|	AK	34907345	10.1038/s41388-021-02147-z
negative Regulation: TP53 ---|	AK	34907345	10.1038/s41388-021-02147-z
negative Regulation: TP53 ---|	AK	34907345	10.1038/s41388-021-02147-z
negative Regulation: TP53 ---|	AK	34907345	10.1038/s41388-021-02147-z
negative Regulation: TP53 ---|	AK	34907345	10.1038/s41388-021-02147-z
negative Regulation: TP53 ---|	AK	34907345	10.1038/s41388-021-02147-z
negative Regulation: TP53 ---|	AK	34907345	10.1038/s41388-021-02147-z
negative Regulation: TP53 ---|	AK	34907345	10.1038/s41388-021-02147-z
negative Regulation: TP53 ---|	AK	34907345	10.1038/s41388-021-02147-z

negative Regulation: TP53 ---| AK34907345 10.1038/s41388-021-02147-z
negative Regulation: TP53 ---| AK34907345 10.1038/s41388-021-02147-z
negative Regulation: TP53 ---| AK33587002 10.1080/01635581.2021.1882509
negative Regulation: TP53 ---| AK26790143 10.1016/j.ejca.2015.12.002
negative Regulation: TP53 ---| AK30827507 10.1016/j.bbrc.2019.02.089
negative Regulation: TP53 ---| AK31733192 10.1016/j.bcp.2019.113713
negative Regulation: TP53 ---| AK12393587 10.1182/blood-2002-02-0504
negative Regulation: TP53 ---| AK32687844 10.1016/j.cbi.2020.109196
negative Regulation: TP53 ---| AK31870856 10.1016/j.yexmp.2019.104362
negative Regulation: TP53 ---| AK30528266 10.1016/j.yexcr.2018.12.002
negative Regulation: TP53 ---| AK30528266 10.1016/j.yexcr.2018.12.002
negative Regulation: TP53 ---| AK30528266 10.1016/j.yexcr.2018.12.002
negative Regulation: TP53 ---| AK30528266 10.1016/j.yexcr.2018.12.002
negative Regulation: TP53 ---| AK30528266 10.1016/j.yexcr.2018.12.002
negative Regulation: TP53 ---| AK20685653 10.1074/jbc.M110.134650
negative Regulation: TP53 ---| AK20685653 10.1074/jbc.M110.134650
negative Regulation: TP53 ---| AK29358327 10.1074/jbc.M117.791145
negative Regulation: TP53 ---| AK24451373 10.1074/jbc.M113.532523
negative Regulation: TP53 ---| AK24451373 10.1074/jbc.M113.532523
negative Regulation: TP53 ---| AK24451373 10.1074/jbc.M113.532523
negative Regulation: TP53 ---| AK24451373 10.1074/jbc.M113.532523
negative Regulation: TP53 ---| AK19339243 10.1074/jbc.M809757200
negative Regulation: TP53 ---| AK30473026 10.1016/j.lfs.2018.10.051
negative Regulation: TP53 ---| AK33333052 10.1016/j.lfs.2020.118814
negative Regulation: TP53 ---| AK32693135 10.1016/j.ijbiomac.2020.07.137
negative Regulation: TP53 ---| AK30170033 10.1016/j.dci.2018.08.017
negative Regulation: TP53 ---| AK30296496 10.1016/j.bbamcr.2018.10.005
negative Regulation: TP53 ---| AK27600832 10.1016/j.bbcan.2016.09.001
negative Regulation: TP53 ---| AK31320250 10.1016/j.prp.2019.152531
negative Regulation: TP53 ---| AK33711551 10.1016/j.biopha.2021.111445
negative Regulation: TP53 ---| AK31953216 10.1016/j.bbadis.2020.165684
negative Regulation: TP53 ---| AK28673691 10.1016/j.mod.2017.06.006
negative Regulation: TP53 ---| AK33773191 10.1016/j.phymed.2021.153534
negative Regulation: TP53 ---| AK31843464 10.1016/j.bmc.2019.115235
negative Regulation: TP53 ---| AK32652464 10.1016/j.jphotobiol.2020.111951
negative Regulation: TP53 ---| AK30641278 10.1016/j.phrs.2019.01.021
negative Regulation: TP53 ---| AK30641278 10.1016/j.phrs.2019.01.021
negative Regulation: TP53 ---| AK33540047 10.1016/j.phrs.2021.105428
negative Regulation: TP53 ---| AK30389549 10.1016/j.biocel.2018.10.008
negative Regulation: TP53 ---| AK31846838 10.1016/j.drup.2019.100670
negative Regulation: TP53 ---| AK32846356 10.1016/j.dnarep.2020.102952
negative Regulation: TP53 ---| AK33717651 10.1016/j.omtn.2021.02.005
negative Regulation: TP53 ---| AK24857662 10.1016/j.celrep.2014.04.046
negative Regulation: TP53 ---| AK32670818 10.1016/j.jtcme.2020.02.003
negative Regulation: TP53 ---| AK15862750 10.1016/j.ejca.2005.02.011
negative Regulation: TP53 ---| AK15862750 10.1016/j.ejca.2005.02.011
negative Regulation: TP53 ---| AK15862750 10.1016/j.ejca.2005.02.011
negative Regulation: TP53 ---| AK22079189 10.1016/j.tibs.2011.10.002
negative Regulation: TP53 ---| AK15209377 10.1016/j.semcdb.2004.01.002
negative Regulation: TP53 ---| AK21999842 10.1186/1476-4598-10-12910.1186/1476-4598-10-
negative Regulation: TP53 ---| AK24451373 10.1074/jbc.M113.53252310.1074/jbc.M113.53252
negative Regulation: TP53 ---| AK24469034 10.1038/onc.2013.58510.1038/onc.2013.585
negative Regulation: TP53 ---| AK24488098 10.1038/cdd.2014.210.1038/cdd.2014.2
negative Regulation: TP53 ---| AK24613379 10.1016/j.freeradbiomed.2014.02.02310.1016/j.

negative Regulation: TP53 ---| AK 25268131 10.1371/journal.pone.010681210.1371/journal.p
negative Regulation: TP53 ---| AK 35414774 10.7150/ijbs.6720010.7150/ijbs.67200
negative Regulation: TP53 ---| AK 34698964 10.1007/s10637-021-01196-1
negative Regulation: TP53 ---| AK 35414774 10.7150/ijbs.67200
negative Regulation: TP53 ---| AK 35414774 10.7150/ijbs.67200
negative Regulation: TP53 ---| AK 35414774 10.7150/ijbs.67200
negative Regulation: TP53 ---| AK 35414774 10.7150/ijbs.67200
negative Regulation: TP53 ---| AK 35146629 10.1007/s10911-022-09511-z
negative Regulation: TP53 ---| AK 35798843 10.1038/s41556-022-00949-1
negative Regulation: TP53 ---| AK 35715817 10.1186/s12964-022-00897-1
negative Regulation: TP53 ---| AK 35682646 10.3390/ijms23115967
negative Regulation: TP53 ---| AK 35879280 10.1038/s41419-022-05106-y
negative Regulation: TP53 ---| AK 36142874 10.3390/ijms231810965
negative Regulation: TP53 ---| AK 36448674 10.1177/15347354221132848
Expression: MAPK1 ---> CLDN1 32553076 10.3967/bes2020.04410.3967/bes2020.044
Expression: MAPK1 ---> CLDN1 30625382 10.1016/j.bbadis.2019.01.008
Expression: MAPK1 ---> CLDN1 35408826 10.3390/ijms23073467
Expression: MAPK1 ---> CLDN1 10.1016/S0002-9440(10)61243-6
Expression: MAPK1 ---> CLDN1 27524860 10.1155/2016/1927348
Expression: MAPK1 ---> CLDN1 18204077 10.1093/carcin/bgn00310.1093/carcin/bgn003
Expression: MAPK1 ---> CLDN1 30362534 10.1002/jcp.2747710.1002/jcp.27477
Expression: MAPK1 ---> CLDN1 30658316 10.1016/j.tranon.2018.12.011
Expression: MAPK1 ---> CLDN1 30227306 10.1016/j.neo.2018.08.010
Expression: MAPK1 ---> CLDN1 35770317 10.1111/exd.14637
Binding: BECN1 ---- MCL1 29555460 10.1016/j.actbio.2018.03.022
Binding: BECN1 ---- MCL1 10.1016/j.canlet.2019.10.030
Binding: BECN1 ---- MCL1 18589438 10.1016/j.jmb.2008.05.071
Binding: BECN1 ---- MCL1 21993018 10.1016/j.canlet.2011.09.020
Binding: BECN1 ---- MCL1 10.1016/j.jdermsci.2011.11.001
Binding: BECN1 ---- MCL1 20159550 10.1016/j.molcel.2010.01.025
Binding: BECN1 ---- MCL1 21353614 10.1016/j.molcel.2011.02.009
Binding: BECN1 ---- MCL1 21353614 10.1016/j.molcel.2011.02.009
Binding: BECN1 ---- MCL1 22735572 10.1016/j.mito.2012.06.003
Binding: BECN1 ---- MCL1 24012660 10.1016/j.semcancer.2013.08.008
Binding: BECN1 ---- MCL1 25194337 10.1016/j.biocel.2014.08.022
Binding: BECN1 ---- MCL1 25194337 10.1016/j.biocel.2014.08.022
Binding: BECN1 ---- MCL1 26009469 10.1016/j.jmb.2015.05.015
Binding: BECN1 ---- MCL1 20200450
Binding: BECN1 ---- MCL1 19912650
Binding: BECN1 ---- MCL1 27527160 10.3390/ijms17081274
Binding: BECN1 ---- MCL1 28234359 10.1038/cdd.2017.22
Binding: BECN1 ---- MCL1 29333953 10.1080/15384047.2018.1423927
Binding: BECN1 ---- MCL1 10.1530/REP-18-0002
Binding: BECN1 ---- MCL1 28622296 10.1038/cdd.2017.82
Binding: BECN1 ---- MCL1 30327467 10.1038/s41467-018-06836-4
Binding: BECN1 ---- MCL1 29808796 10.14715/cmb/2018.64.6.5
Binding: BECN1 ---- MCL1 29611474 10.2174/0929867325666180403145137
Binding: BECN1 ---- MCL1 22263800
Binding: BECN1 ---- MCL1 23152582 10.1194/jlr.R031278
Binding: BECN1 ---- MCL1 22638980 10.1007/s10495-012-0738-x
Binding: BECN1 ---- MCL1 23688351 10.1186/1471-2202-14-56
Binding: BECN1 ---- MCL1 23688351 10.1186/1471-2202-14-56
Binding: BECN1 ---- MCL1 22614243 10.1038/leu.2012.136
Binding: BECN1 ---- MCL1 21139567 10.1038/emboj.2010.327

Binding: BECN1 ---- MCL1	21139567	10.1038/emboj.2010.327
Binding: BECN1 ---- MCL1	21139567	10.1038/emboj.2010.327
Binding: BECN1 ---- MCL1	21139567	10.1038/emboj.2010.327
Binding: BECN1 ---- MCL1	21139567	10.1038/emboj.2010.327
Binding: BECN1 ---- MCL1	21364679	10.1038/cddis.2010.53
Binding: BECN1 ---- MCL1	23681223	10.1038/cddis.2013.155
Binding: BECN1 ---- MCL1	23607597	10.2174/15680096113139990039
Binding: BECN1 ---- MCL1	24107625	10.4161/cc.26696
Binding: BECN1 ---- MCL1	24113155	10.4161/auto.26452
Binding: BECN1 ---- MCL1	24912196	10.1073/pnas.1406425111
Binding: BECN1 ---- MCL1	25885284	10.1186/s12885-015-1239-4
Binding: BECN1 ---- MCL1	26018731	10.1080/15548627.2015.1049800
Binding: BECN1 ---- MCL1	25837021	10.1080/15548627.2015.1029836
Binding: BECN1 ---- MCL1	17643073	10.4161/auto.4713
Binding: BECN1 ---- MCL1	33497829	10.1016/j.drudis.2021.01.021
positive Expression: SFRP1 --+> F	20851881	10.1074/jbc.M110.166231
positive Expression: SFRP1 --+> F	20851881	10.1074/jbc.M110.166231
positive Expression: SFRP1 --+> PPARG		
positive Regulation: NOTCH1 --+> 62		10.1016/j.biopsych.2016.12.021
positive Regulation: NOTCH1 --+> AKT1		10.1053/j.semdp.2020.09.010
positive Regulation: NOTCH1 --+> AKT1		10.1016/j.yjmcc.2012.03.007
positive Regulation: NOTCH1 --+> AKT1		10.1016/j.lfs.2011.10.012
positive Regulation: NOTCH1 --+>	21193018	10.1016/j.bbcan.2010.12.002
positive Regulation: NOTCH1 --+>	22293023	10.1016/j.tcm.2011.11.006
positive Regulation: NOTCH1 --+> AKT1		10.1016/j.urolonc.2011.07.006
positive Regulation: NOTCH1 --+> AKT1		10.1016/j.urolonc.2011.07.006
positive Regulation: NOTCH1 --+>	20675210	10.1016/j.scr.2010.06.002
positive Regulation: NOTCH1 --+>	22465038	10.1016/j.yjmcc.2012.03.007
positive Regulation: NOTCH1 --+>	24095799	10.1016/j.yexcr.2013.09.019
positive Regulation: NOTCH1 --+>	24095799	10.1016/j.yexcr.2013.09.019
positive Regulation: NOTCH1 --+>	24140475	10.1016/j.cellsig.2013.09.021
positive Regulation: NOTCH1 --+>	24140475	10.1016/j.cellsig.2013.09.021
positive Regulation: NOTCH1 --+>	24140475	10.1016/j.cellsig.2013.09.021
positive Regulation: NOTCH1 --+>	24140475	10.1016/j.cellsig.2013.09.021
positive Regulation: NOTCH1 --+>	24140475	10.1016/j.cellsig.2013.09.021
positive Regulation: NOTCH1 --+>	24140475	10.1016/j.cellsig.2013.09.021
positive Regulation: NOTCH1 --+>	24140475	10.1016/j.cellsig.2013.09.021
positive Regulation: NOTCH1 --+>	24140475	10.1016/j.cellsig.2013.09.021
positive Regulation: NOTCH1 --+>	24140475	10.1016/j.cellsig.2013.09.021
positive Regulation: NOTCH1 --+>	24140475	10.1016/j.cellsig.2013.09.021
positive Regulation: NOTCH1 --+>	24140475	10.1016/j.cellsig.2013.09.021
positive Regulation: NOTCH1 --+>	24140475	10.1016/j.cellsig.2013.09.021
positive Regulation: NOTCH1 --+>	21993533	10.1016/j.urolonc.2011.07.006
positive Regulation: NOTCH1 --+>	21993533	10.1016/j.urolonc.2011.07.006
positive Regulation: NOTCH1 --+>	24529510	10.1016/j.jcpa.2013.11.213
positive Regulation: NOTCH1 --+>	25109840	10.1016/j.abb.2014.07.023
positive Regulation: NOTCH1 --+>	22056375	10.1016/j.lfs.2011.10.012
positive Regulation: NOTCH1 --+>	24775271	10.1016/j.imbio.2014.03.020
positive Regulation: NOTCH1 --+>	24775271	10.1016/j.imbio.2014.03.020
positive Regulation: NOTCH1 --+>	24939178	10.1016/j.ejcb.2014.05.004
positive Regulation: NOTCH1 --+>	25799457	10.1016/j.tips.2015.02.002
positive Regulation: NOTCH1 --+> AKT1		10.1016/j.amjsurg.2015.11.029
positive Regulation: NOTCH1 --+>	26850133	10.1016/j.amjsurg.2015.11.029
positive Regulation: NOTCH1 --+>	26967479	10.1016/j.jid.2015.11.006

positive Regulation: NOTCH1 --+> 18924608
positive Regulation: NOTCH1 --+> 18830414
positive Regulation: NOTCH1 --+> 20030805
positive Regulation: NOTCH1 --+> 14583609
positive Regulation: NOTCH1 --+> 14583609
positive Regulation: NOTCH1 --+> 20616044
positive Regulation: NOTCH1 --+> 20616044
positive Regulation: NOTCH1 --+> 18369158
positive Regulation: NOTCH1 --+> 19838160
positive Regulation: NOTCH1 --+> 19265851
positive Regulation: NOTCH1 --+> 15650752
positive Regulation: NOTCH1 --+> 27573203 10.3892/ijo.2016.3598
positive Regulation: NOTCH1 --+> 26846632 10.1007/s13365-015-0394-3
positive Regulation: NOTCH1 --+> 27548161 10.3390/ijms17081357
positive Regulation: NOTCH1 --+> AKT1 10.2174/1381612822666161006160524
positive Regulation: NOTCH1 --+> 28241766 10.1186/s12967-017-1151-6
positive Regulation: NOTCH1 --+> 27593934 10.1038/onc.2016.275
positive Regulation: NOTCH1 --+> 27593934 10.1038/onc.2016.275
positive Regulation: NOTCH1 --+> 27593934 10.1038/onc.2016.275
positive Regulation: NOTCH1 --+> AKT1 10.1074/jbc.M116.759134
positive Regulation: NOTCH1 --+> AKT1 10.1074/jbc.M116.759134
positive Regulation: NOTCH1 --+> 28872614 10.3390/ijms18091904
positive Regulation: NOTCH1 --+> 28849037 10.3892/mmr.2017.7319
positive Regulation: NOTCH1 --+> 28918490 10.1007/s12032-017-1039-6
positive Regulation: NOTCH1 --+> 29095436 10.1038/cddis.2017.543
positive Regulation: NOTCH1 --+> AKT1 10.1074/jbc.M117.790121
positive Regulation: NOTCH1 --+> AKT1 10.1074/jbc.M117.790121
positive Regulation: NOTCH1 --+> AKT1 10.1074/jbc.M117.790121
positive Regulation: NOTCH1 --+> AKT1 10.1074/jbc.M117.790121
positive Regulation: NOTCH1 --+> AKT1 10.1074/jbc.M117.790121
positive Regulation: NOTCH1 --+> AKT1 10.1074/jbc.M117.790121
positive Regulation: NOTCH1 --+> 29321718 10.1186/s12935-017-0496-5
positive Regulation: NOTCH1 --+> 28951560 10.1038/leu.2017.301
positive Regulation: NOTCH1 --+> AKT1 10.1097/PAS.0000000000001042
positive Regulation: NOTCH1 --+> 29545603 10.1038/s41388-018-0188-1
positive Regulation: NOTCH1 --+> 30845764 10.3390/ijms20051151
positive Regulation: NOTCH1 --+> AKT1 10.1139/cjpp-2018-0544
positive Regulation: NOTCH1 --+> AKT1 10.1155/2019/8707053
positive Regulation: NOTCH1 --+> AKT1 10.1186/s13046-019-1319-4
positive Regulation: NOTCH1 --+> AKT1 10.1007/s00280-019-03990-4
positive Regulation: NOTCH1 --+> AKT1 10.1007/s00280-019-03990-4
positive Regulation: NOTCH1 --+> AKT1 10.1007/s00280-019-03990-4
positive Regulation: NOTCH1 --+> 22710837 10.3892/or.2012.1856
positive Regulation: NOTCH1 --+> 22491738 10.3324/haematol.2011.059030
positive Regulation: NOTCH1 --+> 20458516 10.1007/s00018-010-0391-x
positive Regulation: NOTCH1 --+> 22004682 10.1186/1476-4598-10-130
positive Regulation: NOTCH1 --+> 22074495 10.1186/1476-4598-10-138
positive Regulation: NOTCH1 --+> 20616044 10.1073/pnas.1000132107
positive Regulation: NOTCH1 --+> 32792479 10.1038/s41419-020-02873-4
positive Regulation: NOTCH1 --+> 33015852 10.1111/fcp.1260810.1111/fcp.12608
positive Regulation: NOTCH1 --+> 20616044 10.1073/pnas.1000132107
positive Regulation: NOTCH1 --+> 22065781 10.1073/pnas.1104943108
positive Regulation: NOTCH1 --+> 23012411 10.1073/pnas.1206268109
positive Regulation: NOTCH1 --+> 23012411 10.1073/pnas.1206268109

positive Regulation: NOTCH1 --+> 23531541 10.3390/ijms14046863
positive Regulation: NOTCH1 --+> 24140475 10.1016/j.cellsig.2013.09.021
positive Regulation: NOTCH1 --+> 24140475 10.1016/j.cellsig.2013.09.021
positive Regulation: NOTCH1 --+> 18924608
positive Regulation: NOTCH1 --+> AKT1 10.1074/jbc.M116.759134
positive Regulation: NOTCH1 --+> 30105616 10.1007/s00018-018-2905-x
positive Regulation: NOTCH1 --+> 33162798 10.7150/ijms.43184
positive Regulation: NOTCH1 --+> 33176854 10.1186/s13046-020-01750-4
positive Regulation: NOTCH1 --+> 33412518 10.18632/aging.202462
positive Regulation: NOTCH1 --+> 33964962 10.1186/s12935-021-01952-6
positive Regulation: NOTCH1 --+> 34006834 10.1038/s41419-021-03735-3
positive Regulation: NOTCH1 --+> 33802884 10.3390/molecules26061681
positive Regulation: NOTCH1 --+> 34502291 10.3390/ijms22179382
positive Regulation: NOTCH1 --+> 34465724 10.1038/s41389-021-00348-510.1038/s41389-021-
positive Regulation: NOTCH1 --+> 34495871 10.18632/aging.20326110.18632/aging.203261
positive Regulation: NOTCH1 --+> 34495871 10.18632/aging.203261
positive Regulation: NOTCH1 --+> 34495871 10.18632/aging.203261
positive Regulation: NOTCH1 --+> AKT1 10.2298/ABS210423030R
positive Regulation: NOTCH1 --+> AKT1 10.1016/j.jsps.2021.12.009
positive Regulation: NOTCH1 --+> 34995552 10.1016/j.mvr.2021.10430810.1016/j.mvr.2021.1
positive Expression: PTGS2 --+> （33617828 10.1016/j.ejphar.2021.173960
positive Expression: PTGS2 --+> （31884388 10.1016/j.molimm.2019.12.012
positive Expression: PTGS2 --+> （32044411 10.1016/j.jaad.2020.02.006
positive Expression: PTGS2 --+> （32018066 10.1016/j.intimp.2020.106206
positive Expression: PTGS2 --+> （33765613 10.1016/j.intimp.2021.107553
positive Expression: PTGS2 --+> （28231855 10.1186/s12967-017-1150-7
positive Expression: PTGS2 --+> （28231855 10.1186/s12967-017-1150-7
positive Expression: PTGS2 --+> （28231855 10.1186/s12967-017-1150-7
positive Expression: PTGS2 --+> （30061230 10.21873/anticanres.12768
positive Expression: PTGS2 --+> （30061230 10.21873/anticanres.12768
positive Expression: PTGS2 --+> （30061230 10.21873/anticanres.12768
positive Expression: PTGS2 --+> （30061230 10.21873/anticanres.12768
positive Expression: PTGS2 --+> （32234916 10.21873/anticanres.14182
positive Expression: PTGS2 --+> （32823961 10.3390/ijms21165845
positive Expression: PTGS2 --+> （30591531 10.1124/jpet.118.254359
positive Expression: PTGS2 --+> （32942970 10.2174/0929867327999200917150939
positive Expression: PTGS2 --+> （24004819 10.1186/bcr3469
positive Expression: PTGS2 --+> （24004819 10.1186/bcr3469
positive Expression: PTGS2 --+> （24004819 10.1186/bcr3469
positive Expression: PTGS2 --+> （25880839 10.1186/s13075-015-0596-3
positive Expression: PTGS2 --+> （28096371 10.1073/pnas.161292011410.1073/pnas.161292011
positive Expression: PTGS2 --+> （28096371 10.1073/pnas.161292011410.1073/pnas.161292011
positive Expression: PTGS2 --+> （28231855 10.1186/s12967-017-1150-710.1186/s12967-017-1
positive Expression: PTGS2 --+> （30061230 10.21873/anticanres.1276810.21873/anticanres.
positive Expression: PTGS2 --+> （34466408 10.2147/RRU.S32451010.2147/RRU.S324510
positive Expression: PTGS2 --+> CD274 10.1016/j.jaad.2020.02.006
positive Expression: PTGS2 --+> （28231855 10.1186/s12967-017-1150-7
positive Expression: PTGS2 --+> （29808460 10.1007/s10555-018-9729-x
positive Expression: PTGS2 --+> CD274 10.1124/jpet.118.254359
positive Expression: PTGS2 --+> （34987061 10.1158/1940-6207.CAPR-21-0227
positive Expression: PTGS2 --+> （35237322 10.1155/2022/4598573
positive Expression: PTGS2 --+> （35373258 10.1158/1940-6207.CAPR-22-0052
positive Expression: PTGS2 --+> （35373258 10.1158/1940-6207.CAPR-22-0052
positive Expression: PTGS2 --+> （34987061 10.1158/1940-6207.CAPR-21-0227

positive Expression: PTGS2 --+> (34987061 10.1158/1940-6207.CAPR-21-0227
positive Expression: PTGS2 --+> (34987061 10.1158/1940-6207.CAPR-21-0227
positive Expression: PTGS2 --+> (34987061 10.1158/1940-6207.CAPR-21-0227
positive Expression: PTGS2 --+> (35288737 10.1158/1940-6207.CAPR-22-0038
positive Expression: PTGS2 --+> CD274 10.1016/j.immuni.2022.07.001
negative Regulation: MCL1 ---| ch 27847320 10.1016/j.bbrc.2016.11.049
negative Regulation: MCL1 ---| ch 12787138 10.1046/j.1523-1747.2003.12252.x
negative Regulation: MCL1 ---| ch 23954445 10.1016/j.bbamcr.2013.08.006
negative Regulation: MCL1 ---| ch 28343875 10.1016/j.bmcl.2017.03.028
negative Regulation: MCL1 ---| ch 18601905 10.1016/j.bbrc.2008.06.098
negative Regulation: MCL1 ---| ch 20004446 10.1016/j.surg.2009.10.033
negative Regulation: MCL1 ---| ch 20004446 10.1016/j.surg.2009.10.033
negative Regulation: MCL1 ---| ch 12787138
negative Regulation: MCL1 ---| ch 30221675 10.3892/ijo.2018.4557
negative Regulation: MCL1 ---| ch 23257783 10.1038/leu.2012.367
negative Regulation: MCL1 ---| ch 24223823 10.1371/journal.pone.0078570
negative Regulation: MCL1 ---| ch 24982331
negative Regulation: MCL1 ---| ch 18297287 10.1007/s00280-008-0697-710.1007/s00280-008-0
negative Regulation: MCL1 ---| ch 18297287 10.1007/s00280-008-0697-710.1007/s00280-008-0
negative Regulation: MCL1 ---| ch 18297287 10.1007/s00280-008-0697-710.1007/s00280-008-0
negative Regulation: MCL1 ---| ch 27049076
negative Regulation: MCL1 ---| ch 27686452 10.1007/s11010-016-2827-6
negative Regulation: MCL1 ---| ch 31754782 10.1007/s00441-019-03105-810.1007/s00441-019-
negative Regulation: MCL1 ---| ch 746 10.1016/j.bmcl.2017.03.028
negative Regulation: MCL1 ---| ch 34781999 10.1186/s13046-021-02149-5
negative Regulation: MCL1 ---| ch 35031927 10.1007/s11033-021-07040-2
positive Expression: UCA1 --+> MA 31276678 10.1016/j.jid.2019.04.029
positive Expression: UCA1 --+> MA 30468471 10.26355/eurrev-201811-16263
positive Expression: UCA1 --+> MAPK1
positive Expression: UCA1 --+> MA 31276678 10.1016/j.jid.2019.04.029
positive Expression: UCA1 --+> MA 30556875 10.26355/eurrev_201812_16532
positive Expression: UCA1 --+> MA 32843741 10.1038/s41417-020-00214-3
positive Expression: UCA1 --+> MA 33936199 10.1155/2021/5519720
positive Expression: UCA1 --+> MA 32560605 10.2174/1566524020666200619124543
positive Expression: UCA1 --+> MA 31276678 10.1016/j.jid.2019.04.029
positive Expression: UCA1 --+> MA 31276678 10.1016/j.jid.2019.04.029
positive Expression: SNCAIP --+> 20185556 10.1093/hmg/ddq08610.1093/hmg/ddq086
negative QuantitativeChange: hypo 35294329 10.1080/21655979.2022.205126610.1080/21655979
positive Expression: MAPK1 --+> (28237619 10.1016/j.tox.2017.02.008
Regulation: KDM5B ---> RAC1 34455465 10.1007/s00383-021-05005-x
positive Regulation: KDM5B --+> P 1245 10.1016/j.trecan.2017.08.004
positive Regulation: KDM5B --+> P 1245 10.1016/j.trecan.2017.08.004
positive Regulation: KDM5B --+> PTEN 10.3892/mmr.2019.9832
positive Regulation: KDM5B --+> P 25909289 10.18632/oncotarget.3713
positive Regulation: KDM5B --+> P 28958389 10.1016/j.trecan.2017.08.004
positive Regulation: KDM5B --+> P 35946516 10.1097/CAD.0000000000001329
positive Regulation: KDM5B --+> P 28958389 10.1016/j.trecan.2017.08.004
positive Regulation: KDM5B --+> P 30628696 10.3892/mmr.2019.9832
positive Regulation: KDM5B --+> P 35946516 10.1097/CAD.0000000000001329
positive Regulation: KDM5B --+> P 35946516 10.1097/CAD.0000000000001329
positive Regulation: KDM5B --+> P 35946516 10.1097/CAD.0000000000001329
Regulation: GRK4 ---> MAPK1 23693024 10.1016/j.jocn.2012.10.008
Regulation: GRK4 ---> MAPK1 29921886 10.1038/s41401-018-0049-z
Expression: NOTCH1 ---> SPP1 32707422 10.1016/j.cyto.2020.155193

Expression: NOTCH1 ---> SPP1 33162798 10.7150/ijms.43184
Expression: NOTCH1 ---> SPP1 18237408
Expression: NOTCH1 ---> SPP1 16195230
positive Expression: NOTCH1 --+> 29548748 10.1016/j.yexcr.2018.03.014
positive Expression: NOTCH1 --+> 29548748 10.1016/j.yexcr.2018.03.014
positive Expression: NOTCH1 --+> 29548748 10.1016/j.yexcr.2018.03.014
positive Expression: NOTCH1 --+> 29548748 10.1016/j.yexcr.2018.03.014
positive Expression: NOTCH1 --+> 31927049 10.1016/j.lfs.2020.117291
positive Expression: NOTCH1 --+> 30336197 10.1016/j.canlet.2018.09.031
positive Expression: NOTCH1 --+> 28457880 10.1016/j.ctrv.2017.04.003
positive Expression: NOTCH1 --+> 29358129 10.1016/j.biopha.2018.01.086
positive Expression: NOTCH1 --+> 26362310 10.1186/s12885-015-1625-y
positive Expression: NOTCH1 --+> 26362310 10.1186/s12885-015-1625-y
positive Expression: NOTCH1 --+> 26362310 10.1186/s12885-015-1625-y
positive Expression: NOTCH1 --+> 26362310 10.1186/s12885-015-1625-y
positive Expression: NOTCH1 --+> 27468877 10.4149/neo_2016_510
positive Expression: NOTCH1 --+> 27468877 10.4149/neo_2016_510
positive Expression: NOTCH1 --+> 29328395 10.3892/or.2018.6205
positive Expression: NOTCH1 --+> 31357721 10.3390/ijms20153696
positive Expression: NOTCH1 --+> 31964857 10.12659/MSM.919566
positive Expression: NOTCH1 --+> 31571144 10.1007/s12272-019-01183-4
positive Expression: NOTCH1 --+> 31571144 10.1007/s12272-019-01183-4
positive Expression: NOTCH1 --+> 31571144 10.1007/s12272-019-01183-4
positive Expression: NOTCH1 --+> 31571144 10.1007/s12272-019-01183-4
positive Expression: NOTCH1 --+> 31747319 10.1139/cjpp-2019-0448
positive Expression: NOTCH1 --+> 31747319 10.1139/cjpp-2019-0448
positive Expression: NOTCH1 --+> 31747319 10.1139/cjpp-2019-0448
positive Expression: NOTCH1 --+> 31747319 10.1139/cjpp-2019-0448
positive Expression: NOTCH1 --+> 31747319 10.1139/cjpp-2019-0448
positive Expression: NOTCH1 --+> 31747319 10.1139/cjpp-2019-0448
positive Expression: NOTCH1 --+> 31747319 10.1139/cjpp-2019-0448
positive Expression: NOTCH1 --+> 31747319 10.1139/cjpp-2019-0448
positive Expression: NOTCH1 --+> 22143792 10.1073/pnas.1019452108
positive Expression: NOTCH1 --+> 22143792 10.1073/pnas.1019452108
positive Expression: NOTCH1 --+> 22143792 10.1073/pnas.1019452108
positive Expression: NOTCH1 --+> 22143792 10.1073/pnas.1019452108
positive Expression: NOTCH1 --+> 22143792 10.1073/pnas.1019452108
positive Expression: NOTCH1 --+> 22143792 10.1073/pnas.1019452108
positive Expression: NOTCH1 --+> 22143792 10.1073/pnas.1019452108
positive Expression: NOTCH1 --+> 22143792 10.1073/pnas.1019452108
positive Expression: NOTCH1 --+> 22143792 10.1073/pnas.1019452108
positive Expression: NOTCH1 --+> 24700253 10.3892/or.2014.3123
positive Expression: NOTCH1 --+> 24700253 10.3892/or.2014.3123
positive Expression: NOTCH1 --+> 24700253 10.3892/or.2014.3123
positive Expression: NOTCH1 --+> 24700253 10.3892/or.2014.3123
positive Expression: NOTCH1 --+> 25763542 10.1097/CAD.0000000000000227
positive Expression: NOTCH1 --+> 22143792 10.1073/pnas.101945210810.1073/pnas.101945210
positive Expression: NOTCH1 --+> 24782036 10.1007/s11010-014-2069-410.1007/s11010-014-2
positive Expression: NOTCH1 --+> 24782036 10.1007/s11010-014-2069-410.1007/s11010-014-2
positive Expression: NOTCH1 --+> 26362310 10.1186/s12885-015-1625-y10.1186/s12885-015-1
positive Expression: NOTCH1 --+> 27468877 10.4149/neo_2016_51010.4149/neo_2016_510
positive Expression: NOTCH1 --+> 27468877 10.4149/neo_2016_51010.4149/neo_2016_510
positive Expression: NOTCH1 --+> 29548748 10.1016/j.yexcr.2018.03.01410.1016/j.yexcr.20
positive Expression: NOTCH1 --+> 31747319 10.1139/cjpp-2019-044810.1139/cjpp-2019-0448

positive Expression: NOTCH1 --+> ABCC1 10.3390/ijms20153696
positive Expression: NOTCH1 --+> ABCC1 10.12659/MSM.919566
positive Expression: NOTCH1 --+> ABCC1 10.1007/s12272-019-01183-4
positive Expression: NOTCH1 --+> ABCC1 10.1007/s12272-019-01183-4
positive Expression: NOTCH1 --+> ABCC1 10.1007/s12272-019-01183-4
positive Expression: NOTCH1 --+> ABCC1 10.1007/s12272-019-01183-4
positive Expression: NOTCH1 --+> ABCC1 10.1139/cjpp-2019-0448
positive Expression: NOTCH1 --+> ABCC1 10.1139/cjpp-2019-0448
positive Expression: NOTCH1 --+> ABCC1 10.1139/cjpp-2019-0448
positive Expression: NOTCH1 --+> ABCC1 10.1139/cjpp-2019-0448
positive Expression: NOTCH1 --+> ABCC1 10.1139/cjpp-2019-0448
positive Expression: NOTCH1 --+> ABCC1 10.1139/cjpp-2019-0448
positive Expression: NOTCH1 --+> ABCC1 10.1139/cjpp-2019-0448
positive Expression: NOTCH1 --+> ABCC1 10.1139/cjpp-2019-0448
positive Expression: NOTCH1 --+> ABCC1 10.1097/CAD.0000000000000227
positive Expression: NOTCH1 --+> 23458608 10.1016/j.pharmthera.2013.02.003
positive Expression: NOTCH1 --+> 23458608 10.1016/j.pharmthera.2013.02.003
positive Expression: NOTCH1 --+> 24050699 10.1146/annurev-pharmtox-011613-135959
positive Expression: NOTCH1 --+> 22143792 10.1073/pnas.1019452108
positive Expression: NOTCH1 --+> ABCC1
positive Expression: NOTCH1 --+> ABCC1 10.2174/1381612822666160302103646
positive Expression: NOTCH1 --+> ABCC1 10.1016/j.drup.2021.100795
positive Expression: NOTCH1 --+> 35163586 10.3390/ijms23031665
Expression: GPER1 ---> PTGS2 30092317 10.1016/j.dci.2018.08.001
Expression: GPER1 ---> PTGS2 33035625 10.1016/j.mce.2020.111053
Expression: GPER1 ---> PTGS2 29747742 10.1016/j.phymed.2018.03.039
Expression: GPER1 ---> PTGS2 29747742 10.1016/j.phymed.2018.03.039
Expression: GPER1 ---> PTGS2 26241029 10.1016/j.jsbmb.2015.07.019
Expression: GPER1 ---> PTGS2 32791253 10.1016/j.cbpc.2020.108868
Expression: GPER1 ---> PTGS2 30705108 10.1042/CS20180919
Expression: GPER1 ---> PTGS2 30705108 10.1042/CS2018091910.1042/CS20180919
Expression: GPER1 ---> PTGS2 28347854 10.1016/j.jsbmb.2017.03.021
Expression: GPER1 ---> PTGS2 26241029 10.1016/j.jsbmb.2015.07.019
Expression: GPER1 ---> PTGS2 26241029 10.1016/j.jsbmb.2015.07.019
Expression: GPER1 ---> PTGS2 10.1016/j.bbrc.2019.05.045
Expression: GPER1 ---> PTGS2 10.1042/CS20180919
negative Expression: SPP1 ---| N(21747408 10.1038/icb.2011.47
negative Expression: SPP1 ---| N(23800491 10.1016/j.exphem.2013.06.005
positive PromoterBinding: PPARG - 28416286 10.1016/j.stemcr.2017.03.008
positive PromoterBinding: PPARG - 29311623 10.1038/s41418-017-0004-4
positive PromoterBinding: PPARG - 31993929 10.1007/s11010-020-03689-8
positive PromoterBinding: PPARG - 33352766 10.3390/ijms21249721
positive PromoterBinding: PPARG - 19168033 10.1016/j.bbrc.2009.01.06010.1016/j.bbrc.2009
positive PromoterBinding: PPARG - 26942868 10.1080/01635581.2016.1142581
positive PromoterBinding: PPARG - 25700221 10.1042/CS20140631
positive PromoterBinding: PPARG - 25881671 10.1042/CS20130463
positive PromoterBinding: PPARG - 25881671 10.1042/CS20130463
positive PromoterBinding: PPARG - 25881671 10.1042/CS20130463
positive PromoterBinding: PPARG --+> TP53 10.1007/s11010-020-03689-8
positive PromoterBinding: PPARG --+> TP53 10.1042/CS20140631
positive PromoterBinding: PPARG --+> TP53 10.1074/jbc.M113.453886
positive PromoterBinding: PPARG - 21445621 10.1007/s00394-011-0187-2
positive PromoterBinding: PPARG - 21445621 10.1007/s00394-011-0187-2
positive PromoterBinding: PPARG - 16887883 10.1210/me.2006-0192

positive PromoterBinding: PPARG -	27077805	10.1038/cddis.2016.92
positive PromoterBinding: PPARG -	27077805	10.1038/cddis.2016.92
positive PromoterBinding: PPARG -	27077805	10.1038/cddis.2016.92
positive PromoterBinding: PPARG -	27077805	10.1038/cddis.2016.92
positive PromoterBinding: PPARG -	27077805	10.1038/cddis.2016.92
positive PromoterBinding: PPARG -	27077805	10.1038/cddis.2016.92
positive PromoterBinding: PPARG --+> TP53		10.3390/ijms222111360
positive PromoterBinding: PPARG -	35471732	10.1096/fj.202200251R
positive PromoterBinding: TP53 --	31852624	10.1016/j.neuint.2019.104644
positive PromoterBinding: TP53 --	31852624	10.1016/j.neuint.2019.104644
positive PromoterBinding: TP53 --	29073938	10.1186/s13046-017-0617-y10.1186/s13046-017-0
positive PromoterBinding: TP53 --	23220650	10.1016/j.neulet.2012.11.04710.1016/j.neulet.
positive PromoterBinding: TP53 --	20308559	10.1073/pnas.091108210710.1073/pnas.0911082100
positive PromoterBinding: TP53 --	20661255	10.1038/nrg2843
positive PromoterBinding: TP53 --	25197016	10.1186/1471-2407-14-656
positive PromoterBinding: TP53 --	24219989	10.1038/emboj.2013.242
positive PromoterBinding: TP53 --	24219989	10.1038/emboj.2013.242
positive PromoterBinding: TP53 --	23343952	10.1097/PAI.0b013e3182773125
positive PromoterBinding: TP53 --	34666780	10.1186/s12986-021-00615-7
positive PromoterBinding: TP53 --	35410210	10.1186/s12935-022-02557-3
positive PromoterBinding: TP53 --	36612285	10.3390/cancers15010288
positive PromoterBinding: TP53 --	36612285	10.3390/cancers15010288
PromoterBinding: TP53 ---> PPARG	21854837	10.1016/j.brainresbull.2011.07.021
PromoterBinding: TP53 ---> PPARG	23412343	10.1038/cdd.2013.9
PromoterBinding: TP53 ---> PPARG	21510869	10.1186/1471-2407-11-149
PromoterBinding: TP53 ---> PPARG	21445621	10.1007/s00394-011-0187-2
PromoterBinding: TP53 ---> PPARG	30181511	10.3390/ijms19092622
PromoterBinding: TP53 ---> PPARG	30143607	10.1038/s41467-018-05711-6
PromoterBinding: TP53 ---> PPARG	29593329	10.1038/s41556-018-0072-9
PromoterBinding: TP53 ---> PPARG	29593329	10.1038/s41556-018-0072-9
PromoterBinding: TP53 ---> PPARG	29593329	10.1038/s41556-018-0072-9
PromoterBinding: TP53 ---> PPARG	29593329	10.1038/s41556-018-0072-9
PromoterBinding: TP53 ---> PPARG		10.1016/j.brainresbull.2011.07.021
PromoterBinding: TP53 ---> PPARG	29311623	10.1038/s41418-017-0004-4
PromoterBinding: TP53 ---> PPARG	17651710	
PromoterBinding: TP53 ---> PPARG	24675459	10.1038/cddis.2014.87
PromoterBinding: TP53 ---> PPARG	24312426	10.1371/journal.pone.0082494
PromoterBinding: TP53 ---> PPARG	23977392	10.1371/journal.pone.0073709
PromoterBinding: TP53 ---> PPARG	22074662	10.1016/j.biocel.2011.10.023
PromoterBinding: TP53 ---> PPARG	21421225	10.1016/j.juro.2010.12.101
PromoterBinding: TP53 ---> PPARG	19168033	10.1016/j.bbrc.2009.01.060
PromoterBinding: TP53 ---> PPARG		
PromoterBinding: TP53 ---> PPARG	28155209	10.1007/s11999-017-5259-z
PromoterBinding: TP53 ---> PPARG		10.2741/4475
positive QuantitativeChange: hypo	28327194	10.1186/s12943-017-0635-6
Regulation: UCA1 ---> hypopharyng	28327194	10.1186/s12943-017-0635-6
Regulation: UCA1 ---> hypopharyng	28327194	10.1186/s12943-017-0635-6
Regulation: UCA1 ---> hypopharyng	29368602	10.1186/s12943-018-0763-7
Regulation: UCA1 ---> hypopharyng	28327194	10.1186/s12943-017-0635-610.1186/s12943-017-0
Regulation: UCA1 ---> hypopharyng	28327194	10.1186/s12943-017-0635-610.1186/s12943-017-0
Regulation: UCA1 ---> hypopharyng	35836822	10.1155/2022/1562219
Regulation: LRP8 ---> TP53	24840660	10.1016/j.bbadis.2014.05.009
Regulation: LRP8 ---> TP53		
ProtModification: AKT1 ---> BECN1	29921586	10.1074/jbc.RA118.002934

ProtModification: AKT1 ---> BECN124257753 10.1074/jbc.M113.532978
ProtModification: AKT1 ---> BECN124257753 10.1074/jbc.M113.532978
ProtModification: AKT1 ---> BECN124257753 10.1074/jbc.M113.532978
ProtModification: AKT1 ---> BECN1 10.1074/jbc.P113.532978
ProtModification: AKT1 ---> BECN127994061 10.1074/jbc.M116.758268
ProtModification: AKT1 ---> BECN127994061 10.1074/jbc.M116.758268
ProtModification: AKT1 ---> BECN127994061 10.1074/jbc.M116.758268
ProtModification: AKT1 ---> BECN132428566 10.1016/j.biochi.2020.04.025
ProtModification: AKT1 ---> BECN130578829 10.1016/j.cellsig.2018.12.008
ProtModification: AKT1 ---> BECN131260798 10.1016/j.cellsig.2019.109353
ProtModification: AKT1 ---> BECN133838253 10.1016/j.matbio.2021.04.001
ProtModification: AKT1 ---> BECN133540047 10.1016/j.phrs.2021.105428
ProtModification: AKT1 ---> BECN131430557 10.1016/j.semcancer.2019.08.020
ProtModification: AKT1 ---> BECN131972372 10.1016/j.mito.2020.01.002
ProtModification: AKT1 ---> BECN132200042 10.1016/j.gpb.2020.01.001
ProtModification: AKT1 ---> BECN133717651 10.1016/j.omtn.2021.02.005
ProtModification: AKT1 ---> BECN123232185 10.1016/j.bbcan.2012.12.001
ProtModification: AKT1 ---> BECN124269817 10.1016/j.bbrc.2013.11.048
ProtModification: AKT1 ---> BECN123827971 10.1016/j.pneurobio.2013.06.002
ProtModification: AKT1 ---> BECN123811268 10.1016/j.semcancer.2013.06.007
ProtModification: AKT1 ---> BECN124630600 10.1016/j.tibs.2014.02.003
ProtModification: AKT1 ---> BECN124976620 10.1016/j.ajpath.2014.05.010
ProtModification: AKT1 ---> BECN125172632 10.1016/j.jnutbio.2014.06.006
ProtModification: AKT1 ---> BECN127191591 10.1371/journal.ppat.1005652
ProtModification: AKT1 ---> BECN126018563 10.1080/15548627.2015.1043076
ProtModification: AKT1 ---> BECN127335173 10.1152/ajpcell.00129.2016
ProtModification: AKT1 ---> BECN127335173 10.1152/ajpcell.00129.2016
ProtModification: AKT1 ---> BECN127335173 10.1152/ajpcell.00129.2016
ProtModification: AKT1 ---> BECN128428363 10.1530/JOE-17-0081
ProtModification: AKT1 ---> BECN129218076
ProtModification: AKT1 ---> BECN129218076
ProtModification: AKT1 ---> BECN129313410 10.1080/15548627.2017.1422851
ProtModification: AKT1 ---> BECN129921586 10.1074/jbc.RA118.002934
ProtModification: AKT1 ---> BECN129872222 10.1038/s41388-018-0330-0
ProtModification: AKT1 ---> BECN130546075 10.1038/s41418-018-0254-9
ProtModification: AKT1 ---> BECN130204590 10.1515/revneuro-2018-0045
ProtModification: AKT1 ---> BECN131175269 10.12659/MSM.915730
ProtModification: AKT1 ---> BECN131843932 10.1073/pnas.1915139116
ProtModification: AKT1 ---> BECN131843932 10.1073/pnas.1915139116
ProtModification: AKT1 ---> BECN131776982 10.1007/978-981-15-0602-4_5
ProtModification: AKT1 ---> BECN131776982 10.1007/978-981-15-0602-4_5
ProtModification: AKT1 ---> BECN131776982 10.1007/978-981-15-0602-4_5
ProtModification: AKT1 ---> BECN131776982 10.1007/978-981-15-0602-4_5
ProtModification: AKT1 ---> BECN131852899 10.1038/s41467-019-13659-4
ProtModification: AKT1 ---> BECN132121251 10.3390/ijms21051643
ProtModification: AKT1 ---> BECN132187724 10.15252/embj.2019103111
ProtModification: AKT1 ---> BECN1 10.3324/HAEMATOL.2018.212027
ProtModification: AKT1 ---> BECN132715758 10.1152/ajprenal.00594.2019
ProtModification: AKT1 ---> BECN133495338 10.1073/pnas.2020478118
ProtModification: AKT1 ---> BECN123817237 10.1038/ncb2797
ProtModification: AKT1 ---> BECN124006406 10.3324/haematol.2012.079061
ProtModification: AKT1 ---> BECN123980547 10.1165/rcmb.2013-0314TR
ProtModification: AKT1 ---> BECN123980547 10.1165/rcmb.2013-0314TR
ProtModification: AKT1 ---> BECN124257753 10.1074/jbc.M113.532978

ProtModification: AKT1 ---> BECN124257753 10.1074/jbc.M113.532978
ProtModification: AKT1 ---> BECN124257753 10.1074/jbc.M113.532978
ProtModification: AKT1 ---> BECN124257753 10.1074/jbc.M113.532978
ProtModification: AKT1 ---> BECN123708729 10.1007/s10059-013-0140-8
ProtModification: AKT1 ---> BECN123708729 10.1007/s10059-013-0140-8
ProtModification: AKT1 ---> BECN124196588 10.1096/fj.13-235655
ProtModification: AKT1 ---> BECN125412422 10.1371/journal.ppat.1004409
ProtModification: AKT1 ---> BECN125412422 10.1371/journal.ppat.1004409
ProtModification: AKT1 ---> BECN125633274 10.1210/me.2014-1367
ProtModification: AKT1 ---> BECN125633274 10.1210/me.2014-1367
ProtModification: AKT1 ---> BECN124714122 10.1159/000358670
ProtModification: AKT1 ---> BECN124257753 10.1074/jbc.M113.53297810.1074/jbc.M113.53297
ProtModification: AKT1 ---> BECN1 10.1016/j.semcancer.2019.08.020
ProtModification: AKT1 ---> BECN1 10.1530/JOE-17-0081
ProtModification: AKT1 ---> BECN1 10.1074/jbc.RA118.002934
ProtModification: AKT1 ---> BECN1 10.12659/MSM.915730
ProtModification: AKT1 ---> BECN1 10.1073/pnas.1915139116
ProtModification: AKT1 ---> BECN1 10.1073/pnas.1915139116
ProtModification: AKT1 ---> BECN1 10.1007/978-981-15-0602-4_5
ProtModification: AKT1 ---> BECN1 10.1007/978-981-15-0602-4_5
ProtModification: AKT1 ---> BECN1 10.1007/978-981-15-0602-4_5
ProtModification: AKT1 ---> BECN1 10.1007/978-981-15-0602-4_5
ProtModification: AKT1 ---> BECN1 10.1038/s41467-019-13659-4
ProtModification: AKT1 ---> BECN134655756 10.1016/j.jnutbio.2021.108882
ProtModification: AKT1 ---> BECN134655756 10.1016/j.jnutbio.2021.108882
ProtModification: AKT1 ---> BECN134655756 10.1016/j.jnutbio.2021.108882
ProtModification: AKT1 ---> BECN134655756 10.1016/j.jnutbio.2021.108882
ProtModification: AKT1 ---> BECN134655756 10.1016/j.jnutbio.2021.108882
ProtModification: AKT1 ---> BECN134382915 10.1080/15476286.2021.1960689
ProtModification: AKT1 ---> BECN135749708 10.1089/cell.2022.0025
ProtModification: AKT1 ---> BECN136142738 10.3390/ijms231810811
ProtModification: AKT1 ---> BECN136243840 10.1007/978-3-031-06566-8_3
Regulation: SPP1 ---> chemosensit28433634 10.1016/j.bbrc.2017.04.100
Regulation: SPP1 ---> chemosensit30535434 10.3892/ijo.2018.4656
Regulation: SPP1 ---> chemosensit21539449 10.1089/cbr.2010.0838
Regulation: SPP1 ---> chemosensit30627777 10.1007/s00280-018-3759-510.1007/s00280-018-3
Biomarker: hypopharyngeal squamou28327194 10.1186/s12943-017-0635-6
Expression: AKT1 ---> BECN1 27756618 10.1016/j.bbrc.2016.10.043
Expression: AKT1 ---> BECN1 27756618 10.1016/j.bbrc.2016.10.043
Expression: AKT1 ---> BECN1 32750329 10.1016/j.bcp.2020.114188
Expression: AKT1 ---> BECN1 30684759 10.1016/j.jinorgbio.2019.01.010
Expression: AKT1 ---> BECN1 29331396 10.1016/j.pneurobio.2018.01.001
Expression: AKT1 ---> BECN1 31128157 10.1016/j.neulet.2019.134287
Expression: AKT1 ---> BECN1 28188923 10.1016/j.freeradbiomed.2017.02.015
Expression: AKT1 ---> BECN1 22902321 10.1016/j.yjmcc.2012.08.001
Expression: AKT1 ---> BECN1 26306675 10.1016/j.ejphar.2015.08.039
Expression: AKT1 ---> BECN1 26593748 10.1016/j.freeradbiomed.2015.11.022
Expression: AKT1 ---> BECN1 26593748 10.1016/j.freeradbiomed.2015.11.022
Expression: AKT1 ---> BECN1 28151484 10.1038/cddis.2017.7
Expression: AKT1 ---> BECN1 29909423 10.12659/MSM.907853
Expression: AKT1 ---> BECN1 29498006 10.1007/s12035-018-0950-y
Expression: AKT1 ---> BECN1 30183517 10.1080/15384047.2018.1507665
Expression: AKT1 ---> BECN1 31718395 10.1089/jmf.2019.4411
Expression: AKT1 ---> BECN1 31799599 10.1042/BSR20191041

Expression: AKT1 ---> BECN1	33920748	10.3390/ijms22084067
Expression: AKT1 ---> BECN1	22207710	10.1161/CIRCRESAHA.111.255950
Expression: AKT1 ---> BECN1	23519142	10.1097/FJC.0b013e318291c094
Expression: AKT1 ---> BECN1	24678780	10.3171/2014.1.JNS131534
Expression: AKT1 ---> BECN1	16697404	10.1016/j.yjmcc.2006.03.428
Expression: AKT1 ---> BECN1	29909423	10.12659/MSM.90785310.12659/MSM.907853
Expression: AKT1 ---> BECN1	30632514	10.4103/1673-5374.24747610.4103/1673-5374.247
Expression: AKT1 ---> BECN1		10.1089/jmf.2019.4411
Expression: AKT1 ---> BECN1		10.1042/BSR20191041
Expression: AKT1 ---> BECN1		10.3171/2014.1.JNS131534
Expression: AKT1 ---> BECN1	34759291	10.1038/s41419-021-04371-7
Expression: AKT1 ---> BECN1	33086879	10.1080/01635581.2020.1836241
Expression: AKT1 ---> BECN1	35109896	10.1186/s13041-022-00899-7
Expression: AKT1 ---> BECN1	34949740	10.14348/molcells.2021.0183
Expression: AKT1 ---> BECN1	35878674	10.1016/j.yexmp.2022.104814
Expression: AKT1 ---> BECN1	35851059	10.1038/s41419-022-05085-0
Expression: MAPK1 ---> PTEN	19000654	10.1016/j.bbrc.2008.10.157
Expression: MAPK1 ---> PTEN	19000654	10.1016/j.bbrc.2008.10.157
Expression: MAPK1 ---> PTEN	12808053	
Expression: MAPK1 ---> PTEN	12808053	
Expression: MAPK1 ---> PTEN	19002158	
Expression: MAPK1 ---> PTEN	26686085	10.1038/onc.2015.470
Expression: MAPK1 ---> PTEN	26686085	10.1038/onc.2015.470
Expression: MAPK1 ---> PTEN	26686085	10.1038/onc.2015.470
Expression: MAPK1 ---> PTEN	30557170	10.1097/MPH.0000000000001386
Expression: MAPK1 ---> PTEN	31270502	10.1038/s41591-019-0499-y
Expression: MAPK1 ---> PTEN	24025251	10.4161/cbt.26234
Expression: MAPK1 ---> PTEN	24866016	10.1371/journal.pone.0098113
Expression: MAPK1 ---> PTEN	24866016	10.1371/journal.pone.0098113
Expression: MAPK1 ---> PTEN	25862896	
Expression: MAPK1 ---> PTEN	17638924	10.1093/carcin/bgm159
Expression: MAPK1 ---> PTEN	21111719	10.1016/j.brainres.2010.11.06210.1016/j.brain
Expression: MAPK1 ---> PTEN	30735788	10.1016/j.nlm.2019.02.00510.1016/j.nlm.2019.0
Expression: MAPK1 ---> PTEN		10.1097/MPH.0000000000001386
Expression: MAPK1 ---> PTEN		10.1038/s41591-019-0499-y
Expression: MAPK1 ---> PTEN	19118244	
Expression: MAPK1 ---> PTEN	19002158	
Expression: MAPK1 ---> PTEN	19940030	
Expression: MAPK1 ---> PTEN	24866016	10.1371/journal.pone.0098113
Expression: MAPK1 ---> PTEN	25512490	10.1073/pnas.1413186112
Expression: MAPK1 ---> PTEN	17638924	
Expression: MAPK1 ---> PTEN	25512490	10.1073/pnas.1413186112
Expression: MAPK1 ---> PTEN		
negative Expression: PTEN ---| M/	19038262	10.1016/j.yjmcc.2008.10.021
negative Expression: PTEN ---| M/	21194879	10.1016/j.ejso.2010.12.003
negative Expression: PTEN ---| M/	27107786	10.1016/j.toxlet.2016.04.015
negative Expression: PTEN ---| M/	27107786	10.1016/j.toxlet.2016.04.015
negative Expression: PTEN ---| M/	28000852	10.3892/ijo.2016.3811
negative Expression: PTEN ---| M/	29941984	10.1038/s41434-018-0020-0
negative Expression: PTEN ---| M/	22367504	10.1152/ajpheart.00929.2011
negative Expression: PTEN ---| M/	21544242	10.1371/journal.pone.001913910.1371/journal.p
negative Expression: PTEN ---| M/	27107786	10.1016/j.toxlet.2016.04.01510.1016/j.toxlet.
negative Expression: PTEN ---| M/	27388240	10.5966/sctm.2015-0200
negative Expression: PTEN ---| M/	27388240	10.5966/sctm.2015-0200

negative Expression: PTEN ---| MA	34504554	10.3892/etm.2021.1053410.3892/etm.2021.10534
positive Regulation: PRND --+> TF	24339999	10.1371/journal.pone.0082130
positive Regulation: PRND --+> TF	24339999	10.1371/journal.pone.0082130
positive Regulation: PRND --+> TF	24339999	10.1371/journal.pone.0082130
positive Regulation: PRND --+> TF	24339999	10.1371/journal.pone.0082130
positive Regulation: PRND --+> TF	24339999	10.1371/journal.pone.0082130
positive Regulation: PRND --+> TF	24339999	10.1371/journal.pone.0082130
positive Expression: TP53 --+> MI	30130662	10.1016/j.semcancer.2018.08.008
positive Expression: TP53 --+> MI	32343915	10.1089/dna.2020.5457
positive Expression: TP53 --+> MI	30130662	10.1016/j.semcancer.2018.08.008
positive Expression: TP53 --+> MI	20729113	10.1016/j.ymgme.2010.07.016
positive Expression: TP53 --+> MI	21616186	10.1016/j.bbagrm.2011.05.005
positive Expression: TP53 --+> MI	22579961	10.1016/j.bbcan.2012.04.008
positive Expression: TP53 --+> MI	23220650	10.1016/j.neulet.2012.11.047
positive Expression: TP53 --+> MI	23220650	10.1016/j.neulet.2012.11.047
positive Expression: TP53 --+> MI	23220650	10.1016/j.neulet.2012.11.047
positive Expression: TP53 --+> MI	24739178	10.1016/j.bpj.2014.02.032
positive Expression: TP53 --+> MIR107		10.1016/j.canlet.2014.10.011
positive Expression: TP53 --+> MI	24942805	10.1016/j.bbagrm.2014.05.027
positive Expression: TP53 --+> MI	25451319	10.1016/j.canlet.2014.10.011
positive Expression: TP53 --+> MI	25758424	10.1016/j.urolonc.2015.02.003
positive Expression: TP53 --+> MI	20308559	
positive Expression: TP53 --+> MI	20308559	
positive Expression: TP53 --+> MI	29298665	10.1186/s12885-017-3941-x
positive Expression: TP53 --+> MI	29383635	10.1007/s10456-018-9600-2
positive Expression: TP53 --+> MIR107		10.2174/1381612825666190110161843
positive Expression: TP53 --+> MI	21909123	10.1038/aps.2011.103
positive Expression: TP53 --+> MI	24013378	10.3390/ijms140918319
positive Expression: TP53 --+> MI	23343952	10.1097/PAI.0b013e3182773125
positive Expression: TP53 --+> MI	24219989	10.1038/emboj.2013.242
positive Expression: TP53 --+> MI	24219989	10.1038/emboj.2013.242
positive Expression: TP53 --+> MI	25197016	10.1186/1471-2407-14-656
positive Expression: TP53 --+> MI	20661255	10.1038/nrg2843
positive Expression: TP53 --+> MI	20308559	10.1073/pnas.091108210710.1073/pnas.091108210
positive Expression: TP53 --+> MI	23220650	10.1016/j.neulet.2012.11.04710.1016/j.neulet.
positive Expression: TP53 --+> MI	29073938	10.1186/s13046-017-0617-y10.1186/s13046-017-0
positive Expression: TP53 --+> MI	23154181	10.1016/j.bbrc.2012.11.005
positive Expression: TP53 --+> MI	22841759	10.1016/j.freeradbiomed.2012.07.026
positive Expression: TP53 --+> MI	23074224	10.1074/jbc.M112.392076
positive Expression: TP53 --+> MIR107		
positive Expression: TP53 --+> MI	33199193	10.1016/j.trecan.2020.10.005
positive Expression: TP53 --+> MI	33199193	10.1016/j.trecan.2020.10.005
positive Expression: TP53 --+> MI	34666780	10.1186/s12986-021-00615-7
Regulation: DST ---> AKT1	21376539	10.1016/j.jdermsci.2011.01.005
Expression: BECN1 ---> MCL1	27789215	10.1016/j.bcp.2016.10.006
Expression: BECN1 ---> MCL1	31682805	10.1016/j.cbi.2019.108888
Expression: BECN1 ---> MCL1	33545382	10.1016/j.drudis.2021.01.027
Expression: BECN1 ---> MCL1	25472497	10.1038/ncomms6637
Expression: BECN1 ---> MCL1	25472497	10.1038/ncomms6637
Expression: BECN1 ---> MCL1	25472497	10.1038/ncomms6637
Expression: BECN1 ---> MCL1	25837021	10.1080/15548627.2015.1029836
Expression: BECN1 ---> MCL1	25837021	10.1080/15548627.2015.102983610.1080/15548627
Expression: BECN1 ---> MCL1	25472497	10.1038/ncomms663710.1038/ncomms6637
Expression: BECN1 ---> MCL1	25472497	10.1038/ncomms663710.1038/ncomms6637

Regulation: GPER1 ---> PTGS2 28347854 10.1016/j.jsbmb.2017.03.021
Regulation: GPER1 ---> PTGS2 26241029 10.1016/j.jsbmb.2015.07.019
Regulation: GPER1 ---> PTGS2 26241029 10.1016/j.jsbmb.2015.07.019
Regulation: GPER1 ---> PTGS2 10.1016/j.bbrc.2019.05.045
Regulation: GCLC ---> ABCC1 7488097 10.1006/bbrc.1995.2618
Regulation: GCLC ---> ABCC1 7488097 10.1006/bbrc.1995.2618
Regulation: GCLC ---> ABCC1 9063478
DirectRegulation: MCL1 ---> BECN124113155 10.4161/auto.2645210.4161/auto.26452
DirectRegulation: MCL1 ---> BECN123392173 10.1038/cddis.2013.1810.1038/cddis.2013.18
DirectRegulation: MCL1 ---> BECN1
DirectRegulation: MCL1 ---> BECN125484085 10.4161/15548627.2014.981916
DirectRegulation: MCL1 ---> BECN124535641 10.3892/or.2014.3015
DirectRegulation: MCL1 ---> BECN124113155 10.4161/auto.26452
DirectRegulation: MCL1 ---> BECN123681223 10.1038/cddis.2013.155
DirectRegulation: MCL1 ---> BECN123392173 10.1038/cddis.2013.18
DirectRegulation: MCL1 ---> BECN123392173 10.1038/cddis.2013.18
DirectRegulation: MCL1 ---> BECN123392177 10.1038/cddis.2013.17
DirectRegulation: MCL1 ---> BECN123688351 10.1186/1471-2202-14-56
DirectRegulation: MCL1 ---> BECN133931087 10.1186/s13046-021-01948-0
DirectRegulation: MCL1 ---> BECN131485222 10.1155/2019/2024648
DirectRegulation: MCL1 ---> BECN130842276 10.1073/pnas.1819869116
DirectRegulation: MCL1 ---> BECN130792387 10.1038/s41419-019-1407-6
DirectRegulation: MCL1 ---> BECN130066884 10.3892/or.2018.6599
DirectRegulation: MCL1 ---> BECN129976630 10.21873/cgp.20083
DirectRegulation: MCL1 ---> BECN127837217 10.1007/s00018-016-2403-y
DirectRegulation: MCL1 ---> BECN120200450
DirectRegulation: MCL1 ---> BECN123727157 10.1016/j.semcancer.2013.05.007
DirectRegulation: MCL1 ---> BECN123420005 10.1016/j.biocel.2013.02.007
DirectRegulation: MCL1 ---> BECN132307222 10.1016/j.tcb.2020.03.004
DirectRegulation: MCL1 ---> BECN131030977 10.1016/j.tcb.2019.03.004
DirectRegulation: MCL1 ---> BECN128259820 10.1016/j.canlet.2017.02.030
DirectRegulation: MCL1 ---> BECN117643073 10.4161/auto.4713
DirectRegulation: MCL1 ---> BECN125837021 10.1080/15548627.2015.1029836
DirectRegulation: MCL1 ---> BECN126018731 10.1080/15548627.2015.1049800
DirectRegulation: MCL1 ---> BECN125885284 10.1186/s12885-015-1239-4
DirectRegulation: MCL1 ---> BECN124912196 10.1073/pnas.1406425111
DirectRegulation: MCL1 ---> BECN124113155 10.4161/auto.26452
DirectRegulation: MCL1 ---> BECN124107625 10.4161/cc.26696
DirectRegulation: MCL1 ---> BECN123607597 10.2174/156800961131399900039
DirectRegulation: MCL1 ---> BECN123681223 10.1038/cddis.2013.155
DirectRegulation: MCL1 ---> BECN121364679 10.1038/cddis.2010.53
DirectRegulation: MCL1 ---> BECN121139567 10.1038/emboj.2010.327
DirectRegulation: MCL1 ---> BECN121139567 10.1038/emboj.2010.327
DirectRegulation: MCL1 ---> BECN121139567 10.1038/emboj.2010.327
DirectRegulation: MCL1 ---> BECN121139567 10.1038/emboj.2010.327
DirectRegulation: MCL1 ---> BECN121139567 10.1038/emboj.2010.327
DirectRegulation: MCL1 ---> BECN122614243 10.1038/leu.2012.136
DirectRegulation: MCL1 ---> BECN123688351 10.1186/1471-2202-14-56
DirectRegulation: MCL1 ---> BECN123688351 10.1186/1471-2202-14-56
DirectRegulation: MCL1 ---> BECN122638980 10.1007/s10495-012-0738-x
DirectRegulation: MCL1 ---> BECN123152582 10.1194/jlr.R031278
DirectRegulation: MCL1 ---> BECN122263800
DirectRegulation: MCL1 ---> BECN134069373 10.3390/ijms22105330
DirectRegulation: MCL1 ---> BECN129611474 10.2174/0929867325666180403145137

DirectRegulation: MCL1 ---> BECN129808796 10.14715/cmb/2018.64.6.5
DirectRegulation: MCL1 ---> BECN130327467 10.1038/s41467-018-06836-4
DirectRegulation: MCL1 ---> BECN128622296 10.1038/cdd.2017.82
DirectRegulation: MCL1 ---> BECN129363570 10.1530/REP-18-0002
DirectRegulation: MCL1 ---> BECN129333953 10.1080/15384047.2018.1423927
DirectRegulation: MCL1 ---> BECN128234359 10.1038/cdd.2017.22
DirectRegulation: MCL1 ---> BECN127527160 10.3390/ijms17081274
DirectRegulation: MCL1 ---> BECN119912650
DirectRegulation: MCL1 ---> BECN120200450
DirectRegulation: MCL1 ---> BECN125194337 10.1016/j.biocel.2014.08.022
DirectRegulation: MCL1 ---> BECN125194337 10.1016/j.biocel.2014.08.022
DirectRegulation: MCL1 ---> BECN124012660 10.1016/j.semcancer.2013.08.008
DirectRegulation: MCL1 ---> BECN122735572 10.1016/j.mito.2012.06.003
DirectRegulation: MCL1 ---> BECN121353614 10.1016/j.molcel.2011.02.009
DirectRegulation: MCL1 ---> BECN121353614 10.1016/j.molcel.2011.02.009
DirectRegulation: MCL1 ---> BECN120159550 10.1016/j.molcel.2010.01.025
DirectRegulation: MCL1 ---> BECN122305615 10.1016/j.jdermsci.2011.11.001
DirectRegulation: MCL1 ---> BECN121993018 10.1016/j.canlet.2011.09.020
DirectRegulation: MCL1 ---> BECN118589438 10.1016/j.jmb.2008.05.071
DirectRegulation: MCL1 ---> BECN129555460 10.1016/j.actbio.2018.03.022
DirectRegulation: MCL1 ---> BECN133497829 10.1016/j.drudis.2021.01.021
DirectRegulation: MCL1 ---> BECN131030977 10.1016/j.tcb.2019.03.004
DirectRegulation: MCL1 ---> BECN129549710 10.1016/j.conb.2018.02.023
DirectRegulation: MCL1 ---> BECN133508399 10.1016/j.cellsig.2021.109933
DirectRegulation: MCL1 ---> BECN131639425 10.1016/j.canlet.2019.10.030
DirectRegulation: MCL1 ---> BECN131837377 10.1016/j.taap.2019.114857
DirectRegulation: MCL1 ---> BECN130562490 10.1016/j.lfs.2018.12.027
DirectRegulation: MCL1 ---> BECN126009469 10.1016/j.jmb.2015.05.015
DirectRegulation: MCL1 ---> BECN132717194 10.1016/j.yjmcc.2020.07.009
DirectRegulation: MCL1 ---> BECN133831396 10.1016/j.bcp.2021.114544
DirectRegulation: MCL1 ---> BECN133631669 10.1016/j.bbrc.2021.02.045
DirectRegulation: MCL1 ---> BECN134688664 10.1016/j.jbc.2021.101339
DirectRegulation: MCL1 ---> BECN134657993 10.1007/s13105-021-00844-7
DirectRegulation: MCL1 ---> BECN134077348 10.2174/1568009621666210601113144
DirectRegulation: MCL1 ---> BECN134856903 10.2174/1568009621666211202091523
DirectRegulation: MCL1 ---> BECN135904454 10.1042/BCJ20210676
Binding: PRPH ---- DST 17569669 10.1074/jbc.M611703200
Binding: PRPH ---- DST 17569669
Binding: PRPH ---- DST 9971739
Binding: PRPH ---- DST 9971739
Binding: PRPH ---- DST 9971739
Binding: PRPH ---- DST 9971739
positive Expression: BIRC7 --+> 132334197 10.1016/j.bioorg.2020.103863
positive Expression: BIRC7 --+> 125785018
positive Expression: B3GNT3 --+> 33683574 10.1007/s13258-021-01072-510.1007/s13258-021-
PromoterBinding: PPARG ---> PTGS2 14636669 10.1016/S0163-7827(03)00037-7
PromoterBinding: PPARG ---> PTGS2 17316818 10.1016/j.plipres.2007.01.001
PromoterBinding: PPARG ---> PTGS2 10.1016/j.jnutbio.2004.11.001
PromoterBinding: PPARG ---> PTGS2 16303334 10.1016/j.clim.2005.09.018
PromoterBinding: PPARG ---> PTGS2 18362203
PromoterBinding: PPARG ---> PTGS2 14645533
PromoterBinding: PPARG ---> PTGS2 20635307 10.1055/s-0030-1250111
PromoterBinding: PPARG ---> PTGS2 23365414 10.1095/biolreprod.112.107383
PromoterBinding: PPARG ---> PTGS2 24403546 10.1095/biolreprod.113.109223

PromoterBinding: PPARG ---> PTGS2	16443360	10.1016/j.jnutbio.2005.10.007
PromoterBinding: PPARG ---> PTGS2		
ProtModification: AKT1 ---> PHF2(	22334668	10.1074/jbc.M111.333922
ProtModification: AKT1 ---> PHF2(	22334668	10.1074/jbc.M111.333922
ProtModification: AKT1 ---> PHF2(	22334668	10.1074/jbc.M111.333922
ProtModification: AKT1 ---> PHF2(	22334668	10.1074/jbc.M111.333922
ProtModification: AKT1 ---> PHF2(	22975685	10.1016/j.cellsig.2012.09.009
ProtModification: AKT1 ---> PHF2(	22975685	10.1016/j.cellsig.2012.09.009
ProtModification: AKT1 ---> PHF2(	22975685	10.1016/j.cellsig.2012.09.009
ProtModification: AKT1 ---> PHF2(	22975685	10.1016/j.cellsig.2012.09.009
ProtModification: AKT1 ---> PHF2(	22975685	10.1016/j.cellsig.2012.09.009
ProtModification: AKT1 ---> PHF2(	22975685	10.1016/j.cellsig.2012.09.009
ProtModification: AKT1 ---> PHF2(	22334668	10.1074/jbc.M111.333922
ProtModification: AKT1 ---> PHF2(	22334668	10.1074/jbc.M111.333922
ProtModification: AKT1 ---> PHF2(	22334668	10.1074/jbc.M111.333922
ProtModification: AKT1 ---> PHF2(	22334668	10.1074/jbc.M111.333922
ProtModification: AKT1 ---> PHF2(	22334668	10.1074/jbc.M111.333922
ProtModification: AKT1 ---> PHF2(	22334668	10.1074/jbc.M111.333922
ProtModification: AKT1 ---> PHF2(	22334668	10.1074/jbc.M111.333922
ProtModification: AKT1 ---> PHF2(	22334668	10.1074/jbc.M111.333922
ProtModification: AKT1 ---> PHF2(	22334668	10.1074/jbc.M111.333922
ProtModification: AKT1 ---> PHF2(	22334668	10.1074/jbc.M111.333922
ProtModification: AKT1 ---> PHF2(	22864287	10.1038/nsmb.2353
ProtModification: AKT1 ---> PHF2(	22334668	10.1074/jbc.M111.333922
ProtModification: AKT1 ---> PHF2(	22334668	10.1074/jbc.M111.333922
ProtModification: AKT1 ---> PHF2(	22975685	10.1016/j.cellsig.2012.09.009
ProtModification: AKT1 ---> PHF2(	22975685	10.1016/j.cellsig.2012.09.009
ProtModification: AKT1 ---> PHF2(	22334668	10.1074/jbc.M111.333922
ProtModification: AKT1 ---> PHF2(	22334668	10.1074/jbc.M111.333922
ProtModification: AKT1 ---> PHF2(	22334668	10.1074/jbc.M111.333922
ProtModification: AKT1 ---> PHF2(	22975685	10.1016/j.cellsig.2012.09.009
Expression: NOTCH1 ---> MAPK1	32792479	10.1038/s41419-020-02873-4
Expression: NOTCH1 ---> MAPK1	32792479	10.1038/s41419-020-02873-4
Expression: NOTCH1 ---> MAPK1	34037092	10.1590/1414-431X2020E10685
Expression: NOTCH1 ---> MAPK1	34037092	10.1590/1414-431X2020E10685
Expression: NOTCH1 ---> MAPK1	23468978	10.1371/journal.pone.0057382
Expression: NOTCH1 ---> MAPK1	26398771	10.3892/or.2015.4274
Expression: NOTCH1 ---> MAPK1	26398771	10.3892/or.2015.427410.3892/or.2015.4274
Expression: NOTCH1 ---> MAPK1	34109563	10.1007/s12017-021-08671-110.1007/s12017-021-
Expression: NOTCH1 ---> MAPK1	32792479	10.1038/s41419-020-02873-410.1038/s41419-020-
Expression: NOTCH1 ---> MAPK1	24472723	10.1124/jpet.113.20756310.1124/jpet.113.20756
negative Regulation: PTEN ---| MA	16684527	10.1016/j.febslet.2006.04.064
negative Regulation: PTEN ---| MA	27509846	10.3892/ijo.2016.3655
negative Regulation: PTEN ---| MA	16684527	10.1016/j.febslet.2006.04.064
negative Regulation: PTEN ---| MA	19167986	10.1016/j.proghi.2008.07.001
negative Regulation: PTEN ---| MA	22182448	10.1016/j.canlet.2011.12.013
negative Regulation: PTEN ---| MA	20056128	10.1016/j.neulet.2009.12.078
negative Regulation: PTEN ---| MA	17336157	10.1016/j.it.2007.02.004
negative Regulation: PTEN ---| MA	22172720	10.1016/j.ccr.2011.10.030
negative Regulation: PTEN ---| MA	23219716	10.1016/j.ajpath.2012.10.007
negative Regulation: PTEN ---| MA	23206598	10.1016/B978-0-12-398309-1.00007-X
negative Regulation: PTEN ---| MAPK1		10.1016/j.joca.2013.02.280
negative Regulation: PTEN ---| MA	23684551	10.1016/j.canlet.2013.05.007
negative Regulation: PTEN ---| MA	25012567	10.1016/j.bmc.2014.06.046

negative Regulation: PTEN ---| MA 26764128 10.1016/j.ydbio.2015.12.027
negative Regulation: PTEN ---| MA 26460096 10.1016/j.ydbio.2015.10.002
negative Regulation: PTEN ---| MA 26437446 10.1016/j.tiv.2015.09.027
negative Regulation: PTEN ---| MA 27107786 10.1016/j.toxlet.2016.04.015
negative Regulation: PTEN ---| MA 19276401
negative Regulation: PTEN ---| MA 15824107
negative Regulation: PTEN ---| MA 15590688
negative Regulation: PTEN ---| MA 15590688
negative Regulation: PTEN ---| MA 15590688
negative Regulation: PTEN ---| MA 15121878
negative Regulation: PTEN ---| MA 14578361
negative Regulation: PTEN ---| MA 11689561
negative Regulation: PTEN ---| MA 11689561
negative Regulation: PTEN ---| MA 12235291
negative Regulation: PTEN ---| MA 20418918
negative Regulation: PTEN ---| MA 10698513
negative Regulation: PTEN ---| MA 20097771
negative Regulation: PTEN ---| MA 17277022
negative Regulation: PTEN ---| MA 11158326
negative Regulation: PTEN ---| MA 25398317 10.1186/s12964-014-0069-3
negative Regulation: PTEN ---| MA 25398317 10.1186/s12964-014-0069-3
negative Regulation: PTEN ---| MA 25398317 10.1186/s12964-014-0069-3
negative Regulation: PTEN ---| MA 25398317 10.1186/s12964-014-0069-3
negative Regulation: PTEN ---| MA 26135619 10.1038/ncomms8353
negative Regulation: PTEN ---| MA 27605619 10.1523/JNEUROSCI.0936-16.2016
negative Regulation: PTEN ---| MA 27605619 10.1523/JNEUROSCI.0936-16.2016
negative Regulation: PTEN ---| MA 22519449
negative Regulation: PTEN ---| MA 22875989 10.1091/mbc.E12-05-0337
negative Regulation: PTEN ---| MA 22746319 10.1089/ars.2011.4457
negative Regulation: PTEN ---| MA 23064324 10.3892/or.2012.2074
negative Regulation: PTEN ---| MA 20664988 10.3892/or-00000922
negative Regulation: PTEN ---| MA 20664988 10.3892/or-00000922
negative Regulation: PTEN ---| MA 20664988 10.3892/or-00000922
negative Regulation: PTEN ---| MA 21385768 10.1242/dev.058016
negative Regulation: PTEN ---| MA 22106098 10.1530/JOE-11-0296
negative Regulation: PTEN ---| MA 20738847 10.1186/1476-4598-9-224
negative Regulation: PTEN ---| MA 22761812 10.1371/journal.pone.0039520
negative Regulation: PTEN ---| MA 24413171 10.1242/jcs.143743
negative Regulation: PTEN ---| MA 23542173 10.1038/onc.2013.74
negative Regulation: PTEN ---| MA 24595168 10.1371/journal.pone.0090907
negative Regulation: PTEN ---| MA 24595168 10.1371/journal.pone.0090907
negative Regulation: PTEN ---| MA 24552815 10.4161/cc.27837
negative Regulation: PTEN ---| MA 24552815 10.4161/cc.27837
negative Regulation: PTEN ---| MA 24552815 10.4161/cc.27837
negative Regulation: PTEN ---| MA 24552815 10.4161/cc.27837
negative Regulation: PTEN ---| MA 24552815 10.4161/cc.27837
negative Regulation: PTEN ---| MA 24552815 10.4161/cc.27837
negative Regulation: PTEN ---| MA 24552815 10.4161/cc.27837
negative Regulation: PTEN ---| MA 24552815 10.4161/cc.27837
negative Regulation: PTEN ---| MA 24552815 10.4161/cc.27837
negative Regulation: PTEN ---| MA 24552815 10.4161/cc.27837
negative Regulation: PTEN ---| MA 26010537 10.1371/journal.pone.0127942
negative Regulation: PTEN ---| MA 26244501 10.1371/journal.pone.0133699
negative Regulation: PTEN ---| MA 26244501 10.1371/journal.pone.0133699

negative Regulation: PTEN ---| MA 26929372 10.1073/pnas.1523693113
negative Regulation: PTEN ---| MA 10657643
negative Regulation: PTEN ---| MA 11230180
negative Regulation: PTEN ---| MA 12479095
negative Regulation: PTEN ---| MA 12781042
negative Regulation: PTEN ---| MA 15590688
negative Regulation: PTEN ---| MA 19276401 10.1124/jpet.108.150367
negative Regulation: PTEN ---| MA 20147383 10.1242/dev.040535
negative Regulation: PTEN ---| MA 21076047 10.1158/1541-7786.MCR-10-0235
negative Regulation: PTEN ---| MA 22737980 10.3109/10799893.2012.695798
negative Regulation: PTEN ---| MA 22875989 10.1091/mbc.E12-05-0337
negative Regulation: PTEN ---| MA 24595168 10.1371/journal.pone.0090907
negative Regulation: PTEN ---| MA 24552815 10.4161/cc.27837
negative Regulation: PTEN ---| MA 24552815 10.4161/cc.27837
negative Regulation: PTEN ---| MA 24552815 10.4161/cc.27837
negative Regulation: PTEN ---| MA 24552815 10.4161/cc.27837
negative Regulation: PTEN ---| MA 25773930 10.1007/s10549-015-3337-z
negative Regulation: PTEN ---| MA 26135619 10.1038/ncomms8353
negative Regulation: PTEN ---| MA 26316702 10.2147/DDDT.S86184
negative Regulation: PTEN ---| MA 26046303
negative Regulation: PTEN ---| MA 16684527 10.1016/j.febslet.2006.04.064
negative Regulation: PTEN ---| MA 24630093 10.1016/j.biomaterials.2014.02.037
negative Regulation: PTEN ---| MA 12235113
negative Regulation: PTEN ---| MA 23060448 10.1074/jbc.M112.405852
negative Regulation: PTEN ---| MA 20884649 10.1189/jlb.0110001
negative Regulation: PTEN ---| MA 22298084 10.1007/s00395-012-0248-6
negative Regulation: PTEN ---| MA 18726116 10.1007/s00432-008-0465-4
negative Regulation: PTEN ---| MAPK1
negative Regulation: PTEN ---| MA 26686085 10.1038/onc.2015.470
negative Regulation: PTEN ---| MA 27605619 10.1523/JNEUROSCI.0936-16.201610.1523/JNEUROS
negative Regulation: PTEN ---| MA 28367561 10.1039/c6mb00786d
negative Regulation: PTEN ---| MA 29048612 10.3892/ijo.2017.4126
negative Regulation: PTEN ---| MAPK1 10.3892/mmr.2017.8092
negative Regulation: PTEN ---| MA 29788729 10.4149/neo_2018_170208N89
negative Regulation: PTEN ---| MA 29426973 10.1007/s11626-018-0232-z
negative Regulation: PTEN ---| MA 29743588 10.1038/s41388-018-0251-y
negative Regulation: PTEN ---| MA 29537337 10.1152/physrev.00025.2017
Expression: TP53 ---> IGFBP4 21829624 10.1371/journal.pone.0022456
negative PromoterBinding: TP53 -- 18234542 10.1016/j.oraloncology.2007.10.006
negative PromoterBinding: TP53 -- 10196169
negative PromoterBinding: TP53 -- 12411481
negative PromoterBinding: TP53 -- 12411481
negative PromoterBinding: TP53 -- 12411481
negative PromoterBinding: TP53 -- 12411481
negative PromoterBinding: TP53 -- 11085935
negative PromoterBinding: TP53 -- 11085935
negative PromoterBinding: TP53 -- 18254728
negative PromoterBinding: TP53 -- 10196169
negative PromoterBinding: TP53 -- 19247455
negative PromoterBinding: TP53 -- 19247455
negative PromoterBinding: TP53 -- 23384026 10.1186/1471-2407-13-62
negative PromoterBinding: TP53 -- 27367679 10.3390/ijms17071026
negative PromoterBinding: TP53 -- 12411481
negative Expression: MIR107 ---| 31778666 10.1016/j.cbi.2019.108908

negative Expression: MIR107 ---| 31778666 10.1016/j.cbi.2019.108908
Expression: ING4 ---> NGF 24057236 10.1007/s11033-013-2675-3
ProtModification: MAPK1 ---> MCL118948586 10.1182/blood-2008-07-170241
ProtModification: MAPK1 ---> MCL112223490 10.1074/jbc.M207951200
ProtModification: MAPK1 ---> MCL130782845 10.1074/jbc.RA118.006029
ProtModification: MAPK1 ---> MCL130782845 10.1074/jbc.RA118.006029
ProtModification: MAPK1 ---> MCL129232553 10.1016/j.ccell.2017.11.003
ProtModification: MAPK1 ---> MCL129232547 10.1016/j.ccell.2017.11.010
ProtModification: MAPK1 ---> MCL129805077 10.1016/j.ccell.2018.05.001
ProtModification: MAPK1 ---> MCL134182005 10.1016/j.pharmthera.2021.107943
ProtModification: MAPK1 ---> MCL120079433 10.1016/j.bbadis.2009.12.009
ProtModification: MAPK1 ---> MCL120969960 10.1016/j.semcancer.2010.10.011
ProtModification: MAPK1 ---> MCL116710358
ProtModification: MAPK1 ---> MCL117387146
ProtModification: MAPK1 ---> MCL117670986
ProtModification: MAPK1 ---> MCL117670986
ProtModification: MAPK1 ---> MCL117670986
ProtModification: MAPK1 ---> MCL129311244 10.1128/IAI.00504-17
ProtModification: MAPK1 ---> MCL129725697 10.1007/s00018-018-2826-8
ProtModification: MAPK1 ---> MCL129141533 10.2174/1381612823666171115105252
ProtModification: MAPK1 ---> MCL130782845 10.1074/jbc.RA118.006029
ProtModification: MAPK1 ---> MCL130782845 10.1074/jbc.RA118.006029
ProtModification: MAPK1 ---> MCL130819918 10.3324/haematol.2018.201343
ProtModification: MAPK1 ---> MCL131475898 10.2174/1568026619666190902151307
ProtModification: MAPK1 ---> MCL132576977 10.1038/s41580-020-0255-7
ProtModification: MAPK1 ---> MCL123171055 10.1186/1471-2407-12-541
ProtModification: MAPK1 ---> MCL123023732 10.1007/s40291-012-0003-6
ProtModification: MAPK1 ---> MCL121507240 10.1186/1476-4598-10-44
ProtModification: MAPK1 ---> MCL122751450 10.1038/leu.2012.180
ProtModification: MAPK1 ---> MCL124299309 10.4149/neo_2014_019
ProtModification: MAPK1 ---> MCL127004568 10.3892/or.2016.4695
ProtModification: MAPK1 ---> MCL118676833 10.1158/0008-5472.CAN-08-057910.1158/0008-547
ProtModification: MAPK1 ---> MCL118676833 10.1158/0008-5472.CAN-08-057910.1158/0008-547
ProtModification: MAPK1 ---> MCL128522751 10.1158/0008-5472.CAN-16-347010.1158/0008-547
ProtModification: MAPK1 ---> MCL1 10.1128/IAI.00504-17
ProtModification: MAPK1 ---> MCL1 10.1074/jbc.RA118.006029
ProtModification: MAPK1 ---> MCL1 10.1074/jbc.RA118.006029
ProtModification: MAPK1 ---> MCL1 10.2174/1568026619666190902151307
ProtModification: MAPK1 ---> MCL117670986
ProtModification: MAPK1 ---> MCL118846109
ProtModification: MAPK1 ---> MCL121507240 10.1186/1476-4598-10-44
ProtModification: MAPK1 ---> MCL121507240 10.1186/1476-4598-10-44
ProtModification: MAPK1 ---> MCL121730980 10.1038/bjc.2011.242
ProtModification: MAPK1 ---> MCL119249395 10.1053/j.gastro.2008.12.064
ProtModification: MAPK1 ---> MCL125196280 10.1016/j.yexcr.2014.08.039
ProtModification: MAPK1 ---> MCL124779770 10.1186/1476-4598-13-98
positive Expression: MAPK1 --+> M23943652 10.1182/blood-2012-07-445635
positive Expression: MAPK1 --+> M12855556 10.1182/blood-2003-03-0970
positive Expression: MAPK1 --+> M32651234 10.1074/jbc.RA120.013364
positive Expression: MAPK1 --+> M16109713 10.1074/jbc.M506551200
positive Expression: MAPK1 --+> M18198176 10.1074/jbc.M709500200
positive Expression: MAPK1 --+> M20452986 10.1074/jbc.M110.103010
positive Expression: MAPK1 --+> M12963734 10.1074/jbc.M304793200
positive Expression: MAPK1 --+> M12963734 10.1074/jbc.M304793200

positive Expression: MAPK1 --+> M16014620 10.1074/jbc.M505537200
positive Expression: MAPK1 --+> M31301315 10.1016/j.taap.2019.114662
positive Expression: MAPK1 --+> M28526201 10.1016/j.virol.2016.12.011
positive Expression: MAPK1 --+> M28802908 10.1016/j.blre.2017.08.004
positive Expression: MAPK1 --+> M28987383 10.1016/j.canlet.2017.09.040
positive Expression: MAPK1 --+> M28934637 10.1016/j.ctrv.2017.08.012
positive Expression: MAPK1 --+> M31337603 10.1016/j.ymthe.2019.06.014
positive Expression: MAPK1 --+> M29805077 10.1016/j.ccell.2018.05.001
positive Expression: MAPK1 --+> M26299965 10.1016/j.celrep.2015.07.055
positive Expression: MAPK1 --+> M20394737 10.1016/j.bcp.2010.04.010
positive Expression: MAPK1 --+> M15970427 10.1016/j.cellsig.2005.05.021
positive Expression: MAPK1 --+> M20079433 10.1016/j.bbadis.2009.12.009
positive Expression: MAPK1 --+> M20969960 10.1016/j.semcancer.2010.10.011
positive Expression: MAPK1 --+> M19733679 10.1016/j.micinf.2009.08.014
positive Expression: MAPK1 --+> M17097560 10.1016/j.ccr.2006.10.006
positive Expression: MAPK1 --+> M19154985 10.1016/j.chom.2008.11.010
positive Expression: MAPK1 --+> M23524145 10.1016/j.yexcr.2013.03.012
positive Expression: MAPK1 --+> M24121058 10.1016/j.pharmthera.2013.10.001
positive Expression: MAPK1 --+> M24096094 10.1016/j.bone.2013.09.020
positive Expression: MAPK1 --+> M24096094 10.1016/j.bone.2013.09.020
positive Expression: MAPK1 --+> M25747387 10.1016/j.febslet.2015.02.026
positive Expression: MAPK1 --+> M26346348 10.1016/j.leukres.2015.08.007
positive Expression: MAPK1 --+> M26346348 10.1016/j.leukres.2015.08.007
positive Expression: MAPK1 --+> M26367324 10.1016/j.antiviral.2015.09.005
positive Expression: MAPK1 --+> M12107261
positive Expression: MAPK1 --+> M21219631
positive Expression: MAPK1 --+> M19498045
positive Expression: MAPK1 --+> M18198176
positive Expression: MAPK1 --+> M16109713
positive Expression: MAPK1 --+> M12963734
positive Expression: MAPK1 --+> M12963734
positive Expression: MAPK1 --+> M11368774
positive Expression: MAPK1 --+> M21258408
positive Expression: MAPK1 --+> M21258408
positive Expression: MAPK1 --+> M21258408
positive Expression: MAPK1 --+> M18846109
positive Expression: MAPK1 --+> M11314001
positive Expression: MAPK1 --+> M12612065
positive Expression: MAPK1 --+> M27529549 10.1371/journal.pone.0161343
positive Expression: MAPK1 --+> M26243391 10.1189/jlb.2MA1214-599R
positive Expression: MAPK1 --+> M27479182 10.1038/leu.2016.213
positive Expression: MAPK1 --+> M29311244 10.1128/IAI.00504-17
positive Expression: MAPK1 --+> M29311244 10.1128/IAI.00504-17
positive Expression: MAPK1 --+> M27169422 10.1007/82_2016_13
positive Expression: MAPK1 --+> M30150431 10.21873/invivo.11351
positive Expression: MAPK1 --+> M31337603 10.1016/j.ymthe.2019.06.014
positive Expression: MAPK1 --+> M32269717
positive Expression: MAPK1 --+> M32081857 10.1038/s41419-020-2344-0
positive Expression: MAPK1 --+> MCL1 10.1074/jbc.ra120.013364
positive Expression: MAPK1 --+> M33755101 10.1042/BCJ20210006
positive Expression: MAPK1 --+> M33926484 10.1186/s12967-021-02848-9
positive Expression: MAPK1 --+> M21979753 10.2119/molmed.2011.00164
positive Expression: MAPK1 --+> M21979753 10.2119/molmed.2011.00164
positive Expression: MAPK1 --+> M21219631 10.1186/1471-2407-11-7

positive Expression: MAPK1 --+> M23088735 10.1186/1479-5876-10-211
positive Expression: MAPK1 --+> M23773282 10.1186/1475-2867-13-61
positive Expression: MAPK1 --+> M21637292 10.1038/cdd.2011.75
positive Expression: MAPK1 --+> M22064351 10.1038/leu.2011.287
positive Expression: MAPK1 --+> M21170021 10.1038/nchembio.496
positive Expression: MAPK1 --+> M21258408 10.1038/onc.2010.616
positive Expression: MAPK1 --+> M22761917 10.1371/journal.pone.0039870
positive Expression: MAPK1 --+> M22761917 10.1371/journal.pone.0039870
positive Expression: MAPK1 --+> M22761917 10.1371/journal.pone.0039870
positive Expression: MAPK1 --+> M24025415 10.4161/cbt.26341
positive Expression: MAPK1 --+> M23542173 10.1038/onc.2013.74
positive Expression: MAPK1 --+> M24556687 10.1038/cddis.2014.40
positive Expression: MAPK1 --+> M24096476 10.1038/onc.2013.389
positive Expression: MAPK1 --+> M24824212 10.1152/physiolgenomics.00173.2013
positive Expression: MAPK1 --+> M25275600 10.1038/cddis.2014.391
positive Expression: MAPK1 --+> M25879227 10.1371/journal.pone.0124407
positive Expression: MAPK1 --+> M21979753 10.2119/molmed.2011.0016410.2119/molmed.2011.
positive Expression: MAPK1 --+> M433 10.1016/j.blre.2017.08.004
positive Expression: MAPK1 --+> MCL1 10.1016/j.ymthe.2019.06.014
positive Expression: MAPK1 --+> MCL1 10.1189/jlb.2MA1214-599R
positive Expression: MAPK1 --+> MCL1 10.1128/IAI.00504-17
positive Expression: MAPK1 --+> MCL1 10.1128/IAI.00504-17
positive Expression: MAPK1 --+> MCL1 10.1016/j.ymthe.2019.06.014
positive Expression: MAPK1 --+> MCL1 10.1038/s41419-020-2344-0
positive Expression: MAPK1 --+> M10972974
positive Expression: MAPK1 --+> M25422792 10.1186/2162-3619-3-2710.1186/2162-3619-3-27
positive Expression: MAPK1 --+> M25986861 10.1016/j.bbamcr.2015.05.01410.1016/j.bbamcr.
positive Expression: MAPK1 --+> M27765849 10.1158/1535-7163.MCT-16-0017
positive Expression: MAPK1 --+> M28491825
positive Expression: MAPK1 --+> M28765329 10.1158/1078-0432.CCR-17-157410.1158/1078-043
positive Expression: MAPK1 --+> M17463001
positive Expression: MAPK1 --+> M17463001 10.1074/jbc.M610010200
positive Expression: MAPK1 --+> M29105654 10.1038/s41467-017-01477-5
positive Expression: MAPK1 --+> M22898329 10.1186/1750-2187-7-12
positive Expression: MAPK1 --+> M15291360 10.1080/10428190310001639470
positive Expression: MAPK1 --+> M20117835 10.1016/j.leukres.2009.07.013
positive Expression: MAPK1 --+> M19874848 10.1016/j.pharmthera.2009.10.001
positive Expression: MAPK1 --+> M23158473 10.1186/1476-4598-11-84
positive Expression: MAPK1 --+> M23158473 10.1186/1476-4598-11-84
positive Expression: MAPK1 --+> M23158473 10.1186/1476-4598-11-84
positive Expression: MAPK1 --+> M23158473 10.1186/1476-4598-11-84
positive Expression: MAPK1 --+> M23158473 10.1186/1476-4598-11-84
positive Expression: MAPK1 --+> M23158473 10.1186/1476-4598-11-84
positive Expression: MAPK1 --+> M23158473 10.1186/1476-4598-11-84
positive Expression: MAPK1 --+> M22179790 10.3892/ijo.2011.1303
positive Expression: MAPK1 --+> M22179790 10.3892/ijo.2011.1303
positive Expression: MAPK1 --+> M22179790 10.3892/ijo.2011.1303
positive Expression: MAPK1 --+> M19554262 10.1007/s10637-009-9281-1
positive Expression: MAPK1 --+> M23088735 10.1186/1479-5876-10-211
positive Expression: MAPK1 --+> M23696245 10.1124/mol.113.086140
positive Expression: MAPK1 --+> M22064351 10.1038/leu.2011.287
positive Expression: MAPK1 --+> M21996740 10.1038/onc.2011.424
positive Expression: MAPK1 --+> M24523919 10.1371/journal.pone.0088587
positive Expression: MAPK1 --+> M24594907 10.1371/journal.pone.0089064

positive Expression: MAPK1 --+> M	19554262	10.1007/s10637-009-9281-1
positive Expression: MAPK1 --+> M	23158473	10.1186/1476-4598-11-84
positive Expression: MAPK1 --+> M	26005182	10.1016/j.ijmm.2015.04.006
positive Expression: MAPK1 --+> M	16516835	10.1016/j.devcel.2006.01.002
positive Expression: MAPK1 --+> M	17014711	
positive Expression: MAPK1 --+> M	10602518	
positive Expression: MAPK1 --+> M	23158473	10.1186/1476-4598-11-84
positive Expression: MAPK1 --+> M	23773282	10.1186/1475-2867-13-61
positive Expression: MAPK1 --+> M	23056582	10.1371/journal.pone.0047060
positive Expression: MAPK1 --+> M	23678184	10.1128/JVI.00626-13
positive Expression: MAPK1 --+> MCL1		
positive Expression: MAPK1 --+> M	28765329	10.1158/1078-0432.CCR-17-157410.1158/1078-043
positive Expression: MAPK1 --+> M	29792376	10.1089/scd.2017.0287
positive Expression: MAPK1 --+> M	34741044	10.1038/s41419-021-04329-9
positive Expression: MAPK1 --+> M	34654885	10.1038/s41375-021-01443-7
positive Expression: MAPK1 --+> M	35218446	10.1007/s11033-022-07243-1
positive Expression: MAPK1 --+> M	35744993	10.3390/molecules27123865
positive Expression: MAPK1 --+> M	35725771	10.1038/s41375-022-01627-9
positive Expression: MAPK1 --+> MCL1		10.1016/j.blre.2022.101036
positive Regulation: NGF --+> CD	21501463	10.1186/1471-2164-12-19610.1186/1471-2164-12-
positive Regulation: NGF --+> CD	22410557	10.1167/iovs.11-843010.1167/iovs.11-8430
Regulation: DST ---> RAC1	19403692	
Regulation: DST ---> RAC1	19403692	
Regulation: DST ---> RAC1	30059630	10.1146/annurev-cellbio-100617-062534
Regulation: DST ---> RAC1	22718344	10.1242/jcs.097600
Regulation: DST ---> RAC1	24357722	10.1242/jcs.128454
Regulation: DST ---> RAC1	19403692	10.1091/mbc.E09-01-005110.1091/mbc.E09-01-005
Regulation: DST ---> RAC1		
negative Regulation: RAC1 ---| MC	30013448	10.7150/ijms.25527
negative Regulation: RAC1 ---| MC	31413255	10.1038/s41467-019-11490-5
negative Regulation: RAC1 ---| MCL1		10.1038/s41467-019-11490-5
positive Expression: SPP1 --+> CI	28830685	10.1016/j.yexcr.2017.08.028
positive Expression: SPP1 --+> CI	28830685	10.1016/j.yexcr.2017.08.028
positive Expression: SPP1 --+> CI	28830685	10.1016/j.yexcr.2017.08.028
positive Expression: SPP1 --+> CI	30902885	10.1136/gutjnl-2019-318419
positive Expression: SPP1 --+> CI	30902885	10.1136/gutjnl-2019-318419
positive Expression: SPP1 --+> CI	30902885	10.1136/gutjnl-2019-318419
positive Expression: SPP1 --+> CI	30902885	10.1136/gutjnl-2019-318419
positive Expression: SPP1 --+> CI	30902885	10.1136/gutjnl-2019-318419
positive Expression: SPP1 --+> CI	30902885	10.1136/gutjnl-2019-318419
positive Expression: SPP1 --+> CI	30902885	10.1136/gutjnl-2019-318419
positive Expression: SPP1 --+> CI	30902885	10.1136/gutjnl-2019-318419
positive Expression: SPP1 --+> CI	32236573	10.3892/ijo.2020.5035
positive Expression: SPP1 --+> CI	34178613	10.3389/fonc.2021.59285410.3389/fonc.2021.592
positive Expression: SPP1 --+> CI	34423566	10.1111/1759-7714.1410810.1111/1759-7714.1410
positive Expression: SPP1 --+> CD274		10.1136/gutjnl-2019-318419
positive Expression: SPP1 --+> CD274		10.1136/gutjnl-2019-318419
positive Expression: SPP1 --+> CD274		10.1136/gutjnl-2019-318419
positive Expression: SPP1 --+> CD274		10.1136/gutjnl-2019-318419
positive Expression: SPP1 --+> CD274		10.1136/gutjnl-2019-318419
positive Expression: SPP1 --+> CD274		10.1136/gutjnl-2019-318419
positive Expression: SPP1 --+> CD274		10.1136/gutjnl-2019-318419
positive Expression: SPP1 --+> CI	28830685	10.1016/j.yexcr.2017.08.02810.1016/j.yexcr.20
positive Expression: SPP1 --+> CI	30206757	10.1007/s10620-018-5282-2

positive Expression: SPP1 --+> CI	28830685	10.1016/j.yexcr.2017.08.028
positive Expression: SPP1 --+> CI	28830685	10.1016/j.yexcr.2017.08.028
positive Expression: SPP1 --+> CD274		10.1136/gutjnl-2019-318419
positive Expression: SPP1 --+> CD274		10.1136/gutjnl-2019-318419
positive Expression: SPP1 --+> CD274		10.1136/gutjnl-2019-318419
positive Expression: SPP1 --+> CI	36071302	10.1007/s12032-022-01801-0
positive Regulation: RAC1 --+> hy	29410394	10.12659/MSM.907468
positive Regulation: RAC1 --+> hy	29410394	10.12659/MSM.907468
positive Regulation: RAC1 --+> hy	29410394	10.12659/MSM.907468
positive Regulation: RAC1 --+> hy	29410394	10.12659/MSM.907468
positive Regulation: RAC1 --+> hy	29410394	10.12659/MSM.907468
positive Regulation: RAC1 --+> hy	29410394	10.12659/MSM.907468
positive Regulation: RAC1 --+> hy	29410394	10.12659/MSM.907468
positive Regulation: RAC1 --+> hy	29410394	10.12659/MSM.907468
positive Regulation: RAC1 --+> hy	29410394	10.12659/MSM.907468
positive QuantitativeChange: hypc	29410394	10.12659/MSM.907468
positive QuantitativeChange: hypc	29410394	10.12659/MSM.907468
Expression: NOTCH1 ---> PTEN	21035501	10.1016/j.advenzreg.2010.09.012
Expression: NOTCH1 ---> PTEN	18455982	10.1016/j.cell.2008.04.013
Expression: NOTCH1 ---> PTEN	20223597	10.1016/j.ctrv.2010.02.005
Expression: NOTCH1 ---> PTEN		10.1016/j.urolonc.2011.07.006
Expression: NOTCH1 ---> PTEN		10.1016/j.urolonc.2011.07.006
Expression: NOTCH1 ---> PTEN		10.1016/j.urolonc.2011.07.006
Expression: NOTCH1 ---> PTEN	18951834	10.1016/j.drup.2008.09.002
Expression: NOTCH1 ---> PTEN	22897852	10.1016/j.ccr.2012.06.014
Expression: NOTCH1 ---> PTEN	23348702	10.1016/j.canlet.2013.01.020
Expression: NOTCH1 ---> PTEN	24090996	10.1016/j.leukres.2013.09.007
Expression: NOTCH1 ---> PTEN	21993533	10.1016/j.urolonc.2011.07.006
Expression: NOTCH1 ---> PTEN	21993533	10.1016/j.urolonc.2011.07.006
Expression: NOTCH1 ---> PTEN	21993533	10.1016/j.urolonc.2011.07.006
Expression: NOTCH1 ---> PTEN	24780321	10.1016/j.lfs.2014.04.017
Expression: NOTCH1 ---> PTEN	26123366	10.1016/j.exphem.2015.05.017
Expression: NOTCH1 ---> PTEN	26683354	10.1016/j.cca.2015.12.010
Expression: NOTCH1 ---> PTEN	26505911	10.1016/j.exphem.2015.10.004
Expression: NOTCH1 ---> PTEN	26392376	10.1016/j.semcancer.2015.09.009
Expression: NOTCH1 ---> PTEN	18830414	
Expression: NOTCH1 ---> PTEN	18830414	
Expression: NOTCH1 ---> PTEN	20015880	
Expression: NOTCH1 ---> PTEN	20015880	
Expression: NOTCH1 ---> PTEN	19117948	
Expression: NOTCH1 ---> PTEN	19117947	
Expression: NOTCH1 ---> PTEN	27138341	10.1371/journal.pone.0154771
Expression: NOTCH1 ---> PTEN	27070592	10.3390/ijms17040531
Expression: NOTCH1 ---> PTEN		10.1172/JCI84637
Expression: NOTCH1 ---> PTEN	22623890	10.1100/2012/252457
Expression: NOTCH1 ---> PTEN	22074495	10.1186/1476-4598-10-138
Expression: NOTCH1 ---> PTEN	22074495	10.1186/1476-4598-10-138
Expression: NOTCH1 ---> PTEN	22074495	10.1186/1476-4598-10-138
Expression: NOTCH1 ---> PTEN		
Expression: NOTCH1 ---> PTEN	23634247	
Expression: NOTCH1 ---> PTEN	21566651	10.1038/leu.2011.103
Expression: NOTCH1 ---> PTEN	21743488	10.1038/onc.2011.279
Expression: NOTCH1 ---> PTEN	22002304	10.1038/onc.2011.467
Expression: NOTCH1 ---> PTEN	22002304	10.1038/onc.2011.467

Expression: NOTCH1 ---> PTEN	22002304	10.1038/onc.2011.467
Expression: NOTCH1 ---> PTEN	23950991	10.1371/journal.pone.0070737
Expression: NOTCH1 ---> PTEN	24113181	10.1038/cddis.2013.375
Expression: NOTCH1 ---> PTEN	24113181	10.1038/cddis.2013.375
Expression: NOTCH1 ---> PTEN	24113181	10.1038/cddis.2013.375
Expression: NOTCH1 ---> PTEN	24113181	10.1038/cddis.2013.375
Expression: NOTCH1 ---> PTEN	24113181	10.1038/cddis.2013.375
Expression: NOTCH1 ---> PTEN	24113181	10.1038/cddis.2013.375
Expression: NOTCH1 ---> PTEN	24113181	10.1038/cddis.2013.375
Expression: NOTCH1 ---> PTEN	24113181	10.1038/cddis.2013.375
Expression: NOTCH1 ---> PTEN	24113181	10.1038/cddis.2013.375
Expression: NOTCH1 ---> PTEN	23699387	10.1095/biolreprod.113.108100
Expression: NOTCH1 ---> PTEN	24286423	10.3760/cma.j.issn.0366-6999.20130721
Expression: NOTCH1 ---> PTEN	24415757	10.1074/jbc.M113.519686
Expression: NOTCH1 ---> PTEN	24905788	10.1146/annurev-biochem-082411-113907
Expression: NOTCH1 ---> PTEN	24988225	10.1371/journal.pone.0101024
Expression: NOTCH1 ---> PTEN	25781910	10.1080/15384047.2015.1016662
Expression: NOTCH1 ---> PTEN	25043004	10.1038/nature13495
Expression: NOTCH1 ---> PTEN	26228240	10.1038/ncomms8935
Expression: NOTCH1 ---> PTEN	26228240	10.1038/ncomms8935
Expression: NOTCH1 ---> PTEN	26228240	10.1038/ncomms8935
Expression: NOTCH1 ---> PTEN	26491213	10.1155/2015/520590
Expression: NOTCH1 ---> PTEN	25781910	10.1080/15384047.2015.1016662
Expression: NOTCH1 ---> PTEN		10.1071/RD14212
Expression: NOTCH1 ---> PTEN		10.1071/RD14212
Expression: NOTCH1 ---> PTEN	17245125	
Expression: NOTCH1 ---> PTEN	17245125	
Expression: NOTCH1 ---> PTEN	17873882	
Expression: NOTCH1 ---> PTEN	19479935	10.1002/jcb.22199
Expression: NOTCH1 ---> PTEN	19479935	10.1002/jcb.22199
Expression: NOTCH1 ---> PTEN	19479935	10.1002/jcb.22199
Expression: NOTCH1 ---> PTEN	21993533	10.1016/j.urolonc.2011.07.006
Expression: NOTCH1 ---> PTEN	24780321	10.1016/j.lfs.2014.04.017
Expression: NOTCH1 ---> PTEN	25110169	10.3892/ijmm.2014.1889
Expression: NOTCH1 ---> PTEN	28437168	10.1080/15384101.2017.1310351
Expression: NOTCH1 ---> PTEN	28627671	10.3892/mmr.2017.6791
Expression: NOTCH1 ---> PTEN	28627671	10.3892/mmr.2017.6791
Expression: NOTCH1 ---> PTEN	28627671	10.3892/mmr.2017.6791
Expression: NOTCH1 ---> PTEN	28627671	10.3892/mmr.2017.6791
Expression: NOTCH1 ---> PTEN	28603282	10.1038/cmi.2017.30
Expression: NOTCH1 ---> PTEN	29286063	10.3892/ijmm.2017.3319
Expression: NOTCH1 ---> PTEN	29496446	10.1016/j.bbrc.2018.02.18710.1016/j.bbrc.2018
Expression: NOTCH1 ---> PTEN	29496446	10.1016/j.bbrc.2018.02.18710.1016/j.bbrc.2018
Expression: NOTCH1 ---> PTEN	28951560	10.1038/leu.2017.301
Expression: NOTCH1 ---> PTEN	28951560	10.1038/leu.2017.301
Expression: NOTCH1 ---> PTEN	29532851	10.3892/ijmm.2018.3548
Expression: NOTCH1 ---> PTEN	417	10.1016/j.bbamcr.2018.07.006
Regulation: TP53 ---> RAC1	12750388	
Regulation: TP53 ---> RAC1	12578823	
Regulation: TP53 ---> RAC1	12578823	
Regulation: TP53 ---> RAC1	14729984	
Regulation: TP53 ---> RAC1	14729984	
Regulation: TP53 ---> RAC1	14729984	
Regulation: TP53 ---> RAC1	14729984	

Regulation: TP53 ---> RAC1 22745590 10.1593/neo.12352
Regulation: TP53 ---> RAC1 21391906
Regulation: TP53 ---> RAC1 26036474 10.1124/jpet.115.223610
Regulation: TP53 ---> RAC1 12578823
Regulation: TP53 ---> RAC1 25771001 10.1016/j.biomaterials.2015.01.007
Regulation: TP53 ---> RAC1 12750388
Regulation: TP53 ---> RAC1
Regulation: KDM5B ---> PTEN 28958389 10.1016/j.trecan.2017.08.004
Regulation: KDM5B ---> PTEN 28958389 10.1016/j.trecan.2017.08.004
Regulation: KDM5B ---> PTEN 30628696 10.3892/mmr.2019.9832
Regulation: KDM5B ---> PTEN 25909289 10.18632/oncotarget.3713
Regulation: KDM5B ---> PTEN 1245 10.1016/j.trecan.2017.08.004
Regulation: KDM5B ---> PTEN 1245 10.1016/j.trecan.2017.08.004
Regulation: KDM5B ---> PTEN 10.3892/mmr.2019.9832
positive Regulation: CD274 --+> (35818041 10.1186/s12885-022-09838-0
negative Expression: TP53 ---| G(11239498 10.1016/S0006-2952(00)00592-X
negative Expression: TP53 ---| G(24802641 10.1371/journal.pone.009646610.1371/journal.p
negative Expression: GCLC ---| TF 24873722 10.1016/j.freeradbiomed.2014.05.017
positive Expression: AKT1 --+> M(26436206 10.1016/j.bbrc.2015.09.182
positive Expression: AKT1 --+> M(32184020 10.1016/j.bbrc.2020.02.165
positive Expression: AKT1 --+> M(27095788 10.1182/blood-2015-10-675009
positive Expression: AKT1 --+> M(20061561 10.1182/blood-2009-08-239244
positive Expression: AKT1 --+> M(12637318 10.1182/blood-2002-11-3396
positive Expression: AKT1 --+> M(12637318 10.1182/blood-2002-11-3396
positive Expression: AKT1 --+> M(12637318 10.1182/blood-2002-11-3396
positive Expression: AKT1 --+> M(20164468 10.1182/blood-2009-08-239483
positive Expression: AKT1 --+> M(20940415 10.1182/blood-2010-04-189977
positive Expression: AKT1 --+> M(29470962 10.1016/j.yexcr.2018.02.019
positive Expression: AKT1 --+> M(11278698 10.1074/jbc.M010551200
positive Expression: AKT1 --+> M(30782845 10.1074/jbc.RA118.006029
positive Expression: AKT1 --+> M(28073911 10.1074/jbc.M116.755157
positive Expression: AKT1 --+> M(23897826 10.1074/jbc.M113.475913
positive Expression: AKT1 --+> M(17311912 10.1074/jbc.M700445200
positive Expression: AKT1 --+> M(17545167 10.1074/jbc.M701663200
positive Expression: AKT1 --+> M(16051596 10.1074/jbc.M508187200
positive Expression: AKT1 --+> M(30213730 10.1016/j.taap.2018.09.008
positive Expression: AKT1 --+> M(30213730 10.1016/j.taap.2018.09.008
positive Expression: AKT1 --+> M(30213730 10.1016/j.taap.2018.09.008
positive Expression: AKT1 --+> M(30213730 10.1016/j.taap.2018.09.008
positive Expression: AKT1 --+> M(30213730 10.1016/j.taap.2018.09.008
positive Expression: AKT1 --+> M(30213730 10.1016/j.taap.2018.09.008
positive Expression: AKT1 --+> M(30055197 10.1016/j.antiviral.2018.07.015
positive Expression: AKT1 --+> M(30439592 10.1016/j.ejmech.2018.11.001
positive Expression: AKT1 --+> M(28987383 10.1016/j.canlet.2017.09.040
positive Expression: AKT1 --+> M(29864454 10.1016/j.canlet.2018.05.048
positive Expression: AKT1 --+> M(28934637 10.1016/j.ctrv.2017.08.012
positive Expression: AKT1 --+> M(30476750 10.1016/j.ctrv.2018.11.001
positive Expression: AKT1 --+> M(30236849 10.1016/j.biopha.2018.08.158
positive Expression: AKT1 --+> M(32931746 10.1016/j.molcel.2020.08.017
positive Expression: AKT1 --+> M(32931746 10.1016/j.molcel.2020.08.017
positive Expression: AKT1 --+> M(33839456 10.1016/j.neo.2021.03.005
positive Expression: AKT1 --+> M(29805077 10.1016/j.ccell.2018.05.001
positive Expression: AKT1 --+> M(27601002 10.1016/j.clml.2016.08.008
positive Expression: AKT1 --+> M(32898245 10.1182/bloodadvances.2019001393

positive Expression: AKT1 --+> M(19250631 10.1016/j.bbrc.2009.01.097
positive Expression: AKT1 --+> M(19250643 10.1016/j.bbrc.2009.01.094
positive Expression: AKT1 --+> M(15940637 10.1053/j.gastro.2005.03.010
positive Expression: AKT1 --+> M(15940637 10.1053/j.gastro.2005.03.010
positive Expression: AKT1 --+> M(15940637 10.1053/j.gastro.2005.03.010
positive Expression: AKT1 --+> M(15940637 10.1053/j.gastro.2005.03.010
positive Expression: AKT1 --+> M(15940637 10.1053/j.gastro.2005.03.010
positive Expression: AKT1 --+> M(15940637 10.1053/j.gastro.2005.03.010
positive Expression: AKT1 --+> M(15940637 10.1053/j.gastro.2005.03.010
positive Expression: AKT1 --+> M(15940637 10.1053/j.gastro.2005.03.010
positive Expression: AKT1 --+> M(19428480 10.1016/j.dci.2009.02.007
positive Expression: AKT1 --+> M(19874848 10.1016/j.pharmthera.2009.10.001
positive Expression: AKT1 --+> M(21345578 10.1016/j.canlet.2011.01.012
positive Expression: AKT1 --+> M(22343223 10.1016/j.canlet.2012.02.008
positive Expression: AKT1 --+> M(11882383 10.1016/S0898-6568(01)00271-6
positive Expression: AKT1 --+> M(16905331 10.1016/j.smim.2006.07.003
positive Expression: AKT1 --+> M(23524145 10.1016/j.yexcr.2013.03.012
positive Expression: AKT1 --+> M(23524145 10.1016/j.yexcr.2013.03.012
positive Expression: AKT1 --+> M(23524145 10.1016/j.yexcr.2013.03.012
positive Expression: AKT1 --+> M(23524145 10.1016/j.yexcr.2013.03.012
positive Expression: AKT1 --+> M(23524145 10.1016/j.yexcr.2013.03.012
positive Expression: AKT1 --+> M(23524145 10.1016/j.yexcr.2013.03.012
positive Expression: AKT1 --+> M(25199984 10.1016/j.bbcan.2014.08.006
positive Expression: AKT1 --+> M(25199984 10.1016/j.bbcan.2014.08.006
positive Expression: AKT1 --+> M(25533111 10.1016/j.ymgme.2014.11.017
positive Expression: AKT1 --+> M(19815839
positive Expression: AKT1 --+> M(19815839
positive Expression: AKT1 --+> M(17545167
positive Expression: AKT1 --+> M(17014711
positive Expression: AKT1 --+> M(16051596
positive Expression: AKT1 --+> M(15967790
positive Expression: AKT1 --+> M(12960271
positive Expression: AKT1 --+> M(11278698
positive Expression: AKT1 --+> M(19942932
positive Expression: AKT1 --+> M(19407827
positive Expression: AKT1 --+> M(19407827
positive Expression: AKT1 --+> M(14767488
positive Expression: AKT1 --+> M(12445202
positive Expression: AKT1 --+> M(12445202
positive Expression: AKT1 --+> M(34349823 10.1155/2021/5516655
positive Expression: AKT1 --+> M(33469228 10.1038/s41418-020-00717-4
positive Expression: AKT1 --+> M(32598308 10.1515/hmbci-2020-000910.1515/hmbci-2020-000
positive Expression: AKT1 --+> M(18483303 10.1158/1535-7163.MCT-07-218310.1158/1535-716
positive Expression: AKT1 --+> M(19457567 10.1016/j.jhep.2009.03.02410.1016/j.jhep.2009
positive Expression: AKT1 --+> M(21670080 10.1158/0008-5472.CAN-10-453110.1158/0008-547
positive Expression: AKT1 --+> M(30213730 10.1016/j.taap.2018.09.00810.1016/j.taap.2018
positive Expression: AKT1 --+> M(32667864 10.1152/ajpcell.00181.2020
positive Expression: AKT1 --+> MCL1 10.1016/j.bbrc.2020.02.165
positive Expression: AKT1 --+> MCL1 10.1016/j.canlet.2011.01.012
positive Expression: AKT1 --+> MCL1 10.1016/j.canlet.2012.02.008
positive Expression: AKT1 --+> M(11457886
positive Expression: AKT1 --+> M(11457886
positive Expression: AKT1 --+> M(12445202
positive Expression: AKT1 --+> M(12445202

positive Expression: AKT1 --+> MC(11593385
positive Expression: AKT1 --+> MC(11593385
positive Expression: AKT1 --+> MC(11314001
positive Expression: AKT1 --+> MC(11314001
positive Expression: AKT1 --+> MC(11314001
positive Expression: AKT1 --+> MCL1 10.1189/jlb.3A1114-537RR
positive Expression: AKT1 --+> MC(27431988 10.3892/ijo.2016.3624
positive Expression: AKT1 --+> MC(27479182 10.1038/leu.2016.213
positive Expression: AKT1 --+> MC(27460078 10.1007/s13277-016-5260-2
positive Expression: AKT1 --+> MCL1
positive Expression: AKT1 --+> MC(27465322 10.1007/s11596-016-1614-7
positive Expression: AKT1 --+> MC(27465322 10.1007/s11596-016-1614-7
positive Expression: AKT1 --+> MCL1 10.2174/1389450116666150825113035
positive Expression: AKT1 --+> MCL1 10.3390/ijms18061258
positive Expression: AKT1 --+> MC(28192408 10.1038/onc.2016.515
positive Expression: AKT1 --+> MC(29849119 10.1038/s41388-018-0345-6
positive Expression: AKT1 --+> MCL1
positive Expression: AKT1 --+> MCL1
positive Expression: AKT1 --+> MCL1 10.1007/978-981-13-7342-8_5
positive Expression: AKT1 --+> MC(22680924
positive Expression: AKT1 --+> MC(23051914 10.4161/auto.22145
positive Expression: AKT1 --+> MC(23158473 10.1186/1476-4598-11-84
positive Expression: AKT1 --+> MC(23354006 10.3892/ijo.2013.1789
positive Expression: AKT1 --+> MC(21525012 10.1074/jbc.M110.207571
positive Expression: AKT1 --+> MC(23410153 10.2174/09298673113209990106
positive Expression: AKT1 --+> MC(23909734 10.1080/01635581.2013.802001
positive Expression: AKT1 --+> MC(23897826 10.1074/jbc.M113.475913
positive Expression: AKT1 --+> MC(23670294 10.1038/leu.2013.151
positive Expression: AKT1 --+> MC(23825534 10.1371/journal.pone.0066268
positive Expression: AKT1 --+> MC(24130723 10.1371/journal.pone.0075576
positive Expression: AKT1 --+> MC(24327018 10.1038/bjc.2013.754
positive Expression: AKT1 --+> MC(24327018 10.1038/bjc.2013.754
positive Expression: AKT1 --+> MC(24523919 10.1371/journal.pone.0088587
positive Expression: AKT1 --+> MC(23542173 10.1038/onc.2013.74
positive Expression: AKT1 --+> MC(24556687 10.1038/cddis.2014.40
positive Expression: AKT1 --+> MC(28192408 10.1038/onc.2016.515
positive Expression: AKT1 --+> MC(23354006 10.3892/ijo.2013.1789
positive Expression: AKT1 --+> MC(21773964 10.1055/s-0031-1280780
positive Expression: AKT1 --+> MC(24492287 10.1097/CAD.0000000000000081
positive Expression: AKT1 --+> MC(25032861 10.1038/cddis.2014.303
positive Expression: AKT1 --+> MC(25032861 10.1038/cddis.2014.303
positive Expression: AKT1 --+> MCL1 10.1189/jlb.1A0414-212R
positive Expression: AKT1 --+> MCL1 10.1189/jlb.1A0414-212R
positive Expression: AKT1 --+> MCL1 10.1172/JCI75821
positive Expression: AKT1 --+> MC(22252875 10.1128/IAI.05219-11
positive Expression: AKT1 --+> MC(22252875 10.1128/IAI.05219-11
positive Expression: AKT1 --+> MC(22252875 10.1128/IAI.05219-11
positive Expression: AKT1 --+> MC(24824212 10.1152/physiolgenomics.00173.2013
positive Expression: AKT1 --+> MC(25590803 10.1038/cddis.2014.525
positive Expression: AKT1 --+> MC(25275600 10.1038/cddis.2014.391
positive Expression: AKT1 --+> MCL1 10.1128/JVI.02879-15
positive Expression: AKT1 --+> MCL1 10.1128/JVI.02879-15
positive Expression: AKT1 --+> MC(26073081 10.1038/onc.2015.188
positive Expression: AKT1 --+> MC(15940637 10.1053/j.gastro.2005.03.010

positive Expression: AKT1 --+> MC23561469 10.1016/j.hoc.2013.01.002
positive Expression: AKT1 --+> MC19933775
positive Expression: AKT1 --+> MC19933775
positive Expression: AKT1 --+> MC19933775
positive Expression: AKT1 --+> MC18559346
positive Expression: AKT1 --+> MC17014711
positive Expression: AKT1 --+> MC16710358
positive Expression: AKT1 --+> MC11593385
positive Expression: AKT1 --+> MCL1 10.2174/1381612811319660758
positive Expression: AKT1 --+> MC10454566
positive Expression: AKT1 --+> MC10961875
positive Expression: AKT1 --+> MC15940637
positive Expression: AKT1 --+> MC15940637
positive Expression: AKT1 --+> MC16327976
positive Expression: AKT1 --+> MC17009247
positive Expression: AKT1 --+> MC17928528
positive Expression: AKT1 --+> MC18483275 10.1158/0008-5472.CAN-07-6286
positive Expression: AKT1 --+> MC19523441 10.1016/j.bbrc.2009.05.149
positive Expression: AKT1 --+> MC19407827 10.1038/cdd.2009.38
positive Expression: AKT1 --+> MC23524145 10.1016/j.yexcr.2013.03.012
positive Expression: AKT1 --+> MC24859932 10.3892/or.2014.3218
positive Expression: AKT1 --+> MCL1 10.1074/jbc.M115.713545
positive Expression: AKT1 --+> MC28192408 10.1038/onc.2016.51510.1038/onc.2016.515
positive Expression: AKT1 --+> MCL1
positive Expression: AKT1 --+> MC29507660 10.18632/oncotarget.2292610.18632/oncotarget.
positive Expression: AKT1 --+> MC29652658
positive Expression: AKT1 --+> MC30213730 10.1016/j.taap.2018.09.00810.1016/j.taap.2018
positive Expression: AKT1 --+> MC30213730 10.1016/j.taap.2018.09.00810.1016/j.taap.2018
positive Expression: AKT1 --+> MC32305283 10.1016/j.taap.2020.11501310.1016/j.taap.2020
positive Expression: AKT1 --+> MC33132196 10.1016/j.yexcr.2020.11234310.1016/j.yexcr.20
positive Expression: AKT1 --+> MC33839456 10.1016/j.neo.2021.03.005
positive Expression: AKT1 --+> MC34360791 10.3390/ijms22158023
positive Expression: AKT1 --+> MC34715860 10.1186/s12935-021-02282-3
positive Expression: AKT1 --+> MC34654885 10.1038/s41375-021-01443-7
positive Expression: AKT1 --+> MC35136016 10.1038/s41419-022-04551-z
positive Expression: AKT1 --+> MC35315320 10.1016/j.exphem.2022.03.006
positive Expression: AKT1 --+> MC34528729 10.1002/JLB.2RU0221-088RRR
positive Expression: AKT1 --+> MC35218446 10.1007/s11033-022-07243-1
positive Expression: AKT1 --+> MC36180828 10.1186/s10020-022-00547-9
positive Expression: AKT1 --+> MC36243843 10.1007/978-3-031-06566-8_6
positive Expression: AKT1 --+> MC36241868 10.1038/s41388-022-02482-9
negative PromoterBinding: TP53 --15047174 10.1016/j.bbrc.2004.03.029
negative PromoterBinding: TP53 --10792001 10.1172/JCI9290
negative PromoterBinding: TP53 --15548710 10.1158/0008-5472.CAN-04-1612
negative PromoterBinding: TP53 --12647018 10.1007/s00280-002-0551-2
negative PromoterBinding: TP53 --18528466
negative PromoterBinding: TP53 --22094098
negative PromoterBinding: TP53 --11483599 10.1074/jbc.M103429200
negative PromoterBinding: TP53 --14573776 10.1124/mol.64.5.1259
negative PromoterBinding: TP53 --22753715
negative PromoterBinding: TP53 --23969950 10.3892/or.2013.2671
negative PromoterBinding: TP53 ---| ABCC1 10.2174/0929867324666161118130238
negative PromoterBinding: TP53 --18528466
negative PromoterBinding: TP53 --14573776 10.1124/mol.64.5.1259

negative PromoterBinding: TP53 --14573776
negative PromoterBinding: TP53 --14573776 10.1124/mol.64.5.1259
negative PromoterBinding: TP53 --10792001 10.1172/JCI9290
negative PromoterBinding: TP53 ---| ABCC1
negative Expression: CCL3L1 ---| 34153005 10.1172/JCI148542
Expression: PTEN ---> BECN1 27107786 10.1016/j.toxlet.2016.04.015
Expression: PTEN ---> BECN1 27107786 10.1016/j.toxlet.2016.04.015
Expression: PTEN ---> BECN1 26218645 10.1080/15548627.2015.1074372
Expression: PTEN ---> BECN1 829 10.1016/j.cub.2017.02.015
Expression: PTEN ---> BECN1 23811268 10.1016/j.semcancer.2013.06.007
Expression: PTEN ---> BECN1 29412697 10.1089/cbr.2017.230610.1089/cbr.2017.2306
Regulation: PAX8-AS1 ---> PAX8 32293699 10.5603/EP.a2020.0021
Regulation: PAX8-AS1 ---> PAX8 27225188 10.1007/s00438-016-1217-910.1007/s00438-016-1
Regulation: PAX8-AS1 ---> PAX8 36248434 10.1155/2022/2295044
Regulation: CYP2J2 ---> MAPK1 22260463 10.1089/hum.2011.135
Regulation: CYP2J2 ---> MAPK1 22223859 10.1194/jlr.M017319
Regulation: CYP2J2 ---> MAPK1 23835530 10.3892/ijmm.2013.1435
Regulation: CYP2J2 ---> MAPK1 25389363 10.1152/ajpendo.00118.2014
negative PromoterBinding: PPARG -21247904 10.1074/jbc.M110.199612
negative PromoterBinding: PPARG -31277946 10.1016/j.bbrc.2019.06.16010.1016/j.bbrc.2019
negative PromoterBinding: PPARG -31277946 10.1016/j.bbrc.2019.06.16010.1016/j.bbrc.2019
negative PromoterBinding: PPARG -24569375 10.1172/JCI7058710.1172/JCI70587
negative PromoterBinding: PPARG -11861425 10.1161/hh0302.105098
negative PromoterBinding: PPARG -11861425 10.1161/hh0302.105098
negative PromoterBinding: PPARG -11861425 10.1161/hh0302.105098
negative PromoterBinding: PPARG -11861425 10.1161/hh0302.105098
negative PromoterBinding: PPARG -20460605 10.1152/physiolgenomics.00064.2010
negative PromoterBinding: PPARG -23447133 10.1152/ajpregu.00607.2012
negative PromoterBinding: PPARG ---| SPP1 10.1172/JCI70587
negative PromoterBinding: PPARG -21103061 10.1371/journal.pone.0013959
negative PromoterBinding: PPARG -11426586
negative PromoterBinding: PPARG ---| SPP1
negative PromoterBinding: PPARG ---| SPP1 10.1016/j.bbrc.2019.06.160
negative PromoterBinding: PPARG ---| SPP1 10.1016/j.bbrc.2019.06.160
negative PromoterBinding: PPARG -33834866 10.1152/AJPHEART.01009.2020
negative PromoterBinding: PPARG -34462564 10.1038/s41401-021-00756-810.1038/s41401-021-
negative PromoterBinding: PPARG -34462564 10.1038/s41401-021-00756-8
negative PromoterBinding: PPARG -34462564 10.1038/s41401-021-00756-8
Binding: SPP1 ---- PTEN 25035430 10.1074/jbc.M114.554519
Binding: SPP1 ---- PTEN 34181042 10.1007/s00262-021-02995-4
positive Expression: AKT1 --+> G(29792861 10.1016/j.bbrc.2018.05.141
positive Expression: AKT1 --+> G(17681938 10.1074/jbc.M701916200
positive Expression: AKT1 --+> G(17681938 10.1074/jbc.M701916200
positive Expression: AKT1 --+> G(26424114 10.1016/j.freeradbiomed.2015.08.011
positive Expression: AKT1 --+> G(20399244 10.1016/j.tox.2010.04.003
positive Expression: AKT1 --+> G(19463904 10.1016/j.neuroscience.2009.05.025
positive Expression: AKT1 --+> G(18926903 10.1016/j.freeradbiomed.2008.09.013
positive Expression: AKT1 --+> G(18926903 10.1016/j.freeradbiomed.2008.09.013
positive Expression: AKT1 --+> G(18926903 10.1016/j.freeradbiomed.2008.09.013
positive Expression: AKT1 --+> G(22995213 10.1016/j.bbagen.2012.09.008
positive Expression: AKT1 --+> G(17681938
positive Expression: AKT1 --+> G(17681938
positive Expression: AKT1 --+> G(30805762 10.1007/s10565-019-09467-7
positive Expression: AKT1 --+> G(30805762 10.1007/s10565-019-09467-7

positive Expression: AKT1 --+> G(	26324295	10.3892/ijmm.2015.2329
positive Expression: AKT1 --+> G(	17109620	10.2174/156720206778792876
positive Expression: AKT1 --+> G(	25195717	10.1016/j.ntt.2014.08.007
positive Expression: AKT1 --+> G(	21560358	
positive Expression: AKT1 --+> G(	21728338	10.1021/tx200090n
positive Expression: AKT1 --+> G(	25028796	10.3390/md12074214
positive Expression: AKT1 --+> G(	35675271	10.1080/01635581.2022.2069274
positive Regulation: PTGS2 --+> F	21525007	10.1074/jbc.M111.236398
positive Regulation: PTGS2 --+> F	18208815	10.1074/jbc.M703318200
positive Regulation: PTGS2 --+> F	18208815	10.1074/jbc.M703318200
positive Regulation: PTGS2 --+> F	18208815	10.1074/jbc.M703318200
positive Regulation: PTGS2 --+> F	18208815	10.1074/jbc.M703318200
positive Regulation: PTGS2 --+> F	30278282	10.1016/j.bioorg.2018.09.034
positive Regulation: PTGS2 --+> F	15013132	10.1016/j.jacc.2003.08.048
positive Regulation: PTGS2 --+> F	33662521	10.1016/j.alcohol.2020.08.007
positive Regulation: PTGS2 --+> F	33662521	10.1016/j.alcohol.2020.08.007
positive Regulation: PTGS2 --+> F	32559626	10.1016/j.biopha.2020.110388
positive Regulation: PTGS2 --+> F	27376854	10.1016/j.intimp.2016.06.027
positive Regulation: PTGS2 --+> F	27376854	10.1016/j.intimp.2016.06.027
positive Regulation: PTGS2 --+> F	31026757	10.1016/j.pharep.2019.02.011
positive Regulation: PTGS2 --+> PPARG		10.1016/S0002-9440(10)64856-0
positive Regulation: PTGS2 --+> F	16531147	10.1016/j.amjsurg.2005.11.009
positive Regulation: PTGS2 --+> F	20026014	10.1016/j.brainres.2009.12.014
positive Regulation: PTGS2 --+> F	20223456	10.1016/j.atherosclerosis.2010.02.003
positive Regulation: PTGS2 --+> F	20538289	10.1016/j.jss.2010.03.011
positive Regulation: PTGS2 --+> F	12269390	10.1016/S0024-3205(02)01510-2
positive Regulation: PTGS2 --+> F	15629510	10.1016/j.jhep.2004.09.011
positive Regulation: PTGS2 --+> F	16023763	10.1016/j.jhep.2005.02.032
positive Regulation: PTGS2 --+> F	17561353	10.1016/j.psyneuen.2007.04.011
positive Regulation: PTGS2 --+> F	19429187	10.1016/j.brainresbull.2009.02.006
positive Regulation: PTGS2 --+> F	16443360	10.1016/j.jnutbio.2005.10.007
positive Regulation: PTGS2 --+> F	23340376	10.1016/j.atherosclerosis.2012.12.007
positive Regulation: PTGS2 --+> F	23340376	10.1016/j.atherosclerosis.2012.12.007
positive Regulation: PTGS2 --+> F	25529449	10.1016/j.bbrc.2014.12.063
positive Regulation: PTGS2 --+> F	25529449	10.1016/j.bbrc.2014.12.063
positive Regulation: PTGS2 --+> F	18079203	
positive Regulation: PTGS2 --+> F	18208815	
positive Regulation: PTGS2 --+> F	18208815	
positive Regulation: PTGS2 --+> F	18208815	
positive Regulation: PTGS2 --+> F	18208815	
positive Regulation: PTGS2 --+> F	18208815	
positive Regulation: PTGS2 --+> F	18208815	
positive Regulation: PTGS2 --+> F	12065695	
positive Regulation: PTGS2 --+> F	11861647	
positive Regulation: PTGS2 --+> F	11861647	
positive Regulation: PTGS2 --+> F	11861647	
positive Regulation: PTGS2 --+> F	11861647	
positive Regulation: PTGS2 --+> F	11861647	
positive Regulation: PTGS2 --+> F	11861647	
positive Regulation: PTGS2 --+> F	17463321	
positive Regulation: PTGS2 --+> F	17463321	
positive Regulation: PTGS2 --+> F	17463321	
positive Regulation: PTGS2 --+> F	17463321	
positive Regulation: PTGS2 --+> F	17463321	

positive Regulation: PTGS2 --+> F 16385084
positive Regulation: PTGS2 --+> F 27431935 10.3892/ijmm.2016.2674
positive Regulation: PTGS2 --+> F 27339155 10.1007/s00421-016-3414-y
positive Regulation: PTGS2 --+> F 28445238 10.1097/CCM.0000000000002440
positive Regulation: PTGS2 --+> F 28445238 10.1097/CCM.0000000000002440
positive Regulation: PTGS2 --+> F 28445238 10.1097/CCM.0000000000002440
positive Regulation: PTGS2 --+> F 29928066 10.1371/journal.ppat.1007100
positive Regulation: PTGS2 --+> F 31026757 10.1016/j.pharep.2019.02.011
positive Regulation: PTGS2 --+> F 31063413 10.1139/cjpp-2018-0720
positive Regulation: PTGS2 --+> F 31335352 10.1097/COC.0000000000000579
positive Regulation: PTGS2 --+> F 31652556 10.3390/molecules24203801
positive Regulation: PTGS2 --+> F 31519825 10.1523/JNEUROSCI.1249-19.2019
positive Regulation: PTGS2 --+> F 33673390 10.3390/molecules26040985
positive Regulation: PTGS2 --+> F 21443470
positive Regulation: PTGS2 --+> F 21525007 10.1074/jbc.M111.236398
positive Regulation: PTGS2 --+> F 23943857 10.1194/jlr.M042283
positive Regulation: PTGS2 --+> F 25171731 10.1159/000365417
positive Regulation: PTGS2 --+> F 25171731 10.1159/000365417
positive Regulation: PTGS2 --+> F 27109015 10.3892/mmr.2016.5150
positive Regulation: PTGS2 --+> F 15629510 10.1016/j.jhep.2004.09.011
positive Regulation: PTGS2 --+> F 17463321 10.1161/01.RES.0000268411.49545.9c
positive Regulation: PTGS2 --+> F 20159581 10.1016/j.jbiosc.2009.09.00310.1016/j.jbiosc.
positive Regulation: PTGS2 --+> F 22138303 10.1016/j.bbalip.2011.11.00410.1016/j.bbalip.
positive Regulation: PTGS2 --+> F 25171731 10.1159/00036541710.1159/000365417
positive Regulation: PTGS2 --+> F 25529449 10.1016/j.bbrc.2014.12.06310.1016/j.bbrc.2014
positive Regulation: PTGS2 --+> F 26863638 10.18632/oncotarget.721310.18632/oncotarget.7
positive Regulation: PTGS2 --+> F 27376854 10.1016/j.intimp.2016.06.02710.1016/j.intimp.
positive Regulation: PTGS2 --+> F 28445238 10.1097/CCM.000000000000244010.1097/CCM.0000C
positive Regulation: PTGS2 --+> PPARG 10.1523/JNEUROSCI.1249-19.2019
positive Regulation: PTGS2 --+> PPARG 10.1097/CCM.0000000000002440
positive Regulation: PTGS2 --+> PPARG 10.1097/CCM.0000000000002440
positive Regulation: PTGS2 --+> PPARG 10.1097/CCM.0000000000002440
positive Regulation: PTGS2 --+> PPARG 10.1097/COC.0000000000000579
positive Regulation: PTGS2 --+> PPARG 10.3390/molecules24203801
positive Regulation: PTGS2 --+> F 28445238 10.1097/CCM.000000000000244010.1097/CCM.0000C
positive Regulation: PTGS2 --+> F 15876425 10.1016/j.bcp.2005.03.024
positive Regulation: PTGS2 --+> F 23340376 10.1016/j.atherosclerosis.2012.12.007
positive Regulation: PTGS2 --+> F 18208815
positive Regulation: PTGS2 --+> F 11861647
positive Regulation: PTGS2 --+> F 24906289 10.1007/s00441-014-1920-y
positive Regulation: PTGS2 --+> F 14555539
positive Regulation: PTGS2 --+> F 15266333
positive Regulation: PTGS2 --+> F 17459764
positive Regulation: PTGS2 --+> PPARG
positive Regulation: PTGS2 --+> F 36456159 10.21873/anticanres.16084
positive Regulation: PTGS2 --+> F 36456159 10.21873/anticanres.16084
negative PromoterBinding: PPARG - 19684609 10.1038/aps.2009.119
negative PromoterBinding: PPARG - 14636669 10.1016/S0163-7827(03)00037-7
negative PromoterBinding: PPARG - 20656057 10.1016/j.bbalip.2010.07.004
negative PromoterBinding: PPARG - 18055759 10.1124/mol.107.042002
negative PromoterBinding: PPARG - 21333731 10.1016/j.tiv.2011.02.004
negative PromoterBinding: PPARG - 16374840 10.1002/hep.20994
negative PromoterBinding: PPARG - 20554962 10.4049/jimmunol.1000866
negative PromoterBinding: PPARG - 16417216 10.1038/sj.jid.5700028

negative PromoterBinding: PPARG -19578162 10.1194/jlr.M900255-JLR200
negative PromoterBinding: PPARG -17669705 10.1016/j.bone.2007.06.017
negative PromoterBinding: PPARG -16443360 10.1016/j.jnutbio.2005.10.007
negative PromoterBinding: PPARG -17316818 10.1016/j.plipres.2007.01.001
negative PromoterBinding: PPARG -15741054 10.1016/j.jnutbio.2004.11.001
negative PromoterBinding: PPARG -16303334 10.1016/j.clim.2005.09.018
negative PromoterBinding: PPARG -18362203
negative PromoterBinding: PPARG -14645533
negative PromoterBinding: PPARG -20635307 10.1055/s-0030-1250111
negative PromoterBinding: PPARG -23365414 10.1095/biolreprod.112.107383
negative PromoterBinding: PPARG -24403546 10.1095/biolreprod.113.109223
negative PromoterBinding: PPARG -16443360 10.1016/j.jnutbio.2005.10.007
negative PromoterBinding: PPARG ---| PTGS2 10.1016/j.jnutbio.2004.11.001
negative PromoterBinding: PPARG -19684609 10.1038/aps.2009.119
negative PromoterBinding: PPARG -20656057 10.1016/j.bbalip.2010.07.004
negative PromoterBinding: PPARG -18055759 10.1124/mol.107.042002
negative PromoterBinding: PPARG -21333731 10.1016/j.tiv.2011.02.004
negative PromoterBinding: PPARG -16374840 10.1002/hep.20994
negative PromoterBinding: PPARG -20554962 10.4049/jimmunol.1000866
negative PromoterBinding: PPARG -16417216 10.1038/sj.jid.5700028
negative PromoterBinding: PPARG -19578162 10.1194/jlr.M900255-JLR200
negative PromoterBinding: PPARG -17669705 10.1016/j.bone.2007.06.017
positive Regulation: PTEN --+> cł24434152 10.1016/j.bbrc.2014.01.014
positive Regulation: PTEN --+> cł11729185 10.1074/jbc.M108302200
positive Regulation: PTEN --+> cł11495901 10.1074/jbc.M103632200
positive Regulation: PTEN --+> cł11495901 10.1074/jbc.M103632200
positive Regulation: PTEN --+> cł11495901 10.1074/jbc.M103632200
positive Regulation: PTEN --+> cł11495901 10.1074/jbc.M103632200
positive Regulation: PTEN --+> cł27721020 10.1016/j.canlet.2016.10.003
positive Regulation: PTEN --+> cł33220333 10.1016/j.canlet.2020.11.016
positive Regulation: PTEN --+> cł32827577 10.1016/j.bbcan.2020.188405
positive Regulation: PTEN --+> cł28755993 10.1016/j.ijpharm.2017.07.056
positive Regulation: PTEN --+> cł21187093 10.1016/j.febslet.2010.12.027
positive Regulation: PTEN --+> cł22465665 10.1016/j.febslet.2012.03.006
positive Regulation: PTEN --+> cł16545436 10.1016/j.ygyno.2005.12.033
positive Regulation: PTEN --+> cł15072831 10.1016/j.canlet.2003.10.028
positive Regulation: PTEN --+> cł15072831 10.1016/j.canlet.2003.10.028
positive Regulation: PTEN --+> cł23920276 10.1016/j.phymed.2013.07.002
positive Regulation: PTEN --+> cł25744176 10.1016/j.drudis.2015.02.011
positive Regulation: PTEN --+> cł21108789
positive Regulation: PTEN --+> cł21108789
positive Regulation: PTEN --+> cł19299556
positive Regulation: PTEN --+> cł11729185
positive Regulation: PTEN --+> cł11495901
positive Regulation: PTEN --+> cł11495901
positive Regulation: PTEN --+> cł11495901
positive Regulation: PTEN --+> cł11495901
positive Regulation: PTEN --+> cł18458673
positive Regulation: PTEN --+> cł16404430
positive Regulation: PTEN --+> cł11103942
positive Regulation: PTEN --+> cł28887606 10.1007/s10616-017-0134-z
positive Regulation: PTEN --+> cł30616477 10.1177/0300060518819606
positive Regulation: PTEN --+> cł33076450 10.3390/ijms21207633
positive Regulation: PTEN --+> cł33721218 10.1007/s13577-021-00519-x

positive Regulation: PTEN --+> ch	33721218	10.1007/s13577-021-00519-x
positive Regulation: PTEN --+> ch	33721218	10.1007/s13577-021-00519-x
positive Regulation: PTEN --+> ch	33721218	10.1007/s13577-021-00519-x
positive Regulation: PTEN --+> ch	33721218	10.1007/s13577-021-00519-x
positive Regulation: PTEN --+> ch	21108789	10.1186/1476-4598-9-305
positive Regulation: PTEN --+> ch	21108789	10.1186/1476-4598-9-305
positive Regulation: PTEN --+> ch	20736773	10.1097/PGP.0b013e3181de3068
positive Regulation: PTEN --+> ch	26043084	10.1371/journal.pone.0128886
positive Regulation: PTEN --+> ch	11103942	10.1038/sj.onc.1203918
positive Regulation: PTEN --+> ch	16404430	10.1038/sj.bjc.6602926
positive Regulation: PTEN --+> ch	16438844	
positive Regulation: PTEN --+> ch	21820606	10.1016/j.arcmed.2011.06.00810.1016/j.arcmed.
positive Regulation: PTEN --+> ch	28686971	10.1016/j.biopha.2017.06.08910.1016/j.biopha.
positive Regulation: PTEN --+> ch	30925916	10.1186/s13046-019-1119-x10.1186/s13046-019-1
positive Regulation: PTEN --+> ch	33721218	10.1007/s13577-021-00519-x10.1007/s13577-021-
positive Regulation: PTEN --+> chemosensit		10.1016/j.febslet.2012.03.006
positive Regulation: PTEN --+> chemosensit		10.1177/0300060518819606
positive Regulation: PTEN --+> ch	27147467	10.1007/s00018-016-2253-7
positive Regulation: PTEN --+> ch	22781804	
positive Regulation: PTEN --+> ch	27955382	
positive Regulation: PTEN --+> chemosensit		10.1042/BSR20180773
positive Regulation: PTEN --+> ch	34681603	10.3390/ijms222010944
Regulation: ABCC1 ---> GCLC	21728338	10.1021/tx200090n
Regulation: ABCC1 ---> GCLC	21728338	10.1021/tx200090n
positive PromoterBinding: PHF20 -	22334668	10.1074/jbc.M111.333922
positive PromoterBinding: PHF20 -	22334668	10.1074/jbc.M111.333922
positive PromoterBinding: PHF20 -	22334668	10.1074/jbc.M111.333922
positive PromoterBinding: PHF20 -	22334668	10.1074/jbc.M111.333922
positive PromoterBinding: PHF20 -	22334668	10.1074/jbc.M111.333922
positive PromoterBinding: PHF20 -	22334668	10.1074/jbc.M111.333922
positive PromoterBinding: PHF20 -	22334668	10.1074/jbc.M111.333922
positive PromoterBinding: PHF20 -	22334668	10.1074/jbc.M111.333922
positive PromoterBinding: PHF20 -	22334668	10.1074/jbc.M111.333922
positive PromoterBinding: PHF20 -	32555448	10.1038/s41418-020-0580-6
negative Expression: PPARG ---| I	36624108	10.1038/s41598-022-25737-7
negative Expression: TNFAIP8L2 --	29568863	10.3892/mmr.2018.8789
negative Expression: TNFAIP8L2 --	29568863	10.3892/mmr.2018.8789
Regulation: PTEN ---> RAC1	12393431	10.1182/blood-2002-04-1251
Regulation: PTEN ---> RAC1	23806663	10.1016/j.bbamcr.2013.06.009
Regulation: PTEN ---> RAC1		10.1016/j.ijdevneu.2008.09.171
Regulation: PTEN ---> RAC1	15246683	10.1016/j.bbamcr.2004.04.010
Regulation: PTEN ---> RAC1	21943825	10.1016/j.canlet.2011.08.023
Regulation: PTEN ---> RAC1		10.1016/S0955-0674(00)00105-8
Regulation: PTEN ---> RAC1		10.1016/S0955-0674(00)00105-8
Regulation: PTEN ---> RAC1	17560336	10.1016/j.ccr.2007.04.021
Regulation: PTEN ---> RAC1	22995297	10.1016/j.bbrc.2012.09.025
Regulation: PTEN ---> RAC1	23000914	10.1016/j.bcp.2012.09.008
Regulation: PTEN ---> RAC1	23993968	10.1016/j.cellsig.2013.08.017
Regulation: PTEN ---> RAC1	19864456	
Regulation: PTEN ---> RAC1	12960424	
Regulation: PTEN ---> RAC1	16341149	
Regulation: PTEN ---> RAC1	16341149	
Regulation: PTEN ---> RAC1	19940030	
Regulation: PTEN ---> RAC1	26166433	10.1038/ncomms8721

Regulation: PTEN ---> RAC1	26166433	10.1038/ncomms8721
Regulation: PTEN ---> RAC1	26166433	10.1038/ncomms8721
Regulation: PTEN ---> RAC1	26166433	10.1038/ncomms8721
Regulation: PTEN ---> RAC1	27033067	10.1007/978-1-4939-3299-3_2
Regulation: PTEN ---> RAC1	28710285	10.1042/BST20160269
Regulation: PTEN ---> RAC1	29361670	10.1152/ajprenal.00575.2017
Regulation: PTEN ---> RAC1	31481407	10.1128/IAI.00553-19
Regulation: PTEN ---> RAC1	22745590	10.1593/neo.12352
Regulation: PTEN ---> RAC1	23755300	10.1371/journal.pone.0066209
Regulation: PTEN ---> RAC1	23755300	10.1371/journal.pone.0066209
Regulation: PTEN ---> RAC1	23755300	10.1371/journal.pone.0066209
Regulation: PTEN ---> RAC1	24523903	10.1371/journal.pone.0088489
Regulation: PTEN ---> RAC1	24823941	10.1194/jlr.M048207
Regulation: PTEN ---> RAC1	10753747	10.1016/s0960-9822(00)00417-6
Regulation: PTEN ---> RAC1	10753747	10.1016/s0960-9822(00)00417-6
Regulation: PTEN ---> RAC1	19864456	10.1091/mbc.E09-08-069210.1091/mbc.e09-08-069
Regulation: PTEN ---> RAC1	23806663	10.1016/j.bbamcr.2013.06.00910.1016/j.bbamcr.
Regulation: PTEN ---> RAC1	29872505	10.18632/oncotarget.2534910.18632/oncotarget.
Regulation: PTEN ---> RAC1	34480897	10.1016/j.jbc.2021.101161
Regulation: PTEN ---> RAC1	17535963	
Regulation: PTEN ---> RAC1		10.1042/BST20160269
Regulation: PTEN ---> RAC1	28642362	10.1083/jcb.201610102
Regulation: PTEN ---> RAC1		10.1128/IAI.00553-19
Regulation: PTEN ---> RAC1	16973299	10.1016/j.neuroscience.2006.07.049
Regulation: PTEN ---> RAC1	25829446	10.1126/scisignal.200584010.1126/scisignal.20
Regulation: PTEN ---> RAC1	25829446	10.1126/scisignal.200584010.1126/scisignal.20
Regulation: PTEN ---> RAC1		
positive Regulation: PAX8-AS1 --+	29693272	10.1002/jcb.2688110.1002/jcb.26881
positive Expression: PTEN --+> BE	27107786	10.1016/j.toxlet.2016.04.015
positive Expression: PTEN --+> BE	27107786	10.1016/j.toxlet.2016.04.015
positive Expression: PTEN --+> BE	29412697	10.1089/cbr.2017.2306
positive Expression: PTEN --+> BE	29412697	10.1089/cbr.2017.2306
positive Expression: PTEN --+> BE	30875347	10.1097/CAD.0000000000000724
positive Expression: PTEN --+> BE	26218645	10.1080/15548627.2015.1074372
positive Expression: PTEN --+> BE	29361670	10.1152/ajprenal.00575.2017
positive Expression: PTEN --+> BE	23811268	10.1016/j.semcancer.2013.06.007
positive Expression: PTEN --+> BECN1		10.1097/CAD.0000000000000724
positive Expression: PTEN --+> BE	29412697	10.1089/cbr.2017.230610.1089/cbr.2017.2306
positive Expression: PTEN --+> BE	829	10.1016/j.cub.2017.02.015
positive Expression: PTEN --+> BE	35647006	10.3389/fsurg.2022.767611
Regulation: MAPK1 ---> PRR5L	22609986	10.1038/ncb2507
Regulation: MAPK1 ---> PRR5L	22609986	10.1038/ncb2507
Binding: PPARG ---- GPER1	31741264	10.1007/s11033-019-05196-6
Binding: PPARG ---- GPER1		10.1007/s11033-019-05196-6
positive QuantitativeChange: hypo	31620898	10.1007/s00432-019-03047-210.1007/s00432-019-
Regulation: PPARG ---> PAX8	19041825	10.1016/j.beem.2008.09.017
Regulation: PPARG ---> PAX8		10.1016/j.path.2013.10.001
Regulation: PPARG ---> PAX8	16179407	
Regulation: PPARG ---> PAX8	12161538	
Regulation: RBP1 ---> PPARG	20498279	10.1128/MCB.00014-1010.1128/MCB.00014-10
negative Expression: GCLC ---| AE	9813007	
positive Regulation: CD274 --+> N	36266281	10.1038/s41467-022-33739-2
positive Regulation: CD274 --+> N	36266281	10.1038/s41467-022-33739-2
positive Expression: MAPK1 --+> (	29066411	10.1016/j.fct.2017.10.033

positive Expression: MAPK1 --+> GCLC 10.1016/S0006-291X(02)00816-1
positive Expression: MAPK1 --+> (19328227 10.1016/j.freeradbiomed.2009.03.016
positive Expression: MAPK1 --+> (19328227 10.1016/j.freeradbiomed.2009.03.016
positive Expression: MAPK1 --+> (19328227 10.1016/j.freeradbiomed.2009.03.016
positive Expression: MAPK1 --+> (25201354 10.1016/j.tox.2014.09.002
positive Expression: MAPK1 --+> (26424114 10.1016/j.freeradbiomed.2015.08.011
positive Expression: MAPK1 --+> (22864849 10.1007/s00204-012-0913-4
positive Expression: MAPK1 --+> (26424114 10.1016/j.freeradbiomed.2015.08.01110.1016/j.
positive Expression: MAPK1 --+> (15477009 10.1016/j.freeradbiomed.2004.07.021
positive Expression: MAPK1 --+> (15683710 10.1016/j.freeradbiomed.2004.11.012
positive Expression: MAPK1 --+> (18691715 10.1016/j.atherosclerosis.2008.06.024
positive Expression: MAPK1 --+> (20399244 10.1016/j.tox.2010.04.003
positive Expression: MAPK1 --+> (12147223
positive Expression: MAPK1 --+> GCLC
Regulation: STC2 ---> MAPK1 25816401 10.1177/0394632015572745
Regulation: STC2 ---> MAPK1 23187001 10.5483/bmbrep.2012.45.11.086
Regulation: STC2 ---> MAPK1 27878259 10.3892/mmr.2016.595110.3892/mmr.2016.5951
Regulation: STC2 ---> MAPK1 27878259 10.3892/mmr.2016.595110.3892/mmr.2016.5951
Regulation: STC2 ---> MAPK1 31162839 10.1002/ijc.3248710.1002/ijc.32487
Regulation: STC2 ---> MAPK1 35501821 10.1186/s13046-022-02370-w
MolTransport: CYP2J2 ---> AKT1 28554983 10.1194/jlr.M074229
MolTransport: CYP2J2 ---> AKT1 27416746 10.1111/acel.1250710.1111/acel.12507
MolTransport: CYP2J2 ---> AKT1 34639055 10.3390/ijms221910714
MolTransport: CYP2J2 ---> AKT1 25245389 10.1161/HYPERTENSIONAHA.114.0388410.1161/HYPE
MolTransport: CYP2J2 ---> AKT1 23041291 10.1016/j.prostaglandins.2012.09.00410.1016/j
MolTransport: CYP2J2 ---> AKT1 26859195 10.1097/FJC.0000000000000371
MolTransport: CYP2J2 ---> AKT1 22223859 10.1194/jlr.M017319
MolTransport: CYP2J2 ---> AKT1 22223859 10.1194/jlr.M017319
MolTransport: CYP2J2 ---> AKT1 22223859 10.1194/jlr.M017319
Regulation: NET1 ---> AKT1 29769144 10.1186/s13058-018-0966-2
Regulation: NET1 ---> AKT1 32393835 10.1038/s41388-020-1315-3
Regulation: NET1 ---> AKT1 33839702 10.18632/aging.202845
Regulation: NET1 ---> AKT1 33839702 10.18632/aging.202845
Regulation: NET1 ---> AKT1 33839702 10.18632/aging.20284510.18632/aging.202845
Regulation: TP53 ---> BECN1 30529040 10.1016/j.pharmthera.2018.12.005
Regulation: TP53 ---> BECN1 10.1016/j.virusres.2012.03.010
Regulation: TP53 ---> BECN1 22472731 10.1016/j.virusres.2012.03.010
Regulation: TP53 ---> BECN1 25010388 10.1016/j.mam.2014.05.001
Regulation: TP53 ---> BECN1 27118659 10.1016/j.molonc.2016.04.001
Regulation: TP53 ---> BECN1 29417176 10.1007/s00018-018-2759-2
Regulation: TP53 ---> BECN1 29976630 10.21873/cgp.20083
Regulation: TP53 ---> BECN1 30089855 10.1038/s41374-018-0099-3
Regulation: TP53 ---> BECN1 29991800 10.1038/s41388-018-0395-9
Regulation: TP53 ---> BECN1 10.1152/ajpcell.00261.2018
Regulation: TP53 ---> BECN1 23233667 10.1074/jbc.M112.390245
Regulation: TP53 ---> BECN1 21178393 10.4161/cbt.11.2.14627
Regulation: TP53 ---> BECN1 23208502 10.1038/onc.2012.556
Regulation: TP53 ---> BECN1 24937773 10.2119/molmed.2014.00063
Regulation: TP53 ---> BECN1 22345153 10.1158/0008-5472.CAN-11-229110.1158/0008-547
Regulation: TP53 ---> BECN1 24841907 10.1021/jf500903g10.1021/jf500903g
Regulation: TP53 ---> BECN1 25208472 10.1111/jcmm.1238610.1111/jcmm.12386
Regulation: TP53 ---> BECN1 30807795 10.1016/j.neulet.2019.02.03610.1016/j.neulet.
Regulation: TP53 ---> BECN1 10.1172/JCI135536
Regulation: TP53 ---> BECN1 25584615 10.3390/ijms16011544

Regulation: TP53 ---> BECN1 25198662 10.3727/096504014X1402416045908710.3727/09650
Regulation: TP53 ---> BECN1 21765470 10.1038/onc.2011.295
Regulation: TP53 ---> BECN1
Regulation: TP53 ---> BECN1 33297472 10.3390/ijms21239325
Regulation: TP53 ---> BECN1 33556475 10.1016/j.matbio.2021.02.001
Regulation: TP53 ---> BECN1 34119535 10.1016/j.mvr.2021.10420710.1016/j.mvr.2021.1
Regulation: TP53 ---> BECN1 34236569 10.1007/s10495-021-01682-0
positive Expression: MAPK1 --+> F 28363868 10.1016/j.bbrc.2017.03.152
positive Expression: MAPK1 --+> F 28288820 10.1016/j.bcp.2017.03.003
positive Expression: MAPK1 --+> F 31377156 10.1016/j.ejphar.2019.172586
positive Expression: MAPK1 --+> F 19531467 10.1074/jbc.M109.028084
positive Expression: MAPK1 --+> F 19531467 10.1074/jbc.M109.028084
positive Expression: MAPK1 --+> F 10722727 10.1074/jbc.275.12.8817
positive Expression: MAPK1 --+> F 10671533 10.1074/jbc.275.7.4949
positive Expression: MAPK1 --+> F 10652293 10.1074/jbc.275.5.3107
positive Expression: MAPK1 --+> F 15496409 10.1074/jbc.M409885200
positive Expression: MAPK1 --+> F 15496409 10.1074/jbc.M409885200
positive Expression: MAPK1 --+> F 18519570 10.1074/jbc.M800263200
positive Expression: MAPK1 --+> F 10930430 10.1074/jbc.M910237199
positive Expression: MAPK1 --+> F 10930430 10.1074/jbc.M910237199
positive Expression: MAPK1 --+> F 10930430 10.1074/jbc.M910237199
positive Expression: MAPK1 --+> F 16371352 10.1074/jbc.M512722200
positive Expression: MAPK1 --+> F 18854308 10.1074/jbc.M806322200
positive Expression: MAPK1 --+> F 16618699 10.1074/jbc.M600861200
positive Expression: MAPK1 --+> F 18208815 10.1074/jbc.M703318200
positive Expression: MAPK1 --+> F 18208815 10.1074/jbc.M703318200
positive Expression: MAPK1 --+> F 18208815 10.1074/jbc.M703318200
positive Expression: MAPK1 --+> F 18208815 10.1074/jbc.M703318200
positive Expression: MAPK1 --+> F 18208815 10.1074/jbc.M703318200
positive Expression: MAPK1 --+> F 22879597 10.1074/jbc.M112.385542
positive Expression: MAPK1 --+> F 15007072 10.1074/jbc.M311279200
positive Expression: MAPK1 --+> F 15007072 10.1074/jbc.M311279200
positive Expression: MAPK1 --+> F 15007072 10.1074/jbc.M311279200
positive Expression: MAPK1 --+> F 15007072 10.1074/jbc.M311279200
positive Expression: MAPK1 --+> F 15007072 10.1074/jbc.M311279200
positive Expression: MAPK1 --+> F 18534982 10.1074/jbc.M800524200
positive Expression: MAPK1 --+> F 15525649 10.1074/jbc.M408172200
positive Expression: MAPK1 --+> F 11292836 10.1074/jbc.M101801200
positive Expression: MAPK1 --+> F 11292836 10.1074/jbc.M101801200
positive Expression: MAPK1 --+> F 11292836 10.1074/jbc.M101801200
positive Expression: MAPK1 --+> F 20018844 10.1074/jbc.M109.032672
positive Expression: MAPK1 --+> F 12907684 10.1074/jbc.M304887200
positive Expression: MAPK1 --+> F 12902324 10.1074/jbc.M306280200
positive Expression: MAPK1 --+> F 12902324 10.1074/jbc.M306280200
positive Expression: MAPK1 --+> F 12902324 10.1074/jbc.M306280200
positive Expression: MAPK1 --+> F 12796504 10.1074/jbc.M300625200
positive Expression: MAPK1 --+> F 12796504 10.1074/jbc.M300625200
positive Expression: MAPK1 --+> F 12796504 10.1074/jbc.M300625200
positive Expression: MAPK1 --+> F 11901151 10.1074/jbc.M111415200
positive Expression: MAPK1 --+> F 12695520 10.1074/jbc.M302076200
positive Expression: MAPK1 --+> F 12695520 10.1074/jbc.M302076200
positive Expression: MAPK1 --+> F 12695520 10.1074/jbc.M302076200
positive Expression: MAPK1 --+> F 10969080 10.1074/jbc.M006168200
positive Expression: MAPK1 --+> F 10930401 10.1074/jbc.M002324200

positive Expression: MAPK1 --+> F10930401 10.1074/jbc.M002324200
positive Expression: MAPK1 --+> F10930401 10.1074/jbc.M002324200
positive Expression: MAPK1 --+> F31326176 10.3168/jds.2018-15774
positive Expression: MAPK1 --+> F22766885 10.1194/jlr.D029546
positive Expression: MAPK1 --+> F22766885 10.1194/jlr.D029546
positive Expression: MAPK1 --+> F30017554 10.1016/j.jaci.2018.06.033
positive Expression: MAPK1 --+> F33126082 10.1016/j.molimm.2020.10.008
positive Expression: MAPK1 --+> F20691240 10.1016/j.fct.2010.07.046
positive Expression: MAPK1 --+> F29331731 10.1016/j.fct.2018.01.013
positive Expression: MAPK1 --+> F31336132 10.1016/j.fct.2019.110693
positive Expression: MAPK1 --+> F31883986 10.1016/j.fct.2019.111092
positive Expression: MAPK1 --+> F31030852 10.1016/j.vetmic.2019.03.005
positive Expression: MAPK1 --+> F31030852 10.1016/j.vetmic.2019.03.005
positive Expression: MAPK1 --+> F31030852 10.1016/j.vetmic.2019.03.005
positive Expression: MAPK1 --+> F29609010 10.1016/j.jep.2018.03.037
positive Expression: MAPK1 --+> F28095354 10.1016/j.biopha.2017.01.041
positive Expression: MAPK1 --+> F30316005 10.1016/j.micpath.2018.10.008
positive Expression: MAPK1 --+> F26453923 10.1016/j.freeradbiomed.2015.08.023
positive Expression: MAPK1 --+> F28647573 10.1016/j.cellsig.2017.06.012
positive Expression: MAPK1 --+> F27343699 10.1016/j.phrs.2016.06.022
positive Expression: MAPK1 --+> F31546014 10.1016/j.phrs.2019.104446
positive Expression: MAPK1 --+> F32707231 10.1016/j.phrs.2020.105090
positive Expression: MAPK1 --+> F28899709 10.1016/j.pupt.2017.09.002
positive Expression: MAPK1 --+> F28282576 10.1016/j.intimp.2017.03.003
positive Expression: MAPK1 --+> F28666238 10.1016/j.intimp.2017.06.022
positive Expression: MAPK1 --+> F29310107 10.1016/j.intimp.2018.01.001
positive Expression: MAPK1 --+> F26110105 10.1016/j.fob.2015.06.001
positive Expression: MAPK1 --+> F26110105 10.1016/j.fob.2015.06.001
positive Expression: MAPK1 --+> PTGS2 10.1016/j.genrep.2020.100972
positive Expression: MAPK1 --+> F16796978 10.1016/j.ajog.2006.05.014
positive Expression: MAPK1 --+> F17071582 10.2353/ajpath.2006.060178
positive Expression: MAPK1 --+> F21356386 10.1016/j.ajpath.2010.11.074
positive Expression: MAPK1 --+> F17313938 10.1016/j.abb.2007.01.009
positive Expression: MAPK1 --+> F22591774 10.1016/j.archoralbio.2012.04.015
positive Expression: MAPK1 --+> F12220506 10.1016/S0006-291X(02)02124-1
positive Expression: MAPK1 --+> F12901876 10.1016/S0006-291X(03)01393-7
positive Expression: MAPK1 --+> F15325243 10.1016/j.bbrc.2004.07.129
positive Expression: MAPK1 --+> F16527254 10.1016/j.bbrc.2006.02.116
positive Expression: MAPK1 --+> F16930556 10.1016/j.bbrc.2006.07.214
positive Expression: MAPK1 --+> F17097611 10.1016/j.bbrc.2006.10.098
positive Expression: MAPK1 --+> F17222389 10.1016/j.bbrc.2006.12.174
positive Expression: MAPK1 --+> F17222389 10.1016/j.bbrc.2006.12.174
positive Expression: MAPK1 --+> F19319478 10.1007/s00011-009-0030-x10.1007/s00011-009-0
positive Expression: MAPK1 --+> F19327173 10.1186/ar266110.1186/ar2661
positive Expression: MAPK1 --+> F19808956 10.1158/0008-5472.CAN-09-231610.1158/0008-547
positive Expression: MAPK1 --+> F23474920 10.1007/s00011-013-0609-010.1007/s00011-013-0
positive Expression: MAPK1 --+> F23524589 10.1161/CIRCRESAHA.112.28039910.1161/CIRCRESA
positive Expression: MAPK1 --+> F24348809 10.3892/etm.2013.1394
positive Expression: MAPK1 --+> F24520260 10.3892/etm.2014.1484
positive Expression: MAPK1 --+> F24852102 10.1016/j.expneurol.2014.05.01410.1016/j.expn
positive Expression: MAPK1 --+> PTGS2 10.3168/jds.2018-15774
positive Expression: MAPK1 --+> PTGS2 10.1016/j.ajpath.2010.11.074
positive Expression: MAPK1 --+> PTGS2 10.1016/j.archoralbio.2012.04.015
positive Expression: MAPK1 --+> F17222389 10.1016/j.bbrc.2006.12.174

positive Expression: MAPK1 --+> F17222389 10.1016/j.bbrc.2006.12.174
positive Expression: MAPK1 --+> F17222389 10.1016/j.bbrc.2006.12.174
positive Expression: MAPK1 --+> F20883667 10.1016/j.bbrc.2010.09.095
positive Expression: MAPK1 --+> F20883667 10.1016/j.bbrc.2010.09.095
positive Expression: MAPK1 --+> F20883667 10.1016/j.bbrc.2010.09.095
positive Expression: MAPK1 --+> F16256955 10.1016/j.bcp.2005.09.023
positive Expression: MAPK1 --+> F16256955 10.1016/j.bcp.2005.09.023
positive Expression: MAPK1 --+> F17632087 10.1016/j.bcp.2007.06.008
positive Expression: MAPK1 --+> F17632087 10.1016/j.bcp.2007.06.008
positive Expression: MAPK1 --+> F17632087 10.1016/j.bcp.2007.06.008
positive Expression: MAPK1 --+> F17632087 10.1016/j.bcp.2007.06.008
positive Expression: MAPK1 --+> F20096264 10.1016/j.bcp.2010.01.008
positive Expression: MAPK1 --+> F22120676 10.1016/j.bcp.2011.11.008
positive Expression: MAPK1 --+> F22120676 10.1016/j.bcp.2011.11.008
positive Expression: MAPK1 --+> F22120676 10.1016/j.bcp.2011.11.008
positive Expression: MAPK1 --+> F22120676 10.1016/j.bcp.2011.11.008
positive Expression: MAPK1 --+> F22138288 10.1016/j.bcp.2011.11.013
positive Expression: MAPK1 --+> F16185670 10.1016/j.brainres.2005.08.033
positive Expression: MAPK1 --+> PTGS2 10.1016/j.brainres.2007.10.018
positive Expression: MAPK1 --+> F21241676 10.1016/j.brainres.2011.01.013
positive Expression: MAPK1 --+> F22050961 10.1016/j.brainres.2011.08.019
positive Expression: MAPK1 --+> F17767925 10.1016/j.cbi.2007.07.008
positive Expression: MAPK1 --+> F18177848 10.1016/j.cbi.2007.12.001
positive Expression: MAPK1 --+> F19931518 10.1016/j.cbi.2009.11.015
positive Expression: MAPK1 --+> F19931518 10.1016/j.cbi.2009.11.015
positive Expression: MAPK1 --+> F14998726 10.1016/j.chemphyslip.2003.11.004
positive Expression: MAPK1 --+> F14998726 10.1016/j.chemphyslip.2003.11.004
positive Expression: MAPK1 --+> F14998726 10.1016/j.chemphyslip.2003.11.004
positive Expression: MAPK1 --+> F14998726 10.1016/j.chemphyslip.2003.11.004
positive Expression: MAPK1 --+> F14998726 10.1016/j.chemphyslip.2003.11.004
positive Expression: MAPK1 --+> F14998726 10.1016/j.chemphyslip.2003.11.004
positive Expression: MAPK1 --+> F17208222 10.1016/j.ejphar.2006.11.060
positive Expression: MAPK1 --+> F17258194 10.1016/j.ejphar.2006.12.005
positive Expression: MAPK1 --+> F19616539 10.1016/j.ejphar.2009.07.007
positive Expression: MAPK1 --+> F20826149 10.1016/j.ejphar.2010.08.015
positive Expression: MAPK1 --+> PTGS2 10.1016/j.ejphar.2010.12.044
positive Expression: MAPK1 --+> F15530865 10.1016/j.yexcr.2004.05.039
positive Expression: MAPK1 --+> F15225655 10.1016/j.febslet.2004.05.070
positive Expression: MAPK1 --+> F16198345 10.1016/j.febslet.2005.08.077
positive Expression: MAPK1 --+> F17889855 10.1016/j.fertnstert.2007.05.053
positive Expression: MAPK1 --+> F18082740 10.1016/j.fertnstert.2007.09.010
positive Expression: MAPK1 --+> F21762900 10.1016/j.fertnstert.2011.06.041
positive Expression: MAPK1 --+> F21762900 10.1016/j.fertnstert.2011.06.041
positive Expression: MAPK1 --+> F16083714 10.1053/j.gastro.2005.06.003
positive Expression: MAPK1 --+> F21726861 10.1016/j.atherosclerosis.2011.06.009
positive Expression: MAPK1 --+> F21968328 10.1016/j.yjmcc.2011.09.014
positive Expression: MAPK1 --+> F17618875 10.1016/j.jpedsurg.2007.02.006
positive Expression: MAPK1 --+> F15848219 10.1016/j.lfs.2004.12.015
positive Expression: MAPK1 --+> F17822719 10.1016/j.lfs.2007.06.027
positive Expression: MAPK1 --+> F12604095 10.1016/S0028-3908(02)00375-1
positive Expression: MAPK1 --+> F16364386 10.1016/j.taap.2005.11.001
positive Expression: MAPK1 --+> PTGS2 10.1016/j.taap.2007.03.019
positive Expression: MAPK1 --+> PTGS2 10.1016/j.taap.2007.03.019
positive Expression: MAPK1 --+> F16275389 10.1016/j.jaci.2005.08.014

positive Expression: MAPK1 --+> F16275389 10.1016/j.jaci.2005.08.014
positive Expression: MAPK1 --+> F16275389 10.1016/j.jaci.2005.08.014
positive Expression: MAPK1 --+> F16275389 10.1016/j.jaci.2005.08.014
positive Expression: MAPK1 --+> F17320164 10.1016/j.mam.2006.12.009
positive Expression: MAPK1 --+> F19467701 10.1016/j.biomaterials.2009.04.034
positive Expression: MAPK1 --+> F19118901 10.1016/j.molimm.2008.11.006
positive Expression: MAPK1 --+> F19118901 10.1016/j.molimm.2008.11.006
positive Expression: MAPK1 --+> F18187046 10.1016/j.plipres.2007.12.003
positive Expression: MAPK1 --+> F12559775 10.1016/S0165-6147(02)00043-3
positive Expression: MAPK1 --+> F17945363 10.1016/j.bbamcr.2007.09.001
positive Expression: MAPK1 --+> PTGS2 10.1016/j.bbamcr.2008.03.016
positive Expression: MAPK1 --+> PTGS2 10.1016/j.bbamcr.2008.03.016
positive Expression: MAPK1 --+> PTGS2 10.1016/j.bbamcr.2008.03.016
positive Expression: MAPK1 --+> F19110010 10.1016/j.bbamcr.2008.11.013
positive Expression: MAPK1 --+> F19748535 10.1016/j.virusres.2009.09.004
positive Expression: MAPK1 --+> F15963597 10.1016/j.jhep.2005.02.033
positive Expression: MAPK1 --+> F15963597 10.1016/j.jhep.2005.02.033
positive Expression: MAPK1 --+> F15963597 10.1016/j.jhep.2005.02.033
positive Expression: MAPK1 --+> F17084486 10.1016/j.neuint.2006.09.016
positive Expression: MAPK1 --+> F19922759 10.1016/j.fct.2009.11.035
positive Expression: MAPK1 --+> F21385599 10.1016/j.fct.2011.03.003
positive Expression: MAPK1 --+> PTGS2 10.1016/j.fct.2012.02.006
positive Expression: MAPK1 --+> F12225989 10.1152/ajpcell.00609.2001
positive Expression: MAPK1 --+> F27217781 10.2147/OTT.S9619710.2147/OTT.S96197
positive Expression: MAPK1 --+> F27795708 10.1155/2016/5616807
positive Expression: MAPK1 --+> F16385238 10.1097/01.alc.0000191809.29775.41
positive Expression: MAPK1 --+> F29867151 10.1038/s41598-018-26473-710.1038/s41598-018-
positive Expression: MAPK1 --+> F12843292
positive Expression: MAPK1 --+> F22001348 10.1016/j.ajpath.2011.08.012
positive Expression: MAPK1 --+> F17097611 10.1016/j.bbrc.2006.10.098
positive Expression: MAPK1 --+> F17189670 10.1016/j.canlet.2006.11.020
positive Expression: MAPK1 --+> F18848748 10.1016/j.canlet.2008.07.039
positive Expression: MAPK1 --+> F21377269 10.1016/j.canlet.2011.02.004
positive Expression: MAPK1 --+> F15733640 10.1016/j.pain.2004.12.022
positive Expression: MAPK1 --+> F19135800 10.1016/j.pain.2008.11.013
positive Expression: MAPK1 --+> PTGS2 10.2353/ajpath.2007.061035
positive Expression: MAPK1 --+> F12559967 10.1016/S0006-291X(02)02937-6
positive Expression: MAPK1 --+> F20096264 10.1016/j.bcp.2010.01.008
positive Expression: MAPK1 --+> F16185670 10.1016/j.brainres.2005.08.033
positive Expression: MAPK1 --+> F15890324 10.1016/j.cellimm.2005.03.001
positive Expression: MAPK1 --+> F16781693 10.1016/j.cellimm.2006.04.003
positive Expression: MAPK1 --+> F17481603 10.1016/j.ejphar.2007.04.003
positive Expression: MAPK1 --+> F20353767 10.1016/j.ejphar.2010.03.021
positive Expression: MAPK1 --+> PTGS2 10.1016/j.yjmcc.2007.06.007
positive Expression: MAPK1 --+> PTGS2 10.1016/j.yjmcc.2007.06.007
positive Expression: MAPK1 --+> PTGS2 10.1016/j.yjmcc.2007.06.007
positive Expression: MAPK1 --+> PTGS2 10.1016/j.yjmcc.2007.06.007
positive Expression: MAPK1 --+> PTGS2 10.1016/j.yjmcc.2007.06.007
positive Expression: MAPK1 --+> F20970453 10.1016/j.plipres.2010.10.003
positive Expression: MAPK1 --+> F17936921 10.1016/j.regpep.2007.05.007
positive Expression: MAPK1 --+> F17945363 10.1016/j.bbamcr.2007.09.001
positive Expression: MAPK1 --+> F17945363 10.1016/j.bbamcr.2007.09.001
positive Expression: MAPK1 --+> F17945363 10.1016/j.bbamcr.2007.09.001
positive Expression: MAPK1 --+> F27539609 10.1007/s12272-016-0805-x

positive Expression: MAPK1 --+> F27462100 10.1189/jlb.3MR0416-171R
positive Expression: MAPK1 --+> F27663890 10.1007/s00204-016-1826-4
positive Expression: MAPK1 --+> F28054591 10.1038/srep3991410.1038/srep39914
positive Expression: MAPK1 --+> PTGS2 10.1016/S0006-2952(03)00488-X
positive Expression: MAPK1 --+> F15781049 10.1016/j.brainres.2005.01.047
positive Expression: MAPK1 --+> F18571585 10.1016/j.surg.2008.03.024
positive Expression: MAPK1 --+> F19748535 10.1016/j.virusres.2009.09.004
positive Expression: MAPK1 --+> F19796664 10.1016/j.fct.2009.09.029
positive Expression: MAPK1 --+> F15212812 10.1016/j.tox.2004.03.014
positive Expression: MAPK1 --+> F20850495 10.1016/j.tox.2010.09.004
positive Expression: MAPK1 --+> F20850495 10.1016/j.tox.2010.09.004
positive Expression: MAPK1 --+> F18771702 10.1016/j.mce.2008.08.004
positive Expression: MAPK1 --+> F17069969 10.1016/j.canlet.2006.09.011
positive Expression: MAPK1 --+> F17950526 10.1016/j.canlet.2007.09.009
positive Expression: MAPK1 --+> F12654342 10.1016/S0306-4522(02)00839-4
positive Expression: MAPK1 --+> F17307294 10.1016/j.neuroscience.2006.12.063
positive Expression: MAPK1 --+> F17307294 10.1016/j.neuroscience.2006.12.063
positive Expression: MAPK1 --+> F19376214 10.1016/j.toxlet.2009.04.007
positive Expression: MAPK1 --+> F19715751 10.1016/j.jep.2009.08.032
positive Expression: MAPK1 --+> F20152896 10.1016/j.tiv.2010.02.008
positive Expression: MAPK1 --+> F20152896 10.1016/j.tiv.2010.02.008
positive Expression: MAPK1 --+> F20633634 10.1016/j.tiv.2010.07.008
positive Expression: MAPK1 --+> F15331118 10.1016/j.bbi.2004.05.003
positive Expression: MAPK1 --+> F15917189 10.1016/j.freeradbiomed.2005.02.023
positive Expression: MAPK1 --+> F15917189 10.1016/j.freeradbiomed.2005.02.023
positive Expression: MAPK1 --+> F15917189 10.1016/j.freeradbiomed.2005.02.023
positive Expression: MAPK1 --+> F15917189 10.1016/j.freeradbiomed.2005.02.023
positive Expression: MAPK1 --+> F20036733 10.1016/j.freeradbiomed.2009.12.014
positive Expression: MAPK1 --+> F20036733 10.1016/j.freeradbiomed.2009.12.014
positive Expression: MAPK1 --+> F22080084 10.1016/j.freeradbiomed.2011.10.481
positive Expression: MAPK1 --+> F12531424 10.1016/S0898-6568(02)00075-X
positive Expression: MAPK1 --+> F15567061 10.1016/j.cellsig.2004.07.008
positive Expression: MAPK1 --+> F15601629 10.1016/j.cellsig.2004.09.017
positive Expression: MAPK1 --+> F15993751 10.1016/j.cellsig.2004.12.002
positive Expression: MAPK1 --+> F17341442 10.1016/j.cellsig.2007.01.028
positive Expression: MAPK1 --+> F17341442 10.1016/j.cellsig.2007.01.028
positive Expression: MAPK1 --+> F17341442 10.1016/j.cellsig.2007.01.028
positive Expression: MAPK1 --+> F17341442 10.1016/j.cellsig.2007.01.028
positive Expression: MAPK1 --+> F18572386 10.1016/j.cellsig.2008.05.008
positive Expression: MAPK1 --+> F19800403 10.1016/j.cellsig.2009.09.018
positive Expression: MAPK1 --+> F19656660 10.1016/j.jdermsci.2009.06.017
positive Expression: MAPK1 --+> F20133117 10.1016/j.etp.2009.11.005
positive Expression: MAPK1 --+> PTGS2 10.1016/j.phymed.2010.02.006
positive Expression: MAPK1 --+> PTGS2 10.1016/j.plefa.2003.04.005
positive Expression: MAPK1 --+> F16446079 10.1016/j.plefa.2005.12.001
positive Expression: MAPK1 --+> F20004562 10.1016/j.plefa.2009.11.005
positive Expression: MAPK1 --+> F20004562 10.1016/j.plefa.2009.11.005
positive Expression: MAPK1 --+> PTGS2 10.1016/j.jnutbio.2011.04.021
positive Expression: MAPK1 --+> F18656337 10.1016/j.jnutbio.2008.02.004
positive Expression: MAPK1 --+> F22071130 10.1016/j.ejca.2011.09.026
positive Expression: MAPK1 --+> F10708932 10.1016/S0959-8049(99)00318-4
positive Expression: MAPK1 --+> PTGS2 10.1016/j.ejcts.2010.12.006
positive Expression: MAPK1 --+> PTGS2 10.1016/j.ejcts.2010.12.006
positive Expression: MAPK1 --+> F15501074 10.1016/j.critrevonc.2004.08.004

positive Expression: MAPK1 --+> F 21095632 10.1016/j.cyto.2010.10.009
positive Expression: MAPK1 --+> F 19717011 10.1016/j.phrs.2009.03.015
positive Expression: MAPK1 --+> F 19782752 10.1016/j.phrs.2009.09.005
positive Expression: MAPK1 --+> PTGS2 10.1016/j.carpath.2004.03.170
positive Expression: MAPK1 --+> F 16516501 10.1016/j.joca.2006.01.006
positive Expression: MAPK1 --+> PTGS2 10.1016/S1072-7515(00)00551-2
positive Expression: MAPK1 --+> F 17085321 10.1016/j.prostaglandins.2006.08.006
positive Expression: MAPK1 --+> F 17085321 10.1016/j.prostaglandins.2006.08.006
positive Expression: MAPK1 --+> F 16713323 10.1016/j.biocel.2006.03.021
positive Expression: MAPK1 --+> F 18571461 10.1016/j.biocel.2008.05.005
positive Expression: MAPK1 --+> F 19457709 10.1016/j.oraloncology.2009.03.016
positive Expression: MAPK1 --+> F 19457709 10.1016/j.oraloncology.2009.03.016
positive Expression: MAPK1 --+> F 11239825 10.1016/S1388-1981(01)00089-0
positive Expression: MAPK1 --+> F 11239825 10.1016/S1388-1981(01)00089-0
positive Expression: MAPK1 --+> F 14984736 10.1016/j.bbalip.2003.12.002
positive Expression: MAPK1 --+> F 15238218 10.1016/j.bbalip.2004.04.003
positive Expression: MAPK1 --+> F 15238218 10.1016/j.bbalip.2004.04.003
positive Expression: MAPK1 --+> F 15238218 10.1016/j.bbalip.2004.04.003
positive Expression: MAPK1 --+> F 15238218 10.1016/j.bbalip.2004.04.003
positive Expression: MAPK1 --+> F 15238218 10.1016/j.bbalip.2004.04.003
positive Expression: MAPK1 --+> F 15238218 10.1016/j.bbalip.2004.04.003
positive Expression: MAPK1 --+> F 20159581 10.1016/j.jbiosc.2009.09.003
positive Expression: MAPK1 --+> F 19168156 10.1016/j.intimp.2008.12.016
positive Expression: MAPK1 --+> F 19168156 10.1016/j.intimp.2008.12.016
positive Expression: MAPK1 --+> F 19962455 10.1016/j.intimp.2009.11.014
positive Expression: MAPK1 --+> F 22974512 10.1016/j.ygcen.2012.08.020
positive Expression: MAPK1 --+> F 22974512 10.1016/j.ygcen.2012.08.020
positive Expression: MAPK1 --+> F 22732298 10.1016/j.bbamcr.2012.06.021
positive Expression: MAPK1 --+> F 22634430 10.1016/j.bbadis.2012.05.013
positive Expression: MAPK1 --+> F 21937212 10.1016/j.jnutbio.2011.04.021
positive Expression: MAPK1 --+> F 22883813 10.1016/j.molmed.2012.06.010
positive Expression: MAPK1 --+> F 22940184 10.1016/j.intimp.2012.08.011
positive Expression: MAPK1 --+> PTGS2 10.1016/j.biomed.2012.06.004
positive Expression: MAPK1 --+> F 23416211 10.1016/j.ejphar.2013.01.061
positive Expression: MAPK1 --+> F 23376159 10.1016/j.ejphar.2013.01.025
positive Expression: MAPK1 --+> F 23340376 10.1016/j.atherosclerosis.2012.12.007
positive Expression: MAPK1 --+> F 23415682 10.1016/j.taap.2013.01.030
positive Expression: MAPK1 --+> F 23415682 10.1016/j.taap.2013.01.030
positive Expression: MAPK1 --+> F 23267746 10.1016/j.vetimm.2012.11.017
positive Expression: MAPK1 --+> F 23348408 10.1016/j.fct.2012.12.062
positive Expression: MAPK1 --+> F 23376640 10.1016/j.canlet.2012.12.030
positive Expression: MAPK1 --+> F 23376640 10.1016/j.canlet.2012.12.030
positive Expression: MAPK1 --+> F 23376640 10.1016/j.canlet.2012.12.030
positive Expression: MAPK1 --+> F 23416140 10.1016/j.toxlet.2013.02.003
positive Expression: MAPK1 --+> F 23295168 10.1016/j.jep.2012.12.034
positive Expression: MAPK1 --+> F 23376440 10.1016/j.tiv.2013.01.012
positive Expression: MAPK1 --+> F 23266471 10.1016/j.cellsig.2012.12.006
positive Expression: MAPK1 --+> F 23102664 10.1016/j.intimp.2012.10.009
positive Expression: MAPK1 --+> F 23562928 10.1016/j.fct.2013.03.029
positive Expression: MAPK1 --+> F 23523690 10.1016/j.bbagen.2013.03.014
positive Expression: MAPK1 --+> F 23523690 10.1016/j.bbagen.2013.03.014
positive Expression: MAPK1 --+> F 23523690 10.1016/j.bbagen.2013.03.014
positive Expression: MAPK1 --+> F 23747596 10.1016/j.ejphar.2013.05.023
positive Expression: MAPK1 --+> F 24239652 10.1016/j.taap.2013.11.002

positive Expression: MAPK1 --+> F24239652 10.1016/j.taap.2013.11.002
positive Expression: MAPK1 --+> F24239652 10.1016/j.taap.2013.11.002
positive Expression: MAPK1 --+> F23796953 10.1016/j.tips.2013.05.003
positive Expression: MAPK1 --+> F23712098 10.1016/j.fct.2013.04.055
positive Expression: MAPK1 --+> F23712098 10.1016/j.fct.2013.04.055
positive Expression: MAPK1 --+> F23968725 10.1016/j.toxlet.2013.08.008
positive Expression: MAPK1 --+> F24036215 10.1016/j.cellsig.2013.09.005
positive Expression: MAPK1 --+> F24530660 10.1016/j.neuro.2014.02.002
positive Expression: MAPK1 --+> F24378735 10.1016/j.mce.2013.12.014
positive Expression: MAPK1 --+> F24424443 10.1016/j.mce.2014.01.007
positive Expression: MAPK1 --+> F24607321 10.1016/j.neuroscience.2014.02.041
positive Expression: MAPK1 --+> F24607321 10.1016/j.neuroscience.2014.02.041
positive Expression: MAPK1 --+> F21262218 10.1016/j.ejphar.2010.12.044
positive Expression: MAPK1 --+> F24852102 10.1016/j.expneurol.2014.05.014
positive Expression: MAPK1 --+> F24852102 10.1016/j.expneurol.2014.05.014
positive Expression: MAPK1 --+> F24742714 10.1016/j.mrfmmm.2014.04.003
positive Expression: MAPK1 --+> F24742714 10.1016/j.mrfmmm.2014.04.003
positive Expression: MAPK1 --+> F24680956 10.1016/j.antiviral.2014.03.009
positive Expression: MAPK1 --+> F24680956 10.1016/j.antiviral.2014.03.009
positive Expression: MAPK1 --+> PTGS2 10.1016/j.bbamcr.2014.11.022
positive Expression: MAPK1 --+> PTGS2 10.1016/j.fct.2014.11.017
positive Expression: MAPK1 --+> F22353212 10.1016/j.fct.2012.02.006
positive Expression: MAPK1 --+> PTGS2 10.1016/j.mce.2014.11.008
positive Expression: MAPK1 --+> F24747121 10.1016/j.canlet.2014.03.037
positive Expression: MAPK1 --+> F24747121 10.1016/j.canlet.2014.03.037
positive Expression: MAPK1 --+> F24747121 10.1016/j.canlet.2014.03.037
positive Expression: MAPK1 --+> F24747121 10.1016/j.canlet.2014.03.037
positive Expression: MAPK1 --+> F24945401 10.1016/j.jep.2014.06.023
positive Expression: MAPK1 --+> F25269780 10.1016/j.cellsig.2014.09.013
positive Expression: MAPK1 --+> PTGS2 10.1016/j.ijid.2014.03.1356
positive Expression: MAPK1 --+> F25172550 10.1016/j.biocel.2014.08.016
positive Expression: MAPK1 --+> F24933588 10.1016/j.intimp.2014.06.002
positive Expression: MAPK1 --+> F25818189 10.1016/j.lfs.2015.03.006
positive Expression: MAPK1 --+> F25461841 10.1016/j.bbamcr.2014.11.022
positive Expression: MAPK1 --+> F25460360 10.1016/j.fct.2014.11.017
positive Expression: MAPK1 --+> F25451977 10.1016/j.mce.2014.11.008
positive Expression: MAPK1 --+> F25917838 10.1016/j.jep.2015.04.022
positive Expression: MAPK1 --+> F26119525 10.1016/j.tiv.2015.06.024
positive Expression: MAPK1 --+> F25643632 10.1016/j.cellsig.2015.01.014
positive Expression: MAPK1 --+> F25643632 10.1016/j.cellsig.2015.01.014
positive Expression: MAPK1 --+> F25681793 10.1016/j.coph.2014.12.011
positive Expression: MAPK1 --+> F25743463 10.1016/j.bpg.2014.12.003
positive Expression: MAPK1 --+> F26110105 10.1016/j.fob.2015.06.001
positive Expression: MAPK1 --+> F19589865
positive Expression: MAPK1 --+> F18723875
positive Expression: MAPK1 --+> F18559923
positive Expression: MAPK1 --+> F18339712
positive Expression: MAPK1 --+> F18339712
positive Expression: MAPK1 --+> F17537778
positive Expression: MAPK1 --+> F17537778
positive Expression: MAPK1 --+> F17478553
positive Expression: MAPK1 --+> F16998585
positive Expression: MAPK1 --+> F16740977
positive Expression: MAPK1 --+> F16574793

positive Expression: MAPK1 --+> F16574793
positive Expression: MAPK1 --+> F16081631
positive Expression: MAPK1 --+> F16081631
positive Expression: MAPK1 --+> F16081631
positive Expression: MAPK1 --+> F16081631
positive Expression: MAPK1 --+> F15784717
positive Expression: MAPK1 --+> F15784717
positive Expression: MAPK1 --+> F15483103
positive Expression: MAPK1 --+> F15483103
positive Expression: MAPK1 --+> F15256495
positive Expression: MAPK1 --+> F12107235
positive Expression: MAPK1 --+> F12107235
positive Expression: MAPK1 --+> F12107235
positive Expression: MAPK1 --+> F12107235
positive Expression: MAPK1 --+> F12107235
positive Expression: MAPK1 --+> F21044318
positive Expression: MAPK1 --+> F20092633
positive Expression: MAPK1 --+> F20018844
positive Expression: MAPK1 --+> F20018844
positive Expression: MAPK1 --+> F19534809
positive Expression: MAPK1 --+> F19534809
positive Expression: MAPK1 --+> F19228814
positive Expression: MAPK1 --+> F19164179
positive Expression: MAPK1 --+> F19037103
positive Expression: MAPK1 --+> F18854308
positive Expression: MAPK1 --+> F18519570
positive Expression: MAPK1 --+> F18208815
positive Expression: MAPK1 --+> F18208815
positive Expression: MAPK1 --+> F18208815
positive Expression: MAPK1 --+> F18208815
positive Expression: MAPK1 --+> F18208815
positive Expression: MAPK1 --+> F18208815
positive Expression: MAPK1 --+> F18208815
positive Expression: MAPK1 --+> F18208815
positive Expression: MAPK1 --+> F17962194
positive Expression: MAPK1 --+> F17962194
positive Expression: MAPK1 --+> F17962194
positive Expression: MAPK1 --+> F17267381
positive Expression: MAPK1 --+> F17267381
positive Expression: MAPK1 --+> F17077316
positive Expression: MAPK1 --+> F16618699
positive Expression: MAPK1 --+> F16467309
positive Expression: MAPK1 --+> F16467309
positive Expression: MAPK1 --+> F16467309
positive Expression: MAPK1 --+> F16371352
positive Expression: MAPK1 --+> F16083499
positive Expression: MAPK1 --+> F15961395
positive Expression: MAPK1 --+> F15961395
positive Expression: MAPK1 --+> F15767259
positive Expression: MAPK1 --+> F15767259
positive Expression: MAPK1 --+> F15525653
positive Expression: MAPK1 --+> F15525649
positive Expression: MAPK1 --+> F15496409
positive Expression: MAPK1 --+> F15375172

positive Expression: MAPK1 --+> F15329330
positive Expression: MAPK1 --+> F15225374
positive Expression: MAPK1 --+> F15007072
positive Expression: MAPK1 --+> F15007072
positive Expression: MAPK1 --+> F15007072
positive Expression: MAPK1 --+> F15007072
positive Expression: MAPK1 --+> F15007072
positive Expression: MAPK1 --+> F15007072
positive Expression: MAPK1 --+> F15001568
positive Expression: MAPK1 --+> F14985247
positive Expression: MAPK1 --+> F14530261
positive Expression: MAPK1 --+> F14530261
positive Expression: MAPK1 --+> F14530261
positive Expression: MAPK1 --+> F12907684
positive Expression: MAPK1 --+> F12907684
positive Expression: MAPK1 --+> F12907684
positive Expression: MAPK1 --+> F12902324
positive Expression: MAPK1 --+> F12902324
positive Expression: MAPK1 --+> F12902324
positive Expression: MAPK1 --+> F12837757
positive Expression: MAPK1 --+> F12826679
positive Expression: MAPK1 --+> F12796504
positive Expression: MAPK1 --+> F12796504
positive Expression: MAPK1 --+> F12796504
positive Expression: MAPK1 --+> F12695520
positive Expression: MAPK1 --+> F12695520
positive Expression: MAPK1 --+> F12695520
positive Expression: MAPK1 --+> F12695520
positive Expression: MAPK1 --+> F12606757
positive Expression: MAPK1 --+> F12588866
positive Expression: MAPK1 --+> F12239223
positive Expression: MAPK1 --+> F12239223
positive Expression: MAPK1 --+> F12239223
positive Expression: MAPK1 --+> F12130685
positive Expression: MAPK1 --+> F12072440
positive Expression: MAPK1 --+> F11901151
positive Expression: MAPK1 --+> F11861647
positive Expression: MAPK1 --+> F11861647
positive Expression: MAPK1 --+> F11861647
positive Expression: MAPK1 --+> F11861647
positive Expression: MAPK1 --+> F11861647
positive Expression: MAPK1 --+> F11861647
positive Expression: MAPK1 --+> F11861647
positive Expression: MAPK1 --+> F11861647
positive Expression: MAPK1 --+> F11861647
positive Expression: MAPK1 --+> F11854442
positive Expression: MAPK1 --+> F11726530
positive Expression: MAPK1 --+> F11292836
positive Expression: MAPK1 --+> F11292836
positive Expression: MAPK1 --+> F11292836
positive Expression: MAPK1 --+> F11292836
positive Expression: MAPK1 --+> F11259631
positive Expression: MAPK1 --+> F11259631
positive Expression: MAPK1 --+> F11207214

positive Expression: MAPK1 --+> F11179444
positive Expression: MAPK1 --+> F10982805
positive Expression: MAPK1 --+> F10969080
positive Expression: MAPK1 --+> F10930430
positive Expression: MAPK1 --+> F10930430
positive Expression: MAPK1 --+> F10930430
positive Expression: MAPK1 --+> F10930401
positive Expression: MAPK1 --+> F10930401
positive Expression: MAPK1 --+> F10722727
positive Expression: MAPK1 --+> F10671533
positive Expression: MAPK1 --+> F10652293
positive Expression: MAPK1 --+> F10652293
positive Expression: MAPK1 --+> F10652293
positive Expression: MAPK1 --+> F10593866
positive Expression: MAPK1 --+> F10593866
positive Expression: MAPK1 --+> F10506169
positive Expression: MAPK1 --+> F10506169
positive Expression: MAPK1 --+> F10066822
positive Expression: MAPK1 --+> F9988750
positive Expression: MAPK1 --+> F9765305
positive Expression: MAPK1 --+> F9705357
positive Expression: MAPK1 --+> F9705357
positive Expression: MAPK1 --+> F9705357
positive Expression: MAPK1 --+> F9705357
positive Expression: MAPK1 --+> F19218449
positive Expression: MAPK1 --+> F17600311
positive Expression: MAPK1 --+> F17502624
positive Expression: MAPK1 --+> F17463321
positive Expression: MAPK1 --+> F16203985
positive Expression: MAPK1 --+> F15699325
positive Expression: MAPK1 --+> F12578976
positive Expression: MAPK1 --+> F12578976
positive Expression: MAPK1 --+> F11853544
positive Expression: MAPK1 --+> F11085935
positive Expression: MAPK1 --+> F11085935
positive Expression: MAPK1 --+> F10785514
positive Expression: MAPK1 --+> F9494074
positive Expression: MAPK1 --+> F9291137
positive Expression: MAPK1 --+> F16614396
positive Expression: MAPK1 --+> F16278684
positive Expression: MAPK1 --+> F16278684
positive Expression: MAPK1 --+> F15623844
positive Expression: MAPK1 --+> F15623844
positive Expression: MAPK1 --+> F15304095
positive Expression: MAPK1 --+> F15304095
positive Expression: MAPK1 --+> F15304095
positive Expression: MAPK1 --+> F15304095
positive Expression: MAPK1 --+> F15304095
positive Expression: MAPK1 --+> F15304095
positive Expression: MAPK1 --+> F15304095
positive Expression: MAPK1 --+> F15304095
positive Expression: MAPK1 --+> F14708615
positive Expression: MAPK1 --+> F14708615
positive Expression: MAPK1 --+> F14708615

positive Expression: MAPK1 --+> F14708615
positive Expression: MAPK1 --+> F14608118
positive Expression: MAPK1 --+> F14520472
positive Expression: MAPK1 --+> F12881420
positive Expression: MAPK1 --+> F12411481
positive Expression: MAPK1 --+> F12411481
positive Expression: MAPK1 --+> F12411481
positive Expression: MAPK1 --+> F12234923
positive Expression: MAPK1 --+> F12028436
positive Expression: MAPK1 --+> F12028436
positive Expression: MAPK1 --+> F18703638
positive Expression: MAPK1 --+> F18703638
positive Expression: MAPK1 --+> F18703638
positive Expression: MAPK1 --+> F18703638
positive Expression: MAPK1 --+> F18703638
positive Expression: MAPK1 --+> F18441204
positive Expression: MAPK1 --+> F17640979
positive Expression: MAPK1 --+> F17640979
positive Expression: MAPK1 --+> F17640979
positive Expression: MAPK1 --+> F17640979
positive Expression: MAPK1 --+> F17591797
positive Expression: MAPK1 --+> F17591797
positive Expression: MAPK1 --+> F17591797
positive Expression: MAPK1 --+> F17308007
positive Expression: MAPK1 --+> F16705145
positive Expression: MAPK1 --+> F16705145
positive Expression: MAPK1 --+> F16113275
positive Expression: MAPK1 --+> F16113275
positive Expression: MAPK1 --+> F15557607
positive Expression: MAPK1 --+> F15557607
positive Expression: MAPK1 --+> F15557607
positive Expression: MAPK1 --+> F15557607
positive Expression: MAPK1 --+> F15557607
positive Expression: MAPK1 --+> F15557607
positive Expression: MAPK1 --+> F15557607
positive Expression: MAPK1 --+> F15557607
positive Expression: MAPK1 --+> F15485931
positive Expression: MAPK1 --+> F15231484
positive Expression: MAPK1 --+> F15231484
positive Expression: MAPK1 --+> F15231484
positive Expression: MAPK1 --+> F15231484
positive Expression: MAPK1 --+> F15231484
positive Expression: MAPK1 --+> F15231484
positive Expression: MAPK1 --+> F15231484
positive Expression: MAPK1 --+> F15231484
positive Expression: MAPK1 --+> F15231484
positive Expression: MAPK1 --+> F15231484
positive Expression: MAPK1 --+> F15117678
positive Expression: MAPK1 --+> F14764449
positive Expression: MAPK1 --+> F14764449
positive Expression: MAPK1 --+> F14764449
positive Expression: MAPK1 --+> F14764449
positive Expression: MAPK1 --+> F12670830
positive Expression: MAPK1 --+> F12670830

positive Expression: MAPK1 --+> F 19290049
positive Expression: MAPK1 --+> F 17329433
positive Expression: MAPK1 --+> F 15213271
positive Expression: MAPK1 --+> F 22818222 10.1186/1742-2094-9-177
positive Expression: MAPK1 --+> F 22818222 10.1186/1742-2094-9-177
positive Expression: MAPK1 --+> F 22818222 10.1186/1742-2094-9-177
positive Expression: MAPK1 --+> F 22766885 10.1194/jlr.D029546
positive Expression: MAPK1 --+> F 22766885 10.1194/jlr.D029546
positive Expression: MAPK1 --+> F 22824956 10.3892/or.2012.1918
positive Expression: MAPK1 --+> F 22941376 10.3892/or.2012.1998
positive Expression: MAPK1 --+> F 22941376 10.3892/or.2012.1998
positive Expression: MAPK1 --+> F 22941376 10.3892/or.2012.1998
positive Expression: MAPK1 --+> F 22941376 10.3892/or.2012.1998
positive Expression: MAPK1 --+> F 22895548 10.3892/mmr.2012.1023
positive Expression: MAPK1 --+> F 22767315 10.3892/ijmm.2012.1052
positive Expression: MAPK1 --+> F 22767315 10.3892/ijmm.2012.1052
positive Expression: MAPK1 --+> F 22879597 10.1074/jbc.M112.385542
positive Expression: MAPK1 --+> F 22747786 10.1186/1742-2094-9-152
positive Expression: MAPK1 --+> F 22571318 10.1186/1743-8977-9-14
positive Expression: MAPK1 --+> F 22571318 10.1186/1743-8977-9-14
positive Expression: MAPK1 --+> F 23184961 10.1074/jbc.M112.424366
positive Expression: MAPK1 --+> F 22688643 10.1007/s10753-012-9488-8
positive Expression: MAPK1 --+> PTGS2
positive Expression: MAPK1 --+> F 23394447 10.1615/JEnvironPatholToxicolOncol.2013005365
positive Expression: MAPK1 --+> PTGS2 10.1248/bpb.33.1307
positive Expression: MAPK1 --+> F 20200402 10.1189/jlb.1009668
positive Expression: MAPK1 --+> F 20558725 10.1074/jbc.M110.135251
positive Expression: MAPK1 --+> F 21175416
positive Expression: MAPK1 --+> F 21141740 10.3727/096504010X12828372551867
positive Expression: MAPK1 --+> F 21156799 10.1074/jbc.M110.192393
positive Expression: MAPK1 --+> F 21828177 10.1210/en.2011-0255
positive Expression: MAPK1 --+> F 21876694 10.1155/2012/782020
positive Expression: MAPK1 --+> F 21862586 10.1074/jbc.M111.260414
positive Expression: MAPK1 --+> F 22210037 10.1007/s12272-011-1214-9
positive Expression: MAPK1 --+> F 22210040 10.1007/s12272-011-1217-6
positive Expression: MAPK1 --+> F 22210040 10.1007/s12272-011-1217-6
positive Expression: MAPK1 --+> F 22210040 10.1007/s12272-011-1217-6
positive Expression: MAPK1 --+> F 22210040 10.1007/s12272-011-1217-6
positive Expression: MAPK1 --+> F 22210040 10.1007/s12272-011-1217-6
positive Expression: MAPK1 --+> F 22181070 10.1089/jmf.2011.1851
positive Expression: MAPK1 --+> F 22181070 10.1089/jmf.2011.1851
positive Expression: MAPK1 --+> F 22181070 10.1089/jmf.2011.1851
positive Expression: MAPK1 --+> F 22393041 10.1074/jbc.M111.308841
positive Expression: MAPK1 --+> F 20405237 10.1007/s12012-010-9072-0
positive Expression: MAPK1 --+> F 20446020 10.1007/s11010-010-0469-7
positive Expression: MAPK1 --+> F 20446020 10.1007/s11010-010-0469-7
positive Expression: MAPK1 --+> F 20446020 10.1007/s11010-010-0469-7
positive Expression: MAPK1 --+> F 20798930 10.1007/s00204-010-0579-8
positive Expression: MAPK1 --+> F 21044318 10.1186/1476-4598-9-288
positive Expression: MAPK1 --+> F 21140284 10.1007/s11010-010-0682-4
positive Expression: MAPK1 --+> F 21569331 10.1186/1423-0127-18-30
positive Expression: MAPK1 --+> F 21810225 10.1186/1743-8977-8-23
positive Expression: MAPK1 --+> F 21810225 10.1186/1743-8977-8-23
positive Expression: MAPK1 --+> F 21169864 10.1097/HJH.0b013e328342b271

positive Expression: MAPK1 --+> F21169864 10.1097/HJH.0b013e328342b271
positive Expression: MAPK1 --+> F21169864 10.1097/HJH.0b013e328342b271
positive Expression: MAPK1 --+> F21169864 10.1097/HJH.0b013e328342b271
positive Expression: MAPK1 --+> F23184385 10.1161/HYPERTENSIONAHA.112.196303
positive Expression: MAPK1 --+> PTGS2 10.1161/CIRCRESAHA.112.280399
positive Expression: MAPK1 --+> PTGS2 10.1161/CIRCRESAHA.112.280399
positive Expression: MAPK1 --+> F23747881 10.2741/4177
positive Expression: MAPK1 --+> F21412256 10.1038/jid.2011.37
positive Expression: MAPK1 --+> F23680829 10.1097/BRS.0b013e31829a6930
positive Expression: MAPK1 --+> F20697426 10.1038/icb.2010.94
positive Expression: MAPK1 --+> F24159347 10.1155/2013/518301
positive Expression: MAPK1 --+> F24174713 10.1155/2013/964012
positive Expression: MAPK1 --+> F24174713 10.1155/2013/964012
positive Expression: MAPK1 --+> F23943857 10.1194/jlr.M042283
positive Expression: MAPK1 --+> F21490228 10.1523/JNEUROSCI.5986-10.2011
positive Expression: MAPK1 --+> F22140508 10.1371/journal.pone.0028065
positive Expression: MAPK1 --+> F23029074 10.1371/journal.pone.0045529
positive Expression: MAPK1 --+> F23155470 10.1371/journal.pone.0049232
positive Expression: MAPK1 --+> F23118554 10.4196/kjpp.2012.16.5.313
positive Expression: MAPK1 --+> F23755184 10.1371/journal.pone.0065150
positive Expression: MAPK1 --+> F23823806 10.1371/journal.pone.0066561
positive Expression: MAPK1 --+> F23977987 10.5483/BMBRep.2013.46.8.237
positive Expression: MAPK1 --+> F24092824 10.1210/jc.2013-2662
positive Expression: MAPK1 --+> F24092824 10.1210/jc.2013-2662
positive Expression: MAPK1 --+> F24092824 10.1210/jc.2013-2662
positive Expression: MAPK1 --+> F24092824 10.1210/jc.2013-2662
positive Expression: MAPK1 --+> F24092824 10.1210/jc.2013-2662
positive Expression: MAPK1 --+> F24092824 10.1210/jc.2013-2662
positive Expression: MAPK1 --+> F24092824 10.1210/jc.2013-2662
positive Expression: MAPK1 --+> F24092824 10.1210/jc.2013-2662
positive Expression: MAPK1 --+> F24092824 10.1210/jc.2013-2662
positive Expression: MAPK1 --+> F24025354 10.4161/cbt.26427
positive Expression: MAPK1 --+> F23836924 10.1210/me.2013-1130
positive Expression: MAPK1 --+> F24533777 10.2174/1389450115666140217152042
positive Expression: MAPK1 --+> F24569980 10.2478/s11658-014-0186-4
positive Expression: MAPK1 --+> PTGS2 10.1074/jbc.M113.517581
positive Expression: MAPK1 --+> F23728342 10.1038/onc.2013.184
positive Expression: MAPK1 --+> F23728342 10.1038/onc.2013.184
positive Expression: MAPK1 --+> F23728342 10.1038/onc.2013.184
positive Expression: MAPK1 --+> F24738717 10.1089/jmf.2013.2936
positive Expression: MAPK1 --+> F24888317 10.3892/ijmm.2014.1790
positive Expression: MAPK1 --+> F25177723 10.1155/2014/397430
positive Expression: MAPK1 --+> F25180251 10.1530/JME-14-0060
positive Expression: MAPK1 --+> F25180251 10.1530/JME-14-0060
positive Expression: MAPK1 --+> F25319133 10.3892/ijmm.2014.1967
positive Expression: MAPK1 --+> F25319133 10.3892/ijmm.2014.1967
positive Expression: MAPK1 --+> PTGS2 10.1097/MAJ.0000000000000335
positive Expression: MAPK1 --+> PTGS2
positive Expression: MAPK1 --+> F25435376 10.3892/ijmm.2014.2014
positive Expression: MAPK1 --+> PTGS2 10.3233/JAD-141635
positive Expression: MAPK1 --+> PTGS2 10.1179/1743132814Y.0000000426
positive Expression: MAPK1 --+> F21873449 10.1152/ajplung.00092.2011
positive Expression: MAPK1 --+> F21937604 10.1152/ajprenal.00590.2010
positive Expression: MAPK1 --+> F22361730 10.1152/ajpgi.00427.2011

positive Expression: MAPK1 --+> P	24226522	10.1152/ajprenal.00352.2013
positive Expression: MAPK1 --+> P	24872319	10.1152/ajprenal.00634.2013
positive Expression: MAPK1 --+> P	22613973	10.1159/000171028
positive Expression: MAPK1 --+> P	22613973	10.1159/000171028
positive Expression: MAPK1 --+> P	22613973	10.1159/000171028
positive Expression: MAPK1 --+> P	25738262	10.3892/ijmm.2015.2120
positive Expression: MAPK1 --+> P	22095018	10.1159/000331513
positive Expression: MAPK1 --+> P	23382072	10.1128/MCB.01690-12
positive Expression: MAPK1 --+> P	23899566	10.1152/physrev.00004.2012
positive Expression: MAPK1 --+> P	21071999	10.1159/000321327
positive Expression: MAPK1 --+> P	21071999	10.1159/000321327
positive Expression: MAPK1 --+> P	21071999	10.1159/000321327
positive Expression: MAPK1 --+> P	21071999	10.1159/000321327
positive Expression: MAPK1 --+> P	26050653	
positive Expression: MAPK1 --+> PTGS2		10.1530/REP-14-0392
positive Expression: MAPK1 --+> P	25936351	10.3892/ijmm.2015.2195
positive Expression: MAPK1 --+> P	25783839	10.1177/1753425915576345
positive Expression: MAPK1 --+> P	9753607	
positive Expression: MAPK1 --+> P	9830045	
positive Expression: MAPK1 --+> P	10453032	
positive Expression: MAPK1 --+> P	10506169	
positive Expression: MAPK1 --+> P	10652293	
positive Expression: MAPK1 --+> P	10785514	
positive Expression: MAPK1 --+> P	11228545	
positive Expression: MAPK1 --+> P	11179444	
positive Expression: MAPK1 --+> P	11292836	
positive Expression: MAPK1 --+> P	11756226	
positive Expression: MAPK1 --+> P	11811557	
positive Expression: MAPK1 --+> P	11854442	
positive Expression: MAPK1 --+> P	11992399	
positive Expression: MAPK1 --+> P	12096032	
positive Expression: MAPK1 --+> P	12107235	
positive Expression: MAPK1 --+> P	12176102	
positive Expression: MAPK1 --+> P	12377986	
positive Expression: MAPK1 --+> P	12444157	
positive Expression: MAPK1 --+> P	12930301	
positive Expression: MAPK1 --+> P	12837757	
positive Expression: MAPK1 --+> P	14584892	
positive Expression: MAPK1 --+> P	14708615	
positive Expression: MAPK1 --+> P	14984736	
positive Expression: MAPK1 --+> P	15106733	
positive Expression: MAPK1 --+> P	15007072	
positive Expression: MAPK1 --+> P	15238218	
positive Expression: MAPK1 --+> P	15238218	
positive Expression: MAPK1 --+> P	15117678	
positive Expression: MAPK1 --+> P	15470067	
positive Expression: MAPK1 --+> P	15546477	
positive Expression: MAPK1 --+> P	15557607	
positive Expression: MAPK1 --+> P	15623844	
positive Expression: MAPK1 --+> P	15703831	
positive Expression: MAPK1 --+> P	15515144	
positive Expression: MAPK1 --+> P	16088958	
positive Expression: MAPK1 --+> P	16275389	
positive Expression: MAPK1 --+> P	16446079	

positive Expression: MAPK1 --+> F 17047069
positive Expression: MAPK1 --+> F 17222389
positive Expression: MAPK1 --+> F 17307294
positive Expression: MAPK1 --+> F 17267381
positive Expression: MAPK1 --+> F 17189670
positive Expression: MAPK1 --+> F 17463321
positive Expression: MAPK1 --+> F 17341442
positive Expression: MAPK1 --+> F 17640979
positive Expression: MAPK1 --+> F 18162716
positive Expression: MAPK1 --+> F 18162716
positive Expression: MAPK1 --+> F 17964630
positive Expression: MAPK1 --+> F 18285354 10.1093/rheumatology/kem376
positive Expression: MAPK1 --+> F 18208815 10.1074/jbc.M703318200
positive Expression: MAPK1 --+> F 18208815 10.1074/jbc.M703318200
positive Expression: MAPK1 --+> F 18339712 10.1210/en.2007-1633
positive Expression: MAPK1 --+> F 18452714 10.1016/j.bbamcr.2008.03.016
positive Expression: MAPK1 --+> F 18571585 10.1016/j.surg.2008.03.024
positive Expression: MAPK1 --+> F 18566751 10.1007/s11010-008-9843-0
positive Expression: MAPK1 --+> F 18703638 10.1152/ajpgi.90423.2008
positive Expression: MAPK1 --+> F 18958425 10.1007/s12272-001-2114-1
positive Expression: MAPK1 --+> F 18958425 10.1007/s12272-001-2114-1
positive Expression: MAPK1 --+> F 19116446
positive Expression: MAPK1 --+> F 19118901 10.1016/j.molimm.2008.11.006
positive Expression: MAPK1 --+> F 19584262 10.1158/1541-7786.MCR-08-0493
positive Expression: MAPK1 --+> F 19758248 10.1111/j.1600-0722.2009.00669.x
positive Expression: MAPK1 --+> F 20121928 10.1111/j.1600-0722.2009.00682.x
positive Expression: MAPK1 --+> F 20121928 10.1111/j.1600-0722.2009.00682.x
positive Expression: MAPK1 --+> F 19781629 10.1016/j.cellsig.2009.09.014
positive Expression: MAPK1 --+> F 19781629 10.1016/j.cellsig.2009.09.014
positive Expression: MAPK1 --+> F 20386571 10.1038/cr.2010.49
positive Expression: MAPK1 --+> F 20883667 10.1016/j.bbrc.2010.09.095
positive Expression: MAPK1 --+> F 20883667 10.1016/j.bbrc.2010.09.095
positive Expression: MAPK1 --+> F 20883667 10.1016/j.bbrc.2010.09.095
positive Expression: MAPK1 --+> F 20883667 10.1016/j.bbrc.2010.09.095
positive Expression: MAPK1 --+> F 21071999 10.1159/000321327
positive Expression: MAPK1 --+> F 20853849 10.1021/bi100645m
positive Expression: MAPK1 --+> F 21264892 10.1002/cbf.1720
positive Expression: MAPK1 --+> F 21169864 10.1097/HJH.0b013e328342b271
positive Expression: MAPK1 --+> F 21419296 10.1016/j.joen.2011.01.012
positive Expression: MAPK1 --+> F 21311042 10.1161/ATVBAHA.110.216044
positive Expression: MAPK1 --+> F 21385599 10.1016/j.fct.2011.03.003
positive Expression: MAPK1 --+> F 21470077 10.3109/15376516.2011.568982
positive Expression: MAPK1 --+> F 21790943 10.1111/j.1462-5822.2011.01650.x
positive Expression: MAPK1 --+> F 21428715 10.3109/08923973.2011.557383
positive Expression: MAPK1 --+> F 21428715 10.3109/08923973.2011.557383
positive Expression: MAPK1 --+> F 21678415 10.1002/jcp.22880
positive Expression: MAPK1 --+> F 22181070 10.1089/jmf.2011.1851
positive Expression: MAPK1 --+> F 22613973 10.1159/000171028
positive Expression: MAPK1 --+> F 22941376 10.3892/or.2012.1998
positive Expression: MAPK1 --+> F 22974838 10.1016/j.fct.2012.08.041
positive Expression: MAPK1 --+> F 23415682 10.1016/j.taap.2013.01.030
positive Expression: MAPK1 --+> F 23376440 10.1016/j.tiv.2013.01.012
positive Expression: MAPK1 --+> F 23416211 10.1016/j.ejphar.2013.01.061
positive Expression: MAPK1 --+> F 24092824 10.1210/jc.2013-2662

positive Expression: MAPK1 --+> F 23728342 10.1038/onc.2013.184
positive Expression: MAPK1 --+> F 24680956 10.1016/j.antiviral.2014.03.009
positive Expression: MAPK1 --+> F 24680956 10.1016/j.antiviral.2014.03.009
positive Expression: MAPK1 --+> F 25399887 10.1111/1440-1681.12339
positive Expression: MAPK1 --+> F 25399887 10.1111/1440-1681.12339
positive Expression: MAPK1 --+> F 25596430 10.1016/j.taap.2015.01.005
positive Expression: MAPK1 --+> F 25503726 10.1152/ajprenal.00206.2014
positive Expression: MAPK1 --+> F 25783937 10.1186/s12906-015-0583-x
positive Expression: MAPK1 --+> F 25811799 10.1038/cddis.2015.69
positive Expression: MAPK1 --+> F 25888188 10.1186/s12906-015-0583-x
positive Expression: MAPK1 --+> F 25890183 10.1186/s12985-015-0264-x
positive Expression: MAPK1 --+> PTGS2
positive Expression: MAPK1 --+> F 26658076 10.1371/journal.pone.0143663
positive Expression: MAPK1 --+> F 26658076 10.1371/journal.pone.0143663
positive Expression: MAPK1 --+> F 26853158 10.1016/j.semcancer.2016.02.001
positive Expression: MAPK1 --+> F 26466383 10.1371/journal.pone.0129244
positive Expression: MAPK1 --+> F 26466383 10.1371/journal.pone.0129244
positive Expression: MAPK1 --+> F 26466383 10.1371/journal.pone.0129244
positive Expression: MAPK1 --+> F 26658076 10.1371/journal.pone.014366310.1371/journal.p
positive Expression: MAPK1 --+> PTGS2 10.3233/RNN-150559
positive Expression: MAPK1 --+> F 26608166 10.4014/jmb.1510.10070
positive Expression: MAPK1 --+> F 26608166 10.4014/jmb.1510.10070
positive Expression: MAPK1 --+> F 27313009 10.1038/srep2799510.1038/srep27995
positive Expression: MAPK1 --+> F 27548147 10.3390/ijms17081359
positive Expression: MAPK1 --+> F 27548147 10.3390/ijms17081359
positive Expression: MAPK1 --+> F 27664170 10.1093/mmy/myw082
positive Expression: MAPK1 --+> F 27664170 10.1093/mmy/myw082
positive Expression: MAPK1 --+> PTGS2
positive Expression: MAPK1 --+> PTGS2
positive Expression: MAPK1 --+> PTGS2
positive Expression: MAPK1 --+> F 28219441 10.1186/s12974-017-0795-8
positive Expression: MAPK1 --+> F 28288820 10.1016/j.bcp.2017.03.003
positive Expression: MAPK1 --+> F 28288820 10.1016/j.bcp.2017.03.003
positive Expression: MAPK1 --+> F 28245546 10.3390/molecules22030334
positive Expression: MAPK1 --+> F 27814606 10.2741/4476
positive Expression: MAPK1 --+> F 27324043 10.1007/s12013-016-0744-7
positive Expression: MAPK1 --+> F 28325144 10.2174/1389450118666170321105425
positive Expression: MAPK1 --+> F 29038542 10.1038/s41598-017-12648-110.1038/s41598-017-
positive Expression: MAPK1 --+> F 29274705 10.1016/j.pharmthera.2017.12.008
positive Expression: MAPK1 --+> F 29416441 10.1186/s12935-018-0511-5
positive Expression: MAPK1 --+> F 29463003 10.3390/ijms19020599
positive Expression: MAPK1 --+> F 29737477 10.1007/s10753-018-0805-810.1007/s10753-018-0
positive Expression: MAPK1 --+> F 29594389 10.1007/s00018-018-2805-0
positive Expression: MAPK1 --+> F 30119635 10.1186/s13046-018-0839-7
positive Expression: MAPK1 --+> PTGS2 10.1080/01616412.2019.1580459
positive Expression: MAPK1 --+> F 26794660 10.1038/cddis.2015.399
positive Expression: MAPK1 --+> F 31811862 10.1016/j.cbi.2019.10890310.1016/j.cbi.2019.1
positive Expression: MAPK1 --+> F 31811862 10.1016/j.cbi.2019.10890310.1016/j.cbi.2019.1
positive Expression: MAPK1 --+> PTGS2 10.26355/eurrev_201911_19587
positive Expression: MAPK1 --+> F 30773096 10.1177/1933719119828115
positive Expression: MAPK1 --+> PTGS2 10.7150/ijbs.35128
positive Expression: MAPK1 --+> F 32224280 10.1016/j.fsi.2020.03.05610.1016/j.fsi.2020.0
positive Expression: MAPK1 --+> F 32303181 10.1186/s12865-020-00347-y
positive Expression: MAPK1 --+> F 32303181 10.1186/s12865-020-00347-y

positive Expression: MAPK1 --+> P 32303181 10.1186/s12865-020-00347-y
positive Expression: MAPK1 --+> P 32303181 10.1186/s12865-020-00347-y
positive Expression: MAPK1 --+> PTGS2 10.1177/0003319719896472
positive Expression: MAPK1 --+> PTGS2 10.2174/1568009620666200101094736
positive Expression: MAPK1 --+> P 33198771 10.1186/s12974-020-02023-9
positive Expression: MAPK1 --+> P 33505120 10.1007/s10616-020-00449-w
positive Expression: MAPK1 --+> P 33527851 10.1177/0960327120949619
positive Expression: MAPK1 --+> P 33527851 10.1177/0960327120949619
positive Expression: MAPK1 --+> P 33011728 10.1159/000511280
positive Expression: MAPK1 --+> P 32981501 10.2174/1389450121666200925150022
positive Expression: MAPK1 --+> P 33991355 10.1111/pim.1286810.1111/pim.12868
positive Expression: MAPK1 --+> P 33790105 10.1248/BPB.B20-00889
positive Expression: MAPK1 --+> P 34078818 10.1248/bpb.b21-00078
positive Expression: MAPK1 --+> P 34078818 10.1248/bpb.b21-00078
positive Expression: MAPK1 --+> P 34078818 10.1248/bpb.b21-00078
positive Expression: MAPK1 --+> P 34031706 10.1007/s00011-021-01471-0
positive Expression: MAPK1 --+> P 34198981 10.3390/ijms22136894
positive Expression: MAPK1 --+> P 33081671 10.2174/1389450121666201020155010
positive Expression: MAPK1 --+> P 34824589 10.1155/2021/5319584
positive Expression: MAPK1 --+> P 35130451 10.1128/iai.00672-21
positive Expression: MAPK1 --+> P 35382723 10.2174/1568026622666220405143003
positive Expression: MAPK1 --+> PTGS2 10.5483/BMBRep.2022.55.9.061
positive Expression: MAPK1 --+> P 36268909 10.1515/hsz-2022-0218
positive Expression: MAPK1 --+> P 36598592 10.1007/s10753-022-01778-y
positive Regulation: PTGS2 --+> M 29229533 10.1016/j.ejphar.2017.11.045
positive Regulation: PTGS2 --+> M 10969080 10.1074/jbc.M006168200
positive Regulation: PTGS2 --+> M 30679074 10.1016/j.ijcard.2018.12.077
positive Regulation: PTGS2 --+> M 33603542 10.1016/j.jsps.2020.12.011
positive Regulation: PTGS2 --+> M 33221682 10.1016/j.tranon.2020.100940
positive Regulation: PTGS2 --+> M 11891194 10.1016/S0002-9440(10)64918-8
positive Regulation: PTGS2 --+> M 18549804 10.1016/j.ab.2008.05.026
positive Regulation: PTGS2 --+> M 22120676 10.1016/j.bcp.2011.11.008
positive Regulation: PTGS2 --+> M 21565184 10.1016/j.yexmp.2011.04.014
positive Regulation: PTGS2 --+> M 17382352 10.1016/j.lfs.2007.02.008
positive Regulation: PTGS2 --+> M 16446079 10.1016/j.plefa.2005.12.001
positive Regulation: PTGS2 --+> M 20470889 10.1016/j.nbd.2010.05.005
positive Regulation: PTGS2 --+> M 17085321 10.1016/j.prostaglandins.2006.08.006
positive Regulation: PTGS2 --+> M 23810444 10.1016/j.intimp.2013.06.008
positive Regulation: PTGS2 --+> M 24975097 10.1016/j.ejphar.2014.06.027
positive Regulation: PTGS2 --+> MAPK1 10.1016/S1556-0864(16)30013-2
positive Regulation: PTGS2 --+> M 16740977
positive Regulation: PTGS2 --+> M 10969080
positive Regulation: PTGS2 --+> M 9291137
positive Regulation: PTGS2 --+> M 16396944
positive Regulation: PTGS2 --+> M 16396944
positive Regulation: PTGS2 --+> M 27301644 10.1186/s12974-016-0615-6
positive Regulation: PTGS2 --+> M 29156550 10.3390/ijms18112449
positive Regulation: PTGS2 --+> M 29976894 10.3390/ijms19071966
positive Regulation: PTGS2 --+> M 29976894 10.3390/ijms19071966
positive Regulation: PTGS2 --+> M 30265377 10.1002/JLB.3HI0518-193R
positive Regulation: PTGS2 --+> M 28800785 10.3727/096504017X15021536183517
positive Regulation: PTGS2 --+> M 32962418 10.1177/1759091420944658
positive Regulation: PTGS2 --+> M 33221682 10.1016/j.tranon.2020.100940
positive Regulation: PTGS2 --+> M 33383887 10.3390/ijms22010265

positive Regulation: PTGS2 --+> M 22879597 10.1074/jbc.M112.385542
positive Regulation: PTGS2 --+> M 21737546 10.1681/ASN.2010111149
positive Regulation: PTGS2 --+> M 25785965 10.1371/journal.pone.0119416
positive Regulation: PTGS2 --+> M 26500452 10.1186/s12935-015-0255-4
positive Regulation: PTGS2 --+> M 26884798 10.1155/2016/8981650
positive Regulation: PTGS2 --+> M 11891194 10.1016/S0002-9440(10)64918-8
positive Regulation: PTGS2 --+> M 16396944 10.1152/ajprenal.00267.2005
positive Regulation: PTGS2 --+> M 17318410 10.1007/s11010-007-9411-z
positive Regulation: PTGS2 --+> M 19584262 10.1158/1541-7786.MCR-08-049310.1158/1541-778
positive Regulation: PTGS2 --+> M 19584262 10.1158/1541-7786.MCR-08-049310.1158/1541-778
positive Regulation: PTGS2 --+> M 19584262 10.1158/1541-7786.MCR-08-049310.1158/1541-778
positive Regulation: PTGS2 --+> M 19584262 10.1158/1541-7786.MCR-08-049310.1158/1541-778
positive Regulation: PTGS2 --+> M 19671676 10.1158/1541-7786.MCR-09-000410.1158/1541-778
positive Regulation: PTGS2 --+> M 19671676 10.1158/1541-7786.MCR-09-000410.1158/1541-778
positive Regulation: PTGS2 --+> M 20035047 10.2353/ajpath.2010.09022210.2353/ajpath.2010
positive Regulation: PTGS2 --+> M 21807123 10.1016/j.intimp.2011.07.01110.1016/j.intimp.
positive Regulation: PTGS2 --+> M 25238253 10.1039/c4fo00612g10.1039/c4fo00612g
positive Regulation: PTGS2 --+> M 26418808 10.1002/jbt.2176910.1002/jbt.21769
positive Regulation: PTGS2 --+> M 26428849 10.1016/j.intimp.2015.09.01810.1016/j.intimp.
positive Regulation: PTGS2 --+> M 27819025 10.1155/2016/9351787
positive Regulation: PTGS2 --+> M 27819025 10.1155/2016/9351787
positive Regulation: PTGS2 --+> MAPK1 10.3390/ijms221910250
positive Regulation: PTGS2 --+> MAPK1 10.1016/j.ijcard.2018.12.077
positive Regulation: PTGS2 --+> MAPK1 10.1002/JLB.3HI0518-193R
positive Regulation: PTGS2 --+> MAPK1 10.1016/j.jsps.2020.12.011
positive Regulation: PTGS2 --+> M 35850845 10.19540/j.cnki.cjcmm.20220307.702
positive MolTransport: MAPK1 --+> 27174766 10.1016/j.taap.2016.05.003
positive MolTransport: MAPK1 --+> 15567061 10.1016/j.cellsig.2004.07.008
positive MolTransport: MAPK1 --+> 19450684 10.1016/j.phrs.2009.05.001
positive MolTransport: MAPK1 --+> 26410343 10.1016/j.jnutbio.2015.08.026
positive MolTransport: MAPK1 --+> 17537778
positive MolTransport: MAPK1 --+> 15914468
positive MolTransport: MAPK1 --+> 28333094 10.3390/molecules22040506
positive MolTransport: MAPK1 --+> 31999508 10.1152/physrev.00014.2019
positive MolTransport: MAPK1 --+> 23389111 10.1152/ajpregu.00355.2012
positive MolTransport: MAPK1 --+> 17537778 10.1183/09031936.00008707
positive MolTransport: MAPK1 --+> 17097611 10.1016/j.bbrc.2006.10.098
positive MolTransport: MAPK1 --+> 16275389 10.1016/j.jaci.2005.08.014
positive MolTransport: MAPK1 --+> 17537778
positive MolTransport: MAPK1 --+> 17537778
positive MolTransport: MAPK1 --+> 11207214
positive MolTransport: MAPK1 --+> 11207214
positive MolTransport: MAPK1 --+> 15567061
MolTransport: NGF ---> PTGS2 28673205 10.1177/1933719117716778
MolTransport: NGF ---> PTGS2 28673205 10.1177/1933719117716778
MolTransport: NGF ---> PTGS2 28673205 10.1177/1933719117716778
negative PromoterBinding: TP53 -- 24413150 10.1038/cdd.2013.186
negative PromoterBinding: TP53 -- 33512431 10.1182/blood.202000745210.1182/blood.2020007
negative PromoterBinding: TP53 -- 33512431 10.1182/blood.202000745210.1182/blood.2020007
negative PromoterBinding: TP53 -- 24241898 10.1007/s13277-013-1318-610.1007/s13277-013-1
negative PromoterBinding: TP53 -- 18208354 10.1515/BC.2008.03910.1515/BC.2008.039
negative PromoterBinding: TP53 -- 18208354 10.1515/BC.2008.03910.1515/BC.2008.039
negative PromoterBinding: TP53 -- 20739623 10.1152/ajpcell.00080.2010
negative PromoterBinding: TP53 -- 24206455 10.1089/ars.2013.5718

negative PromoterBinding: TP53 --24413150 10.1038/cdd.2013.186
negative PromoterBinding: TP53 --23608884 10.1038/leu.2013.125
negative PromoterBinding: TP53 --23608884 10.1038/leu.2013.125
negative PromoterBinding: TP53 --23608884 10.1038/leu.2013.125
negative PromoterBinding: TP53 --23608884 10.1038/leu.2013.125
negative PromoterBinding: TP53 --21896753 10.1073/pnas.1019312108
negative PromoterBinding: TP53 --18208354 10.1515/BC.2008.039
negative PromoterBinding: TP53 ---| MCL1
negative PromoterBinding: TP53 --29301217 10.3390/ijms19010107
negative PromoterBinding: TP53 --29976747 10.3324/haematol.2018.191650
negative PromoterBinding: TP53 --30097848 10.1007/s12035-018-1277-4
negative PromoterBinding: TP53 --30097848 10.1007/s12035-018-1277-4
negative PromoterBinding: TP53 --34700366 10.1515/hsz-2021-0288
negative PromoterBinding: TP53 --35598030 10.1186/s40164-022-00283-010.1186/s40164-022-
negative PromoterBinding: TP53 --35444234 10.1038/s41418-022-00999-w
negative PromoterBinding: TP53 --35668109 10.1038/s41375-022-01619-9
Regulation: PTGS2 ---> MAPK1 11891194 10.1016/S0002-9440(10)64918-8
Regulation: PTGS2 ---> MAPK1 18549804 10.1016/j.ab.2008.05.026
Regulation: PTGS2 ---> MAPK1 22120676 10.1016/j.bcp.2011.11.008
Regulation: PTGS2 ---> MAPK1 21565184 10.1016/j.yexmp.2011.04.014
Regulation: PTGS2 ---> MAPK1 17382352 10.1016/j.lfs.2007.02.008
Regulation: PTGS2 ---> MAPK1 20470889 10.1016/j.nbd.2010.05.005
Regulation: PTGS2 ---> MAPK1 17085321 10.1016/j.prostaglandins.2006.08.006
Regulation: PTGS2 ---> MAPK1 10.1067/mai.2002.129705
Regulation: PTGS2 ---> MAPK1 23810444 10.1016/j.intimp.2013.06.008
Regulation: PTGS2 ---> MAPK1 24975097 10.1016/j.ejphar.2014.06.027
Regulation: PTGS2 ---> MAPK1 10.1016/S1556-0864(16)30013-2
Regulation: PTGS2 ---> MAPK1 10969080
Regulation: PTGS2 ---> MAPK1 9291137
Regulation: PTGS2 ---> MAPK1 16396944
Regulation: PTGS2 ---> MAPK1 16396944
Regulation: PTGS2 ---> MAPK1 27301644 10.1186/s12974-016-0615-6
Regulation: PTGS2 ---> MAPK1 29156550 10.3390/ijms18112449
Regulation: PTGS2 ---> MAPK1 22879597 10.1074/jbc.M112.385542
Regulation: PTGS2 ---> MAPK1 20597043 10.1055/s-0030-1250074
Regulation: PTGS2 ---> MAPK1 21737546 10.1681/ASN.2010111149
Regulation: PTGS2 ---> MAPK1 25785965 10.1371/journal.pone.0119416
Regulation: PTGS2 ---> MAPK1 26884798 10.1155/2016/8981650
Regulation: PTGS2 ---> MAPK1 11891194 10.1016/S0002-9440(10)64918-8
Regulation: PTGS2 ---> MAPK1 16396944 10.1152/ajprenal.00267.2005
Regulation: PTGS2 ---> MAPK1 17318410 10.1007/s11010-007-9411-z
Regulation: PTGS2 ---> MAPK1 19584262 10.1158/1541-7786.MCR-08-049310.1158/1541-778
Regulation: PTGS2 ---> MAPK1 19584262 10.1158/1541-7786.MCR-08-049310.1158/1541-778
Regulation: PTGS2 ---> MAPK1 19584262 10.1158/1541-7786.MCR-08-049310.1158/1541-778
Regulation: PTGS2 ---> MAPK1 19584262 10.1158/1541-7786.MCR-08-049310.1158/1541-778
Regulation: PTGS2 ---> MAPK1 19671676 10.1158/1541-7786.MCR-09-000410.1158/1541-778
Regulation: PTGS2 ---> MAPK1 19671676 10.1158/1541-7786.MCR-09-000410.1158/1541-778
Regulation: PTGS2 ---> MAPK1 20035047 10.2353/ajpath.2010.09022210.2353/ajpath.2010
Regulation: PTGS2 ---> MAPK1 26418808 10.1002/jbt.2176910.1002/jbt.21769
Regulation: PTGS2 ---> MAPK1 27819025 10.1155/2016/9351787
Regulation: PTGS2 ---> MAPK1 27819025 10.1155/2016/9351787
Regulation: PTGS2 ---> MAPK1 12604350 10.1016/S0006-291X(03)00187-6
Regulation: PTGS2 ---> MAPK1 19008312
Regulation: PTGS2 ---> MAPK1 16624933

Regulation: PTGS2 ---> MAPK1 16467309
Regulation: PTGS2 ---> MAPK1 14530261
Regulation: PTGS2 ---> MAPK1 10671533
Regulation: PTGS2 ---> MAPK1 25810437 10.1152/ajprenal.00514.2014
Regulation: PTGS2 ---> MAPK1 12604350
Regulation: PTGS2 ---> MAPK1 15753395
Regulation: PTGS2 ---> MAPK1 16376453
Regulation: PTGS2 ---> MAPK1 17201164
Regulation: PTGS2 ---> MAPK1 18424914
Regulation: PTGS2 ---> MAPK1 25810437 10.1152/ajprenal.00514.2014
Regulation: PTGS2 ---> MAPK1 10.1002/JLB.3HI0518-193R
positive Expression: GPER1 --+> E31949720
positive Regulation: SFRP1 --+> T14581477 10.1074/jbc.M308102200
positive Regulation: SFRP1 --+> T14581477 10.1074/jbc.M308102200
positive Regulation: SFRP1 --+> T14581477
positive Regulation: SFRP1 --+> T14581477
positive Regulation: SFRP1 --+> TP53
Binding: MCL1 ---- TP53 20404322 10.1074/jbc.M109.081042
Binding: MCL1 ---- TP53 20404322 10.1074/jbc.M109.081042
Binding: MCL1 ---- TP53 20404322 10.1074/jbc.M109.081042
Binding: MCL1 ---- TP53 20404322 10.1074/jbc.M109.081042
Binding: MCL1 ---- TP53 27818144 10.1016/j.molcel.2016.10.002
Binding: MCL1 ---- TP53 27818144 10.1016/j.molcel.2016.10.002
Binding: MCL1 ---- TP53 15865941 10.1016/j.bbrc.2005.03.189
Binding: MCL1 ---- TP53 16376985 10.1016/j.placenta.2005.11.004
Binding: MCL1 ---- TP53 18039530 10.1016/j.intimp.2007.07.013
Binding: MCL1 ---- TP53 22705006 10.1053/j.gastro.2012.06.004
Binding: MCL1 ---- TP53 27465322 10.1007/s11596-016-1614-7
Binding: MCL1 ---- TP53 27465322 10.1007/s11596-016-1614-7
Binding: MCL1 ---- TP53 33315226 10.1007/978-1-0716-1154-8_13
Binding: MCL1 ---- TP53 20885891 10.1593/tlo.10124
Binding: MCL1 ---- TP53 21325876 10.4161/cbt.11.8.15059
Binding: MCL1 ---- TP53 23946421 10.1073/pnas.1303002110
Binding: MCL1 ---- TP53 22451485 10.1128/MCB.06214-11
Binding: MCL1 ---- TP53 20404322 10.1074/jbc.M109.08104210.1074/jbc.M109.08104
Binding: MCL1 ---- TP53 897 10.1016/j.molcel.2016.10.002
Binding: MCL1 ---- TP53 897 10.1016/j.molcel.2016.10.002
Binding: MCL1 ---- TP53 20885891 10.1593/tlo.10124
Binding: MCL1 ---- TP53 23977882 10.1021/bi400690m10.1021/bi400690m
positive Regulation: NGF --+> RAC15629443 10.1016/j.bbrc.2004.11.151
positive Regulation: NGF --+> RAC17320046 10.1016/j.bbrc.2007.02.028
positive Regulation: NGF --+> RAC15302586 10.1016/j.yexcr.2004.05.010
positive Regulation: NGF --+> RAC15302586 10.1016/j.yexcr.2004.05.010
positive Regulation: NGF --+> RAC15302586 10.1016/j.yexcr.2004.05.010
positive Regulation: NGF --+> RAC15302586 10.1016/j.yexcr.2004.05.010
positive Regulation: NGF --+> RAC15890337 10.1016/j.yexcr.2005.02.029
positive Regulation: NGF --+> RAC15890337 10.1016/j.yexcr.2005.02.029
positive Regulation: NGF --+> RAC18678377 10.1016/j.jns.2008.06.031
positive Regulation: NGF --+> RAC17337280 10.1016/j.lfs.2007.01.057
positive Regulation: NGF --+> RAC21816277 10.1016/j.cell.2011.07.008
positive Regulation: NGF --+> RAC21816277 10.1016/j.cell.2011.07.008
positive Regulation: NGF --+> RAC21816277 10.1016/j.cell.2011.07.008
positive Regulation: NGF --+> RAC21816277 10.1016/j.cell.2011.07.008
positive Regulation: NGF --+> RAC1 10.1016/j.cell.2011.07.009

positive Regulation: NGF --+> RAC	17561310	10.1016/j.neuint.2007.04.032
positive Regulation: NGF --+> RAC	20144672	10.1016/j.neuint.2010.01.015
positive Regulation: NGF --+> RAC	20144672	10.1016/j.neuint.2010.01.015
positive Regulation: NGF --+> RAC	20144672	10.1016/j.neuint.2010.01.015
positive Regulation: NGF --+> RAC	20307615	10.1016/j.neuint.2010.03.005
positive Regulation: NGF --+> RAC	20307615	10.1016/j.neuint.2010.03.005
positive Regulation: NGF --+> RAC	20307615	10.1016/j.neuint.2010.03.005
positive Regulation: NGF --+> RAC	17055158	10.1016/j.neulet.2006.10.008
positive Regulation: NGF --+> RAC1		10.1016/j.ijdevneu.2008.09.171
positive Regulation: NGF --+> RAC	18593598	10.1016/j.cellsig.2008.06.002
positive Regulation: NGF --+> RAC	18593598	10.1016/j.cellsig.2008.06.002
positive Regulation: NGF --+> RAC	18558478	10.1016/j.cellsig.2008.05.002
positive Regulation: NGF --+> RAC1		10.1016/j.mcn.2005.08.006
positive Regulation: NGF --+> RAC1		10.1016/j.mcn.2005.08.006
positive Regulation: NGF --+> RAC1		10.1016/j.mcn.2005.08.006
positive Regulation: NGF --+> RAC1		10.1016/j.mcn.2005.08.006
positive Regulation: NGF --+> RAC1		10.1016/j.mcn.2005.08.006
positive Regulation: NGF --+> RAC1		10.1016/j.mcn.2005.08.006
positive Regulation: NGF --+> RAC1		10.1016/j.mcn.2005.08.006
positive Regulation: NGF --+> RAC	20347391	10.1016/j.molmed.2010.02.004
positive Regulation: NGF --+> RAC	22578496	10.1016/j.neuron.2012.04.015
positive Regulation: NGF --+> RAC	23601108	10.1016/j.chom.2013.03.007
positive Regulation: NGF --+> RAC	16168664	10.1016/j.mcn.2005.08.006
positive Regulation: NGF --+> RAC	16168664	10.1016/j.mcn.2005.08.006
positive Regulation: NGF --+> RAC	16168664	10.1016/j.mcn.2005.08.006
positive Regulation: NGF --+> RAC	16168664	10.1016/j.mcn.2005.08.006
positive Regulation: NGF --+> RAC	16168664	10.1016/j.mcn.2005.08.006
positive Regulation: NGF --+> RAC	16168664	10.1016/j.mcn.2005.08.006
positive Regulation: NGF --+> RAC	16168664	10.1016/j.mcn.2005.08.006
positive Regulation: NGF --+> RAC	27018747	10.1016/j.bbamcr.2016.03.020
positive Regulation: NGF --+> RAC	18296444	
positive Regulation: NGF --+> RAC	17535963	
positive Regulation: NGF --+> RAC	17535963	
positive Regulation: NGF --+> RAC	17535963	
positive Regulation: NGF --+> RAC	17535963	
positive Regulation: NGF --+> RAC	16046413	
positive Regulation: NGF --+> RAC	15728722	
positive Regulation: NGF --+> RAC	15728722	
positive Regulation: NGF --+> RAC	15728722	
positive Regulation: NGF --+> RAC	15728722	
positive Regulation: NGF --+> RAC	15728722	
positive Regulation: NGF --+> RAC	15728722	
positive Regulation: NGF --+> RAC	15728722	
positive Regulation: NGF --+> RAC	15728722	
positive Regulation: NGF --+> RAC	15728722	
positive Regulation: NGF --+> RAC	15728722	
positive Regulation: NGF --+> RAC	15728722	
positive Regulation: NGF --+> RAC	15728722	
positive Regulation: NGF --+> RAC	15728722	
positive Regulation: NGF --+> RAC	15630019	
positive Regulation: NGF --+> RAC	15289497	
positive Regulation: NGF --+> RAC	15289497	
positive Regulation: NGF --+> RAC	14570905	
positive Regulation: NGF --+> RAC	14570905	

positive Regulation: NGF --+> RAC(14570905
positive Regulation: NGF --+> RAC(14570905
positive Regulation: NGF --+> RAC(14570905
positive Regulation: NGF --+> RAC(14570905
positive Regulation: NGF --+> RAC(14557270
positive Regulation: NGF --+> RAC(14557270
positive Regulation: NGF --+> RAC(14557270
positive Regulation: NGF --+> RAC(14557270
positive Regulation: NGF --+> RAC(12871977
positive Regulation: NGF --+> RAC(12871977
positive Regulation: NGF --+> RAC(12871977
positive Regulation: NGF --+> RAC(12133829
positive Regulation: NGF --+> RAC(12133829
positive Regulation: NGF --+> RAC(12133829
positive Regulation: NGF --+> RAC(12133829
positive Regulation: NGF --+> RAC(12133829
positive Regulation: NGF --+> RAC(12133829
positive Regulation: NGF --+> RAC(12133829
positive Regulation: NGF --+> RAC(11279039
positive Regulation: NGF --+> RAC(11279039
positive Regulation: NGF --+> RAC(11279039
positive Regulation: NGF --+> RAC(11279039
positive Regulation: NGF --+> RAC(11279039
positive Regulation: NGF --+> RAC(11279039
positive Regulation: NGF --+> RAC(11279039
positive Regulation: NGF --+> RAC(11279039
positive Regulation: NGF --+> RAC1 10.1128/MCB.00255-16
positive Regulation: NGF --+> RAC(11279039
positive Regulation: NGF --+> RAC(11279039
positive Regulation: NGF --+> RAC(11279039
positive Regulation: NGF --+> RAC(11279039
positive Regulation: NGF --+> RAC(11279039
positive Regulation: NGF --+> RAC(11279039
positive Regulation: NGF --+> RAC(11279039
positive Regulation: NGF --+> RAC(11279039
positive Regulation: NGF --+> RAC(11279039
positive Regulation: NGF --+> RAC(11279039
positive Regulation: NGF --+> RAC(11279039
positive Regulation: NGF --+> RAC(11279039
positive Regulation: NGF --+> RAC(11279039
positive Regulation: NGF --+> RAC(11279039
positive Regulation: NGF --+> RAC(11279039
positive Regulation: NGF --+> RAC(11279039
positive Regulation: NGF --+> RAC(11279039
positive Regulation: NGF --+> RAC(11279039
positive Regulation: NGF --+> RAC(11279039
positive Regulation: NGF --+> RAC(11278419
positive Regulation: NGF --+> RAC(11278419
positive Regulation: NGF --+> RAC(11278419
positive Regulation: NGF --+> RAC(11278419
positive Regulation: NGF --+> RAC(11278419
positive Regulation: NGF --+> RAC(11278419
positive Regulation: NGF --+> RAC(11278419
positive Regulation: NGF --+> RAC(11278419

positive Regulation: NGF --+> RAC(11278419
positive Regulation: NGF --+> RAC(11278419
positive Regulation: NGF --+> RAC(11278419
positive Regulation: NGF --+> RAC(11278419
positive Regulation: NGF --+> RAC(11278419
positive Regulation: NGF --+> RAC(11278419
positive Regulation: NGF --+> RAC(11278419
positive Regulation: NGF --+> RAC(11278419
positive Regulation: NGF --+> RAC(11278419
positive Regulation: NGF --+> RAC(11278419
positive Regulation: NGF --+> RAC(11278419
positive Regulation: NGF --+> RAC(11278419
positive Regulation: NGF --+> RAC(9520479
positive Regulation: NGF --+> RAC(12514131
positive Regulation: NGF --+> RAC(16782872
positive Regulation: NGF --+> RAC(12446789
positive Regulation: NGF --+> RAC(20333299
positive Regulation: NGF --+> RAC(20333299
positive Regulation: NGF --+> RAC(20333299
positive Regulation: NGF --+> RAC(20333299
positive Regulation: NGF --+> RAC(20333299
positive Regulation: NGF --+> RAC(20333299
positive Regulation: NGF --+> RAC(20333299
positive Regulation: NGF --+> RAC(20333299
positive Regulation: NGF --+> RAC(20333299
positive Regulation: NGF --+> RAC(20333299
positive Regulation: NGF --+> RAC(20333299
positive Regulation: NGF --+> RAC(20333299
positive Regulation: NGF --+> RAC(20333299
positive Regulation: NGF --+> RAC(20333299
positive Regulation: NGF --+> RAC(20333299
positive Regulation: NGF --+> RAC(20333299
positive Regulation: NGF --+> RAC(20333299
positive Regulation: NGF --+> RAC(20333299
positive Regulation: NGF --+> RAC(15858067
positive Regulation: NGF --+> RAC(15858067
positive Regulation: NGF --+> RAC(21863044 10.1038/nrn3102
positive Regulation: NGF --+> RAC(23223289 10.1523/JNEUROSCI.1079-12.2012
positive Regulation: NGF --+> RAC(23223289 10.1523/JNEUROSCI.1079-12.2012
positive Regulation: NGF --+> RAC(23223289 10.1523/JNEUROSCI.1079-12.2012
positive Regulation: NGF --+> RAC(23223289 10.1523/JNEUROSCI.1079-12.2012
positive Regulation: NGF --+> RAC(23223289 10.1523/JNEUROSCI.1079-12.2012
positive Regulation: NGF --+> RAC(23223289 10.1523/JNEUROSCI.1079-12.2012
positive Regulation: NGF --+> RAC(22384148 10.1371/journal.pone.0032087
positive Regulation: NGF --+> RAC(22384148 10.1371/journal.pone.0032087
positive Regulation: NGF --+> RAC(22384148 10.1371/journal.pone.0032087
positive Regulation: NGF --+> RAC(22384148 10.1371/journal.pone.0032087
positive Regulation: NGF --+> RAC(24752242 10.1371/journal.pone.0095212
positive Regulation: NGF --+> RAC(24752242 10.1371/journal.pone.0095212
positive Regulation: NGF --+> RAC(24752242 10.1371/journal.pone.0095212
positive Regulation: NGF --+> RAC(12879077
positive Regulation: NGF --+> RAC(14557270
positive Regulation: NGF --+> RAC(15629443
positive Regulation: NGF --+> RAC(16168664

positive Regulation: NGF --+> RAC 17535963
positive Regulation: NGF --+> RAC 20144672 10.1016/j.neuint.2010.01.015
positive Regulation: NGF --+> RAC 20333299 10.1371/journal.pone.0009647
positive Regulation: NGF --+> RAC 20333299 10.1371/journal.pone.0009647
positive Regulation: NGF --+> RAC 22384148 10.1371/journal.pone.0032087
positive Regulation: NGF --+> RAC 23223289 10.1523/JNEUROSCI.1079-12.2012
positive Regulation: NGF --+> RAC 24732916 10.1159/000358679
positive Regulation: NGF --+> RAC 25006578 10.1155/2014/547187
positive Regulation: NGF --+> RAC1
positive Regulation: NGF --+> RAC 26966186 10.1242/jcs.184168
positive Regulation: NGF --+> RAC 26966186 10.1242/jcs.184168
positive Regulation: NGF --+> RAC 26966186 10.1242/jcs.184168
positive Regulation: NGF --+> RAC 29106600 10.1093/jrr/rrx03210.1093/jrr/rrx032
positive Regulation: NGF --+> RAC 29106600 10.1093/jrr/rrx03210.1093/jrr/rrx032
positive Regulation: NGF --+> RAC1 10.1691/ph.2018.8726
positive Regulation: NGF --+> RAC1 10.1691/ph.2018.8726
negative Expression: PAX8 ---| PF 20075182
negative Expression: PAX8 ---| PF 16179407
negative Expression: PAX8 ---| PF 12364466
negative Expression: PAX8 ---| PF 23128507 10.3892/ijo.2012.1681
negative Expression: PAX8 ---| PF 32056516 10.2174/0929867327666200214125712
negative Expression: PAX8 ---| PF 12161538
negative Expression: PAX8 ---| PF 12519876
negative Expression: PAX8 ---| PF 16150867
negative Expression: PAX8 ---| PF 16179407
negative Expression: PAX8 ---| PF 26105190 10.1016/j.ctrv.2015.06.002
negative Expression: PAX8 ---| PF 24485648 10.1016/j.ctrv.2014.01.002
negative Expression: PAX8 ---| PF 23602255 10.1016/j.oraloncology.2013.03.437
negative Expression: PAX8 ---| PF 10958784
negative Expression: PAX8 ---| PF 16150867 10.1210/me.2005-0197
negative Expression: PAX8 ---| PF 24405857 10.1016/j.critrevonc.2013.12.007
negative Expression: PAX8 ---| PPARG
Regulation: IGFBP4 ---> MAPK1 28595186 10.1159/000477873
Regulation: IGFBP4 ---> MAPK1 28595186 10.1159/00047787310.1159/000477873
Regulation: IGFBP4 ---> MAPK1 10.2174/1381612828666220608122934
Expression: AKT1 ---> ABCC1 32531682 10.1016/j.ejmech.2020.112359
Expression: AKT1 ---> ABCC1 32992133 10.1016/j.ejmech.2020.112830
Expression: AKT1 ---> ABCC1 29800725 10.1016/j.biocel.2018.05.013
Expression: AKT1 ---> ABCC1 30738829 10.1016/j.ebiom.2018.12.037
Expression: AKT1 ---> ABCC1 22445357 10.1016/j.imlet.2012.03.005
Expression: AKT1 ---> ABCC1 22445357 10.1016/j.imlet.2012.03.005
Expression: AKT1 ---> ABCC1 22537907 10.1016/j.phymed.2012.03.008
Expression: AKT1 ---> ABCC1 19745159
Expression: AKT1 ---> ABCC1 26469962 10.1038/cddis.2015.264
Expression: AKT1 ---> ABCC1 32404870 10.1038/s41419-020-2573-2
Expression: AKT1 ---> ABCC1 32973135 10.1038/s41419-020-02998-6
Expression: AKT1 ---> ABCC1 32973135 10.1038/s41419-020-02998-6
Expression: AKT1 ---> ABCC1 33714198 10.18632/aging.202679
Expression: AKT1 ---> ABCC1 33813658 10.1007/s10555-021-09959-1
Expression: AKT1 ---> ABCC1 23392708 10.1007/s11596-013-1071-5
Expression: AKT1 ---> ABCC1 23392708 10.1007/s11596-013-1071-5
Expression: AKT1 ---> ABCC1 20711237 10.1038/onc.2010.303
Expression: AKT1 ---> ABCC1 25760096 10.3892/mmr.2015.3468
Expression: AKT1 ---> ABCC1 26063481 10.3892/or.2015.4047

Expression: AKT1 ---> ABCC1	17215852	10.1038/sj.leu.2404523
Expression: AKT1 ---> ABCC1	17215852	10.1038/sj.leu.2404523
Expression: AKT1 ---> ABCC1	22082269	10.1134/S000629791109004510.1134/S00062979110
Expression: AKT1 ---> ABCC1	23940624	10.1371/journal.pone.007068510.1371/journal.p
Expression: AKT1 ---> ABCC1	34535128	10.1186/s12935-021-02209-y
Expression: AKT1 ---> ABCC1		10.1016/j.ebiom.2018.12.037
Expression: AKT1 ---> ABCC1		10.1016/j.phymed.2012.03.008
Expression: AKT1 ---> ABCC1	23940624	10.1371/journal.pone.0070685
Expression: AKT1 ---> ABCC1	23940624	10.1371/journal.pone.0070685
Expression: AKT1 ---> ABCC1	23940624	10.1371/journal.pone.0070685
Expression: AKT1 ---> ABCC1	23051672	10.1016/j.ejphar.2012.09.046
Expression: AKT1 ---> ABCC1	15784621	
Expression: AKT1 ---> ABCC1	26722004	10.1096/fj.15-283408
Expression: AKT1 ---> ABCC1		
Expression: AKT1 ---> ABCC1	33231085	10.4149/neo_2020_200605N604
negative Regulation: RAI2 ---| AK	32177171	10.1093/annonc/mdy151.188
negative Regulation: RAI2 ---| AK	32177171	10.1093/annonc/mdy151.188
negative Regulation: RAI2 ---| AK	32177171	10.1093/annonc/mdy151.188
negative Regulation: RAI2 ---| AK	33782811	10.1007/s10549-021-06176-w
negative Regulation: RAI2 ---| AK	29796120	10.1186/s13148-018-0501-410.1186/s13148-018-0
Expression: GPER1 ---> PPARG	35740412	10.3390/biomedicines1006139010.3390/biomedici
Expression: GPER1 ---> PPARG	35256537	10.1530/JOE-21-0398
Regulation: NGF ---> PTEN	20610402	10.1074/jbc.M110.147546
Regulation: NGF ---> PTEN	22465479	10.1016/j.yexcr.2012.03.006
Regulation: NGF ---> PTEN	20869442	10.1016/j.mcn.2010.09.006
Regulation: NGF ---> PTEN	23500101	10.1016/j.neuroscience.2013.02.059
Regulation: NGF ---> PTEN	16723502	
Regulation: NGF ---> PTEN	16723502	
Regulation: NGF ---> PTEN	16723502	
Regulation: NGF ---> PTEN	16723502	
Regulation: NGF ---> PTEN	16723502	
Regulation: NGF ---> PTEN	16723502	
Regulation: NGF ---> PTEN	16723502	
Regulation: NGF ---> PTEN	16723502	
Regulation: NGF ---> PTEN	16723502	
Regulation: NGF ---> PTEN	20237279	
Regulation: NGF ---> PTEN	28264976	10.1523/JNEUROSCI.2923-16.2017
Regulation: NGF ---> PTEN	28264976	10.1523/JNEUROSCI.2923-16.2017
Regulation: NGF ---> PTEN	22027198	10.1074/mcp.M111.013375
Regulation: NGF ---> PTEN	22918949	10.1091/mbc.E12-05-0367
Regulation: NGF ---> PTEN	22918949	10.1091/mbc.E12-05-0367
Regulation: NGF ---> PTEN	20610402	10.1074/jbc.M110.147546
Regulation: NGF ---> PTEN	16723502	10.1091/mbc.e05-12-1144
Regulation: NGF ---> PTEN		10.1016/j.yexcr.2012.03.006
Regulation: NGF ---> PTEN		10.1523/JNEUROSCI.2923-16.2017
Regulation: NGF ---> PTEN		10.1523/JNEUROSCI.2923-16.2017
Regulation: NGF ---> PTEN	20610402	10.1074/jbc.M110.147546
Regulation: NGF ---> PTEN	19070673	10.1016/j.cellbi.2008.11.010
Regulation: NGF ---> PTEN		
Regulation: SFRP1 ---> NOTCH1	18984672	
Regulation: SFRP1 ---> NOTCH1	21478884	10.1038/nn.2794
Regulation: SFRP1 ---> NOTCH1	25616895	10.1242/jcs.163063
Regulation: SFRP1 ---> NOTCH1	21896628	10.1242/dev.065839
Regulation: SFRP1 ---> NOTCH1	27985737	

Regulation: SFRP1 ---> NOTCH1
positive Expression: MIR107 --+> 33651959 10.1089/dna.2020.6356
positive Expression: MIR107 --+> 33651959 10.1089/dna.2020.6356
positive Expression: MIR107 --+> 28276320 10.3727/096504017X1488282907723710.3727/09650
positive Expression: MIR107 --+> 28063928 10.1016/j.bbrc.2017.01.01310.1016/j.bbrc.2017
positive Expression: MIR107 --+> 28276320 10.3727/096504017X14882829077237
positive Expression: MIR107 --+> 28276320 10.3727/096504017X14882829077237
positive Expression: MIR107 --+> 28276320 10.3727/096504017X14882829077237
positive Regulation: NOTCH1 --+> 26302803 10.2174/1389450116666150825113035
positive Regulation: NOTCH1 --+> 29160307 10.1038/nature24998
positive Regulation: NOTCH1 --+> 29160307 10.1038/nature24998
positive Regulation: NOTCH1 --+> 29160307 10.1038/nature24998
positive Regulation: NOTCH1 --+> 29160307 10.1038/nature24998
positive Regulation: NOTCH1 --+> 23688168 10.1186/1471-2407-13-244
positive Regulation: NOTCH1 --+> 23688168 10.1186/1471-2407-13-244
positive Regulation: NOTCH1 --+> 23688168 10.1186/1471-2407-13-244
positive Regulation: NOTCH1 --+> 23688168 10.1186/1471-2407-13-244
positive Regulation: NOTCH1 --+> 23688168 10.1186/1471-2407-13-244
positive Regulation: NOTCH1 --+> 23688168 10.1186/1471-2407-13-244
positive Regulation: NOTCH1 --+> 23688168 10.1186/1471-2407-13-244
positive Regulation: NOTCH1 --+> 23688168 10.1186/1471-2407-13-244
positive Regulation: NOTCH1 --+> 34425253 10.1016/j.cdev.2021.203735
positive Regulation: NOTCH1 --+> RAC1 10.2174/1389450116666150825113035
positive Regulation: NOTCH1 --+> 28268126 10.1016/j.mcn.2017.03.001
positive Regulation: NOTCH1 --+> 29058721 10.1038/ncb3628
positive Regulation: NOTCH1 --+> 36241874 10.1038/s41423-022-00930-w
positive Regulation: NOTCH1 --+> 36241874 10.1038/s41423-022-00930-w
negative Regulation: TP53 ---| CI 12034755
Expression: BECN1 ---> TP53 10.1016/j.semcancer.2019.07.013
Expression: BECN1 ---> TP53 24858802 10.1016/j.bcp.2014.05.009
Expression: BECN1 ---> TP53 24858802 10.1016/j.bcp.2014.05.009
Expression: BECN1 ---> TP53 27559856 10.1080/15548627.2016.1217380
Expression: BECN1 ---> TP53 29230017 10.1038/s41556-017-0007-x
Expression: BECN1 ---> TP53 29991800 10.1038/s41388-018-0395-9
Expression: BECN1 ---> TP53 10.1530/REP-18-0243
Expression: BECN1 ---> TP53 10.1007/978-981-15-0602-4_1
Expression: BECN1 ---> TP53 23486312 10.4161/auto.24132
Expression: BECN1 ---> TP53 23542691 10.1038/nn.3365
Expression: BECN1 ---> TP53 24256242 10.1042/BST20130185
Expression: BECN1 ---> TP53 25180518 10.4161/auto.34398
Expression: BECN1 ---> TP53 26530936 10.3892/ijo.2015.3227
Expression: BECN1 ---> TP53 21962518 10.1016/j.cell.2011.08.03710.1016/j.cell.2011
Expression: BECN1 ---> TP53 30539860 10.4103/0973-1482.20489810.4103/0973-1482.204
Expression: BECN1 ---> TP53 24833845 10.3748/wjg.v20.i17.4953
Expression: BECN1 ---> TP53 23466706 10.4161/cc.24128
Expression: BECN1 ---> TP53 22481206
Expression: BECN1 ---> TP53
Expression: BECN1 ---> TP53 10.1530/ERC-16-0359
Expression: BECN1 ---> TP53 34678225 10.1016/j.bcp.2021.114797
negative Expression: PPP1R3C ---|12727934
Expression: SPP1 ---> PTEN 27063991 10.1016/j.lfs.2016.04.00310.1016/j.lfs.2016.0
Expression: SPP1 ---> PTEN 28875010
Expression: SPP1 ---> PTEN 24039584 10.1371/journal.ppat.1003623
Expression: SPP1 ---> PTEN 19683677 10.1016/j.chom.2009.07.008

Expression: SPP1 ---> PTEN	19286136	10.1016/j.chom.2009.01.005
Expression: SPP1 ---> PTEN	28928218	10.1074/jbc.M117.797787
Expression: SPP1 ---> PTEN	28928218	10.1074/jbc.M117.797787
Expression: PTEN ---> SPP1	33130075	10.1016/j.lfs.2020.118680
Expression: PTEN ---> SPP1	19549529	10.1053/j.gastro.2009.06.039
Expression: PTEN ---> SPP1	28505114	10.3390/ijms18051058
Expression: PTEN ---> SPP1	24893171	10.1371/journal.pone.0097544
Expression: PTEN ---> SPP1	15705860	
Expression: PTEN ---> SPP1	15705860	
Expression: PTEN ---> SPP1	12460992	10.1074/jbc.M209299200
Expression: PTEN ---> SPP1	10970827	10.1165/ajrcmb.23.3.4002
Expression: PTEN ---> SPP1	24379438	10.1128/MCB.01384-13
Expression: PTEN ---> SPP1	12460992	
Expression: PTEN ---> SPP1	34240392	10.1007/s13577-021-00571-7

).04.002
;CI.4024-13.2014
freeradbiomed.2014.12.019

)9.004

.. 2020. 1749790
)19. 05. 015
)8-01-132068
3. 021
301(11)60032-8

)000000000961

g. 2018. 01. 004

010-3

2. 03. 152

.150-7

2014. 06. 020

)4. 003

.1
2014.01.028
-93484-2

pone.0065751
01.004

027-3
027-3
2-4934. 2009. 00709. x
2-4934. 2009. 00709. x

5
2-4934. 2012. 01602. x

0-0277. 2008. 00781. x
0-0277. 2008. 00781. x

2019.01.001

2020.129836

553733

2016.08.015

-05239-3

13113
13113

ıeuroscience.2021.04.010
·00571-x

)17.11.009

). 06. 004

·nta.2008.10.001

32.CCR-15-0016
0421-2
72.CAN-14-3362
72.CAN-14-3362
017.02.006
.986-y

5_18
0.104806
0.104806

-04293-4

.nes10020236

21914
:7
-01372-y

32

›one.0085116

2010. 10. 007

:8

g. 2018. 03. 013

)241

›one. 0111860
›one. 0111860

.3

..2020.1749790
..2020.1749790
..2020.1749790

-129
:3

freeradbiomed.2014.02.023
one.0106812

-00275-3

[illegible]. 12. 002

[illegible]. 03. 014

[illegible]4

14008
14008
[illegible]8-19.2020

-02147-z10.1038/s41580-019-0199-y10.1016/j.cell.2017.04.00110.1038/nrm.2017.9510.1
-02147-z10.1038/s41580-019-0199-y10.1016/j.cell.2017.04.00110.1038/nrm.2017.9510.1
-02147-z10.1038/s41580-019-0199-y10.1016/j.cell.2017.04.00110.1038/nrm.2017.9510.1

:01812_16625

)17. 10. 033

-89220-5
-89220-5

.2.011

017.08.028

.7

24770
)152
.403-9
.403-9
35
-03556-4

.4

-03047-2

2010.02.001

)599-5

)00000000804

ıes9020207

!088-x

;5

ı9-7006.2008.00904.x

;913-1

[illegible]913-1
[illegible]913-1
[illegible]913-1

:9-7006.2009.01302.x
:9-7006.2009.01302.x
:9-7006.2009.01302.x
:9-7006.2009.01302.x

;2.CCR-15-0016
;2
)919-y
!627

;3.MCT-20-1112
;3.MCT-20-1112

2021.119144

.03.010
587305

329788

-02550-w10.1038/s41575-020-0310-z10.1053/j.gastro.2016.11.04810.1038/nrclinonc.201
-01972-0

21914
2018.03.002
2018.03.002
on.2018.e01039
-01372-y
bio.3001199

418-y

418-y

-03185-3

)8. 010

.g. 2013. 02. 018

one. 0071453
51
51
51

.2. 011

)704

)7.021

[illegible]. 2021.1906500

19033
19033
'2.CAN-13-3076

)847-9
32. CCR-08-0629
18518

72. CAN-13-3590
)21. 112631
7. 2020. 1761748
)247-x

-149

-00348-510.3322/caac.2149210.1056/NEJMra171571510.1111/j.1600-0714.2007.00582.x10.

.HA.107.164749
25
25

.04308

.4
.4

)873

j. issn. 1009-2137. 2015. 03. 015

)625-9
)10. 03. 015
›one. 0019139

›one. 0077922
›one. 0102118

:01905_17948

-00504-4

[illegible]01809_15810
[illegible] cnki.cjcmm.20180601.001
[illegible].04.034

)07695

/2046-3758.108.BJR-2020-0127.R2

.05.023
.082-0

:6

ıres. 2010. 11. 062

)5

)3. 020
)373-x
.g. 2014. 06. 007

'959

13113
13113

ıeuroscience.2021.04.010

-00571-x

'. 2016. 4

.150-7

2021.107675

2021.107675

issn.1009-3419.2014.02.13
12984

[illegible]290
[illegible]290

)1.004

)one.0065751

5172

)pat.1007100

)0106N18

.257-9
36.MCR-14-0177

22

)09.06.024
)276-1
.2
.1
)675-2
)0
2014.01.028
rchoralbio.2014.07.013
5070

3. 11. 070

).08.025

ın.2008.09.003

rchoralbio.2009.12.003
)000000000192
)226-x

ın. 2009. 03. 015
ɜ3. MCT-09-0148

016. 05. 003

5.06.057
35
-01718-z10.1016/j.critrevonc.2020.10298810.1016/S0925-5710(98)00038-310.3109/07388
0913-4
freeradbiomed.2015.08.011

'2.CAN-20-0505

›pat.1007100

)040-z

.. 4449

39

-49021-3

. . 02. 093

32-4934.2008.00311.x

3-0538

)0

.g. 2012. 05. 011
.4

)981
·y. 2015. 35

-15170-6
.g. 2018. 01. 004

)981

)-8612-5_9
)-8612-5_9

:. 2021. 1906500
)7. 021

)4. 019

2019.172658

one.0095136
04.019

0/j.issn.1671-5411.2018.07.001

2010-0
2013.10.001
2069-4
0972-x
2019.109306

19281

19281

7

pat.1007100

biomaterials.2014.08.016

)000001806

3
606

)248-0

;759-5

.33231

›rgbio.2019.110989
›85523

›8.010

.. 08.037
:898

one.0066275

). 090222
)7
)7
.647-3

)one. 0106812

⁾. 16

2018. 05. 020

⁾3

)289-6

.0.026
.g.2018.03.013

36.MCR-21-0149

)027-3
)027-3
2015.11.006
2016.12.006

2020.110769

32.CCR-18-0867

›one.0071453
›1
›1
›1

12984

5729.2019.036

-02951-7

freeradbiomed.2021.06.025

.85.2009

)2.006
)13.07.011
:3

.2. 007
)7. 016
2019. 172667

'5
'5

2018.08.102

.211

.5

3e3282f343e1
.8

.649-y
3.11.045

)027-3
)027-3
2015.11.006
2016.12.006
2020.110769

).090803

3e318297d741

5805

)21. 106001
3. 05. 017
pone. 0018823
29
29

.539-9
.539-9

.51. 2009
)90. 2012

)1
)1

)895-9

.g.2017.06.012
ıotobiol.2017.10.006
)0067X

)6.008

:10.1615/JEnvironPatholToxicolOncol.2018026684

)1
)1
2019.172586

5822

36.MCR-20-0173
one.0064771

3e31827912b8
03.018
013.07.011
oids.2016.01.021
.2.007

2018.01.040
2018.08.102

36.MCR-20-0173
2022.113290

j.2022.36.S1.R5122

[illegible]1.2020.08.007
[illegible]3.MCT-12-0342

.361-2
.361-2

'2.CAN-12-2220

)20.101774
)20.101774

2018.08.132

[illegible]. 2020. 1838032

[illegible]one. 0126176
[illegible]4016X14666990347437

[illegible]761-3

[illegible]01826-5

[illegible]. 05. 009

13355
13355

'2. CAN-19-0950
-04958-8

.1
2014.01.028
-93484-2

ıeuroscience.2012.01.002

[illegible]206-5

[illegible]418-y
[illegible]418-y

i3.MCT-10-0336
.2018.1553607
3.12.005
)00000003576

3000-6

one.0122179

g.2011.09.015

ıber. 18. 18

ıc. 2011. 07. 006
)4. 017

3. 02. 187

3. 02. 187

›one. 0065821
›one. 0065821

ın. 2016. 03. 001
3. 08. 037
2019
2019
2019

.720

:7

-01372-y

pone. 0004911
2434-7
pone. 0212749
. 2020. 1711578

i3

.g. 2013. 02. 018

32-0436. 2007. 00261. x

pone.0100515

017.10.033

2019.03.005

38-19.2020

14008
14008

.. 2020. 1749790
.. 2020. 1749790
.. 2020. 1749790

-00275-3

-129
:3

freeradbiomed.2014.02.023

-00348-510.3322/caac.2149210.1056/NEJMra171571510.1111/j.1600-0714.2007.00582.x10.

.04308

.4
.4
.150-7
12768

)697-7
)697-7
)697-7

-03105-8

). 2022. 2051266

)8
2069-4
2069-4
.625-y

)18.03.014

). 01. 060

)617-y
2012.11.047
)7

)635-6
)635-6

'8

:759-5

'476

ıres. 2010. 11. 062
)2. 005

›one. 0019139
2016. 04. 015

)7
2012.11.047
)617-y

'.2015.1029836

-01072-510.1016/j.ygyno.2018.05.01510.1016/j.bulcan.2017.10.00610.2174/13894501179

-08671-110.1046/j.1471-4159.2003.01562.x10.1038/sj.onc.120696510.1016/j.neuroscien
-02873-4
63

SCI.0936-16.2016

'2.CAN-08-0579
'2.CAN-08-0579
'2.CAN-16-3470

00164

2015.05.014

[illegible]2.CCR-17-1574

32. CCR-17-1574

-196

51

2854
08

017. 08. 028

3. 02. 187
3. 02. 187

one. 0096466

)9
33.MCT-07-2183
).03.024
72.CAN-10-4531
3.09.008

22926

[illegible]. 09. 008
[illegible]. 09. 008
[illegible]. 115013
[illegible]20. 112343

.217-9

). 06. 160
). 06. 160

-00756-8

2009.09.003
2011.11.004

[illegible]. 12.063
[illegible]213
2016.06.027
[illegible]00000002440

[illegible]00000002440

2011.06.008
2017.06.089
.119-x
-00519-x10.3322/caac.2133810.1007/s11748-013-0246-010.1016/j.athoracsur.2018.02.01

)2
2013.06.009
25349

)05840
)05840

-03047-2

freeradbiomed.2015.08.011

:RTENSIONAHA.114.03884
j.prostaglandins.2012.09.004

'2.CAN-11-2291

2019.02.036

[illegible]4014X14024160459087

[illegible]04207

)030-x

'2.CAN-09-2316
)609-0
.HA.112.280399

ieurol.2014.05.014

·26473-7

›one.0143663

-12648-1

)805-8

.08903
.08903

)3.056

36.MCR-08-0493
36.MCR-08-0493
36.MCR-08-0493
36.MCR-08-0493
36.MCR-09-0004
36.MCR-09-0004
).090222
2011.07.011

2015.09.018

'452
'452
.318-6

-00283-010.1002/jcb.2008010.1016/j.arcmed.2020.02.00510.1016/j.beha.2019.05.00810.

36.MCR-08-0493
36.MCR-08-0493
36.MCR-08-0493
36.MCR-08-0493
36.MCR-09-0004
36.MCR-09-0004
).090222

2

)90045
›one.0070685

)501-4
.nes10061390

4017X14882829077237
. 01. 013

. 08. 037
898

4. 003

038/sj.onc.120566210.1073/pnas.050285710210.1016/j.cell.2008.06.02810.1016/j.molc
038/sj.onc.120566210.1073/pnas.050285710210.1016/j.cell.2008.06.02810.1016/j.molc
038/sj.onc.120566210.1073/pnas.050285710210.1016/j.cell.2008.06.02810.1016/j.molc

.7. 15710. 1016/j. jhep. 2020. 11. 03710. 1038/s41572-021-00300-210. 1016/j. annonc. 2021. 04

3322/caac.2138810.1016/S0140-6736(08)60728-X10.4065/83.4.48910.1038/sj.bjc.660251

3551.2015.101595710.1038/nature0592510.1080/15548627.2016.116435910.21037/atm.2019

3322/caac.2138810.1016/S0140-6736(08)60728-X10.4065/83.4.48910.1038/sj.bjc.660251

590653410.1186/s12885-018-5074-210.1016/j.ygyno.2014.05.01810.3892/ijo.2017.40931

ıce. 2008. 07. 00110. 1371/journal. pone. 010537210. 1196/annals. 1332. 00810. 1111/j. 1476-5

810.21037/cco.2017.07.0210.1016/j.oraloncology.2008.06.00210.1016/j.tox.2006.07.0

1038/cdd.2017.18610.1016/j.ccell.2019.09.00810.1042/BCJ2017008010.1097/PPO.000000

el. 2010. 09. 02610. 1016/S1097-2765(01)00323-910. 1
el. 2010. 09. 02610. 1016/S1097-2765(01)00323-910. 1
el. 2010. 09. 02610. 1016/S1097-2765(01)00323-910. 1

:. 01210. 1158/2159-8290. CD-20-087310. 1038/s41591-

010.1200/JCO.2015.61.292910.1126/science.120692

). 02. 2210. 1371/journal. pone. 009015110. 1038/onc. 2

010.1200/JCO.2015.61.292910.1126/science.120692

0.1182/blood-2007-05-08868210.1023/A:1010921313

381. 2009. 00022. x10. 1371/journal. pone. 002189110.

01110.1016/j.toxlet.2013.10.00410.1289/ehp.09010

000000040810. 1080/10428194. 2017. 128303210. 3390/
